
JOURNAL INFORMATION
==============================
NLM Title Abbreviation: Schizophr Bull
Journal ID: Schizophr Bull
NLM Journal ID: 0236760
Journal ID: schbul

Schizophrenia Bulletin

ISSN: 0586-7614
EISSN: 1745-1701
Publisher: Oxford University Press

ARTICLE INFORMATION
==============================
PMCID: PMC5905517
DOI: 10.1093/schbul/sby014
Article ID: sby014
Article version: 1
Subjects: Sixth Biennial SIRS Conference, Abstracts for the Sixth Biennial SIRS Conference

Abstracts for the Sixth Biennial SIRS Conference

Print publication date: 2018 Apr
Electronic publication date: 2018 Apr 12
Volume: 44
Issue: Suppl 1
Issue title: Abstracts for the Sixth Biennial SIRS Conference
First page: S1
Last page: S455
Copyright: © Maryland Psychiatric Research Center 2018.
Copyright year: 2018
License: This is an Open Access article distributed under the terms of the Creative Commons Attribution License (http://creativecommons.org/licenses/by/4.0/), which permits unrestricted reuse, distribution, and reproduction in any medium, provided the original work is properly cited.
License URL: https://creativecommons.org/licenses/by/4.0/

Page count: 460

==============================
ISSN 0586-7614 (PRINT)
ISSN 1745-1701 (ONLINE)

VOLUME 44 • SUPPLEMENT 1 • APRIL 2018
academic.oup.com/schizophreniabulletin

### SCHIZOPHRENIA BULLETIN

The Journal of Psychoses and Related Disorders

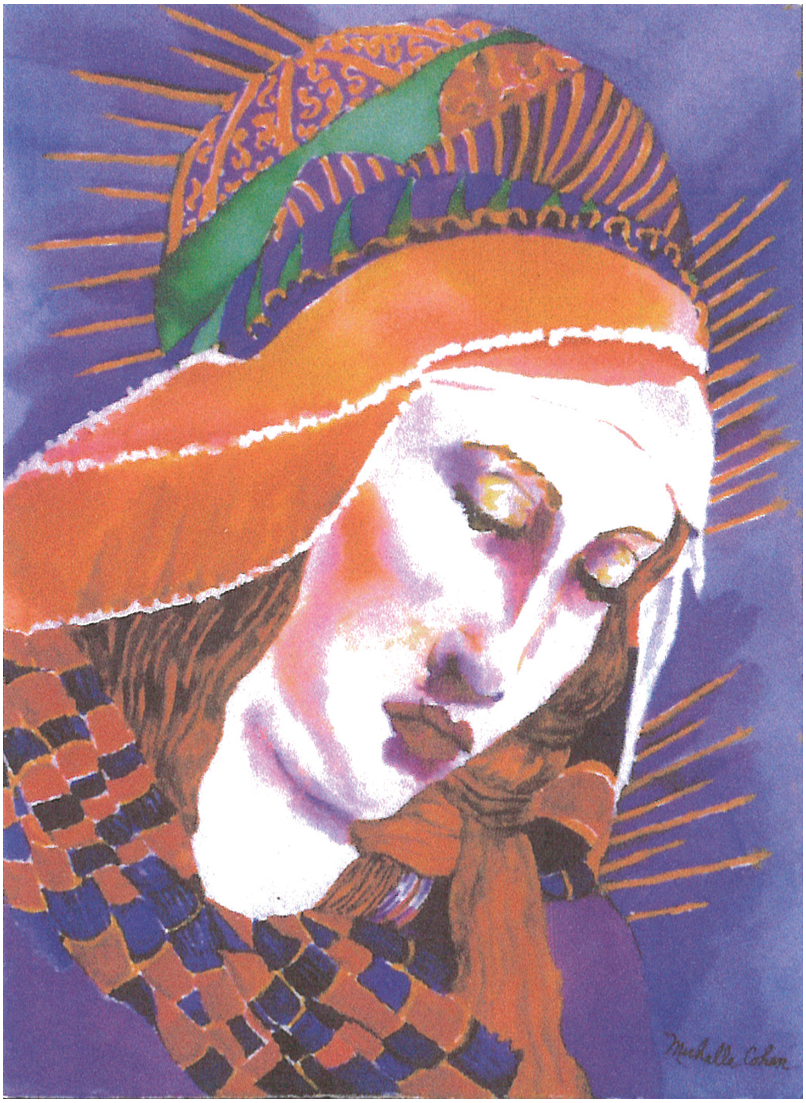

UNIVERSITY of MARYLAND
SCHOOL OF MEDICINE

MPRC
Maryland Psychiatric Research Center

OXFORD
UNIVERSITY PRESS

SCHIZOPHRENIA
BULLETIN
The Journal of Psychoses and Related Disorders

Volume 44, Supplement 1 April 2018

academic.oup.com/schizophreniabulletin

**Editor-in-Chief**
William T. Carpenter, Baltimore, MD

**Managing Editor**
Janet L. Smith, Baltimore, MD

**Associate Editors**
Aiden Corvin, Dublin, Ireland
Peter Falkai, Munich, Germany
Svein Friis, Oslo, Norway
Michael F. Green, Los Angeles, CA
Philip D. Harvey, Miami, FL
John J. McGrath, Brisbane, Australia
Vishwajit L. Nimgaonkar, Pittsburgh, PA
Laura M. Rowland, Baltimore, MD
Paul D. Shepard, Baltimore, MD
Rebecca Syed Sheriff, Adelaide, South Australia
Jim van Os, Utrecht, The Netherlands
Eduard Vieta, Barcelona, Spain

**Editorial Board**
Jean Addington, Calgary, Canada
Anthony O. Ahmed, White Plains, NY
Schrahram Akbarian, Worcester, MA
Ole A. Andreassen, Oslo, Norway
Paul S. Appelbaum, New York, NY
Celso Arango, Madrid, Spain
Deanna M. Barch, St. Louis, MO
Paul Bebbington, London, UK
Richard Bentall, Liverpool, Merseyside, UK
Stefan Borgwardt, Basel, Switzerland
Christopher R. Bowie, Kingston, Ontario, Canada
Alan S. Brown, New York, NY
Robert W. Buchanan, Baltimore, MD
Peter F. Buckley, Augusta, GA
Katherine Burdick, Boston, MA
Juan R. Bustillo, Albuquerque, NM
Pamela Butler, Orangeburg, NY
Mary Cannon, Dublin, Ireland
Raymond C. K. Chan, Beijing, China
Shuo Chen, Baltimore, MD
Alex S. Cohen, Baton Rouge, LA
Cheryl M. Corcoran, New York, NY
Philip R. Corlett, New Haven, CT
John M. Davis, Chicago, IL
Paola Dazzan, London, UK
Jordan E. DeVylder, New York, NY USA
Dwight Dickinson, Bethesda, MD
Lisa Dixon, New York, NY
Shuan M. Eack, Pittsburgh, PA
Charles Fernyhough, Durham, UK
Helen L. Fisher, London, UK
Judith Ford, San Francisco, CA
Frederick J. Frese, Rootstown, OH
Paolo Fusar-Poli, London, UK
Donald Goff, New York, NY
James Gold, Baltimore, MD
Eric L. Granholm, San Diego, CA
Stephan Heckers, Nashville, TN
Elliot Hong, Baltimore, MD
Matthew J. Hoptman, Orangeburg, NY
William P. Horan, Los Angeles, CA
Assen Jablensky, Perth, Australia
Zhao Jingping, Changsha, Hunan, P.R. China
René S. Kahn, New York, NY
John Kane, Glen Oaks, NY
Brian Kirkpatrick, Reno, NV
Nikolaos Koutsouleris, Munich, Germany
Adrienne Lahti, Birmingham, AL
Stephen Lawrie, Edinburgh, Scotland, UK
Junghee Lee, Los Angeles CA
Mark F. Lenzenweger, Ithaca, NY
Stefan Leucht, Munich, Germany
Deborah Levy, Belmont, MA
Jean-Pierre Lindenmayer, New York, NY
Paul H. Lysaker, Indianapolis, IN
Anil H. Malhotra, Glen Oaks, NY
Stephen Marder, Los Angeles, CA
Patrick McGorry, Melbourne, Australia
Andreas Meyer-Lindenberg, Mannheim, Germany
Brian Miller, Augusta, GA
Vijay A. Mittal, Evanston, IL
Romina Mizrahi, Toronto, Canada
Craig Morgan, London, UK
Douglas L. Noordsy, Palo Alto, CA
Merete Nordentoft, Copenhagen, Denmark
Georg Northoff, Ottawa, Ontario, Canada
Sohee Park, Nashville, TN
Josef Parnas, Copenhagen, Denmark
Godfrey D. Pearlson, Hartford, CT
David L. Penn, Chapel Hill, NC
Victor Peralta, Pamplona, Spain
Amy E. Pinkham, Richardson, TX
Thara Rangaswamy, Chennai, India
Norman Sartorius, Geneva, Switzerland
Louis Sass, Piscataway, NJ
Akira Sawa, Baltimore, MD
Jason Schiffman, Baltimore, MD
Jean-Paul Selten, Maastricht, The Netherlands
Emily G. Severance, Baltimore, MD
Iris Sommer, Groningen, The Netherlands
Gregory P. Strauss, Athens, GA
John Strauss, New Haven, CT
Philip R. Szeszko, New York, NY
Carol A. Tamminga, Dallas, TX
Rajiv Tandon, Tallahassee, FL
Peter J. Uhlhaas, Glasgow, UK
Aristotle Voineskos, Toronto, Canada
Sebastian Walther, Bern, Switzerland
James Waltz, Baltimore, MD
Flavie Waters, Perth, Australia
Scott W. Woods, New Haven, CT

### SCHIZOPHRENIA BULLETIN

The Journal of Psychoses and Related Disorders

Volume 44, Supplement 1 April 2018

academic.oup.com/schizophreniabulletin

### Editor-in-Chief
William T. Carpenter, Baltimore, MD

### Former Editors-in-Chief
Loren Mosher (1969–1980)
Samuel Keith (1980–1993)
David Shore (1986–2000)
John Hsiao (1997–2004)

### Editorial Office
Maryland Psychiatric Research Center

### Publisher
Oxford University Press

### Publishing Staff
Anna Hernandez French, Editor
Chloe Miller, Marketing Associate
Jack Cassidy, Production Editor

**Subscriptions**
A subscription to *Schizophrenia Bulletin* comprises 6 issues. Prices include postage; for subscribers outside the Americas, issues are sent air freight. Airmail rates are available on request. *Schizophrenia Bulletin* Advance Access contains papers that have recently been accepted but have not yet been included within an issue. Advance Access is updated weekly.

Annual Subscription Rate (Volume 44, 6 issues, 2018)
*Institutional*
Print edition and site-wide online access: US$614/£410/€614
Print edition only: US$564/£377/€564
Site-wide online access only: US$491/£330/€491
*Personal*
Print edition and individual online access: US$143/£96/€143
Please note: US$ rate applies to US & Canada, Euros applies to Europe, UK£ applies to UK and Rest of World.

There are other subscription rates available; for a complete listing, please visit www.schizophreniabulletin.oxfordjournals.org/subscriptions.

Full prepayment in the correct currency is required for all orders. Orders are regarded as firm, and payments are not refundable. Subscriptions are accepted and entered on a complete volume basis. Claims cannot be considered more than four months after publication or date of order, whichever is later. All subscriptions in Canada are subject to GST. Subscriptions in the EU may be subject to European VAT. If registered, please supply details to avoid unnecessary charges. For subscriptions that include online versions, a proportion of the subscription price may be subject to UK VAT. Personal rates are applicable only when a subscription is for individual use and are not available if delivery is made to a corporate address.

The current year and two previous years' issues are available from Oxford Journals. Previous volumes can be obtained from the Periodicals Service Company at http://www.periodicals.com/oxford.html or Periodicals Service Company, 11 Main Street, Germantown, NY 12526, USA. E-mail: psc@periodicals.com. Tel: (518) 537-4700. Fax: (518) 537-5899.

**Contact information:** Journals Customer Service Department, Oxford University Press, Great Clarendon Street, Oxford OX2 6DP, UK. E-mail: jnls.cust.serv@oup.com.+ Tel: +44 (0)1865 353907. Fax: +44 (0)1865 353485. **In the Americas, please contact:** Journals Customer Service Department, Oxford University Press, 2001 Evans Road, Cary, NC 27513, USA. E-mail: jnlorders@oup.com. Tel: (800) 852-7323 (toll-free in USA/Canada) or (919) 677-0977. Fax: (919) 677-1714. **In Japan, please contact:** Journals Customer Service Department, Oxford University Press, 4-5-10-8F Shiba, Minato-ku, Tokyo, 108-8386, Japan. E-mail: custserv.jp@oup.com. Tel: +81 3 5444 5858. Fax: +81 3 3454 2929.

**Methods of payment:** (i) Cheque (payable to Oxford University Press, to Oxford University Press, Cashiers Office, Great Clarendon Street, Oxford OX2 6DP, UK) in GB£ Sterling (drawn on a UK bank), US$ Dollars (drawn on a US bank), or EU€ Euros. (ii) Bank transfer to Barclays Bank Plc, Oxford Group Office, Oxford (bank sort code 20-65-18) (UK), overseas only Swift code BARC GB 22 (GB£ Sterling to account no. 70299332, IBAN GB89BARC20651870299332; US$ Dollars to account no. 66014600, IBAN GB27BARC20651866014600; EU€ Euros to account no. 78923655, IBAN GB16BARC20651878923655). (iii) Credit card (Mastercard, Visa, Switch or American Express)

**Environmental and ethical policies**
Oxford Journals, a division of Oxford University Press, is committed to working with the global community to bring the highest quality research to the widest possible audience. Oxford Journals will protect the environment by implementing environmentally friendly policies and practices wherever possible. Please see http://www.oxfordjournals.org/ethicalpolicies.html for further information on environmental and ethical policies.

**Postal information**
*Schizophrenia Bulletin* (ISSN 0586-7614) is published six times a year, in January, March, May, July, September, and November, by Oxford University Press, 2001 Evans Road, Cary, NC 27513-2009. Periodicals Postage Paid at Cary, NC and additional mailing offices. Postmaster: Send address changes to *Schizophrenia Bulletin*, Journals Customer Service Department, Oxford Journals, 2001 Evans Road, Cary, NC 27513-2009.

**Digital Object Identifiers**
For information about dois and how to resolve them, please visit http://www.doi.org/.

**Submissions**
**Information** for authors submitting manuscripts for review can be found on the journal website at www.oxfordjournals.org/schbul/for_authors/index.html.

**Permissions**
For information on how to request permissions to reproduce articles or information from this journal, please visit www.oxfordjournals.org/jnls/permissions.

**Advertising**
Advertising, inserts, and artwork enquiries should be addressed to Advertising and Special Sales, Oxford Journals, Oxford University Press, Great Clarendon Street, Oxford, OX2 6DP, UK. Tel: +44 (0) 1865 354767. Fax: +44 (0) 1865 353774.
E-mail: jnlsadvertising@oup.com.

**Drug disclaimer**
The mention of trade names, commercial products or organizations, and the inclusion of advertisements in the journal does not imply endorsement by *Schizophrenia Bulletin*, the editors, the editorial board, Oxford University Press, the Maryland Psychiatric Research Center, or the organization to which the authors are affiliated. The editors and publishers have taken all reasonable precautions to verify drug names and doses, the results of experimental work, and clinical findings published in the journal. The ultimate responsibility for the use and dosage of drugs mentioned in the journal and in interpretation of published material lies with the medical practitioner, and the editors and publishers cannot accept liability for damages arising from any errors or omissions in the journal. Please inform the editors of any errors.

**Disclaimer**
Statements of fact and opinion in the articles in *Schizophrenia Bulletin* are those of the respective authors and contributors and not of *Schizophrenia Bulletin*, the Maryland Psychiatric Research Center, or Oxford University Press. Neither Oxford University Press nor *Schizophrenia Bulletin* or the Maryland Psychiatric Research Center make any representation, express or implied, in respect of the accuracy of the material in this journal and cannot accept any legal responsibility or liability for any errors or omissions that may be made. The reader should make her or his own evaluation as to the appropriateness or otherwise of any experimental technique described.

Copyright © 2018 Oxford University Press and the Maryland Psychiatric Research Center

All rights reserved; no part of this publication may be reproduced, stored in a retrieval system, or transmitted in any form or by any means, electronic, mechanical, photocopying, recording, or otherwise without prior written permission of the publisher or a license permitting restricted copying issued in the UK by the Copyright Licensing Agency Ltd, 90 Tottenham Court Road, London W1P 9HE, or in the USA by the Copyright Clearance Center, 222 Rosewood Drive, Danvers, MA 01923. For those in the USA or Canada not registered with CCC, articles can be obtained by fax in 48 hours by calling: WISE for Medicine™ 1-800-667-WISE.

*Schizophrenia Bulletin* is printed on acid-free paper that meets the minimum requirements of ANSI Standard Z39.48-1984 (Permanence of Paper), beginning with volume 31, number 1.

Oxford University Press is a department of the University of Oxford. It furthers the University's objective of excellence in research, scholarship, and education by publishing worldwide.

Typeset by Newgen Knowledge Works Pvt Ltd, Chennai, India; Printed by Sheridan Press, USA.

*Schizophrenia Bulletin* is covered by the following major indexing services: ISI, Medline (Index Medicus).

### Abstracts for the Sixth Biennial SIRS Conference

### April 2018

**Supported by an unrestricted educational grant from the Schizophrenia International Research Society.**

SCHIZOPHRENIA
BULLETIN
The Journal of Psychoses and Related Disorders

### Contents

OXFORD OPEN

**Plenary/Symposia** . . . . . . . . . . . . . . . . . . . . . . . . . . . . . . . . . . . . . . . . . . . . . . . . . . . . . . . . . . . . **S1**
**Oral Abstracts** . . . . . . . . . . . . . . . . . . . . . . . . . . . . . . . . . . . . . . . . . . . . . . . . . . . . . . . . . . . . . . **S72**
**Posters (Session I, Session II, Session III)** . . . . . . . . . . . . . . . . . . . . . . . . . . . . . . . . . . . . . . **S113**
**Author Index** . . . . . . . . . . . . . . . . . . . . . . . . . . . . . . . . . . . . . . . . . . . . . . . . . . . . . . . . . . . . . . **S429**

**About the Cover**
Michelle Cohen
Scientific theories of the universe and man have influenced my artwork. I enjoy different styles because it's more exciting. As in Picasso's quote: "God doesn't work in only one style." I like painting the natural world, but also fantasy.

### Keynote

### 1. CHANGING THE LENS ON MENTAL HEALTH

Alastair Campbell
*Author*

**Overall Abstract:** Alastair Campbell, talking from personal and family experience, and based on years of campaigning and study, urges a rethink of how we view mental health and mental illness.

### Plenary

### 2. MICROCIRCUITS, MACROCIRCUITS, AND CORTICOL DYSFUNCTION IN SCHIZOPHRENIA: A COMPUTATIONAL AND TRANSLATIONAL NEUROSCIENCE PERSPECTIVE

John Krystal
*Yale University School of Medicine*

**Overall Abstract:** Computational neuroscience may be a critical component of the effort to understand how cortical micro- and macro-circuits support behavior and express the symptoms of neuropsychiatric disorders. This presentation will present an update on an ongoing interdisciplinary effort to understand the role of compromised glutamate synaptic signaling, particularly related to the NMDA glutamate receptor, for the pathophysiology of schizophrenia. This presentation will draw on studies in animal models, healthy humans, and schizophrenia patients. It will draw parallels between the effects of the NMDA receptor antagonist, ketamine, and working memory impairment and abnormalities in cortical functional connectivity in schizophrenia. In so doing, it will highlight examples where computational approaches have affirmed hypotheses arising from experimental work or contributed new predictions that could be tested experimentally. Lastly, it will illustrate a prediction about novel therapeutics for schizophrenia that are embedded in an emerging developmental model for this disorder.

### Concurrent Symposia

### 3. EXCITATION-INHIBITION IMBALANCES IN SCHIZOPHRENIA: MECHANISMS AND INTERVENTIONS

Lawrence Kegeles
*Columbia University & New York State Psychiatric Institute*

**Overall Abstract:** Evidence is accumulating that core features of schizophrenia (SCH) may arise from a fundamental disturbance in the cellular balance of excitation and inhibition (E-I balance) within neural circuitry. In the symposium, we will provide a comprehensive overview of E-I balance alterations in SCH with evidence from preclinical models and in vivo measurements investigating potential neurobiological mechanisms underlying these dysfunctions as well as interventions that remedy these disturbances.
Takao Hensch will summarize findings on critical period plasticity and its potential role in vulnerability to schizophrenia. He will present new preclinical data on the destabilizing consequences of enhanced gamma oscillations, which reversibly prolong juvenile forms of brain plasticity by redox imbalance.
Jan-Harry Cabungcal will address the role of redox dysregulation and oxidative stress in the pathophysiology of schizophrenia. He will present recent data on the relationship of deficits of the perineuronal net and oxidative stress in the anterior cingulate cortex, and evidence that redox dysregulation can be targeted with antioxidants/redox regulators across animal models.
Lawrence Kegeles will present simultaneous EEG and proton MRS measurements of glutamate and GABA during ketamine administration in healthy young adults. These data will be compared with the same modalities acquired in individuals at clinical high risk and patients with SCH, showing disturbed delta and gamma band power and altered E-I balance despite homeostatic rebalancing of glutamate and GABA.
Peter Uhlhaas will summarize evidence from EEG/MEG data examining the potential role of neural oscillations in the pathophysiology of schizophrenia. He will show that alterations in gamma-band oscillations are present prior to the onset of schizophrenia in at-risk individuals and related to aberrant E-I balance parameters revealed by MRS-measured levels of GABA and glutamate. Developmental data on the maturation of neural oscillations suggests that the transition from adolescence to adulthood is a sensitive period for modifications in neuronal dynamics that could potentially explain the manifestation of psychosis during this period.

### 3.1 ENHANCED PARVALBUMIN NETWORK ACTIVITY PROLONGS CRITICAL PERIOD PLASTICITY

Hensch Takao*,[1]
[1]*Harvard University*

**Background:** Oscillations in neuronal activity tie the pathophysiology of schizophrenia to alterations in local processing and large-scale coordination, and these alterations in turn can lead to the cognitive and perceptual disturbances observed in schizophrenia. Here, we focus on the dual role of fast-spiking, parvalbumin (PV+) networks in the generation of gamma oscillations and critical periods of brain plasticity.
**Methods:** We generated a mouse model of reduced recurrent inhibition only within local PV+ cell networks by selective removal of GABAA receptor alpha1 subunits (PV-$\alpha$1 KO mice). Electroencephalography (EEG), PV+ immunohistochemistry, perineuronal net (PNN) labeling and redox balance were compared to cortical measures of brain plasticity (loss of visual acuity, formation of preference behaviors) that are typically limited to a critical period early in life.
**Results:** PV-$\alpha$1 KO mice exhibit chronically enhanced gamma-oscillations and extended juvenile forms of cortical plasticity into adulthood. Acute pharmacological suppression of excitatory input restored E-I balance onto these disinhibited PV+ cells and returned baseline EEG power to normal levels, preventing the extended plasticity. Enhanced gamma oscillations were further found to compromise the integrity of perineuronal nets (PNNs) surrounding PV+ cells, elevating oxidative stress and the turnover of metallopeptidases and structural components of the PNN. All of these aspects were also reversed by pharmacological dampening of excitation onto PV+ cells.
**Discussion:** Cortical gamma oscillations are associated with plasticity and cognition. Our results provide a cellular explanation of how elevated gamma oscillations may promote ectopic brain plasticity by regulating the extracellular matrix which normally stabilizes cortical circuitry. These results carry broad implications for subjects at-risk for schizophrenia who exhibit heightened gamma oscillations prior to psychosis onset (see talk by P Uhlhaas).

### 3.2 PARVALBUMIN INTERNEURON IMPAIRMENT INDUCED BY OXIDATIVE STRESS AS A COMMON PATHOLOGICAL MECHANISM IN ANIMAL MODELS OF SCHIZOPHRENIA

Jan Harry Cabungcal*,[1], Pascal Steullet[1], Joseph Coyle[2], Michael Didriksen[3], Kathryn Gill[5], Anthony Grace[4], Hensch Takao[5], Anthony LaMantia[6], Lothar Lindemann[7],

Thomas Maynard[7], Urs Meyer[8], Hirofumi Morishita[9], Patricio O'Donnell[10], Matthew Puhl[2], Michel Cuenod[1], Kim Q. Do[1]
*[1]Center for Psychiatric Neuroscience, Lausanne University Hospital; [2]Laboratory for Psychiatric and Molecular Neuroscience, Harvard Medical School, McLean Hospital; [3]H. Lundbeck A/S; [4]University of Pittsburgh; [5]Harvard University; [6]George Washington Institute for Neuroscience, The George Washington University; [7]F. Hoffmann-La Roche Ltd., Roche Pharmaceutical and Early Development, Neuroscience, Ophthalmology & Rare Disease (NORD) DTA, Discovery Neuroscience, Roche Innovation Center Basel; [8]Institute of Pharmacology and Toxicology, University of Zurich-Vetsuisse; [9]Friedman Brain Institute, Mindich Child Health and Development Institute, Icahn School of Medicine at Mount Sinai; [10]Pfizer, Inc.*

**Background:** Parvalbumin inhibitory interneurons (PVIs) are crucial for maintaining proper excitatory/inhibitory balance and high-frequency neuronal synchronization. Their activity supports critical developmental trajectories, sensory and cognitive processing, and social behavior. Despite heterogeneity in the etiology across schizophrenia and autism spectrum disorder, PVI circuits are altered in these psychiatric disorders. Identifying mechanism(s) underlying PVI deficits is essential to establish treatments targeting in particular cognition. Based on our previous publications and new data, we propose oxidative stress as a common pathological mechanism leading to PVI impairment in schizophrenia and some forms of autism.
**Methods:** Using immunohistochemistry technique and confocal imaging analysis, we assessed the relationship between oxidative stress (as revealed by 8-oxo-DG immunolabeling) and PVI and their perineuronal net (PNN) in twelve established animal models relevant to autism (i.e., the fmr1 KO and CNV 15q13.3) and schizophrenia (CNV: 22q11, 15q13.3, 1q21, serine racemase (SR) KO, GRIN2A KO, Gclm KO) with or without additional insult (e.g., environmental: Gclm KO + GBR12909, GRIN2A KO + GBR12909, neonatal ventral hippocampal lesion (NVHL), methylazoxymethanol acetate developmental rodent model (MAM) and poly:IC).
**Results:** When PVI deficits in the anterior cingulate cortex were found in these animal models carrying genetic and/or environmental risks relevant to diverse etiological aspects of these disorders, oxidative stress was always present. Specifically, oxidative stress was negatively correlated with the integrity of PVIs and the extracellular perineuronal net enwrapping these interneurons. Oxidative stress may result from dysregulation of systems typically affected in schizophrenia, including glutamatergic, dopaminergic, immune, and antioxidant signaling. As convergent endpoint, redox dysregulation has successfully been targeted to protect PVIs with antioxidants/redox regulators across several animal models (e.g., Gclm KO, NVHL rats, GRIN2A KO and SR KO mice). D-serine, an allosteric modulator of brain NMDA receptor also protected PVIs and PNN against oxidative stress in SR KO mice.
**Discussion:** In view of the fact that the established pathophysiological processes dopamine excess, immune dysregulation and NMDA receptor hypofunction could all induce oxidative stress and are potentiated by additional oxidative insults, this mechanism could be central to damage of the highly metabolically active PVIs and the PNN surrounding them. Antioxidant systems are therefore potential therapeutic targets, assuming that redox regulators could be applied early, during environmental impacts, long before the clinical emergence of the disease.

### 3.3 DISTURBANCES IN NEURAL OSCILLATIONS, GLUTAMATE, AND GABA: EFFECTS OF KETAMINE AND COMPARISON TO SCHIZOPHRENIA

Lawrence Kegeles*[,1], Erin Stolz[2], Xiangling Mao[3], Najate Ojeil[1], Raphael Massuda[4], Mariana Pedrini[4], Maryam Bayatmokhtari[5], Mark Slifstein[6], Anissa Abi-Dargham[6], Matthew Milak[1], Carolyn Rodriguez[7], Chi-Ming Chen[2], Dikoma Shungu[3]
*[1]Columbia University & New York State Psychiatric Institute; [2]University of Connecticut, Storrs; [3]Weill Cornell Medical College; [4]Universidade Federal do Rio Grande do Sul; [5]Mount Sinai School of Medicine; [6]Stony Brook University School of Medicine; [7]Stanford University*

**Background:** In schizophrenia (SCH), proton MRS studies of the medial prefrontal cortex (MPFC) show elevated glutamine (Gln) or the combination of Glu and Gln (Glx) in unmedicated patients. Studies in healthy human subjects demonstrate ketamine-induced acute increases in MPFC Glu or Gln. Together, these findings raise the question of potential disturbances in excitation-inhibition balance in the illness, possibly arising from NMDA receptor deficits in GABAergic interneurons. We investigated these questions following acute ketamine administration by using repeated 15-minute MRS acquisitions of Glx and GABA with simultaneous EEG and comparing the results with the same modalities acquired in SCH.
**Methods:** We enrolled 11 healthy volunteers (age 18–55) who were given a constant i.v. infusion of ketamine 0.5 mg/kg over 40 min during a combined EEG and 1H MRS study. Glx and GABA were acquired in the pregenual MPFC using a 3T GE system and a J-edited PRESS sequence. Sequential MRS acquisitions each of 15 min duration (90 min total) were obtained before, during, and following the infusion. EEG was recorded using an MRI-compatible 64-channel system with direct current BrainAmp MR amplifiers (Brain Products GmbH). Post-ketamine EEG data were analyzed in frontal electrodes for gamma and delta alterations. EEG and MRS data were also acquired in 12 patients with SCH with these systems.
**Results:** Neurochemicals Glx and GABA showed acute increases within 15–30 minutes following the initiation of ketamine infusion, more pronounced for GABA (13% increase, $p = .04$ by paired t test). Gamma amplitude in left and right frontal electrodes increased in the first 15-minute average after initiation of ketamine ($p < .05$), with no evidence of earlier gamma decrease. Left delta amplitude decreased linearly following ketamine ($p < .01$). Peak GABA concentration correlated inversely with average left delta amplitude in the immediately subsequent 15-minute acquisition. Data in SCH showed similar elevations in GABA and gamma amplitude.
**Discussion:** These data show the feasibility of attaining time resolution of Glx and GABA changes in the several-minutes range with standard PRESS J-edited 1H MRS, and simultaneous sub-second resolution with EEG. There were no indications in these frontal electrodes of very early GABAergic inhibition leading to disinhibition of Glx, which may occur in other brain regions following ketamine administration. The GABA, Glx, and EEG alterations found here following ketamine administration are consistent with stable alterations reported in unmedicated patients with SCH and are compatible with an NMDA receptor deficit mechanism in the illness. They show homeostatic rebalancing at elevated levels as found in SCH itself. Excitation-inhibition rebalancing at abnormally elevated levels may pose a risk of neuronal damage that persists in untreated psychosis.

### 3.4 NEURAL OSCILLATIONS AND EXCITATION/INHIBITION BALANCE IN SCHIZOPHRENIA: A DEVELOPMENTAL PERSPECTIVE

Peter Uhlhaas*[,1]
*[1]Institute of Neuroscience & Psychology, University of Glasgow*

**Background:** Schizophrenia (ScZ) is a neurodevelopmental disorder that characteristically emerges during the transition from adolescence to adulthood. However, the mechanisms that underlie the expression of psychotic symptoms and cognitive deficits during this developmental period are still unclear. In my presentation, I will summarize data from EEG/MEG-work that has investigated the maturational changes in in neuronal dynamics during adolescence as well as the possibility that aberrant rhythmic activity is present in clinical high-risk participants.

**Methods:** A sample of participants meeting CHR-criteria (n=100) from the ongoing Youth Mental Health Risk and Resilience (YouR) Study and 50 matched controls were recruited as well as a sample of n = 20 participants meeting first-episode psychosis (FEP) criteria. We examined auditory and visual-induced oscillations as well as resting-state Magnetoencephalographical (MEG)-data and obtained estimates of spectral power and phase-synchronization at source-level. MEG-recordings were accompanied by Magnetic Resonance Spectroscopy (MRS) measurements of GABA and Glutamate-levels in auditory and visual cortices.

In addition, we examined the development of neural oscillations in a sample of n = 100 children and adolescents (age range: 12–21 years) during a working memory task and during spontaneous activity to identify critical periods for the development of neural dynamics.

**Results:** CHR-participants were significantly impaired in the generation of both auditory and visual gamma-band oscillations as well as characterized by an increase in broad-band, resting-state gamma-band power. The latter points towards an increase in excitability-levels of neural circuits which is supported by increased Glutamate-levels in sensory regions while GABA-levels were not different from controls. Similar patterns in both MEG- and MRS-parameters were observed in the FEP-group. Finally, our developmental data highlight that the transition from adolescence to adulthood is characterized by profound changes in both amplitude and synchrony dynamics, highlighting the possibility that critical period mechanisms that underlie the expression of psychosis are impaired in ScZ.

**Discussion:** Together, these data indicate that aberrant neural oscillations in ScZ highlight the crucial contribution of impaired neural dynamics that are likely to result from dysfunctional Excitation/Inhibition balance parameters. Moreover, the onset of schizophrenia during the transition from adolescence to adulthood suggests that critical period mechanisms that support the expression of high-frequency oscillations are impaired.

### 4. INNOVATIVE APPROACHES TO EARLY IDENTIFICATION AND TREATMENT: USING MOBILE HEALTH TECHNOLOGY TO IMPROVE OUTCOMES IN PSYCHOSIS

Laura Tully
*University of California, Davis*

**Overall Abstract:** Smartphone and internet based applications that promote symptom tracking, treatment engagement, and self-management have the potential to improve mental health outcomes and reduce cost of care. This is especially important in the treatment of psychosis, as long-term clinical outcomes to commonly available treatments remain poor and financial costs are high. The speakers in this symposium will present novel approaches using mobile health technology to promote rapid identification and referral (Dr. Niendam), access to treatment (Dr. Hidalgo-Mazzei), treatment engagement and symptom tracking (Dr. Tully), and functional recovery (Dr. Alvarez-Jimenez) for individuals experiencing psychosis.

Dr. Niendam will present initial results of a community-based cluster-randomized controlled trial aiming to increase identification rates of individuals with psychosis and reduce Duration of Untreated Psychosis. Twenty-two school, community, and primary care sites in Sacramento, California were randomized to either standard community education and clinician-based referral versus standard education plus electronic (tablet) screening for psychosis symptoms. Results show electronic screening is feasible across various community settings and significantly increases identification rates compared to clinician-based identification alone.

Dr. Hidalgo-Mazzei will present data examining the feasibility of delivering a psychoeducational treatment program that promotes self-management in bipolar disorder. The SIMPLe platform is accessible from any internet enabled device and provides symptom monitoring and personalized psychoeducation content in Spanish, Italian, and French. Data from a large open trial with over 300 participants from across the globe demonstrate how mobile technologies can increase access to care by extending effective interventions to many people at low cost.

Dr. Tully will present data on the feasibility, validity, and predictive utility of a consumer smartphone application ("app") plus provider web-based Dashboard as an add-on treatment tool in Early Psychosis outpatient programs in Northern California. Data demonstrate that consumers and providers in community-based outpatient clinics are responsive to integrating smartphone technology into treatment services. Consumers willingly use the app to track their symptoms; symptom data gathered via the app appears to be a valid reflection of symptoms experienced over time and can predict symptom exacerbations two weeks later.

Dr. Alvarez-Jimenez will present data demonstrating the feasibility, acceptability, and efficacy of two novel online social media based platforms designed to promote functional recovery in Ultra High Risk and First Episode Individuals. Results indicate online social media platforms are safe, engaging, and improve social functioning in both populations – a domain that is often neglected in most treatment approaches.

Chantel Garrett is the founder of Strong365.org, a website providing consumer and family-focused psychoeducation materials related to psychosis in 103 languages. She also has lived experience as a family member of a loved one with schizophrenia. As discussant, she will speak from her expertise as both a developer and consumer of internet-based and mobile technologies to elucidate how mobile health materials can impact provision of mental health care, and facilitate discussion of the barriers and future directions for the field. Implementation of technology-based care across diverse cultures and languages will also be discussed.

### 4.1 ENHANCING EARLY PSYCHOSIS TREATMENT USING SMARTPHONE TECHNOLOGY: INTEGRATION OF A MOBILE HEALTH PLATFORM IN FOUR EARLY PSYCHOSIS PROGRAMS

Laura Tully*[,1], Ana-Maria Iosif[1], Lauren Zakskorn[1], Divya Kumar[1], Kathleen Nye[1], Aqsa Zia[1], Jennifer Denton[1], Katherine Pierce[1], Taylor Fedechko[1], Tara Niendam[1]
[1]*University of California, Davis*

**Background:** Mobile health applications offer ecologically valid, data-rich methods of modeling daily symptoms and functioning, which could inform treatment delivery and facilitate early intervention in individuals with psychosis. To date, most studies evaluate adoption of technology independent of care providers. However, successful implementation and long-term adoption of mobile technology likely also requires integration into outpatient settings as an add-on tool to enhance treatment. We implemented a smartphone "app" plus clinician Dashboard as an add-on treatment tool in the UC Davis Early Psychosis (EP) Programs and tested feasibility, validity, and predictive utility of symptom tracking via the app as part of EP care. A subsequent pilot study examined barriers to implementation within two additional community outpatient settings in Northern California.

**Methods:** Study 1 implemented the platform within the UC Davis EP Programs. For up to 14 months, EP clients completed daily and weekly surveys examining mood, symptoms, and treatment relevant factors via the app, as well as monthly in-person clinical assessments using the BPRS. Clinicians discussed symptom ratings and surveys during treatment sessions using the Dashboard. We examined client enrollment and survey completion to determine feasibility, and relationships between BPRS and weekly symptom ratings to evaluate validity of self-report symptom data collected via the app. Analysis of predictive utility determined if weekly self-report symptoms predicted symptom exacerbations 2 weeks later. Study 2 expanded recruitment to 2 additional community-based EP outpatient clinics. EP clients and their clinicians used the platform as part of care for 5 months and filled out satisfaction surveys at study-end regarding usability of the platform. Rate of survey completion in the absence of financial incentives was examined to determine real-world implementation of the platform.
**Results:** For study 1, 76 clients enrolled and remained in the study for an average of 183 days (SD=88). Survey completion rates remained high over the course of the study (weekly surveys: 77%; daily surveys: 69%) and were not significantly impacted by baseline symptom severity or length of time in the study. Weekly survey positive and depression/anxiety symptoms were significantly associated with BPRS positive (p<0.001) and BPRS depression/anxiety symptoms (p< 0.001) respectively. EP clients reported high satisfaction with the platform and endorsed continue use of the app if it was made available as part of their treatment. For Study 2, 61 EP clients and 20 clinicians enrolled; 41 EP clients and 20 clinicians participated for 5 months. The majority of EP clients (66%) and clinicians (85%) who completed satisfaction surveys reported a desire to continue to use the platform as part of care. Six (15%) clients and 3 providers (23%) stated that technological glitches impeded their use of the platform.
**Discussion:** These data support the validity and acceptability of implementing smartphone-based assessment of symptoms in community-based EP care. Specifically, results indicate that assessing positive and depression/anxiety symptoms using weekly self-report surveys via smartphone is comparable to gold-standard clinician-led assessments. This approach may be a valid method of monitoring fluctuations in positive and depression/anxiety symptoms in EP populations to anticipate symptom exacerbations. However, solutions to logistical barriers such as technical challenges and clinician engagement with technology are necessary for widespread adoption across EP care.

### 4.2 A TECHNOLOGY-ENHANCED INTERVENTION TO REDUCE THE DURATION OF UNTREATED PSYCHOSIS THROUGH RAPID IDENTIFICATION & ENGAGEMENT

Tara Niendam*,[1], Rachel Loewy[2], Mark Savill[2], Monet Meyer[1], Adi Rosenthal[1], Kevin Delucchi[2], Tyler Lesh[1], Haley Skymba[1], J. Daniel Ragland[1], Howard Goldman[3], Rosemary Cress[1], Richard Kravitz[1], Cameron Carter[1]
*[1]University of California, Davis School of Medicine; [2]Weill Institute for Neurosciences, University of California, San Francisco; [3]University of Maryland School of Medicine*

**Background:** Reducing the duration of untreated psychosis (DUP) is essential to improve long-term outcome in young people with first episode of psychosis (FEP). The US "standard of FEP care" focuses on targeted provider education regarding FEP signs and symptoms to motivate referrals to FEP coordinated specialty care (CSC) services. However, a recent US multisite CSC trial showed a median DUP of 74.5 weeks, suggesting the current approach to engage referral sources is not sufficient to reduce DUP to proposed international standards of 12 weeks. This cluster-randomized controlled trial assesses whether standard targeted provider education plus novel technology-enhanced screening using the Prodromal Questionnaire-Brief version (PQ-B) identifies more individuals with FEP, earlier in their illness, compared to standard targeted provider education alone.
**Methods:** Twenty-two sites were randomized within 3 strata [community mental health, CMH (N=10), middle/high schools, SCH (N=8), primary care, PC (N=4)] to 1 of 2 intervention arms [Education alone (TAU) vs Education + Electronic Screening (Active)]. Active sites screened eligible individuals ages 12–30 at initial presentation for mental health concerns and referred those who passed a liberal PQ-B cut off score for phone evaluation by the CSC clinic. TAU sites referred individuals for phone evaluation based on clinician judgment. Phone evaluations assessed eligibility for FEP services and DUP. Preliminary analyses examined the number of FEP referrals and length of DUP in each arm.
**Results:** Active sites effectively implemented electronic screening within their settings. Of the 822 individuals electronically screened at Active sites between June 2015 and July 2017, 43.2% scored above the PQ-B cutoff (mean±SD PQ-B score=21.25 ± 20.75; median=15; range = 0–95; IQR = 3–35). One in 8 individuals who completed the tablet were identified as experiencing threshold psychosis. Across both Active and TAU sites, 511 individuals were identified, 422 individuals agreed to be referred, and 319 completed a phone interview to determine eligibility: 33.23% reported attenuated and 36.68% fully psychotic symptoms. Active sites identified significantly more individuals with threshold psychosis (p<.001) than TAU. No difference in median days of DUP was observed across arms.
**Discussion:** Preliminary results show the feasibility of electronic screening across various community settings and showed a 3.5 times higher identification rate for electronic screening of self-reported psychosis spectrum symptoms than clinician-based identification alone. Reasons for the lack of difference in DUP will be discussed. While the screening method may shorten the time from entry into mental health care and referral to specialty care treatment, significant DUP reduction may require interventions to reduce time to the first mental health contact. The next phase of the project will examine impact of clinic-based versus community-based treatment engagement to reduce barriers to initiating CSC care.

### 4.3 ENHANCING SOCIAL FUNCTIONING AND LONG-TERM RECOVERY IN YOUNG PEOPLE WITH FIRST EPISODE PSYCHOSIS (FEP) AND YOUNG PEOPLE AT ULTRA HIGH RISK (UHR) FOR PSYCHOSIS: A NOVEL ONLINE SOCIAL THERAPY APPROACH

Mario Alvarez-Jimenez*,[1], John F Gleeson[2], Simon Rice[1], Sarah Bendall[1], Simon D'Alfonso[3], Dina Eleftheriadis[1], Daniela Cagliarini[1], Penni Russon[1], Patrick D. McGorry[1], Barnaby Nelson[1]
*[1]Orygen, The National Centre of Excellence in Youth Mental Health, The University of Melbourne; [2]Australian Catholic University, School of Psychology; [3]The University of Melbourne*

**Background:** Specialized early intervention services have demonstrated improved outcomes in first episode psychosis (FEP); however, functional recovery lags behind symptomatic remission, and many FEP patients remain socially isolated with poor functional outcomes. Similarly, psychological and pharmacological treatments have been demonstrated to reduce rates of transition to psychosis in Ultra High Risk (UHR) patients. However, recent research shows that UHR patients have a poor functional outcome regardless of transition to psychosis. These findings have resulted in widespread calls for new treatments aimed at improving functioning in both FEP and UHR patients.
The aim of these studies was to determine the safety, acceptability, feasibility and treatment effects of an advanced online social media based intervention specifically designed to enhance social functioning in FEP and UHR patients.
**Methods:** Our multi-disciplinary team of 35 researchers, software engineers, professional writers, clinical psychologists, comic developers, experts in human-computer interaction and young people has developed novel online social media platforms for young people with FEP (Horyzons), and UHR patients (Momentum). Our interventions integrate: i) peer-to-peer social networking, ii) tailored therapeutic interventions, iii) expert and peer-moderation, and iv) new models of psychological therapy (strengths-based models, self-compassion and mindfulness). The acceptability and safety of these platforms have been evaluated through 2 pilot studies in FEP (N=20; 1 month intervention), and UHR (N=15; 2 months intervention). In addition, the effectiveness of Horyzons is currently being evaluated in a large 5 year RCT in FEP (N=170; 18 months intervention).

**Results:** UHR pilot: System usage was high, with a total 270 logins (18/user), 749 posts (58/user), 170 therapy modules completed (12/user), and 67% of users being actively engaged over the trial. All participants reported a positive experience using Momentum and would recommend it to others. 93% considered Momentum to be helpful. Analysis revealed a significant increase in social functioning ($p<0.001$; $d=2.39$) and satisfaction with life ($p=0.03$; $d=0.48$) at follow-up. There was a significant increase in therapy mechanisms directly targeted by Momentum including strengths usage ($p=0.03$; $d=0.46$), mindfulness skills ($p=0.04$; $d=0.36$) and components of social support. There were significant correlations between system usage and improvements in social functioning ($r=0.63$ $p=0.02$), social support ($r=0.62$ $p=0.02$) and strengths usage ($r=0.51$ $p=0.06$).
FEP pilot: system use was high. The majority of FEP participants reported feeling safe (100%) and more socially connected (60%) using Horyzons. There was a significant reduction ($d=0.60$; $p=0.03$) in depressive symptoms at follow-up.
FEP RCT: Horyzons' safety outcomes have been consistently strong. System usage is being high, with an average 101 logins, 70 posts, and 11 therapy modules per user, and 60% of users being engaged with the online system for a period of 18 months.
**Discussion:** Horyzons and Momentum are the first online interventions designed to improve functional outcomes in FEP and UHR patients. Momentum is engaging, safe, may improve social functioning and satisfaction with life in UHR patients and appeared to specifically improve therapeutic mechanisms directly targeted by the online intervention. Horyzons is safe and engaging (over prolonged periods of time) and may improve depression and social connectedness in FEP patients.

### 4.4 INTERNET-BASED INTERVENTIONS FOR BIPOLAR DISORDER

Diego Hidalgo-Mazzei*[,1], Ainoa Mateu[2], Maria Reinares[3], Francesc Colom[4], Eduard Vieta[3]
*[1]Hospital Clinic de Barcelona; [2]Centre for Psychiatry, Imperial College London; [3]Institute of Neuroscience, Hospital Clinic, University of Barcelona, IDIBAPS, CIBERSAM; [4]Mental Health Group, IMIM-Hospital del Mar, CIBERSAM*

**Background:** Adjunctive psychological interventions as an add-on to pharmacological treatment in serious mental illnesses have shown to further improve long-term outcome, especially in the case of Bipolar Disorder and first episode psychosis. Among them, psychoeducational programs have a well-established evidence of efficacy and cost-efficiency. However, there are several limitations restricting the broad implementation of these psychological treatments, out of which the most important one is related to a tremendous gap between availability and demand. Therefore, there is an emerging interest to explore new approaches to deliver this kind of treatments tailored to individual needs and in a continuous way (e.g. all year long) from any location while maintaining their efficacy at a low cost. The high availability of Internet connected devices as well as it's user-friendly interfaces could be a potential and feasible window to expand and extend psychoeducational programs in Bipolar disorder and other serious mental illnesses. The main objective of this presentation will be: 1. to review the available internet-based psychological interventions for bipolar disorder, 2. to present the SIMPLe project development, studies protocols, results from a feasibility study and an open study, and finally, 3. to provide some insights and perspectives into the future of the field.
**Methods:** A systematic-review of the literature was undertook to review the available internet-based psychological interventions for bipolar disorder and provide a critical appraisal of the studies and platforms included. A feasibility pilot study was conducted to test the first version of the SIMPLe app in which retention, acceptability and satisfaction were assessed in a group of subsequent samples of bipolar patients using the app, pre and post intervention questionnaires and assessments were conducted during face to face interviews. Regarding the open trial (i.e. OpenSIMPLe), a similar approach was adopted, but involving patients from all around the world and using online questionnaires.
**Results:** During the systematic review we identified over 251 potential entries matching the search criteria and after a thorough manual review, 29 publications pertaining to 12 different projects, specifically focusing on psychological interventions for bipolar patients through diverse Internet-based methods, were selected. In the feasibility study, 51 participants were initially enrolled in the study, 36 (74%) remained actively using the application after 3 months. The whole sample interacted with the application a mean of 77 days (SD=26.2). Over 86% of the participants agreed that the experience using the application was satisfactory. So far, the OpenSIMPLe trial have enrolled more than 300 participants, preliminary results show levels of satisfaction beyond 80%, although a retention of only 5% after 6 months was calculated from servers registries.
**Discussion:** Considering the high rates of retention and compliance reported, they represent a potential highly feasible and acceptable method of delivering this kind of interventions to bipolar patients. The results of the feasibility study confirms that this particular intervention is feasible and represent a satisfactory and acceptable instrument for the self-management of bipolar disorder as an add-on to the usual treatment but future clinical trials must still probe its efficacy. Moreover, preliminary results from the OpenSIMPLe study shows that is feasible to extend this intervention to many people at a low cost. Present and future technologies employing passive data collection and weareables could improve the personalization and accuracy of these interventions.

### 5. RETHINKING THE TAXONOMY, COURSE, AND OUTCOME OF PSYCHOSES: DIMENSIONAL, LATENT TRAJECTORY, AND TRANSDIAGNOSTIC APPROACHES

William T. Carpenter, Jr.
*University of Maryland School of Medicine*

**Overall Abstract:** Evidence continues to accumulate on heterogeneity in phenomenology, course and outcome of non-affective and affective psychotic disorders. Both DSM and ICD classification systems have evolved to include a large number of categories of psychosis. However, doubt remains about this categorical approach because of high comorbidity, common etiological factors and the absence of zones of relative rarity between categorical diagnoses. Some authors have nevertheless argued that categorical representations of psychosis may still be of clinical utility if used in combination with dimensional indicators.
It is now widely accepted that psychotic symptoms partition into several symptom dimensions that would support the heterogeneity of psychotic disorders. However, there is no consensus on the exact number of these dimensions, with previous factor-analytic work pointing towards models with two to twelve specific symptom dimensions. However, recently, there has been evidence for a transdiagnostic dimension underlying affective and non-affective psychotic symptoms in schizophrenia, schizoaffective and bipolar disorder that challenges their classification as distinct diagnostic constructs. There is also considerable heterogeneity in clinical course and outcome of psychotic disorders, but how to best map and model this over time remains to be established. Taken together, this presents significant challenges for the classification of psychotic disorders as separate diagnostic entities.
This symposium brings together international researchers at the forefront of research into the phenomenology, course and outcome of psychotic disorders. Roman Kotov will present novel data on symptom dimensions and examines the course of these dimensions in an epidemiologic cohort of 628 first-admission inpatients with psychosis interviewed 6 times over two decades in the Suffolk County Mental Health Project. Craig Morgan will report new findings from the 10-year follow-up of the Aetiology and Ethnicity in Schizophrenia and Other Psychoses (AESOP-10) study, an epidemiological cohort of 552 patients with a first episode psychosis, using a data driven approach to identify latent trajectory classes to account for heterogeneity in patterns of change in psychotic symptoms over time and characterize these trajectories with the WHO classification, baseline demographic characteristics and diagnoses. Ulrich Reininghaus will present novel data from the Bipolar-Schizophrenia Network on Intermediate Phenotypes (B-SNIP) consortium to investigate whether there is a transdiagnostic dimension cutting across symptoms of schizophrenia, schizoaffective disorder and psychotic bipolar I disorder. Diego Quattrone will report recent findings from EU-GEI Functional Enviromics Study on genetic and socio-environmental factors associated with transdiagnostic and specific symptom dimensions of non-affective and affective psychosis. Robin Murray will discuss these findings in the context of new challenges in the field and directions for future research.

### 5.1 DIMENSIONS OF PSYCHOSIS AND THEIR TRAJECTORIES DURING TWO DECADES AFTER FIRST HOSPITALIZATION

Roman Kotov*[,1]
*[1]Stony Brook University*

**Background:** Heterogeneity of psychosis presents significant challenges for classification. Between two and 12 symptom dimensions have been proposed, and consensus is lacking. The present study sought to identify uniquely informative models by comparing the validity of these alternatives. A critical validator is future course, and we examined trajectory of each dimension.
**Methods:** We investigated this question in the first U.S. study to follow an epidemiological cohort with psychotic disorders for 20 years after first hospitalization. Participants were assessed in person 6 times over 2 decades on Global Assessment of Functioning (GAF), psychotic symptoms, and mood symptoms, and 373 completed 20-year follow-up (68% of survivors) including an electrophysiological assessment of error processing. We first analyzed a comprehensive set of 49 symptoms rated by interviewers at baseline, progressively extracting from one to 12 factors. Next, we compared the ability of resulting factor solutions to (a) account for concurrent neural dysfunction and (b) predict 20-year role, social, residential, and global functioning, and life satisfaction.
**Results:** A four-factor model showed incremental validity with all outcomes, and more complex models did not improve explanatory power. The four dimensions—reality distortion, disorganization, inexpressivity, and apathy/asociality—were replicable in 5 follow-ups, internally consistent, stable across assessments, and showed strong discriminant validity. On all of these measures schizophrenia exhibited a decline that began between years 5 and 10. Correspondingly, GAF scores dropped from 49 (Year 4) to 36 (Year 20). Neither aging nor changes in antipsychotic treatment accounted for the declines.
**Discussion:** These results reaffirm the value of separating disorganization and reality distortion, are consistent with recent findings distinguishing inexpressivity and apathy/asociality, and suggest that these four dimensions are fundamental to understanding neural abnormalities and long-term outcomes in psychosis. They also revealed a substantial symptom burden across psychotic disorders that increased with time and ultimately may undo initial treatment gains. Additional research is needed, but previous studies suggest sociocultural factors and different care models may preempt this decline.

### 5.2 RETHINKING THE COURSE OF PSYCHOTIC DISORDERS: IDENTIFYING LATENT TRAJECTORIES

Craig Morgan*[,1], Paola Dazzan[1], Julia Lappin[2], Margaret Heslin[1], Gillian Doody[3], Robin Murray[1], Peter B. Jones[4], Ulrich Reininghaus[5]
*[1]Institute of Psychiatry, Psychology & Neuroscience, King's College London; [2]University of New South Wales; [3]University of Nottingham; [4]University of Cambridge; [5]Maastricht University*

**Background:** The clinical course of psychotic disorders is highly variable. Typically, researchers have captured course types using broad categories, e.g. the WHO instruments to assess course and outcome distinguish three categories: episodic (i.e., no episode > 6 months), continuous (i.e., no remission > 6 months), and neither (i.e., an episode and a remission > 6 months). However, whether these adequately capture symptom trajectories of psychotic disorders has not been assessed. Using AESOP-10 data, we sought to identify classes of individuals with specific symptom trajectories over a 10 year follow up and to, then, compare trajectories with WHO categories and examine associations between trajectories and baseline demographic characteristics and diagnoses.
**Methods:** AESOP-10 is a follow-up, at 10 years, of a cohort of 552 patients with a first episode psychosis identified in south-east London and Nottingham, UK. At follow-up, we collated detailed information on clinical and social course and outcome. This included collating extensive information on month by month fluctuations in presence of psychotic symptoms. Using this data, we fitted growth mixture models to identify latent trajectory classes that accounted for heterogeneity in patterns of change in psychotic symptoms over time.
**Results:** We had sufficient data on occurrence of psychotic symptoms throughout the follow up on 326 (~ 60%) patients.
A four-class quadratic growth mixture model best fit the data, with four trajectories defined by variations in the mean number of months psychotic per year during the follow-up period: (1) low and reducing [intermittent] (58.5%); (2) persistently high [persistent] (30.6%); (3) high, followed by gradual reduction [late improvement] (5.6%); and (4) intermediate, followed by gradual increase [late decline] (5.4%). When compared with the usual classification of course types (episodic, continuous, neither), the intermittent class included all those in the episodic category (n 94 of 94; 100%) and the persistent class included almost all those in the continuous category (n 72 of 78 persistent; 92%). A majority of those in the neither category had an intermittent trajectory (i.e., n 90 of 145; 62%), with the remainder spread across the other three classes (i.e., n 25, 17% persistent; n 14, 10% late decline; n 16, 11% late improvement).
Compared with those with an intermittent trajectory, patients with a persistent trajectory were less often women (OR 0.6, 95% CI 0.4–0.9), more often of black Caribbean ethnicity (OR 2.3, 95% CI 1.2–4.1), and less often had a diagnosis of affective psychosis (OR 0.2, 95% CI 0.1–0.4). There were no differences by age. Numbers were small, but there were indications that those with a late decline trajectory more closely resembled those with a persistent trajectory (i.e., less often women, more often of black Caribbean ethnicity, less often diagnosis of affective psychosis) than did those with a late improvement trajectory.
**Discussion:** Our current approach to classifying course of psychotic disorders may be flawed, particularly in specifying a group as neither episodic nor continuous. Our findings suggest this group is heterogeneous and includes patients whose outcomes more closely resemble one of the two main trajectories, intermittent or persistent. Only a small proportion of patients fit neither. These patients constitute clinically important sub-groups whose trajectories appear to change, either from an initially positive or initially negative course, some years after first contact with mental health services. Our failure to fully characterise trajectories of psychosis may confound efforts to elucidate predictors of long-term outcome.

### 5.3 EVIDENCE ON A TRANSDIAGNOSTIC PSYCHOSIS SPECTRUM OF SCHIZOPHRENIA, SCHIZOAFFECTIVE AND PSYCHOTIC BIPOLAR DISORDER IN THE BIPOLAR-SCHIZOPHRENIA NETWORK ON INTERMEDIATE PHENOTYPES (B-SNIP)

Ulrich Reininghaus*[,1], Jan Boehnke[2], UnYoung Chavez-Baldini[1], Brett Clementz[3], Godfrey Pearlson[4], Matcheri Keshavan[5], John Sweeney[6], Carol Tamminga[7]

[1]*Maastricht University;* [2]*University of Dundee;* [3]*University of Georgia;* [4]*Yale University;* [5]*Harvard University;* [6]*University of Texas Southwestern;* [7]*The University of Texas Southwestern Medical Center*

**Background:** The validity of the classification of non-affective and affective psychoses as distinct entities has recently been disputed in light of calls for a dimensional and transdiagnostic approach to diagnostic classification and evidence on shared aetiological factors. Despite the shifts in view, there remains a dearth of empirical efforts to clarify and identify a transdiagnostic spectrum of psychosis. Our recent research has demonstrated evidence for a transdiagnostic psychosis spectrum as detailed in a bifactor model with one transdiagnostic symptom dimension and five specific symptom dimensions of positive symptoms, negative symptoms, disorganization, mania, and depression in patients with schizophrenia, schizoaffective and bipolar disorder. The aim of the current study was to investigate whether there is a transdiagnostic dimension cutting across symptoms of schizophrenia, schizoaffective disorder and psychotic bipolar I disorder using widely established measures for assessing psychosis, mania and depression in the large multi-centre Bipolar-Schizophrenia Network on Intermediate Phenotypes (B-SNIP) consortium in the United States.
**Methods:** This study analysed data from the B-SNIP Phenotyping Consortium, which included 933 patients with a diagnosis of schizophrenia (n=397), schizoaffective disorder (n=224), and bipolar disorder (n=312). Multidimensional item-response modelling was conducted on symptom ratings of the Positive and Negative Syndrome Scale (PANSS), the Young Mania Rating Scale (YMRS), and the Montgomery-Åsberg Depression Rating Scale (MADRS) using the mirt package of the R environment.
**Results:** A bifactor model with 1 transdiagnostic symptom dimension and 5 specific symptom dimensions of positive symptoms, negative symptoms, cognitive disorganization, mania, and depression best matched the B-SNIP sample data. The bifactor model with 1 transdiagnostic factor and 5 specific factors based on the PANSS 5-factor solution by Emsley et al. (2003) provided the best model fit (AIC=53209.8, BIC=53920.0, aBIC=53443.7), as compared with a unidimensional model (AIC=55583.1, BIC=56151.3, aBIC=55770.2), a pentagonal model based on the PANSS 5-factor solution by Emsley et al.3 (AIC=53452.6, BIC=54068.1, aBIC=53655.3) as well as pentagonal and bifactor models of other previously reported factor solutions. When we extended analyses to include YMRS and MADRS, again, the bifactor model with 1 transdiagnostic factor and 5 specific factors, again, provided the best model fit.
**Discussion:** Consistent with our previous findings, this study provides evidence on a transdiagnostic symptom dimension that cuts across traditional diagnostic boundaries of schizophrenia, schizoaffective disorder and psychotic bipolar disorder using three widely established measures for assessing psychosis, mania and depression. The best-fitting, bifactor model also included 5 specific symptom dimensions based on the PANSS 5-factor solution by Emsley et al. (2003), which reflects a direct replication of our previous findings on the dimensionality of the PANSS. Overall, our findings lend further support to a transdiagnostic psychosis spectrum encompassing schizophrenia, schizoaffective and bipolar disorder as we have previously proposed.

### 5.4 BIOLOGICAL AND EPIDEMIOLOGICAL EXAMINATION OF TRANSDIAGNOSTIC AND SPECIFIC SYMPTOM DIMENSIONS AT PSYCHOSIS ONSET: FINDINGS FROM THE EUGEI STUDY

Diego Quattrone*[,1], Pak Sham[2], Evangelos Vassos[1], Charlotte Gayer-Anderson[1], Giada Tripoli[1], Laura Ferraro[3], Michael O'Donovan[4], Alex Richards[4], Jim van Os[5], Craig Morgan[1], Ulrich Reininghaus[5], Robin Murray[1], Marta Di Forti[1], Cathryn Lewis[1]
[1]*Institute of Psychiatry, Psychology & Neuroscience, King's College London;* [2]*Centre for Genomic Sciences, The University of Hong Kong;* [3]*University of Palermo;* [4]*MRC Centre for Neuropsychiatric Genetics and Genomics, Cardiff University;* [5]*School of Mental Health and Neuroscience, Maastricht University*

**Background:** Current diagnostic models of psychosis have been questioned since Kraepelin's original dichotomy of dementia praecox and manic depression. Indeed, increasing evidence has suggested that a dimensional approach might be a valid alternative platform for research. However, while an increasing number of studies have investigated how environmental risk factors for affective and non-affective psychosis map onto symptom dimensions, only a few have examined these dimensions in relation to genetic variants as summarised by Polygenic Risk Score (PRS). Furthermore, no studies have examined the putative effect of PRS for Schizophrenia (SZ), Bipolar Disorder (BP), and Major Depressive Disorder (MDD) on previously identified general and specific symptom dimensions. At the same time, only one study has investigated how symptoms vary according to epidemiological factors such as living in urban neighbourhoods. The objectives of this study were to: 1) test whether a bi-factor model statistically fits the conceptualization of psychosis as composed of general and specific dimensions; 2) examine the extent to which SZ, BP, and MDD PRSs explain the phenotypic variance due to general and specific dimensions; 3) test the hypothesis that the general psychosis dimension would be more severe in highly urban environments.
**Methods:** We used clinical and epidemiological data from the EUropean network of national schizophrenia networks studying Gene-Environment Interactions (EUGEI) study, including 2322 First Episode Psychosis (FEP) patients recruited in 17 sites across 6 countries. Genetic variants were collectively analyzed for 800 individuals.
The following analysis steps were performed:

1) Psychopathology items were analysed using multidimensional item response modelling in MPlus to estimate unidimensional, multidimensional, and bi-factor models of psychosis. Model fit statistics included Log-Likelihood, and Akaike and Bayesian Information Criteria to compare these models.
2) SZ, BP, and MDD PRSs for general and specific dimensions were built using PRSice. Summary statistics from large case-control mega-analyses from the Psychiatric Genomics Consortium were used as base data sets and general and specific dimension scores were used as discovery data sets. Individuals' number of risk alleles in the discovery sample was weighted by the log odds ratio from the base samples, accounting for population stratification, and summed into the three PRSs.
3) Multilevel regression analysis was used in STATA 14 to examine the variance in general dimension due to the population density levels across the sites.

**Results:** A bi-factor solution, composed of one general and five specific symptom dimensions, showed the best model fit statistics.
Higher SZ PRS score was associated with higher scores on positive dimensions ($\beta$= 0.27, t=2.11, p<0.05); higher BP PRS was associated with higher scores on mania dimension ($\beta$= 0.17, t=2.11, p<0.05); higher MDD PRS was associated with lower scores on negative dimension ($\beta$= -0.31, t=-2.25, p<0.05). No trends of association were found for SZ, BP, or MDD PRSs and the general psychosis dimension.
The transdiagnostic symptom dimension score was elevated in people living in more densely populated sites ($\eta 2$=0.077, 95% CI 0.057–0.098).
**Discussion:** Our results suggest that a) symptom dimension structure at FEP is best represented by the bi-factor model; b) in FEP patients, there is a trend of associations between SZ PRS and positive dimension, and between BP PRS and mania dimension; and c) elevated level of transdiagnostic symptomatology was observed in more densely populated sites.

### 6. FACT OR ARTIFACT? BENEFITS AND LIMITATIONS OF ADVANCED NEUROIMAGING METHODS FOR PSYCHOSIS

Ofer Pasternak
*Harvard Medical School*

**Overall Abstract:** Neuroimaging tools introduced the ability to non-invasively search for pathological signatures in the brains of subjects suffering from psychosis. In fact, with almost any neuroimaging modality there are studies that report the identification of abnormalities in the brains of schizophrenia subjects. The abundance of findings made it much clearer that brain abnormalities are common and expected in mental disorders. However, the quantity of findings, and especially their inconsistence across studies also raise questions as to the source of these abnormalities. Are they signifying a complicated range of pathologies and interactions? Or do they reflect auxiliary changes that are not directly related to the root, or the etiology of the disorder?
In addition, these inconsistencies raise technical questions such as are our tools sufficiently sensitive to reliably identify brain abnormalities in psychosis? And, to what extent are our tools sensitive to misinterpretation and to artifacts, which may explain some of the group differences found when comparing psychotic populations with controls? Emerging imaging modalities attempt to address these concerns by improving the specificity, i.e., the ability to relate identified abnormalities with underlying pathologies. At the same time analysis must carefully pay attention to common physiological sources of artifacts, such as subject motion, blood flow, brain metabolism, partial volume, etc.
This symposium will bring together five leading neuroimagers from 4 continents, who will present paradigm-shifting state of the art in their field, while providing critical cautionary remarks regarding the shortcomings of these methods, as well as recommendations for proper use in the context of psychosis studies. Speakers are:

1) Prof. Jennifer Caughlin, M.D. Department of Psychiatry and Behavioral Sciences, Johns Hopkins University. She will present state of the art in TSPO-PET acquisition and analysis, and its potential for the evaluation of neuroinflammation in schizophrenia.
2) Prof. Ofer Pasternak, Ph.D. Departments of Psychiatry and Radiology, Harvard Medical School. He will present recent advances in diffusion MRI, and how microstructural imaging may inform the study of psychosis.
3) Prof. Christoph Mulert, M.D. Department of Psychiatry and Psychotherapy. University medical center Hamburg, Germany. He will present new approaches for EEG acquisitions combined with fMRI that may shed light on neuronal activity.
4) Prof. Helen Juan Zhou, Ph.D. Neuroscience & Behavioural Disorders Programme, Duke-Nus Medical School, Singapore. She will present the emerging field of connectomics and its potential application to study functional and structural brain networks in schizophrenia.

The discussant will be Prof. Christos Pantelis, M.D. Department of Psychiatry, University of Melbourne, Australia. Dr. Pantelis will complement the panel by bringing in a more clinical point of view, informed of the needs in psychiatric neuroimaging. The panel will also benefit from his vast experience across all imaging modalities.
This symposium is designed to provide information and considerations that can be essential for psychosis researchers before considering the application of advanced imaging tools. We will describe the main and unique findings that were provided by each modality, towards a discussion of how far are imaging studies from leading to a breakthrough in the understanding of the pathophysiology of psychosis or its treatment.

### 6.1 STUDY OF ALTERED NEUROIMMUNITY IN PSYCHOSIS USING PET-BASED IMAGING OF THE TRANSLOCATOR PROTEIN 18 KDA: PROMISES, PITFALLS, AND FUTURE DIRECTIONS

Jennifer Coughlin*[,1], Tina Notter[2], Yong Du[3], Martin Pomper[3], Akira Sawa[3], Urs Meyer[2]
*[1]Johns Hopkins University; [2]University of Zurich; [3]Johns Hopkins University School of Medicine*

**Background:** Successful development of high affinity radioligands for the translocator protein 18 KDa (TSPO) has contributed to a rapid rise in their use with positron emission tomography (PET) imaging to quantitatively detect the higher density of TSPO in neuropsychiatric conditions with putative microglial activation or reactive gliosis in vivo. [11C]PK11195 has been widely used to study TSPO in many neurological and psychiatric diseases, but the quality of quantified binding estimates using this first-generation radioligand is hampered by low signal to noise ratio, which also limits the sensitivity to detect group differences in binding. Second-generation radiotracers for TSPO such as [11C]DPA-713, [11C]PBR28, or [18F]FEPPA have superior specificity for the target and improved brain penetrance. However, in spite of these promising newer generation radioligands, their use with PET neuroimaging to study the immune response in psychotic diseases like schizophrenia has yielded inconsistent results of low, unchanged, or even tendency toward decreased binding to TSPO compared to data from controls.
**Methods:** In this presentation, we will provide necessary biological and methodological perspective to help interpret better the recent results from imaging TSPO in psychosis. We will first review its expression by many cell types including activated microglia, the resident immune cells in the brain, and its diverse functional roles including TSPO as a biomarker of classic neuroinflammatory processes. We will then present optimized methods for estimating useful binding outcomes that go beyond correction for TSPO genotype to minimize effects of factors, including those related to the diverse roles of TSPO, which otherwise introduce limiting, inter-individual variability in binding.
**Results:** These methods include the reporting of relative binding in one tissue to another, where such global factors are cancelled out by their appearance in the numerator and denominator of the outcome ratio. Use of this ratio approach may decrease inter-individual variability in binding measures and improve the sensitivity and statistical power to detect differences between cohorts. In contrast, use of a relative outcome measure may limit the utility of TSPO imaging since a difference between cohorts or within a subject over time may reflect either abnormal TSPO density or a mere shift in possibly normal, relative distribution between the two tissue regions. The most useful pseudoreference region is therefore one in which the true regional density of TSPO is unchanged in the study population, and is yet unidentified in schizophrenia. Building on this biology and methodology, we discuss misconceptions about imaging TSPO in psychosis and cautiously remind the field that this technique should not be equated with 'imaging microglial activation' or 'imaging neuroinflammation.' Indeed, PET-based TSPO estimates have not correlated with increased peripheral or central pro-inflammatory cytokine levels in schizophrenia or other psychiatric diseases like major depressive disorder.
**Discussion:** Together, a less simplified approach to imaging TSPO may inform its utility in studying other biological processes captured by its use in psychosis, and may guide future, complimentary research in vitro and in vivo to enhance our understanding of altered neuroimmune processes in psychosis.

### 6.2 MICROSTRUCTURAL IMAGING WITH ADVANCED DIFFUSION MRI METHODS – WHAT IS GAINED AND WHAT IS LOST?

Ofer Pasternak*,[1]
[1]*Harvard Medical School*

**Background:** Diffusion MRI is one of the important technological advances that played a crucial role in the discovery of abnormalities related to schizophrenia. While other imaging modalities focused on volumetric changes, and on functional changes (e.g., metabolic, and vascular) diffusion MRI introduced the ability to study microstructural changes. Most diffusion MRI studies focused especially on the white matter, where the diffusion tensor imaging (DTI) analysis, which by now is considered widely available and conventional, provided unique microstructural contrasts. The most important parameter has been the fractional anisotropy (FA), which was perceived as a white matter integrity measure, and sometimes as a myelin integrity measure. The simplicity of the DTI model, and its growing availability on clinical scanners resulted with a considerable body of work demonstrating reduced FA in schizophrenia, leading to new clinical hypotheses, suggesting that mental disorders may involve white matter deficiency, which in turn would lead to mis-wiring, and connectivity issues that may explain some of the unusual symptoms associated with schizophrenia. However, even though DTI measures, and specifically FA, appear to be very sensitive to subtle brain changes, these measures are not specific to any pathology. In fact, while clinical studies attempted to relate DTI measures with white matter and myelin integrity, methodological studies provided clearer evidence that such a relationship is not warranted, since DTI measures could be affected by multitude of sources. This methodological complication raised the need for more advanced microstructural imaging, which could provide superior specificity to underlying pathologies, and especially to pathologies that are related to white matter integrity and connectivity.
**Methods:** In the recent years advanced diffusion acquisition and modeling approaches became available, leading to a significant number of studies that have applied these new tools on psychosis populations. Tools include biological model-based approaches such as free-water imaging, NODDI and permeability-diffusivity index. Other tools select model free approaches such as Kurtosis imaging, Q-space imaging, Diffusion spectrum imaging, and Generalized FA. The advanced methods provide new ways to characterize abnormalities, but at the same time, as the models become more complicated, so are the acquisitions, their length, and their sensitivity to noise. This talk will review findings from advanced diffusion MRI methods and will compare them with those obtained by the conventional DTI approach.
**Results:** The comparison shows that while the sensitivity to identify abnormalities is not necessarily increased by the advanced methods, the fact that in some of these approaches the specificity is improved provides new insights into the nature of the underlying abnormalities. Nevertheless, even though specificity is improved, care must be taken with the interpretation of the result given the fact that diffusion MRI is an indirect measure of microstructure, limited by the assumption embedded in each model.
**Discussion:** The emerging results present dependency on the stage of the psychosis (e.g., first episode, chronic) as well as on the age and gender of the subjects, suggesting that care must be taken in the study design, as well as in the statistical analyses performed. The findings also promote the use of multi-modal acquisitions, as well as the collection of biological, clinical and cognitive parameters. The combined information of these different domains is more likely to truly characterize the underlying abnormalities.

### 6.3 EEG AS A TOOL FOR PSYCHOSIS RESEARCH: CHALLENGES, PITFALLS AND NEW OPPORTUNITIES

Christoph Mulert*,[1]
[1]*University Hospital Hamburg Eppendorf*

**Background:** Electroencephalography (EEG) as the oldest technique currently in use for the analysis of brain function has strong advantages not offered by other techniques: it is a direct measurement of neuronal activity and offers a high temporal resolution. Accordingly, it is very useful for the investigation of neuronal oscillations which are related to disturbed core mechanisms of schizophrenia such as NMDA-receptor dysfunction or E/I imbalance and alterations in connectivity.
**Methods:** On the other hand, the method has also strong limitations, e.g. the difficulty of precise localization, which is due to the inverse problem and also its blindness to subcortical structures that are highly relevant for psychosis research, such as the ventral striatum.
**Results:** Uncritical use of this technique has created widespread skepticism, leading probably to some degree of underestimation of the unique opportunities offered. In this talk, limitations of the technique will be addressed as well as current strategies of proper usage such as the combination of EEG and fMRI.
**Discussion:** Simultaneous EEG-fMRI offers the best from both modalities, that is high temporal and high spatial resolution, but here, too, methodological challenges have to be addressed. Finally, the development of new noninvasive tools for brain stimulation such as transcranial alternating current stimulation (tACS) with the opportunity of frequency-specific modulation of neuronal oscillations ("entrainment") for both brain research and therapy makes detailed information about disturbed oscillations patterns in psychosis even more relevant.

### 6.4 BRAIN FUNCTIONAL CONNECTOMICS BASED ON RESTING STATE FMRI: FROM NODES TO NETWORKS

Juan Zhou*,[1]
[1]*Duke-National University of Singapore Medical School*

**Background:** Emerging evidence suggests that psychosis arises from disrupted communication between distributed neural networks. In the past decade, network-sensitive neuroimaging methods have made it possible to examine vulnerable brain networks in living humans. Previous work has demonstrated that distinct functional intrinsic connectivity networks can be mapped in the healthy brain with task-free or "resting-state" functional magnetic resonance imaging (fMRI). Instead of the changes evoked by specific stimuli, resting state fMRI captures the spontaneous low frequency blood-oxygenation-level-dependent signal fluctuations at rest. Regions showing synchronized spontaneous activities are usually functionally connected and are often supporting highly relevant brain functions.
Being more applicable in patients, recent resting state fMRI studies in psychosis have reported widespread functional dysconnectivity, targeting multiple neural systems that include the default mode network, the salience network, the auditory network, and fronto-striato-thalamic circuits. Such functional connectivity disruptions are also associated with more severe symptoms and more cognitive impairments in patients.
**Methods:** In this talk, I will cover four primary methods for deriving functional connectivity from resting state fMRI data and discuss their pros and cons in the context of schizophrenia. 1) Seed-based approach: correlation between signals of a seed region to other target regions or with the rest of the brain. 2) Independent component analysis: decompose the fMRI data of all brain voxels into spatially non-overlapping and temporally coherent networks. 3) Brain parcellation-based connectivity matrices: based on a set of predefined regions of interest covering the whole brain, the functional connectivity between all pairs of regions are computed and the individual-level connectivity matrices are compared. Lastly, 4) graph theoretical approach is highly useful in capturing and visualizing complex brain interactions embedded in these high dimensional matrices. In a brain graph, each ROI is a node and the functional connectivity between a pair of ROIs is an edge. Graph theoretical measures can then capture the brain functional topology such as functional segregation or modularity at nodal, network, and whole-brain levels.

**Results:** By modelling connectivity as complex networks, this talk will shed some light on whether functional connectomics based on resting state fMRI could 1) reveal symptoms-associated brain network changes; 2) detect early changes in prodromal stage of the disease; 3) predict clinical outcomes in psychosis. Particularly, work from our group and others on persons at-risk for psychosis will be discussed. Moreover, accumulating evidence suggests the influence of vigilance, motion, and physiological noise on functional connectivity measures. I will provide some tips on how to minimize these confounds and increase the reliability and reproducibility of functional connectomics measures.

**Discussion:** Resting state fMRI provides a novel network-sensitive, immediately repeatable, non-invasive tool to examine human functional connectome. Future directions such as dynamic or time-varying functional connectivity which captures neural dynamics at a finer time scale will be briefly discussed. Further developed and integrated with brain structural connectivity measures, brain network functional connectomics may help us better understand heterogeneity in psychosis, reveal disease mechanism, predict and track disease progression, and monitor treatment response.

### 7. RETINAL FUNCTIONS EXPRESSED IN RETINAL IMAGING, CONTRAST PROCESSING AND ELECTRORETINOGRAPHY MAY DECRYPT EARLY RISK MECHANISMS AND PATHOPHYSIOLOGY OF SCHIZOPHRENIA AND MOOD DISORDERS AND ACCELERATE TRANSLATION TO THE CLINIC

Michel Maziade
*Centre de recherche CIUSSS de la Capitale-Nationale*

**Overall Abstract:** The rationale of this symposium is twofold. First, true understanding of the neurobiological and environmental causes of schizophrenia and mood disorders will require the investigation of the human neuronal tissue in function. As an external and accessible extension of the brain, the retina opens this avenue. Existing technologies such as retinal imaging, computerized psychophysical assessment, and electroretinography (ERG) have recently provided evidence that the microanatomy of the retina and strength of rod, cone, and bipolar cell responses to light stimuli can distinguish patients from healthy individuals (Adams & Nasrallah, Schizophr Res 2017; Silverstein & Rosen, Schizophr Res Cogn 2015; Bubl, Biol Psychiatry, 2010; Plos ONE, 2015, Meier, Am J Psychiatry 2014). Second, the search for indicators of brain dysfunction that are detectable both in adult patients and in children at genetic risk is the cornerstone of genetic high-risk research into the neurodevelopmental origins of serious mental disorders and prevention (Maziade, New Eng J Med 2017). In this regard, it has been shown that children at genetic risk show many of the retinal anomalies that adult patients display (Hébert et al., 2010, Biol Psychiatry). Studies of the retina can therefore contribute to clarifying illness pathophysiology and its developmental roots.

This symposium objectives are to: 1) present findings from retinal imaging, visual processing and electroretinography in patients with schizophrenia or mood disorders, and in young healthy offspring of affected parents; 2) to discuss the data in terms of their implications for understanding psychotic and mood disorders; and 3) to clarify the similarities and differences between retinal findings in psychotic and mood disorders and their early developmental trajectories.

Participating scientists: Professor Michel Maziade will be the chair, with Professor Steven Silverstein as the co-chair of the symposium. Professor Anne Giersh, INSERM Strasbourg, France, will act as the discussant.

Professor Emanuel Bubl, Saarland University, Germany, will present findings on the potential of ERG measured retinal background noise as neurobiological correlate for cognitive deficits in ADHD and schizophrenia.

Professor Maziade, Laval University, Canada, will present new ERG findings in young offspring of parents affected by schizophrenia or bipolar disorder and the implications for the illness developmental origin and later transition to illness.

Professor Madeline Meier, Arizona State University, USA, will present results showing phenotypic and genetic associations between schizophrenia and retinal vessel diameter. Findings suggest that individuals with schizophrenia are at increased risk of microvascular complications.

Professor Silverstein, Rutgers University, USA, will present new ERG findings in schizophrenia, and data on the relationships between ERG anomalies, symptoms, retinal structural abnormalities as measured with optical coherence tomography, and antipsychotic medication use.

Based on empirical data, the symposium will offer an integrated view as to: i) how non-invasive measurements of retinal structure and function show consistent anomalies in schizophrenia, bipolar disorder, major depression and ADHD; ii) how findings from children and adolescents at high genetic risk not only indicate a neurodevelopmental process, but also suggest that retinal anomalies in patients are not due to medication use or degenerative effects; iii) how ERG can be administered to adults and children at low cost in clinical studies; iv) how to integrate findings in the staging of the risk status of children at genetic risk.

### 7.1 ELECTRORETINOGRAPHIC ANOMALIES IN SCHIZOPHRENIA AND THEIR RELATIONSHIPS WITH RETINAL STRUCTURE, VISUAL FUNCTIONS, CLINICAL SYMPTOMS, AND MEDICAL COMORBIDITIES

Steven Silverstein*[,1], Docia Demmin[1], Molly Erickson[1], Judy Thompson[1], Danielle Paterno[1], Roni Netser[1]
[1]*Rutgers University*

**Background:** Although several studies have documented retinal cell dysfunction in schizophrenia (Silverstein & Rosen, Scz Res: Cogn, 2015), the extent to which these abnormalities contribute to, and/or result from, other features of the condition is unclear. Thus we sought to: 1) evaluate associations between retinal signaling anomalies as measured with flash electroretinography (fERG) and previously reported changes in visual evoked potentials (VEPs), contrast sensitivity, visual acuity, and contour integration in people with schizophrenia (Silverstein, Neb Symp Motiv, 2016); 2) determine whether fERG anomalies are related to retinal structural abnormalities as indicated by optical coherence tomography (OCT); 3) examine relationships between fERG changes and psychiatric symptoms; 4) determine relationships between fERG anomalies and frequent medical comorbidities in schizophrenia that are known to affect the retina (e.g., diabetes, hypertension); and 5) examine potential medication effects on these findings.

**Methods:** We have assessed 25 patients with schizophrenia and 25 controls who are free of medical comorbidity with fERG and measures of visual function and symptom severity, and data collection is ongoing with patients and controls with diabetes and/or hypertension using these same measures. In addition, we are in the process of completing data collection with two additional groups of patients and controls, one with fERG and OCT (n=12 to date), and another with fERG and VEPs (n=13 to date). fERG data are being collected under both light- and dark-adapted conditions, using a range of flash intensities, backgrounds, and temporal frequencies. The primary fERG variables of interest are a-wave and b-wave amplitudes, which reflect photoreceptor and bipolar cell responses, respectively, and the photopic negative response (PhNR), which reflects ganglion cell activity.

**Results:** On photopic fERG tests, patients with schizophrenia demonstrated significantly weaker photoreceptor response when a flash was presented against an unlit background ($p<.05$), and during a steady-state flicker test ($p<.005$). On scotopic tests, the rate of response gain per unit of intensity increase was significantly weaker for patients than controls ($p=.001$). In both light- and dark-adapted conditions, patients demonstrated weaker signaling of bipolar cells (ps < .005). The schizophrenia group was also characterized by a weaker PhNR (p<.05). Weaker retinal cell responses were related to contrast sensitivity impairments in the schizophrenia group (ps < .05 and .001), but not to visual acuity or contour integration. Reduced responsiveness to low-intensity light was related to more severe negative symptoms, suggesting a reduced dynamic range within which environmental events (i.e., salience) are represented. Measures of retinal cell function were not related to antipsychotic medication dose. Preliminary findings indicate that attenuated fERG signals are not associated with weaker visual cortical responses (EEG-measured VEPs), presumably due to gain control mechanisms. We will report on the extent to which fERG anomalies are related to retinal structural changes and comorbid medical conditions.
**Discussion:** Reduced signaling of photoreceptor, bipolar, and ganglion cells are characteristics of schizophrenia, and are not related to extent of antipsychotic medication use. These changes are related to reduced contrast sensitivity and increased negative symptoms, and may reflect an attenuated ability to accurately represent changes in the intensity of environmental stimuli. Data collection is ongoing for studies examining relationships between ERG indices and VEPs and medical comorbidities.

### 7.2 ELECTRORETINOGRAPHIC ANOMALIES SEEN IN PATIENTS AFFECTED BY SCHIZOPHRENIA OR BIPOLAR DISORDER ARE DETECTABLE EARLY IN CHILDREN BORN TO AN AFFECTED PARENT: IMPLICATIONS FOR THE STAGING OF RISK STATUS IN CHILDHOOD-ADOLESCENCE

Michel Maziade*[,1], Thomas Paccalet[2], Anne-Marie Gagné[1], Elsa Gilbert[3], Daphné Lussier[4], Marc Hébert[3], Valérie Jomphe[1], Nathalie Gingras[3]
*[1]Centre de recherche CIUSSS de la Capitale-Nationale; [2]Université de Québec à Trois-Rivières et Centre de recherche CIUSSS-CN; [3]Université Laval & Centre de recherche CIUSSS-CN*

**Background:** Adult patients having schizophrenia, bipolar disorder or major depression display indicators of brain dysfunctions that may be detectable in healthy children-adolescents at genetic risk, such as those born to an affected parent (Maziade, New Eng J Med 2017; Schizophr Res 2013). For instance, cognitive deficits are displayed by both adult patients and children at risk (Maziade, Schizophr Bull 2011). We had reported that schizophrenia patients present diminished amplitudes and delayed latencies of rod and cone photoreceptor responses (Hébert, Schizophr Res, 2015) and we recently found that bipolar patients have similar ERG anomalies. We had also reported preliminary data in a small sample of 29 children born to an affected parent showing that young offspring had rod diminished amplitudes (Hébert, Biol Psychiatry 2010).
The present objectives were i) under the hypothesis that offspring would display many of the ERG anomalies that schizophrenia or mood disorder patients carry (Hébert, Schizophr Res 2015; Prog Neuropsychopharmacol Biol Psychiatry 2017), to look for cone and rod response anomalies in a large sample of young high-risk offspring; ii) to describe the relationship between ERG anomalies and other risk endophenotypes in the offspring; and iii) look at the relationship between ERG anomalies and the risk clusters already shown to predict later transition to illness.
**Methods:** The sample consisted of 84 young offspring (aged 6 to 27) of a parent affected by schizophrenia or bipolar disorder, compared to 224 healthy controls balanced for age and sex. Full-field cone and rod ERG was measured in non-dilated eyes for all subjects. In the young offspring, we also collected measures of different cognitive domains, attenuated symptoms of psychosis, non-psychotic DSM diagnosis and/or an episode of poor GAF functioning in childhood-adolescence, childhood trauma, and cannabis use (Paccalet, Schizophr Res 2016).
**Results:** In comparison to controls the offspring displayed three ERG anomalies that were observed in adult patients: prolonged cone b-wave latency (p=0.04), diminished rod b-wave amplitude (p=0.04) and prolonged rod b-wave latency (p=0.006). These ERG anomalies were shared by offspring of a parent with schizophrenia or bipolar disorder, an observation of ERG commonality that we had made in adult patients. The three ERG amplitude and latency anomalies tended to aggregate in a child at risk, a trend we also observed in another endophenotype modality such as deficits in different cognitive domains. However, in these high-risk children and adolescents, the patterns of aggregation suggest that ERG anomalies would depict another risk pathway than that marked by cognitive deficits.
**Discussion:** First, ERG anomalies in high-risk children have neurobiological implications for future research on the illness neurodevelopment. Second, as found for other modalities of risk endophenotypes in children at genetic risk (Maziade, New Eng J Med 2017), multiple rod and cone ERG anomalies tended to cluster together in a child. Such an aggregation may be compatible with the multifactorial polygenic theory with a threshold. Remarkably, a clustering of risk indicators is also observed in children at risk of metabolic cardiovascular disorders and is presently considered in practice guidelines for these children. The clustering of risk indicators may provide an empirical basis for the staging of the risk status of children at genetic risk and has immediate implications for their longitudinal surveillance in the clinic.

### 7.3 EVALUATING THE NEUROBIOLOGICAL CORRELATES AND IMPACT OF TREATMENT ON COGNITIVE DYSFUNCTION IN ADHD AND SCHIZOPHRENIA BY MEANS OF THE PATTERN ELECTRORETINOGRAM

Emanuel Bubl*[,1], Lisa Werner[2], Yumin Liang[2], Dieter Ebert[2], Evelyn Friedel[2], Anna Bubl[1], Michael Bach[2], Ludger Tebartz van Elst[2]
*[1]University of Saarland; [2]University of Freiburg*

**Background:** Problems with cognitive function are found in many major psychiatric disorders. In schizophrenia and attention deficit hyperactivity disorder (ADHD), they are among the core symptoms. Based on a growing recognition that there is little diagnostic specificity for any single cognitive impairment, there is an increasing emphasis on investigating impairments across psychiatric disorders. This approach, which is consistent with the NIMH Research Domain Criteria initiative, is expected to lead to a better understanding of the neurobiological mechanisms involved in cognitive deficits.
An increase in neuronal background noise has been identified as a neuronal correlate of inattention. Because the dopamine system has been found to play a critical role in modulating neuronal noise, dopamine dysfunction may play a substantial role in generating the excessive noise that has been found to characterize information processing in both schizophrenia and ADHD. This issue can be studied noninvasively via electrophysiological examination of the retina, a distinct neural network. Both basic research and human studies indicate that retinal information processing is under strong dopaminergic modulation (Bubl, Biol. Psychiatry, 2010; Bubl, Br J Psychiatry, 2012). We have previously demonstrated an elevated level of background noise at a very early stage in visual information processing in untreated patients with ADHD (Bubl, Plos One, 2015). Moreover, background noise was associated with inattention measures in these subjects. To further address the hypothesis that elevated retinal noise reflects dopaminergic dysfunction, we report here on a new study that compared retinal background noise in patients with ADHD both before and after therapy, as well as in patients with schizophrenia.
**Methods:** Neuronal noise was assessed using pattern electroretinogram (PERG), an objective electrophysiological measure for retinal network function from the photoreceptors to the retinal ganglion cells. A total of 20 patients diagnosed with ADHD were tested both before and after treatment with methylphenidate (MPH). The control group consisted of 21 healthy subjects. The PERGs were recorded in a steady state mode in response to checkerboard stimuli of 12 reversals/s. Data collection with people with schizophrenia is ongoing, and results will be reported at SIRS.
**Results:** Before treatment, the patients with ADHD presented with elevated background noise (higher by 127%) in comparison to the control group. After treatment, noise level did not differ from what was observed in the control group. Retinal background noise was found to be highly correlated with the severity of the ADHD symptoms. The results will be discussed in relationship to our findings in patients with schizophrenia.
**Discussion:** These data provide further evidence for the hypothesis that elevated background noise is linked to ADHD and cognitive deficits. The findings are of special relevance because ADHD is a disorder with a dedicated treatment option for cognitive symptoms. Interestingly, a similar pathophysiological mechanism for cognitive dysfunction has been proposed for both schizophrenia and ADHD. However, because ADHD medications, such as MPH, typically elevate dopamine levels, potentially leading to exacerbation of psychotic symptoms, different approaches for treating cognitive symptoms in schizophrenia need to be explored. On this basis, current approaches used to target neuronal noise and cognitive symptoms in patients with schizophrenia will be discussed and their relevance for future research will be addressed.

### 7.4 PHENOTYPIC AND GENETIC ASSOCIATIONS BETWEEN SCHIZOPHRENIA AND RETINAL VESSEL DIAMETER

Madeline Meier*[,1], Enda Byrne[2], Nicholas Martin[3], Tien Wong[4], Xueling Sim[5]
*[1]Arizona State University; [2]University of Queensland; [3]Queensland Institute for Medical Research; [4]Singapore Eye Research Institute; [5]University of Michigan*

**Background:** Individuals with schizophrenia are at increased risk for cardiovascular diseases, and this risk cannot be fully explained by antipsychotic medications or lifestyle factors. Retinal imaging offers a non-invasive means of visualizing the microvasculature in living individuals with schizophrenia. Here we test whether individuals with schizophrenia exhibit retinal microvascular abnormality (Meier et al., AJP, 2013), and test for overlap in the genetic variants associated with schizophrenia and retinal microvascular abnormality.
**Methods:** To test whether individuals diagnosed with schizophrenia showed microvascular abnormality, we used data from the Dunedin Study, a representative cohort of 1,000 New Zealanders followed from birth to age 38. The cohort underwent retinal imaging at age 38, and retinal venular (small veins) and arteriolar (small arteries) diameters were obtained. Analyses compared individuals with schizophrenia (n=27), healthy individuals (n=412), and individuals with medical or psychiatric conditions (ns ranged from 110–210) on retinal vessel diameter. To test for overlap in the genetic variants associated with schizophrenia and retinal vessel diameter, we used linkage disequilibrium-score regression (LD regression) to obtain the genetic correlation between schizophrenia and retinal vessel diameter. This method requires only GWAS (genome wide associate studies) summary statistics rather than actual genotypes. Summary statistics for schizophrenia and retinal vessel diameter came from published meta-analyses.
**Results:** Adults diagnosed with schizophrenia had wider retinal venules (standardized mean=0.59) compared with same-age healthy adults (standardized mean=-0.20) and compared with individuals diagnosed with hypertension, diabetes, and tobacco dependence. Findings could not be explained by antipsychotic medication, as venular diameter was similar in the subset of individuals diagnosed with schizophrenia who had not taken antipsychotic medication in the year prior to retinal imaging (n=22; standardized mean=0.69). There were no differences in arteriolar diameter between individuals diagnosed with schizophrenia and all other groups. Results from LD regression showed a small genetic correlation between schizophrenia and venular diameter (r=0.05, p=.31) and a slightly larger genetic correlation between schizophrenia and arteriolar diameter (r=0.17, p=.02).
**Discussion:** Wider venular diameter is a distinguishing feature of schizophrenia, but genetic variants associated with schizophrenia overlap more strongly with variants associated with arteriolar diameter. It is possible that environmental influences associated with schizophrenia tend to narrow arterioles, obscuring a phenotypic link between schizophrenia and wider arterioles. Pathophysiological mechanisms underlying vessel diameter, including inflammation and endothelial dysfunction, might be related to the development of schizophrenia, and particular genes might contribute to both schizophrenia and arteriolar diameter. Findings will be discussed in relation to links between retinal vessel diameter and IQ (Shalev, Meier et al., Psychol Sci, 2013) and depression (Meier et al., Psychosom Med, 2014).

### Plenary

### 8. DECREASING CARDIOVASCULAR RISK IN PERSONS WITH SCHIZOPHRENIA: INTERVENTIONS AND FUTURE DIRECTIONS

Gail Daumit
*Johns Hopkins Medical Institutions*

**Overall Abstract:** Persons with schizophrenia experience two to three times higher mortality than the overall population. This premature death is due in large part to cardiovascular disease and is potentially preventable. All cardiovascular risk factors are elevated in persons with schizophrenia. This presentation will describe the evidence for interventions to reduce cardiovascular risk factors in this vulnerable population, including obesity and tobacco smoking, and will describe models of integrated physical and mental health care. Ongoing research on interventions to decrease cardiovascular risk in schizophrenia will be presented, and future research needs will be discussed including implementation strategies to scale-up interventions to reduce cardiovascular disease risk in community settings.

### Concurrent Symposia

### 9. DOES BIOLOGY READ THE DSM? TRANSDIAGNOSTIC FINDINGS IN PSYCHOSIS AND IMPLICATIONS FOR TREATMENT

Michael Owen
*Cardiff University*

**Overall Abstract:** A major emerging issue in schizophrenia research is the degree to which the mechanisms underlying the disorder are specific to schizophrenia or are common to a number of disorders, potentially indicating common and distinct pathways to illness. Understanding this is important for diagnosis, biomarkers and the development of new treatments. This symposium will bring together new data to consider the latest findings from different genetic, imaging and clinical approaches.
Dr. Owen, Wales, will present the latest genetic data from the largest genome-wide genetic analyses to date in psychotic disorders (schizophrenia and bipolar disorder) and neurodevelopmental disorders (autism, intellectual disability and ADHD), comprising samples from over 100,000 patients and controls. These data identify novel shared pathways involving neurodevelopmental genes, synaptic function and histone modification that are common across these disorders, but also identify differences in the degree of involvement of particular pathways.
Dr. Howes, England, will present new data from neurochemical and structural imaging studies comparing patients across psychotic and neurodevelopmental disorders (including schizophrenia, bipolar disorder people at risk of autism), showing that there are common dopaminergic alterations linked to psychosis across disorders, as well as showing that structural and neurochemical brain heterogeneity is increased in most brain regions, but also identifying key cortical and sub-cortical regions with increased homogeneity.

Dr. Clementz, USA, will present new EEG and cognitive data from over 400 patients with schizophrenia, and bipolar disorder. This shows differences in intrinsic neural activity that cuts across diagnoses, identifying subtypes that were linked to differences in cognitive functioning.

Dr. Wichers, Netherlands, will present new data from a longitudinal study of adolescents using in-depth real-time phenotyping using experience sampling to investigate the relationship between the coherence of responses and the subsequent development of psychotic and other symptom domains one year later. Her novel application of complex systems theory identifies suspiciousness as a common predictor of the later development of a number of symptoms, but also that other responses, such as low mood, determine the specificity of later outcomes to psychosis.

Overall this symposium will bring together researchers using different, complementary approaches to provide a comprehensive and multi-disciplinary analysis. By bringing researchers from different disciplines together it will enable common mechanisms to be considered, and new insights to be developed. Finally, Dr. DeLisi's wide-reaching experience means she is well placed to lead the discussion of the implications of these findings for understanding the neurobiology of schizophrenia, and for biomarker development as well as considering their implications for the developing new treatments.

The symposium includes gender diversity in presenters and chairs, speakers from multiple institutions across continents, and diversity in career levels with speakers from early, mid and established positions.

### 9.1 GENOMICS AND PSYCHIATRIC DIAGNOSIS

Michael Owen*,[1]
*[1]Cardiff University*

**Background:** Recent genomic studies have begun to reveal the genetic architecture of psychiatric disorder and to give important insights into the relationship between the psychiatric syndromes that form the basis of current taxonomy. These studies have demonstrated the highly polygenic nature of psychiatric disorders, and have indicated that many individual genetic associations are shared across multiple disorders in a way that points to extensive biological pleiotropy and challenges the biological validity of existing diagnostic approaches.

**Methods:** I will present genomic data, predominantly from the study of rare variants, that support the idea of a neurodevelopmental continuum, in which schizophrenia and bipolar disorder, together with childhood neurodevelopmental disorders, such as ID, ASD and ADHD represent the diverse range of outcomes that follow from disrupted or deviant brain development and furthermore that, within the neurodevelopmental continuum, severe mental illnesses occupy a gradient of decreasing neurodevelopmental impairment as follows: ID, ASD, schizophrenia and bipolar disorder. I will also present findings indicating that common genetic variation modifies the outcome of neurodevelopmental impairment explaining in part the diversity of psychiatric outcomes. Finally, I will explore how genetic data might be used to inform novel approaches to patient stratification which will be informative for prognosis and treatment response and facilitate the identification of novel drug targets.

**Results:** Finally, despite the undoubted complexity and the fact that much of the genetic risk remains unaccounted for at the DNA level, there are encouraging signs that the genes implicated in schizophrenia converge onto sets of plausible biological processes. In particular, the data point to synaptic function and histone modification and implicate mechanisms involved in brain plasticity that are important in development and in learning and cognition. While these are almost certainly not the only processes involved, they provide robust entry points for clinical and basic neuroscience research.

**Discussion:** N/A

### 9.2 BRAIN STRUCTURAL AND NEUROCHEMICAL HETEROGENEITY AND HOMOGENEITY IN PSYCHOTIC DISORDERS: TRANSDIAGNOSTIC PET AND MRI IMAGING FINDINGS IN SCHIZOPHRENIA AND BIPOLAR AFFECTIVE DISORDER

Oliver Howes*,[1], Sameer Jauhar[2], Stefan Brugger[3], Fiona Pepper[2]
*[1]MRC LMS and KCL; [2]King's College London & MRC Clinical Sciences Centre; [3]MRC LMS*

**Background:** Psychosis is seen in a number of disorders and treated with the same drugs. However, there is considerable variability in response to treatment and clinical course. Understanding the neurobiology underlying psychosis across diagnoses and in treatment response is important to help guide the development of new treatments and biomarkers for treatment response. Elevated dopamine synthesis and release capacity and structural brain changes have been consistently associated with schizophrenia, but it remains unknown how variable these are, or how they compare across psychotic disorders.

**Methods:** Two cohorts of first episode patients, one with a diagnosis of schizophrenia (n=16) and another with a diagnosis of bipolar affective disorder (n=22) received 18F-DOPA PET and [1H]-MR spectroscopy imaging and clinical measures. All patients had experienced a psychotic episode and received clinical follow-up over 18 months to determine diagnostic stability. We then conducted a meta-analysis using a novel meta-analytic approach to quantify variability in measures to investigate structural and neurochemical heterogeneity in schizophrenia and bipolar affective disorder. The entire PubMed, EMBase and PsychInfo databases were searched from inception to identify relevant studies and the natural log of the measures of dispersion and the coefficient of variance.

**Results:** Striatal dopamine synthesis capacity (Kicer) was significantly elevated in both bipolar (effect size=1.02; $p<0.003$) and schizophrenia (effect size=0.9; $p<0.05$) groups, compared to controls. There was no significant difference in dopamine synthesis capacity between bipolar and schizophrenia groups ($p>0.4$). Kicer was significantly positively correlated with positive psychotic symptom severity in the transdiagnostic group of people with psychosis ($r=0.52$, $p<0.004$), and in the bipolar group after adjusting for manic symptom severity ($r=0.6$, $p<0.01$). There were no differences in glutamate levels in the anterior cingulate cortex.

In the meta-analyses a total of 128 studies were identified including >4000 patients and >4000 controls. Variability ratio was significantly increased in patients relative to controls in gray matter volumes in temporal lobe (VR=1.1, $p=0.004$) and thalamus (VR=1.16, $p<0.001$), and in striatal dopamine receptor density ($p<0.05$) but unaltered in frontal cortex and significantly reduced in the anterior cingulate cortex (VR=0.9, $p=0.02$)

**Discussion:** Elevated dopamine synthesis capacity is associated with psychosis across diagnostic boundaries and linked to the severity of psychotic symptoms, even after adjusting for manic symptom severity. Striatal dopamine receptor density and structural gray matter volumes in a number of cortical and sub-cortical regions show heterogeneity in psychotic disorders, but frontal cortical regions show unaltered and, in the case of the anterior cingulate cortex, reduced heterogeneity, suggesting alterations are homogenous across patients. Taken together these findings striatal dopamine synthesis and structural changes in frontal cortex are common mechanisms linked to psychosis across disorders.

### 9.3 PSYCHOSIS BIOTYPES VERSUS CLINICAL SYNDROMES THROUGH THE PRISM OF INTRINSIC NEURAL ACTIVITY

Brett Clementz*[,1], Godfrey Pearlson[2], Carol Tamminga[3], John Sweeney[3], Matcheri Keshavan[4]
*[1]University of Georgia; [2]Olin Neuropsychiatry Research Center; [3]University of Texas Southwestern Medical Center; [4]Beth Israel Deaconess Medical Center & Harvard Medical School*

**Background:** Deviation in level of intrinsic neural activity (ongoing brain signals recorded with EEG/MEG) is observed in psychosis. Neurophysiological models have proposed this physiological indicator as a genetically mediated core deviation in psychosis. Translational models of intrinsic activity deviations promise to identifying multiple distinct physiological mechanisms for psychosis manifestation. Intrinsic activity deviations may masquerade as higher levels of neural response in sensory cortices, but ultimately may lead to poor signal-to-noise ratios, particularly when psychosis cases are required to identify stimulus salience.

Why do we not hear more about intrinsic activity as a core biomarker for any psychosis variation? An explanation is provided by the current project. The Bipolar-Schizophrenia Network for Intermediate Phenotypes (B-SNIP) published a means for categorizing psychoses by neurobiological homology via use of multiple biomarkers (psychosis Biotypes) rather than by clinical features. B-SNIP demonstrated the superiority of Biotypes versus DSM diagnoses for capturing neurobiological similarity through multiple external validating measures (social functioning, measures of brain volume from structural magnetic resonance images, clinical diagnoses and biomarker features among first-degree relatives). Independent analyses since the initial publication have provided additional support for the usefulness of psychosis Biotypes.

**Methods:** For this project, we analyzed ongoing neural activity from 64 EEG sensors during 150 intervals of 10 sec duration from over 1450 B-SNIP subjects. These data (never before published) were from the inter-trial interval (ITI) of an auditory paired-stimuli task used in Biotypes construction (these ITI data themselves were not used). Although the subjects were engaged in a task (counting the number of stimulus pairs), the data used here were not part of the task itself. Data were evaluated for single trial power (estimate of neural response strength on individual trials) as a function of frequency of neural oscillations (from 2–50 Hz) over the whole head. Data were then averaged over single trials to yield an estimate of the overall strength of nonspecific (unrelated to sensory processing) neural activity.

**Results:** When evaluated by DSM diagnoses (schizophrenia, schizoaffective disorder, bipolar disorder), the 95% confidence intervals for all groups overlapped the healthy group means across all frequencies. When considered by psychosis Biotypes, differences were obvious and statistically significant. In comparison to healthy persons, Biotype-2 probands (the most neurophysiologically activated subgroup in previous analyses) were notably high on nonspecific neural activity, and Biotype-1 probands (the most cognitively and neurophysiologically compromised subgroup in previous analysis) were notably low. Group separations on this metric were better than those obtained with original intrinsic EEG measure used in psychosis Biotypes construction, indicating this more pure intrinsic activity measure is capturing a meaningful component of Biotype neurophysiology. This was true across a range of oscillatory frequencies for the probands. The first-degree relatives of the Biotype probands showed similar patterns, although higher frequency oscillations (above 20 Hz) better differentiated relatives from healthy persons.

**Discussion:** Intrinsic activity deviation is a promising biomarker for translational research programs aimed at differential treatment development, but using DSM psychosis diagnoses would obscure its importance for understanding psychosis.

### 9.4 COMPLEX SYSTEM THEORY AND THE TRANSDIAGNOSTIC USE OF EARLY WARNING SIGNALS TO FORESEE THE TYPE OF FUTURE TRANSITIONS IN SYMPTOMS

Marieke Wichers*[,1], Marieke Schreuder[1], Catharina Hartman[1], Hanneke Wigman[1]
*[1]Interdisciplinary Center Psychopathology and Emotion regulation (ICPE), University Medical Center Groningen*

**Background:** Recently, we showed that assumptions from complex system theory seem applicable in the field of psychiatry. This means that indicators of critical slowing down in the system signal the risk for a critical transition in the near future. In the current study we wanted to explore whether the principle of critical slowing down may also be informative to anticipate on the type of symptoms that individuals are most likely to develop. This is relevant as it may lead to personalized prediction of risk of whether adolescents with mixed complaints are most likely to develop either depression, anxiety, somatic or psychotic symptoms in the near future. For example, we hypothesized that critical slowing down in feeling ‘suspicious’ more strongly indicates risk for a future transition to psychotic symptoms, while critical slowing down in feeling ‘down’ more strongly indicates risk for a transition to depressive symptoms.

**Methods:** We examined this in a population of adolescents (most between 15 and 18 years) as adolescents are an at-risk group for the development of psychopathology. At baseline experience sampling was performed for 6 days, 10 measurements a day. Affect items were used to assess autocorrelation as an indicator of ‘critical slowing down’ of the system. At baseline and follow-up SCL-90 questionnaires were administered. In total, 147 adolescents participated both in baseline and follow-up measures and showed increases in at least one of the defined symptom dimensions. We examined whether autocorrelation was positively associated with the size of symptom transition and whether different type of transitions (in depression, anxiety etc.) were differentially predicted by autocorrelations in specific affect states.

**Results:** The analyses were done very recently, and findings have not been presented before. We found both shared and specific indicators of risk in the development for transition to various symptom dimensions. First, autocorrelation in ‘feeling suspicious’ appeared to be the strongest signal for all assessed psychopathology dimensions (SCL-90 depression: std beta: 0.185; p <0.001; SCL-90 anxiety: std beta: 0.093; p=0.006; SCL-90 interpersonal sensitivity: std beta: 0.176, p<0.001). Second, we found that the combination of ‘feeling suspicious’ and the affect with the second-highest autocorrelation together predicted the precise type of symptom transition. Thus, the combination of feeling suspicious (std beta: 0.185; p<0.001) and down (std beta: 0.108; p=0.001) predicted larger increases in depressive symptoms one year later on the SCL-90, while the combination of feeling suspicious (std beta: 0.093; p=0.006) with feeling anxious (std beta: 0.086; p=0.014) predicted larger increases in anxiety symptoms a year later on the SCL-90.

**Discussion:** These findings support the hypothesis that indicators of slowing down can not only be used to predict risk for a mean level shift in symptoms, but that they can also be informative for the type of symptom transitions at hand. In a next step these findings could be translated to designs measuring personalized early warnings for future direction of symptom shifts, and if successful to clinical implementation of these techniques.

### 10. THE MOLECULAR MECHANISMS OF SCHIZOPHRENIA FROM GLIAL CELLS PERSPECTIVE

Sabina Berretta[1]
*[1]Harvard Medical School, McLean Hospital*

**Overall Abstract:** In the past decade, rapid advances in the field of neuroscience resulted in a dramatic paradigm shift in the way we understand the role of glia in normal brain functions and brain disorder pathology. A growing body of evidence shows that diversified populations of astrocytes, microglia, oligodendrocyte precursors and mature oligodendrocytes play a critical role in the regulation of synaptic functions, blood-brain barrier, immune response regulation, myelination and axonal conduction, and in the synthesis of the extracellular matrix, a key regulator of neural plasticity. Building on this evidence, exciting new findings are beginning to emerge, shedding light on glia abnormalities in schizophrenia and their impact these functions. This symposium aims to discuss and integrate the current state of knowledge on direct evidence for glial abnormalities in schizophrenia and their underlying mechanisms.
Dr. Juliana Nascimiento will present novel findings on the effects of NMDAr antagonists and antipsychotics influence glial cell lines and 3D cultures as neurospheres and cerebral organoids. Results from these studies point to the central role of glycolysis, EIF2 signaling and translational machinery in oligodendrocytes and astrocytes. Dr. Paul Klauser will report on elegant investigations on the implication of developmental redox imbalance inducing oxidative stress leading to impairments of oligodendrocytes, myelin formation and eventually to the disruption of white fibers integrity and conductivity, especially in brain regions where the metabolic demand is high. In patients, alterations of white matter were found to be inversely correlated with blood levels of GSH precursor cysteine and could be prevented by the early administration of the antioxidant N-acetyl cysteine. Dr. Sabina Berretta will discuss recent findings on novel modalities of interaction between glial cells, extracellular matrix and neurons, postulated to affect synaptic structural plasticity and axonal conductance. A growing body of evidence from her group shows disruption of such interactions in schizophrenia, potentially contributing to synaptic pathology and impacting neural connectivity. Dr. Dost Ongur will build on previous work showing abnormal diffusion of neuron-specific metabolite NAA in frontal white matter in patients with chronic schizophrenia in the absence of abnormalities in the diffusion of non-specific metabolites Cr and Cho. State-of-the-art recent studies on first episode psychosis patients and matched healthy controls show that NAA diffusion is normal in first episode patients but Cr and Cho diffusion is abnormal, suggesting that white matter abnormalities in non-neuronal elements in early phases of schizophrenia which are followed by neuronal damage in chronic disease.

### 10.1 STEM CELL-DERIVED IN VITRO MODELS FOR DEPICTING THE ROLE OF GLIA IN SCHIZOPHRENIA FROM A PROTEOMIC PERSPECTIVE

Juliana Nascimento*,[1], Daniel Martins-De-Souza[1], Veronica M. Saia-Cereda[1], Flavia C. A. Gomes[2], Stevens K. Rehen[3]
*[1]University of Campinas; [2]Federal University of Rio de Janeiro; [3]Instituto D'Or de Pesquisa e Ensino (IDOR) and Federal University of Rio de Janeiro*

**Background:** A number of basic and translational studies have clearly indicated the vital role of glia in brain function and the pathophysiological mechanisms of neuropsychiatric disorders, including schizophrenia. The difficulty on studying the molecular basis of glial cells in vivo, led to the development of animal models, which are considered the gold standard to this type of understanding. However, the inherent difficulties in establishing these models for psychiatric disorders and the simplicity of in vitro models, especially given the recent advances in stem cell-based technologies have driven the development of sophisticated in vitro models, which may be attractive for studying the molecular basis of schizophrenia.
**Methods:** Here, we report our investigations in terms of proteome while establishing protocols to generate human pluripotent stem cells-derived cerebral organoids as well as human cerebral organoids-derived astrocytes and oligodendrocytes.
**Results:** The proteome of cerebral organoids show major proteins from neuronal cells as expected, but also several glial markers, supporting the notion that glial cells may be obtained out of these organoids. Besides, the proteome of three schizophrenia and three control organoids have been investigated. Proteins found are broadly distributed on functional activities such as cell growth and maintenance, energy metabolism and cell communication and signaling, and are correlated to cortical brain tissue. We also succeeded in isolating astrocytes out of cerebral organoids. These cells are under investigation in terms of molecular differences associated to schizophrenia.
**Discussion:** The generation of brain organoids and isolation of astrocytes and eventually oligodendrocytes hold great potential for the investigation of the role of glia in schizophrenia, providing an useful approach to drug screening and disease modeling, as our results showed in schizophrenia- and control-derived cells. Additionally, proteomics adds knowledge about information and connections being formed into these models.

### 10.2 REDOX DYSREGULATION, OLIGODENDROCYTES AND WHITE MATTER ALTERATIONS IN SCHIZOPHRENIA

Paul Klauser*,[1], Philipp S. Baumann[1], Margot Fournier[2], Lijing Xin[3], Alessandra Griffa[1], Martine Cleusix[2], Raoul Jenni[2], Michel Cuenod[2], Patric Hagmann[4], Philippe Conus[1], Kim Q. Do[2]
*[1]Lausanne University Hospital; [2]Center for Psychiatric Neuroscience, Lausanne University Hospital; [3]Animal Imaging and Technology Core (AIT), Center for Biomedical Imaging (CIBM), Ecole Polytechnique Fédérale de Lausanne; [4]Signal Processing Laboratory, Ecole Polytechnique Fédérale de Lausanne, Lausanne University Hospital*

**Background:** Widespread (Klauser et al., 2016) and progressive (Cropley et al., 2017) cerebral anomalies of white matter diffusion properties (i.e. fractional anisotropy, FA) have been observed in the Australian Schizophrenia Research Bank (ASRB), one of the largest samples of patients with schizophrenia. From a topological perspective, widespread alterations of white matter tend to concentrate into hub regions that interconnect brain areas over long-distances in a so-called "rich-club" (van den Heuvel et al., 2013; Klauser et al., 2016) in which the metabolic demand is high and thus are most likely to suffer from oxidative stress. Evidence from human and animal models suggests that redox dysregulation leading to oxidative stress during neurodevelopment is implicated in schizophrenia pathogenesis (Steullet et al., 2017). At the cellular level, the triad composed of NMDAR hypofunction, neuroinflammation and dopamine dysregulation interacts with redox imbalance and leads to oxidative stress, affecting oligodendrocytes precursor cells (OPC) and parvalbumine interneurons (Steullet et al., 2016). However, the links between redox imbalance, oligodendrocytes and gross alterations of white matter integrity are largely unexplored. Under oxidative stress induced in vitro by impairing the synthesis of glutathione (GSH), the key player in antioxidant defense, OPC showed a decreased proliferation mediated by an upregulation of Fyn kinase activity. In the prefrontal cortex of a mouse model with impaired GSH synthesis, mature oligodendrocyte numbers as well as myelin markers were decreased at peripuberty (Monin et al., 2014). FA was also reduced in fornix-fimbria and anterior commissure, a change accompanied by a reduced conduction velocity (Corcoba et al., 2015).
**Methods:** 49 patients with psychosis and 64 healthy controls were scanned with the same 3-Tesla scanner. The diffusion spectrum imaging (DSI) sequence included 128 diffusion-weighted images with a maximum b-value of 8000 s mm−2. White matter diffusion properties were estimated using generalized fractional anisotropy (gFA). Total blood cysteine (Cys, protein-bound form, free reduced and free oxidized form), the rate-limiting precursor of GSH, was measured by high performance liquid chromatography from plasma samples collected at the same time-point as MRI brain scans.

Whole brain voxel-based analyses were performed using cluster-based non-parametric permutation testing on gFA maps. Cerebral levels of GSH were assessed by localized 1H-MRS measurements from a volume of interest in medial prefrontal cortex.
**Results:** As previously described in ASRB, we observed widespread abnormalities of white matter in patients. Interestingly, the degree of white matter alterations (i.e. decreased gFA) patients could be predicted by the levels of blood cysteine, a precursor of GSH, strongly suggesting the important role played by oxidative stress in the pathophysiological mechanism. Also, we found that white matter alterations could be reversed by 6 months of add-on treatment with the antioxidant and GSH precursor N-acetyl-cysteine (NAC). Most importantly, this improvement was positively correlated with an increase in prefrontal GSH levels.
**Discussion:** We propose that developmental redox imbalance inducing oxidative stress may lead to impairments of oligodendrocytes, myelin formation and eventually to the disruption of fibers integrity and conductivity, especially in brain regions having high metabolic demand. In patients, alterations of white matter are inversely correlated with blood levels of GSH precursor cysteine and could be prevented by the early administration of the antioxidant NAC.

### 10.3 GLIA-EXTRACELLULAR MATRIX INTERACTIONS IN THE PATHOPHYSIOLOGY OF SCHIZOPHRENIA AND BIPOLAR DISORDER

Sabina Berretta*[,1], Gabriele Chelini[1], Harry Pantazopoulos[1]
[1]*Harvard Medical School, McLean Hospital*

**Background:** Growing evidence from our group and others indicates that key neural functions, including regulation of synaptic plasticity and axonal guidance and connectivity, arise from interactions between glial cells, neurons, and the extracellular matrix. Several distinct populations of glial cells critically contribute to the composition of main components of the extracellular matrix (ECM), synthesizing them and secreting them into the extracellular space, where they become incorporated in organized ECM structures. The brain ECM, and chondroitin sulfate proteoglycans (CSPGs) in particular, play a key role in brain development and adult life, in turn regulating glial functions as well as synaptic plasticity and neural connectivity. We have previously shown that glial cells expressing CSPGs are altered in the amygdala and entorhinal cortex of people with schizophrenia (SZ) and bipolar disorder (BD). These changes are accompanied by marked decreases of perineuronal nets (PNNs), organized ECM structures unsheathing distinct neuronal populations. Recent and ongoing studies are focused on novel CSPG-enriched ECM structures, related to synaptic complexes and myelinated axons, their relationship to glial populations and their involvement in the pathophysiology of SZ and BD.
**Methods:** Postmortem tissue samples from the amygdala, entorhinal cortex and thalamus from a well characterized cohort of healthy control, SZ and BD subjects were included in these studies. Multiplex immunofluorescence combined with quantitative microscopy was used to quantify glial cells and CSPGs, while electron microscopy on human and mouse tissue were used to investigate ultrastructural morphology. Step-wise ANOVA analyses included several potential confounds such as exposure to pharmacological agents and substance abuse.
**Results:** Our results show that at least two novel ECM structures are present in the human brain. The first, enriched in CSPGs bearing chondroitin sulfation in position 6 (CS-6), and named here 'CS-6 clusters' was found to be markedly decreased in the amygdala of people with SZ and BD. Electron microscopy studies show that CS-6 clusters are composed of astrocytes synthesizing and secreting CS-6 CSPGs in the vicinity of adjacent groups of dendrites, where it is incorporated into postsynaptic densities of dendritic spines. The second CSPG-enriched ECM structure, i.e. axonal coats, has been observed in the human thalamus to envelope distinct populations of axons, interweaving with myelin sheets. Its main CSPG components appear to be synthesized and secreted by oligodendrocytes precursor cells located in the vicinity of axon bundles. Preliminary results show abnormalities affecting both oligodendrocyte precursors and axonal coats in SZ.
**Discussion:** In summary, our results show complex interactions between glial cells, neurons and ECM, potentially affecting synaptic functions and axonal conductance. Results in SZ and BD point to a profound disruption of these interactions in several brain regions.

### 10.4 DIFFUSION WEIGHTED SPECTROSCOPY STUDIES OF CELL-TYPE SPECIFIC ABNORMALITIES IN SCHIZOPHRENIA

Dost Ongur*[,1]
[1]*McLean Hospital*

**Background:** In previous work we used diffusion tensor spectroscopy (DTS) to identify abnormal diffusion of the neuron-specific metabolite NAA in frontal white matter in patients with chronic schizophrenia in the absence of abnormalities in the diffusion of cell-type non-specific metabolites Cr and Cho.
**Methods:** DTS relies on the same principles as DTI, but the diffusion characteristics of metabolites are probed, instead of those of water. Since brain metabolites are concentrated in specific cellular and sub-cellular compartments, their diffusion reflects the local geometry of these compartments. We have implemented a DTS approach at a 4 Tesla Varian MRI scanner (described in Du et al 2013).
**Results:** We have now collected similar data from first episode psychosis patients and matched healthy controls. We find that NAA diffusion is normal in the frontal PFC in first episode patients, but Cr and Cho diffusion is abnormal.
**Discussion:** Taken together, our studies suggest white matter abnormalities in non-neuronal elements in early phases of schizophrenia which are followed by neuronal damage in chronic disease.

### 11. AEROBIC EXERCISE TRAINING FOR INDIVIDUALS WITH SCHIZOPHRENIA: THE BROAD BENEFITS ACROSS PHYSICAL HEALTH, COGNITION, AND EVERYDAY FUNCTIONING AND PROMISING MECHANISMS OF ACTION

Keith Nuechterlein
*University of California, Los Angeles*

**Overall Abstract:** Recently aerobic exercise training has begun to be systematically examined in randomized controlled trials (RCTs) in schizophrenia. This symposium will report and discuss the results of RCTs that examined the impact of aerobic exercise on physical health, cognition, and everyday functioning across first-episode and established illness phases of schizophrenia. In addition, data on neurotrophic and brain structural changes will be examined as promising mechanisms of action. Dr. Amal Abdel-Baki of the University of Montreal has focused on the physical health benefits of interval training in her RCT with first episode and multi-episode schizophrenia outpatients. She is demonstrating improved waist circumference, diastolic blood pressure, HDL cholesterol, and social functioning in first episode and multi-episode patients. Dr. David Kimhy of Icahn School of Medicine at Mount Sinai in New York has focused on the impact of aerobic exercise training on cardiovascular fitness, Brain-Derived Neurotrophic Factor (BDNF), cognition, and functional outcome in individuals with an established schizophrenic illness. He has demonstrated beneficial effects at each of these levels. Furthermore, relationships between fitness improvements and BDNF increases and the cognitive and functional gains suggest potential mechanisms of action. Dr. Berend Malchow of Ludwig Maximilian University of

Munich has examined the impact of adding cognitive training to aerobic exercise in multi-episode schizophrenia patients. This combination led to increased verbal memory and improved global functioning. Increase in left temporal grey matter volume is a promising brain mechanism of action. Dr. Keith Nuechterlein from UCLA will present results from a recently completed RCT of first-episode schizophrenia patients in which aerobic exercise training was added to computerized cognitive training to determine the extent to which it could enhance the impact of cognitive training. He will show that this combination significantly enhances cognition and work/school functioning gains beyond the effect of cognitive training alone and leads to increases in prefrontal cortical thickness and functional connectivity. Furthermore, he will examine early BDNF increases in response to treatment as a predictor of later cognitive and functional improvements. Dr. Peter Falkai will lead the discussion of the promise of aerobic exercise as an intervention to improve physical health, cognition, and functional outcome in schizophrenia and consider the potential mechanisms of action.

### 11.1 EFFECT OF INTERVAL TRAINING ON METABOLIC RISK FACTORS IN OVERWEIGHT INDIVIDUALS WITH PSYCHOSIS: A RANDOMIZED CONTROLLED TRIAL

Ahmed Jérôme Romain[1], Cédine Fankam[1], Antony Karelis[2], Elaine Letendre[3], Gladys Mikolajacks[1], Emmanuel Stip[1], Amal Abdel-Baki*[,3]

*[1]University of Montreal Hospital Research Centre; [2]University of Quebec at Montreal; [3]University Hospital of Montreal*

**Background:** Among adults with psychotic disorders, negative symptoms as unhealthy lifestyle habits contribute to a high prevalence of metabolic syndrome and obesity. Lifestyle interventions, mainly physical activity (PA) has emerged as an essential component. Furthermore, interval training (IT) was found to be efficacious in other populations but poorly studied among people with psychosis.

The objective was to determine the effects of a 6-month IT program on metabolic, anthropometric, and psychiatric/functional outcomes.

**Methods:** Randomized controlled trial comparing the effects of a bi-weekly 30 minutes supervised IT program to a waiting list of overweight individuals with psychosis. Body composition and metabolic risk factors (blood pressure, insulin resistance, lipid profile) were measured at baseline and every 3 months. The groups were compared on an intent to treat basis with repeated-measures mixed linear models with the restricted maximum of likelihood method of estimation.

**Results:** Sixty-seven individuals (28 control: waiting list; 39 IT intervention) with psychosis (60.6% men, mean age: 31.0 ± 7.2 years old; BMI: 32.0 ± 6.1 kg/m$^2$, waist circumference: 107.7 ± 13.3 cm) were included in the study, and 67.2% completed the study. Attendance for the IT sessions was 61.8% and the dropout rate was 32%. IT was associated with significant improvements on waist circumference (-2.72 cm, SE = 1.34; p = 0.04), negative symptoms (-2.93, SE = 1.34; p = 0.03), social (SOFAS) (+5.23, SE = 2.39; p = 0.03) and global functioning (+7.34, SE = 2.05; p < 0.001). The effects of exercise in the first-episode psychosis (FEP) sub-group were similar to those of the entire cohort.

**Discussion:** These promising results suggest that IT may be used as a treatment strategy for the management of metabolic complications and possibly improve social functioning in obese individuals with psychotic disorders. Further studies are needed to understand if IT could prevent weight gain and metabolic complications if used before these comorbidities emerge and to understand factors associated with the persistence of exercising.

### 11.2 THE IMPACT OF AEROBIC EXERCISE ON COGNITIVE FUNCTIONING AND BIOMARKERS OF COGNITIVE CHANGE IN INDIVIDUALS WITH SCHIZOPHRENIA

David Kimhy*[,1], Julia Vakhrusheva[2], Matthew Bartels[3], Jacob Ballon[4], Eero Castrén[5], Richard Sloan[2]

*[1]Icahn School of Medicine at Mount Sinai; [2]Columbia University; [3]Albert Einstein College of Medicine; [4]Stanford University; [5]University of Helsinki*

**Background:** Individuals with schizophrenia (SZ) display substantial cognitive deficits across multiple domains. These deficits have been identified as major determinants of poor functioning and disability, representing a serious public health concern and an important target for interventions. At present, available treatments offer only minimal to limited benefits to ameliorate these deficits. Thus, there remains an urgent need to identify novel treatments for cognitive deficits in people with SZ. Emerging evidence from studies of animals, clinical and non-clinical populations suggest that Aerobic Exercise (AE) is efficacious in improving cognition via up-regulation of Brain-Derived Neurotrophic Factor (BDNF). Yet, the impact of AE on cognition and daily-functioning, and the role of BDNF, have not been investigated in schizophrenia. Additionally, limited information is available on the putative link between inflammation markers to cognitive functioning.

**Methods:** Employing a single-blind RCT design, 33 individuals with schizophrenia were randomized to receive "treatment as usual" (n=17; TAU) or attend a 12-week, 3 times-per-week, 60-minutes AE program (n=16) utilizing active-play video-games (Xbox-360 Kinect) and traditional AE equipment.

**Results:** At baseline, cognitive functioning was associated with serum BDNF (r=.51, p=.01), along with TNF-alpha (r=-.38, p=.03), IL-12 (r=-.36, p=.04) and IL-6 (r=-.33, p=.06). Twenty-six participants completed the study (79%). Following the intervention, the AE participants improved their cognitive functioning (MCCB) by 15.1% (vs. -2.0% in the TAU group; p=.03). Hierarchical multiple-regression analyses indicated changes in AF and serum BDNF predicted 25.4% and 14.6% of the cognitive improvement, respectively. Additionally, changes in aerobic fitness (VO2peak ml/kg/min) correlated with informant-reported improvements in work-related daily-functioning skills (SLOF; r=.51, p=.01). Fidelity with target training intensity, was correlated with cognitive improvement (r=.70, p=.02).

**Discussion:** The results indicate AE is effective in enhancing cognitive and daily functioning skills in people with schizophrenia and provide support for the impact of AE-related BDNF up-regulation on cognition. Additional studies are needed to establish the link between inflammation markers and cognitive functioning and the potential impact of AE on this putative pathway. Overall, low aerobic fitness represents a modifiable risk-factor for cognitive dysfunction in schizophrenia for which AE training offer a relatively safe, non-stigmatizing, and side-effect-free intervention.

### 11.3 CLINICAL AND NEUROBIOLOGICAL EFFECTS OF A CONTINUOUS AEROBIC ENDURANCE TRAINING IN MULTI-EPISODE SCHIZOPHRENIA PATIENTS

Berend Malchow*[,1], Sergi Papiol[1], Daniel Keeser[1], Boris Rauchmann[1], Katriona Keller-Varady[1], Alkomiet Hasan[1], Andrea Schmitt[1], Peter Falkai[1]

*[1]Ludwig Maximilian University, Munich*

**Background:** Structural and functional brain alterations as well as cognitive deficits are well-documented findings in schizophrenia patients. Cognitive impairments affect the long-term outcome of schizophrenia and are the main contributors to disability. Aerobic endurance training has been shown to have effects on brain plasticity, gray and white matter volume as well as functional connectivity measures and on cognitive functioning in animal models and healthy humans. However, effects of physical exercise in combination in combination with cognitive remediation (CR) are unknown in schizophrenia.
**Methods:** 21 chronic schizophrenia patients and 21 age- and gender-matched healthy controls underwent 3 months of aerobic exercise (endurance training, 30 min, 3 times per week). 21 additionally recruited schizophrenia patients played table soccer (known as "foosball" in the USA) over the same period. After 6 weeks of endurance training or table soccer, all participants commenced standardized cognitive training with a computer-assisted training program. Clinical symptoms, thorough neuropsychological testing and multimodal neuroimaging with 3D-volumetric T1-weighted sequences, DTI and magnetic resonance spectroscopy (MRS) were performed on a 3T MR scanner at baseline and after the 3-month intervention and 3 additional training-free months. DNA from all subjects was genotyped with the Infinium PsychArray Chip (Illumina, San Diego, CA, USA). Polygenic risk scores were calculated and associated with hippocampal subfield volume change.
**Results:** In summary, a 3-month endurance training program combined with CR therapy for the last 6 weeks of the intervention period was feasible (Keller-Varady et al. 2016) and had positive effects on everyday functioning in multi-episode schizophrenia patients. Deficits improved from medium to mild as assessed with the GAF. Negative symptoms, short- and long-term verbal memory and cognitive flexibility also improved with endurance and cognitive training (Malchow et al. 2015). We could demonstrate grey matter volume increase in the left temporal lobe in schizophrenia patients undergoing endurance training. A non-endurance and coordinative training stimulus like playing table soccer led to a clearly distinct pattern of grey matter alterations in schizophrenia patients (Malchow et al. 2016). There were no effects of the intervention on structural and functional brain networks in schizophrenia patients as well as MRS measures (in preparation). No effects of PRSs were found on total hippocampal volume change. Subfield analyses showed that the volume changes between baseline and 3 months in the left CA4/DG were significantly influenced by PRSs in schizophrenia patients performing aerobic exercise. A larger genetic risk burden was associated with a less pronounced volume increase or a decrease in volume over the course of the exercise intervention. Results of exploratory enrichment analyses reinforced the notion of genetic risk factors modulating biological processes tightly related to synaptic ion channel activity, calcium signaling, glutamate signaling and regulation of cell morphogenesis (Papiol et al. 2017).
**Discussion:** Exercise interventions are feasible and effective interventions for people with schizophrenia and might also help to disentangle the underlying brain pathology of the disorder.

### 11.4 AEROBIC EXERCISE ENHANCES COGNITIVE TRAINING EFFECTS IN FIRST EPISODE SCHIZOPHRENIA: COGNITIVE AND FUNCTIONAL GAINS AND PROMISING BIOLOGICAL MECHANISMS OF ACTION

Keith Nuechterlein*[,1], Sarah McEwen[1], Joseph Ventura[1], Kenneth Subotnik[1], Luana Turner[1], Michael Boucher[1], Laurie Casaus[1], Jacqueline Hayata[1]
[1]*University of California, Los Angeles*

**Background:** The search for treatments to remediate cognitive deficits and their functional outcome consequences remains a critical frontier in schizophrenia. Cognitive training and aerobic exercise both show promising moderate impact on cognition and everyday functioning. Aerobic exercise is hypothesized to increase brain-derived neurotrophic factor (BDNF) and thereby stimulate neurogenesis and synaptic plasticity, leading to increased learning capacity. Systematic cognitive training should take advantage of increased learning capacity and be more effective when combined with aerobic exercise.
**Methods:** In a recently completed randomized controlled trial, we examined the impact of a 6-month program of Cognitive Training & Exercise (CT&E) compared to Cognitive Training alone (CT) in 47 first-episode schizophrenia outpatients. All participants were provided the same Posit Science computerized cognitive training, four hours/week, using BrainHQ and SocialVille programs. The CT&E group also participated in total body circuit training exercises to enhance aerobic conditioning. The exercise intensity was in the 60–80% of aerobic capacity range, combining clinic and home-based exercise for a target of 150 minutes per week.
**Results:** Mixed model analyses demonstrate that the MATRICS Consensus Cognitive Battery Overall Composite improves significantly more by 3 months with CT&E than with CT alone (6.6 vs. 2.2 T-score points, $p<.02$). Work/school functioning improves substantially more with CT&E than with CT alone by 6 months ($p<.001$). BDNF is a promising mechanism of action, improving even after 2 weeks and predicting the amount of cognitive gain at 3 months. The magnitude of cognitive gain by 3 months predicts the amount of work/school functioning improvement at 6 months, suggesting a cascade of effects. Analyses by Dr. McEwen show differential increases in cortical thickness in the left dorsal lateral prefrontal gyrus ($p=.02$) and right superior frontal gyrus ($p=.02$) over 6 months and increased functional connectivity in the central executive network ($p=.04$) with CT&E compared to CT alone and correlations of these increases with cognitive and functional outcome gains.
**Discussion:** We conclude that aerobic exercise significantly enhances the impact of cognitive training on cognition, functional outcome, and frontal cortical thickness in first-episode schizophrenia and that BDNF is a promising mechanism of action for these effects.

### 12. SYNAPTIC DYSFUNCTION IN SCHIZOPHRENIA: EXPLORATION OF NOVEL HYPOTHESES AND PROMISING NEW LEADS

Laura Rowland
*University of Maryland School of Medicine*

**Overall Abstract:** Accumulating evidence suggests that bioenergetic function is impaired in the brain in schizophrenia. In normal brain, glucose is metabolized to lactate and pyruvate, which are monocarboxylate intermediates that serve as the primary energy source for neurons. Working memory and other cognitive domains are dependent on the shuttling of lactate from astrocytes to neurons. Defects in this complex pathway may underlie cognitive dysfunction in schizophrenia. The focus of this symposium is to present evidence of such defects, and to identify substrates that may be targeted for the development of new treatment strategies. Dr. Laura Rowland (University of Maryland, Baltimore, Maryland, USA) will present evidence of bioenergetic dysfunction in living subjects with schizophrenia. Increased levels of lactate ($P < 0.05$) were present in the ACC in schizophrenia ($n = 27$) compared to controls ($n = 29$). Higher lactate levels were associated with lowers scores on the MATRICS Consensus Cognitive Battery. These data establish a direct link between cognition and bioenergetic function in vivo in schizophrenia. Dr. Robert McCullumsmith (University of Cincinnati, Cincinnati, Ohio, USA) will present evidence of alterations in the lactate shuttle and glycolytic enzymes in postmortem samples from schizophrenia ($n = 20$) and control subjects ($n = 20$). Cell-subtype specific changes ($P < 0.05$) in transcripts include increased levels of the lactate transporter MCT4, decreased levels of the glycolytic enzymes PFK1 and hexokinase, and decreased levels of the glucose transporters Glut1 and Glut3. These data suggest attenuated glycolysis in pyramidal neurons, with a shift towards pathways that boost protection from oxidative stress.

The last two speakers will present data that address mechanisms related to these findings, using animal models with behavioral endophenotypes of schizophrenia. Dr. Eduard Bentea (Free University of Brussels (VUB), Brussels, Belgium) will present data from the xCT knockout mouse showing that disruption of system xc-, which supports oxidative stress buffering mechanisms, leads to synaptic dysfunction. Specifically, electron microscopy studies indicate depletion of both pre-and post-synaptic glutamate, while electrophysiological studies show diminished excitatory postsynaptic potentials. These findings directly connect oxidative balance and extracellular glutamate levels with development of "broken" synapses, highlighting a potential mechanism for perturbation of bioenergetic coupling between astrocytes and neurons. Dr. Amy Ramsey (University of Toronto, Toronto, Canada) will present evidence from an animal model of synaptic dysfunction, the GluN1 knockdown mouse. These mice show a bioenergetic defect similar to schizophrenia, with decreased expression of glycolytic enzymes and glucose transporters. These translational findings indicate that genetic risk for schizophrenia may lead to an intermediate bioenergetic phenotype, where diminished supply of lactate and other energetic molecules to neurons could contribute to cognitive dysfunction. Taken together, the work presented by these speakers will provide a fresh look at the bioenergetic defects in schizophrenia, establishing that 1) metabolic perturbations in the brain are prominent and not just an effect antipsychotic treatment, 2) altered neuron-astrocyte coupling leads to synaptic dysfunction, and 3) genetic risk for "broken" synapses disrupts metabolic function.

### 12.1 CELL-SUBTYPE SPECIFIC BIOENERGETIC DEFECTS IN SCHIZOPHRENIA

Courtney Sullivan[1], Robert McCullumsmith*[,1]
*[1]University of Cincinnati*

**Background:** Novel insights into the pathophysiology of schizophrenia are needed to move the field forward by providing the conceptual framework to facilitate development of new treatment strategies. It is well established that glutamatergic systems are disrupted in schizophrenia, which are intimately linked to metabolic function. While there are many promising new directions, accumulating evidence suggests that bioenergetic function is impaired in the brain in schizophrenia. There are multiple mechanisms in the brain to meet neuronal energy demands, including glycolysis, lactate uptake, and oxidative phosphorylation. In normal brain, neurons and astrocytes are coupled through the astrocyte-neuron lactate shuttle, where astrocytes metabolize glucose to lactate and pyruvate, primary energy substrates that are transported to neurons via monocarboxylate transporters (MCTs). Lactate generated by glycolysis in glial cells constitutively supports synaptic transmission even under conditions in which a sufficient supply of glucose and intracellular adenosine triphosphate (ATP) are present. Interestingly, working memory and other cognitive domains are dependent on the shuttling of lactate from astrocytes to neurons. This process highlights the bioenergetic coupling between astrocytes and neurons that develops as the brain matures, forming a critical biological process in the mature adult brain. We assessed elements of these systems in postmortem brain, testing the hypothesis that there are cell-subtype defects in bioenergetics function in the frontal cortex in schizophrenia.
**Methods:** Well-validated assays were used to assess the activity of three glycolytic enzymes in postmortem dorsolateral prefrontal cortex (DLPFC) samples (n=16/group): lactate dehydrogenase (LDH), hexokinase (HXK), and phosphofructokinase (PFK). Each sample was assayed with and without a specific inhibitor (in duplicate) and normalized to protein loaded into the assay. We also probed for differences in protein expression using western blot analysis. Western blot analyses were run in duplicate using the following antibodies optimized for postmortem brain: MCT1, LDH, LDHA, LDHB, HXK1, glucose transporter 3 (GLUT3). We performed real time quantitative polymerase chain reaction (RT-qPCR) using TaqMan PCR assays (MCT1, MCT4, HXK1, HXK2, LDHA, LDHB, PFK1, GLUT1, and GLUT3) in duplicate on cDNA samples in 96-well optical plates on a Stratagene MX3000P (Stratagene, La Jolla, California). We also coupled laser capture microdissection (LCM) with RT-qPCR from superficial and deep layers of DLPFC using the Veritas Microdissection instrument and CapSure Macro LCM caps (Life Technologies, formerly Arcturus, Mountain View, CA, USA). Similar studies were performed in haloperidol-decanoate or vehicle (sesame oil) treated rats (intramuscular injection every 3 weeks for 9 months).
**Results:** We found a 24% decrease in PFK1 mRNA expression in the dorsolateral prefrontal cortex in schizophrenia ($p=0.039$). We also found decreases in HXK (26%, $p=0.002$) and PFK (16%, $p<0.001$) activity in the dorsolateral prefrontal cortex. These changes were not present in haloperidol treated rats. At the cell-level, in pyramidal neurons we found an increase in MCT1 mRNA expression (22%, $p= 0.038$), and decreases in HXK1 (19%, $p= 0.023$), PFK1 (22%, $p=0.003$), GLUT1 (20%, $p=0.008$), and GLUT3 (20%, $p=0.023$) mRNA expression. We found increases in MCT1 (17%, $p<0.05$) and GLUT3 (20%, $p<0.05$), but not HXK1, PFK1, or GLUT1, mRNA expression in enriched pyramidal neuron samples of antipsychotic treated rats.
**Discussion:** As the brain develops, bioenergetic organization and the formation of synapses occur simultaneously, creating a fundamentally interdependent system. There is accumulating evidence of implicating a number of abnormalities associated with glucose metabolism, the lactate shuttle, and bioenergetic coupling in schizophrenia, suggesting energy storage and usage deficits in the brain. Bioenergetic deficits and genetic risk for synaptic dysfunction in schizophrenia could contribute to the pathophysiology of this illness. In normal brain, glucose enters cells through GLUTs and is processed by glycolytic enzymes resulting in bioenergetic substrates such as pyruvate. Pyruvate can then be converted to lactate and transported between cells or intracellularly by MCTs to be oxidized in the TCA cycle when neuronal energy demand is high. Our findings of decreased glycolytic enzyme and lactate transporter mRNA expression suggests a decrease in the capacity of pyramidal neurons to generate bioenergetic substrates from glucose via glycolytic pathways. Additionally, if neurons were unable to take up adequate amounts of glucose for glycolysis, the intracellular pool of available pyruvate/lactate for transport into mitochondria may be diminished, ultimately impacting energy supply. It is also possible that there is attenuated glycolysis in pyramidal neurons, with a shift towards pathways that boost protection from oxidative stress (pentose phosphate pathway). Other studies also report region and cell-subtype specific changes in the expression of genes encoding proteins involved in metabolism in this illness. Importantly, the above changes were not attributable to antipsychotic treatment. Both synaptic function and meeting of energetic demands are essential for cognition, and failure of either could contribute to the cognitive symptoms seen in schizophrenia. Augmenting affected systems such as glucose utilization pathways could offer a novel approach to restoring cognitive function in schizophrenia. This could include targeting pro-metabolic substrates pharmacologically.

### 12.2 METABOLIC CONSEQUENCES OF DEVELOPMENTAL NMDA RECEPTOR HYPOFUNCTION

Adam Funk[1], Catharine Mielnik[2],
Sinead O'Donovan[1], Courtney Sullivan[1],
Yuxiao Chen[2], Robert McCullumsmith[1], Amy Ramsey*[,2]
*[1]University of Cincinnati; [2]University of Toronto*

**Background:** Several imaging and postmortem studies provide evidence that, in the brains of people with schizophrenia, there are alterations in glucose metabolism and energy utilization. However, it is difficult to determine whether altered excitatory transmission alters bioenergetics that then contributes to symptoms of the disorder. We have used a mouse model to begin to address these questions. GluN1 knockdown mice have a mutation that reduces NMDA receptor levels throughout development and maturity.

**Methods:** We affinity purified PSD95 protein complexes from GluN1KD and WT brains (n=3 per group) and ran each sample through our liquid chromatography tandem mass spectrometry (LC-MS/MS) protocol in singlicate. We performed pathway analysis with the EnRICHr suite of bioinformatic tools and compared WT to GluN1KD PSD95 interactomes using the top 20 differentially expressed proteins. We also studied how NMDA receptor hypofunction changes the expression of genes related to glucose metabolism and bioenergetics by quantitative PCR of brain cDNA from WT and GluN1 knockdown mice.
**Results:** Pathway analysis revealed that WT mice showed pathways relevant for synaptic plasticity (as expected), while GluN1KD analyses yielded proteins related to glucose metabolism and utilization. Gene expression analysis revealed that GluN1 knockdown mice have significant decreases in the expression of Slc16a3, Slc2a1, and Slc2a3, which are the genes for the monocarboxylate transporter (MCT4), and glucose transporters 1 and 3 (GLUT1 and GLUT3).
**Discussion:** Our results show that NMDA receptor dysfunction leads to expression changes that would reduce glucose and lactate transport into neurons. The synaptic proteome of NMDAR deficient mice shows an increase in glycolytic enzymes located at the synapse. These data suggest a profound shift in the composition of the cortical excitatory synaptic proteome in GluN1KD mice, with apparent increases in neuroenergetic substrates in neurons. At the same time, there were significant decreases in the levels of transporters that bring glucose and the primary energy substrate, lactate, into neurons. The MCT4 shuttles lactate from astrocytes to neurons, which can then be used for oxidative respiration in neurons. GLUT1 is responsible for transport of glucose across the blood-brain-barrier, and GLUT3 is expressed on neurons and is responsible for glucose uptake in those cells. Notably, we have identified that these transporter gene transcripts are reduced in postmortem brains of people with schizophrenia. Thus, this mouse may be a useful tool to model bioenergetic changes that are observed in schizophrenia, and study functional outcomes when glucose metabolism is improved.

### 12.3 SYSTEM XC- AS A NOVEL MODULATOR OF CORTICOSTRIATAL NEUROTRANSMISSION

Eduard Bentea*[,1], Cynthia Moore[2], Agnès Villers[3], Madeline J Churchill[2], Rebecca L Hood[4], Lauren Deneyer[1], Lise Verbruggen[1], Giulia Albertini[1], Hideyo Sato[5], Laurence Ris[3], Charles K Meshul[6], Ann Massie*[,1]
*[1]Vrije Universiteit Brussel; [2]VA Medical Center, Portland; [3]Université de Mons; [4]Oregon Health & Science University; [5]Niigata University,[6]Oregon Health & Science University, VA Medical Center*

**Background:** System xc- is a plasma membrane amino acid antiporter, of mainly glial origin, that couples the import of cystine with the export of glutamate. System xc- (specific subunit xCT) contributes substantially to ambient extracellular glutamate levels in various regions of the brain, including the striatum and hippocampus. Despite the fact that system xc- is highly expressed in the brain and is a proposed therapeutic target for various neurological disorders, its function under physiological conditions in the central nervous system remains poorly understood. By acting as a source of glial extrasynaptic glutamate, system xc- might modulate synaptic transmission as a mechanism of neuro-glial communication. Previous electrophysiological findings indicate that system xc- delivered glutamate can inhibit excitatory synaptic neurotransmission in the corticoaccumbens pathway and at hippocampal CA3-CA1 synapses. To gain further insight into the proposed function of system xc- as modulator of synaptic transmission, we here focus on corticostriatal synapses.
**Methods:** Single section electron microscopy was carried out on VGLUT1-pre-embed and glutamate immunogold post-embed labeled slices of the dorsolateral striatum of xCT+/+ and xCT-/- mice. Various parameters related to the pre- and post-synaptic compartments were integrated on the obtained electron micrographs, including glutamate immunogold density in the presynaptic terminal and spine, area of the terminal and spine, measures of the postsynaptic density (PSD) (length, area, thickness, and maximum thickness), percentage of PSDs showing perforations, and width of the synaptic cleft. Electrophysiological measures of corticostriatal transmission were obtained by recording the amplitude of field excitatory postsynaptic potentials (fEPSPs) after stimulation of corticostriatal fibers. Finally, grooming behavior was compared between xCT-/- and xCT+/+ littermates.
**Results:** Genetic deletion of xCT led to depletion of glutamate immunogold labeling from corticostriatal terminals and their corresponding dendritic spines. Absence of xCT did not, however, affect the morphology of corticostriatal synapses, as evaluated by the area of the terminals and spines, size of the PSD, and width of the synaptic cleft. Similarly, no changes could be observed in the density of VGLUT1-positive synapses, indicating normal cortical innervation and spine density. Electrophysiological recordings revealed decreased amplitude of fEPSPs in xCT-/- mice after stimulation of corticostriatal fibers. Preliminary investigations revealed that this reduced response can be rescued by restoring physiological levels of glutamate to xCT-/- slices. Changes in corticostriatal transmission were not reflected in aberrant grooming behavior in xCT-/- mice; we could not observe any difference in the total grooming duration, the number of grooming bouts, the average bout duration or the latency to onset to grooming between xCT-/- and xCT+/+ mice.
**Discussion:** Contrary to available evidence at hippocampal and corticoaccumbens pathways, our findings indicate a positive effect of system xc- on basal synaptic transmission at corticostriatal synapses. The decreased response we observed after stimulation of corticostriatal fibers in xCT-/- mice was accompanied by depletion of glutamate immunogold labeling from corticostriatal terminals, suggesting a possible defect in presynaptic glutamate handling. Given the strong decrease (70%) in extracellular glutamate levels previously reported in this strain of mice, we hypothesize that the decreased presynaptic glutamate labeling in xCT-/- mice is related to a loss of extracellular glutamate needed to supply terminals for proper excitatory transmission. This hypothesis is supported by our preliminary results showing increased responses in xCT-/- slices after restoring physiological levels of glutamate. Together, our findings shed new light on the role of system xc- in controlling synaptic transmission, and suggest that it may play an important role in supplying presynaptic terminals with glutamate as an alternative mechanism to the glutamate-glutamine cycle. As a novel modulator of corticostriatal transmission, system xc- may be of interest as a possible therapeutic target for disorders with a corticostriatal component, such as schizophrenia or obsessive-compulsive disorder.

### 12.4 BRAIN LACTATE IS RELATED TO COGNITION IN SCHIZOPHRENIA

Laura Rowland*[,1], Andrea Wijtenburg[2], Subechhya Pradhan[3], Stephanie Korenic[2], Richard Edden[3], Elliot Hong[2], Peter Barker[3]
*[1]University of Maryland School of Medicine; [2]Maryland Psychiatric Research Center; [3]Johns Hopkins University*

Laura Rowland, University of Maryland School of Med: Bioenergetic function may be altered in schizophrenia as supported by post-mortem, preclinical, cerebrospinal fluid, and 31P-magnetic resonance spectroscopy (MRS) research. Impairments in bioenergetic function may lead to cognitive and functional dysfunction, characteristics of the illness. First, a 7T MRS study that tested the hypothesis that frontal lactate concentrations are elevated in schizophrenia and related to cognitive impairments will be presented. Second, recent advances in brain lactate measurements with 3T MRS will be presented.

**Methods:** Twenty-nine controls and 27 participants with schizophrenia completed the study. MRS scanning was conducted on a Philips 'Achieva' 7T scanner, and spectra were acquired from a frontal voxel using STEAM (TE/TM/TR=14/33/3000 ms, 128 NEX, 16 NEX water). Participants completed the MATRICS Consensus Cognitive Battery (MCCB) for cognitive function and UCSD Performance-Based Skills Assessment (UPSA) for functional capacity. The relationships between lactate, MCCB, and UPSA were examined. 3T MRS test-retest measures of lactate were conducted on a Siemens Prisma scanner using spectra editing (TE/TR=140/3, editing pulse at 4.1ppm with 30Hz bandwidth, 360 NEX, 16 NEX water).
**Results:** Patients had significantly higher lactate compared to controls (p = 0.045). Higher lactate was associated with poorer general cognitive function (r=-0.36, p=0.01) Visual learning, processing speed, and reasoning/problem solving cognitive domains showed the strongest relationships with lactate. Poorer functional capacity (r=-0.43, p=0.001) was also related to higher lactate. 3T spectral editing studies showed excellent reproducibility with a mean coefficient of variation of 4%.
**Discussion:** Higher frontal lactate levels in schizophrenia support the hypothesis that brain bioenergetics are altered and related to cognitive and functional impairments in schizophrenia. Higher lactate could be due to inefficient aerobic metabolism causing a shift towards anaerobic metabolism or poor utilization of lactate. Lactate measurements are doable at 3T field strength and may be a useful biomarker of cognition in schizophrenia. Interventions to promote efficient mitochondrial energy metabolism may prove useful for enhancing cognition and alleviating functional impairments in schizophrenia.

### 13. ENDOCANNABINOID MODULATION OF DOPAMINE NEUROTRANSMISSION

Michael Bloomfield
*University College London*

**Overall Abstract:** There are converging lines of evidence that the endocannabinoid system is involved in the pathophysiology of schizophrenia and that understanding these mechanisms may lead to novel treatment targets. In this symposium, we will present a series of experiments that link cannabinoid pharmacology to major fields of schizophrenia research including the dopaminergic, glutamatergic and serotonergic systems, glial cell function and the genetics of cognition.
Dopamine is a major neurotransmitter implicated in the pathophysiology of schizophrenia. Thus, understanding processes that modulate dopaminergic signalling may lead to new insights into the biology and treatment of this disorder. The endocannabinoids anandamide and 2-arachidonoylglicerol (2-AG) modulate dopaminergic neural activity through interactions with CB1 and CB2 receptors. CB1 antagonists inhibit the effects of drugs that potentiate dopaminergic activity, such as cocaine. There is also evidence of interactions between CB1 and CB2 receptors in terms of cannabinoid-mediated changes in dopaminergic function. We will present new evidence that CB2 receptor antagonism opposes the inhibitory effects of rimonabant on cocaine-induced hyperlocomotion. Thus, highlighting the co-modulatory role of CB1 and CB2 receptors on dopaminergic function.
We will then present a new study investigating the antipsychotic mode of action of cannabidiol (CBD). CBD attenuates the psychotomimetic effects of delta-9-tetrahydrocannabinol (THC) and there is evidence that CBD has antipsychotic effects in patients with psychosis. CBD prevents a range of behavioural impairments associated with the NMDA hypofunction model of psychosis measured in the pre-pulse inhibition, social interaction and novel object recognition tests following a two week exposure to the NMDA antagonist MK801. In addition, CBD, prevented neural (measured by delta-FosB) and microglia activation, and the decreased decrease in the number of medial prefrontal parvalbumin-positive neurons. The effects of CBD were blocked by pre-treatment with the 5-HT1A receptor antagonist WAY100635. This indicates that the antipsychotic effects of CBD may be mediated via 5HT1A-mediated mechanisms.
Next, we will describe the effects of CBD on glial cells. Glial cells, which express CB1 and CB2 receptors and synthesise endocannabinoid transmitters, have been implicated in schizophrenia whereby oligodendrocyte dysfunction has been associated with white matter deficits in the illness. In an investigation of the effects of CBD on a human oligodendrocyte culture (MO3.13), CBD administration resulted in diverse changes in the expression of proteins implicated in the pathophysiology of schizophrenia.
Finally, we provide further evidence that polymorphisms in cannabinoid receptor genes are associated with cognitive impairments in humans. In particular, the rs12720071 polymorphism T/T allele is associated with impaired working memory in patients with psychosis.

### 13.1 ENDOCANNABINOID MODULATION OF DOPAMINE NEUROTRANSMISSION

Fabricio Moreira*[,1]
[1]*Federal University of Minas Gerais*

**Background:** Dopamine is the major neurotransmitter implicated in schizophrenia pathology. Thus, understanding the processes modulating dopaminergic signalling may lead to new insights in the biology and treatment of this disorder. The endocannabinoids anandamide and 2-arachidonoyl-glicerol (2-AG) modulate neural activity through interactions CB1 and CB2 receptors. CB1 antagonists inhibit the effects of drugs that facilitate dopamine activity, such as cocaine. Similar to CB1 antagonists, CB2 agonists counteract the effects of cocaine in experimental animals. However, the functions of these receptors have been investigated separately. Here we test the hypothesis that CB1 and CB2 receptors interact to ameliorate the behavioural and molecular processes altered under hyperdopaminergic states. We also sought to identify the endocannabinoid involved in these effects.
**Methods:** Male Swiss mice received cocaine injections to increase dopamine activity in the brain. The biological responses measured were hyperlocomotion, conditioned place preference, cFos expression and Erk protein phosphorylation in the nucleus accumbens. The animals received cannabinoid-related drugs before cocaine injections. The data were analysed with ANOVA followed by the Newman-Keuls test.
**Results:** The CB1 receptor antagonist, rimonabant, and the CB2 receptor agonist, JWH133, inhibited cocaine-induced hyperlocomotion. Moreover, the CB2 antagonist, AM630, reversed the inhibitory effects of rimonabant in cocaine-induced hyperlocomotion, cFos expression, Erk phosphorylation and conditioned place preference. The inhibitors of anandamide and 2-AG hydrolysis, URB597 (FAAH inhibitor) and JZL184 (MGL inhibitor), respectively, were ineffective in inhibiting cocaine hyperlocomotion. However, when combined with a sub-effective dose of rimonabant, JZL184 (but not URB597), inhibited cocaine effects.
**Discussion:** A CB2 antagonist reversed the effect of a CB1 antagonist, suggesting that these receptors modulate cocaine effects in opposite ways. Accordingly, increasing brain 2-AG levels inhibited cocaine effects only if CB1 is blocked and CB2 available. Thus, selective activation of CB2 receptors warrants further investigation as a new strategy for the treatment of psychiatric disorders resulting from hyperdopaminergic states.

### 13.2 CANNABIDIOL AS AN ANTIPSYCHOTIC DRUG

Francisco Guimaraes*[,1], Naielly Rodrigues[1], Nicole Silva[1], Felipe Gomes[2]
[1]*School of Medicine of Ribeirao Preto-University of Sao Paulo;*
[2]*University of Pittsburgh*

**Background:** The phytocannabinoid cannabidiol (CBD) attenuates the psychotomimetic effects produced by high doses of delta-9-tetrahydrocannabinol (THC), the main component of the Cannabis sativa plant.

Corroborating this effect, several preclinical and clinical studies indicate that CBD has antipsychotic properties. The mechanisms responsible for these properties, however, remain unknown (Campos et al., Philos Trans R Soc Lond B Biol Sci 367:3364–782013). We have recently found that repeated CBD administration prevents the behavioral impairments, measured in the pre-pulse inhibition, social interaction and novel object recognition tests, induced in mice by repeated treatment (28 days) with the NMDA receptor antagonist MK-801. CBD also prevented the neural (measured by delta-FosB) and microglia activation, and the decrease in the number of parvalbumin-positive neurons, observed in the medial prefrontal cortex (Gomes et al., Int J Neuropsychopharmacol 18(5)2014, Schizophr Res 164:155–63, 2015). Currently, we are investigating if CBD could also reverse these changes once they have been established and the possible mechanisms of this effect.

**Methods:** Male C57BL/6J mice received intraperitoneal injections of MK-801 (0.25, 0.5 or 1 mg/kg, twice a day) for 7 or 14 days. To determine if these treatments regime would induce acute and long-term deficits, the social interaction (SI) test was performed 1 or 8 days after the end of the MK-801 treatment. Twenty-four hours after the SI, animals were submitted to the novel object recognition (NOR) test. Having established that 14 days of MK-801 induce both acute (24 h after) and long-term (8 days after) behavioral deficits in the SI and NOR tests, we investigated if repeated treatment with CBD (15, 30 or 60 mg/kg daily, i.p.) would reverse these changes. CBD treatment began 24h after the end of the MK-801 treatment and lasted for 7 days. Repeated clozapine (1 mg/kg) was used as a positive control. Forty-eight hours after the last injection, animals were submitted to SI and, 24-h later, to the NOR test. In a second experiment, independent groups of mice received, before each CBD injection, AM251 (a CB1 receptor inverse agonist, 0.1–0.3 mg/kg), AM630 (a CB2 receptor inverse agonist, 0.1–0.3 mg/kg), or the 5HT1A receptor antagonist WAY100635 (0.1–0.3 mg/kg). The data were analyzed by ANOVA followed by the Newman-Keuls test.

**Results:** MK-801 (0.5 mg/kg) administration for 14 days, but not for 7 days, impaired SI and NOR. Repeated CBD or clozapine treatment reversed these impairments. CB1 and CB2 antagonists (AM251 and AM630, respectively) failed to change CBD effect. However, its effect was blocked by pretreatment with the 5HT1A receptor antagonist WAY100635.

**Discussion:** Our findings show that a 2-week treatment with the NMDA receptor antagonist MK801 impairs social interaction and novel object recognition, which have been associated with negative and cognitive symptoms of schizophrenia, respectively. These behavioral deficits last for at least one week and are reversed by the atypical antipsychotic clozapine or CBD, reinforcing the proposal that this latter drug has antipsychotic-like properties. CBD effects seem to depend on facilitation of 5HT1A-mediated neurotransmission.

### 13.3 EFFECTS OF CANNABINOIDS ON A HUMAN OLIGODENDROCYTE CULTURE: IMPLICATIONS FOR SCHIZOPHRENIA

Valéria Almeida*[,1], Daniel Martins-De-Souza[1]
[1]*University of Campinas (UNICAMP)*

**Background:** Preclinical studies have suggested the involvement of the endocannabinoid system in schizophrenia pathobiology. The effects of cannabinoid drugs in several animal models for schizophrenia have been used to understand the pathobiology of the disease, and to investigate potential treatments for schizophrenia symptoms. Alterations in endocannabinoid (eCB) signaling, such as cannabinoid receptor expression and anandamide levels, have also been investigated in animal models. In addition, in vitro studies have shown the molecular pathways and biological processes associated with cannabinoids' effects in some cell types, such as glial cell cultures. Glial cells, which express cannabinoid CB1 and CB2 receptors and synthesize eCBs, have been shown to be implicated in schizophrenia. Thus, the effects of cannabinoid drugs on these cells may contribute to our knowledge about the pathobiology of schizophrenia. Specifically, oligodendrocytes are associated with white matter deficits in schizophrenia. The modulation of their function, survival, and differentiation can result in new approaches to treat schizophrenia's white matter-associated deficits. Here we have investigated the effects of cannabidiol (CBD) on a human oligodendrocyte culture (MO3.13) in terms of protein expression.

**Methods:** MO3.13 oligodendrocytes were treated with CBD (1µM) for 8h. Proteins were extracted from these cells, digested, and processed in a state-of-the-art LC-MS/MS system. Quantitative proteomics approaches were then employed in a label-free fashion. Differentially expressed proteins among the CBD treatment and controls were analyzed using systems biology in silico tools.

**Results:** Analyses identified that several proteins were up- or down-regulated in response to CBD treatment. These proteins were analyzed in terms of biological processes, pathways, and functions. CBD affected the expression of 136 proteins. Some proteins such as the transient receptor potential channel (TRPM7), microtubule-associated proteins (MAP2 and MAP4), Rho GTPase activating proteins (21 and 23), and calcium channel voltage-dependent T type alpha 1H (CACNA1H), among others possibly involved in schizophrenia pathobiology, were increased by CBD-treatment.

**Discussion:** Studies have shown the effects of CBD on the treatment of schizophrenia; but the mechanisms involved in its antipsychotic properties are not fully understood. Herein, we observed that CBD modulated the expression of proteins that can be implicated in schizophrenia pathobiology. For instance, MAPs functions are related to cytoskeleton organization, differentiation, and migration of oligodendrocytes. Studies have shown a decrease of MAPs in schizophrenia patients; thus, increasing MAP2 and MAP4 by CBD may be an interesting mechanism to treat and prevent cytoskeleton impairments in oligodendrocytes and neurons in schizophrenia. Moreover, CBD increased the voltage gated channel (CACNA1H) that is involved in cannabinoid retrograde signaling and glutamate and GABAergic neurotransmission. CACNA1H modulates $Ca2+$ levels and the synaptic vesicle cycle. To note, we also found effects of CBD on pathways and biological processes involved with schizophrenia pathobiology, such as glucose metabolism, axon guidance, and inflammation mediated by cytokine signaling. In summary, these proteomic findings may provide an integrated picture of the role of endocannabinoid signaling in oligodendrocyte cells and possible implications for schizophrenia's pathobiology.

### 13.4 CANNABINOID RECEPTOR GENE POLYMORPHISMS AND COGNITIVE PERFORMANCE IN PATIENTS WITH SCHIZOPHRENIA

Joao Salgado*[,1], Rodrigo Ferretjans[1], Bruna Panizzutti[2], Eduarda Rosa[2], Clarissa Gama[2]
[1]*Federal University of Minas Gerais;* [2]*Federal University of Rio Grande do Sul*

**Background:** Cognition is a major determinant of functioning in patients with schizophrenia. There is evidence that the endocannabinoid system influences cognition in human subjects, and participates in the pathophysiology of schizophrenia. In a previous study, we have shown that the expression of cannabinoid receptors (CB1R and CB2R) on peripheral lymphocytes is inversely correlated with performance in the Brief Assessment of Cognition in Schizophrenia (BACS), in patients with schizophrenia. Recently, CBRs polymorphisms have been associated with an increased risk for schizophrenia, structural changes in the central nervous systems and in cognitive performance of the patients. The aim of the present study was to investigate the association between CBRs polymorphisms and cognitive performance as assessed by the BACS.

**Methods:** A sample of 85 stable medicated patients (61% men; age = 41.6 ± 12.2 years; illness duration = 12.8 ± 10.7 years) was enrolled in this study. Two CB1R polymorphisms (rs1049353; rs12720071) and one CB2R polymorphism (rs2229579) were tested.

**Results:** We did not find any difference in general cognitive performance (BACS total score) regarding the three polymorphisms tested. However, when we analysed specific cognitive domains we have found a significant difference (p=0.002) regarding working memory (assessed by the Digit Span test) in patients with the rs12720071 polymorphism, where those with allele C performed better than those with T/T genotype. Since about a third of the patients (34%) had a history of past use of cannabis and 2.5% reported current use, we performed the rs12720071 polymorphism analysis excluding these patients. In this subgroup of patients, those with allele C also performed significantly better on Digit Span test (p=0.037).
**Discussion:** In this sample, the rs12720071 polymorphism of CB1R appears to influence performance on a working memory task that is sensitive to pre-frontal cortex function.

### 14. VIOLENCE IN SCHIZOPHRENIA: PREVALENCE, MEASUREMENT, PREDICTION AND PREVENTION

Mark Weiser
*Sheba Medical Center*

**Overall Abstract:** Most patients with schizophrenia and bipolar disorders (severe mental illness, SMI) are not violent in their lifetimes, however, a minority of patients are violent at some points in the course of their illness. As the illness appears relatively early in life, and typically runs a chronic course, the number of violent incidents caused by patients can be considerable in some cases. Due to the stigma toward SMI, the media often emphasize reporting of these incidents, which fuel the stigma even more. Although violent behavior is a common cause of concern for patients, their families and clinicians, it is not often discussed in scientific meetings. The purpose of this symposium is to bring this relatively neglected, but very important topic into the spotlight in SIRS, in order to summarize the latest evidence for clinicians and researchers, and to foster new work on reducing these risks of violence.
Dr. Weiser will present an overview of the prevalence of violent behavior in patients with SMI, and will present a population-based, case-control study from Israel, showing increased rates of violent crime in patients with SMI, particularly in female patients and patients who abuse drugs. Secondary analyses will show increased rates of violent behaviour in siblings of patients as well.
Dr. Fazel will present a systematic review on the prognostic (or predictive) accuracy of structured ways to assess violence risk in patients with severe mental illness, and present new work on a scalable and potentially useful predictive model of violent behaviour based on 75,000 patients in Sweden.
Dr. Nijman will present a model based on patient, ward and staff variables focused on the causes and triggers of aggressive behavior on (locked) psychiatric wards. Based on this model, a number of preventive measures can be formulated. At the patient level, the administration of anti-psychotic medication is used to reduce the negative cognitive schemes and delusional thoughts that are depicted in the center of the model. A more novel intervention at the patient level may be the additional administration of nutritional supplements with (among others) high levels of omega 3 fatty acids. The results of two Dutch studies on this topic will be briefly presented in the lecture, among which a RCT on the effects of the use of nutritional supplements on aggressiveness. On the staff level, the use of short-term (daily) risks assessments by the ward nursing staff, among others by means of the six item BrØset Violence Checklist (BVC), has been found to reduce aggressiveness as well as the use of coercive measures on psychiatric wards in two cluster randomized RCTs. On the ward level, studies indicate that aggression on psychiatric wards can be reduced by preventing overcrowding on psychiatric wards, and by providing more space and privacy to the patients.
Dr. Torrey will present data on rates of re-arrest in patients with SMI, showing that the average five-year re-arrest rate is approximately 40% for those released from psychiatric hospitals and 60% for those released from jails or prisons, and will present comparison data from other countries. He will then present data on the effect of extended conditional release, Forensic Assertive Community Treatment (FACT) teams, and Psychiatric Security Review Boards on re-arrest rates.

### 14.1 VIOLENT CRIME IN SCHIZOPHRENIA AND BIPOLAR DISORDER: A POPULATION-BASED STUDY

Anat Fleischman[1], Nomi Werbeloff[2], Rinat Yoffe[3], Michael Davidson[1], Mark Weiser*,[2]
*[1]Sackler Faculty of Medicine, Tel-Aviv University; [2]Sheba Medical Center; [3]Division of Mental Health Services, Ministry of Health, Jerusalem*

**Background:** Previous studies have found that patients with schizophrenia and bipolar disorder are more likely to be violent than the general population. The aim of this study was to investigate the association between schizophrenia and bipolar disorder and violent crime in the Israeli population.
**Methods:** Using the Israeli Psychiatric Hospitalization Case Registry we identified 3187 patients with a discharge diagnosis of schizophrenia and 506 patients with a discharge diagnosis of bipolar disorder. For each proband we identified parents and siblings, and gender- and age-matched controls for patients, parents and siblings. Information on violent crimes was obtained from police records.
**Results:** Patients with schizophrenia were at increased risk for violent crimes compared with controls [odds ratio (OR) 4.3, 95% confidence interval (CI) 3.8–4.9], especially women (OR 9.9, 95% CI 6.2–15.7). Risk for violent crimes was higher among patients with co-morbid substance misuse than in patients without such co-morbidity (OR 5.1, 95% CI 4.2–6.3).
Patients with diagnosis of bipolar disorder were 2.5 times more likely to be convicted or released for mental reasons of violent crimes compared with controls and unaffected full siblings (OR=2.5, 95%CI 1.7–3.7, OR=2.5, 95%CI 1.6–4.0 respectively). Although men were more violent than women, diagnosis of bipolar disorder was a more significant risk factor for female patients than for male patients (OR=16.1 95%CI 1.8–144.6 vs. OR=2.4, 95%CI 1.5–3.7).
**Discussion:** The results of this study suggest that increased risk of violence is part of the clinical picture of schizophrenia and bipolar disorder and needs to be recognized as a legitimate, essential, aspect of clinical management.

### 14.2 STUCTURED RISK ASSESSMENT IN PSYCHIATRY

Seena Fazel*,[1], Achim Wolf[1], Henrik Larsson[2], Thomas Fanshawe[1], Susan Mallett[4]
*[1]University of Oxford; [2]Karolinska Institutet; [4]School of Population and Health Sciences, University of Birmingham*

**Background:** Current approaches to stratify psychiatric patients into groups based on violence risk are limited by inconsistency, variable accuracy, and unscalability.
**Methods:** Based on a national cohort of 75 158 Swedish individuals aged 15–65 with a diagnosis of severe mental illness (schizophrenic-spectrum and bipolar disorders) with 574 018 patient episodes, we developed predictive models for violent offending through linkage of population-based registers. First, a derivation model was developed to determine strength of pre-specified criminal history, socio-demographic, and clinical risk factors, and tested it in external validation. We measured discrimination and calibration for prediction of violent offending at 1 year using specified risk cut-offs.
**Results:** A 16 item model was developed from criminal history, socio-demographic and clinical risk factors, which are mostly routinely collected. In external validation, the model showed good measures of discrimination (c-index 0.89) and calibration. For risk of violent offending at 1 year, using a 5% cut off, sensitivity was 64% and specificity was 94%. Positive and negative predictive values were 11% and 99%, respectively. The model was used to generate a simple web-based risk calculator (OxMIV).
**Discussion:** We have developed a prediction score in a national cohort of patients with psychosis that can be used as an adjunct to decision making in clinical practice by identifying those who are at low risk of violent offending

### 14.3 CAUSES AND PREVENTION OF AGGRESSION FROM PSYCHOTIC INPATIENTS

Henk Nijman*,[1]
[1]*Radboud University Nijmegen*

**Background:** Patients with schizophrenia and other psychotic disorders have an increased likelihood of engaging in violent behavior. These increased risks of dangerous and aggressive behavior, in combination with a lack of insight in their own illness, relatively often make involuntary admission of acutely disturbed psychotic patients on locked psychiatric admissions wards often inevitable. On these locked psychiatric admissions wards, aggression from psychotic patients against staff and fellow patients is a prevalent phenomenon, with the mean in the Netherlands being about 18 aggressive incidents per bed per year on locked psychiatric admissions wards.
**Methods:** In the lecture, a model of what causes or triggers aggressive behavior on (locked) psychiatric wards is presented. In this model, patient, ward and staff variables are integrated to explain why, and in what specific situations, psychotic patients particularly run a high risk of engaging in aggressive behavior.
**Results:** Based on the presented model, a number of preventive measures can be formulated.
On the patient level, the administration of anti-psychotic medication is used to reduce the negative cognitive schemes and delusional thoughts that are depicted in the center of the model. A more novel intervention at the patient level may be the additional administration of nutritional supplements with (among others) high levels of omega 3 fatty acids. The results of two Dutch studies on this topic will be briefly presented in the lecture, among which a RCT on the effects of the use of nutritional supplements on aggressiveness.
On the staff level, the use of short-term (daily) risks assessments by the ward nursing staff, among others by means of the six item BrØset Violence Checklist (BVC), has been found to reduce aggressiveness and the use of coercive measures on psychiatric wards in two cluster randomized RCTs.
On the ward level, studies indicate that aggression on psychiatric wards can be reduced by preventing overcrowding on psychiatric wards, and by providing more space and privacy to the patients.
**Discussion:** The proposed model elucidates how certain patient, staff and ward characteristics may interact in causing aggression. The model also emphasizes that repeated inpatient aggression may be the result of a vicious circle, i.e. inpatient violence is often followed by an increase in environmental and/or communication stress on the patient, thereby heightening the risk of a repeated outburst of violence.

### 14.4 FOLLOW-UP TREATMENT FOR INDIVIDUALS WITH SERIOUS MENTAL ILLNESS WHO HAVE COMMITTED MAJOR CRIMES

E. Fuller Torrey*,[1]
[1]*The Stanley Medical Research Institute*

**Background:** For individuals who have a psychiatric disorder and have committed a major crime, the rate of re-offending is twice as high in the US compared to nine other countries for which there is comparable data. For such individuals the average five-year rearrest rate is approximately 40% for those released from psychiatric hospitals and 60% for those released from jails or prisons. The use of treatment modalities such as extended conditional release, Forensic Assertive Community Treatment (FACT) teams, and Psychiatric Security Review Boards can reduce the rearrest rate from 40–60% to 10% or less.
**Methods:** All 50 states were surveyed to assess how they were doing in providing follow-up treatment for such individuals.
**Results:** Sixteen states were found to be making a moderate effort to provide follow-up treatment, and another 13 states are making a minimal effort. However, the other 21 states, 42% of the total, are making virtually no effort, lending to an unnecessarily high rate of re-offending.
**Discussion:** Using proven treatment approaches the re-arrest rate of individuals with serious mental illness can be reduced from 40–60% to 10% or less.

### Plenary

### 15. ON THE ROAD TO CURING SCHIZOPHRENIA

Cynthia Shannon Weickert
*Neuroscience Research Australia: Schizophrenia Research Laboratory*

**Overall Abstract:** I began my journey to find out what caused schizophrenia around the time me and my twin brother, Scott David, turned 17. My first step was to conceptualize schizophrenia as a biological, cellular and molecular brain problem. This guided my choice of undergraduate and graduate study. I quickly realized that schizophrenia was not a "genetic" disease, nor was it an "environmental" disease, it was both. I prioritized studying RNA as was the active genome, the subcellular substrate where genes and environment interact. Guided from my own experience of watching my normal twin be tortured by schizophrenia in his teens, I sought to find answers by studying the mammalian brain as it developed and changed during adolescence. For my post-doctoral fellowship, I joined the laboratory of Joel Kleinman, who has the largest and best characterized human brain collection of people with schizophrenia in the world. Along my journey, while at NIMH in the USA, I discovered changes in neuronal growth factors and hormone receptors during stages of postnatal life and in the brains of people with schizophrenia compared to controls using the classical hypothesis-driven approach. Since I moved to Sydney Australia, I choose a different, more open-minded approach and let the brains of those who suffered "tell me what happened to them", using a modern, sensitive discovery-driven RNAseq approach. When I listened, more carefully at the molecular level, what I found told me that I may be headed down the wrong path with my research and that I needed to change direction. It suggested that the emphasis I placed on development molecules maybe in some ways blinding me from more clearly seeing the neuropathology that existed only in only some people at the time of death. I found elevated inflammatory cytokine mRNAs in ~40% in the brains of those diagnosed with chronic schizophrenia. In this talk, I will review my latest discoveries on neuroinflammation in schizophrenia including evidence of gliosis, blood-brain barrier (BBB) changes and increased white blood cells in the brains of some with schizophrenia. Today, many of my fellow seekers including geneticists (Chr 6, MHC locus) and epidemiologists (maternal infection) and "animal modelers" (poly I:C) are suggesting that the cause of schizophrenia may involve changes to the immune system. These new discoveries suggest that very first steps I took may have been wrong, that perhaps I should have become an immunologist rather than a neurobiologist. However, from my current vantage point, I believe that even if a fault in the immune system plays a role in the causality of schizophrenia, that neurons are the major protagonist behind the manifestation of schizophrenia. The road ahead suggests that we, as a field, need to do some more trial blazing research that conceptualizes schizophrenia as not a "brain" disease and not as an "immune" disease, but as both.

### Concurrent Symposia

### 16. DEVELOPMENTAL BIOMARKERS OF ENVIRONMENTAL ADVERSITY AND EPIGENETIC RISK FOR MAJOR PSYCHIATRIC DISORDERS: CLUES TO PATHOGENESIS

Kim Do
*Center for Psychiatric Neuroscience, Lausanne University Hospital*

**Overall Abstract:** Interaction between genetic risks and environmental adversities such as childhood trauma may underlie the etiology of psychiatric disorders. Mechanisms underpinning these interactions are poorly understood. This panel will present new data supporting a model in which epigenetic modifications and increased oxidative stress lead to altered trajectories and patients' stratification. Helen Fisher will introduce the field, how exposure to severe stress may have immediate as well as long-lasting damaging effects on learning, behaviour, and health, and has been implicated in the development of psychosis. She will present epigenome-wide analyses of poly-victimisation across childhood and adolescence, utilising data from the Environmental Risk (E-Risk) Longitudinal Twin Study, an epidemiological study of 2,232 children (1,116 twin pairs) born in 1994–1995 in England and Wales and followed to 18 years of age. Results related to associations with DNA methylation in peripheral blood at age 18 years, specific forms of victimization and candidate genes in the stress response will be discussed. Luis Alameda will show that childhood trauma (sexual, physical and emotional abuse) engages oxidative stress in a cohort of early psychosis patients (n=118). Patients with high peripheral oxidation status were found to have smaller hippocampal volumes and more severe clinical symptoms, while those with lower oxidation status evidenced a compensatory antioxidant regulation and better cognition. Linear discrimination analysis highlights blood oxidation status together with childhood trauma as markers for poorer psychopathological profile, allowing patients' stratification. Oussama Kebir will present the first longitudinal prospective study of genomic DNA methylation during psychotic transition in help-seeking young individuals (14 converters, 25 non converters). Alterations in gene promoters and pathways relevant for psychosis, including oxidative stress regulation, axon guidance and inflammatory pathways were observed. In particularly, antioxidant defense gene GTSTM5 is differentially methylated through time and two other genes of GST family might be differentially methylated after conversion to psychosis. These findings suggest that conversion to psychosis may depend on the specific control of oxidative metabolism and regulation between these genes. Darina Czamara will report on how cord blood methylation is affected by genetic and prenatal environment. Analysis on SNP (G) and environmental (E) effects as well as on GxE and G+E were performed using Illumina's Human Omni Express Exom as well as the 450k DNA methylation array in a cohort of 817 Finnish newborns. G and the combination of G and E explained DNA methylation best, environment alone was almost never the best predictor. Furthermore, epigenetic gestational age was associated with prenatal environment as well as with childhood psychiatric problem at age 3 indicating that it might be used as a potential biomarker.
Overall, these new results suggest new biomarkers of environmental and genetic adversity that are related to pathogenic mechanisms, including epigenetic, oxidative stress and structural abnormalities, in epidemiological studies and psychiatric disorders. These biomarkers offer the potential for individualized early intervention and preventive strategies.

### 16.1 EPIGENETIC SIGNATURES OF CHILDHOOD AND ADOLESCENT VICTIMISATION USING A GENETICALLY SENSITIVE LONGITUDINAL COHORT STUDY.

Helen Fisher*[,1], Sarah Marzi[2], Louise Arseneault[2], Chloe Wong[2], Radhika Kandaswamy[2], Terrie Moffitt[3], Susanna Roberts[2], Jonathan Mill[4], Avshalom Caspi[3]
*[1]Institute of Psychiatry, Psychology & Neuroscience, King's College London; [2]King's College London; [3]King's College London, Duke University; [4]Exeter University*

**Background:** Stress is a normal, adaptive response to stressors (e.g. events that make a person feel threatened or upset). However, healthy development can be derailed by excessive or prolonged exposure to stress especially during important developmental periods such as childhood and adolescence. Exposure to severe stress may have immediate as well as long-lasting damaging effects on learning, behaviour, and health, and has been implicated in the development of psychosis. One way these may occur is via changes to epigenetic processes (e.g. alterations in DNA methylation). Initial studies have shown that individuals exposed to severe psychosocial stress have different patterns of DNA methylation (epigenetic 'signatures') compared to individuals exposed to no/minimal stressful life events, but these studies are methodologically limited.
**Methods:** This paper describes our examination of the potential link between exposure to multiple forms of severe victimisation (poly-victimisation) during childhood adolescence and DNA methylation differences utilising data from the Environmental Risk (E-Risk) Longitudinal Twin Study, an epidemiological study of 2,232 children (1,116 twin pairs) born in 1994–1995 in England and Wales and followed to 18 years of age (with 93% retention). Multiple forms of victimisation were ascertained in childhood and adolescence (including physical, sexual and emotional abuse, neglect, exposure to intimate-partner violence, bullying, cyber- and crime victimization) by combining the best practices in survey research with comprehensive interview-based approaches. Whole blood samples were collected from participants at age 18, and the extracted DNA was used to quantify genome-wide patterns of DNA methylation.
**Results:** Epigenome-wide analyses of poly-victimisation across childhood and adolescence revealed several significant associations with DNA methylation in peripheral blood at age 18 years, but these were confounded by tobacco smoking and/or did not survive co-twin control tests. Secondary analyses of specific forms of victimisation revealed sparse associations with DNA methylation that did not replicate across different operationalisations of the same putative victimization experience. Hypothesis-driven analyses of six candidate genes in the stress response (NR3C1, FKBP5, BDNF, AVP, CRHR1, SLC6A4) did not reveal predicted associations with DNA methylation in probes annotated to these genes.
**Discussion:** Findings from this epidemiological analysis of the epigenetic effects of early-life stress do not support the hypothesis of robust changes in DNA methylation in victimised young people. It is possible that epigenetic epidemiology is not yet well matched to experimental, non-human models in uncovering the biological embedding of stress.

### 16.2 CHILDHOOD TRAUMA ENGAGES OXIDATIVE STRESS, HIPPOCAMPUS ALTERATIONS, AND POORER CLINICAL OUTCOME IN EARLY PSYCHOSIS PATIENTS

Luis Alameda*[,1], Margot Fournier[2], Ines Khadimallah[2], Philippe S Baumann[3], Martine Cleusix[2], Alessandra Griffa[4], Paul Klauser[5], Raoul Jenni[2], Michel Cuenod[6], Patric Hagmann[4], Philippe Conus[7], Kim Do[2]

[1]*Institute of Psychiatry, Psychology & Neuroscience, King's College London;* [2]*Center for Psychiatric Neuroscience, Lausanne University Hospital;* [3]*Lausanne University;* [4]*Signal Processing Laboratory, Ecole Polytechnique Fédérale de Lausanne, Lausanne University Hospital;* [5]*The University of Melbourne, Melbourne Neuropsychiatry Centre;* [6]*Center for Psychiatric Neuroscience, University Hospital of Lausanne - CHUV Cery;* [7]*Service of General Psychiatry, Lausanne University Hospital*

**Background:** Exposure to childhood trauma (CT) is a global major public-health and social-welfare problem worldwide. CT increases the vulnerability to major psychiatric conditions including psychosis and is associated with poorer clinical outcome. CT affects the development of brain structures such as hippocampus, possibly through oxidative stress and neuroinflammation, two mechanisms linked to psychosis. We therefore hypothesized that there is an interplay between oxidative stress and CT in psychosis patients. We thus explored in early psychosis patients the relationships between CT and i) hippocampal volume, ii) antioxidant systems; and iii) clinical and cognitive outcomes.
**Methods:** We studied a cohort of 118 early psychosis patients, 36 were exposed to CT (experiences of physical, sexual, or emotional abuse/neglect before16 years old). In a subgroup of 48 patients (18 CT), hippocampal volume was determined by MRI. Antioxidant systems were quantified in blood for the whole cohort. Markers were: glutathione peroxidases (GPx) activity which appeared as a peripheral correlate of brain GSH levels (Xin &al, 2016); peroxiredoxine levels (Prx); Thioredoxine (Trx). Psychopathology (PANSS) and neuropsychology (MCCB) were assessed. The various groups were segregated by linear discriminant analysis.
**Results:** The previously observed decreased hippocampal volume in patients and association of small hippocampal volume with high blood GPx activity (reflecting high oxidative status) (Baumann &al, 2016) was due to the contribution of the traumatized group. Indeed, this association was absent in the no-trauma group, suggesting that the smaller hippocampus is linked to a redox dysregulation. To explore that point further, four groups were then formed, according to trauma and oxidative status: (i) noCT-lowGPx, (ii) noCT-highGPx, (iii) CT-lowGPx and (iv) CT-highGPx. Group CT-highGPx only had smaller hippocampi.
In CT patients, small hippocampal volume was associated with high GPx activity, while hippocampal volume was similar in CT patients with low GPx activity (CT-lowGPx) and in patients not exposed to CT. Interestingly, other antioxidant defense systems such as Trx and oxidized Prx levels correlated negatively with GPx in CT-lowGPx group, suggesting that the Trx/Prx system is able to compensate for changes/decreases in GPx activity. Moreover, CT-lowGPx patients perform better than the other patients on speed of processing, verbal memory and attention tests.
In contrast, hippocampal volume was decreased in CT patients with high GPx activity (CT-highGPx) compared with CT-lowGPx patients and patients not exposed to CT. There was no correlation between GPx and Trx/Prx system in this group. CT-highGPx patients had more severe positive, negative and disorganized symptoms than the other patients.
**Discussion:** We report that traumatized psychosis patients with high peripheral oxidation status (high GPx) had smaller hippocampal volumes and more severe clinical symptoms, while those with lower oxidation status (low GPx) had better cognition and appear to activate a compensatory antioxidant regulation by the Trx/Prx system.
These results suggest that, in early psychosis patients, traumatic experiences during childhood interact with different redox systems and have long term neuroanatomical and clinical impacts. Therefore, redox pathways such as GPx, Trx and Prx systems represent important translational biomarkers for patient selection and stratification in order to aid in diagnostics and treatment decision at early stages of the disease.

### 16.3 METHYLOMIC CHANGES OF OXIDATIVE STRESS REGULATION, AXON GUIDANCE AND INFLAMMATORY PATHWAYS DURING CONVERSION TO PSYCHOSIS

Oussama Kebir*[,1], Boris Chaumette[2], Marie-Odile Krebs[3]
[1]*CH Sainte Anne, Paris;* [2]*INSERM U894 - Ste Anne Hospital;* [3]*INSERM U894, Paris Descartes University, Sainte-Anne Hospital, Service Hospitalo-Universitaire*

**Background:** Epigenetics is hypothesized to mediate the interplay between genes and environment leading to the onset of psychosis
**Methods:** We performed a longitudinal prospective study of genomic DNA methylation during psychotic transition in help-seeking young individuals referred to a specialized outpatient unit for early detection of psychosis and enrolled in a 1-year follow-up (n=39). We used Infinium HumanMethylation450 BeadChip array after bisulfite conversion and analyzed longitudinal variations in methylation at 411 947 cytosine–phosphate–guanine (CpG) sites.
**Results:** We observed that conversion to psychosis was not associated with a global change in methylation and there was no individual CpG significantly associated with psychotic transition. By contrast, we found that conversion to psychosis was associated with specific methylation changes in genes involved in axon guidance, as well as genes of the IL-17 pathway and the glutathione-S-transferase family.
**Discussion:** Alterations in oxidative stress regulation, axon guidance and in inflammatory pathways could represent multiple theaters for the disruption in homeostasis that accompanies the emergence of full-blown psychosis.

### 16.4 EFFECT OF GENOTYPE AND EARLY ADVERSITY ENVIRONMENT ON DNA METHYLATION

Darina Czamara*[,1], Polina Girchenko[2], Anna Suarez Figueiredo[2], Jari Lahti[2], Katri Räikkönen[2], Elisabeth Binder[1]
[1]*Max Planck Institute of Psychiatry;* [2]*Institute of Behavioral Sciences, University of Helsinki*

**Background:** Fetal or prenatal programming, i.e. the process in which environmental events during pregnancy are shaping and determining the development of the embryo, can be embedded by epigenetic changes including DNA methylation. Apart from environment, also the genome plays an important role and a variety of studies which identified meQTLs (methylation quantitative trait loci, i.e. SNPs which are associated with methylation levels) have been published.
**Methods:** Focusing on variably methylated regions (VMRs), we investigated if genotype (G), prenatal environment (E) or the combination of both (GxE, G+E) best explain cordblood DNA methylation in sample of 817 Finnish neonates. Furthermore, we used an epigenetic clock predictor to evaluate if accelerated or decelerated epigenetic age was associated with prenatal environment or with childhood psychiatric problems at age 3.
**Results:** We found that SNP genotype alone best explained methylation status in 44%, SNP x environment in 32% and SNP and prenatal environment in 24% of all VMRs. While functionally relevant meQTLs were located in close proximity to the specific CpG-site, functionally relevant SNPs involved in interaction models showed much broader peaks.
Concerning the epigenetic clock, lower gestational age was associated with maternal depression diagnosis and greater depressive symptoms throughout pregnancy. Epigenetic age deceleration was associated with pre-eclampsia. Furthermore, lower epigenetic gestational age was significantly associated with greater total and internalizing problems in boys.

**Discussion:** Not only environment but also genotype should be considered in epigenetic studies. Furthermore, our results suggest that long-distance effects are present in GxE interactions. The epigenetic clock which mirrors prenatal environment is partially predictive for future development of the child. Lower epigenetic gestational age seems to be developmentally disadvantageous for boys, who in early childhood show greater psychiatric problems.

### 17. CANNABIDIOL AS A TREATMENT IN DIFFERENT STAGES OF PSYCHOSIS- EFFICACY AND MECHANISMS

Sagnik Bhattacharyya
*Institute of Psychiatry, King's College London*

**Overall Abstract:** While drugs that target the dopaminergic and glutamatergic neurotransmitter systems have been extensively investigated as treatments for psychosis, there has been increasing attention in recent years on the endocannabinoid system as a therapeutic target. The CB1 receptor, the main central cannabinoid receptor is ubiquitous and modulates the function of several neurotransmitters, including dopamine and glutamate. A growing body of evidence suggests that psychosis is associated with alterations in the endocannabinoid system, independent of exposure to cannabis.
The CB1 receptor is the main molecular target for delta-9-tetrahydrocannabinol (THC), the major psychoactive ingredient in cannabis. THC is responsible for the psychotogenic effects of cannabis, and is a partial agonist at the CB1 receptor. On the other hand, Cannabidiol (CBD), the second major constituent of cannabis is a non-psychoactive compound that may have an inverse agonist/antagonist effect at CB1 receptors, in addition to a range of other possible mechanisms of action. Interest in the therapeutic potential of CBD stemmed from evidence that it has broadly opposite effects to that of THC, at both the neural and the behavioural level in healthy individuals. Consistent with these results independent evidence that CBD has antipsychotic and anxiolytic properties in patients with mental health disorders has been accumulating. The absence of significant adverse effects associated with CBD, is a critical advantage in relation to the treatment of patients in the various stages of psychosis. Given its tolerability profile, CBD is a treatment of particular interest not just in those with chronic psychosis as in schizophrenia, but also in those in the earlier stages of psychosis. However, published evidence regarding the therapeutic efficacy of CBD as a treatment in psychosis has been limited except a small randomized clinical trial (RCT) some years ago (Leweke et al 2012). Furthermore, the mechanisms that may underlie the beneficial effects of CBD are unclear.
This symposium will bring together state of the art evidence regarding the efficacy of CBD in the different stages of psychosis – from those at clinical high-risk (CHR), through early psychosis to chronic schizophrenia. Furthermore, data from animal and human studies will be presented to give an understanding of the potential mechanisms that may underlie the therapeutic effects of CBD.
The first speaker (Prof Crippa) will set the scene by presenting evidence regarding the different potential mechanisms of action that may underlie the antipsychotic effect of CBD.
The second speaker (Prof McGuire) will present the results of a 6-week placebo-controlled RCT demonstrating the efficacy of CBD as an add-on to existing antipsychotic treatment in schizophrenia.
The third speaker (Dr. Ranganathan) will present the results from an ongoing, placebo-controlled RCT using an within-subject, crossover design to show the effects of 4-week CBD treatment on psychotic symptoms, cognition and electrophysiological markers in patients with established psychosis.
Finally, the fourth speaker (Dr. Bhattacharyya) will present the results from a recently completed proof-of-concept study demonstrating the efficacy of short-term CBD treatment on symptoms and distress in CHR patients as well as on neurocognitive substrates implicated in the CHR state.
Evidence presented here will be discussed by Prof D'Souza, who is an internationally recognized expert in cannabinoid pharmacology and experimental therapeutics development.

### 17.1 A RANDOMIZED CONTROLLED TRIAL OF CANNABIDIOL IN SCHIZOPHRENIA

Philip McGuire*[,1], Philip Robson[2], Wiesław Cubała[3], Daniel Vasile[4], Paul Morrison[1], Rachel Barron[2], Adam Taylor[2], Stephen Wright[2]
*[1]Institute of Psychiatry, Psychology & Neuroscience, King's College London; [2]GW Pharmaceuticals; [3]Medical University of Gdańsk; [4]Central Military Emergency University Hospital, Bucharest*

**Background:** Both preclinical and human research suggest that cannabidiol (CBD) has antipsychotic properties. This study assessed the safety and effectiveness of CBD in patients with schizophrenia.
**Methods:** Patients with schizophrenia (n=88) were randomized to receive CBD (1000 mg/day) or placebo alongside their existing antipsychotic medication for 6 weeks. Participants were assessed before and after treatment using the PANSS, BACS, GAF scales, and the CGI Improvement and Severity scales.
**Results:** Compared those given placebo, patients treated with CBD had lower levels of positive psychotic symptoms (PANSS; p=0.02), and were more likely to have been rated by clinicians as improved (CGI-I; p=0.02) and as not severely unwell (CGI-S; p=0.04). Patients who received CBD also showed trends for greater improvements in cognitive performance (BACS; p=0.07) and in overall functioning (GAF; p=0.08). There was no difference in the frequency of CBD of adverse events between CBD and placebo.
**Discussion:** These data suggest that CBD has beneficial effects in patients with schizophrenia and is not associated with significant adverse effects.

### 17.2 EFFICACY OF CANNABIDIOL IN THE TREATMENT OF EARLY PSYCHOSIS.

Mohini Ranganathan*[,1], Deepak D'Souza[2], Jose Cortes-Briones[1], Patrick Skosnik[1]
*[1]Yale University School of Medicine; [2]Yale University School of Medicine, VA Connecticut Healthcare System*

**Background:** Cannabidiol is a component of herbal cannabis, studied for a number of potential pharmaceutical indications, more recently, its potential anti-psychotic effects with an extremely favorable side effect profile. Cannabidiol content of cannabis may also attenuate the psychotic and cognitive effects associated with cannabis use. Early psychosis is associated with alterations in the endocannabinoid system and is marked by limited engagement in treatment, reluctance to use traditional antipsychotics, sensitivity to medication side effects and heavy cannabis use. Cannabidiol may thus represent a more acceptable and tolerable antipsychotic medication in this phase of illness with a novel mechanism of action.
**Methods:** Data will be presented from an ongoing double blind, placebo controlled, within subject, crossover study examining the effects of Cannabidiol (800mg/day) versus placebo in individuals within the first 7 years of their psychotic illness. Subjects participate in two treatment periods, each four weeks long separated by at least 2 weeks of washout.
**Results:** Data will be presented on the effects of Cannabidiol on psychotic symptoms (measured on the Positive and Negative Syndrome Scale), cognitive deficits (MATRICS battery), electrophysiological biomarkers of information processing (Resting EEG and ERPs relevant to psychosis and cannabinoids), metabolic parameters and general functioning.
**Discussion:** Cannabidiol is a novel drug that has shown potential efficacy in the treatment of psychotic symptoms. Early psychosis is a critical treatment period during which treatment engagement and adherence is critical and duration of untreated psychosis is associated with long term negative consequences. Cannabidiol may thus represent a more acceptable and tolerable medication to target this vulnerable population.

### 17.3 EFFECT OF CANNABIDIOL ON SYMPTOMS, DISTRESS AND NEUROPHYSIOLOGICAL ABNORMALITIES IN CLINICAL HIGH-RISK FOR PSYCHOSIS PATIENTS: A PLACEBO-CONTROLLED STUDY

Sagnik Bhattacharyya*[,1], Robin Wilson[1], Paul Allen[1], Matthijs Bossong[2], Elizabeth Appiah-Kusi[2], Philip McGuire[1]
*[1]Institute of Psychiatry, Psychology & Neuroscience, King's College London; [2]Brain Center Rudolf Magnus, University Medical Center Utrecht*

**Background:** There has been growing interest in the therapeutic potential of Cannabidiol (CBD) stemming from independent evidence that CBD has antipsychotic and anxiolytic properties in patients with mental health disorders. CBD has been found to be non-inferior to antipsychotic medication in a 4-week clinical trial in acute schizophrenia (Leweke et al 2012) and also has been found to reduce anxiety symptoms in social phobia and following public speaking. Human data also suggest that it attenuates the cognitive impairments associated with use of the main psychoactive ingredient in cannabis. However, whether CBD may be useful in relieving symptoms and distress in patients at clinical high-risk of psychosis (CHR) has never been tested. Furthermore, how the beneficial effect of CBD on psychotic and anxiety symptoms may be mediated in the brain remains unclear.
The aim of the present study was to investigate whether short-term treatment with CBD was associated with preliminary evidence of therapeutic benefit and understand the neurocognitive mechanisms.
**Methods:** We investigated the effects of short-term (21 days) treatment with CBD on psychotic and anxiety symptoms in 33 CHR patients, using a placebo-controlled, double-blind, parallel-arm design (CBD arm- 16; placebo arm- 17). In the subjects who received 21 days of treatment, we used fMRI in conjunction with a verbal memory task to assess the effect of CBD relative to placebo treatment on medial temporal and striatal function.
**Results:** Of the 33 CHR patients recruited into the trial, 31 completed treatment for 21 days. Following 21-day treatment (intention-to-treat, last observation carried forward analysis), CBD-treated (n=16) CHR patients showed a significantly greater reduction in anxiety (p=0.02) and in distress associated with psychotic symptoms (p=0.03) and a trend (p=0.14) toward greater reduction in the severity of psychotic symptoms compared to those treated with placebo (n=17) CHR patients (Figure 4). In addition, CBD was tolerated as well as placebo.
Consistent with our predictions, treatment with CBD (n=15) attenuated the engagement during verbal encoding of the parahippocampal cortex, but increased activation in the putamen in CHR patients. A similar pattern of activation was evident during verbal recall, with CBD treatment associated with increased engagement in the putamen.
**Discussion:** Results from our proof-of-concept study suggest that 3-week treatment with CBD has beneficial effects on anxiety, attenuated psychotic symptoms and the distress associated with psychotic symptoms. They also suggest that short-term treatment with CBD modulates both medial temporal and striatal function in CHR patients, regions that are critically implicated in the CHR state. Coupled with the absence of significant adverse effects associated with CBD, a particularly important issue in relation to the treatment of CHR individuals, not all of whom develop a full-blown psychotic disorder, these data indicate that long-term treatment with CBD is likely to be efficacious in CHR patients.

### 17.4 POSSIBLE MECHANISMS INVOLVED IN THE ANTIPSYCHOTIC EFFECTS OF CANNABIDIOL (CBD)

Jose Alexandre Crippa*[,1], Antonio Waldo Zuardi[1], Francisco Silveira Guimaraes[1]
*[1]University of Sao Paulo*

**Background:** Laboratory rodents and human studies have demonstrated that cannabidiol (CBD) presents antipsychotic effects. Several mechanisms of action have been associated to CBD effects. Most of the studies that have investigated these mechanisms have been performed in vitro and their relevance for the in vivo effects of this drug is still uncertain.
**Methods:** In spite of its low affinity for CB1 receptor (CB1R), CBD is capable of antagonizing CB1R agonists at reasonable low concentrations. CBD can also inhibit anandamide uptake and metabolism, enhancing endocannabinoid tonus. It is also possible to attribute the antipsychotic action of CBD to an antagonism of CB1R. This suggestion was reinforced by the observation that patients in the initial prodromal states of psychosis and patients with schizophrenia had higher levels of anandamide in CSF than health controls. Moreover, there would be a decrease in glutamatergic inputs from this area to the prefrontal cortex, impairing locomotor activity and working memory and endocannabinoids could regulate this system. CBD could facilitate endocannabinoids "on demand" synthesis in post-synaptic neurons, acting pre-synaptic terminals and negatively regulating the release of neurotransmitters, particularly GABA and glutamate.
**Results:** Also, an increase in anandamide levels induced by CBD could attenuate GABA release from ventral pallidum neurons, restoring the normal function of this system in psychotic patients. CBD can also increase adult neurogenesis in mice and that this effect has been shown to be dependent on the CB1 receptors. In addition to the mechanisms discussed above, CBD can produce several other effects that could also be involved to be responsible for its antipsychotic properties, including interaction with 5HT1A and TRPV1 receptors and anti-inflammatory/neuroprotective effects. CBD can act as a serotonin 1A receptor (5HT1A) agonist, although the role of 5-HT1A-mediated neurotransmission in schizophrenia is unclear, aripiprazole, an atypical antipsychotic, acts as a partial agonist at this receptor, an effect that could, together with its actions on D2 and 5-HT2A receptors, contribute to therapeutic effects of this drug. CBD can also activate Transient Receptor Vanilloid-1 (TRPV1) receptors that are expressed in several brain areas related to psychosis such as the prefrontal cortex, amygdala and hippocampus. Since CBD is also a potent anti-inflammatory, antioxidant and neuroprotective compound, it is possible that these effects are involved in its antipsychotic action.
**Discussion:** Since CBD is also a potent anti-inflammatory, antioxidant and neuroprotective compound, it is possible that these effects are involved in its antipsychotic action.

### 18. TRACKING THE MECHANISMS UNDERLYING WORKING MEMORY DYSFUNCTION IN SCHIZOPHRENIA FROM CORTICAL MICROCIRCUITS TO THE SYSTEMS LEVEL

Robert Bittner
*University Hospital Frankfurt, Goethe University*

**Overall Abstract:** Converging behavioural and neuroimaging evidence indicates that an inability to regulate behaviour by working memory (WM) is a core feature of people with schizophrenia (PSZ) which significantly influences their level of recovery. WM dysfunction has also gained interest as a target of cognitive enhancement interventions and as an intermediate phenotype in the study of the genetic architecture of schizophrenia. These translational strategies critically depend upon a clear understanding of the underlying cognitive and neurophysiological disturbances. The aim of the symposium is to highlight current approaches to identify the pathophysiological mechanisms underlying WM deficits on both the microcircuit and the systems level.
M. Ichinose will report cognitive modelling data showing that PSZ have elevated internal noise during perceptual processing, which was inversely correlated with WM precision. This indicates that 'noisy' perception contribute to impairments in WM and other cognitive domains.
R. Bittner will present behavioural data from PSZ and imaging genetics fMRI data from a large cohort of healthy participants. The results of these studies indicate, that specific impairments in bottom-up attentional processes associated with genetic risk contribute to the dysfunction of working memory encoding in schizophrenia.

A. Anticevic will present computational microcircuit models, resting state fMRI data and fMRI data using ketamine which provide evidence for a disturbed excitation-inhibition (E-I) balance and resulting in large scale dysconnectivity in schizophrenia in the context of working memory and other cognitive processes.

The research presented in this symposium integrates computational, neuroanatomical, electrophysiological, pharmacological, genetic, behavioural and neuroimaging methods to connect pathophysiological mechanisms at the microcircuit level such as increased neuronal noise and an abnormal E-I balance to disturbances in large scale brain networks at the systems level and to behavioral impairment. Such an integrative approach promises to yield new insights into the pathophysiology of cognitive dysfunction in schizophrenia.

### 18.1 MITOCHONDRIAL ALTERATIONS WITHIN THE PYRAMIDAL-PARVALBUMIN CELL MICROCIRCUIT IN THE PREFRONTAL CORTEX IN SCHIZOPHRENIA

Jill Glausier*,[1]
[1] *University of Pittsburgh*

**Background:** Working memory, a core cognitive function impaired in schizophrenia, depends upon gamma oscillatory neuronal activity in the prefrontal cortex (PFC). Accordingly, individuals with schizophrenia show lower power of gamma oscillations in the PFC during tasks that involve working memory.

Gamma oscillations emerge from the fast and coordinated activity of layer 3 excitatory pyramidal cells and inhibitory parvalbumin (PV) cells. As such, gamma oscillations have a particularly high energetic demand that is met by ATP production via oxidative phosphorylation (OXPHOS) within pyramidal and PV cell mitochondria. PFC layer 3 pyramidal cells have prominent reductions in OXPHOS-related gene pathways in schizophrenia. Importantly, OXPHOS can be regulated by two distinct processes: ATP demand to support neuronal firing, or upstream deficits in OXPHOS enzyme expression.

Layer- and cell type-specific transcriptomic analyses of OXPHOS enzymes and ultrastructural analyses of mitochondrial morphology can help to distinguish between these two possibilities. Reduced ATP demand due to reduced neuronal firing is associated with 1) correlated expression levels of OXPHOS enzyme complexes, 2) lower expression of all subunits comprising Complex IV, the terminal and rate-limiting OXPHOS enzyme complex, and 3) normal mitochondrial morphology. Defective OXPHOS results in 1) elimination of correlated expression of OXPHOS enzyme complexes, 2) variable and inconsistent effects on Complex IV subunit expression, and 3) abnormal mitochondrial morphology.

To determine which upstream factor is likely operative in the illness, we quantified OXPHOS enzyme complex transcripts in layer 3 pyramidal and PV cells, and performed ultrastructural analyses of mitochondrial morphology within pyramidal and PV axon boutons in layer 3 of the PFC in schizophrenia and unaffected comparison subjects.

**Methods:** For mRNA analyses, frozen tissue sections of area 9 from 36 pairs of schizophrenia and comparison subjects were stained for Nissl substance to identify pyramidal cells, or labeled using immunoperoxidase for aggrecan to identify PV cells. Layer 3 pyramidal and PV somata were dissected using laser capture microdissection. Transcriptome profiling was performed by microarray using Affymetrix GeneChips. Analysis of Complexes I, IV, and V expression in each neuronal population was assessed at $q<0.05$ in covariate- and multiple comparisons-corrected analyses.

Electron microscopic analyses were performed in area 46 of 2 matched pairs of schizophrenia and comparison subjects. Mitochondria in pyramidal and PV cell boutons were classified as normal or abnormal using established criteria. Chi-square analysis was used to examine whether the percentages of each type differed between groups.

**Results:** The expression of subunits comprising Complexes I, IV, and V in layer 3 pyramidal and PV cells was significantly lower in subjects with schizophrenia relative to unaffected comparison subjects. In both cell populations, expression of Complexes I, IV, and V were correlated ($r=0.8$-$0.9$) in unaffected comparison and schizophrenia subjects. Complex IV subunits showed 11–26% reductions in pyramidal cells, and 7-31% reductions in PV cells in schizophrenia. In both unaffected comparison and schizophrenia subjects, ≥99% of mitochondria in pyramidal cell boutons and ≥97% of mitochondria in PV cell boutons exhibited normal morphology.

**Discussion:** The current findings are most consistent with the interpretation that lower measures of OXPHOS in schizophrenia reflect lower demand for ATP production due to less neuronal firing.

### 18.2 USING COMPUTATIONAL ESTIMATES OF INTERNAL "NOISE" TO CHARACTERIZE VISUAL PERCEPTUAL AND WORKING MEMORY DEFICITS IN SCHIZOPHRENIA

Megan Ichinose*,[1], Woon Ju Park[2], Duje Radin[2], Sohee Park[1]
[1] *Vanderbilt University;* [2] *University of Rochester*

**Background:** Heightened neural noise serves as a promising explanatory framework for schizophrenia (SZ) pathophysiology, yet its specific contribution to working memory (WM) deficits remains unclear. The perceptual template model (PTM), an established human-observer model of visual perception, asserts that a system's internal noise (IN) is due to both background, 'additive' noise and stimulus-driven 'unfiltered' noise. In this study, we assessed levels of PTM-derived additive and unfiltered IN in SZ during basic visual processing and tested their respective relations to patients' visuospatial WM imprecision.

**Methods:** Individuals with SZ and demographically-matched healthy controls completed a perceptual discrimination task to estimate levels of IN and an analog visual WM task to examine the impact of internal noise on WM precision. The discrimination task involved distinguishing orientations of briefly presented gratings (1 cycle/°; tilted ±45° from vertical) embedded in varying levels of external noise (0–21%). Contrast thresholds were estimated, and additive and unfiltered IN levels were modeled from task performance with the PTM. The WM task required reproducing remembered orientations of high-contrast gratings (same size and spatial frequency as in the discrimination task) with a manual dial at a 1s delay. WM precision was computed as the concentration of the von Mises distribution, fit from subjects' orientation errors.

**Results:** Additive and unfiltered IN during perceptual discrimination were both significantly increased in SZ compared to HC. WM precision was reduced in SZ compared to HC at every set size. Levels of unfiltered IN negatively correlated with WM precision in SZ, while both unfiltered and additive IN negatively correlated with WM precision in HC. For SZ, unfiltered IN was also negatively correlated with IQ, and WM precision was positively correlated with IQ in both groups.

**Discussion:** We found evidence of elevated IN levels during visual perception in SZ, though only unfiltered IN was inversely related to patients' visual WM precision. Thus results indicate overall 'noisy' visual perception in SZ, but point to a more precise model of poorer signal filtering or noise suppression as contributing to WM deficits and potentially broader cognitive impairment. Future work must identify the neural drivers of IN levels, as they may shed light on differential implications of the excitation/inhibition imbalance in WM networks. Findings underscore the link between perception and WM encoding in SZ and offer a novel computational strategy for identifying common and unique pathophysiological mechanisms of SZ cognitive dysfunction.

### 18.3 DISTURBED AND INTACT ATTENTIONAL PROCESSES DURING WORKING MEMORY ENCODING IN SCHIZOPHRENIA: CONVERGING BEHAVIORAL AND IMAGING GENETICS EVIDENCE

Robert Bittner*[,1], Peter Hahn[1], Catherine Barnes[1], Tom Lancaster[2], David Linden[2], Andreas Reif[1]
*[1]University Hospital Frankfurt, Goethe University; [2]MRC Centre for Neuropsychiatric Genetics & Genomics, Cardiff University School of Medicine*

**Background:** Working memory and attention are fundamental and closely linked cognitive domains. This close link is exemplified by the crucial role of selective attention for the selection of information to be encoded into working memory. Patients with schizophrenia are markedly impaired in many aspects of both domains. However, the interplay between these cognitive deficits on both the cognitive and neurophysiological level remains poorly understood. Based on our previous findings regarding the central role of impaired working memory encoding to working memory dysfunction in schizophrenia, we hypothesize, that impaired attentional processes contribute to working memory dysfunction specifically during the encoding stage. This hypothesis was tested in both a behavioral experiment and in an fMRI imaging genetics study.
**Methods:** For the behavioral study, we investigated 35 patients with schizophrenia and 35 matched healthy controls. In a change detection task, participants were simultaneously presented with both highly salient and non-salient spatial information. They were instructed to encode either the highly salient or the non-salient information. In half of the conditions, they were aided by a cue pointing them towards the relevant information. Our goal was to test, whether patients with schizophrenia were biased toward a particular type of information and whether a top-down cue would influence such a bias. In the imaging genetics study, we investigated 100 right-handed individuals without personal or family history of psychiatric disorders, who performed a visuospatial change detection task. The fMRI data were preprocessed and analyzed using Brain Voyager QX 20. For genotyping we used a custom Illumina HumanCoreExome-24 BeadChip array. We calculated polygenic scores (PGS) for schizophrenia based on 108 loci associated with schizophrenia in a recent mega-analysis of the Psychiatric Genetic Consortium (PGC2). We computed whole brain correlations between BOLD activation and PGS to elucidate the relationship between genetic risk for schizophrenia and abnormal brain function.
**Results:** In the behavioral study, patients were significantly more impaired when required to encode non-salient compared to salient information. However, this impairment was specific to conditions without a top-down cue. This demonstrates, that patients could use top-down attention to overcome a bottom-up bias towards highly salient information during working memory encoding. In the fMRI data, we observed a significant negative correlation between BOLD activation in the right temporo-parietal junction (TPJ) during working memory encoding and PGS for schizophrenia. Across all subjects, this area showed robust deactivation during encoding. The TPJ is a crucial region of the ventral attention network and is closely involved in bottom-up attentional processes. Previous fMRI studies observed stronger deactivation of the TPJ during working memory encoding with increasing cognitive demand. Therefore, our results indicate that participants with a higher genetic risk for schizophrenia had to commit more cognitive resources by downregulating their ventral attention network.
**Discussion:** Taken together, the results of both studies point toward specific disturbance of bottom-up attention during working memory encoding, which might be linked to genetic risk for schizophrenia. Conversely, top-down attention appears to be relatively spared. These findings provide new constraints for cognitive and neurophysiological models of impaired working memory encoding in schizophrenia.

### 18.4 CHARACTERIZING LARGE-SCALE NETWORK DYSCONNECTIVITY IN SCHIZOPHRENIA: MODELING ALTERED E/I BALANCE

Alan Anticevic*[,1]
*[1]Yale University*

**Background:** Neuropsychiatric disorders alter the structure and function of neural circuits and distributed neural networks, which leads to profound behavioral disability such as working memory impairment. Non-invasive neuroimaging tools have matured to reliably detect neural systems-level disturbances in neuropsychiatric disorders. However, mechanistic mapping from neural circuit pathology to abnormal behavior remains out of reach for most psychiatric conditions. We focus on the emerging field of 'computational psychiatry' to close these gaps from the perspective of E/I imbalance in schizophrenia with an emphasis on understanding large-Scale network dysconnectivity in schizophrenia.
**Methods:** First, we apply computational microcircuit models to understand cognitive and neural system-level disturbances in schizophrenia as a function of altered E/I balance. In turn, the focus is placed on pharmacological neuroimaging as a powerful causal tool to probe neural circuit perturbations. Specific recent neuroimaging studies are discussed that use the NMDA receptor antagonist, ketamine, to probe glutamate synaptic dysfunction associated with schizophrenia. We leverage resting-state neuroimaging advances to inform biomarker development for network alterations in schizophrenia given its ease of data collection and lack of task-based confounds.
**Results:** Pharmacological and clinical results implicate alterations in cortico-striato-thalamic circuits, which might constitute a final common pathway of neural system disturbances in schizophrenia. Results indicate that this functional marker appears in at-risk states, chronic patients, and following pharmacological manipulations following ketamine. Computational simulations implicate altered E/I balance in cortical microcircuits as a parsimonious upstream mechanism.
**Discussion:** The combined use of biophysical computational modeling, extended to large-scale neural system simulations, has proven particularly powerful to draw inferences about neural circuit alterations that may be 'driving' the resting-state dysconnectivity and cognitive deficits in schizophrenia. In summary, present data illustrates a framework for linking experimental studies in humans with computational models and pharmacological probes will advance to effort to bridge cellular, systems, and clinical neuroscience approaches to psychiatric disorders.

### 19. THE IMPLEMENTATION OF COGNITIVE ADAPTATION TRAINING (CAT) ACROSS COUNTRIES

Dawn Velligan
*University of Texas Health Science Center at San Antonio, School of Medicine*

**Overall Abstract:** Cognitive Adaptation Training is a psychosocial treatment using environmental supports such as signs, checklists, electronic devices and the organization of belongings to cue and sequence adaptive behavior in the home or work environment. In randomized trials, CAT has been found to improve multiple domains of functional outcome for individuals with schizophrenia including, social and occupational functioning, recidivism, medication follow through and community adjustment. Implementation of CAT has taken multiple forms across various countries. In Canada, a model has been adopted in which CAT is provided by occupational therapists (OT) for 4 months. During this time. the OT trains a caseworker to continue CAT after the 4 month time frame. This method has produced large effect sizes for sustained functional changes over time in patients receiving CAT treatment. In the Texas Money Follows the Person

Behavioral Health Pilot, CAT has been used in conjunction with substance use treatment and managed care service coordination to assist people in moving from nursing facilities to community living. 65% of all enrollees in the program have sustained independent living at 1 year. Improvements in functional outcome are evidenced by significant improvement in social and occupational role functioning, community living and quality of life. In addition, cost savings to the state for these Medicaid recipients is sizeable. It takes only 1.4 additional months of community residence to recover intervention costs; 97% of participants met these criteria. Also in Texas, in a program for high utilizers of hospital and emergency services, CAT provided by bachelor's and master's level psychologists has been used to identify the unique cause of multiple hospitalizations and emergency visits, set up supports to address these problems, and reduce inappropriate service utilization. This program has reduced hospitalizations by 80% and saved an average of $40,000 per participant across 9 months. In Finland, CAT has been used to reduce the need for sheltered housing and for improving the quality of life for outpatients with psychosis. CAT has been found to fit well in the Finnish service system and providing interventions in the patient's daily living environment has been found to be more suited to the patients' need than traditional outpatient clinic visits. An outcome study is ongoing. In Australia, CAT has been adapted for the recent onset psychosis population by including greater use of technology and focusing on return to work and school. The versatility of the CAT program, its facility for training, and its ability to be adapted across cultures and countries suggests that CAT could be a useful tool in value-based care in which CAT is used to reduce the overall cost of care and to improve outcomes that matter to patients.

### 19.1 FROM CLINICAL TRIAL TO THE CLINIC: OPTIMIZING COGNITIVE ADAPTATION TRAINING FOR CASE MANAGEMENT TEAMS

Sean Kidd*[,1], Yarissa Herman[2], Gursharan Virdee[2], Chris Bowie[3], Dawn Velligan[4], Natalie Maples[4]
*[1]University of Toronto; [2]Toronto Centre for Addiction and Mental Health; [3]Queen's University; [4]University of Texas Health Science Center at San Antonio, School of Medicine*

**Background:** Cognitive Adaptation Training (CAT) has consistently demonstrated effectiveness in enhancing community functioning in clinical trials of its 9-month application by a specialist. This is a compelling development in the field as clinicians struggle to support gains in independent functioning among patients with schizophrenia. However, outreach interventions delivered by specialists are difficult to support in many contexts where investment in mental health care is insufficient for population needs. This presentation will describe research and implementation efforts that support the delivery of CAT in routine clinical practice.
**Methods:** This program of work began with a feasibility study of a modified version of CAT. CAT was modified to decrease the duration of specialist-delivered CAT to 4 months, with the intervention subsequently supported by the individual's case manager who received rudimentary training and could consult specialists. Twenty-three people with schizophrenia participated in this study of symptom and functional outcomes, evaluating improvements after 4 months of CAT specialist intervention and after an additional 5 months of case manager support. Also described briefly will be (i) preliminary findings from a superiority randomized controlled trial of modified CAT in an early intervention population comparing CAT (n=25) with Action Based Cognitive Remediation (n=23) and (ii) efforts to build out CAT implementation in a tertiary facility enabled through the above clinical trial resources.
**Results:** Analysis of feasibility study findings revealed significant improvements in adaptive functioning, psychiatric symptomatology, and goal attainment that were maintained throughout case management follow-up. Effect sizes for the specialist delivered period ranged from .33 (negative symptoms) to 2.01 (goal attainment scaling) with a modest decline in the follow-up period with community functioning remaining at ES=.66. Improvement in the large effect size range was also observed in community functioning in the trial of modified CAT in early intervention. In this period over 70 allied health clinicians were intensively training in CAT locally and regionally and a community of practice was established. These impacts were further extended through the development of an open-access CAT guide for families that can be used independently or with clinician support.
**Discussion:** This study supports a model for extending the accessibility of CAT in settings that might not otherwise sustain the intervention as it was originally designed. Functional impacts similar to the original clinical trials were observed in a briefer period of specialist delivered CAT and show the promise of being largely sustained over an indefinite period by rudimentary-trained case managers in a consultation model. This observation would appear to apply to both early intervention and general schizophrenia populations. Additionally, this program of work has demonstrated how research-practice synergies can foster implementation that can be sustained after initial research investments.

### 19.2 RECOVERY THROUGH RELOCATION: FROM NURSING HOME TO COMMUNITY USING COGNITIVE ADAPTATION TRAINING

Natalie Maples*[,1]
*[1]University of Texas Health Science Center at San Antonio*

**Background:** Texans with severe mental illness live 29 years less than other Americans and have more health problems earlier in life. Since 2001, over 46,000 Texans have returned home under the State's Money Follows the Person program and federal demonstration grant. Despite this impressive achievement, people with mental health and substance use conditions continue to be institutionalized in nursing facilities (NF). Nationally, the number of NF residents under age 65 with a primary diagnosis of mental illness is nearly three times that of older residents. The Texas Money Follows the Person Behavioral Health Pilot (MFP-BHP), assists nursing facility residents with co-morbid mental and physical illnesses relocate into the community. The transition from institutionalization to independent living is a crucial time for treatment intervention to maintain independence and reduce high risk adverse outcomes, including hospitalization, exacerbation of symptoms or homelessness.
**Methods:** In addition to service coordination from Managed Care Organizations, participants receive Cognitive Adaptation Training (CAT) for six months in the nursing facility and one year in the community. CAT is a home-based psychosocial treatment utilizing environmental supports such as medication containers, signs, checklists and the organization of belongings to bypass deficits in cognitive functioning and cue and sequence adaptive behavior, to improve functional outcomes for individuals with mental illness. This demonstration project assessed the effectiveness of providing CAT to improve functional and social outcomes, measured at baseline, each three months for one year, and each six months post intervention for one additional year.
**Results:** Over 500 individuals have been transitioned into the community since 2008, with 60% maintaining independence. Findings indicate a significant improvement in targeted functional outcomes post facility discharge on the Multnomah Community Ability Scale ($p<.0001$), Social and Occupational Functioning Scale ($p<.001$) and the Quality of Life Scale ($p< .01$). Preliminarily analyses indicate that Medicaid costs for participants are considerably lower on average than costs prior to discharge. At the end of 2015, the savings to the state via the Pilot were tens of millions. The Pilot ends in December 2017 and final cost analyses will be conducted at this time.
**Discussion:** CAT was successfully applied to persons with co-occurring mental and physical disorders relocating from nursing facilities to independent living environments with good preliminary outcomes indicating better quality of life, social and occupational role function and in overall community functioning. The majority of persons have successfully remained in the community. The MFP Behavioral Health Pilot shows Medicaid participants residing in nursing facilities with significant mental health issues can successfully maintain their residence in the community which results in significant cost savings, even taking into account the standard MFP costs plus the intervention. MFP Pilot participants improved their functional status, which extended after the intervention period ended. Current implementation efforts are in place to integrate and sustain CAT in the statewide managed care system.

### 19.3 APPLYING COGNITIVE ADAPTATION TRAINING IN FINLAND: INTERIM RESULTS OF THE FINNISH CAT IMPLEMENTATION PROJECT

Tuukka Mehtala*[,1], Satu Viertio[2], Eila Sailas[3]
*[1]HUS; [2]National Institute for Health and Welfare; [3]Kellokoski Hospital*

**Background:** In Finland, approximately 50 000 people have a diagnosis of schizophrenia. In practice 6% of them reside permanently in mental hospitals. There is a national target to reduce the number of psychiatric hospital beds. However, as hospitals are closed there is a tendency to place schizophrenia patients in different types of sheltered housing instead of supporting them to live independently in the community. In the Danish OPUS-study 94 patients with first episode schizophrenia were followed and even those who had attended a vigorous rehabilitation program lived about two and a half months in sheltered housing in the fifth year after their diagnosis. Thus, with deinstitutionalization we are building up a poorly monitored system of sheltered housing for schizophrenia patients. This system may increase chronic need for support, is expensive and marginalizes a large section of people from the community. When service users are asked they usually prefer having their own homes.
Cognitive adaptation training (CAT) is a home-based, manual-driven treatment that utilizes environmental supports and compensatory strategies to bypass cognitive deficits and improve target behaviors and functional outcomes in individuals with schizophrenia. Unlike traditional case management, CAT provides environmental supports and compensatory strategies tailored to meet the behavioral style and neurocognitive deficits of each individual patient. CAT has been shown to be effective in improving service users' ability live independently.
**Methods:** The study started in 2014. After formal CAT training the program was implemented in the Hyvinkää Hospital and Helsinki University Central Hospital treatment catchment areas (approx. 1 350 000 inhabitants). For the study we selected patients that were in risk of moving to a more supported housing environment due to the presence of cognitive deficits that threatened their ability to live independently. The only exclusion criteria were heavy alcohol and drug abuse and known aggressive behavior. The outcome measurements include both qualitative and quantitative methods: transfer to a different type of housing, need for hospital treatment, psychiatric rating scales, observed measurements and open interviews, and are measured after 4 months after the start of the intervention, at the end of the 9 month intervention and after a 6 months follow-up period.
**Results:** We report here preliminary interim results for the patients who have completed the study so far. Altogether 48 patients were selected for the intervention, which was found to be well-received with 7 patients dropping out. The mean age was 38.9 year, with 39.3 % women and 60.4 % men. 27.6 % were living independently, 22.9 % with their parents, and 29.6 % living in some form of sheltered housing. Participants had severe to moderately-severe psychiatric symptoms and functional impairment (mean GAF 47.8, mean SOFAS 54.8). Apathetic was the most common behavioral subtype (70.7 %), with disinhibited (14.6 %) and mixed (14.6 %) subtypes following.
**Discussion:** Cognitive Adaptation Training can be used to help patients in a wide range of living situations and with severe psychiatric symptoms and functional impairment to maintain their ability to live independently.

### 19.4 CAT IN FIRST-EPISODE PSYCHOSIS: FEASIBILITY, ACCEPTABILITY AND POTENTIAL TO ENHANCE VOCATIONAL RECOVERY

Kelly Allott*[,1]
*[1]Orygen, The National Centre of Excellence in Youth Mental Health*

**Background:** Cognitive and functioning impairments are present early in the course of psychotic disorder and remain one of the greatest treatment challenges in this population. While Cognitive Adaptation Training (CAT) is found to improve a range of outcomes in chronic schizophrenia, it has received limited investigation in first-episode psychosis (FEP). CAT may be particularly useful for addressing vocational recovery in FEP because the cognitive impairments experienced by individuals with FEP predict poorer vocational outcomes and impede the effectiveness of vocational interventions such as supported employment. The aim of this presentation is to present the findings of a pilot study investigating the feasibility and acceptability of CAT in young people with FEP and to describe the clinical considerations and adaptations required when delivering CAT with this population. Preliminary findings on the potential value of CAT in improving vocational outcomes in FEP will also be presented.
**Methods:** This was a single-arm feasibility study of CAT conducted at the Early Psychosis Prevention and Intervention Centre (EPPIC), Melbourne, Australia. Five FEP participants received 9 months of manually-guided CAT. A range of feasibility and acceptability measures were recorded, including participant and case manager satisfaction ratings. Participants' goals, functional needs and clinical observations and adaptations were also recorded. Formal measures of functioning, quality of life and motivation were independently administered pre- and post-intervention.
**Results:** All participants completed the CAT intervention and session attendance rates were very high (95.3%). Participants and their case managers indicated strong satisfaction with CAT as indicated by overall positive mean ratings on the satisfaction items. CAT did not negatively affect existing case management, with case managers reporting that it enhanced their treatment. Vocational recovery (education, employment) was found to be a primary functional goal of most participants. Accordingly, the CAT intervention had a strong focus on vocational functioning, including functional domains that are requisite for successful work or educational outcomes, including organisation and planning, transportation and activities of daily living. Being mindful of factors that may be common in young FEP patients included cognitive heterogeneity, family involvement, flexibility in compensatory and environmental supports used, and the experience of stigma. There were mean improvements from baseline to post-intervention on most formal outcome measures, with the largest effects in global functioning, planning and organisation, and quality of life.
**Discussion:** This study provides encouraging preliminary evidence that CAT is a highly feasible and acceptable intervention in FEP, which may be easily integrated within existing early intervention services. Vocational recovery is important to young people with FEP. CAT is an intervention that appears well suited to addressing this need. The effectiveness of CAT in improving functional outcomes, particularly vocational recovery in FEP warrants further investigation in a larger trial.

### 20. THE APPLICATION OF STEM CELL MODELS TO VALIDATE RARE AND COMMON VARIANTS CONTRIBUTING TO SCHIZOPHRENIA

Kristen Brennand
*Icahn School of Medicine at Mount Sinai*

**Overall Abstract:** As expanding genetic studies increasingly demonstrate that both rare variants of large impact and common variants of small effect contribute to schizophrenia, it becomes increasingly critical that we unravel how these risk factors interact within and between the diverse cell types populating the brain. While mouse models are uniquely suited for demonstrating how aberrant function of single gene products contribute to aberrant neuronal function or behavior, genetic studies of penetrance and complex gene interactions are nearly impossible to address using inbred mouse lines. Similarly, the lack of human post-mortem tissue, coupled with the inability to conduct functional experiments in patient cells, has to date left us with a very limited understanding of how rare and common variants impact gene expression or cellular function. Our panelists have each developed human induced pluripotent stem cell (hiPSC)-based models for the study of predisposition to neuropsychiatric disease, establishing a new mechanism by which to systematically explore the impact of rare and common putative causal variants in human cells.
Given the heterogeneity of schizophrenia and the limited cohort sizes feasible with hiPSC-based cohorts, our panelists will share their successes and struggles in developing cohorts defined by shared clinical or genetic features. They will discuss both the molecular and phenotypic insights they have uncovered, in neurons and glia, from case/control and genetically-edited isogenic cohorts. Our discussant will focus on integrating these findings into consortia-led datasets generated from recent genomic and post-mortem studies of large schizophrenia cohorts. Our overall objective is to consider the role of hiPSC-based studies in dissecting the genetic origins of schizophrenia, validating causal variants identified through ongoing genetic analyses, and serving as a personalized medicine approach to screen for novel therapeutics with which to prevent or reverse disease course.

### 20.1 DISSECTING THE FUNCTIONAL CONSEQUENCES OF RECIPROCAL GENOMIC DISORDERS

Michael Talkowski*,[1]
[1]*Massachusetts General Hospital, Harvard Medical School*

**Background:** Reciprocal genomic disorders (RGDs) represent a unique class of recurrent genomic variation that offer insight into highly dosage sensitive regions of the morbid human genome. However, the genomic architecture mediating RGDs, namely non-allelic homologous recombination (NAHR) of flanking segmental duplications, has rendered these genomic segments recalcitrant to conventional model studies. We recently developed a novel CRISPR method that leverages the homology of segmental duplications and efficiently generates large microdeletions and microduplications that mimic NAHR in humans, including ablation or duplication of one copy equivalent of the segmental duplications. Here, we explore the functional consequences of 16p11.2 RGD in iPS derived neuronal models and across mouse tissues.
**Methods:** We generated CRISPR-engineered 16p11.2 RGD models against an isogenic iPSC background and performed transcriptome profiling in iPSC-derived neural stem cells (NSCs) and induced neurons (iN) (n = 10 isogenic deletions, 10 duplications, 6 controls). We then integrated these data with RNAseq from 306 libraries from multiple tissues in 70 mouse models of reciprocal deletion and duplication of the syntenic 7qf3 region (cortex, striatum, cerebellum, liver, white fat, brown fat in 16 mice; and replication from cortex, striatum, cerebellum in 54 mice).
**Results:** In ongoing analyses, weighted-gene correlation network analysis (WGCNA) identified co-expression modules that were significantly enriched for 16p11.2 genes, evolutionarily constrained genes, genes robustly associated with autism spectrum disorder (ASD; TADA $q < 0.1$) and developmental disorders (DDD). Pathway analyses within modules discovered enrichment of genes critical to synaptic formation and neural connectivity as well as the protocadherin gene family. Network analyses specific to brain tissues within modules further identified a convergence on highly connected, or 'hub' genes, on Wnt signaling, including Ctnnb1 and Ctnnd1. The module was also again enriched for ASD loci (TADA, $FDR < 0.1$), constrained genes (ExAC, $pLI \geq 0.9$) and brain specific genes from the Human Protein Atlas.
**Discussion:** These studies suggest the functional consequences of 16p11.2 RGD across models converge on transcriptional signatures associated with critical neurodevelopmental pathways and individual genes implicated in a spectrum of developmental and neuropsychiatric disorders.

### 20.2 ANALYZING THE MOLECULAR EFFECTS OF LARGE NEUROPSYCHIATRIC CNVS WITH IPSC BASED NEURONAL TISSUE CULTURE MODELS

Alexander Urban*,[1]
[1]*Stanford University*

**Background:** Several large copy number variants (CNVs) in the genomic sequence are strongly associated with schizophrenia. These loci are important objects of study in their own right as well as enticing points of entry for the better understanding of the molecular etiology of schizophrenia. However, most of the schizophrenia-associated large CNVs are larger than one million base pairs and affect up to several dozen genes, presenting a complex challenge for research aiming to determine how these sequence variants are connected on the molecular level to the phenotype.
**Methods:** We have established iPSC based tissue culture models for three of the major schizophrenia associated large CNVs, on chromosomes 22q11 (deletion), 15q13 (deletion) and 16p11 (deletion or duplication). We create neuronal cells with the defined genotypes using either direct induction into the neuronal state (induced neurons, iNs), by slower differentiation via neuronal precursor cells (NPCs) or by generating 3D cultures of cortical spheroids. We then assay the molecular effects of the large CNVs along the trajectory of differentiation by using RNA-Seq (transcriptome), ATAC-Seq (chromatin state) and SeqCap-Epi (DNA-methylation patterns). We also carry out single-cell RNA-Seq analysis using the drop-Seq approach.
**Results:** We detect common effects across the large CNVs as well as locus-specific phenomena. For the most part genes within the CNV boundaries will change their expression patterns in concordance with their new copy number, with notable exceptions. Transcriptome-wide there is a network effect where several hundred genes are differentially expressed, including genes already identified as candidate genes for schizophrenia. Epigenomic states are affected, again most often not only in or nearby the boundaries of the large CNVs but epigenome-wide. Integrative analysis across the layers of molecular signals shows partial concordance as well as a degree of changes in signal being 'offset' between the levels, potentially owing to the dynamic differentiation state of the model system.
**Discussion:** Neuronal tissue culture models based on iPSCs with defined large CNVs strongly associated with Schizophrenia allow for an analysis of the effects of such structural genomic sequence changes in disease-relevant cellular differentiation states. Application of cutting edge genomics and epigenomics assays and integrative data analysis reveals incomplete transcriptional dosage compensation of the genes within the large CNVs as well as transcriptome-wide network effects. Furthermore, there are epigenomic effects in the form of altered chromatin states that may to some extent mediate the gene expression changes. Differences between the large CNV loci as well as potential points of convergence will be discussed.

### 20.3 OPEN CHROMATIN ANALYSES INFORM FUNCTIONAL NONCODING GWAS VARIANTS IN HIPSC MODEL OF MENTAL DISORDERS

Jubao Duan*,[1]
[1]*NorthShore University Health System/University of Chicago*

**Background:** Neuropsychiatric disorders, including schizophrenia (SZ), afflict a significant fraction of the population. Recent genome-wide association studies (GWAS) under the framework of the Psychiatric Genomics Consortium (PGC), along with large-scale sequencing efforts, have identified a plethora of disease risk loci with common and/or rare risk variants. Translating these exciting genomic findings into causation and disease biology offers the promise of developing more tailored therapies in psychiatry. However, understanding the disease biology underlying most GWAS findings remains challenging: (1) The paucity of disease-relevant biological materials for assaying molecular and cellular phenotypes associated with risk loci; (2) Most disease variants lie within poorly-annotated noncoding parts of the genome for which functional interpretation is challenging; and (3) Each locus often contains many genes/variants equivalently associated with the disease due to linkage disequilibrium, and it is difficult to identify which are the likely causal gene/variant. Human neurons derived from induced pluripotent stem cells (iPSCs), both monolayer cultures (2D model) and the emerging brain organoids (3D model), provide a promising alternative to human brains for recapitulating cellular phenotypes relevant to psychiatric disorders. CRISPR/Cas9 editing further strengthens the utility of these models by enabling the generation of isogenic lines with essentially the same genetic background on which allelic effects of a risk variant can be directly compared, thus increasing the sensitivity to detect typically small effects of a GWAS variant.
**Methods:** To functionally assess the relevance of noncoding sequences in neuropsychiatric disorders, we hypothesized that disease-relevant noncoding sequences likely overlap with cell-specific open chromatin regions (OCRs). We have carried out a genome-wide OCR profiling of excitatory neuronal differentiation from human iPSCs using an Assay for Transposase-Accessible Chromatin by sequencing (ATAC-seq).
**Results:** We found that OCRs in neurons were enriched SZ risk variants in neural OCRs and can help prioritize putatively functional SZ risk variants that may impact OCRs and consequently, cellular development. At a leading SZ-risk locus flanking MIR137, we further examined the functional effects of a prioritized common GWAS SNP rs1198588 in CRISPR/Cas9-edited hiPSCs, and found that SZ-risk allele of rs1198588 altered MIR137 expression, OCR dynamics and dendrite arborization/synapse maturation. To systematically identify such disease risk variants that may affect OCR, we further carried out a proof-of-concept analysis of allele-specific open chromatin (ASoC) of in hIPSC-derived neurons. We found that Heterozygous SNPs showing ASoC are more prevalent in neurons than in hiPSCs. Out of the 12 schizophrenia GWAS-implicated SNPs that we found in neuronal OCRs of this single individual, two SNPs showed ASoC and are thus putatively functional: one lies within the 5'-UTR of CHRNA5 (cholinergic receptor, nicotinic, alpha 5) and the other is in the promoter region of VPS45, a Sec1 family gene involved in synaptic transmission. We are currently in the process of replicating the observed landscape of ASoC in iPSC-derived neurons from a larger sample pool.
**Discussion:** Our study suggests that OCR profiling in a human iPSC model of neuron differentiation can predict functional noncoding sequences that regulate neurodevelopment.

### 20.4 MODELING THE CONTRIBUTION OF COMMON VARIANTS TO SCHIZOPHRENIA RISK

Gabriel Hoffman[1], Brigham Hartley[1], Douglas Ruderfer[1], Judith Rapoport[2], Pamela Sklar[1], Kristen Brennand*,[1],
[1]*Icahn School of Medicine at Mount Sinai;* [2]*NIH*

**Background:** Schizophrenia (SZ) is a debilitating psychiatric disorder for which the complex genetic mechanisms underlying the disease state remain unclear. Whereas highly penetrant variants have proven well-suited to human induced pluripotent stem cell (hiPSC)-based models, the power of hiPSC-based studies to resolve the much smaller effects of common variants within the size of cohorts that can be realistically assembled remains uncertain.
**Methods:** We reprogrammed fibroblasts from SZ patients into hiPSCs and subsequently differentiated these disorder-specific hiPSCs into neural progenitor cells (NPCs) and neurons. Our hiPSC neural cells, from controls and patients with SZ, better resemble fetal rather than adult brain tissue, indicating that hiPSC-based models may be best suited for studies of disease predisposition. At the cellular level, we have previously reported aberrant migration in SZ hiPSC NPCs, together with diminished neuronal connectivity and impaired synaptic function in SZ hiPSC neurons.
**Results:** We identified microRNA-9 as having significantly downregulated levels and activity in a subset of SZ hiPSC-derived neural progenitor cells NPCs, a finding that was corroborated by a larger replication cohort and further validated by an independent gene-set enrichment analysis of the largest SZ genome-wide association study (GWAS) to date. Overall, this demonstrated a remarkable convergence of independent hiPSC- and genetics-based discovery approaches. In developing this larger case/control SZ hiPSC cohort of hiPSC-derived NPCs and neurons, we identified a variety of sources of variation, but by reducing the stochastic effects of the differentiation process, we observed a significant concordance with two large post mortem datasets.
**Discussion:** We predict a growing convergence between hiPSC and post mortem studies as both approaches expand to larger cohort sizes. Meanwhile, we have been integrating CRISPR-mediated gene editing, activation and repression technologies with our hiPSC-based neural platform, in order to develop a scalable system for testing the effect of a manipulating the growing number of SZ-associated variants and genes in NPCs, neurons and astrocytes. Altogether, our objective is to understand the cell-type specific contributions of SZ risk variants to disease predisposition.

### 21. IDENTIFYING INDIVIDUALS AT HIGH RISK FOR SCHIZOPHRENIA: LJ SEIDMAN MEMORIAL SYMPOSIUM

Lynn DeLisi
*VA Boston Healthcare System, Brockton Division*

**Overall Abstract:** Lawrence J. Seidman was born and grew up in New York City. He obtained a PhD from Boston University in Psychology and stayed in Massachusetts to work for many years in the Harvard affiliated Hospitals, such as the VA-Boston Healthcare System, Massachusetts General Hospital and at his untimely passing, he was Professor of Psychology in the Department of Psychiatry at Beth Israel-Deaconess Hospital and the Massachusetts Mental Health Center. He was about to move to a new phase in his career, assuming a position at Children's Hospital, Boston. He was a pioneer in the fields of the neuropsychology of schizophrenia, ADHD and related disorders, of using the tools of cognitive assessments and brain imaging to understand the genetic predisposition for serious mental illness, and in the last several years—prediction of conversion to psychosis in individuals at high risk. He contributed to many multicenter collaborations and had several collaborators world-wide, playing an important role in their work. This symposium is conducted in his honor with contributions from key collaborators on different aspects of his work. Drs. Tyronne Cannon and Elaine Walker will both represent the North American Prodrome Longitudinal Study (NAPLS) consortium by reviewing its findings in brain imaging and cognition. Dr. David Braff will review the work of the Consortium on the Genetics of Schizophrenia (COGS) multicenter collaboration, in which Dr. Seidman led one of its sites, and Dr. TianHong Zhang from Shanghai will present current data from the Shanghai-Boston SHARP collaboration on early detection of psychosis. The symposium will be concluded by Dr. Keshavan from Beth Israel-Deaconess, who was a close colleague of Dr. Seidman for the last decade. Together they worked side by side exploring many aspects of early detection of schizophrenia and educating trainees and the public on their findings. He will sum up the legacy of Dr. Seidman to the field and how we can continue it into the future.

### 21.1 STRESS AND COGNITION IN YOUTH AT CLINICAL RISK FOR PSYCHOSIS

Elaine Walker*[,1]
[1]*Emory University*

**Background:** Heightened Stress has been shown to have acute adverse effects on cognitive function, and both stress and cognitive deficits have been found to be inversely associated with hippocampal volume reduction in both healthy and clinical samples. Further, heightened stress and cognitive deficits have been observed in individuals at clinical high risk (CHR) for psychosis in the North American Prodrome Longitudinal Study (NAPLS-2) as well as other studies of CHR groups. The present study utilizes data from NAPLS-2 to examine the relation of acute stress and hippocampal volume with cognitive performance in healthy youth and those at CHR for psychosis. Both the independent and additive relations of daily stress and hippocampal volume (HV) on cognition are examined.
**Methods:** The sample was 666 participants (CHR=476; HC=190) who completed MRI scans, as well as measures of stress and cognitive function at the NAPLS-2 baseline assessment. The self-report stress measure was the Daily Stress Inventory (DSI) and cognitive performance was assessed with tests from the Measurement and Treatment Research to Improve Cognition in Schizophrenia (MATRICS) battery (the Brief Assessment of Cognition in Schizophrenia [BACS]: Symbol Coding, Category Fluency: Animal Naming Trail Making Test: Part A [TMT], Continuous Performance Test—Identical Pairs [CPT-IP], Hopkins Verbal Learning Test—Revised [HVLT-R], Brief Visuospatial Memory Test—Revised [BVMT-R], Neuropsychological Assessment Battery® (NAB): Mazes, and Wechsler memory Scale [WMS]). Hierarchical Linear regression analyses were conducted controlling for subject age, sex and intracranial volume (ICV) with test scores as the dependent measures and DSI scores and hippocampal volume as the predictors.
**Results:** As expected, across subject groups, age was positively associated with performance on all of the cognitive measures, and females scored higher than males on the CPT-IP, WMS, and TMT. ICV was positively linked with performance on the TMT, WMS, NAB Mazes, CPT-IP, and BACS. For HC and CHR subjects combined, after entering covariates (sex, age and ICV), DSI, but not HV, was inversely associated with performance on the BVMT, HVLT, CPT-IP, and WMS. Neither DSI nor HV predicted performance on the NAB mazes or the BACS, beyond the variance accounted for by covariates. When analyses were conducted separately for the HC and CHR groups, DSI was not predictive of performance.
**Discussion:** The present findings indicate that self-reported daily stress is inversely associated with cognitive performance on a variety of measures, and that this relation is not mediated by HV or any of the covariates in the combined sample of HC and CHR youth. Because the two groups differ in DSI scores, with CHR youth showing significantly higher stress scores, the combined samples represent a broader range of scores than either group alone. Thus, the within-group range of DSI scores is constrained and DSI is not predictive of performance within group. Instead, it appears that elevated stress is one factor contributing to cognitive deficits in CHR youth.

### 21.2 FAMILIAL RISK FOR SCHIZOPHRENIA

David Braff*[,1]
[1]*University of California, San Diego*

**Background:** Larry Seidman, Ph.D. was a key contributor to the Consortium on the Genetics of Schizophrenia (COGS) with its focus on understanding the genetic substrates of quantitative endophenotypes in schizophrenia patients. With his deep knowledge of neurocognition related to psychosis, Larry was able to help steer the COGS-1 family study of over 300 families. The subsequent COGS-2 case-control study used the same well curated, quality controlled extensive battery of testing with 1411 schizophrenia patients and 1500 extensively tested healthy control subjects. Larry was first author on the 2015 paper "Factor structure and heritability of endophenotypes and schizophrenia: findings from the Consortium on the Genetics of Schizophrenia." It is important to note that related association studies examined the relationship of quantitative endophenotypes and genetic loci, and this is complementary to but distinct from case-control studies. These COGS studies identified a 42-gene network with a NRGL-ERBB4 hub underlying schizophrenia neurocognitive deficits. Thus, these Ns, modest for case-control studies, are quite powerful for gene finding using quantitative endophenotypic markers related to core "thought disorder" neurocognitive deficits in schizophrenia. These quantitative measures are up to 100 X more efficient and 10 X more powerful for gene finding than case-control studies as explained by Blangero, Williams and Almasy as early as 2005. Genes for SZ overlap with genes for key functionally important quantitative endophenotypes as shown by many groups, including COGENT and COGS, so case-control and endophenotype studies are best viewed as complementary in nature.
**Methods:** Seidman et al (2015) examined 12 heritable neurocognitive and neurophysiological domains (including the Penn CNB Battery), as well as three neurophysiological measures reflecting inhibitory reprocessing from EEG and eye movements. Seidman et al's analysis revealed five distinct factors in the composite COGS battery. These were 1-episodic memory, 2-working memory, 3-perceptual vigilance, 4-visual abstraction and 5-inhibitory processing. The five factors had similar structures across probands, siblings and controls. Also, heritability was significant for all 5 factors. These composite endophenotype factors will be used to enhance our neurobiological and genetic understanding of schizophrenia and its treatment as we move forward, and are related to the Biotype concept of psychosis. Larry Seidman was also an important contributor to the COGS mission as described below.
**Results:** The COGS PsychChip GWAS of quantitative endophenotypes has now identified six regions of association with quantitative neurocognitive measures exceeding genome-wide significance (e.g. NRGL3-Abstaction and Mental Flexibility on the CNB). In addition, many associations between endophenotypes and specific loci exceed the suggestive threshold for further investigation.
**Discussion:** These data will be presented implicating synaptic plasticity and other crucial CNS processes in endophenotype dysfunction in SZ. NB: COGS interrogates the genetic architecture of endophenotypes associated with SZ and its functional outcome, not schizophrenia per se. Still, there is much overlap between risk genes for SZ and neurocognitive endophenotypes. Also neurocognitive endophenotypes are endorsed by the FDA and MATRICS as treatment targets for schizophrenia itself. This allows for data-guided drug and sensory-cognitive remediation of neurocognitive deficits to improve functional outcome in schizophrenia. Using this genomic information to enhance precision based selection of treatment options now seems to be an exciting and viable new treatment pathway.

### 21.3 NEUROIMAGING MARKERS OF RISK FOR AND PROGRESSION TO FULL PSYCHOSIS IN THE NAPLS PROJECT

Tyrone Cannon*[,1]
[1]*Yale University*

**Background:** Dr. Larry Seidman made numerous impactful contributions to our understanding of the roles of disrupted neurocognition and brain function in individuals with or at risk for schizophrenia. Based in part on Larry's seminal work, many in the field have come to view schizophrenia as fundamentally a disorder of dysconnection within and between certain functional networks in the brain. However, what levels or patterns of dysconnection may be sufficient for overt psychosis remains unclear. Because schizophrenia is complexly determined, clinically heterogeneous, and (frequently) chronic and debilitating, neuroimaging studies comparing those with and without this condition cannot by themselves differentiate which neural changes contribute causally, which are epiphenomena, and which are secondary to factors associated with chronicity of illness or antipsychotic drug treatment. A crucial aim is thus isolation of the changes immediately preceding the onset of psychosis that, by virtue of their temporal priority, may represent primary mechanisms in the cascades of events leading to the emergence of psychosis.

**Methods:** Identifying such changes requires a paradigm for ascertaining at-risk individuals prior to psychosis onset and following them over time. Larry Seidman's early work found that both patients and their first-degree relatives fail to disengage the default mode network and fail to engage task-positive networks under cognitive challenge. In the early 2000's, in an effort to isolate changes in brain structure and function more proximal to the onset of psychosis, Larry joined with seven other investigators to launch the North American Prodrome Longitudinal Study (NAPLS). This talk will focus specifically on neuroimaging markers and on results examining baseline and longitudinal changes in brain structure and function among clinical high-risk (CHR) and control subjects, who were scanned at baseline and at 12-months or the point of conversion if it occurred earlier.

**Results:** Converters to psychosis showed a significantly steeper rate of gray matter thinning in right superior and medial prefrontal cortex (PFC) and greater ventricular expansion than non-converters and controls. These effects were significant controlling for multiple testing and independent of exposure to antipsychotic drugs. Higher levels of proinflammatory cytokines at baseline were predictive of steeper rates of gray matter reduction in superior and medial PFC, consistent with the notion that progressive gray matter change in this context is likely to reflect dendritic retraction and synaptic pruning driven by microglial activation. This interpretation is further supported by recent evidence of genetic susceptibility mechanisms involving complement signaling in schizophrenia, variations that appear to result in over-pruning of cortical synapses in animal models. In smaller subsamples using both task-based and resting-state fMRI, CHR subjects who converted to psychosis showed a progressive decrease in global efficiency and increase in network diversity from baseline to follow-up at the point of conversion. The identified network alterations were highly correlated with each other and with progressive gray matter changes in the prefrontal cortex in converters.

**Discussion:** These results are suggestive of a progressive loss of gray matter potentially triggered by altered immune signaling leading to over-pruning of synapses and provide preliminary evidence for longitudinal reconfiguration of resting-state and task-positive brain networks during psychosis development. The latter results appear to converge with Dr Seidman's pioneering work on default mode and task-positive network function in individuals at genetic risk for schizophrenia.

### 21.4 BASELINE CLINICAL AND BIOLOGICAL VARIABLES PREDICTING 1 YEAR OUTCOME OF SUBJECTS AT CLINICAL HIGH RISK OF PSYCHOSIS: INSIGHT FROM SHANGHAI AT RISK FOR PSYCHOSIS (SHARP) PROGRAM

TianHong Zhang*,[1], HuiJun Li[2], YingYing Tang[3], Chunbo Li[1], Kristen Woodberry[4], Daniel I. Shapiro[4], Margaret Niznikiewicz[5], Martha E. Shenton[6], Matcheri S. Keshavan[7], William S. Stone[4], Jijun Wang[1]

*[1]Shanghai Mental Health Center, Shanghai Jiao Tong University School of Medicine; [2]Florida A & M University; [3]Shanghai Mental Health Center, Shanghai Jiao Tong University School of Medicine, Shanghai Key Laboratory of Psychotic Disorders; [4]Beth Israel Deaconess/ Harvard Medical Center; [5]Harvard Medical School, Veterans Affairs Boston Healthcare System; [6]Psychiatry Neuroimaging Laboratory, Brigham and Women's Hospital, Harvard Medical School, Veterans Affairs Boston Healthcare System; [7]Massachusetts Mental Health Center Public, Beth Israel Deaconess Medical Center, Harvard Medical School*

**Background:** In 2010, the "ShangHai At Risk for Psychosis (SHARP)" study was launched at the Shanghai Mental Health Center (SMHC), the largest outpatient mental health clinic in China. The Chinese SHARP research was led by Dr. Larry Seidman, who was also the PI of the Harvard site of the NAPLS project. He had implemented methods very similar to those used in NAPLS for the identification of clinical high risk (CHR) individuals in Mainland China in studies jointly funded by the United States National Institute of Mental Health and Chinese funding agencies.

**Methods:** Dr. Seidman began a collaboration with the SMHC by advising us in carrying out an epidemiological study and then received joint funding for an R21 MH093294 (Fogarty/NIH, "Broadening the Investigation of Psychosis Prodrome to Different Cultural Groups") designed to implement a variety of clinical, neurocognitive and event related potential (ERP) measures in a preliminary study of CHR. That study, which began in April 2012 and ended in March 2015, aimed to build research capacity at the SMHC. He guide us provided 4 in-person research and clinical skills trainings (2 in SMHC, 2 in Boston), translated a widely used CHR diagnostic instrument (the Structured Interview for Prodromal Symptoms/SIPS), and trained China partners to conduct a preliminary study of 100 CHR individuals. Building upon the R21 project, and the North American Prodrome Longitudinal Studies (NAPLS) model, the same group of researchers led by Dr. Seidman is collaborating on an NIMH R01 101052-01 project (2013 to 2016) to examine biomarkers of CHR with 1 year follow-up. The R21 and R01 collaborations between Harvard Medical School (HMS), MIT, Florida A&M University (FAMU), and SMHC investigators have capitalized on the resources and experiences of the Harvard researchers as members of NAPLS, MIT researchers' leading role in functional magnetic resonance imaging (fMRI), and FAMU researchers' expertise in bridging western and Chinese cultures to enhance the existing capacity of Chinese researchers studying the biopsychosocial aspects of CHR. Finally, a stratified cohort of 300 CHR participants was recruited between 2012–2015, and followed up for at least 1 year.

**Results:** With the hope of Dr. Seidman, the SHARP project is ongoing and getting better, larger and stronger. Of the total 417 CHR participants (previous epidemiological survey [CHR, n = 117], R21 [CHR, n = 100], R01 [CHR, n =200]), 349 completed at least a year of follow-up (until August 30, 2017; the longest follow-up case was six and a half years), in which 83 converted to psychosis, and 68 were lost. Preliminary data showed about 20% CHR converted to a psychotic disorder over the course of follow-up, several clinical factors such as 1) functional decline; 2) selected positive symptoms(unusual thoughts and suspiciousness); 3) selected negative symptoms(social anhedonia, expression of emotion, and ideational richness); biological factors such as the P300 auditory ERP; fMRI: Reduced anti-correlation between the bilateral parietal lobule and left dorsolateral prefrontal cortex; Structural MRI: superior temporal gyrus. et al. are account for increasing the risk of conversion to full psychosis.

**Discussion:** This is the first, well-implemented, longitudinal study of CHR in a low and middle-income country to comprehensively investigate clinical and biological factors in predicting psychosis conversion and illness progression. Dr. Seidman provide a critical step in the implementation of CHR concept in China, just as an obvious need and urgency for prevention and early intervention for Chinese patients with schizophrenia.

### Plenary

### 22. THE NEAR FUTURE FOR SCHIZOPHRENIA (PSYCHOSIS) RESEARCH

William T. Carpenter, Jr.
*University of Maryland School of Medicine*

**Overall Abstract:** Implications of a heterogeneous clinical syndrome such as schizophrenia have long been known but little attended. Fundamental problems persist, such as schizophrenia as the phenotype in GWAS studies. But the 21st Century has brought substantial attention to limitations in acquisition of new knowledge. New concepts and methods are being implemented. Selected examples will be reviewed and potential scientific advances that influence clinical care will be outlined. This will include advances in mechanism knowledge, identification of novel targets for therapeutic discovery, re-conceptualization of psychopathology for regulatory purposes, a new integration of behavioral and biological science to inform nosology, enhanced testing of neural circuit hypotheses, and serious attention to primary prevention.

### Concurrent Symposia

### 23. FRONTAL CORTEX DEVELOPMENT AND RISK FOR PSYCHOPATHOLOGY: MOLECULAR AND GENETIC MEDIATORS AS POSSIBLE BIOMARKERS?

Francesco Papaleo
*Instituto Italiano Di Tecnologia*

**Overall Abstract:** This panel includes 4 females and 1 male, 2 early career scientists, 2 clinicians. From 5 different countries, 3 different continents.
Prefrontal cortex (PFC) dysfunction is associated with alterations in cognitive processing impaired in schizophrenia. The development of the PFC is a protracted process, which peaks in adolescence and ends only in early adulthood. Its extended development renders the PFC particularly susceptible to environmental influences, but we know very little about the underlying neurobiological mechanisms. More importantly, we need to understand how risk or protective factors can affect PFC development. This could have an impact towards the development of early and/or preventive treatments for cognitive dysfunctions relevant to a number of psychiatric disorders including schizophrenia.
We will discuss recently-discovered processes involved in different stages of prefrontal cortex development, including gestation and adolescence, and how alterations to these events may lead to schizophrenia-relevant phenotypes. A multidisciplinary group of preclinical and human researchers will discuss recently-identified molecular, genetic, and hormonal events that shape PFC development. We will also show compelling new evidence that disruption to these developmental processes is linked to psychiatric conditions. The data we will present include:

(1) Signaling events implicated in the migration of newborn neurons into emerging cortical layers and their relevance to schizophrenia and autism spectrum disorder (Helen Cooper).
(2) Mechanisms related to adolescent axonal growth and connectivity in the PFC and how they are disrupted by ongoing experiences (Cecilia Flores).
(3) Findings from human and mouse studies regarding the impact of the 22q11.2 microdeletion on PFC development and cognitive maturation (Francesco Papaleo).
(4) The molecular development of the postnatal human cortex, including the maturation of interneurons, molecular changes in neurotransmitter signaling pathways, synaptic development and developmental changes in those immune related molecules that may impact synaptic development (Maree Webster).

Collective discussion of these data (Maude Schneider) will highlight important implications for prevention and early intervention strategies in schizophrenia.

### 23.1 UNDERSTANDING THE ROLE OF SCHIZOPHRENIA/AUTISM GENES IN CORTICAL DEVELOPMENT

Helen Cooper*[,1], Amanda White[2], Conor O'Leary[2]
[1]*Queensland Brain Institute, The University of Queensland;*
[2]*Queensland Brain Institute*

**Background:** The fidelity of neocortical development is dependent on the highly polarized morphology of the neuroepithelial stem cell (NSC) within the embryonic brain. NSCs project long processes to the pial surface along which newborn neurons migrate to establish the cortical plate. Perturbation of NSC morphology prevents neuronal migration into the emerging cortical layers, leading to cortical malformations. Disruption of the laminar architecture due to failed neuronal migration is thought to contribute to the etiology of schizophrenia and autism. Therefore, elucidating the signaling events that precisely control NSC morphology is essential to our understanding of corticogenesis and the aberrant processes that contribute to neuropsychiatric disorders.
Maintenance of NSC morphology and function requires the formation of cadherin-based cell-cell adhesion (adherens junctions) between NSCs and loss of junctional integrity results in failed neuronal migration. Junctional stability is critically dependent on the closely apposed actin cytoskeleton and the actin remodeling protein Cyfip1 known to promote actin polymerization. Cyfip1 has been implicated in schizophrenia and autism and its loss results in cortical malformations. However, the molecular mechanisms governing Cyfip1 activity in NSCs are poorly understood.
**Methods:** In this study we investigate the signaling mechanisms that regulate Cyfip1 activity in the developing mouse cortex using both gain- and loss-of-function approaches. Short interfering RNAs or cDNA expression constructs were electroporated, in utero, into the embryonic day 12 mouse cortex. Phenotypic analysis was then performed several days later.
**Results:** Here we identify the netrin/RGM receptor, Neogenin, as a direct binding partner for Cyfip1. We provide evidence that Neogenin is a critical upstream regulator of Cyfip1 activity during corticogenesis and is therefore a key component of NSC junctions. We show that blocking Neogenin/Cyfip1 interactions in the embryonic mouse cortex results in NSC junctional collapse and severe perturbation of the emerging cortical architecture due to aberrant neuronal migration. Our study therefore reveals that Neogenin's interaction with Cyfip1 is essential for NSC morphology and function.
**Discussion:** In conclusion, we have identified a novel signaling pathway that governs the development of the neocortex. The emergence of neuronal migration defects and cortical malformations when Neogenin-Cyfip1 interactions are prevented emphasizes the fundamental role of this interaction in establishing the correct cortical architecture. Intriguingly, mutations in the Neogenin gene have recently been linked to autism. Therefore, our study implicates the Neogenin/Cyfip pathway in the etiology of neuropsychiatric disorders.

### 23.2 NETRIN-1 RECEPTORS CONTROL MESOCORTICAL DOPAMINE CONNECTIVITY IN ADOLESCENCE

Cecillia Flores*[,1]
[1]*McGill University*

**Background:** Adolescence is an age of heightened vulnerability to develop psychiatric disorders that involve alterations in prefrontal cortex circuitry and cognitive dysfunction. The maturation of prefrontal cortex function is linked to the establishment of dopamine connectivity in this region.

Development of mesocortical dopamine is a gradual process that continues until early adulthood. Because of its extended maturational course, this system is particularly susceptible to environmental influences. Yet there is a significant gap in our knowledge about the cellular and molecular mechanisms underlying adolescent prefrontal cortex dopamine development and how they are influenced by experience.
**Methods:** We examined the role of the Netrin-1 guidance cue receptor, DCC, and its microRNA repressor, miR-218, on adolescent mouse prefrontal cortex development. We used axon-initiated recombination and cell-specific knock-down techniques to characterize the spatiotemporal growth of mesocortical dopamine axons and the role that DCC and miR-218 play in this process. Next, we assessed whether stimulant drugs in adolescence alter miR-218/DCC signaling, thereby disrupting mesocortical dopamine axon growth. Finally, we determined whether altered dopamine axon growth influences prefrontal cortex development by quantifying pyramidal neuron morphology and cognitive performance in adulthood.
**Results:** Here we show, for the first time, that dopamine axons continue to grow from the nucleus accumbens to the prefrontal cortex during adolescence. We discovered that DCC receptors control the extent of this protracted growth by determining where and when dopamine axons recognize their innervation target. Exposure to stimulant drugs or to stress leads to disruption of DCC-dependent adolescent targeting events, causing dopamine axons that should innervate the nucleus accumbens, to grow ectopically to the prefrontal cortex. This effect profoundly changes prefrontal cortex structural and functional development, producing alterations in cognitive processes known to be impaired across psychiatric conditions, including schizophrenia. Importantly, miR-218 controls DCC receptor expression in dopamine neurons across postnatal development and acts as a molecular mediator of the effects of stimulant drugs on prefrontal cortex development.
**Discussion:** The prolonged growth of dopamine axons during adolescence represents an extraordinary period for experience to influence their growth and predispose to or protect against psychopathology. MicroRNA control of DCC receptor in dopamine neurons is a molecular link where genetic and environmental factors seem to interact in adolescence to influence the development and function of the prefrontal cortex.

### 23.3 DEVELOPMENTAL TRAJECTORIES OF SCHIZOPHRENIA-RELEVANT ABNORMALITIES IN A MOUSE MODEL OF 22Q11.2 DELETION SYNDROME

Mariasole Ciampoli[1], Marco Armando[2], Francesco Papaleo*,[1]
*[1]Istituto Italiano di Tecnologia; [2]Hospital Bambino Gesu*

**Background:** The hemizygous genetic deletion in the 22q11.2 locus causes a syndrome (22q11DS) characterized by developmental social and intellectual disabilities, high prevalence of attention deficit hyperactivity disorder (ADHD; ≈37%) during childhood and schizophrenia (≈41%) in adulthood. Although this peculiar behavioral alterations, the specific brain and molecular factors influencing these developmental trajectories are still unknown. Preclinical animal studies could help to disentangle these mechanisms. However, no studies in animal models had so far checked the impact of the 22q11.2 microdeletion in behavioral phenotypes from birth to adolescence, to adulthood.
**Methods:** We used LgDel mutant mice that carry the same 1.5 Mb deletion of the human 22q11.2DS. In parallel, we also used patients with 22q11.2DS.
**Results:** We first unraveled in mice altered startle responses at pre-pubertal ages (postnatal day PND 14) that ameliorated in early development from PND 19. Moreover, sensorimotor gating deficits started to appear as early as PND 19 lasting throughout adulthood. Motor coordination assessed with the Rotarod Test, instead revealed in LgDel mice motor deficits in pre-pubertal period (PND 15–16), that disappeared from adolescence (PND 35). Next, in an implemented 5-Choice Serial Reaction Time Task, we found that LgDel adolescent mice showed selective higher distractibility than controls as it has been shown in patients with schizophrenia. All these developmental behavioral alterations were accompanied by selective altered maturation of the prefrontal cortex, as demonstrated by parallel studies in mice and humans.
**Discussion:** Overall, our experiments are starting to elucidate how clinically-relevant genetic alterations can influence the developmental trajectories of behavioral phenotypes through an altered maturation of the prefrontal cortex. This will be important in the context of the development of early diagnosis and preventive intervention.

### 23.4 MAPPING MAJOR MOLECULAR CHANGES IN THE DEVELOPMENT OF THE HUMAN CORTEX

Maree Webster*,[1], Cynthia Shannon Weickert[2]
*[1]Stanley Medical Research Institute; [2]Neuroscience Research Australia: Schizophrenia Research Laboratory*

**Background:** The predominant neurodevelopmental theory of schizophrenia posits that there is a failure of normal synaptic loss believed to occur during normal adolescence. However, the most consistent neuropathology in the cortex of people with schizophrenia is a deficit in the γ–aminobutyric acid (GABA) inhibitory interneurons, not a reduction in presynaptic and postsynaptic elements. Thus, disruption to the normal development of cortical interneurons may lead to interneuron deficiency in schizophrenia. However, to understand if pathological changes in the brain of an adult with schizophrenia would be consistent with aberrant development, the known neuropathology must be placed in the context of normal human cortical development.
**Methods:** We examined the molecular changes that occur in the synapses, interneurons and the neurotransmitter systems during normal development of the human prefrontal cortex of 68 brains from healthy individuals (1 month - 49 years).
**Results:** Contrary to the prevailing view that synaptic pruning predominates during adolescent brain development, we found presynaptic mRNA and protein levels generally peak between 5–12 years of age and then remain stable through adolescence and into adulthood. Likewise, markers for dendritic spines peak in infancy and while mRNA levels then decline, protein levels are maintained throughout development. The various interneuron markers show three very distinct patterns of expression over development. Parvalbumin and cholecystokinin increase from infancy, whereas somatostatin, calretinin and neuropeptide Y decrease from infancy. Calbindin and vasoactive intestinal peptide peak in the toddlers and then decrease in adults in an inverted U shaped-pattern. Levels of mRNA for the GABA synthesizing enzymes GAD65 (GAD2) and GAD67 (GAD1) peak around 1 year of age and stay consistent through to adulthood. The postsynaptic GABAA receptor α1, β2 and γ subunits increase over the postnatal period to peak in adolescent/young adulthood whereas the α2 subunit shows the inverse pattern and decreases over the postnatal period. The dopamine receptor, D1, increase expression throughout the postnatal period to peak in early adulthood, whereas D2 and D5 show a continual decline throughout life. The NMDA receptors are highest in the first year of life and while subunits GRIN 2B, 2D and 3A all decrease throughout life, GRIN1 remains stable and GRIN 2A decreases after childhood. More recently data supporting the neuroinflammatory hypothesis of schizophrenia has merged with the neurodevelopmental hypothesis and posits that the strongest signal from GWAS studies in schizophrenia is in the C4 gene, which is a component of the complement cascade involved in normal synaptic development. We have initiated an examination of the various components of the complement cascade to determine if/when they are expressed in the human cortex and how they could be impacting the developing circuitry. Preliminary data shows C4 mRNA expressed at very low, but constant levels throughout postnatal life. In contrast, C3 mRNA is expressed at higher levels than C4, peaks in infancy and remains stable into adulthood. MAC protein (CD-59) mRNA which protects cells from complement mediated damage, is expressed at very low levels at birth but then increases significantly with age and is highest in adulthood, suggesting that significant changes in complement may occur after brain maturation is complete.
**Discussion:** Together these findings show very dynamic and complex patterns of expression from birth to adulthood, with the most active growth phase and dynamic changes occurring in the early years before adolescence. Thus, an insult during these early years could profoundly affect the developmental trajectory.

### 24. FROM DUSK TILL DAWN: LIFELONG TRAJECTORIES OF COGNITIVE FUNCTIONING IN PSYCHOTIC DISORDERS AND THEIR IMPLICATIONS FOR FUNCTIONAL RECOVERY AND TREATMENT DECISION

Eva Velthorst
*Icahn School of Medicine at Mount Sinai*

**Overall Abstract:** This symposium will draw together state of the art findings on the lifelong cognitive trajectories, on key-predictors of cognitive functioning and the functional consequences of cognitive impairments in schizophrenia and related psychotic disorders from developmental epidemiological, prodromal, and clinical research. Four speakers will take the audience through new findings on the cognitive course of the lifespan, ranging from childhood to old age. Specifically, the talks will address four key-questions:

1) Which areas of cognitive functioning are impaired and when does this impairment start?
2) How well can cognitive functioning predict the development of a psychotic illness, as well as diagnostic and functional outcome?
3) Does cognitive functioning remain stable after illness onset or are psychotic disorders characterized by continuing decline? When does decline occur and is it possible to predict it?
4) And what is the functional sequelae of specific cognitive impairments in older adults with schizophrenia?

Specifically, Dr. Mollon will present new data examining the origin of cognitive impairment across the psychosis spectrum using a population-based cohort followed prospectively from birth. Her findings demonstrate that while individuals with affective psychosis, subthreshold psychotic experiences and even depression experience some degree of cognitive impairment across the first two decades of life, only those who go on to develop non-affective psychosis exhibit large, widespread and increasing deficits.
Most studies of neurocognitive functioning in Clinical High Risk (CHR) cohorts have examined group averages, likely concealing heterogeneous subgroups. The study of Dr. Velthorst therefore used two independent methods to identify neurocognitive subgroups in a large population at Clinical High Risk for developing psychosis. Her findings show that neurocognitive profiles vary substantially in their severity and are associated with diagnostic and functional outcome, underscoring neurocognition as a predictor of illness outcomes.
Dr. Fett will present recent research on cognitive functioning in a large sample of patients at first hospitalization for a psychotic disorder who have been followed 20-years into the illness. Her findings indicate that cognitive functioning in psychotic disorders continues to decline after illness onset, that this decline is not specific to schizophrenia but present across psychotic disorders, and that, relative to never-psychotic individuals, impairments on some key-cognitive domains worsen with age. Decline could not reliably be predicted by key patient characteristics at baseline.
Lastly, Dr. Harvey will share novel data on the course of cognitive functioning in middle aged and older patients with schizophrenia. His findings demonstrate that cognitive impairments are moderated in their impact on everyday outcomes by the presence of severe communication abnormalities. Interestingly, verbal under-productivity and disconnected speech had different functional correlates, with under-productivity impacting clinician rated social outcomes and performance on measures of interpersonal social competence. A lifetime focus on cognition is paramount in order pinpoint critical periods for prevention and intervention. This symposium seeks to present a comprehensive overview of the cognitive landscape of psychotic disorders by integrating findings on predictors and consequences of lifelong cognitive functioning of individuals diagnosed with a psychotic disorder.

### 24.1 NEUROCOGNITIVE DEVELOPMENT FROM INFANCY TO EARLY ADULTHOOD IN THE PSYCHOSIS SPECTRUM

Josephine Mollon*,[1], Anthony David[2], Stanley Zammit[3], Glyn Lewis[4], Abraham Reichenberg[5]
*[1]Yale University; [2]Institute of Psychiatry, King's College London; [3]Cardiff University; [4]University College London; [5]Icahn School of Medicine at Mount Sinai*

**Background:** The majority of patients with psychotic disorders experience severe neuropsychological impairment. The onset and course of this impairment, however, is debated. Moreover, the course of neuropsychological functioning in other psychiatric conditions remains largely unexamined. This study used longitudinal data from infancy to early adulthood to chart the course of general and specific neuropsychological functions in individuals with psychotic disorders, psychotic experiences and depression.
**Methods:** Data were from the Avon Longitudinal Study of Parents and Children (ALSPAC), a prospective cohort study comprising all live births between 1991 and 1992 in Avon, UK. All participants who underwent cognitive testing at 18 months, 4, 8, 15 and 20 years, and psychiatric assessment at age 18 were included. Individuals with non-affective psychotic disorder, affective psychotic disorder, subclinical psychotic experiences and depression were compared to controls on full-scale, verbal and non-verbal IQ, and measures of processing speed, working memory, language, visuospatial ability and attention.
**Results:** Individuals with non-affective psychosis showed continually increasing deficits between infancy (18 months) and adulthood (20 years) in full-scale IQ (effect size of change ($ES\Delta$) $=-1.09$, p=.02), and non-verbal IQ ($ES\Delta=-0.94$, p=.008). The depression group showed a small, increasing deficit in non-verbal IQ ($ES\Delta=-0.29$, p=.04) between infancy and adulthood. Between ages 8 and 20, the non-affective psychosis group exhibited developmental lags (i.e. slower growth) on measures of processing speed, working memory and attention ($ES\Delta=-0.68$, p=.001; $ES\Delta=-0.59$, p=.004; $ES\Delta=-0.44$, p=.001), and large, static deficits on measures of language and visuospatial ability ($ES=-0.87$, p=.005; $ES=-0.90$, p=.001). There was only weak evidence for neuropsychological deficits in individuals with affective psychosis, depression, and subclinical psychotic experiences.
**Discussion:** These findings suggest that the origins of non-affective psychotic disorder involve dynamic neurodevelopmental processes, which effect both verbal and non-verbal abilities throughout the first two decades of life. These neurodevelopmental processes do not manifest in other psychiatric disorders, such as affective psychotic disorder and depression.

### 24.2 NEUROCOGNITIVE PROFILES IN THE PRODROME TO PSYCHOSIS IN NAPLS-1

Eva Velthorst*,[1], Carrie Bearden[2], Eric Meyer[3], Anthony Giuliano[4], Jean Addington[5], Kristin Cadenhead[6], Tyrone Cannon[7], Barbara Cornblatt[8], Thomas Mcglashan[7], Diana Perkins[9], Ming Tsuang[6], Elaine Walker[10], Scott Woods[7], Larry Seidman[11]
*[1]Icahn School of Medicine at Mount Sinai; [2]University of California, Los Angeles; [3]College of Medicine, College Station;*

[4]*Worcester Recovery Center and Hospital;* [5]*University of Calgary;* [6]*University of California, San Diego;* [7]*Yale University;* [8]*The Zucker Hillside Hospital;* [9]*University of North Carolina;* [10]*Emory University;* [11]*BIDMC*

**Background:** The vast majority of studies of neuropsychological (NP) functioning in Clinical High Risk (CHR) cohorts have examined group averages, possibly concealing a range of subgroups ranging from very impaired to high functioning. Our objective was to assess NP profiles and to explore associations with conversion to psychosis, functional and diagnostic outcome.
**Methods:** Data were acquired from 324 participants (mean age 18.4) in the first phase of the North American Prodrome Longitudinal Study (NAPLS-1), a multi-site consortium following individuals for up to 2½ years. We applied Ward's method for hierarchical clustering data to 8 baseline neurocognitive measures, in 166 CHR individuals, 49 non-CHR youth with a family history of psychosis, and 109 healthy controls. We tested whether cluster membership was associated with conversion to psychosis, social and role functioning, and follow-up diagnosis. Analyses were repeated after data were clustered based on independently developed clinical decision rules.
**Results:** Four neurocognitive clusters were identified: Significantly Impaired (n=33); Mildly Impaired (n=82); Normal (n=145) and High (n=64). The Significantly Impaired subgroup demonstrated the largest deviations on processing speed and memory tasks and had a conversion rate of 58%, a 40% chance of developing a schizophrenia spectrum diagnosis (compared to 24.4% in the Mildly Impaired, and 10.3% in the other two groups combined), and significantly worse functioning at baseline and 12-months. Data clustered using clinical decision rules yielded similar results, pointing to high convergent validity.
**Discussion:** Despite extensive neuropsychological investigations within CHR cohorts, this is one of the first studies to investigate NP clustering profiles as a contributor to heterogeneity in outcome. Our results indicate that the four NP profiles vary substantially in their outcome, underscoring the relevance of cognitive functioning in the prediction of illness progression. Our findings tentatively suggest that individualized cognitive profiling should be explored in clinical settings.

### 24.3 EIGHTEEN-YEAR COURSE OF COGNITIVE FUNCTIONING IN PSYCHOTIC DISORDERS: FINDINGS FROM THE SUFFOLK COUNTY MENTAL HEALTH LONGITUDINAL STUDY

Fett Anne-Kathrin*[,1], Eva Velthorst[2], Avi Reichenberg[2], Camilo Ruggero[3], Jennifer Callahan[3], Evelyn J. Bromet[4], Roman Kotov[4]
[1]*City University of London;* [2]*Icahn School of Medicine at Mount Sinai;* [3]*University of North Texas;* [4]*Stony Brook University*

**Background:** Knowledge about the long-term cognitive course in psychotic disorders is limited. In this 18-year follow-up of participants of the Suffolk County project we report on the longitudinal course of cognitive performance in individuals with schizophrenia spectrum disorders, affective psychoses and other psychoses. We investigate (i) change in functioning in 6 cognitive domains from 2-years to 20-years follow-up after first admission; (ii) 20-year performance and age-related differences in cognitive performance in patients relative to a never psychotic comparison group; and (iii) key predictors of clinically meaningful cognitive decline in patients.
**Methods:** Data came from the Suffolk County Mental Health Project, a prospective study of first-admission patients. Cognitive tests were administered 2 years (n = 399; schizophrenia spectrum: 285, affective psychoses: 226, other psychoses: 117) and 20 years (n = 240; 115, 92, and 34, respectively) after first admission, with 195 individuals completing cognitive tests at both time points. A never psychotic comparison group (N=260) was assessed at year 20.
**Results:** Individuals with schizophrenia spectrum disorders showed lower cognitive functioning than those with affective and other psychoses. Over time, patients declined in cognitive performance on almost all tests (d = 0.24 (range 0.12- 0.44)) with comparable magnitude across diagnoses. Longer duration of untreated psychosis and low childhood IQ were significantly associated with clinically relevant decline (>0.5SD) in general verbal ability and processing speed, but there were no robust predictors of cognitive decline across tests. Cross-sectional comparisons of patients to controls showed increasing impairments with age for general verbal ability, verbal fluency, and executive functioning.
**Discussion:** Our findings indicate that cognitive functioning in psychotic disorders continues to decline after the illness onset, that this decline is not specific to schizophrenia but present across psychotic disorders, and that, relative to never-psychotic individuals, impairments on some key-cognitive domains worsen with age. These results imply that cognitive treatment should not only include cognitive remediation but also prevention of age-related cognitive stagnation and/or decline.

### 24.4 COGNITION AND COMMUNICATION AS DETERMINANTS OF ADAPTIVE DEFICITS IN LATE LIFE SCHIZOPHRENIA

Philip Harvey*[,1], Anjana Muralidharan[2]
[1]*Leonard M. Miller School of Medicine, University of Miami;* [2]*Baltimore VA Medical Center*

**Background:** Older adults with schizophrenia experience poor community integration and social functioning. These individuals are at elevated risk for functional decline and early institutionalization in long-term care facilities. Deficits in thought, language, and communication are core features of schizophrenia and may worsen with age; however, little research focuses on the functional sequelae of these impairments among older adults with schizophrenia.
**Methods:** The present study examined the relationships among age, TLC deficits, and functional outcomes in a sample of community-dwelling middle-aged and older adults with schizophrenia (N=245; ages 40–85). Participants completed assessments of symptoms, neurocognition, TLC deficits, and functional outcomes. Two different categories of TLC deficits were examined: verbal underproductivity (i.e., alogia) and disconnected speech.
**Results:** Regression analyses found that disconnected speech predicted impaired occupational functioning, while verbal under productivity predicted capacity to communicate skillfully in semi-structured social situations, as well as community functioning across interpersonal, occupational, and everyday living domains. Exploratory mediation analyses found that cognitive impairments were mediated by disconnected speed but not under productivity on certain functional outcomes.
**Discussion:** Targeted training to improve TLC deficits, especially verbal underproductivity, among older adults with schizophrenia could have downstream effects on community functioning, improving outcomes for a vulnerable group. It is likely that cognitive training interventions would also facilitate these interventions.

### 25. OLIGODENDROCYTE-BASED IMPAIRMENT OF BRAIN CONNECTIVITY AS TARGET FOR NEW TREATMENT STRATEGIES IN SCHIZOPHRENIA

Johann Steiner
*University of Magdeburg*

**Overall Abstract:** This symposium has a translational approach. First, we present human post-mortem and in-vivo imaging studies on the pivotal role of oligedondrocyte loss and dysfunction with consecutive impairments of brain connectivity in schizophrenia. Natalya Uranova will show morphometric data on ultrastructural alterations of oligodendrocytes, myelin damage and degeneration and disturbed oligodendrocyte-axon interactions in post-mortem prefrontal white matter in schizophrenia. Adrienne Lahti will report diffusion tensor imaging data suggesting impaired axonal and myelin integrity. Because, MR Spectroscopy permits the non-invasive measurement of neurometabolites, such as N-acetylaspartate, a marker of neuronal integrity, and glutamate, which can be neurotoxic when overproduced, this technique provides further understanding of the relationship between white matter microstructure and neuronal function.
Second, we present data from cell culture and animal models suggesting that restoration of oligodendrocyte function (in terms of energy metabolism, maturation and myelin production) is a promising target for the development of novel treatment strategies in schizophrenia. Proteomic studies in postmortem brain by Daniel Martins-de-Souza have suggested a schizophrenia-related energy metabolism dysfunction in oligodendrocytes. These findings have been followed up using oligodendroglia cell lines and induced pluripotent stem cell-derived cerebral organoids, supporting the notion that alterations in glycolysis in oligodendrocytes are pivotal to the overall energy dysfunction in schizophrenia brains. Lan Xiao´s lab has shown that oligodendrocyte dysfunction and impaired myelination in the prefrontal cortex is correlated with schizophrenia-like behavior in mice undergoing prolonged social isolation. Enhancing oligodendrocyte generation and myelin repair by FDA-approved compounds, like quetiapine (an APD) or clemastine (a histamine antagonist) successfully reversed the above phenotype.

### 25.1 OLIGODENDROCYTE PATHOLOGY IN PREFRONTAL WHITE MATTER IN SCHIZOPHRENIA

Natalya Uranova*[,1], Olga Vikhreva[1], Valentina Rakhmanova[1], Diana Orlovskaya[1]
*[1]Mental Health Research Center*

**Background:** Recent neuroimaging studies have shown altered brain connectivity in patients with schizophrenia, associated with disturbed myelination in different fiber tracts and disruptions of white matter (WM) integrity, including prefrontal WM. We aimed to perform a qualitative and morphometric study of the ultrastructure of oligodendrocytes, myelin-forming cells, in prefrontal WM in schizophrenia and normal controls.
**Methods:** WM of the prefrontal cortex (Brodmann's area 10) was studied by transmission electron microscopy and morphometry. Size, volume density (Vv) and the number (N) of organelles in oligodendrocytes were estimated in 21 patients with schizophrenia and 20 normal matched controls. Pearson correlation analysis was performed to assess possible correlations between the parameters measured and age, post-mortem interval, neuroleptic treatment and duration of the disease. ANCOVA tests were used for group comparisons.
**Results:** Qualitative study showed swelling, vacuolation, paucity of ribosomes and mitochondria and accumulation of lipofuscin granules in oligodendrocytes in schizophrenia as compared to controls. Morphometry detected lowered Vv and N of mitochondria and higher Vv and N of lipofuscin granules and vacuoles in oligodendrocytes in the schizophrenic group as compared to the control group (all $p<0.01$).
**Discussion:** Altered metabolism of oligodendrocytes, previously reported reduced number of oligodendrocytes, disrupted myelin/axon integrity, damage and progressive degeneration of myelin sheaths in prefrontal WM in schizophrenia may lead to disturbances in myelination, deficiency of nerve impulses propagation and contribute to network dysfunctions in schizophrenia. Oligodendrocyte and myelin abnormalities may be a target to prevent or restore WM abnormalities and dysfunction of neuronal connectivity in schizophrenia.

### 25.2 UNDERSTANDING WHITE MATTER PATHOLOGY IN SCHIZOPHRENIA USING DIFFUSION TENSOR IMAGING AND MAGNETIC RESONANCE SPECTROSCOPY

Adrienne Lahti*[,1], Meredith Reid[2], David White[1], Nina Kraguljac[1]
*[1]The University of Alabama at Birmingham; [2]Auburn University*

**Background:** Diffusion tensor imaging (DTI) studies in schizophrenia consistently show global reductions in fractional anisotropy (FA), a putative marker of white matter integrity. Because magnetic resonance spectroscopy (MRS) studies permit for the non-invasive measurements of neurometabolites, such as N-acetylaspartate (NAA), a marker of neuronal integrity, and glutamate, which can be neurotoxic when over-released, this technique provides further understanding of the relationship between white matter microstructure and neuronal function.
**Methods:** Twenty-nine schizophrenia patients and twenty controls participated in this 3T imaging study where we used DTI and tract-based spatial statistics (TBSS) to assess white matter integrity of the cingulum bundle and MRS to quantify NAA and glutamate in the anterior cingulate cortex (ACC) and hippocampus, i.e. in cortico-limbic regions connected by the cingulum bundle.
**Results:** We found FA reductions with overlapping radial diffusivity (RD) elevations in patients in multiple tracts, suggesting white matter abnormalities in schizophrenia are driven by loss of myelin integrity. In controls, but not in patients, high hippocampal NAA levels were significantly associated with low RD in the hippocampal part of the cingulum, and low ACC glutamate levels were significantly associated with high FA in the hippocampus part of the cingulum.
**Discussion:** In conclusion, we demonstrate the potential utility of a multimodal neuroimaging approach to help further our understanding of the relationship between white matter microstructure and neurochemistry in distinct cortical regions connected by white matter tracts.

### 25.3 OLIGODENDROCYTES MEDIATE ENERGY METABOLISM ALTERATIONS IN SCHIZOPHRENIA: A PROTEOMIC STUDY

Daniel Martins-De-Souza*[,1]
*[1]University of Campinas (UNICAMP)*

**Background:** While comparing the proteomes and subproteomes of 8 postmortem brain regions and cerebrospinal fluid from schizophrenia patients to controls, we consistently observed alterations in energy metabolism, cell growth and maintenance, synaptic function, and myelinization processes. Considering the nature of these analyses, it was not possible to reveal which particular cell types display such alterations. This is essential information given increasing evidence of glia cells as pivotal players in schizophrenia. With this in mind, we analyzed the proteomes and phosphoproteomes of cultured astrocytes, oligodendrocytes and neurons treated with MK-801, a NMDA-receptor antagonist which impairs glutamatergic transmission as postulated in schizophrenia. We also analyzed biochemical pathways modulated by typical and atypical antipsychotics in human oligodendrocytes. Results led us to employ induced pluripotent stem cell-derived cerebral organoids to deepen our understanding of the data. The central aim of this study is to depict which cell type(s) present proteome changes similarly to those we found in our earlier analysis of human brain tissue as well as identify key pathways for an effective antipsychotic response.
**Methods:** Cell line cultures (astrocytes, oligodendrocytes and neurons) were treated with MK-801 and oligodendrocytes were also treated with a range of typical and atypical antipsychotics. In addition, human embryonic stem cells reprogramed from schizophrenia patients and controls fibroblasts were cultured in mTeSR1 media on Matrigel coated surface and then differentiated into cerebral organoids. All pre-clinical models here employed were submitted to state-of-the art large-scale proteomic analyses. In silico systems biology was employed to identify key pathways in the studied processes.
**Results:** MK-801-treated astrocytes, and especially MK-801-treated oligodendrocytes displayed several proteins differentially expressed which overlapped with previous findings of schizophrenia human brains. On the other hand, MK801-treated neurons displayed very few differences in their proteome, an overlap with previous findings in human brain tissue below 10%. More interestingly, the dysregulation of glycolytic enzymes in MK801-treated oligodendrocytes are very similar to our observations in schizophrenia brain tissue, corroborating with recent findings about of the importance of oligodendrocytes in the energy status of the brain. In oligodendrocytes, antipsychotics displayed differences in translational machinery and eIF2 signaling. Findings on cerebral organoids also showed overlaps with previous postmortem data, mainly on synaptic proteins and specially energy metabolism-associated pathways.
**Discussion:** These findings hold potential for the investigation of developmental and evolutionary features of schizophrenia brains and provides targets to be drug-screened as well as leads to the schizophrenia pathobiology.

### 25.4 PROMOTING MYELIN REPAIR RESCUES MICE FROM SCHIZOPHRENIA-LIKE BEHAVIOR INDUCED BY SOCIAL ISOLATION

Lan Xiao*,[1]
*[1]Third Military Medical University*

**Background:** Although pathological and genetic evidence suggest that oligodendrocyte (OL) or myelin deficits are associated with schizophrenia, the contribution of OL/myelin deficits to its etiology has not been clearly dissected, because OL/myelin abnormalities may be a concomitant phenomenon during the pathogenesis of schizophrenia.
**Methods:** Using olig2 ablation specifically in OLs (olig2 CKO) mice, we detected myelin development status and animal behaviors under normal condition or subjected to social isolation. We also examined the therapeutic effect of FDA-approved compounds, like quetiapine (an APD) or clemastine (a histamine antagonist) on animal behaviors.
**Results:** Our results demonstrated that deleting of olig2 leaded to impaired development of OLs and myelin deficit from postnatal day14 (P14) to P56, preferentially in cerebral cortex, and these young adult Olig2 KO mice showed anxiety-like behavior, motor skill learning deficit and cognitive deficit. Moreover, Olig2 CKO mice exhibited earlier social avoidance behavior than the WT littermates under prolonged social isolation, indicating that myelin deficit may enhance risk of schizophrenia upon environmental stress attacking. Interestingly, enhancing oligodendrocyte generation and myelin repair by quetiapine or clemastine successfully reversed the above phenotype.
**Discussion:** Taking together, promoting myelin repair may present a new therapeutic strategy against schizophrenia.

### 26. NOVEL APPROACHES TO PSYCHOSIS RISK: MOVEMENT, STRESS MODULATION, REWARD AND LANGUAGE

Cheryl Corcoran
*Icahn School of Medicine at Mount Sinai*

**Overall Abstract:** Research on psychosis risk now encompasses novel and innovative approaches for understanding not only positive symptoms, but also impairment in sensorimotor function, stress regulation, reward learning, and language. These include the use of machine learning and cluster analysis with resting state functional connectivity analyses, in vivo measures of dopamine function in response to stress, computational modeling, and automated natural language processing analyses in collaboration with IBM.
First, Vijay Mittal will describe subtypes of clinical risk, identifying a group with aggregated measures of sensorimotor dysfunction, developmental markers, negative symptoms and cognitive deficits, who have a discrete pattern of corticostriatal connectivity.
Second, Romina Mizrahi will present her results from a study of dopamine response to stress in prefrontal cortex, using positron emission tomography, and correlations with cortisol release, across stages of illness, including schizophrenia and clinical risk, with healthy volunteers for comparison.
Third, James Waltz will present data on the computational processes that may underlie both positive and negative symptoms, in respect to dopamine-based signals of salience. These include aberrant or erratic salience signaling, as well as a decreased ability to identify relevant salient stimuli, which could impair reward learning and motivation. His cohort includes individuals with psychosis, and those at clinical risk for it, as well as non-psychosis patient controls.
Fourth, Cheryl Corcoran will describe the use of automated natural language processing (NLP), with machine learning (ML) to identify semantic and syntactic features that predict psychosis onset. She will show data on cross-validation of the classifier in a second risk cohort, and its correlation with demographics and manual linguistic features. Overall, there is an apparent norm of semantic coherence and syntactic complexity from which individuals with psychosis deviate, even prior to its onset.
Finally, the discussant will review these data in the context of his experience and ongoing leadership in the field of psychosis risk research, leading audience discussion, and outlining a roadmap for future research in the field.

### 26.1 MOTOR SUBTYPES AND PREDICTION OF COURSE IN PSYCHOSIS RISK YOUTH

Vijay Mittal*,[1], Derek Dean[2], Sebastian Walther[3], Tina Gupta[1], Teresa Vargas[1], Juston Osborn[1]
*[1]Northwestern University; [2]University of Colorado Boulder; [3]University Hospital of Psychiatry*

**Background:** Prominent etiological conceptions of psychosis implicate abnormal cortico-striatal circuits. Dysfunction in these critical systems, responsible for filtering information and modulating higher-order function, may account for heterogeneous presentations of symptoms and characteristics of psychosis. Collectively, a body of work from our group and from other teams indicates that evaluating select motor behaviors and abnormalities, which directly reflect function of these circuits, may be a useful method for understanding and predicting the neural underpinnings of psychosis. In the context of the psychosis risk period, partitioning clinical high-risk (CHR) youth based on objective behavior may help guide early detection and intervention efforts, and provide a novel perspective on different etiological pathways or patient subtypes.
**Methods:** Using an unsupervised machine learning approach, 69 CHR young adults were included in a K-means cluster analysis based on their performance on instrumental measures of psychomotor slowing, dyskinesia, and neurological soft signs (NSS)—distinct motor domains affected across the psychosis spectrum. We also recruited a group of 70 matched healthy controls (HC) for comparison. All participants were also assessed with a resting-state functional connectivity analysis (rcfMRI). The resulting CHR group clusters and HCs were then compared on positive and negative symptoms, multiple cognitive domains, and cortical-striatal seed based resting state analysis.
**Results:** Results of a 3-cluster solution suggest that there are subtypes of CHR individuals who show psychomotor slowing, average motor performance, and impairment on measures of dyskinesia as well as NSS domains for motor coordination, sequencing and sensory integration. The cluster of individuals showing dyskinesia and abnormal NSS also have more severe negative symptoms and impairment on a number of cognitive domains. Furthermore, the clusters of CHR individuals who show psychomotor slowing and the cluster showing dyskinesia and abnormal NSS have different cortical-striatal connectivity compared to UHR who show average motor behavior and healthy controls.
**Discussion:** These results provide evidence for etiological theories highlighting altered cortico-striatal networks and the importance of examining motor behavior prior to the onset of psychosis. Taken together, this approach may reflect a novel strategy for promoting tailored risk assessment as well as future research developing individualized medicine.

### 26.2 CORTICAL STRESS REGULATION IS DISRUPTED IN SCHIZOPHRENIA BUT NOT IN CLINICAL HIGH-RISK FOR PSYCHOSIS

Romina Mizrahi*[,1], Alan Wilson[2], Sylvain Houle[3], Christin Schifani[2]
*[1]University of Toronto & Centre for Addiction and Mental Health; [2]PET Centre, Centre for Addiction and Mental Health; [3]University of Toronto*

**Background:** While striatal dopamine in psychosis and stress has been well studied, the role of dopamine in the prefrontal cortex (PFC) is poorly understood. To date no study has investigated the PFC dopamine response to stress exclusively in schizophrenia or its putative prodrome, even though medial PFC is known as a key area in stress regulation. The present study uses the high-affinity dopamine D2/3 receptor radiotracer [11C]FLB457 and positron emission tomography (PET) together with a validated psychosocial stress challenge to investigate if the PFC dopamine response to stress is dysregulated in schizophrenia and clinical high risk (CHR) for psychosis.
**Methods:** Fourteen antipsychotic-free patients with schizophrenia, 14 CHR and 12 matched healthy volunteers underwent two [11C]FLB457 PET scans, one while performing a Sensory Motor Control Task (control) and another while performing the Montreal Imaging Stress Task (stress). PET data were analyzed using the Simplified Reference Tissue Model with non-displaceable binding potential (BPND) as outcome measure. Dopamine release was defined as percent change in BPND between control and stress scan (ΔBPND).
**Results:** We observed an increased dopamine release, indexed by ΔBPND, in the medial PFC in schizophrenia patients but not CHR compared to healthy volunteers. Further, associations between stress-induced dopamine release and increase in cortisol levels observed in healthy volunteers and CHR, were absent in schizophrenia, similar to associations with symptoms, distress and anxiety.
**Discussion:** These findings provide first direct evidence of a disrupted cortical dopamine-stress regulation in schizophrenia.

### 26.3 SALIENCE SIGNALING AND THE EMERGENCE OF PSYCHOPATHOLOGY IN YOUTH AT CLINICAL HIGH RISK FOR PSYCHOTIC ILLNESS

James Waltz*[,1], Caroline Demro[2], Zachary Millman[2], Gloria Reeves[3], Jonathan Roiser[4], James Gold[1], Jason Schiffman[2]
*[1]Maryland Psychiatric Research Center; [2]University of Maryland, Baltimore County; [3]University of Maryland School of Medicine; [4]UCL Institute of Cognitive Neuroscience*

**Background:** The early identification of people who appear to be at high risk for conversion to psychosis has become a central thrust of mental health research, with the hope that early intervention may alter the course of psychotic illness. Importantly, both positive and negative symptom dimensions have been found relate to risk for conversion in clinical high risk (CHR) populations. Neuroimaging work points to a role for dopamine pathway activity in both the positive and negative symptoms of psychotic illness. A role for dopamine pathways in signaling various kinds of salience is well-established, and several authors have proposed that excessive dopamine transmission in the striatum might contribute to psychotic symptoms by bringing about erratic, or "aberrant", salience signaling. By contrast, a reduced ability to identify salient events as such, and signal salience "adaptively", could result in impairments in learning and motivation. I will describe results from a study in which we examined the impact of salient events on learning and behavior in adolescents and young adults, a subset of whom were identified as being at CHR for developing psychotic illness.
**Methods:** Participants were 98 adolescents and young adults (mean age = 16.1 ± 3.3 years), assessed clinically using the Structured Interview for Psychosis-Risk Syndromes (SIPS). Eighty-nine participants were receiving mental health services, with 30 of the 89 identified as CHR and 8 identified as already having a psychotic illness. We used two experimental paradigms to investigate the impact of salient events on learning and behavior: the probabilistic stimulus selection task (PSST; Frank et al., 2004) and the Salience Attribution Task (SAT; Roiser et al., 2009). Both adaptive and aberrant salience signals were operationalized in the context of each task. Successful performance of the PSST depends on the adaptive signaling of mismatches between expected and obtained outcomes, called reward prediction errors, which are one form of salient event. The SAT requires participants to respond as quickly as possible to a response prompt, which is preceded by conditioned stimuli that potentially predict reward availability for a fast response. The comparison of reaction time (RT) between responses following the frequently vs. infrequently rewarded conditioned stimuli offers a measure of adaptive salience coding with the expectation of faster RT for reward predicting stimuli. The comparison of RT between responses to the two levels of the irrelevant dimension offers a measure of aberrant salience coding with the expectation of equal RT for stimuli equally-predictive of reward. We assessed whether experimental measures of both adaptive and aberrant salience showed correspondences with SIPS ratings for symptoms along both the positive and negative dimensions.
**Results:** We observed significant correlations between multiple performance measures from the PSST and measures of both positive and negative symptoms. We found that positive symptom severity, in help-seeking youth, correlated positively with an implicit measure of aberrant salience from the SAT, and negatively with an explicit measure of adaptive salience.
**Discussion:** These results, consistent with our previous findings in both first-episode psychosis patients and patients with chronic schizophrenia, suggest that experimental measures of salience signaling may provide a psychosis risk signal in treatment-seeking youth. Further research is necessary to understand the potential predictive role of these measures for conversion to psychosis.

### 26.4 LANGUAGE DISTURBANCE AS A PREDICTOR OF PSYCHOSIS ONSET IN YOUTH AT ENHANCED CLINICAL RISK

Cheryl Corcoran*[,1], Facundo Carrillo[2], Diego Fernández Slezak[2], Casimir Klim[3], Gillinder Bedi[4], Daniel Javitt[5], Carrie Bearden[6], Guillermo Cecchi[7]
*[1]Icahn School of Medicine at Mount Sinai; [2]Universidad de Buenos Aires; [3]University of Michigan Medical School; [4]NYSPI, Columbia University; [5]Columbia University; [6]University of California, Los Angeles; [7]IBM TJ Watson Research Center*

**Background:** Language offers a privileged view into the mind; it is the basis by which we infer others' thoughts. Subtle language disturbance is evident in schizophrenia prior to psychosis onset, including decreases in coherence and complexity, as measured using clinical ratings in familial and clinical high-risk (CHR) cohorts. Bearden et al previously used manual linguistic analysis of baseline speech transcripts in CHR to show that illogical and referential thinking, and poverty of content, predict later psychosis onset.

Then, Bedi et al used automated natural language processing (NLP) of CHR transcripts to show that decreased semantic coherence and reduction in syntactic complexity predicted psychosis onset. To determine validity and reproducibility, we have applied automated NLP methods, with machine learning, to Bearden's original CHR transcripts to identify a language profile predictive of psychosis.

**Methods:** Participants in the Bearden UCLA cohort include 59 CHR, of whom 19 developed psychosis (CHR+) within 2 years, whereas 40 did not (CHR-), as well as 16 recent-onset psychosis and 21 healthy individuals, similar in demographics; speech was elicited using Caplan's "Story Game. Participants in the Bedi NYC cohort include 34 CHR (29 CHR+), with speech elicited using open-ended interview. Speech was audiotaped, transcribed, de-identified and then subjected to latent semantic analysis to determine coherence and part-of-speech tagging to characterize syntactic structure and complexity. A machine-learning speech classifier of psychosis onset was derived from the UCLA CHR cohort, and then applied both to the NYC CHR cohort and to the UCLA psychosis/control comparison, with convex hull (three-dimension depiction of model) and receiver operating characteristics analyses. Correlational analyses with demographics, symptoms and manual linguistic features were also done.

**Results:** A four-factor model language classifier derived from the UCLA CHR cohort that comprised three semantic coherence variables and one syntax (usage of possessive pronouns) predicted psychosis t with accuracy of 83% (intra-protocol) for UCLA CHR, 79% (cross-protocol) for NYC CHR, and 72% for discriminating psychosis from normal speech (UCLA psychosis/control). Convex hulls were defined as the smallest space containing all datapoints within a set for CHR- or healthy controls: these convex hulls showed substantial overlap, with CHR+ and psychosis speech datapoints largely outside these convex hulls. Coherence was associated with age, but speech variables did not vary by gender, race, or socioeconomic status in this study. While automated text features were unrelated to prodromal symptom severity, they were highly correlated with manual text features ($r = 0.7$, $p < .000001$).

**Discussion:** In this small preliminary study, we identified and cross-validated a robust language classifier of psychosis risk that comprised measures of semantic coherence (flow of meaning in language) and syntactic usage (usage of possessive pronouns). This classifier had utility in discriminating speech in individuals with recent-onset psychosis from the norm. It demonstrated concurrent validity in that it was highly correlated with manual linguistic features previously identified by Bearden et al, important as automated methods are fast and inexpensive. Automated language features were unrelated to sex, ethnicity or social class in these small samples, and semantic coherence increased with age, consistent with prior studies of normal language development. Of interest, overlapping convex hulls could be defined for groups of individuals without psychosis (UCLA CHR-, NYC CHR- and UCLA healthy), suggesting a constrained hull of normal language in respect to syntax and semantics, from which pre-psychosis and psychosis speech deviates. The RDoC linguistic corpus-based variables of semantic coherence and syntactic structure hold promise as biomarkers of psychosis risk and expression, with initial validation and reproducibility. Next steps in biomarker development include larger multisite studies with standardization of protocols for speech elicitation, test-retest, and attention to traction/feasibility, acceptability, cost, and utility. Mechanistic studies can also yield neural and physiological correlates of abnormal semantic coherence and syntax.

### 27. THE ROLE OF DOPAMINE IN SHAPING CIRCUITRY RELATED TO SCHIZOPHRENIA AND ADDICTION

Anissa Abi-Dargham
*Stony Brook University School of Medicine*

**Overall Abstract:** Dopamine plays a central role in shaping circuitry within the brain, thus affecting learning and behavior. It also plays a central role in schizophrenia and addiction. This panel will examine the impact of dopaminergic signaling on specific circuits that may create special vulnerability for the emergence of comorbidity between schizophrenia and addiction. The talks will include two presentations in clinical samples using molecular and functional imaging and two presentations in animal models.

Jared Van Snellenberg will discuss connectivity of striatal substructures to the rest of the brain in drug free patients with schizophrenia and their relationship to abnormal cortical D2 signaling and psychotic symptoms. He will present recent unpublished work, motivated by pre-clinical studies with a D2 receptor over-expressing mouse model, using simultaneous multi-slice (multiband) functional MR imaging in these patients. These results suggest that unmedicated patients have altered connectivity between specific basal ganglia subnuclei, consistent with the animal model.

Nora Volkow will focus on the role of bidirectional interactions between dopamine reward system and prefrontal regions in the addicted brain with emphasis on the role of D2 receptor signaling in the striatum. She will discuss the functional impact of these interactions on reward/motivation and executive-function networks and will discuss the variables that influence D2 receptor function including genes, sleep and social stressors and how they interact with drug exposures to provide resilience or vulnerability to substance use disorders or schizophrenia. Finally, she will discuss how this knowledge can be used to tailor interventions to remediate or buffer neurocircuity dysfunction triggered by drugs and for prevention.

Bita Moghaddam will present an animal model of behaviorally induced dysfunction in the mesocortical circuit by using a task where actions are consistently rewarded but probabilistically punished. Spike activity and local field potentials are recorded during this task simultaneously from VTA and mPFC, two reciprocally connected mesocortical regions. Under no risk of punishment, a synchronous interaction at multiple time scales between PFC and VTA dopamine neurons is observed. This synchrony collapsed as a function of punishment contingency during reward-seeking actions, with risk of punishment diminishing VTA-driven neural synchrony between the two regions. These data reveal a dynamic coding scheme in VTA-mPFC neural circuits in representing aversion-based modulation of rewarded actions. These data suggest that driving VTA dopamine neurons by drugs of abuse may reverse the diminished synchrony and serve as self-medication in comorbid conditions.

Finally, Aurelio Galli will discuss the structural, functional, and behavioral insights into the dopamine dysfunction of comorbid conditions as modeled by a deletion of the SLC6A3 affecting the function of the human dopamine transporter (hDAT). Genetic variants in hDAT have been associated with neuropsychiatric disorders. An in-frame deletion in hDAT at N336 (ΔN336) leads to abnormal DA homeostasis. He demonstrated these dysfunctions in brains of Drosophila melanogaster expressing hDAT ΔN336. Furthermore, these flies are hyperactive and display fear and impaired social interactions, traits associated with impaired DA neurotransmission. Insights from X-ray crystallography, electron paramagnetic resonance, molecular dynamic simulations, electrophysiology and behaviors describe how a genetic variation causes DA dysfunction resulting in combined behavioral alterations and psychostimulant use.

### 27.1 TRANSLATIONAL EVIDENCE OF DOPAMINE-RELATED ALTERATIONS OF BASAL GANGLIA AND THALAMO-CORTICAL NEUROCIRCUITRY IN SCHIZOPHRENIA: A FULL CLINIC-TO-BENCH-TO-CLINIC BACK-TRANSLATION

Jared Van Snellenberg*[,1], Guillermo Horga[2], Roberto Gil[1], Christoph Kellendonk[2], Anissa Abi-Dargham[1]
*[1]Stony Brook University Medical School; [2]Columbia University/ New York State Psychiatric Institute*

**Background:** Recent work with a dopamine 2 receptor (D2R) over-expressing (D2R-OE) mouse has suggested that this receptor over-expression leads to a highly plastic increase in bridging collaterals from the associative striatum (AST) to the external segment of the globus pallidus (GPe). Because of the densely interconnected nature of basal ganglia-thalamo-cortical signaling circuitry, we hypothesized and demonstrated in a recent publication that the resting state functional connectivity (RSFC) of AST to multiple cortical and thalamic subregions is broadly disrupted in unmedicated patients with schizophrenia. In this talk, I will present novel simultaneous multi-slice (aka "multiband") fMRI data that provides the spatial resolution necessary to image smaller basal ganglia substructures (such as the GPe/GPi), and show that unmedicated patients with schizophrenia exhibit specifically disrupted AST-GPe connectivity, as predicted directly from the D2R-OE mouse model findings.
In addition, recent work with a 22q11 deletion mouse, which models a similar syndrome in humans that is strongly associated with schizophrenia, has shown that these mice exhibit a D2R-mediated reduction in the strength of excitatory post-synaptic potentials in primary auditory cortex in response to stimulation of the medial geniculate nucleus (MGN) of the thalamus. Consistent with this finding, I will present multiband fMRI data that employs both RSFC and an audio-visual localizer task to demonstrate a specific reduction in RSFC between the MGN and primary auditory cortex, consistent with these findings in the 22q11 mouse.
**Methods:** Partially-overlapping samples of 19 and 14 unmedicated patients with schizophrenia and 15 and 16 matched healthy participants participated in two sets of studies. For both studies, multiband fMRI images were acquired on a GE MR 750 system at the New York State Psychiatric Institute, with a multiband acceleration factor of 6, 2 mm isotropic voxel resolution, and 850 ms TR. Thirty minutes of RSFC data was collected in each participant, and participants in the auditory study also completed a 15 minute audio-visual localizer task that employed sparse temporal sampling with either auditory (9 seconds of a randomized and rapidly-varying musical stimulus) or visual (7.5 Hz alternating checkerboard) stimulation between each acquisition cluster. Basal ganglia subregions were identified via manual drawings conducted by an experienced rater, and the MGN and LGN were identified using the audio-visual localizer task.
**Results:** Unmedicated patients with schizophrenia showed a significant reduction in RSFC strength between the dorsal caudate and GPe (Cohen's $d = 0.87$, $P = 0.017$), but no other striatal or pallidal subregion pairs, consistent with a specific alteration in anatomical projections between these two regions. In addition, patients with schizophrenia showed a reduction in RSFC between the MGN and primary auditory cortex, as well as between the LGN and primary visual cortex ($P < 0.05$, alphasim corrected for whole-brain analysis).
**Discussion:** These findings provide initial support for the existence of D2R-mediated alterations in functional neuroanatomy, first observed in animal models of schizophrenia, in a clinical sample of unmedicated patients. In addition to providing early evidence for potential mechanisms of psychotic phenomena, this work suggests that the use of non-invasive multiband RSFC is a promising approach to translating basic neuroscience findings in animal models back into a clinical setting. Altered circuitry was shown in the D2OE mice to underlie motivational deficits, and we propose that they may have a similar functional impact in patients.

### 27.2 THE DOPAMINE MOTIVE SYSTEM IN ADDICTION

Nora Volkow*[,1]
[1]*DHHS/National Institute on Drug Abuse*

**Background:** We have investigated the role of bidirectional interactions between the dopamine reward and motivation system and executive function in addicted individuals, with a particular focus on the intersection between the role of D2 receptor (D2R) signaling in the striatum and perturbations in prefrontal brain activity.
**Methods:** Using brain imaging we have studied these interaction for various types of addiction and explored how their involvement affect behavior including impulsivity and compulsiveness. We have also investigated the mechanisms associated with vulnerability to drug use disorders as linked with disrupted executive function including the effects of genetics and physiological factors such as circadian rhythms, sleep deprivation and obesity.
**Results:** We found that: a) chronic drug use reduces striatal levels of D2R and perturbs metabolism in frontal brain regions, emotional reactivity and executive control; b) that higher-than-normal striatal D2R availability in nonalcoholic members of alcoholic families appear to play a protective role against alcoholism by regulating circuits involved in inhibiting behavioral responses and in controlling emotions; c) that chronic sleep deprivation is associated with increased striatal dopamine, lower D2R availability, and metabolic changes in several cortical brain regions; and, d) that newly characterized variable number tandem repeat (VNTR) polymorphisms in the genes coding for PER2 and the AKT1 proteins (a kinase that has been implicated in schizophrenia and psychosis) appear to modulate striatal D2R availability in the human brain.
**Discussion:** Although the studies have focused on the effects of drugs, the DA striato cortical pathway is of direct relevance to schizophrenia as well as that of other psychiatric disorders. We will discuss the implications of our findings as they relate to the prevention and treatment of substance use disorders and schizophrenia.

### 27.3 DISCRETE AND COORDINATED ENCODING OF REWARDED ACTIONS BY PREFRONTAL CORTEX AND DOPAMINE NEURONS

Bita Moghaddam*[,1]
[1]*Oregon Health & Science University*

**Background:** Co-morbidity of schizophrenia and drug use has been attribute to common pathophysiology of mesocortical circuit. We modeled a behavioral disruption to this circuit in rodents by using a task where actions were consistently rewarded but probabilistically punished. Our data reveal dynamic coding schemes of the VTA-mPFC neural circuit in representing risk of punishment and punishment-based modulation of rewarded actions.
**Methods:** Spike activity and local field potentials were recorded during simultaneously from ventral tegmental area and medial prefrontal cortex (PFC), two reciprocally connected mesocortical regions, in rodents as they performed a task where actions were consistently rewarded but probabilistically punished. This model allowed us to reveal dynamic coding schemes of the VTA-mPFC neural circuit in representing risk of punishment and punishment-based modulation of rewarded actions.
**Results:** At the single unit level (n=167 mPFC n=102 VTA units), we found that ensembles of VTA and mPFC neurons encode the contingency between action and punishment. At the network level, we found that coherent theta oscillations synchronize the VTA and mPFC in a bottom-up direction, effectively phase-modulating the neuronal spike activity in the two regions during punishment-free actions. This synchrony declined as a function of punishment contingency
**Discussion:** During reward-seeking actions, risk of an aversive outcome and anxiety disrupts dopamine neuron-driven synchrony between PFC and VTA

### 27.4 STRUCTURAL, FUNCTIONAL, AND BEHAVIORAL INSIGHTS OF DOPAMINE DYSFUNCTION REVEALED BY A DELETION IN SLC6A3

Aurelio Galli*[,1]
[1]*Vanderbilt University*

**Background:** The human dopamine (DA) transporter (hDAT) mediates clearance of DA. Genetic variants in hDAT are associated to neuropsychiatric disorders. We investigated the structural and behavioral bases of an in-frame deletion in hDAT at N336 (ΔN336) associated with neuropsychiatric disorders.
**Methods:** This study bridges structural biology, molecular neuroscience and organism physiology culminating in a mechanistic model that relates precise alteration in a transport cycle with behavioral manifestations.
**Results:** We uncovered a previously unobserved conformation of the intracellular gate of the transporter promoted by ΔN336, representing likely the rate limiting step of the transport process. This state is defined by a "half-open and inward facing" state (HOIF) of the intracellular gate that leads to DA dysfunction. The HOIF state is regulated by a network of interactions conserved phylogenetically, as we observed it both in hDAT and in its bacterial homolog leucine transporter. We demonstrated these dysfunctions in brains of Drosophila melanogaster expressing hDAT ΔN336. These flies are hyperactive and display increased fear and impaired social interactions, traits associated with neuropsychiatric disorders.
**Discussion:** Here, we describe how a genetic variation causes DA dysfunction. In this study different techniques and discoveries came together to detail in a translational effort how rare variants in plasma membrane proteins affect complex behavior.

### 28. BEYOND VOICES: MULTISENSORY BODILY SELF DISTURBANCES ACROSS THE SCHIZOPHRENIA SPECTRUM

Sohee Park
*Vanderbilt University*

**Overall Abstract:** Aberrant bodily self experiences are highly salient and disruptive to individuals with schizophrenia. Given that these self disturbances are already present at prodromal stage, persist throughout the course of illness, and impact functional outcome, they should be precisely targeted for intervention. However, in contrast to the prominence of auditory hallucination in schizophrenia research, bodily self disturbances have been largely neglected. This symposium aims to bridge this gap with new theoretical and experimental advances that allow us to capture the full extent of the phenomenology, and at the same time, mechanistically specify the etiology and nature of self disorders with an eye toward implementing new treatments.
Nelson & Sass will present their revised theory of self disturbances in schizophrenia that reconciles phenomenology with neuropsychological and empirical evidence. Raballo and Poletti will present a comprehensive analysis of recent developmental psychopathological studies of self disorders in the schizophrenia spectrum. Giersch and colleagues provide the crucial experimental evidence for the close link between weakened temporal expectancy and the disruption of continuous and unitary self in schizophrenia. Temporal expectancy helps to link and transform a chain of discrete events into our perceptual experience of continuous flow of time, but it is disrupted in schizophrenia. These results point toward potential intervention strategies. Park and colleagues present exteroceptive, proprioceptive and interoceptive contributions to abnormal mapping of bodily sensation and peripersonal space in the schizophrenia-spectrum and argue that the core problems may lie in prediction coding errors across multisensory systems. Potential intervention may lie in studying trained "sensorimotor experts" (athletes) who show precise and sharpened awareness of embodied sensations. The discussant (Ferri) will integrate these findings to highlight a new framework and multi-level approaches for understanding the etiologies of self disturbances in the schizophrenia-spectrum.

### 28.1 VARIETIES OF SELF DISORDER: A BIO-PHENO-SOCIAL MODEL OF SCHIZOPHRENIA

Barnaby Nelson*[,1], Louis Sass[2]
[1]*Orygen Youth Health Research Centre;* [2]*Rutgers University*

**Background:** The self-disorder model offers a unifying way of conceptualizing schizophrenia's highly diverse symptoms (positive, negative, disorganized), of capturing their distinctive bizarreness, and of conceiving their longitudinal development. These symptoms are viewed as differing manifestations of an underlying disorder of 'core-self': hyperreflexivity/diminished-self presence with accompanying disturbances of "grip" or "hold" on reality.
**Methods:** We have recently revised and tested this phenomenological model, in particular distinguishing primary versus-secondary factors, in offering a bio-pheno-social model of schizophrenia spectrum disorders.
**Results:** The revised model is consistent with recent empirical findings and offers several advantages:

1) It helps account for the temporal variations of the symptoms or syndrome, including longitudinal progression, but also the shorter-term, situationally-reactive, sometimes defensive, and possibly quasi-agentive variability of symptom-expression that can occur in schizophrenia (consistent with understanding some aspects of self-disturbance as dynamic and mutable, involving shifting attitudes or experiential orientations).
2) It accommodates the overlapping of some key schizophrenic symptoms with certain non-schizophrenia spectrum conditions involving dissociation (depersonalization and derealization), including Depersonalization Disorder and Panic Disorder, thereby acknowledging both shared and distinguishing symptoms.
3) It integrates recent neurocognitive, neurobiological, and psychosocial (e.g., influence of trauma and culture) findings into a coherent but multi-factorial neuropsychological account.

**Discussion:** An adequate model of schizophrenia will postulate shared disturbances of core-self experiences that nevertheless can follow several distinct pathways and occur in various forms. Such a model is preferable to uni-dimensional alternatives—whether of schizophrenia or core self disturbance—given its ability to account for distinctive yet varying experiential and neurocognitive abnormalities found in research on schizophrenia, and to integrate these with recent psychosocial as well as neurobiological findings.

### 28.2 DISORDERS OF THE EMBODIED SELF IN SCHIZOPHRENIA: AT THE CROSSROAD BETWEEN DEVELOPMENT AND PSYCHOPATHOLOGY

Andrea Raballo*[,1], Michele Poletti[2]
[1]*Norwegian University of Science and Technology;* [2]*Department of Mental Health Reggio Emilia*

**Background:** Basic disorders of the embodied self (BDES) encompass a cloud of related clinical constructs (e.g. cenesthesias, distortions of somatopsychic unity, anomalous bodily experiences in a broad sense) that are immanently related to a profound transformation of subjectivity and with the developmental modulation of bodily awareness. They have been historically ascribed a potential role in the emergence of schizophrenia spectrum disorders. However, the clinical-phenomenological level of description has been only marginally integrated with novel insights from developmental psychopathology and neurosciences.
**Methods:** We conducted a conceptual literature review based on clinical analysis and heuristic synthesis.
**Results:** Despite often occurring in prodromal/clinical at-risk states, as well as in full blown schizophrenia spectrum conditions (where BDES play a pivotal psychopathogenetic role in the genesis of productive symptoms), they are relatively neglected both in research and in routine clinical examination. Furthermore, BDES also discriminate non help-seeking genetic high risk subjects from normal controls.
**Discussion:** Within the superordinate construct of Self-disorders, BDES are a potentially relevant dimensional phenotype for the characterization of broad Schizophrenia Spectrum vulnerability. Their contextualization within a developmental and neurophysiological perspective could further amplify their value for etio-pathogenetic research.

### 28.3 MINIMAL SELF IN SCHIZOPHRENIA: THE TIME PERSPECTIVE

Anne Giersch*[,1], Brice Martin[2], Michel Cermolacce[3], Nicolas Franck[2], Patrick Poncelet[4], Jennifer Coull[5]
*[1]Centre Hospitalier Universitaire de Strasbourg; [2]Université Lyon; [3]Aix-Marseille Université; [4]INSERM; [5]Aix-Marseille Université, Service Universitaire de Psychiatrie, Hôpital Ste Marguerite*

**Background:** The feeling of being one continuous individual in time is a natural evidence, which seems to be lost for patients with schizophrenia who display 'minimal' or 'bodily' self disorders. The continuity in time is a property of the 'minimal' self and its alteration could disrupt the sense of self. It has long been proposed that patients with schizophrenia experience a breakdown of the experience of time continuity. This proposal relies on the patients' self-reports and the phenomenological analysis of their verbal descriptions. We will discuss to which extent recent experimental evidence supports this proposal and provides insight on the mechanisms underlying the perturbation of the experience of time continuity
**Methods:** We used two original experimental approaches to test the link between the sense of self and time disorders in stabilized patients with schizophrenia and controls. The first relies on the parallel measure of time expectation and minimal self disorders, as evaluated with the EASE (phenomenological scale). Time expectation is indexed by the ability to benefit from the passage of time to react to a visual target: expectation increases with time, leading to shorter reaction times. The second approach consists in asking subjects to evaluate their feeling of control when tapping with a stylus on a virtual surface. The feeling of control is a component of agency, i.e. related to the bodily self. It can be altered even when subjects know the action to be their own, and may thus show alterations in the absence of delusions. In order to test the link between the feeling of control and timing, the haptic feedback (tactile and kinesthetic) was manipulated, with perceptible or imperceptible delays.
**Results:** Both tasks show that patients can expect sensory signals and react to unusual events to some extent: they increase their reaction times after trials with missing targets, and their feeling of control decreases when sensory feedbacks are delayed. However, the patients who feel as not being immersed in the world (EASE) do not benefit from the passage of time, consistent with previous results suggesting that patients have a difficulty to fluently follow the events flow. In the motor task, contrary to controls the patients' feeling of control drops as soon as there is an imperceptible delay in the haptic feedback, and patients have difficulty to adjust sensory anticipation in case of delayed haptic feedback
**Discussion:** The results suggest a link between timing and minimal self disorders. The patients are able to expect well-learned sensory signals. However, the patients with minimal self disorders (altered immersion in the world) display time disorders consistent with a breakdown of time continuity. All patients display disrupted time expectation when events become unusual or uncertain. Expecting events in time helps to link events with one another and thus participates to transform a chain of discontinuous events in a continuous flow. Conversely, fragile time expectations may lead to a sense of discontinuity, which could disrupt perceptions and especially the flow of bodily signals, thus contributing to bodily self disorders.

### 28.4 FLEXIBLE BODY BOUNDARY AND ALTERED MAPPING OF THE BODILY SELF IN THE SCHIZOPHRENIA SPECTRUM: CAUSES, PROCESSES AND POTENTIAL INTERVENTION

Sohee Park*[,1], Lénie Torregrossa[1], Taylor Benson[1], Lauri Nummenmaa[2], Matthew Snodgress[1], Enrico Glerean[2], Eon Sol Chon[1], Seok Jin Hong[1]
*[1]Vanderbilt University; [2]University of Turku*

**Background:** Our sense of embodied self depends on continuous spatio-temporal integration and predictive coding of multisensory signals to yield a stable internal landscape. However, schizophrenia is characterized by inconsistent mapping of the physical and parasomatic body space, autoscopic hallucinations and flexible body self boundary. We aimed to elucidate the specific roles of exteroceptive, proprioceptive and interoceptive systems in generating self disturbances. Lastly, if schizophrenia represents one end of the spectrum of bodily self disorders, it is also important to understand what lies at the other extreme end, represented by those whose prediction coding is honed to perfection from years of training (athletes) to gain insight into potential remediation strategies.
**Methods:** In Study 1, components of bodily self-disturbances were examined in individuals with schizophrenia (SZ), matched controls (CO) and prodromal participants (P) with tasks that assessed tactile perception (2-point discrimination task), susceptibility to proprioceptive-tactile illusions, multisensory integration, visual body mapping of emotions (emBODY), and interoceptive awareness (heartbeat detection task). Phenomenological dissociative experiences were captured with a novel picture-based inventory (BODI). In Study 2, we recruited healthy participants with extraordinary expertise to coordinate interoceptive, proprioceptive and exteroceptive signals to perform physical tasks (athletes), and compared their embodiment of emotions with that of matched controls and individuals with schizophrenia.
**Results:** Individuals with schizophrenia and prodromal participants were impaired in interoceptive awareness, exteroceptive tactile discrimination, and audio-visual integration compared with matched control groups. SZ and P also showed increased sensitivity to proprioceptive illusions, which was associated with increased dissociative experiences and positive syndromes. Bodily sensations associated with emotions were reduced in SZ and P compared to CO. Importantly, the spatial locations of embodied emotions were different in SZ compared with CO. Interestingly, athletes showed highly precise localization of embodied emotions compared with matched controls. Self-disturbances were exacerbated by social isolation regardless of diagnosis.
**Discussion:** These results suggest that mapping of internal signals to the experience of external world is inconsistent or incoherent, contributing to fragmented and discontinuous self experience in persons with schizophrenia. More specifically, proprioceptive prediction errors seem to contribute to abnormally flexible self boundary. Diminished access to interoceptive signals may lead to reduced mapping of bodily sensations. Embodied emotions were reduced in SZ and P compared to CO. Athletes seemed to have much more precisely tuned awareness of embodied emotions. These results are consistent with the framework of increased internal neural noise in schizophrenia, which could lead to both weakened and poorly integrated interoceptive, proprioceptive and exteroceptive signaling, and a fragmented sense of self. Athletes data suggest that it may be possible to remediate bodily self disturbances via physical training. These findings underscore the importance of bringing back the body to psychiatry.

### Plenary

### 29. THE COMPLEX INTERACTIONS BETWEEN MIND AND BODY: IT TAKES A BRAIN TO CONTROL IMMUNITY

Asya Rolls
*Israel Institute of Technology*

**Overall Abstract:** Thoughts and emotions can impact physiology. This connection is evident by the emergence of disease following stress or recovery in response to placebo treatment. Nevertheless, this fundamental aspect of physiology remains largely unexplored. We have recently shown that activation of the brain's reward system, which is active in positive emotional states and positive expectations, boosts immunity. In this talk, I will discuss how brain activity can regulate anti-bacterial and anti-tumor immunity and the potential implications for health and cancer therapy. Given the crucial role of the reward system in emotional processes, our findings offer a new mechanistic insight to the association between the patient's psychological state, physical health and cancer progression.

### Concurrent Symposia

### 30. AN IMMUNE PATHOGENESIS OF PSYCHOSIS? EVIDENCE AND CHALLENGES FROM BENCH TO BEDSIDE

Rachel Upthegrove
*University of Birmingham*

**Overall Abstract:** The immune pathogenesis story of schizophrenia is gathering momentum, with increasing evidence from animal models, genetic, circulating biomarker and neuropathological studies. Potentially ground breaking new treatment approaches are proposed. However, it is vital that basic science and is equally matched by deep understanding of the complexity of clinical samples and management of multiple confounding factors when moving from bench to bedside. This presentation will pull together key speakers from a variety of fields, demonstrating the need for continued dialogue in translational, and reverse translational, approach. We will present findings from preclinical studies, genetic insights, longitudinal modelling of immune markers from population-based samples and detailed analysis from clinical samples. Data will include evidence of a prenatal immune activation and the potential transgenerational transmission of behavioural and neuronal abnormalities, co-variation of gene sets associated with both increased risk of schizophrenia and immune function (eg CSMD1, DPP4) together with CRP and peripheral inflammatory cytokine association with symptom profiles in both larger population and clinical samples. Thus, evidence presented will move from large data to fine grain analysis, animals to man and from bench to bedside.
We aim to provide insights into early pathophysiological processes and forward avenues of research to the ultimate aim of elucidating the immune dysfunction impact on psychosis and future avenues for effectiveness of treatment.

### 30.1 IMMUNE PATHOGENESIS OF PSYCHOSIS: THE CHALLENGE OF CO-MORBIDITY

Rachel Upthegrove*[,1], Carl Krynicki[1], Annalisa Gordianno[2], Carmine Pariante[2], Toby Rowland[3], Nicholas Barnes[1], Steven Marwaha[3], Benjamin Perry[3], Paola Dazzan[2], John Deakin[4]
*[1]University of Birmingham; [2]Institute of Psychiatry, Psychology and Neuroscience, King's College London; [3]University of Warwick; [4]University of Manchester*

**Background:** The immune pathogenesis story of schizophrenia is gathering momentum, with increasing evidence from genetic, circulating biomarker and neuropathological studies. New treatment approaches are being trialled. However immune dysfunction is not unique to schizophrenia, and circulating proinflammatory biomarkers identified in schizophrenia have also been identified in bipolar disorder and major depressive disorders. Similarly, in recent times there has been an increasing recognition of commonality across categorical diagnoses at a symptom level; as RDoC criteria acknowledge. For example, depressive symptoms are common in schizophrenia, hallucinations and delusional beliefs common in mood disorders and anhedonia a cross diagnostic challenge
**Methods:** This presentation will include data of altered circulating proinflammatory markers from recently completed meta-analysis in first episode psychosis, established schizophrenia and bipolar disorder, highlighting the potential pluripotent inflammation pathway to mental disorders and outline a circulating cytokine profile at the onset and development of mental disorder as related to symptom specific profiles.
**Results:** Data on circulating inflammatory makers as related to symptom profiles cross-sectional and longitudinally will also be presented from the recently concluded NIHR funded BeneMin (The Benefits of Minocycline on negative symptoms in early phase psychosis) study.
**Discussion:** Future research should recognise co-morbidity, adopt a dimensional approach, or investigate symptom specific biomarkers at early stages of illness with numbers large enough to explore an immune specific clinical profile. This knowledge is essential in the developing story of inflammation and psychosis with the most potential in decades to translate into tailored effective treatment options.

### 30.2 GENETIC VARIATION RELATED TO IMMUNE FUNCTION AND SCHIZOPHRENIA RISK: EVIDENCE FOR EFFECTS ON COGNITION

Gary Donohoe*[,1]
*[1]NUI Galway*

**Background:** Altered immune response is associated with many psychiatric disorders, but whether and how these changes confer increased risk remains unclear. In schizophrenia, robust association between illness risk and the MHC region general, and complement component 4 (C4) specifically, has been demonstrated, along with evidence from both gene enrichment and other genetic analysis highlighting the broader role of genetic variation in additional immune related networks to schizophrenia risk.
**Methods:** In a series of recent studies from our group, we examined the effects of immune-related genetic variation, based on gene ontology, implicated in neural function both behaviourally in samples of ~1200 cases and controls, and cortically in samples of ~150 cases and controls.
**Results:** We found that (1) increased predicted C4A RNA expression predicted poorer performance on measures of memory recall ($p=0.016$, corrected) and a pattern of reduced cortical activity in middle temporal cortex during a measure of visual processing ($p<0.05$, corrected); (2) variation in a curated gene set associated with both increased Schizophrenia risk and immune function (CSMD1, DPP4, SRPK2, TRIM8, STAT6, FES, EP300, TNFRSF13c) were associated with both variation in both episodic memory and general cognitive ability.
**Discussion:** Based on these findings we conclude that schizophrenia risk associated with variation within immune related genes is likely to be conferred at least partly via effects on cognition, and the molecular mechanisms involved may include effects on inflammatory response.

### 30.3 ASSOCIATION BETWEEN SERUM C-REACTIVE PROTEIN, POSITIVE AND NEGATIVE SYMPTOMS OF PSYCHOSIS IN A GENERAL POPULATION-BASED BIRTH COHORT

Golam Khandaker*[,1], Jan Stochl[1], Stanley Zammit[2], Robert Dantzer[3], Glyn Lewis[4], Peter B. Jones[1]
*[1]University of Cambridge; [2]Cardiff University; [3]University of Houston in Texas MD Anderson Cancer Centre; [4]University College London*

**Background:** An association between low-grade inflammation and symptoms commonly shared between psychiatric disorders may explain the trans-diagnostic effects of inflammation, and lead to novel mechanistic hypotheses. Schizophrenia includes diverse symptoms, but the relationship between low-grade inflammation and specific psychotic symptoms has not been examined.
**Methods:** In the general population-based ALSPAC birth cohort, serum CRP levels were assessed around age 16 years. Ten positive and ten negative symptoms of psychosis were assessed using self-report questionnaires around age 17 years. Associations between CRP and psychotic symptoms were examined using regression analysis before and after controlling for concurrent depressive symptoms, substance use, and other confounders. In addition, we used factor analysis to create positive and negative symptom dimension scores, which were then correlated with CRP.
**Results:** About 13% of 5126 participants reported at least one positive symptom. Paranoid ideation (4.8%), visual (4.3%) and auditory hallucinations (3.5%) were the most common. Negative symptoms were correlated with concurrent depressive (P<0.001), and positive symptoms (P<0.001). CRP was associated with auditory hallucinations and anhedonia after controlling for potential confounders including concurrent depressive symptoms. The adjusted OR for auditory hallucinations for those with high compared with low CRP was 2.67 (95% CI, 1.27–5.60). Evidence for this association remained after excluding participants reporting positive symptoms in the context of cannabis/drug use, physical Illness or sleep, and after excluding participants reporting positive symptoms previously at age 12 years. The adjusted OR for anhedonia per SD increase in CRP was 1.13 (95% CI, 1.01–1.26). Factor analysis revealed similar findings. CRP was associated with both positive and negative symptom dimension scores. There was evidence for interaction between CRP and sex; the associations between CRP and psychotic symptoms were stronger in women.
**Discussion:** Low-grade inflammation may be relevant for auditory hallucinations and anhedonia particularly in women. These findings need replication in other samples especially in patients with psychosis.

### 30.4 PRENATAL INFECTION AND LONG-TERM BRAIN PATHOLOGY: FROM PRECLINICAL MODELS TO MECHANISMS

Juliet Richetto*,[1]
[1]*University of Zurich*

**Background:** Prenatal exposure to infection is increasingly recognized to play an important etiological role in neuropsychiatric and neurological disorders with neurodevelopmental components, including schizophrenia, autism, bipolar disorder, and mental retardation. The adverse effects induced by prenatal infection may reflect an early entry into a deviant neurodevelopmental route, but the specificity of subsequent disease or symptoms is likely to be influenced by the genetic and environmental context in which the prenatal infectious process occurs. The epidemiological link between prenatal infection and increased risk of neurodevelopmental disorders also receives strong support from experimental work in animal models. These models are based on maternal gestational exposure to specific infectious agents such as influenza virus or immune activating agents such as the bacterial endotoxin lipopolysaccharide (LPS) or the viral mimic poly(I:C).
**Methods:** Converging evidence form these models suggests that prenatal immune activation can negatively affect early foetal brain development and change the offspring's neurodevelopmental trajectories, which in turn can lead to the emergence of behavioral and cognitive disturbances in later life. Modelling the human epidemiological association between prenatal infection and increased risk of neurodevelopmental disorders in animals has also greatly advanced our understanding of the underlying mechanisms. According to the prevailing view, cytokine-associated inflammatory events, together with downstream pathophysiological effects such as oxidative stress and (temporary) macronutrient and micronutrient deficiency, seem critical in mediating the post-acute effects of maternal infection on the foetal system.
**Results:** Recent findings have further implicated epigenetic processes as possible molecular mechanisms translating the negative effects of prenatal immune activation on the offspring. Not only does prenatal immune activation cause long-lasting epigenetic modifications such as altered DNA methylation and miRNA expression, but it also causes a transgenerational transmission of behavioral and neuronal abnormalities without additional immune exposures.
**Discussion:** Prenatal infection and associated developmental neuroinflammation may have a pathological role in shaping neurodevelopmental disease risk across generations.

### 31. OPTIMISE-ING THE TREATMENT OF FIRST-EPISODE SCHIZOPHRENIA

Celso Arango
*Hospital General Universitario Gregorio Marañon*

**Overall Abstract:** Even if there have been effective antipsychotic treatments for more than fifty years, the implementation of these treatments in clinical practice is still far from optimal, and a significant amount of patients with schizophrenia show poor outcomes. It is unquestionable that antipsychotics remain the first treatment option for patients with first-episode schizophrenia (FES). However, several questions still demand answer in the field. It is unclear how long we should wait before changing an antipsychotic that has not been fully effective, how long we have to wait before starting clozapine in FES, whether we can identify who will respond better to current treatments using biological markers including neuroimaging, and how to improve adherence and functional prognosis after clinical remission is achieved.
OPTiMiSE ('Optimisation of Treatment and Management of Schizophrenia in Europe') is an European Consortium funded under the VII Framework Program that addressed these questions in a sample of nearly 500 patients with FES or schizophreniform psychosis and minimal prior exposure to antipsychotics recruited across Europe and Israel. Within an integrative framework, the Consortium aimed at combining clinical, neuroimaging, genetic and biochemical data in a large representative sample to provide answers to these clinically relevant questions and prepare the field for personalised medication strategies as drugs with novel mechanisms of action become available. Results from this study have not yet been published and most of them will be presented for the first time in a Congress.
Based on previous results from the EUFEST study, all patients received amisulpride as a first step. For those not achieving remission after 4 weeks, OPTiMiSE compared in a randomised double-blind fashion the option of staying on amisulpride or moving to a drug with a different receptor-binding profile, olanzapine. In those not achieving remission after 6 weeks, clozapine was started. OPTiMiSE thus constitutes the first systematic, large-scale testing of the potential benefits of early switching to an antipsychotic with different characteristics in patients not achieving remission, and the application of clozapine in non-remitting patients within the first 10 weeks of treatment. OPTiMiSE also provides information on the added value of psychosocial interventions to improve treatment adherence, reduce relapses and improve functional outcomes after remission is achieved, through a 1-year follow-up randomised controlled trial testing a web-based program comprising a motivational intervention package, psychoeducation, and electronic medication alerts and updates.
OPTiMiSE also collected blood samples for patients participating in the clinical trial and a systematic sample of more than 200 standardised, high-quality, magnetic resonance imaging (MRI) images. Based on this information, OPTiMiSE examined the clinical utility and cost-effectiveness of MRI for screening of underlying organic conditions in FES. In addition, OPTiMiSE also offers novel information on how MRI alterations, genetic and biochemical markers at first presentation of schizophrenia can predict the response to subsequent treatment. For this, OPTiMiSE used two broad strategies—a combination of technology-driven (pharmacogenetics, proteomics, and metabolomics) and hypothesis-driven (neuroimaging, neurochemical, and immune-related) markers.
This symposium offers an overview of the main results obtained in the project in all these areas, with special focus on their clinical and research implications.

### 31.1 OPTIMISING THE TREATMENT AND MANAGEMENT OF FIRST-EPISODE SCHIZOPHRENIA: THE OPTIMISE CLINICAL TRIAL

René Kahn*[,1], OPTiMISE Study Group
[1]*Icahn School of Medicine at Mount Sinai*

**Background:** Very few prospective, sequential studies are available that could guide decisions which have to be made in every day clinical routine. Some of the simplest questions of clinicians remain unanswered. For example: If the first antipsychotic used has not worked, is switching to another drug effective? Or should we perhaps increase the dose? And when should we start clozapine, the most efficacious drug? These questions are most urgent for patients with a first episode of psychosis.
**Methods:** OPTiMiSE is an international clinical trial, conducted in 27 research centers located in 14 countries across Europe and Israel. Among other purposes, OPTiMiSE was designed to test a three-stage treatment algorithm. The 446 participants of OPTiMiSE were diagnosed with a first episode of schizophrenia, schizophreniform or schizoaffective disorder. Each participant started with a 4-week open label amisulpride treatment. After these 4 weeks, non-remitters are randomized to switch to another antipsychotic versus continuation of amisulpride for 6 weeks, in a double-blind fashion (phase 2). After these additional 6 weeks, non-remitters were switched to clozapine for another 12 weeks.
**Results:** Description of the sample:
Mean age at inclusion was 26 years, 30% of the patient sample was female. The median duration of illness was 4 months and none of the participants had been using antipsychotic medication for more than 2 weeks. All patients were treated on a voluntarily basis. The patient sample was moderately ill at baseline, with a mean score on the Positive And Negative Syndrome Scale (PANSS) of 78 (sd 19).
Phase 1
Patients started on 200–800 mg/day amisulpride treatment, with a target dose of 400 mg/day. Drop-out rate was 20%. Out of the 446 patients who were initiated on amisulpride, 56% met remission criteria, based on the eight remission items of the PANSS. Side effects were mild, with a mean weight increase of 2.7 kg (sd 3.3).
Phase 2
At the end of phase 1, 121 patients did not meet remission criteria. Out of the 93 patients who continued into the double-blind treatment phase, 72 patients completed the 6-week treatment phase. Remission rate in phase 2 was 35%. There was no significant difference between the two treatment arms regarding remission rate, nor regarding the drop out rate. Patients randomized to olanzapine gained significantly more weight compared to amisulpride.
Phase 3
At the end of phase 2, 40 patients did not meet remission criteria. Out of the 28 patients who continued into the open-label clozapine treatment phase, 18 patients completed this 12-week treatment from which only 5 patients met remission criteria, translating into a remission rate of only 18%. Non-remitters generally did improve to a great extent but failed to meet the stringent remission criteria.
**Discussion:** Amisulpride is confirmed to be a good option for initiating pharmacotherapy in first episode patients, resulting in a high remission rate. There was no advantage related to remission rates or drop out rates for switching non-responders after 4 week of treatment to another antipsychotic versus continuing the initiated antipsychotic for another 6 weeks; continuing the first antipsychotic may have the benefit of avoiding new side effects related to introducing a new antipsychotic. Despite a low remission rate following clozapine treatment, non-remitters generally did show a substantial reduction in symptoms, demonstrating that clozapine should be an option for non-responding psychotic patients early in the illness.

### 31.2 CAN WE IMPROVE FUNCTIONAL OUTCOME AND ADHERENCE IN OPTIMISE PARTICIPANTS WITH A PSYCHOSOCIAL INTERVENTION?

Richard Drake*[,1], Merete Nordentoft[2], Gill Haddock[1], John Ainsworth[3], Shon Lewis[3]
[1]*MAHSC;* [2]*Mental Health Centre Copenhagen;* [3]*University of Manchester*

**Background:** We hypothesised that a multi-modal psychosocial intervention (PSI) after first episodes of non-affective psychosis would increase antipsychotic adherence, improve functioning and prevent readmission in a multicentre, blind-rated, randomised controlled trial.
**Methods:** Following treatment of first episode non-affective psychosis with amisulpride, olanzapine or clozapine (those remitting after phases 1–3 of the OPTIMISE program or dropping-out but willing to enter the adherence trial) patients with DSM-IV schizophreniform disorder, schizophrenia, or schizoaffective disorder were eligible for allocation to PSI or treatment as usual (TAU). PSI involved: i) e-learning via a psychoeducational website; ii) mHealth intervention with 3 months SMS medication reminders configured by participants; iii) motivational interviewing targeting adherence over 6 weeks.
Primary outcome measures at 3 and 12 month follow-up were Compliance Rating Scale (score 1–7 worst to best), and Social and Occupational Function Assessment Scale (SOFAS; 0–100). Secondary outcomes included remission, Drug Attitudes Inventory (DAI), and EuroQoL quality of life scale. We present interim analyses of 3 month data with general linear and logistic regression models, clustered by centre and adjusted for baseline scores and demographics.
**Results:** Recruitment time, now finished, was extended to reach target sample size, so 12 month data are incomplete. 258 were allocated to PSI or TAU. 18 dropped-out before baseline assessment: 240 entered the modified intention to treat analysis (PSI 121, TAU 119). After PSI, 71% were followed-up at 3 months; after TAU, 80%. No baseline variable significantly predicted this attrition.
Webpages covering illness and treatment had 244–290 hits each. Only 24% of the PSI group set up SMS text reminders. At least 70% attended motivational interviewing, 77% of these for all sessions. Mean California Patient Alliance Scale item score was 5.2 (95% Confidence Interval, CI, 5.0, 5.3; score 1–7 poor-good).
Mean baseline SOFAS (SD) for the PSI group was 62 (14); for TAU 62 (15). General linear modelling of 12 week data included baseline CRS and drug attitudes: marginal mean SOFAS after PSI was 65.3 (CI 62.6, 68.0) and after TAU 61.4 (CI 59.3, 63.4; p0.025; standardised effect size Glass' $\Delta$=0.41). Median (IQR) compliance score was 6 (5,7) at baseline for PSI and 6 (6,7) for TAU; and 6 (5,7) at 3 months in both groups (ordinal logistic regression, p0.36).
In secondary analyses 3 month DAI did not differ significantly between PSI and TAU (marginal means 13.5 v 11.5, bootstrapped p0.164). EuroQoL wellbeing score was significantly better after PSI (marginal means 76.0 v 69.1, bootstrapped p0.003) and remission was significantly commoner (72 v 54%, binary logistic regression p0.007). No analysis result was sensitive to probability weighted adjustment for drop out.
**Discussion:** Interim analyses indicate that immediately after PSI social function and wellbeing improved significantly and remission was commoner. Adherence and DAI did not differ significantly. Either PSI's immediate benefits were non-specific or adherence measures failed to capture its effect. Longer term effects are unclear until definitive analyses planned before 2018.

### 31.3 CLINICAL UTILITY OF MRI SCANNING IN FIRST EPISODE PSYCHOSIS

Paola Dazzan*[,1], AAT Simone Reinders[1], Vasiliki Shatzi[2], Francesco Carletti[1], Celso Arango[3], Wolfgang Fleischhacker[4], Silvana Galderisi[5], Armida Mucci[5], Birte Glenthoj[6],

Alice Egerton[2], Gareth Barker[2], Stefan Leucht[7], Andreas Meyer-Lindenberg[8], René S. Kahn[9], Dan Rujescu[10], Iris Sommer[11], Inge Winter[9], Philip McGuire[1]
*[1]Institute of Psychiatry, Psychology and Neuroscience, King's College London; [2]King's College London; [3]Hospital General Universitario Gregorio Marañon; [4]Medical University Innsbruck Biological Psychiatry Division; [5]University of Naples SUN; [6]University of Copenhagen Center for Neuropsychiatric Schizophrenia Research; [7]Technical University of Munich; [8]Central Institute of Mental Health, Mannheim; [9]University Medical Center Utrecht; [10]University of Halle; [11]UMC Groningen*

**Background:** The response to antipsychotic treatment in patients with psychosis is difficult to predict on the basis of the patient's clinical features. As a result, patients are generally treated in a similar way, even though their response can vary dramatically.
Recent neuroimaging studies suggest that the pattern of brain abnormalities in patients with psychosis may vary in relation to treatment response. However, in many of these studies, patients had already been treated, and it was unclear if this had contributed to the findings.
**Methods:** In Optimise we obtained a structural Magnetic Resonance Imaging data from n=203 minimally treated patients at their first presentation for a psychotic episode. All patients then started treatment with standard doses of amisulpride. After 4 weeks, 56% were in symptomatic remission.
**Results:** We identified brain neoplasms in 3 patients, but the most common radiological findings were non-specific white matter T2-weighted hyperintensities (n=48); cavum septi pelludici (n=34); and arachnoid cysts (n=9).
Cortical thickness, surface area, and gyrification were measured using Freesurfer (). Preliminary analyses applying machine learning to these measures at baseline indicated that symptomatic remission at 4 weeks could be predicted with an accuracy of 64%.
**Discussion:** These findings suggest that radiological assessment can identify abnormalties that require an alternative to conventional treatment in a minority of patients. In most patients with psychosis, neuroimaging abnormalities may be better detected using statistical approaches, and these have greater potential for the stratification of patients according to future antipsychotic response.

### 31.4 GENETIC, IMMUNOLOGICAL AND BIOCHEMICAL MARKERS OF TREATMENT RESPONSE IN SCHIZOPHRENIA

Marion Leboyer*,[1], Stephane Jamain[2]
*[1]AP-HP, Université Paris-est-Créteil; [2]Inserm, Université Paris-Est-Créteil*

**Background:** One of the major shortcomings in the current treatment of schizophrenia is that we have no valid criteria in clinical practice to decide which antipsychotic treatment should be chosen first. This is why we need to define a blood-based biological signature of treatment response that can be easily tested at patient bedside and would also help to identify molecular mechanisms of treatment response by determining biological changes associated with symptom improvements.
**Methods:** Through a European consortium on Optimization of Treatment and Management of Schizophrenia in Europe (FP7, OPTiMiSE), we conducted a clinical trial on treatment response with Amisulpride in 500 subjects with first episode psychosis. For each patient, biological samples (DNA, RNA, plasma and serum) have been collected before treatment and during follow-up visits at weeks 4, 10 and 22, to measure biological changes associated with treatment initiation and with symptoms improvements. We combined multiple high-throughput technologies for transcriptome, genome, metabolome, proteome analyses before and after treatment.
**Results:** The transcriptome analysis conducted on 10,683 genes expressed in peripheral mononuclear cells identified significantly more genes differentially expressed after treatment in 112 patients who will be in remission after 4-weeks treatment than in 51 non-remitters. Using interaction network analysis, we identified biological pathways affected by Amisulpride. For some genes, the expression level was significantly correlated with symptom improvement. Moreover, some genes were already differentially expressed before treatment between remitters and non-remitters, suggesting they might be used to predict treatment outcome. In addition, we identified genetic variations associated with gene expression level and thus may explain individual difference in treatment response.
In parallel, as recent biological data have suggested a preponderant role of innate and adaptive immune system in the vulnerability to schizophrenia or in antipsychotic treatment response (Fond et al, 2015), we paid a particular attention to the analysis of inflammatory markers and the presence of auto-antibodies in patients' sera. Circulating autoantibodies against glutamatergic N-methyl-D-aspartate receptor (NMDAR-Ab) have been reported in up to 10% of patients with psychotic disorders. In our study, we demonstrated the advantage of using cutting-edge methods to ascertain the presence of NMDAR-Ab in seropositive patients that cannot be clinically identified ((Jezequel et al, 2017). Indeed, the only clinical characteristics found in NMDAR-Ab seropositive patients, was the high frequency of female patients, the presence of mild neurologic symptoms and signs of antipsychotic intolerance. In addition, using an advanced statistical classification algorithm, we defined clinically-based subgroups of patients who had specific cytokine signature associated to remission after 4-weeks of treatment, suggesting that these markers may be used to predict treatment response.
**Discussion:** Altogether, our multilevel biological approach resulted in the identification of promising biomarkers, which may be used both to predict drug response and remission in first psychosis episode.

### 32. DIGGING DEEPER IN THE PROTEOME OF SCHIZOPHRENIA

Daniel Martins-De-Souza
*University of Campinas (UNICAMP)*

**Overall Abstract:** Advances in genomics and transcriptomics have yielded novel insights for the pathophysiology of schizophrenia, moving the field forward by providing new substrates for the development of treatment strategies. Interestingly, this has led to a large gap in knowledge in the field, as the impact of genomic variability or alterations in transcript expression is dependent on the next level of gene expression. While proteomics has lagged behind other disciplines in schizophrenia research, several groups are utilizing proteomics approaches to ask and answer the largest possible questions in translational schizophrenia research. Proteomics has evolved as a field very quickly, going beyond the characterization of expression levels of one or a few proteins. With precise quantification of protein expression and degradation, characterization of post-translational modifications as well as the detection of low abundant proteins as putative biomarkers, we will show several different state-of-the-art proteomics approaches applied to the schizophrenia substrate. James Meador-Woodruff (University of Alabama at Birmingham) will provide a brief overview of the field, and present new data showing abnormalities of lipid and carbohydrate modifications on receptor proteins in schizophrenia, as well as abnormal levels of key enzymes associated with these abnormal protein modifications. Robert McCullumsmith (University of Cincinnati, USA) will add pivotal information on the long-standing synaptic hypothesis of schizophrenia by depicting the PSD95 interactome in normal and schizophrenia brain, connecting the excitatory postsynaptic proteome to Big Data analytics. The other two speakers will show have translational proteomics data can be used to develop clinical biomarkers. Dr. Mariana Fioramonte will present how the use of the latest tools in proteomics can tell us more about brain proteomics and protein interactomics. Finally, David Cotter (Royal College of Surgeons, Ireland) will show that proteins associated to the complement system present in the blood plasma of adolescent subjects experiencing psychosis can be predictive broadly in the vulnerability of adult psychiatric disorders. This symposium provides relevant on the biological and biochemical aspects of schizophrenia and proposes potential applications that might be, after adjustments and validations, implemented in the clinic in the future, towards a personalized medicine concept.

### 32.1 MECHANISMS OF ABNORMAL POSTTRANSLATIONAL PROTEIN PROCESSING IN SCHIZOPHRENIA BRAIN

James Meador-Woodruff*,[1]
*[1]University of Alabama at Birmingham*

**Background:** Molecular disturbances of neurotransmitter systems have long been held to be a core feature of the pathophysiology of schizophrenia. Despite years of study of neurotransmitter associated protein expression at multiple levels of gene expression, reports of abnormal neurotransmitter receptor transcript, protein, and signaling complex expression in schizophrenia brain have often been conflicting. These inconsistencies led us to reconsider neurotransmitter-based hypotheses of schizophrenia not as a problem of receptor number, or as a defect of neurotransmitter systems, but rather as a dysregulation of central cellular processes regulating the intracellular distribution of signaling proteins. Our working hypothesis is that a fundamental dysregulation of intracellular processes exists in schizophrenia, resulting in abnormal assembly, trafficking, and intracellular targeting of many key proteins involved in neurotransmission and other critical cellular functions. Previous studies have shown the important roles posttranslational lipid and carbohydrate modifications play in targeting receptors, transporters, and other proteins between intracellular compartments and the synapse, and in the lateral translocation of such molecules between lipid microdomains at the distal end of forward trafficking pathways. Accordingly, we have predicted that abnormal posttranslational lipid and/or sugar modification of proteins by occurs in schizophrenia. We have previously reported changes in extent of N-linked glycosylation as well as of the lipid modifications palmitoylation and N-myristoylation on target proteins in schizophrenia brain. In an ongoing project to elucidate mechanisms of these changes, we have studied expression patterns of key enzymes associated with these posttranslational modifications.
**Methods:** Using well characterized samples of postmortem brain from schizophrenia and matched comparison subjects, we assayed transcript expression of enzymes associated with posttranslational protein modifications by lipids and carbohydrates using microarrays and qPCR. Next, we assayed protein expression of a subset of enzymes using western blot analyses. To determine the brain cell specificity of protein changes, we used laser capture microdissection (LCM) of neuronal and glial cells to harvest specific cell populations from postmortem brains, and developed and validated a capillary electrophoresis system for ultra-low quantity protein concentration (the ProteinSimple WES system) to measure protein expression within LCM harvested cells.
**Results:** Using microarray and qPCR, multiple transcript changes were found in schizophrenia cortex. The most substantial number of altered transcripts were found for those encoding enzymes associated with multiple aspects of posttranslational carbohydrate modifications of proteins, including N-acetylglucosaminyltransferases (GlcNAcTs), glucosyltransferases, glucosidases, N-acetylgalactosaminyltransferases (GalNAcTs), galactosyltransferases, mannosidases, fucosyltransferases, fucosidases, sialyltransferases, and sialidases. Fewer changes were found for lipid modification enzymes.
Subsequently, protein expression of candidate proteins associated with these posttranslational modifications were determined by western blot analyses. Changes in protein expression of mutlple enzymes associated with glycan modification of proteins were found in schizophrenia. These include significant decreases in expression of the N-acetylglucosaminyltransferases B3GNT8 and MGAT4A, and the fucosyltransferase FUT8. Increased protein expression was found for the fucosyltransferase POFUT2, the sialyltransferase ST8SIA2, the glucosyltransferase UGGT2, and the mannosidase EDEM2. Numerous protein changes were also found in enzymes associated with lipid and glycolipid modifications. These include decreased expression of the prenylation associate denzyme subunits farnesyl-protein transferase α-subunit (FNTA), geranylgeranyltransferase type I β-subunit (PGGT1B), and rab geranylgeranyltransferase β-subunit (RABGGTB). Glycophosphatidylinositol (GPI)-anchor attachment 1 protein (GPAA1) is increased in these subjects.
To determine the cell-specific pattern of these protein changes, we have developed an LCM-capillary electrophoresis assay to isolate protein from LCM harvested cells to allow multiplex protein quantification in 500 ng of protein obtained from these cells. We have validated that we can reliably harvest cortical neuronal subtypes and astroglia, are able to measure 4 proteins simultaneously in samples from these cells lines, and are currently collecting cells to extend these findings into cell-specific studies to determine if the changes we have found in posttranslational modification proteins are widely specific or specific to given subpopulations of brain cells.
**Discussion:** These data support our earlier findings of altered patterns of the posttranslational modifications of both glycosylation and lipid modification of proteins in the cortex of schizophrenia. By identifying changes in both mRNA and protein expression of key enzymes associated with these posttranslational modifications, we have begun to elucidate potential mechanisms of these earlier observations.
One of the challenges that has plagued schizophrenia research for decades is that many different neurotransmitter and neurochemical systems have been implicated and studied in this illness, and reconciling this large literature is challenging. These many changes in numerous different systems suggest, however, that rather than schizophrenia being a disorder of a given neurotransmitter system, it is rather a disturbance of core intracellular processes that underlie regulation of multiple neurochemical systems. The machinery associated with posttranslational modifications of proteins is a possible substrate that could reconcile prior abnormalities identified in myriad systems. We have proposed that a fundamental defect in the brains of those affected with this illness is abnormal assembly, trafficking and receptor dynamics of many different proteins in schizophrenia that is due mechanistically to abnormal posttranslational modifications that influence intracellular targeting and trafficking of proteins between subcellular compartments. Dysregulation of lipid and glycan modification of proteins are likely candidates for such a process, and these present data begin to elucidate the mechanisms from which these abnormalities occur.

### 32.2 ABNORMALITIES OF SYNAPTIC PROTEOMES IN SCHIZOPHRENIA

Adam Funk[1], Kenneth Greis[1], Jarek Meller[1], Robert McCullumsmith*,[1]
*[1]University of Cincinnati*

**Background:** The human brain is comprised of billions of neurons that form networks of connections within and between brain regions. These connections facilitate neuroplastic events that underlie learning and memory, critical aspects of cognitive function often perturbed in neuropsychiatric illnesses. Neuronal signaling is mediated by fast and slow transmission events, encompassing receptors, ligands, ions, enzymes, and other substrates. These elements are spatially arranged in subcellular microdomains, facilitating juxtaposition of proteins that coordinate various biological processes. For example, synaptic transmission is modulated via release of neurotransmitter into the synaptic cleft, where receptors are activated and the postsynaptic cell modulated via electrical and chemical signals. The pre- and postsynaptic compartments include highly specialized protein clusters, with elegant and complex regulatory mechanisms that traffic proteins to and from these zones. In particular, postsynaptic densities are microdomains comprised of about 1000 unique proteins that are interacting with one another via specialized multipotent scaffolding molecule. Postsynaptic density-95 (PSD-95) is a multipotent scaffolding, trafficking, and clustering protein that links glutamate receptors, signaling molecules, and other structural proteins at postsynaptic sites. More than 95% of PSD-95 expression is localized to excitatory synapses, and it is the most abundant scaffolding protein within the postsynaptic density. Mounting genetic, proteomic, and pharmacological evidence converges on alterations in the postsynaptic density of excitatory synapses in subjects with schizophrenia. Cognitive and negative symptoms associated with dysfunction of limbic circuitry, including working memory and motivation, are particularly implicated by this mechanism. To investigate excitatory postsynaptic protein hubs in schizophrenia, we assessed the PSD-95 protein interactome from brain tissue of subjects with schizophrenia and controls.
**Methods:** Human brain tissue from fifteen subjects with schizophrenia and fifteen control subjects from the DLPFC was processed for affinity purification of PSD-95 protein complexes. We confirmed PSD-95 capture and enrichment from each sample using Western blot analyses. Samples were then pooled by region and assessed by mass spectrometry for a quality control step. Pooled samples were run in triplicate. Go versus nogo was based on finding more than 500 unique peptides in each pooled sample from each region. Next, individual samples were run through our mass spectrometry protocol in triplicate. We then subtracted any non-specifically captured peptides identified by our IgG control studies that were performed in parallel to PSD-95 affinity purification. Data were normalized within each of the mass spec runs to the most intense PSD-95 peptide. This PSD-95 peptide used for normalization was the same in every sample. Peptides that were present in at least 2 of 3 technical replicates were carried forward and subjected to quantile normalization. Peptides missing in a technical replicate were replaced by imputation, and the dataset subjected to unsupervised clustering. We also performed a semisupervised clustering protocol, non negative matrix factoring (NMF). Consensus signatures of 200 peptides were identified for each brain region, and subjected to traditional and alternative bioinformatics analyses to identify pathways, processes and compounds associated with the signatures for each brain region.
**Results:** Preliminary analyses indicate changes in the PSD-95 interactome consistent with diminished NMDA receptor signaling complex activity in schizophrenia, with lower levels of NMDA-subtype glutamate receptor subunits, as well as protein kinases associated with postsynaptic signaling in this complex. Specific biological pathways and processes identified include metabolic and inflammatory pathways. We will also present proteomic signatures generated from this dataset, and interrogate the iLINCS perturbagen database to identify drugs and genes that simulate or reverse this signature.
**Discussion:** Our preliminary data suggest that NMDA receptor function is compromised in schizophrenia. Additional work is needed to see if this is an effect of antipsychotic medications. Our proteomic findings extend the NMDA receptor hypothesis beyond the transcriptome, highlighting an important new approach for assessing abnormalities of synapses in postmortem brain.

### 32.3 MAGNIFYING THE PROTEOME OF SCHIZOPHRENIA BRAINS

Mariana Fioramonte*[,1], Daniel Martins-De-Souza[1]
[1]*University of Campinas (UNICAMP)*

**Background:** In the post-genomic era, proteomics has emerged as a powerful tool to unravel biomarker candidates and to understand human diseases from the molecular point of view. In the last decade, our group mapped the proteome of several post-mortem brain regions and the cerebrospinal fluid from schizophrenia patients and controls to help deciphering schizophrenia's pathobiology. These results led us to more recently focus on subproteomes and protein interaction analysis.
**Methods:** Using ion mobility-enhanced, data-independent acquisitions and 2D-nano UPLC fractionation, we investigated the mitochondrial, nuclear and cytosolic subproteomes of the dentate nucleus and caudate nucleus as well as the protein interactome of potential protein targets to schizophrenia.
**Results:** As previously observed, have found recurrently the differential expression energy associated proteins as well as signaling pathways associated to glutamatergic dysfunction. Proteins associated to translation machinery were also found differentially expressed, implicating spliceosome dysfunction to schizophrenia. In order to understanding this better, we investigated the protein interactome of hnRNPs by co-immunoprecipitation in the disease context.
**Discussion:** Proteomics findings may provide an integrated picture of schizophrenia´s pathobiology and the identification of proteins associated to energy, signalling and translational pathways may trace back the origins of schizophrenia.

### 32.4 ALTERED COMPLEMENT PATHWAY PROTEIN EXPRESSION IS ASSOCIATED WITH PSYCHOTIC EXPERIENCES AT AGE 11 WHICH PERSIST AT AGE 18

David Cotter*[,1], Jane English[2], Melanie Foecking[1], Mary Cannon[1], Bart Rutten[3], Stanley Zammit[4], Glyn Lewis[5], Sophie Sabhwerwal[1], Lorna Lopez[2], Aoife O'Gorman[1], Gerard Cagney[6]
[1]*Royal College of Surgeons in Ireland;* [2]*Royal College of Surgeons in Ireland, Beaumont Hospital;* [3]*Maastricht University Medical Centre;* [4]*Cardiff University;* [5]*University College London;* [6]*Proteome Research Centre, University College Dublin*

**Background:** The identification of early biomarkers of psychotic disorder is important because early treatment is associated with improved outcome. We have previously shown that altered complement and coagulation pathway associated proteins are associated pathway with psychotic disorder at age 18. In the current study we test the hypothesis that altered complement pathway proteins are associated with persisting psychotic experiences from age 11 to age 18.
**Methods:** The Avon Longitudinal Study of Parents and Children (ALSPAC) is a prospective general population cohort, and a rich resource of demographic, environmental, and clinical data on the individuals involved. We studied a subsample of the cohort who participated in psychiatric assessment interviews at age 11 and 18, and who provided plasma samples at age 11. Semi-targeted proteomic profiling was used to specifically assess the complement pathway proteins in age 11 children who experienced psychotic experiences (but not disorder) at age 11 and age 18 (n=39) compared to age 11 children who only experienced psychotic experiences at age 11.
**Results:** 11 of 34 proteins assessed were significantly differentially expressed at $p<0.05$ and of these 8 remained significant following correction for multiple comparisons. Protein changes were in keeping with increased proteins expression of most complement pathway proteins. Several protein changes represented specific replications of the changes observed in age 11 samples prior to psychotic disorder at age 18, namely increased plasminogen, complement factor H, and complement factor 1r.
**Discussion:** Our findings implicate the blood complement system in the persistence of psychotic experiences from age 11 to age 18. Considering that psychotic experiences are predictive of many psychiatric disorders our findings implicate the complement system not just in psychotic disorders, but more broadly in the vulnerability to a range of adult psychiatric disorders.

### 33. STIGMA AND RECOVERY AMONG YOUNG PEOPLE AT RISK FOR PSYCHOSIS: NOVEL INSIGHTS FROM GLOBAL RESEARCH

Sara Evans-Lacko
*London School of Economics and Political Science*

**Overall Abstract:** The recognition that attenuated clinical symptoms and impaired functioning often precede the onset of psychotic disorders has led to potentially transformative early intervention strategies which could benefit individuals during the prodromal phase through clinical intervention. However, these efforts could present a double-edged sword whereby on one hand earlier treatment could improve symptoms and facilitate recovery, but on the other hand, it could also increase labeling and associated stigma. This set of presentations draws from a diverse set of countries and researcher backgrounds, including peer perspectives to enhance recovery. Our panelists present novel data to address the potential challenges of early intervention strategies and how a better understanding of the stigmatising beliefs and experiences among individuals during the earliest signs of psychotic disorder could improve early intervention efforts.

Two presentations use new perspectives to evaluate the perceptions and impact of stigma among prospective cohorts of young people at elevated risk for psychosis. Lawrence Yang uses one of the largest known cohorts of clinical high-risk young people from a multi-site study in the USA to assess the impact of two types of stigma: stigma associated with symptoms and and stigma associated with labelling of the 'high-risk for psychosis' identification. Sara Evans-Lacko uses two unique prospective community cohorts of young people enriched for risk of psychotic disorder from Brazil and the UK and investigates the levels of personal stigma and mental health literacy in relation to psychosis among young people with and without high risk of developing psychotic disorder. She will then present new data on how this relates to intended help-seeking and actual mental health service use among those at-risk for psychotic disorder. Utilizing a different perspective based on changing medical terminology for at-risk states, Danny Koren explores the attributions made by individuals in the general population and mental health professionals when applying 'attenuated pathology' versus 'compromised health' labels to refer to at-risk psychosis states.

### 33.1 DRIVERS OF STIGMA FOR THE CLINICAL HIGH-RISK STATE FOR PSYCHOSIS—IS STIGMA DUE TO SYMPTOMS OR THE AT-RISK IDENTIFICATION ITSELF?

Lawrence Yang*[,1], Bruce Link[2], Kristen Woodberry[3], Cheryl Corcoran[4], Caitlin Bryant[5], Donna Downing[6], Dan Shapiro[3], Francesca Crump[7], Debbie Huang[7], Drew Blasco[8], William McFarlane[6], Larry Seidman[3]
*[1]Columbia University Medical Center; [2]University of California at Riverside; [3]Beth Israel Deaconess, Harvard Medical Center; [4]Icahn School of Medicine at Mount Sinai; [5]University of Massachusetts/Boston; [6]Maine Medical Center; [7]Columbia University; [8]New York University*

**Background:** The clinical high-risk state for psychosis syndrome (CHR) offers substantial potential benefits in terms of early identification and treatment for at-risk youth. Early treatment might lead to decreased symptoms, thus leading to reduced symptom-related stigma. However, stigma of the clinical high-risk state for psychosis designation might also initiate further stigma through the label of risk for psychosis. Identifying the effects of these sources of stigma is critical in order to best minimize stigma associated with CHR identification and to facilitate recovery.

**Methods:** Baseline stigma assessments were conducted with 170 clinical high risk state for psychosis individuals in a major, NIH-funded longitudinal study at Columbia University Medical Center, Harvard University Medical Center, and Maine Medical Center from 2012 to 2017. Labeling-related measures of stigma (e.g., "shame of being identified as at psychosis-risk") adapted to the CHR group, and a parallel measure of symptom-related stigma (e.g., "shame of the symptoms associated with CHR") were administered. These measures were examined in relation to outcomes of: a) self-esteem, b) quality of life, and c) social functioning, adjusting for sociodemographics and core CHR symptoms (e.g. attenuated psychotic symptoms).

**Results:** Results indicated that stigma related to symptoms was more strongly associated with all outcomes when compared with shame related to the risk-label. Stigma related to symptoms remained a significant predictor of self-esteem and quality of life even after accounting for stigma related to the risk-label and the effects of sociodemographics and CHR symptoms. Conversely, stigma related to the risk-label was no longer a significant predictor for outcomes after accounting for stigma related to symptoms.

**Discussion:** Overall, symptom-related stigma was a more salient correlate and was independently linked with self-esteem and quality of life even after accounting for the effects of stigma related to the risk-label. These results indicate that treating of symptoms through early identification and treatment may provide major benefit for CHR youth by also alleviating symptom-related stigma. These findings also indicate that CHR services should address stigma associated with symptoms immediately at first identification, as these have substantial effects on psychological and functional outcomes. These findings have implications for guiding implementation of specialized CHR services both in the United States and worldwide.

### 33.2 CAN THE STIGMATIZING RISKS OF THE 'AT-RISK' STATE BE REDUCED BY RELABELING IT 'HIGH-RISK HEALTH'? PROMISING PILOT RESULTS FROM TWO EXPERIMENTAL VIGNETTE STUDIES AMONG THE GENERAL POPULATION AND MENTAL HEALTH PROFESSIONALS.

Dan Koren*[,1], Shulamit Radin-Gilboa[1], Yulia Libas[1], Dana Carmi[1]
*[1]University of Haifa*

**Background:** While there is a wide consensus regarding the potential benefits that early detection and intervention in clinical high-risk (CHR) states for psychosis might offer, application of this paradigm in current mental healthcare systems frequently involves concerns about the iatrogenic impact of stigma on patients, families, institutions, and the society at large. Based on examples from other areas in medicine (e.g., 'high-risk pregnancy' as opposed to 'miscarriage risk syndrome', or 'hearing loss' as opposed to 'attenuated deafness') we have recently hypothesized that restructuring CHR for psychosis states as high-risk states for universal functions (e.g., reality-testing) has the potential to reduce these concerns. The goal of this presentation is to introduce this notion and present pilot data that provide preliminary support for its validity.

**Methods:** In the first study, a sample of 125 adults from the general population read an experimental vignette describing a young adolescent experiencing either mild or severe prodromal symptoms who was randomly assigned a 'psychosis-risk' or 'high-risk reality testing' diagnostic label, and answered questions about stigma, hope, and need for care toward the individual in the vignette. In the second study, a sample of 254 mental health professionals read the same experimental vignette who this time was randomly assigned an 'attenuated psychosis' or 'reality-testing loss' diagnostic label, and answered questions about stigma, hope, and need for care toward the individual in the vignette.

**Results:** In the first study, the 'high-risk reality testing' label elicited significantly higher appraisals of self-image, hope, likelihood of seeking help, and need for care than the 'psychosis risk' label. Similarly, in the second study, the 'reality-testing loss' label elicited higher appraisals of self-image, hope, likelihood of seeking help, and importance of providing care than the 'attenuated psychosis' label. In both studies, no effects were found for symptom severity.
**Discussion:** These pilot results provide first empirical support for the social and clinical potential of 'high-risk health' formulations in minimizing the potential stigmatizing harms of 'at-risk' diagnostic labels and improving help-seeking behaviors. If addition, they lay the theoretical and methodological foundation for future studies that will replicate and extend the above findings using more ecologically valid manipulations (e.g., experimental intake meeting clips) among individuals at high risk and their families.

### 33.3 LEVELS OF AND IMPLICATIONS FOR PERSONAL STIGMA AND MENTAL HEALTH LITERACY IN RELATION TO PSYCHOSIS AMONG YOUNG PEOPLE WITH AND WITHOUT RISK OF DEVELOPING PSYCHOTIC DISORDER

Sara Evans-Lacko*[,1], Petra Gronholm[1], Wagner Ribeiro[1], Kristin Laurens[2]
*[1]London School of Economics and Political Science; [2]School of Psychology, Australian Catholic University*

**Background:** Much anti-stigma work suggests that reducing stigma and improving mental health literacy could also improve access to care and support for people with psychotic disorders. This is important given that increasing help-seeking, especially during the early stages of psychosis could reduce the substantial delays to care experienced by people with psychotic disorders. Little is known about levels of personal stigma and mental health literacy among young people at-risk of psychotic disorders, whether there are differences between young people with and without elevated risk for psychosis and how this is associated with actual help-seeking for individuals at-risk of developing psychotic disorders.
**Methods:** We interviewed participants from two existing, ongoing prospective cohorts in the UK and in Brazil. Participants were initially recruited from primary schools. Both samples represent enriched community cohorts (including a greater than average proportion of young people at risk of developing psychotic disorders) in Greater London (n=407) and a similar cohort of young people in Brazil (n=1,500). Participants were presented a vignette depicting a young person with early psychosis symptoms and asked about: recognition of the disorder; intended help-seeking; beliefs about interventions and prevention, stigmatising attitudes and whether they knew someone with a similar problem. We also collected detailed clinical data on psychiatric symptoms (via SDQ [Strengths and Difficulties Questionnaire] in the UK and DAWBA [Development and Well-Being Assessment] in Brazil), presence of psychotic-like experiences, and use of mental health services and personal experiences of seeking support for a mental disorder.
**Results:** Findings on the relationship between personal stigma and mental health literacy in relation to psychotic disorders, intended help-seeking and actual mental health service use, will be presented among young people with and without risk of developing psychotic disorders in the UK and Brazil.
**Discussion:** Reducing personal stigma and improving mental health literacy among young people at risk of psychosis who do not yet use clinical services could be important for future help-seeking. Future research should investigate the impact of anti-stigma interventions among young people with and without risk of developing psychotic disorders and how this facilitates help-seeking and support for this vulnerable group.

### 33.4 UNDER WHAT CONDITIONS DO YOUNG PEOPLE DISCLOSE THEIR DIFFICULTIES? SUBJECTIVE EXPERIENCES OF YOUNG PEOPLE AT RISK OF DEVELOPING PSYCHIATRIC DISORDER

Petra Gronholm*[,1], Graham Thornicroft[2], Kristin R Laurens[3], Sara Evans-Lacko[1]
*[1]London School of Economics and Political Science; [2]King's College London; [3]University of New South Wales*

**Background:** Stigma and discrimination are proposed as critical factors contributing to the underuse of mental health services amongst young people, however these influences remain understudied. Existing research on stigma experienced by young people has focused on individuals in contact with mental health services or with a psychiatric diagnosis. Using a community sample, this study investigates subjective accounts of stigma during the early stages of mental health difficulties with regards to how disclosure and coping are considered, and how help-seeking is approached.
**Methods:** In-depth semi-structured individual interviews were conducted with young people from a Greater London, UK, community cohort. Purposive sampling criteria were used to recruit participants who reported early psychopathology of a persisting nature (emotional and/or behavioural difficulties at a clinical level, and psychotic-like symptoms), thus representing young people at-risk of developing psychiatric disorder. 29 young people aged 12-18 years took part in the study. Thematic analysis was used to analyse the interview data.
**Results:** In-depth semi-structured individual interviews were conducted with young people from a Greater London, UK, community cohort. Purposive sampling criteria were used to recruit participants who reported early psychopathology of a persisting nature (emotional and/or behavioural difficulties at a clinical level, and psychotic-like symptoms), thus representing young people at-risk of developing psychiatric disorder. 29 young people aged 12-18 years took part in the study. Thematic analysis was used to analyse the interview data.
**Discussion:** "Conditional disclosure" is central to how young people cope with their difficulties. Often stigma-related concerns in particular contributed to restricted disclosure, in this way delaying young people's initial help-seeking when difficulties emerge.

### 34. IMPROVING THE DETECTION OF INDIVIDUALS AT RISK OF PSYCHOSIS

Paolo Fusar-poli
*Institute of Psychiatry, Psychology & Neuroscience, King's College London*

**Overall Abstract:** Research findings from the past two decades have opened new opportunities for ameliorating outcomes of psychosis through indicated primary prevention in individuals at clinical high risk for psychosis (CHR-P), which can result in delayed or prevented onset of first episode.
To optimize these benefits, available research has mostly focused on improving the prognostic accuracy and the effectiveness of preventive treatments for individuals at CHR-P.
However, research evidence published in the recent years indicates that despite the prominence of the CHR state, difficulty remains in identifying all individuals who may later develop psychosis. In particular, there is converging evidence indicating that most individuals who will develop a first episode are not currently benefiting from indicated prevention. There is thus a pressing and urgent need to enhance our ability to detect the individuals who are at risk. Identifying at-risk individuals who will later develop psychosis (true positives) is particularly challenging. This symposium acknowledges these challenges by reviewing the empirical validity of the CHR-P construct for detecting individuals at risk of psychosis. At the same time, the symposium suggests specific and differential strategies for overcoming these challenges in secondary mental health care, primary care, or the community.
The first speaker (Dr. Shah) will discuss the relevance of the CHR-P construct for identifying individuals at risk for psychosis. Dr. Shah found that over half of the first episode cases in a catchment area had experienced CHR-P like features prior to their illness onset, while a substantial minority of first episode cases had not. This indicates that not all the first episode cases did pass through a CHR-P like stage, thereby providing an initial estimate of what proportion of first episode cases could be prevented through interventions at the CHR-P stage.
The second speaker (Dr. Fusar-Poli) will discuss the effectiveness of current CHR-P detection strategies in secondary mental health care. Dr. Fusar-Poli found that only a tiny minority (5%) of first episode cases accessing secondary mental health were detected by the local CHR-P service that had been fully established in the Trust. This study developed and validated an individualised risk calculator that can improve the detection of individuals at risk of psychosis in secondary mental health care.
The third speaker (Dr. Perez) will discuss how to improve detection of individuals at risk for psychosis within primary care. Dr. Perez will present a cluster-randomised controlled trial assessing whether increased specific liaison with primary care improves the clinical effectiveness and cost-effectiveness of detection of people at high risk of developing a first psychotic illness. This study showed that intensive outreach to improve liaison with primary care is clinically and cost effective for improving the detection of at risk cases.
The fourth speaker (Dr. Calkins) will discuss the importance of investigating psychosis risk as a dynamic developmental process. Dr. Calkins will present a neurodevelopment prospective study evaluating subclinical symptoms in the community. This study showed that an integrated and multidimensional evaluation of youths with early psychotic-like experiences can enrich our ability to detect individuals at risk of psychosis in the general public.
These findings will be then appraised and critically integrated by the discussant, prof. Craig Morgan.

### 34.1 DO ALL INDIVIDUALS WITH A FEP PASS THROUGH AN EARLIER CHR-P STATE? IMPLICATIONS FOR CLINICAL STAGING, EARLY DETECTION AND PHASE-SPECIFIC INTERVENTIONS

Jai Shah*,[1], Rachel Rosengard[1], Sarah McIlwaine[1], Sally Mustafa[1], Srividya Iyer[1], Martin Lepage[1], Ridha Joober[1], Ashok Malla[1]
[1]*McGill University*

**Background:** The CHR-P syndrome has attracted much attention as a potentially important stage for early intervention aimed at preventing or delaying the onset of psychosis. Knowledge regarding the transition from CHR-P to FEP has been widely described and disseminated, but a major (untested) assumption permeates this literature: that most or all patients with a FEP actually experienced an earlier CHR-P state. Examining this assumption will provide crucial information regarding the potential utility of public mental health efforts such as early case identification and prevention directed at the CHR-P stage.
**Methods:** Semistructured interviews of 351 patients and families with the Circumstances of Onset and Relapse Schedule were supplemented by chart reviews in a catchment area–based sample of FEP patients in Montréal, Canada. Retrospective information was extracted regarding baseline sociodemographic variables, psychiatric and behavioral changes, and help-seeking behavior up to the point of intake in the FEP service. Experts (N=30) working in FEP and CHR settings identified which of 27 early signs and symptoms in the Topography of Psychotic Episode instrument constituted sub-threshold psychotic symptoms if they appeared prior to a syndromal-level psychotic episode. Individuals were then followed within the FEP service for up to 2 years in order to record a range of symptomatic (positive and negative symptoms, depression and anxiety) and functional (global functioning, social and occupational functioning) outcomes.
**Results:** While most clients (between 50–68%) experienced at least one early sub-threshold psychotic symptom prior to their FEP, a substantial minority recalled no CHR-P symptoms en route to psychosis. At entry to FEP services, there were no differences in sociodemographic, cognitive, or functional variables between youth who had experienced a CHR-P state versus those who had not. Youth with a CHR-P profile had significantly longer durations between psychosis onset and making the decision to seek help (median 7.7 weeks versus 3.7 weeks), as well as the total length of the prodrome leading up to psychosis (median 36.4 weeks versus 15.0 weeks). These subgroups also differed in key symptomatic and functional outcomes, with those who passed through CHR-P states en route to FEP having significantly higher depressive and anxiety symptoms at baseline, more positive and negative psychotic symptoms at 1 year, and lower functioning for at least 1 year after the initiation of FEP treatment.
**Discussion:** A substantial minority of FEP cases did not recall a CHR-P state, suggesting that a wide range of psychopathology precedes FEP. Nonetheless, our estimates indicate that over 50% of FEP cases could still be prevented through optimal interventions targeting the CHR-P phase. This adds a novel component to previous arguments regarding the feasibility and relevance of the CHR-P construct for FEP, and underscores the importance of early case identification for this vulnerable population. Implications of these findings for contemporary clinical staging models, prevention and intervention efforts will be discussed.

### 34.2 IMPROVING THE DETECTION OF INDIVIDUALS AT RISK OF PSYCHOSIS IN SECONDARY MENTAL HEALTH CARE

Paolo Fusar-poli*,[1], Grazia Rutigliano[1], Daniel Stahl[1], Cathy Davies[1], Ilaria Bonoldi[1], Thomas Reilly[1], Philip McGuire[1]
[1]*Institute of Psychiatry, Psychology & Neuroscience, King's College London*

**Background:** The overall effect of At Risk Mental State (ARMS) services for the detection of individuals who will develop psychosis in secondary mental health care is undetermined. The objective of the study presented in this lecture is to measure the proportion of individuals with a first episode of psychosis detected by ARMS services in secondary mental health services, and to develop and externally validate a practical web-based individualized risk calculator tool for the transdiagnostic prediction of psychosis in secondary mental health care.
**Methods:** Clinical register-based cohort study. Patients were drawn from electronic, real-world, real-time clinical records relating to 2008 to 2015 routine secondary mental health care in the South London and the Maudsley National Health Service Foundation Trust. The study included all patients receiving a first index diagnosis of nonorganic and nonpsychotic mental disorder within the South London and the Maudsley National Health Service Foundation Trust in the period between January 1, 2008, and December 31, 2015. Data analysis began on September 1, 2016.
The main outcome is risk of development of nonorganic International Statistical Classification of Diseases and Related Health Problems, Tenth Revision psychotic disorders.
**Results:** A total of 91 199 patients receiving a first index diagnosis of nonorganic and nonpsychotic mental disorder within South London and the Maudsley National Health Service Foundation Trust were included in the derivation (n = 33 820) or external validation (n = 54 716) data sets. The mean age was 32.97 years, 50.88% were men, and 61.05% were white race/ethnicity. The mean follow-up was 1588 days. The overall 6-year risk of psychosis in secondary mental health care was 3.02 (95% CI, 2.88–3.15), which is higher than the 6-year risk in the local general population (0.62). Compared with the ARMS designation, all of the International Statistical Classification of Diseases and Related Health Problems, Tenth Revision diagnoses showed a lower risk of psychosis, with the exception of bipolar mood disorders (similar risk) and brief psychotic episodes (higher risk). The ARMS designation accounted only for a small proportion of transitions to psychosis (n = 52 of 1001; 5.19% in the derivation data set), indicating the need for transdiagnostic prediction of psychosis in secondary mental health care. A prognostic risk stratification model based on preselected variables, including index diagnosis, age, sex, age by sex, and race/ethnicity, was developed and externally validated, showing good performance and potential clinical usefulness.
**Discussion:** This lecture will introduce a new online individualized risk calculator which can be of clinical usefulness for the transdiagnostic prediction of psychosis in secondary mental health care. The risk calculator can help to identify those patients at risk of developing psychosis who require an ARMS assessment and specialized care. The use of this calculator may eventually facilitate the implementation of an individualized provision of preventive focused interventions and improve outcomes of first episode psychosis.

### 34.3 IMPROVING THE DETECTION OF INDIVIDUALS AT RISK OF DEVELOPING PSYCHOSIS IN PRIMARY MENTAL HEALTH CARE

Jesus Perez*[,1], Huajie Jin[2], Debra A Russo[1], Jan Stochl[1], Michelle Painter[3], Sarah Byford[2], Peter Jones[1]
*[1]University of Cambridge; [2]King's College London; [3]Cambridgeshire and Peterborough NHS Foundation Trust*

**Background:** General practitioners are usually the first health professionals contacted by people with early signs of psychosis. It is unclear whether increasing the intensity of liaison between primary care and secondary care improves the clinical effectiveness and cost-effectiveness of detecting people with, or at clinical high-risk (CHR-P) of developing, a first-episode psychosis (FEP). This is important given political commitments to facilitate early intervention and decrease waiting times in mental health. We developed and tested a theory-based intervention to improve detection and referral of these mental states.
**Methods:** The LEGS study was a cluster randomised controlled trial (cRCT) involving primary care practices (clusters) in the county of Cambridgeshire and Peterborough, UK. Consenting practices were randomly allocated into two groups: (1) low-intensity liaison between primary care and secondary care, a postal campaign consisting of biannual guidelines to help in the identification and referral of individuals with early signs of psychosis and (2) the high-intensity intervention described in the previous section, which, in addition to the postal campaign, included a specialist mental health professional to liaise with each practice and support the theory-based educational package. Concealed randomisation involved a randomly permuted sequence in blocks, with 12 strata and 96 blocks. Practices that did not consent to be randomised constituted a practice-as-usual (PAU) group. The high- and low-intensity interventions were implemented over a period of 2 years for each practice during the study period April 2010 to October 2013.
The primary outcome was the number of CHR-P referrals to Early Intervention in Psychosis Services per practice site predicated on an assumption that the intensive intervention would double them. New referrals were assessed clinically and stratified into those who met criteria for CHR-P or FEP (together: psychosis true positives) and those who did not fulfil such criteria for psychosis (false positives). Referrals from PAU practices were also analysed.
An economic evaluation quantified the cost-effectiveness of the interventions and PAU, using decision-analytic modelling. Cost-effectiveness was expressed as the incremental cost per additional true positive identified.
**Results:** Of the 104 eligible practices, 54 consented to be randomised. Twenty-eight practices were randomised to low-intensity liaison and 26 practices were randomised to the high-intensity liaison. Two high-intensity practices withdrew. High-intensity practices referred more CHR-P (incidence rate ratio (IRR) 2.2, 95% CI 0.9 to 5.1; p = 0.08)), FEP (IRR 1.9, 95% CI 1.05 to 3.4; p = 0.04) and true positive (IRR 2.0, 95% CI 1.1 to 3.6; p = 0.02) cases. High-intensity practices also referred more false positives (IRR 2.6, 95% CI 1.3 to 5.0; p = 0.005); most (68%) of these were referred on to appropriate services.
The total costs per true positive referral in high-intensity practices were lower than those in low-intensity or PAU practices; the high-intensity intervention was the most cost-effective strategy.
**Discussion:** Increasing the resources aimed at managing the primary–secondary care interface provides clinical and economic value in this setting. Early detection of CHR-P in primary care is clinically and cost-effective
This talk will also introduce the continuation of this work; a 5-year research programme that will focus on the treatment of individuals with psychotic experiences in primary care settings.

### 34.4 IMPROVING THE DETECTION OF INDIVIDUALS AT RISK OF PSYCHOSIS IN THE COMMUNITY: A NEURODEVELOPMENTAL PERSPECTIVE

Monica Calkins*[,1], Tyler Moore[1], Daniel Wolf[1], Theodore Satterthwaite[1], David Roalf[1], Bruce Turetsky[1], Christian Kohler[1], Kosha Ruparel[1], Ruben Gur[1], Raquel Gur[1]
*[1]University of Pennsylvania*

**Background:** Increasing our ability to identify youths at risk of psychosis in the general public is a key step towards an improved ability to prevent the disorder. Prospective evaluation of youths with early psychotic-like experiences can enrich our knowledge of clinical, biobehavioral and environmental risk and protective factors associated with the development of psychotic disorders.
**Methods:** By using a neurodevelopment prospective cohort study we aimed to investigate the predictors of psychosis spectrum features among US youth. This is the first large systematic study to evaluate subclinical symptoms in the community. From a Time 1 screen of 9,498 youth (age 8–21) from the Philadelphia Neurodevelopmental Cohort, a subsample of participants was enrolled based on presence or absence of psychosis spectrum symptoms to participate in an approximately 2-year (n=503, mean age=17) and/or 4-year (n=313; mean age=19) follow-up assessment. Participants were administered the Structured Interview for Prodromal Syndromes, conducted blind to initial screen status, along with the Schizotypal Personality Questionnaire and other clinical measures, computerized neurocognitive testing, and neuroimaging. Age normative references scores of baseline psychosis screening measures were applied to inform interpretation of psychosis symptom endorsements. Clinical and demographic predictors of symptom persistence were examined using logistic regression.
**Results:** At 4-year follow-up, psychosis spectrum features persisted or worsened in 58% of youths endorsing symptoms at baseline. Among youths assessed at all three time-points (n=197), 54% showed temporal stability in presence or absence of psychosis spectrum symptoms, while the remainder exhibited varying patterns of symptom emergence, remission and re-occurrence over time. Baseline depression and social/occupational dysfunction were significant predictors of the occurrence of psychosis spectrum symptoms at either follow-up. Preliminary data on neurocognition, and brain structure and function, will be also discussed with the ultimate aim of integrating them with clinical data, to provide early indices of symptom persistence and worsening in youths at risk for psychosis.
**Discussion:** Together, our findings indicate that varying trajectories of psychosis spectrum symptoms are evident early in US youth representative of the general community, supporting the importance of investigating psychosis risk as a dynamic developmental process.

### 35. PREVENTION OF PSYCHOSIS: AN INDIVIDUAL OR POPULATION APPROACH?

Mary Cannon
*Royal College of Surgeons in Ireland*

**Overall Abstract:** Psychotic disorders and schizophrenia in particular have a profound impact on patients, their caregivers and society. Mental illness is set to overtake cancer and cardiovascular disease to become the most expensive disorder in terms of direct expenditure and disability-adjusted life years over the next decade. Unfortunately, mental health has lagged behind physical disorders in terms of focus on prevention. It is imperative that prevention is taken more seriously for mental health disorders. In this symposium we present novel data and novel perspectives on risk and protective factors for psychosis from the viewpoint of prevention.
Data will be presented from large population based studies from England, France, Italy, Netherlands, Spain, and Brazil and Denmark Danish These include epidemiological studies of first episode psychosis (FEP) (both single centre and multicentre) and large register- based studies.
Olesya Ajnakina will show that only 4.1% of a sample of young adults with first episode psychosis diagnosed over a two year period had actually been seen previously by the prodromal services. This indicates that this "At risk mental state" approach is not useful for prevention of psychosis at a population level. Hannah Jongsma will present data from the EU-GEI large multicentre study showing an association between greater catchment area-level owner-occupancy and lower incidence of psychosis. She also replicated the well-established finding on increased risk for psychosis among minority groups. These findings show that we need to tackle societal factors rather than remaining focused on an individual level approach. Using register data from Denmark, Kristine Engemann Jensen reports a novel protective factor for psychosis – childhood exposure to green space. This shows the importance of the built environment for mental health – particularly for young people. Finally, Sir Robin Murray gives his particular insights on how we can prevent psychosis using data from three first episode psychosis studies. He shows, for instance, that 24% of psychosis cases could theoretically be prevented by eliminating use of high-potency cannabis use in the population. He argues that psychiatrists and psychologists need to get involved in promoting societal and legislative approaches to reducing known risk factors for psychosis. Our discussant Andreas Meyer-Lindenberg will draw on all these findings, along with his own work on risk factors such as urbanicity, in discussing how we can now move to a new prevention-focused paradigm of research on psychosis.

### 35.1 ONLY A SMALL PROPORTION OF PATIENTS WITH FIRST EPISODE PSYCHOSIS COME VIA PRODROMAL SERVICES: A RETROSPECTIVE SURVEY OF A LARGE UK MENTAL HEALTH PROGRAMME

Olesya Ajnakina*[,1], Craig Morgan[1], Charlotte Gayer-Anderson[1], Sherifat Oduola[1], Francois Bourque[1], Robin Murray[1], Anthony David[1]
[1]*Institute of Psychiatry, Psychology & Neuroscience, King's College London*

**Background:** Little is known about patients with a first episode of psychosis (FEP) who had first presented to prodromal services with an "at risk mental state" (ARMS) before making the transition to psychosis. We set out to identify the proportion of patients with a FEP who had first presented to prodromal services in the ARMS state, and to compare these FEP patients with FEP patients who did not have prior contact with prodromal services.
**Methods:** In this study information on 338 patients aged ≤37 years who presented to mental health services between 2010 and 2012 with a FEP was examined. The data on pathways to care, clinical and socio-demographic characteristics were extracted from the Biomedical Research Council Case Register for the South London and Maudsley NHS Trust.
**Results:** Over 2 years, 14 (4.1% of n=338) young adults presented with FEP and had been seen previously by the prodromal services. These ARMS patients were more likely to enter their pathway to psychiatric care via referral from General Practice, be born in the UK and to have had an insidious mode of illness onset than FEP patients without prior contact with the prodromal services.
**Discussion:** In the current pathways to care configuration, prodromal services are likely to prevent only a few at-risk individuals from transitioning to psychosis even if effective preventative treatments become available.

### 35.2 PREVENTING PSYCHOSIS: WHAT, (IF ANYTHING) CAN WE LEARN FROM THE EU-GEI INCIDENCE STUDY?

Hannah Jongsma*[,1], Peter Jones[1], Craig Morgan[2], James Kirkbride[3]
[1]*University of Cambridge;* [2]*Institute of Psychiatry, Psychology & Neuroscience, King's College London;* [3]*University College London*

**Background:** The incidence of psychotic disorders varies across replicable social and environmental gradients at both an individual and a population level, such as higher rates of disorder in urban and migrant populations. However, the factors underpinning this are unclear. The EU-GEI study was established to investigate the incidence as well as the genetic and environmental determinants of first episode psychosis in a multi-national setting. The aim of the present study was to investigate the variance found in the incidence across the 17 catchment areas in the 6 countries (England, France, Italy, the Netherlands, Spain and Brazil) included in this study at both individual and population level, and identify putative predictors of psychosis risk.
**Methods:** We conducted a population-based study of the incidence of non-organic adult ICD-10 psychotic disorders (F20-F33). Demographic data (age, sex, ethnicity) and OPCRIT diagnoses were collected, and denominator data was estimated from government sources. Crude incidence rates were directly standardised to the 2011 England and Wales Census population to account for population differences in age, sex and ethnicity. Multilevel Poisson regression was carried out to investigate variance in incidence between catchment areas by latitude, population density, and percentage of unemployment, owner-occupied houses and single-person households as markers of catchment-area level social fragmentation, using official government statistics and data from the 2011 European Population and Housing Census.
**Results:** We identified a total of 2,774 cases over 12.94 million person-years at risk, leading to a crude incidence of 21.4 per 100,000 person-years (95%CI: 19.4–23.4). The age pattern of incidence differed between men and women: crude incidence peaked in men aged 18–24 (61.0 per 100,000 person-years, 95%CI: 59.0–63.1) and declined sharply thereafter, for women rates also peaked in the youngest age group (27.0 per 100,000 person-years, 95%CI: 24.9–29.1) but decline was more gradual and there was a small but robust secondary peak after age 45. By age 35, 68% of male cases had presented to services, compared to 51% of female cases. Age-sex-ethnicity standardised incidence of all psychotic disorders varied 8-fold across settings. Poisson regression revealed higher rates in minority groups (IRR: 1.6, 95%CI: 1.5–1.7), and an association between greater catchment area-level owner-occupancy and lower incidence (IRR for a 10% increase: 0.8, 95%CI: 0.7–0.8). No relationship was found for other putative environmental risk factors, including latitude and population density. Results were similar for non-affective and affective disorders.
**Discussion:** Variance in treated incidence was substantial and was only partially explained by standardisation for age, sex and ethnicity, and Poisson regression including catchment-area level risk factors. For the prevention of psychosis two main lessons can be learned: services focused on early intervention should not have an upper age limit as half of all female (and 32% of male) cases present after age 35, and future examinations of variance should focus on socioenvironmental and not geographical determinants.

### 35.3 CHILDHOOD EXPOSURE TO GREEN SPACE – A NOVEL RISK-DECREASING MECHANISM FOR SCHIZOPHRENIA?

Kristine Engemann*,[1], Carsten Bøcker Pedersen[1], Constantinos Tsirogiannis[1], Preben Bo Mortensen[1], Jens-Christian Svenning[1]
[1]*Aarhus University*

**Background:** Schizophrenia risk has been linked to urbanization but the underlying mechanistic link remains unknown. Less green space in urbanized areas, where schizophrenia risk is high, could point to green space as an important factor. Green space is hypothesized to positively influence mental health and could mediate schizophrenia risk through noise and particle pollution removal, stress relief or other unknown mechanisms. However, the effect of green space on schizophrenia risk has not been disentangled from that of urbanization and it is unclear if different measures of green space associate differently with risk.
**Methods:** We used satellite data from the Landsat program to quantify green space for Denmark in 30 × 30m resolution for the years 1985–2013. The effect of quantity and heterogeneity of green space and urbanization at place of residence on schizophrenia risk was estimated using cox regression from a longitudinal population-based sample of the Danish population (943 027 persons). Schizophrenia risk was controlled for a range of individual and socioeconomic characteristics that may confound the effect of green space including age, sex and parental education, salary, and employment status.
**Results:** Living at the lowest amount of green space was associated with a 1.52-fold increased risk of developing schizophrenia compared to persons living at the highest level of green space. This association remained after adjusting for known risk factors for schizophrenia: urbanization, age, sex, and socioeconomic status. The strongest protective association was observed during the earliest childhood years and closest to place of residence.
**Discussion:** We found green space to decrease schizophrenia risk independent of urbanization - consequently pointing to green space as a new environmental risk factor for schizophrenia development. This study supports findings from other studies highlighting the natural environment as an important factor for human health, and points to a new methodological framework that combines epidemiological studies with big data approaches.

### 35.4 A PUBLIC HEALTH APPROACH TO THE PREVENTION OF PSYCHOSIS

Robin Murray*,[1], Marta Di Forti[1], Evangelos Vassos[1], Antonella Trotta[1], Harriet Quigley[1], Olesya Ajnakina[1], Diego Quattrone[1], Giada Tripoli[1], Victoria Rodriguez[1], Craig Morgan[1]
[1]*Institute of Psychiatry, Psychology & Neuroscience, King's College London*

**Background:** The main attempt to prevent the development of psychosis has been through clinics for people at clinical high risk. Such an approach is useful for research but can never reach the majority of individuals who will become psychotic. Biological markers could be used to identify individuals with unusual vulnerabilities e.g. those with copy number variations such as VCFS. However, identifying the with such markers is unlikely to impact on the majority of cases, and as yet no useful interventions are available. How therefore to prevent psychosis?
**Methods:** Data will be presented from 3 studies of first onset psychosis (FEP) which used similar methods of ascertainment and assessment of cases and controls; AESOP and GAP from South London and the EU-GEI across 16 sites in 5 European countries.
**Results:** The identified risk factors for psychosis were the polygenic risk score for schizophrenia, childhood abuse, living in a city, being from an ethnic minority, drug abuse, adverse life events. Clearly, reducing some of these (e.g. urbanicity or migration) is not within the powers of psychiatrists. The GAP study showed that the polygenic risk score accounted for the greatest variance in caseness; those with scores in the highest quintile were 7 times more likely to be a psychotic case than those in those lowest quintile. The GAP study also gave estimates of the population attributable fraction (PAF): these indicated that if no one was exposed to child abuse and use of high potency cannabis, then 16% and 24% respectively of psychosis in South London could be prevented. The EU-GEI study showed striking differences in the incidence of psychosis between Northern and Southern Europe; data will be prevented concerning the contribution of risk factors, especially cannabis use, to this.
**Discussion:** The knowledge that schizophrenia is the extreme of a continuum of psychosis has important implications for prevention. Preventive approaches to hypertension or obesity do not focus on identifying individuals carrying biological markers; rather they encourage members of the general population to take exercise and reduce their calorie intake. A similar approach should be adopted for psychosis. In the long-term attempts to reduce risk factors should be made e.g. addressing psychotogenic aspects of city living or by decreasing discrimination of ethnic minorities. This will be difficult. However, an obvious place to start is by attempting to influence society's patterns of consumption of high-potency cannabis. Unfortunately, public policy in the US and certain other countries appears to be moving in the opposite direction with increases in consumption and potency. Are these countries sleep-walking to more psychosis?

### Plenary

### 36. INVESTING IN RECOVERY – AN ECONOMIC AS WELL AS MORAL IMPERATIVE

David McDaid
*London School of Economics and Political Science*

**Overall Abstract:** 'Recovery' is a key concept in mental health policy around the globe. The World Health Organization has called for 'a recovery-based approach that puts the emphasis on supporting individuals with mental disorders and psychosocial disabilities to achieve their own aspirations and goals'. Investing in evidence-based actions to help foster recovery should therefore be core to any system of support for anyone experiencing schizophrenia or other severe mental health problems. While there is clearly a moral imperative to maximise opportunities for recovery, the economic case for action can also be compelling and complementary. However, the opportunity to make an economic argument to support investment in recovery is not always taken, and even when made it is often too narrow in ambition and scope to have a major influence policy and practice. This presentation will highlight examples of the economic potential of recovery-focused services in health, employment, education and housing services. It will look at strengths and weaknesses in the way in which economic evidence is presented to policy makers, including the extent to which implementation challenges have been considered. It will argue that in making the economic case for recovery it is just as vital to look at the role of the messenger as well as the message that is being communicated.

### Plenary

### 37. THE GUT MICROBIOME: A KEY REGULATOR OF NEURODEVELOPMENT AND BEHAVIOUR

John Cryan
*University College Cork*

**Overall Abstract:** The brain-gut-microbiota axis is emerging as a research area of increasing interest for those investigating the biological and physiological basis of neurodevelopmental, age-related and neurodegenerative disorders.

The routes of communication between the gut and brain include the vagus nerve, the immune system, tryptophan metabolism, via the enteric nervous system or by way of microbial metabolites such as short chain fatty acids. These mechanisms also impinge on neuroendocrine function at multiple levels. Studies in animal models have been key in delineating that neurodevelopment and the programming of an appropriate stress response is dependent on the microbiota. Developmentally, a variety of factors can impact the microbiota in early life including mode of birth delivery, antibiotic exposure, mode of nutritional provision, infection, stress as well as host genetics. At the other extreme of life, individuals who age with considerable ill health tend to show narrowing in microbial diversity. Stress can significantly impact the microbiota-gut-brain axis at all stages across the lifespan. Recently, the gut microbiota has been implicated in a variety of conditions including obesity, autism, schizophrenia and Parkinson's disease. Moreover, animal models have been key in linking the regulation of fundamental brain processes ranging from adult hippocampal neurogenesis to myelination to microglia activation by the microbiome. Finally, studies examining the translation of these effects from animals to humans are currently ongoing. Further studies will focus on understanding the mechanisms underlying such brain effects and developing nutritional and microbial-based intervention strategies.

### Concurrent Symposia

### 38. DO NMDAR ANTIBODIES CAUSE SCHIZOPHRENIA?

Belinda Lennox
*University of Oxford*

**Overall Abstract:** NMDAR antibodies have been described in association with some people with schizophrenia. However the finding is still controversial, and in particular some groups describe equal prevalence of antibodies in patients with schizophrenia as other disease controls, or in healthy control subjects. this symposium includes the leading academics undertaking research in this area and will discuss the hot topics in the area, reviewing the latest evidence from a range of perspectives. This will include comparison of testing methods for NMDAR antibodies, discussion of functional effects of NMDAR antibodies with relevance to schizophrenia, an update on prevalence studies of antibodies in psychosis and at risk mental states, and clinical data on the experience of screening patients for NMDAR antibodies in psychiatric hospitals. The discussant is Sarosh Irani, associate professor in neurology at the University of Oxford, who led the first European case series description of NMDAR antibodies.

### 38.1 IMPACT OF ANTI-NMDA RECEPTOR AUTOANTIBODIES FROM PSYCHOTIC PATIENTS ON THE GLUTAMATE SYNAPSE

Laurent Groc*,[1]
[1]*CNRS, University of Bordeaux*

**Background:** The flourishing identification of circulating autoantibodies against neuronal receptors in neuropsychiatric disorders has fostered new conceptual and clinical frameworks. However, their putative presence in different diseases, as well as in healthy subjects, has raised questions about detection reliability and pathogenic role.
**Methods:** Using a combination of single molecule-based imaging approaches, cell calcium imaging, and single-cell electrophysiological recordings, we investigated in hippocampal networks the impact of autoantibodies against glutamate NMDA receptor (NMDAR-Ab) on several aspects of the glutamate synapse.
**Results:** We ascertain the presence of circulating autoantibodies against glutamate NMDA receptor (NMDAR-Ab) in about 20% of psychotic patients diagnosed with schizophrenia and very few healthy subjects. NMDAR-Ab from patients and healthy subjects do not compete for binding on native receptor. Strikingly, NMDAR-Ab from patients, but not from healthy subjects, specifically alter the surface dynamics and nanoscale organization of synaptic NMDAR and its anchoring partner the EphrinB2 receptor. Functionally, only patients' NMDAR-Ab prevent long-term potentiation at glutamatergic synapses while leaving NMDAR-mediated calcium influx intact. Furthermore, we unveil that NMDAR-Ab from first episode psychotic patients produced similar effects.
**Discussion:** By taking advantage of the single molecule imaging and complementary ensemble approaches, we unveil that NMDAR-Ab from psychotic patients (schizophrenic and first episode) profoundly alter NMDAR synaptic transmission and NMDAR-dependent synaptic functions, supporting a pathogenically relevant role.

### 38.2 NEURONAL AUTOANTIBODIES IN PSYCHOSIS: ENOUGH ABOUT PREVALENCE, WHAT'S THE RELEVANCE?

Thomas Pollak*,[1]
[1]*Institute of Psychiatry, Psychology & Neuroscience, King's College London*

**Background:** One source of controversy in the emerging field of autoimmune psychiatry concerns varying prevalence estimates of neuronal surface autoantibodies (NSAbs) in psychiatric disorders, particularly psychotic disorders. Differences in assay methodology and patient selection may contribute to varying case-control estimates.
I will argue that the field needs to move beyond small n prevalence studies, to address the question of the relevance of NSAbs in psychotic disorders, and namely the following questions:

1) Does the presence of NSAbs offer any aetiopathological insights into psychosis i.e. by associating with other disease-relevant biomarkers?
2) Do NSAbs shape the clinical phenotype of psychotic disorders?
3) Do NSAbs have a predictive role in psychotic disorders, in terms of treatment response or course of illness?

**Methods:** To address this issue, we have undertaken measurement of NSAbs using multiple immunoassays in a cohort of individuals at ultra-high risk for psychosis, and another first episode psychosis cohort. Associations between NSAb seropositivity and phenotype, outcome and biomarkers including structural MRI were explored.
**Results:** NSAbs were detected at rates of between 1% and 9% of cases in both cohorts, depending on assay used. Live CBAs detected significantly more NMDAR and GABA-AR IgG antibodies than did fixed CBAs. Rates in cases were not significantly different from controls, regardless of assay.
Nevertheless in UHR subjects NSAbs, and NMDAR Abs in particular, showed clear aetiopathological and phenotypic relevance, associating with cognitive function (poorer verbal memory and IQ), more severe psychopathology and increased volumes of key limbic areas. Significant interactions with a marker of blood-brain barrier integrity offered further aetiopathological insights. NSAbs detected by both fixed and live CBAs demonstrated phenotypic associations and interactions with BBB status, suggesting both assays can detect phenotypically relevant antibodies in the UHR context. In FEP subjects, no such associations were noted. GABA-A receptor antibodies, which have been proposed as NSAbs with emerging disease-relevance, showed no phenotypic associations.
Data on the predictive utility of NSAbs in UHR and FEP subjects will be presented.
**Discussion:** With appropriately fine-grained phenotyping and careful consideration of moderating biological factors and assay variation, clear disease-relevance of NSAbs could be established in UHR subjects but not in FEP subjects. In particular, NMDAR antibodies may have important biomarker potential in the at-risk mental state.
The failure to establish clear disease-relevance in previous psychiatric cohorts may reflect a genuinely irrelevant antibody but could also be due any of the following:

1) Inadequately fine-grained phenotyping of subjects
2) Ignoring the important moderating role of BBB permeability
3) Choosing subjects at 'too late' a stage of illness
4) Inadequately sensitive antibody detection assays

### 38.3 ONGOING GERMINAL CENTRE REACTIONS CONTRIBUTE TO N-METHYL-D-ASPARTATE RECEPTOR (NMDAR) ANTIBODY PRODUCTION IN NMDAR-ANTIBODY ENCEPHALITIS

Mateusz Makuch[1], Robert Wilson[1], Adam Al-Diwani*,[1], James Varley[1], Anne-Kathrin Kienzler[1], Jennifer Taylor[1], Patrick Waters[1], Isabel Leite[1], Belinda Lennox[1], Sarosh Irani[1]
*[1]University of Oxford*

**Background:** Immunoglobulin G (IgG) against the NR1-subunit of the N-methyl-D-aspartate (NMDAR) receptor mediates NMDAR-antibody encephalitis (NMDAR-Ab-E). This multi-stage illness presents with an acute severe psychiatric syndrome, alongside other neurological features, similar to human and animal NMDAR antagonist models. The disease is associated with an ovarian teratoma in around 20% of cases. The cellular immunity underlying this disease is not well understood. While antibody-modifying immunotherapies often promote disease resolution, the illness can be refractory to these treatments correlating with sub-optimal outcomes.
NR1-IgG can be detected several years after clinical resolution, which may be via ongoing germinal centre reactions or the establishment of antibody-secreting cells as long-lived plasma cells in bone marrow niches. These two divergent models implicate use of differing immunotherapies to target these cells. Here we investigate the contribution of ongoing germinal centre reactions to disease progression, potentially informing disease mechanisms and guide targeted immunotherapy.
**Methods:** We hypothesised that recurrent antigen-driven germinal centre reactions would be associated with active generation of NR1-specific IgM and IgG and NR1-specific circulating B cells. We validated a NR1-IgM cell based assay establishing specificity cut-offs by screening healthy and disease control cohorts alongside a previously collected NMDAR-Ab-E cohort (n=46). Following this we went on to explore the temporal evolution of NR1-IgG and NR1-IgM titres in a prospective cohort (n=12).
To investigate the lymphocyte characteristics, we stimulated ovarian teratoma lymphocytes and peripheral blood mononuclear cells (PBMCs) from multiple time points under varying cytokine conditions to understand whether these circulating cells showed capacity for NR1-IgG and IgM generation.
**Results:** We found a 43% prevalence rate of NR1-IgM in the historic cohort. We then confirmed that NR1-IgM binding was specific by its selective depletion after anti-IgM precipitation but not with protein G. In the prospective cohort, we noted often high titres of IgM (up to 1:500) most commonly early in the disease but persisting for around 2 years. NR1-IgM levels varied in titre alongside NR1-IgG spikes. Consistently, culture experiments of patient lymphocytes (PBMCs and tumour-derived) produced varying degrees of NR1-IgM and NR1-IgG under conditions associated with B cell proliferation. The NR1-IgG levels correlated with serum NR1-titres suggesting these circulating B cells made a proportional contribution to serum levels.
**Discussion:** Ongoing germinal centre reactions likely contribute much of the circulating NR1-specific B cell population in NMDAR-Ab-E. Autoimmunisation at these centres represents an as yet unexplored therapeutic target in this and potentially other autoimmune encephalopathies. Regional specificity of these reactions including lymph nodes draining sources of NR1-antigen require further direct evaluation.

### 38.4 PREVALENCE OF ANTI-NEURONAL ANTIBODIES IN PATIENTS ADMITTED WITH FIRST EPISODE OF PSYCHOSIS AND THEIR CLINICAL OUTCOMES

James Scott*,[1], David Gillis[2], Alex Ryan[1], Hethal Hargovan[2], Stefan Blum[1]
*[1]The University of Queensland; [2]Royal Brisbane and Women's Hospital*

**Background:** Anti-neuronal antibodies are associated with psychosis although their clinical significance in first episode of psychosis (FEP) is undetermined. This study examined the prevalence of anti-neuronal antibodies in patients admitted to hospital for treatment of their first episode of psychosis and described clinical presentations and treatment outcomes of those who were antibody positive.
**Methods:** Between July 2013 and May 2015, all consenting patients aged between 12 and 50 admitted for their first episode of psychosis to three mental health hospitals in Queensland, Australia, were tested for anti-neuronal antibodies in serum. Antibody positive patients were referred for neurological and immunological consultation and treatment.
**Results:** During the study, 154 FEP patients were admitted with their first episode of psychosis and 113 consented to participate. Six patients were found to have anti-neuronal antibodies; (anti-NMDAR antibodies [n = 4], VGKC antibody [n = 1], antibody against uncharacterised antigen [n = 1]). Of these, five received immunotherapy, leading to complete resolution of psychosis in four.
**Discussion:** A small, but significant subgroup of patients with first episode psychosis have anti-neuronal antibodies detectable in serum and evidence of central nervous system autoimmune pathology. Early identification of these patients and referral for appropriate treatment is critical to optimise recovery.

### 39. VIRUSES AND SCHIZOPHRENIA: IMPLICATIONS FOR PATHOPHYSIOLOGY AND TREATMENT

Alan Breier
*Indiana University School of Medicine*

**Overall Abstract:** The viral hypothesis of schizophrenia posits that viral infections disrupts cortical circuits that give rise to schizophrenia psychopathology. Prenatal viral exposure during key neurodevelopmental periods, either through direct effects on fetal brain or exposure to excessive maternal cytokines and other chemokines, have been implicated. In addition, abnormal activation of dormant neuro-viruses have been linked to the pathophysiology of schizophrenia. Activation of dormant viruses has potentially important treatment implication for therapies, such as valacyclovir, that suppress viral activity. Among the viruses that have been mostly frequently associated with schizophrenia include herpes simplex virus type 1 (HSV1) and Epstein-Barr virus (EBV). The purpose of this symposium is to focus on the role of viruses in the pathophysiology of schizophrenia and results of antiviral treatment trials in this illness.
Diana Perkins will present data from the North American Prodrome Longitudinal Study (NAPLS2) which is an eight-site observational study of predictors and mechanism of conversion to psychosis and is comprised of a cohort of 763 individuals at clinical high risk for developing psychosis. This paper examines methylation of promoter regions of genes associated with gene expression and reports that 10 markers correctly classified individuals who converted to psychosis. The SIRT1 gene, that is upregulated with HSV, was among the predictive markers.

Faith Dickerson will focus on the association between HSV1 exposure and cognitive impairment in schizophrenia and the potential link between EBV and this illness. In a large cohort of 828 individuals and 573 controls, a significant relationship between HSV1 exposure and cognitive impairment was found. The strongest linkage was in the domain of immediate memory. In 397 subjects with schizophrenia who were compared to 289 controls, significantly higher levels of antibodies to the EBV viral capsid antigens (VCA) was discovered in the schizophrenia cohort.
Vishwajit Nimgaonkar will examine the relationship between HSV1 infection and cognitive performance in a mixed cohort of 226 individuals and present the results of a randomized clinical trial of the anti-viral agent valacyclovir. HSV1 infected participants had significantly lower scores on Emotion Identification and Discrimination (EMOD), spatial memory and spatial ability irrespective of schizophrenia diagnoses. Valacyclovir treatment (1.5 grams BID, 16-week trial) improved EMOD.
Alan Breier will report the results of the VISTA study – 12-site, double-blind, placebo controlled, 16-week trial of the anti-viral medication valacyclovir (3 grams/day) in early phase schizophrenia. 170 subjects were randomized of whom 74 were HSV-1 seropositive and 96 were seronegative. Baseline working memory scores (letter number sequence) were significantly lower in HSV1 positive as compared to HSV1 negative subjects. Analysis of valacyclovir treatment outcomes have only recently commenced and are ongoing. The complete data set (cognitive domains, role function, symptoms and safety) will be presented in full at the meeting.

### 39.1 DNA METHYLATION OF IMMUNE CELLS IN PERSONS AT CLINICAL HIGH RISK FOR PSYCHOSIS

Diana Perkins*[,1], Jeffries Clark[1], Jean Addington[2], Carrie Beardon[3], Kristin Cadenhead[4], Tyrone Cannon[5], Barbara Cornblatt[6], Daniel Mathalon[7], Thomas McGlashan[5], Larry Seidman[8], Ming Tsuang[4], Elaine Walker[9], Scott Woods[5]
*[1]University of North Carolina at Chapel Hill; [2]University of Calgary; [3]University of California, Los Angeles; [4]University of California, San Diego; [5]Yale University; [6]The Zucker Hillside Hospital; [7]University of California, San Francisco; [8]Beth Israel Deaconess, Harvard Medical Center; [9]Emory University*

**Background:** A dysregulated immune system is implicated in the development of psychotic disorders. Persons with schizophrenia have altered levels of circulating immune cell signaling molecules (cytokines), and elevation of specific cytokines predict conversion to psychosis in persons at clinical high risk. Whether these peripheral signals are a causal or a secondary phenomenon is unclear. But, subpopulations of circulating immune cells do regulate the brain from meningeal and perivascular locations influencing cognition, mood, and behavior, and thus may be relevant to schizophrenia vulnerability. Hematopoietic stem cells in the bone marrow differentiate into cascading subtypes depending on signals from other organs, especially the brain. For example, a monocyte subpopulation emerges with repeated social defeat that establish the persistence of anxiety-like behaviors; blocking their release or inhibiting their attachment to brain vascular endothelium prevents the emergence of anxiety-like behaviors. In humans, a similar monocyte subpopulation is associated with social isolation and other adversities including low SES, chronic stress, and bereavement.
**Methods:** The North American Prodrome Longitudinal Study (NAPLS2) is an eight-site observational study of predictors and mechanisms of conversion to psychosis The full cohort includes 763 at clinical high risk (CHR) based on the Criteria of Prodromal State (COPS) and 279 demographically similar unaffected comparison (UC) subjects. Methylation of whole blood DNA collected in PAXgene tubes at baseline was analyzed with the Illumina 450k array in a subgroup of 59 subjects who converted to psychosis (CHR-C), 84 CHR subjects followed for 2 years who did not develop psychosis (CHR-NC) and 67 unaffected subjects (UC). Our analyses focused on methylation of promoter regions of genes, associated with gene expression. Classifier construction used Coarse Approximation Linear Function (CALF) with bootstrapping of 1000 random 80% subsets with replacement to determine statistical likelihood.
**Results:** We found highly overlapping sets of differentially methylated promoter regions in CHR-C subjects compared to CHR-NC and to UC subjects. A set of 10 markers correctly classified CHR-C and CHR-NC subjects with high accuracy (AUC=0.94, 95% CI 0.89–0.98). Included was SIRT1, a gene that is upregulated with HSV reactivation.
**Discussion:** Circulating immune cells excerpt powerful influences on mood, cognition and behavior. An obvious example is the experience of most human with “sickness syndrome”, characterized by apathy, avolition, and withdrawal, and triggered by immune-cell-released cytokines producing an adaptive, resource conserving, behavioral response. While at an early stage, our findings further implicate immune system dysregulation as a mechanism in the development of psychosis.

### 39.2 VIRAL EXPOSURES AND SCHIZOPHRENIA

Faith Dickerson*[,1], Robert Yolken[2]
*[1]Sheppard Pratt; [2]Johns Hopkins University School of Medicine*

**Background:** Epidemiological, immunological, and microbiological studies indicate that infections with members of the family Herpesviridae may be associated with schizophrenia and with cognitive impairment. Herpesviruses are enveloped, double-stranded DNA viruses which are widely prevalent and which are capable of causing persistent infections. The most highly replicated association is that between the alpha herpesvirus Herpes Simplex Virus Type 1 (HSV-1) and cognitive impairment in schizophrenia. Acute HSV-1 infection results in oral lesions which usually resolve spontaneously. However, latency can occur in nerve root ganglia leading to cycles of reactivation in later life. Other herepsviruses may also be associated with schizophrenia. Epstein Barr virus (EBV) is a gamma herpesvirus usually acquired in childhood or adolescence. Acute EBV infection is often associated with fever and adenopathy leading to a vigorous immune response and the suppression of viral replication. However, latency can occur with long term consequences to the infected individual.
**Methods:** We examined the association between HSV-1 seropositivity and cognitive functioning in 828 individuals with schizophrenia from the Sheppard Pratt cohort and 573 control individuals. We also studied antibodies to EBV in a recently enrolled subset of the Sheppard Pratt cohort consisting of 397 individuals with schizophrenia and 289 without a psychiatric disorder. Antibodies to HSV-1 and EBV proteins were measured by immunoassay and confirmed by Western blot. Cognitive functioning was measured by the Repeatable Battery for the Assessment of Neuropsychological Status (RBANS). Regression models were employed to define the independent association between virus exposure and outcome.
**Results:** Serological evidence of exposure to HSV-1 was associated with significantly lower levels of cognitive functioning as measured by the RBANS Total score (coefficient =-3.84, 95% CI -5.60, -2.09, p<.0001). The strongest association was in the domain of Immediate Memory (coefficient= -4.95, 95% CI -7.24, -2.66, p<.0001.) There was a smaller but statistical significant relationship between serological evidence of exposure to HSV-1 and RBANS Total score in control individuals. (coefficient =-1.98, 95% CI -3.88, -.094, p=.04).
In terms of EBV, we found that individuals with schizophrenia had significantly higher levels of antibodies to the EBV viral capsid antigens (VCA) as compared to controls (coefficient= .57, 95% CI .37- .77, p<1.7 10-8). On the other hand, the level of antibody to the EBV Nuclear Antigen (EBNA) and EBV Early Antigen (EA) did not differ between the groups. Within the schizophrenia group, increased levels of EBV VCA antibodies were associated with older age, female gender, and cigarette smoking but not with clinical or cognitive measures.
**Discussion:** The mechanism of the association between HSV-1 exposure and cognitive deficits in individuals with schizophrenia may be due to underlying neuroanatomical deficits, immune dysregulation, or gene x environmental interactions. The aberrant immune response to EBV may represent underlying immunopathy or exposure to variant viral strains. Other herpesviruses, such as Cytomegalovirus, Herpes Simplex Virus Type 2 and Human Herpesvirus Type 6 have also been associated with schizophrenia risk or with cognitive impairment in some populations. The prevention and treatment of herpesvirus infections might lead to new therapeutic modalities for schizophrenia.

### 39.3 CAN NEUROVIRAL INFECTIONS WITH HERPES SIMPLEX VIRUS, TYPE 1 (HSV-1) CONTRIBUTE TO RDOC?

Vishwajit Nimgaonkar*[,1], Smita Deshpande[2], Triptish Bhatia[3], Konasale Prasad[1], Faith Dickerson[4], Raquel Gur[5], Ruben Gur[5], Kehui Chen[1], Joel Wood[1], Robert H Yolken[6]
[1]*University of Pittsburgh;* [2]*Dr. R.M.L. Hospital;* [3]*Indo-US Schizophrenia Study;* [4]*Sheppard Pratt;* [5]*University of Pennsylvania;* [6]*Johns Hopkins School of Medicine*

**Background:** The 'viral hypothesis of schizophrenia' is difficult to prove, partly because it is inherently difficult to find causes for chronic conditions. It is also not easy to date and relate the timing of infections or psychiatric diagnoses. Further, many neuroviruses cannot be isolated from infected persons. Progress could be made using RDoC for cognition as outcomes of infection, since those quantifiable variables cross psychiatric diagnostic domains (as do infections); they can also be monitored longitudinally and assessed with regard to treatment. We have tested this paradigm in relation to Herpes simplex virus, type 1 (HSV-1) that infects over 3.4 billion people. Though it can cause encephalitis, it is asymptomatic in the vast majority, only causing lifelong latent infection in neurons. Over 10 cross-sectional studies and longitudinal studies indicate that even in the absence of overt encephalitis, individuals seropositive for HSV-1 perform significantly worse than seronegative individuals in several cognitive domains, particularly those relating to memory. These associations remain significant following adjustment for demographic variables, such as socio-economic status. Even though post-mortem studies have been inconclusive, brain imaging studies also indicate gray matter volume reductions in frontotemporal regions in infected individuals, consistent with the cognitive impairment. Many neuronal models of latency are also available.
**Methods:** In a prospective naturalistic follow up sample (PNFU), temporal changes in cognitive functions were analyzed in relation to baseline HSV-1 infection in persons with or without schizophrenia (N=226). Separately, in a randomized controlled trial (RCT), HSV-1 infected, clinically stabilized outpatients with SZ received Valacyclovir (VAL, an antiviral, 1.5 G twice daily for 16 weeks) or placebo (PLA) added to standard antipsychotic treatment, using a stratified randomization design, following placebo run-in (N=67). In both samples, HSV-1 infection (seropositivity) was estimated using serum IgG antibodies. All clinical evaluations were blinded to HSV-1 or treatment. Standardized Z scores for accuracy on eight cognitive domains were analyzed for temporal trajectories using generalized linear models (PNFU) and VAL/PLA differences compared with intent to treat analyses (RCT).
**Results:** PNFU: At baseline, HSV-1 infected participants had significantly lower accuracy scores for Emotion Identification and Discrimination (EMOD), Spatial memory and Spatial ability (p=0.025, 0.029, 0.046, respectively), regardless of SZ diagnosis. They also had a significantly steeper temporal worsening for EMOD (p=0.03). RCT: EMOD improved significantly in VAL-treated patients (p=0.048, Cohen's d=0.43).
**Discussion:** HSV-1 infection is associated with time-related dysfunction in EMOD, which indexes social cognition. Conversely, VAL treatment improves EMOD. A portion of HSV-1 associated cognitive dysfunction is progressive, but remediable. Viral infections could be used to investigate and validate RDoC criteria.

### 39.4 A DOUBLE-BLIND TRIAL OF VALACYCLOVIR TO IMPROVE COGNITION IN EARLY PHASE SCHIZOPHRENIA: RESULTS FROM THE VISTA STUDY

Alan Breier*[,1], Faith Dickerson[2], Robert Buchanan[3], Stephen Marder[4], Keith Neuchterlein[4], Deepak D'Souza[5], Michael Francis[1], Alexander Radnovich[1], Robert Yolken[6], Sheldon Preskorn[7], Matthew Macaluso[7], Ziyi Yang[1], Nicole Mehdyoun[1], Rishi Kakar[8], Walter Dunn[4], Debra Hoffmeyer[9], Gerald Maguire[10]
[1]*Indiana University School of Medicine;* [2]*Sheppard Pratt;* [3]*Maryland Psychiatric Research Center;* [4]*University of California, Los Angeles;* [5]*Yale University School of Medicine, VA Connecticut Healthcare System;* [6]*Johns Hopkins University School of Medicine;* [7]*Kansas University Medical Center;* [8]*Segal Trials;* [9]*CI Trials;* [10]*University of California, Riverside*

**Background:** Several lines of evidence suggest that Herpes Simplex Virus type 1 (HSV-1) may contribute to cognitive impairment in schizophrenia. Herpes viruses are enveloped, double stranded DNA viruses that are capable of infecting human CNS resulting in life-long infection with over 40% of the population seropositive for this virus. Valacyclovir is an effective treatment for the suppression of herpes virus infections. A previous small clinical trial (N=24) assessed the efficacy of valacyclovir for cognitive impairment in patients with early phase schizophrenia who were seropositive for HSV-1 (Prasad et al 2013). Results indicate that adjunctive valacyclovir, as compared to placebo, showed improvement in working and visual memory. The current study was undertaken to confirm and extend these findings and determine if HSV-1 seropositive, as compared to those who were HSV-1 seronegative, derived cognitive improvement from adjunctive valacyclovir. We hypothesized that individuals who were HSV-1 positive, but not HSV-1 negative, would demonstrate significant valacyclovir efficacy for cognitive impairment.
**Methods:** An early psychosis network comprised of 12 US sites was established for the valacyclovir trial. Subjects had early phase schizophrenia (within 8 years since psychosis onset) and were randomized 1:1 to a 16-week trial of adjunctive valacyclovir (3 grams/day) or placebo. Assessments included cognitive domains (MATRICS), role functioning (UPSA-B, Q-LES-Q-SF, PSP), psychiatric symptoms (PANSS, NSA-16) and a range of inflammatory markers. The primary outcome was change in working (composite score of Spatial Span and Letter Number span) and visual (Brief Visuospatial Memory Test) as measured by the MATRICS.
**Results:** 170 subjects with early phase schizophrenia were stratified by HSV-1 status and randomized 1:1 to adjunctive valacyclovir or adjunctive placebo. Of those randomized, 74 were HSV-1 seropositive and 96 were seronegative. The valacyclovir vs. placebo groups, respectively, were well matched: age (28.4 vs. 27.5 years), gender (males: 69% vs. 77.9 %), race (white-caucasian: 28.6% vs. 33.7%) and duration of psychosis (3.7 vs. 4.0, years). Baseline working memory scores (Letter-Number Span) were significantly lower in HSV1 positive vs. negative subjects (mean/SD: vs. 35.1/9.0 vs 38.3/11.0, p=0.046). Treatment outcome analyses have only recently commenced and are ongoing. Full results of the cognitive, functioning, symptom and safety measures will be presented at the meeting.
**Discussion:** The current study included 170 patients with early phase schizophrenia randomized to valacyclovir or placebo and stratified by HSV1 sero-status. Baseline working memory scores (Letter-Number Span) were significantly lower in HSV1 positive compared to HSV1 negative subjects. As analyses of treatment effects are ongoing, detailed results of valacyclovir's effects on cognitive domains, symptoms, functioning and safety will be presented at the meeting. Additional research is needed to determine the full therapeutic potential of valacyclovir and other medications targeting herpes viruses in the treatment of schizophrenia.

### 40. NEW INSIGHTS ON THE ROLE OF NEUROINFLAMMATION IN SCHIZOPHRENIA PATHOPHYSIOLOGY FROM POST MORTEM AND ANIMAL STUDIES

Tertia Purves-Tyson
*Neuroscience Research Australia*

**Overall Abstract:** Recent years have witnessed an explosion of clinical and preclinical effort aimed at understanding the involvement of neuroinflammation in schizophrenia (SCZ). The aim of this symposium is to present new and complementary data from human post mortem brain tissue and the rodent maternal immune activation (MIA) model, which together support the involvement of neuroinflammation in SCZ.

Tertia Purves-Tyson will present the first post mortem evidence that neuroinflammation in SCZ extends to the midbrain, a region critical for psychosis and cognitive deficits. She will show that: 1. gene expression of multiple pro-inflammatory cytokines is increased in the post mortem substantia nigra in SCZ compared to controls, in whose brains no such changes are seen; 2. as in the cortex, gene expression changes were found only in a subset of cases (~50%) of the SCZ cohort. She will also explore whether previously identified decreases in dopamine-related transcripts (transporters and receptors) in the substantia nigra in SCZ brains are related to the inflammation status. Ulrike Weber will show in the MIA model that the inflammatory changes identified in the midbrain of patients with SCZ may have a prenatal origin stemming from exposure to inflammation-related environmental insults. Remarkably mimicking the post-mortem data, MIA in mice increased brain pro-inflammatory cytokine gene expression, in not only the prefrontal cortex, but also in the ventral midbrain, and, similarly to humans, only in ~50% of offspring. She will also explore the relationship between changes in immune-related and dopamine-related gene expression in this brain region of MIA offspring. Anthony Vernon will show that MIA exposure on GD15 in rats leads to increased microglia density and soma size in the adult rat striatum and cingulate cortex. Strikingly, chronic haloperidol treatment at clinically comparable doses in adulthood interacts with prenatal MIA exposure, leading to further increases in both microglia density and soma size in both the striatum and frontal cortex. These data suggest adult antipsychotic exposure may increase neuroinflammation in the MIA model. Ina Weiner will report on the effects of subchronic low-dose risperidone treatment (RIS) in adolescence on neuroinflammation in MIA offspring, quantified using radiolabeled [3H]PK11195, a selective TSPO ligand and clinically comparable index of putative microgliosis. Compared to controls, [3H]PK11195 binding was increased in the hippocampus and frontal cortex of adult males and the hippocampus of females, with no changes in adolescence, partially mirroring the results of [11C]PK11195 in-vivo PET in SCZ patients. These increases were prevented in MIA offspring after RIS administration in adolescence, in parallel with prevention of brain volumetric reductions and cognitive deficits. Early intervention with RIS may decrease neuroinflammation and potentially underlie the preventive effects of RIS in the MIA model.

Taken together, these data support the involvement of neuroinflammation in SCZ and MIA model rodents. In particular, they suggest that high inflammatory profile, while distinguishing SCZ/MIA brains from control brains, may characterize only subsets of patients/MIA offspring, or may exist in all individuals but on an on-off basis, implying that preventive and current treatments may interact with neuroinflammation. Indeed, while APDs given to adult symptomatic offspring, interact with neuroinflammation to increase it further, early treatment with APDs in nonsymptomatic offspring prevents neuroinflammation. Evaluation of fluctuations in neuroinflammation over the lifespan and their interactions with treatment effects is a next step.

### 40.1 INFLAMMATORY CYTOKINES ARE ELEVATED IN THE MIDBRAIN IN SCHIZOPHRENIA

Tertia Purves-Tyson*,[1], Debora Rothmond[1], Cynthia Shannon Weickert[1]
[1]*Neuroscience Research Australia*

**Background:** Neuroinflammation is attractive as a candidate mechanism contributing to schizophrenia neuropathology. In ~40% of people with schizophrenia, pro-inflammatory cytokines are elevated in post mortem prefrontal cortex and in peripheral blood of living patients. Dopamine dysregulation contributes to cognitive deficits and psychosis and cytokines can increase dopamine production, yet post mortem midbrain cytokine transcripts have not been examined. We hypothesised that gene expression of inflammatory markers will be elevated in the midbrain of a subset of people that suffered with schizophrenia during their lives.

**Methods:** Pro-inflammatory cytokine mRNAs for interleukin (IL) 1β, IL6, IL6 signal transducer (IL6ST), IL8, 1L18, tumor necrosis factor (TNF) α, SERPINA3, and the microglia marker, allograft inflammatory factor 1 (AIF1), were examined by qPCR in the midbrain from 28 schizophrenia cases and 29 healthy controls. All patients were on antipsychotics at time of death and antipsychotic medication was converted to chlorpromazine (CPZ) equivalents. Inflammatory subgroups were defined using two-step cluster analysis of cytokine mRNAs on the entire cohort. Chi-squared was used to test if the number of individuals in the inflammatory groups differed on the basis of diagnosis. Student's t-tests or ANCOVA were used to detect diagnostic differences and differences between inflammatory/diagnosis subgroups. Student's t-tests were used to compare CPZ equivalent doses in the low and high inflammation schizophrenia groups.

**Results:** SERPINA3, IL1β, IL6 mRNAs were increased by more than 150% and IL6ST mRNA by 17% in the midbrain from schizophrenia patients compared to controls ($F>4.0$, $p<0.0001$–0.05), whilst IL8, IL18 and AIF1 mRNAs were unchanged ($p>0.05$). Cluster analysis revealed 13 individuals as high inflammation and 44 as low inflammation. All 13 individuals in the high inflammatory group were schizophrenia cases and the remaining 15 schizophrenia cases and all the control cases were low inflammation ($\chi2=57.0$, $P<0.0001$, $N=57$). SERPINA3, IL6, IL1β and TNFα mRNAs were all increased in the high inflammation/schizophrenia compared to control and low inflammation/schizophrenia groups ($p<0.002$–0.05). AIF mRNA was not changed by diagnosis, but was increased in the high inflammation/schizophrenia compared to the low inflammation/schizophrenia group ($p=0.015$). The schizophrenia/high inflammation group received higher lifetime, daily and last CPZ equivalent doses ($t(20–26)<-2.7$, $p<0.05$) compared to the schizophrenia/low inflammation group.

**Discussion:** Inflammatory markers were elevated in the midbrain in ~50% of schizophrenia cases, whilst no controls were classified as high inflammation. This data suggests that increases in pro-inflammatory cytokines extend to midbrain regions and may contribute to the neuropathology of the disorder by contributing to dopamine dysregulation. PET studies relate increased microglia activity to at-risk symptom severity in medication naïve people at ultra high risk for schizophrenia, we suggest that the higher dose of antipsychotics in the high inflammation group along with increased microglial marker may indicate that these patients were sicker and thus, required more medication, rather than antipsychotics increasing inflammatory markers. In conclusion, increased cytokine transcripts indicate a neuroinflammatory process in the midbrain in some people with schizophrenia. Future post mortem studies will explore whether previously identified changes in dopamine-related transcripts in the midbrain in schizophrenia are altered according to inflammatory state.

### 40.2 MATERNAL IMMUNE ACTIVATION LEADS TO INCREASED LEVELS OF INFLAMMATORY CYTOKINES IN THE ABSENCE OF OVERT MICROGLIA ANOMALIES IN THE MIDBRAIN

Ulrike Weber-Stadlbauer*[,1], Juliet Richetto[1], Marie Labouesse[2], Urs Meyer[1]
*[1]University of Zurich; [2]College of Physicians and Surgeons, Columbia University/New York State Psychiatry*

**Background:** Inflammatory theories in schizophrenia have gained increasing recognition and acceptance in recent years. The evidence supporting a role of altered inflammatory processes in the etiology and pathophysiology of schizophrenia involves early-life exposure to infectious pathogens or inflammatory stimuli, increased expression of cytokines and other mediators of inflammation in the adult central nervous system (CNS) and periphery, as well as signs of glial anomalies. Given the role of dopaminergic deregulation in the pathophysiology of schizophrenia, inflammatory processes in the midbrain may contribute to dopamine abnormalities in the midbrain and its subcortical and cortical output regions. Here, we tested this hypothesis using an established neurodevelopmental mouse model with relevance to schizophrenia, namely the maternal immune activation (MIA) model.
**Methods:** Pregnant C57BL6/N mice on gestation day 17 were treated with the viral mimetic polyriboinosinic-polyribocytidilic acid (poly(I:C)) or vehicle control solution. We then quantified the gene transcripts of an array of pro-inflammatory cytokines, acute phase proteins, and dopaminergic markers in the midbrain of MIA offspring (N=32) and control offspring (N= 32) at adult age. We also assessed the cell density of microglial cells expressing Iba1 and CD68 by immunohistochemistry to ascertain whether putative inflammatory changes are accompanied by microglia anomalies. Given the large sample sizes, we performed two-step recursive cluster analyses in order to identify possible subgroups of offspring that are characterized by "high" and "low" inflammatory profiles.
**Results:** When considering the entire treatment group, MIA-exposed offspring displayed significantly increased expression of several inflammatory cytokines in the ventral midbrain, including IL-1b ($p < 0.01$), TNF-a ($p < 0.01$) and SERPINA3 ($p < 0.01$). These inflammatory changes occurred in the absence of overt microglia anomalies but were paralleled by changes in dopaminergic markers. The two-step cluster analyses further identified subgroups of MIA-exposed offspring that are characterized by a "high" (41 %, N = 13) and "low" (59 %, N = 19) inflammatory profiles. The "high" inflammatory subgroup of MIA-exposed offspring was defined by marked elevations of SERPINA3, IL-1β, IL-6, and TNFα mRNA levels (all p's < 0.01).
**Discussion:** Maternal immune activation during pregnancy causes persistent signs of inflammation in the offspring's midbrain. In agreement with post-mortem studies in schizophrenia, these inflammatory abnormalities are clearly noticeable in a subgroup of MIA-exposed offspring only. Hence, prenatal immune activation may be one of the factors inducing lasting inflammatory changes relevant to (some cases of) schizophrenia and may contribute to dopaminergic dysfunctions in this disorder.

### 40.3 MATERNAL IMMUNE ACTIVATION AND CHRONIC HALOPERIDOL INTERACT TO INCREASE MICROGLIAL ACTIVATION IN VIVO: DO ANTIPSYCHOTICS INFLAME THE BRAIN?

Marie-Caroline Cotel[1], Romana Polacek[1], Ewelina Lenartowicz[1], Sridhar Natesan[1], Jonathan Cooper[1], Anthony Vernon*[,1]
*[1]Institute of Psychiatry, Psychology and Neuroscience, King's College London*

**Background:** Evidence-based medicine suggests that a subset of schizophrenia is associated with an inflammatory syndrome. To fully harness the potential of novel immunomodulatory therapeutics, it is critical to first determine the impact of antipsychotics on microglial function in vivo. Evidence suggests antipsychotics are anti-inflammatory; however, this is based on in vitro models and non-clinical doses of antipsychotics in vivo. It therefore remains unknown if antipsychotics promote detrimental neuroinflammation or beneficial, homeostatic changes in the brain. To address this question, we explored the effects of chronic haloperidol treatment on microglia in a rat maternal immune activation (MIA) model, representative of schizophrenia pathology.
**Methods:** Pregnant SD rat dams were exposed to poly (I:C) on GD15 (4 mg/kg, i.v.; n=5; POL) to induce MIA, or saline (n=5; CON) as a control. At 4 months of age, male offspring from CON and POL (n=2 per litter), were randomly allocated to treatment with either haloperidol (0.5 mg/kg/d s.c.) or vehicle for 28 days by osmotic minipumps, giving four groups: CON/vehicle; CON/haloperidol; POL/vehicle and POL/haloperidol (all n=10). After 28d treatment, animals were culled and perfused transcardially with 4% PFA. Fixed brain tissues were dissected, cryoprotected and microtome sectioned (1 in 12 series, 40 µm thick). Serial sections were stained for Iba1 as a marker of microglia using an immunoperoxidase protocol. The density and morphology (soma size) of Iba1+ microglia were then assessed in the corpus striatum (CS) and anterior cingulate cortex (ACC) using unbiased stereology. Data were analysed using 2x2 ANOVA in SPSS with main effects of prenatal, postnatal and pre x post-natal interactions.
**Results:** There were significant main effects of prenatal exposure to POL on Iba1+ microglia density in the CS ($F(1,32)=18.09$; $p<0.001$) and the ACC ($F(1,32)=5.04$; $p<0.05$) and for Iba1+ microglia soma sizes (increased) in POL offspring in both the CS ($F(1,32)=88.5$; $p<0.001$) and ACC ($F(1,32)=45.06$; $p<0.001$). There were no main effects of postnatal treatment (vehicle or haloperidol) on Iba1+ microglia density in either the CS or ACC, but there were main effects of postnatal treatment for Iba1+ microglia soma size in both CS ($F(1,32)=17.3$; $p<0.001$) and ACC ($F(1,32)=7.69$; $p<0.01$). Strikingly, there were significant interactions between pre- and post-natal treatments for both Iba1+ density in the CS ($F(1,32)=5.15$; $p<0.05$) and PFC ($F(1,32)=9.43$; $p<0.01$) as well as soma size in the CS ($F(1,32)=11.6$; $p<0.01$) and ACC ($F(1,32)=11.7$; $p<0.01$). Post-hoc testing on this interaction confirmed a significant increase in both Iba1+ density and soma size in poly(I:C)-exposed offspring treated with haloperidol, relative to all other groups in both the CS ($p<0.01$) and ACC ($p<0.01$).
**Discussion:** Our data suggest increased microglial density and activation in the CS and PFC of rats exposed to POL in utero. Haloperidol treatment for 28 days replicating clinically comparable dosing and pharmacokinetics did not affect microglia density in saline-exposed offspring, but increased Iba1+ soma size in both CS and ACC, also suggestive of microglial activation. Strikingly, there were significant interactions between prenatal POL exposure and post-natal haloperidol treatment, leading to significantly increased microglia density and soma size in both CS and ACC. Taken together, these preliminary data suggest adult haloperidol treatment may interact with prenatal immune activation to worsen neuroinflammation.

### 40.4 LOW-DOSE RISPERIDONE TREATMENT IN ADOLESCENCE PREVENTS THE DEVELOPMENT OF NEUROINFLAMMATION IN THE MATERNAL IMMUNE ACTIVATION MODEL

Ina Weiner*[,1], Shani Pery[1], Jasbeer Dhawan[2], Anat Biegon[2], Yael Piontkewitz[1]
*[1]Tel-Aviv University; [2]Stony Brook University School of Medicine*

**Background:** Postnatal consequences of prenatal immune activation mimic a broad spectrum of neuro-psycho-pathological features phenotypic of schizophrenia (SCZ). We previously showed that SCZ-relevant behavioral and brain structural abnormalities emerging in adult offspring of moms exposed to the viral mimic polyI:C, are prevented by treatment with the atypical APD risperidone (RIS) in adolescence, prior to the emergence of structural and behavioral abnormalities. Given the increasing centrality of neuroinflammation in SCZ and its treatment and/or prevention, here we assessed whether adolescent RIS is able to prevent neuroinflammation in the polyI:C offspring.

**Methods:** On gestation day 15, pregnant Wistar rats were injected IV with polyI:C (4 mg/kg/ml) or saline. Pups were weaned on postnatal day (PND) 21. Preventive treatment with RIS (Janssen, Belgium; 0.045 mg/kg) was administered daily on PNDs 34–47. Offspring were sacrificed on PND48, prior to full spectrum of structural and behavioral abnormalities, or on PND90, after the emergence of structural and behavioral abnormalities. Microglial activation was assessed in ten regions (nucleus accumbens, striatum, substantia nigra, frontal, anterior cingulate and occipital cortices, dorsal hippocampus (sub-regions CA1, CA3 and dentate gyrus [DG]) and ventral hippocampus (vHPC), using quantitative [3H]PK11195 autoradiography. Another cohort of offspring underwent behavioral testing and imaging.

**Results:** ANOVAs of [3H]PK11195 binding in offspring sacrificed on PND48 revealed no significant effects of prenatal polyI:C in any of the regions assessed. In adult male offspring, [3H]PK11195 binding was significantly increased in the CA1, CA3 and DG hippocampal subfields as well as in the frontal and occipital cortices, compared to controls. No such increases were observed in polyI:C offspring treated with RIS in adolescence (significant prenatal x preventive treatment interactions, and significant difference in [3H]PK11195 binding between polyI:C-VEH and saline-VEH but not between polyI:C-RIS and saline-VEH offspring in post-hoc analyses, in each of the regions). In females, [3H]PK11195 binding was significantly increased only in the vHPC, occipital cortex, and nucleus accumbens. Such increases were not observed in polyI:C female offspring treated with RIS in adolescence. In a second cohort of offspring, prenatal poly-I:C led to structural abnormalities in the hippocampus, striatum, prefrontal cortex and lateral ventricles, as well to deficits in selective attention, executive function, working memory and social interaction, all of which were prevented by RIS.

**Discussion:** Increased [3H]PK11195 binding in the brains of adult poly-I:C offspring is consistent with increased uptake of [11C]PK11195 in patients with SCZ, measured in-vivo by PET. Microglial activation emerged in adulthood, with no such activation in young (PND48) offspring. Late emergence of microglial activation parallels the developmental course of behavioral and brain structural abnormalities in poly-I:C offspring (Piontkewitz et al, 2011a, 2012a; Piontkewitz et al, 2009), suggesting that these late-emerging abnormalities are linked. The latter is supported by the fact that RIS in adolescence prevented the emergence of behavioral and brain structural abnormalities as well as microgliosis in the adult offspring. These data suggest that prevention of adult microgliosis is one of the mechanisms underlying RIS capacity to prevent polyI:C-induced behavioral and neuroanatomical deficits, however, a causal relationship remains to be established.

### 41. RECONSIDERING THE EVIDENCE FOR CLOZAPINE FOR TREATMENT REFRACTORY SCHIZOPHRENIA

James MacCabe
*Institute of Psychiatry, Psychology and Neuroscience, King's College London*

**Overall Abstract:** The superiority of clozapine for treatment refractory schizophrenia was, until very recently, seen as one of the few unshakeable truths in psychiatry. But the pre-eminence of clozapine has recently been called into question by meta-analyses. What are we to make of the fact that meta-analyses of clinical trials, supposedly the pinnacle of evidence based medicine, fail to show an effect which seems clearly evident to most clinicians, and on which many of our guidelines are based? Have we believed in a fairytale for the past three decades? Or do biases in RCTs and methodological limitations of meta-analyses explain the results? These questions will be discussed by Dan Siskind.

Another way to address the question of efficacy is to examine the pharmaco-epidemiological evidence using population-based registers. Jari Tiihonen will present data from his seminal studies of mortality and readmission rates under clozapine treatment versus other antipsychotics, as well as other data. These data seem to show powerful positive effects of clozapine at the population level. Furthermore, more recent evidence suggests a role for clozapine in reducing rates of violent offending, with new data presented for the first time by Vishal Bhavsar.

Finally, despite clinical guidelines recommending the use of clozapine, the actual rates of clozapine use are much lower than expected, with large regional and international variations. There is evidence that the burden of blood monitoring deters physicians from prescribing clozapine. Yvonne van der Zalm will present new data from a cluster randomised trial testing the efficacy and safety of an intervention to increase rates of clozapine prescribing by employing nurse practitioners trained in the initiation and monitoring of clozapine.

John Kane, author of the first, seminal RCT of clozapine in 1988, will lead the discussion.

### 41.1 WHAT DO META-ANALYSES TELL US ABOUT CLOZAPINE'S EFFICACY AND EFFECTIVENESS FOR TREATMENT REFRACTORY SCHIZOPHRENIA?

Dan Siskind*[,1], Steve Kisely[2], Rachel Land[3], Lara McCartney[4]
*[1]Metro South Addiction and Mental Health Service; [2]University of Queensland, MSAMHS; [3]University of Queensland; [4]University of Melbourne*

**Background:** Clozapine has long been considered the gold standard antipsychotic for treatment refractory schizophrenia (TRS). There have been a number of recent meta-analyses of efficacy of clozapine on psychotic symptoms and effectiveness in reducing hospitalisations that have sparked debate on the role of clozapine.

**Methods:** Current literature regarding the efficacy of clozapine for TRS, including pair-wise and network meta-analyses of RCTs with reported outcomes of total psychotic symptoms, positive symptoms and negative symptoms were reviewed. We also examined the results of a meta-analysis of the effectiveness of clozapine on reducing hospitalisations based in RCTs and observational studies.

**Results:** Two recent meta-analyses: Samara et al (2016), a network meta-analysis in JAMA Psychiatry; and Siskind et al (2016) a pairwise meta-analysis in BJPsych, found similar equivocal results for total psychotic symptoms. However, Siskind et al (2016) found clozapine to be superior to other anti-psychotics for positive symptoms. Factors influencing the difference in results included pair-wise vs network methodology and sensitivity analyses of pharmaceutical industry support. Of note, only 40% of people with TRS responded to clozapine. Clozapine's effectiveness for reducing hospitalisations was significant, with a relative risk of 0.74 (95%CI 0.69–0.80).

**Discussion:** There are a lack of recent non-industry funded randomised control trials of clozapine compared to SGAs, which hinders an equivocal statement about the superiority of clozapine for total psychotic symptoms. However, there is evidence to suggest that clozapine is superior to other antipsychotics, including SGAs, for positive symptoms. In terms of effectiveness, initiation of clozapine can reduce the proportion of people hospitalised and reduce bed days. Use of clozapine needs to be balanced against its adverse drug reaction profile. There remains a need for more effective treatments for TRS, and biomarkers to identify TRS.

### 41.2 WHAT DOES EPIDEMIOLOGICAL DATA TELL US ABOUT CLOZAPINE'S EFFECTIVENESS?

Jari Tiihonen*[,1]
[1]*Karolinska Institutet*

**Background:** The patients included in RCTs represent a small atypical minority of the entire patient population, as up to 80–90% of patients are excluded because of mental or physical comorbidity, suicidal or antisocial behaviour, or substance abuse. Concerning clozapine, this means that those most severely ill patients having the greatest potential benefit from clozapine treatment are excluded from RCTs. Another major limitation of RCTs is that when very important but relatively infrequent phenomena, such as suicide or death is studied, exclusion of high risk patients and insufficient statistical power prevent obtaining statistically significant findings.
**Methods:** Observational studies can overcome these obstacles by using nation-wide electronic databases of hospitalization, mortality, and filled prescriptions. However, the main problem with these observational studies is selection bias. Although the most important covariates could be adjusted in the statistical analysis, there always remains residual confounding associated with the personal characteristics of each patient. One way to overcome this problem is to use within-individual analysis, in which each individual is his or her own control. In this approach, the exposure periods of each individual are compared with the non-exposure periods of the same individual.
**Results:** This far, 3 large observational studies using traditional between-subject analyses have found that when compared with other oral antipsychotics, clozapine is associated with the best outcome concerning risk of re-hospitalization, and 4 large cohort studies have shown that clozapine is associated with the lowest mortality. The only cohort study this far using within-individual analyses showed that in a nation-wide cohort of 29,823 patients, clozapine was associated with the lowest risk of treatment failure (defined as psychiatric re-hospitalization, suicide attempt, discontinuation or switch to other medication, or death).
**Discussion:** A large body of observational studies shows that clozapine has better real-world effectiveness than any other oral antipsychotic treatment.

### 41.3 COULD CLOZAPINE REDUCE VIOLENT OFFENDING?

Vishal Bhavsar*[,1], Kyriaki Kosidou[2], Nicola Orsini[2], Linnea Widman[2], James MacCabe[1], Christina Dalman[2]
[1]*Institute of Psychiatry, Psychology and Neuroscience, King's College London;* [2]*Karolinska Institutet, Vishal Bhavsar, King's College London, Institute of Psychiatry*

**Background:** Clozapine treatment may have beneficial effects on behavioural outcomes in psychotic disorders, including violent offending. Although clozapine and other antipsychotics have been linked to lower levels of violent behaviour, these have been primarily in small selected samples, and population-based estimates have been limited and imprecise.
**Methods:** This study was a within-person cohort study based on linked prescription, hospitalization, and sociodemographic registers. We assessed the effect of clozapine treatment on the rate of violent and non-violent offending in the whole of Sweden, taking account of time-changing sociodemographic characteristics and the combination of violent and non-violent offences within individual convictions.
**Results:** In a group of people treated with clozapine for psychotic disorders, violent offences were much less common during treatment than before. Effects on non-violent offences were smaller in magnitude, and lost precision on adjustments. There was a trend for the effects of antipsychotic treatment to increase with increasing age at initiation. Smaller but similar effects were observed for olanzapine. Clozapine rate reductions for violent offending were twice as strong for those with a history of alcohol-use disorders, compared to those without, RR for alcohol use disorders.
**Discussion:** In patients with psychotic disorders, clozapine treatment is associated with a lower rate of violent offending compared to olanzapine. Clozapine might reduce offending through a direct effect on psychotic symptoms, or indirectly through changes in lifestyle, including use of alcohol.

### 41.4 DEPLOYMENT OF DEDICATED NURSING STAFF TO STIMULATE THE INITIATION OF CLOZAPINE. A CLUSTER-RANDOMIZED TRIAL

Yvonne Van der Zalm*[,1], Raphael Schulte[2], Jan Bogers[3], Machteld Marcelis[4], Iris Sommer[5], Jean-Paul Selten[6]
[1]*Rivierduinen Psychiatric Institute;* [2]*Mental Health Service Noord-Holland Noord, Dutch Clozapine Collaboration Group;* [3]*Rivierduinen Centre for Mental Health / Dutch Clozapine Collaboration Group;* [4]*Maastricht University Medical Center, Institute for Mental Health Care Eindhoven (GGzE);* [5]*University Medical Center Groningen;* [6]*Maastricht University / Rivierduinen Mental Health Care Institute*

**Background:** For patients with refractory schizophrenia, clozapine is the drug of first choice. However, many refractory patients never receive this drug. The underutilization of clozapine may be caused by the labour-intensive white blood cell monitoring during the first months and the concerns about the safety of outpatient clozapine initiation. A recent survey concluded that professionals "perceived the presence of dedicated staff to arrange and monitor the initiation of clozapine in outpatients as the factor that would enable the use of clozapine most". We examined whether the presence of such staff in Dutch teams for ambulatory care makes a difference. The primary objective is to examine whether clozapine monitoring by a Nurse Practitioner (NP) is at least as safe as monitoring by a physician. The secondary objective is to examine whether physicians are more likely to prescribe clozapine if they can delegate the monitoring tasks to a NP.
**Methods:** In this cluster-randomized trial, 23 Dutch ambulatory care teams were randomized into 2 conditions: (A) coordination of clozapine monitoring by a Nurse Practitioner, versus (B) Treatment As Usual: coordination of clozapine monitoring by the responsible physician (usually a psychiatrist). We followed the teams for 15 months, during which period we counted the numbers of patients who started with clozapine. We assessed the safety of the clozapine monitoring by measuring the number of weekly lab exams performed during the first 18 weeks of treatment and counting serious adverse events (SAE). It is important to note that the staff of teams remained blind to the secondary research question.
**Results:** Of the 2643 patients with a diagnosis of non-affective psychotic disorder, 66 patients started using clozapine during the follow-up, 48 in condition A and 18 in condition B (RR: 2.14, 95% CI: 1.24–3.70; p=.005). The provisional results showed no significant differences between conditions A and B in the mean number of lab exams performed. In condition A, 65% of the mandatory lab exams were carried out compared to 60% in condition B. No agranulocytosis or other SAE occurred in Conditions A or B.
**Discussion:** Physicians prescribed over 2 times more often clozapine to patients when they could delegate the white blood cell monitoring to a NP. Clozapine-monitoring by an NP appears to be just as safe as monitoring by a physician. These results strongly support the idea that the presence of dedicated staff to arrange and monitor the initiation of clozapine enables the use of this drug.

### 42. METABOLISM AND CO-MORBIDITIES IN PSYCHOTIC DISORDERS

Matej Oresic
*University of Turku*

**Overall Abstract:** Schizophrenia is associated with a reduced life expectancy of 15–20 years due to a high prevalence of cardiovascular disease and metabolic syndrome. Unhealthy lifestyles and pharmacological side effects have been suggested to be a major cause of excess mortality rates in patients with psychotic disorders. However, abnormal glucose homeostasis, hyperinsulinemia and accumulation of visceral fat are already detected in drug-naïve first episode psychosis (FEP) patients, independently of obesity.

The aim of this symposium is to present the latest research on the theme of metabolic co-morbidities in psychotic disorders. Preliminary and published data from the symposium speakers suggests that FEP is associated with altered composition of gut microbiota, inflammation and lipid dysregulation including changes in the endocannabinoid system in the central nervous system. The studies presented in this symposium may offer new insights into the gut-brain axis in psychotic disorders as well as provide new evidence on the role of lipids as a potential underlying link between the aetiology of psychosis and the associated metabolic disturbances.

All four speakers are principal investigators in the European FP7 project METSY, which aims to identify and evaluate multi-modal peripheral and neuroimaging markers that can predict and monitor psychotic and metabolic symptoms, with specific focus on metabolic co-morbidities in psychotic disorders.

Oliver Howes will first introduce the topic, based on his recent meta-analysis (JAMA Psychiatry 2017; 74; 261–269) as well as present his recent research on neuroimaging of the endocannabinoid system in FEP.

Jaana Suvisaari will present her recent research on gut microbiome and inflammation in FEP. The findings so far suggest that FEP patients have specifically altered gut microbiome composition and increased levels of inflammation.

Tuulia Hyötyläinen will introduce the field of metabolomics in psychosis research and discuss the latest findings from at-risk mental state individuals and FEP patients. Recently published findings suggest that FEP patients who later rapidly gain weight are characterised by increased markers of liver fat at the baseline.

Jarmo Hietala will present neuroimaging studies of the endocannabinoid system using PET and the [18F]FMPEP-d2 tracer, a CB1R radioligand. These studies suggest a major sex difference in the brain CB1 receptor system as well as clear evidence for a dysregulated endocannabinoid system in first-episode psychosis.

Given the endocannabinoid system in the periphery promotes the development of fatty liver, the presented work together suggests that the endocannabinoid system may be the underlying link between the psychosis and the associated metabolic disturbances. This intriguing possibility with potential diagnostic and therapeutic implications will be discussed by the presenters. The discussant Matej Oresic is the coordinator of the METSY project.

### 42.1 BODY AND MIND: CARDIO-METABOLIC AND IMMUNE FUNCTION IN FIRST EPISODE PSYCHOSIS AND COMPARISON WITH CENTRAL NEUROFUNCTIONAL MEASURES

Oliver Howes*[,1], Toby Pillinger[2], Katherine Beck[3], Enrico D'Ambrosio[2]
*[1]MRC London Institute of Medical Sciences and King's College London; [2]Institute of Psychiatry, Psychology & Neuroscience, King's College; [3]King's College London, MRC Clinical Sciences Centre*

**Background:** People with schizophrenia die on average 15–20 years earlier than the general population, and the mortality gap has grown in recent years. Non-CNS, particularly cardio-metabolic, causes account for the majority of this premature mortality. Abnormalities in the cardio-metabolic and immune system are well established in patients with schizophrenia, but it is unknown if abnormalities are present in people at risk of psychosis and at illness onset prior to antipsychotic treatment, and how they compare to neurofunctional measures. To address this, we conducted a series of meta-analyses and meta-regressions to determine the magnitude and consistency of findings and influence of antipsychotic treatment, exercise and other factors across cardio-metabolic parameters at onset of psychosis.

**Methods:** We conducted a meta-analysis of peripheral gluco-regulatory, immune and lipid measures and comparison with brain neurofunctional outcomes (including N-acetyl aspartate, gray matter volume and auditory P300 measures) in studies of clinical high risk samples and first episode psychosis, focusing on drug naïve patients. The entire PubMed, EMBase and PsychInfo databases were searched to identify relevant studies. Then we conducted a meta-review statistical comparison of cardio-metabolic and immune function with neurofunctional measures.

**Results:** 35 studies (>1500 patients and controls) were included in the meta-analyses. Interleukin-6 ($g=2.2$, $p=0.013$), and TNF-alpha ($g=0.94$, $p<0.01$) levels and fasting insulin and post-challenge glucose levels were elevated with moderate-large effect sizes ($g=0.4$, $p=0.01$; $g=0.6$, $p=0.007$ respectively) and cholesterol and low density lipoprotein levels were reduced ($g=-0.2$, $p=0.005$, and $g=-0.22$, $p=0.001$ respectively), whilst triglyceride levels were increased ($g=0.14$, $p<0.05$). These findings remained significant in drug naïve patients and after adjusting for the influence of a number of potential confounders (including body mass, exercise levels, smoking). N-acetyl aspartate levels in frontal cortex and auditory P300 amplitude ($g=0.83$, $p<0.0001$) were significantly altered in first episode patients relative to controls. The median effect sizes for cardiometabolic dysfunction ($g=0.41$) was comparable to that of the neurofunctional measures ($g=0.42$, $p>0.3$). Moreover, the median effect size for immune alterations ($g=1.3$) was significantly greater than that for neurofunctional measures ($p=0.002$).

**Discussion:** We demonstrate that effect sizes for markers of cardio-metabolic and immune disturbance are comparable in magnitude to those for markers of neurofunctional CNS disturbances from the onset of psychosis, suggesting schizophrenia involves multiple organ systems from illness onset. The three main pathoetiological models by which CNS and non-CNS abnormalities may co-occur in schizophrenia will be discussed. The shared genetic and environmental risk architecture between schizophrenia and cardiometabolic disorders suggests common aspects of pathoetiology.

### 42.2 INFLAMMATION AND GUT MICROBIOME IN FIRST-EPISODE PSYCHOSIS

Jaana Suvisaari*[,1], Outi Mantere[2], Jaakko Keinänen[1], Tuula Kieseppä[3], Maria Saarela[4], Robert Yolken[5], Jarno Honkanen[6]
*[1]National Institute for Health and Welfare; [2]McGill University; [3]Helsinki University Hospital; [4]VTT Technical Research Centre of Finland; [5]Johns Hopkins University; [6]University of Helsinki*

**Background:** Patients with first-onset psychosis have evidence of impaired glucose tolerance, but otherwise are metabolically healthy when traditional cardiovascular risk markers are used. After antipsychotic treatment is started, there is rapid weight gain and emergence of dyslipidemias. Weight gain and development of abdominal obesity is accompanied by worsening chronic low-grade inflammation. Activation of innate immunity is often present at the onset of disease. One unexplored mechanism possibly contributing to these problems is altered gut microbiota.

**Methods:** The Helsinki Early Psychosis Study recruited 97 patients with first-episode psychosis and 62 controls into a longitudinal study. Here, data on longitudinal changes in inflammation, weight gain and abdominal obesity during the first year of treatment in patients with first-episode psychosis is presented and compared with matched healthy controls. Three time points (baseline, 2 months, 12 months) are available for patients and two (baseline, 12 month) for controls. The possible contribution of different antipsychotics will be explored, and whether patients who were no longer using antipsychotics at the one-year follow-up have less problems in these measures. First results regarding the gut microbiome will be presented (Schwarz et al. 2017), and the possible contribution of gut microbiota to inflammation and weight gain in first-episode psychosis explored.

**Results:** Our previous findings from a subset of the study sample found most marked changes in innate immunity chemokines (Mäntylä et al. 2015), whereas full longitudinal data on 38 cyto- and chemokines will be available at the SIRS congress. As a preliminary result on the longitudinal course of inflammation, high-sensitivity C-reactive protein showed a significant increase during the first year of treatment in patients (median baseline 0.65 mg/l, 2 months 0.79 mg/l and 12 months 1.68 mg/l). Data on PBMC gene expression will also be presented, revealing notable differences related to different antipsychotic use.

**Discussion:** The findings will be discussed in the context of to what extent they may reflect underlying disease mechanisms and environmental contributions, including gut microbiota alterations, and to what extent inflammation is a secondary phenomenon related to antipsychotic use and weight gain.

**References:**

1) Mäntylä T, Mantere O, Raij TT, Kieseppä T, Laitinen H, Leiviskä J, Torniainen M, Tuominen L, Vaarala O, Suvisaari J. Altered activation of innate immunity associates with white matter volume and diffusion in first-episode psychosis. PLoS One. 2015 May 13;10(5):e0125112.

2) Schwarz E, Maukonen J, Hyytiäinen T, Kieseppä T, Orešič M, Sabunciyan S, Mantere O, Saarela M, Yolken R, Suvisaari J. Analysis of microbiota in first episode psychosis identifies preliminary associations with symptom severity and treatment response. Schizophr Res. 2017 [Epub ahead of print]

### 42.3 METABOLOMICS APPROACHES TO STUDY METABOLIC CO-MORBIDITIES IN PSYCHOTIC DISORDERS

Tuulia Hyötyläinen*[,1], Tommi Suvitaival[2], Dawei Geng[1], Päivi Pöhö[3], Ismo Mattila[2], Jaana Suvisaari[4], Matej Oresic[5]

*[1]Örebro University; [2]Steno Diabetes Center Copenhagen; [3]University of Helsinki; [4]National Institute for Health and Welfare; [5]University of Turku*

**Background:** Psychotic patients are at high risk for developing obesity, metabolic syndrome and type 2 diabetes. These metabolic co-morbidities are hypothesized to be related to both treatment side-effects as well as to metabolic changes occurring during the psychosis. Earlier metabolomics studies have shown that blood metabolite levels are predictive of insulin resistance and type 2 diabetes in the general population as well as sensitive to the effects of antipsychotics. Here we aimed to identify the metabolite profiles predicting future weight gain and other metabolic abnormalities in psychotic patients.

**Methods:** We applied metabolomics to investigate serum metabolite profiles in a prospective study setting in 36 first-episode psychosis patients during the first year of the antipsychotic treatment and 19 controls. Two analytical platforms with broad analytical coverage were used. Molecular lipids were analysed by ultra-high performance liquid chromatography coupled to time-of-flight mass spectrometry (UHPLC_QTOFMS) and polar metabolites were analysed by two-dimensional gas chromatography coupled to TOFMS (GCxGC-TOFMS). Ongoing prospective metabolomics studies ae focusing on the subjects in at-risk mental state.

**Results:** While corroborating several earlier findings when comparing cases and controls and the effects of the antipsychotic medication, we also found that prospective weight gain in psychotic patients was associated with increased levels of triacylglycerols with low carbon number and double-bond count at baseline and independent of obesity, that is, lipids known to be associated with increased liver fat.

**Discussion:** The first-episode psychotic patients who rapidly gain weight in the follow-up have early metabolic disturbances which are associated with insulin resistance and fatty liver. Our studies suggest that metabolite profiles may be used to identify the psychotic patients most vulnerable to develop metabolic co-morbidities, and may point to a pharmacological approach to counteract the antipsychotic-induced weight gain.

### 42.4 THE ENDOCANNABINOID SYSTEM IN FIRST-EPISODE PSYCHOSIS

Jarmo Hietala*[,1]

*[1]University of Turku*

**Background:** The endocannabinoid (EC) system comprises of fatty acid neurotransmitters such as anandamide and 2-acylglycerol, at least two specific cannabinoid (CB) receptor subtypes and enzyme machinery for synthesis and degradation of ECs. The EC system regulates a wide array of functions in central nervous system and peripheral tissues such as brain development, plasticity, reward and stress sensitivity as well as metabolic functions a such as energy/lipid metabolism including liver function and insulin resistance (1). One of the aims of the METSY project (www.metsy.eu) is to study the central and peripheral EC system in FEPs and their involvement in brain mechanisms in psychosis as well as related metabolic co-morbidities. The cannabinoid CB1 receptor (CB1R) is the most abundant cannabinoid receptor subtype in the human brain and is the predominant mediator of various EC effects. In this first series of METSY experiments we report in vivo brain CB1R binding characteristics in patients with first-episode psychosis (FEP) and controls.

**Methods:** Brain CB1R availability was measured using [18F]-FMPEP-d2 and 3D ECAT HRRT positron emission tomography (PET) using distribution volume (DVt) as a proxy for CB1R availability. Our studies in healthy volunteers revealed a marked sex difference in [18F]-FMPEP DVt with males having higher CB1R availability. The overall CB1R sex difference was widespread but also regionally different with most significant effects in the occipital cortex. Therefore, subsequent studies focused first on male patients with FEP and controls (n=18).

**Results:** The results indicate a significant and relatively widespread decrease in CB1R DVt in patients with FEP in fronto-temporal regions, putamen, posterior cingulate as well as parietal regions with large effect sizes. Largely similar results were seen in an independent sample of medication-free male patients with FEP (n=17, the other METSY PET site, King's College, London, UK) (2). Those studies used another validated CB1R tracer, the [11C]-MePPEP. The reduced CB1R in patients was also state-dependent as [18F]-FMPEP DVt correlated negatively with psychotic symptoms (BPRS) in a highly significant manner.

**Discussion:** Epidemiological studies have convincingly shown that cannabis use is associated with an increased risk of both psychotic symptoms and schizophrenia-like psychoses. We now provide direct in vivo evidence for a robust and partly state-dependent dysregulation of the endocannabinoid system in first-episode psychosis. This is well in line with a recent PET study with CB1R tracer, [11C]-OMAR in patients with schizophrenia (3). Based on previous experimental data the reduced CB1R availability is indicative of an increased endocannabinoid drive in psychosis.

**Acknowledgements:** Funding from the EU's 7th Framework Programme; METSY - Neuroimaging platform for characterization of metabolic co-morbidities in psychotic disorders (no. 602478).

**References:**

1) Eriksson O et al The Cannabinoid Receptor-1 Is an Imaging Biomarker of Brown Adipose Tissue. J Nucl Med. 2015 Dec;56(12):1937–41.

2) METSY: Endocannabinoid pathways in early psychosis – brain imaging studies (Hietala J, Howes O, Laurikainen H, Borgan F and the METSY PET Imaging group).

3) Ranganathan M et al Reduced Brain Cannabinoid Receptor Availability in Schizophrenia. Biol Psychiatry. 2016 Jun 15;79(12):997–1005.

### 43. BUILDING ON GENETICS AND PATHOPHYSIOLOGY OF SCHIZOPHRENIA TO GUIDE DISCOVERY OF NEW TREATMENTS

P. Jeffrey Conn
*Vanderbilt University Medical Center*

**Overall Abstract:** Current treatments for schizophrenia (SCZ) are partially effective in treating positive symptoms, but many patients are refractory to available medications and they are ineffective in the treatment of negative symptoms and cognitive impairment. There is a tremendous need to develop novel approaches to the treatment of SCZ that have broader efficacy and fewer adverse effects than existing dopamine D2 receptor and mixed D2/5-HT2A antagonists. To achieve fundamental breakthroughs that provide efficacy for refractory patients and across multiple symptom domains, it will be critical to rely on rigorous studies in SCZ patients that guide development of novel treatment approaches. Major recent advances in the genetics, imaging, and molecular pathophysiology of SCZ point to new potential treatment strategies, and may guide patient selection and outcome measures. Speakers in this symposium will summarize examples of translational studies that may offer novel therapeutic approaches that have the potential to fundamentally change the standard of care for SCZ patients. Jeff Conn (Vanderbilt Univ., USA) will summarize recent genetic studies that point to loss of function mutations in two novel G protein-coupled receptors in SCZ patients, and the discovery of novel positive allosteric modulators for these receptors that are providing a strong proof of concept for advancing selective agents to clinical evaluation in patients, including those that bear these specific mutations. Brian Dean (Univ. Melbourne, Australia) will then review clinical studies showing that a subgroup of SCZ patients show a marked loss of cortical muscarinic M1 receptors and translational studies suggesting that selective activators of M1 receptors could provide benefits in treating this disorder. In addition, he will summarize changes in molecular cytoarchitecture in the cortex of subjects with SCZ that provide important insights in considering M1 activators as a treatment strategy. Clare Beasley (Univ. British Columbia, Canada), will review mounting evidence from postmortem tissue that implicates immune dysregulation and the complement system in SCZ and the potential utility of complement inhibitors in the treatment of this disorder. Finally, Anissa Abi-Dargham (Stony Brook Univ., USA) will discuss recent advances in the use of molecular imaging for discovery of patients with alterations in specific targets that may guide clinical development. Specifically, imaging studies suggest that nigro-striatal dopaminergic projections may play a more important role relative to mesolimbic dopaminergic projections than previously appreciated, and suggest a blunting of dopamine release in extra-striatal areas. These observations can be linked to clinical treatment response or particular domains of pathology and fit nicely with new data suggesting that activation of targets outlined in the first presentation selectively inhibit dopaminergic signaling in nigrostriatal but not in other dopaminergic pathways. In summary, this panel will highlight new therapeutic leads derived from novel insights gathered through imaging, genetic and molecular studies of SCZ patients.

### 43.1 GENETIC INSIGHTS LEAD TO DISCOVERY OF SELECTIVE ACTIVATORS OF MGLU1 AND MGLU3 METABOTROPIC GLUTAMATE RECEPTORS AS POTENTIAL TREATMENTS FOR SCHIZOPHRENIA

P. Jeffrey Conn*[,1], Samantha Yohn[2], Branden Stansley[2], Dan Foster[2], Hyekyung Plumley[2], Craig Lindsley[2]
*[1]Vanderbilt University Medical Center; [2]Vanderbilt University*

**Background:** A large number of clinical and preclinical studies suggest that dysfunction at synapses for the excitatory neurotransmitter glutamate may play a critical role in the pathophysiological changes that underlie each of the major symptom clusters observed in schizophrenia patients. Interestingly, recent genetic studies identified multiple nonsynonymous single nucleotide polymorphisms (SNPs) in the human genes encoding two specific subtypes of metabotropic glutamate (mGlu) receptors that are associated with schizophrenia. These include GRM1 and GRM3, the genes encoding for the mGlu1 and mGlu3 receptor subtypes respectively. Furthermore, postmortem studies suggest that expression of these mGlu receptor subtypes is altered brains of schizophrenia patients compared to controls. Mutations in GRM1 were identified a range of schizophrenia patients, whereas SNPs in the human gene encoding mGlu3 (GRM3) are selectively associated with poor performance on cognitive tests that are dependent on function of the prefrontal cortex (PFC) and hippocampus. These studies raise the possibility that disrupted signaling of mGlu1 and/or mGlu3 could contribute to the symptoms of schizophrenia and that selective modulators of these receptors could provide a novel approach to treatment of this disorder.

**Methods:** Wild-type and mutant forms of mGlu receptors were expressed in cell lines and used for discovery and optimization of highly selective positive allosteric modulators (PAMs) of mGlu1 and mGlu3. Optimized mGlu1 and mGlu3 PAMs were then used along with mouse genetic studies to evaluate the roles of these receptors in specific basal ganglia and forebrain circuits that have been implicated in schizophrenia. Finally, these compounds were used in animal models to assess potential efficacy in rodent models that are relevant for reducing positive, negative, and cognitive symptoms that are observed in schizophrenia patients.

**Results:** GRM1 mutations associated with schizophrenia were found to reduce mGlu1 signaling, suggesting that loss of function of this receptor could contribute to symptoms associated with schizophrenia. Furthermore, we found that highly selective mGlu1 PAMs reverse deficits in mGlu1 signaling observed in these mutant receptors, induced a profound reduction in dopamine release in striatal areas implicated in schizophrenia, and have robust antipsychotic-like effects that are mediated by localized inhibition of dopamine release in striatum. In contrast to existing antipsychotic medications, selective mGlu1 PAMs also improve motivation and reduce anhedonia in animal models. Interestingly, selective mGlu3 PAMs have multiple effects in the prefrontal cortex and hippocampus that would be expected to improve cognitive function. Consistent with this, highly selective mGlu3 PAMs have robust cognition-enhancing effects in rodent models that are relevant for the cognitive deficits observed in schizophrenia patients.

**Discussion:** These studies provide exciting new evidence that highly selective activators of two glutamate receptors identified in human genetic studies have potential utility in treatment of positive (mGLu1), negative (mGlu1), and cognitive (mGlu3) symptoms of schizophrenia patients. Furthermore, the novel mGlu1 and mGlu3 PAMs discovered in these studies provide excellent drug leads for further optimization and ultimate clinical testing.

### 43.2 MUSCARINIC M1 RECEPTORS: INVOLVEMENT IN THE PATHOPHYSIOLOGY AND TREATMENT OF SCHIZOPHRENIA

Brian Dean*[,1], Shaun Hopper[1], Elizabeth Scarr[2]
*[1]Florey Institute for Neuroscience and Mental Health; [2]University of Melbourne*

**Background:** Evidence from postmortem CNS studies and a neuroimaging study suggest that, compared to controls, there are low levels of muscarinic receptors in a number of CNS regions from subjects with schizophrenia. Current data suggests the muscarinic M1 receptor is lower in the cortex of subjects with schizophrenia but other muscarinic receptors may be decreased in sub-cortical regions such as the striatum and hippocampus. In addition, it has been reported that ~25% of subjects with schizophrenia can be divided into a distinct sub-group because they have a marked decrease in cortical muscarinic M1 receptors (muscarinic receptor deficit schizophrenia (MRDS)). These findings have become of clinical significance because proof of principal data shows that treating subjects with schizophrenia with drugs that activate the muscarinic M1 receptor is effective in lessening the symptoms associated with the disorder.
**Methods:** Published and unpublished data will be reviewed to challenge the hypothesis that drugs that activate the muscarinic M1 receptor will be useful in treating schizophrenia.
**Results:** A proof of clinical trial has shown that treating subjects with treatment resistant schizophrenia with the muscarinic M1 and 4 receptor agonist, xanomeline, improves positive and negative symptoms as well as cognitive deficits. Moreover, it has more recently been reported that giving xanomeline on a transdermal patch with a peripheral muscarinic receptor antagonist can lessen the unwanted side effects of the drug to that of placebo. Relevant to these data is the finding that there is a sub-group of subjects with MRDS as the absence of cortical muscarinic M1 receptors in these receptors may make these subjects resistant to treatment with muscarinic M1 receptor agonists. However, novel studies using postmortem CNS from subjects with MRDS and non-MRDS has shown that whilst subjects with MRDS will likely be resistant to muscarinic M1 receptor orthosteric agonists (oxotremorine-M) they will, at least partially, respond to muscarinic M1 receptor allosteric agonists (AC-42) or positive allosteric modulators (BQCA).
**Discussion:** Muscarinic receptor agonism appears to be a promising new treatment for schizophrenia. However, some subjects with MRDS may only respond to activation of the allosteric site on the muscarinic M1 receptor. Evidence from a neuroimaging study suggests subjects with MRDS can be identified whilst living. Hence, establishing the muscarinic receptor status of subjects involved in trials of muscarinic M1 receptor agonists may help in explaining varying levels of treatment responsiveness in subjects with schizophrenia. These conclusions, being directed by data from studies using postmortem CNS, reflect the need for drug discovery and delivery to be based on a growing understanding of the pathophysiology(ies) of schizophrenia.

### 43.3 COMPLEMENT DYSREGULATION IN SCHIZOPHRENIA: IMPLICATIONS FOR POTENTIAL TREATMENT STRATEGIES

Clare Beasley*,[1]
*[1]University of British Columbia*

**Background:** Dysregulation of the immune system and inflammation likely play a role in the development or course of schizophrenia, at least in a sub-population of patients. In particular, recent evidence has implicated the complement system in this disorder. The complement system is a key effector of innate immunity, mediating elimination of pathogens and debris via initiation of phagocytosis, inflammation and cell lysis. Intriguingly, early complement components also participate in synaptic pruning and plasticity. Further evaluation of the role of the complement system in schizophrenia may reveal novel treatment strategies.
**Methods:** Investigations of the complement system in blood and postmortem brain tissue in schizophrenia will be reviewed. Furthermore, the relationship between blood and brain complement levels and measures of central and peripheral inflammation, genotype and synaptic density will be explored. Finally, the potential utility of anti-complement therapies in the treatment of schizophrenia will be discussed.
**Results:** Recent genome-wide association studies have revealed an association with genetic markers within the major histocompatibility complex locus in schizophrenia, with this association suggested to reflect diversity in complement component 4 (C4) genes. Consistent with genetic data, higher complement hemolytic activity has been reported in this disorder, while our studies in postmortem brain tissue have revealed increased expression of several complement components. Notably, C4 expression is impacted by C4 genetic architecture.
**Discussion:** Overall, data suggests a role for the complement system in schizophrenia. Increased complement expression may be indicative of an inflammatory response. However, given that early complement components have also been implicated in synaptic pruning and circuit remodeling, disturbances in complement activity may also contribute to synaptic deficits previously identified in this disorder. Anti-complement therapies are currently available, while the complement system provides numerous additional options for future drug development. Further research is required to elucidate the potential utility of anti-inflammatory therapies, including those targeting the complement system, in the treatment of schizophrenia, and to identify which patients may benefit most from this strategy and when treatment would be most effective.

### 43.4 THE ROLE OF MOLECULAR IMAGING IN GUIDING DRUG DEVELOPMENT

Anissa Abi-Dargham*,[1], Lawrence Kegeles[2], Mark Slifstein[3]
*[1]Stony Brook University School of Medicine; [2]Columbia University & New York State Psychiatric Institute; [3]Stony Brook University*

**Background:** Molecular imaging with Positron Emission Tomography (PET) has the unique capability of examining molecules in the brains of human subjects in real time. This has been well exploited in the study of dopaminergic transmission in schizophrenia. The results have on one hand confirmed some predictions, and on the other hand they have brought up unexpected findings, that have the potential to affect drug development in a major way.
**Methods:** PET studies of dopamine release, synthesis, storage, receptors and transporters across labs and different experiments will be reviewed to extract findings that differ from preconceived notions. These have the capability of re-focusing drug development based on new evidence.
**Results:** Expected findings that PET studies have confirmed are those of striatal dopaminergic excess, measured as enhanced presynaptic release capacity and presynaptic enzymatic synthesis capacity. Also expected was hypodopaminergic transmission in the dorso-lateral prefrontal cortex.
The unexpected findings from PET have been as follows: 1) the striatal excess is most predominant in the associative striatum, rather than the limbic striatum. 2) hypodopaminergia is generalized to all extrastriatal areas of the brain
These observations of opposite presynaptic dopaminergic dysfunction between striatal and extrastriatal regions suggest that the striatal excess may not be driven by activity levels of midbrain DA cells but may result from abnormal striatal local regulation of presynaptic DA release, affecting specifically the associative striatum, region which shows largest magnitude of excess DA. Furthermore alterations in dopaminergic signaling in opposing directions may have a dual effect on learning across functional domains by weakening learning from salient or relevant signals and reinforcing stimulus independent signals, thus explaining different symptom domains.
**Discussion:** More work is needed to understand the cellular mechanisms of dopaminergic dysfunction in schizophrenia. The evidence presented here, while limited, highlights the challenges of drug development in the absence of real in vivo information from patients, and the need for multidisciplinary cross talk to address these challenges.

### O1. Oral Session: Biomarkers

### O1.1 ALTERED COMPLEMENT PATHWAY PROTEIN EXPRESSION IS ASSOCIATED WITH PSYCHOTIC EXPERIENCES AT AGE 11 WHICH PERSIST AT AGE 18

David Cotter*[,1], Jane English[2], Melanie Foecking[1], Mary Cannon[1], Bart Rutten[3], Stanley Zammit[4], Glyn Lewis[5], Sophie Sabhwerwal[1], Lorna Lopez[2], Aoife O'Gorman[1], Gerard Cagney[6]
*[1]Royal College of Surgeons in Ireland; [2]Royal College of Surgeons in Ireland, Beaumont Hospital; [3]Maastricht University Medical Centre; [4]Cardiff University; [5]University College London; [6]Proteome Research Centre, University College Dublin*

**Background:** The identification of early biomarkers of psychotic disorder is important because early treatment is associated with improved outcome. We have previously shown that altered complement and coagulation pathway associated proteins are associated pathway with psychotic disorder at age 18. In the current study we test the hypothesis that altered complement pathway proteins are associated with persisting psychotic experiences from age 11 to age 18.
**Methods:** The Avon Longitudinal Study of Parents and Children (ALSPAC) is a prospective general population cohort, and a rich resource of demographic, environmental, and clinical data on the individuals involved. We studied a subsample of the cohort who participated in psychiatric assessment interviews at age 11 and 18, and who provided plasma samples at age 11. Semi-targeted proteomic profiling was used to specifically assess the complement pathway proteins in age 11 children who experienced psychotic experiences (but not disorder) at age 11 and age 18 (n=39) compared to age 11 children who only experienced psychotic experiences at age 11.
**Results:** 11 of 34 proteins assessed were significantly differentially expressed at $p<0.05$ and of these 8 remained significant following correction for multiple comparisons. Protein changes were in keeping with increased proteins expression of most complement pathway proteins. Several protein changes represented specific replications of the changes observed in age 11 samples prior to psychotic disorder at age 18, namely increased plasminogen, complement factor H, and complement factor 1r.
**Discussion:** Our findings implicate the blood complement system in the persistence of psychotic experiences from age 11 to age 18. Considering that psychotic experiences are predictive of many psychiatric disorders our findings implicate the complement system not just in psychotic disorders, but more broadly in the vulnerability to a range of adult psychiatric disorders.

### O1.2 PERIPHERAL INFLAMMATORY MARKERS ARE PREDICTIVE OF CLINICAL CHARACTERISTICS AND OUTCOME IN PSYCHOSIS

Graham Blackman*[,1], Thomas Pollak[1], Megan Pritchard[1], John Hanrahan[2], Anthony Dalrymple[2], Amalia Velarde[3], Vivienne Curtis[1], Robert Stewart[1], Anthony David[1]
*[1]Institute of Psychiatry, Psychology and Neuroscience, King's College London; [2]King's College London; [3]Ramón y Cajal University Hospital*

**Background:** Dysregulation of the immune system represents an important vulnerability factor for schizophrenia. A rise in peripheral inflammatory markers has previously been demonstrated in psychosis; however, its significance remains uncertain. Characterising this relationship aids our understanding of the role of immunological factors in psychosis, as well as potentially identifying candidate biomarkers to guide diagnosis, treatment and prognosis. Whilst specialized inflammatory marker assays have been found to be associated with outcome and treatment, these tests are not typically available in clinical practice. We sought to establish whether routine inflammatory markers are associated with clinical characteristics and outcomes in patients with schizophrenia and related disorders.
**Methods:** A multi-site cohort study of patients admitted to an acute psychiatric ward between January 2013 and December 2016 within a large Mental Health Trust was undertaken. Cases were identified from an electronic database containing full clinical records. Inclusion criteria were patients aged 18 and 65 years with a discharge diagnosis of schizophrenia, or related disorder and a routine blood test within 3 days of admission. Exclusion criteria were diagnoses of drug-induced psychosis, organic brain disorder, or admission during the perinatal period. Pro-inflammatory (white blood cell total and differential count, C-reactive protein) and anti-inflammatory markers (albumin) recorded during the admission were extracted. Clinical characteristics were based upon the Health of the Nation Outcome Scale, a 12-item clinician rated tool contemporaneously rated at admission and discharge.
**Results:** A total of 968 patients met the inclusion criteria. 309 patients were female and mean age was 38 years. The most frequent ethnicities were White, Black African, Black Other and Black Caribbean and the commonest diagnoses were schizophrenia, unspecified non-organic psychosis and schizoaffective disorder. Mean interval from admission to admission blood test was 0.8 days.
Patients with affective psychosis had a significantly higher white cell count, monocyte count and lymphocyte count than patients with non-affective psychosis on admission. Furthermore, among patients with affective psychosis, a partial correlation controlling for age, body mass index, blood pressure, physical health and smoking status found a significant association between symptom severity and monocyte count. There was a highly significant association between both neutrophil count and white cell count with hallucinatory symptoms. There was also a highly significant positive association between C-reactive protein and self-injurious behaviour, replicating recently published findings in smaller samples. There was a significant reduction in overall psychiatric symptoms over the course of admission, which was significantly associated with admission monocyte count. A partial correlation found white cell count and neutrophil count at admission were associated with reductions in hallucinatory symptoms. Eosinophil count was significantly associated with admission length.
**Discussion:** In a large cohort of patients admitted due to psychotic disorder, pro-inflammatory markers were associated with affective psychosis and overall symptom severity, and predicted admission length and reduction in symptom severity. The study supports an association between immune dysregulation and psychosis. Furthermore, the study highlights the role of routinely and inexpensively measured peripheral inflammatory markers as potential diagnostic and prognostic biomarkers in psychosis.

### O1.3. A COMPUTATIONAL TRIAL-BY-TRIAL EEG ANALYSIS OF HIERARCHICAL PRECISION-WEIGHTED PREDICTION ERRORS

Sara Tomiello*[,1], Dario Schöbi[2], Lilian Weber[2], Helene Haker[2], Iglesias Sandra[2], Klaas Enno Stephan[2]
*[1]ETH Zurich; [2]University of Zurich & ETH Zurich*

**Background:** Action optimisation relies on learning about past decisions and on accumulated knowledge about the stability of the environment. In Bayesian models of learning, belief updating is informed by multiple, hierarchically related, precision-weighted prediction errors (pwPEs). Recent work suggests that hierarchically different pwPEs may be encoded by specific neurotransmitters such as dopamine (DA) and acetylcholine (ACh). Abnormal dopaminergic and cholinergic modulation of N-methyl-D-aspartate (NMDA) receptors plays a central role in the dysconnection hypothesis, which considers impaired synaptic plasticity a central mechanisms in the pathophysiology of schizophrenia.
**Methods:** To probe the dichotomy between DA and ACh and to investigate timing parameters of pwPEs, we tested 74 healthy male volunteers performing a probabilistic reward associative learning task in which the contingency between cues and rewards changed over 160 trials between 0.8 and 0.2.
Furthermore, the current study employed pharmacological interventions (amisulpride / biperiden / placebo) and genetic analyses (COMT and ChAT) to probe DA and ACh modulation of these computational quantities. The study was double-blind and between-subject.
We inferred, from subject-specific behavioural data, a low-level choice PE about the reward outcome, a high-level PE about the probability of the outcome as well as the respective precision-weights (uncertainties) and used them, in a trial-by-trial analysis, to explain electroencephalogram (EEG) signals (64 channels). Behavioural data was modelled implementing three versions of the Hierarchical Gaussian Filter (HGF), a Rescorla-Wagner model, and a Sutton model with a dynamic learning rate. The computational trajectories of the winning model were used as regressors in single-subject trial-by-trial GLM analyses at the sensor level. The resulting parameter estimates were entered into 2nd-level ANOVAs. The reported results were family-wise error corrected at the peak-level ($p<0.05$) across the whole brain and time window (outcome phase: 0 - 500ms).
**Results:** A three-level HGF best explained the data and was used to compute the computational regressors for EEG analyses. We found a significant interaction between pharmacology and COMT for the high-level precision-weight (uncertainty). Specifically:

- At 276 ms after outcome presentation the difference between Met/Met and Val/Met was more positive for amisulpride than for biperiden over occipital electrodes.
- At 274ms and 278 ms after outcome presentation the difference between Met/Met and Val/Met was more negative over fronto-temporal electrodes for amisulpride than for placebo, and for amisulpride than for biperiden, respectively.

No significant results were detected for the other computational quantities or for the ChAT gene.
**Discussion:** The differential effects of pharmacology on the processing of high-level precision-weight (uncertainty) were modulated by the DA-related gene COMT.
Previous results linked high-level PEs to the cholinergic basal forebrain. One possible explanation for the current results is that high-level computational quantities are represented in cholinergic regions, which in turn are influenced by dopaminergic projections. In order to disentangle dopaminergic and cholinergic effects on synaptic plasticity further analyses will concentrate on biophysical models (e.g. DCM). This may prove useful in detecting pathophysiological subgroups and might therefore be of high relevance in a clinical setting.

### O1.4. CEREBROSPINAL FLUID FINDINGS IN TWINS WITH PSYCHOTIC SYMPTOMS – NOVEL FINDINGS AND FUTURE PROSPECTS

Viktoria Johansson*[,1]
[1]*Karolinska Institutet*

**Background:** Schizophrenia and bipolar disorder are severe mental disorders with unknown etiology. Our research group has studied biomarkers in the cerebrospinal fluid (CSF) of twins with schizophrenia and bipolar disorder to be able to determine the genetic and environmental influences. In brain disorders, CSF is the most appropriate substrate to study as it may reflect the brain biochemistry better than blood. In this presentation I aim to give an overview of our findings and their relation to psychotic disorders. I intend to present our most recent preliminary finding and to discuss future prospects.
**Methods:** We studied CSF-markers from a cohort of 50 monozygotic (MZ) and dizygotic (DZ) twins with schizophrenia or bipolar disorder. The twins have gone through diagnostic assessments and have been extensively phenotyped with questionnaires, symptom scales for psychiatric symptoms as well as neuropsychological testing. We have analyzed monoamines, microglia-, neurodegenerative-, kynurenine-, and inflammatory markers using immunoassays and high-performance liquid chromatography techniques. We have also studied microscopic structures with scanning electron microscopy.
**Results:** One of our main findings was that soluble cluster of differentiation 14 protein (sCD14) was higher in twins with schizophrenia or bipolar disorder compared to their not affected co-twins. A later analysis showed that the difference within the discordant twin-pairs was higher in the DZ twin pairs ($\beta=28697.1$, $t=3.20$, $p=0.024$) compared with the MZ twin pairs ($\beta=5577.5$, $t=2.10$, $p=0.081$) suggesting that genetic components along with unique environmental effects have an influence on the higher sCD14 levels in patients with schizophrenia and bipolar disorder. We also found that sCD14 was higher in those patients with more psychotic symptoms.
In our study on microscopic structures in CSF we found that the structures were prevalent not only in the patients with schizophrenia and bipolar disorder but also in their not affected co-twins. The finding suggests that genetic factors may be partly involved in the formation of the structures.
**Discussion:** We have analyzed inflammatory and neurodegerative markers in the CSF of twins with psychotic disorders to be able to study genetic and environmental influences. Our results indicate that sCD14 may have an influence on microglia activation in psychosis. We have continued with analyses on the correlations between all the markers, the monoamine metabolites and associations with symptoms and cognitive ability and the preliminary results from these analyses will be presented.
To conclude CSF analyses for biomarkers in twins may result in extended knowledge regarding the genetic and environmental relationships. Our unique twin data gives us the possibility to study CSF-markers in relation to psychiatric symptoms and cognitive measures. For future studies it would be of interest to assemble twin-samples from several research groups to be able to study research questions regarding gene and environment interactions.

### O1.5. ICAM-1 IS INCREASED IN BRAIN AND PERIPHERAL LEVELS OF SOLUBLE ICAM-1 IS RELATED TO COGNITIVE DEFICITS IN SCHIZOPHRENIA

Thomas Weickert*[,1], Helen Cai[2], Maryanne O'Donnell[3], Ryan Balzan[4], Ruth Wells[5], Dennis Liu[6], Cherrie Galletly[6], Cynthia Shannon Weickert[5]
[1]*University of New South Wales, NeuRA;* [2]*University of New South Wales;* [3]*Prince of Wales Hospital;* [4]*Flinders University;* [5]*Neuroscience Research Australia;* [6]*University of Adelaide*

**Background:** Schizophrenia is a disabling and often unremitting mental illness with an unknown cause that is characterized by heterogeneity in psychotic symptom presentation, cognitive deficits and treatment response. There is accumulating evidence for the role of inflammation in the etiology of schizophrenia. Inflammatory markers have been identified in the brains and peripheral blood of chronically ill patients with schizophrenia and in first episode patients and these markers have been associated with structural and functional brain abnormalities and cognitive deficits. Intercellular adhesion molecule 1 (ICAM-1) is a transmembrane protein expressed on endothelial cells which binds to leukocyte receptors that promotes transmigration of white blood cells into tissue. While peripheral inflammatory markers are altered in people with schizophrenia relative to controls, the extent to which ICAM-1 is elevated in the brains of people with schizophrenia and peripheral levels of soluble ICAM-1 (sICAM-1) is increased in relation to cognitive impairment in schizophrenia is unknown.
**Methods:** In a post-mortem cohort, 8 mRNAs relating to BBB function and 3 immune cell markers were measured by qPCR in the prefrontal cortex of

37 people with schizophrenia and 37 matched controls. In an independent living cohort, sICAM-1 was measured with a Luminex immunoassay from the plasma of 78 chronically ill patients with schizophrenia (all receiving antipsychotic medication) and 73 healthy controls. All participants from the living cohort received the following cognitive assessments: Wechsler Adult Intelligence Scale – 3rd edition to assess current IQ, Controlled Oral Word Association Test verbal fluency and Wechsler Memory Scale-Revised to assess verbal memory. Pearson's or Spearman's correlations were performed between cognitive measures and sICAM1 levels as appropriate in schizophrenia patients and healthy controls.

**Results:** ICAM-1 was elevated in the brains of people with schizophrenia relative to controls and CD163+ perivascular macrophages were found in the parenchyma. Peripheral sICAM1 was elevated by 29.2% in people with schizophrenia compared to healthy controls, $t(140) = -3.988$, $p < 0.01$. In people with schizophrenia, sICAM1 was inversely correlated with immediate verbal memory (r=-0.30, p=0.01), delayed verbal memory (rho=-0.29, p=0.01), verbal abstract reasoning (r=-0.23, p=0.05), and processing speed (rho=-0.28, p=0.02). In healthy controls, sICAM1 levels were inversely correlated with verbal fluency (r=-0.27, p=0.03) and processing speed (rho=-0.26, p=0.03).

**Discussion:** The brain endothelium of people with schizophrenia can attract more immune cells via increased ICAM-1. sICAM-1, a cleavage product of ICAM which enables white blood cell migration into tissue (including brain) is significantly elevated in peripheral blood of patients with schizophrenia. sICAM-1 is associated with poor verbal memory, reasoning and processing speed in people with schizophrenia and accounts for variation in cognition of healthy controls. This suggests that increased inflammatory processes, measured in blood, may reflect brain related cognitive deficits that are the hallmark of schizophrenia. Anti-inflammatory treatments may reverse cognitive impairment in schizophrenia.

### O1.6. INCREASED COMPLEMENT FACTORS C3 AND C4 IN SCHIZOPHRENIA AND THE EARLY STAGES OF PSYCHOSIS: IMPLICATIONS FOR CLINICAL SYMPTOMATOLOGY AND CORTICAL THICKNESS

Vanessa Cropley*[,1], Liliana Laskaris[1], Andrew Zalesky[1], Cynthia Shannon Weickert[2], Maria Di Biase[1], Gursharan Chana[3], Bernhard Baune[4], Chad Bousman[5], Barnaby Nelson[6], Patrick D McGorry[6], Ian Everall[7], Christos Pantelis[1]

*[1]Melbourne Neuropsychiatry Centre, The University of Melbourne; [2]Neuroscience Research Australia; [3]Centre for Neural Engineering, The University of Melbourne; [4]University of Adelaide; [5]University of Calgary; [6]Orygen, The National Centre of Excellence in Youth Mental Health; [7]Institute of Psychiatry, Psychology & Neuroscience, King's College London*

**Background:** The complement system - a key component of the innate immune system, has been proposed to contribute to the pathogenesis of schizophrenia. Recently, complement C4 was associated with increased risk of schizophrenia, and in a mouse model, developmentally-timed synaptic pruning. These observations have led to proposals that abnormal activation of the complement system might contribute to the development of schizophrenia by disrupting synaptic pruning during key developmental periods. However, despite renewed interest in the complement system in schizophrenia it remains unclear whether peripheral complement levels differ in cases compared to controls, change over the course of illness and whether they are associated with current symptomatology and brain cortical thickness. This study aimed to: i) investigate whether peripheral complement protein levels are altered at different stages of illness, and ii) identify patterns among complement protein levels that predict clinical symptoms and grey matter thickness across the cortex.

**Methods:** Complement factors C1q, C3 and C4 were quantified in 183 participants [n=83 Healthy Controls (HC), n=10 Ultra-High Risk (UHR) for psychosis, n=40 First Episode Psychosis (FEP), n=50 Chronic schizophrenia] using Multiplex ELISA. Permutation-based t-tests were used to assess between-group differences in complement protein levels at each of the three illness stages, relative to age- and gender-matched healthy controls. Canonical correlation analysis was used to identify patterns of complement protein levels that correlated with clinical symptoms and regional thickness across the cortex.

**Results:** C3 and C4 were significantly increased in FEP and UHR patients, whereas only C4 was significantly increased in chronic patients. A molecular pattern of increased C4 and decreased C3 was associated with positive and negative symptom severity in the pooled patient sample. Increased C4 levels alone, or decreased C3 levels alone, did not correlate with symptom severity as strongly as the pattern of increased C4 in combination with decreased C3. Preliminary canonical correlation analyses revealed that, in healthy controls, a molecular pattern characterised by increased C3 and decreased C4 was associated with relatively thinner paracentral, inferior parietal and inferior temporal cortices, but relatively thicker insular, in the left hemisphere. In the pooled patient group, a trend for increased C3 in combination with decreased C1q was associated with relatively thinner left lateral occipital cortex and pars orbitalis but relatively thicker pars opercularis and precuneus.

**Discussion:** Our findings indicate that peripheral complement concentration is particularly increased early and preceding psychosis and its imbalance may be associated with symptom severity and variation in regional grey matter thickness across the cortex.

### O1.7. PROTEOMIC ANALYSIS OF BLOOD BASED SAMPLES FROM THE OPTIMISE (OPTIMIZATION OF TREATMENT AND MANAGEMENT OF SCHIZOPHRENIA IN EUROPE) STUDY POINT TOWARDS COMPLEMENT PATHWAY PROTEIN CHANGES

Melanie Föcking*[,1], Thomas Pollak[2], Patrick Dicker[1], Gerard Cagney[3], Inge Winter[4], Rene Kahn[4], Philip McGuire[2], David Cotter[8]

*[1]Royal College of Surgeons in Ireland; [2]Institute of Psychiatry, Psychology and Neuroscience, King's College London; [3]Proteome Research Centre, University College Dublin; [4]University Medical Centre Utrecht; [8]Royal College of Surgeons in Ireland, Beaumont Hospital*

**Background:** The OPTiMiSE (Optimization of Treatment and Management of Schizophrenia in Europe) trial may help in the identification of predictors of treatment response. Medication naïve patients with first episode schizophrenia or schizophreniform disorder were enrolled in the study and treated open-label for a four-week period with amisulpride. PANSS ratings were undertaken at baseline and following the four-week treatment. 30 non-remitters (as defined by the Andreasen criteria) with the worst change in PANSS scores and the 30 remitters with the best change in PANSS scores were selected to represent good and poor outcome groups.

**Methods:** We compared proteomic markers in serum collected prior to treatment in 30 patients who subsequently showed a good response to amisulpride ("responders", and 30 patients who did not show a good response ("non-responders"). Serum samples were depleted using High Performance Liquid Chromatography (HPLC) attached to a MARS column to remove the 14 most abundant plasma proteins (albumin, IgG, antitrypsin, IgA, transferrin, haptoglobin, fibrinogen, alpha2-macroglobulin, alpha1-acid glycoprotein, IgM, apolipoprotein AI, apolipoprotein AII, complement C3, and transthyretin). The groups were matched for ethnicity, gender and age.

50 µg from each sample were reduced, alkylated, tryptically digested, then zip-tipped to concentrate and purify. Samples were then run for a 90-min gradient on a Thermo Scientific Q- Exactive Hybrid Quadrupole-Orbitrap Mass Spectrometer. Raw MS data were processed by MaxQuant software and searched against the human Uniprot database, for label free quantitation of peptides and proteins. False discovery rates (FDR) were set at 1% for both peptide and protein levels in target/decoy to minimize false positives. The match between runs feature was utilized.

**Results:** Four hundred and sixty-four protein identifications were obtained. Samples were excluded where >30% of proteins were missing, and we used imputation, where the missingness depends upon a threshold of detection. This left 228 proteins for analysis. Of these, 21 were significantly different between responders and non-responders (p<0.05), one was FDR positive, one at trend FDR level. Pathway analysis (KEGG, David NIH) of the significant proteins determined "complement and coagulation cascades" to be the top pathway affected with six proteins from the list assigned to the pathway. These were CFI, C4A, C6, F9, VWF and SERPING1, all found to be up-regulated and with p-values ranging from 0.002 to 0.044. C6 is a constituent of the membrane attack complex (MAC) that plays a key role in the innate and adaptive immune response, while CFI belongs to the alternative pathway, C4A belongs to the classical pathway and F9 and VWF play roles in the intrinsic coagulation pathway.

**Discussion:** These data complement results by Sekar et al., implicating excessive complement activity in the development of schizophrenia. Our data identifies the complement proteins in treatment response and this is also consistent with our previous findings of up-regulation of the complement pathway among those at risk of future psychotic experiences.

### O1.8. STRESS-INDUCED AMYGDALA HYPERACTIVITY LEADS TO INTERNEURON LOSS AND SCHIZOPHRENIA-LIKE PATHOLOGY IN A DEVELOPMENTAL DISRUPTION MODEL OF SCHIZOPHRENIA

Anthony Grace*[,1], Felipe Gomes[1], Xiyu Zhu[1]
[1]*University of Pittsburgh*

**Background:** Studies of the MAM rat model of schizophrenia show that rats exhibit higher anxiety levels, greater response to stress, and amygdala hyperactivity prepubertally before the emergence of the hyperdopaminergic state later in life. Furthermore, administration of diazepam prepubertally prevents this transition. These data suggest that MAM may predispose to psychosis via increased response to environmental stressors. If this is accurate, then one would predict that sufficiently strong stressors administered to normal rats during the critical period prepubertally would lead to psychosis-like state in the adult.

**Methods:** Rats are exposed to either 3 sessions of 1-hour restraint, 25 footshocks daily for 10 days, or both stressors delivered either at PD 31–40 (prepuberty) or PD 65–74 (adult) in intact rats and rats with prelimbic PFC (plPFC) lesions at PD25. Rats were tested for amphetamine locomotion, novel object recognition (NOR), and VTA DA neuron firing. Valproic acid (VPA) was administered to adults 5 days before and during the combined stressors to reopen the critical period. DREADD activation of the amygdala was also evaluated.

**Results:** While individual stressors prepubertally augmented anxiety and disrupted NOR in the adult, only the combined stressors resulted in amphetamine hyperlocomotion and increased DA neuron population activity similar to the MAM rats. plPFC lesions enabled the footshock alone to lead to anxiety, NOR deficits, and the hyperdopaminergic state in the adult. Given that stress is known to impact parvalbumin (PV) interneurons in the hippocampus when administered during the critical period in prepubertal rats, we tested the impact of opening the critical period in the adult rats with VPA. Normal rats given combined stressors at PD 65–74 showed attenuated DA neuron activity similar to that observed in depression models. However, administration of VPA caused the combined stressors to lead to the schizophrenia phenotype. This was accompanied by hippocampal hyperactivity driven by an overactive amygdala, since DREADD activation of the amygdala produced similar effects.

**Discussion:** These data suggest that factors that increase the response to environmental stressors during the prepubertal critical period lead to activation of the stress-activated amygdala-hippocampal pathway and PV interneuron loss, which leads to the hyperdopaminergic state in the adult thought to underlie psychosis. Furthermore, re-opening the critical period in the adult makes the adult sensitive to stress-induced psychosis. This suggests that controlling the impact of stress early in life in susceptible individuals may be an effective means to circumvent the transition to psychosis later in life.

### O2. Oral Session: Cognition

### O2.1. FIRST EPISODE PSYCHOSIS PATIENTS ACROSS EUROPE DIFFER IN INTELLECTUAL QUOTIENT (IQ) AND EXPOSURE TO ENVIRONMENTAL HAZARDS

Laura Ferraro*[,1], Marta Di Forti[2], Caterina La Cascia[1], Giada Tripoli[2], Lucia Sideli[1], Ilaria Tarricone[3], Diego Quattrone[2], Robin Murray[2], Daniele La Barbera[1]
[1]*University of Palermo;* [2]*Institute of Psychiatry, Psychology & Neuroscience, King's College London;* [3]*Bologna University*

**Background:** Children who later develop Schizophrenia on average are more likely to present with lower IQ; this has been considered evidence for the neurodevelopmental theory of schizophrenia. Though, recent studies have shown that first episode psychosis patients with a history of cannabis use have significantly higher premorbid and current IQ compared to those who never used it. This suggests that abnormal early neurodevelopment does not explain the aetiology of all cases of Schizophrenia, leaving space to environmental hazards.

The present study aims to: investigate differences in IQ, as a marker of neurodevelopment, and in exposure to environmental risk factors in a large sample first episode psychosis patients recruited across five different European countries, in comparison with their respective control groups.

**Methods:** We analysed data on IQ, socio-demographics and cannabis use from FEP=705 (51.1 % males) and healthy controls=1.034 (48.9 % males), as part of the European network of national schizophrenia networks studying European Gene-Environment-Interaction (EUGEI) study. Patients met ICD-10 criteria for psychosis, ascertained by using OPCRIT (McGuffin et al., 1991).

The CEQmv(Di Forti et al., 2009) further modified for the EUGEI study, was used to collect data on cannabis use. We used ANOVAs where IQ was used as the outcome variable and case/control status and Country were respectively entered as independent predictors, along with other predictors.

**Results:** Case-control status (F (1,1.484)=133.1, p<0.001) and Country (F (4, 1.484)=32.1, p<0.001) resulted in interaction in predicting IQ after controlling for gender, age, ethnicity, education, occupation relationship and living status. That means, being a case and being from France (mean IQ=73.4), Spain (mean IQ=74.6) and Italy (mean IQ=75.4) was associated with the lowest IQ (F (4,1.484)=3.7, p=0.004), compared with cases from UK (mean IQ=87.1) and Holland (mean IQ=85.5). Among controls the pattern was similar but not significant.

We then grouped countries as North- (UK and Holland) and South – Europe (Italy, Spain, France) and we compared the presence of the main risk factors between the two groups. Both patients and controls from the northern part of Europe, were more likely to be from other ethnicities (chi2(2)=93.3, p<0.001) and living alone (chi2(2)=39.6, p<0.001) than patients and controls from the southern part, who were, for instance, more likely to be married (chi2(2)=34.1, p=0.007). There were no differences in education, nor in gender distribution between cases from the north and from the south of Europe and cases from the south, but not controls, were more likely to be employed than patients from the North (chi2(1)=19.1, p<0.001). A significant difference emerged in patterns of cannabis use, that resulted more dangerous in the northern part, where cases, but not controls, were more likely to have used cannabis (chi2 (1)=18.2, p<0.001) on a daily basis (chi2(2)=17.6, p<0.001) with a higher concentration in THC (chi2 (2)=43.8, p<0.001) and before their 15 years (chi2 (2)=20.8, p<0.001), compared to patients from the south.

**Discussion:** Our findings on the higher IQ reported in the first episode psychosis patients from northern Europe sites might indicate this as a group with less neurodevelopmental abnormalities and more likely to have developed Psychosis because of adverse social environment and more harmful pattern of cannabis use, compared to patients from the southern countries.

### O2.2. CHILDHOOD ADVERSITIES AND PSYCHOTIC SYMPTOMS: THE POTENTIAL MEDIATING OR MODERATING ROLE OF NEUROCOGNITION AND SOCIAL COGNITION

Giovanni Mansueto*[,1], Koen Schruers[2], Fiammetta Cosci[3], Jim van Os[4], GROUP Investigators[2]

*[1]School for Mental Health and Neuroscience Maastricht University; [2]Maastricht University Medical Center, School for Mental Health & Neuroscience; [3]Florence University; [4]University of Maastricht*

**Background:** Childhood abuse and neglect are risk factors for psychotic symptoms. The exposure to early adversities might lead to poor functioning in the area of neuro/social cognition, which in turn is associated to psychosis. This study aimed to explore the mediating and moderating role of neuro/social cognition in the relationship between childhood abuse, neglect, and psychotic symptoms.

**Methods:** 1.119 psychotic patients were enrolled from university hospitals in the Netherlands and Belgium (i.e., Groningen, Amsterdam, Maastricht, Utrecht, Leuven) and their affiliated mental healthcare institutions. Childhood adversities were evaluated with the Dutch version of the Childhood Trauma Questionnaire. Psychotic symptoms were assessed with the Positive and Negative Syndrome Scale, using the five-factor model proposed by van der Gaag et al. (2006). Verbal learning-memory, attention-vigilance, working memory, information processing speed, reasoning-problem solving were evaluated via the Word Learning Task, the Continuous Performance Test, the Wechsler Adult Intelligence Scale 3rd. Mentalizing abilities were evaluated as a measure of social cognition using the Hinting Task. Mann-Whitney U test were performed to compare patients with to without early adversities. Correlation was used to ensure that independent variables (childhood neglect or childhood abuse), dependent variables (psychotic symptoms), and hypothesized mediator (M)/moderator (MR) (i.e., neurocognition or social cognition) were associated. Mediation and moderation analyses were run according to Baron and Kenny's criteria. A bootstrapping procedure was used to assess indirect effects. Mediation and moderation models were adjusted for age, sex, and lifetime cannabis use as a priori potential cofounders

**Results:** Patients with childhood neglect, compared with those without childhood neglect, showed more severe psychotic symptoms (p<.01) and lower scores on retention rate, and attention (p<.01).

Patients with childhood abuse showed more severe psychotic symptoms (p<.01) than those without childhood abuse, while no statistically significant differences were found for neurocognition (p=.87) and social cognition (p=.77). Mentalizing abilities partially mediate the relationship between childhood neglect and negative symptoms (Total Effect: 1.01, BCa: .27-.75; Indirect effect: .17, BCa: .02-.40), disorganization (Total Effect: 1.29, BCa .46-1.95; Indirect effect: .23, BCa: .03-.49), and excitement (Total Effect: .76, BCa: .32-1.21; Indirect effect: .05, BCa: .009-.14). The association between childhood neglect and psychotic symptoms is neither mediated nor moderated by neurocognition. Neurocognition and social cognition neither mediate nor moderate the association between childhood abuse and psychotic symptoms.

**Discussion:** The aetiological role of neurocognition in the association between childhood adversities and psychosis seems unlikely. Mentalizing abilities could be an aetio-pathogenetic pathway linking childhood neglect to negative symptoms, disorganization, and excitement.

### O2.3. AUTOMATED ANALYSIS OF RECENT-ONSET AND PRODROMAL SCHIZOPHRENIA

Guillermo Cecchi*[,1], Cheryl Corcoran[2]

*[1]IBM Research; [2]Icahn School of Medicine at Mount Sinai*

**Background:** Psychosis has significant effects on language, to the extent that its disturbance is one of the principal components of diagnosis and prognosis. In particular, two features of language seem to be prominently affected: discourse coherence, observed in patients as derailment (or tangentiality), and discourse richness, observed as poverty of speech. Using automated linguistic analysis on baseline interviews, we have shown in a previous study that it is possible to predict with high accuracy of conversion to psychosis (100%) among a cohort of clinical high-risk youth, by quantifying the subjects' semantic coherence and syntactic complexity as proxies for derailment and poverty of speech, respectively.[1] In the present study, we seek to explore to what extent the prodromal prediction model can discriminate recent-onset schizophrenia patients from matching controls, with the intent of understanding how the prodromal-onset transition is reflected in language.

**Methods:** Eighteen recent-onset schizophrenic patients and twelve matching controls had baseline interviews, using an open-ended protocol previously introduced.[2] Using automated analysis, transcripts of interviews were evaluated for semantic and syntactic features predicting psychosis onset in an independent cohort. These features were then used to discriminate between patients and controls, applying the same classifier that predicted conversion to psychosis, namely converts laying outside the convex hull of non-converts. Additionally, we compared the discrimination power of this approach against alternative models including alternative linguistic features (e.g. metaphoricity[3]) and protocols (e.g. short prompts[4]).

**Results:** The convex hull of the controls subjects misclassifies only one of the patient samples, a result that amounts to 95% true positive rate. Surrogating by label randomization and accounting for false and positive negative rates results in a balanced accuracy of 80%, which is comparable to those obtained with alternative automated models, which range from 70% to 85%. Moreover, the present cohort is clearly separable from both converts and non-converts in the CHR cohort when projected in the feature space.

**Discussion:** The automated features optimized for prediction of psychotic onset convey highly significant information regarding the discrimination between recent-episode patients and controls. The directionality of effects, however, is not obviously derived from that observed in the prodromal-onset transition. This preliminary study provides the basis for a larger study to better understand language disturbances across these cohorts.

**References:**

1. Bedi, G., et al. npj Schizophrenia 1 (2015): 15030.
2. Ben-David, S., et al. Psychiatric Services 65.12 (2014): 1499–1501.
3. Gutiérrez, E.D., et al. Proceedings of the 2017 Conference on Empirical Methods in Natural Language Processing. 2017.
4. Mota, N.B., et al. PloS one 7.4 (2012): e34928.

### O2.4. THE MISSING PIECE IN THE PUZZLE: COGNITIVE DECLINE IN SCHIZOPHRENIA AND BIPOLAR PATIENTS AFTER THE FIRST EPISODE

Abraham Reichenberg*[,1], Jolanta Zanelli[2], Josephine Mollon[2], Sven Sandin[1], Paola Dazzan[2], Craig Morgan[2], Izabela Pilecka[2], Tiago Reis Marques[3], Gillian Doody[4], Kevin Morgan[8], Paul Fearon[5], Anthony David[2], Peter Jones[6], Robin Murray[2]
*[1]Icahn School of Medicine at Mount Sinai; [2]Institute of Psychiatry, Psychology and Neuroscience, King's College London; [3]Psychiatric Imaging Group, MRC Clinical Sciences Centre, Institute of Clinical Science, Imperial College London; [4]University of Nottingham; [8]University of Westminster; [5]St. Patrick's University Hospital and Trinity College Dublin; [6]University of Cambridge*

**Background:** Schizophrenia is associated with a severe cognitive impairment. While it is widely believed that cognitive deficits in schizophrenia remain stable after illness onset, few studies have comprehensively examined longer-term cognitive change from soon after the first episode through to late adulthood. We examined whether schizophrenia patients experience cognitive decline following the first episode, whether this decline is generalized or confined to individual neuropsychological functions, and whether decline is specific to schizophrenia.
**Methods:** Participants were from a population-based, case-control study of patients with first-episode psychosis followed prospectively up to 10 years post first admission. A neuropsychological battery was administered at index presentation and at follow-up to patients with a diagnosis of schizophrenia and bipolar disorder or mania (n=83), as well as to healthy comparison subjects (n=103).
**Results:** The schizophrenia group exhibited declines in IQ and individual neuropsychological functions tapping verbal knowledge, executive function, language and visual memory (group by time interaction p values<0.01). The age when progression of deficits where observed differed between functions. There was no decline in verbal memory and processing speed. These functions showed large deficits at the first episode, which remained static thereafter. Cognitive decline in IQ, verbal knowledge and language was not specific to schizophrenia and was also apparent in the bipolar/mania group (p values<0.05). Healthy individuals with low IQ, on the other hand, showed no evidence of decline, suggesting that a progressive course of cognitive impairment is specific to psychosis.
**Discussion:** Schizophrenia and bipolar/mania patients experience cognitive decline after onset of psychosis. Cognitive remediation efforts should target individual functions during specific time periods.

### O2.5. MULTISENSORY INTEGRATION UNDERLYING BODY OWNERSHIP IN SCHIZOPHRENIA AND INDIVIDUALS AT FAMILIAL RISK TO DEVELOP PSYCHOSIS: A STUDY USING THE RUBBER HAND ILLUSION PARADIGM

Neeltje Van Haren*[,1], Merel Prikken[1], Anouk van der Weiden[2], Heleen Baalbergen[1], Manon Hillegers[3], Henk Aarts[2], René Kahn[4]
*[1]University Medical Centre Utrecht, Brain Centre Rudolf Magnus; [2]Utrecht University, Social and Behavioural Sciences; [3]Erasmus MC, Rotterdam; [4]Icahn School of Medicine at Mount Sinai*

**Background:** Patients with schizophrenia suffer from fundamental self-disturbances and have difficulties integrating and distinguishing between the self and others. For example, they experience that bodily boundaries vanish, that body parts are located at the wrong part of the body or that they are not the subject of their own movements. Such experiences are referred to as disturbances in the sense of body ownership. Although these are well-described psychotic symptoms, surprisingly little is known about their etiology and development. Our aim was to replicate a more flexible sense of body ownership in patients, thereby using a well-controlled experimental procedure (with proprioceptive drift and subjective strength of the illusion. Second, we examine whether increased familial risk to develop psychosis (i.e., offspring of patients with schizophrenia), relative to increased familial risk to develop mood disorders or the absence of familial risk, is related to alterations in RHI measures.
**Methods:** With a Rubber Hand Illusion (RHI) paradigm, body ownership was assessed in two different cohorts: 1) 54 patients with schizophrenia and 56 age and gender matched controls and 2) 24 children/adolescents with at least one parent with schizophrenia, 33 children/adolescents with at least one parent with bipolar disorder, and 18 age and gender matched controls. In this paradigm, a visible rubber hand and the invisible real hand were stroked either synchronously or asynchronously. Subsequently, proprioceptive drift and subjective RHI were measured.
**Results:** All groups showed the rubber hand illusion, i.e., a stronger proprioceptive drift and higher subjective ratings of the RHI after synchronous compared with asynchronous stroking (all p<0.001). The effect of synchronicity on subjective RHI was significantly stronger in patients with schizophrenia as compared with healthy individuals (p=0.03). No significant differences were found between children/adolescents with and without increased familial risk to develop psychosis. Last, in patients the subjective RHI was related to severity of delusions (rho=0.36).
**Discussion:** This study confirms alterations in embodied ownership experiences in patients with schizophrenia, but no evidence was found for impairments in children/adolescence with increased familial or clinical risk to develop psychosis. Longitudinal data are needed to reveal whether impairments in body ownership are predictive of psychosis onset, however, our findings provide suggestive evidence that this is not the case. In addition, that group differences were found in multisensory integration processes related to the embodiment, but not proprioceptive drift, implicates different underlying mechanisms. A possible explanation might come from the distinction between bottom-up (i.e., sensory input) and top-down (i.e., cognitive representation of body schema) mechanisms that influence multisensory integration, that is, altered cognitive representations may influence embodiment but not proprioceptive drift.

### O2.6. SOCIAL SIMULATION IN VIRTUAL REALITY IMPROVES EMBODIMENT OF EMOTIONS IN SCHIZOPHRENIA

Lénie Torregrossa*[,1], Matthew Snodgress[1], Laura Hieber Adery[1], Megan Ichinose[1], Joshua Wade[1], Dayi BIan[1], Lauri Nummenmaa[2], Eric Granholm[3], Nilanjan Sarkar[1], Sohee Park[1]
*[1]Vanderbilt University; [2]Aalto University, University of Turku; [3]UCSD*

**Background:** Bodily-self disturbances and anomalous emotional functioning are core features of schizophrenia that play a major role in social and functional outcome. Much has been written about abnormal perception and expression of emotions in schizophrenia but less is known about the bodily experience of emotions in this population. The prevalence of anomalous bodily-self experiences (Parnas & Handset, 2003), impaired simulation (Park et al, 2008) and interoception deficits (Ardizzi et al., 2016) in schizophrenia suggests that embodiment of emotions might be altered in this population.
**Methods:** We investigated emotional embodiment in individuals with schizophrenia (SZ) and demographically-matched controls to determine whether SZ experience anomalous bodily sensations of emotions. We then implemented a novel Virtual Reality (VR) social skills intervention that required participants to simulate social interactions with avatars. The VR training was designed to target social attention and improve simulation of other people's emotions, intentions, and actions in SZ, thereby improving embodiment of emotions.

We recruited twenty-six individuals with schizophrenia (SZ) and 26 demographically matched controls (CO). At baseline, we assessed social functioning, cognitive functions, symptom and embodied emotions. An online body mapping task (Nummenmaa et al., 2014) was used to generate spatial maps of bodily sensations experienced during 14 emotions categories. Then, SZ participated in the 5-week, novel VR social skills intervention that targeted social attention and simulation. Naturalistic scenarios, in which subjects moved through variable sequences of steps to attain the goal of a "mission" (e.g. find out the birthday of the avatar etc.). Subject interacted with an avatar and practiced perspective-taking and pragmatics to advance to the next level of difficulty.

**Results:** At baseline, bodily sensation maps show overall reduced embodiment of emotions in SZ as compared to CO. Statistical pattern recognition with Linear Discriminant Analysis (LDA) revealed less unique bodily sensations of emotions in SZ. Similarity scores between the maps of CO and SZ revealed a specific deficit in embodiment of low-arousal emotions (i.e. depression, sadness, shame) in SZ. After five weeks of VR training, negative symptoms and emotional embodiment improved in SZ. Specifically, embodiment of low-arousal emotions increased. Moreover, changes in the body maps of emotion indicated increased concordance among SZ.

**Discussion:** Anomalous embodiment of emotions plays an important role in the poor social outcome of individuals with schizophrenia, but a 5-week VR training of social attention, simulation, perspective taking, and communication skills was effective in improving emotional embodiment. Further research is warranted to elucidate underlying social cognitive mechanisms that link self-disturbances and embodiment of emotions.

### O2.7. CAN THEY HEAR IT? DO PATIENTS WITH AUDITORY VERBAL HALLUCINATIONS HAVE AUDITORY PROCESSING DEFICITS?

Susan Rossell*[,1], Eric Tan[2], Nathan Wilson[1], Wei Lin Toh[1], Sean Carruthers[1], Philip Sumner[1], Matthew Hughes[1], Neil Thomas[1]

*[1]Swinburne University; [2]CMH, Swinburne University, Monash Alfred Psychiatry Research Centre, The Alfred Hospital and Monash University*

**Background:** It has been well established for over half a century that patients with a diagnosis of schizophrenia have auditory processing deficits. More recently, there has been a small body of work that has suggested patients with a history of auditory hallucinations have particularly pertinent auditory processing deficits, with the importance of pitch perception being noted in a single study. This current body of work has systematically investigated whether auditory perception, using a tone detection task, are related to auditory verbal hallucinations (AVH).

**Methods:** Four studies will be presented each of which used a tone detection task that manipulated pitch, with the study 1 also have a tone duration manipulation.

**Results:** Study 1 compared 15 AVH with 15 non-AVH patients with schizophrenia with 34 healthy controls, AVH patients demonstrated the greatest impairments on pitch and tone duration deficits compared to the other two groups. Study 2 compared 99 patients with schizophrenia (65 with a history of AVH and 34 without) with 95 healthy controls and confirmed pitch deficits in schizophrenia, specifically those with AVH. Study 3 established in 100 healthy controls that unusual experiences or auditory hallucination proneness were significantly correlated to pitch perception performance. Lastly, study 4 demonstrated that relatives of patients with schizophrenia also showed a significant correlation between usual experiences and pitch perception.

**Discussion:** This research indicates auditory processing deficits are a core feature of AVH in schizophrenia, and potentially represent an endophenotype for AVH. The authors will discuss a potential cognitive model which explains the relationship between AVH and pitch perception. There are clear translational elements to this research, and we suggest there might be some utility in using auditory training as an intervention to reduce the impact of AVH.

### O2.8. TRAJECTORIES OF NEUROCOGNITIVE FUNCTIONING OVER TIME IN YOUTH AT CLINICAL HIGH RISK WHO DO AND DO NOT TRANSITION TO PSYCHOSIS

Kristen Woodberry*[,1], William S. Stone[1], Daniel I. Shapiro[1], Cole M. Chokran[2], Jean Addington[3], Carrie Bearden[4], Kristin Cadenhead[5], Tyrone D. Cannon[6], Barbara A. Cornblatt[7], Thomas H. McGlashan[6], Daniel H. Mathalon[8], Diana O. Perkins[9], Ming T. Tsuang[10], Elaine F. Walker[11], Scott W. Woods[9], Larry J. Seidman[1]

*[1]Beth Israel Deaconess Medical Center, Harvard Medical School; [2]Beth Israel Deaconess Medical Center; [3]University of Calgary; [4]University of California, Los Angeles; [5]University of California, San Diego; [6]Yale University; [7]Zucker Hillside Hospital; [8]University of California, San Francisco; [9]University of North Carolina, Chapel Hill; [10]University of California, San Diego, Institute of Genomic Medicine, University of California, La Jolla; [11]Emory University*

**Background:** In spite of evidence for the premorbid and prodromal onset of cognitive deficits in schizophrenia and related psychotic disorders, there is some limited evidence to suggest that deficits may progress with psychosis onset. Cognitive remediation in youth at risk for psychosis is being touted as an opportunity not only to remediate deficits but to potentially prevent this progression. Yet trajectories of cognitive functioning over time remain poorly understood in youth at risk, including the degree to which age at assessment or illness onset, sociodemographic factors, or symptom progression influence these trajectories.

**Methods:** The North American Prodrome Longitudinal Study (NAPLS) -2 collected data on an extensive battery of neuropsychological (NP) tests at baseline, one year, two years, and post-conversion in a sample of clinical high risk (CHR) youth and healthy comparison (HC) subjects ages 12–35 (N= 960, 92% of the full sample) followed clinically for up to 2 years. NP data were available for 694 at CHR and 265 HC. Linear mixed effects analyses were used to test the effects of group, age, gender, age of onset, maternal education, and clinical outcome on cognitive trajectories.

**Results:** Those who transitioned to a psychotic disorder over the course of follow-up performed significantly below those who did not and well below healthy comparisons. Tasks reliant on attention, visual and auditory working memory, visuospatial and verbal memory, and processing speed best differentiated those who transitioned from those who did not at one year (Cohen's d from -0.33 to -0.54). Discrepancies from normal functioning on these tests were generally large (Cohen's d from -0.67 to -1.02) consistent with findings for first episode samples. Although clinical outcome was not associated with a significantly different trajectory over time on any cognitive domain, these are likely due to high rates of conversion in this sample within the first year. Predictors of different trajectories will be presented.

**Discussion:** These data from one of the largest CHR studies to date suggest that much of the neuropsychological dysfunction in major psychotic disorders is present early in the course of illness and prior to its full expression. However, trajectories are highly heterogeneous. More frequent assessment prior to and during the onset of illness are needed to fully understand the cognitive correlates of psychosis onset and the implications for early intervention.

### O3. Oral Session: fMRI

### O3.1. NEUROTYPING UNTREATED FIRST EPISODE SCHIZOPHRENIA ON THE BASIS OF SLOW-WAVE RESTING-STATE DYNAMICS

Tushar Das[1], Mingli Li[2], Lena Palaniyappan*[,1], Tao Li[2]
*[1]University of Western Ontario; [2]Mental Health Center, West China Hospital of Sichuan University*

**Background:** Coherence in the phase of oscillatory neuronal activity indicates functional interaction among brain regions at rest. Infra-slow fluctuations in BOLD signal (band 5 - 0.01 to 0.027 Hz - and band 4 - 0.027 to 0.073 Hz) has been observed using resting state fMRI when employing frequency-domain analysis, and has been previously shown to be altered in schizophrenia. In the current study, we examined the strength and the dynamic variance of phase coherence in these 2 bands (using a sliding window approach) among 6 large-scale brain networks (default-mode, fronto-parietal, salience, sensorimotor, visual, cerebellar) in 129 drug-naïve patients with schizophrenia and 197 healthy controls. Our motivation was to exploit the large-scale resting-state slow-wave oscillatory dynamics to parse the heterogeneity of schizophrenia.
**Methods:** Four 6*6 matrices depicting patient vs. control differences in dynamic variance and mean of phase coherence (vPC and mPC respectively) in the slow 4 and slow 5 bands were constructed from resting state fMRI time series obtained from 6 networks based on 160 nodes of Dosenbach's atlas. Deviations in mean/variance of phase coherence among the 6 networks were identified after FDR correction for each matrix ($p<0.05$). A latent profile analysis (LPA) was undertaken on the basis of the identified deviant features in the patient group. LPA is a Finite Mixture Modelling approach to identify naturally occurring sub-groups of patients on the basis of multivariable data. The identified subgroups were compared in terms of the severity of clinical symptoms across van der Gaag's 5 factors of PANSS scale.
**Results:** Patients with schizophrenia showed increased vPC between salience-sensorimotor and visual-cerebellar networks in band 5; decreased vPC between DMN-sensorimotor and DMN-cerebellar networks in band 4. Patients also had a decrease in mPC between DMN-visual and DMN-cerebellar networks in band 5. We were able to identify 3 subgroups of patients using LPA. SZ1 (n=28) and SZ2 (n=45) had higher overall burden of symptoms compared to SZ3 (n=56). SZ2 had the highest burden of negative syndrome score and showed most deviance from healthy controls (5 out of 7 features significantly different from the healthy cohort). SZ1 had the highest burden of positive syndrome scores and had 4 out of 7 features deviant from HC. In contrast, SZ3 had least deviation from HC (3 out of 7 features) and also had less symptom burden across all symptom dimensions.
**Discussion:** Various abnormalities have been reported in the interactions among the large-scale networks in schizophrenia, with lack of consistency ascribed to syndromic heterogeneity. We illustrate how deviations in time-varying nature of slow-wave oscillations in resting state fMRI can be exploited to meaningfully reduce heterogeneity of this illness. The 3 subgroups thus identified not only show differential symptom burden but also exhibit hierarchical deviation from a normative group of healthy controls (SZ2>SZ1>SZ3>HC). To our knowledge this is the first attempt to stratify 'neurotypes' among drug-naïve patients with schizophrenia on the basis of large-scale network dynamics. Given the widespread availability of resting-fMRI data, we anticipate independent replication of our results in the near future.

### O3.2. BRAIN HYPERACTIVATION DURING MEMORY RETRIEVAL PRECEDES AND PREDICTS CONVERSION TO PSYCHOSIS IN INDIVIDUALS AT CLINICAL HIGH RISK

Hengyi Cao*[,1], Sarah McEwen[2], Yoonho Chung[1], Carrie Bearden[2], Jean Addington[3], Bradley Goodyear[3], Kristin Cadenhead[4], Barbara Cornblatt[5], Doreen Olvet[5], Daniel Mathalon[6], Thomas McGlashan[1], Diana Perkins[7], Aysenil Belger[7], Larry J. Seidman[8], Ming Tsuang[6], Theo van Erp[9], Elaine Walker[10], Stephan Hamann[10], Scott Woods[1], Tyrone Cannon[1]
*[1]Yale University; [2]University of California, Los Angeles; [3]University of Calgary; [4]University of California, San Diego; [5]Zucker Hillside Hospital; [6]University of California, San Francisco; [7]University of North Carolina; [8]Harvard Medical School, Beth Israel Deaconess Medical Center; [9]University of California, Irvine; [10]Emory University*

**Background:** Memory deficits are a hallmark of psychotic disorders such as schizophrenia. However, whether neural dysfunction underlying these deficits is present prior to onset of illness and potentially predicts conversion to psychosis are unclear. This study aimed to investigate: 1) baseline brain functional alterations during memory processing in subjects at clinical high risk (CHR); 2) whether alterations are more severe in converters compared with non-converters and are thus predictive of psychosis; and 3) associations of these alterations with task performance, baseline symptoms and memory ability.
**Methods:** A sample of 155 individuals at CHR (including 18 subjects who later converted to psychosis (age 17.22 ± 3.44 years, 10 male) and 137 subjects who did not convert (age 19.01 ± 4.19 years, 81 male)) and 108 healthy controls (age 20.30 ± 4.85 years, 58 male) were drawn from the second phase of the North American Prodrome Longitudinal Study (NAPLS-2) consortium. All participants underwent functional magnetic resonance imaging (fMRI) with a paired-associate memory paradigm, which consisted of one run for encoding and another run for retrieval. During encoding, participants were presented a series of semantically unrelated word pairs and were asked to remember the presented word pair. During retrieval, a pair of words was presented on the screen on each trial and subjects were asked to indicate whether the given word pair had been presented during the encoding session. Active baseline conditions were included in the task for both encoding and retrieval runs.
Data processing was performed for each run, following the standard procedures using the Statistical Parametric Mapping software (SPM12). At individual level, preprocessed images were entered into a general linear model (GLM), generating individual contrast maps (task vs baseline). These contrast maps were further used for a group-level GLM analysis, modeling group, sex, age and site as regressors. Significance was determined using family-wise error (FWE) correction across all voxels in the brain.
The observed activation alterations were further tested for potential associations with task performance, clinical symptoms and/or general memory ability. Task performance was measured using the percentage of correct responses and the mean reaction time during retrieval. Clinical symptoms were evaluated by the summed scores of each domain (positive, negative, disorganization, general) in the Scale of Prodromal Symptoms (SOPS). Memory ability was quantified by the Brief Visuospatial Memory Test- Revised (BVMT-R) and the Hopkins Verbal Learning Test- Revised (HVLT-R) total recall scores.
**Results:** No significant group differences in activation were found during encoding. However, during retrieval, a significant group effect was observed in five brain regions: left dorsolateral prefrontal cortex ($T = 4.75$, $P_{FWE} = 0.034$), left ventrolateral prefrontal cortex ($T = 4.99$, $P_{FWE} = 0.013$), left inferior parietal lobule ($T = 4.73$, $P_{FWE} = 0.035$), left superior temporal gyrus ($T = 5.71$, $P_{FWE} = 0.001$), and right middle temporal gyrus ($T = 4.89$, $P_{FWE} = 0.019$). This effect was indicative of greater activation in converters than non-converters and controls and was particularly manifest in unmedicated subjects ($P < 0.001$). Baseline hyperactivation was correlated with retrieval reaction time during scan in converters ($R = 0.61$, $P = 0.009$), and with baseline positive, negative and disorganization symptoms ($R > 0.18$, $P < 0.003$) and memory scores ($R < -0.15$, $P < 0.01$) in the whole sample.
**Discussion:** These findings suggest that hyperactivation during memory retrieval may mark processes associated with conversion to psychosis; such measures have potential as biomarkers for psychosis prediction.

### O3.3. REWARD PROCESSING AS A VULNERABILITY INDICATOR FOR PSYCHOSIS: RESULTS FROM A TWIN STUDY

Mette Nielsen*,[1], Egill Rostrup[2], Rikke Hilker[2], Christian Legind[2], Maria H Jensen[2], Simon Anhøj[2], Brian V. Broberg[2], Birgitte Fagerlund[2], Birte Glenthøj[2]
*[1]Center for Neuropsychiatric Schizophrenia Research; [2]Center for Neuropsychiatric Schizophrenia Research, & Center for Clinical Intervention and Neuropsychiatric Schizophrenia Research, Psychiatric Center Glostrup, University of Copenhagen, Glostrup University Hospital*

**Background:** Disturbances of the brain reward system is a common finding among patients with schizophrenia. Recent studies have found similar alterations in healthy relatives to patients with schizophrenia and reward disturbances have been suggested to be an endophenotype for schizophrenia. Here we compare brain reward activity between patients with schizophrenia, their healthy co-twin and a sample of matched healthy controls (HC). We hypothesize that patients as well as the healthy co-twins will show reward alterations compared to HC.
**Methods:** By coupling information from The Danish Twin register to the Danish Psychiatric Central Research Register, twins with a diagnosis of schizophrenia spectrum disorder were identified and invited to participate in the study. A sample of age and gender matched HC twins were invited as well. All participants went through a diagnostic interview, and psychopathology was characterized with the Positive and Negative Syndrome Scale (PANSS) and Clinical Global Impression (CGI). Current level of function was estimated with Global Assessment of Function (GAF). The reward system was examined with a modified version of the monetary incentive delay task during functional magnetic resonance imaging (fMRI).
**Results:** A total of 219 participants were included in the study. Of these, 162 participants completed the fMRI scanning. For statistical reasons (independent observations), only one healthy twin from each HC couple was included in the analyses, which resulted in 116 participants: 42 patients with schizophrenia spectrum disorder, 34 healthy co-twins and 40 HC. Mean age for the whole group was 40.7(10.6) and 64 (55%) were males. There were no group differences in age and sex ($p>0.6$).
**Psychopathology:** Patients had a PANSS total score of 60(18.9), a CGI score of 4.1(1.5) and a GAF score of 55 (14.9). For the co-twins, PANSS-total was 36(7.1), CGI was 1.5(0.9) and a GAF was 78(11.1), whereas the HC had a PANSS total score of 31(1.6), CGI was 1(0.2) and a GAF was 86(5.6). One-way ANOVA showed a significant effect of group for PANSS total, all PANSS sub-scores, CGI and GAF. For all measures, post hoc t-tests showed significant group-differences between co-twins and HC (all $p<0.03$), co-twins and patients (all $p<0.001$) and HC and patients (all $p<0.001$).
Reward related fMRI activity: Whole brain group differences were analyzed in FSL by performing three pairwise comparisons: co-twins versus HC, co-twins versus patients, and HC versus patients. A corrected cluster significant threshold of $P=0.05$ was used.
During reward anticipation there were no group differences between co-twins and HC or co-twins and patients. Compared to HC, patients had a decreased contrast activity in the bilateral dorsolateral prefrontal cortex during anticipation of uncertain events. Likewise, patients showed decreased activity in left caudate during anticipation of loss.
During reward outcome evaluation, co-twins showed increased contrast activity compared to HC in the miss contrast and in the monetary loss contrast. Additionally, both co-twins and HC had increased contrast activity compared to patients in the miss contrast.
**Discussion:** Although the healthy co-twins were all without clinical diagnoses, they had a subtle but significantly higher level of psychopathology and a lower level of function compared to HC. Interestingly, the healthy co-twin had an aberrant increased activity during evaluation of negative outcome compared to the HC but also compared to their diagnosed twin. This may indicate that reward abnormalities can be observed along with even subtle psychopathology and thus may serve as vulnerability indicator of psychiatric conditions, whereas these abnormalities are partly normalized by medication in the diagnosed twin.

### O3.4. INCREASED ENGAGEMENT OF THE FRONTO-PARIETAL NETWORK AND DECREASED ENGAGEMENT OF THE DEFAULT-MODE – CINGULO-OPERCULAR – SENSORIMOTOR BETWEEN-NETWORK CONNECTIVITY IN FIRST-EPISODE PSYCHOSIS PATIENTS

Eva Rikandi*,[1], Teemu Mäntylä[1], Tuula Kieseppä[2], Jaana Suvisaari[1], Tuukka Raij[3]
*[1]National Institute for Health and Welfare, Helsinki; [2]Helsinki University and Helsinki University Hospital; [3]Aalto University*

**Background:** The brain basis of psychotic disorders is still inadequately understood; however, evidence strongly suggests a central role of the dysfunctional integration of signaling between brain systems, i.e. "dysconnectivity". Resting-state functional connectivity (FC) studies of chronic schizophrenia have revealed several illness-related, network-level changes. Patients have shown reduced modular structure, changes in the subcortical-cortical interactions, increased FC within the default-mode network (DMN) and reduced FC between fronto-parietal (FP) network components. Network-level changes in earlier stages of psychotic illnesses are less studied but DMN hyperconnectivity, loss of anticorrelation between task-positive and task-negative networks and both hypo- and hyper- corticostriatal connectivity have been reported in early and at-risk stages of psychosis. While studies using tasks and resting-state have yielded plenty of valuable information, regular fmri tasks capture only a narrow field of brain functioning and during rest, behavior may vary greatly between subjects. In this study we assessed subnetworks of first-episode psychosis (FEP) patients during processing of a movie stimulus that includes everyday-like rich variety of stimuli.
**Methods:** We recorded 3T fMRI of 71 FEP patients and 57 control subjects, recruited from the Helsinki Early Psychosis Study, while they watched scenes from the movie Alice in Wonderland (Tim Burton, 2010). We then constructed a network of 160 nodes based upon a meta-analysis of regions related to a wide range of cognitive and emotional processing, we extracted signal time courses from each node and created a 160x160 correlation matrix for each subject. Using GraphVar software, we first identified all pairs of nodes where FC was statistically significantly different between groups ($p < 0.05$, FDR-corrected for multiple comparisons) and then extracted Graph-Components, or subnetworks, in which all pairs of nodes are connected by significant links.
**Results:** We identified a statistically significant subnetwork of 49 nodes with mainly decreased but also some increased links of FC in patients. Nodes that had a high number of decreased FC links in patients were mostly situated bilaterally in the medial prefrontal (mPFC) regions of the DMN and subcortical regions of the cingulo-opercular (CO) network, concentrated in the basal ganglia. The decreased FC links of the DMN were relatively wide-spread, connecting to some nodes within the DMN and several nodes of the CO and sensorimotor (SM) networks as well as the cerebellum. The decreased FC links of the basal ganglia were mainly connected to nodes of the SM network and the cerebellum. Patients had nodes with several links of increased FC mainly in the FP network, connecting to nodes within the FP as well as nodes of the CO network and the cerebellum.
**Discussion:** Our results indicate that during naturalistic stimulus, network-level changes in FC are already present at the early stages of psychoses implicating similar networks and links as seen in earlier studies using resting state and simple stimuli in mainly chronic patients. However, we found the within FP as well as FP-CO connectivity to be increased in patients, seemingly contradicting earlier evidence of reduced FC between

FP components. It would seem that during movie viewing patients engage more regions involved in attentional control (perhaps for compensatory purposes) whereas control subjects have stronger involvement of regions related to spontaneous cognition and high-order integration.

### O3.5. TESTING THE DOPAMINE HYPOTHESIS OF PSYCHOSIS USING POSITRON EMISSION TOMOGRAPHIC IMAGING IN FIRST EPISODE BIPOLAR AFFECTIVE DISORDER AND SCHIZOPHRENIA

Sameer Jauhar*[,1], Matthew Nour[1], Mattia Veronese[2], Maria Rogdaki[2], Ilaria Bonoldi[1], Matilda Azis[3], Federico Turkheimer[3], Philip McGuire[1], Oliver Howes[2]
*[1]Institute of Psychiatry, Psychology & Neuroscience, King's College; [2]King's College London; [3]Northwestern University*

**Background:** The dopamine hypothesis of psychosis suggests that dopamine abnormalities are present in psychotic illness, irrespective of diagnostic class. Meta-analyses of Positron Emission Tomography (PET) studies of the dopamine system have shown elevated dopamine synthesis capacity in schizophrenia, though there is a dearth of studies examining this in other psychotic disorders.
We therefore sought to answer the question of whether abnormalities of the presynaptic dopamine system are seen in bipolar psychosis, how this compared to schizophrenia, and whether positive psychotic symptoms were associated with dopamine synthesis capacity, irrespective of diagnostic class.
**Methods:** Cross-sectional, case-control 18F-DOPA Positron Emission Tomography (PET) study in people with first episode bipolar psychosis, schizophrenia and control subjects. Clinical measures included the Positive and Negative Syndrome Scale (PANSS), Young Mania Rating Scale and Global Assessment of Functioning (GAF).
**Results:** Mean (SD) ages were 23.6 (3.6) years in 22 people with bipolar psychosis (13 male), 26.3 (4.4) years in 16 people with schizophrenia (14 male), and 24.5 (4.5) years in controls (14 male). There was a significant group difference in striatal dopamine synthesis capacity (Kicer) ($F_{2,57}=6.80$, $P=.002$), post-hoc tests indicating Kicer was significantly elevated in both the bipolar group (mean [SD], 13.18 [1.08]×10–3 min-1; $P=.002$) and the schizophrenia group (mean [SD], 12.94 [0.79]×10–3 min-1; $P=.04$) compared with controls (mean [SD], 12.16 [0.92]×10–3 min-1). Kicer was positively correlated with positive psychotic symptom severity in the combined bipolar and schizophrenia sample currently experiencing psychosis, explaining 27% of the variance in symptom severity ($n=32$, $r=0.52$, $P=.003$).
**Discussion:** This is the first study to examine the presynaptic dopamine system in bipolar psychosis, finding an elevation compared to controls, equivalent to schizophrenia, from first onset of illness. A relationship was found between dopamine synthesis capacity and positive psychotic symptoms, across diagnostic classes, indicating a transdiagnostic role for dopamine synthesis capacity and positive psychotic symptoms.

### O3.6. DEFICITS IN CONTEXT-DEPENDENT ADAPTIVE CODING IN EARLY PSYCHOSIS AND HEALTHY INDIVIDUALS WITH SCHIZOTYPAL PERSONALITY TRAITS

Matthias Kirschner*[,1], Amelie Haugg[1], Andrei Manoliu[1], Erich Seifritz[1], Philippe N. Tobler[2], Stefan Kaiser[3]
*[1]University Hospital of Psychiatry, University of Zurich; [2]University of Zurich; [3]University Hospitals, Switzerland*

**Background:** Adaptive coding of reward values is a fundamental principle of brain functioning to efficiently represent a theoretically infinite range of rewards in the natural environment with the limited coding range of reward-processing neural machinery. Patients with schizophrenia show impaired neural adaptation to the current reward context. However, it is unknown if and how generally this impairment extends across the psychosis spectrum.
**Methods:** We studied 27 patients with first-episode psychosis, 26 individuals with schizotypal personality traits and 25 healthy controls using functional magnetic resonance imaging in combination with a variant of the monetary incentive delay task. We assessed adaptive reward coding in two reward conditions with different reward ranges.
**Results:** Compared to healthy controls, patients with first-episode psychosis and individuals with schizotypal personality traits showed less efficient neural adaptation to the current reward context in the caudate. The two groups therefore showed a similar deficit in reward representation as patients with schizophrenia. In addition, we find impaired adaptive coding of reward in the caudate and putamen to be associated with total symptom severity across the psychosis continuum.
**Discussion:** Deficits in adaptive coding were prominent across the psychosis continuum and even detectable in unmedicated healthy individuals with schizotypal personality traits. In addition, the association between total symptom severity and impaired adaptive coding in the right caudate and putamen suggests a dimensional mechanism underlying imprecise neural adaptation. Our findings support the idea that impaired adaptive coding may be a general information-processing deficit across the psychosis spectrum and not limited to schizophrenia.

### O3.7. EFFECT OF N-ACETYLCYSTEINE ON BRAIN GLUTAMATE LEVELS AND RESTING PERFUSION IN SCHIZOPHRENIA

Grant McQueen*[,1], Alice Egerton[1], John Lally[1], Fernando Zelaya[1], David J. Lythgoe[1], Gareth J. Barker[1], James Stone[1], Philip McGuire[1], James MacCabe[1], Tracey Collier[1]
*[1]Institute of Psychiatry, Psychology & Neuroscience, King's College London*

**Background:** Schizophrenia may be associated with elevations in glutamate levels in the anterior cingulate cortex (ACC), and this may be particularly apparent in patients who have not responded well to conventional antipsychotic treatment (Egerton et al., 2012; Mouchliantis et al., 2016). This suggests that compounds that can decrease ACC glutamate levels may have therapeutic potential for this group. N-acetylcysteine (NAC) is one such compound, currently under investigation as an adjunctive therapy for schizophrenia. The effects of NAC on brain glutamate levels and physiology in schizophrenia have not been previously evaluated. The primary aim of this study was to examine whether a single oral dose of NAC can alter brain glutamate levels in schizophrenia. The secondary aim was to characterise the effects of NAC on regional brain perfusion.
**Methods:** In a double-blind placebo-controlled crossover study, twenty patients with a diagnosis of schizophrenia underwent two 3 Tesla MRI scans, performed one week apart, and following administration of a single oral dose of 2400mg NAC or matching placebo. Proton magnetic resonance spectroscopy (1H-MRS) was used to investigate the effect of NAC on glutamate and Glx (glutamate plus glutamine) levels scaled to creatine (Cr) in the anterior cingulate cortex (ACC) and in the right caudate nucleus. Pulsed continuous arterial spin labelling (pCASL) was used to measure the effects of NAC on resting cerebral blood flow (CBF) in the same regions. 1H-MRS spectra were analysed using LCModel version 6.3-0I using a standard basis set. Individual CBF maps were pre-processed in the Automatic Software for ASL Processing (ASAP) toolbox running in SPM-8 in Matlab 6.5. The effects of NAC on 1H-MRS metabolite levels were determined using paired samples t-tests. Changes in rCBF were determined using within-subjects, second-level analysis implemented in SPM-8.

**Results:** In the ACC, Glx/Cr was significantly reduced in the NAC compared to placebo condition (t(17) = 2.40; P = .03, d = 0.64). There was no significant effect of condition on Glu/Cr in the ACC, or on Glx/Cr or Glu/Cr in the right caudate nucleus, or on any of the other metabolites quantifiable from the 1H-MRS spectra. There were no significant differences in CBF in the ACC (mean (SD) placebo = 47.22 (8.81); NAC = 46.83 (7.29); t(18) = .349, P = .73) or in the right caudate nucleus (mean (SD) placebo = 37.51 (7.48); NAC = 37.77 (6.71); t(18) -.310, P = .76) in the NAC compared to placebo condition. There was also no significant difference in global CBF between conditions (mean (SD) placebo = 39.64 (10.02); NAC = 40.03 (9.13); t(18) = -.398, P = .70).

**Discussion:** These results provide preliminary evidence that NAC may reduce ACC glutamate metabolites in schizophrenia. Future studies will need to determine the extent to which reductions in glutamate metabolites following a single dose of a glutamatergic compound are indicative of longer-term efficacy in improving symptoms.

### O3.8. DORSOLATERAL PREFRONTAL CORTEX IN DRUG-NAÏVE FIRST EPISODE SCHIZOPHRENIA: DYNAMIC PHASE COHERENCE OF INFRASLOW OSCILLATIONS

Tushar Das[1], Mingli Li[2], Lena Palaniyappan*[,1], Tao Li[2]
*[1]University of Western Ontario; [2]Mental Health Center, West China Hospital of Sichuan University*

**Background:** Dorsolateral Prefrontal Cortex (DLPFC) has been identified as the seat of many synaptic changes in schizophrenia. Functional neuroimaging studies indicate inefficient recruitment of DLPFC, often termed as hypofrontality, that appears in conjunction with disrupted connectivity of DLPFC with other brain regions. Very Low Frequency Oscillations (VLFO or infraslow oscillations) stem from AMPA currents, and are thought to represent a slow, cyclic modulation of cortical gross excitability. These oscillations are phase-synchronised enabling long-distance communication. This synchrony fluctuates across time (dynamic), indicating state-shifts. Dynamic synchrony can be captured using variance of phase coherence (vPC). We aimed to isolate the brain regions showing abnormal vPC with right DLPFC in drug-naïve first episode schizophrenia compared to healthy controls. Based on our prior work indicating fronto-insular dysconnectivity, we hypothesized that anterior insula would show the most disrupted dynamic phase coherence with DLPFC among all other brain regions.

**Methods:** 129 drug-naïve patients with first episode of schizophrenia (FES) and 197 age- sex- and education-level matched healthy controls (HC) were recruited. Based on Dosenbach's atlas applied to 7.67 minutes (230 timepoints with TR=2 s) of eyes-open resting fMRI scan, we extracted timeseries of 160 functional network nodes, and identified right DLPFC with MNI coordinates (x=40,y=36,z=29). Wavelet-transformation was done to enable time-frequency analysis that decomposed the timeseries data into 3 bins of very low frequency oscillations (0.02–0.04 Hz, 0.04–0.06 Hz, 0.06–0.08 Hz). We then estimated phase coherence between DLPFC and other 159 regions at each timepoint, and the variance (vPC) across the entire acquisition. Regions showing aberrant vPC with right DLPFC were identified using FDR corrected 2-tailed $p<0.05$ as the threshold of statistical significance in a two-sample t test comparing HC and FES groups. A two-step clustering procedure using likelihood-distance measure was employed to stratify sub-groups of FES with or without vPC disruption. The resulting subgroups were compared based on clinical symptom scores using van der Gaag's 5-factor PANSS model.

**Results:** The FES group showed a significant reduction (FDR corrected p = 0.03) in vPC between right DLPFC and left anterior insula within the frequency band: 0.02 - 0.04 Hz. The rDLPFC-lAI path can be termed as a long-distance connection based on anatomical distance measure (82.6 mm, compared to a median of 75mm) when compared to HCs. FES group was split into 2 subgroups using the clustering procedure. FES-2 (n=51) had higher vPC of rDLPFC-lAI connectivity compared to HC (Hedges' g: -2.0, p=0.001) while FES-1 (n=78) had lower vPC compared to HC (Hedges' g: 0.47, p=0.009). FES-1 had more 'emotional' and 'positive symptoms' but had similar symptom loadings in other PANSS domains compared to FES-2.

**Discussion:** Reduced variability of phase coherence between anterior insula and DLPFC in schizophrenia confirms our prior hypothesis of Salience Executive Loop dysfunction in this illness. Current results indicate that aberrant connectivity with anterior insula is the major lateral prefrontal disruption even in a drug naïve state at rest. Further, this aberration seems to be specific to infraslow rather than other frequency bands, raising the possibility that this may be a key mechanism of modulating overall cortical excitability in schizophrenia. It is possible that this dysfunction is specific to a subgroup with more psychotic symptoms at first presentation.

### O4. Oral Session: Genetics

### O4.1. GENETIC VULNERABILITY TO DUSP22 PROMOTOR HYPERMETHYLATION IS INVOLVED IN THE RELATION BETWEEN IN UTERO FAMINE EXPOSURE AND SCHIZOPHRENIA

Marco Boks*[,1], Lotte Houtepen[1], Zhida Xu[1], Yujie He[1], Gianluca Ursini[2], Adam Maihofer[3], Prashanth Rajarajan[4], Hilleke Hulshoff Pol[5], Bart Rutten[6], Andrew E. Jaffe[7], Joel E. Kleinmann[7], Dewleen Baker[3], Elly Hol[8], Schahram Akbarian[4], Caroline Nievergelt[3], Lot D. Witte[9], Christiaan Vinkers[9], Daniel R. Weinberger[7], Yaqin Yu[10], René Kahn[4]
*[1]University Medical Centre Utrecht; [2]Lieber Institute for Brain Development; [3]University of California, San Diego; [4]Icahn School of Medicine at Mount Sinai; [5]Rudolf Magnus Institute of Neurosciences, University Medical Center Utrecht; [6]Maastricht University Medical Centre; [7]Lieber Institute for Brain Development, Johns Hopkins Medical Campus; [8]Rudolf Magnus Center; [9]Brain Center Rudolf Magnus, University Medical Center Utrecht; [10]School of Public Health, Jilin University*

**Background:** Epigenetic changes may account for the doubled risk to develop schizophrenia in individuals exposed to famine in utero.

**Methods:** We therefore investigated DNA methylation in a unique sample of patients and healthy individuals conceived during the great famine in China. To further examine the causality of the identified DNA methylation differences we also exposed human fibroblasts to nutritional deprivation and analyzed changes in expression and DNA methylation.

**Results:** In the famine exposed schizophrenia patients we found significant hypermethylation of the dual specificity phosphatase 22 (DUSP22) gene promoter (Chr6:291687–293285) (N=153, p=0.01). The presence of a direct link between famine exposure and DUSP22 transcription was supported by increased methylation (p=0.048) and expression (p=0.019) in response to nutritional deprivation in the cultured human fibroblasts (N=10). These findings are in line with previous research that implicated hypermethylation of DUSP22 in the environmental risk to neuropsychiatric disorders. In postmortem brain samples from schizophrenia patients, variation in DUSP22 methylation was genetically regulated across chromosomes by a region on chromosome 16. This cross chromosomal regulation of variability in DUSP22 methylation is consistent with new 3D genome interaction data obtained using Hi-C capture in brain and previously published data on lymphocytes.

**Discussion:** Together our results identify an epigenetic locus at which the response to prenatal famine exposure is genetically regulated across chromosomes and that is relevant for a major psychiatric disorder.

### O4.2. HERITABILITY AND CORRELATION TO SCHIZOPHRENIA SPECTRUM DISORDER OF GLUTAMATE AND OTHER NEUROMETABOLITE LEVELS IN ANTERIOR CINGULATE AND LEFT THALAMUS: A REGISTER BASED MAGNETIC RESONANCE TWIN STUDY

Christian Legind*,[1], Brian Broberg[2], Rene Mandl[3], Rachel Brouwer[3], Simon Anhøj[4], Rikke Hilker[4], Maria H. Jensen[4], Philip McGuire[5], Hilleke E. Hulshoff Pol[6], Birgitte Fagerlund[7], Egill Rostrup[8], Birte Glenthoj[4]

*[1]Psychiatric Center Glostrup; [2]Center for Neuropsychiatric Schizophrenia Research; [3]University Medical Center Utrecht; [4]Center for Neuropsychiatric Schizophrenia Research and Center for Clinical Intervention and Neuropsychiatric Schizophrenia Research, Copenhagen University Hospital, Mental Health Center Glostrup,[5]Institute of Psychiatry, Psychology & Neuroscience, King's College London; [6]Brain Center Rudolf Magnus, University Medical Center Utrecht; [7]Center for Neuropsychiatric Schizophrenia Research, Psychiatric Center Glostrup, Mental Health Services Capital Region of Denmark, University of Copenhagen and The Lundbeck Foundation Initiative for Integrative Psychiatric Research (iPSYCH); [8]Center for Neuropsychiatric Schizophrenia Research, & Center for Clinical Intervention and Neuropsychiatric Schizophrenia Research, Psychiatric Center Glostrup, University of Copenhagen, & Glostrup University Hospital*

**Background:** Glutamatergic changes in schizophrenia may precede dopaminergic alterations leading to psychopathology as part of a dopaminergic pathway, or they may represent a distinct pathophysiology. It is unknown if these glutamatergic alterations are due to genetic influences, although findings in gene studies implicate the NMDA receptor. Higher levels of glutamate and glutamine have been reported in the thalamus of patients with schizophrenia, while studies of the anterior cingulate cortex (ACC) have reported increased, decreased, and unaltered levels compared to healthy controls.
By studying discordant mono- (MZ) and dizygotic (DZ) twins it is possible to estimate heritability and correlation to disease in the same population. This kind of study has only been done once on glutamate levels, but no heritability estimates were reported.[1] A study of older healthy twins found N-acetyl aspartate (NAA), choline (Cho), creatinine (Cr), and myo-inositol (MI) levels to be heritable in posterior cingulate cortex.[2] Of these metabolites, NAA has generally been found to correlate negatively with schizophrenia. Here we present our final results on heritability, and correlation to liability for schizophrenia spectrum disorder (ICD-10 F2x.x) of the neurometabolite levels in the ACC and the left thalamus.
**Methods:** By linking The Danish Twin Register and The Danish Psychiatric Central Research Register, 25 complete MZ and 21 complete DZ twin pairs con- or discordant for schizophrenia spectrum disorder (ICD 10 F2x.x) and 29 complete MZ and 20 complete DZ healthy control pairs were included. Thirteen additional twins were scanned without their siblings. Spectra of glutamate, Glx, NAA, Cho, Cr and MI were obtained by [1H]-MR spectroscopy at 3 tesla and analyzed by using LCModel. Additive genetic, common environmental and unique environmental effects on metabolite levels were calculated by structural equation modeling with openMX software. The best fitting model was determined by the Akaike Information Criterion.
**Results:** In the ACC heritability estimates were significant for glutamate (29%), Glx (31%), NAA (39%), Cho (38%), Cr (37%) and MI (33%). In the left thalamus we found significant estimates of heritability for glutamate (16%), Glx (31%), Cho (60%), and of common environment for Cr (29%). A significant positive correlation to schizophrenia spectrum liability was found for glutamate in the left thalamus ($r=0.16$; $p = 0.03$), and negative correlations were found for NAA ($r = -0.16$; $p = 0.02$) and Cr ($r = -0.25$; $p = 0.006$) in the ACC. For glutamate in the thalamus and Cr in the ACC the significant correlation to disease was due to overlapping genetic effects influencing both metabolite and disease.
**Discussion:** In this the first study to estimate heritability of glutamate levels in the brain, the primary findings are that glutamate levels in both the ACC and the left thalamus are heritable, and in the left thalamus also correlated to disease with a significant genetic overlap. This emphasizes glutamate levels in the left thalamus as a potential endophenotypic marker for schizophrenia. NAA and Cr were negatively correlated to disease in the ACC, which could point to disturbances of neuronal health and metabolism. For Cr an overlap of genes influencing both metabolite levels and disease suggests Cr as a possible candidate endophenotype.

**References:**
1. Lutkenhoff ES, van Erp TG, Thomas M a, Therman S, Manninen M, Huttunen MO et al. Proton MRS in twin pairs discordant for schizophrenia. Mol Psychiatry 2010; 15: 308–18.
2. Batouli SAH, Sachdev PS, Wen W, Wright MJ, Suo C, Ames D et al. The heritability of brain metabolites on proton magnetic resonance spectroscopy in older individuals. Neuroimage 2012; 62: 281–289.

### O4.3. INCREASED CEREBRAL BLOOD FLOW AFTER SINGLE DOSE OF ANTIPSYCHOTICS IN HEALTHY SUBJECTS DEPENDS ON DOPAMINE D2 RECEPTOR DENSITY PROFILES EVALUATED WITH PET AND MRNA EXPRESSION DATA.

Pierluigi Selvaggi*,[1], Mattia Veronese[1], Peter CT Hawkins[1], Ottavia Dipasquale[1], Gaia Rizzo[2], Alessandro Bertolino[3], Juergen Dukart[4], Fabio Sambataro[5], Steven CR Williams[1], Federico Turkheimer[1], Mitul Mehta[1]

*[1]Institute of Psychiatry, Psychology and Neuroscience, King's College London; [2]Imanova Ltd., Centre for Imaging Sciences, Hammersmith Hospital; [3]University of Bari 'Aldo Moro'; [4]Translational Medicine Neuroscience and Biomarkers, F. Hoffmann-La Roche Ltd, Basel, Switzerland; [5]University of Udine*

**Background:** Previous studies measuring cerebral blood flow (CBF) with magnetic resonance sequences like Arterial Spin Labelling (ASL) showed that patients with schizophrenia (SCZ) have increased CBF in basal ganglia and reduced blood flow in cortical areas like the prefrontal cortex. It is still not clear whether these abnormalities are related to antipsychotic treatment or rather they reflect a disease trait independent from medication. Interestingly, administration of single dose of antipsychotics in healthy volunteers produce marked functional effects that are in the same region reported as altered in SCZ. These effects are thought to depend on dopamine D2 receptor (D2R) blockade, although their relationship with antipsychotic pharmacodynamics has not been fully established yet. In fact, the haemodynamic nature of CBF measures makes difficult to interpret drug effects in terms of altered neurotransmission function. Here, we tested whether CBF changes induced by different antipsychotics mirror receptor distribution profiles of D2R. We evaluated the correlation of CBF variation with receptor density as measured with PET and brain mRNA expression extracted from the Allen Human Brain Atlas (ABA).
**Methods:** Forty-two healthy male subjects were enrolled in a double blind, randomized, placebo-controlled, crossover study. Participants were randomized in two equal parallel groups to receive a single dose of antipsychotic/placebo in three separate sessions. In Group 1 placebo, olanzapine 7.5mg (OLA) or haloperidol 3mg (HAL) were administered before the MRI scan. In Group 2 participants received placebo, 0.5mg (lowRIS) or 2mg (highRIS) of risperidone. Regional CBF was assessed with pseudo-continuous ASL (pCASL) sequence. For each antipsychotic, a paired T-test was performed in SPM12 with global CBF values as covariate of no interest.

A template image of dopamine D2 receptor density was derived from 6 PET scans in healthy volunteers using the high affinity D2/D3 antagonist ligand [18F]-Fallypride. Brain mRNA expression values for DRD2 gene (coding for D2R) were extracted from the ABA dataset by using the MENGA toolbox. CBF contrast images and the [18F]Fallypride BPND template were segmented into 83 ROIs by using the Desikan-Killiany Atlas. The regional changes in CBF against placebo (ΔCBF) were compared with regional BPND values and gene expression maps using multivariate correlations.

**Results:** For all antipsychotics, CBF changes in each ROI were directly proportional to [18F]Fallypride non displaceable binding potential (BPND) values (OLA R2= 0.24, HAL R2= 0.61, lowRIS R2= 0.54, highRIS R2= 0.52, all p<0.001) and DRD2 mRNA expression levels (OLA R2= 0.04, HAL R2= 0.15, lowRIS R2= 0.19, highRIS R2= 0.20, all chance likelihood <2%).

**Discussion:** In the present study, we were able to show that the CBF increase induced by antipsychotic is directly proportional to D2R concentration in the brain, as indexed by PET BPND maps and mRNA expression levels. Interestingly, the association strength between ΔCBF and brain receptor distribution profiles mirrored differential D2R affinity between the tested drugs. Overall, these results indicate that CBF increases after administration of a single dose of antipsychotics actually reflect known pharmacodynamics profile of these compounds. In addition, these results further reinforce previous evidence suggesting the role of D2R blockade as a mechanism behind increased CBF induced by antipsychotics. Finally, CBF is ultimately a functional marker and this work is important in bridging the considerable gap between the pharmacokinetic and pharmacodynamic effects of compounds with unclear brain functional effects like antipsychotics.

### O4.4. DOES POLYGENIC RISK SCORE FOR SCHIZOPHRENIA MODERATE THE MOMENTARY AFFECTIVE AND PSYCHOTIC REACTIONS TO DAILY-LIFE STRESSORS?

Lotta-Katrin Pries*[,1], Sinan Guloksuz[2], Claudia Menne-Lothmann[1], Jeroen Decoster[3], Ruud van Winkel[3], Dina Collip[1], Philippe Delespaul[1], Marc De Hert[4], Catherine Derom[5], Evert Thiery[6], Nele Jacobs[7], Marieke Wichers[8], Boris Klingenberg[1], Ozan Cinar[1], Bochao Lin[9], Jurjen Luykx[9], Bart Rutten[1], Jim van Os[10]

*[1]Maastricht University Medical Centre; [2]Maastricht University Medical Centre, Yale School of Medicine; [3]Maastricht University Medical Centre, University Psychiatric Centre KU Leuven; [4]University Psychiatric Centre KU Leuven; [5]Centre of Human Genetics, University Hospitals Leuven, Ghent University Hospitals, Ghent University; [6]Ghent University Hospital; [7]Maastricht University Medical Centre, Open University of the Netherlands; [8]Maastricht University Medical Centre, University of Groningen, University Medical Center Groningen, Interdisciplinary Center Psychopathology and Emotion Regulation; [9]Brain Center Rudolf Magnus, University Medical Center Utrecht; [10]Maastricht University Medical Centre, Brain Center Rudolf Magnus, University Medical Center Utrecht, King's College London, King's Health Partners, Institute of Psychiatry*

**Background:** Studies using the event sampling method (ESM), a structured diary technique measuring subjective experiences and emotional fluctuations in daily life, have consistently shown that individuals reporting psychotic experiences display a heightened emotional reactivity to minor stressors—a neuropsychological mechanism that likely contributes to the development and perpetuation of psychotic experiences. Except a few undersized non-replicated candidate-gene studies showing an association between genetic variations and elevated momentary stress reactivity, genetic underpinnings of emotion reactivity to momentary stressors have not been investigated. Therefore, by leveraging a large general population twin dataset of ESM, we aimed to investigate—for the first time—whether the polygenic risk score (PRS) for schizophrenia moderates stress reactivity (psychotic experiences (PE) and negative affect (NA) in response to momentary stress).

**Methods:** Data were derived from a general population adolescent and young adult twin sample. The total sample included 638 participants (Monozygotic = 202, Dizygotic = 436). ESM variables were randomly measured at 10 times/day over 6 consecutive days. For the main analyses, we assessed ESM information on PE (suspiciousness, loss of control, racing thoughts, pervasive thoughts, difficulties to express thoughts), NA (feeling lonely, anxious, listless, down, guilty), and event-related stress (pleasantness of the most important event since last entry); and for additional explorative analyses we assessed social stress (participants were asked with whom they are (e.g. nobody or family) and to rate the pleasantness of the social situation). PRS were trained on the results from the Psychiatric Genetics Consortium-2 SZ. Multilevel regression analyses, taking into account of multiple observations nested within twins who were clustered within family, were used to analyze the moderating effects of PRS (at p-value < 0.05) on the relationship between momentary stress and NA or PE. All analyses were adjusted for age, sex and 2 principle components.

**Results:** There were significant main effects of momentary stress (event stress: b = 0.065, p < 0.001, 95% CI = 0.048, 0.082; social stress: b = 0.128; p < 0.001; 95% CI = 0.111, 0.145) on PE. However, neither the main effects of PRS on PE nor the interaction between PRS and momentary stress on PE were significant. The analysis with NA as dependent variable indicated main effects of momentary stress (event stress: b = 0.096; p <0.001; 95% CI = 0.081, 0.111; social stress: b = 0.180; p < 0.001; 95% CI = 0.164, 0.197), but no main effect of PRS. There was a significant negative interaction between PRS and both event-related stress (b = -0.016; p = 0.024; 95% CI = -0.031, -0.002) and social stress (b = -0.017; p = 0.024; 95% CI = -0.031, -0.002) on NA.

**Discussion:** This is the first study investigating the influence of molecular genetic risk for schizophrenia on momentary stress reactivity, measured using an ecologically valid diary method. These results suggest that PRS for schizophrenia does not have an effect on psychotic stress responses, while increased genetic risk for schizophrenia showed a buffering effect on the association between momentary stress and NA. It is possible that individuals with high PRS for schizophrenia might have emotional response deficits, a characteristic of the clinical phenotype. Alternatively, these individuals might have been less accurate in self-evaluating momentary stress, attributing high values to stressors that genuinely do not have an impact on their emotion regulation. Future studies, investigating both clinical and general populations, are required to elucidate the impact of PRS on stress reactivity.

### O4.5. INVESTIGATING GENETIC PROFILES ASSOCIATED WITH 'REAL WORLD' CLINICAL OUTCOMES IN PSYCHOSIS: A RETROSPECTIVE COHORT STUDY

Emma Francis*[,1], Olesya Ajnakina[2], Marta Di Forti[2], Robin Murray[2], Robert Stewart[2], Conrad Iyegbe[2]

*[1]South London and Maudsley NHS Foundation Trust; [2]Institute of Psychiatry, Psychology & Neuroscience, King's College London*

**Background:** The Clinical Record Interactive Search (CRIS) database provides anonymised data from the full electronic health records of all patients at the South London and Maudsley NHS Foundation Trust, a large provider of secondary mental health care. We have previously shown how the large volumes of available CRIS data pertaining to outcomes can be mined and integrated with patient data collected by historical research interview. The applications of this futuristic translational research model are yet to be fully explored. The aim of this study is to determine whether transcriptomic profiles at the onset of psychosis can discern the likely trajectory symptoms over the subsequent 5 years of illness.
**Methods:** The study sample consists of 200 first-episode psychosis cases (ICD-10 codes: F20-F29 or F30-F33) aged 18–65 years who presented to SLAM (South London and Maudsley NHS Trust) mental health services between the 1st of January 2010 and the 1st of January 2015. Patients were subsequently recruited to the GAP study. Patients were followed-up electronically for 5 years post recruitment using the CRIS research platform.
RNA samples were collected at the baseline timepoint via PAXgene blood tubes and interrogates, using the Illumina HumanHT-12.v4 beadchip array. Samples were run at the National Institute for Health Research's (NIHR) Biomedical Research Centre for Mental Health (BRC-MH) at the Institute of Psychiatry, Psychology and Neuroscience. A total of 4756 probes passed a stringent quality control across the 200 samples.
**Results:** CRIS data pertaining to the GAP cohort was interrogated for information on clinical symptoms over a 5-year period using text-mining and natural language processing apps that represent over 70 different dictionary definitions of psychotic and affective symptoms. Confirmatory factor analysis was used to reduce this to a much smaller set of orthogonal symptom dimensions which were then the subject of a genetic interrogation using gene expression data. The analysis was conducted using a statistical learning framework which combines Elastic net penalised regression methodology with K-fold cross-validation (via the GLMnet package in R). This identified gene transcripts that were predictive of longer term symptom trajectories in half of the available sample. The veracity of the model was further validated using the second withheld portion of the sample.
**Discussion:** The results of this discovery phase may provide a rationale for subsequent multi-modal investigations whose aims will be to further enrich the biomarker signature and to also understand the molecular mechanisms that sustain them.

### O4.6. GENOME-WIDE ASSOCIATION STUDY, HERITABILITY ESTIMATION AND POLYGENIC RISK ANALYSIS OF SUSCEPTIBILITY TO INFECTIONS IN 65,534 INDIVIDUALS WITH SEVERE MENTAL DISORDERS AND POPULATION CONTROLS

Michael Benros*[,1], Ron Nudel[2], Yunpeng Wang[2], Vivek Appadurai[2], Andrew Schork[2], Esben Agerbo[3], Thomas Werge[2], Merete Nordentoft[4], Preben Mortensen[3], Alfonso Buil[2], Wesley Thompson[2]
*[1]Mental Health Centre Copenhagen; [2]Institute of Biological Psychiatry, Mental Health Center St. Hans, Mental Health Services Copenhagen; [3]National Centre for Register-based Research, Aarhus University; [4]Mental Health Centre Copenhagen, Copenhagen University Hospital*

**Background:** Infections are one of the major disease burdens internationally; however, the genetic architecture of infections is largely unknown. The human leukocyte antigen (HLA) loci have been implicated in susceptibility for infections; however, to date, largescale HLA type and genome wide association studies (GWAS) of infections have been lacking. We aim to investigate the genetic architecture of infections with GWAS of single-nucleotide polymorphisms (SNPs) and HLA types, including associations with mental disorders.
**Methods:** We conducted case-cohort association analysis using both SNP's and HLA types from a Danish population-based sample born after 1981 comprising of 65.534 unrelated Danish individuals. All individuals were linked utilizing nationwide population-based registers with virtually complete registration of all hospital contacts for infections from birth, where 28.472 (43%) individuals had ≥1 infection requiring hospitalization. Among the 45.889 cases with mental disorders, a total of 21.728 (47%) cases had hospitalizations for infections, whereas among the 19.645 individuals with no severe mental disorders, a total of 6.744 (34%) cases had hospitalizations for infections. All analyses were adjusted for age, sex, and principal components.
**Results:** We will present GWAS findings of the overall susceptibility for acquiring infections among individuals with severe mental disorders exploring differences to population controls. Furthermore, we will present SNP heritability of acquiring infections among individuals with severe mental disorders exploring differences to population controls. Moreover, we will present findings from association analysis of HLA types investigating the role of HLA alleles in susceptibility to infections and mental disorders. Lastly, we will present results on possible gene-infection interaction regarding the risk of mental illness.
**Discussion:** Our findings will illuminate the genetic architecture of acquiring infections, and the genetic associations with mental disorders exploring the possible genetic component of the known association between infections and severe mental disorders, such as schizophrenia. Furthermore, we will for the first time in a large population based study explore the associations with HLA alleles to infections and severe mental disorders.

### O4.7. PLACENTAL GENE EXPRESSION, OBSTETRICAL HISTORY AND POLYGENIC RISK FOR SCHIZOPHRENIA

Gianluca Ursini*[,1], Giovanna Punzi[1], Qiang Chen[1], Stefano Marenco[2], Joshua F. Robinson[3], Annamaria Porcelli[4], Emily Hamilton[3], Marina Mitjans[5], Giancarlo Maddalena[4], Martin Begemann[5], Jan Seidel[5], Hidenaga Yanamori[6], Andrew E. Jaffe[7], Karen F. Berman[2], Michael F. Egan[8], Richard Straub[1], Carlo Colantuoni[1], Giuseppe Blasi[4], Ryota Hashimoto[6], Dan Rujescu[9], Hannelore Ehrenreich[5], Alessandro Bertolino[4], Daniel Weinberger[1]
*[1]Lieber Institute for Brain Development; [2]National Institute of Mental Health, National Institutes of Health; [3]UCSF; [4]University of Bari 'Aldo Moro'; [5]Max Planck Institute of Experimental Medicine, DFG Research Center for Nanoscale Microscopy and Molecular Physiology of the Brain (CNMPB); [6]Osaka University Graduate School of Medicine, Molecular Research Center for Children's Mental Development, United Graduate School of Child Development, Osaka University; [7]Lieber Institute for Brain Development, Johns Hopkins Medical Campus; [8]Merck and Co., Inc.; [9]University of Halle*

**Background:** Early life events influence later susceptibility to many adult diseases and may contribute to define the environmental context in which genes enhance risk for complex disorder like schizophrenia. Here we analyze the role of intrauterine and perinatal environment in modulating the association of schizophrenia with genomic risk.
**Methods:** We evaluated whether genomic risk for schizophrenia interacts with intrauterine and perinatal complications (Early Life Complications, ELCs) on case-control status, in three independent samples of healthy subjects and patients with schizophrenia from USA (n=501), Italy (n=273) and Germany (n=919). We further analyzed the relationship between genomic risk and ELCs in two samples of only patients with schizophrenia from Germany (n=1019) and Japan (n=172). Genomic risk was measured with polygenic risk profile scores based on GWAS-significant alleles (PRS), while ELCs history was assessed with the McNeil-Sjöström Scale. We tested whether genes overlapping the schizophrenia loci interacting with ELCs are enriched in placenta and differentially expressed in placental samples from complicated pregnancies, in 8 independent placental datasets. Finally, we evaluated whether GWAS SNPs marking loci containing genes highly expressed and dynamically modulated in placenta (PlacPRS genes) drive the interaction between PRS and ELCs, and performed pathway analyses on PlacPRS genes.

**Results:** PRS interacts with ELCs on case-control status, in the three independent samples from USA (p= p=0.004), Italy (p=0.018) and Germany (p=0.018); in each sample the variance of schizophrenia explained by PRS is multiplicatively higher in the presence of a history of ELCs compared with the absence of such events. The relationship between genomic risk and ELCs is further replicated in the two independent samples of only cases from Germany (p=0.047) and Japan (p=0.044). The gene-set based on PRS loci interacting with ELCs is highly expressed in multiple placental tissues (p<0.001) and dynamically regulated in placental samples from complicated, in comparison with normal, pregnancies (p<0.05). These differences are significantly greater in placentae from male compared with female offspring (p<10–8). The interaction between PRS and ELCs is largely driven by PlacPRS genes (p=0.002); PRS constructed from the remaining loci do not interact with ELCs (NonPlacPRS, p=0.60). Pathways and biological functions associated with NonPlacPRS genes are reminiscent of previous analyses about schizophrenia risk-genes, while PlacPRS genes implicate an orthogonal biology, with roots in the fetal/placental response to hypoxic stress.
**Discussion:** Our data suggest that the most significant schizophrenia GWAS variants contribute to risk at least partly by converging on a developmental trajectory sensitive to ELCs and altered placental gene expression. The sex-associated effects on placental transcription suggest that the male preponderance of schizophrenia may arise from gene-environment interactions that influence placental biology. These results highlight placental health as a new public health frontier for primary prevention, particularly in high-risk males.

### O4.8. VULNERABLE PERIODS FOR COGNITIVE DEVELOPMENT IN INDIVIDUALS AT HIGH GENOMIC RISK OF SCHIZOPHRENIA

Sinead Morrison*[,1], Samuel Chawner[1], Therese van Amelsvoort[2], Ann Swillen[3], Elfi Vergaelen[3], Michael Owen[1], Marianne van den Bree[1]
*[1]Cardiff University; [2]Maastricht University; [3]KU Leuven*

**Background:** 22q11.2 Deletion Syndrome (22q11.2DS) is caused by the deletion of approximately 60 genes on chromosome 22 and represents one of the strongest known genetic risk factors for schizophrenia. Approximately 1 in 4 adults with 22q11.2DS are diagnosed with schizophrenia spectrum disorders, presenting with psychotic symptomatology analogous to that exhibited in idiopathic schizophrenia.
Cognitive deficits are a core feature of schizophrenia. 22q11.2DS presents a valuable model for understanding vulnerable periods of cognitive development which may be associated with psychosis development. Most previous studies report greater deficits in older individuals with 22q11.2DS than younger individuals but these studies have often focused solely on IQ, neglecting other neurocognitive domains associated with schizophrenia. Additionally, many studies of 22q11.2DS have not included adults, missing a crucial group at increased risk for schizophrenia. The first aim was therefore to examine whether there are increasing deficits in cognitive functioning on a wide range of domains in 22q11.2DS across developmental stages (children, adolescents and adults) compared to typically developing (TD) controls. The second aim was to take into account the presence of a psychotic disorder, and whether this explained variance in functioning.
**Methods:** We conducted the largest study to date of neurocognitive functioning beyond IQ in 22q11.2DS. This work was the result of international collaboration across 3 sites. The same battery of tasks measuring processing speed, attention and spatial working memory were completed by 219 participants with 22q11.2DS and 107 TD controls. Wechsler IQ tests were completed, yielding Full Scale (FSIQ), Verbal (VIQ) and Performance IQ scores (PIQ). An age-standardised difference score was produced for each participant taking into account TD control performance. The average performance of children (6–10 years), adolescents (10–18 years) and adults (18–56 years) was compared using an ANOVA approach. No children or adolescents reached diagnostic criteria for a psychotic disorder, but 13% of adults with 22q11.2DS were either diagnosed with a DSM-IV psychotic disorder. The cognitive performance of adults with or without a psychotic disorder was compared with independent t-tests with correction for unequal variance.
**Results:** Children and adults with 22q11.2DS displayed a greater deficit in working memory than adolescents (p=0.017 and p<0.001 respectively). Adults displayed greater deficits in FSIQ and PIQ than adolescents (p=0.018 and p=0.001 respectively). Adults diagnosed with a psychotic disorder displayed a greater deficit in VIQ than those without a psychotic disorder (p=0.040).
**Discussion:** Magnitude of cognitive deficit in individuals with 22q11.2DS varied by cognitive domain and developmental stage. There were specific deficits in working memory, PIQ and FSIQ in adults with 22q11.2DS compared to children and adolescents. The lack of differences between children and adolescents contradicts previous research which proposes that older children exhibit greater cognitive deficits, and suggests that there may be a longer developmental window to intervene and maintain cognitive functioning in a group at high genomic risk of schizophrenia. Adults with 22q11.2DS and psychotic disorder had a greater deficit in VIQ, which supports previous research. This international sample provides unique insights into cognitive functioning in 22q11.2DS across developmental stages.

### O5. Oral Session: Comorbidity

### O5.1. CLOZAPINE AND LONG-TERM MORTALITY RISK IN PATIENTS WITH SCHIZOPHRENIA: PRELIMINARY RESULTS FROM A META-ANALYSIS

Jentien Vermeulen*[,1], Geeske van Rooijen[1], Marita van de Kerkhof[1], Arjen Sutterland[1], Christoph Correll[2], Lieuwe de Haan[1]
*[1]Academic Medical Center, University of Amsterdam; [2]Zucker Hillside Hospital, Hofstra North Shore LIJ School of Medicine*

**Background:** Patients with schizophrenia have a high mortality risk. The role of clozapine in the long-term mortality risk is insufficiently known. The objectives of the current study were to determine in i) all-cause long-term mortality rates and ii) specific-cause mortality rates and ratios in patients with schizophrenia with and without clozapine treatment.
**Methods:** We systematically searched EMBASE, MEDLINE and PsycINFO and included studies that used a long-term follow-up design (i.e., ≥52 weeks) and reported on mortality in adults diagnosed with schizophrenia-spectrum disorders receiving clozapine treatment.
**Results:** Altogether, 23 studies fulfilled our criteria, reporting on 1,166 deaths during 203,231 patient years for patients treated with clozapine. Pooling five cohort studies that included sufficient sample sizes and length of follow-up, we found an unadjusted mortality rate of 7.34 per 1,000 patient years (95%CI=4.39–10.28). Long-term, crude mortality rate ratios were significantly lower in patients treated with clozapine compared to patients without clozapine treatment (mortality rate ratio=0.59, 95%CI=0.43–0.81, p-value<0.001) as well as compared to other antipsychotic medications (mortality rate ratio=0.61, 95%CI=0.45–0.84, p-value=0.002). We found incomplete and inconsistent reporting of specific-cause mortality rates. Statistical heterogeneity was high in all analyses.
**Discussion:** Future studies with substantial length of follow-up and uniform reporting of confounders are needed to validate these findings of a significantly lower mortality risk in patients using clozapine, in particular for the risk of cardiovascular mortality.

### O5.2. PREDICTORS OF CARDIOMETABOLIC RISK IN THE YEAR AFTER ONSET OF PSYCHOSIS: A PROSPECTIVE COHORT STUDY

Fiona Gaughran*[,1], John Lally[2], Khalida Ismail[2], David Hopkins[1], Shubulade Smith[2], Kathryn Greenwood[3], Poonam Sood[2], Brendon Stubbs[2], Dominic Stringer[1], Daniel Stahl[2], Anthony David[2], Robin Murray[2], Anita Patel[4]
*[1]King's College London; [2]Institute of Psychiatry, Psychology & Neuroscience, King's College London; [3]University of Sussex; [4]Queen Mary, University of London*

**Background:** The first episode of psychosis (FEP) is a critical time to prevent the onset of weight gain, cardiovascular and metabolic disease. However, little is known about the influence of patient characteristics and lifestyle on these outcomes
**Methods:** We conducted a prospective cohort study over 12 months of 294 people with FEP investigating the influence of lifestyle factors and medication on cardiometabolic outcomes over 12 months. Information on sociodemographics, lifestyle (physical activity (PA), sedentary behaviour (SB) nutrition, smoking), medication and service use and mental health symptoms was collected at baseline and after twelve months.
**Results:** There were high rates of cardiometabolic abnormalities and unhealthy lifestyle choices on first presentation with psychosis, increasing over the subsequent 12 months. Obesity rates rose from 17.8% to 23.7% while the proportion with Hba1c levels >/= 39mmol/mol rose from 12% to 23.7%. White participants were more at risk of developing central obesity while there were highly clinically relevant increases in mean Hba1c in those of non-white ethnicity from 36.4 to 39.7mmol/mol. We found no association between lifestyle or medication with either baseline or 12-month cardiometabolic outcomes.
**Discussion:** Cardiometabolic risk factors and unhealthy lifestyle behavior are already prevalent in those with early psychosis and worsen in the year following first presentation, making this an important time for preventative strategies. We found no evidence however that such strategies should be preferentially directed towards those reporting less healthy lifestyle habits. Patterns of emergence of cardiometabolic risk over the first year of psychosis varied by ethnicity.

### O5.3. A COMPREHENSIVE NATIONWIDE STUDY OF COMORBIDITY WITHIN TREATED MENTAL DISORDERS – A DANISH REGISTER-BASED STUDY

Oleguer Plana Ripoll*[,1], Carsten Pedersen[1], Esben Agerbo[1], Thomas Munk Laursen[2], Preben Bo Mortensen[1], John McGrath[3]
*[1]Aarhus University, National Center for Register-based Research; [2]Aarhus University; [3]University of Queensland, Queensland Brain Institute*

**Background:** Comorbidity has been the focus of a substantial body of research and it is acknowledged that different mental disorders tend to co-occur more frequently than expected. Many studies of mental disorder comorbidity have been published in recent decades, but these studies tend to be restricted to subsets of disorders, and are difficult to combine based on different methodologies. There is a need to examine comorbidity within mental disorders in a manner that covers a comprehensive range of mental disorders. The aim of this study was to use high-quality registers to (a) provide bidirectional pairwise estimates between the major groups of mental disorders, (b) investigate if associations changed depending on time since first diagnosis, (c) explore sex-specific patterns of comorbidity, and (d) estimate absolute risks rather than only incidence rate ratios of developing certain disorders after being diagnosed with one specific disorder. This abstract focus on results based on schizophrenia.
**Methods:** We designed a population-based cohort study including all Danish residents between 2000 and 2016 (N = 5,940,778). Information on incident cases of mental diseases was obtained from the Danish Psychiatric Research Register, and we classified different disorders into 10 main groups: organic mental disorders (ICD10 F00-F09), substance abuse disorders (F10-F19), schizophrenia spectrum disorders (F20-F29), mood disorders (F30-F39), neurotic disorders (F40-F48), eating disorders (F50), personality disorders (F60), mental retardation (F70-F79), pervasive developmental disorders (F84) and behavioural and emotional disorders (F90-F98). We examined associations between all pairs of mental disorders. Hazard ratios (HR) were estimated using Cox Proportional Hazards models with age as time scale, and adjusting for sex, calendar time and other psychiatric comorbidity. Finally, we estimated the absolute risk of being diagnosed with other mental disorders after being diagnosed with a specific disorder.
**Results:** All mental disorders were associated between them, with HR ranging from 1.1 to 19.5. There were 21,909 men and 20,106 women who were diagnosed with schizophrenia spectrum disorder (SSD) for the first time between 2000 and 2016. After onset of SSD, the rate of being diagnosed with substance abuse disorders was more than 4 times higher, compared to those without SSD (HR=4.4 [95%CI: 4.3–4.5]); the difference was larger within the first 6 months after being diagnosed with SSD (HR=31.8 [95%CI: 30.5–33.1]), although the rates remained higher even 15 years after the diagnosis (HR=3.2 [95%CI: 3.0–3.4]). Within the first 10 years after diagnosis of SSD, 23.8% [95%CI: 23.1–24.5] of men and 10.6% [95%CI: 10.2–11.1] of women were diagnosed for the first time with substance abuse disorders. Regarding mood disorders, the incidence rate was almost 3 times higher on individuals previously diagnosed with SSD than undiagnosed (HR=2.7 [95%CI: 2.6–2.7]). Analogous time-trends were observed, with larger differences within the first 6 months after diagnosis (HR=18.8 [95%CI: 18.1–19.6]), which diminished but remained higher 15 years later (HR=2.3 [95%CI: 2.2–2.4]). A total of 15.1% [95%CI: 14.5–15.6] of men and 20.8% [95%CI: 20.2–21.5] of women with a diagnosis of SSD were diagnosed with mood disorders within 10 years.
**Discussion:** In this population-based comprehensive study, we observed that comorbidity is pervasive and usually bidirectional. We observed that after being diagnosed with a specific disorder, the risk of being diagnosed with an additional disorder is particularly higher in the first 6 months, but even after 15 years the risk is higher compared to undiagnosed individuals.

### O5.4. NATURAL CAUSE MORTALITY IN PERSONS WITH SCHIZOPHRENIA AND BIPOLAR DISORDER

Faith Dickerson*[,1]
*[1]Sheppard Pratt*

**Background:** It is now well established that persons with schizophrenia and with bipolar disorder have a reduced life expectancy but the reasons for this premature mortality are not known with certainty. The aim of the current investigation was to identify the determinants of natural-cause mortality in a cohort of individuals with schizophrenia or bipolar disorder. To our knowledge, our investigation is unique in studying patients who were assessed at baseline with an in-person clinical assessment and blood sample and then subsequently evaluated regarding their mortality status and cause of death.
**Methods:** Persons with schizophrenia (n=789) and bipolar disorder (n=498), mean age of 38 (s.d. 12.6) years, underwent an in-person clinical assessment. They also had a blood sample drawn which was tested by enzyme immunoassay tests for IgG class antibodies to Herpes Simplex Virus type 1 (HSV-1), Cytomegalovirus (CMV), Epstein Barr Virus Nuclear Antigen (EBV), Human Herpesvirus Type 6 and Toxoplasma gondii. Participants were followed for a median observation period of 7.87 years (range 1 day – 16.9 years); the total number of person-years of observation was 10,859.3 person-years. Mortality was subsequently determined utilizing data from the US National Death Index.

**Results:** A total of 6.8% (87/1287) of persons died of natural causes. There were 70 deaths in the schizophrenia and 17 in the bipolar disorder participants. The mean age at death of those who died from natural causes was 56.7 years (range 19.4 – 79.1 years). The standardized mortality ratio (SMR), the age-adjusted ratio of the number of observed deaths in this study sample to that expected in the general population, was 2.57 (95% CI 1.24 – 4.75).
Natural cause mortality was predicted in a multivariate model by baseline cigarette smoking (RR=6.29, 95% CI 1.41, 3.72, p=0.00076); divorced or widowed status (RR=1.90, CI 1.21, 2.99); reduced cognitive score (RR=0.73, CI 0.61, 0.87); receipt of antidepressant medication (RR=1.74, CI 1.12, 2.71); elevated levels of antibodies to Epstein Barr Virus (EBV) (RR=1.29, CI 1.01, 1.66); and a genitourinary (RR 1.82, CI 1.16, 2.86), respiratory (RR 1.82, CI 1.16, 2.86), or cardiac (RR 2.09, CI 1.33, 3.29) condition.
Interaction models showed evidence of additive effects of smoking and both cardiac and respiratory condition. Compared to non-smokers without a cardiac condition, non-smokers with a cardiac condition had a more than threefold elevation of mortality risk (RR=3.76, 95% CI 1.47 – 9.63, p=0.0057) as did smokers without a cardiac condition (RR=3.63, 95% CI 1.49 – 8.85, p=0.0046), while the presence of both smoking and a cardiac condition increased mortality risk by more than six-fold (RR=6.75, 95% CI 2.84 – 16.0, p<0.0001). Compared to non-smokers without a respiratory condition, mortality risk more than doubled for non-smokers with a respiratory condition (RR=2.30, 95% CI 0.97 – 5.46, p=0.058), as well as for smokers without a respiratory condition (RR=2.37, 95% CI 1.31 – 4.28, p=0.0044), while the mortality risk more than quadrupled for smokers with a respiratory condition (RR=4.72, 95% CI 2.45 – 9.09, p<0.0001). There was not a significant interaction between smoking and elevated EBV antibody levels. There was a synergistic effect of antidepressant use and cardiac disease on mortality risk: participants with both risk factors had a more than threefold elevation of mortality risk compared to persons with neither risk factor (RR=3.10, 95% CI 1.71 – 5.63, p=0.0002).
**Discussion:** Multiple factors contribute to the excess mortality of persons with schizophrenia and bipolar disorder, but cigarette smoking is a major preventative cause. The delivery of smoking cessation treatments should be a high priority.

### O5.5. SLEEP IN MAJOR PSYCHIATRIC DISORDERS: RESULTS FROM NATIONWIDE SUPER FINLAND STUDY

Minna Torniainen-Holm*[,1], Erik Cederlöf[1], Willehard Haaki[2], Jarmo Hietala[2], Katja Häkkinen[3], Erkki Isometsä[4], Kaisla Joutsenniemi[4], Tuomas Jukuri[5], Risto Kajanne[3], Olli Kampman[6], Tuula Kieseppä[4], Nina Lindberg[4], Markku Lähteenvuo[7], Jouko Lönnqvist[1], Teemu Männynsalo[8], Jussi Niemi-Pynttäri[9], Kimmo Suokas[9], Jaana Suvisaari[1], Jari Tiihonen[10], Annamari Tuulio-Henriksson[11], Asko Wegelius[12], Juha Veijola[5], Aarno Palotie[3], Tiina Paunio[1]
*[1]National Institute for Health and Welfare, University of Helsinki; [2]University of Turku; [3]Institute for Molecular Medicine Finland, Niuvanniemi Hospital; [4]Helsinki University Hospital, University of Helsinki; [5]University of Oulu; [6]University of Tampere, Seinäjoki Hospital District; [7]Institute for Molecular Medicine Finland, University of Tampere; [8]City of Helsinki, Social Services and Health Care Sector; [9]University of Tampere; [10]Niuvanniemi Hospital, University of Eastern Finland, Karolinska Institutet; [11]University of Helsinki, The Social Insurance Institution of Finland; [12]Helsinki University Hospital*

**Background:** People with psychotic disorders demonstrate a wide spectrum of sleep abnormalities. Abnormalities include changes in total sleep time, increased sleep onset latency, increased wake-up time after sleep onset and abnormalities in sleep architecture. The study aimed to characterize the sleep difficulties in a large sample of persons with psychotic disorders and to examine association with age and gender.
**Methods:** Altogether, 5046 persons with a major psychiatric disorder (schizophrenia: 2972, schizoaffective disorder: 640, bipolar disorder: 1097 and psychotic depression: 330) and aged 18–80 participated in a nationwide Super project. The Finnish SUPER (Finnish acronym for "Finnish study on genetic mechanisms of psychotic disorders") study is a part of the international Stanley Global Neuropsychiatric Genomics Initiative. The results were compared with a representative general population sample of 8018 adults (Health 2000). Sleep was assessed in a self-report questionnaire. In the present study we used total sleep time, tiredness (defined as feeling more tired than other people of the same age during day time at least weakly, yes/no), difficulties in getting sleep without sleep medication often or almost daily (yes/no) and early morning or night awakenings occurring either often or nearly every night (yes/no).
**Results:** Long sleep (> 10h) was most common in persons with schizophrenia or schizoaffective disorder reported by approximately 30% when age was 18–40. The corresponding proportion was approximately 15% in persons with bipolar disorder or psychotic depression and less than 1% in the general population. Tiredness, difficulties in getting sleep and early morning or night awakenings were reported most by persons with bipolar disorder and psychotic depression, but also persons with schizophrenia reported those more than the general population in people with under 60 years of age. Schizoaffective disorder was between schizophrenia and affective psychoses in the sleep variables. In persons over 60, the difference between the groups was smaller than in persons under 60 years of age, because sleeping long and tiredness decreased in all patient groups, and difficulties getting sleep and awakenings increased in the general population sample more than in psychosis patients.
**Discussion:** Sleep disorders seem to be prominent in persons with major psychiatric disorders. Tiredness was common in all diagnosis groups. Long sleep was most common in schizophrenia and difficulties in getting sleep and early morning or night awakenings in affective psychoses. More research is needed on possibilities to prevent and treat sleep disorders in major psychiatric disorders.

### O5.6. SUBMISSION WITHDRAWN

### O5.7. RISK OF DIABETIC COMPLICATIONS AND SUBSEQUENT MORTALITY AMONG INDIVIDUALS WITH SCHIZOPHRENIA AND DIABETES MELLITUS: A NATIONWIDE POPULATION-BASED REGISTER STUDY

Anita Tønder Nielsen*[,1], Mogens Vestergaard[2], Trine Munk-Olsen[3], Jette Kolding Kristensen[4], Thomas Munk Laursen[5]
*[1]The National Centre for Register-based Research, Aarhus University; [2]Institute of General Medical Practice, Aarhus University; [3]National Centre for Register-Based Research, Aarhus University, The Lundbeck Foundation Initiative for Integrative Psychiatric Research (iPSYCH); [4]Research Unit for General Practice, Aalborg University; [5]Aarhus University, The Lundbeck Foundation Initiative for Integrative Psychiatric Research (iPSYCH)*

**Background:** Schizophrenia constitutes a high risk of morbidity and mortality from physical illness. Individuals with comorbid schizophrenia and diabetes mellitus have been found to have a three- to four-fold higher rate of death than the general population, which may be explained by a higher rate of diabetic complications. We aimed to study incidence of diabetic complications diagnosed in hospitals following a diabetes diagnosis and subsequent mortality in individuals with schizophrenia compared to individuals without.

**Methods:** The entire Danish population was followed in 1997–2016 using population-based registries. Incident diabetes was defined as prescription redemptions of insulin or oral antidiabetic drugs, ICD-10 diagnoses E10 or E11 related to hospital contacts, whichever came first. Diabetic complications were separated into macrovascular complications (coronary heart disease, peripheral artery disease, stroke+TCI, heart failure, myocardial infraction, foot ulcer) and microvascular complications (retinopathy, neuropathy, nephropathy).

Cox regression was used to estimate incidence rate ratios (IRR) and mortality rate ratios (MRR) and all estimates were adjusted for age and calendar time.

**Results:** The incidence rate of macrovascular complications was similar in individuals with schizophrenia and in those without; IRR=1.09 (95% CI: 0.96–1.23) for females and IRR=0.91 (95% CI: 0.83–1.01) for males. For foot ulcer the incidence rate was higher in females with schizophrenia than in females without; IRR=1.79 (95% CI: 1.12–2.85), p=0.015 and for heart failure the incidence rate was higher in males with schizophrenia than in males without; IRR=1.52 (95% CI: 1.21–1.91), p<0.000. The incidence rate for microvascular complications was similar for females with and without schizophrenia; IRR=0.88 (95% CI: 0.75–1.04), but lower in males with schizophrenia than in males without schizophrenia; IRR=0.79 (95% CI: 0.69–0.89), P<0.001.

The mortality rate following a diagnosis of a macrovascular complication was higher in individuals with schizophrenia than those without; MRR=2.17 (95% CI: 1.84–2.56) for females and MRR=2.40 (95% CI: 2.06–2.80) for males. For microvascular complications the subsequent mortality rate was also higher in individuals with schizophrenia than in those without; MRR=2.17 (95% CI: 1.67–2.80) for females and MRR=2.30 (95% CI: 1.90–2.79) for males. P<0.001 for all estimates. The Mortality rates for every single complication showed similar estimates.

**Discussion:** Unexpectedly, we found individuals with comorbid schizophrenia and diabetes mellitus to have a similar or lower rate of diabetic complications diagnosed in hospitals compared to individuals with diabetes mellitus only. However, we still found an excess mortality following a diagnosis of a diabetic complication among individuals with schizophrenia. These results may indicate that individuals are not even seen in hospitals with their diabetic complications and hence indicate an increased need for improved somatic care of individuals with schizophrenia if the burden of diabetes mellitus morbidity and mortality should be reduced.

### O6. Oral Session: Neuroimaging

### O6.1. HIPPOCAMPAL VOLUME IN ADOLESCENTS WITH PERSISTENT PSYCHOTIC EXPERIENCES: A LONGITUDINAL POPULATION-BASED MRI STUDY

Ana Calvo*[,1], Erik O'Hanlon[2], Helen Coughlan[2], Ian Kelleher[2], Mary Clarke[2], Mary Cannon[2]

*[1]Universidad Internacional de La Rioja; [2]Royal College of Surgeons in Ireland*

**Background:** Individuals with schizophrenia show significant brain morphological abnormalities. The ENIGMA consortium identified that patients with schizophrenia had smaller hippocampus, amygdala, thalamus, accumbens and intracranial volumes.[1] Reduced hippocampal volume is one of the most consistent findings in schizophrenia research.[2–4] Also, Previous research has reported differences in hippocampal volume and white matter integrity in young adolescents who report psychotic experiences.[5,6] However there has been little longitudinal research to investigate the developmental trajectory of these regions in adolescence with an increased susceptibility to psychotic disorders.

**Aims:** to investigate two-year longitudinal changes in hippocampal volume in a sample of adolescents who reported psychotic experiences relative to their peers. To investigate the role of presence of co-morbid DSM IV mental disorders and stressful life events in influencing hippocampal volume and study the differences in hippocampus volume between adolescents who were having persistent symptoms versus adolescents with remitting symptoms.

**Methods:** A longitudinal case-control study of 50 community-based adolescents aged 13–16 years (25 with psychotic experiences and a matched sample of 25 without psychotic experiences), compared hippocampal volume. All participants were assessed at baseline and two years follow up. T1 weighted anatomical high-resolution imaging and high angular resolution diffusion imaging data were used to conduct quantitative anatomical volumetric evaluations of global hippocampal volume. Clinical interviews also provided information on psychotic experiences, co-morbid disorders and adverse life events.

**Results:** There were significant differences in the Right and Left Whole hippocampus between PE and Control group at baseline and 2-year follow up (p≤ 0.05). There were significant differences between PE persist and Control group in the left and right whole hippocampus (p≤ 0.05).

**Discussion:** The differences identified in our study suggest that early hippocampal reductions, may play a role in increasing vulnerability to psychosis.

**References:**

1. van Erp TG, Hibar DP, Rasmussen JM, et al. Subcortical brain volume abnormalities in 2028 individuals with schizophrenia and 2540 healthy controls via the ENIGMA consortium. Mol Psychiatry. 2016;21(4):585.
2. Wright IC, Rabe-Hesketh S, Woodruff PW, David AS, Murray RM, Bullmore ET. Meta-analysis of regional brain volumes in schizophrenia. Am J Psychiatry. 2000;157(1):16–25.
3. Nelson MD, Saykin AJ, Flashman LA, Riordan HJ. Hippocampal volume reduction in schizophrenia as assessed by magnetic resonance imaging: a meta-analytic study. Arch Gen Psychiatry. 1998;55(5):433–440.
4. Stein JL, Medland SE, Vasquez AA, et al. Identification of common variants associated with human hippocampal and intracranial volumes. Nat Genet. 2012;44(5):552–561.
5. Drakesmith M, Caeyenberghs K, Dutt A, et al. Schizophrenia-like topological changes in the structural connectome of individuals with subclinical psychotic experiences. Hum Brain Mapp. 2015;36(7):2629–2643.
6. Satterthwaite TD, Wolf DH, Calkins ME, et al. Structural Brain Abnormalities in Youth With Psychosis Spectrum Symptoms. JAMA psychiatry. 2016;73(5):515–524.

### O6.2. NEUROBIOLOGY OF PSYCHOMETRIC SCHIZOTYPY: INSIGHTS FROM MULTIMODAL IMAGING RESEARCH

Gemma Modinos*[,1], Alice Egerton[1], David J. Lythgoe[1], Gareth J. Barker[1], Katrina McMullen[1], Christian Keysers[2], André Aleman[3], Veena Kumari[1], Steven CR Williams[1]

*[1]Institute of Psychiatry, Psychology & Neuroscience, King's College London; [2]Netherlands Institute for Neuroscience, Netherlands Academy for Arts and Sciences, KNAW, University of Amsterdam; [3]University of Groningen, University Medical Hospital Groningen*

**Background:** The continuum approach to psychosis proposes a dimensional continuity between the neurobiology of subclinical psychotic-like experiences in healthy individuals (or schizotypy) and psychotic symptoms in clinically relevant psychosis (Linscott and van Os, 2013, Nelson et al., 2013). Preclinical models propose that cortical glutamate dysfunction related to cortico-limbic-striatal hyper-responsivity to stress may underlie both hippocampal and striatal overdrive as well as gray matter loss associated with schizophrenia-like behaviors (Berretta et al., 2001, Lodge and

Grace, 2011). Our recent studies investigated whether changes in brain glutamate are present in healthy individuals with high psychometric schizotypy, and whether these are related to changes in (1) corticolimbic response to emotion and (2) gray matter volume (GMV).

**Methods:** Forty-eight healthy participants were recruited based on their score on the O-LIFE questionnaire (Mason et al., 2005), after pre-screening 250 respondents to online advertisement. Participants with high levels of unusual experiences (HS group; that is, scored >7 on the Unusual Experiences (UE) subscale of the O-LIFE), and participants with low UE (LS group; that is, <2 on O-LIFE UE subscale), were invited to participate. Groups were matched by age, gender and IQ. A structural MRI scan, glutamate proton magnetic resonance spectroscopy in the anterior cingulate cortex (ACC), and functional magnetic resonance imaging (fMRI) measuring corticolimbic response during emotional processing were acquired at 3T in a single session. Glutamate levels were analyzed using LCModel 6.3-1L. Voxel-based morphometry was applied to quantify GMV and both GMV and fMRI group level analyses were run using SPM12. Standalone imaging results as well as fMRI/sMRI × glutamate interactions were considered significant after voxel-wise $P<0.05$ family-wise error correction.

**Results:** While viewing emotional pictures, HS individuals showed greater activation than did subjects with LS in the caudate, and marginally in the ACC, hippocampus, medial prefrontal cortex (MPFC) and putamen. Although no between-group differences were found in glutamate concentrations, within the HS group ACC glutamate was negatively correlated with striatal activation (bilaterally in caudate and in left putamen at $P < 0.05$) and marginally with MPFC ($P = 0.052$) and amygdala (left: $P = 0.062$; right: $P = 0.079$), correlations that were not present in LS subjects. Structurally, subjects with HS showed GMV decreases in the rolandic operculum/superior temporal gyrus ($P < 0.05$) at the whole-brain level, and significant increases in GMV were also detected using ROI in the precuneus and ACC (both $P < 0.05$). Furthermore, in HS subjects ACC glutamate levels were negatively correlated with GMV in the ACC ($P < 0.05$). Such association was absent in LS. These findings provide, to our knowledge, the first evidence that brain glutamate levels are associated with emotional hyper-responsivity and volumetric changes in HS in brain regions thought to be critical in the pathophysiology of psychotic symptoms.

**Discussion:** Collectively, these results are in line with a dimensional view of psychosis by suggesting that interactions between brain structure, neurochemistry, and functional response to emotion within a corticolimbic circuit are involved in the expression of psychotic-like experiences at non-clinical and clinical levels. These findings may also serve as evidence of potentially protective mechanisms, as our studies involved high-functioning individuals with HS and some of the observed effects are opposite to what would have been predicted from studies in clinical groups.

### O6.3. PATTERNS OF GRAY MATTER ABNORMALITIES IN PATIENTS WITH FIRST-EPISODE AND TREATMENT-NAÏVE SCHIZOPHRENIA

Mingli Li*[,1], Tushar Das[2], Deng Wei[1], Yinfei Li[1], Xiaohong Ma[1], Hua Yu[1], Xiaojing Li[1], Ya-jing Meng[1], Liansheng Zhao[1], Yingcheng Wang[1], Qiang Wang[1], Lena Palaniyappan[2], Tao Li[1]
*[1]Mental Health Center, West China Hospital of Sichuan University; [2]University of Western Ontario*

**Background:** To detect schizophrenia-related anatomical changes that are not confounded by antipsychotic treatment and to establish clinically identifiable subgroups that differ in underlying neuroanatomical patterns.

**Methods:** This case-control study was conducted at West China hospital in China, and analysis was undertaken in Robarts Research Institute, London, Canada. 206 patients with schizophreniform psychosis and schizophrenia and 170 healthy controls were scanned on a Signa 3.0-T MR scanner; 137 patients with schizophreniform psychosis and schizophrenia and 172 healthy controls were scanned on a 3.0 T MR scanner. All the patients were first-episode and treatment-naïve. Source based morphometry (SBM) performed to analyze the gray matter (GM) concentration. Latent class analysis used to identify clinical subtypes of patients using the scores of symptom dimensions. GMC component-based connectomes were constructed to study the graphic organization of structural brain network of subtypes of schizophrenia.

**Results:** Patients showed prominent reduction in GM in two components; one including anterior insula, inferior frontal gyrus, anterior cingulate and another with superior temporal gyrus, and precuneus, inferior/superior parietal lobule, cuneus, and lingual gyrus. Increased GM was seen in one component of cerebellar tonsil and inferior semi-lunar lobule, and the other component of middle temporal gyrus, superior temporal gyrus, middle frontal gyrus and putamen. Greater GM of latter component was associated with less severe positive symptoms and better performance on cognitive tests. Reduced global efficiency only existed in a subgroup of patients with severe negative and disorganization symptoms.

**Discussion:** These findings delineate a common pattern of gray matter changes in schizophrenia, and a subgroup of patients with robust cortical reorganization suggestive of compensatory plasticity after first episode.

### O6.4. AUDITORY AND LANGUAGE AREAS DISTINGUISH CONVERTERS FROM NON–CONVERTERS AT BASELINE IN SHARP CLINICAL HIGH-RISK SUBJECTS FOR PSYCHOSIS STUDY

Elisabetta Del Re*[,1], William S. Stone[2], Sylvain Bouix[3], Nathaniel Somes[3], Huijun Li[4], YinYin Tang[5], TianHong Zhang[5], Susan Whitfield-Gabrieli[6], Robert McCarley[7], Larry J. Seidman[2], Matcheri Keshavan[2], JiJun Wang[5], Martha Shenton[8], Margaret Niznikiewicz[7]
*[1]Harvard Medical School, Veterans Affairs Boston Healthcare System, Brigham and Women's Hospital; [2]Harvard Medical School, Beth Israel Deaconess Medical Center; [3]Psychiatry Neuroimaging Laboratory, Brigham and Women's Hospital, Harvard Medical School; [4]Florida A&M University; [5]Shanghai Key Laboratory of Psychotic Disorders, Shanghai Mental Health Center, Shanghai Jiao Tong University School of Medicine; [6]Massachusetts Institute of Technology, McGovern Institute for Brain Research; [7]Harvard Medical School, Veterans Affairs Boston Healthcare System; [8]Psychiatry Neuroimaging Laboratory, Brigham and Women's Hospital, Harvard Medical School, Veterans Affairs Boston Healthcare System*

**Background:** Frontal and temporal lobes abnormalities are often reported in schizophrenia. In the present study, we tested whether or not these abnormalities exist in individuals at clinical high risk for psychosis (CHR), and whether they distinguish between those CHR who convert to psychosis versus those who do not convert to psychosis at one year. We analyzed both cortical thickness (CT) and surface area (SA) given the fact that CT and SA develop along different developmental genetically mediated pathways. Since CHR individuals also experience a deterioration of cognitive functions and sub-threshold psychotic symptoms, we also explored the relationship between cognition and symptomatology and the two brain regions.

**Methods:** Magnetic resonance images, clinical and cognitive data were acquired in 130 CHR who did not convert to psychosis (CHR-NC), 22 CHR who converted to psychosis (CHR-C) and 92 healthy controls (HC) at the Shanghai Mental Health Center, in Shanghai, China, who were tested as part of a NIH funded China and Harvard Medical School collaboration. An internal pipeline developed at the Psychiatry Neuroimaging Laboratory (PNL), Brigham and Women's Hospital, Harvard Medical School, was used to process the scans. The pipeline includes several quality control steps and FreeSurfer 5.3 (FS) processing, the latter modified to include an automated PNL developed masking methodology, the MABS. FS output was 9 temporal and 11 frontal regions in the left and right hemisphere. All data were Z-scored to the mean and standard deviation of HC. Gender and group differences were investigated using multivariate analyses, and Spearman's correlations were employed to investigate the relationship between brain measures and cognitive and clinical measures.
**Results:** SA analysis of the frontal and temporal lobes showed no significant differences among the three groups, while specific and significant group differences were found in CT. More specifically, for the temporal lobe a main effect of Group (p=0.021) and a significant interaction of Region x Group (p=0.01) were found. Post hoc analyses showed that CT of Heschl's gyrus and of the posterior region of the superior temporal sulcus distinguished CHR-C from CHR-NC (p=0.027) and from NC (p=0.002), with CT of CHR <CHR-NC=NC. For the middle temporal gyrus (MTG) CT was also significantly smaller in CHR-C than in NC (p=0.004) and at trend level in CHR-NC (p=0.098). With respect to the frontal lobe, no significant main effect of Group was found but a significant region X Group interaction was identified. Post hoc analyses showed smaller CT of the pars triangularis in CHR-C with CHR-C<CHR-NC (p=0.02) and NC (p=0.012). The CT of the pars opercularis was smaller in CHR-C compared to NC (p=0.036). In CHR-C, the CT of MTG was significantly and positively correlated with the Verbal Learning test and with the Hopkins Verbal Learning test (rho= 0.64; p=0.002), with strength of correlation decreasing with task repetition. Further CT of MTG was correlated with the Brief Visual Memory Test (rho=0.6, p=0.004). A significant and positive correlation was also found between CT of the pars opercularis (rho=0.7; p=0.002) and the Brief Visual Memory test. The same correlation was also present with the pars triangularis. None of these correlations were present in NC or CHR-NC.
**Discussion:** These results indicate that specific CT abnormalities in circumscribed areas of the frontal and temporal lobes at baseline distinguish between CHR individuals who convert to psychosis versus those who do not at one-year follow-up. The brain regions involved belong to language circuits and their CT abnormalities correlate with verbal learning suggesting that these brain circuits are among the first affected by processes leading to frank psychosis.

### O6.5. LINKING CORTICAL AND CONNECTIONAL PATHOLOGY IN SCHIZOPHRENIA

Maria Di Biase*[,1], Vanessa Cropley[2], Luca Cocchi[3], Alexander Fornito[4], Fernando Calamante[5], Eleni Ganella[2], Christos Pantelis[2], Andrew Zalesky[2]
*[1]Melbourne Neuropsychiatry Centre; [2]The University of Melbourne, Melbourne Neuropsychiatry Centre; [3]QIMR Berghofer Medical Research Institute; [4]School of Psychology and Psychiatry & Monash Biomedical Imaging, Monash University; [5]University of Melbourne*

**Background:** Schizophrenia is associated with cortical thinning and breakdown in white matter microstructure. Whether these pathological processes are related remains unclear. We used multimodal neuroimaging to investigate the relation between regional cortical thinning and breakdown in adjacent infracortical white matter as a function of age and illness duration.
**Methods:** Structural magnetic resonance and diffusion images were acquired in 218 schizophrenia patients and 167 age-matched healthy controls to map cortical thickness (CT) and fractional anisotropy (FA) in regionally adjacent infracortical white matter at various cortical depths.
**Results:** Between-group differences in CT and infracortical FA were inversely correlated across cortical regions (r=−0.5, p<0.0001), such that the most anisotropic infracortical white matter was found adjacent to regions with extensive cortical thinning. This pattern was evident in early (20 years: r=−0.3, p=0.005) and middle life (30 years: r=−0.4, p=0.004, 40 years: r=−0.3, p=0.04), but not beyond 50 years (p>0.05). Frontal pathology contributed most to this pattern, with extensive cortical thinning in patients compared to controls at all ages (p<0.05); in contrast to initially increased frontal infracortical FA in patients at 30 years, followed by rapid decline in frontal FA with age (rate of annual decline; patients: 0.0012, controls 0.0006, p<0.001).
**Discussion:** Cortical thinning and breakdown in white matter anisotropy are inversely related in young schizophrenia patients, with abnormally elevated white matter myelination found adjacent to frontal regions with extensive cortical thinning. We argue that elevated frontal anisotropy reflects regionally-specific, compensatory responses to cortical thinning, which are eventually overwhelmed with increasing illness duration.

### O6.6. LACK OF ANTIPSYCHOTIC MEDICATION EFFECTS ON WHITE MATTER MICROSTRUCTURE IN SCHIZOPHRENIA

Nina Kraguljac*[,1], Anthony Thomas[1], Frank Skidmore[1], David White[1], Adrienne Lahti[1]
*[1]University of Alabama at Birmingham*

**Background:** A number of studies have reported decreased white matter integrity in patients with schizophrenia, but little is known about the relationship between white matter integrity and antipsychotic medications.
**Methods:** We enrolled 42 unmedicated patients (thirty were medication-naïve) with schizophrenia in a longitudinal trial with risperidone. Symptom severity was assessed with the Brief Psychiatric Rating Scale (BPRS). We obtained diffusion weighted images before medication was started, and after six weeks of treatment. Healthy controls matched 1:1 on age, gender, and parental socioeconomic status were also scanned twice six weeks apart. 30 diffusion sampling directions spanning the whole sphere were acquired twice and concatenated (in plane resolution 2.2mm, slice thickness 2.2mm, b-value 1000 s/mm2, 5 b0 images). After visual inspection of raw images we used TORTOISE for correction of bulk motion, eddy currents and susceptibility artifacts using a single interpolation step in DIFF_PREP. For each dataset, the first B0 image was selected as the reference for registration. Prior to registration, diffusion weighted and structural images were upsampled at a factor two and smoothed with a Perona-Malik anisotropic edge favoring gradient based filter to compute the transformations from moving to fixed images. After computation of transformations, original images were used to create the registered images. Bspline correction was done with the T2 weighted image (approximated from a T1 image using AFNI's FATCAT). Diffusion and structural images were resampled to 1.5mm isotropic voxels. Gradient tables were rotated along with motion correction. To obtain a summary measure of motion, the root-mean-square (RMS) was calculated both for absolute (RMSabs) and relative (RMSrel) movement. Datasets with RMSabs of greater than the voxel edge length were excluded from further analysis. Tensors were computed with DIFF_CALC using a linear fitting algorithm. To spatially normalize diffusion images to the Illinois Institute of Technology atlas (IIT2) space, we implemented an optimized non-linear image registration using a modified version of 3dQwarp in AFNI. The warping optimization implements an iterative refinement, where an input image is repeatedly processed through an optimizer in smaller and smaller patches, incorporating convergence criteria at each patch level to better resolve artifacts, with a final patch size of 3x 5x 3 mm. To assess whole brain voxel-wise group differences and changes over time in scalar indices used AFNI's 3dttest++ (age, sex, and RMSrel as covariates) with clustsim, a bootstrapping method used to correct for multiple comparisons.
**Results:** Mean age of patients was 26.62 years, 62% of subjects were male. Of the 42 patients included here, 33 completed the study. BPRS total scores decreased significantly after six weeks of treatment, average risperidone dose at that time was 3.73+/-1.72mg. Fractional anisotropy (FA) was significantly decreased in a small area of the medial temporal lobe and mean diffusivity (MD) was significantly increased in the hippocampal part of the cingulum in unmedicated patients (n=40) compared to healthy controls (n=41). Longitudinal analyses showed no changes in FA, MD, RD or white matter macrostructure in healthy controls over time, and no changes in patients after six weeks of treatment with risperidone.

**Discussion:** With state of the art data-processing methods we only found small areas of white matter integrity deficits in our predominantly medication-naïve patients. This is consistent with prior reports of limited white matter alterations at disease onset that may progress with illness duration. Our data suggests that a short-term course of antipsychotic medication may not alter white matter microstructure, but studies with longer follow up durations will be important to determine long term effects of antipsychotic medications.

### O6.7. COMMON NEUROANATOMICAL ABNORMALITIES IN FIRST EPISODE PSYCHOSIS ACROSS SEVERAL INDEPENDENT SAMPLES

Sandra Vieira*[,1], Cristina Scarpazza[1], Benedicto Crespo-Facorro[2], Diana Tordesillas-Gutierrez[2], Victor Ortiz-García de la Foz[2], Esther Setien-Suero[2], Floor Scheepers[3], Qiyong Gong[4], Tiago Reis Marques[1], Robin Murray[1], Anthony David[1], Paola Dazzan[1], Andrea Mechelli[1]

*[1]Institute of Psychiatry, Psychology and Neurosciences, King's College London; [2]Marqués de Valdecilla University Hospital, Instituto de Investigación Valdecilla, CIBERSAM; [3]University Medical Center Utrecht; [4]School of Public Administration, Sichuan University*

**Background:** Structural abnormalities in first episode psychosis (FEP) tend to be subtle and widespread. Most studies investigating structural abnormalities in this clinical population have used small samples, and therefore may be under-powered. In addition, most studies have examined participants at a single research site, and therefore the results may be specific to the local sample investigated. Consequently, findings from existing studies have often been heterogeneous. This study aimed to overcome these issues by testing for neuroanatomical abnormalities in individuals with FEP relative to healthy controls that are expressed consistently across five independent datasets.

**Methods:** Structural Magnetic Resonance Imaging data were acquired from a total of 572 patients with FEP and 502 age and gender comparable healthy controls (HC) at five sites - London (UK), Utrecht (Netherlands), Chengdu (China) and two independent sites at Santander (Spain). Voxel-based morphometry (VBM) as implemented in Statistical Parametric Mapping software (SPM12) was used to investigate differences in gray matter volume (GMV) between the two groups. The statistical analysis was carried out using an analysis of variance (ANCOVA), with diagnostic group and scanning site as factors, and age and gender as covariates of no interest. Neuroanatomical alterations in patients with FEP relative to HC common to the five datasets were identified by comparing the total FEP group against the total HC group, and then using the inclusive masking option (at p<0.05 uncorrected) to identify those regions that survived the comparison between FEP and HC within each dataset. Individual clinical scores from each site were normalised and then used to examine their association with GMV in the total sample. Statistical inferences were made at p<0.05 after family-wise error correction for multiple comparisons.

**Results:** Relative to HC, FEP showed a widespread pattern GMV reduction in fronto-temporal regions bilaterally, including the gyrus rectus, orbitofrontal, temporal, fusiform, precentral and lingual gyri, anterior cingulate and insula as well as in the parietal lobe in the precuneus gyrus. The largest GMV reduction was found in the left gyrus rectus which is part of the inferior frontal lobe. Negative correlations were found between this region and positive symptoms severity (r=-.2, p<.001) and duration of illness (r=-.1, p<.012), but not with negative symptoms (r=.0, p=.991). Patients also showed GMV increases in the temporal gyrus bilaterally, left inferior frontal gyrus and right cerebellum relative to HC.

**Discussion:** This study identified a common pattern of fronto-temporal-parietal reductions in five independent FEP samples; in addition, some of these reductions were more pronounced in patients with more severe positive symptoms and longer duration of illness. This pattern of results suggests the presence of symptom- and stage-dependent neuroanatomical alternations in FEP that are expressed above and beyond site-related differences in recruitment criteria and scanning parameters.

### O6.8. GLUTAMATERGIC DYSFUCTION AND TREATMENT RESPONSE IN MINIMALLY TREATED AND CHRONIC SCHIZOPHRENIA PATIENTS

Elias Mouchlianitis*[,1], Lucy Vanes[1], Erica Barry[1], Krisna Patel[1], Katie Wong[1], Lilla Porfy[1], Sukhi Shergill[1]

*[1]Institute of Psychiatry, Psychology and Neuroscience, King's College London*

**Background:** Glutamatergic dysfunction as a result of NMDA receptor hypofunction has been implicated in antipsychotic treatment-resistant schizophrenia, however its nature in very early stages and chronic stages of the disease is still unknown. Data on glutamate and treatment response are currently limited in two separate studies, one in first-episode patients (Egerton et al., 2012) and one in chronic patients (Mouchlianitis et al., 2016). Here we acquired proton magnetic resonance spectroscopy measures from a large sample of minimally treated first episode and chronic schizophrenia patients, and a group of matched healthy controls. Both first-episode and chronic schizophrenia groups were further stratified by treatment response. This allowed us to investigate glutamatergic dysfunction in both early and later stages of the diseases in relation to treatment-response.

**Methods:** We acquired proton magnetic resonance spectroscopy (1H-MRS) at 3 Tesla from bilateral anterior cingulate cortex (ACC) from 170 participants. 137 participants with a diagnosis of schizophrenia (according to ICD-10 criteria) and 33 healthy controls matched for age, sex, and socioeconomic background consented to participate in this study. The patient sample included 95 minimally treated first-episode patients, with illness duration less than 36 months, of which 65 has shown good response and 26 have shown persistent psychotic symptoms; and a group of 42 chronically-ill patients with illness duration over 3 years. The chronic group was classified into 21 antipsychotic treatment-resistant patients and 21 antipsychotic treatment-responsive patients. 1H-MRS data were analyzed using a standard basis function within LC-Model. Our primary measure was glutamate to creatine ratio (Glu/Cre) and its correlation to N-Acetylaspartic acid to creatine ratio (NAA/Cre).

**Results:** The main new finding is that first-episode patients with persistent psychotic symptoms show significantly higher Glu/Cr and NAA/Cr correlation R(23)=0.76, P<0.001.compared to first-episode patients in remission R(65)=0.43, P<0.00, Fisher's r-to-z, Z=1.97, P<0.05, effect size d=0.48. Compared to healthy controls (who did not show any Glu/Cr to and NAA/Cr correlation R(33)=0.24, P=0.33) the FEP-resistant group showed a significant difference, Z=2.6, P<0.005, representing a large effect size of d=0.87 but not the FEP-responsive group, Z=0.97, P=0.17. Remarkably, when we examined first-episode patients with antipsychotic exposure of less than 6 months, we found an extremely high correlation in the non-responsive group R(5)=0.95, P=0.01, compared to the responsive-group,R(20)=0.44, P<0.05, which reflected a large effect size of d=0.99. Chronically-ill resistant patients showed a significant correlation R(21)=0.48, P<0.05 and responsive trend-level correlation R(21)=0.41, P=0.07, but neither group differed from healthy controls.

**Discussion:** Our study provides the first 1H-MRS evidence for acute metabolic perturbations in glutamatergic neurotransmission in minimally treated schizophrenia patients with persistent psychotic symptoms. These are absent in later stages of the disease for both treatment-resistant and treatment-responsive patients. It is likely that neurodegenerative processes resulting from excitotoxity due glutamatergic dysfuntion are most impactful within the first few months from illness onset. Our data point to the urgent need to identify reliable biomarkers for the prediction of antipsychotic treatment-response and the development of novel interventions to address glutamatergic perturbations at the beginning of their illness.

### O7. Oral Session: Pharmacology

### O7.1. MIDBRAIN DOPAMINE NEURON ACTIVITY CONTROLS THE EFFECTS OF REPEATED KETAMINE ON STRIATAL DOPAMINERGIC FUNCTION

Michelle Kokkinou*[,1], Oliver Howes[2]
[1]*Medical Research Council, London Institute of Medical Sciences;* [2]*Medical Research Council, London Institute of Medical Sciences, King's College London*

**Background:** Schizophrenia is a chronic debilitating disorder which affects about 21 million people worldwide (WHO 2017). Elevated pre-synaptic striatal dopamine synthesis capacity is a robust neurochemical alteration seen in patients with schizophrenia compared to controls, with a large effect size Cohen's d=0.79 (Howes et al., 2012). Ketamine, a non-competitive N-methyl-D-aspartate receptor (NMDAR) antagonist induces psychotomimetic effects in healthy human (Krystal et al., 1994, Stone et al., 2007) and exacerbates psychotic symptoms in patients with schizophrenia (Lahti et al., 1991). For these reasons, it has been used to model the neurochemical alterations seen in schizophrenia such as dopaminergic overactivity (Usun et al., 2013, Kokkinou et al., 2017). However, the effect of sub-chronic ketamine on dopamine synthesis capacity in vivo is not known. Here we investigated the effect of sub-chronic ketamine on striatal dopamine synthesis capacity in vivo using Positron Emission Tomography (PET) imaging and on locomotor activity in the mouse. Moreover, via a chemogenetics approach (Roth 2016) we explored the role of midbrain dopamine neuron activity in mediating ketamine-induced effects.
**Methods:** All procedures were conducted under licence in accordance with the UK Animals (Scientific Procedures) Act of 1986. Mice received a sub-anaesthetic dose of ketamine or an equivalent volume of saline for five consecutive days. Locomotor activity was assessed in the open field test. Moreover, mice received a dynamic 3,4-dihydroxy-6-[(18)F]-fluoro-L-phenylalanine Positron Emission Tomography (PET) scan to assess striatal dopamine synthesis capacity in vivo. Data were analysed using an extended Patlak graphical analysis approach (Walker et al., 2013). Further midbrain dopamine neurons were transduced with an adeno-associated virus vector expressing Gi-coupled (hM4Di) inhibitory receptors under the control of the dopamine transporter (DAT) promoter in DATCre positive mice. Standard immunohistochemistry was used to label dopamine neurons and mCherry expression in dopamine neurons was confirmed using confocal microscopy. Two weeks following the stereotaxic injection of the viral construct, mice received clozapine N-oxide (CNO) to study the effects of inhibiting dopamine neuron firing on locomotor activity and striatal dopamine synthesis capacity in the sub-chronic ketamine model. Data were analysed by two-tailed independent samples t-tests, one-way ANOVA and repeated measures two-way ANOVA followed by Bonferroni post hoc tests where appropriate. $p<0.05$ was considered statistically significant.
**Results:** Sub-chronic ketamine treatment significantly increased striatal dopamine synthesis capacity ($p<0.05$, effect size=1.2) and induced locomotor sensitization ($p<0.01$). hM4Di-mCherry viral construct was successfully transduced in midbrain dopamine neurons with over 98% specificity. Chemogenetic inhibition of midbrain dopamine neurons prevented the ketamine-induced elevation in striatal dopamine synthesis capacity ($p<0.05$, effect size= 0.64) and locomotor sensitization ($p<0.05$).
**Discussion:** Our data show that sub-chronic ketamine results in the elevation in striatal dopamine synthesis capacity and locomotor sensitization and that these effects require midbrain dopamine neuron activation. Furthermore, our data are in support of the hypothesis that NMDA receptor hypofunction on GABAergic interneurons leads to disinhibition of glutamatergic projections and subsequently increase in dopamine neuron activity and dopamine synthesis capacity in projection targets such as the striatum.

### O7.2. BREAKTHROUGH ON ANTIPSYCHOTIC MAINTENANCE MEDICATION IN A CLINICAL COHORT

Jose Rubio*[,1], Christoph Correll[2], Anil Malhotra[2], Majnu John[2], John Kane[1]
[1]*Northwell Health;* [2]*Zucker Hillside Hospital*

**Background:** Antipsychotic drugs are effective in reducing the severity of psychotic symptoms both in the short and long term, and in reducing risk of relapse. However, some patients may develop a relapse of their psychotic symptoms despite continued antipsychotic treatment. Arguably, this phenomenon would be best studied in patients treated with long-acting injectable (LAI) formulations, where the dates of exposure can be confirmed, removing the potential confounder of non-adherence. The characterization of this phenomenon can add important knowledge about the intrinsic efficacy of antipsychotic drugs, potential mechanisms involved in the decrement of their efficacy, and the underlying pathophysiology of psychosis that is not modulated via primarily dopaminergic mechanisms. Despite the implications of this clinical phenomenon, research on breakthrough on antipsychotic maintenance medication (BAMM) in models not confounded by non-adherence has been limited. To date, little is known about the incidence and predictors of BAMM in clinical populations.
**Methods:** We extracted data from a cohort of individuals with a psychotic disorder who were initiated on their first LAI treatment between 2010 and 2015 in the injection clinic at The Zucker Hillside Hospital (New York, USA). We defined BAMM as hospitalization during the period of continuous treatment with LAI, which we used as the primary outcome. LAI treatment was considered continuous for each treatment episode if it was administered following the manufacturer's recommendations for the first 2 months, and until there was a delay in the administration that would have required additional oral supplementation according to the manufacturer instructions (typically >1.5 times the scheduled interval of administration). We measured the cumulative incidence and time to BAMM in individuals with continuous LAI administration, and conducted univariate and multivariate analyses of covariates.
**Results:** A total of 291 episodes of continuous treatment were observed. Of those, 44 (15.1%) led to hospitalization despite continuous treatment with a LAI antipsychotic. The median time to hospitalization was 204.5 days. In the multivariate analysis, the number of hospitalizations prior to onset of LAI treatment (5 vs 2, OR=2.75; 95%CI=1.60–4.72) and time since last hospitalization (4 vs 24.8 weeks, OR 0.70; 95%CI=0.53–0.91) were significantly associated with greater odds of hospitalization during continuous antipsychotic treatment. Individuals who were hospitalized despite continuous treatment were more likely to subsequently be treated with clozapine or ECT (18.2% vs 0, OR=4.93; 95%CI=1.25–19.40). We conducted a mutivariate Cox regression analysis for time to hospitalization and a sensitivity analysis comparing BAMM with individuals that completed 2 years of continuous treatment without being hospitalized and the results were consistent.
**Discussion:** In a clinical cohort, a meaningful proportion of patients with a psychotic disorder treated with LAIs were hospitalized, despite confirmed continuous treatment. The median time to this event occurred about 7 months after onset of LAI treatment, suggesting that these patients had been stable and had reached steady state antipsychotic levels prior to hospitalization. Patients with a more active illness at the time of initiation of LAI treatment were more likely to relapse. These data suggest that more comprehensive investigation of BAMM is feasible; and therefore research focused on this unique group of individuals may provide novel insights into the pathophysiology of psychosis and into the mechanism of action of antipsychotic drugs.

### O7.3. DOSE-RESPONSE META-ANALYSIS TO IDENTIFY THE OPTIMUM AND EQUIVALENT DOSES OF ANTIPSYCHOTIC DRUGS FOR SCHIZOPHRENIA

Stefan Leucht*,[1], Alessio Crippa[2], Nicola Orsini[2], John Davis[3]
*[1]Technical University of Munich; [2]Karolinska Institutet; [3]University of Illinois at Chicago*

**Background:** It is important to better understand the optimum doses and equivalent doses of antipsychotic drugs. Several methods to understand these relationships have been published, but all these methods have weaknesses. In this paper we present a dose-response meta-analysis which theoretically is the most appropriate method for this purpose.
**Methods:** We identified all double-blind, placebo-controlled, studies that compared at least two fixed doses of second-generation antipsychotic drugs or haloperidol in people with acute schizophrenia or with predominant negative symptoms. For this purpose, we searched multiple electronic databases, the website of the FDA, and the clinical trial database clinicaltrials.gov. The method applied was dose response meta-analyses with a spline model. The outcome was the reduction of the PANSS or BPRS total score from baseline or – for negative symptoms - a negative symptom scale. With this method we identified 95% effective doses (these have also been called “near-to-maximum” doses). We applied linear splines to examine whether the dose-response curves had already reached a plateau. Moreover, the identified dose-response relationships of each drug were used to derive risperidone equivalent doses.
**Results:** We identified 67 randomized-controlled trials that were eligible. The following 1mg risperidone equivalent doses were identified: amisulpride 86.6mg/day, aripiprazole 1.9mg/day, asenapine 2.4mg, brexpiprazole 0.56mg clozapine 91mg, haloperidol 1.01mg, iloperidone 3.2mg, lurasidone 23.5mg, olanzapine 2.4mg, paliperidone 13.4/2.1, quetiapine 77mg, risperidone 1mg, sertindole 3.6mg, ziprasidone 30mg.
**Discussion:** From a conceptual point of view, dose-response meta-analysis is the most appropriate method to identify maximally effective doses and equivalent doses. The results of this meta-analysis will be compared with other published methods to define dose-response, in particular the minimum-effective dose method, the classical mean dose method, the daily-defined-doses (DDD) method and expert consensus methods. The results of this analysis are likely to provide information with impact for treatment decisions.

### O7.4. SYSTEMATIC REVIEW, META-ANALYSIS AND META-REGRESSION OF PREDICTORS OF PLACEBO RESPONSE IN ACUTE SCHIZOPHRENIA

Claudia Leucht*,[1], Anna Chaimani[2], Stefan Leucht[1]
*[1]Technical University of Munich; [2]Paris Descartes University*

**Background:** The drug-placebo differences (“effect sizes”) in acute phase, randomised, double-blind trials have become smaller and smaller over the decades. In a recent meta-regression analysis, it had been shown that the degree of placebo response is the strongest predictor of drug-placebo differences. Thus, the open question now was what the predictors of placebo response are.
**Methods:** Placebo-controlled, randomised, double-blind trials that compared any licensed antipsychotic drug with placebo were searched in multiple electronic databases, the website of the Food and Drug Administration and the clinical trial database ClinicalTrials.gov. The mean change from baseline of the PANSS or the BPRS total score from baseline to endpoint in the placebo-groups was extracted from each identified trial. The outcome was the degree of placebo response measured by the BPRS or PANSS change from baseline to endpoint. 24 patient-, and study design related parameters were analysed as potential predictors of placebo response in univariate and multivariate meta-regression analyses.
**Results:** Of 167 included RCTs 99 provided the necessary data. In univariate analyses more recent publication year, larger sample size (total number of participants and sites), use of PANSS rather than the BPRS, studies conducted outside the US or mixed, shorter wash-out phases and shorter study duration, lower participant mean age and lower mean duration of illness were associated with higher placebo-response.
**Discussion:** This meta-regression included approximately two times more studies than previous attempts to resolve this issue and it is therefore the to date by far largest analysis of this kind. Multiple potential moderators of placebo response were identified. Importantly, these moderators of placebo response were not identical with those identified in a previous analysis[1] as significant moderators of drug-placebo differences in the same dataset. Thus, different factors appear to play a role in this complex area.
**References:**
1. Leucht S, Leucht C, Huhn M, Chaimani A, Mavridis D, Helfer B, Samara M,Rabaioli M, Bächer S, Cipriani A, Geddes JR, Salanti G, Davis JM. Sixty Years of Placebo-Controlled Antipsychotic Drug Trials in Acute Schizophrenia: SystematicReview, Bayesian Meta-Analysis, and Meta-Regression of Efficacy Predictors. Am J Psychiatry. 2017;174(10):927–942.

### O7.5. LONG-TERM SAFETY AND TOLERABILITY OF BREXPIPRAZOLE IN PATIENTS WITH SCHIZOPHRENIA

Mika Hakala*,[1], Mette Gislum[1], Aleksandar Skuban[2], Stine Meehan[1]
*[1]H. Lundbeck A/S; [2]Otsuka Pharmaceutical Development & Commercialization, Inc.*

**Background:** Long-term maintenance treatment is recommended to control the symptoms of schizophrenia[1,2]; therefore, safety monitoring for longer than the period required to treat an acute exacerbation is warranted. The aim of the present study (Lighthouse extension; NCT01810783) was to assess the long-term safety and tolerability of open-label treatment with brexpiprazole (flexible dose 1–4 mg/day) in adult patients with schizophrenia. Brexpiprazole is a serotonin-dopamine activity modulator that acts as a partial agonist at the serotonin 5-HT1A and dopamine D2 receptors, and as an antagonist at the 5-HT2A and noradrenaline α1B/2C receptors, all with subnanomolar potency. Brexpiprazole is approved in the US as adjunctive therapy to antidepressants for the treatment of major depressive disorder (MDD) and in the US, Australia and Canada as monotherapy for treatment of schizophrenia.
**Methods:** Patients rolled over into this 52-week open-label study from a randomized, double-blind, placebo-controlled, active referenced, Phase 3 study (Lighthouse[3]; NCT01810380). The primary endpoint was safety and tolerability. Efficacy was assessed as an exploratory endpoint using the Positive and Negative Syndrome Scale (PANSS), Clinical Global Impressions – Severity of illness (CGI-S) and Improvement (CGI-I) scales, and the Personal and Social Performance (PSP) scale. Changes from baseline were analyzed using a mixed model repeated measurements (MMRM) approach.
**Results:** 210 patients were enrolled, and 101 (48.3%) completed the study. The mean and mean modal doses of brexpiprazole were 3.07mg/day and 3.18mg/day, respectively; at last visit, 50% of the patients received 4mg/day. Among patients who took ≥1 dose of brexpiprazole, the incidence of discontinuation due to treatment-emergent adverse events (TEAEs) was 17.2%. TEAEs with an incidence of ≥5.0% were schizophrenia

(worsening of the underlying disease; 11.5%), weight increase (10.5%), headache (8.6%), and insomnia (8.1%). Most TEAEs were mild or moderate in severity. The mean increase in body weight from baseline to Week 52 was 2.6kg (observed cases [OC]) and 19.6% of patients had a weight increase ≥7% at any time during the study. There were no clinically relevant findings for either metabolic parameters (lipids and glucose), or for events related to ECGs, vital signs, extrapyramidal symptoms, or prolactin. Patients' symptoms and functioning showed continual improvement; at Week 52, mean change from baseline in PANSS was -6.8 (95% confidence interval [CI]: -9.3, -4.2); CGI-S: -0.4 (95%CI: -0.5, -0.2); and PSP: 4.2 (95%CI: 2.2, 6.1). The CGI-I score at Week 52 was 2.8 (95%CI: 2.6, 3.1). The percentage of responders (reduction of ≥30% from baseline in PANSS total score or a CGI-I score of 1 [very much improved] or 2 [much improved]) increased throughout the study, from 16% (OC and last observation carried forward [LOCF]) at Week 1 to 48% (OC) and 35% (LOCF) at Week 52.

**Discussion:** Treatment with open-label brexpiprazole 1–4 mg/day was generally well tolerated for up to 52 weeks in patients with schizophrenia. Further, long-term treatment with brexpiprazole was associated with continued improvement in efficacy measures and functional outcomes.

**References:**

1. Hasan A et al., World J Biol Psychiatry. 2013;14(1):2–44.
2. Lehman AF et al., Am J Psychiatry. 2004;161(2 Suppl):1–56.
3. Marder SR et al., Acta Neuropsychiatr. 2016; 16:1–13.

### O7.6. META-ANALYSIS OF EFFICACY OF COGNITIVE ENHANCERS FOR PATIENTS WITH SCHIZOPHRENIA-SPECTRUM DISORDERS

Igne Sinkeviciute*[,1], Marieke Begemann[2], Merel Prikken[3], Bob Oranje[3], Erik Johnsen[4], Wan U Lei[5], Kenneth Hugdahl[6], Rune A. Kroken[4], Carina Rau[7], Jolien Jacobs[8], Silvia Mattaroccia[9], Iris E Sommer[10]

*[1]Haukeland University Hospital; [2]University Medical Centre Utrecht, Brain Centre Rudolf Magnus, University Medical Center Groningen; [3]University Medical Centre Utrecht, Brain Centre Rudolf Magnus; [4]Haukeland University Hospital, NORMENT Center of Excellence, University of Oslo, University of Bergen; [5]School of Medicine, Medical Sciences and Nutrition, University of Aberdeen; [6]University of Bergen; Norwegian Center of Excellence for Mental Disorders Research, University of Oslo; Haukeland University Hospital; [7]University of Konstanz; [8]Utrecht University; [9]University of Rome; [10]University of Groningen, University of Bergen*

**Background:** Cognitive impairment is a core feature of schizophrenia, which is predictive for functional outcomes and should therefore be one of the main treatment targets. Pharmacological enhancement of cognition has been a main field of research in the last decades, investigating almost all neurotransmitter systems and reporting positive as well as negative findings. The pathophysiology of cognitive dysfunctions in schizophrenia is complex and many different neurotransmitter systems are involved. This quantitative review provides an overview of studies of pharmacological agents targeting neurotransmitter systems relevant for cognitive deficits in schizophrenia.

**Methods:** In our systematic search we included pharmacological agents targeting glutamatergic, cholinergic, serotonergic, dopaminergic, GABAergic and noradrenergic neurotransmitter systems, and also a miscellaneous group of agents, including modafinil/armodafinil. We evaluated the effects of cognitive enhancers on overall cognitive functioning as well as on seven cognitive domains, including attention/vigilance, processing speed, reasoning, verbal learning and memory, visual learning and memory, working memory, and verbal fluency.

**Results:** In total, 93 studies with 5630 patients were suitable for inclusion in the meta-analysis. The mean sample size was 28.73 (SD=27.13, range=4–203), mean age of the participants was 44.15 years (SD=6.36, as reported by 91 study samples), 68.54% of the sample were men (as reported by 87 study samples), and average illness duration was 15.57 years (SD=6.47, as reported by 63 study samples). Combining all cognitive enhancers across different neurotransmitter systems for the effect on overall cognitive functioning resulted in fifty-one study samples, with a total of 3635 patients. Cognitive enhancers showed a small but significant positive effect size of 0.10 over placebo treatment (p=.023; 95%CI=0.01 to 0.18). Overall, cognitive enhancers showed no positive effects as compared to placebo for the separate domains. When analysing each neurotransmitter system separately, agents acting predominantly on the glutamatergic system showed a small but significant effect size on overall cognitive functioning (Hedges' g=0.19, p=.01), as well as on working memory (Hedges' g=0.13, p=.04). A sub-analysis of acetylcholinesterase inhibitors (AChEI) within cholinergic system showed a small effect on working memory (Hedges' g=0.26, p=.03). No other positive effects of cognitive enhancers as compared to placebo were revealed.

**Discussion:** The current meta-analysis showed very few favorable effects of cognitive enhancers for patients with schizophrenia spectrum disorders. The overall analysis showed small difference between cognitive enhancers and placebo. Most studies were on agents acting on the glutamatergic and the cholinergic system. There is some evidence of positive effects on cognitive functioning for agents acting on glutamatergic system and acetylcholinesterase inhibitors within cholinergic system. There is still a major lack of studies involving agents acting on other systems. Important issues such as dose, treatment duration, including a younger population and subtyping heterogeneous samples should be taken into account for future studies.

### O7.7. COGNITIVE FUNCTIONING FOLLOWING DISCONTINUATION OF ANTIPSYCHOTIC MEDICATION: A SUB-GROUP ANALYSIS FROM THE OPUS II TRIAL

Nikolai Albert*[,1], Lasse Randers[1], Kelly Allott[2], Heidi Jensen[1], Marianne Melau[1], Carsten Hjorthøj[1], Merete Nordentoft[3]

*[1]Mental Health Centre Copenhagen; [2]Orygen, The National Centre of Excellence in Youth Mental Health; [3]Mental Health Centre Copenhagen, University of Copenhagen*

**Background:** The presence of cognitive defects in patients suffering from schizophrenia is well established. While the earlier "Kraepelinian" view was one of deteriorating cognitive functioning, more recent studies have found that cognitive deficits tend to be stable or improving over time. Cognitive impairments are associated with poorer functional outcomes and understanding the factors that influence cognitive functioning is critical for understanding how to improve cognitive and functional outcomes in patients. The effect of antipsychotics medication on cognitive functioning in patients diagnosed with schizophrenia is poorly understood. Some studies of second-generation antipsychotics indicated that they improved cognitive functioning while other studies have found that they decrease the level of cognitive functioning.

**Methods:** We included patients with schizophrenia who were in treatment with antipsychotics 1.5 years (baseline) after initiation of treatment and followed them up 3.5 years later (n=189). At follow-up 60 (32%) had discontinued their antipsychotic treatment and 129 (68%) were still taking antipsychotics. Using the Brief Assessment of Cognition in Schizophrenia (BACS) we assessed cognition at baseline and follow-up.

**Results:** The patients who had discontinued their medication had a higher level of cognitive functioning in all domains at baseline, as well as Global cognitive function (mean z-score -1.50 (SD 1.24) vs. -2.27 (SD 1.30), p<.001). After controlling for relevant confounders (age, sex, baseline functioning and negative symptoms) those who discontinued antipsychotic medication improved significantly more than those who remained on antipsychotic medication during the course of the follow-up on the Token Motor Task (estimated mean change difference -0.46, 95% CI(-0.89; -0.04), p=0.031), the Speed of Processing Domain (estimated mean change difference -0.38, 95% CI(-0.68; -0.08), p=0.012), and Global cognition (estimated mean change difference -0.36, 95% CI(-0.66; -0.07), p=0.016).
**Discussion:** Due to the naturalistic design we cannot conclude on the direction of the relationship between antipsychotic medication and cognition. There is no evidence that discontinuation of medication had a negative effect on cognitive functioning. Rather, we find that that discontinuation of medication was associated with better cognitive functioning.

### O8. Oral Session: Outcome

### O8.1. EXAMINING RELATIONSHIPS BETWEEN PSYCHOTIC EXPERIENCES AND SUICIDAL IDEATION IN ADOLESCENTS USING A NETWORK APPROACH

Daniel Nunez*[,1], Andrés Fresno[1], Claudia van Borkulo[2], Philippe Courtet[3], Víctor Arias[1], Viviana Garrido[4], Johanna Wigman[5]
*[1]Universidad de Talca, Chile; [2]University of Amsterdam; [3]CHU Montpellier, University of Montpellier; [4]Universidad Católica del Maule; [5]University of Groningen, University Medical Center Groningen*

**Background:** Suicide is the second cause one of the leading causes of death in young individuals. Timely and adequate identification of individuals with suicidal ideation could prevent from suicidal behavior. Psychotic experiences (PE) have been shown to increase levels of suicidal ideation (SI) in the general population. Therefore, detailed investigation of the relationship of PE and SI is relevant. However, the exact nature of the relationship between these two phenomena remains unclear, which is intensely debated nowadays. Given both the high complexity of SI and behavior and the fact that its expression has a trans-diagnostic nature, a fruitful approach to gain new insights about its relationships with psychiatric symptoms might be the application of network analysis, which could be helpful to elucidate specific associations existing between PE and SI.
**Methods:** A specific type of network analysis, the Ising model, was used to examine connections between dichotomized questions on psychotic experiences and suicidal ideation in a cross-sectional study with 1685 adolescents from the general population aged 13–18 years. To assess psychotic experiences, we used an item generation deductive method (Hinkin, 1995) of two pre-existing scales that we adapted in previous studies: the Brief Self-report Questionnaire for Screening Putative Pre-psychotic States (BQSPS; Liu et al., 2013), and the Community Assessment of Psychic Experiences—Positive scale (CAPE-P15; Capra et al., 2013). The questionnaire encompassed 15 items addressing the following dimensions: perceptual anomalies (PA; 3 items); bizarre experiences (BE; 6 items); social anxiety (SA; 3 items); and negative symptoms (NS; 3 items). Suicidal ideation (SI) was assessed by six items of the Columbia–Suicide Severity Rating Scale (C-SSRS; Posner et al., 2011), adapted for being used as a self-report questionnaire. Severity of SI was rated on a 6-point ordinal scale in which 1 = wish to be dead, 2 = nonspecific active suicidal thoughts, 3 = thoughts about how to commit suicide, 4 = suicidal thoughts and intentions, 5 = suicidal thought with detailed plan, and 6 = intentions to conduct plan.
**Results:** SI was mostly connected to the PE domains perceptual anomalies (PA) and bizarre experiences (BE), which have higher strength values in the network. Central nodes within these domains, as indexed by higher centrality measures (strength and betweenness) were: auditory experiences (PA1: hearing voices when you are alone), persecutory ideation (BE1: feelings of being persecuted; BE2: conspiracy against you), and social anxiety (SA) (SA1: I cannot get close to people).
**Discussion:** Through a network analytic approach, our results add new insights to previous findings concerning the associations between psychotic experiences and suicidal ideation, suggesting that perceptual anomalies (mainly auditory experiences), social anxiety (being distant to people), and bizarre experiences (paranoid beliefs) are connected in a meaningful way to suicidal ideation in a network of symptoms in a sample of non-help-seeking adolescents. Given the potential advantages of the network analysis to study psychopathology and suicidal behavior, its usage can contribute to a better understanding of the nature of the complex relationships between these phenomena. Future network analysis studies should include additional symptom domains to analyze whether the associations between PE and suicidal behavior are undifferentiated; are specifically and independently associated, regardless of antecedents of mental disorders; or if the associations are not specific, but merely reflect a higher underlying risk of suicidal behavior as a function of psychiatric symptoms or mental distress.

### O8.2. DURATION OF UNTREATED PSYCHOSIS (DUP) IS ASSOCIATED WITH WORSE RESPONSE TO TREATMENT IN ANTIPSYCHOTIC NAÏVE FIRST EPISODE PSYCHOSIS

Daniel Cavalcante*[,1], Bruno Ortiz[1], Mariane Noto[1], Quirino Cordeiro[2], Luccas Coutinho[1], Vanessa Ota[1], Sintia Belangero[1], Rodrigo Bressan[1], Ary Gadelha[1], Cristiano Noto[1]
*[1]Universidade Federal de São Paulo; [2]Santa Casa de Misericordia de São Paulo*

**Background:** Longer duration of untreated psychosis (DUP) predicts poor functional outcomes in patients with schizophrenia. The results are not so clear when addressing effects on specific dimensions of symptoms, which may be due to confounding effects of previous antipsychotic treatment. We investigated the association between DUP, specific dimensions and response to treatment in a 10-week follow up study of patients at First-Episode of Psychosis (FEP) with no previous antipsychotic use.
**Methods:** We assessed 158 antipsychotic naïve individuals with first-episode psychosis, admitted to a psychiatric emergency service. Diagnosis was established according to the Structured Clinical Interview for DSM-IV (SCID). Symptom severity was measured with the Positive And Negative Symptoms Scale (PANSS) and the Clinical Global Impressions Scale (CGI). Functionality was assessed with the Global Assessment of Functioning Scale (GAF). All patients were treated with risperidone and reassessed after 10 weeks of treatment. For analyses, we performed non-parametric correlation tests (Spearman's correlation).
**Results:** At baseline, we did not find a correlation between DUP and symptom severity and functionality. After the follow-up, DUP became significantly correlated to both symptomatic and functional outcomes. DUP showed significant association with PANSS positive score (r=0.282; p=0.008), PANSS negative score (r=0.295; p=0.005), PANSS total score (r=0.258; p=0.017), CGI total (r=0.305; p=0.003) and GAF (r=-0.294; p=0.004). We also found a negative correlation between DUP and response to treatment considering 30% of reduction of PANSS' scores (r=-0.288; p=0.027).
**Discussion:** Our findings support that DUP does not affect the severity of illness at baseline, but modifies the response to treatment and clinical severity after 10 weeks. This finding suggests that longer exposition to psychosis might be involved in biological abnormalities that modulate the response to antipsychotics, which could mediate poor response to treatment.

### O8.3. CLINICAL AND FUNCTIONAL OUTCOMES IN YOUNG ADULTHOOD OF CHILDREN WITH PSYCHOTIC SYMPTOMS: A LONGITUDINAL TWIN COHORT STUDY

Antonella Trotta*[,1], Louise Arseneault[2], Avshalom Caspi[3], Andrea Danese[2], Terrie Moffitt[3], Carmine Pariante[1], Helen Fisher[1]

*[1]Institute of Psychiatry, Psychology & Neuroscience, King's College London; [2]King's College London; [3]King's College London, Duke University*

**Background:** Childhood psychotic symptoms, such as hallucinations and delusions, are relatively common and have been shown to increase risk of psychotic disorders in adulthood. However, less is known about their association with other forms of psychopathology and more broadly with social and occupational functioning during the crucial transition to adulthood. Using a prospective genetically-sensitive birth cohort we investigated associations between age-12 psychotic symptoms and a range of mental health problems and functional outcomes at age 18.
**Methods:** Data from utilized from the Environmental Risk (E-Risk) Longitudinal Twin Study, a birth cohort of 2,232 twins born in 1994–1995 in England and Wales, followed to age 18 with 93% retention. Childhood psychotic symptoms were assessed in private interviews at age 12. At age 18, interviews were conducted to assess psychopathology, social and occupational functioning, physical health, quality of life, risky and offending behaviors.
**Results:** Children with psychotic symptoms were at greater risk of psychotic phenomena, depression, anxiety, and suicide attempts or self-harm in young adulthood than children without such symptoms. They were also more likely to be obese, smoke cigarettes, be lonely, already have children, and report a lower quality of life at age 18 compared with their unaffected peers. These associations held when controlling for sex, age-5 IQ, other psychopathology at age 12, and family environment.
**Discussion:** In our genetically sensitive cohort, we showed strong evidence of continuity between early psychotic symptoms in childhood and persistence of psychotic phenomena to young adulthood. Psychotic symptoms in childhood are also important risk markers for a wide range of non-psychotic disorders and poor functional outcomes and therefore should be carefully assessed and treated to prevent adverse consequences in adulthood.

### O8.4. THE EFFECT OF EARLY MEDICATION DISCONTINUATION ON LONG-TERM CLINICAL OUTCOME IN FIRST EPISODE PSYCHOSIS

Eric Chen*[,1], Christy Hui[1], WC Chang[1], Sherry Chan[1], Edwin Lee[1], William Honer[2]

*[1]University of Hong Kong; [2]University of British Columbia*

**Background:** Clinical decision to dis/continue antipsychotics in patients remitted from first-episode psychosis is important. Existing short-term evidence suggests that patients who discontinued antipsychotics had more relapses. Data on long-term outcomes are lacking; with only one open-label study suggesting better long-term recovery outcome in patients who had early medication discontinuation. We examined the long-term effect of early medication discontinuation in year 2 following first-episode remission for patients with no residual psychotic symptoms.
**Methods:** We followed-up 178 first-episode psychosis patients who participated in a 1-year randomized controlled trial (RCT) on medication discontinuation. Patients were randomized into receiving either a medication maintenance group or a placebo discontinuation group. After the RCT, all patients received usual psychiatric care. Poor long-term clinical outcome was defined as a composite of persistent psychotic symptoms, a requirement for clozapine, or suicide.
**Results:** There were no differences between patients who were included (n=142) and excluded (n=36) from the study with regard to their baseline demographics, clinical and functioning. At 10 years, more patients in the early discontinuation group (35/89, 39%) had poor clinical outcome than patients in the maintenance group (19/89, 21%) (P<0.01). Relapse during the RCT has partly mediated the significant relationship between early medication discontinuation and poor outcome at 10-year.
**Discussion:** Whether to discontinue medication following successful treatment of first episode psychosis is a difficult clinical decision. In first episode psychosis with a full initial response to antipsychotic treatment, continued need for medication is important for the first three years after starting treatment, to prevent relapse, and decrease the risk for a poor long-term outcome.

### O8.5. SCHIZOPHRENIA AND BIPOLAR DISORDER DIAGNOSIS PATTERNS: REAL-WORLD EVIDENCE FROM US CLAIMS DATABASES

Mallik Greene[1], Tingjian Yan[2], Eunice Chang[2], Ann Hartry*[,3], Maelys Touya[3], Maxine Chen[1], Jennifer Munday[4]

*[1]Otsuka Pharmaceutical Development & Commercialization, Inc. [2]Partnership for Health Analytic Research, LLC; [3]Lundbeck; [4]Phar LLC*

**Background:** Schizophrenia and bipolar disorder (BD) are typically understood as separate and non-concurrent psychiatric disorders both in the clinical setting and in the DSM-V and ICD-10 classification systems. However, patients may experience both mood and schizophrenia symptoms simultaneously. Several studies have shown overlap between schizophrenia and BD symptoms, which may lead to diagnostic confusion. Additionally, molecular studies have confirmed that schizophrenia and BD share susceptibility genes. This study explored diagnosis patterns of patients with schizophrenia and/or type I bipolar disorder (BD-I) diagnoses in a real-world setting.
**Methods:** This was a retrospective cohort study using Truven MarketScan® Commercial, Medicaid, and Medicare Supplemental databases from the study period 01/01/2012 and 06/30/2016. Patients were considered to have a diagnosis of schizophrenia if 1 inpatient claim or 2 outpatient claims for schizophrenia were identified within a selected identification period (01/01/2013 and 06/30/2015). BD-I was defined in an analogous way, and the following five mutually exclusive cohorts were defined: 1) schizophrenia (SCZ) alone (cohort I): newly diagnosed with schizophrenia alone (e.g., met the claims-based diagnostic criteria for schizophrenia, but not for BD-I), 2) BD-SCZ (cohort II): met BD-I criteria only in the year prior to meeting the schizophrenia criteria, 3) SCZ-BD (cohort III): met schizophrenia criteria only in the year prior to, or on the same day as, meeting BD-I criteria, 4) BD-SCZ-BD (cohort IV) met BD-I criteria both in the year before and the year after meeting the schizophrenia criteria, and 5) BD alone (cohort V): newly diagnosed with BD-I alone (e.g., met the claims-based diagnostic criteria for BD-I, but not for schizophrenia). Descriptive statistics are reported for all cohorts.
**Results:** Of the 63,725 patients in the final analytic sample, 11.5% (n=7,336) had schizophrenia alone (cohort I), 7.7% (n=4,909) had a dual diagnosis (cohorts II-IV), and 80.8% (n=51,480) had BD-I alone (cohort V). The dual diagnosis patients included 1.0% (n=615) with BD-SCZ (cohort II), 2.8% (n=1,794) with SCZ-BD (cohort III), and 3.9% (n=2,500) with BD-SCZ-BD (cohort IV). Patients with different diagnosis patterns significantly differed in age, gender, and insurance type (p<.001). Considering the dual diagnosis cohorts, 927 received both diagnoses on the same day. Of those occurring on the same day, the majority (n=753) were on claims from the hospital/emergency department setting.

**Discussion:** This analysis of real-world data found a sizable number of patients with dual diagnoses of schizophrenia and BD-I. Among all patients with either BD-I, schizophrenia, or both, about 2/3 as many met the criteria for both disorders as for schizophrenia alone. Fifteen percent of patients who met criteria for both did so on the same day, likely reflecting patients presenting to acute care exhibiting mixed features. A review of medical records would be useful to determine if dual diagnosis is more common than suspected, and claims data should be examined to determine if these patients differ sufficiently from those with a single diagnosis to warrant exclusion from single-disease cohorts.

### O8.6. THE RELATIONSHIP BETWEEN COGNITION AND FUNCTIONAL IMPROVEMENT IN THE CONTEXT OF A PSYCHOSOCIAL INTERVENTION TARGETING SOCIAL DISABILITY IN FIRST EPISODE PSYCHOSIS

Sian Griffiths*,[1], Stephen Wood[2], Renate Reniers[1], Max Birchwood[3]
*[1]University of Birmingham; [2]Orygen, the National Centre of Excellence in Youth Mental Health; [3]Warwick*

**Background:** Whilst Early Intervention Services (EIS) are the 'gold standard' treatment for young people with psychosis, in a recent study of over 1000 First Episode Psychosis (FEP) cases, 66% of individuals were experiencing a high level of poor functioning, despite receiving care under EIS for a period of 12 months (Hodgekins et al., 2015). This highlights the need to develop new interventions to target functional impairments in FEP.
A specialised Social Recovery Cognitive Behavioural Therapy (SRCBT) has been developed which aims to address the underlying factors impeding social recovery, and has shown to be effective at improving structured activity in individuals with established illness and FEP (Fowler et al., 2013). Identifying the factors that contribute to functional change will ensure that targeted psychosocial therapies are being delivered appropriately. Impaired social cognition (SC) and neurocognition (NC) are closely related to poor functioning in psychosis. Exploration of SC and NC pre- and post-intervention will therefore be important to test underlying mechanisms of functional change, and identify individuals who are more likely to benefit from the specialized SRCBT.
**Methods:** This study ran alongside a multi-site proof of concept trial of SRCBT, for individuals with FEP experiencing social disability. Participants (M age = 25 years) had less than 30 hours a week of structured activity before entering the trial. At baseline, 123 participants completed a battery of SC and NC assessments. 59 participants were randomly allocated to the therapy group (SRCBT + EIS), and 64 were randomly allocated to the standard care group (care from an EIS alone). Participants completed a follow-up assessment at 9 months on the same cognitive battery, and a further assessment of their structured activity. The assessors were blind to group allocation. A small sub-sample of participants (N=6) allocated to the SRCBT group underwent functional magnetic resonance imaging (fMRI) scanning pre- and post- SRCBT, to explore any changes in the social brain regions following successful intervention.
**Results:** Regression analyses showed that SC was a significant predictor of treatment response (i.e. improved structured activity). Specifically, those who had better social knowledge at baseline were most likely to benefit from the SRCBT (Wald $\chi^2$ = 4.073; p = .044), accounting for 16% of the overall variance. To further illustrate this, individuals scoring in the top quartile for social knowledge achieved an additional 11 hours on average of structured activity post-intervention.
Furthermore, in the group that underwent fMRI scanning pre- and post - intervention, there were increased activations in the social brain regions, namely the temporo-parietal junction (TPJ), which became more refined and localized by follow-up. There was also a trend for increased signal intensity in the TPJ, with increased structured activity post-SRCBT. Although this was not significant (r = .455; p = .365), there was a moderate strength relationship
**Discussion:** No studies to-date have examined predictors of treatment response to a CBT intervention targeting functional impairment in FEP. These findings have implications for practice where remediation of SC may improve the efficacy of the SRCBT, particularly for individuals who have poorer social knowledge. This study is also the first to provide preliminary insights into a functional brain network associated with improved structured activity in psychosis; however, replication of these findings in a larger sample is needed.

### O8.7. COGNITIVE SUBTYPES IN FIRST-EPISODE PSYCHOSIS AND ASSOCIATION TO TREATMENT RESPONSE

Manuela Russo*,[1], Simone Ciufolini[1], Olesya Ajnakina[1], Tiago Reis Marques[1], Avi Reichenberg[2], Anthony David[1], Marta Di Forti[1], Carmine Pariante[1], Robin Murray[1], Paola Dazzan[1]
*[1]Institute of Psychiatry, Psychology and Neuroscience, King's College London; [2]Icahn School of Medicine at Mount Sinai*

**Background:** Psychotic disorders are characterized by large heterogeneity in clinical presentation, response to treatment and cognitive functioning. Indeed, there is evidence of the presence of cognitive subgroups of patients across affective and non-affective psychosis. However, very little is known about these subgroups in first episode psychosis (FEP) and whether they can be informative about course of illness, particularly response to treatment. The aim of this study is to investigate the number and the pattern of cognitive clusters in FEP, their external validity and association with treatment response at 12-week and 1-year follow up.
**Methods:** The sample was composed by a total of 212 participants including 105 FEP patients from the South London and Maudsley Foundation Trust and 107 Healthy Controls (HC). All participants underwent a comprehensive clinical and neurocognitive battery. Z-score [mean=0, and standard deviation (SD)=1] were created for the whole sample based on the neurocognitive performance of the HCs. Treatment response at 12-week and 1-year follow-up was used to explore potential utility of subtypes in predicting response to treatment. Hierarchical cluster analysis was carried out to determine the number of cognitive clusters in FEP patients. A series of analyses of variance were carried out to determine if FEP clusters differed among each other in relation to demographic and clinical characteristics, level of functioning and from the HC sample in term of cognitive performance. Logistic regression was used to explore whether cognitive clustering was predictive of treatment response at 12-week and 1-year FU.
**Results:** Four cognitive clusters emerged: one with near normal cognition (42.9% of the FEP patients) with a general cognitive score of z=-0.20, one with selected cognitive deficits (14.3%) in the domains of verbal memory, processing speed and executive functions (general cognitive score of z=-0.55); and two severe deficit clusters consisting in one cluster with severe deficits (33.3%; general score of z=-1.48) and the other with a deeply compromised cognitive ability (9.55%; general cognitive score of z=-2.34). There were no significant differences between clusters in terms of clinical features at baseline (including diagnosis, positive and negative symptoms, medication), apart from the level of functioning that was significantly lower in the severely compromised cluster compared to the near normal cognition cluster.
It emerged that majority (about 68%) of the patients from the near normal cognition cluster were responsive to treatment, whilst the majority of the selective and severely impaired clusters did not respond to treatment at 12-week follow-up. There were no significant results with regard to treatment response at 1-year FU.
**Discussion:** Distinct patterns of cognitive impairments exist within FEP that might be characterized by different response to treatment. Clinical presentation at the onset of the illness is not useful in predicting response to treatment later on in the course of the illness, while cognitive functioning might be a more valid indicator. Cognitive stratification could represent a promising way forward to elucidate pathophysiology of psychosis and to provide tailored interventions.

### O8.8. NEUROCOGNITION IN ULTRA-HIGHRISK INDIVIDUALS AND RELATIONSHIP TO TRANSITION TO PSYCHOSIS, DEPRESSIVE DISORDER, AND FUNCTIONING: FINDINGS FROM THE NEURAPRO TRIAL

Kelly Allott*[,1], Patrick McGorry[2], Luke Bolt[3], Barnaby Nelson[2], Connie Markulev[2], Hok Pan Yuen[2], Miriam Schaefer[1], Nilufar Mossaheb[4], Monika Schlögelhofer[4], Stephan Smesny[5], Ian Hickie[6], Gregor Berger[7], Eric Chen[8], Lieuwe de Haan[9], Dorien Nieman[9], Merete Nordentoft[10], Anita Riecher-Rössler[11], Swapna Verma[12], Andrew Thompson[13], Alison Yung[14], G. Paul Amminger[1]

*[1]Orygen, The National Centre of Excellence in Youth Mental Health; [2]Orygen, The National Centre of Excellence in Youth Mental Health, The University of Melbourne; [3]La Trobe University; [4]Medical University of Vienna; [5]University Hospital, Jena; [6]Brain and Mind Centre, University of Sydney; [7]Child and Adolescent Psychiatric Service of the Canton of Zurich; [8]University of Hong Kong; [9]Academic Medical Center, University of Amsterdam; [10]Mental Health Center Copenhagen, University of Copenhagen; [11]University of Basel Psychiatric Clinics; [12]Institute of Mental Health, Singapore; [13]University of Warwick; [14]Institute of Brain Behaviour and Mental Health, University of Manchester*

**Background:** Neurocognitive impairments are a core feature of psychosis and major depressive disorder (MDD) and are associated with poorer functioning outcomes in these illnesses. Individuals at ultra-high risk (UHR) for psychosis have been shown to experience mild but significant cognitive impairments relative to healthy controls. Evidence suggests neurocognitive deficits are useful predictors of transition to psychotic disorder, although there is inconsistency regarding the specific domains implicated. Furthermore, depression is common in the UHR population, but the relationship between neurocognitive impairment and depression in UHR has not been investigated. The aim of this study was to examine neurocognitive functioning in UHR participants at baseline and whether neurocognitive performance is associated with i) transition to psychosis; ii) depressive disorder; and iii) functioning at medium-term follow-up (median 24 months).
**Methods:** Secondary analysis of data collected as part of a multi-centre RCT of omega-3 fatty acids and cognitive behavioural case management (NEURAPRO) for individuals at UHR for psychosis was conducted. Baseline, 6, 12 and 24-month (median) assessments relevant to the current study included the Comprehensive Assessment of the At-Risk Mental State (CAARMS), Structured Clinical Interview for DSM-IV (SCID), Brief Assessment of Cognition for Schizophrenia (BACS), Montgomery–Åsberg Depression Rating Scale (MADRS) and Social and Occupational Functioning Assessment Scale (SOFAS). The analysis was conducted on the 287 UHR participants (129 males, 158 females) who completed these measures. Hierarchical Cox proportional hazards regression was used to examine neurocognitive predictors of transition to psychosis, while hierarchical logistic and linear regressions were used to identify neurocognitive predictors of MDD and functioning, respectively.
**Results:** Mean z-scores at baseline indicated that the UHR participants performed on average a quarter to half a standard deviation below healthy people in most domains (range mean z=-0.24 to -0.47), except for executive functioning (mean z=0.16, SD=1.21). One hundred and nineteen (41.5%) participants met criteria for MDD at baseline. Thirty-eight (13.2%) participants transitioned to psychosis over 24 months. Results showed that poorer Executive (p=.01) and Motor (p=.03) functions were predictive of transition to psychosis over 24 months, after controlling for other clinical and treatment variables. Forty-eight (16.7%) participants met criteria for MDD at 24 months. Faster Processing Speed significantly predicted MDD at 24 months (p=.01), but failed to retain significance after controlling for other factors, with baseline and past MDD history emerging as the strongest predictors of MDD at medium-term follow-up (both p<.001). After adjustment for IQ and baseline functioning, functional outcomes at 6, 12 and 24 months were predicted by baseline Processing Speed.
**Discussion:** These findings suggest that neurocognitive abilities are important predictors of transition to psychosis and functional outcomes within the UHR population, but hold minimal value in predicting MDD after controlling for history of MDD. Further research is needed that examines trajectory of neurocognition over time in UHR and in relation to psychopathology and functioning outcomes.

### O9. Oral Session: Prediction

### O9.1. CANNABIS AND OTHER SUBSTANCE USE DISORDERS PREDICT CONVERSION FROM SCHIZOTYPAL DISORDER TO SCHIZOPHRENIA

Carsten Hjorthoj*[,1], Nikolai Albert[2], Merete Nordentoft[3]

*[1]Copenhagen University Hospital, Mental Health Center Copenhagen; [2]Mental Health Center Copenhagen; [3]Mental Health Centre Copenhagen, The Lundbeck Foundation Initiative for Integrative Psychiatric Research (iPSYCH)*

**Background:** Schizotypal disorder has been linked to conversion to schizophrenia, with a quarter to half of patients converting over a course of two to five years. Substance use disorders are common in patients with schizotypal disorder, and cannabis has been shown to be a risk factor for developing schizotypal disorder. Cannabis has also been linked to an increased risk of schizophrenia. Other substance use disorders, in particular alcohol, may also increase risk of later schizophrenia. These previous results suggest that substance use, in particular cannabis, may predict conversion to schizophrenia in people with schizotypal disorder.
**Methods:** We used the nationwide, unselected Danish registers. The study population was all people born since 1981 in Denmark with incident diagnosis of schizotypal disorder, without previous diagnosis of schizophrenia. Information on substance use disorders was combined from five different registers. Cox regression using time-varying information on substance use disorders and antipsychotic medication, and adjusted for parental history of mental disorders, sex, birth year, and calendar year were used to estimate hazard ratios (HR) and 95% confidence intervals (CI).
**Results:** After two years, 16.3% (95% CI 14.8%-17.8%) of the people with schizotypal disorder had converted to schizophrenia. After 20 years, the conversion rate was 33.1% (95% CI 29.3–37.3) overall, and 58.2% (95% CI 44.8%-72.2%) in those with cannabis use disorders. In fully adjusted models, any substance use disorder predicted conversion to schizophrenia (HR=1.34, 95% CI 1.11–1.63). Dividing by substances, cannabis use disorders (HR=1.30, 95% CI 1.01–1.68), amphetamine use disorders (HR=1.90, 95% CI 1.14–3.17), and opioid use disorders (HR=2.74, 95% CI 1.38–5.45) predicted conversion to schizophrenia. These associations were not explained by concurrent use of antipsychotic medication, functional level before incident schizotypal disorder, or parental history of mental disorders.
**Discussion:** Substance use disorders, in particular cannabis, amphetamines, and opioids, are important predictors of conversion from schizotypal disorder to schizophrenia. However, conversion rates are high even in those without substance use disorders, indicating a need for both universal and substance-targeted prevention in people with schizotypal disorder.

### O9.2. IDENTIFYING PSYCHOTIC SYMPTOMS AND PREDICTING RELAPSE THROUGH SOCIAL MEDIA

Michael Birnbaum*[,1], Asra Rizvi[2], Munmun De Choudhury[3], Sindhu Ernala[3], Guillermo Cecchi[4], John Kane[5]
*[1]Northwell Health-Early Treatment Program; [2]Zucker Hillside Hospital; [3]Georgia Institute of Technology; [4]IBM Research; [5]Northwell Health*

**Background:** The internet and social media provide an unprecedented opportunity to transform early psychosis intervention services. This study aimed to capture concerning patterns of social media activity associated with the onset and persistence of psychotic symptoms.
**Methods:** Facebook and Twitter archives were extracted from over 150 participants with psychotic disorders, mood disorders and healthy controls. Machine learning was used to build classifiers aiming to identify patterns and distinguish between groups.
**Results:** Linguistic analysis of Twitter commentary identified significantly increased use of interpersonal pronouns ($p < 0.001$), decreased emphasis on friendship ($p < 0.001$) and increased emphasis on health ($p < 0.001$) in individuals with psychosis. Preliminary classifiers correctly recognized participants with psychotic disorders (n=62) from healthy controls (n=24) with an average accuracy of 80% and distinguished participants with psychosis from those with mood disorders (n=39) with an average accuracy of 70%. Further analysis identified shifts in language use of participants with psychosis who experience a relapse (n=18) including significant increases in the use of swearing ($p<0.05$), first-person pronouns ($p<0.05$) and negations ($p<0.05$). We additionally identified significant differences in the profile pictures ($p<0.005$) and structure of messages posted ($p<0.005$) by youth with psychosis who experienced a psychotic relapse.
**Discussion:** Identifying markers in social media activity associated with worsening psychotic symptoms offers the prospect that social media may be a clinically useful tool to identify patients in the earliest phases of relapse.

### O9.3. PSYCHOTIC EXPERIENCES IN COMMON MENTAL DISORDERS AND AS CLINICAL MARKERS OF RISK FOR SUICIDAL BEHAVIOUR

Ian Kelleher*[,1]
*[1]Royal College of Surgeons in Ireland*

**Background:** A high risk for suicidal behaviour has long been recognized in psychotic disorders. More recently, research has demonstrated that subclinical psychotic experiences are strong markers of risk for suicidal behaviour. Whether PEs are specific risk markers for suicidal behaviour, beyond the indirect risk resulting from co-occurring psychopathology, remains unclear.
**Methods:** This study used a stratified, multi-stage probability sample of households in England to recruit a nationally representative sample aged 16 years and over (N=7,403). Participants were assessed for psychotic experiences, suicide attempts, common mental disorders and borderline personality disorder/traits.
**Results:** Psychotic experiences were reported by approximately 4% (n=323) of the total sample and were prevalent across the full range of mental disorders: the highest prevalence in non-psychotic disorders was in individuals with agoraphobia, nearly a quarter of whom reported psychotic experiences. Eighteen percent of individuals with social phobia reported hallucinations, as did 17% of individuals with OCD, 14% of individuals with depression, and 11% of individuals with generalised anxiety disorder. Psychotic experiences were risk markers for suicide attempts, regardless of whether they occurred in individuals with a common mental disorder (OR=2.47, 95%CI=1.37–4.43), individuals without a common mental disorder (OR=3.99, 95%CI=2.47–6.43), individuals with high borderline personality disorder traits (OR=2.23, 95%CI=1.03–4.85) or individuals without significant borderline personality disorder traits (OR=2.47, 95%CI=1.37–4.43).
**Discussion:** Psychotic experiences are prevalent across a wide range of (non-psychotic) mental disorders. They demonstrate a strong relationship with suicidal behaviour, beyond that explained by co-occurring mental disorder diagnoses.

### O9.4. PREDICTING SCHIZOPHRENIA: IDENTIFICATION OF MULTIMODAL MARKERS OF DISEASE THROUGH A MACHINE LEARNING APPROACH

Linda Antonucci*[,1], Giulio Pergola[1], Dominic Dwyer[2], Silvia Torretta[1], Maria Teresa Attrotto[1], Raffaella Romano[1], Barbara Gelao[1], Rita Masellis[1], Antonio Rampino[3], Grazia Caforio[4], Giuseppe Blasi[3], Nikolaos Koutsouleris[2], Alessandro Bertolino[3]
*[1]University of Bari "Aldo Moro"; [2]PRONIA Consortium, Ludwig Maximilians Universitat – Munich; [3]University of Bari "Aldo Moro", Bari University Hospital; [4]Bari University Hospital*

**Background:** Diagnosis of schizophrenia is based on a collection of symptoms which are heterogeneous from one patient to the other. Therefore, improving the reliability of this diagnosis is a currently unmet need. Schizophrenia risk is associated with genetic variation and with environmental factors potentially affecting neurodevelopment. Moreover, among the symptoms, cognitive abnormalities are heritable and predate its clinical onset.
Multivariate techniques can leverage the high dimensionality of data in order to study the combined effect of multiple risk factors and symptoms on clinical predictions. The aim of the current study is therefore to assess the predictability of schizophrenia diagnosis applying machine learning techniques to an ensemble of genetic, early environmental and cognitive deficits variables.
**Methods:** 442 subjects (339 healthy controls – HC – and 103 patients with schizophrenia – SCZ) were recruited for the study. Participants underwent a full neuropsychological evaluation (Modality 1, assessment of working memory, verbal fluency, intelligence quotient, attention, speed of processing and cognitive control), a broad environmental assessment (Modality 2, investigation of urbanicity, obstetric complications, developmental anomalies, socio-economic parental status and age of parents at birth) and genome-wide genotyping (Modality 3). Following published procedures, we computed individual risk scores for each of the single nucleotide polymorphisms (SNPs) associated with risk for schizophrenia in the Psychiatric Genomics Consortium (PGC) study. Data from Modalities 1, 2 and 3 entered NeuroMiner v0.998 and underwent preprocessing procedures through scaling, pruning of non-informative variables and imputation of missing values through Euclidean distance-based nearest-neighbor search. Then, these three modalities were included in a Support Vector Machine HC vs. SCZ classification algorithm, which applied decision-based data fusion strategies to integrate the individual predictions of the three modalities in a nested cross-validation framework.
**Results:** Our cross-validated results revealed that Modality 1 (cognition) predicted schizophrenia diagnosis with the highest Balanced Accuracy (BAC, 87.3%) and that the most selected cognitive indices were intelligence quotient scores and attentional abilities. Modality 2 (environment) classified HC and SCZ with a BAC of 67.2%, and the most predictive environmental features were the parental socio-economic status, the presence of developmental anomalies during the first year of life and the age of father at birth. On the other hand, Modality 3 (genetics) predicted schizophrenia diagnosis with BAC=54,1%. The most informative SNPs were FUT9 rs117074560, TCF4 rs72934570 and STAG1 rs7432375. Decision-based fusion combining individual cognitive, environmental and genetic decision scores predicted the classification of SCZ from HC with a 78.9% BAC.

**Discussion:** Our results using a novel machine learning approach suggest that an ensemble of cognitive, early environmental and genetic features can predict schizophrenia with significant accuracy. Our results also give key information on cognitive and environmental factors that can be targeted in early identification programs and offer novel insights about genetic loci that may be prioritized in future investigations of the pathophysiology of the disease. However, the near chance-level predictive ability of the genetic modality alone calls for the implementation and testing of more complex models of interaction between multiple risk factors.

### O9.5. ABERRANT DOPAMINE SYSTEM FUNCTION REVERSED BY THE OREXIN RECEPTOR ANTAGONIST TCS1102 IN A RODENT MODEL OF SCHIZOPHRENIA

Stephanie Perez*[,1], Daniel Lodge[1]
[1]*University of Texas Health Science Center at San Antonio*

**Background:** Aberrant regulation of dopamine system function is thought to contribute to psychosis in schizophrenia patients; however, the brain regions associated with this dysregulation have not been conclusively demonstrated. We have recently demonstrated that medium spiny neurons in the nucleus accumbens (NAc) receive convergent input from the ventral hippocampus (vHipp) and paraventricular nucleus of the thalamus (PVT). Furthermore, inactivation of either the vHipp or PVT is sufficient to reverse aberrant dopamine system function in rodent models of schizophrenia. Using, chemogenetic experiments we now provide conclusive evidence that the thalamic input to the NAc plays a role in the regulation of dopamine neuron activity. These data demonstrate that the vHipp and thalamus (specifically the PVT) work in concert to regulate VTA dopamine neuron population activity. Such data are important as they provide evidence that thalamic abnormalities may contribute to the aberrant dopamine system function observed in schizophrenia and suggest that the PVT may be a novel site for intervention in psychosis. To examine this, we explored the orexin system, which is known to provide a dense innervation of the PVT.
**Methods:** Pregnant Sprague Dawley (SD) rats were treated on gestational day (GD) 17 with either methylazoxymethanol acetate (MAM; 22 mg/kg, i.p.) or saline. For Poly I:C, pregnant dams were treated on GD12 (7.5 mg/kg Poly I:C or saline). Male pups weaned on post-natal day 21 in groups of 2–3 until adulthood (>60 days). For chemogenetic experiments, normal SD rats were bilaterally micro-injected with AAV2 vectors (Addgene) expressing hm3D(Gq)(pAAV-h8yn-HA-hm3D(Gq)-mcherry; 0.5μL) into the PVT or mPFC. Control rats were administered the viral vector lacking the hm3D encoding gene. Prior to testing, CNO (0.75ul; 300uM) was injected into the nucleus accumbens. In vivo extracellular recordings were performed to measure dopamine neuron activity in the VTA. Spontaneously active VTA dopamine neurons were recorded using previously established electrophysiological criteria.
**Results:** NMDA activation of the PVT induces a significant increase in VTA dopamine neuron population activity. MAM- and Poly I:C-treated rats (both verified rodent models of schizophrenia) consistently display aberrant VTA dopamine neuron population activity, which is restored by pharmacological inactivation of the PVT with TTX. Chemogenetic activation of PVT neurons projecting to the mPFC do not affect VTA dopamine neuron activity; however, activation of PVT neurons projecting to the nucleus accumbens induces a significant increase in dopamine neuron population activity. This effect can be replicated in rats that receive microinjections of the endogenous orexin peptide A or B into the PVT. Consequently, dopamine neuron function can be restored in MAM-treated rats that received a systemic injection of the orexin peptide antagonist TCS 1102.
**Discussion:** We now demonstrate that orexin receptors are expressed on PVT neurons projecting to the NAc and may serve as a substrate for pharmacological manipulation of this pathway. Here, we provide evidence that both systemic and intracranial (PVT) administration of the orexin receptor antagonist, TCS1102, can normalize aberrant dopamine system function in a rodent model of schizophrenia. Collectively, these data suggest that targeting orexin signaling in the thalamus, specifically, the PVT, may represent a novel site of intervention for psychosis associated with schizophrenia.

### O9.6. SPECIFIC SYMPTOMS IN ADOLESCENCE PREDICT PSYCHOSIS IN THE NORTHERN FINLAND BIRTH COHORT 1986

Juha Veijola*[,1], Tuula Hurtig[1], Graham Murray[2], Pirjo Mäki[1]
[1]*University of Oulu;* [2]*University of Cambridge*

**Background:** A number of psychological symptoms have been found to predict psychosis. Many studies have found no specificity to separate symptoms predicting non-psychotic psychiatric disorders from those predicting psychotic disorders. We were able to conduct prospective study comparing adolescent symptoms predicting non-psychotic psychiatric disorders and psychotic psychiatric disorders.
**Methods:** Members of the of the Northern Finland Birth Cohort 1986 were asked to fill in PROD-screen questionnaire at age 15–16 years. PROD-screen includes 21 items both measuring positive prodromal symptoms, negative prodromal symptoms and general symptoms.
We were able to follow 6,514 participants using Finnish Hospital Discharge Register detecting new hospital treated mental disorders till 23 years.
**Results:** The highest prevalence of positive symptoms in the PROD-screen were in the group of subjects who developed psychotic disorder (65% over the cut off) compared to subjects who developed non-psychotic disorder (36%; OR 5.7; 95%CI 2.1–15.4, p<0.001, adjusted for parents' psychiatric disorder, family structure, family SES, adolescent's cannabis use and gender), and to subjects without any disorder (27%; adjusted OR 6.5; 2.8–15.0, p<0.001). Respective figures for negative symptoms were 55% in the group of psychotic subjects compared to 30% in subjects with non-psychotic disorder (3.3; 1.4–7.7, p=0.01) and 24% in the 'healthy' (4.1; 1.9–8.6, p<0.001). When comparing separate symptoms in those having psychiatric hospital treatments, we found four positive symptoms and one negative symptom predicting specifically psychotic disorders.
**Discussion:** In this large prospective population sample both positive and negative symptoms in adolescence associated specifically with development of first episode psychosis.

### O9.7. INDIVIDUALIZED LONG-TERM OUTCOME PREDICTION OF PSYCHOSIS IN AN OBSERVATIONAL STUDY: A MACHINE LEARNING APPROACH

Jessica De Nijs[1], Daniel P.J. van Opstal[1], Ronald J. Janssen[1], Wiepke Cahn[1], Hugo Schnack*[,2], GROUP Investigators
[1]*Brain Centre Rudolf Magnus, University Medical Center Utrecht;* [2]*University Medical Centre Utrecht*

**Background:** Schizophrenia and related disorders have heterogeneous outcomes. Predicting long-term psychosis outcome may be helpful in improving treatment decision making. The aim of our study was to develop and validate a long-term outcome prediction model of psychosis in individual patients. Many studies have shown that outcome is related to symptoms, demographic, clinical, cognitive, genetic and environmental data – at the level of correlations. We hypothesized that, using machine learning (ML), it is possible to predict individual long-term outcome based on patterns that are present in these data at baseline. Second, we test if variables that were recently found to be predictive of short-term outcome (European First Episode Schizophrenia Trial (EUFEST), Koutsouleris et al, 2016) can yield accurate long-term outcome predictions in our sample.

**Methods:** This study included 523 patients (mean (SD) age = 27.6 (7.4) year) from the Genetic Risk and Outcome of Psychosis study. The study extensively assessed patients at baseline, 3- and 6-year follow-up. Outcome was defined in two ways: 1) Symptomatic: being in remission (good outcome) or not in remission (poor outcome), according to the Remission Tool (i.e. a consensus definition which defines remission as maintaining core DSM symptoms, based on Positive and Negative Symptom Scale [PANSS] on a low level during ≥6 months); and 2) Functional, using Global Assessment of Functioning (GAF) scale, divided into good (GAF≥65) and poor (GAF <65) outcome. A support vector machine was trained to predict outcome based on (combinations of) the following sets of baseline data: PANSS, clinical and demographic variables, substance use, neurocognitive/ social cognitive tasks, premorbid adjustment, need of care items (CANSAS), extrapyramidal symptoms, genetic features, environmental variables; and the sets of predictors from 4- and 52-week GAF-based outcome prediction models from the EUFEST study. We trained full and leaner models, using recursive feature elimination (RFE). We tested performance of outcome prediction models using nested cross-validation, i.e., predicting outcome in patients not part of the training set.
**Results:** 6-year functional outcome (i.e. GAF status) was best predicted by a multi-modal model based on baseline PANSS, CANSAS, clinical and demographic variables, using RFE: 75% of the patients was correctly predicted. Significant predictions using single-modal models were obtained for baseline PANSS (62.7%), clinical (60.9%) and CANSAS predictors (58.0%). For functional outcome (GAF) at 6 years, also baseline PANSS, clinical and CANSAS related features produced highest accuracies (61.1%, 63.1% and 59.3% resp.). Classification of symptomatic and functional outcome at 3 years yielded comparable results. Replication using the best scoring predictors of 4 and 52 weeks outcome in the EUFEST study resulted in accuracies of 61.5% and 56.5% for remission 3-year outcome; 61.6% and 61.0% for remission 6-year outcome; 60.1% and 57.7% for GAF 3-year outcome; 62.3% and 64.6% for GAF 6-year outcome.
**Discussion:** Our results show that predicting long-term symptomatic and functional outcome can be done with reasonable accuracies of up to 75%. Training a ML algorithm revealed that PANSS, clinical and need of care features predicted our multiple endpoints best. Interestingly, EUFEST predictors included these three types of data as a main part of best performing predictors. We showed that these short-term outcome predictors are, to certain extent (up to 65%), also predictive of long-term outcome. Our study is a promising step in pursuit of personalized medicine applicability in mental care institutes. However, our model needs replication in independent samples.

### O9.8. STRESS AND COGNITIVE FUNCTION AMONG INDIVIDUALS AT CLINICAL HIGH-RISK FOR PSYCHOSIS: FINDINGS FROM THE NAPLS COHORT

Alexis Cullen*,[1], Kristin S. Cadenhead[2], Jean Addington[3], Carrie E. Bearden[4], Tyrone Cannon[5], Barbara A. Cornblatt[6], Daniel Mathalon[7], Thomas H. McGlashan[5], Diana O. Perkins[8], Larry Seidman[9], William S. Stone[10], Ming Tsuang[2], Scott W. Woods[5], Elaine F. Walker[11]
*[1]Institute of Psychiatry, Psychology & Neuroscience, King's College London; [2]University of California, San Diego; [3]University of Calgary; [4]University of California, Los Angeles; [5]Yale University; [6]Zucker Hillside Hospital; [7]University of California, San Francisco; [8]University of North Carolina, Chapel Hill; [9]Harvard Medical School; [10]Harvard Medical School, Beth Israel Deaconess Medical Center; [11]Emory University*

**Background:** Accumulated evidence from non-human animal studies suggests that the prominent deficits in memory and executive function that characterise individuals with psychosis may, at least in part, be due to the effects of stress on the brain regions that support these functions. However, studies of patients with established psychosis have yielded inconsistent findings with regards to the relationship between stress and cognition, and research in high-risk populations is notably lacking. Utilising data from the North American Prodrome Longitudinal Study 2 (NAPLS 2), we aimed to further elucidate the relationship between stress (daily stressors, life events, and childhood trauma) and cognitive function in clinical high-risk (CHR) individuals and healthy controls (HC). We additionally explored the role of potential mediators [hypothalamic-pituitary-adrenal (HPA) axis function] and moderators (group status, sex, family history of illness).
**Methods:** The sample comprised 885 participants (CHR=646; HC=239) who completed measures of stress and cognitive function at the NAPLS 2 baseline assessment. Stress measures included the Daily Stress Inventory and a modified version of the Psychiatric Epidemiology Research Interview Life Events Scale, both of which provided continuous measures of stress exposure (number of events) and distress (subjective feelings of distress). Participants were also interviewed using the Childhood Trauma and Abuse Scale to determine any exposure to childhood trauma (abuse, neglect, and bullying occurring prior to age 16 years). Basal HPA axis activity was determined via salivary cortisol samples obtained at the baseline assessment and standardised scores from selected subtests from the Measurement and Treatment Research to Improve Cognition in Schizophrenia (MATRICS) were used to derive two cognitive domain scores (memory and executive function). To examine relationships between stress and cognitive domain scores, linear regression analyses were performed on standardised variables.
**Results:** Daily stressor exposure, daily stressor distress, and life event exposure exhibited negative quadratic (i.e., inverted U-shaped) associations with both memory and executive function ($P < 0.01$ for all). In contrast, the reverse pattern (i.e., a negative linear relationship and a positive quadratic relationship) was shown in the model for life event distress and memory domain scores ($P < 0.01$) whilst trauma history showed only a trend-level association with poorer memory performance ($P = 0.084$). These relationships, which did not differ across CHR and healthy control groups, were largely unchanged after adjusting for demographic factors and salivary cortisol. Exploratory analyses suggested that trauma exposure and a family history of psychosis may moderate the relationship between daily stressors/ life events and cognitive function.
**Discussion:** In this large sample of predominately CHR individuals, we observed that the association between stress and cognition is complex and differs across stressor types. The negative quadratic associations that we observed for daily stressor exposure, daily stressor distress, and life event exposure imply that whist lower levels of stress may facilitate memory and executive function, there may be a negative impact on cognition when these stressors become more frequent and distressing. Interventions aiming to minimise stress exposure and promote effective coping strategies might feasibly improve cognition in CHR individuals.

### O10. Oral Session: Risk Factors

### O10.1. DISORGANIZED GYRIFICATION NETWORK PROPERTIES DURING THE TRANSITION TO PSYCHOSIS

André Schmidt*,[1], Tushar Das[2], Daniel Hauke[1], Fabienne Harrisberger[1], Lena Palaniyappan[2], Stefan Borgwardt[1]
*[1]University of Basel; [2]University of Western Ontario*

**Background:** There is urgent need to improve the limited prognostic accuracy of psychopathology-based classifications to predict the onset of psychosis in clinical high-risk (CHR) subjects for psychosis. However, as yet no reliable biological marker has been established to differentiate CHR subjects who will develop psychosis from those who will not. This study investigated abnormalities in graph-based gyrification connectome in CHR subjects and patients with first-episode psychosis (FEP) and tested the accuracy of this systems-based approach to predict the transition to psychosis among CHR individuals.

**Methods:** 44 healthy controls (HC), 63 at-risk mental state (ARMS) subjects without later transition to psychosis (ARMS-NT), 16 ARMS subjects with later transition (ARMS-T), and 38 antipsychotic-free patients with FEP were recruited from the specialized clinic for the early detection of psychosis at the Department of Psychiatry, University of Basel, Basel, Switzerland. Gyrification-based structural covariance networks (connectomes) were constructed to quantify global integration, segregation and small-worldness. Extremely randomized trees with repeated, nested cross-validation was performed to differentiate ARMS-T from ARMS-NT individuals. Permutation testing was used to assess the significance of classification performance measures.
**Results:** Small-worldness is reduced in both ARMS-T and FEP patients, secondary to reduced integration and increased segregation in both groups. In addition, we also found that transitivity (segregation) was significantly higher in ARMS-T and FEP groups compared to both ARMS-NT and healthy controls. Using the connectome properties as features, we obtained a high classification accuracy of 90% (balanced accuracy: 81%, positive predictive value: 85%, negative predictive value: 92%.) All performance measures were highly significant as indicated by permutation tests (all $p < 0.01$).
**Discussion:** Our findings suggest that there is poor integration in the coordinated development of cortical folding in patients who develop psychosis. This study further indicates that gyrification-based connectomes might be a promising means to generate systems-based measures from anatomical data that improves individual prediction of psychosis transition in CHR subjects.

### O10.2. PSYCHOTIC EXPERIENCES ARE ASSOCIATED WITH HEALTH ANXIETY AND FUNCTIONAL SOMATIC SYMPTOMS IN PRE-ADOLESCENCE

Martin K. Rimvall*[,1], Cecilia Pihl Jespersen[2], Lars Clemmensen[1], Anne Mette Skovgaard[3], Frank Verhulst[1], Jim van Os[4], Charlotte Ulrikka Rask[2], Pia Jeppesen[1]
*[1]Child and Adolescent Mental Health Center, Mental Health Services, The Capital Region of Denmark; [2]Research Clinic for Functional Disorders and Psychosomatics, Aarhus University Hospital; [3]University of Copenhagen; [4]Maastricht University Medical Centre*

**Background:** Psychotic experiences (PE) in children and adolescents include hallucinations, delusions and thought-disturbances in the absence of psychotic disorders. Psychosis can be viewed on a continuum ranging from subclinical PE throughout the life span, to clinical psychosis syndromes. Psychosis and PE often co-occur with anxiety and depression, and several studies point towards an affective pathway to psychosis.
Health anxiety (HA) is a relatively new concept in child and adolescent psychiatry, characterized by obsessive rumination, with thoughts about suffering from a disease and misinterpretation of benign bodily sensations and changes. HA at age 11–12 years are associated with emotional disorders and functional somatic symptoms (FSS). In adolescence extensive physical changes occur, and it has been suggested that increased bodily awareness in some cases is accompanied aberrantly by anxiety regarding somatic sensations and somatic health.
We hypothesized that PE would be associated with HA and FSS, and that the associations would remain significant after adjustment for general psychopathology, suggesting a particularly strong specific link between these specific psychopathologies over and above the general multidimensionality of psychopathology.
**Methods:** The study population consists of 1572 children from the general population who participated in the 11–12 year follow-up of the Copenhagen Child Cohort 2000 (CCC2000). PE were assessed face-to-face by the Kiddie Schedule for Affective Disorders and Schizophrenia present and life-time version, and were rated dichotomously as either present (likely or definitely) or not present. HA was self-reported using the Childhood Illness Attitude Scale and FSS were self-reported using the Children's Somatization Inventory, Child Report Form, revised. HA and FSS were scored dichotomously into high (high 10%) and low (bottom 90%) scores. The associations between PE and HA + FSS were adjusted for i) general psychopathology, rated by parents, using the Strengths and Difficulties Questionnaire total score, ii) chronic physical conditions assessed by parent report, iii) onset of puberty onset defined by Tanner-stage I vs II-IV and iv) sex.
**Results:** PE were associated with HA (OR 2.91 (CI95% 1.86–4.57)) and FSS (OR 4.61 (CI95% 3.08–6.89)) in univariate analyses. In a mutually adjusted multivariate model which was further adjusted for general psychopathology, puberty, chronic physical conditions and sex, the associations still held significance for both HA (OR 1.73 (CI95% 1.03–2.90)) and FSS (OR 3.39 (CI95% 2.15–5.35)).
**Discussion:** Our study is, to our knowledge, the first to estimate the role of HA and FSS with regard to PE. Our hypothesis, that PE are associated with HA and FSS in pre-adolescence, was confirmed. The statistical effects were reduced, but remained significant after mutual adjustment and adjustment for general psychopathology. This shows that part of the association is confounded by a general load of psychopathology, but also indicates that HA and FSS contribute to PE over and above general psychopathology. Our study warrants further longitudinal studies, exploring if HA and FSS might constitute a specific pathway in psychosis development.

### O10.3. EARLY BRAIN AND COGNITIVE DEVELOPMENT IN CHILDREN AT RISK FOR SCHIZOPHRENIA

Veronica Murphy[1], Sarah Short[2], Emil Cornea[1], Barbara Goldman[1], Gang Li[1], Dinggang Shen[1], John Gilmore*[,1]
*[1]University of North Carolina; [2]University of Wisconsin*

**Background:** Currently, most attempts at early identification and intervention for individuals at risk for schizophrenia focus on the prodromal phase of the illness during adolescence. However, cognitive and other deficits likely arise well before the prodromal phase. Many risk genes for schizophrenia play a role in early brain development, and recent studies indicate that the basic structural and functional networks of the brain are in place by the second year of life. This suggests that schizophrenia likely has origins in prenatal and early childhood brain development, and that early identification and intervention may need to be shifted to this developmental period to have a real impact on the incidence and severity of schizophrenia.
**Methods:** We studied early childhood brain development 25 children of mothers with schizophrenia and 178 control children. Children had a 3T MRI after birth and at 1 and 2 years of age, and global tissue volumes (gray matter, white matter, CSF), ventricle volumes, and cortical thickness and surface area were determined. Children were also assessed with the Mullen Scales of Early Learning at 1 and 2 years.
**Results:** Children at risk for schizophrenia had significantly lower Mullen Composite scores at both age 1 (p=0.0078) and 2 years (p=0.0001) compared to control children. Reductions were present in fine motor, expressive and receptive language scales at both ages. Overall, high-risk children did not differ from controls in global tissue volumes, though there was evidence of a gender effect. Female high-risk children tended to have reduced gray matter volumes after birth and at age 1 year (significant reduction after birth, p =0.018), while males tended have increased gray matter volumes at age 1 and 2 years (significant at 1 year, p = 0.037). Cortical thickness and surface area results tended to reflect the gray matter volume findings. Females had regions of significant cortical surface area reduction after birth, while males had several regions of significant of cortical surface area expansion at 1 year. Males had a few regions of significant changes of cortical thickness after birth.
**Discussion:** In the context of its limitations, this study confirms previous studies that find alterations of very early childhood development in children at risk for schizophrenia. It also indicates that alterations of cortical gray matter are evident in very early childhood, and that there is a gender difference in these alterations, with females having reduced gray matter volumes and males having increased gray matter volumes. Brain structure and cognitive abnormalities associated with risk for schizophrenia are present shortly after birth; future studies may be able to identify very early biomarkers of risk that will not only improve our understanding of how brain abnormalities associated with schizophrenia develop, but also define periods of childhood development that can be targeted with early intervention.

### O10.4. INCREASED RISKS FOR NON-AFFECTIVE PSYCHOTIC DISORDER AND BIPOLAR DISORDER IN AUTISM SPECTRUM DISORDER

Rik Schalbroeck*[,1], Fabian Termorshuizen[2], Jean-Paul Selten[3]
*[1]Maastricht University; [2]Rivierduinen Mental Health Care Institute; [3]Maastricht University, Rivierduinen Mental Health Care Institute*

**Background:** Young adults with autism spectrum disorder (ASD) appear to be at increased risk for non-affective psychotic disorder (NAPD) and bipolar disorder (BD). However, previous studies have mostly examined the co-occurrence of ASD with NAPD and BD, which is problematic given substantial overlap in symptoms between these disorders. As such, previous risk estimates may have been influenced by diagnostic bias (i.e. NAPD/BD symptoms being mistakenly diagnosed as ASD) or selection bias (i.e. individuals being recognized and/or registered with ASD due to the development of NAPD/BD). In the present study, we used longitudinal data from two Dutch psychiatric case registers to obtain more reliable risk estimates for NAPD and BD among young adults with ASD.
**Methods:** ASD cases were followed between ages 16 and 35 (n = 17,234). Kaplan-Meier estimates were used to calculate risks for NAPD and BD. We conducted separate analyses to reduce possible bias, taking into account the age of ASD diagnosis (ASD diagnosed before or after age 16) and sequence of diagnoses (ASD before or after NAPD/BD). We conducted prognostic analyses using Cox regression to examine possible risk factors for NAPD and BD in ASD.
**Results:** ASD cases were at an increased risk for NAPD and BD compared to previously-reported risks in the general population, even when ASD had already been diagnosed at an early age, before a diagnosis of NAPD or BD. Among cases who were diagnosed with ASD at least one year before a diagnosis of NAPD or BD, an estimated 7.90% (95% CI, 6.70–9.31) developed NAPD, whereas 1.35% (95% CI, 0.89–2.04) developed BD, prior to age 36. Prognostic analyses showed that men with ASD were at a relatively greater risk for NAPD, whereas women with ASD were at a greater risk for BD.
**Discussion:** Young adults with ASD are at an increased risk to develop NAPD and BD, which is not only the result of diagnostic or selection bias. More research is necessary to examine possible mechanisms underlying these risks.

### O10.5. ABNORMAL MODULAR ORGANIZATION OF THE FUNCTIONAL CONNECTOME PREDICTS CONVERSION TO PSYCHOSIS IN CLINICAL HIGH-RISK YOUTH

Guusje Collin*[,1], Larry Seidman[2], Matcheri S. Keshavan[3], Zhenghan Qi[4], William S. Stone[5], TianHong Zhang[6], Yingying Tang[6], Martha E. Shenton[7], Jijun Wang[6], Susan Whitfield-Gabrieli[8]
*[1]University Medical Center Utrecht; [2]Harvard Medical School; [3]Massachusetts Mental Health Center, Beth Israel Deaconess Medical Center, Harvard Medical School; [4]University of Delaware; [5]Harvard Medical School, Beth Israel Deaconess Medical Center; [6]Shanghai Mental Health Center, Shanghai Jiao Tong University School of Medicine; [7]Brigham and Women's Hospital, Harvard Medical School, Veterans Affairs Boston Healthcare System; [8]Massachusetts Institute of Technology, McGovern Institute for Brain Research*

**Background:** The first episode of schizophrenia is typically preceded by a prodromal phase characterized by sub-threshold symptoms and declining functioning. Elucidating the neurobiological substrate of prodromal symptoms that progress into overt psychotic illness is crucial to the development of early detection and intervention strategies for schizophrenia. In this study, we performed a functional connectome analysis in a large group of adolescents and young adults at Clinical High Risk (CHR) for schizophrenia. We aim to assess whether, and if so how, baseline connectome organization distinguishes CHR youth that go on to develop psychosis.
**Methods:** This study comprises a total of 251 subjects, including 158 psychotropically-naïve CHR subjects (CHRs) and 93 healthy controls (HCs), who were matched to CHRs on age, gender, and level of education. Prodromal symptoms and cognition were assessed using the SIPS structured interview and MATRICS cognitive battery. Anatomical T1 MRI and resting-state fMRI scans were collected at baseline and processed using Freesurfer v6.0 and CONN v17.d software. For each subject, a functional connectome map was reconstructed consisting of 162 nodes representing 148 cortical regions from the Destrieux atlas and 14 subcortical structures. Functional connectomes were analyzed in terms of modular topology using the Louvain community detection method. Modular network partitions of individual CHRs were compared to a group-averaged HC network using the rand similarity coefficient (SR), providing a measure of the level of (ab)normality of the CHRs' modular partitions. Analysis of covariance (correcting for age- and gender) was used to compare SR levels between CHRs who developed psychosis during follow-up (CHR+; N = 23) as compared to CHRs who did not develop psychosis (CHR-; N = 135). Kaplan-Meier analysis was used to estimate psychosis-free survival functions for CHRs with below- versus above-average SR, which were compared using log-rank tests. Cox regression analysis was used to assess how baseline connectome organization and clinical measures (i.e., demographics, symptoms, IQ) predicted time to conversion.
**Results:** Modular community detection in HCs yielded five major modules including a posterior 'visual', central 'sensorimotor', medial frontoparietal 'default-mode', lateral frontoparietal 'central-executive', and inferior 'limbic' module. Modular connectome organization of CHR+ was significantly less similar to HCs than CHR- ($F(1,154) = 7.14$, $p = 0.008$). A region-specific analysis to identify which regions contributed most to aberrant modular connectome organization in CHR+ showed that superior temporal (including STG), medial temporal (including amygdala), and ventromedial prefrontal regions were most abnormal in terms of their modular assignment. Psychosis-free survival functions of CHRs with low versus high SR were significantly different ($z = 2.5$, $p = 0.013$), with a Hazard ratio of 3.3 indicating an over 3-fold relative event rate (i.e., conversion to psychosis) in CHRs with abnormal baseline connectome organization. Cox regression analysis indicated that baseline connectome organization ($z = -2.3$, $p = 0.019$), IQ ($z = -2.7$, $p = 0.007$), and gender ($z = 2.0$, $p = 0.048$) predicted time to conversion.
**Discussion:** This study indicates that abnormalities in functional connectome organization precede the first psychotic episode. Conversion to psychosis was found to be over three times more likely in CHRs with abnormal modular organization of the functional connectome at baseline. Our results suggest that functional connectome reorganization may underlie the gradual manifestation of prodromal symptoms. These findings may contribute to early diagnosis and intervention in schizophrenia.

### O10.6. OLANZAPINE IMPAIRS CENTRAL INSULIN ACTION: EFFECTS ON BODY FUEL PREFERENCE IN RATS

Laura Castellani[1], Jennifer Wilkin[1], Zohra Ahsan[1], Celine Teo[1], Chantel Kowalchuk[1], Adria Giacca[2], Gary Remington[2], Margaret Hahn*[,1]
*[1]Centre for Addiction and Mental Health; [2]University of Toronto*

**Background:** Antipsychotics (APs) remain the cornerstone of treatment in schizophrenia, with increasing use on- and off- label. Olanzapine (OLZ)

is a highly prescribed, high metabolic liability AP. Olanzapine has also been found to shift major fuel preference from carbohydrates to fats (while impairing fat breakdown and decreasing availability of fat as a substrate). The shift toward fat utilization is demonstrated by decreases in respiratory exchange ratio (RER). Notably, APs may alter signaling pathways in insulin-sensitive brain areas, particularly in the hypothalamus, that play a role glucose regulation and energy expenditure.

**Methods:** We investigated the effects of intracerebroventricular (ICV) insulin administration on OLZ-induced disruptions in energy homeostasis. Male Sprague Dawley rats were assigned to 4 treatment groups (ICV-peripheral): Vehicle (VEH)-VEH (n = 5), Insulin (INS)-VEH (n = 7), INS-OLZ (n = 6), VEH-OLZ (n = 5). Following acclimatization to the metabolic cages, rats received injections of INS (10mU) or VEH into the 3rd ventricle, and OLA (3mg/kg) or VEH subcutaneously at the beginning of the light (7AM, t=0) and dark (7PM, t=12h) cycle. Dose of OLZ was chosen based on clinically relevant >65% dopamine (D2) brain occupancy. Dose of ICV insulin was chosen based on established decreases in food intake. Indirect calorimetry was used to calculate RER, and heat production. Cumulative food intake was measured at 12-hour intervals (t=12h and 24 h).

**Results:** Treatment with OLZ reproduced the previously established downward shift in RER during the dark phase (p=0.016), which occurred independently of changes in food intake or heat production. Central insulin also decreased RER (p=0.013), an unexpected finding as insulin has been associated with increased carbohydrate oxidation. This may have been secondary to decreased food intake associated with central INS (p=0.011), leading to a shift towards fat-oxidation characteristic of fasting. Co-administration of OLZ with central INS (INS-OLZ) abolished the treatment effect seen in the INS-VEH group relative to VEH-OLZ, shifting the RER profile to become similar to the VEH-OLZ group. Similarly, when OLZ was co-administered with ICV-INS, the effect of central INS to reduce food-intake was lost. An interaction effect (p=0.007) was observed between central insulin and subcutaneous OLZ treatments on RER, supporting the notion that these compounds may work through independent mechanisms to influence fuel preference.

**Discussion:** Taken together, these findings suggest that: 1) central insulin stimulation alters metabolism but is unable to modulate OLZ-specific associated changes in RER; 2) this is likely occurring due to rapid induction of central insulin resistance by OLZ. Our data thus warrants further investigation into the effects of APs on central insulin sensing, and mitigation strategies (i.e. use of central insulin sensitizers) with the goal of mitigating the metabolic burden of these compounds.

### O10.7. INVESTIGATING THE MECHANISMS UNDERLYING THE BENEFICIAL EFFECTS OF ESTROGENS IN SCHIZOPHRENIA

PJ Michael Deans[1], Carol Shum[1], Leo Perfect[1], Marco Conforti[1], Rodrigo Duarte[1], Sagnik Bhattacharyya[1], Nicholas Brandon[2], Jack Price[1], Deepak Srivastava*[,1]

*[1]Institute of Psychiatry, King's College London; [2]AstraZeneca Neuroscience iMED*

**Background:** Estrogens, in particular 17β-estradiol (estradiol) have repeatedly been shown to exert powerful influences over cognitive function, and in particular, on a range of cognitive behaviours associated with neurodevelopmental disorders. This includes depressive and anxious behaviours as well as learning and memory (including working memory). These cognitive enhancing effects have been shown to be dependent on increases in the number of dendritic spines as well as alterations in glutamate receptor transmission and regulation of synaptic protein expression. Modulation of these synaptic functions can result in long-term increases in synaptic connectivity.

Interestingly, there is growing evidence that estrogenic-based compounds may have a positive effect in the treatment of a number of neuropsychiatric disorders, including schizophrenia. Importantly, recent clinical studies have demonstrated that adjunct treatment with estradiol or the selective estrogen receptor modulator (SERM) raloxifene, ameliorates positive and negative symptoms and improves working memory and attention deficits in male and female schizophrenic patients. However, it has been argued that estrogenic-based compounds are not an effective treatment option owing to potential serve side effects associated with long-term administration. It is, however, of note that the precise mechanisms that underlie the positive effects of estradiol, or estrogenic-based compounds, in this disease are currently unclear. Therefore, determining how estradiol exerts its positive effects in health as well as in disease, will aid in the development of safer and more effective estrogenic-based compounds.

**Methods:** Here, we have used human induced pluripotent stem cell (iPSC)-derived from healthy or patients diagnosed schizophrenic but with no common genetic background to study the potential mechanism that may underlie estrogens beneficial effects in disease. iPSCs were differentiated into young, developing, cortical neurons using well established methods. First, we assessed the ability of estrogens to modulate key neuronal and synaptic structures as well as synaptic and inflammatory genes. Next, we assessed the expression and distribution of synaptic proteins were determined in both healthy iPSC-neurons and patient iPSC-neurons (from 3–6 individuals from each group). Subsequently, using a pharmacological approach, we have explored the ability of estrogens to rescue cellular and molecular deficits in iPSC-neurons derived from schizophrenic patients.

**Results:** Both healthy and patient iPSC differentiated into neuroepithelium, neural progenitors cells and finally into TBR1- and EMX1-positive neurons efficiently. Assessment of synaptic protein expression revealed reduced expression of key synaptic proteins involved in excitatory transmission compared to control lines. When healthy iPSC-neurons were treated with a range of estrogenic compounds, we observed a robust increase in the expression of key synaptic protein including GRIN1 and DGL4. Consistent with previous reports, patient iPSC-neurons displayed reduced synaptic protein expression compared with healthy iPSC-neurons. Critically, when patient iPSC-neurons were treated with 17β-estradiol or raloxifene, we observed an increase in synaptic protein expression to a level similar to that observed in untreated healthy iPSC-neurons.

**Discussion:** These data are the first to demonstrate that estrogens are capable of regulating synaptic proteins in human neurons taken from patients diagnosed with schizophrenia. Collectively, we hope these data will help us understand how estrogens may confer their positive effects in psychiatric disorders.

### O10.8. A PLURIPOTENTIAL AT RISK MENTAL STATE: INITIAL RESULTS

Jessica A. Hartmann*[,1], Barnaby Nelson[1], Spooner Rachael[1], G Paul Amminger[1], Andrew Chanen[1], Aswin Ratheesh[1], Christopher G. Davey[1], Patrick D. McGorry[1]

*[1]Orygen, The National Centre of Excellence in Youth Mental Health*

**Background:** The development of the ultra-high risk (UHR) criteria for psychosis over 20 years ago created a new framework for research into subthreshold states in psychiatry. Since (i) early clinical phenotypes are overlapping and non-specific, and (ii) prevention research faces the challenge of achieving adequate statistical power when focusing on low incidence syndromes such as schizophrenia, we introduce an extension of the UHR approach in order to encompass trans-diagnostic targets. The 'CHARMS' (Clinical High at Risk Mental State) study aims to validate a set of pluripotential criteria to prospectively identify help-seeking young people at risk of developing a range of serious mental disorders.

**Methods:** The CHARMS study is a cohort study of help-seeking young people aged 12–25 attending youth mental health services in Melbourne, Australia. New referrals meeting the CHARMS criteria are allocated to the CHARMS+ group; referrals under CHARMS threshold are allocated to CHARMS- (control) group. Transition status and clinical/functional outcomes are re-assessed at 6 and 12 months. The CHARMS criteria consist of subthreshold states for psychosis, mania, severe depression and borderline personality disorder. A range of clinical predictors, including anxiety, stress, sleep/circadian disturbance, and cognitive biases are being assessed as well.
**Results:** To date, a sample of N=73 participants have been recruited: N=49 (67%) met CHARMS criteria (CHARMS+) at baseline with N=24 (33%) allocations to the control group (CHARMS-). Of these, N=48 participants have been followed up to 6 months and a sample of N=35 has been followed up to 12 months. At 6 months, 32% of the CHARMS+ group have transitioned to a full-threshold mental disorder which increased to 37% at 12 month follow-up. 0% of the CHARMS- control group has transitioned.
**Discussion:** Our initial results indicate that the CHARMS criteria can be applied in the context of a youth mental health service and validly identify help-seeking young people at substantial risk of progressing to serious mental disorder over a short time frame (within 12 months). This study is the first to introduce and validate a set of clinical criteria to identify a broader 'at risk' patient population, and represents an important advance from the UHR for psychosis approach. It will foster understanding of risk factors and pathogenic mechanisms that drive the onset of severe mental disorder transdiagnostically and introduce a new case identification paradigm for the next generation of preventive intervention trials.

### O11. Oral Session: Services and Other Interventions

### O11.1. A RANDOMISED CONTROLLED TRIAL OF SMARTPHONE ACTIVE SYMPTOM MONITORING IN PSYCHOSIS

Shon Lewis*[,1], Paolo Fraccaro[1], John Ainsworth[1], Matt Machin[1], Caroline Sanders[1], Zhimin He[2], Pauline Whelan[1], Charlotte Stockton-Powdrell[1], Richard Hopkins[1], Til Wykes[3]
*[1]University of Manchester; [2]Aberystwyth University; [3]King's College London*

**Background:** We developed a smartphone-based personalised technology to monitor symptoms in real time and showed good acceptability, reliability and validity for active remote monitoring of symptoms in previous published studies (www.clintouch.com). We report a randomised trial testing its efficacy in improving psychotic symptom control, and its potential as an early warning system for relapse when embedded into the ICT systems of mental health provider organisations, and as a tool for identifying new phenotypes for precision medicine.
**Methods:** Participants with SMI receive a semi-random beep 2–4 times per day on their smartphone app and answer 14 key symptom rating items using a touchscreen slider. Responses are uploaded wirelessly in real time to a central server and build into a graphical readout on the handset, allowing active symptom monitoring and attempts at self-management. We built this into an end-to-end system in two NHS Hospital Trusts (Manchester and South London) to stream data into electronic care records and enable detection by the clinical team of early signs of relapse in people with SMI when key symptoms exceeded a personalised severity threshold. We conducted an open randomised controlled trial of this active symptom monitoring (ASM) using the smartphone app compared to usual management with the aim of assessing: (i) acceptability of continuous monitoring over 3 months; (ii) impact of active self-monitoring on PANSS positive symptoms and Empowerment Rating Scale score assessed at 6 and 12 weeks; (iii) efficiency of detecting early warning signs of relapse. Eligible participants with a DSM5 diagnosis of schizophrenia and related disorders and a history of relapse within the previous two years were included from an early intervention team (early psychosis group) and a community team (chronic psychosis group).
**Results:** Of 181 eligible, 81 were randomised to either active symptom monitoring or management as usual. 90% stayed in the trial for 12 weeks. Of the 38 in the ASM arm who completed 12-week follow up, adherence defined as responding to >33% of alerts was 84%, >50% of alerts was 60%. At 12 weeks, ASM compared to usual management was associated with no difference on empowerment scale. PANSS positive subscale score showed a significant mean reduction in the ASM group over 12 weeks in the early psychosis group (n= 22, planned ANCOVA p<0.02), but no effect in the chronic psychosis group (n=19). Early warning sign alerts generated by the system occurred in 92% of cases and blind comparison with electronic case record data suggested good sensitivity and lower specificity, but with clear indications of how to adjust the gain of the system to improve future event-detection efficiency. Multivariate analyses pointed to the ability of the system to identify clinical subtypes.
**Discussion:** The active smartphone monitoring system is feasible and acceptable over three months in people with schizophrenia and related disorders. It was associated with psychotic symptom improvement in recent onset participants, supporting the notion of improved self-management. When built into clinical management workflows to enable personalised alerts of symptom deterioration, it was shown to have potential use in promoting earlier intervention for relapse.

### O11.2. CHANGES IN PSYCHOPATHOLOGY PREDICT CHANGES IN WORKING ALLIANCE IN FIRST EPISODE PSYCHOSIS

Marianne Melau*[,1], Nikolai Albert[2], Merete Nordentoft[2]
*[1]Mental Health Centre Copenhagen, Copenhagen University Hospital; [2]Mental Health Centre Copenhagen*

**Background:** The cooperative and dynamic relationship between patients and therapist known as Working Alliance, has in two meta-analysis shown to be an important factor for positive outcome in psychotherapy regardless the modality of therapy. Studies investigating the association between working alliance and outcome conducted in cohorts of patients with mental illness treated in a case manager setting has reported an association between a strong working alliance and reduced symptom severity, better social function, adherence to psycho-social treatment.
For this study, we used data from a trial testing the effect of five years of specialized early intervention (SEI) compared to two years of SEI for patients diagnosed with first episode of schizophrenia spectrum disorder. We aimed to study the effect of the intervention on the working alliance and the change in working alliance as a dynamic factor in the two treatment conditions from baseline to follow-up.
When extending specialized early intervention from two to five years' vs transferring to treatment as usual, we hypothesized a change in working alliance and psychopathology favoring the patient in the extended SEI group.
**Methods:** Participants were recruited from SEI teams (OPUS) in Denmark. All newly diagnosed within the schizophrenia spectrum (ICD-10, F2), age between 18 and 35. Participants were included 1 ½ year after initiation of SEI treatment (baseline) and followed up 5 years after initiation of treatment. At both assessments participants were examined with a comprehensive assessment battery including working alliance, psychopathology, social function, cognitive function, adherence to medication and client satisfaction. Assessors were blind to treatment allocation. The primary outcome, working alliance inventory (WAI), was assessed by self-assessment.
A change score was calculated by subtracting the baseline score from the follow-up score. Multivariable linear regression analyses were conducted, corrected for the baseline value of the independent and dependent variable.
**Results:** Of the 289 participants who attended the follow-up interview 258 (89%) had completed the WAI at baseline and follow-up. Participants who were randomized to prolonged SEI had a stable WA from baseline to follow-up, while participants who were randomized to TAU had a mean drop in WA over the same period.
Change in WA was associated with change in negative-, psychotic-, and disorganized symptoms dimension, and social function in the extended OPUS group. In the TAU group, we found that change in WA were negatively associated with change in cognitive function measured with BACS. In both groups, there were an association between the change in WA and change in client satisfaction.

**Discussion:** This indicates that those participants' who continued the extended SEI treatment maintained their experiences of a strong WA with their case manager, while those participants who were transferred to TAU experiences a lower degree of WA with their case manager compared to their time in SEI treatment. Furthermore, the participants who increased on their cognitive functioning were less likely to assess WA positively if they were transferred to TAU.

### O11.3. A LONGITUDINAL ANALYSIS OF THE EFFECTS OF NEUROTICISM AND EXTRAVERSION ON SUBJECTIVE WELL-BEING IN PATIENTS WITH SCHIZOPHRENIA

Floor van Dijk*[,1], Frederike Schirmbeck[1], Lieuwe de Haan[1]
[1]*Academic Medical Centre, University of Amsterdam*

**Background:** One in five patients with a psychotic disorder has a persistent low subjective well-being over three years. This group has a poorer prognosis for social functioning. This presentation reports on the first longitudinal study evaluating whether neuroticism and extraversion influence subjective well-being (SWB) in patients with a schizophrenia spectrum disorder. SWB is generally defined as 'the subjective experience, as constituting aspects of mental or physical state, which patients report regardless of etiological attributions'. It is an independent determinant for recovery in patients with a psychotic disorder. Two cross-sectional studies on quality of life in schizophrenia suggest that personality traits are associated with the way patients value life. If personality traits predict the trajectories of subjective well-being, our results would provide a clinical reference point for patients at risk for a persistent low subjective well-being.

**Methods:** We included 186 patients and 126 healthy control subjects from the Dutch Genetic Risk and Risk and Outcome of Psychosis cohort. SWB was measured with the Subjective Well-being under Neuroleptics-20 (SWN) scale. Assessments took place at baseline, three years and six years follow-up. We used the Five-Factor Inventory to assess neuroticism and extraversion. Positive, negative and depressive symptoms in patients were assessed by the Positive and Negative Symptoms Scale. For controls we used the Community Assessment of Psychic Experiences for investigating subclinical symptoms. By using linear mixed model analyses we investigated the relation between SWB and the personality traits, including the moderating associations of positive, negative, depressive symptoms and a range of psychosocial indicators (among which antipsychotic use and smoking cannabis). An exploratory analysis in the patient sample, investigated the predictive values of personality traits and symptoms at baseline on the course of SWB over 3 and 6 years. Patients were accounted to one of three SWB-trajectories 'stable low', 'low start and improving' and 'stable high'.

**Results:** Mixed model analyses revealed that in patients, high scores of neuroticism and low scores of extraversion were associated with lower SWN-scores: at 3 years: $t = -3.07$ and $t = 4.34$ for $p < 0.05$ and at 6 years: $t = -2.62$, $p = 0.009$ and $t = 3.51$, $p = 0.001$. We found no interaction effect of time and personality traits. Neuroticism and extraversion were related to SWB to the same extent in the control group.

Regarding trajectories over time, we found a stable low SWB in 15.1% of the patients, forming the 'stable low' trajectory group. This group scored highest on neuroticism and lowest on extraversion compared to patients with an increase in SWB or a stable high SWB: neuroticism scores showed post hoc compared mean differences (MD) of 4.25, $p = 0.03$ for the 'low start increasing'-group and MD 10.75, $p < 0.001$ for the 'stable high'-group).

**Discussion:** We found an association between personality traits and subjective well-being regardless of (subclinical) psychotic or depressive symptoms. Extraversion can be regarded as a resilience factor, whereas neuroticism is associated with a persistent low well-being. In patient with a schizophrenia spectrum disorder, neuroticism could be a focus for therapeutic interventions that diminish negative affectivity. Additionally, an assessment of neuroticism and extraversion early in the process of treatment could be considered.

### O11.4. EDUCATION, EMPLOYMENT AND DISABILITY AMONG YOUNG PERSONS WITH EARLY PSYCHOSIS PARTICIPATING IN A COORDINATED SPECIALTY CARE PROGRAM

Thomas Smith*[,1], Jennifer Humensky[1], Jennifer Scodes[1], Melanie Wall[1], Ilana Nossel[1], Lisa Dixon[1]
[1]*Columbia University & New York State Psychiatric Institute*

**Background:** Comprehensive early treatment programs for individuals with early psychosis have demonstrated success internationally, spurring rapid expansion of the model in the United States. Between 2014–2016, U.S. federal funding to states to support Coordinated Specialty Care (CSC) for individuals with early psychosis increased to $50 million annually (Dixon, 2017). New York State (NYS) was an early adopter and has rapidly expanded CSC across the state. This study prospectively evaluated education and employment outcomes over time within NYS's CSC program, OnTrackNY.

**Methods:** Employment and education trajectories were assessed for individuals with early psychosis who had at least one three-month follow-up assessment, from the program's inception in October 2013, through September 2016 (N=325). Rates of Social Security Administration (SSA) disability enrollment were assessed for individuals enrolled from October 2013 to June 2017 (n=679).

Education and employment status was estimated using longitudinal logistic models utilizing generalized estimating equations with an autoregressive covariance structure to account for within-subject correlations over time. To test how education/employment changed over time, pre-specified contrasts were tested from the longitudinal model for the mean change in sequential follow-up visits. A Kaplan-Meier estimator with discrete time to event and censoring at last observed follow-up month with no event was used to estimate the probability of any education/employment by one year after admission and to estimate the risk of disability by two years after admission.

**Results:** Approximately 40% of individuals with early psychosis were engaged in school or work upon enrollment in a CSC program; engagement increased to 80% after 6 months of care. The estimated probability of being employed or in school at some time during the year after admission was 87.9% (95% Confidence Interval (CI)= [82.9, 92.0]). Relative to women, men had significantly lower odds of education/employment. Relative to non-Hispanic whites, individuals who were Asian, Hispanic or Black had lower odds of education/ employment. Relative to individuals who had not yet completed high school, individuals whose highest educational attainment was High School (HS) or GED had lower odds of educational/ employment.

At admission, 2.5% (17/679) clients were receiving SSA disability benefits. The Kaplan-Meier estimates that 18.3% (95% CI= [13.9, 23.9]) of clients followed for two years obtained disability benefits. In bivariate cox regression analyses, individuals with lower (worse) occupational and social functioning scores have significantly greater risk of disability enrollment than individuals with higher scores (in multivariate analysis, only lower occupational functioning remains significant). Age, gender, race, ethnicity, and symptom scores were not significantly associated with disability enrollment.

**Discussion:** This study demonstrates that individuals with early psychosis who receive CSC in non-research community settings achieve significant improvements in education and employment. Gender, race/ethnicity, and baseline education predicted education and employment outcomes, while poorer functioning was associated with risk of SSA disability benefits. CSC teams should make particular efforts to support the work and school goals of individuals who may be more likely to struggle in achieving engagement in work and school.

### O11.5. EFFECTIVENESS OF COORDINATED SPECIALTY CARE FOR EARLY PSYCHOSIS

Britta Galling*[,1], Christoph Correll[2]
*[1]The Zucker Hillside Hospital; [2]The Zucker Hillside Hospital, Hofstra North Shore LIJ School of Medicine*

**Background:** The value of early intervention in psychosis and allocation of public resources has long been debated since outcomes in people with schizophrenia-spectrum disorders have remained suboptimal. Several research programs for early psychosis yielded promising results for team-based, multi-element coordinated specialty care (CSC).
**Methods:** Systematic literature search of PubMed/PsycInfo/Embase/clinicaltrials.gov without language restrictions until 06/06/2017. Random effects meta-analysis of randomized trials comparing CSC versus Treatment as Usual (TAU) in in first episode psychosis or early-phase schizophrenia-spectrum disorders (schizophrenia, psychotic disorder not otherwise specified, schizoaffective disorder, schizophreniform disorder, delusional disorder), calculating standardized mean differences (SMDs) and risk ratios (RRs) for continuous and categorical outcomes as well as prespecified subgroup and meta-regression analyses.
Co-primary outcomes were all-cause treatment discontinuation and ≥1 psychiatric hospitalization during the treatment period. Key secondary outcomes were total symptom improvement, functioning, and work or school involvement.
**Results:** Across 10 trials (n=2,176; age=27.5 ± 4.6 years; male=62.3%; trial duration=16.2 ± 7.4 (range=9–24) months), CSC outperformed TAU at the end of treatment regarding all meta-analyzable outcomes. This included all-cause discontinuation (studies=10, n=2,173, RR=0.70, 95% confidence interval (CI)=0.61–0.80, p<0.001; number-needed-to-treat (NNT)=12.4), ≥1 hospitalization (studies=10, n=2,105, RR=0.74, 95%CI=0.61–0.90, p=0.003; NNT=10.1), total symptom severity (studies=8, n=1,179, SMD=-0.32, 95%CI=-0.47, -0.17, p<0.001), positive symptoms (studies=10, n=1,532, SMD=-0.22, 95%CI=-0.32, -0.13, p<0.001), negative symptoms (studies=10, n=1,432, SMD=-0.28, 95%CI=-0.42, -0.14, p<0.001), general symptoms (studies=8, n=1,118, SMD=-0.30, 95%CI=-0.47, -0.13, p=0.001), depressive symptoms (studies=5, n=874, SMD=-0.19, 95%CI=-0.35, -0.03, p=0.017), functioning (studies=7, n=1,005, SMD=0.21, 95%CI=0.09–0.34, p=0.001), involvement in school/work (studies=6, n=1,743, RR=1.13, 95%CI=1.03–1.24, p=0.012; NNT=17.8), and quality of life (studies=4, n=505, SMD=0.23, 95%CI=0.004–0.456, p=0.046). Superiority of CSC regarding all outcomes was also evident at 6, 9–12, and 18–24 months of treatment (except general symptoms and depression at 18–24 months).
**Discussion:** In early psychosis, CSC is superior to TAU across all meta-analyzable, highly relevant outcomes with small-to-medium effect sizes. These results support the need for funding and utilization of CSC in patients with early-phase psychosis.

### O11.6. WHO GETS IN TO EARLY PSYCHOSIS INTERVENTION SERVICES? A COMPARISON OF SERVICE USERS AND NON-USERS IN HEALTH ADMINISTRATIVE DATA

Kelly Anderson*[,1], Ross Norman[2], Arlene MacDougall[2], Jordan Edwards[2], Lena Palaniyappan[2], Cindy Lau[3], Paul Kurdyak[3]
*[1]University of Western Ontario; [2]Western University; [3]Institute for Clinical Evaluative Sciences*

**Background:** There is a dearth of information on people with first-episode psychosis who do not access specialized early psychosis intervention (EPI) services. With this notable gap in knowledge comes the implicit assumption that nearly all cases of first-episode psychosis are detected and treated by EPI services. We sought to estimate the proportion of incident cases of non-affective psychosis who do not access these services, and to examine factors associated with EPI admission.
**Methods:** Using health administrative data, we constructed a retrospective cohort of incident cases of non-affective psychosis in the catchment area of the Prevention and Early Intervention Program for Psychoses (PEPP) in London, Ontario between 1997 and 2013. This cohort was linked to primary data from PEPP to identify EPI-users. We used multivariate logistic regression to model socio-demographic and service factors associated with EPI admission.
**Results:** Over 50% of suspected cases of non-affective psychosis did not have contact with the EPI program for screening or admission. Our findings suggest a clear gradient by age, with a decreasing likelihood of being treated in the EPI program with increasing age strata (age 46–50 years vs. age 16–20 years: OR=0.03, 95%CI=0.01–0.05). EPI-users are more likely to be male (OR=1.58, 95%CI=1.24–2.01), and less likely to live in areas of socioeconomic deprivation (OR=0.51, 95%CI=0.36–0.73). EPI-users also had a higher odds of psychiatrist involvement at the index diagnosis (OR=7.35, 95%CI=5.43–10.00), had a lower odds of receiving the index diagnosis in an outpatient setting (OR=0.50, 95%CI=0.38–0.65), and had a lower odds of prior alcohol-related (OR=0.42, 95%CI=0.28–0.63) and substance-related (OR=0.68, 95%CI=0.50–0.93) disorders.
**Discussion:** Much of the prior research on EPI services is predicated on the belief that nearly all patients with first-episode psychosis are represented in these services, with little discussion or consideration of people who may be receiving care elsewhere in the health system. We need greater consideration of patients with first-episode psychosis who are not accessing EPI services – our findings suggest this group is sizable, and there may be socio-demographic and clinical disparities in access. Non-psychiatric health professionals could be targeted with interventions aimed at increasing detection and referral rates.

### O11.7. DISCHARGE PLANNING PRACTICES AND FAMILY INVOLVEMENT IN TRANSITIONS TO OUTPATIENT CARE FOLLOWING DISCHARGE FROM HOSPITAL PSYCHIATRIC UNITS

Morgan Haselden*[,1], Thomas Smith[1]
*[1]Columbia University & New York State Psychiatric Institute*

**Background:** Individuals with mood and psychotic disorders treated in hospital psychiatric units have high rates of discontinuing treatment following discharge, a time that poses substantial risks of serious and even life threatening adverse outcomes. Hospital provider care transition practices believed to improve transitions include communication with outpatient providers, scheduling timely appointments for outpatient follow-up care, forwarding case summaries to aftercare providers, and involving family or support persons in discharge planning. While these are standards of care, little is known about how often they are adequately delivered and their impact on post-discharge aftercare adherence.
**Methods:** As part of a larger project looking at over 30,000 hospital admissions of Medicaid patients with serious mental illness, this study examined hospital medical records for 217 admissions at two urban US hospitals. Trained raters reviewed records for evidence of inpatient providers completing discharge planning practices. Medicaid data were used to measure demographics and attendance of seven- and 30-day outpatient appointments.
**Results:** The sample of 217 admissions was 51% male and 82% were adults, with discharge diagnoses including schizophrenia and related disorders (45%), bipolar disorders (28%) and depressive disorders (17%). The average length of stay was 14 ± 13 days with a median of nine days. The medical records showed evidence of inpatient providers communicating with outpatient providers 64% (n=139) of the time. There was evidence of an outpatient appointment scheduled within seven days of discharge for 81% (n=176) of the sample. A case summary was made available to the aftercare provider within one day of discharge for 66% (n=144) of the sample. Records showed that the inpatient team communicated with family members or support persons about the patient's post-discharge treatment plan for 53% (n=114) of the sample, and 36% (n=79) attended a family meeting or therapy session. Rates of attending an aftercare behavioral health appointment were 55% (n=120) at seven days post-discharge and 80% (n=174) for 30 days.
**Discussion:** This study found varying rates of providers completing care transition practices. Only half of the sample had attended an aftercare appointment in the seven days post discharge, however the majority had attended an appointment by 30 days. Planned analyses will present demographic and clinical differences among those who received discharge planning activities and had family involvement. We will examine predictors of attending follow-up care and report the effectiveness of discharge planning practices. Findings will help inform strategies to improve care-coordination and discharge planning for individuals with serious mental illnesses treated in psychiatric hospitals.

### O11.8. PREVALENCE AND PREDICTORS OF INTERVIEW-ASSESSED CLINICAL HIGH-RISK SYMPTOMS IN THE GENERAL POPULATION

Frauke Schultze-Lutter*[,1], Chantal Michel[2]
*[1]Heinrich-Heine University Düsseldorf; [2]University Hospital of Child and Adolescent Psychiatry and Psychotherapy, University of Bern*

**Background:** In clinical samples, symptomatic ultra-high risk criteria and the basic symptom criterion "cognitive disturbances" perform well in predicting psychosis, and best when both approaches are combined. However, little-to-nothing is known about the prevalence, clinical relevance, and moderators of these clinical high risk (CHR) criteria and their constituent symptoms in the community.
**Methods:** Regression analyses involved 2683 community participants (age 16–40 years; response rate: 63.4%). Semi-structured telephone interviews were performed by well-trained psychologists.
**Results:** Lifetime and current CHR symptoms were reported by 21.1% and 13.8% of interviewees. Frequency of symptoms was mostly low, only 2.4% met any CHR criterion. A stepwise relationship underlay the association of the two types of CHR symptoms and criteria with the presence of mental disorders and functional deficits, with odds ratios being highest (7.4–31.8) when ultra-high risk and basic symptoms occurred together. Report of a family history of mental disorder generally increased risk for CHR symptoms. While younger age increased risk for basic symptoms, lifetime substance misuse and trauma increased risk for ultra-high risk symptoms.
**Discussion:** Prevalence of CHR criteria was within the range to be expected from the prevalence rates of psychoses. Clinical relevance of both CHR symptoms and criteria increased in a stepwise manner from basic symptoms via ultra-high risk symptoms to their combined presence, reinforcing the clinical utility of their combined use. The risk factors selectively associated with basic and ultra-high risk symptoms seem to support developmental models relating basic symptoms to neurobiological and ultra-high risk symptoms to psychological factors.

### O12. Oral Session: Socio-Economic/Environment

### O12.1. EXAMINING THE NEUROBIOLOGICAL IMPACT OF CHILDHOOD TRAUMA: AN IMPORTANT ROLE FOR FRONTAL AND INSULAR REGIONS

Marieke Begemann*[,1], Maya Schutte[2], Lucija Abramovic[1], Marco P.M. Boks[1], Neeltje Van Haren[2], Rene C. W. Mandl[2], Roel Ophoff[3], Christiaan H. Vinkers[1], Marc Bohlken[1], Iris Sommer[3]
*[1]University Medical Center Utrecht; [2]University Medical Center Utrecht & Brain Center Rudolf Magnus; [3]University of California, Los Angeles; [9]University Medical Center Groningen*

**Background:** Childhood trauma may increase the risk for psychiatric illness by its negative impact on brain development. Studies investigating the association between childhood trauma and deviations in gray matter volume have shown inconsistent findings, often restricted by a region-of interest approach with a sole focus on the amygdala and hippocampus and without controlling for the presence of psychiatric illness.
**Methods:** First, using a whole-brain approach in a large cross-diagnostic sample (n=554) of healthy individuals and patients with a bipolar type-I or psychotic disorder, we investigated the neurobiological correlates of childhood trauma by evaluating gray matter volume. Follow-up analyses were conducted to evaluate the effect of psychiatric illness. Second, we investigated to what extent these trauma-related structural correlates could be observed in both groups separately (healthy individuals versus patients). Participants were recruited as part of three different studies, all conducted in the University Medical Center Utrecht (the Netherlands) between 2007 and 2016. We included 554 participants: 220 healthy individuals without a psychiatric history, 250 patients with a bipolar-I disorder and 84 patients with a psychotic disorder. Childhood trauma was evaluated with the Childhood Trauma Questionnaire (CTQ-SF). Anatomical T1 MRI scans were acquired at 3T. FreeSurfer was used to assess regional brain morphology.
**Results:** In the total sample, childhood trauma severity was associated with bilateral reductions in frontal and insular gray matter volumes. In the right hemisphere, medial orbitofrontal and superior frontal volume reductions were related to childhood trauma. These associations remained when adjusting for psychiatric illness, with the exception of the right superior frontal subregion. However, when evaluating both groups separately, these structural correlates of childhood trauma were mainly observed in patients. Healthy controls did show trauma related reductions in right medial orbitofrontal region, while this association was not significant in the patient group.
**Discussion:** Our results suggest that gray matter reductions in the frontal and insular regions are important neurobiological correlates of childhood trauma. For future research, a whole brain approach should be applied, as cortical rather than subcortical areas may be the main correlate of childhood trauma contributing to the development of psychopathology.

### O12.2. STICKS AND STONES MAY BREAK MY BONES BUT WORDS INCREASE THE RISK OF PSYCHOTIC EXPERIENCES

Colm Healy*[,1], Mary Clarke[1], Ian Kelleher[1], Mary Cannon[1]
*[1]Royal College of Surgeons in Ireland*

**Background:** There has been a surge of interest into the relationship between psychotic experiences (PEs) and bullying. However, the methods of bullying and impact of bullying varies across individuals and the prevalence may also vary by respondent (parent or children). For this reason, a thorough investigation into this relationship is warranted.
**Methods:** A longitudinal analysis was conducted on waves 1 and 2 (ages 9 and 13) of the nationally representative Growing Up in Ireland study. Data from n=7163 families were included in this study. Information regarding bullying, being a bully, bullying type, reasons for the bullying, the impact of the bullying was collected from the participating child and their primary care giver (PCG) at both waves. Psychotic experiences were reported by the child at the second wave using the Adolescent Psychotic Symptoms Screener.
**Results:** 13.12% of children met validated criteria for psychotic experiences. Based on the PCG's account, 32.89% of those with PEs at age 13 were bullied at age 9 and this was independently associated with PEs even after accounting for bullying at 13 (OR: 1.40, CI: 1.19–1.65). Physical, verbal, electronic bullying and bullying by exclusion were associated with an increased risk of PE. However, in a multivariate analysis only verbal bullying was independently associated with an increased risk of psychotic experiences (OR: 1.56, CI: 1.27–1.93; adjusted for bullying at 13: OR: 1.47, CI: 1.19–1.82). There was a linear relationship between the number of different methods of bullying experienced at 9 and the risk of PEs at 13 (continuous OR: 1.24, CI: 1.14–1.34). Of the reasons for bullying given by the PCG, only ethnicity (OR: 2.36, CI: 1.46–3.80), being a teacher's pet (OR: 2.09, CI: 1.17–3.73) and jealously (OR: 2.28, CI: 1.5–3.39) were significantly associated with PEs. Persistent bullying was associated with a higher risk of PEs relative to their peers (never bullied OR: 2.31, CI: 1.73–3.08; and bullied at one-time point: OR: 1.49, CI: 1.10–2.03).

Based on the child's account, the vast majority of those who report being a bully (13.87%) at age 9 were also bullied (76.48%, OR: 7.04, 5.97–8.31). Both being a bully and being bullied at age 9 were associated with an increased risk of PEs (16.91%, OR: 1.34, CI: 1.09–1.64; and 50.48% OR: 1.71, CI: 1.48–1.98, respectively). In a multivariate analysis only being bullied was independently associated with PEs (OR: 1.68, CI: 1.44–1.96; adjusted for bullying at 13: OR: 1.57, CI: 1.34–1.83). Verbally bullying another was the only method of bullying associated with an increased risk of PEs at 13 (OR: 1.59, CI: 1.06–2.39). Of those reporting being bullied, verbal and written bullying at age 9 were associated with an increased risk of PEs at age 13 (OR: 1.25, CI: 0.97–1.6; and OR: 1.44, CI: 1.05–1.97, respectively). In a multivariate analysis only written bullying was associated with an increased risk of PEs (OR: 1.47, CI: 1.05–2.06; adjusted for bullying at 13: OR: 1.41, CI: 1.01–1.99). The impact of the bullying on well-being was also associated with an increased risk of PEs at 13 (OR: 1.36, CI: 1.09–1.72; adjusted for bullying at 13: OR: 1.30, CI: 1.04–1.63). Persistent bullying was associated with a vastly higher risk of PEs relative to their peers (never bullied: OR: 4.42, CI: 3.44–5.69; and bullied at one time point OR: 2.71, CI: 2.10–3.50).

**Discussion:** Bullying is pervasive in the childhood of those who subsequent report PE. Bullying at age 9, particularly verbal and written bullying methods are risk factors for PEs in adolescence even when controlling for adolescent bullying. Persistent bullying was associated with a vastly higher risk of PEs. Reducing the rates of bullying in childhood may moderate the likelihood of PEs in adolescents.

### O12.3. PROTECTIVE FACTORS FOR PSYCHOTIC EXPERIENCES AMONGST ADOLESCENTS EXPOSED TO MULTIPLE FORMS OF VICTIMIZATION

Eloise Crush[1], Louise Arseneault[2], Terrie Moffitt[3], Andrea Danese[2], Avshalom Caspi[3], Sara Jaffee[4], Helen Fisher*[,1]
*[1]King's College London, Institute of Psychiatry; [2]King's College London; [3]King's College London, Duke University; [4]University of Pennsylvania*

**Background:** Experiencing multiple types of victimization (poly-victimization) during adolescence is associated with onset of psychotic experiences. However, many poly-victimized adolescents will not develop such subclinical phenomena and the factors that protect them are unknown. This study investigated whether individual, family, or community-level characteristics were associated with an absence of psychotic experiences amongst poly-victimized adolescents.

**Methods:** Participants were from the Environmental Risk (E-Risk) Longitudinal Twin Study, a nationally-representative cohort of 2232 UK-born twins. Exposure to seven different types of victimization between ages 12–18 was ascertained using a modified Juvenile Victimization Questionnaire at age 18. Adolescents were also interviewed about psychotic experiences at age 18. Protective factors were measured at ages 12 and 18.

**Results:** Exposure to poly-victimization during adolescence was associated with age-18 psychotic experiences (OR=4.62, 95% CI 3.59–5.94, P<0.001), but more than a third of the poly-victimized adolescents reported having no psychotic experiences (40.1%). Greater social support was found to be protective against adolescent psychotic experiences amongst those exposed to poly-victimization. Notably, social support was also generally associated with a reduced likelihood of age-18 psychotic experiences in the whole sample (along with engaging in physical activity and greater neighborhood social cohesion).

**Discussion:** Increasing social support from friends and family appears to be an important area for preventive interventions targeting adolescent psychotic experiences. Such prevention efforts would be most effectively targeted at poly-victimized adolescents who are at high-risk of developing psychotic phenomena.

### O12.4. SOME OF THE INDIVIDUAL DIFFERENCES IN RISK TO DEVELOP PSYCHOSIS AMONG CANNABIS USERS CAN BE EXPLAINED BY WHERE THEY LIVE AND BY THEIR AGE AT FIRST USE

Marta Di Forti*[,1], Diego Quattrone[1], Giada Tripoli[1], Caterina La Cascia[2], Laura Ferraro[2], Charlotte Gayer-Anderson[1], Craig Morgan[1], Robin Murray[1]
*[1]Institute of Psychiatry, Psychology & Neuroscience, King's College London; [2]University of Palermo*

**Background:** Cannabis use remains the most widely used recreational drug worldwide. Following from several USA states legalisation policies, European countries are reconsidering their cannabis laws. While a significant amount of Epidemiological evidence has reported that cannabis use increases the risk of psychosis it is still unclear: 1) what underpins individual differences in developing a psychotic disorder following cannabis use; 2) if variations in availability of cannabis have affected rate of Psychotic disorders across Europe.

**Methods:** Using detailed data on lifetime pattern of cannabis use from the EUGEI first episode case-control study (N=2300) and the available Incidence rates of Psychosis calculated for each European site of the same study, we aim 1) to estimate if differences in age at first use, especially of high potency cannabis among cannabis users resulted in differences in their probability to develop psychosis across the study sites; 2) to calculate the proportion of new cases of psychosis attributable to early adolescence-high Potency cannabis in the 5 countries; 3) to relate data on prevalence of cannabis use in each study site with the corresponding Incidence rates for psychotic disorders.

**Results:** Cannabis users starting using cannabis at age 15 and younger who live in those EU countries where high potency cannabis is available have the highest probability to develop psychosis, compared to never users (Adj ORs from 2.6–5.9; p<0.01). Moreover, the proportion of new cases of Psychosis attributable to heavy use started in adolescence was between 20% and 37%. Finally, the correlation between lifetime use of cannabis in population controls from the study sites was significantly correlated with the corresponding incidence rates for Psychosis (r=0.6; p<0.001)

**Discussion:** Before Europe rushes into the USA legalisation "moda" more public education effort might need to be invested in reducing the use of high potency type of cannabis among young adolescents. The latter could lead to a significant reduction in the proportion of new cases of psychosis across Europe.

### O12.5. GENETIC AND ENVIRONMENTAL PREDICTORS OF MAIN OUTCOMES IN THE DANISH HIGH RISK AND RESILIENCE STUDY - VIA 7. A STUDY OF 522 7-YEAR-OLD CHILDREN OF PARENTS WITH SCHIZOPHRENIA, BIPOLAR DISORDER OR NEITHER OF THESE DISORDERS

Merete Nordentoft*[,1], Nicoline Hemager[2], Camilla Jerlang Christiani[2], Ditte V. Ellersgaard[2], Aja Greve[3], Ditte Lou Gantriis[4], Birgitte Klee Burton[5], Katrine Spang[6],

Kerstin von Plessen[7], Jens Richardt Møllegaard Jepsen[8], Ole Mors[9], Anne Amalie Thorup[10]
[1]*Mental Health Centre Copenhagen;* [2]*Research Unit at Mental Health Center, Capital Region of Denmark and The Lundbeck Foundation Initiative for Integrative Psychiatric Research (iPSYCH);* [3]*Psychosis Research Unit, Aarhus University Hospital;* [4]*The Lundbeck Foundation Initiative for Integrative Psychiatric Research, Psychosis Research Unit, Aarhus University Hospital;* [5]*Child and Adolescent Mental Health Centre, Mental Health Services Capital Region, Copenhagen;* [6]*Mental Health Services – Capital Region of Denmark, Child and Adolescent Mental Health Centre;* [7]*University Hospital Lausanne;* [8]*Mental Health Services – Capital Region of Denmark, Child and Adolescent Mental Health Centre, The Lundbeck Foundation Initiative for Integrative Psychiatric Research, Mental Health Services - Capital Region of Denmark, Center for Neuropsychiatric Schizophrenia Research and Center for Clinical Intervention and Neuropsychiatric Schizophrenia Research;* [9]*Aarhus University Hospital;* [10]*Child and Adolescent Mental Health Center Capital Region of Denmark*

**Background:** Studies of children born to parents with schizophrenia and affective disorders can allow us to study the processes preceding the manifestation of the disease, and thereby provide a possibility for identifying early amendable risk factors such as poor parenting, deviances in cognitive functioning, and early, subtle signs of psychopathology at a point where preventive intervention can be applied.
**Methods:** The Danish High Risk and Resilience Study - VIA7 is a representative nationwide cohort study of 522 7-year-old children of parents with schizophrenia, bipolar disorder or neither of these disorders recruited during 2013–2015. The sample consists of: 202 children with a parent diagnosed with schizophrenia spectrum psychosis, 120 children with a parent diagnosed with bipolar disorder, and 200 children with neither of the parents treated in mental health services for the above diagnoses.
We have collected blood and saliva samples from the children and their parents and polygenic risk scores were calculated. We have thoroughly assessed the home environment with the instrument HOME. We have assessed main outcomes such as psychopathology, PLIKS, neurocognition and social cognition. We will analyse the influence of genetic and environmental exposures and their interaction.
**Results:** Generally, the children with a familial risk of schizophrenia had lower neurocognitive, social cognitive and neuromotor functioning, more child psychiatric diagnoses, and more severe symptoms compared to control children. In most comparisons, children of parents with bipolar disorder were not different from controls, but in some tests they performed poorer or had more symptoms compared to than control children.
We will present data on genetic and environmental risk factors for these outcomes
**Discussion:** This is the largest high-risk study ever conducted. It is unique that we have access to detailed phenotyping and extensive information on environmental and genetic risk factors. Studies like this can inform about patogenesis and possibilities for future preventive interventions

### O12.6. SUBMISSION WITHDRAWN

### O12.7. RISK OF PSYCHOSIS IN OFFSPRING OF PARENTS WITH A HISTORY OF HOMELESSNESS DURING CHILDHOOD AND ADOLESCENCE: A NATIONWIDE, REGISTER-BASED, COHORT STUDY

Sandra Feodor Nilsson*[,1], Thomas Munk Laursen[2], Carsten Hjorthøj[3], Anne Thorup[4], Merete Nordentoft[5]
[1]*University of Copenhagen;* [2]*Aarhus University, The Lundbeck Foundation Initiative for Integrative Psychiatric Research (iPSYCH);* [3]*Copenhagen University Hospital, Mental Health Center Copenhagen;* [4]*Research Unit, Child and Adolescent Mental Health Center, Capital Region of Denmark and The Lundbeck Foundation Initiative for Integrative Psychiatric Research (iPSYCH);* [5]*Mental Health Centre Copenhagen, The Lundbeck Foundation Initiative for Integrative Psychiatric Research (iPSYCH)*

**Background:** Children and adolescents with deprived backgrounds have high rates of psychiatric problems. Parental and social factors are regarded crucial for children's healthy and positive development but whether children's risk of psychosis in an early age is associated with parental social marginalisation is unknown. We aimed to analyse the association between mothers' and fathers' history of homelessness and the offspring's risk of psychiatric disorders during childhood and adolescence with attention to the specific child and adolescent psychiatric diagnosis: psychosis, as well as to the combined effect of mother's and father's schizophrenia and bipolar disorder and homelessness experiences according to the child's risk of any psychiatric disorder.
**Methods:** We conducted a nationwide, register-based cohort study of 1,072,882 children aged 0−16 years living or being born in Denmark between Jan 1, 1999 and Dec 31, 2015. Parental homelessness was the primary exposure and offspring's risk of psychosis and other psychiatric disorders the outcome. We analysed the association by survival analysis using Poisson regression and incidence rate ratios (IRRs), adjusted for year and offspring characteristics, and additionally adjusted for parental factors (age at offspring's birth and parental psychiatric disorders).
**Results:** In total, 17,238 (2%) offspring had either one or two parents with a history of homelessness, and 56330 (5%) offspring were diagnosed with any psychiatric diagnosis during the study period. Of these, 850 (1.5%) had a diagnosis of psychosis before their 16th birthday. The incidence rate of any psychiatric disorder was 28.2 cases per 1000 person-years (20.7–38.4) in offspring with at least one parent with a history of homelessness and a mother with a schizophrenia or bipolar disorder, compared with 18.3 cases per 1000 person-years (16.8–20.0) in those whose parents had no history of homelessness.
The IRR of psychosis in offspring born to a mother with a history of homelessness was 3.1 (95% CI 1.9–5.0) compared with those whose parents had no history of homelessness. A similar risk was found if both parents had a history of homelessness (IRR 5.4, 95% CI 2.7-1.9), whereas no association was found when only the father had experiences of homelessness. Also after full adjustment including parental psychiatric disorders, an increased risk of psychosis was found in offspring if the mother (IRR 1.8, 95% CI 1.1–3.0) or both parents (IRR 2.9, 95% CI 1.4–5.9) had a history of homelessness. Highest risk was found for attachment disorder when both parents had a history of homelessness (IRR 32.5, 95% CI 24.6−42.9) and substance use disorder when only the mother experienced homelessness (6.9, 95% CI 4.9−9.7). In offspring whose mother had a history of homelessness and a psychiatric disorder, 36% (95% CI 27%−45%) had received a psychiatric diagnosis themselves by the age of 15. If the mother or father had a schizophrenia or bipolar disorder, homelessness experiences did not add further to the increased risk of any psychiatric disorder in offspring.
**Discussion:** Parental homelessness was strongly associated with an increased risk of psychosis and several other severe psychiatric disorders in offspring during childhood and adolescence. These findings have important implications for public health and policy because they suggest a need for improvement in the support of socially marginalised families to help prevent psychiatric illness in offspring. However, our findings also suggested that the risk of any psychiatric disorder in offspring associated with parental homelessness depended on the parental psychiatric diagnoses.

### O12.8. LOWER FAMILY INCOME PREDICTS PSYCHOTIC EXPERIENCES IN A COMMUNITY SAMPLE OF YOUTHS IN BRAZIL: RESULTS FROM A 3-YEAR FOLLOW-UP STUDY

Lais Fonseca*[,1], Luisa von Nielander[2], Felipe Argolo[1], Pedro Pan[1], Rodrigo Bressan[1], Ary Gadelha[3]
*[1]Universidade Federal de Sao Paulo; [2]Federal University of Sao Paulo, University of Sao Paulo Medical School; [3]Laboratório Interdisciplinar de Neurociências Clínicas (LiNC), Universidade Federal de São Paulo*

**Background:** Up to 17% of community youths, from 9 to 12 years old, report subthreshold psychotic experiences (PE). Besides increasing conversion risk to psychotic disorders, PE also predicted suicide attempts and were associated with more severe general psychopathology. Understanding predisposing factors to PE during development can inform on risk to mental disorders, a crucial step to future prevention interventions. Adversity in early life has been associated with psychotic symptoms, and low socioeconomic status (SES) is an established environmental risk factor for several mental disorders. However, few studies investigated the effect of low SES on PE risk in youths, prospectively. This topic is highly relevant in underprivileged countries like Brazil, where large populations are exposed to poverty. We hypothesized that low income at baseline would predict later report of PEs.
**Methods:** We analyzed data from the Brazilian High Risk Cohort Study for Psychiatric Disorders (HRC), in which 2,512 youths (6–12 years old, mean age at baseline 9.7 years, SD = 1.92; 53,1% male) completed the baseline assessment and 2,012, the 3-year follow-up (T1). PE were assessed at each time-point through two sources of information: parental report, using the Child Behavior Checklist (CBCL), and youth self-report, using the positive dimension of the Community Assessment of Psychic Experiences (CAPE). A single latent variable for each time-point was created to encompass both sources of information using Confirmatory Factor Analysis, yielding good model fit. Total family income (T0) was correlated to the psychotic latent variable (T1), controlling for age, gender and baseline report of PE. Then, we investigated how any mental disorder diagnosis and exposure to trauma, two possible confounding factors, affected the results. The Development and Well-Being Assessment (DAWBA) was used to investigate mental disorders diagnoses. No conversion to psychotic disorder was observed at the follow-up. A latent variable encompassing parental and youths reports, measured by Childhood Trauma Questionnaire (CTQ), was used as trauma exposure.
**Results:** The mean family income reported was approximately US$ 394 per month (p25 = US$ 195, p50 = US$ 307, p75 = US$ 516). Income (baseline) and PE (T1) had a negative significant correlation (pcorr = -0.064; df = 2004; p = 0.004). Subjects with any mental disorder reported PE more frequently, either at baseline or at the follow-up. Low income inversely correlated with trauma, whereas trauma was strongly associated with PE and having any mental disorder at both time points. The correlation between income and PE did not remain significant, after controlling for any mental disorder (T1) (pcorr = -0.049; df = 1597; p = 0.050) or trauma exposure (T0) (pcorr = -0.043; df = 2003; p = 0.053).
**Discussion:** Other studies have previously reported a positive correlation between adversity and PE, but few have described a specific association between family income and psychotic experiences. Our additional analyses suggest that this is not specific, considering the influence of non-psychotic mental disorders, and mediated by low-income related exposures, i.e. childhood trauma. Even not surviving multivariate analyses, income could work as a proxy for adversity exposures and may be useful as a tool to define populations at risk for psychotic and non-psychotic mental disorders. We expect that the longer follow-up assessments help us answer the questions raised.

### Poster Session I

### T1. STRESSFUL LIFE EVENTS AND PERCEIVED STRESS IN THE SAMPLE OF PATIENTS WITH FIRST-EPISODE PSYCHOSIS AND HEALTHY CONTROLS: PRELIMINARY RESULTS

Anna Butjosa[1], Regina Vila-Badia[2], Núria Del Cacho[3], Itziar Riera-López de Aguileta[3], Mar Álvarez[4], Daniel Muñoz[4], Carme Saltó[3], Núria Grases[3], Susana Ochoa*[,3], PROFEP Group, Judith Usall[3]
*[1]Parc Sanitari Sant Joan de Déu, University of Barcelona; [2]Fundació Sant Joan de Déu; [3]Parc Sanitari Sant Joan de Déu; [4]Hospital Sant Joan de Déu*

**Background:** Despite the evidence related to the role of major life events and childhood trauma in the development of first-episode psychosis (FEP; Varese et al., 2012; Morgan & Fisher, 2007), there are few studies on environmental exposure to stressful life events (SLEs) and how SLEs might influence the onset of a psychotic disorder, and the role of perceived stress in this population. The proposed analyses will investigate the association between the categories of SLEs (education, work, partner, family, home, legal, finances, social and health) and perceived stress between patients with FEP and healthy controls (HC).
**Methods:** Participants were patients with FEP (n=15) and HC (n=21). This research was part of a longitudinal observational study called the 'PROFEP group' in Catalonia. Stressful life events were assessed with the Questionnaire of stressful life events (QSLE) (Butjosa et al., 2017). We analysed the frequency of the categories of SLEs. Perceived stress was assessed with the Perceived Stress Scale (PSS; Cohen & Williams, 1988).
**Results:** There are more frequency of SLEs in the education ($p<0.05$) and health ($p<0.05$) categories, and perceived stress ($p<0.05$) in FEP sample than HC.
**Discussion:** Results show the relevance of the presence of SLEs (e.g. education and health) and a potent source of perceived stress in FEP sample. Therefore, more studies are needed to evaluate these stressors to apply future psychological interventions in relation to stress management in FEP population. In addition, it would add protective variables in the analyses such as resilience, coping and social support.

### T2. DO ADVERSE LIFE EVENTS AT FIRST ONSET OF AUDITORY VERBAL HALLUCINATIONS INFLUENCE SUBSEQUENT VOICE-CHARACTERISTICS? RESULTS FROM AN EPIDEMIOLOGICAL STUDY

Josef Bless*[,1], Frank Larøi[2], Julien Laloyaux[2], Kristiina Kompus[3], Bodil Kråkvik[4], Einar Vedul-Kjelsås[5], Anne Martha Kalhovde[6], Kenneth Hugdahl[7]
*[1]University of Bergen; [2]University of Bergen, University of Oslo, University of Liège; [3]University of Bergen, University of Oslo; [4]St. Olavs University Hospital; [5]St. Olavs University Hospital, Norwegian University of Science and Technology; [6]Jaeren District Psychiatric Center; [7]University of Bergen, University of Oslo, Haukeland University Hospital*

**Background:** Understanding what happens at first onset of auditory verbal hallucinations (AVHs) is important at both a clinical and theoretical level. Previous studies have focused on age with regard to first onset of AVHs. In the current epidemiological study, we investigated the role of adverse life events (e.g. accidents, divorce, bullying, unemployment) at the time of first onset of AVHs regarding symptom severity and general mental health later in life.
**Methods:** Using data from the Launay-Slade Hallucination Scale (LSHS), we compared participants who reported having experienced at least one adverse life events at first onset of AHVs (Trigger group; N = 76) to those who did not report any specific events at first onset of AVHs (No-trigger group; N = 59) on a large array of variables using Fisher's exact test.
**Results:** Results revealed that the Trigger group experienced the AVHs as more emotional and they were also more troubled by the AVHs compared to the No-trigger group (all $p < 0.01$). Also, the Trigger group more often reported hallucinations in other (non-auditory) sensory modalities (e.g. visual, $p = 0.012$) compared to the No-trigger group. Furthermore, the Trigger group reported poorer mental health in general, and having had more frequent contact with mental health professionals, and also reported more frequently taking medication for mental problems in general (all $p < 0.01$).
**Discussion:** Adverse life events at first onset of AVHs appear to have a negative influence on subsequent voice-characteristics and general mental health, suggesting their presence to be an important factor to take into account when determining the risk for psychosis or other mental disorders. However, future longitudinal studies are needed in order to corroborate these findings.

### T3. METACOGNITIVE BELIEFS IN SEVERE MENTAL DISORDERS

Tiril Østefjells*[,1], Ingrid Melle[1], Sofie R. Aminoff[2], Ole A. Andreassen[1], Akiah O. Berg[3], Roger Hagen[4], Tone Hellvin[1], Trine V. Lagerberg[1], Rachel Loewy[5], June U. Lystad[1], Kristin L. Romm[6], Leiv Sandvik[6], Nasrettin Sönmez[6], Jan Ivar Røssberg[6]
*[1]Oslo University Hospital and Institute of Clinical Medicine, University of Oslo; [2]Oslo University Hospital and Institute of Clinical Medicine, University of Oslo, Akershus University Hospital; [3]Oslo University Hospital and Institute of Clinical Medicine; [4]Norwegian University of Science and Technology; [5]University of California, San Francisco; [6]Oslo University Hospital*

**Background:** Affective dysregulation and psychotic experiences or symptoms often co-occur in the general population as well as in bipolar and psychotic disorders, suggesting a complex interplay. Early trauma is hypothesised to be important for the aetiology of both, and individuals with early traumatic experiences often develop disorders characterised by an admixture of affective and psychotic symptoms. Early emotional abuse seems to be particularly relevant for both disorders. Studies of common factors associated with affective dysregulation and psychosis in bipolar and psychotic disorders could help further theoretical understanding and tailor therapeutic interventions. Metacognitive beliefs – beliefs that outline the importance or consequence of thoughts – have been proposed as one possible common factor. Compared to healthy controls, patients with affective or psychotic disorders hold higher levels of metacognitive beliefs that could be maladaptive. Metacognitive beliefs have been linked to affective and/or psychotic diagnoses and symptoms in these disorders, and to early trauma in general. However, little is known about the specific relationships between symptoms of bipolar/psychotic disorders, early emotional abuse, and metacognitive beliefs.
This project had three objectives: (1) to examine the prevalence of metacognitive beliefs in bipolar and psychotic disorders, compared to controls; (2) explore whether illness-related factors were linked to metacognitive beliefs; (3) examine if symptomatic responses (depression or positive symptoms) to early emotional abuse were mediated by metacognitive beliefs.
**Methods:** Patients with a bipolar or psychotic disorder, and healthy controls, were included through the on-going Thematically Organised Psychosis (TOP) Study in Oslo, Norway. Analyses included t-tests for group comparisons, regression analyses, and regression based mediation pathway analyses where the indirect effects were tested with bootstrapped confidence intervals.

**Results:** Patients with bipolar or psychotic disorders reported higher levels of metacognitive beliefs compared to controls. Metacognitive beliefs were significantly related to depression for all patients. Higher levels of metacognitive beliefs were also related to illness-factors related to a poorer long-term outcome, specifically an earlier age at onset of affective disorder in bipolar disorders, and poorer premorbid social adjustment in psychotic disorders. Metacognitive beliefs significantly mediated the relationship between early emotional abuse and depression. The combination of metacognitive beliefs and depression significantly mediated the relationship between early emotional abuse and positive symptoms. The mediation models explained a moderate amount of the variance in symptoms ($R2 = .21$ and .29) compared to direct models of early emotional abuse impacting on symptomatic responses directly ($R2 = .04$ and .03)

**Discussion:** Our results show that patients with bipolar or psychotic report higher levels of metacognitive beliefs compared to controls, and that such beliefs relate to current symptoms of depression in both patient groups. Our results also suggest that metacognitive beliefs relate to factors present before or at the onset of illness, which are often linked to a poorer long-term outcome in the disorders. Further, our findings suggest that in regards to early emotional abuse, metacognitive beliefs could play a role in an affective pathway to psychosis. Metacognitive beliefs could thus be relevant treatment targets in regards to depression and positive symptoms in bipolar and psychotic disorders.

### T4. IDENTIFICATION OF NEUROANATOMICAL SURROGATE MARKERS OF CHILDHOOD TRAUMA

Theresa Haidl*,[1], Marlene Rosen[1],
Mauro Seves[1], Thorsten Lichtenstein[2],
Anne Ruef[3], Joseph Kambeitz[3], Nikolaos Koutsouleris[3]
*[1]University of Cologne; [2]University Hospital of Cologne;*
*[3]Ludwig-Maximilian-University*

**Background:** Childhood trauma (CT) plays an important role in psychiatric disorders. It is associated with an increased risk for psychiatric disorders like major depression, anxiety disorders, dependency, post-traumatic stress disorders and even psychosis. There is a high incidence of CT in patients with psychosis, especially for physical and sexual abuse. Already in UHR-individuals increased CT could be observed. A study of Thompson and colleagues showed that 97% of their UHR sample reported a trauma in the past. 83% of the cases were physical abuse, 67% emotional abuse and 27% sexual abuse. Our aim was to investigate if there are neurobiological surrogate markers of trauma existing which can be detected by a multi pattern analysis.

**Methods:** PRONIA ('Personalized Prognostic Tools for Early Psychosis Management') is a prospective collaboration project funded by the European Union under the 7th Framework Programme (grant agreement n° 602152). Considering a broad set of variables (sMRI, rsMRI, DTI, psychopathological, life event related and sociobiographic data, neurocognition, genomics and other blood derived parameters) as well as advanced statistical methods, PRONIA aims at developing an innovative multivariate prognostic tool enabling an individualized prediction of illness trajectories and outcome. Seven clinical centers in five European countries and in Australia participate in the evaluation of three clinical groups (subjects clinically at high risk of developing a psychosis [CHR], patients with a recent onset psychosis [ROP] and patients with a recent onset depression [ROD]) as well as healthy controls; planned sample size is n=1680. CT was assessed by the Childhood Trauma Questionnaire (CTQ). To identify neuroanatomical and functional surrogate markers of CT, a multi pattern analysis via Neurominer (NM) was conducted. An additional VBM analysis was performed to evaluate the results of the NM analysis.

**Results:** We found that patients and HC could be separated very well by the CTQ pattern. Moreover, the classification among the patient groups yielded results that were not much better than a random classification. This finding underlines that CT is an overarching risk factor for mental diseases and not specific for single clinical entities. Furthermore, the decision scores that were conducted in the first NM analysis revealed highly significant negative correlations with grey matter changes in frontotemporal cortical areas, the anterior cingulate and the insular cortex. These areas are already discussed for CT in the literature.

**Discussion:** CT seems to be a global risk factor for psychiatric disorders. Moreover, we could re-examine the results of our multi variate analysis successfully in a VBM procedure.

### T5. LURASIDONE AND RISK FOR METABOLIC SYNDROME IN PATIENTS WITH SCHIZOPHRENIA: A COMPREHENSIVE DATABASE ANALYSIS

Michael Tocco*,[1], Andrei Pikalov[1],
Jay Hsu[1], Josephine Cucchiaro[1],
John W. Newcomer[2], Antony Loebel[1]
*[1]Sunovion Pharmaceuticals, Inc.; [2]Florida Atlantic University Charles E. Schmidt College of Medicine*

**Background:** Patients with schizophrenia are at increased risk for developing metabolic syndrome, with an estimated prevalence of approximately 35–50% (Correll et al. Psychiatr Serv 2010;61:892–98; Vancampfort et al. World Psychiatry 2015;14:339–47). Treatment with atypical antipsychotic medications have been shown to increase rates of metabolic syndrome, with differences observed among antipsychotic agents, most notably in propensity for weight gain: higher for olanzapine, clozapine, and iloperidone; intermediate for quetiapine, risperidone, and paliperidone; and lower for amisulpride, aripiprazole, asenapine, lurasidone, and ziprasidone (Leucht et al. Lancet 2013;382:951–62). Independent of weight gain, atypical antipsychotics also appear to have direct effects on lipid metabolism and glucose regulation. The aim of this safety analysis was to assess the effects of treatment with lurasidone on metabolic syndrome risk in patients with schizophrenia.

**Methods:** Changes in the rate of metabolic syndrome during treatment with lurasidone (40–160 mg/d) versus active comparators (olanzapine, quetiapine, risperidone) were analyzed using pooled short-term data from 3 randomized, double-blind, placebo-controlled studies; long-term data from 2 active-controlled studies; and switch data from 2 open-label extension studies. Metabolic syndrome was defined based on the National Cholesterol Education Program criteria (NCEP ATP III; 2005 revision).

**Results:** In short-term studies, risk of treatment-emergent metabolic syndrome was similar for patients in the lurasidone and placebo groups (odds ratio [OR]=0.97; week 6 LOCF-endpoint); and was significantly greater for patients in the olanzapine (OR=2.68; $P<0.001$) and quetiapine (OR=3.70; $P<0.001$) groups compared to placebo. In long-term studies, risk of treatment-emergent metabolic syndrome after 12 months was significantly lower for lurasidone compared with risperidone (OR=0.374; 95% CI, 0.180–0.774; $P<0.01$) and non-significantly lower for lurasidone compared with quetiapine XR (OR=0.267; 95% CI, 0.040–1.806; $P>0.05$). In open-label switch studies, the rate of metabolic syndrome decreased in patients switched to lurasidone after 6 weeks of treatment with olanzapine or 12 months of treatment with risperidone.

**Discussion:** In this comprehensive analysis of the lurasidone clinical trial data base, treatment with lurasidone (40–160 mg/d) was not associated with the development of metabolic syndrome in patients with schizophrenia. Rates of metabolic syndrome increased in patients treated with olanzapine, risperidone, and quetiapine XR.

### T6. SECOND GENERATION ANTIPSYCHOTIC DRUGS AND MORTALITY: A META-ANALYSIS OF PLACEBO-CONTROLLED RANDOMISED CONTROLLED TRIALS

Johannes Schneider-Thoma*[,1], Stefan Leucht[1]
[1]*Technical University of Munich*

**Background:** Despite intensive research it is unknown if treatment with antipsychotic drugs contributes to the reduced life-expectancy observed in patients with schizophrenia and other severe mental disorders. As randomized controlled trials (RCT) are considered the best evidence to examine causal relationship, we aimed to analyze mortality related to antipsychotics based on all placebo-controlled RCTs conducted so far.
**Methods:** We evaluated mortality in pair-wise meta-analysis of RCTs comparing second-generation-antipsychotics versus placebo across all diagnostic fields.
Information about deaths was extracted from summary data of clinical trials as identified by two searches (last 01/28/2017) in several electronic databases. Furthermore, manufacturing companies and regulatory authorities were contacted, and their websites were searched for trial reports and supplemental information about fatal events. We examined mortality due to any reason (primary outcome), due to natural causes, suicide, and other unnatural causes. We synthesized the results with odds ratios (OR) in a common-effect meta-analysis. We addressed the effects of age, diagnostic field, gender, study duration, antipsychotic drug used, drug dose and polypharmacy with subgroup- and meta-regression-analyses. We used the GRADE framework to evaluate the confidence in the evidence.
**Results:** We included 596 randomized trials that reported 207 deaths in 53804 patients on placebo (0.38%) and 99 deaths in 31184 patients on drug (0.32%). There was no evidence of significant difference between antipsychotics and placebo regarding mortality due to any reason (OR 1.19; 95% CI 0.93, 1.53), natural causes (OR 1.29; 95% CI 0.85, 1.94), suicide (OR 1.15; 95% CI 0.47, 2.81) and other unnatural causes (OR 1.55; 95% CI 0.66, 3.63). The number-needed-to-harm for all-cause mortality was 6 more deaths in 10 000 drug-treated patients compared to placebo, with a 95% CI ranging from 2 helped to 14 harmed. Most deaths occurred in patients with dementia and these patients also had the highest drug-related mortality in subgroup analysis (OR 1.56; 95% CI 1 .10, 2.21). The OR for patients with schizophrenia (based on 32807 patients) was 0.69 (95 CI 0.35, 1,35).
**Discussion:** We found no evidence of increased mortality related to treatment with second generation antipsychotic drugs, neither in the overall sample nor in the subgroup of patients with schizophrenia. Results of subgroup analyses indicated however that mortality of patients with dementia might be increased when they are exposed to antipsychotics. Strength of the analysis are the use of data from RCTs and the comprehensive search, which aimed to identify all placebo-controlled RCTs of second generation antipsychotics conducted so far. Limitations of our analysis are the remaining imprecision of the results with a 95% confidence interval including the risk of more than 1 patient harmed in 1000 patients treated with antipsychotics. Moreover, we could only address short-term effects leading to death, but not long-term effects - such as induction of metabolic syndrome – which also contribute to the increased mortality over the whole life-span.

### T7. PHARMACOGENETIC OF TARDIVE DYSKINESIA -- A FOLLOW-UP ON THE VALBENAZINE TARGET VMAT2/SLC18A2

Clement Zai*[,1], Arun Tiwari[1], Daniel Mueller[1], Aristotle Voineskos[1], Steven G. Potkin[2], Jeffrey Lieberman[3], Herbert Meltzer[4], Gary Remington[1], James Kennedy[1]
[1]*Centre for Addiction and Mental Health;* [2]*University of California, Irvine;* [3]*Columbia University;* [4]*Northwestern University Feinberg School of Medicine*

**Background:** Tardive dyskinesia (TD) is a motor side effect that may arise after long-term treatment of antipsychotic drugs. Its etiology is not well understood, but a number of risk factors have been associated with TD. TD occurrence appears to be familial, thus suggesting a genetic component. We previously reported on an association between the SLC18A2 gene that codes for the vesicular monoamine transporter 2 (VMAT2) that packages monoamines including dopamine from the cytoplasm into synaptic vesicles (Zai et al, 2013). In the present study, we examined the dopamine transporter gene SLC6A3 by itself and in conjunction with SLC18A2 for possible association with TD.
**Methods:** We genotyped and analyzed the variable-number tandem repeat (VNTR) polymorphism in the 3' untranslated region of the SLC6A3 gene in our European sample of 187 schizophrenia/schizoaffective disorder patients assessed for TD occurrence based on the Abnormal Involuntary Movement Scale (AIMS). We also explored the interaction between the VNTR and the TD-associated SLC18A2 marker rs363224.
**Results:** Our preliminary analysis did not show the SLC6A3 VNTR to be associated with TD occurrence or severity. There also appeared to be no significant interaction between SLC6A3 VNTR and SLC18A2 rs363224 in TD occurrence or severity ($p>0.05$).
**Discussion:** Our findings did not support a major role of the dopamine transporter gene in TD risk or severity, but we will examine additional putative functional markers in this gene.

### T8. NEUROLOGICAL SOFT SIGNS (NSS) IN SCHIZOPHRENIA: AN UPDATE ON THE STATE- VERSUS TRAIT-PERSPECTIVE

Silke Bachmann*[,1], Johannes Schröder[2]
[1]*Clienia Littenheid;* [2]*University of Heidelberg*

**Background:** Neurological soft signs (NSS) represent minor neurological signs which indicate non-specific cerebral dysfunction. Numerous studies have confirmed their presence in schizophrenia across all stages of the disease. NSS have been looked at as an endophenotype or a trait phenomenon for many decades. However, during the past years studies increasingly reported on fluctuations of the NSS scores. To shade further light on the question whether NSS represent a state or a trait component or both, a review of longitudinal studies on schizophrenia patients was performed, because only measurements at two or more points in time can answer the question at hand.
**Methods:** A search of studies which had assessed NSS in adult schizophrenia patients and included at least one follow-up examination was undertaken. Studies which had been published between January 1966 and June 2017 and listed in relevant databases were included. Due to the fact, that ongoing brain maturation lasts until adulthood and is paralleled by a loss of those NSS which are present in childhood, studies on teenagers were excluded.
**Results:** Twenty-nine follow-up studies were identified which overall used well-known instruments for the investigation of NSS. Patients were at different disease stages. All expressed abnormally increased NSS, however to different extents. An NSS reduction during the course was detected in most first episode patients and those with a remitting course whereas chronically ill patients exhibited stable or increasing NSS scores. The change over time could for the most part be attributed to changes in the motor system subscales and to a lesser amount to sensory integration scales. As opposed to the earlier notion that medication evokes or worsens NSS, studies largely agreed on a positive interrelation between medication response and improvement of NSS. The type of antipsychotic was of small importance and when side-effects were commented on there was a weak relationship with NSS. On the other hand, studies gave some hints at relationships between NSS and symptoms, i.e. negative and cognitive symptoms.
**Discussion:** The reviewed studies confirmed the presence of abnormal, i.e. elevated NSS in patients diagnosed with schizophrenia. Studies disagreed on the amount of abnormalities in NSS. However, subgroups emerged with either stable or fluctuating properties of NSS. The latter speaks very much in favor of a state-trait dichotomy being present in NSS and thus challenges the view that NSS depict an endophenotype.

### T9. CROSS-SECTIONAL ASSOCIATION OF MEMBRANE FATTY ACID COMPOSITION AND PSYCHOPATHOLOGY IN THE NEURAPRO-E STUDY

Maximus Berger[1], Patrick D. McGorry[2], Barnaby Nelson[2], Connie Markulev[2], Hok Pan Yuen[3], Miriam Schaefer[1], Nilufar Mossaheb[4], Monika Schlogelhofer[4], Stefan Smesny[5], Ian Hickie[6], Gregor Berger[7], Eric Chen[8], Lieuwe De Haan[9], Dorien Nieman[10], Merete Nordentoft[11], Anita Riecher-Rössler[12], Swapna Verma[13], Andrew Thompson[14], Alison Yung[15], G. Paul Amminger*[,2]
*[1]James Cook University; [2]Orygen, The National Centre of Excellence in Youth Mental Health; [3]Orygen, The National Centre of Excellence in Youth Mental Health, The University of Melbourne; [4]Medical University of Vienna; [5]University Hospital Jena; [6]University of Sydney; [7]Child and Adolescent Psychiatric Service of the Canton of Zurich; [8]University of Hong Kong; [9]AMC-Academisch Psychiatrisch Centrum; [10]Academic Medical Center; [11]Mental Health Centre Copenhagen; [12]University of Basel Psychiatric Clinics; [13]Institute of Mental Health, Singapore; [14]University of Warwick; [15]University of Manchester*

**Background:** Converging evidence suggests that people at ultra-high risk (UHR) for psychosis have depleted levels of several fatty acids (FAs), and that changes in omega-3 (n-3) FA levels may indicate a higher risk for transition to psychosis. However, limited information is available on how FA deficiencies relate to psychopathology in individuals with UHR phenotypes. Here, we report the relationship between membrane FA levels and symptom severity in a study of individuals at UHR for psychosis.
**Methods:** Data from 280 of 304 (92%) of participants of the NEURAPRO study, a multi-centre randomized-controlled trial of omega-3 fatty acids versus placebo, were used for the present analysis. All participants were aged between 13 and 40 years and met criteria for UHR for psychosis. Blood samples were collected at study baseline and month 6 (end-of-intervention). Membrane fatty acids were analysed using mass spectrometry as percentage of total fatty acids in erythrocytes. Pearson correlation coefficients were calculated between baseline erythrocyte fatty acid levels and scores on the Scale for the Assessment of Negative Symptoms (SANS) and Brief Psychiatric Rating Scale (BPRS).
**Results:** Negative symptoms were positively correlated with one saturated FA (Tetracosanoic acid [24:0], R=0.272, p<0.0001), one n-3 FA (Eicosapentaenoic acid [20:5], R=0.142, p=0.017) and one n-9 FA (Nervonic acid [24:1], R=0.274, p<0.0001), and negatively correlated with one saturated FA (Palmitic acid [16:0], R=-0.224, p<0.0001), two n-6 FAs (Dihomo-y-linolenc acid [20:3], R=-0.201, p<0.001 and Linolelaidic acid [18:2], R=-0.333, p<0.0001), and one n-7 FA (Vaccenic acid [18:1], R=-0.172, p=0.004). BPRS scores were positively correlated with one saturated FA (Tetracosanoic acid [24:0], R=0.363, p<0.0001) and one n-9 fatty acid (Nervonic acid [24:1], R=-0.346, p<0.0001), and negatively correlated with two n-3 FAs (Dihomo-y-linolenc acid [20:3], R=-0.153, p=0.010 and Docosahexaenoic acid [22:6], R=-0.193, p<0.001), and two n-6 FAs (Arachidonic acid [20:4], R=-0.125, p=0.037 and Linoleic acid [18:2], R=-0.340, p<0.0001).
**Discussion:** Consistent with a previous study, negative symptoms and general psychopathology were associated with levels of several classes of FAs in the present study. These findings support the relevance of membrane fatty acids for the onset of psychotic symptoms and indicate that FAs should be further evaluated as biomarkers in people at UHR for psychosis.

### T10. HERITABILITY OF AMYGDALA ACTIVITY AND ITS GENOME WIDE ASSOCIATION WITH THE SCHIZOPHRENIA RISK LOCUS OF MIR137

Tiziana Quarto*[,1], Giulio Pergola[1], Pasquale Di Carlo[1], Vittoria Paladini[1], Marco Papalino[1], Raffaella Romano[1], Antonio Rampino[1], Daniela Marvulli[1], Alessandro Bertolino[1], Giuseppe Blasi[1]
*[1]University of Bari*

**Background:** It is well known that heritability plays a prominent role in risk for schizophrenia, and that this brain disorder is crucially characterized by emotional symptoms. Less known is how heritability shapes brain activity during emotion processing and whether this brain phenotype is also associated with genetic variation increasing risk for schizophrenia. Here, we implemented a multi-step, data-driven approach in order to assess the relevance of the link between heritability, genetic variation, and schizophrenia for brain activity during emotion processing.
**Methods:** We investigated three samples of healthy individuals and one sample of schizophrenia (SCZ) patients: i) 28 healthy twin pairs (16 monozygotic and 12 dizygotic twin pairs); ii) 289 unrelated healthy participants (genome-wide association study - GWAS -discovery sample); iii) 90 unrelated healthy participants (replication sample); iv) 40 SCZ patients. During fMRI, participants approached or avoided threatening angry faces (explicit emotion processing). Intra-class correlations (ICC) between twin pairs and ACE models (A: additive genetics; C: common environment; E: unique environment) were used to identify regions of interest (ROIs) with heritable functional activity. Then, we extracted BOLD signal from these ROIs and conducted a GWAS on 565,137 single nucleotide polymorphisms (SNPs) (selected with the following criteria: minor allele frequency>0.15, Hardy–Weinberg equilibrium<0.001, linkage disequilibrium pruning $r^2$>0.9) using robust linear models of allelic dosage corrected for multiple comparisons (Gao et al. 2008 Genetic Epidemiology). Finally, we assessed the effect of surviving SNPs in the replication sample of healthy individuals as well as in the sample of SCZ patients.
**Results:** In healthy twins, we identified bilateral amygdala as the brain region with the highest heritability during explicit emotion processing as evaluated with our task (ICC=.79; h2=0.54; p<.001). The subsequent GWAS in healthy non-twins indicated that bilateral amygdala activity during the task was associated with a polymorphism close to miR-137 (rs1198575) (p=1.5 × 10–7), with the C allele corresponding to lower activity than the t allele. A similar effect was found in the replication sample (p=.01) and in patients with SCZ (p=.03).
**Discussion:** Our data-driven approach revealed that amygdala activity as evaluated with our task is heritable. Furthermore, our results indicate that a polymorphism in miR-137 has genome wide association with amygdala response during emotion processing which is also replicated in two independent samples of healthy subjects and of patients with schizophrenia. Previous findings indicated that this polymorphism has genome-wide association with schizophrenia (Ripke et al. 2014). Other results reveal that miR-137 is a key regulatory neuronal factor linked to SCZ and involved in emotion processing (Cosgrove et al., 2017). Our findings are consistent with these previous findings and further highlight a crucial role for miR-137 in emotion processing and SCZ (Anticevic et al., 2012 Schizophr Bull).

### T11. CEREBROSPINAL FLUID (CSF) MARKERS OF INFLAMMATION AND INFECTIONS IN SCHIZOPHRENIA AND AFFECTIVE DISORDERS: A SYSTEMATIC REVIEW AND META-ANALYSIS

Sonja Orlovska[1], Ole Köhler-Forsberg[1], Sophie Brix[1], Merete Nordentoft[1], Daniel Kondziella[2], Jesper Krogh[1], Michael Benros*[,3]

[1]Mental Health Centre Copenhagen, Copenhagen University Hospital; [2]Copenhagen University Hospital; [3]Mental Health Centre Copenhagen

**Background:** Infections and inflammatory processes have been associated with the development of schizophrenia and affective disorders; however, no study has yet synthesized all the available data on cerebrospinal fluid (CSF) immune-alterations. We conducted the first systematic review of the immunological findings from CSF studies among patients with schizophrenia or affective disorders.
**Methods:** All studies investigating CSF inflammatory markers in persons with schizophrenia or affective disorders published prior to March 23, 2017 were identified searching PubMed, CENTRAL, EMBASE, Psychinfo, and LILACS. The literature search, data extraction and assessment of risk of bias were performed by two independent reviewers. Meta-analyses with standardized mean difference (SMD) including 95% confidence intervals (CI) were performed on studies including healthy controls.
**Results:** We identified 112 CSF studies published between 1942–2016, of which 32 were included in meta-analyses; however, only few studies investigated identical biomarkers. The CSF/serum albumin ratio was increased in both schizophrenia (54 patients; SMD=0.62; 95%CI=0.24–1.00) and affective disorders (302 patients; SMD=0.43; 95%CI=0.25–0.61, I2=0%), compared to healthy controls. Total CSF protein was elevated in both schizophrenia (97 patients; SMD=0.38; 95%CI=0.12–0.65, I2=0%) and affective disorders (53 patients; SMD=0.77; 95%CI=0.36–1.18, I2=0%). The IgG ratio was increased in schizophrenia (54 patients; SMD=0.60; 95%CI=0.23–0.98), whereas the IgG Albumin ratio was decreased (32 patients; SMD= -0.62; 95%CI= -1.13 to -0.12). Interleukin-8 (IL-8) (95 patients; SMD=0.46; 95%CI=0.17–0.75, I2=0%) and IL-6 levels (230 patients; SMD=0.38; 95%CI=0.02–0.74; I2=64%) were increased among individuals with schizophrenia but not significantly increased in affective disorders. None of the remaining inflammatory markers were significantly different compared to healthy controls in the meta-analyses. However, in the studies which did not include healthy controls, CSF abnormalities were more common, and CSF dependent re-diagnosis occurred in 3.2–6% in the two studies investigating this.
**Discussion:** Our findings suggests that schizophrenia spectrum and affective disorders are associated with CSF abnormalities including signs of blood-brain barrier impairment and inflammation, supporting a role of the immune system in mental disorders. However, only few studies investigated the same parameters with healthy controls and high quality longitudinal CSF studies are lacking, including impact of psychotropic medications and potential benefits of anti-inflammatory treatment in subgroups with abnormal CSF inflammatory markers

### T12. VITAMIN D STATUS AND PSYCHOTIC DISORDER: ASSOCIATIONS WITH CLINICAL VARIABLES AND RISK FACTORS

Christine van der Leeuw*[,1], Lot de Witte[2], Claude van der Ley[3], Richard Bruggeman[3], Jim van Os[4], Machteld Marcelis[5]
*[1]Maastricht University Medical Center; [2]Icahn School of Medicine, University Medical Center Utrecht, Utrecht University; [3]University of Groningen, University Medical Center Groningen; [4]University Medical Center Utrecht, Utrecht University, Maastricht University Medical Center, Institute of Psychiatry, King's College London; [5]Maastricht University Medical Center, Institute for Mental Health Care Eindhoven (GGzE)*

**Background:** The association between schizophrenia and decreased vitamin D levels is well documented. Low maternal and postnatal vitamin D levels suggest a possible etiological mechanism. Vitamin D deficiency in patients diagnosed with schizophrenia is presumably (also) the result of disease-related factors. Furthermore, certain demographic risk factors such as urbanicity may be associated with vitamin D.
**Methods:** In a large study population of 347 patients with psychotic disorder and 282 controls, associations between vitamin D levels in blood and clinical variables and risk factors were investigated.
Regression analyses were conducted correcting for gender, age, ethnicity, body mass index (BMI), smoking and sampling season. Group × symptomatology and group × urbanicity interactions were investigated. Both current urbanicity and urbanicity at birth were assessed.
**Results:** Vitamin D concentrations were significantly lower in patients (B= -8.05; 95% confidence interval (CI) -13.68 to -2.42; p=0.005). There were (trend) significant interactions between group and vitamin D for symptomatology (positive symptoms: $\chi 2$=2.81 and p=0.094; negative symptoms: $\chi 2$=5.63 and p=0.018). A small but significant effect was detected: higher vitamin D concentration was associated with lower positive (B= -0.02; 95% CI -0.04 to 0.00; p=0.049) and negative symptom levels (B= -0.03; 95% CI -0.05 to -0.01; p=0.008) in patients. The group × current urbanicity interaction was not significant. However, the group × urbanicity at birth was significant when corrected for current urbanicity ($\chi 2$=11.26 and p=0.001).
**Discussion:** Vitamin D levels in patients with psychotic disorder were lower than in controls, with an interaction between group and urbanicity at birth. In the patient group, symptom levels were lower when vitamin D concentration was higher.

### T13. PROGRESSIVE SPONTANEOUS AND SYNCHRONY GAMMA-BAND OSCILLATION DEFICITS IN FIRST EPISODE SCHIZOPHRENIA

Yoji Hirano*[,1], Naoya Oribe[1], Toshiaki Onitsuka[1], Shigenobu Kanba[1], Martha Shenton[2], Margaret A. Niznikiewicz[3], Larry J. Seidman[4], Robert W. McCarley[3], Kevin M. Spencer[3]
*[1]Kyushu University; [2]Brigham & Women's Hospital, Harvard Medical School; [3]Harvard Medical School, Veterans Affairs Boston Healthcare System; [4]Harvard Medical School, Beth Israel Deaconess Medical Center*

**Background:** Deficits in the gamma-band (30–100 Hz) auditory steady-state response (ASSR) and progressive volumetric decreases in the primary auditory cortex have been detected shortly after the onset of schizophrenia (SZ), and may be associated with symptoms such as auditory hallucinations. Disruption of gamma-band oscillation has received considerable interest, as the basic mechanisms underlying these oscillations are understood and are conserved across species. Despite the importance of abnormal gamma-band oscillations in SZ, it remains unclear whether the gamma-band ASSR deficit shows progressive change over time during the early stages of the disease. Moreover, animal models based on NMDA receptor hypofunction demonstrate an increase in spontaneous gamma power, which has been reported in chronic SZ (Hirano et al., JAMA Psychiatry 2015), yet it still remains unclear in first-episode schizophrenia (FESZ). Hence, a longitudinal electroencephalogram study of the spontaneous and synchrony gamma-band oscillation in FESZ is important to better understand the pathophysiology and trajectory of early-stage schizophrenia.
**Methods:** Subjects were 23 FESZ (14 treated and 9 untreated with antipsychotics), and 39 matched healthy controls (HC). Dipole source localization of dense electrode EEG data was used to examine oscillatory activities in auditory cortices during auditory steady-state stimulation (20/30/40-Hz rates). ICA was used to remove artifacts. Phase locking factor (PLF) and induced power (not phase-locked) were calculated from artifact-free single trial source estimates. Clinical symptoms were assessed by SAPS and SANS. Subjects were recruited as part of the Boston CIDAR Center (www.bostoncidar.org). Test sessions (Time-1/Time-2) were 11.9 months apart.
**Results:** Compared to HC, FESZ showed reduced 40-Hz ASSR PLF (synchrony gamma) and increased induced gamma power (spontaneous gamma) during continuous auditory stimuli at time-1. Longitudinally,

FESZ showed overall progressive reductions in 40-Hz ASSR PLF and progressive increases in induced gamma power, especially within the left auditory cortex. These progressive deficits were not related to antipsychotic medication. Progressive increase of induced gamma power was correlated with increased positive symptoms.
**Discussion:** We found coincide disruptions of auditory gamma-band oscillation, which showed progressive increase in spontaneous gamma (cortical excitability) and progressive decrease in synchrony gamma (cortical synchrony failure) during continuous auditory stimuli in FESZ. These two apparently distinctive circuit progressive abnormalities already occurred in the very early stage of the disease. We propose that assessing ASSR-PLF and spontaneous gamma in FESZ may provide a sensitive translatable biomarker for the integrity of neural networks that are fundamentally altered in the very early stage of SZ.

### T14. ASSESSING DIFFERENCES IN INFLAMMATORY MARKERS BETWEEN FIRST EPISODE PSYCHOSIS PATIENTS AND HEALTHY CONTROLS: THE IMPORTANCE OF CONTROLLING FOR CONFOUNDING FACTORS

Annalisa Giordano*[,1], Valeria Mondelli[1], Mitul A. Mehta[1], Steve Williams[1], Carmine M. Pariante[1], Paola Dazzan[1]
*[1]Institute of Psychiatry, Psychology & Neuroscience, King's College London*

**Background:** Although there is a cumulative evidence for increased level of markers of inflammation in psychosis, it has not been conclusively proven yet whether this is due to the disorder in itself or to other factors. One reason for this is the lack of studies that have controlled for major confounding factors such as obesity, smoking, antipsychotic use and stress. The BMI, as an indirect measure of body fat level, and tobacco smoking are known to play a role in modulating the immune system, but little research has been done in this area in patients with psychosis. The aim of this study was to investigate the differences in markers of inflammation and neuroplasticity between FEP patients and HC while controlling for a priori confounding factors, such as BMI and tobacco smoking.
**Methods:** Nineteen First Episode Psychosis (FEP) patients and 21 healthy controls (HC) matched for age, gender, ethnicity and marital status were recruited in South London (UK). Blood samples were collected to measure High Sensitivity C-Reactive Protein (hs-CRP), Interleukin (IL)-6, IL-8 and Brain-Derived Neurotrophic Factor (BDNF). Body Mass Index (BMI) was also assessed. Moreover, patients were asked whether they were tobacco smokers. Differences in continuous variables were analysed using independent samples t-tests. Categorical variables were assessed using the chi-square ($\chi 2$) test. One-way ANCOVAs were conducted to assess difference between FEP patients and HC in markers of inflammation and BDNF while controlling for the effect of BMI and tobacco smoking. All analyses were conducted using IBM SPSS statistical software version 23. The significance value for all tests was set at $\alpha = 0.05$.
**Results:** FEP patients had higher serum level of hs-CRP compared to controls (N = 19, M = 2.29 mg/L, SD = 2.76; N = 21, M = 0.56 mg/L, SD = 0.41; respectively, t(27.79)=-2.41, p=0.02), suggestive of a hyperactivation of the immune system. There was no significant difference in IL-6, IL-8 and BDNF serum levels between the two groups. BMI was significantly higher in patients than controls (N = 19, M = 27.67, SD = 4.91; N = 21, M = 23.55, SD = 3.31; respectively, t(38)=-3.14, p=0.003) and there was a significantly higher number of smokers in the FEP patient group than in the HC group (smokers= 57.9% of FEP patients; smokers=14.3% of HC; $\chi 2(1)=8.34$, p=0.004). The one-way ANCOVAs showed that there was not significant effect of group on hs-CRP, IL-6, IL-8 and BDNF values when controlling for BMI and tobacco smoking. In fact, BMI was significantly related to the hs-CRP level, F(1,36) = 7.20, p = 0.01.
**Discussion:** We found that BMI and tobacco smoking may be important confounding factors when investigating disease-related alterations in the levels of hs-CRP, IL-6, IL-8 and BDNF. More studies are needed to assess the role of potential confounding factors on markers of inflammation in psychosis and to understand the role of immune activation in the pathophysiology of these disorders.

### T15. LONGITUDINAL ASSOCIATIONS BETWEEN CHILDHOOD SALIVARY CORTISOL LEVELS AND PRODROMAL SYMPTOMS IN LATE ADOLESCENCE: FINDINGS FROM A HIGH-RISK COHORT

Alexis Cullen*[,1], Elizabeth Fraser[1], Helen Fisher[1], Ruth Roberts[1], Uzma Zahid[1], Carmine Pariante[1], Patricia Zunszain[1], Philip McGuire[1], Robin Murray[1], Valeria Mondelli[1], Kristin R Laurens[2]
*[1]Institute of Psychiatry, Psychology & Neuroscience, King's College London; [2]Australian Catholic University*

**Background:** Individuals with established psychosis are characterised by a distinct pattern of hypothalamic-pituitary-adrenal (HPA) axis dysfunctions which include both elevated daytime cortisol levels and a blunted cortisol awakening response (CAR). Whilst these patterns of dysfunction have also been observed among those at elevated risk for the disorder, longitudinal studies are scarce. As such, the relevance of these HPA axis abnormalities for the progression of psychopathology in high-risk populations is unknown. Utilising data from a well-characterised, longitudinal cohort of youth at elevated risk for schizophrenia and their typically-developing peers (The Child Health and Development Study), we aimed to investigate the extent to which HPA axis function determined in childhood is a significant predictor of putative prodromal status and psychopathology in late adolescence/early adulthood.
**Methods:** The sample comprised high-risk individuals who presented either multiple antecedents of schizophrenia (developmental delays, psychopathology, and psychotic-like experiences: ASz=21) or a family history of illness (FHx=13), and typically-developing youth with neither antecedents nor a family history (TD=36). Participants were recruited at age 9–12 years using a community screening method and assessed biennially throughout adolescence. At the age 11–14 years assessment phase, participants collected salivary cortisol samples in their home environment which were used to determine diurnal cortisol secretion and the CAR. At the age 17–21 years assessment phase, participants completed measures of prodromal symptoms (Prodromal Questionnaire: PQ), depression (Quick Inventory of Depressive Symptomatology questionnaire: QIDS), and anxiety (Social Interaction Anxiety Scale: SIAS). Established PQ thresholds were used to identify participants who met probable prodromal status. Logistic and linear regression analyses were used to examine the extent to which salivary cortisol measures at age 11–14 years predicted probable prodromal status and continuous psychopathology measures at 17–21 years, respectively.
**Results:** Relative to the TD group, ASz youth were characterised by higher depression (B=0.24, p=0.05) and disorganised symptoms (B=0.36, p=0.007) at 17–21 years whilst FHx youth obtained higher scores on the PQ general symptoms scale (B=0.24, p=0.048). Analyses performed in the total sample indicated that the CAR was negatively associated with depression symptoms (B=-0.28, p=0.006) and PQ negative symptoms (B=-0.30, p=0.004) at age 17–21 years. Positive associations were observed between diurnal cortisol and positive (B=0.41, p=0.02), disorganised (B=0.30, p=0.04), and general (B=0.29, p=0.03) PQ symptoms. Diurnal cortisol levels were also significantly associated with probable prodromal status at follow-up (OR=1.04, p=0.04). No significant interactions were observed between group status and salivary cortisol levels in any model. After adjustment for potential confounders (age, follow-up time, sex, BMI, and pubertal status), the CAR continued to show significant associations with both depression (p=0.006) and PQ negative symptoms (p=0.007) whilst only a statistical trend was observed for the relationship between diurnal cortisol levels and positive symptoms (p=0.055).

**Discussion:** The current study is the first to examine the extent to which HPA axis function can predict development of prodromal symptoms in a high-risk cohort. Our finding that more abnormal HPA axis function (i.e., a decreased CAR and higher diurnal cortisol) at age 11–14 years is associated with both prodromal and depression symptoms at age 17–21 has important implications for aetiological theories and for clinical practice.

### T16. GLUTAMATERGIC CHANGES IN UHR

Christina Wenneberg*[,1], Brian Broberg[2], Egill Rostrup[3], Louise Birkedal Glenthøj[4], Birte Glenthoj[5], Merete Nordentoft[4], Tina Dam Kristensen[4]

*[1]Psychiatric Centre Copenhagen; [2]Center for Neuropsychiatric Schizophrenia Research; [3]Center for Neuropsychiatric Schizophrenia Research, Center for Clinical Intervention and Neuropsychiatric Schizophrenia Research, Psychiatric Center Glostrup, University of Copenhagen, Glostrup University Hospital; [4]Mental Health Centre Copenhagen; [5]Center for Neuropsychiatric Schizophrenia Research, Center for Clinical Intervention and Neuropsychiatric Schizophrenia Research, University of Copenhagen*

**Background:** The search for biomarkers may prove significant for short-term identification of UHR individuals (remission/non-remission). On a long-term basis, biomarkers might give the opportunity to delay or prevent psychotic episodes. Disturbances of the neurotransmitters glutamate and GABA have long been suspected to be involved in the pathophysiology of psychosis. These disorders have also found been found in people at UHR, making it a promising area for early detection.

Cognitive deficits in schizophrenia are present prior to the onset of psychosis, and may be linked to perturbed glutamate and GABA function. Data suggest that this link is already present in UHR states.

**Methods:** Participants: UHR individuals who meet the CAARMS criteria recruited from Mental Health Services in the Capital Region of Denmark and matching healthy controls.

**Examinations:** 1H-MRS of the ACC and thalamus. Diagnostic and psychopathological tests: CAARMS, SCID, SOFAS, PSP, Cornblatt, SANS, BPRS, MADRS, YMRS, CGI, PAS, SPI-A, AQoL Cognitive tests as part of collaborative studies.

**Results:** So far 116 UHR individuals and 42 healthy controls have been scanned (December 2017) Very early preliminary analysis of the baseline data finds no significant difference in glutamate levels (in ACC and thalamus) in UHR patients compared to matched healthy controls. Baseline data remains to be analysed in relation to relevant subgroups of patients e.g. based on clinical outcome. GABA analysis and analysis of follow-up data are also yet to be performed. Data will be ready for the meeting, and will be presented.

**Discussion:** More studies are needed in this field, since results so far have been diverging.

Baseline data remains to be analysed in relation to relevant subgroups of patients e.g. based on clinical outcome. GABA analysis and analysis of follow-up data are also yet to be performed. Glutamate data will be presented at the meeting.

### T17. OXIDATIVE STRESS BIOMARKERS AND NEGATIVE DIMENSION IN THE FIRST TEN YEARS OF SCHIZOPHRENIA: A 1-YEAR FOLLOW-UP STUDY

Leticia González-Blanco*[,1], M Paz Garcia-Portilla[1], Leticia Garcia-Alvarez[1], Lorena De La Fuente-Tomas[1], Pilar Saiz-Martinez[1], Celso Iglesias[1], Ana Coto[1], Julio Bobes[1]

*[1]University of Oviedo*

**Background:** Several studies have documented changes in oxidative parameters and antioxidant enzymes in patients with schizophrenia (1, 2). However, their relation to negative symptoms and the longitudinal clinical course is still unclear.

The objectives of the present study are to: 1) analyze the association between oxidative stress biomarkers and negative dimension; 2) identify if these biomarkers could predict clinical outcomes in stable patients with schizophrenia at 1-year follow-up.

**Methods:** A 1-year follow-up study of 57 stable outpatients with schizophrenia (≤10 years of illness) (mean age=31.5 ± 6.5; 63.2% males).

**Assessment:** PANSS, Clinical Assessment Interview of Negative Symptoms (CAINS) -Motivation/Pleasure (MAP) & Expression (EXP) domains-, Brief Negative Symptom Scale (BNSS). Oxidative stress biomarkers: homocysteine, hemolysis test (% hemolysis), lipid peroxidation subproducts (LPO), catalase activity in erythrocytes (CAT).

Pearson correlations were performed to determine associations between biomarkers and clinical scores at baseline, and they were included in stepwise multiple linear regression analyses, considering potential confounding factors.

The clinical course for each psychopathological domain was determined using the formula: [follow-up-baseline scores]. Positive values were interpreted as worsening, while negative improvement. Pearson correlation and multiple linear regression analyses were performed to determine if baseline levels of oxidative stress parameters were predictors of clinical changes at follow-up.

**Results:** 1) Baseline associations: Final regression models identified that LPO level was a significant predictor of lower scores in PANSS-N, BNSS total, Avolition and Blunted Affect subscale of BNSS and CAINS-EXP ($\beta$= -0.408; -0.290, -0.254, -0.296, -0.247, respectively).

2) Longitudinal course: At 1-year follow-up, patients only improved significantly ($p<0.05$) in PANSS-Total [59.4 ± 16.4 - 54.5 ± 16.0 (t=3.362)], PANSS-General [29.7 ± 8.9 - 26.9 ± 7.9 (t=3.362)], Blunted Affect subscale [6.9 ± 5.0 - 5.9 ± 4.7 (t=2.489)], and almost significant ($p<0.069$) in CAINS-EXP and BNSS total score. No significant changes in BMI, waist circumference, smoking or antipsychotic equivalent doses were detected, but they were also considered in regression analyses. A higher percentage of hemolysis at baseline, with a decrease in equivalent doses of antipsychotics, both significantly predict an improvement in scores of PANSS-N (R2=0.140, F=7.166), BNSS (R2=0.246, F=6.193) and CAINS-EXP (R2=0.186, F=5.259).

**Discussion:** Lower concentrations of LPO were related to greater severity of negative symptoms as avolition and blunted affect (inner world). Longitudinal analyses showed that higher % of hemolysis at baseline predict an improvement of negative dimension at 1-year follow-up. From our results, we hypothesize that there is an inverse relationship between oxidative stress and negative dimension in stable patients with schizophrenia during the first ten years of illness.

### T18. MULTIVARIATE BRAIN ANATOMICAL DIFFERENCES IN POSITIVE AND NEGATIVE SCHIZOTYPY: PRELIMINARY RESULTS FROM THE TYPIA STUDY

Maria F. Urquijo*[,1], Eliana Faiola[2], Anne Ruef[3], Anna Kasparbauer[2], Inga Meyhöfer[2], Maria Steffens[2], Ulrich Ettinger[2], Nikolaos Koutsouleris[1]

*[1]Ludwig Maximilian University; [2]University of Bonn; [3]Ludwig Maximilians University, University Psychiatric Hospital*

**Background:** In schizotypy, a factor structure similar to the one observed in schizophrenia has been unraveled, being the positive and negative the most consistently replicated dimensions. Despite this fact, most of the studies on brain volume patterns in schizotypy consider it as an unitary rather than a multidimensional construct. Hence, based on previous results showing that schizophrenia and schizotypal personality traits share common neurodevelopmental patterns, it is hypothesized that brain volumetric patterns in individuals with high positive schizotypy are intrinsically different to those observed in persons reporting high negative schizotypy and to individuals with overall low schizotypal traits. The present study aims to evaluate this hypothesis using novel machine learning techniques to address the multivariate nature of psychotic diseases and the brain itself.

**Methods:** Data from the TYPIA Study, an ongoing project conducted at the Ludwig-Maximilian University of Munich and the University of Bonn in Germany, was used to investigate whether brain volumetric patterns are distinct in healthy individuals with high positive (HPS) and high negative schizotypy (HNS) when compared to one another (HPS vs HNS) and to individuals with self-reported low schizotypy (LS vs HNS and LS vs HPS). A preliminary analysis on grey matter volumetric patterns from 29 LS (19 f., mean age: 24.6 y.), 28 HNS (20 f., mean age: 26.8 y.) and 23 HPS (17 f., mean age: 26.4 years) individuals from the general population without any current psychiatric diagnosis was performed. Group divisions were based on the introvertive anhedonia and unusual experiences subscales from the Oxford-Liverpool Inventory of Feelings and Experiences (O-LIFE). Structural images were preprocessed with a standard voxel based morphometry pipeline using the SPM-based CAT12 toolbox in Matlab. After age, sex and grey matter intracranial volume and center corrections, a linear support vector classification (SVC) algorithm was used to assess separability between the groups.

**Results:** Our preliminary cross-validated results showed that LS and HNS can be separated with 56.0 % balanced accuracy (BAC), whereas LS vs HPS and HNS vs HPS allowed for only 42.87% and 48.8% BAC respectively. Interestingly, a post-hoc analysis comparing LS vs both high schizotypy groups merged together showed the highest BAC (59.2%). As expected, the brain differences between groups are rather small, since the sample consists fully of healthy controls. However, these results indicate that personality traits related to HNS are linked to more pronounced changes in the brain as compared to HPS. Nevertheless, schizotypy as a combination of the positive and negative dimensions allowed for a higher classification accuracy when compared to LS, supporting the notion of schizotypy as a unitary construct as observed from the post-hoc analysis. Furthermore, HNS and HPS were not separable by the algorithm, most likely due to the intrinsic heterogeneity of the construct.

**Discussion:** Our results align with previous studies claiming that negative symptoms are associated with structural changes in the CNS whereas positive symptoms relate to changes in functioning and activation of the brain. A larger sample as well as using other data modalities will confirm the stability of our findings. Research on volumetric patterns of the brain areas related to negative symptoms in non-clinical samples might lead to a better understanding of the underlying causes of schizophrenia. Above all, our results show that investigating non-clinical expression of psychosis-like symptoms is a promising strategy to understand the prodromal stadium of schizophrenia.

### T19. MULTIMODAL IMAGING IN FIRST EPISODE PSYCHOSIS: MAGNETOENCEPHALOGRAPHY, 7T FMRI STROOP, AND 7T MRS SPECTROSCOPY

Timothy Gawne*[,1], Greg Overbeek[1], Jefferey Killen[1], David White[1], Meredith Reid[2], Noah Salibi[2], Thomas Denny[2], Ellis Charles[3], Adrienne Lahti[1]

*[1]University of Alabama at Birmingham; [2]Auburn University; [3]Louisiana Tech*

**Background:** Schizophrenia (SZ) is an illness whose heterogeneity has impeded understanding the underlying pathophysiology. In order to better understand this heterogeneity, here we used magnetoencephalography (MEG), 7T Magnetic Resonance Spectroscopy (MRS), and 7T fMRI during the Stroop task, on the same set of patients with first episode psychosis (SZ).

**Methods:** 22 minimally treated first episode SZ and 24 healthy controls (HC) matched for age, gender, and family socio-economic status were recruited. Neurometabolite levels were obtained from the bilateral anterior cingulate cortex using 7T proton MRS with an ultra-short echo time (5 ms) STEAM sequence, and referenced to water. MEG was performed in a 4D systems 148 channel magnetometer, and both the auditory evoked potential to 40 Hz tone clicks, and the resting state (eyes closed) were recorded. The fMRI BOLD response to the Stroop task was also recorded in a 7T scanner.

**Results:** The magnitude of the audio-evoked MEG responses to 40 Hz tone clicks was not significantly different between SZ and HC. However, many SZ showed high levels of theta-band activity during the resting state. The ratio of theta to alpha band activity in the anterior MEG sensors significantly differentiated SZ from HC, $P<0.05$ by t-test. MRS levels of glutamate and total NAA (tNAA), also separated HC from SZ, $P<0.05$, t-test. An across-groups whole brain analysis of the Stroop fMRI BOLD response to incongruent trials relative to congruent trials was performed ($p < .01$, FDR-corrected). The strongest signal came from a region in the left parietal (MNI, -30, -58.5, 48), and between-group analysis of the BOLD signal from a 4mm sphere surrounding this location revealed that the activation was greater for SZ than HC by t-test, $P<0.05$. The MEG theta/alpha ratio, and the left parietal fMRI Stroop effect, were significantly correlated, $r=0.45$, $P=0.005$. However, the fMRI Stroop was uncorrelated with the MRS tNAA ($r = -0.11$, $P=0.491$) and also uncorrelated with the MRS glutamate levels ($r = -0.16$, $P=0.334$).

**Discussion:** We speculate that the MEG and fMRI data, and the MRS neurometabolite levels, may reflect two relatively independent underlying pathological mechanisms in SZ. Possibly the MEG and fMRI results are indicative of dysfunction in long-range cortical-cortical networks, while the MRS data is more indicative of local neurometabolic dysfunction. Further exploration of SZ using multiple imaging modalities on the same subjects may help to untangle the underlying pathophysiological basis of the heterogeneity of this disorder.

### T20. SEARCHING FOR NOVEL AUTOANTIBODIES WITH CLINICAL RELEVANCE IN PSYCHIATRIC DISORDERS

Mats Persson*[,1], Arasch Zandian[2], Louise Wingård[1], Hanna Nilsson[1], Evelina Sjöstedt[3], Daniel Johansson[1], David Just[2], Cecilia Hellström[2], Mathias Uhlén[2], Jochen Schwenk[2], Anna Häggmark-Månberg[2], Oscar Norbeck[1], Björn Owe-Larsson[1], Peter Nilsson[2]

*[1]Karolinska Institutet; [2]Royal Institute of Technology; [3]Uppsala University*

**Background:** Immunological reactions may have a role in subgroups of patients suffering from psychiatric disorders. Possible markers for such subgroups may be autoantibodies of currently unknown nature. If identified, they could indicate which patients that would benefit from immunomodulatory treatment in addition to standard interventions. Modern proteomic methods allow analyses of antibody binding to thousands of different human proteins, facilitating the identification of currently undiscovered autoantibodies.

**Methods:** We have explored the association between any seroreactivity in plasma samples from first episode psychosis patients against more than 2000 randomly chosen protein fragments derived from human proteins, and the development of disorders characterized by chronic or relapsing psychotic symptoms. Plasma from 53 patients and 41 non-psychotic controls were assessed; the clinical course of the patients were followed for a mean duration of 7 years. The plasma samples were analyzed for IgG reactivity to 2304 fragments (approx 100 a.a. residues in length) of human proteins using a multiplexed affinity proteomic technique, and positive hits validated for binding in two additional assays.

**Results:** Thirty patients were diagnosed with schizophrenia, delusional disorder, schizoaffective disorder, bipolar disorder or a long-term unspecified nonorganic psychosis during follow-up, while 23 patients achieved complete remission. Eight patient samples showed autoreactivity to the N-terminal fragment of the PAGE protein family (PAGE2B/PAGE2/PAGE5), whereas no such autoreactivity was seen among the controls. PAGE autoreactivity was associated with a significantly increased risk of being diagnosed with schizophrenia during follow-up (odds ratio 6.7). An antisera raised against the N-terminal fragment stained an unknown extracellular target in human cortical brain tissue (Zandian et al., Transl Psychiatry 7: e1177; doi:10.1038/tp.2017.160).
We are currently investigating the identity of this target. In addition, two other putative new autoantibodies found primarily among the patients, and rarely in the controls, will be discussed at the meeting.
**Discussion:** Our findings suggest that autoreactivity to the N-terminal portion of the PAGE protein family is associated with schizophrenia in a subset of patients with first-episode psychosis. In addition, we propose that searching for novel autoantibodies in an unbiased way may be feasible using state-of-the-art proteomic methods, and can yield useful biological markers for immune involvement in subgroups of individuals diagnosed with psychiatric disorders.

### T21. ALTERATIONS OF CRY2 AND PER3 GENE EXPRESSION ARE ASSOCIATED WITH GRAY MATTER ABNORMALITIES OF THALAMIC-LIMBIC NETWORK IN UNIPOLAR DEPRESSION AND BIPOLAR DEPRESSION

Chengcheng Zhang*,[1], Peiyan Ni[1], Tao Li[1]
[1]*West China Hospital, Sichuan University*

**Background:** The current study aimed to identify shared and distinct brain structure abnormalities and their relationships with circadian gene expression in patients with bipolar depression and unipolar major depression.
**Methods:** A total of 103 subjects participated in this study, including 32 patients with bipolar depression (BDP), 26 patients with unipolar depression (UDP) and age, sex-matched 35 healthy controls were conducted brain structural magnetic resonance imaging scans and then used optimized voxel-based morphometry to explore group differences in regional gray matter volume (GMV). Circadian gene mRNA expressions in peripheral blood were measured on a subset of 12 patients with BDP, 19 patients with UDP and 33 control subjects using reverse transcription quantitative real-time polymerase chain reaction.
**Results:** The GMV of the thalamus-limbic pathways had significantly increased in BDP cases relative to comparison subjects, while in UDP the increased GMV focused on the thalamus. The circadian-related gene mRNA expressions have significantly decreased in the patients with BDP, however with higher expression levels in the UDP cases. In addition, the GMV of right thalamus in the UDP was positively associated mRNA level of CRY2 gene, while the GMV of right hippocampus in the UDP was negatively associated mRNA level of PER3 gene.
**Discussion:** Our study identified the relationship between abnormalities of thalamic-limbic network and alterations of circadian gene pathway in BDP and UDP. The shared GMV abnormality was the right thalamus. PER3 might be critical to hippocampus dysfunction in UDP, and CRY2 might be critical to thalamus dysfunction at a right-hemisphere function in BDP.

### T22. PITUITARY GLAND VOLUME DIFFERENCES IN INDIVIDUALS WITH PSYCHOSIS: RESULTS FROM THE BIPOLAR-SCHIZOPHRENIA NETWORK ON INTERMEDIATE PHENOTYPES (B-SNIP) STUDY

Synthia Guimond*,[1], Samantha Tingue[2], Gabriel A. Devenyi[3], Yun-Xiang Tang[4], Luke Mike[5], M. Mallar Chakravarty[3], John A. Sweeney[6], Godfrey D. Pearlson[7], Brett A. Clementz[8], Carol A. Tamminga[9], Matcheri S. Keshavan[1]
[1]*Harvard Medical School, Beth Israel Deaconess Medical Center;* [2]*Emmanuel College, Beth Israel Deaconess Center;* [3]*McGill University;* [4]*Second Military Medical University;* [5]*Beth Israel Deaconess Center;* [6]*University of Texas Southwestern;* [7]*Yale University;* [8]*University of Georgia;* [9]*University of Texas Southwestern Medical Center*

**Background:** When exposed to stress, the hypothalamic-pituitary-adrenal axis is hyperactivated, which can cause the enlargement of the pituitary gland. Hence, pituitary gland volume could be a biomarker of stress present in psychosis. However, it remains unclear if individuals with psychosis have larger pituitary gland than healthy people. Previous studies investigating this question used small samples and reported inconsistent results. In the current study, we used an automated multi-atlas segmentation method to investigate the differences between pituitary gland volumes in a large sample of individuals on the psychosis spectrum.
**Methods:** Data collection was completed across six sites in the Bipolar-Schizophrenia Network on Intermediate Phenotypes (B-SNIP) consortium with a total of 755 participants included in the study - 174 individuals with schizophrenia (SZ), 115 with schizoaffective disorder (SZA), 167 with psychotic bipolar disorder (PBD), and 299 healthy controls (HC). Structural magnetic resonance images were acquired and pituitary gland volumes were obtained using the automated MAGeT-Brain algorithm. General linear model and post-hoc independent t-tests were used to analysis the differences between subgroups of patients using clinical diagnosis and agnostic Biotype classification (Biotype 1 being the most cognitively impaired). We also explored potential effect of antipsychotic intake, symptoms severity and duration of illness. In all analyses, we used Bonferroni correction for multiple comparisons and entered confounds as covariates (age, sex, race, intracranial volume, and site).
**Results:** Overall, the pituitary gland volumes were not significantly different between patients and HC. No significant main effect of diagnosis was observed, but SZ patients had trending larger pituitary volume compared to HC ($p=.033$, uncorrected). We observed a significant main effect of Biotype ($p=.003$), with Biotype 1 having significantly larger pituitary gland than HC and Biotype 2 ($p=.004$ and $p=.013$). In the patients group, no significant relationship between the pituitary gland and the amount of antipsychotic intake was observed ($r=.02$, $p=.68$). Significant correlations with the pituitary gland volume were observed with symptoms severity ($r=.22$, $p=.000$), and with the duration of illness ($r=-.18$, $p=.002$). Importantly, Biotypes did not significantly differ in terms of symptoms severity nor duration of illness.
**Discussion:** As a group, individuals with psychosis do not have abnormal pituitary gland volume, but larger pituitary gland is related to shorter duration of illness and greater symptoms severity. Therefore, larger pituitary gland volume could be a state-related biomarker of psychosis. Moreover, while we did not observe any significant subgroup differences using clinical diagnosis, our results suggest an increase in pituitary volume in biotype 1 patients compared to HC. These findings clarify previous inconsistent reports, and encourage further investigation of stress biomarkers in individual with psychosis with lower cognitive abilities. In the future, this could lead to the development of more targeted treatments for this specific subgroup of patients.

### T23. DYNAMICS OF NEURONAL METABOLISM AFTER THE ACUTE ONSET OF PSYCHOSIS – A TWO YEARS FOLLOW-UP 1H/31P-MR-SPECTROSCOPY STUDY IN NEUROLEPTIC NAÏVE UHR TRANSITION PATIENTS

Stefan Smesny*,[1], Diana Berberich[1], Alexander Gussew[1], Kerstin Langbein[1], Mario Walther[2], Juergen Reichenbach[1]
[1]*University Hospital Jena;* [2]*University of Applied Sciences Jena*

**Background:** Glutamatergic dysfunction, deregulated mitochondrial metabolism and alterations of membrane phospholipids are considered as core pathology of psychosis, and have been studied in schizophrenic illness using magnetic resonance spectroscopy (MRS). Combining 1H- and 31P-MRS, this study investigates these aspects in Ultra-high risk (UHR-T) patients right after transition to psychosis (T0) and after a two years interval (T1) in a naturalistic longitudinal design, including treatment as usual by cognitive-behavioral therapy (CBT) and pharmacotherapy with second generation antipsychotics.
**Methods:** We applied 3 T chemical shift imaging (3D 31P-MRS, 2D 1H-MRS) and hippocampal single-voxel 1H-MRS in 29 neuroleptic-naïve UHR-T patients and 27 healthy controls matched for age and gender. Glutamate (Glu) and N-acetyl-aspartate (NAA) reflect neuronal functioning, phosphocreatine (PCr), adenosine triphosphate (ATP) and NAA indicate mitochondrial function and energy metabolism, and phosphomono- and diester indicate the balance of phospholipid synthesis (PME) and -breakdown (PDE). Psychopathology was assessed using the CAARMS, BPRS-E and SCL-90-R. Generalized linear mixed models were used to examine case-control differences in metabolite changes over time, and associations with clinical improvement.
**Results:** At T0, cross-sectional analysis revealed decreased NAA, Glu and PME levels in the left dorsolateral prefrontal cortex (DLPFC) and thalamus of UHR-T patients as well as higher PCr and lower PDE levels in the right hippocampus. (ii) Follow-up analysis (T1) showed in patients a significant increase of Glu in the bilateral DLPFC and the right thalamus, while a decrease of PCr was observed in the right hippocampus.
**Discussion:** The observed metabolite pattern at T0 likely reflects a hypofunction of glutamatergic neurons and a disturbance of membrane phospholipid turnover in fronto-thalamo-hippocampal networks during the first acute onset phase of psychotic illness. The pattern of changes at T1 is suggestive for an improvement of neuronal functioning in these networks that is caused by therapy, and presumably underlies the observed clinical improvement in terms of negative symptoms and cognitive impairment.

### T24. REDOX REGULATORS AND OXIDATIVE STRESS IN SCHIZOPHRENIA

Havard Bentsen*,[1], Dag Solberg[2], Helge Refsum[2], Ole Andreassen[3]
*[1]Haukeland University Hospital; [2]Diakonhjemmet Hospital; [3]Oslo University Hospital*

**Background:** Elevated levels of oxidative stress have been reported in schizophrenia and may play a role in the underlying psychopathology. The antioxidant defense system may be disrupted in schizophrenia. We have earlier shown how levels of membrane polyunsaturated fatty acids (PUFA) change during the course of schizophrenia, increasing from a low level in a subgroup, remaining stable in another. Here, we aimed at comparing levels of redox regulators, oxidative stress, and related genotypes in schizophrenia patients and healthy controls, and at identifying how these biomarkers are related to membrane fatty acids and clinical characteristics in acute and chronic phase of schizophrenia.
**Methods:** Patients with schizophrenia spectrum disorders (n=55) examined during an acute phase and five years later during a chronic phase, and healthy controls (n=51) were included. We assessed blood levels of redox regulators [(alpha-tocopherol, bilirubin, uric acid, glutathione (GSH), glutathione peroxidase (GPx)], glutathione reductase (GR), markers of oxidative stress [F2-isoprostane, reactive oxygen metabolites (D-ROMs)] and PUFA. We examined genotypes and gene expression related to glutamate cysteine ligase (GCL), the rate-limiting enzyme of GSH synthesis, and its catalytic (GCLC) and modulator (GCLM) subunits. Links between redox measures and Positive and Negative Syndrome Scale (PANSS) were studied.
**Results:** In the chronic phase, levels of alpha-tocopherol (p=0.03) and bilirubin (p<0.001) were lower, while uric acid (p=0.02) was higher in patients than in controls. In patients, the levels of alpha-tocopherol were higher in the acute phase than in the chronic phase (p=0.001). However, the changes depended on the PUFA levels in the acute phase. In the low PUFA group, alpha-tocopherol levels remained stable, whereas in the high PUFA group, they decreased to those of the low PUFA group. Levels of D-ROMs were linked to PUFA (r=0.39, p=0.007) and long-chain PUFA (r=0.42, p=0.003) in controls but not in patients.
There was no significant difference in the distribution of GCLC genotypes between patients and controls. Compared to other GAG trinucleotide-repeat (TNR) genotypes, 7/9 GAG genotype was linked to higher gene expression of GCL (mRNA GCLC, p=0.049; mRNA GCLM, p=0.02), higher levels of GSH in blood (p=0.02) and higher GR activity in blood (p=0.007). Only when combined with C-129T high risk genotype, the 7/7 GAG genotype induced a strong reduction of CGLC expression (p=0.045, bootstrapping 10000 samples). Gene expression related to glutathione synthesis was non-significantly (p=0.10–0.19) higher among patients. Levels of long-chain PUFA were significantly positively linked to gene expression of glutathione related enzymes.
**Discussion:** The findings of abnormal levels of alpha-tocopherol, bilirubin, uric acid and glutathione synthesis in the chronic phase of schizophrenia indicate oxidative stress. Dysregulation of antioxidant defenses may be involved in the pathophysiology of schizophrenia and warrants further experimental studies.

### T25. ALTERATIONS OF PLASMA PHOSPHOLIPID FATTY ACID PROFILES ARE ASSOCIATED WITH LOCAL BRAIN STRUCTURAL ABNORMALITIES IN NEUROLEPTIC NAÏVE FIRST-EPISODE PSYCHOSIS PATIENTS

Kerstin Langbein*,[1], Christian Fleischer[1], Katrin Kuhnt[2], Christian Gaser[1], Stefan Smesny[1]
*[1]Jena University Hospital; [2]Friedrich-Schiller-University Jena*

**Background:** Alterations in brain structure are among the most robust biological findings in schizophrenia. Also changes in fatty acid profiles including reduced levels of polyunsaturated fatty acids (PUFA) are well replicated in schizophrenia. Being essential for neurodevelopmental processes and structural plasticity and remodeling, alterations of PUFA metabolism might be also associated with the occurrence of brain structural abnormalities. To investigate this assumption in vivo we examined the interrelation of PUFA profiles and brain structure in neuroleptic naïve first-episode psychosis (FEP) patients and healthy controls (HC) matched for age and gender.
**Methods:** High-resolution T1-weighted 3T MRI were acquired from 29 FEP patients (age 26.4 ± 5.3y; 13 females/16 males) and 31 HC (age 25.1 ± 4.7y; 14 f/17 m). Fatty acid profiles were analyzed using gas chromatography in the plasma phospholipid (PL) fraction that is rather independent of recent fat consumption, and that potentially indicates PUFA availability in phospholipid structural components essential in the brain. To investigate brain structural abnormalities in FEP and effects of illness on interactions between fatty acid profiles and local grey (GM) or white (WM) matter density, voxel-based morphometry (VBM) with the computational anatomy toolbox (CAT12) was used.
**Results:** VBM analyses revealed a reduction of GM in FEP in left frontal operculum, left middle frontal gyrus, left superior frontal gyrus and bilateral temporal gyrus (TFCE, FWE<0.05). The group comparisons of fatty acid profiles in the PL fraction showed reduced omega-6 PUFA and MCFA (C10-C14) levels in FEP patients. Interaction analyses revealed an influence of illness on the association between omega-6 PUFA and GM density at the left supramarginal gyrus/left postcentral gyrus and left superior temporal gyrus (TFCE, FWE<0.05) with a regression slope FEP patients > HC. For MCFA interaction analyses revealed effects of illness on associations with GM density in the bilateral superior medial frontal gyrus and left anterior cingulate gyrus with a regression slope HC > FEP patients.
**Discussion:** Our results support the notion that the availability of PUFA and MCFA potentially affects brain structural development and remodeling.

They also support the notion of PUFA and phospholipids metabolism as a biochemical basis to create early prevention and intervention strategies, e.g. by PUFA supplementation.

### T26. PERINATAL STRESS AND PSYCHOSIS: RESULTS FROM THE BOLOGNA FIRST EPISODE PSYCHOSIS (BO-FEP) STUDY

Ilaria Tarricone[1], Marcello Lanari[1], Marta Di Forti*[,2], Robin Murray[2], Domenico Berardi[1]
*[1]Bologna University; [2]Institute of Psychiatry, King's College London*

**Background:** According to the gene-environment interaction model the pathogenesis of psychosis relies on an adverse neuro-socio-developmental pathway. Perinatal stress represents an important risk factor for the development of psychosis because it could interfere with socio-neuro-development early in life. We aim to investigate the correlation of perinatal risk factors with the onset of psychosis.
**Methods:** Case-control – incidence study. Patients (and their mothers) were eligible if they presented for the first time with first episode psychosis at the Bo West CMHC between 2002 and 2012. The Bo West CMHC serves a catchment area of about 200,000 people. The controls were recruited in the same catchment area and study period.
**Results:** 42 patients and 26 controls and their mothers were included. Adjusted logistic regression showed that psychosis onset was significantly associated with: stressful situations during pregnancy; lower level of maternal physical health before or during pregnancy; use of anti-inflammatory drugs during pregnancy; low level of maternal education.
**Discussion:** The results of our study suggest that stress during perinatal period increases the risk of developing psychosis. More attention should be given to the containment of perinatal stress and the prevention of its adverse effects on mother and child mental health.
Tarricone I, Mimmi S, et al.. First-episode psychosis at the West Bologna Community Mental Health Centre: results of an 8-year prospective study. Psychol Med. 2012 Nov;42(11):2255–64. PubMed PMID: 22404368

### T27. DYSFUNCTIONAL ATTITUDES IN ADOLESCENTS WITH EARLY-ONSET PSYCHOSIS: PRELIMINARY RESULTS

Olga Puig-Navarro*[,1], Francina Badia[1], Yuli Gallegos[3], Mireia Forner[1], Inmaculada Baeza[1], Gisela Sugranyes[1], Eva Varela[1], Anabel Martinez-Aran[4], Eric Granholm[5]
*[1]Hospital Clinic of Barcelona; [3]VA San Diego Healthcare System; [4]Hospital Clínic of Barcelona, CIBERSAM; [5]University of California San Diego, VA San Diego Healthcare System*

**Background:** Dysfunctional attitudes such as defeatist performance beliefs (DPB) and asocial beliefs (AB) have been linked to negative symptoms and functional outcome in chronic schizophrenia (Campellone et al., 2016; Grant and Beck, 2010) and in adults with recent onset schizophrenia (Ventura et al., 2014). Adolescents with early-onset psychosis (EOP) are at major risk of having persistent negative symptoms (Puig et al., 2017) but no previous study has examined dysfunctional attitudes in this population. We aimed: (1) to examine if more DPB and AB were present in adolescents with EOP compared with normal controls, and (2) to study the relationships between DPB and AB with symptoms and functioning.
**Methods:** Sample: 15 adolescents with EOP (11♀, age=15.33 ± 1.23) and 10 healthy controls (8♀, age=15.60 ± 1.51), participants in a trial about cognitive and behavioral social skills treatment in EOP developing in Hospital Clínic of Barcelona (baseline data). Patients were under antipsychotic meds. Inclusion criteria: having an early-onset schizophrenia spectrum disorder diagnosed between 9–18 years-old (schizophrenia, schizoaffective disorder, psychosis n.o.s.); being within the 5 first years after the disease onset; clinical stability. Exclusion criteria: IQ<70; comorbid substance dependence disorder; neurological disorders. Instruments: PANSS (Kay et al., 1987), Calgary Depression Scale (CDS, Addington et al., 1990), Children's Global Assessment Scale (C-GAS, Shaffer et al., 1983), Social and Role Functioning Scales (GF:S, GF:R, Cornblatt et al., 2007), Life Skills Profile - Adolescent version (Puig et al., 2013), Dysfunctional Attitudes Scale - Spanish version (DAS, Sanz et al., 1993); Asocial Beliefs Scale (Grant and Beck, 2010). DPB was extracted from the DAS scale following Beck et al. (2013). Groups were compared with T-test or Chi-square tests. Pearson correlations were computed for examining relationships between variables.
**Results:** EOP and control groups were homogeneous in age (t=-0.49, p=0.632) and sex (X2=0.15, p=0.702). All subjects were living with their parents. Family SES was lower in EOP group (X2=10.69, p=0.030). All subjects but one patient were studying although patients had repeated more courses (t=4.00, p=0.001). EOP subjects had a predominance of negative symptoms (positive symptoms=14.07 ± 3.85; negative symptoms=23.50 ± 13.79; general symptoms=31.86 ± 7.28). Mean score of CDS in EOP was low (3.77 ± 4.76). EOP group had lower scores in all functional measures than controls (C-GAS: 51.14 ± 9.38 vs 93.70 ± 4.45, GF:S: 5.71 ± 1.07 vs 9.00 ± 0.47, GF:R: 5.21 ± 1.12 vs 8.80 ± 0.42, LSP: 67.64 ± 12.30 vs 44.13 ± 3.60; t>6.67, p<0.001). Regarding dysfunctional attitudes, EOP group had higher scores in dysfunctional attitudes scales than healthy controls (DPB: 55.60 ± 19.57 vs 33.00 ± 9.37, AB: 6.67 ± 3.42 vs 3.00 ± 2.11; t>3.02, p<0.006). In EOP, DPB and AB scores were negatively correlated with functioning (DPB: C-GAS r=-0.60, GF:S r=-0.66, GF:R r=-0.66, LSP r=0.65; p<0.002; AB: C-GAS r=-0.45, GF:S r=-0.55, GF:R r=-0.49, LSP r=0.53; p<0.029). No significant correlation were found between dysfunctional attitudes and negative symptoms in EOP.
**Discussion:** These preliminary results showed that adolescents with EOP had higher levels of dysfunctional attitudes than controls. Accordantly to recent literature, defeatists performance beliefs and asocial beliefs were correlated with poorer functioning. However, dysfunctional attitudes were not associated with negative symptoms. It might be that dysfunctional attitudes do not contribute to negative symptoms but in functional outcome in youngs with EOP although larger samples are required. Acknowledgments: Fundación Alicia Kolplowitz.

### T28. QUALITY OF PARENTAL BONDING AMONG INDIVIDUALS WITH ULTRA-HIGH RISK OF PSYCHOSIS AND SCHIZOPHRENIA PATIENTS

Oon Him Peh[1], Attilio Rapisarda*[,1], Kang Sim[1], Jimmy Lee[1]
*[1]Institute of Mental Health*

**Background:** A child's parental bonding, measured using the Parental Bonding Instrument (PBI), has been found to be associated with psychiatric illnesses. In particular, a significantly higher proportion of patients with schizophrenia tend to report affectionless-controlling mothers as compared to healthy controls.
This study aims to (i) investigate the applicability of the PBI tool in Singapore, using exploratory factor analysis, and (ii) explore the association between parental bonding, symptom severity and functioning across schizophrenia patients, individuals at ultra-high risk of psychosis (UHR), and healthy controls.
**Methods:** Data from 59 schizophrenia patients, 164 UHR, and 510 healthy controls (N = 733) were collected. The Structured Clinical Interview for DSM-IV (SCID) was used to ascertain any psychiatric diagnoses. Positive and Negative Symptoms of Schizophrenia (PANSS) and Global Assessment of Functioning (GAF) were administered on UHR and patients. Social and Occupational Functioning Assessment Scale (SOFAS) was administered on HC and UHR. Calgary Depression Scale for Schizophrenia (CDSS) was administered on UHR only.

Two exploratory factor analyses of the PBI were conducted on maternal items and paternal items (oblimin rotation). PBI factor scores were calculated for each individual and compared across groups using one-way ANOVA. Multivariate backward regressions were conducted to elucidate the association(s) between parental bonding factors and the clinical scales.
**Results:** Factor analyses revealed three-factor solutions for both maternal and paternal items, with factors 'care', 'autonomy', and 'overprotection'. All the original 'care' items loaded onto the 'care' factor for maternal and paternal analyses. The original 'control' items were split into 'autonomy' (the degree to which children were allowed to make their own decisions, e.g. 'gave me as much freedom as I wanted') and 'overprotection' (e.g. 'felt I could not look after myself'). Fit statistics suggested a good fit for both maternal items and paternal items (CFI > 0.9, TLI > 0.9). UHR were 1.61 times as likely to report affectionless-controlling mothers (OR = 1.61, 95% CI: 1.13–2.30, p = .008) and 0.52 times as likely to report having optimal mothers (OR = 0.52, 95% CI: 0.29–0.93, p = 0.028). No significant differences in paternal styles were reported.
Compared to HC, patients and UHR reported significantly lower maternal care ($F(2,729) = 27.51$, $p < .001$), higher maternal overprotection ($F(2,729) = 17.00$, $p < .001$) and paternal overprotection ($F(2,711) = 9.30$, $p < .001$) (bonferroni-corrected). Among UHR, higher paternal overprotection was significantly associated with higher total PANSS scores ($\beta = .162$, $p = .045$), higher PANSS general psychopathology subscores ($\beta = .185$, $p = .022$), lower GAF scores ($\beta = -.188$, $p =.021$), lower SOFAS scores ($\beta = -.183$, $p = .024$), and worse CDSS scores ($\beta = .210$, $p = .009$). Among patients, higher maternal overprotection ($\beta = .444$, $p = .022$) and paternal care ($\beta = .400$, $p = .036$) were associated with higher GAF functioning.
**Discussion:** This psychometric investigation of the PBI among Asian participants yielded three-factor models, which deviate from the original two factors. Our findings replicate previous evidence of higher proportion of affectionless-controlling mothers among UHR and patients. Lower maternal care, lower maternal and paternal overprotection were reported. Paternal overprotection was associated with worse positive and negative symptoms of schizophrenia, worse social, occupational, and psychological functioning, and more severe depressive symptoms among UHR. Our results highlight the importance of addressing childhood parental bonding issues in early intervention services for UHR.

### T29. ELECTRORETINOGRAPHIC RESPONSE IN YOUTHS AT GENETIC RISK OF SCHIZOPHRENIA AND BIPOLAR DISORDER AND IN NORMAL CONTROLS: TRANSVERSAL AND LONGITUDINAL DIFFERENCES AND IMPLICATIONS FOR THE RISK TRAJECTORY

Anne-Marie Gagné[1], Thomas Paccalet*,[1], Valérie Jomphe[1], Daphné Lussier[1], Michel Maziade[1]
[1]*Centre de Recherche CIUSSS-CN*

**Background:** Visual defects have been widely reported in major psychosis. This includes altered eye tracking, retinal thinning and electrophysiological anomalies.[1] One of the most replicated alterations is decreased electroretinographic (ERG) responses that are observed in both bipolar disorder and schizophrenia. Our previous study showed a diminished rod b-wave amplitude in a small sample of children born to an affected parent.[2] The fact that an anomaly found in patients would also be observed in children at genetic risk suggests a neurodevelopmental origin and may represent a vulnerability marker. Little data exists on the stability of ERG measures in childhood and adolescence. We wanted to evaluate rod and cone ERG response in larger samples of young offspring of an affected parent (HR) and age and gender balanced controls. By comparing a subsample of 33 offspring to controls, we were able to evaluate the stability and change of ERG over time.
**Methods:** ERGs of 71 offspring (mean age of 19 y.o.) and 224 healthy controls (mean age of 20 y.o.) was recorded. From this sample, 33 HR and 26 healthy controls had ERG recordings at 2 different moments (mean interval of 4 years). We then compared the amplitudes obtained at Time 1 and Time 2 in order to assess whether the ERG amplitudes remained stable or varied over time.
**Results:** Congruent with our 2010 report, this larger HR sample showed a reduced rod b-wave amplitude ($p<.05$). Probably due to higher statistical power, two other differences were found: an increased cone b-wave latency ($p<.05$) as well as a diminished mixed rod/cone ERG amplitude ($p <.05$). None of the ERG amplitudes of the healthy controls changed over time. In contrast, 12 out of 33 HR participant showed a variation of more than one standard deviation (either increase or decrease) on the rod b-wave amplitude which was significantly more frequent than in healthy controls (2/26; $p<.05$). Change in offspring occurred in both directions: some of them had an increased ERG amplitude response that was sufficient to end up in the confidence interval of the controls whereas others experienced a decreased of their rod amplitude over time.
**Discussion:** These young high-risk offspring displayed three ERG anomalies that we have already reported in adult patients.[2] Our finding bolstered the evidence that ERG anomalies observed in patients may have neurodevelopmental or childhood roots. We observed only little variation in the ERG of the healthy controls over time in that early age range and this appears concordant with existing literature. Of particular interest is the finding that rod b-wave amplitudes were diminished in patients and in offspring. The offspring also showed increased variability over time in comparison to controls. Future studies will seek to understand the relationship between the transversal or longitudinal patterns of rod b-wave amplitudes, as an indicator of risk, and the risk endophenotypes previously reported in the children born to an affected parent.[3,4]
**References:**
1. Gagné et al. Prog Neuropsychopharmacol Biol Psychiatry. 2015
2. Hébert et al. Bill Psychiatry, 2010
3. Maziade, N Eng J Med, 2017
4. Paccalet et al., Schizophr Res, 2016

### T30. TIPPING POINTS – PREDICTING TRANSITIONS TO PSYCHOSIS IN AT-RISK YOUNG PEOPLE

Jessica A. Hartmann*,[1], Patrick D. McGorry[1], Marieke Wichers[2], Barnaby Nelson[1]
[1]*Orygen, the National Centre of Excellence in Youth Mental Health;* [2]*University of Groningen, University Medical Center Groningen, Interdisciplinary Center Psychopathology and Emotion regulation*

**Background:** In traditional psychosis prediction research, the assumption is that a single "snapshot" of clinical disturbance at time point one (i.e. baseline) can reliably predict the future emergence of psychosis over time (i.e., follow-up). This is a linear, static approach to psychosis prediction. However, the field increasingly recognizes that mental health behaves as a non-linear, dynamic system, common to other complex structures such as ecosystems, financial markets or the climate.
**Methods:** Increasing evidence points toward the existence of generic "tipping points" in these complex dynamic systems. A tipping point refers to a critical threshold whereby a system shifts from one state into another. Evidence suggests there are universal early warning signals/resilience indicators (such as a phenomenon called 'critical slowing down'), which predict close proximity to a critical tipping point.
**Results:** There is growing evidence for the presence of these early warning signals in psychopathology. This presentation will introduce theoretical concepts of tipping points and resilience indicators in the context of transitioning from at-risk mental state to frank psychosis.

**Discussion:** This new framework may represent a paradigm shift from static prediction approaches to dynamic, individualized models of psychosis prediction and may inform the development of new clinical identification tools and early and individualized interventions to prevent such transitions.

### T31. TEN YEAR CLINICAL FOLLOW-UP OF YOUTH WITH EARLY ONSET PSYCHOSIS

Sugranyes Gisela*[,1], Elena De la Serna[2], Marina Redondo[1], Daniel Ilzarbe[1], Josefina Castro-Fornieles[3], Inmaculada Baeza[1]
*[1] Hospital Clínic of Barcelona; [2]Centro De Investigación Biomédica En Red De Salud Mental, CIBERSAM; [3]Hospital Clínic de Barcelona, Centro de Investigación Biomédica en Red de Salud Mental, CIBERSAM*

**Background:** Early onset psychosis (onset prior to age 18) may be associated with poorer long-term outcomes than when onset takes place in adulthood. Studies so far prospectively following youth with early onset psychosis have suggested that only 20% of patients experienced a "good" outcome, and that nearly 50% experience a "poor" outcome. However, the major body of evidence so far has focused on cohorts recruited over 20 years ago, while a recent review (Clemmensen 2012) has noted that outcomes may be more favourable in more recent studies.
We set out to update the evidence by prospectively assessing youth with a first episode of early onset psychosis after a 10 year follow-up period with clinical and functional measures.
**Methods:** Patients were recruited from a child and adolescent psychiatry unit at a university hospital in Barcelona, Spain between 2003 and 2008. Inclusion criteria were: age 7 to 17 years and presence of positive psychotic symptoms of less than 6 months duration. Exclusion criteria: presence of a concomitant Axis I disorder that could account for the psychotic symptoms (e.g., substance abuse, autistic spectrum disorders, post-traumatic stress disorder, or acute stress disorder); learning disability according to DSM-IV criteria; neurological disorders. Occasional substance use was not an exclusion criterion. A sample of age matched healthy controls was recruited from the same geographical area. Controls had the same exclusion criteria in addition to no family history of psychotic disorders in first degree relatives. All participants were assessed with clinical (K-SADS, PANSS, GAF, CGI) measures by a psychologist and psychiatrist with experience with child and adolescent population at baseline and 10 year follow-up.
**Results:** Sixty-nine patients and thirty-one controls were assessed at baseline; 36 patients (52%) and 24 controls (77%) were re-assessed at ten year follow-up. There were no differences in age (M=26.4 SD=1.4 vs. M=26.0 SD=2.0; t=.84; p=.41) or gender (47% vs. 46% females, Chi square=.011; p=.92). We were unable to locate 9 patients (13%) and 4 controls (12.5%). Two patients were deceased at follow-up (one committed suicide; one from metastasic cancer), one patient was in prison, and the rest declined participation. Patients who attended follow-up had trend-level baseline poorer functioning (17.85 vs. 22.4, p=.065), and significantly greater baseline clinical severity 6.44 vs 6.0, p=.017) than those who did not. Diagnoses at baseline were as follows: n=16 (23.2%) schizophrenia; n=20 (29%) affective disorders with psychotic symptoms; n=29 (42%) psychosis not otherwise specified. At follow-up: n=18 (50%) schizophrenia, n=11 (30.6%) affective disorders, n=1 (2.8%) personality disorder; n=1 (2.8%) eating disorder and n=5 (13.9%) had no psychiatric diagnosis. Twenty patients (58.8%) had been hospitalized during the follow-up period and thirty (88.2%) were currently receiving at least one antipsychotic drug. Seven patients (19.4%) were categorised as "poor outcome" (GAF < 50), 18 patients (50%) as "moderate" outcome (GAF 51–70) and 9 patients (26.5%) as "good outcome" (GAF >70). Seven patients (19.4%) were in full time employment and five (13.9%) were in full time education. The rest were either unemployed or working part time or in an assisted setting.
**Discussion:** Early onset psychosis is associated with poor long term outcomes in a portion of patients, although current functional outcomes in these patients may be comparatively better in relation to data from historical reports (Clemmensen 2012). Factors related to improved healthcare services, such as reduction in duration of untreated psychosis and new treatment modalities may potentially underlie these differences.

### T32. USING PSYCHOSOCIAL INTERVENTION TO ENHANCE KNOWLEDGE OF ATTENUATED PSYCHOSIS SYMPTOMS AND HELP SEEKING BEHAVIORS AMONG AFRICAN AMERICAN YOUNG ADULTS

Huijun Li*[,1], Tanisha Pelham[2]
*[1]Florida A&M University; [2]Florida State Hospital*

**Background:** The lack in knowledge of mental illnesses is of primary concern with regard to help-seeking and treatment outcomes, especially when faced with chronic and severe illnesses such as psychotic disorders. Where mental health knowledge lacks, so does the ability to recognize the signs and symptoms, risk factors, and causes of mental disorders; as well as the appropriate routes of care for these illnesses. Psychotic disorders and attenuated/subclinical psychotic symptoms are often the target of stigma due to the distinctive symptoms, disruptive behavior and perceived dangerousness of both. Furthermore, the social stigma and discrimination historically faced by African Americans in the United States magnifies the disparity in treatment outcomes among this population. The enrollment of minority college students has increased from 15 percent to 33 percent over the past three decades; cases of students with mental illnesses have also increased. It is becoming more important to explore psychosocial intervention strategies geared to promote knowledge of attenuated psychotic symptoms and help-seeking behavior among African Americans young adults.
**Methods:** The sample consists of 177 students from a Historically Black College and University (HBCU) in the Southeast region of the United States. The participants ranged in age from 18–25. A within group test-retest design was used to conduct the study. The participants received a pretest, participated in a psychosocial training on attenuated psychosis syndrome, and a posttest.
**Results:** The results suggest that the training was effective in enhancing the participants' knowledge of early warning signs of psychosis and improving their help-seeking behavior. However, stigma unexpectedly increased after the training.
**Discussion:** Discussion: Enhancing mental health literacy has implications for influencing the effects of stigma and discrimination. Colleges and universities are optimal settings for improving mental health literacy because of the high-risk age groups served at these institutions. Mental health literacy is an important life skill that should be taught before the need arises. In order to increase the likelihood that African American college students seek the appropriate help for mental health problems and understand the effects of stigma on help seeking behaviors, more cultural specific interventions are necessary among this population. Interventions should include strategies to cope with stigma and discrimination in order to reduce the effects of both. Future research in this area should also consider how one's ethnic identity correlates with stigma and help seeking behavior.

### T33. EARLY INTERVENTION FOR EARLY PSYCHOSIS IN FRANCE, MAPPING OF PROGRAMS

Sophie Meunier-Cussac*[,1], Guy Gozlan[2], Laurent Lecardeur[1], Anne Duburcq[3], Laurène Courouve[3]
*[1]EMSI CHU Caen; [2]SAMSAH Prepsy; [3]Cemka*

**Background:** Early intervention programs (EIP) have been developed in many countries (United States, Europe, Canada, Australia) and are now widely considered effective in the treatment of early psychosis.

In France, current national policies in the field of mental health promote the development of early intervention but France has not yet met national standards of care for EIP.
A recent report from the London School of Economics (2016) comparing different European countries even mentioned the delay of France in this area, referring to only one EIP in the country.
However, different early intervention initiatives have emerged in France during the last decade without such information being centralized and therefore with no visibility on the current situation over the whole territory.
The aims of this study were to draw up a comprehensive inventory of existing or planned programs (in metropolitan France and in the overseas territories) in 2017 and to describe how they operate in 2016.
**Methods:** This was a two-phase study; phase one was to identify and create an inventory of existing initiatives, and phase two was to describe and conduct an analysis of each initiative.
To be included, identified initiatives had to offer an early, intensive and multidisciplinary approach with at least 0.5 dedicated full-time equivalent staff. A secondary inclusion criterion concerned the out-patient setting of the initiative.
Inventory was achieved through many contacts across the country, among physicians/psychiatrists, healthcare facilities (hospitals, clinics, adolescent centers…) or administrative institutions (Health Regional Agencies…) which may either provide this kind of care or know of such initiatives.
An online declarative survey was administered between March and July 2017 to the identified psychiatrists with questions that covered administrative and clinical topics: structure of attachment, dedicated team, funding, targeted population, activity in 2016, partners of the program, difficulties encountered and prospects.
**Results:** Between March and July 2017, 37 EIP for management of early psychosis were identified in France: 18 were operational, 8 were being established, and discussions were under way for the remaining 11.
The 18 identified operational programs were located throughout the country with a few regional disparities. All programs operated with multidisciplinary teams, including at least one psychiatrist and one nurse, and with a mean of 4.3 dedicated full-time equivalents healthcare workers (median: 3.7).
Most programs offered case management (12/18), with caseloads ranging from 4:1 to 22:1. The mean caseload was 10:1 (standard deviation 8:1).
All programs included 15 to 35 year-old early psychosis patients. Four programs also included patients at ultra-high risk for psychosis (UHR), while 4 others continued patient management during the chronic stages; 4 initiatives included all these stages of the disease.
Half of the programs had been existing for 2 to 5 years (50%); 89% were created less than 5 years ago.
The surveyed professionals described an increasing number of patients under their care.
**Discussion:** Numerous projects and discussions appear to be under way (some programs should open very soon).
A real dynamic has been launched in France with an increasing focus and this evaluation will help to improve visibility of the identified programs and promote the development of new programs.

### T34. SUBMISSION WITHDRAWN .

### T35. DIFFUSION MEASURES OF EXTRACELLULAR FREE WATER IN A NON-HUMAN PRIMATE MODEL OF MATERNAL IMMUNE ACTIVATION: EXPLORING NEUROIMMUNE MECHANISMS OF PSYCHIATRIC DISORDERS

Cameron Carter*,[1], Tyler Lesh[2], Costin Tanase[2], Jeffrey Bennett[2], Ana-Maria Iosif[2], Judy Van Der Water[2], Richard Maddock[2], David Amaral[2], Melissa Baumann[2]
*[1]UC Davis Health System, Imaging Research Center; [2]UC Davis*

**Background:** Evidence has been accumulating for an immune-based component of psychiatric disorder etiology, particularly schizophrenia. Early epidemiological studies found an increased incidence of schizophrenia in offspring of mothers who had an infection during pregnancy. Recent work has identified genetic links to the MHC complex, pro-inflammatory cytokine elevations in cerebrospinal fluid and plasma. We have developed a non-human primate (NHP) model of maternal immune activation (MIA) using a modified form of the viral mimic polyIC (polyICLC) examine the relationship between altered neuorimmune function may contribute to psychosis risk through effects on the developing brain and behavior of NHP offspring. In a previous cohort of MIA-exposed offspring, our group observed evidence for increased pre-synaptic dopamine levels in the striatum using 6-[18F]fluoro-L-m-tyrosine (FMT) positron emission tomography, in addition to pubertal-onset behavioral abnormalities, which may model part of the neurodevelopmental pathway towards psychosis. This study builds on this model and examines the effect of maternal immune activation on in vivo--extracellular free water--a diffusion magnetic resonance imaging measure obtained with a multi-shell acquisition. We sought to test the hypothesis that offspring of pregnant monkeys who received polyICLC injections at the end of the first trimester would show increased extracellular free water compared to control offspring.
**Methods:** Fourteen pregnant rhesus monkeys (Macaca mulatta) receiving polyICLC at the end of the first trimester were compared to 14 controls. The offspring from both groups underwent a multi-shell diffusion MRI scan at 3 Tesla. Diffusion data was collected when the offspring were one month, 6 months, and 12 months of age. Six month preliminary findings are currently presented. Diffusion images were aligned to individual subject MPRAGE scans. Individual subject structural scans were then nonlinearly aligned to generate a common group average template and the group average template was subsequently nonlinearly aligned to a neurodevelopmental rhesus atlas. For this preliminary analysis, the frontal cortex was selected as an a priori region of interest in addition to the more global whole-brain gray and white matter masks.
**Results:** Six month old MIA-exposed rhesus offspring showed a trend for increased whole-brain white matter extracellular free water ($p=.09$) with no significant difference in whole-brain gray matter free water ($p=.27$) compared to control offspring. However, analysis of the frontal ROI revealed significantly increased gray matter free water in the left hemisphere ($p=.013$) with a trend towards increased gray matter free water in the right hemisphere ($p=.081$). There were no significant differences between MIA-exposed and control offspring in basic motor and reflex development or growth trajectories.
**Discussion:** These data suggest that despite the lack of behavioral abnormalities at this early age, extracellular free water values are increased in MIA-exposed offspring, particularly in frontal gray matter. More global whole-brain free water group differences did not reach statistical significance, which may indicate some regional specificity to these changes early in development. The NHP MIA model complements the human schizophrenia literature in which extracellular free water increases have been repeatedly identified. Ultimately, these data provide validation of the clinical relevance of the NHP MIA model and improve our understanding of neuroimmune mechanisms in the development of psychiatric disorders, particularly schizophrenia.

### T36. THE ANTIPSYCHOTIC-LIKE PROPERTIES OF EVENAMIDE (NW-3509) REFLECT THE MODULATION OF GLUTAMATERGIC DYSREGULATION

Marco Bortolato*,[1], Laura Faravelli[2], Ravi Anand[3]
*[1]University of Kansas; [2]Newron Pharmaceuticals S.p.A.; [3]Anand Pharma Consulting*

**Background:** The lack of adequate benefit with current 5HT2 / D2 antipsychotics in large proportions of schizophrenic patients suggests it is essential to modulate other mechanisms for improving symptoms of schizophrenia (SCZ). Increasing evidence implicates NMDAr hypofunction, and hippocampal hyperactivity, in the dysregulation not only of mesolimbic DA neurons but also of Glu neurons, leading to increasing synaptic activity of Glu in the PFC. Injection of NMDAr antagonists (PCP, ketamine) at doses that produce psychotomimetic effects lead to a downstream increase of Glu neurotransmission at non-NMDAr. The excessive firing and the hyper-glutamatergic tone represent alternative targets of treatment for SCZ ultimately affecting positive, negative, cognitive symptoms. The addition of Glu release inhibitors may augment the benefits of the antipsychotics in patients showing inadequate response.

Evenamide uniquely does not interact with monoaminergic (DA, 5-HT, NA, H) pathways affected by current antipsychotics, or with more than 130 different targets that are involved in CNS activity, except sodium channels. Preclinical data suggests that by the modulation of the firing abnormalities, evenamide normalizes the aberrant spread of Glu excitatory transmission that occurs in the brains of patients with SCZ. Evenamide showed efficacy in animal models relevant to SCZ (sensory motor gating, mania, psychosis, depression, impulse control, cognition, social interaction), in monotherapy and as an add on to first or second generation antipsychotics irrespective of whether impairment was either spontaneous, induced by amphetamine or NMDAr antagonists or stress. Evenamide, has also shown significant benefit in a p.c phase 2 trial as an add-on to risperidone and aripiprazole in patients worsening on dopaminergic/serotoninergic antagonist medication, suggesting it acts through other mechanisms. New animal data further confirm evenamide's activity in reducing SCZ symptoms provoked by Glu alteration.

**Methods:** Effects of evenamide (EVE 1.25, 5, 15 mg/kg PO) to restore the impaired information processing (a deficit observed in SCZ), were evaluated in the rat model of the Pre-Pulse Inhibition (PPI) deficit induced by injection of the NMDAr antagonist ketamine (KET 6 mg/kg, SC). Clozapine (CLO 7.5 mg/kg, IP) was used as a positive control.

**Results:** PPI analysis showed significant main effects for KET to lower PPI levels [$F(1,264)=139.67$, $P<0.0001$], for EVE [$F(3,264)=3.14$, $P<0.05$] and CLO to enhance PPI levels [$F(1,98)=30.89$, $P<0.001$]. Notably, significant EVE x KET [$F(3,264)=2.79$, $P<0.05$] and CLO x KET interactions [$F(1,98)=5.45$, $P<0.05$] were found.

Post-hoc analyses (Tukey's) revealed that KET significantly lowered PPI ($P<0.0001$) for each group; both EVE (5 mg/kg) and CLO significantly increased PPI in KET-treated rats ($P=0.02$; $p<0.001$).

**Discussion:** Evenamide as monotherapy has similar effect to clozapine in reversing ketamine- induced worsening of PPI. Together with previously demonstrated effects to reverse PCP-induced PPI and social interaction deficits, this further supports its potential to affect both positive and negative symptoms of SCZ by targeting altered Glu transmission.

Efficacy of evenamide as an add-on to antipsychotics would revolutionize development of novel antipsychotics that would target aberrant firing and Glu transmission in SCZ. Two clinical trials have been planned to support the hypothesis that the addition of evenamide should add a non-dopaminergic mechanism for augmenting antipsychotic efficacy in patients who are not responding adequately to current antipsychotic, and in patients with treatment resistant SCZ who are not responding/worsening on clozapine.

### T37. THE LONELY MOUSE: A MODEL FOR STUDYING MATERNAL PSYCHOLOGICAL STRESS AND ITS CONSEQUENCES IN THE OFFSPRING

Joseph Scarborough[1], Flavia Müller[1], Annamaria Cattaneo[2], Juliet Richetto*,[1]
*[1]Institute for Veterinary Pharmacology and Toxicology, University of Zurich; [2]IRCCS Fatebenefratelli San Giovanni di Dio*

**Background:** It is well established that antenatal psychopathology affects obstetric outcomes and maternal behavior, and that it has long-term consequences on the offspring's wellbeing and mental health, which are relevant for multiple psychiatric disorders. Against this background, it is of pivotal importance to investigate the precise mechanisms that underlie such association, and evaluate the potential beneficial and/or detrimental impact of pharmacological treatment during pregnancy and the postpartum period. To date, rodent models rely mainly on exposure to chronic or acute unpredictable stress during pregnancy, which is mainly based on physical stressors characterized by medium translational value. Therefore, we propose the use of a social isolation-rearing paradigm to investigate the effects of antenatal maternal stress on the offspring. This model has the advantage of implementing psychological stressors, as opposed to physical stressors, to induce depressive-like behaviours in female mice. Moreover, the depressive-like state can be induced and assessed before pregnancy, thus eliminating possible confounding factors that arise from physical stressful manipulations applied during pregnancy.

**Methods:** C57BL/6 female mice were socially isolated, or group housed, from weaning (PND21) to adulthood (PND91). After 5 weeks of social isolation, the animals were tested to confirm the development of a depressive-like phenotype. At PND91, both group housed and socially isolated mice were bred and left undisturbed during pregnancy. The offspring were subjected to cognitive and behavioral testing in adulthood. A subgroup of socially isolated and grouped females were treated with the antidepressant Fluoxetine (10mg/kg) for the last 3 weeks of social isolation, pregnancy and weaning, and the offspring were once again subjected to cognitive and behavioral testing in adulthood.

**Results:** Social isolation rearing induced weight gain, basal plasma corticosterone reduction and depressive-like behavioural traits, such as reduced social interaction and increased anxiety. Both female and male offspring of socially isolated mothers displayed a variety of behavioural abnormalities relevant to different psychiatric disorders, such as increased anxiety and altered fear expression. Male offspring also presented metabolic alterations and cognitive deficits in the form of spatial working memory and recognition memory. Prenatal fluoxetine was effective in rescuing some of the above-mentioned behavioural abnormalities but detrimental for others.

**Discussion:** Our results demonstrate, for the first time, that long-lasting psychological stress preceding pregnancy is sufficient for inducing long-term behavioural and metabolic alterations in the offspring. Specifically, social isolation can be considered a strong etiological factor for stress in rodents, just as loneliness is a significant precursor to depression in humans. The social isolation-rearing model could thus offer a translationally-relevant setting in which to further investigate the mechanisms underlying the association between prenatal stress and psychopathology in the offspring, and the contribution of pharmacological treatments.

### T38. SUBMISSION WITHDRAWN

### T39. NEURAL MECHANISMS OF METABOTROPIC GLUTAMATE RECEPTOR 3 MEDIATED ENHANCEMENT OF SYNAPTIC PLASTICITY AND COGNITION

Branden Stansley*,[1], Max Joffe[1], Samantha Yohn[1], Craig Lindsley[1], Colleen Niswender[1], P. Jeffrey Conn[2]
*[1]Vanderbilt University; [2]Vanderbilt University Medical Center*

**Background:** The group II metabotropic glutamate receptor 3 (mGlu3) is an emerging therapeutic target for schizophrenia, as research has demonstrated a link between mutations in the human gene encoding for mGlu3, GRM3, and clinical diagnosis of schizophrenia. Schizophrenia is known to be accompanied by debilitating cognitive impairments that negatively impact the overall quality of life of the patient. While current pharmacological therapeutics mainly target the positive symptoms, cognitive symptoms are often not effectively treated. Our recent discovery that mGlu3 and mGlu5 can act as signaling partners to modulate synaptic plasticity in the prefrontal cortex led us to hypothesize that mGlu3 may subserve similar functions to those of mGlu5 during hippocampal synaptic plasticity and hippocampal-dependent behaviors.
**Methods:** We directly tested this hypothesis using acute slice electrophysiology to investigate basal synaptic transmission as well as long-term plasticity in hippocampal slices. To test cognition, the associative fear learning behavioral assay, termed trace-fear conditioning, was used. C57bl/6 mice or CaMKII-cre;mGlu5-/- mice were used in all studies.
**Results:** We report that mGlu2/3 activation enhances hippocampal theta-burst (TBS)-induced LTP but was without effect on group I mGlu agonist-induced LTD The group II mGlu agonist enhancement of TBS-LTP was blocked by antagonists of mGlu3 or mGlu5.
We next tested downstream mechanisms of group II mGlu induced LTP by chemically activating LTP with the group II agonist LY379268 in combination with selective antagonists. We verified the LTP was induced by mGlu3 activation but not mGlu2 using selective negative allosteric modulators of each subtype. Furthermore, mGlu5 negative allosteric modulation with MTEP blocked mGlu3-LTP, and conversely the mGlu5 positive allosteric modulator, VU0092273, enhanced mGlu3-LTP. The cannabinoid receptor type 1 antagonist AM251 was also capable of blocking mGlu3-LTP, suggesting cannabinoid signaling mechanistically drives this LTP.
Having confirmed a role for mGlu5 in the mGlu3-LTP, we next verified that postsynaptic mGlu5 located on pyramidal neurons was necessary for mGlu3-LTP by utilizing CaMKII-cre;mGlu5-/- mice. It was found that hippocampal slices from these mice showed no enhancement of LTP when LY379268 was bath applied alone or in combination with TBS-stimulation. Behaviorally, we discovered that selective activation of mGlu3 by systemically injecting the group II mGlu agonist in combination with a selective mGlu2 negative allosteric modulator, VU6001966, causes an enhancement in the acquisition of trace-fear conditioning learning. This was also confirmed to be dependent on mGlu5 as both systemic pharmacological inhibition or genetic deletion of mGlu5 abolished this learning enhancement. Further testing of the ability of mGlu3 activation to augment other cognitive tasks is currently underway.
**Discussion:** These results taken together demonstrate mGlu3 enhances hippocampal LTP and hippocampal-dependent learning through mechanisms that involve both mGlu5 and CB1 receptor activation. This work provides a basic biological mechanism and preclinical therapeutic validation for mGlu3 as a target for neurological disorders in which cognition is disrupted such as schizophrenia.

### T40. GPR52 AGONISTS REPRESENT A NOVEL APPROACH TO TREAT UNMET MEDICAL NEED IN SCHIZOPHRENIA

Andrew J. Grottick[1], Ben Grayson[2], Giovanni Podda[2], Nagi Idris[2], Cornelia Dorner-Ciossek*[,3], Joanna Neill[2], Scott Hobson[3]
*[1]Beacon Discovery, Inc.; [2]University of Manchester; [3]Boehringer Ingelheim Pharma*

**Background:** There are currently no treatment options for key symptom domains in certain psychiatric and neurological diseases. For example, antipsychotics effectively treat the positive symptoms of schizophrenia, however both the cognitive impairments associated with schizophrenia (CIAS) and negative symptoms, both key predictors of functional outcome, are not treated by current therapies. Additionally, psychotic symptoms associated with neurological diseases such as Alzheimer's Disease (AD) are not adequately treated with current antipsychotics. Therefore, novel mechanisms to address these unmet medical needs are urgently required and are under investigation.
GPR52 is a Gs-coupled orphan g-protein coupled receptor which has an intriguing pattern of brain expression. In cortex, GPR52 is expressed primarily on glutamatergic neurons and co-localized with the Gs-coupled D1 receptor (D1R). Deficiencies in D1R activation are associated with both cognitive deficit and negative symptoms of schizophrenia. In contrast, in the striatum, GPR52 is almost exclusively co-expressed with the Gi-coupled D2 receptor (D2R), which mediates the reduction in positive symptoms by antipsychotics. Based on GPR52's functional coupling and co-localization, agonists may be predicted to resemble D1R agonists in cortical regions, thus treating cognitive or negative symptoms, while resembling D2R antagonists in striatal regions. Thus, GPR52 agonists have the potential to provide a novel therapeutic strategy for the currently untreated CIAS and negative symptom domains in addition to the psychotic symptoms of AD.
**Methods:** To assess the antipsychotic potential of GPR52 agonists, they were tested for their ability to decrease psychostimulant-induced hyperlocomotion. The efficacy of GPR52 agonists for CIAS and sociability, an aspect of negative symptoms, was assessed in the sub-chronic phencyclidine (scPCP) model for schizophrenia, known to induce long-lasting cognitive and social behaviour deficits, in addition to a reduction in parvalbumin-positive GABAergic interneurons in hippocampus and pre-frontal cortex. Rats were treated with PCP twice daily for 7 days followed by 7 days washout and then tested in the attentional set shifting task (ASST) for executive function and the social interaction test for sociability respectively following treatment with a GPR52 agonist.
**Results:** GPR52 agonist 1 dose-dependently reversed psychostimulant-induced hyperlocomotion in rats at doses which were behaviorally quiescent when administered alone. Additionally, GPR52 agonist 2 showed a robust, dose-dependent rescue of scPCP induced deficits in the extra dimensional shift phase of the ASST, achieving significance after a 4 mg/kg p.o. application. Likewise, GPR52 agonist 2 significantly rescued scPCP induced deficits in social interaction at identical doses as in ASST without effects on object exploration or locomotor activity.
**Discussion:** GPR52 agonists were efficacious in animal models assessing the three main symptoms domains associated with schizophrenia. Efficacy in ASST and SI demonstrate both pro-cognitive efficacy and restoration of an aspect of negative symptoms, respectively, in a well-established model inducing behavioral and neuropathological deficits associated with schizophrenia. Furthermore, GPR52 agonists reduced psychostimulant-induced hyperlocomotion, an effect associated with antipsychotic efficacy. Taken together, these data demonstrate the potential of this innovative mechanism to simultaneously treat the three core symptoms domains of schizophrenia as well as potentially treat the psychotic symptoms associated with other neurological disorders.

### T41. MODIFIED COGNITIVE BEHAVIORAL THERAPY FOR ELDERLY PATIENTS WITH SCHIZOPHRENIA: A RANDOMIZED, CONTROLLED PILOT TRIAL

Bernhard Müller*[,1], Christian Kärgel[2], Magdalena Horacek[1], Ute Darrelmann[3], Anja Heger[3], Daniela Kariofillis[4], Beate Nentwich[5], Christoph Meisner[5], Claudia Ose[1], Svenja Unsöld[5], Jens Wiltfang[6], Stefan Klingberg[5], Gudrun Sartory[4]
*[1]University of Duisburg-Essen; [2]LWL-University Hospital Bochum; [3]University of Bonn [4]University of Wuppertal; [5]University of Tübingen; [6]University of Göttingen*

**Background:** An European society growing older sets the need to explore treatment options for elderly patients. Here we aimed to gather evidence on the effectiveness of a modified cognitive behavioural therapy (mCBT) in elderly schizophrenia patients as compared to treatment as usual (TAU).
**Methods:** 43 schizophrenia patients > 55y (mean 60 y), were assessed in a randomized, single blind controlled pilot trial with parallel groups: TAU+mCBT vs. TAU and intention to treat (ITT) last observation carried forward (LOCF) analysis. Subjects were recruited in Germany among in- and outpatients. MCBT comprised 30 sessions in 9 month, and a 6 month follow-up including a) physical and social activation, b) problem solving c) social skills. Primary outcomes were pre/post change in either PANSS total score, UPSA-brief score or Calgary Depression Scale for Schizophrenia.

**Results:** From 43 patients, 40 were randomized. 15 mCBT and 16 TAU-patients comprised the ITT sample. None of the primary outcome measures reached significance. When assessing effect sizes we found little pre-/post change in PANSS total score (d=0.14) and the UPSA-brief (d=0.14). Depression symptoms however improved with treatment (pre/post d=0.75 and pre/FU d=0.52). Among secondary outcomes, global assessment of functioning significantly improved in the mCBT-group pre/post (d=.074) and pre/follow-up (d=.080).

**Discussion:** Our results provide evidence for the feasibility of a sufficiently powered phase-III study targeting depressive symptoms and global functioning in long-term treated elderly schizophrenia patients. A history of about twenty years of pharmacological treatment does not imply there is no room for improvement in depression and global functioning following a modified cognitive behavioral therapy in this specific patient group.
Supported by the German Federal Ministry of Education and Research (BMBF-01GV0909); ICTRP/DRKS: DRKS00003623.

### T42. WHEN SHOULD EARLY INTERVENTION START, AND FOR HOW LONG SHOULD IT LAST?

Nikolai Albert*[,1], Marianne Melau[1], Heidi Jensen[2], Lene Halling Hastrup[3], Carsten Hjorthøj[1], Merete Nordentoft[1]
*[1]Mental Health Center Copenhagen; [3]Region Hovedstadens Psykiatri; [3]Psychiatric Research Unit, Region Zealand*

**Background:** Early intervention in psychosis facilities have often failed to integrate the two main elements of early intervention. While some facilities have focused on early, and have had Duration of Untreated Psychosis (DUP) as their main target, others have focused on the intervention and the treatment provided when patients were diagnosed. As both DUP reduction and specialized early intervention (SEI) has proved to have an effect on the treatment of patients with first-episode psychosis one could hope of a synergetic effect if the two strategies were integrated. In this study, we use data from a randomized clinical trial testing the effect of prolonged early intervention (5 years) compared to standard specialized early intervention (2 years). Overall the study found that both treatment groups remained stable or improved in psychopathology, functioning and cognition and that there was no further beneficial effect of the prolonged the treatment. Participants had a long DUP (median 52 weeks). For this specific sub-study we hypothesized that patients who were treated early in their course of illness would have a beneficial effect of the prolonged treatment compared to those who only received 2 years of specialized treatment.

**Methods:** 296 participants with a psychotic diagnosis within the schizophrenia spectrum (ICD 10 – F2x, excluding F21) were included. DUP start was assessed from first psychotic symptom equivalent to 3 or above on a global SAPS item. DUP stop was when patients started antipsychotic treatment or specialized early intervention treatment. To assess if there were a delay within the mental health referral system we used the national register to identify when participants first were diagnosed with a schizophrenia spectrum diagnosis and calculated the time until they started SEI treatment. Finally, we added the DUP and the treatment delay together to assess the time from first psychotic symptom until the start of adequate treatment (both antipsychotic medication and specialized early intervention treatment), called total treatment delay. We analyzed if there were a treatment effect for participants with DUP shorter than 3 months (n=79) and if there were an effect for participants with a total treatment delay shorter than 6 months (n=54). We used multiple imputations to correct for missing data at the follow-up. The data were analyzed using binary logistic regression for the dichotomous variables and linear regression for the continuous variables.

**Results:** At the five year follow-up, the participants who had a short DUP and had received 5 years of SEI treatment had lower psychopathological scores and higher level of functioning and cognition than those who only received 2 years of SEI treatment. The difference was not significant. For the patients who had a short total treatment delay there was a clear trend favoring the prolonged treatment and for negative symptoms there was a near significant effect of the prolonged treatment (estimated mean difference -0.61, 95% CI -1.2;0.006, p=0.05).

**Discussion:** Our findings are results of a sub-group analysis and should be interpreted with caution. Even if the results from the main trail did not find a significant effect of prolonged SEI treatment this sub-group analysis indicates that some of the explanation could be the delay prior to the start of treatment and that there could be a beneficial effect of the prolonged treatment if it actually were provided within the early years of illness and not just in the early years after diagnosis.

### T43. TRANSCRANIAL DIRECT-CURRENT STIMULATION (TDCS) IN PATIENTS WITH ULTRA-TREATMENT-REFRACTORY AUDITORY HALLUCINATIONS

Jean-Pierre Lindenmayer*[,1], Tania Sultana[2], Amandeep Kaur[2], Rang Yang[2], Anzalee Khan[2], Mila Kirstie-Kulsa[3], Isidora Ljuri[2], Benedicto Parker[2]
*[1]New York University; [2]Manhattan Psychiatric Center; [3]Columbia University*

**Background:** Transcranial direct-current stimulation (tDCS), a noninvasive neurostimulation treatment, has been reported to show improvements in treatment-resistant auditory hallucinations in patients with schizophrenia. tDCS administered over a limited number of sessions effectively produced lasting attenuation of auditory hallucinations in otherwise stable outpatients. It has also been shown that tDCS may be a useful intervention for ameliorating cognitive deficits in patients with chronic schizophrenia. The purpose of this study was to test tDCS for auditory hallucinations in ultra-treatment resistant schizophrenia to assess if this form of neurostimulation can alleviate treatment-refractory auditory hallucination symptoms up to 4 weeks after the final treatment. In addition, we also wanted to examine the effects of tDCS on cognitive functions.

**Methods:** 28 inpatients with DSM-V schizophrenia and long-standing treatment resistance and persistent auditory verbal hallucinations were recruited. Each individual participated in behavioral assessments at baseline, endpoint and follow-up [PANSS and Auditory Hallucinations Rating Scale (AHRS) and MCCB cognitive battery] and were randomized to receive active vs. sham tDCS treatments. For active treatment, patients had the inhibitory (cathodal) tDCS electrode placed over left auditory cortex relative to an excitatory (anodal) electrode placed over frontal cortex on the right side. tDCS treatments took place for 20 min twice daily for 5 consecutive days. Assessment batteries were repeated following the 4 weeks of treatment. The Chattanooga, dual channel CHA-1335 stimulator with two 7 × 5 cm (35 cm2) sponge electrodes soaked in a saline solution (0.9% NaCl) was used for the delivery of 2 mA current.

**Results:** A total of 28 subjects were enrolled (tDCS, n = 13; Control, n = 15). 20 subjects completed the trial. 3 subjects dropped out of the active tDCS treatment group, while 4 subjects did not complete the control treatment due to early discharge from the hospital. Most subjects were male (tDCS n = 10, 76.9%; Control n = 6, 40.0%). Length of present psychiatric admission ranged from 1–25 months, with a mode of 2 months (n = 12) and average of 2.9 months. Participating inpatients were on clozapine, haloperidol, paliperidone depot, fluphenazine decanoate, paliperidone, olanzapine, and risperidone as primary medications. Repeated Measures ANOVA showed a significant difference for the auditory hallucination total score, frequency and number of voices over time ($p < 0.05$) with greater reduction in scores observed for the tDCS group. Improvements were maintained after 4 weeks.

There was no significant change over time observed for the PANSS positive symptoms or total score, or for the PANSS Hallucinatory Behavior item score. When assessing cognitive functioning, only Working Memory change was significant (p = 0.048) between the tDCS and the Control group with the tDCS group showing significant improvement in T-Score as compared to the Control group.

**Discussion:** Subjects who received tDCS treatment showed a significant reduction in the frequency, number of voices, and total scores of their auditory hallucination. Additionally, subjects in the tDCS group showed significant improvement in the Working Memory. Our results indicate that patients who have been ultra-resistant to antipsychotic treatments and who received tDCS treatment presented with robust diminution of their auditory hallucinations. We conclude that tDCS seems to be effective not only for ambulatory, higher functioning patients, but also for much lower functioning patients with medication-refractory auditory verbal hallucinations.

### T44. A RANDOMISED, DOUBLE-BLIND, PLACEBO-CONTROLLED TRIAL OF THE EFFECTS OF VITAMIN B12, B6 AND FOLIC ACID ON COGNITION AND SYMPTOMS IN FIRST-EPISODE PSYCHOSIS: THE VITAMINS IN PSYCHOSIS STUDY

Kelly Allott*[,1], Patrick McGorry[1], Hok Pan Yuen[1], Colin O'Donnell[2]
*[1]Orygen, The National Centre of Excellence in Youth Mental Health; [2]Donegal Mental Health Service*

**Background:** Vitamin B12, vitamin B6 and folic acid are homocysteine-reducing agents. People with schizophrenia have been found to have increased homocysteine levels. Elevated homocysteine has been associated with impaired cognition. Previous research in chronic schizophrenia has shown that supplementation with folate plus vitamin B12 can improve cognition and clinical symptoms. Whether homocysteine lowering agents are effective in first-episode psychosis is unknown. The aim of this study was to investigate if adjunctive vitamin B12, B6 and folic acid can lower homocysteine and improve symptomatology and cognition in people with first-episode psychosis.

**Methods:** This was a randomised, double-blind, placebo-controlled trial conducted at the Early Psychosis Prevention and Intervention Centre (EPPIC) in Melbourne, Australia. One hundred and twenty patients aged 15–26 years with first-episode psychosis consented and were randomised to receive folic acid 5mg, vitamin B12 0.4mg, and vitamin B6 50mg or placebo, each taken once-daily for 12 weeks as an adjunct to antipsychotic medication. Co-primary measures were change in cognition as measured by a composite score from a battery of 11 tests and total symptomatology (PANSS) over 12 weeks. Secondary outcomes included additional cognitive, symptom, functioning, tolerability and safety measures.

**Results:** Of the 120 participants randomised in the study, 20 dropped out with no follow-up assessments and were excluded from analysis. Of the remaining 100 participants, 52 were in the vitamins group and 48 the placebo group. At baseline, the two treatment groups had lower levels of folate and vitamin B12 intake than healthy controls, but did not differ from each other. Vitamin B12, B6 and folic acid reduced homocysteine levels in the vitamin group over 12 weeks. The homocysteine lowering effects of the vitamins did not confer a major advantage over placebo therapy in improving the co-primary PANSS (p=.75) or composite cognition (p=0.79) outcomes over 12 weeks. There was a significant difference between groups among females in the cognitive domain of speed of processing (p=.049) and attention/vigilance (p=0.002), in which the mean performance of the placebo group declined over 12 weeks, whereas performance in the vitamin group remained showed improvement.

**Discussion:** Folic acid, B12 and B6 supplementation appears well tolerated and safe in first-episode psychosis and lowers homocysteine levels in this population. However, supplementation may not offer extra benefits to all patients with first-episode psychosis, with the possible exception of speed of processing and attention/vigilance. Although previous research suggests that males preferentially benefit, our findings suggest that there may be a specific beneficial effect on cognition for females with first-episode psychosis.

### T45. A COMPARISON OF SCHIZOPHRENIA RELAPSE RATES OF 3 PALIPERIDONE FORMULATIONS, ONCE-DAILY, ONCE-MONTHLY AND ONCE EVERY-3-MONTH: POST-HOC ANALYSIS FROM 3 RANDOMIZED CONTROLLED TRIALS

Maju Mathews[1], Srihari Gopal*[,1], Arun Singh[1], Isaac Nuamah[1], Anne Marie Quinn[1], Katalin Pungor[2], Wilson Tan[1], Bernardo Soares[2], Edward Kim[1]
*[1]Janssen Inc.; [2]Janssen-Cilag*

**Background:** Poor adherence to antipsychotic treatment in patients with schizophrenia can result in recurrent relapses, worsening disease, functional impairment and reduction in treatment responsiveness. Long-acting antipsychotic formulations can maintain therapeutic plasma levels for longer durations, reducing dosing frequency and delaying time to relapse compared to oral formulations. Consequently, relatively lower rates of relapse can be expected in patients on long-acting injectables (LAIs) who have discontinued treatment versus those discontinuing oral medications of the same antipsychotic. However, there is no available evidence to support this association. In this post hoc analysis, the percentage of patients who relapsed and the time to relapse for three different formulations of the same molecule (oral paliperidone extended release [ER]; paliperidone palmitate once monthly [PP1M] LAI, and paliperidone palmitate three monthly [PP3M] LAI) were evaluated in adults with schizophrenia, comparing the active and placebo arms.

**Methods:** Data from three similarly designed, randomized, double-blind, placebo-controlled relapse prevention studies in adult patients with schizophrenia (DSM-IV-TR criteria) with similar inclusion/exclusion and relapse criteria were analyzed. Patients stabilized during an open-label stabilization phase with either paliperidone ER, PP1M or PP3M were then randomized to receive either placebo (analogous to non-adherent patients in the real-world) or the same active treatment used during stabilization phase (analogous to adherent patients). The primary outcome in each study was the time to relapse after entering the randomization phase, estimated using Kaplan-Meier method. In this report, the percentage of patients who relapsed as well as time to relapse in the three studies were indirectly compared.

**Results:** In total 922 patients were included in this analysis, 473 continued to receive the same active treatment and 449 patients were randomized to receive placebo. The percentage of patients who relapsed was lowest with PP3M as compared with PP1M and paliperidone ER in both the active treatment group (PP3M, 9% < PP1M, 18% < paliperidone ER, 22%) and placebo group (PP3M, 29% < PP1M, 48% < paliperidone ER, 52%) patients. The post discontinuation median time to relapse (95% confidence interval) in placebo group was highest with PP3M, 395 days (274 days to not reached) > PP1M, 172 days (134 to 222 days) > paliperidone ER, 58 days (42 to 114 days) but was not estimable in the paliperidone group.

**Discussion:** Treatment with longer acting formulations of paliperidone are associated with lower percentage of relapse and longer time to relapse in patients with schizophrenia. Lower percentage of patients with relapse observed with LAI therapy (PP1M and PP3M) could presumably be due to ensured therapeutic plasma levels. The lower percentage of relapse observed with PP3M treatment as compared with PP1M and oral paliperidone ER treatment in the placebo group could be advantageous to non-adherent patients, as this mimics the real-world scenario where patients discontinue their antipsychotics suddenly. These findings are of relevance in schizophrenia patients as fewer and delayed relapses over the course of a lifetime of schizophrenia may provide higher protection against grey matter damage and help preserve functioning.

### T46. TARGETING THE IMMUNE SYSTEM TO TREAT DEPRESSION AND NEGATIVE SYMPTOMS IN SCHIZOPHRENIA

Carl Krynicki*[,1], Rachel Upthegrove[1], John Suckling[2], Paola Dazzan[3], Eileen Joyce[4], Stephen Lawrie[5], Nusrat Husain[6], Imran Chaudhry[6], Graham Dunn[6], Peter Jones[2], Danuta Lisiecka[9], Shon Lewis[6], Thomas Barnes[7], Stephen Williams[6], Stephen Hopkins[6], Richard Drake[6], Richard Smallman[6], Annalisa Giordano[3], Carmine Pariante[3], Bill Deakin[6]
*[1]University of Birmingham; [2]University of Cambridge; [3]Institute of Psychiatry, Psychology and Neuroscience, King's College London; [4]UCL Institute of Neurology; [5]University of Edinburgh; [6]University of Manchester; [7]Imperial College London*

**Background:** Negative symptoms consist of impaired quality of life, social isolation, reduced emotional responsiveness, self-neglect and anhedonia, which have been categorised into avolition-apathy and expressive deficits sub-domains. The treatment of negative symptoms remains a challenge. Depression is commonly seen in schizophrenia and previous findings have suggested a relationship between depression and negative symptoms via the avolition-apathy sub-domain, (Barnes et al., 2016). It is possible this is the result of a common aetiology, distinct from expressive deficit or other symptoms of schizophrenia. Immune dysfunction has been implicated in both psychotic and depressive illnesses; increased circulating pro-inflammatory markers (such as IL-6, TNF-α & CRP). This suggests a novel target for treatments. A putative neuroprotective role of minocycline has been suggested via reducing microglial activation, and decrease in the production of cytokines including IL-6. Minocycline has been shown to be effective in the treatment of negative symptoms (Xiang et al., 2017) and depression (Soczynska et al., 2012). Within schizophrenia, we predict that that minocycline will lead to a longitudinal improvement in depression and the avolition-apathy sub-domain of negative symptoms
**Methods:** Data from the BeneMin study will be presented. BeneMin recruited 207 patients with a current research diagnosis of schizophrenia within 5 years of onset and randomised to minocycline (300mg/day) or matching placebo for 12 months adjunctive to antipsychotic medication. For this analysis the primary outcome variable is the negative symptom subscale from the Positive and Negative Syndrome Scales (and broken down into avolition-apathy and expressive deficits sub-domains), Calgary Depression Scale in Schizophrenia (CDSS) and circulating IL-6, TNF-α and CRP over 4-time points 2, 6, 9, and 12 months.
**Results:** At baseline, 40% were depressed (mean CDSS score = 5). The mean avolition-apathy PANSS score was 9.5 and expressive deficits was 9, and was comparable across placebo and minocycline arms. Preliminary results show that markers of inflammation were low in both treatment arms, compared with previous research (baseline CRP Md = 1.45, IL-6 Md = .57, TNF-α Md = 2.43) and this was comparable across depressed and non-depressed patients. TNF-α was significantly associated with expressive-deficits (B = .75, p = .005). Conversely, no marker of inflammation was associated with avolition-apathy or depression. However, in four linear mixed effect models across the 2, 6, 9 and 12-month follow-up assessments compared with placebo, minocycline had no effect on total negative symptoms, avolition-apathy, expressive deficits or depression. Further analysis stratifying patients by depression scores and markers of inflammation will be presented.
**Discussion:** Preliminary results indicate that minocycline does not lead to a reduction in avolition-apathy or depression in early schizophrenia. This may be the result of a medicated, sample recruited within 5 years of illness onset, and low levels of depression. Future studies should target depression in psychosis as a primary aim with samples of individuals with increased inflammatory response to fully investigate minocycline's potential in targeted intervention.

### T47. IS THERE A DIURNAL VARIATION IN PSYCHIATRIC SYMPTOMS IN SCHIZOPHRENIA?

David Daniel*[,1], Alan Kott[1]
*[1]Bracket Global, LLC*

**Background:** Anecdotally we have observed subgroups of schizophrenic patients who experienced a diurnal variation in a wide range of psychiatric symptomology. Such a pattern could have substantial ramifications in both clinical care and clinical trials. For example, two commonly used measures of symptom severity in clinical trials, the Positive and Negative Symptom Scale (PANSS) and the Negative Symptom Assessment Scale (NSA-16) contain numerous items that are rated based entirely or in part on observations of the subject during the interview. We hypothesized that inconsistency in the time of day of assessment in subjects whose symptoms were influenced by circadian rhythms could introduce an erratic "noise" element in the longitudinal measure of their symptoms.
To investigate this hypothesis we compared the change in PANSS total score and individual PANSS items across consecutive visits by whether the assessments had been conducted at consistent vs. inconsistent times of day.
**Methods:** 2109 individual subject visits from multiple schizophrenia clinical trials for which PANSS interview start time was available were included in the analysis. 1,764 pairs of consecutive PANSS interviews within individual subjects were identified and the time difference between the start times of the interviews were calculated. The absolute time difference was divided into quartiles and the first quartile (assessments least disparate in time) and the fourth quartile (assessments most disparate in time) were compared in the analyses. The mean absolute change in PANSS total and PANSS individual items between the consecutive visits was compared between the 2 groups using a t-test. Given the exploratory and hypothesis driven nature of the analysis we did not correct for multiple comparisons.
**Results:** The average absolute difference in interview start times was 79.5 minutes. The group with assessments closest in time (N = 446) had their interviews start no later than 30 minutes apart while the group with assessments most disparate in time (N = 445) had their interview start with at least 170 minutes difference. Of the 30 PANSS items, items P4 (Excitement 0.33 vs. 0.44, p =0.0077), P7 (Hostility 0.31 vs. 0.47, p < 0.001), N5 (Difficulty in abstract thinking 0.28 vs. 0.35, p = 0.043), N7 (Stereotyped thinking0.26 vs. 0.35, p = 0.021) G1 (Somatic concern 0.34. vs. 0.43, p = 0.041), G2 (Anxiety 0.42 vs. 0.53, p = 0.019), G6 (Depression 0.38 vs.0.52, p = 0.0044), G7 (Motor retardation 0.28 vs. 0.37, p = 0.025), and G8 (Uncooperativeness 0.29 vs. 0.39, p = 0.02) had an absolute difference significantly higher in the disparate group than in the close group. The mean absolute difference in the PANSS total was 5.1(+/-5.8) in the close group and 5.9(+/-5.4) in the disparate group, (p = 0.08).
**Discussion:** The results demonstrate a statistically significant effect of variation in time of day on symptom severity. These findings underscore the risk of potential noise (erratic changes) in longitudinal assessment of symptom severity when ratings are done at different times of day. Moreover, it suggests that symptom severity assessed in a standard PANSS interview may not generalize over the entire day, much less over the standard one week rating period. This is important because many PANSS items are rated partially or entirely on the interview which may not be a representative "biopsy" of the subject's mental state or behavior during the one week rating period. This highlights the potential remedies of more frequent or ecological measurements.
Hufford et al (2014) demonstrated a statistically significant effect of variation in the time of day on signal from the MCCB cognitive battery. Our results confirm our anecdotal observations that severity of non-cognitive schizophrenic symptoms are impacted by circadian rhythms as well.

### T48. ANTIPSYCHOTIC EFFICACY OF EVENAMIDE (NW-3509) IS DUE TO MODULATION OF GLUTAMATERGIC DYSREGULATION

Ravi Anand*[,1], Emma C. Forrest[2], Richard D. Hartman[3], Stephen M Graham[2], Laura Faravelli[2]
*[1]Anand Pharma Consulting; [2]Newron Pharmaceuticals; [3]NeurWrite LLC*

**Background:** Over 70% of schizophrenic patients discontinue treatment with first (F)- or second-generation antipsychotics (SGA) due to dissatisfaction with their therapeutic effects; median time to discontinuation ranges from 3–7 months (1). Switching to another antipsychotic, except clozapine, did not yield better results (2). These results indicate it is essential to modulate mechanisms other than dopaminergic (DA)/serotoninergic (5-HT) systems to improve symptoms of schizophrenia (SCZ). Increasingly, NMDA receptor (NMDAr) hypofunction (3) and hippocampal hyperactivity (4) are implicated in the dysregulation of mesolimbic DA and glutamate (Glu) neurons, leading to increasing synaptic activity of Glu in the PFC (5). Augmenting the effects of current antipsychotics with Glu release inhibitors may improve symptoms of psychosis in patients with SCZ.
Evenamide does not interact with monoaminergic (DA, 5-HT, NA, H) pathways affected by current antipsychotics, or with >130 different targets involved in CNS activity, except for sodium channels, leading to modulation of Glu release. Evenamide shows efficacy in animal models of SCZ as monotherapy and as an add-on to FGA or SGA, irrespective of whether impairment was spontaneous, or induced by amphetamine, NMDAr antagonists or stress.
**Methods:** In a pilot, proof of mechanism, randomized, double-blind, placebo-controlled, parallel group, 4-week trial, evenamide (n=50; 15–25 mg bid) or placebo (n=39) was added to patients with SCZ worsening on their current antipsychotic doses of risperidone (RIS; ≥2 mg/day) or aripiprazole (ARI; ≥10 mg/day), in 2 sites in the US (n=61) and 3 in India (n=28).
**Results:** 89 patients with SCZ (mean baseline PANSS total: 62.9 ± 7.4; CGI-S: 3.5 ± 0.5), experiencing break-through psychotic symptoms on previously effective and stable doses of RIS (mean dose: 4.2 ± 2.0 mg/day; n=70) or ARI (mean dose: 19.7 ± 7.0 mg/day; n=19) were randomized (1.3:1 ratio) to treatment with evenamide or placebo. Analyses demonstrated the addition of evenamide to RIS or ARI was associated with statistically significant efficacy, based on the PANSS Positive Symptoms sub-scale (mean change, responders), and CGI-C responder rates. The study treatments were very well tolerated; 2 patients on evenamide discontinued treatment due to AEs (atrial fibrillation and seizure). The most common AEs (evenamide vs placebo [%]), were somnolence (16 vs 12.8%), insomnia (10 vs 6%) and headache (6 vs 0%).
**Discussion:** Addition of evenamide in patients worsening on SGAs modulating DA/5-HT significantly improved positive symptoms and CGI. No AEs such as EPS, endocrine, or sexual side effects, or weight gain were noted. These data indicate that evenamide's Glu antagonism, demonstrated in preclinical experiments, is of value in patients worsening on current antipsychotics. Evenamide, as monotherapy or add-on, has reversed ketamine- and PCP-induced worsening of PPI. The results in the pilot clinical trial demonstrated an absence of side effects common with DA/5-HT blockers, and a rapid onset of action mediated by evenamide targeting altered Glu transmission in patients in whom SGA treatment had lost its efficacy.
Efficacy of evenamide as add-on to antipsychotics would revolutionize development of novel antipsychotics targeting aberrant firing and Glu transmission in SCZ. Potentially pivotal studies with evenamide are in planning to demonstrate that the addition of evenamide, a Glu release inhibitor, augments antipsychotic efficacy in patients worsening on current antipsychotics, and in patients with treatment-resistant SCZ not responding/worsening on clozapine.

### T49. THE NEURAPRO STUDY: ADHERENCE TO STUDY MEDICATION

Monika Schlögelhofer*[,1], Patrick D. McGorry[2], Barnaby Nelson[2], Connie Markulev[2], Hok Pan Yuen[2], Miriam Schäfer[3], Nilufar Mossaheb[1], Stefan Smesny[4], Ian B. Hickie[5], Gregor Berger[6], Eric Y. H. Chen[7], Lieuwe de Haan[8], Dorien H. Nieman[8], Merete Nordentoft[9], Anita Riecher-Rössler[10], Swapna Verma[11], Andrew Thompson[2], Alison Ruth Yung[12], G. Paul Amminger[13]
*[1]Medical University of Vienna; [2]Orygen, the National Centre of Excellence in Youth Mental Health; [3]Orygen, the National Centre of Excellence in Youth Mental Health, Medical University of Vienna; [4]University Hospital Jena; [5]University of Sydney; [6]Child and Adolescent Psychiatric Service of the Canton of Zürich; [7]University of Hong Kong; [8]Academic Medical Center; [9]Psychiatric Centre Bispebjerg; [10]Psychiatric University Clinics Basel; [11]Institute of Mental Health; [12]University of Manchester, Greater Manchester Mental Health NHS Foundation Trust; [13]Orygen, the National Centre of Excellence in Youth Mental Health, Centre for Youth Mental Health, University of Melbourne, Medical University of Vienna*

**Background:** Adherence to a medication is generally defined as the extent to which patients take medications as prescribed by their health care providers. Poor adherence to study medication is not uncommon posing a major challenge to treatment trails. However, poor adherence may not be randomly distributed but rather be associated with demographic or illness factors. The aim of the present study was to identify factors associated with adherence to study medication in young people at ultrahigh risk of psychosis who participated in the NEURAPRO study.
**Methods:** Secondary analysis of data collected in a multi-centre, double-blind, placebo-controlled, randomized clinical trial to prevent or delay the onset of psychosis in participants at ultrahigh risk of psychosis testing omega-3 polyunsaturated fatty acids (omega-3 PUFAs) vs. placebo, in combination with cognitive behavioural case management (NEURAPRO) were included in this analysis. Measures included the Brief Psychiatric Rating Scale (BPRS), the Scale for the Assessment of Negative Symptoms (SANS), the Montgomery Asberg Depression Rating Scale (MADRS), the Young Mania Rating Scale (YMRS), the Social and Occupational Functioning Assessment Scale (SOFAS), and the Global Functioning: Social and Role scales. Adherence to the study medication was assessed monthly for each participant based on capsule count. The mean adherence rating over the 6-month intervention period was then computed and categorized as either adherent (≤25% of capsules returned) or non-adherent (>25% of capsules returned). Transition to psychosis was defined on the basis of operationalized criteria and assessed with the Comprehensive Assessment of the At-Risk Mental State. Levels of ω-3 PUFAs in fish oil, eicosapentaenoic acid (EPA) and docosahexaenoic acid (DHA) (amongst other fatty acids) were measured as percentage of total fatty acids in erythrocytes at baseline and at month 6 (end-of-intervention).
**Results:** Of 304 randomised participants, 57.9% (N = 176) were non-adherent (>25% of capsules returned) and 128 (42.1%) were adherent (≤25% capsules returned) to the study medication.
No sex differences were observed for adherence rates. At baseline the omega-3 index (EPA+DHA) was significantly lower in the non-adherent group (P = 0.018). The non-adherent group had significant lower scores on the SOFAS (P = 0.001) and the Global Functioning: Social and Role Scale at baseline assessment (P < 0.001 and P = 0.020, respectively) compared to the adherent group. No statistically significant differences were observed on symptom measures at baseline (BPRS, SANS, MADRS, YMRS). The cumulative transition to psychosis rate at month 12 was significantly higher in the non-adherent group compared to the adherent group (14.8% vs. 4.7%; Log rank test: P < 0.001).

**Discussion:** Adherence to study medication was relatively low in NEURAPRO. Poor functioning and lower levels of ω-3 PUFAs at baseline were associated with non-adherence. Young people who were non-adherent had a significantly higher risk of progressing to first episode psychosis. Knowledge about factors associated with adherence could help to improve the delivery of interventions in young people at risk of psychosis.

### T50. SYMPTOMATIC AND FUNCTIONAL RESPONSE TO BREXPIP RAZOLE TREATMENT IN PATIENTS WITH ACUTE SCHIZOPHRENIA BY AGE

Catherine Weiss*[,1], Erin MacKenzie[2], Francois Therrien[1], Peter Zhang[1], Stine Meehan[3]
*[1]Otsuka Pharmaceutical Development & Commercialization, Inc; [2]Lundbeck Canada, Inc; [3]H. Lundbeck A/S*

**Background:** Atypical antipsychotics are the mainstay of treatment for schizophrenia, and have a meaningful effect on positive symptoms and agitation/aggression. More recently, treatment goals have shifted to target functioning; a cycle of deterioration often occurs in early schizophrenia in which recurring relapse results in decreased functioning.
Brexpiprazole is a serotonin-dopamine activity modulator that is a partial agonist at 5-HT1A and dopamine D2 receptors, and an antagonist at 5-HT2A and noradrenaline alpha1B/2C receptors, all at subnanomolar potency. The efficacy of brexpiprazole has been shown in both short- and long-term studies. In this post-hoc analysis from three short-term studies, the proportion of patients achieving symptomatic and functional response was assessed, grouped by age at baseline.
**Methods:** Efficacy and functioning data were pooled from three 6-week, double-blind, placebo-controlled studies in hospitalized patients with acute exacerbation of schizophrenia (Vector [NCT01396421]; Beacon [NCT01393613]; and Lighthouse [NCT01810380]), and stratified according to age at baseline (18–35 years; and 36–65 years). For the current analyses, response was defined as reduction in PANSS score of ≥30% from baseline; a CGI-I score of 1 or 2 (much improved or improved); or reduction in PANSS score of ≥30% OR CGI-I score of 1 or 2. Functional response was defined as an increase in PSP total score of at least 10 points. The analyses were conducted using a mixed-model repeated measures (MMRM) approach with all brexpiprazole doses pooled (2-4mg/day).
**Results:** 557 patients aged 18–35 years and 857 patients aged 36–65 years were analysed. For patients aged 18–35 years, a statistically significantly greater proportion of brexpiprazole-treated vs placebo-treated patients had symptomatic response after 6 weeks of treatment (PANSS ≥30%: 40.5% vs 28.7%, p<0.01; CGI-I 1 or 2: 39.9% vs 25.4%, p<0.001; PANSS ≥30% OR CGI-I 1 or 2: 46.2% vs 32.3%, p<0.01). Similar results were observed for patients aged 36–65 years (PANSS ≥30%: 48.7% vs 37.6%, p<0.01; CGI-I 1 or 2: 47.1% vs 32.7%, p<0.0001; PANSS ≥30% OR CGI-I 1 or 2: 54.8% vs 41.6%, p<0.001). For patients aged 18–35 years, a statistically significantly greater proportion of brexpiprazole-treated vs placebo-treated patients had functional response after 6 weeks of treatment (PSP 10 points change: 46.3% vs 33.0%, p<0.01); similar results were observed for patients aged 36–65 years (49.2% vs 38.2%, p<0.01).
The proportion of patients meeting both symptomatic (using ≥30% PANSS improvement or CGI-I score of 1 or 2) and functional response was statistically significantly greater in brexpiprazole-treated patients vs placebo-treated patients regardless of the age group (18–35 years: 37.4% vs 25.4%, p<0.01; 36–65 years: 41.8% vs 30.2%, p=0.01).
**Discussion:** The results of these analyses confirm that 6 weeks of treatment with brexpiprazole results in symptomatic and functional response in acutely ill schizophrenia patients in both younger patients (age 18 to 35 years) as well as older patients (age 36–65).

### T51. TREATMENT OF NEGATIVE SYMPTOMS OF SCHIZOPHRENIA WITH TRANSCRANIAL CURRENT STIMULATION (TDCS): RESULTS OF RANDOMIZED, DOUBLE-BLINDED, SHAM-CONTROLLED TRIAL

Leandro Valiengo*[,1], Pedro Gordon[1], Mauricio Serpa[1], Acioly Lacerda[2], Wagner Gattaz[1], Martinus Van de Bilt[1], Helio Helkis[1], Andre Brunoni[1]
*[1]University of Sao Paulo; [2]Centro de Pesquisa e Ensaios Clínicos Sinapse-Bairral*

**Background:** The negative symptoms of schizophrenia cause significant distress and impairment. The treatment of them is a challenge, with medications having none or little effect. So, new treatments are necessary for this condition. The aim of the study was to ascertain the efficacy of tDCS in treating negative symptoms of schizophrenia
**Methods:** This study was designed to be a randomized, sham-controlled, double-blinded trial using tDCS for the treatment of negative symptoms of schizophrenia. One-hundred (here we analyzed only 70% of the sample, the remaining will be presented at the meeting) patients will be enrolled and submitted to ten tDCS session over the left dorsolateral prefrontal cortex (anodal stimulation) and left temporo-parietal junction-left (cathodal stimulation), over 5 consecutive days, with 2 mA of current. Participants were assessed with clinical and neuropsychological tests before and after the intervention. The primary outcome was change (over time and across groups) in the scores of the Negative Subscale of Positive and Negative Symptoms Syndrome (PANSS). Our secondary outcomes consist of others scales as SANSS (Scale of Assessment of Negative Symptoms), Calgary and the AHRS (Auditory Hallucinations Rating Scale).
**Results:** From 70% of the sample the active tDCS was significantly superior to sham at endpoint at 6 weeks by negative sub scale of PANSS (mean difference, 3,5 points; SD=6.2; P<.05). The total PANSS and the hallucinations scale had no differences between both groups. The other times of analysis were not found differences between sham and active groups. The others scales (Calgary and SANSS have not being evaluated yet).
**Discussion:** The results of our studies suggests a potential role of tDCS for the treatment of negative symptoms of schizophrenia. The effect size was small. This is the biggest study with tDCS for treating negative symptoms of schizophrenia until now. At the meeting all the data will be analyzed (100 patients), it these could change our preliminary results.

### T52. N-ACETYL-CYSTEINE ADD-ON TREATMENT LEADS TO AN IMPROVEMENT OF FORNIX WHITE MATTER INTEGRITY IN EARLY PSYCHOSIS

Paul Klauser[1], Lijing Xin[1], Margot Fournier[1], Alessandra Griffa[1], Martine Cleusix[1], Raoul Jenni[1], Michel Cuenod[1], Rolf Gruetter[1], Patric Hagmann[1], Philippe Conus[1], Philipp Baumann*[,2], Kim Q. Do[1]
*[1]Lausanne University Hospital; [2]DP-CHUV (TIPP)*

**Background:** Beneficial effects of N-acetyl-cysteine (NAC) on negative symptoms in chronic schizophrenia have been reported in two studies. A recent study in early psychosis from our group, did not report significant improvement in negative symptoms (potentially linked to the modest baseline levels) but showed improvement in cognition (i.e. processing speed) and an increase in the brain antioxidant glutathione (GSH) levels, indicating good target engagement.[1] Indeed, research in animal models highlights the critical role of redox regulation by brain GSH for white matter maturation and maintenance. Given the strong evidence of white matter (WM) alterations in schizophrenia as well as the current lack of etiological treatments, redox dysregulation is an interesting target. The current study aims at investigating the impact of NAC, a precursor of GSH, the main antioxidant in the brain, on WM integrity in patients in the early psychosis phase. We focused on the fornix bundle that has been shown to be impaired in an animal model of oxidative stress[2] (i.e. impaired GSH synthesis) as well as in early psychosis patients.[3]

**Methods:** WM diffusion properties were estimated using generalized fractional anisotropy (gFA) computed from diffusion spectrum imaging (DSI) brain scans acquired in patients who received either NAC (n=10; mean age=25.3 ± 5.7; males/females 9/1) or placebo (n=10; mean age=24.8 ± 7.9; males/females 5/5) as add-on treatment over 6-months. GSH levels were measured in the medial prefrontal cortex using Magnetic Resonance Spectroscopy (MRS).

**Results:** A non-parametric longitudinal voxel-based analysis limited to the fornix revealed a time x treatment interaction which reached significance in the body of the fornix (corrected p<.04) with NAC patients showing an increase in gFA over 6-month of treatment. Importantly, improvement of gFA (i.e. increase) in the fornix of early psychosis patients (NAC and placebo) correlated with increase in cerebral GSH levels (r=.67; p<.005).

**Discussion:** This study is the first to assess the effect of NAC on WM integrity as assessed by diffusion weighted-imaging in the early phase of psychosis. WM alterations appear early in the illness and become widespread in a more chronic phase of the disease.[4] To the best of our knowledge there is currently no approved medication for schizophrenia that show significant effect on WM integrity. In this study, effects of NAC on WM integrity in the fornix were significant despite the limited sample size. This is a small-scale proof of concept study, which was very demanding for early psychosis patients and needs replication in a larger study. Its potential properties to counteract WM deficits may be even more important in individuals at clinical high risk for psychosis. As NAC add-on treatment is safe with no side effects, this study paves the way for preventive approach at the early stages of psychosis.

**References:**

1. Conus P, Seidman L, Fournier M, et al. N-Acetyl-Cysteine in a double-blind randomized placebo- controlled trial: Towards biomarker guided treatment in early psychosis. Schizophr Bull 2017. DOI:10.1093/schbul/sbx093.
2. Corcoba A, Steullet P, Duarte JMN, et al. Glutathione Deficit Affects the Integrity and Function of the Fimbria/Fornix and Anterior Commissure in Mice: Relevance for Schizophrenia. Int J Neuropsychopharmacol 2015; 19: 1–11.
3. Baumann P, Griffa A, Fournier M, et al. Impaired fornix-hippocampus integrity is linked to peripheral glutathione peroxidase in early psychosis. Transl Psychiatry 2016; 6: 1–8.
4. Klauser P, Baker ST, Cropley VL, et al. White Matter Disruptions in Schizophrenia Are Spatially Widespread and Topologically Converge on Brain Network Hubs. Schizophr Bull 2017; 43: 425–35.

### T53. USING ARTIFICIAL INTELLIGENCE PLATFORMS TO ENHANCE STUDY DESIGN IN SCHIZOPHRENIA TRIALS

Laura Shafner*[,1], Chloe Chah[1]

*[1]AiCure*

**Background:** Remote patient monitoring is critical in ensuring optimal drug exposure. Between 30–50% of CNS trials fail because patients are not following the assigned protocol. It is estimated that non-adherence based on pharmacokinetic (PK) data is as high as 39% in schizophrenia trials. An AI platform that uses software algorithms on smartphones to visually and automatically confirm medication ingestion has been used extensively in schizophrenia trials, phases I-IV. Aggregated data demonstrate the feasibility of using the technology in patients with schizophrenia – where smartphone ownership is estimated to be well above 50% - and the potential value of enhancing study design through predictive data and statistical power.

**Methods:** Aggregated data were collected across seven schizophrenia studies; three trials are completed and four are ongoing. Protocols varied by geography, treatment duration, study design, inclusion/exclusion criteria, dosing regimens, and assessment frequency (US and global; six to 52 weeks' treatment duration, lead-in or washout periods, ages 16–65 years, dosing QD or BID, 1–3 units per dose, inpatient and outpatient). Study subjects used the AI application for each dosing administration. In addition to tracking medication intake, the patient-facing interface also provided automated reminders, alarms, dosing windows, clinic visit scheduling, and protocol-specific dosing instructions. Study teams and sites had access to data, analytics, automated notifications, and intervention dashboards.

**Results:** So far, 43,340 adherence parameters have been collected in studies with target enrollments of 1,312 subjects with schizophrenia. For randomized subjects who received at least 1 dose of the study drug, cumulative average adherence as measured by the AI platform (visual confirmation of ingestion) across all treatment groups, including placebo, is 83.6%. Adherence, as measured by the percentage of PK samples above the lower limit of quantification (LLOQ), is 91.2%. Between 3.9% and 12.5% of subjects triggered fraudulent activity alerts (intentional misuse of the technology). The average number of site interventions per subject per study was 4.7 (33.8% text messages; 34.7% phone calls; 31.5% in-person clinic visits). Adherence data logged on the AI platform were used in most studies as the primary measure of adherence (for at home and in-clinic dosing) and as the basis to evaluate eligibility criteria for randomization following placebo lead-in periods.

**Discussion:** Subjects with schizophrenia (stable, acute, positive and negative symptoms, cognitive impairment) treated with antipsychotics demonstrate high rates of adherence using a smartphone-based AI application. Non-adherence based on PK data ranged from 8.3% to 10.4%; a significant reduction from the 39%-50% non-adherence rates observed in clinical trials and real-world settings. Traditional methods to monitor adherence – pill count and self-report methods – are not reliable enough to be of predictive value in lead-in periods, demonstrate poor concordance with PK data, and do not allow sponsors to resolve issues that may affect adherence in real time. Use of the AI platform in schizophrenia studies demonstrates the potential of the technology to enhance data quality, enable the sponsor to estimate the effect of the investigational drug - when used as directed – and improve the likelihood of detecting a signal.

### T54. TAILOR – TAPERED DISCONTINUATION VERSUS MAINTENANCE THERAPY OF ANTIPSYCHOTIC MEDICATION IN PATIENTS WITH NEWLY DIAGNOSED SCHIZOPHRENIA SPECTRUM DISORDERS IN REMISSION OF PSYCHOTIC SYMPTOMS

Signe Dolmer*[,1], Mai Nielsen[1], Merete Birk[1], Ole Mors[1], Anne Emilie Stürup[2], Nikolai Albert[2], Heidi Dorthe Jensen[2], Lene Eplov[2], Carsten Hjortshøj[2], Bjørn Epdrup[2], Merete Nordentoft[2]

*[1]Aarhus University Hospital; [2]Copenhagen University Hospital, Mental Health Center Copenhagen*

**Background:** Schizophrenia spectrum disorders have major implications for the individuals, their families and society.

Antipsychotic medication is the cornerstone in the treatment of psychotic symptoms and is effective in the reduction of psychotic symptoms and of relapse after remission of psychotic symptoms. This is the reason for recommending maintenance treatment with antipsychotic medication in national and international guidelines for the treatment of schizophrenia, one year after remission of psychotic symptoms in first episode psychosis.

The aim of the study is to investigate the effect of tapered discontinuation versus maintenance therapy with antipsychotic medication in patients with newly diagnosed schizophrenia or persistent delusional disorder and with minimum three months remission of psychotic symptoms, and to find minimal effective dose of antipsychotic medication. Negative symptoms, cognitive impairments and the side effects of antipsychotic medication can cause a serious and long-term burden for patients and can reduce their quality of life. The TAILOR study will investigate these important aspects.

**Methods:** The study is a randomized multicenter single blinded clinical trial. The aim is to include 250 patients from the outpatient early intervention program, OPUS, a 2 years manualized psychiatric treatment programme. At baseline patients must have 3 months remission of psychotic symptoms as documented by the SAPS (Schedule for Assessment of Positive Symptoms in Schizophrenia).

The patients will be randomized to either tapered discontinuation or dose reduction of antipsychotic medication or treatment as usual stratified according to substance abuse. The intervention will last for 1 year, and follow up interviews will be made after 1,2 and 5 years.

The patients will receive a user-developed mobile phone application to make daily registrations.

**Results:** The study has been including patients since May 2017.

The first data is expected in 2019.

**Discussion:** The TAILOR trial will contribute to knowledge about the effect of tapering/discontinuation of antipsychotic medication in early phases of schizophrenia spectrum disorders and hopefully the results may guide future clinical treatment regimens of antipsychotic medication.

The trial is a complex medical intervention, and it raises ethical, practical and organizational challenges.

When designing the TAILOR trial ethical questions were raised regarding blinding and the design of the intervention. In the trial only the researchers are blinded, neither clinicians nor patients, because they should be attentive of the high risk of relapse in the discontinuation group. The design gives the clinicians the possibility to adjust the dose of the antipsychotic medication to ensure sufficient treatment. Therefore, the trial only includes assessor blinding and the groups might end up being more similar than intended.

In general, it is of ethical consideration that the trial participants in the tapering/discontinuation group will be subjected to a higher risk of relapse. On the other hand, it seems unethical if research were not to discover the group of patients who can discontinue antipsychotic medication without relapsing.

Practical challenges will be sufficient recruitment or patient motivation and dropout.

### T55. DRIVING ABILITIES IN CLINICALLY STABLE OUTPATIENTS WITH SCHIZOPHRENIA

Falko Biedermann*[,1], Alex Hofer[1], Theresia Pichker[1], Sylvia Pardeller[1], Georg Kemmler[1], Bernhard Holzner[1], Ilse Kurzthaler[1]

[1]*Medical University Innsbruck*

**Background:** According to the UN convention of human rights, individual mobility is an important aspect for people suffering from chronic disease. Recent studies have shown that 30% of patients suffering from schizophrenia have a driving license for motorized vehicles, however, studies on driving abilities among this patient group are scarce. Accordingly, the current study investigates the parameters, which are relevant in this regard.

**Methods:** In this naturalistic study, stable patients, diagnosed with schizophrenia according to ICD-10, between 18 and 60 years of age, are recruited on an outpatient basis. They have to be clinically stable without hospitalization for at least 6 months and have to be on the same medication for at least 6 months. Psychopathology and extrapyramidal motor symptoms (EPS) are assessed by means of the Positive and Negative Syndrome Scale (PANSS) and the Modified Simpson-Angus Scale (MSAS), respectively. Driving abilities are investigated by means of a computerized test battery of the Wiener Testsystem, measuring visual perception, reactivity and stress tolerance, concentration, vigilance, and motor coordination.

**Results:** So far, 42 outpatients suffering from schizophrenia, with a mean age of 42.7 ± 8.9 years and a mean duration of illness of 11.2 ± 5.5 years, have been included into the study. 52 % were male and the mean education was 14.4 ± 4.0 years. The mean PANSS total score was 56.3 ± 20.3 (positive symptoms: 12.9 ± 5.4, negative symptoms: 13.6 ± 5.5, general symptoms: 29.7 ± 13.6). All patients were treated with second generation antipsychotics, and only one had a combination therapy with an additional first generation antipsychotic. We found significant positive correlations between driving abilities and both years of education and EPS, whereas residual symptoms (PANSS) were not associated with driving abilities.

**Discussion:** The relationship between EPS and driving abilities was not surprising, since motor flexibility might be seen as basic requirement in traffic situations. The missing correlation between residual symptomatology and driving abilities, on the other hand, may be explained by very low mean PANSS scores and the small range of scores in our sample. To summarize, these data suggest that in clinically stable outpatients suffering from schizophrenia driving abilities are primarily influenced by EPS rather than by residual symptomatology. Altogether, further studies are needed with a larger sample size.

### T56. AN EXPLORATORY ANALYSIS CONVERTING SCORES BETWEEN THE PANSS AND BNSS

Alan Kott*[,1], David Daniel[1]

[1]*Bracket Global, LLC*

**Background:** The Brief Negative Symptom Scale is a relatively new instrument designed specifically to measure the negative symptoms in schizophrenia. Recently more clinical trials include the BNSS scale as a secondary or exploratory outcome, typically along with the PANSS. In the current analysis we aimed at establishing the equations that would allow conversion between the BNSS scale total score and the PANSS negative subscale and PANSS negative factors score as well as conversion equations between the expressive deficits and avolition/apathy factors of the scales. (Kirkpatrick, 2011; Strauss, 2012)

**Methods:** Data from 518 schizophrenia clinical trials subjects with both PANSS and BNSS data available were used. Regression analyses predicting the BNSS total score with the PANSS negative subscale score, and the BNSS total score with the PANSS Negative factor (NFS) score were performed on data from all subjects. Regression analyses predicting the BNSS avolition/apathy factor (items 1, 2, 3, 5, 6, 7, and 8) with the PANSS avolition/apathy factor (items N2, N4 and G16) and the BNSS expressive deficits factor (items 4, 9, 10, 11, 12, and 13)with the expressive deficits factor (items N1, N3, N6, G5, G7, and G13)of the PANSS were performed on a sample of 318 subjects with individual BNSS item scores available. In addition to estimating the equations we as well calculated the Pearson's correlations between the scales.

**Results:** The PANSS and BNSS avolition/apathy factors were highly correlated (r=0.70) as were the expressive deficit factors r=0.83). The following equations predicting the BNSS total score were obtained from regression analyses performed on 2,560 data points:

BNSS_total = -11.64 + 2.10*PANSS_negative_subscale

BNSS_total = -9.26 + 2.11*PANSS_NFS

The following equations predicting the BNSS factor scores from the PANSS factor scores were obtained from regression analyses performed on 1,634 data points:

BNSS_avolition/apathy = -2.40 + 2.38 * PANSS_avolition/apathy

BNSS_expressive_deficit_factor = -4.21 + 1.27 * PANSS_expressive_deficit_factor

**Discussion:** The BNSS differs from the PANSS negative factor because it addresses all five currently recognized domains of negative symptoms including anhedonia and attempts to differentiate anticipatory from consummatory states. In our analysis we have replicated the strong correlation between the BNSS total score and PANSS negative subscale and newly identified strong correlations between the BNSS total score and NFS as well as strong correlations between the avolotion/apathy and expressive deficit factors of the BNSS and the PANSS scales. (Kirkpatrick, 2011)The provided equations offer a useful tool allowing researchers and clinicians to easily convert the data between the instruments for reasons such as pooling data from multiple trials using one of the instruments, to allow interpretation of results within the context of previously conducted research, etc. but as well offer a framework for risk based monitoring to identify data deviating from the expected relationship and allow for a targeted exploration of the causes for such a disagreement. The data used for analysis included not only subjects with predominantly negative symptoms but as well acutely psychotic subjects as well as subjects in stable conditions allowing therefore to generalize the results across the majority of schizophrenic subjects. This post-hoc analysis is exploratory. We plan to further explore the potential utility of equations addressing the relationships among schizophrenia measures of symptom severity in an iterative manner with larger datasets.

### T57. EFFECTS OF 0.5MS AND 1.5MS PULSE-WIDTHS ON CARDIOVASCULAR FUNCTION IN SCHIZOPHRENIA PATIENTS RECEIVING ELECTROCONVULSIVE THERAPY

Dhruva Ithal*[,1], Sayantanava Mitra[1], Shyam Sundar A[1], C. Naveen Kumar[1], Jagadisha Thirthalli[1], V. J. Ramesh[1], B. N. Gangadhar[1]
[1]*National Institute of Mental Health and Neurosciences*

**Background:** Electroconvulsive therapy (ECT) has been shown to have a profound effect on cardiovascular functions. The initial parasympathetic response, followed by the sympathetic surge and the second parasympathetic peak characterize a typical ECT session and in patients with pre-existing cardiac disorders, this 'roller-coaster ride' of autonomic discharges can drastically increase morbidity and mortality; albeit such incidences are rare nowadays with the advances in medical technology. While laterality and stimulus dose (in terms of millicoulombs, mC) are known to affect cardiovascular response, the effect of pulse-width (PW) on the latter has not been explored. Compared to 1.5-milisecond (ms) stimulus pulse trains, trains with 0.5ms PW last 3 times longer for equivalent stimulus charges, other parameters remaining constant. This would translate to greater initial parasympathetic response duration, and the implications of such occurrences for cardiovascular well-being are largely unknown.
**Methods:** Seventy-one consenting adult (M=33, F=38; mean age 30.87 ± 9.59 years, mean duration of illness 89.68 ± 77.98 months) patients, with a diagnosis of Schizophrenia, were randomly assigned to receive bilateral ECT with either 0.5ms (n=35) or 1.5ms (n=36) PW stimulus; after obtaining institutional ethical-committee's approval. Seizure threshold was determined during the first session. Rate-Pressure product (RPP; pulse*systolic blood-pressure) was calculated during the second ECT session, in which stimulus was administered at 1.5–2 times the threshold for the two groups, at 5 time points (RPP1-5, viz. pre-anaesthesia, during anaesthesia, during convulsive motor seizure, 1 and 2 minutes post seizure, respectively). They were compared between the groups using independent-sample t-test. At baseline, the patients were assessed on PANSS for psychopathology.
**Results:** Two groups did not differ on socio-demographic and clinical characteristics at baseline. Mean administered dose of anaesthetic agent and muscle relaxant were comparable. While the mean seizure threshold and mean charge administered at 2nd ECT were significantly lower in the 0.5 ms group, they were otherwise comparable on mean duration of seizure (motor and EEG), and the RPPs at all 5 time-points. Both Max.RPP (18102.84 ± 4477.4 mmHg/min in 0.5ms, 17935.33 ± 3598.5 mmHg/min, p=0.864) and Max.RPP-RPP2 (5010.58 ± 2893.3 mmHg/min in 0.5ms, 5811.12 ± 4270.9 mmHg/min in 1.5ms, p=0.389) were comparable between the two groups.
**Discussion:** The characteristic sequence of cardiac events unfolding in an ECT session comprises of a temporary asystole during the administration of the stimulus, followed by an increase in blood pressure and pulse rate during clonic phase, and another slowing of heart rate at the end of motor seizure. The stimulus train duration in 0.5ms group lasts 3 times longer than in 1.5ms group for an equivalent amount of charge, thus increasing the asystole duration and theoretically altering subsequent autonomic responses. However, the groups failed to demonstrate any significant effects of these alterations in terms of altered cardiac activity implying that such alterations might not be clinically relevant. It is well known that briefer PWs cause lesser cognitive side-effects, are more efficient in eliciting seizures. present analysis shows that the two PWs of 0.5ms and 1.5ms might have similar effects on cardiovascular function, at least in otherwise-healthy adult schizophrenia patients, for similar anaesthetic agents, even if the train with 0.5ms PW lasts for double the time as with 1.5ms PW.

### T58. SARCASM COMPREHENSION AS A SOCIAL COGNITION MEASURE IN SCHIZOPHRENIA – A SYSTEMATIC LITERATURE SEARCH AND META-ANALYSIS ON THE USE OF THE TASIT

Alexander Rapp*[,1], Franziska Purr[1], Anne Felsenheimer[1]
[1]*University of Tuebingen*

AQ1

**Background:** Social cognition tasks with higher ecologically validity could be helpful both as an outcome measure for training and for social cognition impairment in schizophrenia. The comprehension of sarcasm and irony is a candidate for a valid, replicable task.
**Methods:** Tests and paradigms as well as studies in schizophrenia are available in English, Dutch, German, Italian, Greek, Japanese and other languages. The Awareness of Social Inference Test (TASIT) (McDonald et al.,J head trauma rehabil 2003,) is currently the by far most applied paradigm. Here, we present a systematic literature research and meta-analysis on application of these paradigms in patients with schizophrenia.
**Results:** 25 studies with data from n=2185 patients with schizophrenia and n=1474 controls used the TASIT. This exceeds the numbers for other irony comprehension paradigms. Separate meta-analyses were calculated for the "sarcasm-enriched" and "sarcasm-minimal" subtests with data from 5 different English language studies. In both subtests, patients with schizophrenia showed significant impairment. Non-English translations of the TASIT show a comparable picture. Longitudinal data are available from 4 studies. Studies in high risk populations showed mixed results, however the TASIT is included in longitudinal cohort studies such as NAPLS-2.
**Discussion:** We discuss differences with other task such as paradigms without prosodic or face information or the available fMRI investigations.

### T59. VIRTUAL REALTY ASSESSMENT OF FUNCTIONAL CAPACITY IN EARLY SCHIZOPHRENIA: ASSOCIATIONS WITH NEUROCOGNITION, FUNCTIONAL CAPACITY PERFORMANCE, AND DAILY FUNCTIONING

Joseph Ventura*[,1], Tamara Welikson[2], Kenneth L. Subotnik[1], Arielle Ered[1], Richard Keefe[2], Gerhard H. Hellemann[1], Keith H. Nuechterlein[1]
[1]*UCLA Semel Institute for Neuroscience & Human Behavior;*
[2]*Duke University*

**Background:** Research using virtual reality assessment of functional capacity has shown promise as a reliable and valid way to assess treatment response in patients with established schizophrenia. There has been little work on virtual reality based assessments of functional capacity for patients in the early phase of schizophrenia. We examined whether virtual reality based assessment methods reveal functional capacity deficits in young patients and relevant relationships with established measures of neurocognition, functional capacity performance, and daily functioning.
**Methods:** The sample consisted of UCLA Aftercare Research Program patients (n=42) who were diagnosed by trained raters administering the SCID and who met criteria for schizophrenia, schizoaffective disorder, or schizophreniform disorder, and screened normal control subjects (n=13). Patients were within 2 years of their first psychotic episode upon clinic entry, were an average of 23.2 years old, and had an average of 12.9 years of education. The Virtual Reality Functional Capacity Assessment Tool (VRFCAT) was the computer-based measure of functional capacity. We used the MATRICS Consensus Cognitive Battery (MCCB) as an objective measure of neurocognition and the UCSD Performance-Based Skills Assessment (UPSA) to assess functional capacity performance. The Global Functioning Scale: Role and Social, and the Role Functioning Scale were used to assess work and school performance, familial interactions, and social functioning.
**Results:** We were able to confirm that the deficit in functional capacity performance measured using VRFCAT is present in the early course of schizophrenia in that the patients were slower and committed more errors (M=830.41) as compared with normal controls (M=716.84; t=3.0, p<.01). Virtual reality based assessment of functional capacity was correlated with objective measures of neurocognition (MCCB Overall Composite), r=-.71, p=<.01, standard approaches to functional capacity assessment (UPSA), r=-.66, p=<.01, work and school functioning (r=-.52, p<.01), and level of social relationships (r=-.43, p=<.03), but not familial relationships (r=-.03, p=.87). Interestingly, neither neurocognition (MCCB) nor functional capacity performance (UPSA) were correlated with the level of familial relationships.
**Discussion:** We extend previous findings in that even patients in the early course of schizophrenia showed virtual reality based functional capacity performance deficits when compared with normal control subjects. Virtual reality based performance was correlated with neurocognition, suggesting that it may be sensitive to changes in cognition. Furthermore, correlations with everyday work/school and social functioning indicate promise as a co-primary measure to index change in functioning in response to treatment. Interestingly, none of our measures of functional capacity or neurocognition were correlated with familial relationships indicating that the determinates of family interactions might be driven by factors other than cognitive capacities.

### T60. HEALTH LITERACY IN PEOPLE WITH SCHIZOPHRENIA ATTENDING COMMUNITY MENTAL HEALTH CLINICS

Sumana Thomson[1], Cherrie Galletly*[,1], Christopher Prener[2], Suzanne Garverich[3], Dennis Liu[1], Alisa Lincoln[3]
*[1]The University of Adelaide; [2]Saint Louis University; [3]Northeastern University*

**Background:** Health literacy (HL) has been defined as the degree to which individuals possess the capacity to obtain, process, understand and utilise basic health information. For people with schizophrenia, important aspects of their HL include the ability to understand information about their illness and treatment, taking medications correctly, and interacting with clinicians. Schizophrenia is associated with lower levels of education, which has been found to negatively impact on HL. Further, schizophrenia is often associated with cognitive impairment, but the relationship between HL and cognitive function in this patient population is not known.
Studies of HL in people with physical disorders have demonstrated that people with poor HL have poorer outcomes, with greater morbidity and mortality. There has been very little research into HL in schizophrenia, although it may be expected that those with poor HL might have more difficulty managing their illness and interacting with clinical services.
**Methods:** People living in the community, and attending public community mental health outpatient clinics in the Northern suburbs of Adelaide, Australia, were invited to participate in the study. They were interviewed by trained research staff, and completed the WAIS VI digit symbol coding (DSC), Verbal Fluency (VF; animal naming), the short version of the Test of Functional Health Literacy in Adults (S-TOFHLA) along with two parts of the Woodcock-Johnson III measuring aural literacy (Part 4; WJ4) and reading literacy (Part 9; WJ9). Of 101 participants, 62 had schizophrenia, while the other 39 had a range of other diagnoses.
**Results:** The 62 participants with schizophrenia had a mean age of 41.2 (SD 9.9) years and 61% were male. They had a mean of 11.02 (SD 1.5) years of education. The remaining participants had a mean age of 43.3 (SD 13.4) years, 46% were male, and the mean years of education was 11.3 (SD 2.5). 90% of the schizophrenia group were at or below 8th grade (Year 8) level for aural literacy, and 63% were at or below 8th grade (Year 8) for reading literacy.
Those with a schizophrenia diagnosis had lower scores on the WJ9 (mean 8.3, SD 4.5) compared with the non-schizophrenia group (mean 11, SD 5.1); t = 2:739; p = 0:007, medium Cohen's D effect size (D = 0:548). However, there was not a significant difference (t = 1.975, p = .051) in aural literacy between the schizophrenia and non-schizophrenia groups.
Using the S-TOFHLA, 81% of the schizophrenia group had adequate HL; 6% were marginal and 13% were inadequate. In contrast, 97% of the non-schizophrenia group had adequate HL. The schizophrenia group had lower mean S-TOFHLA scores (mean 28.6, SD 7.5; compared to mean 31.8, SD 4.8); t = 2.369; p = 0.020, medium Glass's effect size (G = 0.657).
In all subjects, there was a moderate, positive relationship (r = 0.359; p < .05) between education and the TOFHLA score. There was also a positive correlation between the S-TOFHLA score and the aural and reading literacy scores
**Discussion:** The majority of people with schizophrenia had very poor aural and verbal literacy, and there was a correlation between education and literacy skills, and HL. People with schizophrenia tend to have less years of school attendance, and cognitive impairment is a core component of the disorder.
However, only 19% of the schizophrenia group had inadequate HL. This finding suggests that their HL skills are better than expected, given their educational and literacy deficits. This may reflect their engagement in case management and psychoeducation whilst attending the community MH clinic; the clinicians might have been providing effective ongoing education about managing their mental illness, medication and interactions with health services.

### T61. ANTISACCADE AND MEMORY GUIDED SACCADE PERFORMANCE ACROSS THE SCHIZOPHRENIA CONTINUUM

Elizabeth Thomas[1], Susan Rossell*[,2], Eric Tan[2], Sean Carruthers[3], Philip Sumner[3], Kiymet Bozaoglu[4], Caroline Gurvich[5]
*[1]Monash Alfred Psychiatry Research Centre; [2]Monash Alfred Psychiatry Research Centre, Swinburne University, St. Vincent's Hospital; [3]Swinburne University, Monash Alfred Psychiatry Research Centre, The Alfred Hospital; [4]University of Melbourne; [5]Monash Alfred Psychiatry Research Centre, The Alfred Hospital, Monash University*

**Background:** Saccadic (ocular motor) deficits are one of the most replicated findings in schizophrenia. However, less research has been conducted investigating the broader schizophrenia continuum. Recent research suggests that the personality characteristics and symptoms observed in schizophrenia lie on a continuum with subclinical symptoms, known as schizotypy, observed in the non-clinical population. Schizotypy is considered a suitable model for investigating schizophrenia as it mirrors the symptoms, albeit in a more subtle manner. As saccadic deficits are a cognitive hallmark of schizophrenia, it is believed that saccadic deficit may be associated with higher schizotypy. The aim of the current study was to 1) replicate previous findings of impairments in antisaccade and memory-guided saccade performance in schizophrenia and 2) investigate the relationship between antisaccade and memory-guided saccade performance and schizotypy.

**Methods:** 105 adults (35 patients with schizophrenia/schizoaffective disorder and 70 healthy controls) completed the antisaccade and memory-guided saccade tasks, which engage spatial working memory and inhibition processes. The variables analysed for both saccade paradigms were error rate, latency (ie. reaction time) and gain (ie. spatial accuracy). Schizotypy was assessed using the Oxford-Liverpool Inventory of Feelings and Experiences (O-LIFE), a 104 item questionnaire which measures the three main schizotypy factors: Unusual Experiences, Introvertive Anhedonia and Cognitive Disorganisation. A total O-LIFE score was also calculated from these three schizotypy factors as a representation of global schizotypy. Controls were then divided into low (n = 35) and high schizotypy groups (n = 35). A MANOVA was conducted to observe differences in eye movement variables between low schizotypy individuals, high schizotypy individuals and patients. Correlations were also conducted to further investigate these relationships.

**Results:** Antisaccade error rate, ($p < 0.001$), antisaccade latency ($p = 0.007$), memory-guided saccade error rate ($p = 0.009$) and latency ($p < 0.001$) were significantly different between patients and controls. When comparing low schizotypy, high schizotypy and patient groups, the MANOVA revealed significant differences for antisaccade and memory-guided saccade latency and a non-significant trend for antisaccade gain. However, post-hoc analyses revealed that there was only a significant difference between low schizotypy and patient groups ($p < 0.001$), but not between low schizotypy and high schizotypy nor between high schizotypy and patient. Looking across the schizophrenia continuum, there were significant correlations between the total O-LIFE score and antisaccade gain ($p = 0.033$), memory-guided saccade latency ($p = 0.014$) and memory-guided saccade gain ($p = 0.011$). A non-significant trend was also observed between the total O-LIFE score and antisaccade latency ($p = 0.088$).

**Discussion:** This study replicated previous findings of impaired saccade performance in schizophrenia. In addition, it also replicated findings of impaired antisaccade performance in higher schizotypy and is the first study to investigate and demonstrate the relationship between higher schizotypy and impaired memory-guided saccade performance. Overall, these findings supporting the use of schizotypy as a model for schizophrenia and also support the theory of schizotypy and a broader schizophrenia continuum.

### T62. COMPARISON OF NEUROCOGNITIVE FUNCTIONS IN FIRST-EPISODE SCHIZOPHRENIA PATIENTS, NON-PSYCHOTIC SIBLINGS, AND INDIVIDUALS AT CLINICAL HIGH-RISK FOR PSYCHOSIS

On Ki Chu[1], Wing Chung Chang*[,1], Hoi Ching Lee[1], Suet In Chan[1], Sanyin Chiu[1], Lai Ming Hui[1], Kit Wa Chan[1], Ho Ming Lee[1], Yi Nam Suen[1], Eric Chen[1]
[1]*The University of Hong Kong*

**Background:** Neurocognitive impairment is a core feature of schizophrenia, and has been observed among healthy non-psychotic siblings of schizophrenia patients as well as individuals at clinical high-risk (CHR) for psychosis. Thus far, few studies have directly contrasted neurocognitive performance between non-psychotic siblings and CHR samples. Potential differential patterns of neurocognitive deficits among schizophrenia patients, familial high-risk and CHR samples remain to be clarified. This study aimed to compare neurocognitive functions among first-episode schizophrenia (FES) patients, their non-psychotic siblings, CHR individuals, and healthy controls.

**Methods:** FES patients (n=69, mean age=25.3) and CHR individuals (n=97, mean age=21.1) without family history of psychosis were recruited from a territory-wide specialized early intervention service for psychosis in Hong Kong. A group of non-psychotic siblings of FES patients (n=50, mean age=25.4) and healthy controls (HC) (n=68, mean age=24.5) were also recruited. A standardized battery of neurocognitive tests encompassing working memory, processing speed, executive function, visual memory, verbal learning, and sustained attention was administered. Group differences were examined using analysis of covariance (ACOVA) with Bonferroni correction applied for statistical significance ($P<0.008$), controlling for age and years of education.

**Results:** Compared with HC, FES patients exhibited significantly poorer performance across all neurocognitive domains (Hedges g ranged: 0.48–1.73), while CHR individuals demonstrated significantly worse neurocognitive functioning in all domains (Hedges g ranged: 0.53–1.15) but sustained attention. Non-psychotic performed significantly worse than HC in executive function (Hedges g=0.63, $p<0.001$), visual memory (Hedges g=0.57, $p=0.002$), verbal learning (Hedges g=0.52, $p=0.001$), and working memory (Hedges g=0.37, $p=0.003$). Among four groups, FES patients displayed the most severe neurocognitive impairment. The pattern of neurocognitive dysfunction was similar between CHR and non-psychotic sibling groups, except for processing speed, of which CHR individuals demonstrated greater degree of impairment than siblings in digit symbol coding test ($p<0.001$).

**Discussion:** Our results indicate a gradient of neurocognitive impairment across FES, CHR and non-psychotic sibling samples, reflecting differential degrees of psychosis liability. Processing speed, as measured by digit symbol coding test, demonstrated the highest discriminant utility in discriminating CHR from familial high-risk individuals. Our findings thus confirm the critical role of neurocognitive dysfunction as a reliable risk indicator and an endophenotype for schizophrenia and related psychoses.

### T63. TOWARDS A COMPREHENSIVE SEMANTIC MEMORY NETWORK IN SCHIZOPHRENIA: PRELIMINARY RESULTS USING MAGNETOENCEPHALOGRAPHY (MEG) IN SCHIZOTYPY

Rachel Batty*[,1], Will Woods[1], Susan Rossell[2]
[1]*Swinburne University of Technology;* [2]*Swinburne University, Monash Alfred Psychiatry Research Centre*

**Background:** Semantic memory (memory for facts, concepts, and knowledge of the external world) abnormalities are predicted to underlie disturbances in thought and language, deficits in cognitive domains, and the development and maintenance of delusions in patients with schizophrenia. Electroencephalographic (EEG) recordings have successfully identified the neural time course for the processing of semantic information as an electrophysiological response between 300 and 500ms post stimulus (i.e., the N400). The N400 is a remarkably consistent and highly sensitive neural response to semantic relationships, and is thought to index the binding of current stimuli into context by detecting whether meaning is shared with recently processed stimuli or items in memory. To date, the N400 appears to be amodal: an index of semantic processing irrespective of stimulus type (e.g., word/picture stimuli alike), and has shown mixed findings in schizophrenia. However, existing literature has largely relied on EEG or functional magnetic resonance imaging (fMRI) techniques, and these are constrained in spatial and temporal resolution, respectively. Comparatively, MEG provides excellent spatio-temporal resolution, not possible from other stand-alone neuroimaging techniques. We aimed to determine the neuromagnetic correlates of novel semantic triads in both lexical and picture form, and to determine N400m differences in high/low schizotypal samples.

**Methods:** MEG was recorded (whole-head 306 channel Elekta Neuromag® TRIUX magnetometer system) in 35 nonclinical controls (18 male) while completing a novel explicit semantic association task. MEG data were continuously sampled at 1KHz (0.1Hz high pass filter). Following MaxFiltering, data was processed using MNE for Python. Data were filtered offline (40Hz lowpass) and epoched at -300ms to 800ms post- target stimulus onset. The largest peak was measured at sensor triplets at temporo-parietal sites in both hemispheres. High/low schizotypal samples were determined by a median split of the Oxford-Liverpool Inventory of Feelings and Experiences (cognitive disorganisation scale; high=17, low=18).
**Results:** Preliminary sensor level analyses demonstrated an N400m at temporo-parietal sites in response to both word and picture stimulus sets (with an earlier peak to pictures). Neither amplitude nor latency was significantly different between schizotypal samples, however a significant task x hemisphere x group interaction was found for N400m latency, $F(1.00,33.00) = 6.18$, $p<.02$.
**Discussion:** An N400m was confirmed in response to the novel lexical task. The earlier peak (~200ms) to picture stimuli suggests that pictorial semantic information may be processed more rapidly than lexical information. The significant schizotypal group latency interaction demonstrated that while individuals low in schizotypal traits process lexical stimuli first in the right hemisphere (followed by the left) and picture stimuli first in the left hemisphere (followed by the right), individuals high in schizotypal traits do not demonstrate hemispheric specificity/laterality according to stimulus type. The data is currently being analysed for (i) source localisation, (ii) deep source contributions (e.g., hippocampus), and (iii) de/synchronisation of neural oscillations (across six frequency bands; 1-8Hz, 8-30Hz, 30-50Hz, 70-120Hz, 120-200Hz, and 200-300Hz).

### T64. SUBMISSION WITHDRAWN

### T65. EVALUATING PATTERNS OF SEMANTIC AND EXECUTIVE DYSFUNCTION IN SCHIZOPHRENIA: A CLUSTER ANALYSIS APPROACH

Eric Tan*[,1], Denny Meyer[1], Erica Neill[2], Caroline Gurvich[4], Susan Rossell[3]
*[1]Swinburne University; [2]University of Melbourne; [3]The Alfred Hospital and Monash University, Swinburne University*

**Background:** Semantic and executive dysfunction are among the most prominent of the cognitive impairments in schizophrenia. Using a cluster analysis (CA) approach, the primacy of semantic and executive dysfunction and their relationship to psychopathology was examined in a two-step investigation.
**Methods:** In Study One, 76 schizophrenia/schizoaffective disorder (SZ) patients completed three semantic (category fluency productivity, category errors, Hopkins Verbal Learning Test) and three executive function (inhibition, switching, verbal fluency) measures. Three groups were predicted: semantic-dominant (SD), executive-dominant (ED) and mixed. In Study Two, 52 SZ patients and 48 healthy controls completed the MATRICS Consensus Cognitive Battery (MCCB) alongside the previous semantic/executive battery.
**Results:** For Study 1, the CA results confirmed the first two specific groups but revealed a third group unimpaired in both domains (UN). Positive and negative symptoms did not differ between all groups. For Study 2, the CA results confirmed the presence of the same three groups: SD, ED and UN. One-way ANOVAs confirmed that MCCB overall cognitive scores for UN group were significantly higher compared to the SD and ED groups, which did not differ from each other; however, all three clinical groups still performed significantly worse than healthy controls. Psychopathology again did not differ between the three clinical groups.
**Discussion:** The findings confirm semantic and executive dysfunction as two main areas of cognitive impairment in SZ while also affirming the presence of cognitively impaired patients without these two primary deficits. Symptomatology patterns do not appear to differ between cognitive impairment profiles, highlighting the complexity of symptomatology mechanisms and cognitive deficits being a discrete entity within the illness. These conclusions have implications for the nosology of schizophrenia and the delivery of cognition-based therapies.

### T66. PSYCHOMETRIC VALIDATION OF A NOVEL PATIENT-REPORTED OUTCOME MEASURE FOR ASSESSING PATIENTS' SUBJECTIVE EXPERIENCE OF COGNITIVE IMPAIRMENT OF SCHIZOPHRENIA (PRECIS)

Raymond Rosen[1], Jeremiah Trudeau[2], Steven Silverstein[3], David Henderson[4], Adam Smith[5], David Walling[6], Miguel Garcia[2], Bethany Davis[7], Leonard Derogatis[8], Michael Sand*[,9]
*[1]New England Research Institutes; [2]Boehringer Ingelheim Pharmaceuticals Inc.; [3]Rutgers University; [4]Boston University School of Medicine, Boston Medical Center; [5]Richmond Behavioral Associates; [6]CNS Network, Inc.; [7]Synexus; [8]Maryland Center for Sexual Health; [9]Boehringer Ingelheim Pharma GmbH & Co. KG*

**Background:** We have previously described the development and content validity of a new patient-reported outcome measure (PRO) to assess patients' subjective experience of cognitive impairment in schizophrenia: The Patient-Reported Experience of Cognitive Impairment in Schizophrenia (PRECIS). Here we assess the psychometric properties of the PRECIS PRO in patients with schizophrenia and healthy age-matched controls with the aim of developing a revised version for use in clinical studies.
**Methods:** The PRECIS PRO is a 35-item scale comprising eight concept domains (memory, communication, control, planning, handling problems, attention, sharp thinking and overall experience), each with multiple individual items. PRECIS was administered to psychiatrically-healthy controls (single visit), and a subset of patients in a large, clinical trial assessing patients with schizophrenia on stable antipsychotic treatment (NCT02281773) at baseline and Weeks 6, 9 and 12. Analysis of the original 35-item PRECIS PRO included factor structure, factor analysis (FA), internal consistency, test-retest reliability and discriminant (known groups) validity testing. FA was performed on all pre-treatment scores in the patient group (n=410) and patients and controls combined (n=498). Individual items with less than adequate reliability or validity were then identified and eliminated or modified.
**Results:** Questionnaire responses were collected from 410 patients with schizophrenia and 88 healthy controls. The mean (standard deviation [SD]) total PRECIS score was significantly lower for healthy controls (1.39 [0.7]) compared with patients (2.06 [1.2]; $p<0.0001$), as was overall experience domain score (1.41 [0.7] vs 2.35 [1.3]; $p<0.0001$). For each domain of patient experience, PRECIS mean scores were also significantly lower for healthy controls compared to patients with schizophrenia. The mean differences between groups ranged from -0.94 (overall experience domain) to -0.52 (control domain; $p<0.0001$, all domains). Patients with schizophrenia had wider response distributions compared with controls, while the control group had marked "floor effects" across most items. Initial exploratory FA of the 35-item PRECIS PRO identified a 6-domain solution that accounted for 62% of total item variance, and Cronbach's alpha (0.959) indicated an extremely high level of internal consistency. Following analyses of the 35-item PRECIS PRO, a total of 11 items were eliminated based on pre-specified criteria (poor loading onto identified factors, marked floor effects in patient groups or <50% test-retest reliability). Confirmatory FA of the revised 24-item PRECIS PRO identified 1 primary domain (attention) and 3 secondary additional domains (memory, executive function, communication). An additional domain included items related to patient distress or bother related to cognitive impairment. There was a high level of internal consistency both for the overall 24-item PRECIS PRO (Cronbach's alpha= 0.942) and individual domains (Cronbach's alphas: 0.743–0.873). Intraclass correlation coefficients were 0.78 for the overall 24-item PRECIS PRO and ranged from 0.49–0.74 for individual domains. Finally, discriminant validity testing confirmed there were significant differences between the patient group and the control group in each of the 5 domains of the revised 24-item PRECIS PRO (p<0.0001).
**Discussion:** This large validation study demonstrated that the revised 24-item PRECIS PRO is a valid and reliable PRO measure with good internal consistency, adequate test-retest reliability and strong discriminant validity. PRECIS may therefore serve to define key patient-based endpoints for use in future clinical studies.
**Funding:** Boehringer Ingelheim (1289.20)

### T67. NEUROLOGICAL SOFT SIGNS IN THE COURSE OF SCHIZOPHRENIA: A LONGITUDINAL ANALYSIS IN CHRONIC SCHIZOPHRENIA

Christina Herold*[,1], Céline Duval[1], Marc Lässer[2], Ulrich Seidl Seidl[3], Dusan Hirjak[1], Philipp Thomann[1], Johannes Schröder[1]
*[1]University of Heidelberg; [2]Rehabilitation Hospital Zihlschlacht; [3]Center for Mental Health, Klinikum Stuttgart*

**Background:** Neurological soft signs (NSS) are minor ('soft') neurological abnormalities in sensory and motor performance, which are frequently reported in patients with schizophrenia at any stage of their illness. It has been demonstrated that NSS vary in the clinical course of the disorder: Longitudinally NSS seem to decrease in parallel with remission of psychopathological symptoms, an effect which mainly applies to patients with a remitting course. However, these findings are mainly based on patients with a first episode of the disorder and the course of NSS in patients with chronic schizophrenia and persisting symptoms is rather unknown.
**Methods:** Therefore, we investigated 21 patients with chronic schizophrenia (duration of illness: 22.8 years ± 11.5) twice with a follow-up time interval of 7 years. Baseline and endpoint NSS scores were evaluated by the Heidelberg Scale, established instruments were used to rate psychopathological symptoms and neuropsychological performance.
**Results:** Results show a significant increase of the NSS subscales "motor coordination" and "integrative functions" with stable positive and negative symptoms, including apathy, as well as chlorpromazine equivalents. Along with this, neuropsychological parameters as verbal memory, verbal fluency and cognitive flexibility deteriorate significantly. Regression analyses show that the TMT B performance/cognitive flexibility and the SANS global score/negative symptoms at baseline are strong predictors for NSS increase at follow-up.
**Discussion:** These results illustrate a significant aggravation of NSS in patients with chronic schizophrenia over time, while psychopathological symptoms remain stable. In addition, cognitive performance is deteriorating as well, with cognitive flexibility together with negative symptoms as strongest predictors for NSS changes.

### T68. DIFFERENT INFLUENCES OF INTELLECTUAL FUNCTIONING AND COGNITIVE PERFORMANCE TO FUNCTIONAL OUTCOMES IN SCHIZOPHRENIA AND HEALTHY CONTROLS

Leticia Czepielewski*[,1], Clarissa Gama[1], Cameron Carter[2], Angus MacDonald, III[3], James Gold[4], Steven Silverstein[5], Deanna Barch[6]
*[1]Universidade Federal do Rio Grande do Sul; [3]University of California, Davis [4]University of Minnesota; [5]University of Maryland Baltimore; [6]Rutgers University; [7]Washington University*

**Background:** Individuals with schizophrenia (SZ) have marked functional impairments in wide range of domains, such as employment, independent living and interpersonal relationships. Several clinical, cognitive and psychological factors have been shown to predict functional outcomes. However, current pharmacological and psychosocial treatments have failed to rehabilitate patients, which indicates that the mechanisms of functional outcomes are not completely clear. Therefore, we aimed to better understand the relationship between intellectual and cognitive performance to functional outcome of subjects with SZ compared to unaffected individuals. Considering the neurodevelopmental course of SZ, our hypothesis was that premorbid crystallized IQ would interact with cognition to influence functionality.
**Methods:** We included 188 individuals with confirmed diagnosis of SZ and 268 unaffected subjects (HC) from two separate multisite studies conducted by the Cognitive Neuroscience Test Reliability and Clinical Applications for Schizophrenia (CNTRACS) consortium. We used the following variables for the analysis: a) Estimated crystallized premorbid intellectual functioning (IQ): Wechsler Test of Adult Reading (WTAR); b) Cognitive performance: Dot Probe Expectancy task (DPX) and Relational and Item-Specific Encoding task (RISE) from CNTRACS; c) Functional capacity: UCSD Performance-based Skills Assessment – Brief (UPSA-B). We conducted linear regressions to predict functional outcome considering demographic, intellectual, and cognitive variables, as well as the interaction between cognition and IQ in participants with SZ and HC separately.
**Results:** Participants with SZ had worse cognitive performance and premorbid IQ, and poorer functional outcome compared to HC. For the prediction of UPSA-B, the regression model that included cognition and IQ as predictors and age and parental SES as covariates was significant in SZ ($F(4, 124) = 8.473$, $p < .001$, Adj. $R^2 = .189$), with the both variables showing significant main effects: IQ ($\beta = .311$, $t = 3.324$, $p = .001$) and cognition ($\beta = .216$, $t = 2.630$, $p = .0010$). When we included the IQ x cognition interaction ($F(5, 123) = 7.035$, $p < .001$, Adjusted $R^2 = .191$), it did not significantly improve the model ($F = 1.224$, $p = 0.27$), and the interaction was not significant ($\beta = -.11$, $t = -1.106$, $p = .27$). In HC, the regression model with only main effects was similar to what was seen in SZ ($F(4,313) = 27.62$, $p < .001$, Adj. $R^2 = .25$), with main effects of IQ ($\beta = .239$, $t = 4.418$, $p < .001$) and cognition ($\beta = .349$, $t = 6.891$, $p < .001$) (Figure 1). However, when we included the IQ x cognition interaction ($F(5,312) = 24.15$, $p < .001$, Adjusted $R^2 = .27$), the interaction was significant ($\beta = -.139$, $t = -2.801$, $p = .005$) and accounted for a significant increase in variance over and above the other main effects ($F = 7.8452$, $p = .005$).
**Discussion:** In SZ, both higher IQ and better cognitive performance were independent predictors of better functioning. However, in HC, functionality was predicted by the interaction between IQ and cognition, with the form of the interaction suggesting that for HC participants with higher IQ, there was less effect of cognition on predicting better functioning. Conversely, in HC with lower premorbid IQ, better cognitive performance has a stronger effect in predicting better functioning. The fact that both IQ and cognition had independent relationships to functional outcome in SZ could help explain the limited clinically significant results found in previous studies of cognitive remediation and psychosocial interventions that did not also consider the impact of premorbid IQ. Future studies could focus on early interventions to prevent functional impairments through the stimulation of early intellectual development.

### T69. PERFORMANCE OF PATIENTS WITH NMDAR-AB ENCEPHALITIS COMPARED TO PATIENTS WITH SCHIZOPHRENIA AND HEALTHY CONTROLS USING FUNCTIONAL MAGNETIC RESONANCE IMAGING (F-MRI) AND A WORKING MEMORY PARADIGM

Eric Kelleher*,[1], David Mothersill[2], April Hargreaves[3], Angela Vincent[4], Gary Donohoe[2], Aiden Corvin[3]
*[1]Trinity Centre for Health Sciences; [2]National University of Ireland; [3]Trinity College Dublin; [4]University of Oxford*

**Background:** N-methyl-D-aspartate receptor (NMDAR) encephalitis has prominent early psychotic features. It has been rarely misdiagnosed as schizophrenia. NMDAR encephalitis causes deficiencies in working memory in the short & long term. Early immunotherapy/tumour removal improves outcomes. Schizophrenia can cause significant cognitive performance deficits with working memory often severely affected.
Clinical MRI in NMDAR encephalitis is usually normal, despite severe clinical course. Resting state fMRI studies have shown reduced functional connectivity of the left & right hippocampus. To date, to the best of our knowledge, functional neuroimaging utilising a working memory task such as the 1-back task (participant has to identify the number onscreen immediately before the number currently on screen) has not been utilised in NMDAR encephalitis. Potentially this may highlight alterations in brain regions through altered neuronal activity underlying working memory.
We hypothesised that patients with NMDAR encephalitis would demonstrate a difference in BOLD response (a proxy for neuronal activity) compared to healthy controls during fMRI while performing the 1-back task. Additionally, we wished to compare task evoked BOLD response in patients with NMDAR-Ab encephalitis compared to patients with schizophrenia.
**Methods:** Using G*Power 3.1 software with effect size f (0.8), total sample size was calculated from data obtained from a previous study on working memory performance in NMDAR encephalitis for the 3 groups (NMDAR encephalitis, healthy controls & schizophrenia) (N=30). Following ethical approval, 12 individuals diagnosed with NMDAR encephalitis were recruited from neurological centres. Participants were trained in the 1-back task prior to scanning. Results were compared to previously collected data from cohorts of healthy controls (N=14) and schizophrenia (N=14). Cohorts were matched insofar as possible. Data was analysed using SPSS and ART toolbox.
**Results:** In the NMDAR encephalitis (N=12) cohort, gender ratio F:M was (11:1), mean(s.d.) age 37.5(12.2) years. 50% identified ongoing memory problems. Schizophrenia cohort (N=14) gender ratio (7:7), mean (s.d.) age 39 (12.3) years. Healthy controls gender ratio (10.4) mean (s.d.) age 29.8 (6.5) years. There was no difference for gender, age or educational level between cohorts. FSIQ was higher ($p<0.01$) in controls & NMDAR encephalitis compared to schizophrenia cohorts. NMDAR encephalitis cohort had acute symptoms [mean (s.d.)] for 7.09 (2.43) weeks until treatment. 9 individuals were still on immunotherapy at time of entry.Duration since diagnosis to entry into this study was 55.18(23.13) months for NMDAR encephalitis and 12.6 (10.91) years for schizophrenia. There were no significant differences in number of ART motion outliers ($p >0.05$) between cohorts. Age & gender were added to second level analyses as covariates.
One-way ANOVA was used to compare N-back accuracy & reaction time. There was a significant difference ($p<0.01$) between groups on accuracy but not reaction time, with only the schizophrenia group showing lower accuracy.1-back working memory condition was associated with significantly increased BOLD response across several brain regions that play a role in working memory, including the dorsolateral prefrontal cortex. However, there were no significant main effects on BOLD response for the 1-back task for any cohort.
**Discussion:** Patients with NMDAR encephalitis report ongoing memory difficulties. However, in this study, there were no identifiable deficits compared to healthy controls using fMRI. This may relate to regeneration of NMDA receptors, the relatively long time until from diagnosis to follow up and early treatment with immunotherapy.

### T70. RECOVERY IN FIRST –EPISODE PSYCHOSIS: COGNITIVE, CLINICAL AND RESILIENCE (PERSONAL RESOURCES) TRAJECTORIES ACROSS 6 YEARS

Anne-Kari Torgalsboen*,[1], Christine Mohn[2]
*[1]University of Oslo; [2]Vestre Viken Hospital Trust*

**Background:** There is increasing recognition that people with schizophrenia can experience recovery. Based on a recent review of long-term outcome studies of first-episode psychosis (FEP), the authors argued that remission and recovery rates may be more favourable than previously thought.[1] In order to improve timing of interventions and personalize treatment, it must be determined when improvement take place and how cognitive, clinical and resilience trajectories develop over time.
The Oslo Schizophrenia Recovery study is one of very few long-term prospective studies of FEP investigating the rate of recovery and the longitudinal course of cognitive, clinical and personal resources in the same study. Limitations of previous studies include high attrition and samples consisting of relapsing patients most often seen in inpatient settings. In the present study, fully recovered subjects no longer in treatment have not been lost to follow- up.
The present study has a multi-follow-up design, is ongoing, and includes data from eight assessment points across six years. Thus, it is possible to assess sustained remission and full recovery as well as to show trajectories of neurocognition, symptoms and personal resources in the long term.
**Methods:** 28 (17 men, 11 women, mean age 21.0, SD 2.6 years) individuals with first-episode schizophrenia and receiving a combination of medication, case management and cognitive therapy are assessed with the Positive and Negative Syndrome Scale (PANSS), the MATRICS Consensus Cognitive Battery (MCCB) and measures of self-efficacy, hope and resilience at each assessment point. Repeated measures ANOVAs were conducted.
**Results:** At 6-year follow-up 45.5 % fulfilled criteria for full recovery, i.e. sustained improvement in both symptoms and social/vocational functioning for two years or longer. The retention rate is high (79%). As expected, there were statistically significant reductions in both positive and negative symptoms from baseline, with stabilization from 2 years to 6 years follow-up: F 21.77, p .000, $\eta 2$ .53 and F 46.60, p .000, $\eta 2$ .71. The same significant reduction across 6 years was shown for general psychopathology (F 31.36, p .000, $\eta 2$ .62.) There were statistically significant increases in overall neurocognitive test results from baseline, with stabilization from 2 years to 6 years follow-up: F 11.25, p .000, $\eta 2$ .35.
A significant increase in reported hope was found from baseline with stabilization from 6 months to 6 years follow-up: F 5.53, p .000, $\eta 2$ .21. The trajectory of general self-efficacy followed almost the same pattern as hope: There were statistically significant increases in reported self-efficacy from baseline, with stabilization from 1 year to 6 years follow-up: F 3.37, p .002, $\eta 2$ .14. Finally, resilience also increased significantly from baseline, with stabilization from 6 months to 6 years follow-up: F 5.12, p .000, $\eta 2$ .21.
**Discussion:** The recovery rate in this sample is higher (45.5%) compared to other studies. Modern treatment, as well as keeping the fully recovered in the study, may all contribute to this higher rate of recovery. The results indicate that the greatest improvement happens during the two first years of treatment and thus seems to represent a “window of opportunity” for recovery. Increases in reported self-efficacy, hope and resilience were observed 1., 5 years before improvements in neurocognition and clinical symptoms, underlining the importance of focusing on personal resources and patients’ hope of recovery early in treatment with the ultimate aim of increasing the rate of recovery in FEP.

**References:**
1. Lally, J., et al. Remission and recovery from first-episode psychosis in adults: systematic review and meta–analysis of long –term outcome. British Journal of Psychiatry, 2017.

### T71. CHANGE AND STABILITY IN COGNITIVE TRAJECTORIES FROM CHILDHOOD TO LATE ADOLESCENCE IN YOUNG OFFSPRING AT GENETIC RISK OF SCHIZOPHRENIA AND MOOD DISORDER: IMPLICATIONS FOR THE RISK STATUS

Elsa Gilbert*[,1], Thomas Paccalet[2], Valérie Jomphe[3], Daphné Lussier[3], Michel Maziade[4]
*[1]Université Laval; [2]Université de Québec; [3]CERVO Brain Research Centre; [4]Université Laval, CERVO Brain Research Centre*

**Background:** Cognitive impairments are a core feature of schizophrenia (SZ).[1] The few existing retrospective or prospective population-based studies indicate that patients who develop psychoses have cognitive impairments in childhood and adolescence.[2,3] Offspring at high genetic risk also present neurocognitive impairments before the age of disease incidence.[4,5] The form of the childhood cognitive trajectory may be a predictor of transition to illness but more data are needed on the early cognitive trajectories in children at genetic risk to inform about the most sensitive periods in risk progression to psychosis and to orient interventions.[6] The objective was to investigate the cognitive trajectories in children born to a parent affected by a SZ or BP from early childhood to late adolescence, in terms of changes in cognitive 4 domains known to be impaired in major psychosis. A special attention was given to the timing of changes in childhood, and their association with well documented clinical risk indicators.
**Methods:** The sample consisted of 79 offspring (age from 6 to 21) born to parents affected by SZ or BP from our multi-affected kindreds of Eastern Quebec. Our cognitive battery covered: episodic memory, working memory, speed of processing and executive functioning evaluated at two-time points (mean duration between assessments +/- 6y). A Cognitive domain was considered impaired when mean performance was below -1 SD. We had measurements of established childhood risk indicators [4]: psychotic-like experiences, non-psychotic DSM diagnoses and social functioning (GAF).
**Results:** Three distinct developmental trajectories were identified according to the progression in number of impaired cognitive domains from baseline to follow-up: i) A “steady” trajectory with stable and intact performances across all cognitive domains (n=52; 66%); ii) a “deteriorating” trajectory with an accumulation of cognitive impairments (n=18; 23%) and; iii) an “improving” trajectory with a diminishing number of cognitive impairments at follow-up (n=9; 11%). IQ and neuropsychological performances were similar at baseline between the “deteriorating” and “improving” trajectories (p=.4), while the steady group performed best. The 3 subgroups were comparable in terms of the parent diagnosis and offspring gender. Regarding clinical risk indicators, the deteriorating subgroup presented a worsening of social functioning between the two-time points (-7 GAF points vs -0.8 for steady, +0.56 for improving) and a higher rate of childhood non-psychotic DSM diagnosis ($p \leq .01$). Importantly, we observed striking differences in cognitive trajectories among siblings suggesting that change or stability go beyond the heritability of cognitive capacities.
**Discussion:** Our results suggest three types of cognitive developmental trajectories among offspring of parents affected by SZ or BP. The progressive deteriorating trajectory was associated with an aggregation of other clinical risk indicators shown to predict transition.[4] Cognitive deterioration was slightly more frequent in childhood and pre-adolescence than in late adolescence which has implications for the timing of detection, the need of care, the type of longitudinal surveillance and the design of future prevention research.[6]

**References:**
1. Keefe & Kahn, JAMA Psychiatry, 2017
2. Meier et al., Am J Psychiatry, 2014
3. MacCabe et al., JAMA Psychiatry 2013
4. Paccalet et al., Schizophr Res, 2016
5. Maziade et al., Schizophr Bull, 2011
6. Maziade, N Eng J Med, 2017

### T72. VERBAL MEMORY AND VOXEL BASED MORPHOMETRY IN FIRST EPISODE NON-AFFECTIVE PSYCHOSIS: A PROCESS ORIENTED APPROACH

Rosa Ayesa-Arriola*[,1], Esther Setien-Suero[2], Diana Tordesillas-Gutierrez[2], Benedicto Crespo-Facorro[2]
*[1]Marqués de Valdecilla University Hospital, IDIVAL, University of Cantabria; [2]Marques de Valdecilla University Hospital, IDIVAL, University of Cantabria, CIBERSAM*

**Background:** Deficits in auditory-verbal memory have been reported by the vast majority of published research in schizophrenia and also detected in first episode psychosis (FEP), confirming they are already present at the early stages of the illness. However, the specific neurocognitive constructs underlying defective verbal memory and their neuroanatomical correlates remains poorly understood in schizophrenia spectrum disorders patients.
**Methods:** Data on the Rey Auditory Verbal Learning Test (RAVLT), a widely used verbal memory measure that provides a range of performance indexes to evaluate distinct memory processes including: a) acquisition/learning b) sensitivity to interference c) retrieval; d) retention or rate of forgetting; e) and retrieval efficiency was available for 388 FEP patients and 184 healthy controls (HC). In 218 FEP patients and 146 HC, structural magnetic resonance imaging data were analysed using Voxel based morphometry (VBM) toolbox.
**Results:** The FEP group showed significantly lower results on acquisition/learning, delayed recall as well as higher rates of forgetting. They also exhibited a significant sensitivity to retroactive but not proactive interference. We also found significant correlations between bilateral frontal lobe morphometry and proactive interference as well as between right frontal lobe morphometry and retroactive interference. Rate of forgetting was significantly correlated with right occipital cortex morphometry. Those with higher rates of forgetting and proactive and retroactive interference demonstrated further gray matter reductions in frontal and occipital cortical areas.
**Discussion:** The application of a process oriented approach to the neuropsychological evaluation of verbal memory allows a finer-grained analysis of the neurocognitive constructs underlying defective verbal memory in FEP. The results suggest specific relationships between different neuroanatomical structures and discrete memory processes with these structures playing an important role in verbal memory deficits found in FEP.

### T73. NOVEL VISUAL TRAINING FOR COGNITIVE REMEDIATION IN SCHIZOPHRENIA

Toral Surti*[,1], Dayshalis Ofray[2], Bruce Wexler[2]
*[1]Yale University School of Medicine, VA Connecticut Healthcare System; [2]Yale University School of Medicine*

**Background:** Though cognitive remediation has shown modest benefits for schizophrenia, relatively few training programs for have targeted the visual processing deficits common in the illness. Residual visual processing deficits following cognitive remediation may explain why cognitive remediation has had limited effects on visual learning and memory compared to auditory learning and memory. We sought to test whether training early visual processing would improve visual memory and facial affect recognition by targeting a well-characterized visual deficit in schizophrenia: visual backward masking (VBM). The deficit is so common in schizophrenia, and in non-affected family members to a lesser degree, that it is viewed as an endophenotype. The VBM deficit, however, can normalize as we have previously shown. In our prior open-label pilot study, individuals with schizophrenia (Surti and Wexler, 2012) made substantial gains in VBM performance with rudimentary computerized VBM training. The VBM improvements were also accompanied by improvements in visual memory.
To test the hypothesis that improved early sensory training could lead to other cognitive gains in the same domain, we conducted a randomized control study with a new, more sophisticated computerized VBM training program, and compared it to an active control condition. We expected that visual memory and facial affect recognition would improve with the novel visual training (VT), and that VBM would improve with the VT as well.
**Methods:** 23 individuals with stable schizophrenia or schizoaffective disorder were randomized to receive 20 sessions of VT or an active control. The VT consisted of VBM training with multiple levels of difficulty, adaptive tracking, virtual rewards, and a variety of letters, numbers, and shapes to train different areas of the visual field. The active control condition was a commercially available computerized typing tutorial (TT) with animation, game narrative, and multiple typing activities. Participants were tested before and after training with: the Matrics Cognitive Consensus Battery (MCCB), including the Brief Visuospatial Memory Test-Revised (BVMT) as the study's primary outcome; the Profile of Nonverbal Sensitivity (mini-PONS) to assess non-verbal social cues; standardized VBM tests; and typing assessments. Repeated measure ANOVAs were conducted in SPSS24 after checking for normality.
**Results:** 22 of 23 individuals completed the study, and by participants' reports, both interventions were well tolerated, equally enjoyable and equally motivating, though the VT was slightly more frustrating for participants. Even when co-varying for education, which was higher in the VT group, there were no condition by time interactions for the BVMT, the mini-PONS, overall MCCB, or typing ability. There was a significant condition by time interactions for VBM performance ($F = 5.8$, $p =0.028$), with a substantial improvement in the VT group (Cohen's $d = 0.54$; $p=0.004$).
**Discussion:** Patients with schizophrenia equally tolerated a computerized visual training designed in-house and an off-the shelf highly gamified control training, but only the visual training, specifically designed for individuals with schizophrenia, had effects on the trained task. The effects of the visual training did not generalize to visual memory, facial affect recognition, or global cognition, so further work is needed to facilitate generalization.

### T74. ACADEMIC ACHIEVEMENT AND SCHIZOPHRENIA: A META-ANALYSIS

Hannah Dickson*[,1], Alexis Cullen[1], Sheilagh Hodgins[2], James MacCabe[1], Kristin Laurens[3]
*[1]Institute of Psychiatry, Psychology & Neuroscience, King's College London; [2]University of Montreal; [3]Australian Catholic University*

**Background:** The extent to which poor academic achievement is associated with later schizophrenia is unclear. The aim of the present study was to update our prior meta-analyses which examined academic achievement in youth aged 16 years or younger who later developed schizophrenia or schizophrenia spectrum disorders (SSD) and those who did not (Dickson et al, 2012, Psychological Medicine, 42, 743–755). We also conducted a new meta-analysis on published studies that reported on general academic achievement in youth at-risk for schizophrenia/SSD aged 16 years or younger compared to typically developing youth
**Methods:** In addition to the five studies included in our earlier meta-analyses, a further three prospective investigations of birth or genetic high-risk cohorts were identified that reported results using objective measures of general academic achievement and of mathematics achievement for individuals who did and did not develop schizophrenia/SSD in adulthood. For our new meta-analysis we identified a total of seven studies that met the following inclusion criteria: (1) written in English; (2) objective measure of general academic achievement consisting of scores on least two core academic subjects (i.e., literacy and mathematics) at age 16 years or younger; (3) results provided for youth at high risk for developing schizophrenia/SSD in adulthood by virtue of having at least one first-degree relative with the disorder or reporting psychotic like-experiences (PLEs); and (4) sufficient data to calculate effect sizes.
**Results:** Meta-analyses showed that by age 16 years, individuals who later developed schizophrenia/SSD presented with significantly poorer general academic achievement ($d=-0.26$) and mathematics achievement ($d=-0.21$). Findings also indicated that during adolescence, youth with a family history of schizophrenia/SSD and youth reporting PLES were characterised by significantly lower general academic achievement than healthy peers ($d=-0.39$; $d=-0.53$, respectively).
**Discussion:** These results show that poor academic achievement precedes illness onset, and may represent an easily identifiable non-specific marker of biological, psychological and social risk processes underpinning the development of schizophrenia/SSD.

### T75. GENERAL AND EXECUTIVE COGNITIVE PROFILES: GENERAL COGNITIONS INFLUENCE ON WCST PERFORMANCE

Sean Carruthers*[,1], Caroline Gurvich[2], Philip Sumner[3], Eric Tan[4], Elizabeth Thomas[2], Susan Rossell[1]
*[1]Swinburne University; [2]The Alfred Hospital, Monash University; [3]Swinburne University, The Alfred Hospital; [4]The Alfred Hospital, Monash University, Swinburne University*

**Background:** Executive functions (EF) have been conceptualized as a set of higher-level control processes that enable an individual to adapt to diverse situations, inhibit inappropriate responses, formulate, initiate and persevere plans and mediate the organisation of goal-directed thoughts and actions and are commonly reported as being compromised in schizophrenia. Complex measures designed to assess EF suffer from task impurity, activating and reporting on the performance of non-EF processes. The present study examined the potential contribution discrete cognitive subtypes might have on performance on the Wisconsin Card Sorting Test
**Methods:** Ward's method hierarchical cluster analysis was performed on the MATRICS Consensus Cognitive Battery (MCCB) and the Wisconsin Card Sorting Test (WCST) collected from 105 healthy controls and 100 patients with schizophrenia/schizoaffective disorder.
**Results:** Two cognitive profiles were identified for general cognition (High/Low) and the WCST (High/Low). For controls, only 53% of low performing participants performed low on the WCST. For patients, 73.5% of patients who performed poorly on the MCCB were found to perform poorly on the WCST.
**Discussion:** Results indicate that the contribution of general cognitive domains on poor WCST performance differs between patients and control participants. For patients, there appears to be a substantial contribution of impaired general cognition to performance the WCST.

### T76. INVESTIGATION OF NAV1.1 POSITIVE MODULATOR EFFECTS ON FAST SPIKING INTERNEURONS IN SOMATOSENSORY CORTEX SLICES

Piszar Ildiko[1], Just Stefan[1], Draheim Henning*[,1]
*[1]Boehringer Ingelheim*

**Background:** GABAergic inhibition is essential for normal cortical function as it serves the purpose of proper excitation/inhibition (E/I) balance in many circuits. In contrast, inappropriate interneuron signaling leads to E/I imbalance, reduced gamma oscillations in EEG measurements and has serious behavioural consequences. Among the heterogeneous group of GABAergic cells, fast-spiking, parvalbumin positive interneurons (FS-PV+) play a key role in the generation and maintenance of gamma oscillations. E/I imbalance due to interneuron dysfunction has been implicated in the pathophysiology of various psychiatric disorders.
Cognitive impairment has been associated with altered gamma oscillation in schizophrenia and there is accumulating evidence for involvement of FS-PV+ interneuron deficit in the disease. Hypofunction of FS-PV+ neurons leads to disinhibition of pyramidal cells which cause network desynchronization. Therefore, it is hypothesized that activation of these neurons could restore high-frequency oscillations and consequently improve cognitive functions. However selective modulation of different interneuron types is still challenging due to limited number of known cell type specific targets.
**Methods:** A possible starting point for the treatment could be pharmacological activation of voltage gated sodium channels (Nav) which have a pivotal role in action potential initiation. Of the four subtypes of Nav channels expressed in the CNS Nav 1.1 comprises the majority of the sodium current in FS-PV+ but not in pyramidal neurons. Based on this we looked for selective Nav 1.1 activators and found a recently published promising compound (Compound 3a, see Crestey et al, 2015) which has been shown to increase the electrical activity of FS-PV+ interneurons in the CA1 area of the hippocampus. However, the dysfunction in information processing found in schizophrenic patients is not only restricted to the hippocampus and high-order association cortices but also influences the sensory cortex. Thus, our aim was to explore the effect of the selective Nav 1.1 positive modulator Compound 3a on FS interneurons in the mouse somatosensory cortex. We performed whole-cell patch clamp recordings from mouse cortical brain slices and recorded the electrical activity of single FS cells before and after the drug application.
**Results:** Surprisingly the excitatory effect of the compound 3a could only partly be confirmed in the way that positive modulation of Nav1.1 in terms of action potential number and threshold only takes place under particular conditions, i.e. at physiological temperature and under specific ion compositions of the recording solutions
**Discussion:** The discrepancy of our results from published data might be attributed to the different experimental conditions such as recording temperature and ionic composition of solutions and highlight the importance of selecting near physiological conditions during brain slice patch clamp experiments.

### T77. DIAGNOSTIC AND NEUROCOGNITIVE CORRELATES OF SCHIZOTYPY WITHIN AND ACROSS THE PRONIA STUDY GROUPS

Carolina Bonivento*[,1], Maria Fernanda Urquijo[2], Stefan Borgwardt[3], Eva Meisenzahl[4], Marlene Rosen[5], Raimo Salokangas[6], Rachel Upthegrove[7], Stephen Wood[8], Nikolaos Koutsouleris[2], Paolo Brambilla[9]
*[1] University of Udine; [2]Ludwig-Maximilian-University; [3]University of Basel; [4]Heinrich-Heine University Düsseldorf; [5]University of Cologne; [6]University of Turku; [7]University of Birmingham; [8]The University of Melbourne; [9]University of Milan,*

**Background:** Schizotypy traits range from odd behaviors to symptoms that resemble full schizophrenia, although less severe. Previous studies associated different degrees of positive and negative schizotypal traits to variations in the persons' cognitive profiles while others related them to the risk to develop psychosis.
We hypothesize that similar pattern of positive and negative schyzoypy traits characterize individuals at risk of psychosis and patients meeting the criteria for recent onset psychosis, although with different degrees of severity. Also, both should differ from depressed patients. Moreover, specific combinations of schizotypy traits and neurocognitive alterations should be associated to the different psychopathological profiles. The final goal of the study is to identify candidate predictors of risk of psychosis that will be used as features in next machine learning analyses.
**Methods:** The present is a multi-centric study that was conducted as part of the project titled 'Personalised Prognostic Tools for Early Psychosis Management' (PRONIA).
115 participants at high-risk for psychosis (CHR), 114 recent onset psychosis (ROP), 123 recent onset depression (ROD) and 252 healthy controls (HC) took part in the study.
All were aged between 15 and 40 years.
The participants filled the Wisconsin Schizotypy questionnaire, measuring positive (Magical Ideation Scale - MIS; Perceptual Aberration Scale - PAS) and negative schizotypy traits (Social Anhedonia Scale - SAnS; Physical Anhedonia Scale - PAnS).
Moreover, they were administered the PRONIA Cognitive Battery (PCB), comprising measures of visuo-spatial dexterity and memory (Rey Figure, copy and delayed drawing), short-term memory (Digit Span - DS), Verbal Learning, Verbal Fluency, Attention (Continuous Performance Test - CPT, Digit Symbol Substitution Test - DSST), Emotions' Recognition, General Intelligence (WAIS Vocabulary, Matrix Reasoning).
**Results:** We run i) a Multivariate Analysis of Covariance with 'WSS subscales' as dependent variable; 'Group' as between subject factor; 'Age' and 'Gender' as covariates; ii) a Multinomial logistic regression with 'Group' as dependent variable; HC 'Group' as reference parameter; 'WSS subscales' and scores at the PCB's tests as predictors; 'Age' and 'Gender' as covariates. ROP and CHR reported both positive and negative schizotypy traits, although only the negative symptoms involving social aspects were clearly evident in CHR. Also, ROP and CHR differed for the positive symptoms, as they were present but at a lower level in CHR than in ROP. ROD instead scored high at the negative symptoms. Interestingly, ROP, CHR and ROD did not differ between each other for the negative symptoms, probably reflecting the effect of the psychopathology on the patients' general motivation to life.
The regressions analysis highlighted different patterns of associations of WSS and neurocognitive scores with the clinical status. In particular, the scores at the MIS, PAS and SanS combined with the Rey Figure (delayed drawing), predicted that the participants were CHR; the MIS, PAS and SAnS with measures of attention (CPT, DSST) predicted that the participant were ROP; the PAS; SAnS and short-term memory (DS) predicted to being ROD.
**Discussion:** Coherently with the hypotheses, different schizotypy traits or grade of severity characterized patients with distinct psychopathology profiles. Also, the association of WSS subscales with the cognitive measures differentiated between groups, with visuo-spatial long-term memory being associated to CHR, measures of attention relating to ROP and verbal short term memory relating to ROD.
This makes these measures good candidates for the upcoming machine learning analyses.

### T78. LONG-TERM PROGNOSIS OF SCHIZOPHRENIA - RESULTS FROM THE NORTHERN FINLAND BIRTH COHORT 1966

Erika Jääskeläinen[1], Matti Penttilä[1], Jani Moilanen[1], Marianne Haapea[1], Anja Hulkko[1], Irina Rannikko[1], Nina Rautio[1], Tanja Nordström[1], Juha Käkelä[1], Annika Seppälä[1], Johanna Immonen[1], Hanna Korpela[1], Jussi Seppälä[1], Graham Murray[2], Hannu Koponen[3], Matti Isohanni*[,1], Jouko Miettunen[1]
*[1]University of Oulu; [2]University of Cambridge; [3]University of Helsinki, Helsinki University Hospital*

**Background:** The aim of this study was to explore the prognosis and predictors of outcomes in schizophrenia in a birth cohort sample followed since mid-pregnancy until the age of 45 years.
**Methods:** The sample included subjects with schizophrenia (n=29–161, depending on the analyzed topic) from the Northern Finland Birth Cohort 1966. Outcomes and their predictors were analyzed by utilizing national registers, questionnaires and personal examinations made on several time points (e.g. during pregnancy, at age 1 year, 34- and 43- years). Functioning, amount of psychiatric symptoms, utilization of treatments, physical illnesses and mortality, and cognition were used as measures of outcomes. Several plausible factors associating to outcomes were studied, e.g. gender, family history of psychosis, development and childhood related factors, school performance, and illness related factors around the onset of psychosis, brain morphology and cognitive functioning, and lifetime antipsychotic medication.
**Results:** Around the age of 34-years recovery was possible though quite uncommon (3.4%), some persons achieved symptomatic remission (21%), and many were on disability pension (54%). Around the age of 43–45 years only 11.2% were employed, and 19% were in remission. Earlier age of illness onset, longer duration of untreated psychosis, suicidal ideation and poorer functioning around illness onset, brain morphological changes and poorer cognition, and higher lifetime doses of antipsychotics associated to poor outcomes. Cognition did not markedly decline from 34 to 43 years of age, but poorer premorbid school performance and higher lifetime doses of antipsychotics predicted more decline of cognition. For some cases, the cumulative amount of used antipsychotics was extensive. Somatic comorbidities were common, and mortality high.
**Discussion:** Based on this naturalistic sample, progression of schizophrenia may follow a variety of different trajectories. Poor clinical course is common but not necessary outcome. Our results indicate heterogeneous and still relatively unsatisfactory prognosis of schizophrenia in this sample. Several predictors of outcomes have been found, and especially factors related to illness onset and high lifetime cumulative dose of antipsychotics are of interest. Birth cohort setting offers unique possibility to study long-term prognosis of schizophrenia.

### T79. AFFECTIVE FACE PROCESSING IN SCHIZOPHRENIA: DISORDER-SPECIFIC OR TRANSDIAGNOSTIC DEFICIT?

Jack Cotter*[,1], Kiri Granger[1], Jennifer Barnett[1]
[1]*Cambridge Cognition*

**Background:** Social cognitive dysfunction is common in patients with schizophrenia and is associated with marked and persistent functional disability. Facial emotion recognition is a core aspect of social cognition and has been consistently demonstrated to be impaired in this population. However, it remains unclear whether these deficits are unique to patients with schizophrenia. We compared the severity of facial emotion recognition deficits in patients with both sub- and full-threshold psychotic symptoms to those observed across a range of psychiatric, neurological and developmental disorders in order to determine to what extent this represents a disorder-specific or transdiagnostic aspect of cognitive dysfunction.
**Methods:** We conducted an electronic database search in order to identify published, peer-reviewed meta-analyses that compared facial emotion recognition task performance between individuals meeting clinical criteria for a psychiatric, neurological or developmental condition against healthy controls. Facial emotion recognition standardized mean difference effect size estimates (Cohen's d or Hedges' g) were required to have been derived from tasks in which participants had to identify, label or match images of faces consisting of all or any combination of the six basic emotions (happiness, sadness, anger, fear, surprise or disgust). Where possible, a 'total' score was used, comprising performance across multiple emotions. Effect size estimates must have been derived from two or more independent studies in order for the meta-analysis to be included. Where there were multiple publications for a given medical condition that met our inclusion criteria, we included the most recently published paper.
**Results:** We identified 19 meta-analyses eligible for inclusion that examined performance across relevant tasks among 24 different clinical populations. Though the effect sizes are not directly comparable across clinical conditions (due to methodological differences between studies and in meta-analytic procedures), they demonstrate consistent and statistically significant deficits in facial emotion recognition across almost all of the clinical groups included in this review. Effect size estimates indicated that deficits among patients with schizophrenia were among the largest and most robust. Deficits were also evident even among those individuals with sub-threshold psychotic symptoms who met clinical criteria for being at ultra-high risk of developing a psychotic disorder.
**Discussion:** Facial emotion recognition deficits are a transdiagnostic issue, potentially serving as a biomarker of neurological abnormality. However, these impairments appear to be particularly severe and debilitating among people with schizophrenia. There are currently no recognized treatments for these deficits. This in part is due to a lack of outcome measures suitable for use in clinical trials. Improved characterization and operationalization of social cognition and other 'hot' cognitive processes are necessary to facilitate and advance treatment efforts, both in schizophrenia and across other clinical groups. We are currently in the process of developing and acquiring normative data for a series of computerized tasks which can be used to assess these domains. This includes new variants of established tests which have been used to assess facial emotion recognition, as well as novel tasks to detect emotional biases and assess responses to socially-relevant information. These tasks will help to facilitate further research into these complex social processes and potentially assist in the development of interventions for those patients that are adversely affected.

### T80. CALCIUM AND POTASSIUM VOLTAGE-GATED CHANNELS GENES ASSOCIATION ANALYSIS: EVIDENCE ON THEIR ROLE IN COGNITIVE PERFORMANCE OF SCHIZOPHRENIA PATIENTS AND HEALTHY SUBJECTS

Maria Guardiola*[,1], Carmen Almodóvar[1], Alba Lubeiro Juárez[2], Silvia Alonso-Lana[4], Jordi Ortiz-Gil[5], Amalia Guerrero-Pedraza[6], Salvador Sarró[4], Edith Pomarol-Clotet[4], Mar Fatjó-Vilas[7]
[1]*University of Barcelona;* [2]*School of Medicine, University of Valladolid;* [4]*FIDMAG Research Foundation, CIBERSAM;* [5]*FIDMAG Research Foundation, Hospital General de Granollers;* [6]*Benito Menni - CASM, Sant Boi de Llobregat;* [7]*FIDMAG Research Foundation, CIBERSAM, University of Barcelona*

**Background:** Cognitive deficits are considered core features of schizophrenia (SZ) (Green & Harvey 2014). Subtle variations in the perfectly coupled mechanism that maintains the potential of membrane in neurons may have repercussion on neuronal processing. Therefore, genetic variability related to the functioning of excitable cells and linked to pathways essential for neuronal survival and plasticity may underlie the observed differences in cognitive abilities (Carr et al 2016). CACNA1C and KCNH2 genes encode for calcium and potassium voltage-gated channels, ultimately related to neuronal functioning (Dolmetsch et al 2001, Schwarz et al 2004). These two genes have been previously related with SZ (Atalar et al 2010, Ripke et al 2014). The aim of this study was to evaluate whether the genetic variability of CACNA1C and KCNH2 is associated with: i) the risk for schizophrenia, ii) the cognitive performance of SZ patients and healthy subjects.
**Methods:** Our sample consisted of 348 SZ patients and 387 unrelated healthy controls (HC). DNA was extracted from blood/saliva samples using standard procedures and two Single Nucleotide Polymorphisms (SNPs)

were genotyped: rs1006737 (G/A) in CACNA1C gene, rs3800779 (G/A) in KCNH2. A subsample (296 SZ/157 HC) underwent neurocognitive assessment, which included: i) premorbid IQ (Word Accentuation Test - Test de Acentuación de Palabras (TAP)); ii) memory (Wechsler Memory Scale (WMS-III)) and, iii) executive function (Behavioural Assessment of the Dysexecutive Syndrome (BADS)). The association between the SNPs and neurocognitive performance was explored (adjusted by sex and age) separately in patients and in controls groups.

**Results:** In our sample, we did not detect an association of CACNA1C and KCNH2 with the risk for SZ. Patients performed significantly worse than controls in all cognitive measures (p<0.005). SZ patients homozygous for the risk allele (A) of the CACNA1C polymorphism showed lower premorbid IQ (TAP scores) than patients carriers of the C allele (rs1006737: B=-1.39 p=0.027). Within HC, the minor allele (A) of KCNH2 was associated with WMS global score (rs3800779: B=3.01 p=0.010): subjects carrying the AA genotype presented better memory performance.

**Discussion:** Our findings add evidence on the role of CACNA1C and KCNH2 on modulating cognitive performance in SZ patients and HC (Huffaker et al 2009, Krug et al 2010, Zhang et al 2012, Hashimoto et al 2013). Our results in patients are in line with previous studies that suggest an association of CACNA1C risk allele on different cognitive domains. As regards to KCNH2, our results are opposite in terms of the direction of the effect observed in previous studies, probably as a consequence of the sample size and heterogeneity in methods used to assess memory. The different direction of the genetic effects among patients and controls reflects the complex relationship between genetic factors and cognitive performance variability. It is suggested that genes that enhance cognitive abilities under normal circumstances turn to be pernicious under the modulation effect of a disease (Crespi et al 2007). Further research is needed and we expect to extend the present results with neuroimaging genetics approaches.

**Acknowledgements:** Spanish Ministry of Economy and Competitivity, Instituto de Salud Carlos III (PI15/01420 and PI15/00299), Ayuda cofinanciada por el Fondo Europeo de Desarrollo Regional (FEDER) "Una manera de hacer Europa".

### T81. LONG-TERM COURSE OF COGNITIVE PERFORMANCE IN PATIENTS WITH CHRONIC SCHIZOPHRENIA

Thaís Martins*,[1], Thalita Fernandes[1], Diego Mendes[1], Gustavo Mustafé[1], Luis Fernando Pegoraro[1], Clarissa Dantas[1]
[1]*Univeristy of Campinas*

**Background:** Cognitive deficits are prevalent among patients with schizophrenia and are robustly associated with functioning and outcome. Although cognitive deficits are known to be present at the prodromal phase and to worsen at the onset of the disease, the long-term course of cognitive impairments are less well established. Many studies have focused on first episode psychosis with relatively short lengths of follow-up. Thus, the aim of this study is to investigate changes in cognitive performance of chronic schizophrenia patients in a variety of neurocognitive tests over a seven-year test-retest period.

**Methods:** We will contact 85 patients with schizophrenia (as defined by the DSM-IV-TR), considered clinically stable in the previous year, who participated in a study about the deficit syndrome of schizophrenia carried out in 2009 and 2010. Back then, they were recruited in two sites: an outpatient psychiatric service of a university general hospital (49 patients) and a community-based mental health service for patients with severe mental illness (36 patients), both in Campinas, Sao Paulo, Brazil. Patients will be assessed with the same instruments adopted in the first study: a questionnaire for clinical and demographic information; SAPS, SANS, Calgary Depression Scale and a battery of neurocognitive tests comprising: Digit Span Forward (DSF), Digit Span Backward (DSB), Rey Complex Figure Copy (RCFC), Rey Complex Figure Memory (RCFM), Digit symbol-coding (DSC), Picture Completion (PC), Matrix Reasoning (MR), Vocabulary (V), Trail Test A (TTA), Trail Test B (TTB), Phonological Fluency (PF) and Semantic Fluency (SF). To test differences in neurocognitive performance, and in symptoms severity at base and at follow-up we used the Wilcoxon Test.

**Results:** We present in this poster partial results. Among the 20 reassessed patients the mean age at baseline was 36.9 ± 8.9 years, mean duration of mental illness was 16 ± 10.1 years, 75% were men. They had, in mean, 10.7 ± 3.3 years of education, only 20% had any work activity, and 15% were married. Mean length of test-retest interval was 6.9 years (minimum 6 and maximum 7.7). At follow-up, 4 patients had improved their education, but only 3 (15%) had any work activity. Up to now 19 patients completed the cognitive reassessment. Severity of positive and of depressive symptoms was low at base line (mean score on SAPS 5.5 ± 4.8; mean score on Calgary 1.5 ± 1.9) and remained low at follow-up (SAPS 6.2 ± 4.4, Calgary 2.2 ± 2.2), with no significant change. Patients, as a group, had moderate negative symptoms were at baseline (mean SANS score 10.5 ± 6.9) and had their negative symptoms worsened at follow-up (SANS 14.8 ± 7.1), p=0.005. Patients had a worse performance at follow-up in 4 out of 12 tests: DSF (3.8 ± 1.5 at follow-up versus 10.1 ± 2.8 at baseline, p < 0.000), DSB (3.4 ± 1.9 at follow-up versus 4.3 ± 2.2, p=0.005), RCFC (14.8 ± 9.4 versus 30.2 ± 8.6, p < 0.000) and RCFM (5.9 ± 6.5 versus 13.9 ± 9.8, p < 0.000). In the remaining 8 tests: DSC, PC, MR, V, TTA, TTB, PF and SF, there were no significant differences in performance between baseline and follow-up assessments.

**Discussion:** Our preliminary results are derived from a small sample. Although we cannot draw definite conclusions, we identified different patterns of longitudinal course for different cognitive domains with attention, shot-term memory, working memory, visual-spatial ability and executive functions presenting a decline over time; whereas other domains, such as visual memory, visual perception, learning memory, verbal comprehension, motor function, remaining stable in patient through patients' 4th and 5th decades of life.

### T82. THE RELATIONSHIP BETWEEN SOURCE MONITORING DEFICITS AND NEUROPSYCHOLOGICAL FUNCTIONING IN SCHIZOPHRENIA

Martyna Krężołek*,[1], Łukasz Gawęda[2]
[1]*Medical University of Warsaw;* [2]*Medical University of Warsaw, University Medical Center Hamburg Eppendorf*

**Background:** Source monitoring (SM) is a metacognitive process involved in making judgments about the origin of memories, knowledge and beliefs. Many studies have demonstrated that people with schizophrenia perform more poorly on tasks of source monitoring when compared to non-schizophrenic. Although source of monitoring is considered as an important cognitive biases implicated in reality distortions/psychotic symptoms, the knowledge on its neurocognitive mechanisms is far from being conclusive. The main aim of our study was to investigate the relationship between SM and neuropsychological functioning in schizophrenia.

**Methods:** A total of 84 (43 females; mean age 42.01, SD=11.55) patients diagnosed with schizophrenia were assessed with neuropsychological tests, including executive functions, verbal memory, working memory, processing speed and attention. SM was assessed with an action memory task. Simple actions were presented to the participant verbally (text) or non-verbally (icons). Some actions were physically performed and others were imagined. Following the learning phase, participants were presented with each action as well as new ones, were asked whether the action was presented verbally or non-verbally (action's presentation type discrimination), and whether the action was performed or imagined (self-monitoring). A knowledge corruption for self-monitoring (proportion of high confident errors on all high confident responses) was also obtained. The symptoms severity was assessed with the PANSS. The relationship between SM biases and neuropsychological functioning was investigated with correlation analyses.
**Results:** The correlations were found between incorrect action's presentation type discrimination and the results of test such as CTT 1 (r=-0.22, p<0.05), D2 (r=-0.25, p<0.05) and Block Design (r= -0.40, p<0.01). Correlational analyses showed no relations between incorrect self-monitoring and neuropsychological functioning. Knowledge corruption for self-monitoring turned out to be correlated with WCST (r=0,22, p<0.05), CVLT (r= -0.26, p<0.05) and Backward Digit Span (r= -0.27, p<0.05). These correlations remain significant when controlled for positive symptoms severity. Incorrect self-monitoring showed a significant relation with the PANSS positive subscale (r=0.23, p<0.05). Knowledge corruption was related to PANSS disorganization subscale (r=0.25, p<0.05).
**Discussion:** In line with previous studies we found that deficits in self-monitoring are related to symptoms severity and not to neuropsychological functioning. On the other hand, deficits in action's presentation discrimination are related exclusively to neuropsychological functioning. These results suggest that the relationship between SM and neuropsychological functioning depends on the type of SM deficits. The conclusions of the study may be of clinical importance - in light of our results it might be advisable to combine cognitive remediation techniques with those interventions that focus on cognitive biases like source monitoring deficits

### T83. PROCESSING SPEED PERFORMANCE AND FUNCTIONING IN YOUNG ADOLESCENTS EXPERIENCING AUDITORY HALLUCINATIONS

Joanne Kenney*[,1], Ian Kelleher[1], Colm Healy[1], Niamh Dooley[1], Eleanor Carey[1], Amy Adair[1], Donal Campbell[1], Helen Coughlan[1], Erik O' Hanlon[1], Mary Cannon[1]
[1]*Royal College of Surgeons in Ireland*

**Background:** Neurocognitive impairments are a prevalent aspect of psychosis which, on average, begin in early adolescents, with particular impairment apparent in speed of processing and nonverbal working memory in early stages (Kelleher et al., 2012). It is important to understand the impact of cognitive impairment on functional ability, particularly in early stages of illness which may assist in the development of targeted therapeutic strategies.
**Methods:** A population sample of 212 school going adolescents aged 11–13 years partook in the study, which included community-based adolescents who report experiencing psychotic symptoms but who were not clinically diagnosed. Psychotic symptoms were assessed using the psychosis section of the Schedule for Affective Disorders and Schizophrenia. Six cognitive domains were assessed using the MATRICS consensus cognitive battery. Functioning was assessed using the Children's Global Assessment Scale. Six separate linear regression analyses were performed to test if each cognitive domain of the MATRICS battery predicted functioning.
**Results:** In the entire sample (including those who experienced psychotic experiences and those who did not) (n=211), speed of processing significantly explained 8% of the variance in functioning (F(1, 76) = 6.61, p = .0012, R-squared = 0.08.), (Beta = 0.39, p = 0.012). When the sample was subdivided into those who ever experienced auditory hallucinations (AH) (n=62) versus those that did not (n=149), speed of processing significantly predicted 18% of the variance in functioning in the group experiencing AHs (F(2, 33) = 3.82, p =0.032, R-squared = 0.18), (Beta = 0.43, p = 0.06). However, no effect was found in the group without AVs (F(1,40) = 1.19, p =0.28). No other cognitive domain predicted functioning.
**Discussion:** Speed of processing appears to be a core cognitive deficit in psychosis which impacts on functioning in young adolescents particularly in those experiencing psychotic symptoms such as auditory hallucinations, however the variance predicted by processing speed is relatively low. This research highlights the potential of speed of processing as a possible viable target for early intervention in psychotic disorders.

### T84. DO SIMILAR COGNITIVE MECHANISMS ENCOURAGE DELUSION-LIKE IDEATION AND BELIEF IN FAKE NEWS?

Michael Bronstein*[,1], Gordon Pennycook[1], Adam Bear[1], Tyrone Cannon[1], David Rand[1]
[1]*Yale University*

**Background:** Increasingly, the positive symptoms of psychosis are recognized as being on a continuum with phenomena that are experienced by many members of the general population (i.e., non-clinical samples). Delusions are no exception. These fixed false beliefs, which are common in individuals with psychosis, are echoed by inflexible false beliefs in the general population that have delusion-like qualities (e.g., belief in clairvoyance). In a series of studies, we sought to determine whether belief in a particular type of disinformation (fake news) might represent a point on the same continuum as delusions and delusion-like ideation. To this end, we examined whether individuals who endorsed more delusion-like ideation were also more prone to believing fake news. We then examined whether the cognitive mechanisms behind any relationship between delusion-like ideation and fake news were similar to those associated with delusion-like ideation generally.
**Methods:** 503 participants were recruited using Amazon's Mechanical Turk (MTurk). Participants completed a test of ability to discriminate real from fake news along with several individual difference measures. These included measures of delusion-like ideation (the Peters et al. Delusion Inventory [PDI]), engagement in analytic thinking (the Cognitive Reflection Test [CRT]), and the degree to which one values evidence in forming and revising beliefs (the Actively Open-Minded Thinking Questionnaire [AOT]). Mediation tests were conducted using the PROCESS macro for SPSS (model 4, with 5000 bootstrapped samples and bias-corrected 95% confidence intervals).
**Results:** Delusion-like ideation was positively correlated with belief in fake news (rho(501) = .20, p < .001). The relationship between belief in fake news and delusion-like ideation was partially explained by lower levels of analytic thinking ability (as measured by the CRT; completely standardized 95% CI = [.02 .07]) and lower evidence valuation (as measured by AOT scores; completely standardized 95% CI = [.01 .06]). These indirect effects accounted for 39% of the relationship between delusion-like ideation and belief in fake news. Delusion-like ideation and belief in real news were not correlated (rho(501) = 0.01, p = .927).
**Discussion:** Consistent with the notion that belief in fake news represents a point on the same continuum as belief in delusional and delusion-like ideas, belief in fake news was associated with increased endorsement of delusion-like ideation. This relationship was partially explained by factors previously associated with delusions and delusion-like ideation (e.g., lower engagement in analytic thinking, lower valuation of evidence in belief formation and revision). The link between delusion-proneness and belief in fake news (which was established for the first time in these studies) may prove useful in helping to inoculate the public against the deleterious effects of purposely-spread misinformation. Identifying individuals who might be at high risk of falling for fake news is an essential first step in this direction. The present results suggest that individuals who endorse delusion-like ideation may be one population toward which interventions aimed at preventing belief in misinformation might usefully be aimed.

### T85. PRELIMINARY ANALYSES OF THE NEUROCOGNITIVE DATABASE OF PRONIA USING UNIVARIATE STATISTICS: CLINICAL GROUP DIFFERENCES

Marco Garzitto*[,1], Lana Kambeitz-Ilankovic[2], Carolina Bonivento[3], Sara Piccin[1], Stefan Borgwardt[4], Eva Meisenzahl[5], Marlene Rosen[6], Raimo Salokangas[7], Rachel Upthegrove[8], Stephen Wood[9], Nikolaos Koutsouleris[2], Paolo Brambilla[10]

[1]Scientific Institute IRCCS; [2]Ludwig Maximilian University; [3]University of Udine; [4]University of Basel; [5]Heinrich-Heine University Düsseldorf; [6]University of Cologne; [7]University of Turku; [8]University of Birmingham; [9]Univeristy of Melbourne; [10]University of Milan

**Background:** Neuro-cognitive deficits are a core feature of psychosis. In the clinical high risk stages of psychosis, neuro-cognitive deficits qualitatively affect the same functions while being quantitatively less marked compared to those in full-blown disorder. Therefore, cognitive impairments are considered to be an important intermediate phenotype for transition to psychosis. Partially overlapping deficits were also reported in depressive disorders, so it is important to identify deficits specifically associated to psychotic symptoms from those common to other conditions. We aimed to identify and differentiate cognitive deficits specifically associated to [i] psychopathology in general (i.e., presence of clinical diagnosis); [ii] psychotic symptoms; [iii] sub- and threshold levels of psychotic symptoms.
**Methods:** We compared four groups of participants within the project Personalised Prognostic Tools for Early Psychosis Management (PRONIA; www.pronia.eu). The PRONIA Cognitive Battery (PCB) includes 10 tests selected as reliable measures of neuropsychological difficulties in patients at high-risk of psychosis. The scores were obtained from the PRONIA Discovery Sample, which included 707 participants: 278 healthy controls (HC); 138 recent-onset depression (ROD); 139 clinical high-risk (CHR); 152 recent-onset psychosis (ROP), tested in seven sites across Europe. At first the norms were calculated correcting the HC's raw scores by sex, age, cognitive level, education, and mother language (English, Finnish, German, Italian, or other). Then, univariate analyses of variance with a priori contrasts were used for directly comparing [i] HC vs ROD/CHR/ROP; [ii] ROD vs CHR/ROP; [iii] CHR vs ROP.
**Results:** The difference in cognitive performance between the clinical groups (ROD, CHR, ROP) as compared to the HC [i], was shown in measures of: speed of execution ($\omega P2$ range 0.016–0.123; all $p \leq 0.035$); sustained attention ($\omega P2$: 0.024–0.080; $p \leq 0.022$); verbal fluency ($\omega P2$: 0.020–0.031; $p \leq 0.002$); emotion recognition ($\omega P2 = 0.026$; $p = 0.001$); visuo-spatial ($\omega P2$: 0.018–0.049; $p \leq 0.006$) and verbal ($\omega P2$: 0.038–0.075; $p < 0.001$) both short- and long-term memory. Three clinical groups did not show significant difference in salience measures when compared with HC ($p \geq 0.053$), beyond a main effect of group ($\omega P2 = 0.015$). Differences between ROD and CHR/ROP groups [ii] were detected in: speed of execution (all $p \leq 0.001$); sustained attention ($p \leq 0.011$); short-term and working memory ($p \leq 0.004$); long-term memory ($p \leq 0.001$); semantic verbal fluency ($p = 0.024$); emotion recognition ($p = 0.005$); and estimation of adaptive salience ($p = 0.021$). When compared with ROP, CHR [iii] performed significantly better in the same domains that differentiated ROD from CHR/ROP, with the important exception of long-term memory measures ($p \geq 0.094$).
**Discussion:** These results are consistent with the expectations drawn from previous literature on the neuropsychological impairments in psychotic disorders and CHR participants. Furthermore, PCB showed to be useful in [i] psychopathology in general, [ii] differentiating between recent-onset depression and psychotic symptoms, and [iii] between threshold and subthreshold psychotic symptoms. Interestingly, long-term memory deficits contributed more in differentiating psychotic symptoms from other psychopathological entities (ROD vs CHR/ROP comparison) than along the spectrum of attenuated psychotic symptoms, resulting in full clinical picture of psychosis (CHR vs ROP). Finally, salience attribution difficulties were confirmed to be associated with (sub-)threshold psychotic symptoms, more than to general psychopathology.

### T86. COGNITIVE SUBTYPES IN FIRST-EPISODE PSYCHOSIS: AN EMPIRICAL LONGITUDINAL STUDY OF RELATIONSHIP TO COGNITIVE, SYMPTOM AND FUNCTIONAL OUTCOMES

Andrew Watson*[,1], Bill Deakin[2], John Suckling[3], Paola Dazzan[4], Stephen Lawrie[5], Rachel Upthegrove[6], Nusrat Husain[2], Imran Chaudhry[2], Graham Dunn[2], Peter Jones[3], Danuta Lisiecka[3], Shon Lewis[2], Thomas Barnes[7], Stephen Williams[2], Stephen Hopkins[2], Emma Knox[2], Kelly Byrne[2], Richard Drake[2], Richard Smallman[2], Eileen Joyce[1]
[1]UCL Institute of Neurology; [2]University of Manchester; [3]University of Cambridge; [4]Institute of Psychiatry, Psychology and Neuroscience, King's College London; [5]University of Edinburgh; [6]University of Birmingham; [7]Imperial College London

**Background:** Variable outcomes following a first-episode of psychosis are partly attributable to heterogeneity in cognitive functioning. Previous work in first episode psychosis has identified clinically meaningful cognitive subtypes based on pre-specified differences in estimated premorbid and current cognitive functioning. We used an empirical clustering technique to examine whether these cognitive profiles can be replicated with an unbiased method, their relationship with clinical, cognitive and global functioning at psychosis onset as well as their stability over time.
**Methods:** Patients attending NHS early intervention services following a first episode of psychosis were recruited to a double-blind clinical trial of minocycline for negative symptoms of psychosis (BeneMin) which found no treatment effect. Participants were assessed on clinical, cognitive and global functioning at baseline (n=169) and 12-month follow-up (n=107). K-means analysis was used to empirically cluster participants on the basis of estimated premorbid IQ (WTAR), and baseline cognitive functioning (derived from 4 sub-tests of the WAIS IV, verbal fluency (COWAT) and verbal learning scores (AVLT)). Clinical and global functioning was assessed using the Positive and Negative Syndrome Scale (PANSS), Calgary Depression Scale (CDS) and Global Assessment of Functioning (GAF).
**Results:** K-means cluster analysis revealed three cognitive subgroups: 28% showing preserved premorbid and current IQ (PIQ); 29% displaying compromised premorbid and current IQ (CIQ); and 43% with normal premorbid but deteriorated current IQ (DIQ). There were no significant differences between groups in age, gender, CDS, cannabis use or olanzapine equivalent antipsychotic dose. The PIQ group performed significantly better than the DIQ group on all baseline cognitive measures, who performed significantly better than the CIQ group. At baseline, there were significant differences in GAF scores ($F(2,168) = 4.267$, $p=0.016$; PIQ >DIQ, LIQ). At 12-months, all three groups had improved over time on cognitive function with no group x time interactions except verbal fluency ($F(2, 97) = 5.204$, $p=0.007$) where only the DIQ group improved. All groups improved on positive and total symptom scores but only the PIQ and DIQ groups significantly improved on negative and general symptom scales. There was no significant change in GAF score in any of the groups over time. At follow-up there were significant differences between groups in negative syndrome scores ($F(2, 104) = 8.720$, $p=0.004$: LIQ > PIQ, DIQ) and global functioning ($F(2,101) = 5.880$ $p=0.004$; PIQ > DIQ, LIQ).
**Discussion:** Using an unbiased method to define cognitive subgroups at first episode, we confirmed in a new sample, previous findings which used pre-specified criteria. A large subgroup showed evidence of a decline in IQ at psychosis onset and 12-months later this subgroup had neither continued to deteriorate nor returned to premorbid levels of cognition. Patients with preserved normal and compromised cognitive function at psychosis onset showed no deterioration over 12 months. The cognitive sub-groupings were clinically meaningful. The preserved group showed better general function which persisted over 12 months. General functional outcome in the IQ decline group was as poor as the compromised group and the compromised group had more persistent negative symptoms.

### T87. TOWARD DEVELOPING CLINICAL CUTOFF VALUES FOR THE BECK COGNITIVE INSIGHT SCALE

Danielle Penney*[,1], Genevieve Sauve[1], Ashok Malla[2], Ridha Joober[2], Martin Lepage[2]
[1]Douglas Mental Health University Institute; [2]McGill University

**Background:** Cognitive insight represents the ability to question and criticize the validity of one's beliefs, to recognize when beliefs may be faulty, and to then rely on external feedback to make correct assessments of a situation. Cognitive insight is characteristically impaired in persons with schizophrenia and related psychoses. The Beck Cognitive Insight Scale (BCIS) is the most widely used tool to assess cognitive insight, yet there is no consensus regarding clinical cutoff values. Cognitive insight is predictive of better response to psychosocial treatment and the ability to accept critical feedback from treatment teams, thus cutoffs are an important next step needed to facilitate the clinical interpretation of the BCIS. Some studies have attempted to develop diagnostic cutoffs, yet no study has proposed clinical cutoffs to differentiate levels of cognitive insight between patients with schizophrenia.
**Methods:** Three hundred and eighty-five English or French-speaking patients with a schizophrenia spectrum disorder (203 first-episode and 182 multiple-episode psychosis patients) and 185 healthy controls completed a battery of clinical and neuropsychological tests, including the BCIS. Patients and controls were matched on age, sex, level of education, and socio-economic-status. Correlations were calculated between the composite index and previously identified correlates of cognitive insight. Variables significantly correlating with the BCIS composite index were then included in a clustering analysis to classify patients according to their clinical profile. Two clinical profiles representing low and high cognitive insight were identified, and were based on global functioning and IQ. Composite index scores at the 33rd percentile in the low cognitive insight cluster and the 66th percentile in the high cognitive insight cluster were calculated.
**Results:** Functioning and IQ significantly correlated with the BCIS composite index and were included in a clustering analysis, using a pre-determined number of two clusters. Independent samples t-tests revealed that the 2 clusters differed significantly on the BCIS self-reflectiveness score ($t(372) = -3.93$, $p < .001$) and on the composite index ($t(372) = -3.17$, $p = .002$). There was no difference between clusters on self-certainty ($t(372) = .31$, $p = .76$). Patients in cluster A had a mean SR, SC, and composite index of 12.65 (SD = 4.3, Range = 2 to 26), 7.78 (SD = 3.3, Range = 0 to 18) and 4.87 (SD = 5.8, Range = -11 to 20), respectively, while mean scores for patients in cluster B were 15.11 (SD = 4.1, Range = 3 to 25), 7.64 (SD = 2.9, Range = 1 to 15) and 7.47 (SD = 4.8, Range = -3 to 22). In cluster A, the values of the 33rd and 66th percentiles were 2.6 and 7 respectfully. In cluster B, these values were 5 and 9. We are proposing that 33% of patients with the lowest composite index scores in cluster A represent those with low cognitive insight. Accordingly, 33% of patients with the highest composite index scores classified in cluster B represent those with high cognitive insight. Low cognitive insight is thus represented by a score of 3 or below, borderline scores range from 4 to 9, and high cognitive insight is represented by a score of 10 or above.
**Discussion:** We proposed clinical cutoffs for the BCIS with a theoretical basis anchored in patient clinical profiles (functioning and IQ). Clinical cutoffs will facilitate and better orient treatment teams in the clinical interpretation of the BCIS and ergo to patients' level of cognitive insight. The development of such cutoffs will help to reduce heterogeneity in psychosocial group intervention, will facilitate interventions aimed at increasing cognitive insight, and improve communication between patients and their treatment teams.

### T88. CLUSTER ANALYSIS IDENTIFIES TWO NEUROCOGNITIVE PROFILES AMONG OFFSPRING AT GENETIC RISK OF A MAJOR MENTAL DISORDER

Rossana Kathenca Peredo Nunez de Arco*[,1], Michel Maziade[1], Valérie Jomphe[2], Elsa Gilbert[1], Thomas Paccalet[3], Chantal Merette[1]
*[1]Université Laval, CERVO Brain Research Centre; [2]CERVO Brain Research Centre; [3]Centre de Recherche CIUSSS-CN*

**Background:** Offspring of patients diagnosed with Schizophrenia (SZ) or Bipolar Disorder (BP) are at high risk (HR) of developing either SZ or BP and show impairment in various cognitive domains (Mortiz et al 2017, Gilbert et al 2014,). Also, the performance gradually decreases from relatives of patients with psychosis to individuals at prodromal phase and finally to subjects at first episode of psychosis (Hou et al. 2016). Recently a meta-analysis found that various cognitive domains were impaired in a pooled sample of subjects including clinical high risk for psychosis and first episode of psychosis with effect sizes ranging from -0.30 to -0.85 (Hauser et al. 2017). However, theses deficits were obtained from data of the entire sample of subjects at risk even though only a small percentage of all offspring at HR risk transit toward to a major mental disorder (Rasic et al.2014). Hence, the effect size reported may represent a mixture of larger and smaller deficits, referring to those who will eventually convert versus those who won't, respectively. This present study addresses this issue by attempting to separate offspring of individuals with SZ or BP into two subgroups according to their cognitive profile in order to differentiate a subgroup with healthy or close to healthy cognitive performance from another having a lower performance.
**Methods:** Our sample was composed of a HR group of 131 offspring from 6 to 24 years old. The sample was drawn from previous independent studies that targeted all multigenerational families densely affected by SZ or BP in the Eastern Québec (Canada) catchment area for genetic analysis purposes (Maziade et al. 2011). All subjects were assessed on: Processing speed, Verbal memory (VEM), Visual Memory (VISEM), Working memory and Executive functioning. An average hierarchical cluster analysis, using the Ward's method, was performed by age group on all five cognitive domains to separate the HR group into two subgroups according to their cognitive functioning. The pseudo F statistics and Pseudo T square index were used to estimate the number of clusters and ANOVA was also performed by age group to verify that the two clusters differed in their average cognitive scores. Then, both subgroups were compared to a control group of n= 131 subjects that matched the HR group by age and gender.
**Results:** The cluster analysis yielded two different groups, referred to as HR1 and HR2. For Processing speed and VEM, differences between HR1 and HR2 were statistically significant in almost all age groups (6-10,11-15,16–20 years old), for VISEM the two groups were different from 11 to 24 years old, while for Working memory and Executive functioning, HR1 differed from HR2 from age 16 to 24. Moreover, the HR1 group performed very similarly to the control group in all functions, while the HR2 group presented significant differences from control subjects in most cognitive performance with effect sizes often exceeding those previously seen and even reaching -2.3 for VISEM.
**Discussion:** One of the most striking results from our study was to detect one subgroup of HR with cognitive performance very similar to non at risk individuals, while the other subgroup performed even worse than what was presented in the literature. To our knowledge, this is the first study to reveal such two neurocognitive profiles across different age groups in the HR population. Still, further research is needed in longitudinal studies to investigate whether these findings are associated with the transition to a psychiatric disorder in the following years. Nevertheless, our study suggests that interventions with a neurocognitive target should be addressed earlier, due to the apparition of a breach in cognitive performance at very early stages in life.

### T89. DEFINING COGNITIVE "NORMALITY" IN SCHIZOPHRENIA: PREVALENCE OF BROAD AND NARROW CRITERIA AND RELATION TO CLINICAL AND FUNCTIONAL STATUS

Melissa Parlar[1], Walter Heinrichs[1], Stephanie McDermid Vaz[2], Clayton Wilson[3], Farena Pinnock*[,1]
*[1]York University; [2]McMaster University; [3]Ryerson University*

**Background:** Cognitive dysfunction is considered a core feature of schizophrenia. Nonetheless, patients with the illness overlap with healthy controls on many tasks, giving rise to the identification of subpopulations with relatively normal cognitive performance. However, the prevalence and implications of these subgroups for understanding schizophrenia are unclear because "normality" criteria vary. Estimates of the frequency of normal range performance in the patient population are as low as 0% and as high as 89%. This study examines the relation between different normality criteria and normality prevalence. It also assesses functional outcome and symptom severity in cognitively normal and impaired subgroups.

**Methods:** "Narrow" (IQ) and "broad" (MATRICS Consensus Cognitive Battery; MCCB) cognitive normality criteria were applied to data from schizophrenia (n = 99) and healthy control samples (n = 80). Functional outcome was assessed with the Multidimensional Scale of Independent Functioning (MSIF). The Positive and Negative Syndrome Scale (PANSS) was administered to measure symptom severity.

**Results:** Cognitive normality ranged from 13% (broad criterion) to 47% (narrow criterion) among patients. Patients meeting both broad (MCCB) and narrow (IQ) definitions were functionally disadvantaged compared to cognitively normal controls (t(63) = 7.05, p < .01; t(72) = 9.97, p < .01, respectively). However, cognitively normal patients showed no functional (MSIF) advantage relative to cognitively impaired patients based on both broad and narrow definitions of cognitive normality (t(95) = .43, p = .67; t(74) = -1.04, p = .30, respectively). Functioning did not differ between IQ and MCCB based cognitively normal patients (t(51) = .61, p = .55). Moreover, broad and narrow definitions of cognitive normality were not associated with differences in symptom severities relative to cognitively impaired patients. This held true for both positive (t(97) = 1.39, p = .17; t(76) = -.72, p = .47, broad and narrow definitions, respectively) and negative (t(97) = .98, p = .33; t(76) = -1.07, p = .29, broad and narrow definitions, respectively) symptom severity on the PANSS.

**Discussion:** Our data show that the prevalence of cognitive performance normality varies widely with the breadth of the normality criterion. However, regardless of the criterion applied, cognitively normal patients remain functionally disadvantaged relative to cognitively normal controls. Perhaps more importantly, however defined, cognitively normal patients demonstrate no advantage in functionality relative to cognitively impaired patients. Thus, patients meeting the broad definition of cognitive normality are not functionally advantaged relative to those meeting the narrow definition. We also found that varying definitions of cognitive normality/impairment have no implications for the severity of psychotic psychopathology in treated outpatients. Overall, the current study suggests that the reported prevalence of cognitive normality in schizophrenia is largely a product of definitional approaches. At the same time, the data cast doubt on the functional importance of preserved and proficient cognition regardless of definition and suggest that cognitive normality does not confer an advantage in terms of reduced symptom severity.

### T90. MEMBERSHIP IN A SCHIZOTYPY TAXON PREDICTS HOPELESSNESS AND THOUGHTS OF SELF-HARM 7 YEARS LATER

Richard Linscott*,[1]
[1]*University of Otago*

**Background:** Expressions of liability for schizophrenia are associated with suicidal thinking and behaviour. This relationship appears not to be specific to different expressions of suicide, although there is evidence suggesting that schizophrenia liability is associated with greater lethality. The relationship is evident among help-seeking and non-help-seeking volunteers and using measures of psychosis experience or self-reported schizotypy; it persists despite controlling for other psychopathologies. Given these observations, the link between suicide and schizophrenia liability may be rooted in shared pathogenic mechanisms. With this in mind, I tested whether stress sensitivity could contribute to the link between schizophrenia liability and suicidality in a prospective study.

**Methods:** At baseline (T1), n = 1074 undergraduates (M = 19.8 years, SD = 3.1; 30% male) completed the Schizotypal Personality Questionnaire (SPQ) and the Acute Hassles Scale (AHS), a self-report measure of stress sensitivity. Participants were classified as schizotypal or non-schizotypal by taxometric analyses of items for specific SPQ facets (cognitive-perceptual, interpersonal, and disorganization) and a general SPQ item set. Participants were classified as schizotypal (n = 43) if they were classified to the general schizotypy class to two or more specific-facet classes. At follow-up (T2) 7.8 years later (SD = 1.9, range = 5.5 to 10.5 years), the T1 schizotypy group (n = 43) and an age- and sex-matched control group (n = 216) were invited to participate in an online follow-up study. Of those invited, n = 84 (M = 27.7 years, SD = 3.3; 26% male; n = 15 schizotypal at T1) provided consent and completed the SPQ and AHS. At T2, hopelessness was assessed using 3 items from the Depression, Anxiety, and Stress Scales and thoughts of self-harm were assessed with one item from DSM-5 Self-Rated Level 1 Cross-Cutting Symptom Measure for adults. The small n at T2 prevented taxometric analyses of SPQ ratings at T2.

**Results:** At T2, 9.5% of participants reported thoughts of self-harm and 17.8% hopelessness. Cross-sectional regression analyses of T2 data showed that the SPQ and AHS total scores each predicted concurrent self-harm thoughts (β = .52, p < .001, and β = .45, p < .001, respectively) and hopelessness (β = .43, p < .001, and β = .24, p < .05, respectively). When T2 SPQ and AHS were entered simultaneously, only SPQ scores predicted self-harm thoughts (β = .39, p = .001 for SPQ; β = .20, p = .10, for AHS) and hopelessness (β = .46, p = .001, for SPQ; β = -.04, p = .74 for AHS). In longitudinal analyses, T1 taxon membership predicted T2 self-harm thoughts (β = .28, p = .011) and hopelessness (β = .33, p = .002) but T1 AHS did not (β = .09, p = .43, and β = .09, p = .40, respectively). T1 taxon membership remained a significant predictor of T2 self-harm thoughts (β = .28, p = .016) and hopelessness (β = .34, p = .004) when T1 AHS was entered as a concurrent predictor, whereas AHS predicted neither outcome (β = -.01, p = .95, and β = -.02, p = .84, respectively).

**Discussion:** Schizotypy classification during the late teens or early 20s predicted hopelessness and thoughts of self-harm 5 to 10 years later. Although stress sensitivity was correlated with concurrent thoughts or self-harm, stress sensitivity could not have accounted for the link between schizotypy and self-harm thoughts or hopelessness. The study is limited by the rudimentary nature of the assessment of self-harm thinking, the modest sample size, and the large rate of loss to follow-up.

### T91. DEVELOPMENT OF NOVEL BIS-AMIDINES FOR THE TREATMENT OF TOXOPLASMOSIS

Lorraine Jones-Brando*,[1], Claudia Bordón[1], Robert H. Yolken[1], Varvara Misheneva[1], Mikhail Pletnikov[1], Omkar Revu[2], James McNulty[2]
[1]*Johns Hopkins University School of Medicine;* [2]*McMaster University*

**Background:** Toxoplasma infections constitute a worldwide public health problem responsible for significant morbidity. Symptoms of acute infection by the agent, the apicomplexan parasite Toxoplasma gondii, can range from mild to life-threatening depending upon the immune status of the host. Toxoplasma infections are often unrecognized in immune competent hosts but infection can lead to the long-term establishment of cysts in the brain and thus a persistent infection. Such tissue cysts are associated with altered behavior and cognition in mouse models. Furthermore, serological evidence of Toxoplasma infection has been associated with an increased risk of recent onset psychosis, schizophrenia, and other psychiatric disorders in humans. Adjunct treatment of Toxoplasma-seropositive individuals afflicted with schizophrenia with effective anti-parasitic medications could be beneficial by eliminating the parasite cysts, thereby possibly alleviating psychiatric symptoms. However, currently available medications are ineffective against the cyst form of the organism responsible for persistent infection. Others have published the anti-Toxoplasma potential of derivatives of the bis-amidine pentamidine. We designed and synthesized a panel of novel bis-amidines and explored their anti-Toxoplasma efficacy.
**Methods:** We designed and synthesized an array of twelve novel bis-amidines. These compounds were examined for in vitro activity against T. gondii tachyzoites first using a colorimetric assay employing the β-gal producing strain RH-2F to examine the effect of the compounds over 5 days of parasite growth in human fibroblast host cells. This was followed by several immunofluorescence-based assays to determine if compounds can directly act on tachyzoites (red/green invasion assay), on an established infection of host cells (replication assay), and whether the compounds are parasiticidal, ie. can rid the host cells of tachyzoite infection after one dose (recovery assay). A rodent model of acute toxoplasmosis was established for examining in vivo efficacy of lead compounds.
**Results:** Four of these compounds proved highly efficacious in vitro with 50% inhibitory (IC50) values ranging from 200 nM to 1.3 μM. Therapeutic indices based on the ratio between the median cell cytotoxic dose and the IC50 ranged from 4 to 84. These four compounds all inhibited tachyzoite invasion and significantly inhibited in vitro replication over a 24-hour period at nanomolar concentrations. Additionally, two of the compounds evaluated thus far appear to be parasiticidal in vitro at 1 - 2 μM.
**Discussion:** Our results suggest that bis-amidines can be designed to be effective against experimental Toxoplasma infections. The parasiticidal activity of some of the compounds make them serious candidates for further drug development. Further experiments to determine 1) in vivo efficacy in mouse models of persistent infection, and 2) synergy with antipsychotics and mood stabilizers will investigate the possibility that these compounds could be used as adjunct treatment in Toxoplasma-positive individuals suffering with schizophrenia and other psychiatric disorders.

### T92. QUITLINK: ACCESSIBLE SMOKING CESSATION SUPPORT FOR PEOPLE LIVING WITH SEVERE AND ENDURING MENTAL ILLNESS

Amanda Baker[1], Ron Borland[2], Billie Bonevski[1], David J. Castle*[,3], Jill Williams[4], Catherine Segan[2], Peter Kelly[5], Alyna Turner[6], Lisa Brophy[7], Rohan Sweeney[6]
*[1]University of Newcastle; [2]Cancer Council Victoria; [3]St. Vincent's Hospital, University of Melbourne; [4]UMDNJ-Robert Wood Johnson Medical School [5]University of Wollongong; [6]Deakin University; [7]University of Melbourne*

**Background:** People with severe mental illness (SMI) typically die 20 years earlier than the general population, largely due to smoking related diseases. Their smoking rate is alarmingly high and persistent, which contrasts sharply with the steady decline in the general population's smoking rate. Smokers with SMI are equally motivated to quit smoking, but report less encouragement to quit by health professionals and are less able to succeed. When engaged in a program, some can quit successfully, but at lower rates than for the general population. Evidence-based smoking cessation interventions, such as quitlines, are underutilised by smokers with SMI. There is an urgent need to develop highly accessible, appropriately tailored cessation services for smokers with SMI to which mental health services can routinely refer smokers, and to explore why low smoking cessation rates persist among people with SMI receiving cessation treatment.
Quitlink will utilise peer workers to identify, support, and refer smokers with SMI in mental health services to Quitline, who will deliver a tailored, proactive and accessible smoking cessation intervention. Additionally, we wish to investigate participant and health worker perceptions of the support provided by Quitlink, the nature of barriers encountered and their impact on initiating and succeeding with cessation.
**Methods:** Design: A multi-centre prospective, randomised, open, blinded endpoint (PROBE) design will be utilised to compare standard smoking care alone against Quitlink. Assessments will be conducted at baseline, end of treatment, 3 months post treatment and 6 months post treatment.
Setting and sample: 382 smokers will be recruited from three mental health services in Victoria: Mind Australia, Neami and Melbourne's St Vincent's hospital.
**Intervention:** The manual guided Quitlink intervention consists of standard smoking care plus: Referral to Quitline, Quitline counselling, Quitline provision of NRT, Quitline engagement with mental health services, Quitline engagement of support network and peer worker help with participant contact.
**Results:** As recommended by the Society for Research on Nicotine and Tobacco expert workgroup, the primary outcome measure in this study will be 6 months prolonged abstinence at the 8-month follow-up (allowing participants up to 2 months to stop). Prolonged 6-month abstinence will be defined as per the Russell Standard, i.e. self-report of abstinence for 6 months (allowing up to 5 cigarettes in total) and biochemical verification of self-reported abstinence (validated in a face-to-face visit using expired CO monitoring using a Micro+ Smokelyzer, with a reading of over 8ppm defined as a treatment failure).
**Discussion:** Outcomes of the project will determine effectiveness and cost-effectiveness of a novel and highly translatable intervention resulting from linking two existing services (Quitline and mental health peer workers). This will be the world first RCT of a Quitline intervention delivered to people with SMI that also includes a concurrent economic evaluation. As smoking is the leading cause of preventable death in people with SMI, if Quitlink is shown to be effective, it has the potential to greatly improve individuals' longevity, quality of life, mental health and reduce health care costs. This is an innovative and practical service delivery model that has the potential to ensure that smokers with SMI have access to best-practice smoking cessation treatment. Secondly, regardless of effectiveness outcomes, the project's qualitative study will provide greater insights into the barriers faced by smokers with SMI and will assist in the development of even more effective interventions.

### T93. PERSONALITY TRAITS AND PSYCHOTIC SYMPTOMS IN RECENT ONSET OF PSYCHOSIS PATIENTS

Julia Sevilla-Llewellyn-Jones[1], Olga Santesteban-Echarri *[,2], Pablo Cano-Domínguez[3], Antonia de-Luis-Matilla[3], Alberto Espina-Eizaguirre[3], Berta Moreno-Kustner[4], Susana Ochoa[5]
*[1]San Carlos Clínico Hospital; [2]University of Calgary; [3]Hospital Clínico Universitario Virgen de la Victoria; [4]Málaga University; [5]Hospital Sant Joan de Deu*

**Background:** Personality in patients with psychosis, and particularly its relation to psychotic symptoms in recent onset of psychosis (ROP) patients, is understudied. The aims of this research were, first, to study the relation between each clinically significant personality trait (CSPT) and psychotic symptoms, and second, to study the variance of each CSPT that was explained by psychotic symptoms, sex and age.
**Methods:** Sample: Data was obtained from 94 ROP patients (78.7% males; mean age: 24.67(4.59)). Measures: The Millon Clinical Multiaxial Inventory (MCMI) and the Positive and Negative Syndrome Scale were used to assess CSPT (schizoid, avoidant, dependent, depressive, histrionic, narcissistic, antisocial, sadistic, compulsive, negativistic, masochistic, schizotypal, borderline, paranoid) and psychotic symptoms (positive, negative, disorganized, exited, and anxiety and depression). Statistical analyses: U Mann–Whitney tests and multivariate logistic regressions were carried out to test the association between clinical personality traits adjusting for symptoms, sex and age.
**Results:** From the 94 patients, 13.9% had schizoid, 20.8% had avoidant, 7.9% had depressive, 7.9% had dependent, 5% histrionic, 15.8% narcissistic, 9.9% antisocial, 6.9 % sadistic, 14.9% compulsive, 1% negativistic, 0% masochistic,

2% schizotypal, 2% borderline, 5% paranoid CSPT. Negative psychotic symptoms were higher in patients with schizoid CSPT. The excited symptoms were lower for those with avoidant and depressive CSPT. The anxiety and depression symptoms were higher for patients with dependent CSPT. The positive psychotic symptoms were lower for patients with histrionic and higher for patients with compulsive CSPT. Logistic regression demonstrated that gender and positive and negative symptoms explained 35.9% of the variance of the schizoid CSPT. Excited symptoms explained 9.1% of the variance of the avoidant CSPT. Anxiety and depression symptoms and age explained 31.3% of the dependent CSPT. Gender explained 11.6% of the histrionic CSPT, 14.5% of the narcissistic CSPT and 11.6% of the paranoid CSPT. Finally, gender and positive dimension explained 16.1% of the compulsive CSPT.
**Discussion:** The study highlights the importance of studying personality in patients with psychosis as it broadens understating of the patients themselves and the symptoms suffered.

### T94. HS-CRP TO EVALUATE CHRONIC INFLAMMATION AND CARDIOVASCULAR DISEASES IN SCHIZOPHRENIA

Rahma Nefzi*[,1], Amine Larnaout[1], Ben Ammar Hanen[1], Emira Khelifa[1], Aissa Amina[1], El Hechmi Zouhaier[1], Guemira Fethi[2]
*[1]Razi Hospital; [2]Salah Azaiz Institut*

**Background:** The metabolic syndrome is a combination of risk factors for cardiovascular disease (RFCV). These complications are responsible for a significant excess mortality found in patients with schizophrenia. C-reactive protein (CRP), the main protein of the acute phase of the inflammatory process, has been chosen as one of the most informative biomarkers for predicting vascular death and major cardiovascular events at 10 years of age. It is the moderate and chronic increase in CRP levels measured by high-sensitivity C – reactive protein (hs-CRP) that represents a risk factor for cardiovascular disease. In the meanwhile, the results of research on autoimmunity and inflammation during psychosis described high levels of inflammatory markers in schizophrenia. In fact, chronic inflammation, measured by high blood C-reactive protein level, has been described in schizophrenia. The aim of this work was to evaluate the association between serum levels of high-sensitivity C – reactive protein, as a marker of chronic inflammation, metabolic syndrome and cardiovascular risk in a cohort of Tunisian patients with schizophrenia during remission.
**Methods:** A cross-sectional and retrospective descriptive study was conducted at the “F” psychiatry department at the Razi Hospital, including 80 patients with schizophrenia in period of clinical remission. The evaluation focused on 11 cardiovascular risk factors: age, family history of early heart disease, physical inactivity, alcohol consumption, smoking, type 2 diabetes, android obesity, the elevation of total cholesterol, the decrease of hdl-cholesterol, high blood pressure, elevation of triglycerides. A dosage of high-sensitivity C – reactive protein was performed.
**Results:** 25 patients (31%) met the criteria for metabolic syndrome of the International Diabetes Federation (2006). 13 patients (16%) had none of the 5 diagnostic criteria for metabolic syndrome. The average number of cardiovascular risks was 3.66.
22% of patients had significant cardiovascular risk (number of risk factors ≥ 5).
The average measured CRP us was 3.43 ± 2.08 mg / l. Taking only the measure of hs-CRP as RFCV, 64% of our patients had a moderate cardiovascular risk and 38% had a significant risk.
Hs-CRP levels were not associated with metabolic syndrome (p=0.4). However, a strong association was found between high levels of hs-CRP and high risks for cardiovascular disease (p=0.006).
**Discussion:** Chronic inflammation plays a role in the pathophysiology of many chronic diseases, including cardiovascular diseases. It also plays an important role in the pathogenesis of schizophrenia. The role of the immuno-inflammatory system in schizophrenia arouses interest in immuno-psychiatric research. The association between chronic inflammation and cardiovascular diseases in schizophrenia could lead to treatments that would prevent the progression of both diseases overall.

### T95. PREVALENCE AND CONSEQUENCES OF CARDIAC AUTONOMIC DYSFUNCTION (CADF) IN 112 UNMEDICATED PATIENTS WITH SCHIZOPHRENIA

Karl Bär[1], Andy Schumann[1], Alexander Refisch[1], Steffen Schulz[1], Andreas Voss[1], Berend Malchow*[,2]
*[1]University of Jena; [2]Ludwig Maximilian University*

**Background:** A shortened life expectancy of about 15–20 years in patients with schizophrenia (SZ) is very well documented. Cardiovascular disease has been identified as one of the leading causes of premature death. Beyond the effects accounted for by lifestyle and antipsychotic medications, several lines of evidence indicate a shared underlying pathophysiology between both schizophrenia and cardiovascular disease. The most obvious link lies in the relation between cardiac autonomic dysfunction (CADF) and the development of cardiovascular diseases. CADF has been extensively described in patients with schizophrenia, with main features like an increased heart rate at rest, reduced baroreflex sensitivity or increased variability of the QT interval. However, the definite influence of autonomic dysfunction for reduced life expectancy is still unknown. Thus, one has to identify patients at increased risk. Therefore, we established a scoring system based on heart rate variability (HRV)-measures from unmedicated SZ patients to quantify autonomic changes associated with an assumed cardiac risk profile.
**Methods:** Autonomic measures were obtained from electrocardiogram recordings at rest in 112 unmedicated SZ patients and 112 age and gender matched healthy controls (HC). A rating score was obtained by relating 13 different, independent heart rate variability indices from every SZ patient to the 1st, 1.5th and 2nd standard deviations (SD) of the HC sample. According to the total amount of rating points, every SZ patient was classified into a corresponding subgroup of cardiac autonomic dysfunction (< 4 points = absent, 4 – 13 points = moderate, > 13 points = severe CADF). The selected HRV parameters contain different information of autonomic system modulation and have been proven very reliable in clinical research. Besides, symptom severity (Positive and negative syndrome scale) were determined as well as cardiac risk markers like BMI, smoking habits and physical activity.
**Results:** Severe CADF was present in 29% of the tested unmedicated SZ patients, whereas moderate CADF was present in 44% and absent in 27% of the cases. Therefore, about one third of the patients revealed severe cardiac autonomic changes correlating with a potential risk for cardiovascular events. Only 27 % of the SZ patients showed no pathological findings. Patients with severe CADF showed significantly higher heart rates at rest when compared to patients with moderate CADF [$F(2,109) = 23{,}089$; $p < 0{,}001$]. The ratio of Low Frequency/High Frequency was also significantly higher in SZ patients with severe CADF compared to moderate CADF [$F(2,109) = 38{,}321$; $p < 0{,}001$] which points to a shift of the autonomic balance to sympathetic modulation. The three subgroups of SZ patients did not differ significantly in terms of symptoms, BMI, smoking habits or physical activity. SZ patients with severe CADF had a significantly longer duration of the illness [$F(2,109) = 12{,}810$; $p < 0{,}001$].
**Discussion:** This study demonstrated that almost one third of unmedicated SZ patients show severe CADF. The close relation between CADF and the development of cardiac diseases or arrhythmias is well described. Therefore, these patients might need close cardiological follow-up appointments to reduce the likelihood of sudden cardiac death. In addition, the definite relation between the degree of autonomic dysfunction and the potential risk of cardiovascular events needs to be investigated in this patient population prospectively. Future studies need to design interventional strategies for everyday clinical settings to improve physical health and quality of life.

### T96. A RETROSPECTIVE DATABASE STUDY OF THE RELATIONSHIP BETWEEN ALCOHOL AND CANNABIS USE AND CLINICAL MEASURES IN EARLY PHASE PSYCHOSIS

Jacob Cookey[1], Philip Tibbo*[,2], Jacob McGavin[3], Sherry Stewart[1]
*[1]Dalhousie University; [2]Capital District Health Authority; [3]University of Toronto,*

**Background:** Alcohol is the most commonly abused substance in Canada (18%), with cannabis use being the second most commonly abused substance (7%). People diagnosed with psychotic disorders have similar or increased risk of alcohol and cannabis use disorders compared to the general population. While there is ample data investigating the negative impact cannabis has on the development and course of psychosis, there is very limited data examining the potential role that alcohol might play. This study, therefore, investigates the pattern of alcohol and cannabis use and the clinical impact it might have in those at the early phase of psychotic illness.
**Methods:** This is a cross-sectional, retrospective, database study of 264 patients at time of admission to the NSEPP (Nova Scotia Early Psychosis Program), in Nova Scotia, Canada. Outcome measures included the following domains: demographics/pattern of use, symptomatology, cognition and function. Four groups of patients with early phase psychosis (EPP) were analyzed according to risk level of current substance use: 1) low risk substance use (LR, n = 44), 2) moderate-high alcohol use only (AU, n = 33), 3) moderate-high cannabis use only (CU, n = 55), and 4) moderate-high combined alcohol and cannabis use (AU+CU, n = 132).
**Results:** Between group comparisons revealed statistically significant differences in: age (with the AU group being oldest), gender (with LR and AU groups with higher % females compared to CU and CU+AU groups), Positive psychotic symptoms (with AU group having the least positive symptoms), anxiety (with AU group having most anxiety symptoms), and functioning (with AU group having higher social/occupational functioning scores).
**Discussion:** Our findings reveal significant between group differences in a group of 264 patients at time of entry into the NSEPP. The age differences may suggest that those who develop psychosis at an older age could be more predisposed to use alcohol primarily. Alternatively, it is possible that alcohol somehow delays the onset of psychosis. The gender differences suggest that females with EPP use cannabis less commonly, and seem to choose alcohol use only if they are substance users. There are less intense positive psychotic symptoms (as measured by the positive and negative syndrome scale, PANSS) and more intense trait anxiety symptoms (measured by the state trait anxiety inventory, STAI) in the AU group which may either indicate that anxiety is worsened and positive symptoms are somehow suppressed with alcohol use, or else might reflect a subgroup in the EPP population (with less positive symptoms and more anxiety symptoms) that self-select for alcohol use. Finally, the AU group have the highest social/occupational functioning (measured with the social and occupational functioning assessment scale, SOFAS), which may indicate that alcohol facilitates social interaction/functioning; however based on existing literature, this finding more likely represents the fact that the more social someone is, the more likely they are to be exposed to alcohol in their peer group, and are therefore more likely to drink alcohol.

### T97. CANNABIS USE IMPACTS SYMPTOM PRESENTATION IN ANTIPSYCHOTIC NAIVE PATIENTS IN FIRST EPISODE OF PSYCHOSIS (FEP)

Luccas Coutinho*[,1], Cinthia Higuchi[1], Daniel Azevedo Cavalcante[1], Rodrigo Bressan[1], Quirino Cordeiro[2], Cristiano Noto[5], Ary Gadelha[1]
*[1]Universidade Federal de São Paulo; [2]Faculdade de Ciências Médicas da Santa Casa de São Paulo*

**Background:** Psychotic disorders induced by cannabis may present distinct symptomatic profile, course and underlying biology. Most studies on symptomatic effects of cannabis exposure are limited by examining patients after antipsychotic treatment. We investigated if antipsychotic naive FEP patients that reported cannabis use present higher symptom's severity and if affects deferentially any of standard psychosis dimensions (positive, negative, disorganized, excitement and depressive).
**Methods:** The sample comprised 194 antipsychotic naive FEP individuals. The baseline assessment was performed right after the admission at the emergency room and the follow-up assessment two months after antipsychotic treatment. The cannabis exposure was measured by ASI-6 (Addiction Severity Index) and additional questions addressing the relation to onset of psychotic disorders and how many times cannabis has been used. Cannabis use was reported by 41,2% of patients, and 25.8% reported heavy use (more than 50 times).
Dimensional psychopathology was assessed with the Positive and Negative Syndrome Scale (PANSS) and the symptom dimensions were constructed based on previous studies (positive, negative, disorganized, depressive, excitement). Considering cannabis use, we analyzed the following variables: 1 - Number of days of cannabis consumption in the last 30 days (Acute Use); 2 – Age of first cannabis use; 3 – Total cannabis use lifetime, categorized in no use, less than 50, more than 50 (total use).
**Results:** The mean age was 25.52 (sd=7.17), mean duration of untreated psychosis was 176 days (sd= 291) and most of the subjects were male (63%). Acute use of cannabis was associated with higher scores in the positive symptom dimension ($p = 0.017$, df= 40, R-squared = 0.132). Also, the earlier age of first cannabis use was related to higher presentation scores of the negative symptom dimension ($p = 0.002$, df = 33, R-squared = 0.238). No significant association was found between any cannabis exposure variable and other symptomatic dimensions excitement, depressive and disorganized symptoms´ dimensions. Cannabis use did not associate with duration of untreated psychosis ($p=0.443$, W= 2709.5). All the analyses were controlled by gender, age and duration of untreated psychosis.
**Discussion:** Acute and total exposure to cannabis affected deferentially the symptoms dimensions in patients at first episode of psychosis. Previous studies on the relationship between cannabis use and negative symptoms produced mixed results. This may be biased by antipsychotic exposure prior to first assessment. We will investigate the course of the symptoms of those patients to verify if the symptomatic differences are maintained.

### T98. THE EFFECT OF SUBSTANCE USE ON 10-YEAR OUTCOME IN FIRST EPISODE PSYCHOSIS – EARLY CESSATION RESULTS IN BETTER OUTCOMES

Melissa Weibell*[,1], Ingrid Melle[2], Inge Joa[1], Jan Olav Johannessen[1], Robert Jørgensen[1], Wenche ten Velden Hegelstad[1]
*[1]Stavanger University Hospital; [2]University of Oslo; [3]*

**Background:** The pre-TIPS study in 1994–95 showed that the duration of untreated psychosis (DUP) was long in our region with a mean value of 2.1 years, and median 26 weeks. This set the stage for the TIPS-study (1997–2000), reducing DUP through information campaigns targeted to the general population and other referral agents (GP's, schools and others) in Rogaland County (Norway). The information campaigns were launched together with a low threshold organization with direct access to an early detection team, for a diagnostic interview and help. No referral other than a phone call needed, and a guarantee of assessment within 24 hours if there was suspicion of psychotic symptoms. The hypothesis was that this could change help seeking behavior, awareness towards psychosis and thus reduce the DUP. The information campaigns and the early detection team were introduced in an early detection(ED) area (Rogaland county, Norway) comparing DUP with two usual-detection control sites in Oslo (Norway)

and Roskilde (Denmark). As a result, DUP in the early detection area was reduced from 26 weeks median to 4 weeks median.
All patients with First Episode Psychosis in the early detection area have been followed for ten years, and a twenty-year follow-up is to take place shortly. Symptom and function advantages of early detection and DUP reduction have been demonstrated as being significant throughout the follow-up period. Social and functional outcome have been increasingly emphasized as being key parameters, as these contribute to both quality of life and to financial costs in society.
Substance use is common in first-episode psychosis (FEP) and has been linked to poorer outcomes with more severe psychopathology and higher relapse rates. Early substance discontinuation appears to improve symptoms and function. However, studies vary widely in their methodology, and few have examined patients longitudinally, making it difficult to draw conclusions for practice and treatment.
**Methods:** We aimed to investigate the relationship between substance use and early abstinence and the long-term course of illness in a representative sample of FEP patients.
Out of 301 included patients, 266 could be divided into four groups based on substance use patterns during the first two years of treatment: persistent users, episodic users, stop-users and non-users. Differences in clinical and functional during the follow-up period were assessed using linear mixed effects (lme) models for the analysis of repeated measures data.
**Results:** Patients who stopped using substances within the first two years after diagnosis had outcomes similar to those who had never used with less symptoms than episodic or persistent users. Both episodic and persistent users had lower rates of symptom remission than non-users, and persistent users also had more negative symptoms than those who stopped using.
**Discussion:** Our findings emerge from one of very few long-term longitudinal studies examining substance use cessation in FEP with 10-year follow-up. The results convey hope that the detrimental effects of substance abuse on mental health may be significantly reversed if one stops the abuse in time. This can help patients who struggle with addiction with their motivation to embrace abstinence.

### T99. LONG-TERM CANNABIS USE ASSOCIATED WITH ALTERED FUNCTIONING DURING VERBAL LEARNING

Grace Blest-Hopley*[,1], Aisling O'Neill[2], Robin Wilson[3], Vincent Giampietro[4], Sagnik Bhattacharyya[3]
[1]*King's College London, Institute of Psychiatry, Psychology, & Neuroscience*

**Background:** Long-term use of cannabis has long been associated with changes in cognition, including memory and learning, particularly verbal learning in man. However, evidence regarding the neurobiological underpinnings of impairments in memory following long-term cannabis use has not been consistent. Furthermore, to our knowledge none of the studies published to date have specifically investigated whether brain function differed between cannabis users and non-users while learning new information as estimated over repeated trials. Therefore, we aimed to investigate this.
**Methods:** Twenty-one predominantly cannabis users (CU) who started using cannabis during adolescence and 21 healthy non-using controls (NU), completed a block design verbal paired associates learning task whilst undergoing functional Magnetic Resonance Imaging. The task required participants to learn and recall a set of word-pairs over 4 repeated trials. We examined the interaction between repetition and group (CU vs NU) on brain activation during encoding and recall condition using non-parametric repeated measures analysis of variance.
**Results:** There was no significant difference in total recall score between CU and NU. However, there was a significant effect of repetition ($p<0.001$) on recall score, suggesting that there was a significant improvement in recall score over repeated trials across the two groups of participants. Furthermore, there was a significant interaction between repetition and group on recall score such that the change in recall score over repeated trials significantly differed ($p = 0.032$) between the CU and NU groups. This was associated with a significant interaction ($p = 0.009$) between group and repetition on activation in the midbrain bilaterally, extending to the, parahippocampus, caudate and cingulate gyrus during the encoding condition. There was greater engagement of these regions in CU than in NU over repeated encoding trials.
**Discussion:** These results suggest that verbal learning is slower and more effortful requiring greater engagement of critical brain areas involved in learning in cannabis users compared to non-users.

### T100. NICOTINE USE IMPACTS NEGATIVE SYMPTOMS SEVERITY IN SCHIZOPHRENIA

Hianna Oliveira*[,1], Luccas Coutinho[1], Cinthia Higuchi[1], Cristiano Noto[1], Rodrigo Bressan[1], Ary Gadelha[1]
[1]*Universidade Federal de São Paulo*

**Background:** Nicotine use is higher among patients with schizophrenia (50–98%) than in general population (25–30%). This association can reflect a non-specific liability to substance use or specific effects of tobacco on symptoms severity or side effects. Studies about nicotine use and schizophrenia symptoms dimensions are controversial. Some of them showed a relation between severe nicotine use and higher positive symptoms and others presented a correlation between lower negative symptoms and nicotine use. That is why we aimed to verify whether nicotine use is associated with symptoms dimensions in patients with schizophrenia.
**Methods:** Two hundred and seven outpatients were enrolled from the Programa de Esquizofrenia da Universidade Federal de São Paulo (PROESQ/UNIFESP). Schizophrenia diagnosis was confirmed by Structured Clinical Interview for DSM-IV Axis I Disorders (SCID-I). Dimensional psychopathology was assessed with Positive and Negative Syndrome Scale (PANSS) and Fagerstrom Test for Nicotine Dependence. The PANSS items were grouped in five dimensions: positive, negative, disorganized/cognitive, mood/depression and excitement/hostility. The total score of Fagerstrom Test for Nicotine Dependence was the index used for severity in nicotine dependence. We used Wilcoxon-mann- whitney test to compare the means of PANSS dimensions between nicotine users versus non nicotine use.
**Results:** The patients mean age was 36.75 (SD 10.648), 69.1% were male, 48.3% reported lifetime tobacco use and 34.3% reported current tobacco use. Lower scores on negative dimension were associated with nicotine use ($W = 5642.5$, p-value = 0.046, effect size = 0.446). All p-values were corrected by Bonferroni test. Tests that evaluated the relationship between nicotine use and the total PANSS score or other dimensions were not statistically significant.
**Discussion:** This study shows that nicotine use impacts negative symptoms of schizophrenia. Increase in hepatic metabolism leading to low antipsychotic blood levels has been previously documented in patients with schizophrenia. Thus, the observed results can either indicate effect on primary negative symptoms or indirect effects through reduced D2 blockade caused by lower antipsychotic levels. Future quantitative analyzes and Longitudinal studies may better inform on direction of the association between nicotine dependence and negative symptoms in schizophrenia.

### T101. ENRICHING PSYCHOTIC DISORDER CLASSIFICATION USING NATURAL LANGUAGE PROCESSING

Rashmi Patel*[,1], Richard Jackson[1], Robert Stewart[1], Philip McGuire[1]
[1]*King's College London*

**Background:** Advances in molecular biology, genetics and neuroimaging have the potential to improve our understanding of psychotic disorders.

However, the clinical classification of psychotic disorders has remained largely unchanged and is based on criterion-based diagnostic systems (such as ICD-10 and DSM-5) which do not necessarily reflect their underlying aetiology and pathophysiology. A more refined characterisation of clinical phenotype could help to improve our understanding of these disorders. Clinical data are increasingly recorded in the form of electronic health records (EHRs). Automated information extraction methods such as natural language processing (NLP) offer the opportunity to quickly extract and analyse large volumes of clinical data from EHRs. We sought to characterise the range of presenting symptoms in a large sample of patients with psychotic disorders using NLP.
**Methods:** Dataset: South London and Maudsley NHS Trust (SLaM) Biomedical Research Centre (BRC) Case Register comprising pseudonymised EHRs of over 270,000 people.
Clinical sample: 18,761 patients with an ICD-10 diagnosis of a psychotic disorders (F20, F25 or F31) and a control group of 57,999 patients with a non-psychotic disorder diagnosis (mood/affective/personality disorders without psychotic symptoms).
Data collection: The NLP software package TextHunter was used. All sentences containing keywords relevant to the following symptom categories were analysed using a support vector machine learning (SVM) approach: positive symptoms, negative symptoms, disorganisation, mania and catatonia. Data on 46 symptoms were obtained with 37,211 instances annotated to contribute training and gold standard data for machine learning. 2,950 instances were independently annotated to determine inter-annotator agreement.
**Outcomes:** prevalence of psychotic symptoms and their association with ICD-10 diagnosis.
**Results:** A good degree of inter-annotator agreement was achieved (Cohen's κ: 0.83). Machine learning NLP achieved a mean precision (positive predictive value) of 83% and recall (sensitivity) of 78%. Among patients with psychotic disorders, the most frequently documented symptoms were paranoia, disturbed sleep and hallucinations. Psychotic symptoms were not limited to patients with an ICD-10 diagnosis of a psychotic disorder and were also present in the control group.
**Discussion:** We found that psychotic symptoms were not limited to patients with a specific ICD-10 diagnosis and were present in a wide range of ICD-10 disorders. These findings highlight the utility of detailed NLP-derived symptom data to better characterise psychotic disorders.

### T102. AN INVESTIGATION OF SCHIZOPHRENIA-BIPOLAR SUBGROUPS WITH GENETIC AND PROGNOSTIC VALIDATION

Dominic Dwyer*,[1], Janos Kalman[1], Monika Budde[1], Urs Heilbronner[1], Anne Ruef[1], Heike Anderson-Schmidt[2], Katrin Gade[4], Nikola Mueller[5], Ivan Kondofersky[3], Sergi Papiol[1], Peter Falkai[1], Thomas G. Schulze[2], Nikolaos Koutsouleris[1]
*[1]Ludwig Maximilian University; [2]University Medical Centre Göttingen; [3]Institute of Computational Biology*

**Background:** Separation of individuals into schizophrenia and bipolar diagnoses has long been questioned, with some suggesting that the classification impairs the understanding of etiology, the accuracy of prognoses, and treatment selection. In this study, we employed unbiased statistical techniques to identify subgroups of individuals with chronic illness using a large array of variables commonly evaluated at the bedside. We then validated the resulting groups by investigating age of onset, schizophrenia polygenic risk scores (PRS), and functional outcomes at a 1-year follow-up period. Our hypothesis was that transdiagnostic subgroups would be stratified based on illness onset whereby individuals with earlier onset would have higher genetic risk loading and poorer functional outcomes.
**Methods:** Participants were selected from a longitudinal, naturalistic, multi-site project (PsyCourse) designed to investigate psychiatric illness course and outcomes. A total of 329 participants (age(SD)=45.7(12.6); 54% female; years of illness duration(SD) = 13.7(10.3)) with a DSM-IV diagnosis of schizophrenia, schizoaffective disorder, or bipolar disorder were assessed from 17 centers at baseline and 1-year follow-up periods. A clinical battery measuring sociodemographic, illness history, symptoms, cognition, and personality questionnaires (199 variables) was used to subgroup individuals. A non-negative factor analytic consensus clustering MATLAB toolbox was created based on previous methodological work in oncology. PRS were generated using widely used strategies, and differences between resulting subgroups were investigated with MANCOVA controlling for ancestry effects. Differences in functional outcomes were investigated with repeated measures ANOVA.
**Results:** A 4-subgroup solution was robustly defined as the optimal solution using resampling techniques and cluster validity indices. Diagnoses were mixed in two subgroups, but predominantly bipolar or schizophrenia in the other two. All subgroups had equal illness durations ($p>0.05$), but the age of onset showed a decreasing trend with the earliest age being linked to two subgroups: a mixed bipolar-schizophrenia group with intermediate levels of general functioning and in a schizophrenia group with low levels of functioning ($p<0.001$). PRS scores were significantly increased in the early-onset, mixed bipolar-schizophrenia subgroup ($p=0.007$, uncorrected) and in the schizophrenia group ($p=0.025$, uncorrected). Prognoses differed between the four groups ($p=0.003$), with the greatest increases in functional outcomes in a late-onset mixed diagnostic subgroup ($p=0.006$) and in the schizophrenia group ($p=0.002$).
**Discussion:** Four subgroups were detected and our hypothesis was supported by a relationship between earlier illness onset and higher schizophrenia genetic risk loading. While one of the subgroups with an earlier onset mostly consisted of individuals with schizophrenia, the other subgroup was diagnostically mixed. Our results tentatively suggest that transdiagnostic clustering may identify subgroups that could be effectively used to understand etiology and prognoses. Future research will investigate the possibility of differential treatment effects in these subgroups.

### T103. ODIP (OUTIL DE DIAGNOSTIC INFORMATISÉ DES PSYCHOSES / PSYCHOSIS COMPUTERIZED DIAGNOSTIC TOOL): A NEW, SIMPLE METHOD FOR GENERATING DSM DIAGNOSES FOR PSYCHOTIC DISORDERS

Jean-Romain Richard*,[1], Baptiste Pignon[1], Franck Schurhoff[1], Andrei Szoke[1]
*[1]INSERM*

**Background:** OPCRIT was designed as a powerful tool to diagnose psychotic and affective psychoses. It has been frequently used in international psychiatric research. However, with 90 items it is time-consuming to complete and the diagnoses provided include many which are no longer used. Furthermore, this application is no updated for certain operating systems or psychiatric classifications.
For these reasons, we have developed, a similar but much simpler tool focused on DSM classification of affective and non-affective psychoses.
**Methods:** ODIP is based on the DSM-IV psychotic disorders classification, focusing on psychotic disorders (affective and non-affective). We identified 13 criteria that allow for the distinction between affective disorders with psychotic features (Bipolar or Depressive episode), schizophrenia, schizophreniform, schizoaffective, delusional, brief or non-specified psychotic disorders. We also designed a form to collect data on these 13 items.
To assess how ODIP performs we tested it against the more complete OPCRIT and discordances in diagnosis were compared with the clinical diagnosis or, in a subsample of patients, with a research diagnosis.
This was done in a total sample of 464 patients with a first episode of psychosis.
First, we observed that only 34 out of 90 OPCRIT items are required to obtain a coherent DSM-IV diagnosis and that we could complete the items automatically using an algorithm based on the ODIP form.

All the process was first tested with 212 patients to avoid any computer generated errors. Then we compared results for all patients together and discordance between ODIP and OPCRIT diagnosis was then analysed to determine which corresponded better to the Clinician's diagnosis when available (unavailable for 17 patients with discordant diagnoses).
**Results:** 88.2% of diagnoses for the 364 patients were equivalent when comparing ODIP and OPCRIT results. For the discordant diagnoses most of them (7.2%) were so mainly because of lack of needed information and when one of the systems provided a wrong diagnosis it was more often the OPCRIT (4.1%) than ODIP (0.5%).
**Discussion:** We demonstrated the ability of our 13 item ODIP tool to provide more reliable diagnosis than OPCRIT in the context of first episode psychosis with no organic or toxic origin.
**Limitations:** This tool was not intended to assess affective disorder diagnosis but only specify the diagnosis for the episode. As yet it was tested only on first psychotic episodes.
The primary interest of this new tool is the speed of administration and the relatively simple algorithm implemented in an excel file and available from the authors on request.

### T104. ASSESSING THE UTILITY OF COPULA FUNCTIONS FOR RISK PREDICTION OF PSYCHOSIS

Landan Zhang*[,1], Charlotte Gayer-Anderson[1], The EUGEI Study, Craig Morgan[1], Robin Murray[1], Michael Pitt[1], Conrad Iyegbe[1]
*[1]Institute of Psychiatry, Psychology & Neuroscience, King's College London,*

**Background:** Studies which have attempted to assess the predictive potential of socio-environmental risk factors for psychosis have used such a variety of datasets and methodologies. As a result, it is not possible for policy-makers to understand how different models compare, or might inform evidence-based policy-making. Thus, the cumulative predictive potential of non-genetic risk factors for psychosis has not yet been studied systematically. An important question which has not been considered previously is whether correlation structure between multivariate risks can be detrimental to the goal of prediction, particularly across different populations. Model fitting to locally-relevant correlation structures can limit the generalizability of a prediction model. Copulas are mathematical functions which allow the joint risk function of two or more correlated variables to be modelled in spite of this inherent bias. The copula approach is a foundation methodology with applications in the fields of finance, insurance and banking, where it is used for risk-management purposes. This study examines the impact of copulas on the stability of prediction power for psychosis across different populations.
**Methods:** The data used in this work comes from work package 2 (WP2) (entitled "Functional Enviromics") of The European Union (EU)-funded European Network Study of Gene-Environment Interactions (EUGEI). The total dataset available consists of 1180 cases of first episode psychosis (ICD10 diagnostic criteria F20-F29 or F30-F33) and 1528 healthy controls recruited by 16 centres across 6 countries (United Kingdom, Holland, Spain, France, Italy, Brazil). We sought to compare the predictive performance of copulas against that of summary risk scores for formulating disease risk for a common set of socio-environmental risk factors. The copula methodology allows joint risks to be modelled as a distribution whilst summary scores convey the number of risk factors encountered by an individual, weighted by literature-derived odds ratios for association. Gaussian copula with non-Gaussian marginal distributions were used to capture the correlation structure of 9 discrete variables in total. These incorporated: Lifetime Cannabis Use, frequency of Cannabis Use, Household discord, severity of psychological abuse, severity of physical abuse, severity of sexual abuse, severity of bullying, number of adverse adult life events and intrusive adult life events. We applied a fully Bayesian approach which uses Markov Chain Monte Carlo to simulate latent variables from multivariate ordered probit model and also estimate the threshold parameters and parameters from copula model. The resulting joint distribution (a copula) mapped the relationship between cumulative exposure to these factors and risk of psychosis.
**Results:** A proportion of subjects were withheld from the copula, so that the performance of the finished function could be evaluated on unseen data. The performance of the 2 prediction methods was compared within and between recruitment centres and are conveyed in terms of:

- Sensitivity and false positive rates (The area under the Receiver Operating Characteristic curve)
- Percentage of variance explained (Nagelkerke R2)
- Calibration (whether predicted risks were correct)
- Discrimination (whether high risk subjects could be distinguished from low risk ones)
- Reclassification (model behaviour close to specific thresholds)

**Discussion:** The application of the copula methodology to the multi-centre EUGEI dataset provides us with the opportunity to tackle a major limitation of the summary scoring approach which is the default method for aggregating risks across most areas of health research.

### T105. FACTOR ANALYSES OF SUCCESSIVE ASSESSMENTS BY MULTIPLE SCALES HAVE A CONSISTENT STRUCTURE IN A COHORT OF FIRST EPISODE PSYCHOSES

Alexandra Berry*[,1], Max Marshall[1], Max Birchwood[2], Shon Lewis[1], Samei Huda[1], Alison Yung[1], Richard Drake[1]
*[1]University of Manchester; [2]University of Warwick*

**Background:** Depending on the nature of their items factor analyses of different scales impose different structures on the underlying psychopathological dimensions, so a broader range of scale items should be more revealing. Few studies repeat analyses over successive interviews to investigate whether psychopathology has a consistent structure or evolves, especially after first presentations when the illness is most plastic and cohorts are unselected by chronicity.
**Methods:** A cohort was recruited from consecutive presentations aged 16–35 to NHS Early Intervention in Psychosis services from 14 catchments over 5 years during the National EDEN project. All met DSM IV-R criteria for schizophrenia spectrum psychoses, brief or substance induced psychoses, mania or severe depression with psychosis. At recruitment, after 6 and 12 months each was assessed with Positive and Negative Symptom Scale (PANSS), Calgary Depression Scale (CDS), Young's Mania Rating Scale (MRS) and Birchwood's Insight Scale (IS). At each point principal axis factoring with oblique (Promax) rotation included all scale items simultaneously, apart from using total scores for IS. Items below communality thresholds were excluded and the analyses repeated until stable solutions were achieved with fit metrics meeting conventional thresholds. Factor solutions were selected using breaks in the scree plot and eigenvalues>1.0.
**Results:** 1003 met diagnostic criteria and 948 provided data. Each time point produced 6 factors featuring consistent items: psychosis (PANSS delusions, hallucinations, suspicion, stereotyped thinking & bizarre ideation; MRS grandiose content); excitement/mania/disorganisation (PANSS agitation; MRS elation, overactivity, pressured and disorganised speech); hostility/suspiciousness (PANSS hostility, uncooperativeness & impulsive irritability; MRS irritability & aggression); depression/anxiety (PANSS anxiety, guilt, depression; CDS subjective & objective depression, guilt & guilty ideas of reference, hopelessness, self-depreciation, suicidality, early waking); negative symptoms (PANSS blunting, emotional & social withdrawal, poor rapport, poverty of speech, retardation and avolition), and poor insight (PANSS insight, MRS insight, IS total). Depression explained 29–32% of variance at different stages, Psychosis 28–29%, Negative 25–26%, Excitement 19–24%, Hostility 16–23% and Poor Insight 16–23%.
**Discussion:** The cohort, recruited from consecutive presentations, included a full range of psychoses in sufficient numbers to factor analyse the scales' 51 parameters. There was evidence for 6 factors slightly different from the traditional 3 SAPS/SANS (Scales for the Assessment of Positive and Negative Symptoms) or 5 PANSS factors derived using chronically unwell samples with non-affective psychosis. There was more consistency than in previous first episode follow-up studies and affective and insight dimensions were more clearly defined.

### T106. IMPROVING PSYCHIATRIC DIAGNOSIS BY ADDING MOTOR FUNCTION NEXT TO MENTAL HEALTH FUNCTION: A NETWORK APPROACH

Galoeh Adrian Noviar*[,1], Didi Rhebergen[1], P. Roberto Bakker[2]
*[1]Vrije Universiteit Amsterdam; [2]Maastricht University*

**Background:** Currently, the predictive value of psychiatric diagnosis is inadequate compared to other medical fields. It has been suggested that the use of a network model might aid in acquiring new insights into the underlying connections between symptoms (Bringmann et al., 2013). In addition, previous research (Bakker et al., 2012) has revealed associations between dysregulated mental- and motor function. As such, the network graphs might be enhanced by adding non-mental factors.
**Methods:** Baseline data from a 4-year prospective naturalistic study (Bakker et al., 2012) was used to obtain data about 207 psychiatric long-stay patients. (i) Drug-induced movement disorders: tardive dystonia (TD), akathisia, parkinsonism, and dyskinesia. (ii) ratings of the clinical global impression-schizophrenia (CGI) scale, and (iii) age and total defined daily dose to account for potential confounders. Statistical programming environment R (Epskamsp, Cramer, Waldorp & Borsboom, 2012) was used to visualise several psychopathology-severity networks.
**Results:** Interpretation of the graphs is based on the "centrality" of the symptoms. Centrality indicates the influence of a symptom on the network. Parkinsonism scored a low centrality score in graphs depicting high psychopathology in contrast with the other levels. Dyskinesia scored a low centrality score in medium psychopathology contrary to the other levels. The network graphs show a consistent positive correlation between age and parkinsonism (0.25, 0.53, and 0.19 for low, medium, and high psychopathology, respectively.), and a negative correlation between age and akathisia (-0.32, -0.47, and -0.21, respectively). High severities of psychopathology negatively correlated with parkinsonism (-0.16) and positively correlated with high levels of TD (0.33).
**Discussion:** The usage of a network model including motor factors has provided useful information to take into consideration when examining psychopathology of a patient. TD and parkinsonism draw the most attention. More research with the dataset, combined with further developing the network architecture technique is needed to accurately map motor- and mental factors.

### T107. WHY VALIDATION MATTERS: A DEMONSTRATION PREDICTING ANTIPSYCHOTIC RESPONSE USING 5 RCTS

Adam Chekroud*[,1]
*[1]Spring Health*

**Background:** Machine learning methods hold promise for making more effective, personalized treatment decisions to improve outcomes and reduce the cost of care. The use of these techniques remains nascent in psychiatry, and relatively little research has focused on the extent to which models derived in one sample make accurate predictions in unseen samples. Statistical research indicates that model performance in unseen samples is generally lower than performance in the derivation sample.
**Methods:** We investigate the generalizability of machine learned models using data from five multi-site randomized controlled trials of antipsychotic efficacy (total N = 1511). We include 125 predictor variables collected at baseline in all five trials, including demographics, psychological/behavioral scales (AIMS, BARS, CGI, PANSS, and SARS), vital signs, complete blood count, blood chemistry, and urinalysis. Using elastic net regression, we predicted 4-week treatment outcomes according to a binary cut-point of 25% reduction in PANSS scores. This study compared model performance for a range of internal and external validation methodologies.
**Results:** First, we trained a separate model on each of the five trials with no internal or external validation and obtained single-trial balanced accuracies from 74.6% to 100%. When each trial was split into a 50% training set and 50% holdout set, the balanced accuracies on the holdout test set were between 48% and 60.6%. When models were trained on each trial using 10-fold cross validation, balanced accuracies ranged from 50% to 73.7%. When each model was trained on a single trial and then sequentially tested on each of the four other trials, the mean balanced accuracy for each trial ranged from 50.5% to 54.2%. Finally, when the model was trained on four trials combined and tested on the one trial left out (leave-one-trial-out validation), balanced accuracies ranged from 48.9% to 58.7%.
**Discussion:** The performance of models predicting antipsychotic treatment response is highly affected by the validation routine chosen. Performance estimated using one trial – even using internal cross-validation – is drastically higher than the performance obtained when the same model is tested on independent data from other clinical trials with similar protocols. These findings present considerable cause for concern regarding the interpretation of predictive analyses based on a single, multi-site trial.

### T108. ANALYTICAL AND PREDICTIVE VALIDITY OF HALLUCINATIONS IN FIRST PSYCHOTIC EPISODES

Maria Hernandez-Garcia[1], Carlos Gonzalez[2], Pablo Soldevilla[2], Olga Sparano[3], Miguel Hernandez[4], Carlos Cañete[4], Julio Sanjuan*[,1]
*[1]University of Valencia; [2]Clinic Hospital, Valencia INCLIVA; [3]Rivera Hospital; [4]Clinic Hospital, Valencia INCLIVA, CIBERSAM*

**Background:** Some studies of first psychotic episodes have suggested the association between childhood trauma, such as sexual abuse, and the risk of hallucinations.[1] Furthermore, other studies indicated that environment can alter the phenomenological presentation of first psychotic episodes.[2]
However, there are no studies about the association between hallucinations in first psychotic episodes and the prognosis of the disease. This is the main objective of this study. We also compared the phenomenological differences between hallucinations in first episode psychosis and persistent hallucinations in patients with chronic psychosis.
**Methods:** Naturalistic, longitudinal follow-up study in a sample of 173 patients of first psychotic episode attending public mental health service in Area 5 of Valencia region (Spain) in a period between 2010–2017.
We compared first-episode patients with hallucinations (N=38) with two samples: A) First-episode patients without hallucinations (N=137). B) Chronic patients with persistent hallucinations (N=45) from a previous study.[3]
In the first comparison we used the following variables: sociodemographic data, risk factors (such as cannabis consume and immigration), psychiatric pathology (CIE-10), psicopathology (including clinical scales GAF, CGI and PANSS), number of emercengy visits and number of hospitalization after the first psychotic episode.

In the second one, we use the PSYRATS scale to compare both groups.

**Results:** In the first comparison, First Episode Psychotic patients with and without hallucinations,we only found significant differences in the number of hospital income, with more hospitalizations in the non hallucinating group (P= 0.001).

In the second comparison, First Episode Hallucinations versus Chronic Persistent Hallucinations, significant differences were only found in the duration of the hallucinations, which was much higher in chronic persistent hallucinations group (P= 0.001)

**Discussion:** Consequently, it seems that first psychotic episode patients without hallucinations have more hospitalizations than first-episode patients with hallucinations. Moreover, we can conclude that the duration of voices is higher in chronic patients with persistent hallucinations than in first psychotic episode hallucinations.

Both results have practical implications in the prognostic importance of hallucinations in first psychotic episodes.

**References:**

1. Misiak, B. et al "Childhood traumatic events and types of auditory verbal hallucinations in first-episode schizophrenia patients", Comprehensive Psychiatry 66 (2016) 17–22.

2. Oher, F.J. et al "The effect of the environment on symptom dimensions in the first episode of psychosis: a multilevel study", Psychological Medicine 44 (2014) 2419–2430.

3. González, J.C. et al J., "Persistent Auditory Hallucinations", Psychopathology 39 (2006) 120–125.

### T109. CLUSTERING OF SCHIZOPHRENIA PATIENT SUBTYPES BY SPECIFIC SYMPTOM DIMENSIONS USING AN UNCORRELATED PANSS SCORE MATRIX (UPSM)

Seth Hopkins*[,1], Ajay Origala[1], Antony Loebel[1], Kenneth S. Koblan[1]

[1]*Sunovion Pharmaceuticals*

**Background:** Interpretation of the efficacy of antipsychotic agents in treating schizophrenia using standard (Marder) Positive and Negative Syndrome Scale (PANSS) factors is confounded by moderate-to-high between-factor correlations. In previous pooled analyses of short-term, placebo-controlled lurasidone clinical trials, clustering and factor analysis identified an uncorrelated PANSS score matrix (UPSM) that generated transformed PANSS factor scores with high face validity (good correlation with standard [Marder] PANSS factors), and high specificity/orthogonality (low levels of between-factor correlation) at both baseline, and when measuring change during short-term treatment. In a validation analysis using 12 separate clinical trials, we previously confirmed that the weighted UPSM coefficients had generalizable utility, yielding transformed PANSS factors with high specificity while retaining good levels of correlation with standard PANSS factors. The aim of the current analysis was to determine whether distinct clinical subtypes of schizophrenia could be empirically derived from the transformed PANSS factor scores at baseline.

**Methods:** In a new analysis of a pooled sample of 5 placebo-controlled trials (N=1,710 patients), K-means clustering of baseline UPSM factor scores in MATLAB was used to identify whether clinical sub-groups could be empirically derived that were characterized by predominant symptom severity in one or more of the transformed PANSS factor domains. For each empirically derived domain thus identified, key demographic and clinical variables were examined, including baseline transformed PANSS factor severity scores [note: the weighted UPSM coefficient yields factor scores with numerical values that are much smaller than are observed with standard Marder factor scores]; and Montgomery-Åsberg Depression Rating Scale (MADRS) and Negative Symptom Assessment Scale (NSA) scores.

**Results:** Cluster analysis using the UPSM transformed PANSS Factor scores identified 5 distinct clinical subtypes defined by the severity of the UPSM Factor score relative to the mean score for all patients on the respective transformed PANSS factors. For the predominant positive cluster, the mean transformed PANSS positive factor score was 3.9 (vs. a mean score of 2.9 ± 0.9 SD for all patients); for the predominant hostile cluster, the hostility factor score was 2.6 (vs. a mean score of 1.4 ± 1.1); for the predominant disorganized cluster, the disorganized factor score was 3.0 (vs. 2.5 ± 1.0); for the affective cluster, the anxiety and depression factors, respectively, were 2.3 (vs. 1.8 ± 0.9) and 2.7 (vs. 1.7 ± 1.0); and for the predominant negative cluster, the apathy/avolition and deficit of expression factors, respectively, were 3.1 (vs. 2.5 ± 0.9) and 2.5 (vs. 1.8 ± 0.9). Patients in the predominant negative cluster had the highest NSA score (61 vs. a mean score overall of 53); and patients in the predominant affective cluster had the highest MADRS score (16 vs. a mean score overall of 11).

**Discussion:** These results provide evidence for a consistent underlying schizophrenia symptom structure and suggest the utility of UPSM transformed PANSS factors for characterizing clinical differences among clearly delineated clinical subpopulations, even within a clinical trial population of acute schizophrenia.

### T110. FIRST EPISODE PSYCHOTIC PATIENTS WITH A HISTORY OF FREQUENT CANNABIS USE EXPRESS MORE POSITIVE SYMPTOMS AT ILLNESS ONSET THAN THOSE WHO NEVER USED CANNABIS

Diego Quattrone*[,1], Charlotte Gayer-Anderson[1], Laura Ferraro[2], Giada Tripoli[1], EUGEI group[3], Evangelos Vassos[1], Pak Sham[4], Jim van Os[5], Craig Morgan[1], Cathryn Lewis[1], Ulrich Reininghaus[5], Robin Murray[1], Marta Di Forti[1]

[1]*Institute of Psychiatry, Psychology & Neuroscience, King's College London;* [2]*University of Palermo;* [3]*European Network;* [4]*The University of Hong Kong;* [5]*Maastricht University*

**Background:** Robust evidence has demonstrated that cannabis use increases the risk to develop psychotic disorders. However, a limited number of studies have investigated if and how cannabis use influences psychopathology profiles at first episode psychosis (FEP).

Based on the evidence that dopamine dysfunction contributes to explain positive symptoms in psychosis, and that the main cannabis' psychoactive component, Δ9-Tetrahydrocannabinol (THC), modulates the dopamine system, we hypothesise that: 1) positive symptoms at FEP are more common among psychotic patients who used cannabis compared with never users; 2) this association is a dose-response relationship.

**Methods:** We analyzed a sample of 1130 FEP patients as part of the EUGEI study, recruited across six countries. The MRC Socio-demographic Schedule was used to collect sociodemographic information. Psychopathology was assessed with the OPerational CRITeria (OPCRIT), and symptom items were analyzed using Mplus to estimate a multidimensional model of psychosis. The Cannabis Experience Questionnaire modified version (CEQmv) was administered to collect information on cannabis, and different patterns of use were computed based on frequency of consumption and type of cannabis, as a proxy of exposure to THC.

**Results:** The lifetime rate of cannabis use was 63%. Fifty-five percent of cannabis users consumed mostly high-potency cannabis, and 46% showed a daily frequency. Mixed-effects linear regression revealed that frequency of cannabis use was associated with the positive symptom dimension score. Daily users of high-potency cannabis presented with the strongest association (B=0.19, 95%CI=0.02–0.38), even after gender, age, ethnicity, other drug use, and study site were controlled for.

**Discussion:** Our results show that patients with a history of daily use of high potency cannabis express more positive symptoms at psychosis onset, even after taking into account other substance use and relevant sociodemographic factors.

### T111. PANSS NEGATIVE SYMPTOM DIMENSIONS ACROSS GEOGRAPHICAL REGIONS: IMPLICATIONS FOR SOCIAL, LINGUISTIC AND CULTURAL CONSISTENCY

Anzalee Khan*[,1], Lora Liharska[2], Philip Harvey[3], Alexandra Atkins[4], Richard Keefe[5], Danny Ulshen[4]
*[1]Nathan S. Kline Institute for Psychiatric Research; [2]New York University School of Medicine; [3]Leonard M. Miller School of Medicine, University of Miami; [4]NeuroCog Trials; [5]Duke University Medical Center*

**Background:** Recognizing the discrete dimensions that underlie negative symptoms in schizophrenia and how these dimensions are conceptualized across geographical regions may result in better understanding and treatment. The expressive-experiential distinction has been shown to have vast importance in relation to functional outcomes in schizophrenia. Previous studies have shown that the PANSS may not be equivalently rated across counties and cultures, suggesting regional differences in both symptom expression and rater judgment of symptom severity. Items that perform in markedly different ways across demographic, regional, cultural, or clinical severity characteristics may not offer valid representations of the target construct. 1) Will the expressive and experiential dimensions of the PANSS vary over 15 geographical regions and will the item ratings defining each dimension manifest similar reliability across these regions? 2) In large multi-center, international trials where data are combined, which of the two dimensions are disposed to social, linguistic and cultural inconsistency?

**Methods:** Data was obtained for the baseline PANSS visits of 6,889 subjects. Using Confirmatory Factor Analysis (CFA), we examined whether the expressive-experiential distinction would be replicated in our sample. We investigated the validity of the expressive-experiential distinction using Differential Item Functioning (DIF; Mantel-Haenszel) across 15 geographical regions – South America-Mexico, Austria-Germany, Belgium-Netherlands, Brazil, Canada, Nordic regions (Denmark, Finland, Norway, Sweden), France, Great Britain, India, Italy, Poland, Eastern Europe (Romania, Slovakia, Ukraine, Croatia, Estonia, Czech Republic), Russia, South Africa, and Spain - as compared to the United States.

**Results:** Expressive Deficit: More DIF was observed for items in the Expressive deficit factor than for items relating to experiential deficits. The following regions showed at least moderate to large DIF for all items: Austria-Germany, Nordic, France, and Poland. Of all the items, N3 Poor Rapport showed the most moderate and large DIF (n = 13; 86.67%) across countries, with 7 countries reporting large DIF. Similarly, N6 Lack of Spontaneity and Flow of Conversation showed moderate and large DIF for 66.67% countries (n=10). Experiential Deficit: Item G16 Active Social Avoidance reported negligible DIF for 14 of the 15 countries investigated (93.33%). Large DIF was observed for N2 Emotional Withdrawal and N4 Passive Apathetic Social Withdrawal for Brazil and India. Seven regions demonstrated no DIF across all items of the PANSS experiential deficit factor (South America-Mexico, Belgium-Netherlands, Nordic, Great Britain, Eastern Europe, Russia, and Spain). Overall, there were many fewer observed items with large DIF for PANSS experiential domain.

**Discussion:** These results suggest that the PANSS Negative Symptoms Factor can be better represented by a two-factor model than by a single-factor model. Additionally, the results show significant differences in ratings on the PANSS expressive items, but not the experiential items, across regions. This could be due to a lack of equivalence between the original and translated versions, cultural differences in the interpretation of items, rater training, or understanding of scoring anchors. Knowing which items are challenging for raters across regions can help guide PANSS training to improve results of international clinical trials aimed at negative symptoms.

### T112. TRADITIONAL RISK FACTORS NOT ENOUGH TO EXPLAIN THE SHORT LIFETIME EXPECTANCY IN PATIENTS WITH SCHIZOPHRENIA

Moradi Hawar*[,1], Anna-Karin Olsson[2], Fredrik Hjärthag[1], Madeleine Johansson[3], Maivor Olsson-Tall[3], Lars Helldin[2]
*[1]Karlstad University; [2]NU Health Care Hospital, Karlstad University; [3]NU Health Care Hospital*

**Background:** Patients with schizophrenia have about 20 years shorter lifetime expectancy compared to healthy population. The cause of this excess in mortality is due to both unnatural and natural causes. While the lifetime prevalence of death due to suicide among patients with schizophrenia is estimated to be 4.9%, Cardiovascular (CV) disease contributes to as much as 50% of the excess mortality in patients with schizophrenia.

This study focuses on whether hypertension, diabetes, hyperlipidemia and tobacco could be related to the reduced lifetime expectancy in patients with schizophrenia spectrum disorder.

**Methods:** From the Clinical Long-term Investigation of Psychosis in Sweden (CLIPS) study, 79 patients now deceased were analyzed at baseline. Data regarding occurrence of hypertension, diabetes, hyperlipidemia, tobacco but also data on the type of antipsychotic treatment were collected. Two patients, one with zero risk factors and one with 5 risk factors were omitted from the study. We created four categories based on the number of risk factors. 31 patients with one risk factor, 24 patients with two risk factors, 12 patients with three risk factors and 4 patients with four risk factors.

**Results:** The mean age for death was 61 years and the age varied between 35–83 years old. 18 percent were treated with typical antipsychotics and 61 percent with atypical antipsychotics. 18 percent had both atypical and typical antipsychotic treatment. 17 percent had treatment for diabetes, 27 percent had treatment for hypertonia, 8 percent had treatment for hyperlipidemia and 43 percent were using tobacco. The data collected pictures the occurrence of the different risk factors on average 6 years before their death. We compared the age of death for the four different risk factor groups with a Kruskal-Wallis Test and could not find any significant difference between them.

**Discussion:** Compared to the general population in Sweden there is an increased risk for diabetes in patients with schizophrenia, however the prevalence of hypertonia is the same, 27 percent for 18 years old and elder, in the general population. Daily tobacco use was rather high among patients with schizophrenia. Compared to general population, women and man with 10 percent respective 8 percent higher. Even if both diabetes and tobacco use has a high prevalence in patients with schizophrenia, it may not be enough to explain the reduced lifetime expectancy in patients with schizophrenia This study indicates that metabolic syndrome and the risk factors it contains need to be further studied in order to find its association to early death in patients with schizophrenia.

### T113. THE LINK BETWEEN BLUNTED AFFECT AND SUICIDE IN SCHIZOPHRENIA: A SYSTEMATIC REVIEW

Markella Grigoriou*[,1], Rachel Upthegrove[1], Lisa Bortolotti[1]
*[1]University of Birmingham*

**Background:** The lifetime risk of suicide and suicide attempt in patients with schizophrenia are 5% and 25%–50%, respectively. Understanding the suicide risk factors is of great significance in research and clinical practice. The current systematic review is the first attempt to examine and demonstrate the associations between the core negative symptom of blunted affect and suicide in people with schizophrenia. We believe this review may have important implications for suicide epidemiology and helps us improve prevention tools.
**Methods:** A comprehensive search strategy using PRISAMA guidelines was used to identify potential studies and data that met inclusion criteria. We searched original studies published since 2016 via MEDLINE (R) from 1946 to February 2016, EMBASE from 1947 to February 2016, and PsychINFO from 1806 to February 2016. Inclusion criteria were met if an article reported any kind of correlation between negative symptoms and suicide ideation, attempted suicide or completed suicide in patients with schizophrenia. The used search terms were: schizophreni* AND suicid* AND negative symptom* OR affective symptom* OR expressed emotion* OR emotional internal*. Studies with original data related to the blunted affect and suicide in schizophrenia were examined by manual reviewing.
**Results:** The initial search found 878 papers about negative symptoms and suicidal behaviour. From those only 12 papers fulfilled the inclusion criteria. Eight of twelve eligible papers found a positive association between blunted affect and suicide in schizophrenia indicating the link between social isolation and blunted affect with suicide ($p<0.018$), the impact blunted affect has on completed suicides on female population with schizophrenia ($p<0.034$) and the link between blunted affect and suicide in the stage of hospital admission ($p<0.001$). Two of twelve papers report no significance between blunted affect and suicide. One paper shows blunted affect did not have direct relation with suicide but can lead to the development of a suicidal behaviour. The last paper demonstrates blunted affect is important as suicide risk factor in schizoaffective disorder only.
**Discussion:** Based on the best available data, our results demonstrate a challenging link between blunted affect or related emotional disturbances and suicide in schizophrenia. Despite major suicide risk factors such as hopelessness, positive symptoms and depression, blunted affect is another factor we need to consider as it relates to social engagement and emotion regulation which are essential elements for eliminating suicides and improve interventions in psychiatry. Our outcomes may help with future development of preventive strategies and tools to combat suicide but also with gaining better understanding around what determines suicidal behaviour in schizophrenia.

### T114. SCHIZOTYPAL PERSONALITY QUESTIONNAIRE-BRIEF: FACTOR STRUCTURE ANALYSIS IN A NONCLINICAL ROMANIAN SAMPLE

Aziz Ferchiou*[,1], Maria Ladea[2], Mihai Bran[2], Marion Leboyer[3], Ruxandra Slavu[4], Franck Schurhoff[3], Andrei Szoke[5]
*[1]INSERM; [2]University of Medicine and Pharmacy "Carol Davila"; [3]H Mondor Hospital, DHU Paris-Est University, Fondation FondaMental; [4]Clinical Hospital of Psychiatry; [5]H Mondor Hospital, Fondation FondaMental*

**Background:** The study of schizotypal traits in the general population has been proposed as a way to understand aetiology and pathophysiology of schizophrenia. Self-report measures of psychometric schizotypy like the Brief version of the Schizotypal Personality Questionnaire (SPQ-B) have been shown to be valid, inexpensive and non-invasive tools. Few studies used a Likert-type scale format, which could be better able to allow partial endorsement and to detect more defended respondents than the forced choice format. At our knowledge, no studies of the SPQ-B used validity and social desirability items, to assess the potential impact of random or biased answers.
**Methods:** We examined factor structure and internal reliability of a Romanian version of the Schizotypal Personality Questionnaire-Brief (SPQ-B), in a Likert format in a sample of 580 students of Universities of Bucarest, Craiova and Brasov, in Romania. 3 validity items and 5 items of social desirability were added to the 22-items SPQ-B. We investigated the dimensional structure of the Romanian version of the SPQ-B first in the entire sample, and then after eliminating "bad" responders (i.e. those with aberrant answers on the validity items). We used a Principal Components Analysis (PCA) followed by a promax rotation. Factor selection was based on Eigenvalues over 1.0 (Kaiser's criterion), Cattell's scree plot test, and interpretability of the factors. We calculated Cronbach's Alpha for total SPQ-B and for each dimension.
**Results:** Our sample was constituted of 433 women and 147 men. The mean age was 25.5 ± 4.5 years. SPQ-B Likert total scores ranged from 23 to 90 points (mean = 55 ± 12). 71 participants were excluded after taking account of validity questions. Factor analysis of the entire sample resulted in a 3-factor solution that explained 43.8% of the variance. Factor 1 (Cognitive-perceptual; 10 items) includes items related to "ideas of reference", "magical thinking", "unusual perceptual experiences" and "suspiciousness". Factor 2 (Interpersonal; 5 items) includes items related to "social anxiety", "no close friends", and "constricted affect". Factor 3 (Disorganized; 7 items) includes items related to "odd behavior" and "odd speech".
Coefficient Alpha for the three subscales and total scale, respectively, were 0.74, 0.78, 0.78 and 0.86. There were no significant differences when the analyses were conducted in the sample of 509 "good" responders' students.
**Discussion:** Factor analysis of the Romanian version of the SPQ-B in a Likert format confirmed the three-factor structure of schizotypy. The SPQ-B and its subscales demonstrated good internal reliability. The use of items of validity and social desirability did not change significantly the results.

### T115. REASONING BIAS, WORKING MEMORY PERFORMANCE, AND A TRANSDIAGNOSTIC PHENOTYPE OF AFFECTIVE DISTURBANCES AND PSYCHOTIC EXPERIENCES IN THE GENERAL POPULATION

Christian Rauschenberg*[,1], Ulrich Reininghaus[1], Margreet ten Have[2], Ron de Graaf[2], Saskia van Dorsselaer[2], Nicole Gunther[1], Lotta-Katrin Pries[1], Sinan Guloksuz[1], Rajiv Radhakrishnan[3], Maarten Bak[1], Jim van Os[4]
*[1]Maastricht University; [2]Trimbos Institute; [3]Yale University School of Medicine; [4]University Medical Center Utrecht*

**Background:** There is robust evidence that reasoning biases such as a tendency of jumping to conclusions (JTC) as well as cognitive deficits are associated with psychosis, but evidence on their association with affective disturbances remains inconclusive. Recent findings also suggest a transdiagnostic phenotype of co-occurring affective disturbances and psychotic experiences. This study aimed to investigate whether JTC bias and decreased working memory performance are associated with the co-occurrence of affective disturbances, psychotic experiences (PEs), and psychosis-related help-seeking behaviour (HS) in the general population.
**Methods:** The second Netherlands Mental Health Survey and Incidence Study (NEMESIS-2). Trained interviewers administered the Composite International Diagnostic Interview (CIDI) at three time points in a representative general population sample, with N=4.596 individuals who completed all assessments. The beads task and digit-span task were completed to assess JTC bias and working memory performance, respectively. CIDI was used to measure affective disturbances (i.e. depression, anxiety, mania) and an add-on instrument to measure PEs and HS.
**Results:** We found that, compared to individuals with neither affective disturbances nor PEs, JTC bias was more likely to be present in individuals with co-occurring affective disturbances, PEs, and HS (moderate psychosis [1–2 PEs]: relative risk ratio [RRR]=1.23, 95% CI 1.03 – 1.48, p=0.023; high psychosis [3 or more PEs or HS]: RRR=1.66, 95% CI 1.26 – 2.19, p<0.001) in models adjusted for socio-demographic characteristics and socio-environmental factors. However, when we additionally adjusted for working memory performance this association was attenuated (moderate psychosis: RRR=1.17, 95% CI 0.98 – 1.41, p=0.088; high psychosis: RRR=1.57, 95% CI 1.19 – 2.08, p=0.002). In line with previous findings, there was no evidence that JTC bias was more likely to occur in individuals with sole presence of affective disturbances (RRR=1.03, 95% CI 0.94–1.13, p=0.492). Further, there was some evidence of a dose-response relationship, as JTC bias was progressively more likely to occur in individuals with affective disturbances as the level of PEs increased or HS was reported (high vs. moderate psychosis: p=0.052). In contrast, compared to individuals with neither affective disturbances nor PEs, a decreased working memory performance was evident in all groups (i.e., affective disturbances only: RRR=0.94, 95% CI 0.90–0.98, p=0.006; PEs only: RRR=0.79, 95% CI 0.69–0.91, p=0.001; co-occurring affective disturbances and moderate psychosis: RRR=0.83, 95% CI 0.75–0.91, p<0.001; co-occurring affective disturbances and high psychosis: RRR=0.76, 95% CI 0.65–0.89, p=0.001).
**Discussion:** Our findings suggest that JTC bias and decreased working memory performance may contribute to a transdiagnostic phenotype of co-occurring affective disturbances and PEs. Further, findings support the notion that JTC bias may be specifically associated with psychosis, including in those presenting a transdiagnostic phenotype, while a lowered working memory performance may represent a more broadly distributed vulnerability factor across various symptom domains. Overall, results point to the need to further investigate whether established mechanism and risk factors, described to be involved in the development and maintenance of psychosis, extend to transdiagnostic phenotypes to further corroborate proposed aetiological models and overcome shortcomings of focussing only on specific domains of mental health.

### T116. CAFFEINE-INDUCED PSYCHIATRIC MANIFESTATIONS

Kwanghun Lee*[,1], Won-Myong Bahk[2], Bo-Hyun Yoon[3], Duk-In Jon[4], Sang-Yeol Lee[5], Moon Doo Kim[6], Beomwoo Nam[7], Min-Kyu Song[8]
*[1]Dongguk University Hospital; [2]Yeouido St. Mary's Hospital, The Catholic University of Korea; [3]Naju National Hospital; [4]Hallym University; [5]Wonkwang University, School of Medicine; [6]School of Medicine, Jeju National University; [7]School of Medicine, Konkuk University; [8]Yesan Mental Health Clinic*

**Background:** The association between caffeine consumption and various psychiatric manifestations has long been observed.
**Methods:** We present two cases that show the ability of caffeine to induce psychotic and manic symptoms, and we also review the extant literature on caffeine-induced psychiatric manifestations.
**Results:** On the basis of our own and others' findings, we suggest that caffeine may be related to not only de-novo psychotic or mood symptoms but also to aggravation of pre-existing psychotic or mood disorders.
**Discussion:** We therefore suggest that caffeine consumption among patients with mood or psychotic symptoms should be assessed carefully in clinical practice as part of routine psychiatric evaluations.

### T117. INVESTIGATION OF FORMAL THOUGHT DISORDER AND RESPONSE TO TREATMENT IN SCHIZOPHRENIA

Fernando Rocha Loures Malinowski[1], Bruno Bertolucci*[,1], Cristiano Noto[1], Deyvis Rocha[1], Cinthia Higuchi[1], Rodrigo Bressan[1], Ary Gadelha[1]
*[1]Federal University of Sao Paulo (UNIFESP)*

**Background:** Formal thought disorder (FTD) is a multidimensional dysfunction characterized by inability to maintain a coherent speech in spoken or written language, poor social cognition and disorganized thought itself.
Presence of formal thought disorder has been associated with poor prognosis in schizophrenia, but the association with treatment response is yet to be determinate. Formal thought disorder has a close relation to disorganized symptoms in schizophrenia, which were independently associated with treatment resistance and poor response to standard antipsychotics. Formal thought disorder investigation could provide a clinical construct better delimited to assess disorganized symptoms in schizophrenia.
We investigated the association between FTD, remission and treatment resistance in patients with schizophrenia.
**Methods:** This study reunite a sample of 213 patients, between 14 and 69 years, who met DSM-IV criteria for schizophrenia.
The analyses were conducted in two samples conducted independently. In both samples, Diagnostic evaluation was performed with the Structured Clinical Interview for DSM-IV Axis I Disorders (SCID-I), response to treatment was primarily assessed through PANSS, functional impairment was assessed by GAF and disease severity, by CGI. The first sample was a follow-up study that enrolled inpatients. Participants were rated at baseline and after four weeks of antipsychotic treatment. If the participant did not reduce a minimum of 40% of baseline PANSS, the antipsychotic was switched. If the participant did not reduce a minimum of 40% in total PANSS in the following antipsychotic trial, the participant was considered as treatment resistant schizophrenia (TRS) and clozapine, introduced. The second sample was enrolled in an outpatient clinic specialized in schizophrenia.
Illness remission was defined as a severity of mild (score of 3 on a scale of 1 to 7) or less for P1, P2, P3, G9, G5, N1, N4 and N6 PANSS`s items.
To stablish FTD severity, PANSS items related to high scores at the Thought and Language Index (TLI) were considered: P2, P6, N1, N2, N5, N6, G7 and G9.
**Results:** Most subjects were male (56.8%) and the mean age was 34.42 (±12.33 SD).
The FTD failed to associate with remission (t = 4.007, p = 0,491) or treatment resistance (t = -3.768, p = 0.988) in both samples.
FTD had a negative correlation with GAF (r = -0.473, p<0.01) and a positive correlation with CGI (r = 0.530, p<0.01).
**Discussion:** FTD had a stronger association with global functioning and severity measures, rather than treatment symptomatic outcomes. In future studies, we will investigate whether FTD show distinctive clinical features commonly related to disorganized syndrome, i.e. earlier age of onset.

### T118. IMPACT OF DYSFUNCTIONAL METACOGNITIONS AND WORRY ON DEVELOPMENT OF PARANOIA: A 1-YEAR LONGITUDINAL STUDY IN A NON-CLINICAL SAMPLE

Xiaoqi Sun*[,1], Suzanne So[1], Raymond Chan[2], Chui-de Chiu[1], Patrick Leung[1]
*[1]The Chinese University of Hong Kong; [2]Institute of Psychology, Chinese Academy of Sciences*

**Background:** A worry thinking style has been identified as one of the proximal causal factors for paranoia (Freeman & Garety, 2014). This argument has been supported by the finding that patients with paranoia worry as much as patients with generalized anxiety disorder, and that worry predicts paranoia in non-clinical individuals. Wells (1995) argued that it is when metacognitions about worry (i.e. beliefs about worry and meta-worry) exaggerate worrying that anxiety disorders emerge. It was not clear how metacognitions interact with trait worry in the development of non-clinical paranoia.

**Aims:** To examine the predictive effect of dysfunctional metacognitions and trait worry on change in paranoia over one year within a large university sample.
**Methods:** An online survey encompassing measures of metacognitions, trait worry, and paranoia was conducted at baseline (valid N=2291) and one year (N=1746). A series of longitudinal structural equation models were tested, with baseline level of metacognitions as latent variable, baseline trait worry and paranoia at both time points as observed variables. Model fit indices were compared across models (CTI, RMSEA, AIC, BIC).
**Results:** A final trimmed model with the best goodness-of-fit ($\chi^2$=82.78, p<.001, CFI=0.99, RMSEA=0.069) suggested that dysfunctional metacognitions contributed to paranoia at 1-year follow-up, both directly ($\beta$=0.21, p<.001) and via baseline paranoia ($\beta$=0.09, p=.001). Trait worry at baseline did not predict paranoia at either time point.
**Discussion:** Our results indicated a critical role of dysfunctional metacognitions in paranoid ideation both concurrently and prospectively. Future interventions may focus more on modifying beliefs and worry about worry.

### T119. CAN SOME YOUNG PEOPLE RECOVER FROM FIRST-EPISODE PSYCHOSIS WITH INTEGRATED PSYCHOSOCIAL TREATMENT WITHOUT ANTIPSYCHOTIC MEDICATIONS? AN RCT TO ASSESS RISKS, BENEFITS, AND RANGE OF OUTCOMES

Patrick McGorry*[,1], Shona Francey[1], Barnaby Nelson[1], Graham Jessica[1], Baldwin Lara[1], Harrigan Suzy[1], Hok Pan Yuen[1], Fornito Alex[2], Allott Kelly[1], Alvarez-Jimenez Mario[1], O'Donoghoe Brian[1]
*[1]Orygen Youth Health Research Centre for Youth; [2]Monash University*

**Background:** While antipsychotic medication (AP) is a very effective treatment for positive psychotic symptoms in first-episode psychosis (FEP), it is also associated with risks. These include adverse neurological and metabolic effects and measurable changes in brain structure. APs may even be associated with poorer functional recovery. Due to advances in the detection of, and psychosocial treatments for FEP, it is now ethically feasible to study the relative risks and benefits of offering AP as a first line treatment, and conversely, of withholding it, on a background of comprehensive evidence-based psychosocial care. This non-inferiority design randomised double blind placebo controlled study examines whether a (low-risk) subgroup of people with FEP can recover without AP, and considers the effects on functioning, physical health, cognition, and brain structure of AP versus withholding AP.
**Methods:** Young people with FEP were screened for study eligibility and invited to participate if they met stringent inclusion criteria indicating low-risk of harm to self or others, and adequate social support. Hence a large proportion of patients were assumed a priori to be too high risk to withhold antipsychotic medication. Participants were randomly assigned to receive either low dose AP (MIPT group) or placebo (PIPT group) for six months, and all participants received intensive psychosocial treatment. Randomisation was stratified with three levels of DUP and gender creating six cells. Assessments of psychopathology, neurocognitive performance, and neuroimaging occurred regularly until two years after study entry.
**Results:** 90 young people were randomised and 81 commenced trial medication. They were 44% male and mean age 18.5 years (SD = 2.7). Thirty-four percent of participants completed the six month medication phase and there were more completers in the placebo group than the medication group. On the primary outcome measure of SOFAS there was significant evidence that the placebo group was not inferior to the medication group (SOFAS: MIPT mean = 61.5, SD = 13.4; PIPT mean = 61.7, SD = 16.8). The two groups were found to be very similar on all psychopathology assessments and measures of functioning at both baseline and following treatment, suggesting that the outcomes of the two treatment regimes were not different with respect to symptoms and functioning.
**Discussion:** The results of this study demonstrate that it is feasible and acceptable to conduct AP-free research in carefully selected FEP to examine the risk-benefit ratio of current treatments under carefully controlled conditions that prioritise patient outcomes and safety. Although only one-third of the participants completed the six month trial intervention period, more of those on placebo completed the trial phase and they had higher mean, minimum and maximum time in the experimental intervention phase than those on medication. In addition, there were no differences between the groups on measures of psychopathology and functioning, suggesting that the intensive psychosocial intervention provided to all participants is complementary and may be more important than antipsychotic medication in the early phases of psychotic illness for a subgroup of young people. However this subgroup is very small as a % of total FEP patients treated during the study period. Further analysis of physical health and neuroimaging data and completion of the 24 month follow-up assessments will allow detailed examination of the risk-benefit ratio regarding antipsychotic medication in FEP.

### T120. SUBMISSION WITHDRAWN

### T121. RATES AND PREDICTORS OF RELAPSE FOLLOWING DISCONTINUATION OF ANTIPSYCHOTIC MEDICATION AFTER A FIRST EPISODE OF PSYCHOSIS

Meghan Bowtell[1], Scott Eaton[1], Kristen Thien[1], Melissa Bardell-Williams[1], Linglee Downey[1], Aswin Ratheesh[1], Eoin Killackey[1], Patrick D. McGorry[1], Brian O'Donoghue*[,1]
*[1]Orygen, the National Centre of Excellence in Youth Mental Health*

**Background:** There is uncertainty about the required duration of long-term antipsychotic maintenance medication after a first episode of psychosis. Robust predictors of relapse after discontinuation are yet to be identified. The present study aimed to determine the proportion of young people who discontinue their antipsychotic medication after a first episode of psychosis, the proportion who experience relapse, and predictors of relapse.
**Methods:** A retrospective study of all individuals presenting to the Early Psychosis Prevention and Intervention Centre between 01/01/11 and 31/12/13 was conducted. A Cox regression analysis was conducted to identify predictors of relapse.
**Results:** A total of 544 young people with a FEP were included. A trial of discontinuation was undertaken by 61% of the cohort. Median duration of antipsychotic medication prior to first trial of discontinuation was 174.50 days. Amongst those trialing discontinuation, 149 (45.8%) experienced relapse in a median follow-up time post discontinuation of 372 days. On multivariate analysis, predictors of relapse were a diagnosis of cannabis abuse disorder (HR: 1.40), and longer duration of antipsychotic medication (HR: 1.05).
**Discussion:** Antipsychotic discontinuation frequently occurs earlier than guidelines recommend. Individuals with a diagnosis of cannabis abuse are more likely to experience relapse and addressing this substance abuse prior to discontinuation could possibly reduce relapse rates.

### T122. UNMET NEEDS IN PATIENTS WITH ACUTE TRANSIENT PSYCHOTIC DISORDERS (ATPD): ANALYSIS OF PATHWAYS TO CARE: AN 8 YEARS FOLLOW-UP STUDY

Amedeo Minichino*[,1], Grazia Rutigliano[2], Sergio Merlino[3], Cathy Davies[2], Dominic Oliver[2], Andrea De Micheli[4], Philip McGuire[2], Paolo Fusar-Poli[5]
*[1]University of Oxford; [2]Institute of Psychiatry, Psychology & Neuroscience, King's College London; [3]University of Pisa; [4]Institute*

*of Psychiatry Psychology and Neuroscience, King's College London, University of Pavia; [5]Institute of Psychiatry, Psychology & Neuroscience, King's College London, Maudsley NHS Foundation Trust, University of Pavia*

**Background:** Acute and transient psychotic disorders (ATPDs) constitute a highly heterogeneous category of brief psychotic disorders. The long-term course and outcomes of ATPDs is not completely clear, with more than half of patients initially diagnosed with ATPDs shifting towards other psychotic spectrum diagnoses. Uncertainties in the real-world clinical care of these patients is further complicated by the diagnostic overlap with the Brief Limited Intermittent Psychotic Symptoms (BLIPS). Thus, patients with similar diagnostic features may either be recommended conventional antipsychotic treatment (if diagnosed with ATPD and according to the current guidelines for first episode psychosis - FEP) or be contraindicated antipsychotic treatment and receive psychological therapies (recommended for BLIPS cases).

Given the complexity of the clinical presentation, admission to highly specialized services for early intervention in psychotic disorders (EIP) should represent the best therapeutic pathway for these patients. However, it is not known how many individuals with ATPDs are effectively detected and treated by EIP services.

This study aims at overcoming such a gap in knowledge by describing the pathways to care of patients with ATPDs and the treatments received across eight follow-up time-points (3, 6, 12, 18, 24, 48, 72, and 96 months).

**Methods:** Electronic health record-based retrospective cohort study including all patients who received a first index diagnosis of ATPD (F23, ICD-10) within the South London and Maudsley (SLaM) National Health Service Trust, between 1st April 2006 and 15th June 2017. Sociodemographic and clinical characteristics were analyzed using one-way ANOVA and Tukey post-hoc tests for continuous variables and chi square test for categorical variables. Logistic regression analyses were used to investigate the association between sociodemographic characteristics and detection/treatment by EIP.

**Results:** A total of 3074 patients receiving a first index diagnosis of ATPD (F23, ICD-10) within SLaM were included. The mean follow-up was 1495 days. After 8-year, 1883 cases (61.26%) retained the index diagnosis of ATPD; the remaining developed schizophrenia (23.8%), affective-spectrum psychoses (4.8%), and other psychotic disorders.

Only 7.5 % of ATPDs was detected and treated by Early intervention in Psychosis services (EIP). The remaining quote of patients were treated with general mental health services (91.5%).

Active treatment by EIS was more common among males, caucasian, and younger individuals (odds ratio (OR) = 1.35, 95% CI 1.01–1.7, P<0.001; OR = 0.6, 95% CI 0.46–0.78, P<0.001; and OR = 0.91, 95% CI 0.90–0.92, P<0.001, respectively).

Almost half of the total sample (48.5%) was in treatment with antipsychotic medications after 1 year of follow-up. This proportion dropped to 25% after 3 years of active treatment.

Less than 1% of ATPDs were offered psychotherapy interventions at any of the 8 time points of interest.

**Discussion:** The present study shows that the largest majority of individuals with ATPD (91.5%) is never detected and treated by the EIP services, which should be the best clinical option for these patients.

This suggests that they are receiving neither the best first-episode care nor the best preventative care. Efforts should be done to promote outreach campaigns in general mental health services to persuade clinicians referring these patients to local EIP services, with the aim of providing the best possible care.

### T123. PERSISTENT NEGATIVE SYMPTOMS IN FIRST EPISODE PSYCHOSIS: PREVALENCE, PREDICTORS AND PROGNOSIS

Stephen Austin*[,1], Carsten Hjorthøj[2], Ole Mors[3], Rikke Gry Secher[4], Pia Jeppesen[5], Lone Petersen[4], Anne Thorup[4], Merete Nordentoft[4]

*[1]Psychiatric Research Unit; [2]Copenhagen University Hospital, Mental Health Center Copenhagen; [3]Aarhus University Hospital; [4]University of Copenhagen; [5]Child and Adolescent Mental Health Center*

**Background:** Negative symptoms are a core component of schizophrenia. These symptoms have been shown to impact on a range of outcomes, and often are resistant to pharmacological and psychosocial interventions treatment. The goal of this study was to investigate the prevalence, baseline predictors and long-term impact of persistent negative symptoms (PNS) within a large representative cohort of people with first episode psychosis.

**Methods:** The study had prospective design. Patients recruited into the OPUS trial (1998–2000) with a first time diagnosis within the schizophrenia spectrum (F20-28) were included. People were classified with persistent negative symptoms, if they experienced enduring negative symptoms that were not secondary to psychotic symptoms, depression or due to medication side effects. Clinical data collected at baseline, 1 year, 2 years and 10 years was used to identify predictors of PNS and long-term outcomes.

**Results:** Full clinical data was available on 369 people. A total of 90 people (24%) displayed PNS, two years after diagnosis. Significant univariable predictors of PNS at baseline were low functioning, male sex, cannabis use, poor pre-morbid social functioning and high levels of negative symptoms. People that displayed PNS had significantly lower functioning and higher levels of psychopathology at 10 year follow-up. A total 3% of people with PNS were recovered at 10 year follow-up compared to rate of 20% recovered without PNS (OR 7.42, p<0.01).

**Discussion:** A significant proportion of the cohort displayed persistent negative symptoms and these symptoms significantly impacted on long-term outcomes. Researchers and clinicians need to continue to develop effective interventions that can ameliorate these symptoms and potentially impact on illness prognosis within schizophrenia.

### T124. CLINICAL DIFFERENCES BETWEEN URBAN AND RURAL SCHIZOPHRENIA

Amine Larnaout*[,1], Rahma Nefzi[1], Amina Aissa[1], Rouaa Trabelsi[1], Emira Khelifa[1], Hanen Ben Ammar[1], Zouhaier El Hechmi[1]

*[1]Hospital Al Razi*

**Background:** Schizophrenia is a highly heritable disease; yet heritability rate could never exceed 80% in most studies. Genetic factors could never then, by their own, account for the whole risk for developing schizophrenia. Thus, environmental factors co-play a major role. Urbanity is now well established to be one of the most influential environmental factors for developing severe mental illnesses. A strong association between exposure to urban environment and the risk of developing schizophrenia, was demonstrated and several studies have shown that living in an urban environment increases the risk for developing schizophrenia.

The aim of this study was to examine the clinic biological characteristics of patients with schizophrenia according to their rural or urban condition.

**Methods:** This study was based on 106 patients with a DSM 5 diagnosis of schizophrenia in period of remission. Sociodemographic and clinical characteristics were assessed. PANSS scale, Calgary depression scale, CGI scale were used to assess different features of the disease and biological variables (CBC, renal and liver markers, inflammatory markers: CRP and CRP hs) were evaluated. Two groups were formed according to the patient's urban or rural birth and early living.

**Results:** There were 71 urban and 35 rural patients. The mean age was 42,7 years in the urban group and 42,3 years in the rural group. Socio demographic characteristics were similar between the two groups. Patients with urban background had a higher score on the negative subscale (p=0,01) and the general psychopathology subscale (P=0,025) of the PANSS compared with rural patients. Otherwise, compared to patients with a rural birth and early living, urban patients showed more depressive symptoms on the Calgary depression scale (p=0,039). There were no significant differences between the two groups regarding the biological variables tested.
**Discussion:** The association between urban upbringing and risk for developing schizophrenia is well established. Nevertheless, relationship between epidemiological factors and different symptom dimensions is still poorly understood. Our study found a significant association between patients' urban background and negative as well as disorganization and depressive symptoms. Most of published studies found a correlation between urban upbringing and positive psychotic symptoms in schizophrenia. To our knowledge, no similar result has ever been found before. Otherwise, there were several studies showing an association between disorganization and depressive symptoms with urban upbringing of the patients.

### T125. INCIDENCE OF TREATMENT RESISTANCE SCHIZOPHRENIA IN A COMMUNITY SAMPLE USING THE TRRIP CONCENSUS

Dan Joyce*[,1], Rodrigo Bressan[2], Sukhi Shergill[3]
[1]*Institute of Psychiatry, King's College;* [2]*Universidade Federal de Sao Paulo - UNIFESP;* [3]*Cognition Schizophrenia and Imaging (CSI) Lab*

**Background:** Estimates of treatment resistant schizophrenia (TRS) vary due to lack of consensus definition. The Treatment Response and Resistance in Psychosis (TRRIP) consensus provides a rigorous prospective definition for TRS, but has not yet been applied to data. We provide the first prospective estimate of the incidence of TRS in a large community cohort using TRRIP.
**Methods:** The publicly available CATIE (Clinical Antipsychotic Trials of Intervention Effectiveness) data were mined using bespoke implementations of algorithms that operationalise the minimum TRRIP concensus criteria. Survival curves for transition to treatment resistance status (versus treatment responsive and censoring) were estimated. Inferential methods were used to establish baseline patient characteristics that are associated with TRS. Machine learning methods were also applied to estimate patient-level prediction of TRS status from baseline data.
**Results:** 1369 patients were included in the analysis, with 992 patients at risk for developing TRS at baseline. A total of 48 cases of TRS were identified, yielding a crude incidence of 36.2 cases per 1000 person years. There were no strong associations with baseline demographics or clinical state at enrolment to the trial and the predictive modelling failed to identify any patient-level predictor of future TRS.
**Discussion:** The CATIE trial protocol excluded patients with retrospective evidence of TRS, however, prospectively applying the TRRIP consensus revealed that there were patients with TRS in the cohort. Our results suggest a small incidence, and that baseline clinical and demographic data is not a robust predictor of future resistance status. Analysis of individual TRRIP criteria reveals a significant unmet need for patients with poor treatment response, but who do not meet criteria for TRS, particularly in social and occupational functioning.

### T126. PSYCHOTIC EXPERIENCES AND COMMON MENTAL DISORDERS IN CHILDHOOD AND ADOLESCENCE: BIDIRECTIONAL AND TRANSDIAGNOSTIC ASSOCIATIONS IN A LONGITUDINAL COMMUNITY-BASED STUDY

Pedro Pan*[,1], Giovanni Salum[2], Felipe Argolo[1], Ary Gadelha[1], Rodrigo Bressan[1]
[1] *Universidade Federal de São Paulo;* [2]*Universidade Federal do Rio Grande do Sul*

**Background:** The prevalence of Psychotic Experiences (PE) in the general population is approximately 7%. Several studies report on the association of PE with non-psychotic mental disorders and dimensional psychopathology. However, few have addressed this relationship during adolescence using longitudinal data. Here, we aim to explore bidirectional associations of PE and common mental disorder in youth in a 3-year follow-up community-based study. We hypothesized that there is a link between PE and depression, corroborating findings from adult studies, and that mental disorders comorbidity significantly correlates to PE, showing a nonspecific effect of PE as a risk for a broad "psychiatric load/liability".
**Methods:** We analyzed data from the Brazilian High Risk Cohort (HRC), a large multi-site school-based study. At baseline, we evaluated 2,244 subjects (6–12 years old) using the Community Assessment of Psychotic Experiences (CAPE) and an adapted version of the Comprehensive Assessment of At-risk Mental States (CAARMS) by self-report and clinician ratings, respectively. Mental disorders in youth were assessed by the Development and Well-Being Assessment (DAWBA). We grouped mental disorders into 4 DSM-based categories: any depressive disorder, any anxiety disorder, any Attention Deficit Hyperactivity Disorder (ADHD), and any Oppositional Defiant Disorder or Conduct Disorder (ODD/CD). Subjects were reassessed after 3 years, with a retention rate of 75%. We used regression analyses to explore predictors of PE and mental disorders at follow-up. Finally, we investigated the bidirectional effect of PE as a nonspecific psychiatric "load/liability" by creating count variables for the number of comorbid psychiatric disorders for each participant. Poisson regression models tested the effect of PE (as a predictor) in the count variable (the outcome) controlling for potential confounders.
**Results:** We found bidirectional associations between PE and mental disorders in youth. Baseline PE increased the risk of any depressive disorder at follow-up, and baseline ADHD was associated with PE at 3-year follow-up. Comorbidity analyses showed significant relationships in both directions, with an increased risk of PE according to the number of comorbid psychiatric disorders.
**Discussion:** We showed that subthreshold psychotic symptoms predict subsequent depressive disorder, and nonspecifically relate to psychiatric comorbidity. These findings are concordant with the notion that psychotic experiences are part of the same psychiatric vulnerability conferred to common mental disorders, such as depression and ADHD.
Our results may inform future research on testing subclinical psychotic symptoms to further our understating on identifying high-risk groups for early intervention.

### T127. OFFSPRING OF ANTENATALLY DEPRESSED MOTHERS AND PARENTS WITH SEVERE MENTAL DISORDER – A LONG FOLLOW-UP IN THE NORTHERN FINLAND 1966 BIRTH COHORT

Pirjo Mäki*[,1], Tiina Taka-Eilola[2], Graham Murray[3], Juha Veijola[1]
[1]*University of Oulu, Oulu University Hospital;* [2]*University of Oulu;* [3]*University of Cambridge*

**Background:** Depression during pregnancy is common, but long-term outcomes in the offspring of antenatally depressed mothers are unknown. Among severe mental disorders at least schizophrenia is considered to be a neurodevelopmental disorder acting already in utero with high genetic vulnerability. The aim was to study whether offspring of antenatally depressed mothers have an elevated risk for severe mood disorders till middle adulthood, taking account parental severe mental disorder.
**Methods:** The general population-based Northern Finland 1966 Birth Cohort includes 12,058 children, whose mothers were asked at mid-gestation if they felt depressed. The offspring were followed for over 40 years, and hospitalised severe mental disorders were detected using the Finnish

Hospital Discharge Register. It was also used for identifying severe mental disorders in the parents till 1984, when the offspring were of age.
**Results:** Of the mothers, 14% self-reported depression during pregnancy. Of the parents, 10% had suffered from a hospitalised severe mental disorder.
Adult offspring of antenatally depressed mothers had modestly increased risk for mood disorders both non-psychotic (crude OR 1.6; 95%CI 1.1–2.2) and psychotic (2.0; 1.0–4.1) but not for schizophrenia nor substance use disorder, when compared with the children of mothers without antenatal depression.
Maternal depression during pregnancy combined with parental severe mental disorder increased the risks for severe mental disorders in the offspring widely. The risks for both non-psychotic (crude OR 3.8; 95%CI 2.1–6.6) and psychotic mood disorder (5.4; 1.6–18.1) and also for schizophrenia (4.3; 2.3–8.2) and substance use disorder (2.8; 1.7–4.7) were higher in the offspring with both maternal antenatal depression and parental severe mental disorder than in those without maternal depression and with severe mental disorder in the parent (for non-psychotic 1.5; 1.0–2.4 and psychotic mood disorder 4.2; 1.9–9.2, for schizophrenia 1.3; 0.8–2.4 and for substance disorder 1.5; 1.1–2.2) or in those with a depressed mother but without parental severe mental disorder (for non-psychotic 1.3; 0.9–1.9, and for psychotic mood disorder 2.1; 0.9–5.0, for schizophrenia 0.9; 0.5–1.6 and substance disorder 1.4; 1.1–2.0). The reference group was birth cohort members without maternal antenatal depression and without paren¬tal hospital-treated mental disorder. The risks remained statistically significant even after adjustment for maternal smoking during pregnancy, perinatal complications, father's social class and family type at birth. In the offspring of antenatally depressed mother and a father with severe mental disorder the risk was elevated only for schizophrenia (7.5; 2.2–26.2).
**Discussion:** Maternal depression during pregnancy increased the risk for mood disorders in the offspring slightly but not for schizophrenia nor substance use disorder when compared with the children of mothers without antenatal depression. Maternal antenatal depression combined with parental severe mental disorder increased the risks for all of these severe mental disorders in the adult offspring.
The risk was highest for schizophrenia in the offspring of antenatally depressed mother and a father with severe mental disorder.
To our knowledge, this is the first study of mood disorders, schizophrenia and substance use disorder in the offspring of antenatally depressed mothers with long follow-up till middle age where familial vulnerability for severe mental disorders was taken into account in a general population-based sample.
**References:**
1. Mäki P, et al. Am J Psychiatry 2010;167(1):70–7,
2. Taka-EilolaT et al. Eur Psychiatry 2017; 42:36–43

### T128. THE ASSOCIATION BETWEEN GENETIC RISK FOR SCHIZOPHRENIA AND PATTERNS OF CIGARETTE AND CANNABIS USE IN ADOLESCENCE

Stanley Zammit*[,1], Hannah Jones[2], Suzanne Gage[3], Jon Heron[2], George Davey Smith[2], Glyn Lewis[4], Michael O'Donovan[1], Michael Owen[1], James Walters[1], Marcus Munafò[2]
*[1]Cardiff University; [2]University of Bristol; [3]University of Liverpool; [4]University College London*

**Background:** Schizophrenia is associated with a higher prevalence of cannabis use and cigarette use. However, it is unknown to what extent these associations are due to a shared genetic aetiology. We therefore aim to examine how schizophrenia genetic risk associates with patterns of cigarette and cannabis use in adolescence.
**Methods:** We analysed repeated measures of cigarette and cannabis use during adolescence in a sample of 5,300 individuals in the Avon Longitudinal Study of Parents and Children (ALSPAC) birth cohort who had at least 3 measures of cigarette and cannabis use between ages 14–19 years. Cigarette and cannabis use data were summarised using longitudinal latent class analysis to identify longitudinal classes of substance use, and associations between polygenic scores for schizophrenia and resulting classes were assessed.
**Results:** The schizophrenia polygenic score based on single nucleotide polymorphisms (SNPs) meeting a discovery sample threshold of $p \leq 0.05$ was associated with late onset cannabis use as compared to non-use (OR = 1.20; 95% CI = 1.05, 1.37) but not with early onset or cigarette only use latent classes. This association persisted after excluding the CHRNA5-CHRNA3-CHRNB4 nicotinic receptor gene cluster (OR = 1.25; 95% CI = 1.08, 1.44), a locus which has previously been found to strongly associate with schizophrenia.
**Discussion:** This study found that genetic risk of schizophrenia (as captured by polygenic scores) is associated with late-onset cannabis use but not with other smoking phenotypes in adolescence in ALSPAC. Possible explanations for these results are that schizophrenia and cannabis use have a shared genetic aetiology or that biological risk of schizophrenia leads to cannabis use through secondary mechanisms. These secondary mechanisms may include stress of childhood behavioural problems occurring as a result of biological processes underling schizophrenia. Future analyses involving mediation models may shed some light on factors influencing patterns of substance use in individuals with a high genetic liability for schizophrenia.

### T129. CHARACTERISTICS OF PREMORBID FUNCTIONING IN MALE ADOLESCENTS WHO LATER SUFFERED FROM PSYCHOTIC DISORDERS

Katya Rubinstein*[,1], Ortal Bhuknik-Atzil[1], Rivka Tuval-Maschiach[2], Eyal Fruchter[3], Avi Reichenberg[4], Mark Weiser[1]
*[1]Sheba Medical Center; [2]Bar-Ilan University; [3]Israeli Defense Forces; [4]Icahn School of Medicine at Mount Sinai*

**Background:** Previous research has shown that people with psychotic disorders have impaired functioning prior to the onset of the illness. The main goal of the proposed study was to deepen understanding of the characteristics of premorbid impairment in persons later diagnosed with psychotic disorders.
**Methods:** We examined unique premorbid data from IDF archives, including narrative summaries of pre-induction interviews of 17 years-old adolescents. This nested case-controlled study sample included two groups: 168 male adolescents who were later hospitalized for psychotic disorders, and 168 matched control subjects who were not diagnosed with psychotic illness during their military service. All subjects underwent pre-induction assessments between 2001 – 2010. The data were analyzed using mixed-method analysis, combining qualitative and quantitative research methods, in order to present an integrated characterization of pre-morbid functioning of future cases, compared to controls. Themes that arose from qualitative analyses, were conceptually divided into life conditions (for instance, death of a close person), and personal characteristics (i. e., mature, responsible). Each theme group was clustered into factors using categorical principal components analysis (CATPCA). Between-group comparisons on the identified factors were performed. Afterwards, the factors that were identified as significantly different in between-group comparisons, were included in a classification tree analyses to examine possible predictors of outcome.
**Results:** The analyses identified 5 factors in the "states" category: adaptation difficulties, negative family environment, suicidal thoughts and experience, medical conditions, and loss and instability in the family. In the "traits" category, 5 additional factors were identified: high-functioning, unpleasant interpersonal impression, interpersonal trust issues, strange impression, and low social skills. Future psychotic disorder patients, compared with matched controls, showed more premorbid adaptation difficulties. Their family environments were characterized with more serious medical or psychiatric conditions, experience of loss and instability, as well as family disruption and violence. Their personality traits were characterized by low interpersonal skills, while controls were described as more “high-functioning”.
**Discussion:** The current study partially replicated previously published findings and provided detailed description of the characteristics of environment, functioning and personal traits of people who experienced first outbreak of psychotic disorder, in the years before the outcome. Unlike most of the studies that focused on premorbid period, the current study used unique premorbid data and a combination of in-depth qualitative analyses, performed blinded to outcome, and novel machine learning techniques.

### T130. ASSOCIATIONS BETWEEN DIFFERENT TYPES OF CHILDHOOD ADVERSITY AND 5-YEAR OUTCOMES IN FIRST-EPISODE PSYCHOSIS PATIENTS

Olesya Ajnakina*[,1], Antonella Trotta[1], Marta Di Forti[1], Simona Stilo[1], Anna Kolliakou[1], Poonam Gardner-Sood[1], Javier-David Lopez-Morinigo[1], Fiona Gaughran[1], Anthony David[1], Paola Dazzan[1], Carmine Pariante[1], Valeria Mondelli[1], Robin Murray[1], Helen Fisher[1]
*[1]Institute of Psychiatry, Psychology and Neuroscience, King’s College London*

**Background:** Little is known about the impact of different forms of childhood adversity (CA) on outcomes in first episode psychosis (FEP) patients beyond the first year of treatment. We investigated associations between different types of CA and 5-year outcomes in a well-characterised sample of FEP patients.
**Methods:** A total of 237 FEP cases aged 18–65 years were followed on average for 5 years after first presentation to psychiatric services in South-London, UK. CA was assessed at service entry using the Childhood Experience of Care and Abuse Questionnaire. Using electronic clinical notes, extensive information was collected on clinical and social outcomes, service use, and self-injurious behaviours. As case analysis with missing data provides the most severely biased results we conducted multiple imputations to handle the missing data. We imputed the missing values using multiple imputations by chained equations (MICE). MICE has been shown to be a robust method for dealing with missing data across empirical and longitudinal studies.
**Results:** 72.1% of the sample reported at least one form of CA. Childhood parental separation was associated with greater likelihood of non-compliance with antipsychotic medications (OR=2.44, 95% CI=1.11–5.39), compulsory admission (OR=2.40, 95% CI=1.32–4.37), and living alone (OR=1.99, 95% CI=1.04–3.81) by the end of the follow-up. Institutional care was associated with longer total length of inpatient stay (IRR=1.34, 95% CI=1.01–1.79); parental death was associated with compulsory admissions (OR=2.87, 95% CI=1.02–8.05) during the follow-up.
**Discussion:** Our findings suggest some specificity in the detrimental impact of CA on service use and social functioning over a 5-year period following first contact with mental health services for psychosis. Clinicians should screen patients for CA and tailor interventions accordingly to improve outcomes.

### T131. INCIDENCE OF FIRST-EPISODE PSYCHOSIS IN THE CATCHMENT AREA OF RIBEIRÃO PRETO, BRAZIL

Cristina Del-Ben*[,1], Shuhama Rosana[1], Camila Loureiro[1], Taciana Ragazzi[1], Daniela Zanatta[1], Silvia Tenan[1], Jair Ferreira-Santos[1], Paulo Louzada-Junior[1], Antonio dos Santos[1], Craig Morgan[2], Paulo Menezes[1]
*[1]Ribeirão Preto Medical School, University of São Paulo; [2]Institute of Psychiatry, Psychology & Neuroscience, King’s College London*

**Background:** In the study “Schizophrenia and Other Psychoses Translational Research: Environment and Molecular Biology” (STREAM), we estimated the incidence of first-episode psychosis (FEP) in the catchment area of Ribeirão Preto, Brazil, and investigated the role of environmental and biological factors in the aetiology of the psychoses. STREAM is part of the international consortium “European Network of National Schizophrenia Networks Studying Gene-Environment Interactions” (EU-GEI).[1] Here, we describe the incidence of FEP in this Brazilian catchment area according to some demographic characteristics of the population.
**Methods:** Twenty-six counties compose the catchment area under study. Ribeirão Preto is the main city of the region, with a population of 604,682 inhabitants, population density of 929.8 inhabitants/Km2, per capita gross intern product (GIP) of U$ 9,143,20 and human development index (HDI) of 0.800, ranked 40th among 5,570 Brazilian municipalities. The population of the remaining 25 counties varied from 1,953 to 110,074 inhabitants (median=23,862), median population density of 75.0 inhabitants/Km2 (interval 13.2–308.4 inhabitants/ Km2), median GIP of U$5,254,80 (interval U$2,234,80–U$20,747,40), and HDI values ranging from 145th to 2,282nd in the national ranking. The sample was composed by individuals aged 16 to 64 years old, and with a first contact with mental health services due to psychotic symptoms in a 3-year period (1st April 2012 – 31st March 2015). Statistical analyses were carried out using Stata 13 software. We used the estimated population during the 3-year period based on the 2010 Brazilian Census to calculate person-years at risk. Incidence rates were estimated by sex, age, self-reported skin colour, and city of living.
**Results:** We identified 588 FEP patients over 3 years. The incidence rate of psychosis was higher in male (21.8, 95%CI=19.5–24.4) than female (18.1, 95%IC=16.1–20.5), non-white (20.9, 95%IC=18.1–23.9) than white patients (15.8, 95%IC=14.1–17.6), and those living in counties (22.3, 95%IC=20.1–24.5) other than the main city, Ribeirão Preto (17.3, 95%IC=15.2–19.6). The incidence rate of FEP declined with age.
**Discussion:** The proportion of patients reporting themselves as white (52.1%) was lower than that described for the catchment area under study (66.3%), confirming the higher risk of psychosis among those from minority groups.[2] The incidence rates of FEP observed in the Ribeirão Preto catchment area are roughly similar to those reported in São Paulo city[3] and in a large city in Southern Italy[4], but lower to those described in large urban centres in some European countries[5], which converges to the heterogeneity of the incidence of psychosis worldwide. We found a lower incidence rate of FEP in the main city of the catchment area, in comparison with the remaining cities of the region, which have a lower population density, but worse socioeconomic indicators. These preliminary results suggest an effect of socioeconomically deprived contexts in the incidence of psychosis in Brazil, as described in developed countries.[6] Further studies are needed to explore the environmental risk factors associated with differences in the incidence of psychosis in low and middle-income countries.

**References:**
1. van Os et al. Schizophr Bull. 2014; 40(4):729–36.
2. Jongsma et al. JAMA Psychiatry, in press.
3. Menezes et al. Br J Psychiatry Suppl. 2007:51:102–6.
4. Mulè et al. Soc Psychiatry Psychiatr Epidemiol. 2017;52(2):155-62
5. Kirkbride et al. Arch Gen Psychiatry. 2006;63(3):250–8.
6. Kirkbride et al. Am J Psychiatry. 2017;174(2):143–53.

### T132. ASSESSMENT OF CROSS-NATIONAL EQUIVALENCE OF THE COMMUNITY ASSESSMENT OF PSYCHIC EXPERIENCES (CAPE)

Baptiste Pignon*[,1], Hugo Peyre[2], Aziz Ferchiou[1], Jim van Os[4], Bart P. F. Rutten[3], Robin M. Murray[4], Craig Morgan[4], Marion Leboyer[3], Franck Schürhoff[1], Andrei Szöke[1]
*[1]INSERM; [2]Robert Debré Hospital; [3]University Medical Center Utrecht; [4]King’s College London*

**Background:** The Community Assessment of Psychic Experiences (CAPE) is a self-report questionnaire that has been developed to measure the dimensions of psychosis in the general population. The cross-national equivalence of a questionnaire allows the comparability of a scale across different populations in different countries, i.e., using different versions of the scale according to the considered language. In this study, our aim was to investigate the equivalence of the CAPE across different countries.
**Methods:** Data were drawn from the European Union Gene-Environment Interaction (EU-GEI) study. Participants (incident case of psychotic disorder, controls and siblings of cases) were recruited across in six countries: United Kingdom, the Netherlands, Italy, Spain, Brazil and France. To analyse the cross-national equivalence of the dichotomised version of the CAPE, we used the multigroup categorical confirmatory factory analysis (MCCFA). The cross-national equivalence can be stated after the establishment of three invariances characterised by increased constraints: the configural invariance, the metric invariance and the scalar invariance across the multiples groups.
**Results:** The configural invariance model fits well, providing evidence for identical factor structure across countries. The assumption that factor loadings are identical across countries is granted based on the negligible change in the fit indices in the metric invariance model. Moreover, the fit indices suggest that the CAPE shows scalar invariance across countries.
**Discussion:** These findings suggest that comparisons across countries of factor and observed means of the CAPE are possible. Thus, differences observed in scores between samples from different countries can be considered as different levels of psychosis.

### T133. MENTAL HEALTH OF ASYLUM SEEKERS AT THE HUMANITARIAN CAMP IN PARIS

Andrea Tortelli*[,1], Florence Perquier[2], Norbert Skurnik[3], René Wulfman[2], Marina Ibad-Ramos[2], Andrei Szoke[1], Alain Mercuel[2]
*[1]INSERM; [2]GHT Paris; [3]EPS Maison Blanche*

**Background:** Asylum seeker status is associated to higher prevalence of psychiatric disorders due to not only pre-migration events but also to the trajectory of migration and post- migration adversities. Mental problems and need of care may vary according to these different stages. In France, after the cleaning of the “Calais jungle” in October 2016, most asylum seekers moved to Paris, where they live as homeless. An asylum seeker center (CPA- Centre Premier accueil) was then created a few weeks later, to allow rapid access to asylum seeker procedure, shelter, and medical care (including mental health).
**Methods:** We will analyze 1- year data (socio-demographic characteristics, migration history, psychiatric symptoms, hospital admissions and medical prescriptions) of about 1000 recently arrived asylum seekers who consulted for psychiatric examination in the refugee camp (CPA). Prevalence and bivariate analyses will be done. Factors associated with mental health problems will be identified using multivariate analyses.
**Results:** Findings will be discussed in the light of the French/European immigration policy and the organization of the public mental health system.
**Discussion:** The results of our study will contribute to the identification of mental health problems and correlates at the arrival of asylum seekers in France, in the aim to develop adequate service planning, prevention and support.

### T134. EXPOSURE TO NITROGEN DIOXIDE DURING CHILDHOOD IS ASSOCIATED WITH INCREASED RISK OF SCHIZOPHRENIA

Sussie Antonsen[1], Pearl L.H. Mok[2], Roger T. Webb[2], Preben Bo Mortensen[1], John McGrath[3], Esben Agerbo[1], Jørgen Brandt[4], Camilla Geels[4], Carsten Pedersen*[,4]
*[1]Aarhus University, The Lundbeck Foundation Initiative for Integrative Psychiatric Research; [2]The University of Manchester, Manchester Academic Health Science Centre (MAHSC); [3]Aarhus University, The Lundbeck Foundation Initiative for Integrative Psychiatric Research, University of Queensland; [4]Aarhus University*

**Background:** Urban-rural differences in schizophrenia incidence have been reported from numerous published studies. Though as yet unknown, the underlying causes responsible for these differences have been postulated to include urban-rural differences in pollution, diet, infections, stress, or selective migration. Exposure to the air pollutant nitrogen dioxide (NO2) has been linked with increased rates of mortality, lung cancer, and psychotropic medication prescribing. However, no study to date has examined whether NO2 exposure during early childhood is associated with schizophrenia risk.
**Methods:** Utilizing individual-level information in the rich Danish population-based registers enriched with longitudinal information on residential exposure to air pollution, we investigated the putative link between NO2 exposure during childhood and schizophrenia risk. For each cohort member, exposure to NO2 was estimated longitudinally from birth to 10th birthday, expressed as mean of daily exposures at residence across the first 10 years of life. Incidence rate ratios per 10μg/m3 increase in mean NO2 exposure were estimated using survival analysis techniques. Air pollutant exposures were modelled using the UBM model in 1*1km grids covering Denmark from 1979 onwards.
**Results:** We observed a dose response relationship between childhood NO2 exposure and elevated risk for developing schizophrenia. Risk increased 1.36 fold (95%CI: 1.31–1.41) per 10μg/m3 increase in mean NO2 exposure during childhood. This association remained materially unaltered when adjusted for potential confounders such as family socioeconomic position and history of severe mental disorders. In absolute risk terms, at age 35 a person exposed to more than 25μg/m3 mean NO2 faced a 1.4% risk of schizophrenia whereas persons exposed to less than 10μg/m3 per day faced a 0.8% risk.
**Discussion:** This is the first population-based study demonstrating that exposure to NO2 during childhood is linked with elevated schizophrenia risk. The potential mechanism of NO2 on the risk of schizophrenia remains to be identified, however, if causality is proven this finding offers great potential for prevention of some cases of schizophrenia.

### T135. NEIGHBORHOOD-LEVEL PREDICTORS OF AGE AT ONSET AND DURATION OF UNTREATED PSYCHOSIS IN FIRST-EPISODE PSYCHOSIS

Benson Ku*[,1], Luca Pauselli[2], Marc Manseau[3], Michael Compton[4]
*[1]Hofstra Northwell School of Medicine; [2]Columbia University, New York State Psychiatric Institute; [3]New York State Office of Mental Health; [4]Columbia University*

**Background:** Recently, there has been increasing interest in the role of the social environment in the development and outcomes of schizophrenia. We investigated whether or not several neighborhood characteristics would be associated with two important prognostic factors in early-course psychosis, age at onset of psychosis (AOP) and duration of untreated psychosis (DUP). It is known that certain risk factors such as gender, family history, and history of cannabis use are associated with an earlier AOP and other risk factors such as history of being incarcerated, history of cannabis use, and mode of onset of psychosis (MOO) are associated with longer DUP. We sought to determine whether neighborhood characteristics would also be risk factors for these two prognostic indicators.
**Methods:** Data were collected as part of the Atlanta Cohort on the Early Course of Schizophrenia project, which included patients hospitalized for a first episode of a schizophrenia-spectrum disorder. Diverse variables were obtained from interview-based measures, data from chart reviews, and informant/family member collateral interviews, including gender, family history of psychosis, history of cannabis use, history of incarceration, MOO, patient-level residential mobility, Neighborhood Disorder Scale (NDS), AOP, and DUP. We retrieved 13 neighborhood characteristics using census tract-level data from the American Community Survey linked to individual addresses. Factor analysis with orthogonal rotation produced four neighborhood-level factors. With the addition of NDS, five independent variables were entered into two separate linear regression models with AOP and DUP as the dependent variables respectively; final models were derived from stepwise backward elimination, controlling for known predictors of AOP and DUP, and individual-level socioeconomic variables.

**Results:** Reliable census tract data were available for 143 participants. For the linear regression model pertaining to AOP, after stepwise backward elimination, the remaining independent predictor was neighborhood-level residential instability (β=-0.210; p=0.018). This variable remained significant after controlling for known risk factors such as gender, family history, and age at first cannabis use (β=-0.237; p=0.017) and after controlling for patient-level residential mobility (β=-0.195; p=0.031). Regarding the linear regression model for DUP, after stepwise backward elimination, the remaining independent predictors were the General Socioeconomic Status neighborhood factor (β=0.269; p=0.007), the Low Household Value neighborhood factor (β=0.190; p=0.046), and NDS (β=0.339; p<0.001). After controlling for known predictors of DUP, including MOO, history of incarceration, and age at first cannabis use, NDS and MOO remained significant (NDS: β=0.326; p=0.008; MOO: β=0.466; p<0.001).

**Discussion:** We found initial evidence that neighborhood-level characteristics are associated with important outcomes that affect the prognosis of early psychosis. Residential instability was associated with an earlier AOP and perceived neighborhood disorder was associated with a longer DUP. The association between the social environment and prognostic factors, widely explored in other health conditions, may have significant implications on the understanding and management of psychosis and should be further explored. Area-level context, beyond the individual level, might be considered when implementing services and policies that could contribute to improved outcomes among those with early schizophrenia.

### T136. DO VITAMIN D SUPPLEMENTATION DURING THE FIRST YEAR OF LIFE PREDICT COGNITION IN PSYCHOSES DURING MIDLIFE?

Jussi Seppälä*[,1], Laura Tröger[2], Erika Jääskeläinen[2], Jouko Miettunen[2], Matti Isdohanni[2], Marianne Haapea[2]
*[1]Carea, Psychiatric Services; [2]University of Oulu*

**Background:** Schizophrenia (SCZ) has been associated with cognitive impairment. The lack of vitamin D was associated with over 2-fold risk for mild cognitive impairment, and vitamin D could also associate with cognitive performance which may be explained by the role of vitamin D in the development of central nervous system or in neuroprotection. Vitamin D supplementation during the first year of life has been associated with a reduced risk of SCZ in males within the Northern Finland Birth Cohort 1966(NFBC 1966), but no studies have examined it`s possible association with cognition in SCZ during midlife. The aim of this study was to examine the association of vitamin D supplementation during the first year of life with the cognition at the age of 43 years separately among those having psychosis and among non-psychotic controls in prospective NFBC 1966.

**Methods:** The study is based on the NFBC 1966 concerning 12.058 live-born children in 1966 in Northern Finland. The final study population of this study (N= 257) consisted of 60 persons with schizophrenia spectrum disorder (SSD) and of 26 individuals with non-schizophrenic psychoses (NSSD) while 171 non-psychotic participants formed the reference group.

The daily dose of vitamin D was calculated based on the concentration of vitamin D in the product used and the reported amount of the product consumed. At the time when cohort was born, the recommended dose of vitamin D was 2000 IU/day. Based on maternal interviews in the year after birth, two measures of vitamin D supplementation were available: (a) frequency of intake (coded as regularly or irregularly/none) and (b) dose of vitamin D (<1600 IU/day, 1601–2000 IU/day, or >2000 IU/day.

The following tests were performed at the age of 43 years: California Verbal Learning Test (CVLT), Abstraction Inhibition and Working Memory Task (AIM), Visual Object Learning Test (VOLT), Vocabulary (WAIS-III), Visual Series (WMS-III), Digit Span (WAIS-III), Grooved Pegboard, Matrix Reasoning and Verbal Fluency.

**Results:** The study population (N= 2579 included 60 subjects with SSD, 26 persons had NSSD, and 171 non-psychotic controls formed the reference group. There were more men among those having psychosis (52.3% vs. 47.7%, respectively while the control group had more women (49.7 vs. 50.3, respectively). Only 13.2% of participants in the entire study population had received vitamin D supplementation irregularly or not at all. On the other hand, 5.1% had taken vitamin D supplementation more than the recommended dose. Because the number of those who got vitamin supplementation under recommended dose (<2000IU/day) was not more than 3 persons (1.2% of the whole study population), the association of the dose vitamin D supplements with later cognition was not analyzed. Therefore, the frequency of vitamin D supplementation (coded as regular or irregular/none) was utilized in final analyses. The frequency of vitamin D supplementation was not associated with cognition in midlife either among those having psychosis or in the control group (p-values for global cognitive performance in psychoses and controls were 0.89 and 0.61, respectively).

**Discussion:** The main finding of this study was that no association was found between the frequency of vitamin D supplementation during first year of life and cognition in midlife either among those having psychosis or in the control group.

The only earlier study (N=9.114) evaluating a link between the use of nutritional supplements during early life and risk of SCZ was carried out in NFBC 1966. In males, the use of either irregular or regular vitamin D supplements was associated with a reduced risk of SCZ compared with no supplementation.

### T137. CLASSIFICATION OF RECENT-ONSET PSYCHOSIS BASED ON RESTING-STATE FUNCTIONAL CONNECTIVITY AND THE RELATIONSHIP TO NEUROCOGNITIVE IMPAIRMENT

Johanna Weiske*[,1], Anne Ruef[1], Shalaila Haas[1], Carolina Bonivento[2], Nikolaos Koutsouleris[1], Lana Kambeitz-Ilankovic[1]
*[1]Ludwig Maximilians University, University Psychiatric Hospital; [2]University of Udine*

**Background:** Impairments in cognitive functioning are a core feature of psychotic disorders and they have been associated with resting-state functional connectivity (rsFC) alterations in patients suffering from psychosis (Dauverman et al., 2014). Multivariate pattern analysis (MVPA) has proven to be a useful tool in the investigation of rsFC alteration in psychosis and in detecting subtle differences in multidimensional data sets (Kambeitz et al., 2015). In this study, we differentiated recent-onset psychosis patients (ROP) from healthy controls (HC) using a Support Vector Machine (SVM) classification based on rsFC. Furthermore, we investigated the relationship of the discriminative rsFC pattern to neurocognitive measures.

**Methods:** Resting-state fMRI and neurocognitive measures were obtained from 220 HC and 115 ROP across 7 sites of the PRONIA consortium. The rsFC matrix was estimated for each subject by calculating pairwise correlations between mean time series of 90 brain regions based on AAL parcellation. A L1-regularized L2-loss SVM was trained to classify ROP vs. HC based on rsFC in a repeated nested cross-validation. Decision scores for ROP were correlated with cognitive measures derived from the following neuropsychological tests: Rey Auditory Verbal Learning Task (RAVLT), Phonetic and Semantic Verbal Fluency, Diagnostic Analysis of Nonverbal Accuracy, Forward and Backward Digit Span, Self-ordered Pointing Task, and Salience Attribution Test.

**Results:** The classification algorithm was able to differentiate ROP and HC with a balanced accuracy (BAC) of 71.3% based on rsFC. The discriminative connectivity pattern included short-range connections between left putamen and left hippocampus, right putamen and right caudate nucleus, left superior frontal and right inferior orbitofrontal regions, as well as long-range connections between left and right occipital cortex and left cingulate gyrus, left supramarginal gyrus and right temporal pole. Two negative correlations between the SVM decision scores for ROP and measures of the RAVLT were significant (delayed recall: r=-0.3, Bonferroni –adjusted p<.04; recall after interference: r=-0.3, Bonferroni-adjusted p<.02).
**Discussion:** The classification performance was driven by a rsFC pattern including areas involved in memory processing, such as hippocampus and cingulate gyrus (Allen et al., 2007) as well as regions related to language processing, such as the supra marginal gyrus (Li et al., 2009). The negative correlation of rsFC-based decision scores with RAVLT measures shows that patients whose verbal learning and memory is more severely impaired exhibit a more distinctive rsFC pattern than patients with less impaired verbal memory.

### T138. ACOUSTIC PATTERNS IN SCHIZOPHRENIA: A SYSTEMATIC REVIEW AND META-ANALYSIS

Alberto Parola*[,1], Arndis Simonsen[2], Vibeke Bliksted[2], Riccardo Fusaroli[2]
*[1]University of Turin; [2]Aarhus University*

**Background:** Individuals with schizophrenia are characterized as presenting atypical voice patterns: poverty of speech, increased pauses, distinctive pitch (mean and variability). Voice atypicalities may play a role in the social impairment experienced by patients, and could constitute a window into motor, cognitive, emotional and social components of the disorder. Indeed, they have already been generally associated with negative symptoms. However, the state of the evidence for atypical voice patterns and their relation to clinical features is uncertain. Studies using clinical rating scales indicate that voice alterations are severe across many voice properties. In contrast, quantitative acoustic studies seem to have found less robust and more variable results limited to specific features. We therefore systematically reviewed the literature quantifying acoustic patterns in schizophrenia, and performed a meta-analysis of the evidence. We aimed at identifying evidence for acoustic markers of schizophrenia and its clinical features, needs for further research and barriers to collective advancements on these issues.
**Methods:** We adopted the “PRISMA Statement” guidelines for transparent reporting of a systematic review. The literature search was conducted on Pubmed and Google Scholar (details and pre-registration at https://goo.gl/H1yDpm). Study selection was conducted according to the following inclusion criteria: (a) empirical study, (b) quantification of acoustic features in the vocal production of participants with schizophrenia, (c) sample including at least two individuals with schizophrenia, (d) inclusion of a comparison group, or an assessment of variation in acoustic features in relation to severity of clinical features. We identified 54 studies as eligible for inclusion and contacted all authors to obtain missing estimates and individual-level data, where possible. 34 studies availed enough information to be included in a meta-analysis. The meta-analysis consisted of mixed effects regression models, one per each relevant acoustic feature.
**Results:** Of the 37 authors contacted, 59% responded and 5% provided at least some of the requested data. Chief reasons of denials were: i) data loss (n = 8), ii) effort required (n = 5), iii) ethical concerns with data sharing (n = 1). On the results available we found significant meta-analytic effects of schizophrenia in percentage of spoken time (n = 6, d = -1.16, 95% CIs: -2.06 -0.27) and proportion of pauses (n = 5, d = 0.56, 95% CIs: 0.15 0.96). After controlling for influential studies, we found significant differences also in pitch mean (n = 5, d = 0.40, 95% CIs: 0.12 0.68) and pitch variability (n = 6, d = -0.46, 95% CIs: -0.70 -0.23). No effects were found for pause duration (n = 7), speech rate (n = 9), speech duration (n = 5) and pitch intensity (n = 5). We found evidence for publication bias for studies investigating pause duration and pitch variability.
Key concerns on the meta-analysis are: i) small sample sizes, ii) heterogeneity of task and acoustic processing methods, iii) lack of demographic and clinical individual-level data necessary to control for confounds (e.g. medication and relation to clinical features).
**Discussion:** We found clear effects of increased pause behavior in schizophrenia and less clear effects of pitch. However, the magnitude of these abnormalities is limited and contrast with the large effect sizes reported by studies using clinical rating scales. Future research should focus on larger sample sizes, systematic assessment of multiple acoustic features and multiple speech tasks, standardized acoustic processing methods, and individual level data available. More reflection is needed on how to make data sharing possible within privacy and ethical constraints.

### T139. ELECTRORETINOGRAM ABNOMALIES IN SCHIZOPHRENIA PATIENTS WITH VISUAL HALLUCINATIONS

Vincent Laprevote*[,1], Florent Bernardin[1], Thomas Schwitzer[1], Raymund Schwan[1]
*[1]Centre Psychothérapique de Nancy*

**Background:** Retinal dysfunctions have been integrated in cognitive models of visual hallucinations in several pathologies such as Parkinsonian syndromes or eye diseases. Besides, structural abnormalities of the retinal ganglion cells are documented in schizophrenia and have been associated to visual hallucinations (VH) in neurological disorders. We aim to study functional abnormalities of retinal ganglion cells in schizophrenia patients with VH.
**Methods:** We measured the activity of retinal ganglion cells using electroretinogram according to ISCEV criteria. We compared the amplitude and implicit time of the P50 and the N95 waves of the pattern electroretinogram in schizophrenia patients with VH (VH group, n = 7), Auditory Hallucinations or no hallucination (AH/NH group, n = 8) and controls (n = 30).
**Results:** Preliminary findings show a significant increase of the N95 implicit time in the HV group compared with controls (p = .05). No difference was found between the HV and HA/NH groups but a gradient appeared to emerge between the 3 groups.
**Discussion:** Functional impairment of the retinal ganglion cells appears to be more pronounced in schizophrenia patients with HV. The increase of N95 implicit time may be interpreted as a dysfunction of retinal ganglion celles rather than a cell loss. These preliminary results need to be confirmed with a larger sample.

### T140. RESTING STATE NETWORKS ALTERATION IN SCHIZOPHRENIA

Gianluca Mingoia*[,1], Igor Nenadic[2]
*[1]RWTH Aachen University; [2]Jena University Hospital*

**Background:** While functional MRI and PET studies have shown altered task-related brain activity in schizophrenia, recent studies suggest that such differences might also be found in the resting state (RS). Here we used ICA based analysis to investigate RS fMRI data to compare connectivity of 11 well known networks (Auditory, Cerebellum, DMN, Exectutive Control, Fronto-parietal 1, Fronto-parietal 2, Salience, Sensorimotor, Visual1, Visual2, Visual3 network) between patients with schizophrenia and healthy controls suggesting deficits in related neuropsychological functions.
**Methods:** We obtained RS fMRI series (3T, 3x3x3mm resolution, 45 slices, TR 2.55s, 210 volumes) in 25 schizophrenia patients (mean age 30a±7.3), on stable antipsychotic medication and 25 matched healthy controls (30.3a±8.6). Subjects were asked to lie in the scanner keeping eyes closed with no further specific instructions. Data were pre-processed; we applied

FSL MELODIC (pICA) yielding IC, we used FIX to auto-classify ICA components which represent artifacts and an automated routine to select for each subject the component matching the anatomical definition of resting state networks. SPM12 was used for second level analysis, we used two sample t-test to compare networks functional connectivity between groups. We then analysed the power spectrum density (PSD) for the extracted networks, estimating the power of the signal at different frequencies.

**Results:** Our method reliably identified all networks in every control and patients. We found significant differences in the anatomical pattern of areas. Patients showed decreased functional connectivity in comparison to healthy controls in portions of Cerebellum, DMN, Executive Control, Fronto-parietal1 and sensorimotor networks; in addition patients showed increased functional connectivity in comparison to healthy controls in portions of DMN network. Finally, PSD was found altered in patients with Schizophrenia when compared to healthy controls in Fronto-parietal1, Sensorimotor, visual1and visual2 networks.

**Discussion:** Well known resting state networks were reliable identified from RS fMRI in Schizophrenia patients. The differences in anatomical distribution point to possible alterations in functional connectivity in Schizophrenia, which suggests disruption in Cerebellum, DMN, Executive control, Fronto-parietal and sensorimotor activity. The comparison of the PSD suggests changes in Fronto-parietal1, Sensorimotor and in addition visual1, visual2 networks.

### T141. CHARACTERIZATION OF HEMODYNAMIC ALTERATIONS IN SCHIZOPHRENIA AND BIPOLAR DISORDER AND THEIR EFFECT ON RESTING-STATE FUNCTIONAL CONNECTIVITY

Wenjing Yan[1], Lena Palaniyappan*[,2], Peter Liddle[3], Gopikrishna Deshpande[1]

*[1]Auburn University; [2]University of Western Ontario; [3]University of Nottingham*

**Background:** Schizophrenia (SZ) and bipolar disorder (BP) have both common and distinct clinical symptomatology. Their neural bases have been explored using functional connectivity between brain regions using resting-state functional magnetic resonance imaging (rs-fMRI). However, fMRI is an indirect measure of neural activity and is modeled as a convolution of the hemodynamic response function (HRF) and latent neural activity. The HRF varies across both individuals and different brain regions within an individual. Consequently, it is plausible for two brain regions to appear synchronized in the BOLD space while being desynchronized in latent neural space and vice versa.

**Methods:** In order to address this issue, we estimated voxel-specific HRFs by deconvolving rs-fMRI time series obtained from SZ (N=19), BP (N=35) and matched healthy individuals (N=34). The shape of the HRF was significantly different between the three groups in many regions previously implicated in SZ and BP. Specifically, we found voxels within the mediodorsal, habenular and central lateral nuclei of the thalamus to have HRFs with aberrations in all three of its shape parameters: time to peak, response height and full width half max. Therefore, we defined this region as the seed, estimated seed-based functional connectivity maps in all three groups and characterized pairwise differences between them. Further, we performed a 2-way ANOVA and estimated regions exhibiting an interaction between the group and deconvolution factors.

**Results:** We found voxels within the mediodorsal, habenular and central lateral nuclei of the thalamus to have HRFs with aberrations in all three of its shape parameters: time to peak, response height and full width half max. Results indicated that functional connectivity differences between the groups are inferred significantly differently with raw BOLD and deconvolved latent neural time series. Since the variability of the HRF could be driven by both neural and non-neural factors, we feel that it is preferable to estimate functional connectivity using deconvolved data.

**Discussion:** Neurochemicals such as GABA, glutamate, serotonin and nitric oxide have a role in controlling neurosignaling pathways underlying neurovascular coupling and hence the HRF. Previously documented alterations of these neurochemicals in SZ and BP could, at least in part, explain the significant differences in HRF shapes observed between the groups. Functional connectivity group differences obtained from raw BOLD data must be interpreted cautiously in the light of systematic HRF differences between groups.

### T142. PARIETAL CONNECTIVITY IN SCHIZOPHRENIA AND PSYCHOTIC BIPOLAR DISORDER: A COMBINED STRUCTURAL AND DYNAMIC FUNCTIONAL CONNECTIVITY STUDY

Tushar Das[1], Peter Liddle[2], Lena Palaniyappan*[,1]

*[1]University of Western Ontario; [2]University of Nottingham*

**Background:** The role of parietal lobe in psychotic disorders is poorly understood. In independent previous studies, we have observed that (1) the severity of disorganization is associated with reduced cerebral blood flow to bilateral parietal angular gyrus in patients with schizophrenia (2) disorganization is more pronounced in patients who have morphological abnormalities in left parietal supramarginal gyrus (3) the global connectivity of right parietal supramarginal region is reduced in schizophrenia compared to bipolar disorder with psychosis. We aimed to delineate the nature of parietal dysconnectivity in the 2 major psychotic disorders and to study the relationship between the syndrome of disorganization and structural and functional connectivity of the parietal lobe with rest of the brain. We also related parietal connectivity to the global assessment of functional status (GAF scores) and processing speed scores among patients with schizophrenia.

**Methods:** We recruited 16 subjects with psychotic bipolar disorder and 34 subjects with established schizophrenia, age- and sex- matched with 32 healthy controls. Both patient groups were medicated, and were in a clinically stable state. Diffusion Tensor Imaging (DTI) and resting state fMRI data were obtained using a 3T MRI scanner. Using 90 regions as defined in the AAL atlas, deterministic tractography was performed (FSL v5.0 and TrackVis). For each of the 90*90 connections, fractional anisotropy weighted by number of streamlines, and normalised by average fiber length was used as the index of structural connectivity. 90*90 functional connectivity values were also obtained for each subject using the fMRI data (SPM8 and DPARSFA). Dynamic connectivity (variance) was estimated using a sliding window approach (13 bins; 240 time points; TR=2.5s). The primary variable of interest across the 2 imaging modalities was the graph metric of weighted average degree from all parietal lobe nodes in the AAL atlas with all other nodes of the brain. Using ANOVA, we compared the degree of parietal connectivity among the 3 groups of subjects. Three multiple regression analyses were conducted to assess relationships between parietal connectivity (degree of right and left structural and dynamic functional connectivity) and severity of disorganisation, processing speed (digit symbol substitution test -DSST) and GAF scores.

**Results:** The 3 groups differed significantly on the degree of left parietal structural connectivity (F=6.5, p=0.002, HC>BIP=SCZ) and on the degree of left (F=6.4, p=0.003; BIP=HC>SCZ) as well as right parietal (F=5.2, p=0.008; BIP=SCZ>HC) dynamic functional connectivity. Parietal dysconnectivity predicted the severity of disorganisation (model F=4.1, p=0.01) in SCZ. Disorganization was particularly associated with reduced left parietal structural ($\beta$=-0.45, p=0.02) and dynamic connectivity ($\beta$=0.40, p=0.04) but not with the right parietal dysconnectivity. DSST scores were associated with reduced left parietal structural connectivity ($\beta$=0.44, p=0.04). GAF was increased in patients with higher right parietal dynamic functional connectivity ($\beta$=0.38, p=0.04).

**Discussion:** Both structural and dynamic functional parietal dysconnectivity are seen in the 2 major psychotic disorders - schizophrenia and bipolar disorder. Left-right asymmetry in parietal dysconnectivity is notable, especially among patients with schizophrenia. Parietal dysconnectivity plays a role in the processing speed as well as global functioning deficits in schizophrenia. Taken together, these findings suggest that the degree of connectivity of parietal lobe could be an important determinant of symptom burden, specific cognitive deficits as well as functional capacity in psychotic disorders.

### T143. NOT A NUISANCE ANY MORE: GLOBAL FMRI SIGNAL AT REST, PROCESSING SPEED AND SYMPTOM SEVERITY IN SCHIZOPHRENIA

Annabel Umeh[1], Peter Liddle[2], Susan Fancis[2], Lena Palaniyappan*[,3]

*[1]University College Cork; [2]University of Nottingham; [3]University of Western Ontario*

**Background:** Before data from resting-state functional magnetic resonance imaging (rs-fMRI) is analyzed, the global signal (GS) - average blood-oxygen level dependent (BOLD) signal across all voxels in the brain - is normally removed through global signal regression (GSR). This convention arose in order to control for changes in brain activity that are usually of no interest but may be caused by non-neuronal factors, including changes in respiratory rate, arterial CO2 levels or cardiac pulsation. However, recent studies have indicated that GS may systematically vary between patients with schizophrenia and healthy controls. In addition to testing this notion, we also studied if the dynamic variance of global signal across time carried any meaningful information that relates to overall symptom severity and information processing speed in patients with schizophrenia.
**Methods:** Data was collected from 39 in a clinically stable, medicated early stage of schizophrenia (median duration of illness= 6.5 years), and 34 sex, age and parental socioeconomic-status matched healthy controls over a 10-minute period of eyes-open rest at TR=2.5s (3T Philips Achieva, 240 time points, dual-echo, gradient-echo EPI). Scores were obtained from the Signs and Symptoms of Psychotic Illness (SSPI) scale and the Digit Symbol Substitution Test (DSST). Rs-fMRI time-series data were motion-corrected (using 6 parameters), slice-time corrected, reoriented with structural images, band-pass filtered (0.01–0.1 Hz), scrubbed using ArtRepair for framewise displacement and transformed to MNI space. SPM8 and the advanced version of the Data Processing Assistant for resting-state fMRI (DPARSFA) were used for this purpose. GS mean was computed from all grey matter voxels using a template mask in MNI space, using grand mean scaling to a base of 1000, averaged across all time-points. The variance of GS across time (dynamic GS variance) was computed from the entire 10-minutes of acquisition (240 time points).
**Results:** Independent-sample t-tests used to compare GS mean between controls (mean[sd] = 3135.1[1244.4]) and the SCZ group (mean[sd] = 3207.8[1191.7]) yielded no significant results [t(71) = -0.14, p = .89]. The temporal variance of GS did not differ between controls (mean[sd] = 113.05[59.41]) and the SCZ group (mean[sd] = 118.02[57.93]) [t(71) = -0.36, p = .72]. In the SCZ group there was a significant correlation between the total SSPI score reflecting overall illness severity (□ = -.322, $p < 0.05$) and the mean GS. This relationship was especially pronounced for the syndrome of Reality Distortion (rho = -.344, $p < 0.05$) and Disorganization (rho = -.303, p = 0.065), where higher symptom severity was seen in patients with lower mean GS.
Dynamic variance in GS was higher in healthy controls with lower mean DSST (r = -.364, p = 0.04), but no such relationship was seen in the SCZ group (r = .066, p = .694). Notably, when compared to controls (mean[sd] = 57.4[9.40]), patients (mean[sd]=42.4[9.97]) had significantly lower DSST scores [t(69) = 6.47, $p < 0.001$]. Neither GS nor GS variance related to root mean square of framewise displacement in the 2 groups.
**Discussion:** We did not find an overall increase or reduction in the global signal in patients with schizophrenia. Nevertheless, the strength of global signal obtained from resting fMRI is related to severity of persisting symptoms of schizophrenia, whereas the dynamic variance of this signal relates to the speed of processing ability assessed outside the scanner in healthy subjects. With emerging evidence relating global signal to cognitive vigilance and overall brain connectivity, our results indicate that global signal is a parameter of interest that should not be automatically discarded in resting fMRI studies of schizophrenia

### T144. THE ROLE OF TRANSIENT BETA OSCILLATIONS IN ABERRANT SELECTIVE ATTENTION TO SALIENT EVENTS IN SCHIZOPHRENIA

Elizabeth Liddle[1], Jyothika Kumar[1], Siân Robson[1], Emma Hall[1], Lauren Gascoyne[1], Mohammad Katshu[1], Lena Palaniyappan[2], Peter Morris[1], Matthew Brookes[1], Peter Liddle*[,1]

*[1]University of Nottingham; [2]University of Western Ontario*

**Background:** Selective attention to situationally salient information is aberrant in schizophrenia. Following the presentation of behaviourally relevant stimuli, oscillatory power in the beta-band (13-30Hz) typically decreases (Event-Related Desynchronisation – ERD) then increases (Event-Related Synchronisation – ERS). The ERD-ERS pattern is a potential marker for the processing of behaviourally salient events. In a previous magnetoencephalography (MEG) study (Liddle et al Hum. Brain Mapp. 2016; 37:1361–74) we found that in people with schizophrenia, ERS was reduced. Recently, Jones (Curr. Opin. Neurobiol, 2016; 40: 72–80) proposed that the relatively continuous beta-synchronisation observed in trial-averaged data may reflect the probability distribution of transient beta events discernible in single trial data. She cited both animal and human data consistent with a neural model in which these beta bursts are generated by transient input to pyramidal neurons via distal dendrites concurrent with input to deeper layers presumed to be from thalamus. External stimuli are less likely to be perceived during the time period immediately following a transient beta event. The model is consistent with the hypothesis that transient beta bursts are an index of top-down modulation of the processing of perceptual information, and raises the possibility that aberrant control over this modulation might contribute to aberrant selective attention in schizophrenia. We hypothesized that in relevant trials, the beta-burst probability distribution would be skewed towards the latter part of the trial, reflecting a period of suppressed beta-burst probability, and thus of enhanced stimulus perception, followed by a period of increased burst probability, possibly reflecting sensory suppression following stimulus processing.
**Methods:** We recorded MEG data in 23 patients with schizophrenia and 37 healthy controls during the performance of a relevance modulation task designed to assess neural effects of situational salience. Data were recorded using a 275-channel CTF system (Coquitlam, Canada). Visual stimuli that were either task-relevant or task-irrelevant were presented in alternating, predictable, order. Beamformed data time courses were computed for 8 previously defined brain networks. Time-frequency spectrograms were computed for each trial, from 0 to 1500 ms following stimulus presentation. A 2-D peak-detection algorithm was used to identify transient increases in oscillatory power. The time point of any peak occurring within the beta band (~15–25 Hz) was recorded, and the median of these time-points computed for each trial. These medians were averaged within each participant for each trial type (relevant; irrelevant) as a measure of central tendency of the probability distribution of the beta-bursts.
**Results:** On average, between one or two beta-bursts were recorded per trial. As predicted, these occurred significantly later during behaviorally relevant trials than during irrelevant trials, in all networks, F(1,58)= 93.5, p<0.001), consistent with normal post-event beta enhancement. This effect was significantly attenuated in schizophrenia, F(1,58)=6.01, p=.017.

**Discussion:** These findings add to the evidence that patients with schizophrenia have reduced ability to allocate attention to behaviorally relevant information. Furthermore, the demonstration of an abnormality potentially accounted for by neural modelling of top-down influence on perceptual processing opens the way to understanding the relevant neural mechanism and to developing neuromodulatory treatments that might alleviate aberrant selective attention in schizophrenia.

### T145. ALTERATIONS IN SUPERFICIAL WHITE MATTER IN THE FRONTAL CORTEX IN SCHIZOPHRENIA: A DWI STUDY USING A NOVEL ATLAS

Ellen Ji*[,1], Sarrazin Samuel[1], Marion Leboyer[1], Miguel Guevara[2], Pamela Guevara[2], Cyril Poupon[3], Antoine Grigis[3], Josselin Houenou[1]
*[1]INSERM; [2]University of Concepción; [3]Neurospin*

**Background:** Alterations in brain connectivity are strongly implicated in the pathophysiology of schizophrenia (SZ). Very recently, evidence is mounting to suggest that changes in superficial white matter (SWM) U-shaped short range fibers are integral components of disease neuropathology, a theory that is supported by findings from postmortem studies and less often in vivo in patients with SZ. This diffusion weighted imaging (DWI) study aimed to investigate SWM microstructure in the frontal cortex in people with SZ.
**Methods:** Whole brain tractography was performed in 31 people with SZ and 54 healthy controls using BrainVISA and Connectomist 2.0 software. Segmentation and labelling of superficial white matter tracts were performed using a novel atlas characterizing 100 bundles. Principal Components Analysis (PCA) using a varimax orthogonal rotation was performed on mean generalised fractional anisotropy (gFA) of bundles located in the frontal cortex. Composites scores were computed for each subject, reflecting a linear combination of mean gFA values.
**Results:** PCA revealed three components explaining 19.7 %, 5.8 %, and 5.4 % of the total variance. The mean score of the second component was significantly lower in the people with SZ compared with controls ($p = 0.01$) and included 13 bundles connecting regions in the pars orbitalis, insula, pars triangularis, pars opercularis, orbitofrontal cortex, anterior cingulate, superior frontal cortex and middle frontal cortex.
**Discussion:** Our results support findings of reduced white matter integrity in the frontal cortex in people with SZ. Moreover, PCA may be helpful in identifying specific networks as the deficits do not appear to be widespread. Identifying patterns of superficial white matter dysconnectivity may be helpful in understanding the prominent symptoms and cognitive deficits and observed in SZ.

### T146. AROUSAL AFFECTS DIFFERENTIALLY FIRST-EPISODE PSYCHOSIS PATIENTS AND CONTROL SUBJECTS' DEFAULT MODE NETWORK FUNCTIONING DURING MOVIE VIEWING

Teemu Mantyla*[,1], Jussi Alho[2], Eva Rikandi[3], Tuula Kieseppä[4], Jaana Suvisaari[1], Tuukka Raij[5]
*[1]National Institute for Health and Welfare; [2]Aalto University School of Science; [3]National Institute for Health and Welfare, University of Helsinki; [4]University of Helsinki, Helsinki University Hospital; [5]Aalto University School of Science, University of Helsinki, Helsinki University Hospital*

**Background:** Functional alterations of the default mode network (DMN) are frequently reported in psychotic disorders, but the functional role of these alterations remains poorly known. In addition to previous studies that have applied different types of tasks or recorded resting-state neuroimaging data, there has recently been more interest in the use of movie stimuli in studying brain functioning in patient populations, because this could provide a more naturalistic account of brain functioning in real life-like situations.
**Methods:** Seventy-one first-episode psychosis (FEP) patients (mean age = 26.0 yrs, 47 (66%) males) and 57 controls (mean age = 26.86 yrs, 24 (42%) males) from the Helsinki Early Psychosis Study watched scenes from the movie Alice in Wonderland (Tim Burton, 2010) during 3 T fMRI-BOLD imaging. We used intersubject correlation (ISC) analysis, in which the correlation between voxel-wise BOLD time series in every within-group pair of subjects is calculated. In this study, time-windowed ISC was calculated with a 10-TR (time of repetition, 1.8 s) window with 1-TR steps over the fMRI time series. In each ISC window, a two-sample t test was performed to obtain a t-statistic time series of differences between the groups. An independent group of control subjects (n = 17, 10 males, mean age 26.5 yrs) rated how emotionally arousing the currently seen events of the stimulus are, producing a time-varying rating used as a regressor. General linear model was used to identify brain regions where the t-statistic time series covaries with the arousal rating. To make the interpretation of results less ambiguous, the arousal rating was divided into high and low arousal regressor by z scoring the rating and taking only the positive and negative values, respectively. Nonparametric clusterwise permutation test was used for statistical inference (cluster-defining threshold of $p = 0.05$, familywise error corrected threshold of $p = 0.05$, number of permutations = 5000). Furthermore, by using an experience-sampling setup during the same brain-scanning session, a partially overlapping sample of participants reported how emotionally aroused they were feeling during scanning.
**Results:** The results show significant correlation between the t-statistic time series and low arousal regressor, especially in the DMN including the anterior and posterior cingulate cortex, medial prefrontal cortex, precuneus, and bilateral lateral temporoparietal regions. Closer inspection reveals that during moments of low arousal in the movie stimulus, the ISC of healthy controls goes up but the ISC of patients does not. In the experience-sampling portion of the study, the patients reported more arousal than the control subjects.
**Discussion:** Intersubject correlation in the DMN depended differentially on arousal in FEP patients and control subjects. More specifically, during moments when the stimulus was rated less emotionally arousing, control subjects' DMN functioning synchronized more while the patients' did not. In connection with the difference in reported arousal during the same imaging session, our findings provide preliminary evidence for a contribution of arousal on the functional alterations of the DMN and suggest that this may be related to higher baseline arousal in the patients. Higher arousal and the related distortion of high order integrative functioning that characterizes DMN could contribute to the pathogenesis of psychosis.

### T147. DECREASED STRIATAL REWARD PREDICTION ERROR CODING IN UNMEDICATED SCHIZOPHRENIA PATIENTS

Teresa Katthagen*[,1], Jakob Kaminski[1], Andreas Heinz[1], Florian Schlagenhauf[2]
*[1]Charité – Universitätsmedizin Berlin; [2] Charité – Universitätsmedizin Berlin, Max Planck Institute for Human Cognitive and Brain Sciences*

**Background:** Reinforcement learning involves flexible adaptation towards a changing environment and is driven by dopaminergic reward prediction error (RPE; outcome (R) – expectation (Q)) signaling in the midbrain and projecting regions, such as the ventral striatum (Schultz, 1998). Schizophrenia patients show heightened dopamine levels in the striatum (Howes et al., 2012) as well as deficits in reinforcement learning (Waltz, 2016) which may be mediated by disrupted prediction error signaling

(Heinz and Schlagenhauf, 2010; Schlagenhauf et al., 2014). Using model-based fMRI, the present study aims to assess these neural signals during a reversal learning paradigm in unmedicated schizophrenia patients and healthy individuals.

**Methods:** In the current study, 19 schizophrenia patients and 23 age- and gender-matched healthy controls completed a reversal learning paradigm (Boehme et al., 2015) during fMRI scanning where subjects had to choose between two neutral stimuli to maximize their reward. A Rescorla Wagner learning model (Single Update, one learning rate) was fitted against the individual choice data using a softmax function. Individual RPE trajectories from the fitted Rescorla Wager learning model were correlated with the BOLD response during feedback onset. Parameter estimates of ventral striaral RPE trajectories were correlated with psychopathology scores from the PANSS (Kay et al., 1987).

**Results:** In the reversal learning task, schizophrenia patients chose the correct stimulus less often compared to healthy individuals (percent correct choices: 65.7 ± 10.7 vs. 76.7 ± 7.7; t=3.7, p=0.001). Across all participants, the RPE trajectories correlated with BOLD response in the bilateral ventral striatum (left ventral striatum [-10 12 10], t=7.40, pFWE <0.001, right ventral striatum [10 12 -10], t=6.56, pFWE=0.006). Schizophrenia patients displayed decreased RPE coding in the right ventral striatum compared to healthy individuals ([14 14 -10], t=3.69, pSVC for nucleus accumbens = .015). In patients, extracted parameter estimates from the right ventral striatum correlated negatively with the PANSS total symptoms score (Spearman's rho =-0.55, p=0.018).

**Discussion:** We found that unmedicated schizophrenia patients performed worse in the reversal learning task and displayed decreased striatal prediction error signaling. This neural deficit was increased in patients with overall higher symptom severity. While RPE coding seems to be intact in patients receiving antipsychotic medication (Culbreth et al., 2016), our findings are in line with previous studies in unmedicated schizophrenia patients (Reinen et al., 2016; Schlagenhauf et al., 2014). Therefore, deficient neural coding of this core reinforcement learning mechanism may reflect a characteristic of the disorder of schizophrenia and does not result from antipsychotic medication.

### T148. THALAMIC-CORTICAL CONNECTIVITY IN PATIENTS WITH AUDITORY HALLUCINATIONS IN SCHIZOPHRENIA

Yulia Zaytseva*[,1], Eva Kozakova[1], Eduard Bakstein[1], Jaroslav Hlinka[1], Maya Schutte[2], Iris Sommer[2], Jiri Horacek[1], Filip Spaniel[1]
*[1]National Institute of Mental Health; [2]Utrecht Medical Centre*

**Background:** The auditory verbal hallucinations (AVH) prevalence among patients diagnosed with schizophrenia is more than 70% (Hugdahl et al, 2008). The brain mechanisms of AVH are not yet clarified. In the currect study, we explore the specific brain connections that are usually associated with perception and reception of the speech, being present in patients with AVHs.

**Methods:** In total 70 patients with first psychotic episode, 41 with auditory hallucinations and 29 patients who had never experienced hallucinations were included into the study based on the following criteria: non-hallucinated patients were defined using PANSS scores (Hallucinations P3 scored as 1)/hallucinated patients (PANSS P3 score 3). All patients underwent resting MRI scans. We analysed the resting state functional connectivity between temporal cortices including TPJ structures (supramarginal gyrus and angular gyrus, parahypocampal gyrus) and predefined thalamic nuclei that are parts of the auditory pathways, specifically: the medial geniculate nuclei(MGB), the mediodorsal nuclei (MDN), having a big spectrum of connections with the limbic system and the reticular thalamic nuclei (RTN) which is a main source of inhibitory signals (using detailed atlas of thalamus, Morel, 2003).

**Results:** The analysis revealed the increased connectivity between mediodorsal nucleus L and Hesch gyrus (L, R), pahahippocampal gyrus (L,R) as well as between mediodorsal nucleus (R) and parahipocampal gyrus (L). The decreased connectivity was detected between medial geniculate body (L), Medial geniculate body (R) and inferior parietal lobule (L) as well as between medial geniculate body (L) x parahyppocampal gyrus (L) (FDR corr p=0.05).

**Discussion:** We confirmed our hypothesis on the altered connectivity between thalamic nuclei and auditory cortex (Heshl gyrus, parahippocampal gyrus) and inferior parietal cortex in patients with AVHs. The findings go in line with other studies on functional dysconnectivity with right hippocampal formation and mediodorsal thalamus compared to patients without lifetime AHs (Diederen et al, 2010; Shinn et al, 2013). The patterns of decreased connections presumably indicate a sensory defect: decreased connectivity between medial geniculate nuclei (main auditory pathway) and parahippocampal gyrus (memory retrieval) and inferior parietal cortex (interpretation of sensory information, Radua et al, 2010). The patterns of increased connections presumably imply alterations of emotional processing (projections from mediodorsal nuclei to auditory cortex) and deserve further investigation.

### T149. METACOGNITIVE DEFICITS IN INTEROCEPTION ARE ASSOCIATED WITH DISSOCIATIVE EXPERIENCES IN PATIENTS WITH FIRST EPISODE PSYCHOSIS

Sarah Garfinkel[1], Kathy Greenwood*[,1], Charlotte Rae[1], Geoff Davies[1], Cassandra Gould van Praag[4], Anil Seth[1], Medford Nick[1], Hugo Critchley[1]
*[1]University of Sussex*

**Background:** Dissociative experiences, including depersonalization and derealisation, represent perturbations of consciousness and selfhood, and are commonly reported by patients during early stages of a psychotic illness. The continuity and integrity of a conscious sense of self is proposed to be grounded upon the control of internal physiological state and its predictive representation through interoception, i.e. the sensing of internal bodily changes. We tested the hypothesized relationship between dissociation and interoceptive deficits in patients with first episode psychosis (FEP), combining behavioural testing with functional neuroimaging.

**Methods:** Individuals with first episode psychosis (N=41) and matched community control participants (N=21) performed an interoceptive task (heart-tone synchrony judgments) during functional magnetic resonance imaging (fMRI). Trial-by-trial confidence ratings indexed subjective performance, and measures of metacognitive interoceptive awareness (insight) were derived from confidence-accuracy correspondence. We tested for regional brain activity relating to dissociative symptom scores and objective, subjective and metacognitive aspects of interoception.

**Results:** In patients with FEP, metacognitive impairments in interoception predicted magnitude of dissociative symptoms, accompanied by hypoactivation of right insula cortex. Other dimensions of interoception, and accuracy, confidence and metacognitive insight on an exteroceptive task were unrelated to dissociative symptoms and there were no group differences between FEP patient and control groups.

**Discussion:** Our findings suggest that symptoms of disturbed conscious integrity and selfhood in early psychosis arise through selective disruption of higher-order metacognitive representations of interoceptive signals. Brain systems supporting the conscious integration of bodily feelings may represent a target for interventions to enhance functioning and, speculatively, mitigate illness progression in psychosis.

### T150. REAL-TIME FMRI NEUROFEEDBACK TO DOWN-REGULATE SUPERIOR TEMPORAL GYRUS ACTIVITY IN PATIENTS WITH SCHIZOPHRENIA AND AUDITORY HALLUCINATIONS: A PROOF-OF-CONCEPT STUDY

Natasza Orlov*[,1], Vincent Giampietro[1], Owen O'Daly[1], Gareth Barker[1], Katya Rubia[1], Philip McGuire[1], Sukhi Shergill[2], Paul Allen[3]
*[1]Institute of Psychiatry, Psychology & Neuroscience, King's College London; [2]Cognition Schizophrenia and Imaging (CSI) Lab; [3]University of Roehampton*

**Background:** Neurocognitive models and previous neuroimaging work posit that auditory verbal hallucinations (AVH) arise due to increased auditory cortex (AC) activity and altered connectivity between the AC and other speech and language regions [e.g. 1]. In the present study we examined if patients with schizophrenia (SCZ) and AVH could be trained to down-regulate AC activity using real time functional Magnetic Resonance Imaging neurofeedback (rtfMRI-NF) [2]. We also examined the effects of rtfMRI-NF training on functional connectivity between the AC and other speech and language regions.
**Methods:** Eleven patients with SCZ (Table 1) and treatment refractory AVH were recruited to participate in the study and were trained to down-regulate auditory cortex (AC) activity over an average of fourteen rtfMRI-NF runs conducted during a two-week training period (Fig 1). We used a functional localiser to identify the speech sensitive superior temporal cortex (STG) (Figure 2A). At the end of the training period, AC activity, functional connectivity and AVH symptom levels were compared pre and post training.
**Results:** Patients successfully learnt to down-regulate activity in their AC over the rtfMRI-NF training period. Post training, patients showed increased connectivity between the AC, the inferior prefrontal gyrus and the inferior parietal lobe Figure. There was also a modest reduction in AVH symptom levels post compared to pre training (Table 2).
**Discussion:** The AC is as suitable target region for rtfMRI-NF in patients with SCZ and treatment refractory AVH. Successful down-regulation of AC activity can increase functional connectivity between speech motor and perception regions. These findings raise the possibility that rtfMRI-NF training could be used as a novel therapeutic intervention in this clinical population.

### T151. APATHY AND DIMINISHED EXPRESSION ARE NOT ASSOCIATED WITH VENTRAL OR DORSAL STRIATUM VOLUME IN SCHIZOPHRENIA

Achim Burrer*[,1], Matthias Kirschner[1], Erich Seifritz[1], Kaiser Stefan[2]
*[1]University Hospital of Psychiatry, University of Zurich; [2]Geneva University Hospitals*

**Background:** Negative symptoms are core features of schizophrenia and can be grouped into two domains. These are apathy including anhedonia, avolition and asocialty as well as diminished expression including blunted affect and alogia. A large body of research found that ventral striatal hypoactivation is linked to negative symptoms. In particular, it has been shown that this neural correlate is specific for apathy but not diminished expression. Here, we investigated whether this dissociation can also be found in ventral striatum volume.
**Methods:** We included brain structural T1 MRI data of 60 patients diagnosed with schizophrenia (SZ) and 58 healthy controls (HC). Negative symptoms in these groups have been assessed using the Brief Negative Symptom Scale (BNSS). We performed voxel-based morphometry (VBM) using the statistical parametric mapping package (SPM 12; Wellcome Trust Centre for Neuroimaging, London). We performed a region of interest (ROI) analysis of ventral and dorsal striatal volume between patients with schizophrenia and healthy controls. Furthermore, we analyzed the correlation of right and left ventral striatal volume with apathy and diminished expression in patients with schizophrenia. Moreover, we analyzed potential group differences in gray matter volume in an exploratory whole-brain analysis. Finally, we performed an exploratory whole-brain linear regression to identify potential correlations between the two negative symptom dimensions and gray matter volume. (cluster-defining threshold of $p < 0.001$, cluster-level pFWE < 0.05)
**Results:** Patients with schizophrenia showed no differences in ventral striatal volume compared to healthy controls. Apathy or diminished expression did not correlate with ventral or dorsal striatal gray matter volume in patients with schizophrenia. In the exploratory whole-brain analysis we found significant less gray matter volume in the right insula of schizophrenia patients compared to healthy controls (cluster-level pFWE = 0.03, peak (x,y,z = 46,-15,20). Our exploratory whole-brain linear regression revealed no significant correlation between apathy or diminished expression and gray matter volume changes in patients with schizophrenia.
**Discussion:** Although a correlation of apathy and ventral striatal volume has been shown in a previous study with fewer subjects, we could not reproduce this finding in a larger group of 60 patients with schizophrenia (Roth et al. 2016). However, while these negative findings do not support the association between apathy and ventral striatal volume, there may be more subtle brain structural changes linked to the pathophysiology of apathy, which cannot be detected by voxel based morphometry. The gray matter reduction in the right insula in subjects with schizophrenia replicated findings from previous studies in schizophrenia (Fornito et al. 2009).

### T152. NRN1 GENE AND FUNCTIONAL MRI: ASSOCIATION ANALYSIS IN SCHIZOPHRENIA PATIENTS AND HEALTHY SUBJECTS

Carmen Almodóvar*[,1], Maria Guardiola[2], Pilar Salgado-Pineda[3], Marcos Moreno[4], Carme Gallego[4], Claudia Prats[5], Barbara Arias[5], Edith Pomarol-Clotet[3], Peter J. McKenna[3], Mar Fatjó-Vilas[2]
*[1]FIDMAG Research Foundation, University of Barcelona; [2]FIDMAG Research Foundation. CIBERSAM, University of Barcelona; [3]FIDMAG Research Foundation. CIBERSAM [4]Molecular Biology Institute of Barcelona (IBMB-CSIC); [5]University of Barcelona, CIBERSAM*

**Background:** Alterations of synaptic plasticity are currently accepted to play a critical role in schizophrenia (SZ). Among genes of neuronal plasticity there is Neuritin1 gene (NRN1), which has been associated with SZ, age at onset and differences in general cognitive performance in this disorder. However, little is known about the brain imaging correlates of NRN1 gene. We aimed: i) to investigate the association of NRN1 with schizophrenia-spectrum disorders (SZ-SD), exploring its role in age at onset through a family-based study, ii) to examine the brain functional correlates of NRN1 sequence variants through a neuroimaging genetics approach using a case-control design.
**Methods:** A family-based association analysis was carried out with a sample of 588 individuals from 159 families (74 early onset / 85 adult onset) with an offspring with a diagnosis of SZ-SD. An independent sample consisting of 45 subjects (26 patients / 19 controls) was used to perform a case-control neuroimaging genetics analysis. DNA was extracted from blood/buccal mucosa samples and eleven Single Nucleotide Polymorphisms (SNPs) in NRN1 were genotyped. The linkage disequilibrium between the SNPs was estimated in the family-based sample with Haploview v4.1.

Three haplotype blocks were defined: 1) rs2208870, rs12333117, rs582186, 2) rs645649, rs582262, 3) rs3763180, rs10484320, rs4960155, rs9379002, rs9405890, rs1475157. PLINK-v1.06 was used for the tabulation of possible individual haplotype phases and for the family-based association analyses (Transmission Disequilibrium Test). To explore the brain functional correlates of NRN1, the subjects belonging to case-control sample underwent a single MRI scanning session and performed a virtual reality spatial navigation task (Salgado-Pineda et al. 2016). The standard atlas provided in the FSL package was used to define three separate ROIs (left and right hippocampus and medial frontal region (mPFC)) and the mean value of activation per each subject was used to test the effect of each SNP/haplotypes by means of a linear regression. All the analyses were adjusted by age, sex and premorbid intelligence coefficient (IQ-TAP).

**Results:** Two haplotypes including SNP4 and SNP5 (rs645649-rs582262) were associated with early onset SZ-SD: the haplotype CG was undertransmitted from parents to patients (p=0.011, OR (95%CI=0.08(0.01–0.71) - protective haplotype), while the haplotype GG showed an overtransmission trend (p=0.055, OR (95%CI=3.83 (1.40–10.48)). No effect was observed in the adult onset subsample.

No differences between patients and controls were observed in the activation of the three ROIs. Within patients, an effect of the haplotype CG (SNP4-5) was detected in the mPFC: carriers of no copies of the protective haplotype showed a higher mean activation (n=15, mean(SD)=-1.17(17.37)) than individuals with at least one copy of the haplotype (n=9, mean(SD)=-21.19(21.94)) (□=-0.507 p=0.035).

**Discussion:** First, our family-based results are consistent with evidence of a genetic association between NRN1 gene and SZ-SD and extend the knowledge on that NRN1 has a selective impact on early age at onset (Fatjó-Vilas et al. 2016). Second, our data suggest that NRN1 is involved in the regulation of the de-activation of mPFC in patients with SZ during a spatial navigation task. This result is of special interest since mPFC is an area included in the Default Mode Network (DMN) and alterations in this network have been highly documented in SZ patients during performance of different tasks (Pomarol-Clotet et al. 2008; Mannell et al. 2010; Salgado-Pineda et al. 2011).

**Acknowledgements:** Fundación Alicia Koplowitz.

### T153. CAN COGNITIVE TRAINING DECREASE REACTIVE AGGRESSION IN SCHIZOPHRENIA?

Anthony Ahmed*[,1], Matthew Hoptman[2], Jean-Pierre Lindenmayer[3]

*[1]Weill Cornell Medical College, New York Presbyterian Hospital; [2]Nathan Kline Institute; [3]New York University*

**Background:** Cognitive deficits contribute to aversive social behaviors such as impulsive aggression. Studies have shown that cognitive training interventions may decrease the risk for impulsive aggression. The current study sought to illuminate the underlying mechanism of cognitive training effects on impulsive aggression—particularly, changes in the neural circuitry and in behavioral expressions of emotion regulation and emotion-based impulsivity.

**Methods:** Participants (N=28) with schizophrenia or schizoaffective disorder were recruited from New York Presbyterian Hospital and Manhattan Psychiatric Center and randomized into one of two cognitive training groups—a cognitive remediation training plus social cognition training (CRT+SCT) group versus CRT alone. At baseline and following 36 hours of training, participants completed the MATRICS Consensus Cognitive Battery (MCCB), Eyes Task, and the Emotion Recognition-40 (ER-40) as measures of neurocognition, mentalizing, and facial affect recognition. We indexed emotion regulation capacity using the Positive and Negative Affect Scale (PANAS) and by obtaining heart rate, respiration, and electrodermal activity while participants viewed pictures selected from the International Affective Picture System (IAPS). A subsample of participants completed fMRI scans during the completion of the emotion regulation task. The Go No-go task and the Emotional Stop Signal task served as measures of impulsivity. Aggression was measured using the Overt Aggression Scale (OAS), the Point Subtraction Aggression Paradigm (PSAP), and the Taylor Aggression Paradigm (TAP).

**Results:** Participants were 31.93 years old (SD=10.46) and had completed 12.07 (SD=2.59) years of education. Both groups showed improvements from baseline on the composite cognition score of the MCCB with a slight edge to the combined CRT+SCT group (Cohen's d=0.22). Both groups showed pre-to-post reductions in aggression with only minimal differences. Although both groups showed pre-to-post improvements in affect recognition and mentalizing, the CRT+SCT group showed greater improvements in affect recognition (Cohen's d = 0.21) and mentalizing (Cohen's d = 0.39). Both groups showed reductions in negative affectivity scores from baseline (Cohen's d =-0.48) but reductions were greater in the CRT+SCT group (Cohen's d = -0.24). Both groups demonstrated pre-to-post reductions in their Low Frequency/High Frequency heart rate variability ratio (Cohen's d=-0.83) and pre-to-post reductions in skin conductance (Cohen's d = -0.48). Pre-to-post differences in HRV and skin conductance were very minimal.

Both groups demonstrated large pre-to-post reductions in misses on the No-Go trials of the Go No-Go Task (Cohen's d =-1.74). Reductions were greater in the CRT+SCT than the CRT only group (Cohen's d=0.49) suggesting that the CRT+SCT group show greater improvements in impulse control after cognitive training.

Baseline fMRI scans showed that amygdalofrontal network activation was greater when emotionally evocative pictures were preceded by a reappraisal statement compared to conditions in which they were preceded by negative descriptions. This shows that the emotion regulation task engages relevant neural targets. The presentation will include accumulated follow-up fMRI scans. It is expected that there will be increased BOLD signaling following cognitive training.

**Discussion:** The study adds to evidence of cognitive training prospects for decreasing aggressive impulses. A mechanistic model with improved emotion regulation and impulse control contributing to reduced aggression may characterize cognitive training effects. Change in neural circuitry of emotion regulation will demonstrate strong proof-of-concept.

### T154. RESTING STATE PERFUSION IN THE REWARD SYSTEM LINKED TO DIMENSIONS OF NEGATIVE SYMPTOMS IN SCHIZOPHRENIA

Katharina Stegmayer*[,1], Andrea Federspiel[2], Roland Wiest[3], Sebastian Walther[2]

*[1]University Hospital of Psychiatry, University of Bern; [2]University Hospital of Psychiatry; [3]University Institute of Diagnostic and Interventional Neuroradiology*

**Background:** Negative symptoms (NS) are central for the symptomatology of schizophrenia associated with poor functional outcome. Two dimensions of NS have consistently been proposed: apathy and diminished expression. Even though distinct pathophysiological mechanism have been hypothesised resting state perfusion and dimensions of NS have not been studied. Here, we therefore focused on dimensions of NS and the link to whole brain resting state perfusion in schizophrenia patients.

**Methods:** We included 45 schizophrenia spectrum patients and 44 age- and gender-matched healthy controls. We assessed NS with the Scale for the Assessment of Negative Symptoms (SANS) and imaging on a 3T MRI scanner. Apathy was currently present in 31 patients and diminished expression in 27 patients. Patients did not differ in antipsychotic medication or positive symptoms. We compared whole-brain perfusion over all, and between the groups using 1-way ANCOVAs (F and T tests). A uniform threshold of $p < 0.5$ (FWE-corr) was applied.

**Results:** Diminished expression was most prominently associated with perfusion within the right orbital cortex, insula, ventral striatum and head of caudate nucleus, while apathy was associated with perfusion bilateral within the SMA, the insula and the thalamus.

**Discussion:** Dimensions of NS at rest were associated with altered resting state perfusion, in particular in brain areas relevant for reward processing. Distinguishable associations of rCBF with NS dimensions point to distinct underlying pathophysiology.

### T155. SEPARABLE AND REPLICABLE NEURAL STRATEGIES DURING SOCIAL BRAIN FUNCTION IN PEOPLE WITH AND WITHOUT SEVERE MENTAL ILLNESS

Colin Hawco*,[1], Robert Buchanan[2], Navona Calrco[1], Benoit Mulsant[1], Joseph Viviano[1], Erin Dickie[1], Miklos Argyelan[3], James Gold[2], Marco Iacoboni[4], Pamela DeRosse[3], George Foussias[1], Anil Malhotra[3], Aristotle Voineskos[1]
*[1]University of Toronto; [2]Maryland Psychiatric Research Center; [3]The Zucker Hillside Hospital; [4]David Geffen School of Medicine at UCLA*

**Background:** The case-control design and disease heterogeneity may be major limiting factors impeding biomarker discovery in brain disorders, including serious mental illness such as schizophrenia spectrum disorder (SSD) or bipolar disorder (BPD). We propose that this heterogeneity represents an opportunity for discovery by uncovering relevant biologically driven sub-types within disorders. Individuals with schizophrenia spectrum disorder (SSD) have deficits in social cognition related to poor functional outcome.
**Methods:** A total of 109 SSD and 70 matched healthy controls (HC) were recruited across three sites. Participants performed an fMRI task in which they observed or imitated emotional faces. For each participant, an individual pattern of activity (Imitate > Observe for emotional faces) was identified. Hierarchical clustering (Ward's method) identified clusters of individuals with similar patterns of activity. We then examined whether new data-driven groups of participants (based on patterns of brain activity) demonstrated performance differences on a batter of social and neuro cognitive tests completed out of the scanner. As a validation of the importance of cluster membership, Euclidean distance was compared between participants to members of their own cluster, diagnosis, or site. The clustering analysis was repeated on a replication sample consisting of 32 SSD, 37 euthymic BPD, and 39 HC.
**Results:** Three clusters with distinct patterns of neural activity were found. Cluster one (24 HC and 44 SSD) represented 'typical activators' (lateral frontal and parietal activity). Cluster two (21 HC and 31 SSD) were identified as 'hyper-activators', showing more intense and extended activity. This was interpreted as a 'compensatory' response of over-activation related to impaired neural circuits, such as is seen in aging. Interestingly, cluster three (25 Controls and 35 SSD) showed a very atypical pattern, including suppression of activity during imitation in regions involved in the default mode network and/or higher order social cognition (e.g. theory of mind). This group also had improved social cognitive performance relative to the other clusters. Participants were found to have more similar patterns of brain activity to members of their cluster rather than to members of their diagnostic group or scanning site. Importantly, when clustering was applied to the replication sample, the same three patterns (typical activators, hyper activators, and deactivators) were identified.
**Discussion:** In independently collected samples, our findings demonstrate different patterns of neural activity among individuals during a socio-emotional task that were independent of DSM-diagnosis or scan site. Our findings may provide objective neuroimaging endpoints (or biomarkers) for subgroups of individuals in target engagement research aimed at enhancing cognitive performance independent of diagnostic category.

### T156. IN VIVO CHARACTERIZATION OF THE FIRST AGONIST DOPAMINE D1 RECEPTORS PET IMAGING TRACER [18F]MNI-968 IN HUMAN

Gilles Tamagnan*,[1], Olivier Barret[1], David Alagille[1], Vincent Carroll[1], Jennifer Madonia[1], Cristian Constantinescu[2], Christine SanDiego[1], Caroline Papin[1], Thomas Morley[1], David Russell[1], Timothy McCarthy[3], Lei Zhang[3], David Gray[3], Anna Villalobos[3], Chewah Lee[3], Jianqing Chen[3], John Seibyl[1], Kenneth Marek[1]
*[1]inviCRO; [2]Constantinescu; [3]Pfizer, Inc.*

**Background:** D1 receptors, which couple to inhibitory G-proteins, have been shown to regulate neuronal growth and development, mediate some behavioral responses. Its function has been shown to be altered in both neurologic and psychiatric disorders. To date, there is a lack of agonist PET tracers for the D1 receptors labeled with 18F with relevance in clinical studies. We report the evaluation in non-human primates of [18F]MNI-968 (PF-06730110), a novel PET radiotracer of the D1 receptors
**Methods:** Four brain PET studies, 2 baselines and 2 blockade studies using PF-2562, a D1 partial agonist compound, were conducted for 90 min in two rhesus monkeys with [18F]MNI-968 (169 ± 31 MBq). [18F]PF-06730110 was administered at the same dose level for both monkeys as a bolus followed by a 2-hour infusion, with [18F]MNI-968 administered 30 min into the infusion. Additionally, six brain PET studies were conducted over 180 min (317 ± 49 MBq) in 6 healthy human volunteers (3 test/retest and 3 test). PET data were modeled with 2-tissue compartmental model (2T), Logan graphical analysis (LGA), and non-invasive Logan graphical analysis (NI-LGA) with cerebellar cortex as reference region to estimate total distribution volume VT, and binding potential BPND.
For the blockade studies in rhesus monkeys, occupancy was estimated from BPND at baseline and post blockade.
**Results:** In rhesus monkeys, [18F]MNI-968 (PF-06730110), penetrated the brain with a peak whole-brain uptake up to ~3% of the injected dose at ~ 6 min post injection and showed a fast washout. The highest signal was found in the caudate, putamen, with moderate extrastriatal uptake. The lowest signal was in the cerebellum. BPND values were up to ~1.4 in the putamen. All three quantification methods (2T, LGA and NI-LGA) were in excellent agreement, with a similar estimated D1 receptors occupancy of PF-06730110 of ~40% for both monkeys in the caudate and putamen.
In human, [18F]MNI-968 kinetics appeared to be faster compared to non-human primates, with a BPND in the putamen of ~0.8. Initial measurement of test-retest reproducibility was ≤ 7% for BPND in the striatal regions.
**Discussion:** Our work showed that [18F]MNI-968 ([18F]PF-06730110), is a promising agonist PET radiotracer for imaging D1agnist receptors that can be quantified non-invasively. Studies are currently ongoing both in non-human and human primates to further characterize the tracer.

### T157. FRONTOSTRIATAL CONNECTIVITY IN TREATMENT-RESISTANT SCHIZOPHRENIA: RELATIONSHIP TO POSITIVE SYMPTOMS AND COGNITIVE FLEXIBILITY

Vanessa Cropley*,[1], Eleni Ganella[1], Cassandra Wannan[1], Andrew Zalesky[1], Tamsyn Van Rheenen[1], Chad Bousman[2], Ian Everall[3], Alexander Fornito[4], Christos Pantelis[5]
*[1]The University of Melbourne; [2]University of Calgary; [3]Institute of Psychiatry, Psychology & Neuroscience, King's College London; [4]School of Psychology and Psychiatry & Monash Biomedical Imaging, Monash University; [5]Melbourne Neuropsychiatry Centre, University of Melbourne*

**Background:** The frontostriatal circuits linking different parts of the frontal cortex to subregions of the striatum are proposed to regulate different aspects of cognition, executive function, affect and reward processing. Dysregulation of these brain circuits is also known to be important in the etiology of psychotic disorders, with the magnitude of dysfunction correlating with the severity of positive symptoms. These observations suggest that the integrity of brain circuits connected to the striatum is important for antipsychotic treatment response as well as specific cognitive processes. However, not all individuals with schizophrenia benefit from antipsychotic treatment, with up to 20% of individuals considered to be treatment-resistant. These individuals also show pervasive impairments in cognition, including cognitive flexibility. Nevertheless, few studies have examined striatal connectivity in treatment-resistant schizophrenia (TRS), particularly in relation to positive symptomatology and specific cognitive deficits subserved by the striatal circuits. This study therefore aimed to (i) assess for disruptions in frontostriatal connectivity in a sample of TRS and (ii) assess the relationship between the frontostriatal circuits with positive symptoms and attentional set-shifting (cognitive flexibility) given recent associations with the dorsal striatal circuit.
**Methods:** Resting-state functional magnetic resonance imaging was used to investigate functional connectivity (FC) in 42 TRS participants prescribed clozapine (30 males, mean age=41.3(10)), and 42 healthy controls (24 males, mean age=38.4(10)). The whole striatum (caudate, putamen and nucleus accumbens) and the left and right dorsal striatum were separately seeded as regions of interest, and Pearson's correlations between the seeds and all other voxels comprising cortical and subcortical gray matter were investigated. For brain regions that showed significant group differences in FC with the striatal seeds, Pearson's correlations explored the relationship between the strength of connectivity with positive symptoms and attentional set-shifting (extradimensional shift errors) as measured with the CANTAB intra-/extradimensional set shift task.
**Results:** In comparison with healthy controls, TRS patients displayed significantly reduced FC between the whole striatum and the bilateral anterior cingulate, cerebellum, precuneus, right and left frontal pole and left insular/temporal pole, and reduced FC of the left and right dorsal striatum with cerebellum, and between the right dorsal striatum and bilateral cingulate and right frontal pole. Reduced FC between the whole striatum and precuneus and insular/temporal pole was associated with greater delusions of jealousy ($p<.002$ uncorrected); no other associations with positive symptoms were detected. In the entire sample, reduced FC from all striatal seeds was associated with greater extradimensional errors, indicating worse cognitive flexibility. These associations were not detected in TRS and controls separately.
**Discussion:** Our preliminary findings reveal reduced striatal FC in TRS, including hypoconnectivity of the dorsal striatal circuit. In contrast to early psychosis, reduced dorsal striatal connectivity does not appear to mediate positive symptoms. Our finding relating hypoconnectivity of the striatal circuits with impaired cognitive flexibility is partly consistent with recent observations in other psychiatric disorders, although such deficits appear not specific to the dorsal circuit and to TRS. Future work will examine connectivity of the ventral striatum, as well as striatal connectivity in early-onset psychosis and siblings of patients with schizophrenia.

### T158. THE VALUE OF ACTIGRAPHY FOR MEASURING APATHY IN PATIENTS WITH SCHIZOPHRENIA: ASSOCIATIONS WITH CLINICAL MEASURES AND NEUROIMAGING OF ACTION INITIATION

Marie-José van Tol*[,1], Claire Kos[1], Michelle Servaas[1], Jan Bernard Marsman[1], Nicky Klaasen[1], Oliver Tucha[1], Henderikus Knegtering[1], André Aleman[1]
*[1]University of Groningen*

**Background:** Apathy is a highly debilitating and frequently occurring behavioral characteristic, present in approximately half of the patients with schizophrenia. Apathy is considered a core negative symptom that relates strongest to lack of initiative and is the strongest predictor of poor functional outcome, poor medication compliance, and high caregiver burden. Notwithstanding, measuring apathy is still a challenge. Therefore, we aimed to investigate whether actigraphy, an objective and continuous measurement of activity levels, could yield an objective quantitative measure of apathy severity in schizophrenia patients. Moreover, we aimed to investigate whether actigraphy related to relevant functional neuroimaging underpinnings of apathy, i.e. self-initiated goal-directed behavior.
**Methods:** Quantity, variability, and initiation of motor behavior were studied in relation to apathy severity as measured with clinical measures, and in relation to neural correlates of self-initiated behavior using functional Magnetic Resonance Imaging (fMRI). All patients (N=58) suffered from clinical significant apathy, as measured with the Apathy Evaluation Scale and Scale for the Assessment of Negative Symptoms and wore an actigraph for 48 continuous hours. Physical activity was quantified as the total activity counts over the patients' ten most active hours of each day, summed over two weekend days (Activity-total, i.e. 20 hours in total). Variability of motor behavior (Activity-variability) was calculated by taking the root of the Mean Squared Successive Difference of the activity counts. For 31 of these patients, fMRI data during a task tapping into self-initiative was available.
**Results:** Results showed that quantity, variability, and initiation of motor behavior were associated with negative symptoms, but not specifically with apathy. Motor behavior parameters were associated with brain activation during the self-initiative task in various brain regions including inferior parietal regions. The results were only observed during the condition wherein participants were asked to promptly reply to specific cues and not during the condition where more freedom in timing and selection of behavior was allowed.
**Discussion:** Actigraphy can be used to measure quantity as well as variability of motor behavior in patients with schizophrenia and with specific relevance for negative symptoms, and that it correlates with selective neural substrates of action selection and activation of motor programs. However, actigraphy may not capture higher-order motivational processes that contribute to apathy severity.

### T159. ASSOCIATION BETWEEN VITAMIN D INSUFFICIENCY AND METABOLIC SYNDROME IN PATIENTS WITH PSYCHOTIC DISORDERS

Sung-Wan Kim*[,1], Taeyoung Yoo[1], Jung Jin Kim[2], Jae-Kyeong Kim[3], Ji-Eun Hong[4], Ju-Yeon Lee[5], Jae-Min Kim[1], G. Paul Amminger[6], Michael Berk[7], Jin-Sang Yoon[2]
*[1]Chonnam National University Medical School; [2]The Catholic University of Korea Kangnam, St. Mary's Hospital; [3]Mindlink, Gwangju Bukgu Mental Health Center; [4]Gwangju Mental Health Commission; [5]Chonnam National University Medical School, Gwangju Bukgu Community Mental Health Center; [6]Orygen - The National Centre of Excellence in Youth Mental Health; [7]Deakin University, School of Medicine*

**Background:** Vitamin D levels are low in patients with schizophrenia. This study examined the association between vitamin D and metabolic syndrome in patients with psychotic disorders.
**Methods:** The study enrolled 302 community-dwelling patients with psychotic disorders. Sociodemographic and clinical characteristics, including blood pressure, physical activity, and dietary habit were gathered. Laboratory examinations included vitamin D, lipid profile, fasting plasma glucose, HbA1c, liver function, and renal function. Vitamin D insufficiency was defined as < 20 ng/ml. Clinical characteristics associated with vitamin D insufficiency were identified.
**Results:** Among the 302 participants, 236 patients (78.1%) had a vitamin D insufficiency and 97 (32.1%) had metabolic syndrome. Vitamin D insufficiency was significantly associated with the presence of metabolic syndrome ($p = 0.006$) and hypertension ($p = 0.017$). Significant increases in triglycerides and alanine transaminase were observed in the group with a vitamin D insufficiency (p = 0.002 and 0.011, respectively). After adjusting for physical activity and dietary habit scores, vitamin D insufficiency remained significantly associated with metabolic syndrome and hypertension.

**Discussion:** Vitamin D insufficiency was associated with metabolic syndrome and was particularly associated with high blood pressure, although the nature, direction and implications of this association are unclear.

### T160. TREATMENT OF CLOZAPINE-ASSOCIATED OBESITY AND DIABETES WITH EXENATIDE (CODEX) IN ADULTS WITH SCHIZOPHRENIA: A RANDOMISED CONTROLLED TRIAL

Dan Siskind*[,1], Anthony Russell[2], Clare Gamble[2], Karl Winckel[2], Sam Hollingworth[2], Steve Kisely[3]
*[1]Metro South Addiction and Mental Health Service; [2]University of Queensland; [3]University of Queensland, MSAMHS*

**Background:** Clozapine is the most effective anti-psychotic for treatment refractory schizophrenia, but causes significant metabolic disturbances including obesity and type 2 diabetes. The metabolic adverse reactions may be mediated in part by clozapine induced dysregulation of Glucagon-like-peptide-1 (GLP-1). GLP-1 is an intestinal epithelial derived peptide, released with ingestion of food, that triggers satiety, reduces glucagon production, promotes insulin production and slows gut motility. Clozapine has been shown to interfere with GLP-1 function in animal models, leading to metabolic dyregulation including obesity and preference for high calorie meals. Administration of exogenous GLP-1 agonists such as exenatide to animals have been shown to counter this effect of clozapine. Exenatide subcutaneous weekly injections may assist obese people on clozapine lose weight.

**Methods:** This randomised, controlled, open-label, pilot trial aimed to evaluate the effect of exenatide on weight loss among clozapine-treated obese adults who have schizophrenia, with or without stable diabetes. Twenty-eight out-patients were randomised to once weekly extended release subcutaneous exenatide or treatment as usual for 24 weeks. This trial examined the safety, tolerability and acceptability of exenatide among obese people with schizophrenia on clozapine, with an evaluation of change in weight, glycaemic control, psychosis severity and metabolic parameters.

**Results:** All 28 participants completed the study. (Exenatide=14 (3 T2DM), control=14 (2 T2DM)). Six people on exenatide achieved >5% weight loss, compared to only 1 control (p=0.029). Mean weight loss was greater for exenatide than control at week 24 (-5.29kg vs -1.12kg, p=0.015) as were. BMI (-1.78 vs -0.39 p=0.019), fasting glucose (-0.34 vs 0.39, p=0.036) and HbA1c (-0.21 vs 0.03, p=0.004). There was no significant difference for other metabolic syndrome components. There were higher rates of transient nausea (n=8), vomiting (n=7) and diarrhoea (n=7) in the exenatide group.

**Discussion:** Exenatide is a promising therapeutic agent for glycaemic control and weight loss in clozapine-treated people with obesity. These results suggest good tolerability and a consistent and favourable pattern of weight loss effects with exenatide. GLP-1 agonists could assist in reducing the cardio-metabolic associated morbidity and mortality secondary to clozapine.

### T161. HEARING VOICES AMONG INDIGENOUS MAASAI WOMEN IN TANZANIA: IMPLICATIONS FOR GLOBAL MENTAL HEALTH

Neely Myers[1], Luca Pauselli*[,2], Michael Compton[3]
*[1]Southern Methodist University; [2]College of Physicians and Surgeons, Columbia University/New York State Psychiatric Institute; [3]College of Physicians and Surgeons, Columbia University*

**Background:** Studies of the health of indigenous and tribal people can shed light on health inequalities with implications for global mental health. Almost no previous studies of mental health among the Maasai in Kenya or Tanzania or even pastoralist groups in general are available, with the exception of one mentioning the stress of rural-to-urban migration. While engaged in ethnographic research in 2013, Myers interviewed 13 Maasai women in northern Tanzania about their everyday lives. Interested in the phenomenology of voice-hearing, she also asked them if they heard voices. Eleven of the women (85%) reported regularly hearing voices that they found to be distressing. Myers returned in 2015 to collect data from a larger sample, which has resulted in this report.

**Methods:** For this study, we used a convenience sample (n=73) of females taken from a broader study whose eligibility criteria included being a Maasai person living in the Arusha Region of Tanzania and over the age of 18. We excluded people who reported being psychiatric patients or family members of patients being seen by the local mental health coordinator to create a nonclinical community sample. This project conducted an initial survey to: 1) estimate the community prevalence of voice-hearing, or auditory verbal hallucinations (AVHs) in this specific population; and, 2) examine any demographic correlates and two specific hypothesized correlates based on previous literature about voice-hearing (e.g., psychological stress and potentially traumatic events).

**Results:** The prevalence of AVHs in this community sample was quite high compared to other studies in sub-Saharan Africa, at 34.3%. There were no differences between participants who did and did not experience AVHs in terms of demographics, but those experiencing AVHs had a statistically significantly higher level of psychological distress (30.1, SD=6.0, compared to 25.1, SD=6.0), with a Cohen's d effect size of .87. Even though a numerical difference was observed in terms of potentially traumatic events (4.6, SD=2.1, compared to 3.8, SD=2.3), this difference was not statistically significant (d=.38).

**Discussion:** Hearing distressing voices may be an indicator of mental ill health that is easily recognizable to community health workers and brings much-needed attention to communities in need. Maasai women face tremendous social adversity in this time of rapid social, economic, and climate change in the region. Evidence for a link between stressful life events, social disadvantage, and the development of psychotic symptoms is strong in Europe, but not as well-developed in this region. The high level of psychosocial stress and AVHs in our sample may also be indicative of extreme social adversity. Maasai women have been historically disenfranchised since the advent of colonial and postcolonial policies favoring men. Women in this region also experiences extreme states of deprivation, including a 21.5 year gap in life expectancy at birth compared to the local population (for all Maasai), 81% severe food insecurity, and a rate of 59% for the stunted growth of children (as compared to their neighbors, the Meru, with 21% of children with stunted growth). Due to local livelihood insecurity, the age of first marriage has been decreasing, resulting in increased pressure on women. This analysis contextualizes these findings and calls for further research on the epidemiology of voice-hearing in this region, as well as further work on the phenomenology of these AVHs so that we can best understand how to address them and improve mental health outcomes for this marginalized group.

### T162. LEFT PREFRONTAL GYRIFICATION IN HEALTHY SUBJECTS ASSOCIATED WITH SUBCLINICAL PSYCHOTICISM PHENOTYPE

Igor Nenadic*[,1], Katharina Frisch[2], Bianca Besteher[2], Christian Gaser[2]
*[1]Philipps-Universität Marburg - UKGM; [2]Jena University Hospital*

**Background:** Recent studies have shown aberrant gyrification, i.e. folding of the cortical surface, as a marker of disturbed neocortical development in schizophrenia. Unlike more commonly used markers like voxel-based morphometry, these parameters might be more sensitive to early neurodevelopmental pathologies. It is unclear, however, whether such structural changes might be evident across the schizophrenia spectrum, involving at-risk subjects as well as even healthy subjects with subclinical or attenuated psychotic(-like) symptoms

**Methods:** We analysed high-resolution MRI scans (3 Tesla, T1-weighted MPRAGE, 1x1x1mm resolution) from n=177 healthy subjects with no current or previous psychiatric condition recruited from the local community. Subjects completed the SCL90R, a general symptom checklist (i.e. self-rating of symptoms), which includes subscales for psychoticism (with subclinical psychotic/-like symptoms) and paranoid ideation. We used the CAT12 toolbox to analyse both gyrification using the absolute mean curvature approach (Luders et al., NeuroImage 2006, as well as cortical thickness and voxel-based morphometry (VBM).

**Results:** Correcting for effects of age and gender, we found a significant negative correlation between SCL90R psychoticism scores and gyrification in a left prefrontal / frontopolar cluster, but no similar finding for wither cortical thickness analysis nor analyses of the paranoid ideation subscale.

**Discussion:** Our results suggest that prefrontal gyrification might be a marker for psychotic phenotypes spanning a spectrum from subclinical symptom expression to frank psychosis. This association seems linked to gyrification (rather than other markers of brain structure), which would suggest a relative specificity. Hence, this would be consistent with the assumption that gyrification is related to early neurodevelopmental effects, which lead to liabity to experiencing psychotic symptoms later in life, and might thus serve as an imaging phenotype for early risk detection and intervention in high-risk groups.

### T163. SUBMISSION WITHDRAWN

### T164. STRUCTURAL COVARIANCE IN DRUG-NAÏVE FIRST EPISODE PSYCHOSIS: AN ULTRA-HIGH FIELD MRI STUDY

Tushar Das[1], Kara Dempster[2], Michael Mackinley[2], Peter Jeon[1], Joe Gati[3], Jean Theberge[4], Ali Khan[3], Lena Palaniyappan*[,1]

*[1]University of Western Ontario; [2]London Health Sciences Center; [3]Robarts Research Institute; [4]St. Joseph's Health Care London*

**Background:** Structural neuroimaging studies report disrupted morphological relationship in the grey matter volume (structural covariance) in patients with schizophrenia, indicating an impairment in functional and/or developmental plasticity. To our knowledge, no studies have examined the alterations in structural covariance across the entire brain in drug-naïve first episode psychosis. TOPSY (Tracking Outcomes of Psychosis) is one of the first studies intending to track the neurobiological trajectory using ultra-high field (7T) imaging starting from a drug-naïve first episode state. Here, we report the initial findings from the structural covariance of grey matter volume. To our knowledge, this is the first structural covariance analysis being reported using a 7T anatomical MRI acquisition.

**Methods:** We used ultra-high field (7 Tesla) MRI in 28 patients with FEP (satisfying criterion A of DSM-5 schizophrenia) and 18 controls, to estimate grey matter volume in a voxelwise manner. FEP and controls were matched for age, sex and parental socioeconomic status. Patients were recruited at an early intervention unit (PEPP, London Ontario) and had active psychotic symptoms at the time of scanning. Morphometric analysis was done using SPM12, after DARTEL based registration and segmentation but without spatial smoothing on 160 brain regions (6mm spheres) obtained using Dosenbach's atlas. Correlation matrix for each group was constructed from 160*160 pearson correlation coefficients, followed by estimation of a bias matrix for each subject using jack-knife bias estimation. Bias values for each pair of nodes in an individual subject quantified the contribution of that subject to the overall within-group covariance. Higher positive values meant greater covariance between the two given nodes in that subject, relative to the rest of the group. These bias matrices can be considered equivalent to demeaned and normalised matrices of structural covariance. Structural covariance across all possible regional pairwise connections was tested using 2-tailed voxelwise T-test with FDR correction (p=0.05, 5% rate for false positives).

**Results:** Patients had a significant reduction in structural covariance affecting between right posterior insula and right precentral gyrus (within sensorimotor network, t=3.86, Hedge's g = 1.15); between right posterior insula and left ventral prefrontal cortex (between sensorimotor and salience network, t=3.71, Hedge's g = 1.10); and between right anterior cingulate cortex and right dorsal prefrontal cortex (between sensorimotor and default-mode network, t=3.10, Hedge's g = 0.92). There were no pairwise connections with increased structural covariance among FEP subjects compared to healthy controls.

**Discussion:** Our findings suggest that (1) structural covariance is disrupted even by the time of first-episode of psychosis; thus, the disruptions in morphological relationships reported in schizophrenia are not explicable by antipsychotic usage or illness duration (2) sensorimotor network regions show a predominant disruption in structural covariance, affecting morphological relationships with both salience and default mode regions. The functional and developmental plasticity of sensorimotor networks may be crucial for the early trajectory of psychosis.

### T165. ULTRA-HIGH FIELD MORPHOMETRY IN DRUG-NAÏVE FIRST EPISODE PSYCHOSIS

Tushar Das[1], Kara Dempster[2], Michael Mackinley[2], Peter Jeon[1], Joe Gati[3], Jean Theberge[1], Ali Khan[3], Lena Palaniyappan*[,1]

*[1]University of Western Ontario; [2]London Health Sciences Center; [3]Robarts Research Institute*

**Background:** Structural neuroimaging studies report distributed grey matter volume (GMV) deficits in drug-naïve first episode psychosis (FEP), though their relevance to symptom burden and cognitive deficits is currently unclear. When compared to studies in medicated patients and/or patients with established later-stage of psychosis, the GMV deficits reported have been limited in both spatial distribution and effect size, indicating the possibility of stage-specific progression during the clinical course of psychosis. TOPSY (Tracking Outcomes of Psychosis) is one of the first studies intending to track the neurobiological trajectory using ultra-high field (7T) imaging starting from a drug-naïve first episode state. Here, we report the initial findings from the voxel-based morphometry (VBM) of GMV. To our knowledge, this is the first VBM report from drug-naïve FEP subjects obtained using a 7T MRI acquisition.

**Methods:** We used ultra-high field (7 Tesla) MRI in 28 patients with FEP (satisfying criterion A of DSM-5 schizophrenia) and 18 controls, to evaluate differences in the grey matter. Volume in a voxelwise manner. FEP and controls were matched for age, sex and parental socioeconomic status. Patients were recruited at an early intervention unit (PEPP, London Ontario) and had active psychotic symptoms at the time of scanning. We also obtained abbreviated PANSS (8 items) scores to index the severity of psychosis. Analysis was done using SPM12, after DARTEL based registration and segmentation but without spatial smoothing. 2-tailed voxelwise T-test with FDR correction (p=0.05, 5% rate for false positives) was used. We used multiple regression analysis to predict the scores from processing speed measure (modified Symbol Substitution Test) and the severity of Delusions and Unusual Thought Content (P1 and G9), the 2 symptoms for which most subjects sought treatment in the first place.

**Results:** Patients had a significant reduction in GMV in left fusiform gyrus (Hedge's g = 1.98, T= 6.7), and increased GMV in the right precuneus (Hedge's g = 1.63, T= 5.5) and lingual cortex (Hedge's g = 1.19, T= 4.0). We did not find any other areas of significant GMV change. Of these 3 circumscribed GMV changes, reduced fusiform GMV was found among FEP patients with lower processing speed (ß=0.45, p=0.04), higher severity of delusions (ß=-0.43, p=0.049) and unusual thought content (ß=-0.59, p=0.01). Increased precuneus GMV was found among FEP patients with higher severity of delusions (ß=0.62, p=0.008) and unusual thought content (ß=0.50, p=0.03). Right lingual changes were not related to the severity of delusions or processing speed scores.
**Discussion:** Our findings suggest that (1) GMV deficits are minimal in drug-naïve FEP subjects, with large effect-size changes concentrated around face processing (fusiform) region (2) GMV increases co-occur with GMV reduction especially in those with most severe delusions and cognitive deficits indicating a role for compensatory plasticity. Subtle early brain structural changes appear to predict symptom burden and cognitive deficits at the time of first clinical presentation with psychosis. Focusing on treatments that manipulate the structure of fusiform cortex could potentially reduce the severity of some of the early symptoms in FEP.

### T166. SPATIAL INCOHERENCE OF LARGE-SCALE CORTICAL NETWORKS RELATES TO FORMAL THOUGHT DISORDER IN SCHIZOPHRENIA: A 7T MRI-BASED THICKNESS STUDY

Lena Palaniyappan*[,1], Ali Al-Radaideh[2], Penny Gowland[3], Peter Liddle[3]
*[1]University of Western Ontario; [2]The Hashemite University; [3]University of Nottingham,*

**Background:** The thickness of cerebral cortex varies across individuals as well as across different regions within an individual. Shared trophic or plastic influences such as repeated task-related recruitment of extant brain regions results in morphological covariance within large-scale brain networks. Pathological processes disrupting functional co-activation can result in higher than expected degree of variability within large-scale networks in an individual level, resulting in spatial incoherence. We studied spatial incoherence of cortical thickness in 17 cortical networks identified on the basis of well-known patterns of intrinsic connectivity, to identify the spatially incoherent networks and relate them to differences in severity of thought disorder among patients with schizophrenia.
**Methods:** Ultra-high field 7T anatomical MRI scans (MPRAGE) were obtained from 20 subjects in a clinically stable, medicated early stage of schizophrenia, and 19 sex, parental socioeconomic-status and age matched healthy controls. Cortical thickness was estimated using Freesurfer v5.0, across 17 networks based on the parcellation scheme of Yeo et al. We computed within-network coefficient of variation in thickness (CVT) across vertices that constitute each network. Higher CVT of a network in a subject indicates higher spatial incoherence within the network for that individual. Independent 2-tailed t-tests were used to compare CVT of 17 networks between the 2 groups with FDR-corrected p=0.05 considered as statistically significant. We related CVT of affected networks to the scores of positive and negative Formal Thought Disorder measured using Thought and Language Index in patients.
**Results:** Salience Network (aka Ventral Attention Network as per Yeo atlas), Default Mode Network and Central Executive Network (aka dorsal Attention Network in Yeo atlas) showed most significant reduction in MRI-derived cortical thickness (networks #8, #12, #15 as well as #16 of Yeo atlas). Only the Salience and Executive Networks (network #8 and #12) showed higher coefficient of variation in patients compared to controls, indicating either a failure of coordinated maturation or co-ordinated function. Higher spatial incoherence of Salience Network related to reduced mean thickness of Central Executive Network in patients with schizophrenia; this relationship was not seen in healthy controls (Fisher's z test, p=0.02). Both higher coefficient of variation in Salience Network and lower mean thickness in Central Executive Network predicted the severity of positive but not negative thought disorder scores.
**Discussion:** Our results indicate that (1) large-scale cortical networks involved in information processing (Salience and Executive Networks) show spatial incoherence in schizophrenia (2) the degree of spatial incoherence relates to the severity of disorganisation of thoughts and language in patients. Spatial incoherence may be the result of a dysmaturational or functional dysplastic effect reflecting inefficient cortical recruitment in schizophrenia. Within-subject morphological variability carries useful information that can potentially explain the elusive neural basis of complex symptoms such as formal thought disorder.

### T167. ABERRANT MYELINATION OF THE CINGULUM BUNDLE IN PATIENTS WITH SCHIZOPHRENIA: A 7T MTI/DTI STUDY

Lena Palaniyappan*[,1], Ali Radaideh[2], Olivier Mougin[3], Penny Gowland[3], Peter Liddle[3]
*[1]University of Western Ontario; [2]The Hashemite University; [3]University of Nottingham*

**Background:** The structural integrity of the anterior cingulum has been repeatedly observed to be abnormal in patients with schizophrenia. Reduced glutathione levels, indicating oxidative stress, is associated with reduced structural integrity of cingulum bundle in patients with schizophrenia. Variations in neuregulin-1, a well-established candidate marker for schizophrenia, results in oligodendrocyte dysfunction and defective myelination, and is shown to affect the structural integrity of the anterior cingulum in patients with schizophrenia. While the evidence to date has been obtained using diffusion tensor imaging, abnormal tract-specific changes in myelin content can be more directly inferred by combining multiple modalities of WM imaging such as diffusion tensor (DTI) and magnetization transfer imaging (MTI) in parallel.
**Methods:** We used ultra-high resolution (7 Tesla) MTI in 17 patients with schizophrenia and 20 controls, to evaluate the macromolecular (predominantly myelin) content of the brain. Immediately after the 7T scanning, we also obtained a 3T diffusion tensor image (DTI) and undertook probabilistic tractography using FSL software (AutoPtx, ProbTrackX) to delineate anterior cingulum bilaterally. Unpaired t tests were used for group comparisons along with estimates of Cohen's d or Hedge's g for effect sizes.
**Results:** Patients had a significant reduction in magnetization transfer ratio (MTR) in right (Cohen's d=0.91, p=0.007) but not left (d=0.03, p=0.92) cingulum bundle. There was also a trend level reduction in fractional anisotropy of right (d=0.60, p=0.07) but not left (d=0.47, p=0.17) cingulum bundle. We did not find any significant relationship between the 3 major symptom dimensions of schizophrenia (Reality Distortion, Disorganization, Psychomotor Poverty) and Cingulum MTR. Patients with Schneiderian delusions (n=5) showed a significantly reduced MTR of left cingulum compared to patients (n=12) with no Schneiderian delusions (Hedges' g=1.36, p=0.02).
**Discussion:** Our findings suggest that MTR changes in anterior cingulum, resulting from either dysmyelination or neuroinflammation, is present in clinically stable patients with schizophrenia despite their medicated status. We lacked sufficient power to detect association between MTR changes of cingulum and symptom dimensions. Nevertheless, our results suggest that MTR changes are of higher magnitude than changes in fractional anisotropy, indicating the sensitivity of measuring myelination as a biological marker of white matter aberrations in schizophrenia.

### T168. STRUCTURAL COVARIANCE AND CORTICAL REORGANIZATION IN SCHIZOPHRENIA: AN MRI-BASED MORPHOMETRIC STUDY

Lena Palaniyappan*[,1], Peter Liddle[2]
*[1]University of Western Ontario; [2]University of Nottingham*

**Background:** In patients with schizophrenia, distributed abnormalities are observed in grey matter volume. A recent hypothesis posits that these distributed changes are indicative of a plastic reorganization process occurring in response to a functional defect in neuronal information transmission. We investigated the structural covariance across various brain regions in early-stage schizophrenia to determine if indeed the observed patterns of volumetric loss conform to a coordinated pattern of structural reorganization.
**Methods:** Structural MRI scans were obtained from 40 healthy adults and 41 age, gender and parental socioeconomic status matched patients with schizophrenia. Volumes of grey matter tissue was estimated at regional level across 90 atlas-based parcellations. Group level structural covariance was studied using a graph theoretical framework.
**Results:** Patients had distributed reduction in grey matter volume, with high degree of localized covariance (clustering) compared to controls. Patients with schizophrenia had reduced centrality of anterior cingulate and insula but increased centrality of the fusiform cortex, compared to controls. Simulating targeted removal of highly central nodes resulted in significant loss of the overall covariance patterns in patients compared to controls.
**Discussion:** Regional volumetric deficits in schizophrenia are not a result of random, mutually independent processes. Our observations support the occurrence of a spatially interconnected reorganization with systematic de-escalation of conventional 'hub' regions. The resulting morphological architecture may be primed for compensatory functions, albeit with a high risk of inefficiency.

### T169. COGNITIVE INSIGHT AND CORTICAL THICKNESS IN SCHIZOPHRENIA

Arpitha Jacob[1], Mariamma Philip[1], Rose Bharath[1], Shivarama Varambally[1], Ganesan Venkatasubramanian[1], Gopikrishna Deshpande[2], Naren Rao*,[1], Ayushi Shukla[1]
*[1]National Institute of Mental Health and Neurosciences; [2]Auburn University*

**Background:** Diminished cognitive insight is exhibited by substantial proportion of patients suffering from schizophrenia and is an important determinant of poor treatment adherence. While the clinical correlates of cognitive insight are well examined the neural correlates of cognitive insight is less explored. We examined relation between cortical thickness and cognitive insight in schizophrenia patients.
**Methods:** We examined 37 schizophrenia patients in comparison with 19 healthy volunteers. We measured cortical thickness using a high resolution anatomical magnetic resonance image and cognitive insight using Beck's Cognitive Insight Scale (BCIS). We measured the difference between schizophrenia patients and healthy volunteers using Analysis of covariance and relation between cortical thickness and BCIS scores in schizophrenia patients using stepwise regression analysis.
**Results:** Patients had significantly thinner cortices than healthy volunteers in orbitofrontal cortex, superior temporal gyrus, occipital cortex, dorsomedial prefrontal cortex and posterior cingulate cortex. Significant positive correlations were found between self-reflection and cortical thickness in posterior cingulate cortex, dorso-medial frontal gyrus, occipital lobe. Significant negative correlations were observed between self-certainty scores and bilateral Posterior cingulate and orbitofrontal cortex.
**Discussion:** We found significant differences in cortical thickness between SCZ and HV in brain regions implicated in cognitive insight. Our findings also suggest higher self-certainty to be associated with thinner cortices in bilateral PCC and OFC. Significant relations between cortical thickness and cortical midline structures supports the critical role of these self-evaluative brain regions in cognitive insight in schizophrenia.

### T170. THE SUPERIOR LONGITUDINAL FASCICULUS: CAN CSD BASED TRACT DELINEATION AND NODAL ANALYSIS CLARIFY THE PRESENCE OF TARGETED DIVERGENT DEVELOPMENTAL STRUCTURAL CONNECTIVITY IN ADOLESCENTS REPORTING PSYCHOTIC EXPERIENCES

Erik O'Hanlon*,[1], Niamh Dooley[1], Colm Healy[1], Mary Cannon[1]
*[1]Royal College of Surgeons in Ireland*

**Background:** Superior Longitudinal Fasciculus (SLF) differences are consistently reported in psychotic disorders. The SLF is a complex large bundle of association white matter fibers that bidirectionally connect caudal, temporal cortex and inferior parietal cortex to locations in the frontal lobe. Advances in tractography methodologies detail four discrete subdivisions (SLF I, II, III and IV, more often referred to as the arcuate fasciculus (AF)). Greater specificity of the SLF subdivisions and associated cognitive networks may clarify the mechanisms of divergent tract developmental and functional aspects associated with psychotic experience symptomology in population based samples of adolescents within the extended psychosis continuum
**Methods:** A case-control sample of 25 adolescents reporting psychotic experiences versus 25 controls (mean age 13.7 years). We employed High Angular Resolution Diffusion Imaging (HARDI) based data with constrained spherical deconvolution (CSD) based fibre tractography to delineate the discrete subdivisions of the SLF including the arcuate fasciculous. Following tract identification, standard diffusion metrics, (fractional anisotrophy (FA), and Diffusivity measures MD, AD and RD), were assessed. A secondary supportive "along-tract" analysis to ascertain more subtle patterns of variation of tract integrity over the tract length was applied. White matter nodal analysis exploring the structural connectivity was applied to investigate additional functional networks recruited by and interconnected via the SFL. We investigate the ability of tractography of the SLF subdivisions and nodal analysis to identify possible differences between adolescents experiencing subclinical psychotic like experiences and those who don't.
**Results:** Our results agree with recent studies of the SLF I and AF (Fernandes-Miranda et al 2015) revealing a pattern of asymmetry of these tracts with more extensive tract bundles being consistently identified in the left hemisphere compared to the right. Along-tract analysis revealed subtle patterns of change in discrete subdivisions of the SLF while nodal analysis shows promise in its ability to define precise organisational networks
**Discussion:** Delineating the SLF subdivisions may clarify potential developmental trajectories between frontal and parieto-temporal speech-related areas contributing to the pathogenesis of psychotic like experiences. These results reveal the presence of aberrant structural connectivity in young adolescents with psychotic experiences.

### T171. REDUCED FRONTAL CORTICAL THICKNESS AND SURFACE IN A 10 YEARS FOLLOW-UP OF EARLY ONSET PSYCHOSIS

Daniel Ilzarbe*,[1], Elena de la Serna[2], Inmaculada Baeza[3], Jose Pariente[4], Adriana Fortea[1], Marina Redondo[1], Nuria Bargallo[7], Josefina Castro-Fornieles[3], Gisela Sugranyes[3]
*[1]Hospital Clinic de Barcelona; [2]Hospital Clinic of Barcelona, Centro de Investigación Biomédica en Red de Salud Mental (CIBERSAM); [3]Hospital Clinic of Barcelona, Centro de Investigación Biomédica en Red de Salud Mental (CIBERSAM), Institut d'Investigacions Biomèdiques Agustí Pi i Sunyer (IDIBAPS); [4]Magnetic Resonance Image Core Facility, Institut d'Investigacions Biomèdiques Agustí Pi i Sunyer (IDIBAPS); [7]Hospital Clinic of Barcelona, Institut d'Investigacions Biomèdiques Agustí Pi i Sunyer (IDIBAPS)*

**Background:** Structural volume loss of cortical gray matter over time in schizophrenia has been widely reported (Vita et al. 2012), and may be more pronounced when the disorder has an onset prior to age 18 (Early Onset Psychosis, EOP; Arango et al. 2008). More recently, studies have focused on measures of cortical morphology. The single study in EOP so far has identified greater loss of cortical thickness (CTH) in patients with schizophrenia over time (van Haren et al. 2011), whereas to our knowledge, no so far study has examined measures of surface area (SA) in EOP following a longitudinal design. We set out to examine measures of both CTH and SA in a sample of EOP at 10-year-follow-up.
**Methods:** Patients with EOP were recruited at first episode, matched by sex and age with healthy controls (HC) and re-assessed at 10 years. Subjects were evaluated clinically and structural T1 volumes were acquired using magnetic resonance imaging at baseline and 10-year-follow-up. Images were preprocessed, segmented and analysed with FreeSurfer. Quality control procedure was carried out by two raters. Images were segmented and CTH and SA values were extracted for each parcellation employing Desikan-Killiany Atlas; these were grouped in frontal, occipital, temporal, parietal and cingulate lobes so as to reduce multiple comparisons. When group or group by time effects were detected, parcellations were individually examined. A linear mixed model was built using Stata IC 13.1 to evaluate the effect of group and time on CTH and SA, including hemisphere as fixed effects and correcting by total intracranial volume and setting a critical p-value of .05.
**Results:** Thirty-nine subjects completed the follow-up. After removing 9 due to poor quality T1 images (technical problems, excess of movement), 28 subjects were finally included (13 EOP, 15 HC). There were no significant differences in age (EOP=26.9 ± 0.6 vs HC=27.2 ± 0.3 at follow-up) or sex distribution (%female: EOP=43% vs HC=38%) between groups. The distribution of diagnosis in the case group was: schizoaffective disorder (n=5), bipolar disorder with psychotic features (n=3), schizophrenia (n=2) and others (n=3).
There was a trend-level group effect in global CTH ($p = .07$) which was significant in the frontal lobe ($p = .014$). EOP exhibited less CTH in the caudal middle frontal ($p = .016$) and pars opercularis ($p = .03$) and orbitalis ($p = .007$) of the inferior frontal gyrus. There was an effect of time in the parietal ($p = .013$) and occipital ($p = .004$) lobes consisting of thinner CTH at follow-up in both groups.
There were no differences in SA between groups. Both showed an increase in total SA ($p < .001$) and for parietal ($p < .001$), temporal ($p = .009$) and occipital ($p = .003$) regions at follow-up. There a group by time effect in frontal SA, consisting of an increase over time in HC and a decrease in EOP ($p = .044$), specifically in medial orbito-frontal cortex ($p = .039$).
**Discussion:** Our results have identified: 1) thinner cortices in frontal regions in EOP compared to HC, which seems to be constant over time; and 2) a decreased in SA in frontal areas in EOP along time, contrasting with HC, whose frontal surface increased at follow-up. These findings are consistent with another study (Greenstein et al. 2006) which also reported reduced CTH in frontal areas in EOP during development, while we found no abnormalities in temporal regions (Vita et al. 2012). Despite the small sample size, to our knowledge this is the longest follow-up of an EOP sample employing magnetic resonance imaging so far.

### T172. MULTIMODAL QUANTIFICATION OF MEMORY CIRCUIT MICROSTRUCTURE IN FIRST EPISODE PSYCHOSIS

Carolina Makowski*[,1], Christine Tardif[1], Gabriel Devenyi[2], Robert Amaral[3], Gabriella Buck[3], Ridha Joober[2], Ashok Malla[2], Jai Shah[2], Mallar Chakravarty[2], Martin Lepage[2]
*[1]McGill University; [2]McGill University, Douglas Mental Health University Institute; [3]Douglas Mental Health University Institute*

**Background:** Integrity of hippocampal subfield structure and associated limbic circuitry subserves various memory processes, a domain that is impaired in psychosis and an important predictor of functional outcome. We use a novel atlas that encapsulates both hippocampal subfields and surrounding white matter (WM), forming the 'memory circuit', to assess volumes with high-resolution MRI, and microstructure with quantitative T1 (qT1). Our aims were to examine 1) group by time interactions on memory measures and the memory circuit, and 2) explore the relationships between the chosen memory measures and limbic structures, informed by results from 1), in a longitudinal sample of first episode of psychosis (FEP) patients.
**Methods:** Nineteen FEP and 20 controls with baseline and 3-month follow-up data were included. Logical Memory and Visual Reproduction Subscales of the Weschler Memory Scale, and MRI scans on a 3T scanner were collected. High-resolution T2-weighted images (0.64 mm3) were input to the MAGeT Brain algorithm to obtain volumes of hippocampal subfields and surrounding WM, defined by fimbria, alveus, fornix, and mammillary bodies. Mean qT1 values for each hippocampal subfield and WM structure were sampled from MP2RAGE (1 mm3) qT1 maps. Linear mixed models were used to assess group by time interactions on memory measures, volumes and qT1. To begin, total hippocampal volumes and WM structure for each hemisphere were examined using a Bonferroni correction for multiple comparisons, followed by post-hoc tests of individual subfields and WM structures. Linear models were then used to assess relationships between baseline memory and change in anatomical measures of interest in FEP. Models controlled for sex, education, age, and brain volume.
**Results:** Significant group by time interactions emerged on bilateral mean WM qT1 (left: $F_{1,65}=9.3$, $p=.003$; right: $F_{1,65}=10.6$, $p=.002$), where it was found that within the FEP group, qT1 (relaxation time in ms) increased over the 3-month follow-up period. Looking at WM structures separately, the interaction was driven by qT1 changes in fimbria, fornix, and mammillary bodies bilaterally (p's<.05). No significant group by time interactions were found with respect to volumes or memory, although a trend-like group by time interaction on right fornix volume was found ($F_{1,64}=5.6$, p-uncorrected=.02). Finally, brain-behaviour relationships were explored, restricting our anatomical measure of interest to mean qT1 values within bilateral WM. Although no tests passed correction for multiple comparisons, there was a trend association between better delayed recall of Visual Reproduction and decreases in qT1 of combined WM on the right hemisphere ($F_{1,11}=3.72$, $p=.08$), driven by changes in qT1 of the right fornix ($F_{1,11}=4.4$, $p=.06$).
**Discussion:** This study reveals significant microstructural changes in WM output circuitry of the hippocampus shortly after a FEP. Specifically, increases in qT1 were found within fimbria, fornix, and mammillary bodies bilaterally. Given that T1 relaxation times are typically shorter in WM, an increase in qT1 may reflect a combination of decreased myelin content and increased inflammation. Furthermore, preliminary data suggest better visual memory at baseline is associated with lower qT1 within WM microstructure over a 3-month period, suggesting that preserved non-verbal memory ability shortly after a FEP may manifest in a protective anatomical phenotype, particularly within the fornix. Given the importance of the hippocampal-fornix circuit in FEP, both with respect to memory and as a theorized hub of pathophysiology in psychosis, a better understanding of WM microstructure in relation to cognitive profiles in patients may offer a new perspective for treatment targets.

### T173. GABA AND GLUTAMATE IN PATIENTS WITH 22Q11.2 DELETION SYNDROME AND HEALTHY VOLUNTEERS AND THE RELATION WITH COGNITION: A RANDOMIZED DOUBLE-BLIND 7TESLA PHARMACOLOGICAL MRS STUDY

Claudia Vingerhoets*[,1], Desmond Tse[1], Mathilde van Oudenaren[2], Esther van Duin[1], Dennis Hernaus[1], Jan Ramaekers[1],

Jaap Janssen[1], Grainne McAlonan[3], Oswald Bloemen[4], Therese van Amelsvoort[1]
*[1]Maastricht University; [2]Academic Medical Center; [3]Institute of Psychiatry; [4]GGz Centraal/Maastricht University*

**Background:** 22q11.2 deletion syndrome (22q11DS) is characterized by a microdeletion on the long arm of chromosome 22. The clinical phenotype of this syndrome is highly variable but symptoms include cognitive impairment, heart malformations, auto-immune problems and a high risk of developing a psychotic disorder. One of the genes located in the deleted region is PRODH which encodes proline dehydrogenase (PRODH). This enzyme is involved in converting proline to glutamate (GLU). GLU is involved in the pathophysiology of psychosis, particularly in cognitive symptoms (Lewis and Moghaddam 2006). Gamma-aminobutyric acid (GABA) is involved in cognition and psychosis as well (Vinkers et al. 2010). With this study we aimed to investigate GLUergic and GABAergic reactivity in the anterior cingulate cortex (ACC) and striatum in medication-free patients with 22q11DS with no psychiatric history and healthy controls (HC).
**Methods:** This was a randomized double-blind placebo controlled cross-over study. Groups were matched for age and gender. 12 patients with 22q11DS (mean age 35 years) and 20 HCs (mean age 31 years) were enrolled in the study. GABA and GLU, levels in the ACC and striatum were obtained twice with 7Tesla Magnetic Resonance Spectroscopy (MRS, STEAM): once after placebo and once after oral administration of 50 mg. riluzole (agent with anti-glutamate and pro-GABA action). Striatal and ACC GLU/GABA ratios were computed as well as GLUergic and GABAergic reactivity (placebo minus riluzole). In addition, within the 22q11DS group, the relationship between cognitive functions (memory and attention) measured with the CANTAB and GABA, GLU, GLU/GABA ratio, GABAergic reactivity and GLUergic in the ACC and striatum were examined.
**Results:** Analyses of Covariance (ANCOVA) showed no baseline group differences in glutamate and GABA levels and GLU/GABA ratios (corrected for fraction of cerebral spinal fluid, CSF) in both brain regions. A repeated measures ANCOVA showed a trend level significant increase in striatal GABA concentrations after (p= 0.065). Riluzole had no significant effect on GLU (p= 0.303) and GLU/GABA ratios (p= 0.150) in the striatum. No medication X group interaction effects were found. Riluzole had no significant effect on GABA (p= 0.101), GLU (p= 0.847) and GLU/GABA ratio (p= 0.108) in the ACC. No group main effects and no medication X group interactions effects were found. However, a significant negative correlation was found between verbal memory (r= -0.650, p= 0.030) and ACC GLU levels, as well as GLUergic reactivity (r= -0.733, p= 0.010). Moreover, in the 22q11DS group, a significant negative correlation was found between attention (target sequence detection) and ACC GLU levels (r= -0.704, p= 0.016) as well as GLU/GABA ratio (r= -0.602, p= 0.050). Furthermore, sustained attention was positively associated with ACC GABA levels (r= 0.700, p= 0.024) and negatively associated with GLU/GABA ratio r= -0.639, p= 0.047) in these patients. Finally, a positive correlation was found between visual memory and striatal GLU levels (r= 0.616, p= 0.043).
**Discussion:** The present study did not demonstrate differences in ACC and striatal GLU and GABA levels, nor in GLUergig or GABAergic reactivity in response to riluzole between 22q11DS patients and controls. However, these results suggest a role for GLU and GABA in cognition in the 22q11DS group. Therefore, influencing these neurotransmitter systems might enhance cognitive functioning in these patients. More studies are required to replicate these findings.

### T174. STRUCTURAL ABNORMALITIES IN THE CINGULATE CORTEX IN ADOLESCENTS AT ULTRA-HIGH RISK WHO LATER DEVELOP PSYCHOSIS

Adriana Fortea*[,1], Phillip van Eindhjoven[2], Jose Pariente[3], Anna Calvo[3], Albert Batalla[2], Elena de la Serna[4], Daniel Ilzarbe[5], Jordina Tor[6], Montserrat Dolz[7], Inmaculada Baeza[8], Gisela Sugranyes[8]
*[1]Hospital Clínic de Barcelona, Universitat de Barcelona; [2]Radboud University Medical Center, Donders Institute for Brain Cognition and Behavior, Centre for Cognitive Neuroimaging; [3]Magnetic Resonance Image Core Facility, Institut d'Investigacions Biomèdiques Agustí Pi i Sunyer (IDIBAPS); [4]Hospital Clinic of Barcelona, Centro de Investigación Biomédica en Red de Salud Mental (CIBERSAM); [5]Hospital Clinic of Barcelona; [6]Hospital Sant Joan de Déu, Centro de Investigación Biomédica en Red de Salud Mental (CIBERSAM); [7]Hospital Sant Joan de Déu, Centro de Investigación Biomédica en Red de Salud Mental (CIBERSAM); [8]Hospital Clinic of Barcelona, Centro de Investigación Biomédica en Red de Salud Mental (CIBERSAM), Institut d'Investigacions Biomèdiques Agustí Pi i Sunyer (IDIBAPS).*

**Background:** Identification of biomarkers of transition to psychosis in individuals at ultra-high risk (UHR) has the potential to improve future outcomes (McGorry, 2008). Structural MRI studies with UHR samples have revealed steeper rates of cortical thinning in temporal, prefrontal and cingulate cortices in individuals who later develop psychosis in both baseline and longitudinal comparisons (Fusar-Poli, 2011; Cannon, 2014). However, little is known about how onset of prodromal symptoms during adolescence impacts on changes in cortical thickness (CTH) (Ziermans, 2012).
**Methods:** Multicentre cross-sectional case-control study, including youth aged 10–17 years, recruited from two child and adolescent mental health centres. UHR individuals were identified using the Structured Interview for Prodromal Syndromes criteria with some modifications. Healthy controls (HC) were recruited from the same geographical area. Exclusion criteria comprised personal history of psychotic symptoms, IQ<70, autism spectrum disorder, presence of neurological disorder, or antecedents of head trauma with loss of consciousness. The study was approved by the local Ethical Review Boards. All participants underwent a comprehensive socio-demographic and clinical evaluation at baseline and after 6, 12 and 18 months follow-up to identify which individuals converted to psychosis (UHR-P) and which did not (UHR-NP).
High-resolution magnetic resonance structural images were acquired at baseline on a 3Tesla and 1.5Tesla scanners. An inter-site compatibility study was conducted with healthy controls which revealed high inter-site correlation coefficients (r>.6) for CTH measures. Images were pre-processed employing automated procedures implemented in FreeSurfer 5.3.0, cortical parcellation employed the Desikan-Killiany brain atlas. Analyses: First, mean global and lobar (frontal, parietal, temporal, occipital, insula and cingulate) CTH measurements were computed. Then, within lobes showing group effects, CTH was measured for each parcellation. ANCOVA was performed to test differences between groups in SPSS 22.0, including gender, age, total intracranial volume and site as covariates. Significance was set at p<.05, corrected using the false discovery rate (FDR).
**Results:** 122 subjects were included (59 UHR-NP vs. 18 UHR-P vs. 45 HC, mean ages: 15.2 ± 1.5 vs. 15.0 ± 1.8 vs. 15.8 ± 1.5, F=1.9, p=.15; gender (%female): 61.0% vs 61.1% vs 68.9%, $\chi2$=.76, p=.68). There were no significant differences in case-control proportion between centres: $\chi2$=1.3, p=.25. No significant differences in global CTH in UHR-P (2.57 ± 0.13mm) relative to UHR-NP (2.56 ± 0.11mm) and HC (2.58 ± 0.09mm) were found.
There was a significant group effect on the right cingulate cortex (F=6.6, pFDR=.024): UHR-P showed lower CTH in this area relative to controls (p=.007 uncorrected). Within the right cingulate cortex, a significant group effect was found in the posterior cingulate (F=5.7, pFDR=.016) and isthmus (F=4.6, pFDR=.024), and a trend level in the caudal anterior cingulate (F=2.9, p=.057): with smaller CTH in UHR-P relative to HC in the isthmus cingulate (p=.025) and the posterior cingulate (p=.066). No significant differences were observed between UHR-P and UHR-NP groups.

**Discussion:** UHR-P showed significant cortical thinning in several regions of the right cingulate cortex in comparison to HC, giving support to the notion that structural alterations in the cingulate cortex may be present in children and adolescents prior the onset of psychosis. Longitudinal changes in CTH have the potential to increase understanding of changes related to transition to clinical illness.

### T175. A 10-YEAR LONGITUDINAL STUDY OF GREY MATTER VOLUME IN FIRST EPISODE OF NON-AFFECTIVE PSYCHOSIS

Diana Tordesillas-Gutiérrez*[,1], Ayesa-Arriola Rosa[2], Victor Ortiz-García de la Foz[3], Esther Setien-Suero[2], Javier Vázquez-Bourgon[3], Benedicto Crespo-Facorro[3]
*[1]Valdecilla Biomedical Research Institute IDIVAL; [2]Marqués de Valdecilla University Hospital, IDIVAL, CIBERSAM; [3]University Hospital Marques de Valdecilla – IDIVAL, University of Cantabria, CIBERSAM*

**Background:** Structural abnormalities in First Episode of Non- Affective Psychosis (FEP) are shown to be present at the time of onset of the illness. Although there are multiple cross-sectional studies in chronic patients there is no clear evidence how these alterations progress years after the appearance of the first episode.
**Methods:** Data for the present investigation were obtained from an ongoing epidemiological and longitudinal intervention programme of first-episode psychosis (PAFIP) conducted at the Marqués de Valdecilla University Hospital (HUMV), Spain. Images for 62 FEP patients and 47 healthy controls were acquired at baseline and 10 year follow-up on the same 1.5-T whole-body scanner (SIGNA, GE, Milwaukee, WS, USA). Three-dimensional T1-weighted images, using a spoiled gradient-recalled acquisition in the steady state (GRASS) (SPGR) sequence, were acquired in the coronal plane with the following parameters: TE=5 msec, TR =24 msec, NEX=2, rotation angle =45°, FOV= 26 x 19.5 cm, slice thickness =1.5 mm and a matrix of 256 x 192.
Structural imaging data for each subject was analyzed using serial longitudinal Statistical Parametric Mapping software (SPM12). After segmenting the mid-point average and multiply the result by the jacobian maps, DARTEL was applied to spatially normalise de diferences. T-test between both groups was performed, allowing voxel-wise comparison of progressive structural change. All results were p<0.05 FWE corrected.
**Results:** FEP patients exhibited progressive bilateral atrophy of the anterior cingulate bilaterally, the right inferior orbital, middle and superior frontal giri, left precentral and postcentral giri and cerebellum. We found no areas were grey matter was greater in controls than in patients.
**Discussion:** In this study we analyze a well characterized sample of patients with a first episode of non-affective psychosis in the first weeks after onset and 10 years later. Our results confirm that, apart from the grey matter volume reduction presented at baseline, patients show a progressive grey matter loss in anterior cingulate, frontal and parietal lobes as well as cerebellum.

### T176. REDUCED WHITE MATTER ‘CONNECTIVITY’ IN THE SPLENIUM OF THE CORPUS CALLOSUM IN TREATMENT-RESISTANT SCHIZOPHRENIA

Idaiane Assunção-Leme[1], André Zugman[2], Luciana Monteiro de Moura[3], João Ricardo Sato[4], Deyvis Rocha[5], Bruno Bertolucci Ortiz[2], Cristiano Noto[1], Vanessa Ota[1], Sintia Belangero[1], Rodrigo Bressan[2], Andrea Parolin Jackowski[2], Ary Gadelha*[,5]
*[1]Laboratório Interdisciplinar de Neurociências Clínicas(LiNC); [2]Laboratório Interdisciplinar de Neurociências Clínicas(LiNC), Universidade Federal de São Paulo(UNIFESP); [3]Universidade Federal do ABC (UFABC); [4]Universidade Federal do ABC, Laboratório Interdisciplinar de Neurociências Clínicas(LiNC), Universidade Federal de São Paulo(UNIFESP); [5]Universidade Federal de São Paulo (UNIFESP)*

**Background:** Resistance to treatment affects up to 30% of patients with schizophrenia (SCZ). Current criteria for treatment-resistant schizophrenia (TRS) require failure to respond to two antipsychotic trials for adequate dose and duration. Clozapine is the only antipsychotic that is more effective to treatment resistant patients. Increasing evidence suggest that TRS may represent a subgroup of patients with distinct biological signature. Brain dysconnectivity was proposed as a major feature of schizophrenia and more intense in TRS patients. Earlier identification of TRS may anticipate the clozapine trial and, thus, reduce disability and treatment costs. In our study, we investigated whether there were differences in white matter integrity among first episode of psychosis (FEP), treatment-resistant schizophrenia (TRS), and non treatment-resistant schizophrenia (NTRS) patients.
**Methods:** Diffusion-tensor brain MRI images were obtained for 34 TRS (19 males), 50 NTRS (26 males) and 35 FEP individuals (18 males), on a Siemens 1.5T MRI scanner. Treatment resistance was defined as persistence of moderate to severe symptoms, after failure to respond to 4–6 week trials of at least two different antipsychotic medications in adequate doses (equivalent to at least 400 mg/day of chlorpromazine or 5 mg/day of risperidone). All participants were receiving antipsychotic medication. All TRS patients were in clozapine use. Analysis of diffusion parameters was performed using a tract-based spatial statistics (TBSS), yielding a total two contrasts: i) mean FA is lower (or higher) in the TRS compared to the FEP, ii) mean FA is lower (or higher) in the NTRS compared to the FEP corrected for multiple comparisons using family-wise error (FWE) < 0.05. Gender and age were used as covariates.
**Results:** FEP patients were younger than TRS (mean±SD; 27.2 ± 7.93 y/o vs 37.06 y ±7.98 y/o;t=5.08, p <0.001) and NTRS (27.2 ± 7.93 y/o vs 37.71 y ±11.18 y/o; t=4.57, p<0.001) patients. Reduced in FA value was observed in the splenium of the corpus callosum (CC) in TRS patients when compared to FEP (47,598 voxels and thresholded at p<0.05). No differences between NTRS and FEP patients were observed.
**Discussion:** Our results showed reduced FA value in the splenium of the CC in TRS when compared to FEP. The splenium of corpus callosum connects the temporal and occipital cortices, and have been previously associated with schizophrenia, but not specifically to treatment resistance in schizophrenia. Our data might suggest that patients with resistance to treatment have inefficiency in the connectivity of the white matter between these regions. Further studies will be required to replicate these findings and to explore the significance of white matter changes in the brain in order to determine if these are consequence of disease progression or related to clozapine exposure.

### T177. STRUCTURAL ORGANIZATION OF THE PRAXIS NETWORK PREDICTS GESTURE PRODUCTION: EVIDENCE FROM HEALTHY SUBJECTS AND PATIENTS WITH SCHIZOPHRENIA

Petra Viher*[,1], Ahmed Abdulkadir[2], Katharina Stegmayer[2], Peter Savadijev[3], Marek Kubicki[3], Nikos Makris[3], Sarina Karmacharya[3], Andrea Federspiel[2], Stephan Bohlhalter[2], Tim Vanbellingen[2], René Müri[2], Roland Wiest[4], Werner Strik[2], Sebastian Walther[2]
*[1]University Hospital of Psychiatry Bern; [2]University Hospital of Psychiatry Bern, University of Bern; [3]Brigham and Women´s*

*Hospital, Harvard Medical School; [4]Institute of Neuroradiology, University of Bern*

**Background:** Hand gestures are an integral part of social interactions and are involved in nonverbal and verbal communication. The convey language that is expressed by motor actions, and thus depend on the interplay of various brain regions. Several functional magnetic resonance imaging studies in healthy subjects suggest the praxis network for gesture production, involving distinct frontal, parietal and temporal regions. Lesions studies in subjects with apraxia, following left brain damage corroborate these findings. However, little is known about the structural connectivity underlying gesture production. We aimed to provide novel insights into the structural connectivity of the praxis network and how it is related to gesture production.
**Methods:** Our sample consisted of 41 healthy subjects and of 40 patients with schizophrenia, demonstrating gesture impairments and structural network abnormalities. All participants performed a gesture production test, the test of upper limb apraxia and underwent diffusion weighted magnetic resonance imaging. Finsler geometry was used to investigate structural connectivity and graph theory to estimate global and local efficiency of the praxis network, which consists of 13 bilateral regions of interest.
**Results:** Our findings showed an association of gesture production with network attributes and specific connections within the praxis network. Thus, global and local efficiency and most of the intra- and interhemispheric connections within the gesture network predicted gesture production across groups. Global efficiency of the praxis network further predicted gesture production only in the patient group. Local efficiency of many ROIs and connections of interest predicted production in patients at trend-level. In contrast, there were no significant or trend-level associations of gesture production with network attributes in controls.
**Discussion:** The results revealed an association of impaired gesture performance with structural alterations of the praxis network, including global and local efficiency and many connections of interest. Our findings are of great importance in the understanding of the structural correlates of gesture production and shed further light on the neural underpinnings of gesture deficits in a patient group with severe social deficits.

### T178. PRIOR SUB-THRESHOLD PSYCHOTIC SYMPTOMS ASSOCIATED WITH THICKER RIGHT INFERIOR FRONTAL GYRUS AMONG PATIENTS IN A FIRST EPISODE OF PSYCHOSIS

Rachel Rosengard*[,1], Carolina Makowski[1], Jai Shah[2], Ashok Malla[2], Ridha Joober[2], Martin Lepage[2]
*[1]McGill University; [2]McGill University, Douglas Mental Health University Institute*

**Background:** Individuals with attenuated or sub-threshold psychotic symptoms (STPS) are considered at-risk for psychosis. The notion that STPS represent "early psychosis" holds promise as it suggests the possibility of charting the developmental course of psychotic illness with neuroimaging. However, recent evidence suggests that a significant minority of patients in a first episode of psychosis (FEP) do not recall pre-onset STPS, suggesting diversity in early positive symptom course. This diversity may be reflected at the level of neurodevelopment. While imaging studies of at-risk youth and FEP patients reveal progressive trends in cortical thinning across stages of illness, none have considered the STPS history of FEP patients. To better understand neurobiological trends across illness stages, we investigate the relationships between STPS history and cortical thickness in FEP patients using a whole-brain approach.
**Methods:** Patients (N=93) were recruited from a specialized early intervention clinic for FEP at the Douglas Mental Health University Institute. The Circumstances of Onset and Relapse Schedule was administered to identify youth who recalled at least one of nine expert-selected STPS prior to their FEP (STPS+, N=67) compared to those who did not (STPS-, N=26). These STPS include: Suspiciousness or odd ideas of reference, odd or bizarre ideas that are not delusional, unusual or eccentric behavior, unusual perceptual experiences that are not clearly psychotic, disorganized or odd speech, inappropriate affect, hallucinations or delusions (sub-threshold), and passivity experiences. Age and sex-matched healthy controls were recruited (N=83) for comparison. Participants were scanned on a 1.5T MRI scanner between 1 and 3 times at baseline, at 1-year follow-up, and at 2-year follow-up. Structural T1-weighted images were processed through the CIVET 2.1 pipeline. Cortical thickness values of 320 scans (143 HC, 123 STPS+, 54 STPS-) that passed quality control were extracted for group analysis. Linear mixed effects models controlling for effects of age, sex, education, and mean whole-brain thickness were applied to obtain vertex-wise F-test maps comparing groups.
**Results:** Post-hoc vertex-wise t-test maps were thresholded with Random Field Theory (p-cluster=0.001) and revealed that compared to controls, only STPS- patients exhibited significantly thinner cortical thickness in the right inferior frontal gyrus (peak t(162.3)=4.13, p<0.001). Examination of mean cortical thickness within this cluster, comparing patient groups only, revealed that compared to STPS+ patients, STPS- patients exhibited significantly thinner cortical thickness (t(172)=-2.55, p=0.01). This difference was most pronounced at baseline.
**Discussion:** These results indicate that within the right prefrontal cortex, STPS+/- patients may undergo different cortical maturation trajectories leading up to and through a first episode of psychosis. These differences may explain differential vulnerability to sub-threshold psychotic symptomology before a full-blown episode. In addition to suggesting differential underlying neurobiology related to STPS, these results suggest the importance of considering STPS history in mapping the trajectories of cortical thickness among FEP patients.

### T179. DO INDIVIDUALS IN A CLINICAL HIGH-RISK STATE FOR PSYCHOSIS DIFFER FROM HEALTHY CONTROLS IN THEIR CORTICAL FOLDING PATTERNS?

Rachele Sanfelici*[,1], Dominic B. Dwyer[1], Anne Ruef[1], Linda A. Antonucci[2], Nikolaos Koutsouleris[1], PRONIA Study Group
*[1]Ludwig Maximilian University; [2]University of Bari "Aldo Moro"*

**Background:** Volumetric brain differences between persons meeting criteria for a clinical high-risk state for psychosis (CHR) and healthy controls (HC) have been previously reported, yet little is known about potential abnormalities in surface-based morphological measures. Gyrification (i.e., the amount of cortical convolution) remains relatively stable across the lifespan and is minimally influenced by ubiquitous confounding factors (e.g., drug use, medication, or stress). Recently, a multi-site analysis conducted in 104 CHR persons found global increases in cortical gyrification compared to HC (Sasabayashi et al. 2017). If replicated, gyrification abnormalities in CHR could potentially serve as early neuromarkers of elevated risk, and thus could eventually be used to identify objectively and efficiently the CHR state.
**Methods:** A total of 124 CHR and 264 HC subjects were recruited as part of the PRONIA consortium (www.pronia.eu), a large-scale international longitudinal study currently consisting of 10 European sites. Cortical surfaces were reconstructed from structural MRI images using a volume-based, newly introduced technique called the Projection-Based-Thickness (PBT) as available in the SPM-based-toolbox CAT12. Local gyrification was quantified automatically across the whole brain as absolute mean curvature for each vertex of the brain surface mesh consisting of thousands of individual measurement points. Vertex-wise differences of curvature values were calculated applying a General Linear Model, corrected for age, gender and site effects. Results were investigated at corrected and uncorrected levels.

**Results:** We found no significant differences in vertex-wise gyrification between CHR and HC at either corrected or uncorrected levels (p>0.05). Further investigations of potential confounding site effects also did not reveal differences.
**Discussion:** Our preliminary findings suggest that CHR subjects do not show whole-brain gyrification abnormalities when compared with healthy subjects. These negative results agree with literature suggesting that cortical convolution might be more affected by neurodevelopmental or genetic factors, and thus deviations from normal patterns might not be detectable in heterogeneous samples of at-risk subjects wherein the etiology and ultimate prognosis is unknown. In order to better investigate differences in cortical folding and address the role of gyrification as neuroanatomical biomarker for psychosis, future investigations should focus on subgroups within CHR populations (e.g. patients groups defined by basic symptoms, ultra-high risk, or familial risk) in addition to specific analyses of individuals with higher neurodevelopmental (e.g., obstetric complications) or genetic (e.g., polygenic risk) loadings.

### T180. LOWER GLUTAMATE LEVEL IN TEMPORO-PARIETAL AREA MAY PREDICT A BETTER RESPONSE TO TDCS IN SCHIZOPHRENIA: A PILOT STUDY

Junhee Lee*[,1], Youngwoo Yoon[2], Andrea Wijtenburg[3], Laura Rowland[3], In Chan Song[4], Kang Ik Cho[2], Minah Kim[4], Tae Young Lee[4], Jun Soo Kwon[4]
*[1]Seoul National University Hospital; [2]Institute of Human Behavioral Medicine, SNU-MRC; [3]Maryland Psychiatric Research Center; [4]Seoul National University College of Medicine*

**Background:** Transcranial Direct Current Stimulation (tDCS) is a non-invasive neuromodulation technique which uses a weak electric current from electrodes across the scalp to modulate targeted brain areas. It has been suggested that tDCS may be useful in reducing psychotic symptoms such as auditory hallucination. The aim of this study was to find alteration of key neurotransmitters in schizophrenia in temporo-parietal area (TPA) after tDCS intervention, using magnetic resonance spectroscopy (MRS) technique.
**Methods:** Ten schizophrenia patients with auditory hallucination were recruited from the outpatient clinic of Seoul National University Hospital (SNUH). The anode was placed over the left dorsolateral prefrontal cortex (DLPFC), and the cathode was placed over the left TPA. Patients underwent MRS scan with the very short echo time phase rotation STEAM sequence before and after the tDCS sessions, respectively.
**Results:** Seven of the participants completed MRS scans before and after the tDCS sessions. Positive and Negative Symptom Scale (PANSS) total and general psychophathology scale showed a significant improvement after tDCS. There was no significant difference between glutamate/creatinine (Cr) level before and after tDCS sessions. However, a significant positive correlation between the pre-tDCS glutamate/Cr value in left TPA and the improvement in auditory hallucination measured by Auditory Hallucination Rating Scale (AHRS) after tDCS was found.
**Discussion:** The results of this investigation show that the schizophrenia patients whose auditory hallucination benefits the most from tDCS treatment had lower glutamate/Cr level in left TPA. Previous studies regarding the relationship between glutamatergic system and treatment response mostly have only focused on the frontal area and striatum. However, this study suggests a potential role of glutamatergic system in TPA in predicting treatment response of auditory hallucination.

### T181. ABNORMAL FRONTAL AND PARIETAL SYNAPTIC GAIN RESPONSES IN FIRST EPISODE SCHIZOPHRENIA DURING A P300 TARGET DETECTION TASK

Alvaro Diez*[,1], Alba Lubeiro Juárez[1], Benjamín Cañas[2], Aldara Alvarez-Astorga[2], Angel Nevado[3], Vicente Molina[1]
*[1]Universidad de Valladolid; [2]Hospital Clínico Universitario de Valladolid; [3]Universidad Complutense de Madrid*

**Background:** The "dysconnection hypothesis" proposes that schizophrenia is best understood in terms of aberrant brain functional integration and synaptic neuromodulation, which may underlie illness psychopathology and cognitive decline. Impairments in the P300 potential are well documented in schizophrenia and progressive over the years with the illness. We used Dynamic Causal Modeling (DCM) to investigate intrinsic (self-) connectivity in a frontoparietal cortical hierarchy during a P300 task; that is, how evoked activity results from the dynamics of coupled neural populations and how neural coupling changes with the experimental factors.
**Methods:** Thirty-one patients with schizophrenia (16 first episode and 15 chronic patients) and 31 healthy controls underwent EEG recordings during an auditory oddball paradigm to elicit the P300 response. We studied 16 frontoparietal models (primary auditory, superior parietal, and superior frontal sources) and identified an optimal model of neural coupling, explaining illness 'diagnosis' and 'chronicity' effects, as well as their interactions with 'task condition'.
**Results:** The winning model included changes in connectivity in all 3 hierarchical levels. Compared to healthy controls, all patients (chronic and first episode) showed decreased self-inhibition – i.e., increased cortical excitability – in right superior parietal gyrus across task conditions. On the other hand, first episode patients – but not chronic – showed in the left frontal and parietal source a reversal of the normal synaptic gain changes in response to targets, relative to standard tones.
**Discussion:** We confirmed that both subjects with chronic and first episode schizophrenia show a context-independent loss of parietal synaptic gain control. Importantly, in the highest levels of the hierarchy, first episode patients showed a specific abnormal gain modulation pattern in response to task-relevant stimuli not present in those chronically treated. Abnormal synaptic gain is plausibly caused by NMDA-receptor and/or GABAergic pathologies that change the excitability of superficial pyramidal cells, and may be independent of illness advance and chronic pharmacological treatment.

### T182. SHARED AND DISTINCT ALTERATIONS IN THE WHITE MATTER TRACTS OF REMITTED AND NON-REMITTED PATIENTS WITH SCHIZOPHRENIA

Tzung-Jeng Hwang*[,1], Jing-Ying Huang[2], Chih-Min Liu[1], Yu-Jen Chen[2], Yung-Chin Hsu[2], Hai-Gwo Hwu[1], Yi-Tin Lin[1], Ming-Hsien Hsieh[1], Chen-Chung Liu[1], Yi-Ling Chien[1], Wen-Yih Isaac Tseng[2]
*[1]National Taiwan University Hospital; [2]National Taiwan University*

**Background:** Antipsychotic drugs are the standard treatment for schizophrenia; however, the treatment outcomes vary. Different treatment outcomes may be attributed to the genetic and molecular heterogeneity of patients, which may be represented in the white matter structures of the brain. In the present study, we assessed the association between white matter tract integrity and treatment outcomes in patients with schizophrenia.
**Methods:** We evaluated 96 patients with schizophrenia (remitted, 53; non-remitted, 43) and 50 healthy controls through diffusion spectrum imaging with a 3 Tesla magnetic resonance imaging scanner. Patients were categorized into the remission and non-remission groups according to the criteria proposed by The Remission in Schizophrenia Working Group (RSWG) on the basis of PANSS scores. White matter tract integrity was assessed through an automatic tract-specific analysis method to determine the mean generalized fractional anisotropy (GFA) values of the 76 white matter tract bundles in each participant.
**Results:** Analysis of covariance revealed that 7 tracts, namely the bilateral fornices, the bilateral uncinate fasciculi, and the callosal fibers (CFs) of the bilateral temporal poles, bilateral hippocampi, and bilateral amygdalae, had significantly different GFA values among the 3 groups. Posthoc between-groups analysis showed that the non-remission group had lower GFA values in all 7 tracts than the control group; the remission group had lower GFA values than the control group only in 4 tracts, namely the bilateral fornices and the CFs of the bilateral temporal poles, and bilateral hippocampi. Compared with the remission group, the non-remission group had lower GFA values in all 7 tracts.
**Discussion:** All 7 tracts that were altered in the non-remission group are a part of the limbic system, which supports various functions, including emotions, memory, and learning. Our results suggest that patients who had poor outcomes to antipsychotic treatments might have more severe disruptions in the limbic system. The 7 altered tracts in the non-remission group are compatible with those reported in previous studies on white matter or gray matter alterations. In a cross-sectional tractography-based study on 3 pairs of association fibers (i.e., the cingulum, superior longitudinal fasciculus, and uncinate fasciculus), Luck et al reported that compared with patients with good outcomes, patients with poor outcomes had reduced FA in the uncinate fasciculus and superior longitudinal fasciculus. Marques et al performed a longitudinal study using tract-based spatial statistics and reported that non-responders had more tracts with a significantly lower FA than did the responders, particularly in the uncinate fasciculus and corpus callosum. In addition to the uncinate fasciculus, we also observed reduced fiber integrity in the bilateral fornices and the CFs of the bilateral temporal poles, bilateral hippocampi, and bilateral amygdalae; these tracts connect the gray matter in the limbic system. Jääskeläinen et al revealed that a reduction in gray matter volume in the frontal and limbic areas is associated with overall poor outcomes. In addition, Van Haren et al reported significantly reduced gray matter volumes in the frontal and temporal cortices of the individuals with poor outcomes. Because the gray matter regions are anatomically connected by the fiber tracts, gray matter reduction in the limbic system might affect the interconnecting fiber tracts; this finding accords with the findings of the present study.
In conclusion, differences in the severity of white matter tract alterations in the remission and non-remission groups might indicate biologically distinct subgroups in schizophrenia.

### T183. AUDITORY-STEADY-STATE RESPONSES AND CORTICAL VOLUME IN PATIENTS WITH SCHIZOPHRENIA

Do-Won Kim*[,1], Sungkean Kim[2], Seon-Kyeong Jang[3], Jihoon Kang[1], Chang-Hwan Im[2], Seung-Hwan Lee[4]
[1]*Chonnam National University;* [2]*Hanyang University;* [3]*Clinical Emotion and Cognition Research Laboratory;* [4]*Clinical Emotion and Cognition Research Laboratory, Inje University IIsan Paik Hospital*

**Background:** The 40-Hz auditory steady-state response (ASSR) probing gamma-band oscillations may reflect N-methyl-D-aspartate receptor (NMDAR) dysfunction in patients with schizophrenia (SZ). Diminished gamma oscillations are reported in SZ, although increased spontaneous gamma oscillations are also reported. We investigated the 40-Hz ASSR and its association with brain volumes and clinical symptoms of SZ.
**Methods:** The 40-Hz ASSR was measured using electroencephalography in 33 patients with SZ and 30 healthy controls (HCs). Four gamma oscillation components (evoked power, spontaneous oscillations (baseline and total power), and inter-trial phase coherence (ITC)) were assessed. Brain volumes were assessed using high-resolution magnetic resonance imaging and voxel-based morphometry.
**Results:** Patients with SZ had larger evoked and total powers and higher ITC than HCs. In HCs, evoked power showed significant positive correlations with bilateral superior temporal gyrus (STG) volume. In SZ, the effect of positive symptoms on the path from evoked power to left STG volume was significantly moderated. In SZ with elevated positive symptoms, large evoked power predicted small left STG volume, whereas large evoked power predicted large left STG volume in those with low positive symptoms. Increased baseline power was associated with a smaller left middle frontal gyrus (MFG) volume in SZ, whereas increased ITC correlated with larger MFG volume in HCs.
**Discussion:** Our results support the NMDAR hypofunction model of SZ, and suggest significant involvement of the STG and MFG in gamma oscillations.

### T184. BRAIN-WIDE FUNCTIONAL DYSCONNECTIVITY IN SCHIZOPHRENIA: PARSING DIATHESIS, RESILIENCE AND THE EFFECTS OF CLINICAL EXPRESSION

Shuixia Guo[1], Wei Zhao[1], Haojuan Tao[2], Liu Zhening[2], Lena Palaniyappan*[,3]
[1]*Hunan Normal University;* [2]*Mental Health Institute, The Second Xiangya Hospital of Central South University;* [3]*University of Western Ontario*

**Background:** The functional dysconnectivity observed from resting-state fMRI studies in schizophrenia is also seen in unaffected siblings indicating its association with the genetic diathesis of the illness. Nevertheless, when compared to patients, the extent of dysconnectivity appears to be limited both in spatial distribution and magnitude in siblings, suggesting that some of the abnormalities could be exclusively linked to the clinical expression or treatment effect rather than genetic diathesis. We investigated brain-wide functional connectivity using a graph theory approach to apportion resting-state dysconnectivity into components that represent genetic diathesis, clinical expression or treatment effect and resilience.
**Methods:** Resting state functional MRI data acquired from 116 subjects (28 patients with schizophrenia, 28 unaffected siblings and 60 matched healthy controls). Based on Dosenbach's atlas applied to 6 minutes (180 time points with TR=2 s) of eyes-open resting fMRI scan, we extracted time series of 160 functional network nodes. After constructing a 160*160 functional network, we investigated between-group differences in strength and diversity of functional connectivity and topological properties of undirected graphs constructed from thresholded correlation matrices. We also used Support Vector Machine approach to estimate the ability of functional connectivity metrics to discriminate the three groups from each other.
**Results:** Using ANOVA [FDR corrected $p<0.05$], we found 88 out of 12720 pairs of functional links to be significantly different among the three groups. 48.8% of these 88 links included nodes from the Default Mode Network (DMN), with the largest portion of these involving Salience Network/DMN connectivity (14.8%). Post-hoc t tests revealed that 62.5% of these disconnected links were associated with genetic diathesis of schizophrenia (i.e. both patients and siblings showing same direction of significant post-hoc difference compared to HC) and 21.6% were associated with clinical expression or treatment effect (i.e. patients differed from siblings and healthy controls, but no difference between controls and siblings). Topologically, we observed increased degree, clustering coefficient and global efficiency but reduced local efficiency in the sibling group compared to both patients and controls, indicating a resilience (or compensation) effect. Support vector machine analysis revealed a high degree of accuracy when classifying the genetically predisposed (patients and siblings) vs. healthy controls (Area Under the Curve - AUC 0.97) or the patient groups vs. healthy controls (AUC 0.97) but not when discriminating patients vs. siblings (AUC 0.58)
**Discussion:** A large portion of the resting-state functional dysconnectivity seen in patients with schizophrenia represent a genetic diathesis effect. The most prominent network level disruption in this context is the dysconnectivity among nodes of the default-mode and salience networks. Despite their predisposition, unaffected siblings show a pattern of resilience in the emergent connectomic topology. Our findings could potentially help refine imaging genetics approaches currently used in the pursuit of the pathophysiology of schizophrenia.

### T185. DIFFERENTIAL ACTIVITY OF TRANSCRIBED ENHANCERS IN THE PREFRONTAL CORTEX OF 592 CASES WITH SCHIZOPHRENIA AND CONTROLS

Panos Roussos*,[1]
[1]*Mount Sinai School of Medicine*

**Background:** Transcription at enhancers is a widespread phenomenon, which produces so-called enhancer RNA (eRNA) and occurs in an activity dependent manner. The role of eRNA and its utility in exploring disease-associated changes in enhancer function and the downstream coding transcripts that they regulate is however not well established. We here used transcriptomic and epigenomic data to interrogate the relationship of eRNA transcription to disease status and how genetic variants alter enhancer transcriptional activity in the human brain.
**Methods:** We combined RNA-seq data from 537 post mortem brain samples from the CommonMind Consortium with cap analysis of gene expression and enhancer identification, using the assay for transposase-accessible chromatin followed by sequencing.
**Results:** We find 118 differentially transcribed eRNAs in schizophrenia and identify schizophrenia-associated gene/eRNA co-expression modules. Perturbations of a key module are associated with the polygenic risk scores. Further, genetic variants affecting expression of 927 enhancers, which we refer to as enhancer expression quantitative loci or eeQTLs, are identified. Enhancer expression patterns are consistent across studies, including differentially expressed eRNAs and eeQTLs. Combining eeQTLs with a genome-wide association study of schizophrenia identifies a genetic variant that alters enhancer function and expression of its target gene, GOLPH3L.
**Discussion:** Here, we expanded the scope of the CommonMind Consortium to interrogate enhancer function in schizophrenia, to examine how genetic variation affects enhancers, and to evaluate specific effects on enhancer and gene expression for previously identified schizophrenia risk variants. Our novel approach to analyzing enhancer transcription is adaptable to other large-scale, non-poly-A depleted, RNA-seq studies.

### T186. ASSOCIATION BETWEEN POLYMORPHISMS OF THE NEUREGULIN 1 (NRG1) GENE AND SCHIZOPHRENIA

Junzhe Xu*,[1], Jianxin Yuan[1], Te-An Chen[1], Anna Li[2], Meghan Helm[2], Steve Dubovsky[1]
[1]*School of Medicine, SUNY at Buffalo;* [2]*VHAWNY Healthcare System*

**Background:** The dysfunction of neuregulin 1 (NRG1) is one of the plausible hypotheses for the pathogenesis of schizophrenia. The neuregulin 1 (NRG1) is located on chromosome 8p, as suggested by multiple linkage studies. The aim of this study is to clarify the contribution of polymorphisms of the neuregulin 1 (NRG1) with schizophrenia
**Methods:** After informed consent was obtained, 100 schizophrenia patients and 100 control subjects were enrolled in this study. All subjects were administered the Diagnostic Interview for Genetic Studies (DIGS) (National Institute of Mental Health-Molecular Genetics Initiative, 1992; Nurnberger et al., 1994) by a research assistant with extensive training in this interview. Blood samples were collected in anonymously identified 10-ml Vacutainer tubes (Becton Dickinson). DNA was prepared by a modified SDS/Proteinase K procedure (Gusells et al., 1979). We genotyped polymorphism neuregulin 1 (NRG1) with the PCR-RFLP methods. The PCR products were digested by restricted enzyme.
**Results:** We observed a significant association between the polymorphism neuregulin 1 (NRG1) and the schizophrenia (Chi-Square Test P= 0.0449).
**Discussion:** The NRG1gene was originally identified as a susceptibility gene for schizophrenia by using a combination of a linkage and association approaches based on microsatellite markers and then using SNPs after microsatellite at risk haplotypes were identified. We found there is the frequency of the polymorphism of neuregulin 1 (NRG1) was significantly increased in schizophrenia patients. This allelic association suggests that the functional polymorphism neuregulin 1 (NRG1) may play a role in susceptibility to schizophrenia. Further study with larger sample sizes is required.

### T187. ALTERED DNA METHYLATION OF THE OXYTOCIN RECEPTOR GENE IS ASSOCIATED WITH SUSCEPTIBILITY TO PSYCHOSIS AND ANHEDONIA-ASOCIALITY IN FEMALES: EPIGENETIC EVIDENCE IN RECENT-ONSET SCHIZOPHRENIA AND ULTRA-HIGH RISK FOR PSYCHOSIS

Minji Bang[1], Se Joo Kim[1], Kyung Ran Kim[1], Su Young Lee[1], Jin Young Park[1], Eun Lee[1], Jee In Kang[1], Suk Kyoon An*,[1]
[1]*Yonsei University College of Medicine;* [2]*Dankook University College of Medicine*

**Background:** Oxytocin is one of the key hormones involved in human social and emotional processing. In this regard, abnormal functioning of the oxytocin system has been suggested to influence on the clinical manifestation of schizophrenia, especially negative symptoms. The aim of the present study was to investigate epigenetic modification of the oxytocin receptor gene (OXTR) and its association with negative symptoms in individuals with recent-onset schizophrenia (SCZ) and at ultra-high risk (UHR) for psychosis.
**Methods:** Sixty-four SCZ patients (< 5 years of duration of illness; 25 men, 39 women), 46 UHR individuals (27 men, 19 women), and 98 healthy controls (HCs; 46 men, 52 women) participated in the present study. DNA methylation was quantified from peripheral blood using pyrosequencing at CpG sites in OXTR intron 1 (hg19, chr3: 8,810,729–8,810,845) and exon 3 (hg19, chr3: 8,809,281–8,809,534). The severity of negative symptoms in clinical groups was measured using the Scale for the Assessment of Negative Symptoms (SANS) and Scale for the Assessment of Positive Symptoms (SAPS).
**Results:** A multivariate analysis of covariance revealed significant differences in OXTR methylation between groups ($F = 16.00$, $p < 0.001$) and gender ($F = 2.84$, $p = 0.025$). Compared to HCs, both UHR and SCZ participants showed lower levels of OXTR intron 1 methylation, particularly at CpG site -934 upstream of the OXTR start codon (HCs = 47.3 ± 4.1 [mean ± SD], UHR = 38.8 ± 4.8, SCZ = 40.2 ± 5.3; $F = 73.74$, $p < 0.001$). Besides, female participants showed higher OXTR intron 1 methylation at CpG site -934 than male participants (male = 42.3 ± 6.1, female = 44.1 ± 5.8, $F = 9.08$, $p = 0.003$). Multiple linear regression analysis with clinical symptoms demonstrated that the degree of DNA methylation at CpG site -934 was significantly associated with the SANS anhedonia-asociality subscale scores in the entire group of female UHR and SCZ participants (beta = -0.44, $p = 0.001$).
**Discussion:** The present study demonstrated decreased OXTR methylation in both UHR and SCZ individuals compared to HCs. Furthermore, the severity of anhedonia-asociality was significantly associated with the degree of OXTR methylation in female UHR and SCZ individuals. These findings suggest that epigenetic aberration of OXTR may confer susceptibility to schizophrenia spectrum psychosis and influence the early pathogenesis of schizophrenia prior to the onset of overt psychosis, particularly in females.

### T188. MASSIVE OPEN ONLINE DISCOVERY 'MOOD' FOR SCHIZOPHRENIA RESEARCH

Madhavi Ganapathiraju*[,1], Narayanaswamy Balakrishnan[2]
*[1]University of Pittsburgh; [2]Indian Institute of Science*

**Background:** We have recently presented Schizophrenia Interactome, i.e., the network of protein-protein interactions (PPIs) of schizophrenia associated genes. PPIs predicted by our High-precision Protein-Protein Interaction Prediction model (HiPPIP) which uses machine learning to classify features of protein-pairs such as colocalization, coexpression, common molecular functions and biological processes could explain the apparent discordance between modern and historical genetic basis of Schizophrenia (published in npj Schizophrenia), and also were instrumental in discovering that OASL interacts DDX58 to activate the RIG-I immunity pathway during viral infection. These novel predicted PPIs were found to be highly accurate based on computational and experimental validations, and gave insights into possible functions of SZ genes that previously had no-known functional information. Even a single novel PPI can have enormous impact on advancement of biology, when translated effectively. How can we ensure that 500+ of these novel PPIs of schizophrenia interactome are translated effectively?
**Methods:** We developed a platform for Massive Open Online Discovery for Schizophrenia Research, or "MOOD for Schizophrenia Research", that brings together trained biologists and bioinformaticians including those who are currently not affiliated with research labs (non-research students, PhDs who gave up science careers for administrative/corporate jobs or to take care of families, etc), as well as scientists who are active researchers, to hypothesize, discuss and prioritize the novel PPIs. Hypotheses are written as nanopublications with authorship credit. We are developing a number of features on the portal that allow and encourage scientists to create knowledge around the predicted PPIs and be recognized and given credit.
**Results:** The first version of our website that disseminates the Schizophrenia Interactome is receiving hundreds of unique users each month. We have since developed MOOD for Schizophrenia Research and will present the key features of the website in this work. Each PPI can be viewed, researched on and written about, by participating scientists. Comprehensive information about the proteins in the PPI regarding their known functions, pathways, diseases and drug associations, is made readily available to scientists, allowing them to hypothesize the importance of the specific PPI. We present methods that we employ to promote collaboration in this work. Initially, the portal starts with a few members and it grows through referral webs (i.e. current members invite new members). The portal has a number of features that recognize and thus entice users to participate. We will also present the user feedback and participation.
**Discussion:** PPIs are central to cellular systems. Yet less than 10% of estimated PPIs are known today. Thus, the computationally predicted PPIs which are deemed accurate, can accelerate advancement of schizophrenia biology research. The time is ripe to benefit from computer science and information technologies methods for not only discovering aspects of computational biology but to create new mechanisms to promote online collaboration to achieve big things as a summation of nanocontributions. The knowledge potential generated through this system would aid various principal investigators in well established (but ill funded) research labs by giving them access to bioinformaticians and biologists around the world who are eager be recognized for their contributions. We present here, not merely a website but a novel approach to promote collaborative research between people with heterogeneous skills and commitments by benefiting from the untapped talent of researchers around the world.

### T189. PEPTIDE SHARING BETWEEN SCHIZOPHRENIA-RELATED PROTEINS AND THE INFLUENZA A VIRUS MAY OFFER A WINDOW INTO THE IMMUNE AETIOLOGY OF PSYCHOTIC DISORDERS

Adrianna Kepinska*[,1], Thomas Pollak[1], Conrad Iyegbe[1], Robin Murray[1]
*[1]Institute of Psychiatry, Psychology & Neuroscience, King's College London*

**Background:** Schizophrenia is a complex disorder in which infection and immune mechanisms are thought to play a role. Epidemiological and ecological studies have implicated influenza infection in particular and it is possible that cross-reactivity, or molecular mimicry, between the influenza virus and brain proteins underlies this association. Proteins might share amino acid sequences, which could thus provide the basis for an autoimmune response that targets endogenous proteins. This study is the first to characterise sequence alignment between schizophrenia-related brain proteins and the proteome of the influenza A virus, and comparing it with sequence alignment in proteins not implicated in schizophrenia.
**Methods:** The software Peptide Match Service (https://research.bioinformatics.udel.edu/peptidematch/index.jsp; Protein Information Resource, University of Delaware and Georgetown University Medical Center) was used to obtain sequence alignments between protein sequences. A case-control study design was used to compare schizophrenia-related proteins to proteins not involved in schizophrenia. Schizophrenia-related proteins were operationalised as proteins found significant in the Psychiatric Genomics Consortium schizophrenia genome-wide association studies (GWAS). The control group consisted of null proteins ($p$-value > .75) in the GWAS. Null proteins were also selected to represent genes expressed in tissues other than central nervous system tissues. Both groups were equalised for the total amino acid count. Perfect pentapeptide matches (i.e. 5 amino acids) in proteins and the influenza proteome were explored.
**Results:** There was a link between schizophrenia-related (GWAS-significant) proteins and presence of perfect matches between proteins and the influenza proteins polymerase acidic protein ($\chi2$ (1) = 5.284, $p$ = .022, two-sided) and RNA-directed RNA polymerase catalytic subunit ($\chi2$ (1) = 6.132, $p$ = .013, two-sided). Pentapeptide-sharing was found to be highly significant between schizophrenia-related proteins and the hemagglutinin precursor ($\chi2$ (1) = 17.723, $p$ = .000026, two-sided). There was no significant difference ($p$ > .05) between schizophrenia-related proteins and proteins not implicated in schizophrenia (GWAS-null proteins) in the frequency of proteins having perfect matches with the influenza A proteins PB2-S1, polymerase basic protein 2, matrix protein 1 and 2, and neuraminidase. However, the result for matrix protein 1 approached statistical significance ($\chi2$ (1) = 3.319, $p$ = .068, two-sided).
**Discussion:** We find evidence to suggest there is significant overlap between the linear structures of proteins involved in schizophrenia and those integral to the influenza virus. Future research should establish the biological relevance of this finding, particularly regarding the antigenicity of the peptide sequences which we have identified. Extra studies should also go beyond sequences and address structural homologies. Future research could assess whether an immune reaction against particular schizophrenia-related proteins is a plausible mechanism contributing to psychotic disorders. Also, exploring peptide sharing in different influenza strains could offer insights into links between influenza pandemics, maternal infection, and psychosis. Elucidating peptide sharing might have implications for schizophrenia risk management and safe influenza prevention.

### T190. ASSOCIATION OF THE HUMAN MU-OPIOID RECEPTOR GENE POLYMORPHISM ASN40ASP WITH SCHIZOPHRENIA

Anna Li*[,1], Jiaxin Yuan[1], Te-An Chen[1], Meghan Helm[1], Steve Dubovsky[2], Junzhe Xu[2]
*[1]VHAWNY HealthCare System; [2]Medical School, SUNY at Buffalo*

**Background:** In humans, opioidergic neurotransmission appears to modulate a variety of behaviors, including the stress response and cognitive processes, as well as anxiety and psychosis. One neurobiological process which may be modified by the Asn40Asp polymorphism of the μ opioid receptor is the HPA axis response to stress. Hypothalamic corticotropin-releasing hormone (CRH) neurons, which affects glucocorticoid release by stimulating pituitary adrenocorticotropin (ACTH) secretion, are directly and indirectly inhibited by β-endorphin-producing neurons via the μ opioid receptor (OPRM). Both exaggerated and blunted HPA responses to stress have been associated with high risk for psychosis. Many studies have suggested that opioids play an important role in response to stress, motivation, and numerous psychiatric entities. The present association study tested the hypothesis that the Asn40Asp substitution polymorphism confers susceptibility to schizophrenia.

**Methods:** After informed consent was obtained, 100 schizophrenia patients and 100 control subjects were enrolled in this study. Genomic DNAs were extracted from peripheral blood by using the modified SDS/Proteinase K procedure. The genotypes of the Asn40Asp polymorphism of the μ opioid receptor were assessed by allele-specific polymerase - chain reaction. The PCR products were digested by restricted enzyme.

**Results:** The frequency of the Asp40 allele was significantly increased in all schizophrenia patients (Fisher's Exact Test P= 0.0118). There were no associations the Asn40Asp polymorphism of the μ opioid receptor with substance dependence among schizophrenia patients and normal control.

**Discussion:** The opioidergic neurotransmitter system plays an important role in regulating activation of the hypothalamic-pituitary-adrenal (HPA) axis. Initial activation of the HPA axis occurs at the level of the paraventricular nucleus of the hypothalamus, where neurons that produce corticotropin releasing factor (CRF) are located [Bell et al., 1998]. CRF neurons in this area express μ-opioid receptors and are under tonic inhibition by neurons of the arcuate nucleus that contains β-endorphin [Wand et al., 1998]. Genetic factors appear to be important modulators of HPA axis activation. The HPA axis appears to be involved, including the normal stress response [Bond et al., 1998; LaForge et al., 2000] and psychosis in which HPA axis dynamics appear to be abnormal. Similarly, there is growing evidence that altered opioidergic neurotransmission and HPA axis dynamics may affect alcohol- and drug-seeking behaviors [Piazza and Le Moal, 1997; Kreek and Koob, 1998].

### T191. RANDOMIZED DOUBLE-BLIND FEASIBILITY STUDY OF A GLUTEN-FREE DIET IN PEOPLE WITH SCHIZOPHRENIA AND ELEVATED ANTIGLIADIN ANTIBODIES (AGA IGG)

Deanna Kelly*[,1], Haley Demyanovich[1], Katrina Rodriguez[2], Daniela Cihakova[2], Monica Talor[2], James Gold[1], Sharon August[1], Robert Buchanan[1], Stephanie Feldman[1], Fang Liu[1], William Carpenter[1], William Eaton[2]

*[1]University of Maryland Baltimore, Maryland Psychiatric Research Center; [2]Johns Hopkins Bloomberg School of Public Health*

**Background:** Deconstructing schizophrenia is important, since different disorders within the syndrome may have distinct pathophysiological mechanisms and treatment targets. Emerging evidence suggests inflammation may play a role in at least a subgroup of people with this illness. One specific subgroup with known inflammation is a group with elevations in antigliadin antibodies (AGA IgG). These elevations (>20 U) are found in about 1/3 of people with schizophrenia. Gliadin is a protein found in wheat, barley and rye. This subgroup with AGA IgG elevations may be distinct as they have fewer positive symptoms, higher kynurenic acid levels, and may benefit from a gluten free diet. The effectiveness of gluten removal has been controversial with mixed results in previous studies, however no former study has examined gluten removal in those with high AGA IgG, that is, the population who may be expected to benefit.

**Methods:** Sixteen people with a DSM-IV-TR diagnosis of schizophrenia or schizoaffective disorder and who also had elevated AGA IgG (≥20 U) were recruited and admitted to an inpatient unit for a 5-week randomized double blind trial. Participants were randomized in a double blind fashion to either a diet containing gluten or a complete strictly enforced gluten free diet. This was accomplished by given 10 gm of gluten flour or 10 gm of rice flour daily in a protein shake, with all meals for all participants being standardized and gluten free. Participants had cooking classes, received cookbooks, and went on shopping trips for gluten free foods and meals. Participants were rated on the Brief Psychiatric Rating Scale (BPRS), Scale for the Assessment of Negative Symptoms (SANS) and the MATRICS Cognitive Battery (MCCB) at baseline and 5 weeks. The study was not powered to find a treatment effect, but designed to examine the feasibility of conducting an inpatient gluten removal study and examine trends in treatment as measured by Cohen's D effect size (ES) differences.

**Results:** Fourteen of 16 people completed the 5-week trial and all tolerated the diet (2 discontinued early in the trial for housing and administrative reasons). The mean age of participants was 37.9 ± 13.2 years, 56% male and 75% African-American. During the clinical trial, participants receiving the gluten free diet had an improvement in negative symptoms as compared to placebo (treatment difference) with an ES=0.53. There was no improvement in BPRS total score or positive symptoms. The MCCB composite score did not improve, but an ES=0.6 was noted in the domain of attention favoring the gluten free diet. The AGA IgG levels decreased by 35% in the five weeks in the gluten free diet group relative to a 17% decrease in the gluten containing group. It is also important to note that the correlation between the change in SANS total score and AGA IgG in the gluten free group (r=0.57) was strong and notably different than the correlation between the change in SANS total score and AGA IgG in the gluten containing group (r=-0.017), suggesting a possible marker of treatment effect. Adverse effects were similar between treatment groups.

**Discussion:** This is the very first study of a gluten free diet in schizophrenia with elevated AGA IgG. This feasibility study suggests that removal of gluten is associated with improvement in negative symptoms and attention, but not positive symptoms. Participants tolerated the diet. The feasibility study provided data to design the now ongoing fully powered confirmatory double-blind trial in people with schizophrenia with negative symptoms using a higher gluten amount (30 grams daily) and with aims to examine associated mechanisms, with targets of inflammation, neuroimaging and gut permeability.

### T192. THE GUT MICROBIOME IN SCHIZOPHRENIA AND ANTIPSYCHOTIC INDUCED METABOLIC DYSFUNCTION

Sarah Kanji[1], Ilona Gorbovskaya[1], Trehani Fonseka[1], Kazunari Yoshida[1], Victoria Marshe[1], Malgorzata MacKenzie[1], Premysl Bercik[2], Elena Verdu[2], Margaret Hahn[1], Daniel Mueller*[,3]

*[1]Centre for Addiction and Mental Health; [2]McMaster University; [3]University of Toronto*

**Background:** Antipsychotic (AP) medications are the cornerstone of treatment for schizophrenia (SCZ), with off-label prescription rapidly increasing in youth and adolescent populations. However, APs have been associated with metabolic side effects including diabetes and obesity. Although several mechanisms have been proposed, the gut microbiome (GMB) has been suggested as a potential mediator of AP-induced metabolic side effects due to its role in weight and metabolic regulation; as well as emerging evidence demonstrating a shift in the microbiome of AP-treated animals and humans. The purpose of the current study is to 1) Investigate the GMB in SCZ patients compared to healthy individuals and 2) To examine the role of GMB in SCZ and AP-induced metabolic side effects.

**Methods:** Three groups of 25 participants are being recruited. Group A: Long-term AP-treated patients (for at least 6 months) taking clozapine (CLZ). Group B: Healthy controls matched with Group A for BMI, age, sex, and smoking status. Group C: Treatment-naive SCZ patients starting an AP or patients newly switching to CLZ. Groups A and B will be assessed at a single time point (week 0) whereas Group C will be assessed prospectively at weeks 0, 3, and 12 with the same measures collected. The following clinical measures and metabolic indices are collected at baseline, and if applicable, at follow up visits: the Questionnaire of Eating and Weight Patterns (QEWP), the Dutch Eating Behavior Questionnaire (DEBQ), the Power of Food Scale (POFS), the Positive and Negative Syndrome Scale (PANSS), the Gastrointestinal Symptom Rating Scale (GSRS), Exercise/Physical Activity Evaluation, body mass index, oral glucose tolerance test, lipid profile, and serum clozapine levels. We also collect fecal samples for DNA extraction and microbiome sequencing. We are currently performing a baseline comparison across groups A, B, and C in order to evaluate intrinsic differences between schizophrenia and healthy controls, as well as, effects of new AP exposure versus chronic exposure. The difference between analytical groups (i.e. groups A, B, and C) at different time points (i.e. baseline, week 3 and 6) for variables such as BMI and metabolic parameters marker change are analyzed using two-way repeated measures ANCOVA, including significant covariates. The relationship between different measures, such as body weight and metabolic parameters, GMB composition (abundance of each species), and AIWG will be tested using Spearman's correlation. Analysis of the Operational Taxonomic Unit network generated using QIIME will be further analyzed using linear discriminant analysis (LDA) effect size (LEfSe) method. This method uses LDA scores to estimate the effect size of differentially abundant taxa (at phyla, class or other levels) and ranks the relative difference of microbial taxa that discriminate groups with biological consistency and statistical significance. The GMB composition of within group weight gainers versus non-weight gainer will also be assessed in a subgroup of subjects using LEfSe.

**Results:** To date, 17 patients enrolled (13 patients in group A (9 men, mean±SD age: 31.6 ± 5.1 years; BMI, 31.4 ± 5.5), two control in group B (1 men, mean±SD age: 29.0 ± 1.4 years; BMI, 30.3 ± 0.8), two in group C (1 men, mean±SD age: 30.0 ± 9.9 years; BMI, 25.8 ± 5.6)). Further analyses including GMB composition in each group are now being conducted. We are currently analyzing these individuals for their GMB composition and results will be presented at the SIRS meeting.

**Discussion:** To our knowledge, this is the first study to assess GMB sample in SCZ and investigate the association the GMB and AP-induced metabolic side effects. Our study has the potential to help to unravel the role of GMB in SCZ and AP response.

### T193. CHRONIC HALOPERIDOL TREATMENT INDUCES SIGNIFICANT CHANGES IN MICROGLIA AND IN THE EXPRESSION OF THE 18 KDA TRANSLOCATOR PROTEIN TSPO IN NAIVE ADULT RAT BRAINS

Marie Cotel*[,1], Ewelina Lenartowicz[2], Sridhar Natesan[1], Maria Dadabhoy[1], Anthony Vernon[1]
*[1]Institute of Psychiatry, Psychology & Neuroscience, King's College London; [2]Research Center for Molecular Medicine of the Austrian Academy of Sciences*

**Background:** Positron emission tomography (PET) using radiolabeled ligands selective for the 18 kDa translocator protein (TSPO) is the most widely used technique to assess putative neuroimmune abnormalities in vivo. However, the results of TSPO PET in schizophrenia patients are ambiguous. In particular, the influence of antipsychotic medication exposure has not been definitively tested. We have previously established that chronic exposure to two different antipsychotics resulted in increased density and amoeboid morphology of brain microglia, particularly in the cortex (ACC) and corpus striatum (STR) (Cotel et al., 2015). Here we explore the consequences of such treatment on TSPO, in relation to changes in microglia. We hypothesize that chronic haloperidol treatment would increase the expression of TSPO in microglia and correlate with increased levels of circulating proinflammatory cytokines.

**Methods:** Adult male Sprague-Dawley rats (12 week-old) were implanted with subcutaneous osmotic mini-pumps (ALZET® 2ML4) delivering a continuous dose of either common vehicle (20% β-cyclodextrin + 5% ascorbic acid) or haloperidol (2mg/kg/d) for 28 days (Kapur et al., 2003). Plasma was collected before perfusing the animals with 4% buffered PFA. Brains were carefully removed and processed for immunostaining against Iba1 and TSPO. Levels of plasma cytokines were measured using the pro-inflammatory 9-plex panel from Meso Scale Discovery (MSD®). TSPO expression was measured by its area fraction using FIJI software (10 snaps per animal per brain region, 20x magnification) in the ACC, STR, hippocampus (HPC), secondary somatosensory cortex (S2), primary motor cortex (M1). Numbers of resting and amoeboid microglia were determined by unbiased stereology (Gundersen, 1987) in the ACC, STR, and HPC. The Cavalieri estimator (StereoInvestigator 12.0,MBF Bioscience) was used to determine the volume of these regions. Data were analyzed by t-tests corrected for multiple comparisons, using GraphPad Prism 6.0.

**Results:** Only 5 cytokines from the panel were above the limit of detection. Of these, IL-6 is significantly increased in haloperidol-treated animals (+186%, p<.01). IL-10, IL-4, KC/GRO, and TNFα plasma levels were not significantly affected. In parallel, TSPO expression was significantly increased in the ACC (+56%, p<.01), the STR (+77%, p<.01) and the S2 (+158%, p<.001). In the STR, the density of both resting and amoeboid microglia were significantly elevated in the treatment group (+22%, p<.05 and +45%, p<.001, respectively). A 2-way ANOVA revealed a significant interaction between treatment and brain region for amoeboid but not resting microglia. Peripheral IL-6 levels correlated negatively with TSPO levels in the STR in saline and haloperidol-treated groups (r=-0.82 and r=-0.62, respectively, p<.05). In addition, KC/GRO, although not different between groups, correlated positively with TSPO levels in STR in controls (r=0.77, p<.05), but negatively in HPC, M1, and S2 after haloperidol treatment (r=-0.66, r=-0.62, r=-0.6, respectively, p<.05).

**Discussion:** As hypothesized, TSPO expression is significantly affected by chronic haloperidol, with augmented levels in the ACC, STR and S2. These data are in line with an increased TSPO PET signal in medicated schizophrenia patients as compared to matched antipsychotic-naïve patients (Holmes et al., 2016). Furthermore, these data confirm and extend our prior findings demonstrating that microglial activation is present already after 28 days of exposure to haloperidol and this effect is most prominent in the STR. Further investigations are needed to determine which cell types drive changes in TSPO (glia, endothelium, astrocytes) and how this relates to their functions at a molecular level.

### T194. BRAIN STRUCTURAL AND BEHAVIORAL ABNORMALITIES FOLLOWING PRENATAL EXPOSURE TO MATERNAL INFLAMMATION ARE PREVENTED BY EARLY BUT NOT LATE INTERVENTION

Yael Piontkewitz*[,1], Michal Arad[1], Ina Weiner[1]
*[1]Tel-Aviv University*

**Background:** Prenatal exposure to the viral mimic poly-I:C via the pregnant dam has been shown to lead to a broad spectrum of neuro-psychopathological features phenotypic of schizophrenia, recapitulating the well established link between maternal infection in pregnancy and increased schizophrenia risk in the offspring. We previously showed using longitudinal imaging that prenatal poly-I:C leads postnatally to volumetric brain abnormalities that precede the emergence of behavioral abnormalities (selective attention deficits and excessive response to amphetamine) in adulthood, and that an ultra-low dose of risperidone (RIS) given prior to "symptom" emergence, prevents the emergence of behavioral as well as structural brain deficits. Here we aimed at showing that the efficacy of RIS

(assessed at adulthood) is a function of time of administration, with earlier but not later being effective.
**Methods:** On gestation day 15, pregnant Wistar rats were injected IV with polyI:C (4 mg/kg/ml) or saline. Offspring received daily injections of RIS (0.045 mg/kg) at 4 different time windows (TW): PNDs 34–47 (TW1), PNDs 48–61 (TW2), PNDs 62–75 (TW3) or PNDs 106–120 (TW4), and underwent structural imaging (scanned on a 7.0 T/30 cm; Bruker, Rheinstetten, Germany) and behavioral assessment of amphetamine-induced activity (AIA) and latent inhibition (LI). Excessive AIA is considered to mimic the exacerbation of psychotic symptoms in response to amphetamine in schizophrenia patients. LI reflects the normal attentional capacity to ignore stimuli that were experienced as irrelevant in the past; disrupted LI is considered to reflect a failure to ignore irrelevant stimuli and is seen in amphetamine-treated rodents and humans, in schizotypal persons, and in acutely psychotic schizophrenia patients. Testing was conducted on PNDs >90 (for TWs 1–3) and PNDs 106 and 150 (for TW4).
**Results:** Poly-I:C offspring had larger lateral ventricles (LV) and smaller prefrontal cortex (PFC), striatal (STR) and hippocampal (HIP) volumes, as well as disrupted LI and enhanced AIA compared to their controls. The effects of RIS on these abnormalities was a function of the administration window. RIS was fully effective when given in TW1 (significant prenatal treatment x drug treatment interaction for each of the brain regions as well as for both behavioral indices, and significant differences between poly-IC-vehicle but not between poly-I:C-RIS and the control groups). In TW2, RIS prevented all abnormalities with the exception of HIP volume reduction (statistics as above except for HIP region). In contrast, in both TW 3 and 4, RIS failed to prevent both structural and behavioral abnormalities (only main effect of prenatal treatment for volumetric and AIA measures and only prenatal treatment x preexposure interaction for LI).
**Discussion:** These results provide the first demonstration that intervention must indeed be provided early in order to be effective, with efficacy declining as the animal matures. Furthermore, they provide strong support for structure-function relationship because there is no prevention of behavioral abnormalities without prevention of structural abnormalities. Finally, since at the 0.045mg/kg dose RIS is a selective 5HT2A/2C antagonist, our results suggest that such antagonism is a promising route for prevention in high risk individuals.

### T195. GAMMA SYNCHRONY IS DYSFUNCTIONAL DURING COGNITIVE PROCESSING IN FIRST ONSET SCHIZOPHRENIA

Anthony Harris*[,1], Annie Brennan[1], Leanne Williams[2]
*[1]University of Sydney; [2]Stanford University*

**Background:** Schizophrenia is characterised by marked cognitive deficits that are central to the disability caused by the disorder. These deficits are present prior to the onset of schizophrenia, are severe by the time of first presentation and persist. They are thought to arise from a core problem in neural connectivity. Cortical connectivity can be measured using changes in the synchronous activity of the brain in the gamma band (30-100Hz) of the electroencephalogram. This study characterizes the profile of gamma synchrony and behavioural performance during higher-order cognitive processing in schizophrenia.
**Methods:** Gamma synchrony was acquired during a Continuous Performance Test (CPT) in 59 young people with First Onset Schizophrenia (FOS) and 59 matched controls We quantitated synchrony for regions associated with the fronto-parietal attention and visual networks. Groups were compared on gamma synchrony for intrinsic (pre-stimulus), task-evoked change (relative to baseline) and absolute (not relative to baseline) measures. The relationship between gamma synchrony, CPT accuracy, psychopathology and functioning was also explored.
**Results:** FOS showed a reduced ability to modulate task-evoked changes in gamma synchrony, in the context of generally higher intrinsic and absolute synchrony, particularly in frontal regions. These gamma synchrony abnormalities in FOS were associated with performance on the CPT, but not with symptoms or functioning.
**Discussion:** This study is the first to demonstrate that gamma synchrony abnormalities are not limited to perceptual or lower-order cognitive processing. Task-relevant changes in synchrony were constrained by an overall excess of intrinsic background synchrony that was unrelated or not amenable to modulation by specific task demands. This excess in regional synchrony disturbed functional connectivity in central executive circuits and affected cognitive performance but not symptomatically. The results are consistent with gamma synchrony being a measure of dysconnectivity, a core abnormality in schizophrenia.

### T196. CALRETININ INTERNEURON DENSITY IN THE CAUDATE NUCLEUS IS LOWER IN SCHIZOPHRENIA

Istvan Adorjan*[,1], Bin Sun[2], Virginia Feher[1], Teadora Tyler[1], Bori Damo-Csorba[1], Benedek Pour[1], Daniel Veres[1], Olaf Ansorge[2], Steven Andrew Chance[2], Francis Szele[2]
*[1]Semmelweis University; [2]University of Oxford*

**Background:** The excitatory/inhibitory imbalance theory is widely accepted in the pathology of autism spectrum disorder. Recent results suggest its relevance in the aetiology of schizophrenia as well (Jardri 2016, Yang 2017, Gao and Penzes 2015). In order to discover the possibly altered neuronal composition in schizophrenia numerous studies have been focussing mainly on different cortical regions such as the ventromedial prefrontal cortex and dorsolateral prefrontal cortex. In particular, various interneuronal populations have been found altered.[2] However, relatively little is known about the neuroanatomical changes of subcortical structures, such as the caudate nucleus, in the pathology of schizophrenia.
**Methods:** Therefore, we examined the immunohistochemical distribution of calretinin (CR) and NPY-immunopositive neurons in the caudate nucleus and the dorsolateral prefrontal cortex. The state of microglial activation was controlled by the detection of Iba1 and TMEM119. In order to corroborate our results obtained by immunohistochemistry (IHC) qPCR analyses were also conducted.
**Results:** The present study provides evidence for the altered interneuronal composition of caudate nucleus in schizophrenia without signs of microglial activation. There were small, medium and large CR-immunopositive (CR-ip) interneurons detected in the caudate nucleus. There was a 32% decrease in the density of all CR-ip interneurons ($p=0.020$, statistical power=0.747) that was driven by the loss of the small CR-ip interneurons ($p=0.017$, statistical power=0.777) while the densities of the medium and large CR-ip and NPY-ip interneurons were not significantly altered ($p=0.078$, $p=0.436$, $p=0.125$, respectively). Our experiments were also extended to the dorsolateral prefrontal cortex (medial frontal gyrus and superior frontal gyrus) where no significant changes were seen by IHC. However, qPCR analyses revealed a trend of decreased CR mRNA levels in schizophrenia ($p=0.061$, statistical power=0.485) while returned no significant changes regarding mRNA levels of NPY, Iba1 and TMEM119. No significant interactions between variables were seen controlling for PMI, age and gender by univariate, multifactorial ANOVA.
**Discussion:** We have discovered one of the most striking examples of altered neuronal densities in the forebrain in schizophrenia; a highly significant decrease in CR-ip neuronal density in the caudate nucleus. Future studies are warranted to elucidate neuronal inputs to CR-ip neurons in the caudate nucleus and whether they innervate local interneurons or medium spiny neurons. If they primarily regulate local interneurons they may disinhibit medium spiny neurons. If they directly innervate medium spiny neurons they may inhibit the principal cells of the striatum. It will also be important to determine if the large, medium and small CR-ip neurons have different connectivity and thus segregate function. Interestingly our results regarding the decreased density of CR-ip interneurons are in line with our previous observations in ASD[1] that underline the possible shared pathomechanisms between schizophrenia and ASD.

**References:**
1. Adorjan I, Ahmed B, Feher V, Torso M, Krug K, Esiri M, Chance SA, Szele FG. 2017. Calretinin interneuron density in the caudate is lower in autism spectrum disorder. Brain 140:2028–2040.
2. Brisch R et al. 2015. Calretinin and parvalbumin in schizophrenia and affective disorders: a mini-review, a perspective on the evolutionary role of calretinin in schizophrenia, and a preliminary post-mortem study of calretinin in the septal nuclei. Frontiers in Cellular Neuroscience 9:393.

### T197. A DRD2 CO-EXPRESSION GENE SET ENRICHED FOR SCHIZOPHRENIA RISK GENES IS CHARACTERIZED BY A COMMON TRANSCRIPTIONAL REGULATION INVOLVING NURR1 TRANSCRIPTION FACTOR

Silvia Torretta*[,1], Antonio Rampino[1], Giulio Pergola[1], Maria Pennuto[2], Manuela Basso[3], Pasquale Di Carlo[1], Rita Masellis[1], Giuseppe Blasi[1], Alessandro Bertolino[1]
*[1]University of Bari "Aldo Moro"; [2]University of Trento, University of Padova; [3] University of Trento*

**Background:** Genome-wide association studies demonstrated that multiple genetic variants are associated with schizophrenia (SCZ). Additional evidence revealed that genes are prone to operate in functional molecular networks that subtend complex clinical phenotypes. This knowledge raises the need to investigate how genes linked to SCZ and their possible co-regulators are inserted into molecular networks with a key impact in disease pathogenesis. Using post-mortem brain mRNA data sets (Pergola et al. 2017), we have previously identified a co-expression network enriched for SCZ risk genes, including DRD2, the gene coding for the D2 dopamine receptor, and predicted cognitive and neuroimaging phenotypes of SCZ, as well as response to antipsychotic treatment. Given the relevance of DRD2 to the pathophysiology of SCZ, in the current study we sought to further our understanding of biological mechanisms underpinning co-expression of the DRD2 network. In detail, we aimed at probing the hypothesis that expression of genes within the DRD2-related co-expression network is modulated by a common transcriptional regulation involving one or more Transcription factors (TFs).
**Methods:** In order to identify TF binding sites (TFBSs) in the promoter region of the 85 genes belonging to the DRD2 co-expression network, we performed a motif enrichment analysis using Pscan and Genomatix MatInspector tools. Biological validation experiments were performed in primary mouse cortical neurons. By real-time PCR analysis we measured the mRNA transcript levels of a group of genes included in the DRD2 co-expression module in basal conditions and upon viral vector-mediated overexpression (OE) and knockdown (KD) of the predicted TFs. We studied expression of genes linked to either DRD2 in the co-expression gene set, such as Gpld1, Chit1, Btg4 and Osr1, or SCZ risk, such as Gatad2a and Slc28a1. We also analyzed transcript levels of Cnr1, which mediates cannabinoid-induced transmission and is relevant to SCZ. Moreover, we analyzed the transcript levels of D2 long splicing isoform (D2L), which was included in the co-expression network, and D2 short splicing isoform (D2S), to verify whether TF regulation was specific for D2L.
**Results:** Promoters of the DRD2 co-expression gene set were enriched for two TFBSs, recognized by Nur-Related Factor 1 (NURR1, FDR-adjusted p=0.03) and Estrogen-Related Receptor Alpha (ERR1, FDR-adjusted p=0.02), respectively. Validation experiments in mouse primary cortical neurons established that NURR1, and not ERR1, is a regulator of genes of the DRD2 co-expression module analyzed in this study. In detail, Nurr1 gain of function (OE) decreased Cnr1 transcript levels (p=0.0002), whereas it increased Gpld1 transcript levels (p=0.03). The transcript levels of these genes showed an opposite expression trend upon Nurr1 KD. D2L (p=0.008), but not D2S, Gatad2a (p=0.00001), Slc28a1 (p=0.0005) and Chit1 (p=0.02) showed significant expression profile changes only upon Nurr1 OE and vice versa.
**Discussion:** NURR1 participates in developmental, differentiation and survival processes of dopaminergic neurons. It is implicated in transcriptional modulation of genes involved in dopaminergic transmission, including DRD2, as well as in behavioral phenotypes related to dopaminergic anomalies and reminiscent of SCZ in animal models. Finally, NURR1 genetic variation has been associated with SCZ. Taken together, our results are consistent with previous literature and with the hypothesis that molecular mechanisms responsible for co-expression in DRD2 network involve transcriptional regulation by NURR1. They also suggest reliability of our DRD2 co-expression network, and add new insights on mechanisms linked to DRD2-related molecular ensembles and to SCZ.

### T198. A SCHIZOPHRENIA-LIKE BIRTH SEASONALITY AMONG MATHEMATICIANS AND AN OPPOSITE SEASONALITY AMONG BIOLOGISTS: MORE EVIDENCE IMPLICATING BIMODAL RHYTHMS OF GENERAL BIRTHS

Giovanni Marzullo*[,1]
*[1]Per Aspera Research Foundation*

**Background:** Based on early-20th century births, a pre-electric illumination time of comparatively normal human exposure to sunlight, studies of schizophrenia (SCZ) found a birth seasonality with two opposite effects: a SCZ-liability peak among subjects born around late-February and an equally significant SCZ-resistance peak among those born six months later, around late-August. We previously investigated this rhythm in connection with a sunlight-dependent bimodal rhythm of general births that, prior to the full advent of electric lighting (but not later), occurred ubiquitously in non-equatorial parts of the world. We found that the SCZ-liability peak coincided with a first, Feb-Mar peak of general-population births (the GP1) while the SCZ-resistance peak coincided with a second, Aug-Sep peak of those births (the GP2). Moreover, in a study of hand and visual-field preferences among professional baseball players, we found the SCZ-liability, GP1-coincident seasonality among players with preferences denoting cerebral asymmetry "deficits" (CADs) and the SCZ-resistance, GP2-coincident seasonality among those with preferences denoting cerebral asymmetry "excesses." Also, in a study suggested by associations of CADs with artistic abilities, we found the SCZ-liability, GP1-coincident seasonality among groups representing visual, performing and literary art "creators" (VPL-Artists) and the SCZ-resistance, GP2-coincident seasonality among groups representing art critics, historians, curators and other art "observers" (Para-Artists). Together, these findings suggested, as one possibility (but see later), that the SCZ-liability, CAD effects and artistic abilities could all three represent traits genetically or otherwise selected into the GP1 excess population of newborns and out of the GP2 population. The present study of "scientists" was initially aimed at the purported arts/science antithesis.
**Methods:** Birth seasonalities were examined among early-20th century born American scientists and among yet earlier European biologists and mathematicians.
**Results:** A group representing 1,925 American scientists showed the SCZ-resistance, GP2-coincident seasonality. However, this effect proved to be mostly due to biologists because biochemists, chemists, and physicists showed gradually less seasonality while mathematicians suggested an altogether artist-like, GP1-coincident seasonality. This intimation of a biologist-mathematician antithesis was pursued with an investigation of most major figures in the history of the two sciences from the 15th to the early-20th century. The two groups, numbering 576 mathematicians and

787 biologists, shared the same mean decade of birth, the 1780s, and essentially the same geographic origin in Western Europe. The mathematicians showed a very significant SCZ liability-like, GP1-coincident seasonality while the biologists showed an even more significant SCZ resistance-like, GP2-coincident seasonality. The latter effect was particularly strong among naturalists, anatomists and other groups representing biological "observationalism" as opposed to "experimentalism."
**Discussion:** The findings are discussed in light of a) new evidence that the annual photoperiod is indeed alone responsible for both peaks of general births, with the GP1 and the GP2 being caused by maternal periconceptional exposure to, respectively, the summer-solstice sunlight maximum and the winter-solstice minimum, and b) an approach/withdrawal theory of lateralization of basic emotions where the left cerebral cortex would handle external stimuli eliciting complacent emotions towards external realities while the right cortex would handle internal stimuli eliciting disdain for those realities.

### T199. DEVIANT CORTICAL SULCATION RELATED TO SCHIZOPHRENIA, BUT NOT COGNITIVE DEFICITS, LIKELY PREDATE BRAIN DEVELOPMENT IN THE SECOND TRIMESTER

Lena Palaniyappan*,[1]
*[1]University of Western Ontario*

**Background:** Gestational disruptions are linked to the risk of schizophrenia; but in most cases, there is a lack of a clear history or observable anomaly indicating that the disruptions are likely to be subtle (Murray et al., 2017). The time-locked development of cortical sulci in a human embryo is highly sensitive to developmental disruptions (Chi et al., 1977). We can retrospectively infer the likely timing of embryonic/fetal disruption in schizophrenia by studying the structure of major cortical sulci that represent lobar development in adults with schizophrenia.
**Methods:** Anatomical T1 MRI scans from a publicly available dataset (COBRE) of 68 patients with schizophrenia and 72 controls were used to evaluate the sulcal depth. 5 major primary sulci that are invariable, representing lobar development (calcarine sulcus, superior temporal sulcus, superior frontal sulcus, interparietal sulcus and inferior frontal sulcus) with formation representing distinct developmental periods (16, 23, 25, 26 and 28 weeks respectively (Chi et al., 1977)) were chosen. Sulcal depth was measured using Morphologist interface of BrainVISA 4.5 (http://brainvisa.info/). Following the construction of 3-dimensional models of cortical folds, various sulci were automatically classified using a probabilistic algorithm with maximum depth computed for each identified sulcus. The 5 sulci were consistently labeled automatically across all subjects. The identified sulci were visually inspected to ensure that the boundaries are in accordance with Ono's Atlas of Cerebral Sulci (Ono et al., 1990).
**Results:** A repeated measure ANOVA with 5 sulci and 2 hemispheres as within-subject factors and gender, age and intracranial volume as covariates revealed a significant between-subjects effect for diagnosis ($F[1,134]=14.8$, $p=0.0002$). Gender ($F[1,134]=7.4$, $p=0.007$) and age ($F[1,134]=4.5$, $p=0.035$) also had significant effect in the model. Parameter estimates revealed a significant effect of diagnosis (Controls>Patients) for left superior temporal ($t=3.2$, $p=0.002$), right superior temporal ($t=2.8$, $p=0.006$), right inferior frontal ($t=2.7$, $p=0.007$) and left calcarine ($t=2.2$, $p=0.03$) sulci. 5 non-collinear factors representing the 5 bilateral sulci were obtained using varimax rotation, and related to overall MATRICS standardized composite score in patients using multiple regression. The depth of the superior frontal sulcus was the only predictor of the variation in the cognitive score ($F[1,54]=8.7$, $p=0.005$).
**Discussion:** The above findings suggest that the gestational cortical disruption underlying schizophrenia is likely to predate, if not, coincide with the appearance of calcarine sulcus (i.e. 16 weeks, early second trimester) and affects frontal, temporal and occipital lobes. Nevertheless, the burden of cognitive deficits may relate specifically to aberrant superior frontal development occurring in late second trimester.

### T200. DISTINCT ASSOCIATIONS OF MOTOR DOMAINS WITH THE GENETIC RISK FOR PSYCHOSIS – DIFFERENT PATHWAYS TO MOTOR ABNORMALITIES IN SCHIZOPHRENIA?

Lea Schäppi*,[1], Katharina Stegmayer[1], Petra Viher[1], Sebastian Walther[1]
*[1]University Hospital of Psychiatry*

**Background:** Aberrant motor function is an integral part of Schizophrenia. In fact, abnormalities are frequently found in patients, in populations at risk, and in unaffected relatives. Motor abnormalities are suspected to be relevant for the clinical outcome and could probably predict the conversion from at-risk individuals to schizophrenia. Furthermore, motor function and has been argued as endophenotype of the disorder. Yet, which particular motor domain may classify as a potential endophenotype is unknown. We aimed to compare schizophrenia patients, unaffected first degree relatives and healthy controls for different motor domains. We expected impairments in all domains in patients and in some domains in relatives.
**Methods:** We included 43 schizophrenia patients, 34 unaffected first degree relatives of schizophrenia patients and 29 healthy control subjects, matched for age, gender and education level. We compared motor function of five domains between the groups. The domains comprise neurological soft sings (NSS), abnormal involuntary movements (dyskinesia), Parkinsonism, complex fine motor function applying the coin rotation task as well as finger tapping. Furthermore, we tested the association of motor function of the five domains with working memory, frontal lobe function and nonverbal intelligence for each group separately using within-group bivariate correlations.
**Results:** Schizophrenia patients showed poorer motor function in all tested domains compared to healthy controls. First-degree relatives had intermediate ratings with aberrant function in two motor domains. In detail, relatives had significantly more NSS and performed poorer in the finger tapping task than controls. In contrast, in relatives complex fine motor function was intact. Relatives did not differ from controls in dyskinesia or Parkinsonism severity.
**Discussion:** Taken together, schizophrenia patients have motor abnormalities in all tested domains. Thus, motor abnormalities are a key element of the disorder. Likewise, first degree relatives presented motor deficits in two domains. A clear difference between relatives and healthy controls was found for NSS and finger tapping. Thus, NSS and finger tapping may be a potential marker of vulnerability for schizophrenia. The lack of association between genetic risk and dyskinesia or Parkinsonism suggests distinct pathobiological mechanisms in the various motor abnormalities in schizophrenia.

### T201. THE STUDY OF WHITE MATTER MATURATION IN THREE POPULATIONS OF GENETIC HIGH RISK FOR SCHIZOPHRENIA INDIVIDUALS SPANNING THE DEVELOPMENTAL TIMELINE

Amanda Lyall*,[1], Nathaniel Somes[1], Fan Zhang[1], James Robertson[1], Lauren J. O'Donnell[1], Yogesh Rathi[1], Ofer Pasternak[1], Peter Savadjiev[1], Martin Styner[2], Zachary Fitzgerald[1], Raquelle Mesholam-Gately[3], Heidi Thermenos[4], Susan Whitfield-Gabrieli[5], Matcheri Keshavan[3], Lynn DeLisi[6], John Gilmore[2], Larry J. Seidman[3], Marek Kubicki[1]
*[1]Brigham and Women's Hospital, Harvard Medical School; [2]University of North Carolina; [3]Beth Israel Deaconess Medical*

*Center, Harvard Medical School [4]Massachusetts Mental Health Care Center, Harvard Medical School; [5]Massachusetts Institute of Technology; [6]VA Boston Healthcare System,*

**Background:** While the etiology of schizophrenia (SZ) is still unclear, it has been characterized as a neurodevelopmental disorder because patients exhibit deviations from normal maturational trajectories that are evident prior to the onset of psychotic symptoms. White matter (WM) has been purported to play a central role in the development of SZ, however, the timing and nature of WM changes in SZ is still poorly understood. This study uses diffusion imaging from three independent Genetic High Risk (GHR) populations spanning the developmental timeline from infancy to young adulthood. The aim of this study is to understand the extent and the time-course of WM maturational pathologies as a function of age and genetic risk for psychosis.
**Methods:** Two datasets of 3T diffusion-weighted images of children aged 7 to 12 (24 HC and 16 at GHR) and young adults aged 19 to 29 (26 HC and 43 GHR) were collected at the Massachusetts Institute of Technology. The third dataset of 3T images of infants aged 2 years (35 HC and 18 GHR) was collected at the University of North Carolina – Chapel Hill. Whole brain two-tensor tractography was performed and 4 bilateral WM tracts (arcuate fasciculus (AF); inferior longitudinal fasciculus (ILF); cingulum bundle (CB); superior longitudinal fasciculus-ii (SLF-ii)), were extracted utilizing an atlas-guided fiber clustering algorithm. The fractional anisotropy of the tissue (FA-t) was obtained. We carried out group comparisons of FA-t between GHR and HCs utilizing Mann-Whitney-U tests and Cohen's d effect sizes for each WM tract.
**Results:** Preliminary analyses reveal significant reductions in FAt between GHR and HC in the right CB (p = 0.013) in the child GHR population. This is mirrored by medium to large effect sizes in the bilateral CB in GHR children (CB-left, d = 0.51; CB-right, d = 0.79). Reductions in FAt in the adult GHR population within the right CB was the largest effect observed in the adult analysis (CB-right, d = 0.46). Effect sizes in the bilateral CB were minimal in the infant GHR population (CB-left, d = 0.14, CB-right, d = 0.11). Significant decreases were also seen in the right SLF-ii in the adult GHR population (p = 0.012), but not in the infant or child GHR populations, though the reductions in FAt in the child GHR population exhibited a small effect (d = 0.35). All other white matter tracts in the adult analysis showed minor effects ranging from d = 0.033 (ILF-right) to 0.28 (ILF-left). The children and infant population also exhibited small effect sizes for all other tracts, with the child GHR dataset ranging from 0.036 (ILF-left) to 0.41 (ILF-right) and the infant GHR dataset ranging from d = 0.038 (SLF-left) to 0.34 (ILF-left).
**Discussion:** Our preliminary results suggest that abnormal WM maturation may occur in the right CB and right SLF-ii in individuals with increased genetic risk for SZ, specifically after early childhood (7 to 12 years) and into adulthood (19 to 29 years). The CB and SLF-ii are highly implicated in working memory performance, an ability that retrospective studies have shown begins to decline during the peripubertal period in those that develop SZ (~7 to 9 years). The lack of structural findings in GHR infants, may suggest that WM alterations are more likely to arise later in development, thereby possibly identifying childhood as a vulnerable period. Taken together, the preliminary results of this study provide possible evidence of subtle divergences from a healthy WM maturational trajectory in the right CB and right SLF-ii in early to late childhood that may persist into adulthood and these deviations may contribute to cognitive phenotypes described in other studies.

### T202. HUMOR-SKILLS TRAINING IN PATIENTS WITH SCHIZOPHRENIA: EFFECTS ON SYMPTOMS AND SOCIAL FUNCTIONING

Irina Falkenberg*[,1], Florian Bitsch[2], Philipp Berger[1], Arne Nagels[1], Benjamin Straube[1]
*[1]Philipps-University*

**Background:** Humor can provide a method of coping with a variety of stressful situations. Training of humor-related skills has proven effective in clinical samples, although humor training in patients with schizophrenia is relatively rare.
**Methods:** In the present study, patients with schizophrenia have been randomly assigned to either a training of humor abilities or a training of social skills. Training effects on measures of psychopathology, psychosocial functioning and stress were compared between groups.
**Results:** Preliminary analyses revealed that level of negative symptoms, stress and psychosocial dysfunction were significantly reduced in the humor group over the course of the training.
**Discussion:** These results suggest that humor training may improve important clinical and functional outcomes in patients with schizophrenia.

### T203. ILLICIT DRUGS USE AND ULTRA-HIGH RISK (UHR) FOR PSYCHOSIS STATUS IN A LATIN-AMERICAN SAMPLE

Mauricio Serpa*[,1], Alexandre Andrade Loch[1], Camille Chianca[1], Elder Freitas[1], Julio Cesar Andrade[1], Tania Maria Alves[1], Lucas Hortêncio[1], Martinus Theodorus van de Bilt[1], Wagner Gattaz[1], Wulf Rossler[1]
*[1]University of Sao Paulo*

**Background:** In recent years, a number of investigations have evaluated the effect of cannabis use on the risk of presenting ultra-high risk for psychosis (UHR) status as well as its influences on transition rate, suggesting a dose-dependent interaction. On the other hand, the association between cocaine (snorted or smoked) - an increasing health issue in several countries worldwide - and the UHR state was not appropriately examined. Also, exposure to other psychotomimetic drugs, as amphetamines and lysergic acid diethylamide (LSD), has not been investigated yet. We sought to examine differences in the prevalence of drug use between UHR subjects and epidemiologic controls (EC).
**Methods:** Over 2500 individuals from the city of São Paulo (Brazil), aged between 18 and 30 years old, were screened with the Prodromal Questionnaire. Subjects with scores higher than 18 points in the positive subscale were invited to be thoroughly assessed with the application of SIPS (Structured Interview for Psychosis-Risk Syndromes). Drug use (lifetime use, age of first use and more intense use) was assessed using South Westminster scale.
**Results:** 100 individuals presented UHR state; other 110 were enrolled as EC. A subsample of 50 UHR subjects and 82 HC with data on drugs consumption were evaluated herein. UHR subjects history of lifetime drug use was: 19 (38%) cannabis; 5 (10%) snorted cocaine; 1 (2%) crack; 1 (2%) amphetamine; 2 (6.9%) LSD. EC history of lifetime drug use was: 20 (24.4%) cannabis; 6 (7.3%) snorted cocaine; 0 crack; 2 (2.4%) amphetamine; 1 (1.2%) LSD. No differences were observed for snorted cocaine (p=0.589), crack (p=0.379), amphetamine (p=1.0), or LSD (P=0.167). At a trend level, cannabis lifetime use (p=0.096) was more prevalent in the UHR group. Additional analyses showed that UHR subjects initiate cannabis use at earlier age than EC (p=0.006). In this group, 20% of subjects had used cannabis prior to 15 years of age, in comparison to 3.6% in the EC group.
**Discussion:** Our results reinforce the view that cannabis use is linked to psychosis risk and that subjects at early age of exposure are at greatest risk. Nonetheless, studies with larger number of participants are warranted to confirm our findings, particularly on the lack of association between less frequently consumed drugs and the UHR for psychosis state.

### T204. NOVEL VIRTUAL REALITY SOCIAL SKILLS TRAINING FOR INDIVIDUALS WITH SCHIZOPHRENIA

Lénie Torregrossa*[,1], Laura Hieber Adery[1], Megan Ichinose[1], Heathman Nichols[1], Alena Gizdić[1], Joshua Wade[1], Dayi BIan[1], Eric Granholm[2], Nilanjan Sarkar[1], Sohee Park[1]
*[1]Vanderbilt University; [2]University of California, San Diego*

**Background:** Social impairment is a core feature of schizophrenia presenting a major barrier to recovery. Although antipsychotic medications can reduce psychotic symptoms, social functioning often remains poor, contributing to the financial burden of schizophrenia. Validated behavioral interventions, such as Social Skills Training (Liberman & Martin, 1988), target a broad range of social domains by practicing pragmatic living skills. But they yield only modest effect sizes for social outcome (Pfammatter et al, 2006). Conventional social interventions present further limitations including: time and effort required from patients and therapists, low adherence, lack of personalization, and low generalizability. Importantly, most people with mental illness do not currently have access to social interventions. The aim of this study was to design and implement an effective, high-compliance virtual reality (VR) social skills training game for people with schizophrenia.
**Methods:** The advantages of the VR environment include flexibility, controllability, extensive repertoire of stimuli, low-burden, low-cost and safety (Strickland, 1997). The goal of the training game was to support social attention to improve social skills learning. We trained social skills by exercising problem-solving in naturalistic scenarios: the grocery store, a bus stop, and a cafeteria. Subjects moved through variable steps in a social "mission" to obtain personal information through conversations with avatars. Each mission began with the participant fixating on the avatar. Subjects then had to decide which avatar to approach and choose an appropriate response to the avatar's prompts. If they chose an incorrect response, oral feedback was provided on why this response was not the most effective, and instructed them to try again. This occurred until the participant identified the most appropriate response, thus completing the mission and getting access to the next level of difficulty. Each training session concluded after completion of 12 total conversation missions.
Eighteen individuals with schizophrenia (SZ) and twenty demographically matched controls (CO) participated in this study. At baseline, SZ and CO completed pre-training assessments. The CO group did not undergo VR training but participated in behavioral assessments so that we could compare SZ performance to normative data. SZ participated in the VR training twice a week for 5 weeks (10 sessions). After the 10th session, we re-examined social functioning, cognitive functions, and symptoms. SZ also completed a satisfaction survey upon training completion.
**Results:** Of the eighteen SZ participants enrolled in the study, sixteen completed the 10 sessions of training, yielding a retention rate of 89%. 80% of SZ subjects reported being "extremely satisfied" with the training. None reported not being satisfied. 93.3% rated the training as "acceptable" and the effort required to attend the study as "easy." Regarding outcome, negative symptoms significantly decreased from pre-training to post-training. Performance on BLERT and CogState Social Emotional Cognitive Task significantly improved. The average time taken to complete a mission was significantly lower in the last session compared to the first, showing that participants became increasingly better at efficiently solving these social missions.
**Discussion:** These results show evidence for VR training as an acceptable and feasible intervention improving social functioning in SZ. Future work will test the adaptive social VR training against an active control condition in a pilot randomized controlled trial to evaluate the relative efficacy of the VR training on enhancing social attention and associated neural circuitry.

### T205. CHANGES IN SOCIAL ATTENTION AND EMOTION RECOGNITION FOLLOWING A PILOT SOCIAL SIMULATION COMPUTER GAME INTERVENTION FOR INDIVIDUALS WITH SCHIZOPHRENIA

Megan Ichinose*[,1], Joshua Wade[1], Laura Hieber Adery[1], Lénie Torregrossa[1], Heathman Nichols[1], Dayi BIan[1], Alena Gizdic[1], Nilanjan Sarkar[1], Sohee Park[1]
*[1]Vanderbilt University*

**Background:** Individuals with schizophrenia (SZ) exhibit significant difficulties processing and perceiving socioemotional information conveyed by others. Increasing evidence suggests that SZ deficits in facial emotion recognition, in particular, contribute to impaired daily social functioning. Studies show that improving SZ patients' visual attention to socially-relevant facial areas (eyes, nose, mouth) with targeted computer interventions will ameliorate deficits in emotion recognition. We tested whether 10 sessions of a novel, VR-based social simulation computer game would indirectly improve facial emotion recognition in SZ, and additionally whether potential gains were associated with changes in gaze patterns.
**Methods:** Fifteen SZ outpatients completed a social simulation computer game intervention involving a pre-training visit, 10 training sessions scheduled approximately twice per week (days until completion: M=38.8, SD=16), and a post-training visit. During training sessions, participants played a novel, adaptive VR-based computer game that involved approaching and engaging in conversations with various Avatar game characters across several naturalistic settings (a bus stop, café, grocery store). Each game session required completion of 12 "social missions" to determine information about different characters (e.g., food preference), achieved by selecting the appropriate conversational prompts and follow-up questions from multiple options. At pre- and post-training visits, emotion recognition was assessed with a novel dynamic facial affect recognition task (DFAR) and the Bell Lysaker Emotion Recognition Task (BLERT). During the DFAR, participants viewed adult Avatar characters (50% female) making one of 8 dynamic facial expressions (anger, sadness, fear, disgust, joy, surprise, contempt) while gaze data and behavioral responses were recorded. The VR-based computer game and DFAR were developed in-house with Autodesk Maya 3D animation and Unity software (unity3d.com).
**Results:** Patients' emotion recognition accuracy (BLERT) significantly improved from pre- to post-training. Patients' accuracy on the facial affect recognition task (DFAR) also significantly improved following training for specific negative emotions (anger, contempt, fear, and sadness). Regarding changes in visual attention, patients made overall fewer fixations at post-training (fixation duration threshold = 200ms) across relevant social areas (eyes, nose, mouth) when viewing emotional Avatar faces compared to pre-training. A general reduction in fixations was not accompanied by an increase in mean fixation duration. Rather, shorter fixation durations were positively associated with DFAR performance accuracy.
**Discussion:** SZ patients' participation in a novel, VR-based computerized social simulation training may yield indirect benefits in emotion recognition. Specifically, patients exhibited improvements on a validated assessment of emotion perception (BLERT) following the 10-session computer training. A decrease in the number of fixations on socially-informative facial regions during Avatars' emotion expression on the DFAR may indicate an increased efficiency in scanning for socioemotional information. Though much work remains in probing the exact nature of treatment mechanism and durability of these improvements, these promising initial results demonstrate the potential of an VR-based computer game for improving core deficits in social cognition.

### T206. DOES AGE INFLUENCE RESPONSE TO COGNITIVE REMEDIATION?

Benedetta Seccomandi*[,1], Deborah Agbedjro[1], Morris Bell[2], Richard Keefe[3], Matcheri Keshavan[4], Silvana Galderisi[5], Alice Medalia[6], Joanna Fiszdon[7], Mario Maj[5], Armida Mucci[5], Roberto Cavallato[8], Til Wykes[1], Matteo Cella[2]
*[1]King's College London; [2]Yale University School of Medicine; [3]Duke University Medical Center; [4]Harvard University; [5]University of Naples SUN; [6]Columbia University; [7]VA Connecticut Health, Yale University; [8]University Vita-Salute San Raffaele.*

**Background:** Cognitive deficits are common in people with schizophrenia and have a negative impact on functioning. Cognitive Remediation (CR) is an effective approach to reduce the burden of cognitive difficulties however there are individual differences in therapy response. Previous research suggests that participants age may be a significant moderator of therapy efficacy but results are inconclusive. This study attempts to fill this gap by exploring how age may influence CR outcomes.
**Methods:** Data from ten trials from the NIMH Database of Cognitive Training and Remediation Studies (DoCTRS) were used. We considered the following therapy outcomes: Executive function as assessed by the Trail making test part B (TMTB), the Wisconsin Card Sorting Test (WCST) and Verbal fluency (FAS) scores. Working memory was assessed with the Letter-Number Span (LNS) and the Digit span. Symptoms were evaluated with the Positive, Negative and General scores from the Positive and Negative Syndrome Scale (PANSS). Functioning was assessed using the Heinrichs-Carpenter quality of life (HCQOL) scale. To evaluate the effect of age on outcomes we classified participants into under 40 and over 40 years old. We compared outcomes across age groups using mixed linear models.
**Results:** We considered data from 711 people with schizophrenia (407 received CR and 304 the control condition). For the under 40 group the average age was 29.26 (SD 6.83) while the average yeas spent in education was 12.11 (2.61). The over 40 group had a mean age of 40.09 (SD 6.09) and 12.11 (2.54) years of education.
We found a significant interaction between age and working memory and functioning improvement for the over 40 group. The younger group showed a larger effect of CR in term of general symptoms reduction. We did not find an effect of age on executive function, positive and negative symptom.
**Discussion:** The results indicate that CR may benefit people with schizophrenia in different way depending on their age. Age may represent a large number of complex factors and more work is needed in this area to better understand how individual characteristics and illness history may influence CR response. Work in this sense will help to reduce CR response heterogeneity and improve therapy personalisation.

### T207. A REVIEW OF PREDICTORS OF RESPONSIVENESS TO CBT FOR PSYCHOSIS

Ana Elisa Farias de Sousa*[,1], Martin Lepage[1], Mathieu Brodeur[1]
[1]*McGill University*

**Background:** Pharmacological and psychological intervention combined are proved to be more effective for treating psychosis than pharmacological treatment alone. Cognitive Behavioral Therapy for psychosis (CBTp) has been empirically supported as conjoint treatment providing a significant improvement in positive and negatives symptoms, and functional outcomes for psychosis. However, rates of patient discontinuation in CBTp and occasional lack of improvement in symptoms show it is important to refine the identification of the individual characteristics related to better response to CBTp. This literature review aims to accomplish a comprehensive analysis of the evidence-based studies that have searched predictors in the last decades, focusing on individual factors that directly predicts responsiveness to CBTp, rather than therapist or treatment factors. The scope of knowledge gathered here intends to guide practical application of CBTp to people with psychosis that can benefit more from this intervention. Adaptations to improve the effectiveness of CBTp and gaps to be addressed in further research are also considered.
**Methods:** Thirty (30) studies (18 RCT) were included to determine which characteristics are relevant for a distinctive response to CBTp in people with schizophrenia and other psychotic disorders. The word "predictor" was used to discriminate pertinent studies. Articles were included if they reported in a population within a Psychosis Spectrum Disorder; reported on CBT or derived intervention; reported on individual predictors of outcome in CBT or derived therapy. Articles that reported on a high-risk psychosis population or on comorbidities with psychosis; reported non-individual predictors; were case studies or literature reviews; had a small sample; and had mixed interventions and did not report results specific to CBT were not included.
**Results:** Studies have shown divergences in methodology, focus on different domains and time-points of disorders outcome and great heterogeneity in results. There is strong evidence that greater clinical and cognitive insight, cognitive flexibility, greater positive symptom severity and less pronounced negative symptoms at baseline, shorter duration of psychosis, a greater number of hospitalization in the previous five years and pre-therapy coping styles can predict better outcome in CBTp, although their significance has varied between studies. While impairment in verbal memory was related to a shortage of improvement in symptoms and a greater likelihood to abandon of treatment before completion, most studies did not find neurocognitive functioning to be a predictor of outcome in CBTp.
**Discussion:** Further investigation is needed to determine the extent and validity of these predictors in different populations within the scope of psychosis. Professionals can benefit from the gathered knowledge, using these findings to better target CBTp and to focus early stages of intervention on developing patient's abilities such as cognitive flexibility and insight, working memory, coping skills and clinical awareness in order to improve their receptiveness to therapy and successful outcome.
Future research should aim to replicate findings with larger and more diagnosis-comprehensive samples to enable generalization of the present results. Aspects such as personality traits, metacognition and sociodemographic characteristics require more thorough investigation to confirm their predictive value before being taken into consideration when selecting patient suitability to CBTp and similar interventions.

### T208. LONGITUDINAL FEASIBILITY AND ACCEPTABILITY OF THE EXPRESS SMARTPHONE APP: RECRUITMENT, RETENTION AND PRELIMINARY FINDINGS

Emily Eisner*[,1], Sandra Bucci[1], Richard Emsley[1], Christine Barrowclough[1], Richard Drake[1]
[1]*University of Manchester*

**Background:** Relapse of schizophrenia is common, has profound, adverse consequences for patients and is costly to health services. Early signs interventions aim to use warning signs of deterioration to prevent full relapse. These interventions show promise but could be further developed. There is preliminary evidence that adding 'basic symptoms' to conventional early signs of relapse may improve relapse prediction. Basic symptoms are subtle, subclinical disturbances in one's experience of oneself and the world that can include, for example, perceptual changes, mild subjective cognitive problems and decreased tolerance of stressors. This study aimed to evaluate the feasibility and acceptability of using the ExPRESS smartphone app to monitor both conventional early signs and basic symptoms as possible predictors of relapse.
**Methods:** Patients who had experienced a relapse of schizophrenia within the past year took part in a screening interview. Those with at least one basic symptom emerging prior to a previous relapse were eligible for the longitudinal feasibility study. Consenting participants were asked to use the ExPRESS smartphone app once a week for 6 months, answering questions on their experience of conventional early signs, basic symptoms and psychotic symptoms. When app responses indicated an increase in psychotic symptoms above a pre-defined threshold, the researcher conducted the PANSS positive symptoms interview over the phone to assess whether the symptom increase was indicative of relapse. At the end of the follow-up period, face-to-face qualitative interviews were conducted to explore participants' experiences of using the phone app and reasons for study dropout.
**Results:** 82% (18/22) of those screened were eligible for the longitudinal feasibility study and consented to participate. Of these, 72% (13/18) completed at least half of the weekly phone app assessments, with two participants dropping out of the study without completing any assessments on the phone app. Two participants met pre-defined relapse criteria during the 6 month follow up period. Initial findings from sixteen qualitative interviews are discussed, including interviews with the two participants who met relapse criteria and two study drop-outs.

**Discussion:** The rate of recruitment to the longitudinal study was much higher than expected, since a higher than expected proportion of screened participants had experienced basic symptoms prior to a previous relapse. Retention rates were as expected, suggesting that the ExPRESS app is acceptable to patients with psychosis. Qualitative feedback from participants supports this conclusion. The results from this longitudinal feasibility study will inform the design of a well-powered definitive study prospectively examining the sensitivity and specificity of basic symptoms in predicting relapses of schizophrenia.

### T209. TESTING CORTICAL RTMS TARGETS TO IMPROVE PSYCHOMOTOR SLOWING IN SCHIZOPHRENIA AND MAJOR DEPRESSION IN A RANDOMIZED, DOUBLE-BLIND, PLACEBO-CONTROLLED TRIAL

Sebastian Walther*,[1], Lea Schäppi[1], Danai Alexaki[1], Katharina Stegmayer[1], Georgios Schoretsanitis[1]
*[1]University Hospital of Psychiatry*

**Background:** Psychomotor retardation is a frequent symptom of schizophrenia spectrum disorders and major depressive disorders, which hampers functional outcome. Neuroimaging studies have pointed to aberrant connectivity between cortical and subcortical components of the motor system in subjects with psychomotor retardation. Furthermore, increased neural activity was noted in premotor areas in subjects with severe motor inhibition. Interventional trials targeting aberrant brain function with noninvasive brain stimulation in this field are missing.
**Methods:** In a randomized, sham-controlled, double-blind clinical trial we test whether three different repetitive transcranial magnetic stimulation (rTMS) protocols may ameliorate psychomotor retardation after 15 daily sessions in patients with schizophrenia spectrum disorders and patients with major depressive disorder. Randomization is performed in parallel for both diagnoses. rTMS protocols include facilitatory stimulation (15 Hz) of left dorsolateral prefrontal cortex (DLPFC), facilitatory stimulation (iTBS) of the supplementary motor area (SMA), inhibitory stimulation (1 Hz) of the SMA, and sham stimulation of the occipital cortex. Assessments are performed at baseline and every five rTMS sessions. Motor retardation is assessed with wrist actigraphy and the Salpetriere Retardation Rating Scale (SRRS). The primary outcome variable is the proportion of responders per group, with SRRS score reduction of 30% from baseline. We apply the last observation carried forward method to the intention to treat population
**Results:** The ongoing study has enrolled 24 patients (17 SZ, 7 MDD), and 15 patients completed the study. The proportion of responders differs significantly between groups (X2 = 7.7 p = 0.05) in favor of the inhibitory SMA stimulation (83%). Repeated measures ANOVA of SRRS in all participants with LOCF indicated a significant effect of time (F = 9.6, p = 0.001), but no time x protocol interaction. However, the completer analysis indicated an effect of time (F = 14.4, p < 0.001) and a time x protocol interaction (F = 2.5, p = .05). Positive effects were also noted for fine motor performance and negative symptoms.
**Discussion:** Inhibitory stimulation of the SMA seems to improve psychomotor retardation in these preliminary analyses. The result fits to findings of increased neural activity in premotor areas during behavioral motor inhibition in schizophrenia and major depression. Given, the effect is stable over the whole planned study population, inhibitory rTMS would become an interesting treatment for psychomotor retardation in affective and nonaffective psychoses.

### T210. PSYCHOSOCIAL CORRELATES OF INTERPERSONAL PLEASURE IN SCHIZOPHRENIA-SPECTRUM PATIENTS

Diane Gooding*,[1], Yael Ratner[2], Nina Mendyk[2], Herman Farkash[2], Michael Ermiyev[2], Michael S. Ritsner[3]
*[1]University of Wisconsin-Madison; [2]Shaar Menashe Mental Health Center; [3]National Insurance Institute, Shaar Menashe Mental Health Center*

**Background:** Although many people with schizophrenia-spectrum disorders report high levels of social anhedonia, it is not clear what differentiates those patients who self-report social anhedonia from those who do not. Moreover, the extent to which the hedonic functioning of severely disordered patients is associated with their clinical symptoms or with personality-related factors remains unresolved.
**Methods:** We administered the Anticipatory and Consummatory Interpersonal Pleasure Scale (ACIPS; Gooding & Pflum, 2014), a self-report measure designed to assess hedonic capacity for social and interpersonal pleasure, to 125 consecutively admitted inpatients with schizophrenia-spectrum disorder. The (81 schizophrenia, 44 schizoaffective disordered) patients were assessed in terms of their illness and symptom severity. They were also administered measures of self-efficacy (GSES; Jerusalem & Schwarzer, 1992), quality of life (Q-LES-Q-18; Ritsner et al., 2005), and recovery level (RAS-20; Salzer, 2010). Based on total ACIPS scores, two cut-off points were defined in order to classify participants as ‘normally hedonic’, ‘hypohedonic’ or ‘anhedonic’.
**Results:** The ACIPS negatively correlated with 8 PANSS items: conceptual disorganization (P2, r=-0.24, p<0.01), hallucinatory behavior (P3, r=-0.28, p<0.01), suspiciousness (P6, r=-0.31, p<0.001), emotional withdrawal (N2, r=-0.24, p<0.01), stereotyped thinking (N7, r=-0.19, p<0.05), tension (G4, r=-0.23, p<0.01), G5 mannerism and posturing (G5, r=-0.22, p<0.05), and disturbance of volition (G13, r=-0.26, p<0.01).In addition, the ACIPS positively correlated with self-efficacy, self-esteem, perceived social support, subjective quality of life, and recovery scale scores.
**Discussion:** The ACIPS is a reliable and valid means to measure social anhedonia in a clinical sample. The findings revealed that the self-reported hedonic functioning of schizophrenia-spectrum patients is associated with both clinical symptomatology as well as some personality-related variables. Suggestions for further clinical and research applications using the ACIPS will be provided.

### T211. BASIC SELF-DISTURBANCE AS A PREDICTOR OF DETERIORATION IN ATTENUATED PSYCHOSIS: A 1-YEAR FOLLOW-UP STUDY AMONG COMMUNITY-DWELLING ADOLESCENTS

Dan Koren*,[1], Liza Lacoua[1], Lily Rothshield[1], Maya Rothbaum[1], Neta Cohen[1], Josef Parnas[2]
*[1]University of Haifa; [2]University of Copenhagen*

**Background:** Phenomenological research indicates that disturbance of the basic sense of self may be a core phenotypic marker of schizophrenia spectrum disorders. Basic self-disturbance (SD) refers to a disruption of the sense of first-person perspective and self-presence that is associated with a variety of anomalous subjective experiences. Recent studies including from our group provided first, preliminary support for the notion that SD is related to attenuated psychosis symptoms (APS) and depression among clinical (i.e., treatment-seeking) and non-clinical samples of non-psychotic adolescents. However, very few studies, if any at all, have looked at the ability of SD to predict change in APS and depression over time. The goal of this study was to address this lacuna in the literature by examining the unique and added contribution of SD to the prediction of change over time in APS and depression among community-dwelling adolescents.
**Methods:** The 1-year longitudinal relationship between SD and change in APS and depression were explored in a sample of 100 non-help-seeking adolescents (age 13–15) from the community. SD was assessed with the Examination of Anomalous Self-Experience (EASE), prodromal symptoms and syndromes were assessed with the Structured Interview for Prodromal

Syndromes (SIPS), present and lifetime diagnoses of schizophrenia-spectrum and depression disorders were assessed with the Kiddie Schedule for Affective Disorders and Schizophrenia (K-SADS), level of distress with the Mood and Anxiety States Questionnaire (MASQ), and psychosocial functioning with the with the Strength and Difficulties Questionnaire (SDQ).
**Results:** Seventy-seven (77%) adolescents out of the 100 that had been assessed at baseline were available and agreed to participate in the 1-year follow-up (Mean=1.4, S.D.=0.8). Except for a diagnosis of an affective disorder, which was slightly less prevalent among those who returned for the follow-up assessment, there were no significant differences between those who were available and those who lost for the follow-up assessment on any of the major socio-demographic or clinical variables at baseline. Consistent with our first hypothesis, SD at baseline predicted a significant amount of variance in APS change over time (R-squared=0.10, F= 8.61, p=0.004). However, inconsistent with our second hypothesis, SD at baseline did not have a significant added contribution to the prediction of APS change when APS at baseline was controlled for (R-squared difference=0.02, F=1.83, p=0.18).
**Discussion:** These results provide preliminary support for a prospective association between SD and deterioration in prodromal symptoms among adolescents from the community. However, they fail to support an added value of SD over and above baseline APS for the prediction of APS deterioration. Because SD was assessed only at baseline, they leave unanswered the degree to which change in SD is associated with a change in APS and depression.

### T212. THE INTRINSIC ORGANIZATION OF SYMPTOMS MARKS TRANSITION FROM HIGH-RISK STATE TO EARLY PSYCHOSIS: A PHENOMENOLOGICAL CONNECTIVITY STUDY

Lena Palaniyappan*[,1], Tushar Das[1], Fabienne Harrisberger[2], Undine E. Lang[2], Anita Riecher-Rössler[2], André Schmidt[2], Stefan Borgwardt[2]
[1]*University of Western Ontario;* [2]*University of Psychiatric Clinics Basel (UPK)*

**Background:** In psychiatric practice, when symptoms “come together” we call the resulting construct as a diagnosis. We believe that there is a disease process that binds together, enabling co-occurrence of varied symptoms. We use either diagnostic or syndromic labels to describe this construct (e.g. positive syndrome, negative syndrome, schizophrenia, at-risk mental state). An emerging idea, promoted by network theorists, is that symptoms may relate by their own intrinsic nature, with no external constructs bringing them together e.g. paranoia leads to social withdrawal, loss of appetite leads to loss of weight etc. This intrinsic organisation of symptom relationships can be studied using network models by applying graph theory to symptom data.
**Methods:** We recruited 63 subjects with at-risk mental state [on the basis of Melbourne PACE criteria] but no transition (ARMS-NT), 16 that later developed psychosis (ARMS-T) and 38 drug-naïve patients with first-episode psychosis (FEP) from Basel, Switzerland. Symptoms were measured using Brief Psychiatric Rating Scale. Clinical symptoms can be construed as a system of individual elements (24 nodes) and their relationship (24x23 possible edges) within a group. We estimate each individual’s contribution to the intrinsic organisation of symptoms using a jack-knife bias estimation procedure. Bias values for each pair of symptoms in an individual subject quantified the contribution of that subject to the overall within-group relationship for that symptom pair. Higher values meant greater relationship between the two given nodes in that subject, relative to the rest of the group. We then used Graph Analysis Toolbox, with a range of binarization thresholds based on cost-density of connectivity to extract adjacency matrices.
**Results:** None of the 24 individual symptoms of BPRS significantly differentiated ARMS-NT from ARMS-T, though a number of symptoms (suspiciousness, hallucinations, disorganisation, motor retardation, hostility and suicidality) showed a gradient of FEP>ARMS-T>ARMS-NT (F test, FDR corrected p<0.05). The small-worldness (F=4.8, p=0.01) and the clustering coefficient (F=10.9, p<0.001) and modularity (F=10.9, p<0.001) of the symptom networks were notably different among the 3 groups, with a gradient of FEP>ARMS-T>ARMS-NT (except for modularity where FEP=ARMS-T). Post-hoc tests revealed significantly high clustering (Hedges’s g = 0.60, p<0.05) and high modular organisation (Hedges’s g = 0.81, p<0.01) of symptoms in ARMS-T compared to ARMS-NT. There were no differences between ARMS-T and FEP groups. In both ARMS-T and FEP groups, anxiety was the most central symptom. In addition to anxiety, the FEP group also had unusual thought content emerging as a central feature.
**Discussion:** To our knowledge, this is the first study to investigate the intrinsic phenomenological connectivity and its relevance to psychosis in the clinical high-risk population. Risk of transition to psychosis relates to the consolidation of relationship among symptoms (clustering and modularity), but appears unrelated to the severity of symptoms per se. First episode of psychosis could be thought of as a state of high modular clustering among otherwise sparsely connected symptoms. Incongruent clustering (e.g. blunting with anxiety) is reminiscent of Bleuler’s concept of ambivalence being a fundamental feature of psychosis. Deconsolidation of symptom clustering could be the key to prevent transition to frank psychosis in high-risk individuals. Reducing the bridging symptoms (esp. anxiety) could weaken the clinical core of a psychotic episode, complementing the pharmacological approaches of reducing dopamine transmission.

### T213. THE EFFECT OF ACUTE STRESS ON PARANOID THINKING AND CORTISOL DURING SOCIAL INTERACTION IN HIGH AND LOW SCHIZOTYPES

James Gilleen*[,1]
[1]*Institute of Psychiatry*

**Background:** Paranoid thinking, a common symptom of psychosis and schizophrenia, manifests as a sense of threat and may also be indexed by a lack of trust. Stress, in turn, exacerbates psychosis and paranoia, and is a well-established risk factor for schizophrenia as well as a component in several models of psychosis. The present study aimed to determine the impact that acute stress has on paranoid thinking during social interaction in vivo in high (HSZ) vs low (LSZ) schizotypy using an iterated social reciprocity game. The main hypothesis was that HSZ would anticipate greater social threat and paranoia at baseline compared to LSZ, and moreover that experimentally-induced stress would exacerbate those differences, and thus show that stress differentially modulates how HSZ model the intentions of others.
**Methods:** Matched healthy participants were stratified into HSZ (N=17) and LSZ (N=17) groups and were administered a non-financial, social-reciprocity game against benevolent and malevolent opponents under both stress and no-stress conditions. Stress was manipulated using the MIST (Dedovic et al., 2005) stress paradigm. Cortisol was measured from saliva samples acquired before and after the MIST stress task. Anticipation of threat and trust scores were derived from the social interaction task.
**Results:** At baseline, cortisol levels were not significantly different between HSZ and LSZ but were significantly raised by the stressor task in HSZ (p<.05) but not in LSZ. Higher cortisol at baseline (pre-stress) predicted greater initial and average anticipation of threat (both r=.5, p<.05) (no-stress) to other players; and lower initial trust ratings of malevolent, but not benevolent, players. The MIST task significantly elevated stress ratings compared to baseline (p<.001) and following stress, greater change in cortisol from baseline to post-MIST was associated with lower trust ratings

(r=.51, p<.05). In HSZ greater stressor-related cortisol levels correlated with greater anticipation of threat (r=.58, p<.05) and lower trust levels (r=.57, p<.05) which was not apparent in low schizotypes.
**Discussion:** Acute stress elevates cortisol and paranoia in HSZ – in the form of greater anticipation of threat, and lower trust compared to LSZ. The study adds evidence to the role of stress in exacerbating schizophrenia-like experiences in those who are sub-threshold for schizophrenia and psychosis.

### T214. STIGMA ABOUT SCHIZOPHRENIA: THE EFFECTS OF DIAGNOSTIC LABELS, SYMPTOMS, AND ILLNESS PHASE

Christopher Groot*,[1], Kelton Hardingham[2]
*[1]Melbourne School of Psychological Sciences, The University of Melbourne; [2]Melbourne School of Psychological Sciences*

**Background:** Schizophrenia is possibly the most stigmatised of all mental disorders. It has been argued that the label of schizophrenia itself has become stigmatically charged through over a century of association with misleading and sensationalist representations in news media and pop-culture. There has, therefore, been considerable recent debate on the topic of changing the label of schizophrenia. Label change advocates argue that public stigma about schizophrenia would be reduced should the label be replaced with either an eponymous or descriptive alternative. However, few empirical studies to date have directly investigated this possibility. Moreover, no known single study has investigated the effects of descriptive and eponymous relabelling of schizophrenia on public stigma as a function of symptomatology and illness phase. The current study aimed to fill this gap in the literature.
**Methods:** Australian university students (m = 181, f = 176, other/unspecified = 4, Mage = 19.58 years, age range 17–60 years) participated in the study by reading a brief vignette depicting a protagonist with schizophrenia, and by subsequently completing a number of stigma measures. Participants were randomly allocated to vignette conditions that varied systematically by disorder label (schizophrenia vs eponymous label vs descriptive label), symptoms (positive vs negative symptoms), and illness phase (active vs remittent symptoms). Stigmatised thoughts, attitudes, and behaviour were measured using the Social Distance Scale (SDS; Link et al., 1987) and selected items forming six factors (Fear/Dangerousness, Help/Interaction, Responsibility, Forced Treatment, Pity, and Anger; Brown, 2008) from the Attributional Questionnaire (AQ; Corrigan et al., 2004). Socially desirable response bias and familiarity with mental illness were controlled using the Marlow-Crowne Social Desirability Scale (Crowne & Marlow, 1960), and Level-of-Contact Report (Holmes, Corrigan, Williams, Canar & Kubiak (1999).
**Results:** Data was analysed with a series of ANCOVA and ANOVA analyses. No group differences in either SDS or AQ scores were observed as a function of disorder label. Positive symptoms were observed to elicit significantly greater levels of fear/dangerousness responses and endorsement of forced treatment when compared to negative symptoms. However, negative symptoms elicited significantly greater anger responses. While only these isolated differences on stigma measures were observed for symptom profile contrasts, vignettes depicting an active illness phase elicited significantly greater levels of stigma on the SDS and most AQ factors when compared with vignette conditions depicting a symptomatically remittent protagonist. Lastly, no statistically significant interactions were observed between labels, symptomatology and illness phase.
**Discussion:** The results suggest that diagnostic label change may not be an effective strategy to reduce public stigma about schizophrenia in western countries. It is important to note, however, that public stigma elicitation is just one of the numerous considerations as regards the utility of label change, and that others, such as consumer perspectives, hold value independent of the current findings. The current findings also highlight that the symptomatology and illness phase of schizophrenia are likely to be implicated in eliciting public stigma about the disorder, and are worthy of further attention. While the current study investigated these phenomena at the broad symptom profile level, future research should investigate individual symptoms and their subtypes discretely, in order to inform a comprehensive, symptom-focussed model of elicited public stigma pertaining to schizophrenia.

### T215. CLINICAL PREDICTORS OF HOSPITALIZTIONS IN FIRST EPISODE PSYCHOTIC PATIENTS: A NATURALISTIC FOLLOW UP STUDY

Olga Sparano*,[1], Pau Soldevila-Matias[2], Carlos Gonzalez[2], Lucia Bonet[3], Esther Lorente[4], Jose Miguel Carot[5], Julio Sanjuan[4]
*[1]Hospital Universitario de la Ribera; [2]Hospital Clinico INCLIVA; [3]Universidad Valencia; [4]Hospital Clinico INCLIVA, CIBERSAM; [5]Universidad Politecnica*

**Background:** Some naturalistic longitudinal studies of first psychotic episodes of the last 50 years have suggested associations between psychopathology and the remission of symptoms and the clinical course of disease.[1] A recent study in a large sample of patients with schizophrenia has obtained significant results using the number of hospitalizations as outcome variable.[2]
The main objective of this study is to know if clinical and sociodemographic variables predict the number of hospitalizations after the first psychotic episode
**Methods:** Naturalistic, longitudinal follow-up study in a sample of 212 patients of first-episode psychosis attending public mental health service in Area 5 of Valencia (Spain) in a period between 2010–2017. Of 212 patients, a total of135 were included, excluding patients lost due to abandonment and death.
The study included a) baseline variables: sociodemographic, risk factors (Cannnabis use), clinical scales; PANSS, CGI (clinical global impression) and GAF (global assessment of functioning scale) and kind of treatment (oral versus injectable). b) outcome variables: number of visits to the emergency room, hospitalizations, and outpatient consultations.
**Results:** None of the psychopathological or treatment variables at baseline were significantly associated with the outcome variables.
The younger patients have a significant (p < 0.01) higher number of emergencies room visit in the follow up.
**Discussion:** In contrast with previous reports[1,2] Tihonen J et al2017)) we were not able to find any relationship between severity of illness (at baseline) or the kind of treatment (oral versus injectable) with the emergency rooms visits or number of hospitalizations.
The only significant result was related with the age of the patients. Younger patients have more probability of having more visit to the emergency room.

**References:**
1. Capdeville D. A multi-dimensional approach to insight and its evolution in first- episode psychosis: a 1 -year outcome naturalistic study. Psychiatry Res. 2013 dec 30;210(3):835–41
2. Tihonen J.Real-World effectiveness of antipsychotic treatments in a Nation wide Cohort of 29.823 patients with schizophrenia. Jamapsych. 2017;74(7)686–693

### T216. THE CULTURAL EXPERIENCES OF PATIENTS DIAGNOSED WITH SCHIZOPHRENIA IN SOUTH AFRICA

Wilna Basson*,[1]
*[1]Sefako Makgatho Health Sciences University*

**Background:** Schizophrenia is a debilitating mental illness that affects people from all walks of life. Individuals attach meaning to their illness based on their cultural point of view; for some traditional black South Africans, causes of ill health are ascribed to culturally laden inferences. Some patients seek spiritual help before consulting medical doctors. This study aimed to explore how black South Africans diagnosed with schizophrenia experience their illness from their cultural point of view.
**Methods:** The study followed a hermeneutic phenomenological approach. In-depth interviews were conducted with three patients diagnosed with schizophrenia and on medication for their illness. Their stories were analysed using thematic content analysis.
**Results:** Five themes emerged during the study. Theme 1 related to Naming Things. The name given to their illness significantly affected the meanings that were attached to the illness. Theme 2 referred to Being Without. Losses as well as gains became apparent. Participants had lost their roles, independence and intimacy; however, they developed other coping strategies and some relationships became stronger. Theme 3 pertained to Connections and Disconnections. While participants were connected to their families and their community, they also felt disconnected due to the stigma perceived. Theme 4 was the theme of Being Spiritual. Spirituality played a vital role in how participants attached meaning to their illness, and it helped them to cope with challenges. Theme 5 was Rainbow after the Rain. The negative connotation of having a mental illness turned into personal, inter-personal and spiritual growth. The devastating illness became a gift to all participants; they demonstrated immense levels of resilience and they found their own way of being and relating.
**Discussion:** Culture played a crucial role during the initial stages of the illness; all participants sought spiritual help and it determined the meanings attached to the illness. This study proposes a need for mental health workers to explore the challenges that hamper openness within families and communities in order to lessen the perceived stigma experienced by the patients, and to acknowledge and encourage different coping and meaning-making structures, such as spirituality.

### T217. FACTORS RELATED TO SUICIDAL BEHAVIOR IN KOREAN PATIENTS WITH BIPOLAR DISORDER: THE EFFECT OF MIXED FEATURES ON SUICIDALITY

Hye-Jin Seo*[,1], Hee Ryung Wang[2]
*[1]Yong-In Mental Hospital; [2]Yeouido St. Mary's Hospital, The Catholic University of Korea*

**Background:** The aim of the present study was to investigate various risk factors of suicidal behaviors, including mixed feature specifier, in Korean patients with bipolar disorder.
**Methods:** We retrospectively reviewed medical charts from 2005 to 2014. A total of 334 patients diagnosed with bipolar disorder using DSM-IV TR were enrolled. The subjects were categorized into two groups according to history of suicidal behaviors and compared regarding demographic and clinical characteristics including mixed feature specifier. We re-evaluated the index episode using the DSM-5 criteria and classified into index episode with and without mixed feature. Logistic regression was performed to evaluate significant risk factors associated with suicidal behavior.
**Results:** Suicidal behavior had independent relationship with mixed feature at index episode using DSM-5 criteria and number of previous depressive episodes in Korean bipolar patients. The mixed feature specifier was the strongest risk factor in the present study.
**Discussion:** Suicidal behavior had independent relationship with mixed feature at index episode using DSM-5 criteria and number of previous depressive episodes in Korean bipolar patients. The mixed feature specifier was the strongest risk factor in the present study.

### T218. IDENTIFICATION OF FIRST-EPISODE PSYCHOSIS SUBGROUPS BASED ON POSITIVE SYMPTOM DOMAINS AND THEIR SOCIODEMOGRAPHIC AND CLINICAL CORRELATES

Chiara Galletti*[,1], Luca Pauselli[2], Marc Manseau[3], Alfonso Tortorella[1], Michael T. Compton[2]
*[1]University of Perugia; [2]Columbia University; [3]New York University*

**Background:** Although the heterogeneous nature of psychosis is well established in the literature, genetics, neurophysiology, and neuroimaging have not yet succeeded in an unequivocal classification of the diverse clinical presentations. For the time being, the field relies mostly on a symptom-based, descriptive diagnostic system. The purpose of the current study was to use seven previously identified positive symptom domains to identify possible subgroups of first-episode psychosis (FEP), using cluster analysis. In addition, we tested whether possible subgroups differed across a number of sociodemographic and clinical variables.
**Methods:** We analyzed data from a large FEP sample (n=247) to identify possible clinical subgroups of psychosis based on positive symptoms; specifically, delusion and hallucination domains resulting from previous factor analyses of Scale for the Assessment of Positive Symptoms (SAPS) items in this sample. A rigorous methodology was applied to perform cluster analysis and identify FEP subgroups. Kruskal-Wallis tests with pairwise post-hoc comparisons were used to check the differences in each positive symptom domain between the subgroups. Bivariate analyses comparing subgroups on a number of sociodemographic and clinical characteristics were performed.
**Results:** Five FEP subgroups were identified based on the severity of seven domains of delusions and hallucinations. Three of them (Mild Positive Symptoms Subgroup, Moderate Positive Symptoms with Sin/Guilt and Jealousy Delusions Subgroup, Severe Positive Symptoms Subgroup) shared a similar psychopathological profile, with typical psychotic symptoms differing in severity. Another subgroup was characterized by high severity on typical and atypical symptoms (Severe Mixed Atypical Positive Symptoms Subgroup), and the other by high severity in somatic delusions (Somatic Delusions Subgroup). The five subgroups did not significantly differ in terms of gender, age at onset, family history, mode of onset of psychosis, premorbid functioning, and alcohol and non-cannabis drug use disorders, though some potential signals were identified, (but not reaching statistical significance due to small sample sizes in the subgroups). Significantly different prevalence rates of cannabis use disorder were found across the five subgroups.
**Discussion:** In our analysis, five possible FEP subgroups were identified based on the severity of seven domains of delusions and hallucinations: three of them shared a similar psychopathological profile differing in severity, and two were characterized by different atypical symptoms. Despite several acknowledged limitations, our results highlight the potential to identify clinical phenotypic subgroups of FEP, which may be helpful in future research aimed at filling the gaps between clinical, neuropathological, and genetic explanations of psychosis etiology. Such an approach may also lead to better targeted preventive interventions and more individualized and effective treatments.

### T219. THE ROLE OF MELATONIN AND MELATONIN AGONISTS IN COUNTERACTING ANTIPSYCHOTIC-INDUCED METABOLIC SIDE EFFECTS: A SYSTEMATIC REVIEW

Won-Myong Bahk*[,1], Young Joon Kwon[2], Bo-Hyun Yoon[3], Sang-Yeol Lee[4], Kwanghun Lee[5], Duk-In Jon[6], Moon Doo Kim[7], Eunsung Lim[8]
*[1]Catholic University of Korea; [2]Soonchunhyang University; [3]Naju National Hospital; [4]Wonkwang University, School of Medicine; [5]College of Medicine, Dongguk University; [6]College of Medicine, Hallym University; [7]School of Medicine, Jeju National University [8]Shinsegae Hospital*

**Background:** Melatonin administration to high cholesterol-treated and high fat-treated rats has been shown to suppress body weight and visceral adiposity. In addition, in various animal models related to obesity, metabolic syndrome, and diabetes, melatonin has beneficial efficacy in ameliorating various metabolic symptoms, including attenuating weight gain, lowering blood pressure (BP), and improving insulin resistance. This systematic review aims to investigate whether melatonin or melatonin agonists significantly attenuate metabolic side effects among psychiatric populations treated with atypical antipsychotics.
**Methods:** Four randomized controlled trials were identified through a comprehensive literature search using MEDLINE, EMBASE, and the Cochrane Library on 22 October 2015. These four trials (including three melatonin studies and one ramelteon study) included 138 patients, of whom 71 were treated with melatonin or ramelteon and 67 were treated with a placebo. Because of high heterogeneity, we did not carry out a meta analysis.
**Results:** Melatonin was beneficial in lowering blood pressure among bipolar disorder patients; this blood pressure-lowering effect was not prominent among schizophrenic patients. Melatonin appeared to improve lipid profiles and body composition and attenuated weight gain among both schizophrenic and bipolar disorder patients. Ramelteon showed a significant efficacy in lowering total cholesterol level.
**Discussion:** Despite the few studies included, this systematic review provided promising evidence of the potential benefits of melatonin and its agonists in attenuating one or more components of metabolic syndrome among psychiatric patients using atypical antipsychotics.

### T220. THE GLUTAMINASE INHIBITOR EBSELEN PREVENTS AMPHETAMINE SENSITIZATION IN MICE

Lior Heffetz-Giterman[1], Sharon S. Lander[1], Reut Cohen[1], Amit Gross[1], Inna Gaisler-Salomon*[,1]
[1]*University of Haifa*

**Background:** Dysregulated glutamatergic neurotransmission has been strongly implicated in the pathology of schizophrenia (SZ). Glutaminase 1 (GLS1) plays a critical role in the recycling of glutamate. GLS1 deficient mice were previously shown to display an attenuated response to the acute and chronic effects of the dopamine-releasing psychotomimetic drug amphetamine and have a pro-cognitive profile. A recent large-scale drug screening study identified ebselen as a potent CNS-available GLS1 inhibitor. Here, we asked whether ebselen (10 mg/kg) would attenuate sensitization to amphetamine (4 mg/kg) and induce pro-cognitive behavior.
**Methods:** Sensitization to amphetamine (4mg/kg) was tested in the open field. Mice received either saline, amphetamine or amphetamine+ebselen (10mg/kg) i.p. on 4 consecutive days. Seven days later, mice were challenged with amphetamine, amphetamine+ebselen or saline. We further assessed the effect of ebselen administration on Gls1 mRNA in the hippocampus, prefrontal cortex and striatum, and on dopamine receptor expression in the striatum. Finally, we measured social preference and recognition in genetically modified GLS1 deficient mice and in ebselen (10mg/kg)-treated wild-type mice.
**Results:** We found decreased sensitization to amphetamine in mice that received pre-treatment with ebselen. Gene expression studies revealed reduced Gls1 expression in hippocampus, and altered expression of dopamine markers in the striatum of ebselen-treated mice. Finally, ebselen-treated mice show enhanced social recognition, similarly to GLS1 deficient mice.
**Discussion:** Similarly to genetically modified GLS1 deficient mice, ebselen-treated mice demnstrate resilience to the sensitizing effects of the pro-psychotic drug amphetamine and a pro-cognitive phenotype. These findings provide evidence for the potential of GLS1 inhibition in addressing some of the central clinical features of SZ and related pathology.

### T221. LURASIDONE DISPLAYS ANTIDEPRESSANT AND PRO-COGNITIVE EFFECTS IN THE CHRONIC MILD STRESS MODEL: A ROLE FOR REDOX MECHANISMS AND PARVALBUMIN EXPRESSION

Andrea Carlo Rossetti[1], Maria Serena Paladini[1], Paola Brivio[1], Giulia Sbrini[1], Mariusz Papp[2], Francesca Calabrese[1], Raffaella Molteni[1], Marco Andrea Riva*[,1]
[1]*University of Milan;* [2]*Polish Academy of Sciences, Krakow*

**Background:** Exposure of rodents to chronic stress is able to recapitulate a number of functional alterations that are associated with psychiatric disorders, including anhedonia and cognitive impairment. Stress-based experimental models are also useful to investigate the ability of pharmacological intervention in normalizing such defects as well as the molecular alterations associated with the behavioral phenotype. On these bases, the aim of the present study was to investigate the ability of a chronic treatment with the multi-receptor modulator lurasidone in normalizing behavioral changes produced by chronic mild stress (CMS) in rats. Moreover, we investigated the potential contribution of parvalbumin expression and of redox mechanisms in the alterations brought about by CMS exposure.
**Methods:** Adult male Wistar rats were exposed to CMS for 2 weeks and sucrose consumption was used to identify rats that were susceptible to the stressful manipulation. Control and CMS-susceptible rats were then randomized to receive chronic vehicle or the multi-receptor modulator lurasidone (3 mg/kg/day) for 5 more weeks, while continuing the stress procedure. Animals were tested for anhedonia, using the sucrose intake test, and for cognitive impairment, using the novel object recognition (NOR) test. Rats were sacrificed at the end of the procedures and the brain regions of interest were dissected and used for the molecular analyses.
**Results:** Exposure to CMS produced a persistent anhedonic phenotype as well as a significant deficit in the NOR test. Both behavioral abnormalities were normalized by chronic lurasidone treatment. Rats exposed to CMS display a marked and selective reduction in the expression of parvalbumin, which identifies a subpopulation of GABAergic interneurons, in dorsal (but not ventral) hippocampus, an effect that was normalized by chronic lurasidone administration. CMS rats also show a significant up-regulation of (NADPH) oxidase 2 (NOX2), which is critically involved in oxidative stress, as well as a down-regulation of Nrf-2, a master regulator of antioxidant defense. These alterations in dorsal hippocampus were normalized in animals that received chronic lurasidone treatment that was also capable of reducing the levels of Keap-1, an important player that exerts a repressive control over Nrf-2.
**Discussion:** Our results demonstrate the ability of lurasidone in normalizing anhedonia and cognitive deficits associated with CMS exposure, suggesting its effectiveness on different ‘domains’ (RDoC) that characterize psychiatric disorders. Lurasidone was also able to normalize the effects produced by CMS exposure on parvalbumin expression, possibly through its ability in promoting anti-oxidative mechanisms within the dorsal hippocampus. All in all, these effects may promote resilience toward the alterations produced by adverse environmental conditions, such as stress, which represents a major vulnerability element in the etiology of psychiatric disorders.

### T222. EARLY TREATMENT RESISTANCE IN A LATIN-AMERICAN COHORT OF PATIENTS WITH SCHIZOPHRENIA

Cristian Mena[1], Alfonso Gonzalez-Valderrama[1], Barbara Iruretagoyena[2], Juan Undurraga[1], Nicolas Crossley*[,2]
[1]*J. Horwitz Psychiatric Institute;* [2]*Universidad Católica de Chile*

**Background:** Failure to respond to antipsychotic medication in schizophrenia is a common clinical scenario with significant morbidity. Recent studies have highlighted that many patients present treatment-resistance from disease onset. We here present an analysis of clozapine prescription patterns, used as a real-world proxy marker for treatment-resistance, in a cohort of 1195patients with schizophrenia from a Latin-American cohort, to explore the timing of treatment resistance during the course of the disease and possible subgroup differences.
**Methods:** We used survival analysis from national databases of clozapine monitoring system, national disease notification registers, and discharges from an early intervention ward.

**Results:** Echoing previous studies, we found that around 1 in 5 patients diagnosed with schizophrenia were eventually prescribed clozapine, with an over-representation of males and those with a younger onset of psychosis. The annual probability of being prescribed clozapine was highest within the first year (probability of 0.11, 95% confidence interval of 0.093–0.13), compared to 0.018 (0.012–0.024) between years 1 and 5, and 0.006 (0–0.019) after 5 years. There were no differences in age at psychosis onset or gender related to the onset of treatment resistance. A similar pattern was observed in a subgroup of 230 patients discharged from an early intervention ward with a diagnosis of non-affective first episode of psychosis.
**Discussion:** Our results highlight that treatment resistance is frequently present from the onset of psychosis. Future studies will shed light on the possible different clinical and neurobiological characteristics of this subtype of psychosis.

### T223. REAL WORLD EFFECTIVENESS OF ANTIPSYCHOTIC DRUGS IN PATIENTS WITH SCHIZOPHRENIA: A 10-YEARS RETROSPECTIVE STUDY

Tae Young Lee*[,1], Minah Kim[1], Junhee Lee[1], Jun Soo Kwon[1]
[1]*Seoul National University College of Medicine*

**Background:** Discontinuation of antipsychotics in patients with schizophrenia has increasing attention as a representative treatment effectiveness of the medication. The objective of present study is to determine which antipsychotic medication is highly effective in a real-world clinical setting considering adjuvant pharmacotherapy over a 10-year follow-up period.
**Methods:** A total of 2300 patients with schizophrenia were recruited at the Seoul National University Hospital, and participants received amisulpride, aripiprazole, clozapine, haloperidol, olanzapine, paliperidone, quetiapine, risperidone, ziprasidone for up to 10-years. Time-to-discontinuation of antipsychotic medications was analyzed using Kaplan-Meier survival analyses. Group differences were compared using log-rank tests.
**Results:** The most frequently used drugs were Risperidone, Aripiprazole, Olanzapine in the antipsychotic drug, Valproate, Lamotrigine, Topiramate in the anticonvulsant agents, Escitalopram, Sertraline, Milnacipran in antidepressants, Lorazepam, Clonazepam, Zolpidem in anxiolytics or sedatives, Propranolol, Benztropine, Trihexyphenidyl in antiparkinson drugs. About half of the patients discontinued taking antipsychotics before 1.5 years. Clozapine showed significantly longer time to discontinuations compared to other antipsychotic drugs. Aripiprazole also showed a lower incidence of discontinuation except for Clozapine and Olanzapine.
**Discussion:** Clozapine was found to be the most effective antipsychotics in terms of time to discontinuations. Aripiprazole is the most highly recommended 1st line antipsychotics.

### T224. THE FUNCTIONAL CONNECTIVITY DERIVED WITH BIVARIATE ANALYSIS, COHERENCE AND PHASE LOCKING VALUE IN PATIENTS WITH SCHIZOPHRENIA UNDER CLOZAPINE

Yong Sik Kim*[,1], In Won Chung[1], Hee Yeong Jung[2], Tak Youn[1], Se Hyun Kim[1], Nam Young Lee[1], Seong Hoon Jeong[3], Kyung Tae Park[4], Sang Hoon Yi[4], Yong Min Ahn[2]
[1]*Dongguk University School of Medicine;* [2]*Seoul National University;* [3]*Eulji University Hospital;* [4]*Inje University*

**Background:** Coherence (COH) and Phase Locking Value (PLV) may have considerable potentials for investigating anomalies of functional connectivity in schizophrenia but results are still conflicting. This study is aimed to investigate relationships between plasma levels of clozapine (p-CZP) and norclozapine (p-NCZP), and total and cognitive factor scores of Positive and Negative Syndrome Scale (PANSS-T, -C), and functional connectivity by COH and PLV.
**Methods:** Fifty-eight patients who were diagnosed as schizophrenia with DSM-5 criteria and under CZP were recruited (duration of illness, 15.5 ± 8.0 years; duration of CZP, 6.8 ± 4.6 years; mean daily dose of CZP, 233.6 ± 88.4 mg). COH and PLV were calculated with Neurophysiological Biomarker Toolbox from qEEG and were averaged from the signals of electrodes in the designated brain regions, frontal (F), temporal (T), central (C) and occipitoparietal (OP). For interhemispheric connectivity, electrodes except all midline channels were combined into Odd (O) and Even (E). The results were presented at ≥0.30 of Spearman correlation.
**Results:** 1) Correlation coefficient between p-CZP and p-NCZP was 0.84, and those of CZP dose with p-CZP and p-NCZP were 0.38 and 0.53, respectively. 2) p-CZP showed correlations with OCEC in delta and alpha, OTEC in delta, OCEOP in theta, OTEF in alpha, and OOPEF in gamma band in COH, and OOPEOP in beta band in PLV. 3) p-NCZP showed correlations with ETEOP in delta, theta, and gamma, OCEC in delta and alpha, OFOC and OCEOP in delta, OFET and OTET in alpha, OCEF in beta, OOPEC in gamma band in COH, and with ETEOP in delta, theta, and beta, OTET and OCEC in alpha, OCEF in beta band in PLV. 4) CZP dose showed correlations with ETEC in beta and gamma, ETEOP in theta, OCEF in alpha, OTET in beta, OOPEF and OOPET in gamma band in COH, and with OTET in alpha and beta, ETEOP in theta, OTEOP in alpha, ETEC in beta, OFOC in gamma band in PLV. 5) PANSS-T showed correlations with OFEOP and EFEOP in alpha, OCEOP in beta, OTOC and OTEF in gamma band in COH, and with OTEF in beta and gamma, OFET in delta, OOPEF in beta, OTOC and OCEOP in gamma band in PLV. 6) PANSS-C showed correlations with EFEOP in delta, theta, alpha, and beta, OOPEOP in delta, alpha, and beta, OFET and OTEF both in alpha and beta, OOPEF in delta, OFEOP in alpha, OFEC and OCEOP in beta, OTOC in gamma band in COH, and with EFEOP in theta, alpha, and beta, OFEOP and OOPEOP both in alpha and beta, OFET in delta and beta, OTOC, OOPEF, OOPEOP in beta, OTOC and OCEOP in PLV. 7) PANSS-T and -C showed no correlations with p-CZP, p-NCZP and CZP dose. 8) However, the clinical and drug variables showed significant simultaneous correlation with certain functional connectivity, but sometimes the direction correlation was opposite.
**Discussion:** The relationship between functional connectivity and clozapine parameters seems to demonstrate inter- and intra-hemispheric connections in brain regions. However, there were same and/or opposite directions of correlations between COH and PLV dependent EEG band frequencies and clinical and drug variables. Taken together, investigating the functional connectivity with COH and PLV could give the information about p-CZP and p-NCZP before the laboratory reports, the degree of psychopathology in patients with schizophrenia under CZP, and the differentiations of surface symptoms whether derived from pathophysiology of schizophrenia or from clozapine effects.

### T225. OPERATIONAL DEFINITIONS FOR ANTIPSYCHOTIC RESPONSE IN DELUSIONAL DISORDER: A SYSTEMATIC AND CRITICAL REVIEW

Alexandre González-Rodríguez*[,1], Francesc Estrada[1], José Antonio Monreal[2], Diego Palao[2], Javier Labad[2]
[1]*Parc Taulí University Hospital;* [2]*Parc Taulí University Hospital, CIBERSAM, Autonomous University of Barcelona (UAB)*

**Background:** Recent research in schizophrenia has revealed that there is no consensus to which is the most appropriate definition for antipsychotic response. Response rates allow the clinician to know how many subjects have responded to a specific treatment. However, once again, levels of response or the cutoff chosen have been subject of controversy, as a high variety of values have been applied in schizophrenia research. This systematic review aimed to examine all the definitions used for antipsychotic response in delusional disorder (DD), to analyze them and provide a discussion of the methodology used.
**Methods:** A systematic computerized literature search was performed by using Pubmed, Scopus and PsycINFO databases (1990-September 2017) according to the Preferred Reporting Items for Systematic Reviews and Meta-Analyses (PRISMA) statement. In addition, reference searches were manually conducted through identified studies in order to obtain other relevant articles not previously found on the initial search strategy. The following search terms were used: [(Antipsychotic response) OR (antipsychotics) OR (treatment response)] AND [(delusional disorder)]. Studies were only included if the met our inclusion criteria: (a) be published in a peer-reviewed journal, (b) prospective or retrospective studies focusing on antipsychotic response, (c) studies assessing response in DD based on clinical judgment or clinical records, (d) studies including a definition of antipsychotic response based on assessment scales and (e) diagnostic criteria based on ICD or DSM. The literature search strategy was conducted independently by two of the authors (A.G. and F.E.). The last search was conducted on 30th October, 2017.
**Results:** Seventy-four studies were initially identified. 39 studies were excluded after titles and abstracts were read, as they did not meet our initial inclusion criteria or met any of the exclusion criteria. 22 studies were excluded after reading the full text-document as they failed to meet our inclusion criteria or met any exclusion criteria, and 2 articles were excluded as studies for the assessment were duplicated. After the screening and selection processes, a total of 11 studies met our inclusion criteria, using different methods to define antipsychotic response in DD. Chart review (n=5) and observer-rated scales (n=6), from which 2 of them used the CGI improvement scale for assessing response, 2 studies evaluated it by mean changes from baseline to endpoint scores (PANSS, BPRS), one study combined the CGI improvement scale and mean changes from baseline scores (PANSS), and one study reported responder rates based on a scale-derived cutoff (PANSS).
**Discussion:** A lack of consensus in the definitions of antipsychotic response in delusional disorder and a high degree of heterogeneity of the methods used are reflected on this systematic review. Although no consensus for the response definition appears to exist in delusional disorder, there is a need to better quantify the treatment response in terms of percentages of response, and linking these findings with those derived from the CGI. Recommendations from Leucht (2014) in schizophrenia would be a first step in defining response among delusional disorder patients.

### T226. CLINICAL PREDICTORS OF FUNCTIONAL CAPACITY IN TREATMENT RESISTANT SCHIZOPHRENIA PATIENTS: COMPARISON WITH RESPONDER PATIENTS, ROLE OF NEGATIVE SYMPTOMS, PROBLEM SOLVING DYSFUNCTIONS, AND NEUROLOGICAL SOFT SIGNS

Felice Iasevoli*[,1], Luigi D'Ambrosio[1], Danilo Notar Francesco[1], Eugenio Razzino[1], Camilla Avagliano[1], Elisabetta Filomena Buonaguro[1], Thomas Patterson[2], Andrea de Bartolomeis[1]
*[1]University of Naples "Federico II"; [2]University of California, San Diego*

**Background:** Treatment Resistant Schizophrenia (TRS) patients show more severe impairments in community functioning compared to Antipsychotic Responder Schizophrenia (ARS) patients. The scope of this work was to assess whether TRS patients suffer from more severe alterations in functional capacity, i.e. the baseline potential of a patient to function in the community, and whether factors affecting functional capacity differ between TRS and ARS patients.
**Methods:** 60 out of 182 eligible patients were included. A multistep diagnostic procedure to separate TRS from ARS was then used. Patients were administered a range of assessment tools including (but not limited to): the PANSS; cognitive performances tests; the Specific Level of Functioning (SLOF); the Neurological Evaluation Scale (NES); the UCSD Performance-Based Skills Assessment (UPSA) extended version. Univariate and multivariate statistics were performed. Significance was set at $p<.05$.
**Results:** After controlling for covariates, no significant differences in both total and subscales UPSA scores were found between TRS and ARS patients. However, TRS patients constantly scored lower than ARS patients. Stepwise regression was used to determine predictors of UPSA score. The first group encompassed clinical variables. In the whole sample, the final significant model, $F(2,57)=18.848$, $p<.0005$, adjusted $R2=.37$, included: PANSS negative subscale score and NES score. In TRS patients, the final significant model, $F(3,24)=16.552$, $p<.0005$, adjusted $R2=.63$, included PANSS negative scale score, education years, and NES score. In ARS patients, no significant models were found.
The second group included cognitive performance variables. In the whole sample, the final significant model, $F(2,57)=7.64$, $p=.001$, adjusted $R2=.18$, included Problem Solving and Verbal Memory. In TRS patients, the final significant model included Problem Solving and VisuoSpatial Memory. In ARS patients, the final significant model included Verbal Memory only.
The third group included psychosocial variables. In the whole sample, the final significant model, $F(1,58)=18.82$, $p<.0005$, adjusted $R2=.23$, included SLOF Area5 score only. In TRS patients the final significant model included SLOF Area1 score only, while in ARS patients, no significant models were found.
By hierarchical multiple regressions, NES score was found to be predictive of the highest UPSA score variance ratio among schizophrenia patients. The addition of PANSS Negative scale score and Problem Solving (in this order) led to a statistically significant increase in R2. No further models were found to add significant increase in R2. In TRS patients, PANSS Negative scale score was the variable that explained the most variance in UPSA score. The addition of Problem Solving and education years (in this order) led to a statistically significant increase in R2. No further models were found to add significant increase in R2, although NES score showed a trend toward significance.
At last, we performed a path analysis to evaluate the type (direct or indirect) and the direction of relationships among these variables and UPSA score. The only variables that were in direct relationship with UPSA score were PANSS Negative Scale score and Problem Solving. SLOF Area5 was in indirect relationship with UPSA score by PANSS Negative Scale score, while NES score relationship with UPSA score was mediated by Problem Solving.
**Discussion:** Our study demonstrated that negative symptoms, altered cognitive performances, and more severe neurological soft signs were the major factors influencing functional capacity in schizophrenia patients. These factors were more relevant in TRS than in ARS patients.

### T227. THE METABOTROPIC GLUTAMATE RECEPTOR SUBTYPE 1 REGULATES STRIATAL DOPAMINE RELEASE VIA AN ENDOCANNABINOID-DEPENDENT MECHANISM: IMPLICATIONS FOR THE TREATMENT OF SCHIZOPHRENIA

Samantha Yohn*[,1], Daniel Covey[2], Daniel Foster[1], Mark Moehle[1], Jordan Galbraith[1], Joseph Cheer[2], Craig Lindsley[1], P. Jeffrey Conn[3]
*[1]Vanderbilt University; [2]University of Maryland School of Medicine; [3]Vanderbilt University Medical Center*

**Background:** Clinical and preclinical studies suggest that selective activators of the muscarinic M4 receptor have exciting potential as a novel approach for treatment of schizophrenia. M4 reduces striatal dopamine (DA) though release of endocannabinoids (eCB), providing a mechanism for local effects on DA signaling in the striatum. M4 signals through Gαi/o and does not couple to Gαq/11 or induce calcium (Ca++) mobilization. This raises the possibility that M4-induced eCB release and inhibition of DA release may require co-activation of another receptor that activates Gαq/11. If so, this receptor could provide a novel target that may be more proximal to inhibition of DA release. Interestingly, the group 1 metabotropic glutamate (mGlu) receptors (mGlu1 and Glu5), couple to Gαq/11 and activate eCB signaling in multiple brain regions.
**Methods:** We tested the hypothesis that M4-induced reductions in DA release and subsequent antipsychotic-effect requires co-activation of group 1 mGlu receptors. The effect of M4 activation on electrically-evoked DA release in striatal slices was assessed using fast-scan cyclic voltammetry (FSCV) in the absence or presence of selective negative allosteric modulators (NAMs) of group 1 mGlu receptor subtypes. To evaluate the potential role of mGlu1, we determined the effects of a selective mGlu1 positive allosteric modulators (PAMs) on striatal DA release and antipsychotic-like activity in rodent models that are dependent on increased DA transmission. Since reductions in DA signaling, including D1 signaling have been implicated in reduced motivation, we also determined the effects of an mGlu1 PAM, M4 PAM, and the typical antipsychotic haloperidol on motivational responding in a progressive ratio (PR) schedule.
**Results:** We now present exciting new data in which we found that activation of mGlu1 through application of exogenous agonists or selective stimulation of thalamostriatal afferents induces a reduction of striatal DA release and that selective mGlu1 PAMs have robust antipsychotic-like effects in rodent models. Interestingly, our studies also suggest that mGlu1 activation is required for M4 PAM-induced inhibition of DA release and antipsychotic-like effects. However, in contrast to available antipsychotic agents, the present results and previous studies suggest that mGlu1 and M4 PAMs reduce DA signaling through local release of an eCB from striatal SPNs and activation of CB2 receptors on neighboring DA terminals to reduce DA release. While these studies suggest that the effects of M4 PAMs on DA release require activation of mGlu1, we have also found that these targets have important differences. Most notably, M4 PAMs also directly inhibits D1 signaling in D1-SPN terminals in the substatnia nigra pars reticulata (SNr). Unlike M4, mGlu1 does not directly inhibit DA D1 receptor signaling and does not induce behavioral changes that could be associated with negative symptoms.
**Discussion:** Our findings are especially interesting in light of recent findings that multiple loss of function single nucleotide polymorphisms (SNPs) in the human gene encoding mGlu1 (GRM1) are associated with schizophrenia, and points to GRM1/mGlu1 as a gene within the "druggable genome" that could be targeted for treatment of schizophrenia. Recent clinical imaging studies suggesting that symptoms in schizophrenia patients are associated with selective increases in striatal DA signaling and while extrastriatal regions display hypo-dopaminergic function; thus, mGlu1 and M4 PAMs may provide a mechanism for selective inhibition of DA release in striatal regions that are important for antipsychotic efficacy, without further disruptions in extrastriatal DA signaling.

### T228. VARIABILITY AND UNDERUTILISATION OF CLOZAPINE IN SPAIN

Francisco Javier Sanz-Fuentenebro*[,1], Jose Juan Uriarte Uriarte[2], Pere Bonet Dalmau[3], Vicente Molina Rodriguez[4], Alba Lubeiro Juárez[4], Miguel Bernardo Arroyo[5]
*[1]Hospital 12 de Octubre; [2]Gestión Clínica, Red de Salud Mental de Bizkaia; [3]Salud Mental Fundación Althaia, "Consell Assessor de Salut Mental"; [4]University of Valladolid; [5]Hospital Clínic, Universitat De Barcelona, IDIBAPS, CIBERSAM*

**Background:** The analysis of the information available in many countries on the use of clozapine, systematically indicates low prescription, underdosing and delay in the start of treatment. But as striking as this underutilization is the great variability between territories studied, which are related to multiple factors, and have led to various initiatives to improve its use. We do not have studies that evaluate these aspects in the Spanish population, so we have considered a first approximation through samples from four territories.
**Methods:** The authors analyzed the prescription data of clozapine in Castilla y León, the Basque Country, Catalonia and a Southern Madrid Area.
**Results:** The patients diagnosed with schizophrenia who receive treatment in the territories studied oscillate around 0.3%; the treatments with clozapine / 10000 inhabitants between 33.0 and 57.0; and patients diagnosed as schizophrenia receiving clozapine account for between 13.7% and 17.9% of those treated. The coefficient of variation between centers and prescribers is frequently higher than 50%.
**Discussion:** The global clozapine prescription data in the territories studied are in the range of countries in our environment. The variability in the prescription is very high and increases as we analyze smaller territories, until a great heterogeneity of the individual prescription.

### T229. ANTIPSYCHOTIC DRUG USE AND THYROID FUNCTION IN PATIENTS WITH SEVERE MENTAL DISORDERS

Trude Iversen*[,1], Nils E. Steen[1], Kåre I. Birkeland[1], Ragni H. Mørch[1], Elina J. Reponen[1], Jannicke F. Anderssen[1], Linn Rødevand[1], Ingrid Melle[1], Ole A. Andreassen[1], Espen Molden[2], Erik Jönsson[3]
*[1]University of Oslo, Oslo University Hospital; [2]Diakonhjemmet Hospital; [3]Oslo University Hospital, University of Oslo, Karolinska Institutet*

**Background:** Altered levels of free Thyroxin (fT4) and Thyroid Stimulating Hormone (TSH) have been associated with severe mental disorders and the use of antipsychotic drugs. Still, there is a lack of studies systematically investigating commonly prescribed antipsychotic drugs and thyroid function. We investigated the association of antipsychotic drugs and thyroid hormones levels in patients with severe mental disorders and compared thyroid function tests between patients and healthy controls under real-life conditions.
**Methods:** We included 1345 patients with schizophrenia or bipolar disorders and 989 healthy controls from the on-going Thematically Organized Psychosis (TOP) study, recruiting participants between 18–65 years of age in and around Oslo, Norway. All patients underwent a thorough clinical investigation including diagnostic evaluation, somatic screening and assessment of medication data. Serum drug concentrations were measured. Plasma levels of fT4 and TSH were measured in patients and healthy controls, and thyroid status was determined based on the combined hormone levels. Participants with known thyroid function disorders (N=28) were excluded. Mann-Whitney U tests and chi-square tests were performed for comparison between groups. For evaluation of influence from antipsychotics, multiple linear regression analyses were performed, adjusting for patient/control status, age, sex and use of other psychopharmacological agents. Associations with the use of olanzapine, quetiapine, aripiprazole or risperidone in monotherapy were analyzed in a subsample of patients (N=480), adjusting for age, sex and diagnosis. Spearman correlation analyses were performed for hormone levels and drug serum concentrations.
**Results:** We found significant lower levels of fT4 (median 13.70 vs 14.00, $p<0.001$) and higher levels of TSH (median 1.92 vs 1.57, $p<0.001$) in patients compared to healthy controls. A significant difference between patients and controls in occurrence of hyper- and hypothyreosis was observed ($p<0.001$), with more than three times as many patients compared to controls with hypothyroid status (11.1% vs 3.4%), and a doubling of hyperthyroid status (2.3% vs. 1.2%). Use of antipsychotics was significantly associated with lower fT4 level (p=0.001), but not with the TSH level. We also found significant associations between lower fT4 level and current use of quetiapine (p=0.005) and olanzapine (p=0.018), but again no significant associations were found with TSH level. No significant correlations were found between drug serum concentrations and fT4 or TSH. In the regression analyses we also observed that female sex and increasing age was associated with lower levels of both fT4 and TSH.

**Discussion:** In this large, cross-sectional study we found significant differences in thyroid hormone levels between patients with schizophrenia and bipolar disorders and healthy controls, and our data indicate a notable prevalence of undetected deviant thyroid function in the patient population. There was a significant association between fT4 level and the use of antipsychotics, particularly with the use of quetiapine and olanzapine. This suggests a possible contribution to altered thyroid hormone levels from the use of commonly prescribed antipsychotic agents. These findings call for renewed attention towards the role of thyroid function in severe mental disorders and the associations with antipsychotic drugs.

### T230. DOSE TRENDS OF ARIPIPRAZOLE FROM 2004 TO 2014 IN PSYCHIATRIC INPATIENTS IN KOREA

Young-Joon Kwon*[,1], Won-Myong Bahk[2], Bo-Hyun Yoon[3], Duk-In Jon[4], MoonDoo Kim[5], Beomwoo Nam[6], Eunsung Lim[7], Sung-Yong Park[8]
*[1]Soonchunhyang University Chunan Hospital; [2]Yeouido St. Mary's Hospital, The Catholic University of Korea; [3]Naju National Hospital; [4]College of Medicine, Hallym University; [5]School of Medicine, Jeju National University; [6]School of Medicine, Konkuk University; [7]Shinsegae Hospital; [8]Keyo Hospital*

**Background:** The purpose of the present study was to evaluate the initial and maximum doses of aripiprazole over a decade to estimate appropriate dosage in clinical practice. We hypothesized that there was a measurable change in dosing patterns during 2004–2014 in Korean psychiatric inpatients.

**Methods:** In this retrospective study, we reviewed the medical records of patients who were hospitalized in the psychiatric ward of five university hospitals in Korea from March 2004 to December 2014. The patients were at least 18 years of age, prescribed aripiprazole during the index hospitalization and were given at least one prescription for oral aripiprazole. We compared baseline demographic variables among Waves 1 (2004–2006), 2 (2007–2010) and 3 (2011–2014) using univariate one-way analysis of variance (ANOVA) with Bonferroni correction for continuous variables and a chi-square test for categorical variables.

**Results:** There was a significant difference in mean age among waves (p = 0.012). The use of concomitant medications with aripiprazole was significantly different among waves, as well. The use of other atypical antipsychotics in Wave 1 was 27.0% (n = 20) and 27.4% (n = 55) in Wave 2 and increased to 36.5% (n = 129) in Wave 3, but the difference between Waves 1 and 3 (p = 0.118) and 2 and 3 (p = 0.027) did not reach statistical significance after Bonferroni's correction. In total, the initial dose of aripiprazole was significantly lower in Wave 3 (7.0 ± 3.9 mg/day) when compared to Waves 1 (10.9 ± 4.6 mg/day, p<0.001) and 2 (10.7 ± 5.6 mg/day, p<0.001). The initial doses of aripiprazole in all diagnostic groups were significantly lower in Wave 3 than in Wave 2.

**Discussion:** The results from the present study show that the initial doses of aripiprazole, and not the maximum doses, decreased in hospitalized psychiatric patients with the accumulation of clinical experience in aripiprazole use.

### T231. PALIPERIDONE LONG-ACTING INJECTABLE (LAI) IS ASSOCIATED WITH A LOWER INTAKE OF BENZODIAZEPINES AND A LOWER NUMBER OF ADMISSIONS COMPARED WITH OTHER LAIS IN A COHORT OF PATIENTS WITH SCHIZOPHRENIA

Juan-Antonio Garcia-Carmona*[,1]
*[1]Reina Sofia University Hospital*

**Background:** Schizophrenia is characterized by a chronic course that in most of cases requires continued and long-term treatment by the use of long acting injectables (LAI). Several LAI formulations have been available and increased the number of pharmacotherapeutic options for schizophrenia. However, there are no formal recommendations regarding the use of specific LAIs for the treatment because there are only a few available studies and even fewer studies assessing which LAI formulation is more effective or suppose an advantage. Many studies has been published regarding the prescription patterns of oral anti-psychotic and the transition to a LAI treatment in real-life settings but the patterns and predictors of use of different LAI formulations, and the effect of introducing new LAI drugs has drawn even less attention. The aim of this study was to analyze the antipsychotic LAI prescription patterns during a 3-years follow-up period in Murcia, Spain, and to identify predictors of medication changes associated to the use of LAIs.

**Methods:** we designed a non-interventional, naturalistic and retrospective study using all patients from the health department corresponding to the Reina Sofía University Hospital, Murcia, Spain who were diagnosed of schizophrenia and treated with long-acting injectables (LAIs) between the years 2015–2017. Data, pertaining to patients older than 18 years old, were extracted from electronic medical records. Demographic variables, the use of LAIs, the rates for antipsychotic polypharmacy, combined use of different antipsychotic classes with a special focus on atypical antipsychotics, and psychotropic polypharmacy using benzodiazepines, mood stabilizers, and other relevant drugs were identified, as well as, the number of admissions and suicidal attempts.

**Results:** No statistical differences were observed regarding demographic variables between LAIs users. However, the number of admissions to a hospital or acute relapses where significantly lower (p<0.05) in the group treated with paliperidone LAI versus the others LAIs. In addition, our results showed that paliperidone, aripiprazole, zuclopentixol and riperidone LAIs are associated with 15.85; 47.86; 25 and 49.25 mg/day of diazepam, respectively. One way ANOVA showed that paliperidone LAI was associated with a significant (p<0.01) less intake of benzodiazepines when compared to others LAIs. ANOVA failed to show differences when the dose of oral anti-psychotics co-administered with LAIS were compared.

**Discussion:** Polypharmacy is the most common pattern of antipsychotic use in this region of Spain. Use of atypical antipsychotics is extensive. Most patients receive psychiatric co-medications such as anxiolytics. Polypharmacy is associated with the use of aripiprazole or zuclopentixol and these groups of patients showed more admissions to a hospital. In contrast, paliperidone LAI group showed less admissions and less use of benzodiazepines. Our results indicate that paliperidone LAI could improve the long-term management of patients with schizophrenia.

### T232. EFFICACY AND SAFETY OF ANTIDEPRESSANT AUGMENTATION OF ANTIPSYCHOTICS IN SCHIZOPHRENIA

Britta Galling*[,1], Christoph Correll[2]
*[1]The Zucker Hillside Hospital; [2]The Zucker Hillside Hospital, Hofstra North Shore LIJ School of Medicine*

**Background:** Although antidepressants are commonly used in patients with schizophrenia, meta-analytic guidance on the efficacy and safety of antidepressant augmentation evaluated as a single clinical strategy in patients with insufficient response to antipsychotic monotherapy is missing.
**Methods:** Systematic literature search of PubMed/MEDLINE/PsycINFO/Cochrane Library without language restrictions from database inception until 07/20/2015 for randomized, double-blind, efficacy-focused trials comparing adjunctive antidepressants vs placebo to antipsychotics in schizophrenia. Random effects meta-analysis in accordance with the Preferred Reporting Items for Systematic Reviews and Meta-Analyses (PRISMA) standard to evaluate the efficacy and safety of antidepressant augmentation of antipsychotics in schizophrenia.
**Results:** In 48 studies (n=2192, duration=10.2 ± 7.6 weeks), antidepressant augmentation outperformed placebo regarding total symptom reduction (studies=28, n=1249, standardized mean difference (SMD)=-0.36, 95% confidence interval (CI)=-0.57, -0.15, p=0.001), driven by negative (studies=32; n=1384, SMD=-0.25, 95%CI=-0.44, -0.06, p=0.010), but not positive (p=0.284) or general (p=0.118) symptom reduction. Significant improvements extended to core negative symptoms avolition/apathy (SMD=-0.54, 95%CI=-0.84, -0.24, p<0.001) and anhedonia/asociality (studies=8, n=284, SMD=-0.50, 95%CI=-0.90, -0.10, p=0.013). In predefined subgroup-analyses, superiority regarding negative symptoms was confirmed in studies augmenting first-generation antipsychotics (FGAs) (studies=10, n=433, SMD=-0.42, 95%CI=-0.76, -0.08, p=0.016), but not second-generation antipsychotics (studies=13, n=452, p=0.385). Uniquely, superiority in total symptom reduction by noradrenergic-and-specific-serotonergic-antidepressants (SMD=-0.72, 95%CI=-1.24, -0.20, p=0.007) was not driven by negative (p=0.467), but by positive symptom reduction (SMD=-0.43, 95%CI=-0.78, -0.09, p=0.013). Antidepressants did not improve depressive symptoms more than placebo (studies=24, n=1111, p=0.207). Bupropion was superior to placebo regarding smoking cessation (studies=7, n=327, RR=2.75, 95%CI=1.60–4.72, p<0.001, number-needed-to-treat (NNT)=6). Except for more dry mouth (RR=1.57, 95%CI=1.04–2.36, p=0.03) and dizziness (RR=2.01, 95%CI=1.06–3.82, p=0.032), antidepressants were not associated with more adverse effects or all-cause/specific-cause discontinuation than placebo.
**Discussion:** For schizophrenia patients on stable antipsychotic treatment adjunctive antidepressants are effective for total and particularly negative symptom reduction, and bupropion helps smoking cessation. However, effects are small-to-medium, differ across individual antidepressants, and negative symptom improvement seems restricted to the augmentation of FGAs.

### T233. DEFINING TREATMENT RESPONSE AND RESISTANCE IN FIRST EPISODE SCHIZOPHRENIA

Kara Dempster*[,1], Lena Palaniyappan[2], Ross Norman[3]
*[1]London Health Sciences Center; [2]University of Western Ontario; [3]Western University*

**Background:** Although a substantial proportion of individuals with schizophrenia fail to respond to first-line dopaminergic blocking medications and are Treatment Resistant (TR), identifying these subjects prospectively remains challenging. Though clinically defined only after multiple treatment trials, TR is suspected to reflect a stable neurobiological phenomenon that can be identified even at the first episode of schizophrenia (FES). Establishing clear expectations for symptom improvement following antipsychotic initiation would facilitate development of objective thresholds for determining lack of efficacy. The Treatment Response and Resistance in Psychosis (TRIPP; Howes et al, 2017) working group has recently published consensus guidelines which define lack of response as a <20% improvement in psychotic symptoms. However, given that most patients with FES respond robustly to antipsychotics (Robinson et al, 1999), FES specific criteria for prospective identification of TR are warranted. We examined two symptom improvement thresholds across positive and negative symptom domains at 6 months in FES to investigate poor response (PR) as a proxy measure of early TR. We then examined the baseline/early clinical features that best prospectively predicted PR+ status. Given the estimated prevalence of TR is approximately 33%, we hypothesized that a comparable number (ie, 1/3rd) of individuals with FES would meet PR criteria using less a 50% response threshold, rather than a more stringent 20% threshold for determining symptomatic response. Furthermore, we hypothesized that very early lack of response would be associated with PR at 6 months.
**Methods:** Data from a longitudinal naturalistic cohort study of patients treated at the Prevention and Early Intervention Program for Psychosis (PEPP) in London, Ontario, Canada collected between 2002 and 2007 were used for this analysis. Only individuals meeting criteria for a primary psychotic disorder that were medication compliant were included. Positive and negative symptoms of psychosis were assessed using the SAPS (Andreasen, 1983) and SANS (Andreasen, 1984) at baseline, and at months 1, 2, 3, and 6. Treatment was administered in a naturalistic setting and followed clinical guidelines for the treatment of FES.
**Results:** Applying a 20% and 50% symptom improvement threshold for defining PR resulted in 2.2% and 14% rates for positive symptom PR, 33% and 60.9% rates of negative symptom PR, and 12% and 37.0% rates of total symptom PR at 6 months. Logistic regression analyses demonstrated that poor premorbid functioning, having a longer duration of untreated illness, and limited overall treatment response at months one and two were significantly associated with being PR+ (<50% improvement in total symptoms) at 6 months.
**Discussion:** This is the first study to our knowledge to investigate the symptom response thresholds suggested by TRIPP in FES. Our results suggest that including negative symptoms (either alone, with a 20% criteria for improvement, or in addition to positive symptoms, with a 50% improvement threshold) is necessary to identify the expected proportion of TR subjects prospectively in a FES sample. We propose that failing to achieve at least a 50% improvement in total symptoms, or at least 20% change in negative symptom severity by 6 months may be an early clinical indicator of eventual TR. On an optimistic note, we speculate that it may be possible to determine clozapine-eligibility as early as 6 months by using this approach. However, further studies are warranted to investigate the utility of this symptom threshold criteria in larger samples of patients with FES.

### T234. BREAKTHROUGH ON ANTIPSYCHOTIC MAINTENANCE MEDICATION: A STUDY-LEVEL META-ANALYSIS AND META-REGRESSION ANALYSIS

Jose Rubio*[,1], Kinza Ahmed[1], Christoph Correll[2], Katsuhiko Hagi[3], Taishiro KIshimoto[1], John Kane[1]
*[1]Northwell Health; [2]The Zucker Hillside Hospital; [3]Dainippon Sumitomo Pharma*

**Background:** The study of psychotic relapse despite ongoing antipsychotic maintenance treatment can serve as a paradigm to study the intrinsic efficacy of antipsychotic drugs, their mechanism of action, the pathophysiology of psychosis, and potentially the evolution of treatment-resistant schizophrenia. This phenotype is referred to as Breakthrough on Antipsychotic Maintenance Medication (BAMM). Despite the fact that the efficacy of long-acting injectable (LAI) treatment (where adherence can be confirmed) has been researched for decades, the study of individuals breaking through LAI treatment has received little attention. A reanalysis of the literature on studies of LAI formulations can serve as an initial approach to understand BAMM in a paradigm not confounded by non-adherence.
**Methods:** We re-analyzed data from 3 meta-analyses of randomized controlled trials (RCTs), mirror image, and cohort studies including LAIs. We extracted the data of study-defined relapse from each LAI arm, which was our outcome. We also extracted data for various covariates regarding the study design, participant, and LAI treatment characteristics. We conducted a random-effects study-level meta-analysis, and a meta-regression analysis of covariates of interest. We examined the risk of publication bias using the fail-safe test.
**Results:** In a pooled analysis of studies from the 3 separate meta-analyses, we identified a total of 51 LAI treatment arms, including 13,071 individuals (mean age=38.8 ± 6.2, 60.5 ± 11.9% male, illness duration=12.1 ± 5.7 years, mean total PANSS score=74.3 ± 7.8). The mean planned follow-up duration was 71.5 ± 37.0 (range=24–154) weeks, and the defined daily dose (DDD) of LAI treatment was 1.1. The pooled weighted relapse rate in a random effects model was 28% (95% CI=24–31%), being 22% (95% CI=15–30%) in RCTs (mean duration= 66.2 wks), 33.0% (95% CI=24–43%) in mirror image studies (mean duration= 55.4 wks), and 30% (95% CI=26–34%) in naturalistic cohort studies (mean duration= 79.4 wks). In a meta-regression analysis, RCT design (p=0.04), and industry sponsorship (p=0.03) were associated with lower relapse rates on LAI treatment, whereas studies conducted in Europe (p=0.03) or North America (p<0.01), and longer duration of follow-up (p<0.01) were associated with greater relapse rates. In the fail-safe test, we found that we would need 4,629 studies to bring the p Value>α, suggesting low risk of bias.
**Discussion:** About one in four individuals receiving LAI treatment relapsed according to study definitions, suggesting that this is a relatively common phenomenon, even during assured medication adherence. At the study level, no major differences were observed in terms of sociodemographic characteristics, type of drugs, mean drug daily dose, or baseline severity at study entry in relation to relapse rate in individuals treated with LAIs. The relapse rate though was lower in RCTs, which could reflect a difference in the patient population participating and/or the nature of observation in LAI RCTs. These results suggest either that the examined study design, illness severity and treatment related covariates are limited to identify potential mediators and moderators of BAMM, or that the data about the covariates at the study level was not precise enough to detect clinical characteristics associated with BAMM. Given the relative frequency of BAMM, future research should explore potential mediators and moderators of the failure to maintain response to antipsychotic treatment even during periods of assured adherence. Neurobiological markers, which may be more closely related to the pathophysiology of BAMM, and individual participant data meta-analyses, which can identify clinical predictors with greater precision, should be the next steps.

### T235. SELF ASSESSMENT OF SOCIAL COGNITIVE ABILITY IN SCHIZOPHRENIA: ASSOCIATION WITH SOCIAL COGNITIVE TEST PERFORMANCE, INFORMANT ASSESSMENTS OF SOCIAL COGNITIVE ABILITY, AND EVERYDAY OUTCOMES

Philip Harvey*[,1], Amy Pinkham[2], David Penn[3]
*[1]Leonard M. Miller School of Medicine, University of Miami; [2]University of Texas at Dallas; [3]University of North Carolina*

**Background:** Impairments in self-assessment are commonly found in people with schizophrenia and impairments in introspective accuracy (IA) predict impaired functional outcome. previous studies have suggested mis-estimation of cognitive and functional skills predict impairment in everyday functioning at least as much as ability scores. In this study, we examined self-assessment of social cognitive ability and related these self-assessments to assessments of social cognition from informants, to performance on tests of social cognitive ability, and to everyday outcomes. The difference between self-reported social cognitive abilities and informant ratings was our measure of IA.
**Methods:** People with schizophrenia (n=135) performed 8 tests of social cognitive abilities. They also rated their social cognitive abilities on the Observable Social Cognition Rating Scale (OSCARs). High contact informants also rated social cognitive ability and everyday outcomes, while unaware of the patients' other scores. Social competence was also measured with a performance-based assessment and clinical ratings of negative symptoms were also performed.
**Results:** Patient reports of their social cognitive abilities were uncorrelated with performance on social cognitive tests and with three of the four domains of everyday functional outcomes. IA, in specific overestimation of performance compared to informant ratings, predicted impaired everyday functioning across all four functional domains. IA scores predicted functional outcomes even when the influences of social cognitive performance, social competence, and negative symptoms were considered in regression models. Thus, self-assessment of social cognition had a relatively specific impact social outcomes.
**Discussion:** Mis-estimation of social cognitive ability was a more important predictor of social and nonsocial outcomes in schizophrenia than performance on social cognitive tests. These results suggest that consideration of IA is critical when attempting to assess causes of everyday disability and when implementing interventions aimed at disability reduction.

### T236. TECHNOLOGY LITERACY AND ENGAGEMENT IN PEOPLE WITH SCHIZOPHRENIA

Kwok Tung Gordon Wong[1], Daniel King[2], Ryan Balzan[3], Dennis Liu[4], Cherrie Galletly*[,2]
*[1]Northern Adelaide Local Health Network; [2]University of Adelaide; [3]Flinders University; [4]Northern Adelaide Local Health Network, University of Adelaide*

**Background:** Information and interventions for mental illness are increasingly being provided on-line. There is an expectation that citizens have access to the internet and are competent in using technology. People with schizophrenia are often excluded from social engagement, have cognitive impairment and have very limited income; all of which may reduce their use of technology.
This project aimed to assess the use of technology and the internet in people with schizophrenia or schizoaffective disorder, living in the community.
**Methods:** Face-to-face structured interviews were used to evaluate technology literacy, attitudes towards technology, and access and engagement with technology in 50 people with schizophrenia or schizoaffective disorder aged 18–65 years, living in the community.
**Results:** About half of the study population had access to a computer, and half had access to the internet at home. Participants' most common uses of technology were voice-calling, messaging, surfing the internet and accessing Facebook. The use of more advanced functions of technology (calendar, banking, news, health information) were rare. The general attitude among participants was that technology was not a significant part of their lives.
**Discussion:** Technology literacy and internet access were limited in this population. This needs to be addressed before the on-line delivery of educational information, service information and e-health interventions can be widely utilised in people with schizophrenia.

### T237. USER EXPERIENCE TOWARDS AN INTEGRAL INTERVENTION MODEL BASED ON M-HEALTH SOLUTION FOR PATIENTS WITH TREATMENT-RESISTANT SCHIZOPHRENIA (M-RESIST): QUALITATIVE INFORMATION FROM PATIENTS, CAREGIVERS AND CLINICIANS IN A PILOT STUDY

Silvia Marcó-García[1], Elena Huerta-Ramos[1], Susana Ochoa *[,1], Eva Grasa[2], Anna Alonso[3], Erika Jääskeläinen[4], Elisenda Reixach[5], Jesús Berdún[5], Charlotte Jewel[6], m-RESIST GROUP Iluminada Corripio[7], Judith Usall[8]

[1]Parc Sanitari Sant Joan de Deu; [2]IIB-Hospital Santa Creu I Sant Pau; [3]Hospital Sant Pau; [4]University of Oulu; [5]Fundació TicSalut; [6]IMEC; [7]Hospital Santa Creu i St. Pau, CIBERSAM; [8]Parc Sanitari San Joan de Dèu, CIBERSAM

**Background:** In the European Union approximately 5 million people suffer from psychotic disorders. Patients with schizophrenia make up the largest subgroup of these, and between 30–50% of them are considered resistant to treatment. Despite the proven potential of m-health solutions, there remains a lack of technological solutions in the treatment of patients with this disease. To improve the quality of care of these outpatients, an m-health solution termed Mobile Therapeutic Attention for Patients with Treatment Resistant Schizophrenia (m-RESIST) has been created in European Union and implemented in three countries (Spain, Hungary and Israel). m-RESIST is an innovative project aimed to empower patients with Treatment Resistant Schizophrenia, which integrates pharmacological and psychosocial approaches, develops knowledge of the illness using predictive models, and includes the following m-Health tools: a Dashboard, a Smartwatch and a Smartphone. Prior to the implementation in the healthcare reality, the solution has been tested in pilot groups to assess the acceptability, usability and satisfaction of all m-RESIST components in each country. In addition to online and onsite visits, this phase has included an anonymous online questionnaire, with the aim of capturing more consistently the opinion of participants in their experience with m-RESIST. We summarize their opinions about services and devices included in the solution, as well as the improvement proposals of each group.
**Methods:** During three months (from August to October), a case manager from Spain sent out an interval question to the Spanish participants via m-RESIST Dashboard, in order to collect information about the users experience with the system. It was administered weekly on different days and at different times, being anonymous for both parts. We have obtained qualitative information from nine patients, one caregiver and two clinicians.
**Results:** Patients consider m-RESIST a useful tool, in terms of immediacy of contact with clinicians, improvement of disease awareness, better follow-up of their disease, less-worries from caregivers and feeling protected by having a team with whom they can share their concerns. As cons, patients have a strong feeling of being observed and with too much repetitive questionnaires to answer. They consider a bit difficult to use the devices, with several errors in its operation. They do not like to carrying the smartwatch and to check the battery of the devices. Also, the program is not available on weekends, which leads to a feeling of being somehow disregarded. For patients, this solution should also include the possibility of changing programmed location when on vacations and it should not be a substitute for traditional treatment. Regarding caregivers, m-RESIST is considered as a good tool to have in their daily lives, because it helps in terms of disease improvement, to have a better follow-up about pharmacological issues and symptoms, and to feel secure knowing there is a support for both patient and caregiver. No cons were reported. For clinicians, m-RESIST is a system with high potential, being easy, intuitive and useful, specially to share psychoeducational content with patients and to improve communication with them. However, several technological problems must be solved in the future, there still provide a poor patient monitoring and much more time is needed than regarding the traditional treatment.
**Discussion:** The three user groups consider m-RESIST as a useful tool, with pros and cons being described regarding their specific needs and provided proposals for improvement.

### T238. THE ASSOCIATION OF PSYCHOSOCIAL FUNCTIONING WITH BRAIN VOLUME IN THE EARLY STAGES OF (PSYCHOTIC) ILLNESS

Renate Reniers*[,1], Rachel Upthegrove[1], Kareen Heinze[1], Katharine Chisholm[1], Ashleigh Lin[2], Beth Charters[1], Hannah Ball[1], Graham Hall[3], Stephen Wood[4]
[1]University of Birmingham; [2]Telethon Kids Institute, The University of Western Australia; [3]University of Manchester; [4]Orygen, the National Centre of Excellence in Youth Mental Health

**Background:** In recent years, psychosocial functioning has received a lot of attention with discussions around its importance in terms of early identification of illness, prediction of outcome, and targeting of treatment. Regardless of diagnostic outcome, both groups of individuals at ultra-high risk for psychosis (UHR) and those with a first episode of psychosis (FEP) show a wide range of functional outcomes. In light of these clinical outcomes, effort has been made to identify neuroanatomical markers for functioning and functional outcome independent of diagnostic status. The present study aimed to increase insight into the association of brain volume with psychosocial functioning in the early stages of (psychotic) illness by investigating the association between grey matter volume and current levels of social and occupational functioning (SOFAS) in healthy individuals, those with emerging mental health problems (EMH), UHR individuals, and those with a FEP.
**Methods:** Twenty nine healthy controls (12M:17F; mean age 20.97), 27 EMH individuals (6M:21F; mean age 21.24), 31 UHR individuals (14M:17F; mean age 24.40), and 31 FEP individuals (25M:6F; mean age 25.24) were recruited from mental health services, through posters, social media and opportunity sampling, in the wider area of Birmingham, UK. They underwent magnetic resonance imaging at the Birmingham University Imaging Centre and completed the Social and Occupational Functioning Assessment Scale (SOFAS: healthy controls mean 84.41, range 70–95; EMH mean 63.93, range 32–89; UHR mean 54.68, range 35–80; FEP SOFAS 56.65, range 21–95). Images were analysed using the CAT12 toolbox in SPM12. Grey matter volumes were examined controlling for age, gender and total intracranial volume.
**Results:** Compared to healthy controls, EMH individuals displayed a pattern of grey matter volume reduction in association with reduced functioning scores in medial prefrontal and cingulate areas. The areas spanning volumetric differences between the two groups in their association with SOFAS scores were similar to those identified in previous work investigating the association between brain volume and functional outcome in UHR individuals (Reniers et al., 2016, doi:10.1093/;schbul/sbw086) but were more widespread and disperse. Similar areas of association were observed in UHR and FEP individuals compared to healthy controls but here the pattern was much more specific and more pronounced in the FEP group than the UHR group in the comparison with healthy controls.
**Discussion:** The present findings provide novel evidence that while those in the early stages of psychotic illness present a unified pattern of association between psychosocial functioning scores and grey matter volume, those with EMH present with a more pronounced but more dispersed pattern, possibly reflecting a more disperse diagnostic outcome. This indicates specificity with psychotic illness in the association between psychosocial functioning and brain volume and suggests importance concerning our ability to predict outcome and target interventions. In addition, it provides support for the recent focus on functioning in addition to distinct diagnostic categories.

### T239. SINGLE-SUBJECT PREDICTION OF FUNCTIONAL OUTCOMES IN CLINICAL HIGH RISK SUBJECTS USING CLINICAL DATA

Marlene Rosen*[,1], Nathalie Kaiser[1], Theresa Haidl[1], Mauro Seves[1], Frauke Schultze-Lutter[2], Stefan Borgwardt[3], Paolo Brambilla[4], Eva Meisenzahl[2], Christos Pantelis[5], Stephan Ruhrmann[1], Raimo Salokangas[6], Rachel Upthegrove[7], Stephen Wood[8], Nikolaos Koutsouleris[9], The PRONIA Group
[1]University of Cologne; [2]Heinrich-Heine University Düsseldorf; [3]UPK, University of Basel; [4]University of Milan; [5]Melbourne Neuropsychiatry Centre, The University of Melbourne; [6]University of Turku; [7]University of Birmingham; [8]Orygen, the National

*Centre of Excellence in Youth Mental Health; [9]Ludwig Maximilian University*

**Background:** Psychotic disorders are associated with serious deterioration in functioning even before the first psychotic episode. Also on clinical high risk (CHR) states of developing a first psychotic episode, several studies reported a decreased global functioning. In a considerable proportion of CHR individuals, functional deterioration remains even after (transient) remission of symptomatic risk indicators. Furthermore, deficits in functioning cause immense costs for the health care system and are often more debilitating for individuals than other symptoms. However in the past, CHR research has mostly focused on clinical outcomes like transition and therefore, functioning in CHR patients is under-investigated. The current study aims at predicting functioning at a single subject level applying multi pattern recognition to clinical data for the first time.

**Methods:** PRONIA ('Personalized Prognostic Tools for Early Psychosis Management') is a prospective collaboration project funded by the European Union under the 7th Framework Programme (grant agreement n° 602152). Considering a broad set of variables (sMRI, rsMRI, DTI, psychopathological, life event related and sociobiographic data, neurocognition, genomics and other blood derived parameters) as well as advanced statistical methods, PRONIA aims at developing an innovative multivariate prognostic tool enabling an individualized prediction of illness trajectories and outcome. Seven university centers in five European countries and in Australia (Munich, Basel, Birmingham, Cologne, Melbourne, Milan/Udine, Turku) participate in the evaluation of three clinical groups (subjects clinically at high risk of developing a psychosis [CHR], patients with a recent onset psychosis [ROP] and patients with a recent onset depression [ROD]) as well as healthy controls.

In the current study, we analysed data of 114 CHR patients. Functioning was measured by the 'Global Functioning: Social and Role' Scales (GF S/R). Features were derived from the large pool of clinical data that were assessed in PRONIA including questionnaires measuring CHR criteria as well as psychopathology, family history of psychotic disorders or treatment and various self-rating scales. Feature Elimination method of a strict Wrapper was used to identify most predictive variables from the multitude of clinical data included into the analysis.

**Results:** Balanced Accuracy of predicting social functioning in CHR patients was acceptable (pooled cross-validation: BAC = 74.3%, Sens = 72.8%, Spec = 60.3%; leave-site-out cross-validation: BAC = 69.9%, Sens = 84.3%, Spec = 55.6%). In contrast, applying the strict wrapper model revealed worse prediction performance for role functioning. Which might indicate that predicting level of role functioning requires more information than social functioning. As expected, prior functioning levels were identified as main predictive factor but also distinct protective and risk factors were selected into the prediction models.

**Discussion:** Identifying single predictive variables is in purpose of a much more efficient prognostic process. Moreover, understanding the mechanisms underlying functional decline and its illness related pattern might enable an improved definition of targets for intervention. Future research should aim at further maximisation of prediction accuracy and cross-centre generalisation capacity. In addition, other functioning outcomes as well as clinical outcomes need to be focused on.

### T240. CAREGIVER BURDEN OF OUTPATIENTS WITH SCHIZOPHRENIA IN UNIVERSITY CLINIC IN SAO PAULO, BRAZIL

Elaine Di Sarno*[,1], Isabel Cristina Napolitano[1], Mario Rodrigues Louzã Neto[1]

[1]*Institute of Psychiatric, Hospital Clinics, IPQ-HC-FMUSP*

**Background:** The impact of schizophrenia on the family is complex[1] and affects not only the patient, but his/her whole family. The adverse consequences involve physical, emotional, social, and economic restrictions and imply an objective and subjective burden for caregivers.[2]

This study aims to evaluate the burden of caregiving in a sample of outpatients with schizophrenia, in Sao Paulo, Brazil.

**Methods:** Cross-sectional observational study. Patients with diagnosis of schizophrenia (DSM-5), 18–50 years, both sexes, and a relative/caregiver, both sexes, aged 18 to 70 years, living in contact with the patient at least 20 hours/week. Measures included patients and caregivers' demographic variables. Family burden was evaluated using the Brazilian version of the Family Burden Interview Schedule (FBIS-BR), a semi-structured interview, considering objective and/or subjective burden, distributed in five subscales (assistance to the patient in daily life [objective and subjective burden]; supervision of patients' problematic behaviors [objective and subjective burden]; financial burden; impact on family routine [objective and subjective]; worries about the patients' present and future life [subjective]). The questions of FBIS-BR refer to the last thirty days prior to the interview, except for one item, which evaluates the overload during the last year. The objective burden is assessed in a Likert scale (1 = never to 5 = every day), and subjective burden, in Likert scale (1 = not at all to 4 = very much).

**Results:** Patients: n= 56: 69.6% male; mean age: 36.04 ± 9.62 years; 89.3% single; duration of disease: 15.07 ± 9.83 years; number of hospitalizations: 2.95 ± 3.76; 76.8% with elementary or middle school; 66.1% without social security.

Caregivers n=56: 76.8% female; mean age: 56.30 ± 11.46 years; 57.1% mothers; 10.7 % fathers; 23.2% siblings; 57.1% married; 62.5% with elementary or middle school; in contact with the patient 81.71 ± 37.04 hours/week, most of them live with the patient; 53.6% without social security.

The mean total score of the objective and subjective burden was 2.43 ± 0.57 and 2.14 ± 0.53, respectively.

In the analysis of subscales the assistance to the patient in daily life (objective) was 3.26 ± 0.71 and it subjective aspect was 1.82 ± 0.89; supervision of patients' problematic behaviors (objective) was 1.80 ± 0.53 and it subjective aspect was 0.95 ± 0.71. The impact on family routine (objective and subjective) was 2.21 ± 0.93 and worries about the patients' present and future life (subjective) 3.64 ± 0.61; financial burden: 3.39 ± 1.54. The mean total family income was US$1008.49 ± $526.02.

There were no significant differences in FBIS-BR scores between male and female patients, except for "supervision of patients' problematic behaviors", both objective (p=.013, uncorrected for multiple comparisons) and subjective (p= .032. uncorrected for multiple comparisons) aspects, in which female patients were responsible for a higher burden for their caregivers. Regarding the family's perception of the financial burden in the last year, 57.3% considered their spending on patients as frequent, almost always or always heavy, in the same period.

**Discussion:** Our results are consistent with the study of Barroso et al. (2007), according to which providing care to psychiatric patients generates the feeling of overload, since the caregiver undergoes changes in his / her routine of life, failing to satisfy his / her needs to meet the needs of the patient. The burden affects almost equally male and female patients.

**References:**

1. Caqueo-Urízar A.,Castillo M., Giráldez S. et al.. Psicothema 2014: 26(2): 235–243

2. Barroso et al. Rev. Psiq. Clín 34 (6); 270–277

### T241. INTERPERSONAL COGNITIVE RIGIDITY AFFECTS SOCIAL FUNCTIONING IN PSYCHOSIS MORE THAN THEORY OF MIND: A STUDY WITH THE REPERTORY GRID TECHNIQUE

Helena García-Mieres*[,1], Susana Ochoa[2], Victoria Furlan[1], Raquel Lopez Carrilero[2], Anna Villaplana[2], Regina Vila-Badia[3], Eva Grasa[4], Ana Barajas[5], Esther Pousa[6], Guillem Feixas[1]

[1]*Universitat de Barcelona;* [2]*Parc Sanitari Sant Joan de Déu;* [3]*Fundació Sant Joan de Déu;* [4]*IIB-Hospital Santa Creu I Sant Pau;* [5]*Centre de Salut Mental Les Corts;* [6]*Hospital del Mar*

**Background:** Social functioning impairment is one of the core features for schizophrenia diagnosis and are also present in other psychotic spectrum disorders, being determinant for disability. This impairment has multiple domains, which are linked but separate. Previous research has shown that social functioning is multiply determined by neurocognition, social cognition and symptoms, being social cognition the domain that accounts for more of the variance in daily functioning. However, cognitive rigidity in interpersonal perception has received less attention and much variance remains unexplained. The aim of this study was to test the role of interpersonal cognitive rigidity, as measured with the Repertory Grid Technique (RGT) in social functioning in psychosis.
**Methods:** Sample of 40 out-patients with a psychotic spectrum diagnosis from the network of mental health services of Parc Sanitari Sant Joan de Déu (Barcelona, Spain). Cross-sectional study, assessment was carried out by a predoctoral researcher (GMH), using a sociodemographic questionnaire, the Social Functioning Scale (SFS), the Hinting Task (Theory of Mind, ToM), the Beck Cognitive Insight Scale (BCIS), and the RGT (to measure interpersonal cognitive rigidity, two indices were selected: Percentage of Variance Accounted for the First Factor, PVAFF, and Polarization). Pearson correlations and multiple regression analysis were performed.
**Results:** Results showed that social engagement/withdrawal was explained by PVAFF, accounting for 16% of the variance. Independence-competence was explained by polarization, explaining 14.6% of the variance and by sex, which accounted for 11.1% of the variance. Independence-performance was explained by theory of mind, explaining 22.5% of variance. Employment/occupation was explained by years of illness accounting for 21.6% of variance, and by polarization (beta=-0.318, p=0.026) which explained 10% of variance. Finally, the total score of the SFS was explained by polarization, explaining 14.4% of variance, and sex, which accounted for 12.6% of variance. For prosocial activities and interpersonal communication, none of the variables entered for the linear regression analysis.
**Discussion:** Despite ToM and cognitive insight are common variables reported in the research literature, in our study the cognitive rigidity measures of the RGT, based on the patients' own terms (personal constructs) in rating their significant others, were better predictors of social functioning. These findings support the importance and utility of an idiographic instrument like the RGT to investigate cognitive processes related to social perception and their impact on functioning. Regarding PVAFF, a higher tendency to perceive the interpersonal world from a unidimensional manner predicted a worse outcome in social relationships/withdrawal. Regarding interpersonal polarized thinking, it was the best cognitive predictor of social functioning measures. Our results suggest that a dichotomous thinking style in interpersonal perception might also be relevant for elucidating the dysfunction in social adjustment domains. These findings are still preliminary, and form part of an ongoing study.

### T242. DEVELOPMENT OF SELF-STIGMA INVENTORY FOR PATIENTS WITH SCHIZOPHRENIA (SSI-P): RELIABILITY AND VALIDITY STUDY IN TURKEY

Mustafa Yildiz[1], Fatma Kiras[1], Aysel İncedere[1],
Fatma Betül Abut*[,1]
[1]*Kocaeli Universitesi*

**Background:** Stigmatization is defined as discrimination and loss of social status which is triggered by negative stereotypes related to certain human characteristics such as having mental illnesses. Most of the people with mental illnesses are aware of the stigmatization in society and some of them internalize this social stigma by stigmatizing themselves. Schizophrenia is known as the most stigmatized mental illness by the society, healthcare professionals, and the patients themselves. In Turkey, there is no scale that evaluates the self-stigmatization of people with schizophrenia. The purpose of this study was to develop a culturally-sensitive and easy-to-use instrument to measure self-stigma of the people with schizophrenia.
**Methods:** After examining the existing stigma and self-stigma scales for people with mental illnesses, 25-item self-stigma inventory was formed. Focus group interviews were conducted with 20 patients with schizophrenia and the items of the newly developed form were reviewed and rephrased into more comprehensible statements for the patients. The pilot study was conducted with a sample of 15 patients with schizophrenia and the inventory was finalized as 19-item self-stigma inventory for the patients. One hundred and sixty-two outpatients with schizophrenia or schizoaffective disorder were given sociodemographic form, Self-Stigma Inventory (SSI-P), Beck Depression Inventory (BDI), Internalized Stigma of Mental Illness (ISMI) Scale, Rosenberg Self-Esteem Scale (RSES), Beck Hopelessness Scale (BHS), Positive and Negative Syndrome Scale (PANSS), Clinical Global Impression-Severity (CGI-S), and Global Assessment of Functioning (GAF). For reliability analyses; split-half reliability, internal consistency coefficient, and item-total correlation were assessed. For validity analyses; explanatory factor analysis and convergent validity were conducted.
**Results:** The sample of the study was 162 outpatients. Seventy-seven percent of the participants were males, 70% were single, mean age was 37, and level of education was 10 years. Cronbach's alpha coefficient for SSI-P total score was 0.93, and Cronbach's alpha scores for SSI-P subscales were between 0.60 and 0.91. Split-half reliability of the inventory was 0.90. For factor analysis, Kaiser-Meyer-Olkin value was found as 0.913 and Barlett test was significant (p<0.001). In explanatory factor analysis, three factors (perceived incompetency, internalized stereotypes and social withdrawal, and concealment of the illness) were defined and 63% of the variance was explained by the factors. Two items were removed from the questionnaire as they had lower item value than 0.40. In the final form, perceived incompetency factor consisted of 8 items, internalized stereotypes and social withdrawal factor had 7 items, and concealment of the illness factor had 2 items. SSI-P total score was found significantly and positively correlated with PANSS negative symptoms subscale (r=0.19, p<0.05), Beck Depression Inventory (r=0.53, p<0.001), Beck Hopelessness Scale (r=0.40, p<0.001), ISMI total score (r=0.73, p<0.001), and Rosenberg Self-Esteem Scale (r=-0.59, p<0.001).
**Discussion:** The results of the current study show that SSI-P is a reliable and valid instrument for assessing the self-stigmatization of the patients with schizophrenia. It consists of 17 items that are comprehensible and user-friendly for the patients. The scale could be considered as an important instrument in psychotherapy practices and for research purposes.

### T243. RESOURCE GROUP-ACT: RELATIVES' PERSPECTIVES

Nils Sjöström*[,1], Mats Ewertzon[2], Bente Weimand[3],
Anita Johansson[4], Zophia Mellgren[5], Ola Johansson[1],
Jane Ek-Persson[4], Margda Waern[6]
[1]*Sahlgrenska University Hospital;* [2]*Ersta Sköndal Bräcke University College;* [3]*Akershus University Hospital;* [4]*Skaraborgs Hospital;* [5]*Dish Association of Local Authorities and Regions;* [6]*Sahlgrenska University Hospital, University of Gothenburg*

**Background:** Relatives often take on great responsibility for helping the patient in his or her daily life, and many relatives experience lack of support from health care services. Cooperation with relatives is a central component in Resource groups Assertive Community Treatment (R-ACT). This person-centered model has been found to decrease symptoms, increase level of function, and strengthen well-being in patients with psychotic disorders. However, little is known about relatives' experiences of the model.
**Aim:** To examine relatives experiences of R-ACT. Further, to compare relatives' experiences of treatment and feelings of being alienated from care services in relatives' with and without experience of R-ACT. We hypothesize higher levels of family burden, and family stigma and lower quality of life in relatives without R-ACT.
**Design:** Cross-sectional study focusing on relatives of persons with psychotic disorders during the period of October 1, 2017 – May 31, 2018.
**Participants:** Relatives of next of kin suffering from psychotic disorders, treated in health care clinics with and without R-ACT in Västra Götaland County in Sweden.
**Measurements:** The postal questionnaire includes four self-reported instruments: the Family Involvement and Alienation Questionnaire, the Burden Inventory for Relatives of Persons Psychotic Disturbances, the Inventory of Stigmatizing Experiences (family version), and RAND-36.
**Results:** Recruitment is ongoing. Preliminary results will be presented at the conference.
**Discussion:** Increased knowledge about relatives' experiences of psychosis care can inform the development of R-ACT, a care model that focuses on participation of both patients and their relatives.

### T244. SELF-DEFINING MEMORIES PREDICT ENGAGEMENT IN STRUCTURED ACTIVITY IN FIRST EPISODE PSYCHOSIS

Abigail Wright*[,1], Geoff Davies[1], David Fowler[1], Kathryn Greenwood[1]
[1]*University of Sussex*

**Background:** Self-defining memories (SDM) are vivid personal events, related to important life memories and narrative identity. Self-defining memories reported by individuals with schizophrenia have been found to be less specific, more negative, and individuals extract less meaning from the memories compared to a healthy control group. Research in healthy control participants has demonstrated that self-defining memories (specific and integrated SDMs) may be predicted by neurocognition, associated with metacognition, the way one thinks about one's abilities, and linked to goal outcomes. Neurocognition and metacognition are known predictors of poor functional outcome in psychosis, and recently metacognition was demonstrated to mediate between neurocognition, functional capacity, and functional outcome in first episode psychosis (FEP) (Davies, Fowler and Greenwood 2017). Self-defining memories may also have a role in predicting poor functional outcome. However, previous studies have only assessed those with chronic schizophrenia, none have looked at the relationship to functional outcome or pattern of SDMs in First Episode Psychosis. This study aimed to investigate the pattern of SDMs in FEP and the independent contribution of self-defining memories to outcome.
**Methods:** This was a cross-sectional study involving a sample of 71 people with First Episode Psychosis who completed measures for neurocognition, metacognition (Metacognitive Assessment Interview and Beck's Cognitive Insight Scale), self-defining memories, functional capacity (UCSD Performance-Based Skills Assessment) and functional outcome (hours spent in structured activity per week) using Time-Use Survey (Fowler et al., 2009). Research has demonstrated time spent in structured activity is 63.5 hours in healthy non-clinical population, 25.2 hours in a First Episode Psychosis sample, and 19.7 hours in a psychosis sample with delayed recovery (Hodgekins et al., 2015). Data was compared to a matched healthy control sample. It was hypothesised that self-defining memories would be less specific, less integrated and more negative in First Episode Psychosis compared to healthy controls, and self-defining memories would mediate between neurocognition and functional outcome in a multiple mediation model.
**Results:** Self-defining memories reported by individuals with First Episode Psychosis were less specific, less integrated, and more negative, focused on relationships, failure and life threatening events, compared to matched healthy control group. Within the First Episode Psychosis sample, holding less specific memories was associated with engagement in significantly fewer hours of structured activity per week (14.9 hours for non-specific memories and 43.3 hours for specific memories), and this effect remained after controlling for neurocognition and metacognition. A multiple mediation model demonstrated that the specificity of SDMs mediated the relationship between neurocognition and functional outcome, independent of functional capacity and metacognition.
**Discussion:** This study demonstrated that the types of self-defining memories reported are different between First Episode Psychosis and healthy controls, and may play a key role in functioning. This study was able to demonstrate a significant difference between the individuals with FEP reporting a specific compared to a non-specific memory on hours spent in structured activity. In such that participants who provided a specific memory were likely to have a better functional outcome and able utilise their neurocognitive ability to participate in more activities. Given these results, self-defining memories could be considered as a key factor to be explored within current FEP interventions.

### T245. THE ROLE OF PROTECTIVE FACTORS IN THE FIRST-EPISODE PSYCHOSIS: PRELIMINARY RESULTS

Regina Vila-Badia*[,1], Anna Butjosa[2], Núria Del Cacho[1], Itziar Riera-López de Aguileta[1], Mar Alvárez[1], Marta Pardo[3], Marta Coromina[1], Núria Grases[1], Susana Ochoa[1], PROFEP Group[1], Judith Usall[4]
[1]*Parc Sanitari Sant Joan de Déu;* [2]*Parc Sanitari Sant Joan de Déu, University of Barcelona;* [3]*Hospital Sant Joan de Déu;* [4]*Parc Sanitari San Joan de Dèu, CIBERSAM*

**Background:** Currently, there is a great interest in stress since many diseases can be affected by stress, including psychotic disorders. Interpretation and capacity of the person to tackle situations of psychosocial stress and their recovery capacities are relevant factors in the prevention of psychotic disorders (López-Soler, 2008; N Pereda, 2009, 2010; Noemí Pereda, Guilera, Forns, & Gómez-Benito, 2009). Some of protector factors that have been studied are the following: Resilience (R), Coping Strategies (CS) and Social Support (SS). Furthermore, few studies have been performed with FEP population.
**Methods:** This research was part of a longitudinal observational study called 'PROFEP Group' in Catalonia. The patients belong to Mental Health Parc Sanitari Sant Joan de Déu (for adults) and Hospital Sant Joan de Déu (for children and adolescents) health care sector. Participants were FEP patients (N=15); males= 9, females= 6) and HC (N=19; males=6, females=13) between 14 and 42 years. We used the PANSS scale (positive, negative and general) to evaluate psychotic symptoms and DUKE (social support), EMA (coping strategies) and CD-RISC-17 (resilience) scales to evaluate protective factors.
**Results:** FEP patients showed worse resilience ($p<0.05$), less social support ($p<0.05$) and more avoidance coping strategies ($p<0.05$) than HC. On the other hand, in FEP patients, some protective factors correlate with the symptomatology. The DUKE scale and the EMA cautious action subscale correlate with the total PANSS, while the EMA social joining subscale correlates with the positive symptoms ($p<0.05$).
**Discussion:** Resilience, Coping Strategies and Social Support seem to have an important role in the appearance and severity of an FEP. It is necessary to carry out more studies with more sample, even so, the results indicate that these factors may be important for the prevention of an FEP and could be worked on in future interventions in FEP patients as well as in HC.

### T246. DECREASING AGGRESSIVE BEHAVIOR IN PATIENTS WITH COGNITIVE IMPAIRMENTS BY TRAINING PSYCHIATRIC STAFF IN INTERACTIVE SKILLS

Daniel Abrams*[,1], Anneli Goulding[2], Margda Waern[2], Nils Sjöström[2]

[1]University of Gothenburg; [2]University of Gothenburg, Sahlgrenska University Hospital

**Background:** Preventive measures to decrease aggressive incidents in psychiatric care range from friendly responses to advanced de-escalation techniques.

But interventions have not often been systematically evaluated and often have different emphasis. There is also large variation in the outcome measurements used.

A method that has been used in Sweden is an interactive training approach, which aims to establish and maintain calmness and security for patients with cognitive impairments. Experiences from Gothenburg indicate decreased levels of coercive measures after training staff and providing supervision. The in-patient-unit where such training and application has been carried out most consistently, won a national award in 2016 for having no coercive measures taken in six months, despite 90 percent of the patients receiving compulsory care.

The intervention is a well defined 3-day-course, with two trainers and twelve participants. The main part of the course is devoted to the role playing of conflict situations with patients, based on the participants' own experiences and examples. Visual analysis tools are used to make the role plays into learning situations.

**Aim:** We describe here the study protocol for a planned project that will test the Interactive Training approach in four regional hospitals. In addition, group interviews will be applied to increase understanding of staff experiences, as well as the evaluation of the implementation process.

**Methods:** Planned sub-studies:

1. Staff's experience of using interactive methods will be analyzed through focus-groups; four group interviews with 5 people in each group. (Assisting nurses and nurses working full-time, who have been educated in interactive conflict-handling and worked according to the method for at least one year).
2. Intervention study. The staff at the psychiatric departments of four different hospitals will receive training in interactive conflict handling, and after the course, supervision. The purpose is to compare the number of aggressive events before and after the intervention.

The instruments that will be used for measurement of the effect are the Staff Observation Aggression Scale - revised (primary outcome), the Social Dysfunction Aggression Scale and the Clinical Global Impression - Severity Scale.

We will also document the type of care (voluntary or compulsory), the number of psychiatric hospital beds, the number of inpatient patients, the number of staff employed, if the patient was affected by alcohol or illegal drugs and several other variables. Diagnoses will be retrieved from patient records.

3. Evaluation of implementation. The purpose is to analyze the implementation of the intervention at four hospitals. Group interviews will be conducted and the data will be analyzed qualitatively by using Normal Process Theory (NPT) as a framework. NPT is an action research perspective that focuses on what actors actually do and discerns between, implementation, embedding integration as different levels of change.

**Results:** Data collection for the first sub-study will be completed in June 2018 and results from the second and third are anticipated to be available by March 2019 and December 2019, respectively.

**Discussion:** Possible methodological problems are that data from focus-groups may not be possible to generalize. However, qualitative data may capture experiences that shed light on the psychological working-mechanisms of the intervention.

The intervention study is expected to generate rich data, where essential variables are controlled for, for example organizational features, distribution of diagnoses and severity of symptoms. However, in a complex organization, it may not be possible to control for all variables that might explain variations in outcome.

### T247. INSIGHT INTO NEGATIVE SYMPTOMS AS AN IMPORTANT TARGET FOR PSYCHOSOCIAL REHABILITATION IN RELATION TO CLINICAL CHARACTERISTICS

Maria Minyaycheva*,[1], Igor Gladyshev[1], Oleg Papsuev[1]
[1]Moscow Research Institute of Psychiatry

**Background:** Apathy and amotivation are considered as the core features of negative symptoms in patients with schizophrenia spectrum disorders. It's well know that schizophrenia patients often lack insight into their symptoms. Insight bias affects self-representation, social functioning and social outcomes, reduces effects of psychosocial treatment and rehabilitation.

**Objective:** To research key aspects of insight into apathy depending on diagnostic categories in patients with schizophrenia spectrum disorders. The aim of the study was to analyze correlations of insight into apathy/amotivation with clinical symptoms, compliance with treatment and social cognition.

**Methods:** 103 patients with schizophrenia and schizophrenia spectrum disorders were recruited to participate in the study. Only patients in stabilized state that met criteria of PANSS total score ≤ 80 points were included. Demographic data was collected along with the clinical description on prevailing symptoms during acute phase. Discrepancy score for Apathy Evaluation Scale clinical (AES-C) and self-rated (AES-S) versions was used to assess insight into amotivation syndrome. Hinting Task, Ekman-60 and RAD-15 were used to assess social cognition and BACS was used for neurocognition.

**Results:** Overall, moderate positive correlations between AES-C and PANSS amotivation subscale N2 and N4 items, N6 item with total PANSS negative subscale were revealed. No significant correlations with G16 item were registered. AES-C/AES-S discrepancy ratio also modestly correlated with paranoid schizophrenia (r=0,29) and prevailing delusional symptoms during acute phase (r=0,33) of manifest psychoses, age of onset (r=0,28) and inpatient only treatment intake (r=0,27). It was negatively correlated with number of hospital admissions (r=-0,43). It is worth noting that we found no correlation between AES discrepancy ratio and social cognition and neurocognition.

**Discussion:** Patients with prevailing paranoid symptoms not only lack insight into positive symptoms, but tend to underestimate their negative symptoms such as motivation and apathy. Clinically this can be described by overestimated strengths, overstated expectations, exaggerated hopes, mistakenly overrated beliefs. These phenomena often biases the recovery process and need to be addressed during motivational enhancement therapy. Patients with more difference between the results in AES-C and AES-S are less critical to their conditions and less committed to therapy while being more paranoid in their beliefs. It is also harder to identify problems and targets for these patients as they often see no reasons for treatment at all. Probably with some of these patients indirect methods (metacognitive training) would be preferable rather than psychoeducation-based approaches when choosing psychological therapies. Interestingly no relationship of insight and social cognition was revealed. That needs further investigation as motivation is often considered to be a mediator for neurocognitive and social cognitive functions while there is still little works on the role of insight in relation to social cognition.

### T248. PSYCHOPATHOLOGY IN F2X.X-UNAFFECTED CO-TWINS AS A VULNERABILITY INDICATOR OF PSYCHOSIS

Rikke Hilker*,[1], Mette Nielsen[2], Christian Legind[3], Maria H. Jensen[4], Simon Anhøj[4], Brian Broberg[5], Birgitte Fagerlund[6], Merete Nordentoft[6], Birte Glenthøj[4]
[1]Mental Health Center Glostrup; [2] Center for Neuropsychiatric Schizophrenia Research (CNSR); [3]Center for Neuropsychiatric

*Schizophrenia Research (CNSR), Center for Clinical Intervention and Neuropsychiatric Schizophrenia Research (CINS), Mental Health Centre Glostrup, University of Copenhagen; [4]Center for Neuropsychiatric Schizophrenia Research (CNSR), Center for Clinical Intervention and Neuropsychiatric Schizophrenia Research (CINS), Copenhagen University Hospital; [6]University of Copenhagen*

**Background:** Studies have shown that the risk of developing schizophrenia is associated with an increased risk of most other psychiatric disorders[1] and that the familial transmission of risk extends across diagnostic categories.[2] In twin studies, unaffected twins may not be completely free of symptomatology even when they do not fulfill diagnostic criteria for a psychiatric illness, but this has not been systematically tested in a twin design.
The aim of the study was to investigate subtle psychopathology in unaffected co-twins from proband twin pairs, where one twin had a schizophrenia spectrum disorder diagnosis (ICD-10: F2x.x), and compare the level of psychopathology of F2x.x-unaffected co-twins to that of healthy twins.
**Methods:** We conducted a multimodal, cross-sectional combined clinical and register-based nation-wide twin study, by including twin pairs where one or both twins had a diagnosis in the schizophrenia spectrum (identified by linking The Danish Twin Register and the Danish Psychiatric Central Research Register). A group of age-and gender matched healthy twin pairs were included. All subjects underwent ratings of psychopathology with Positive and Negative Syndrome Scale (PANSS), Comprehensive Assessment of At Risk Mental State (CAARMS), and Clinical Global Impression (CGI). The current level of functioning was estimated with Global Assessment of Function (GAF).
**Results:** A total of 219 twins were included; i.e. proband twins (n=65), F2x.x-unaffected co-twins (n=56) and healthy twins n=98). For unaffected co-twins, the mean PANSS total score was 38 (SD 12.6), the CGI score was 1.7 (SD 1.1) and the GAF score was 74 (SD 13.3), which were all significantly higher than in healthy twins (all $p<0.02$), who had a mean PANSS total score of 32 (SD 5.7), a CGI score of 1 (SD 0.3) and a GAF score of 85 (SD 7.3). For CAARMS, the following items were significantly more severe in the unaffected co-twins compared to healthy twins: anxiety (p=0,021), OCD (p=0.008), stress tolerance (p=0.001), aggression (p=0.01), inappropriate affect (p=0.03), social isolation (p=0.01), and impaired role-function (p=0.001).
**Discussion:** These preliminary results indicate a subtle but significant level of psychopathology in unaffected co-twins of probands affected with a schizophrenia spectrum disorder compared to healthy twins, measured by PANSS and CGI. Results from CAARMS indicate specific areas of interest, clustered around emotional and behavioral symptoms like anxiety, stress intolerance, social isolation, inappropriate affect/aggression and impaired role-function. This may suggest subtle symptomatology in the f2x.x-unaffected co-twins, which may contribute to the significantly lower level of function in the co-twins compared to healthy twins. The heritability of these measures of psychopathology will be examined using Structural Equation Modelling on the whole cohort of probands, unaffected co-twins and healthy twins.
**References:**
1. Mortensen PB, Pedersen MG, Pedersen CB. Psychiatric family history and schizophrenia risk in Denmark: which mental disorders are relevant? Psychol Med 2010; 40: 201–10.
2. Rasic D, Hajek T, Alda M, Uher R. Risk of mental illness in offspring of parents with schizophrenia, bipolar disorder, and major depressive disorder: a meta-analysis of family high-risk studies. Schizophr Bull 2014; 40: 28–38.

### T249. THE ROYAL AUSTRALIAN AND NEW ZEALAND CLINICAL PRACTICE GUIDELINES FOR SCHIZOPHRENIA AND RELATED DISORDERS (2016) – A STEP TOWARDS BETTER CARE?

Cherrie Galletly*[,1], David Castle[2], Frances Dark[3], Verity Humberstone[4], Assen Jablensky[5], Eoin Killackey[6], Jayashri Kulkarni[7], Patrick McGorry[6], Olav Nielssen[8], Nga Tran[2]
*[1]The University of Adelaide; [2]University of Melbourne, St Vincent's Hospital; [3]Metro South Health Services; [4]Northland District Health Board, University of Auckland; [5]The University of Western Australia; [6]Orygen Centre for Youth Mental Health, The University of Melbourne; [7]Monash Alfred Psychiatry Research Centre; [8]St. Vincent's Hospital*

**Background:** Clinical Practice Guidelines are developed to improve clinical standards, encourage use of evidence-based treatments, and provide a foundation for audits, service evaluation, and research. This presentation by the expert writing group responsible for the updated RANZCP Clinical Practice Guidelines for Schizophrenia and Related Disorders describes the process, the challenges and the barriers in writing these new clinical guidelines. Once published, dissemination, discussion and utilisation of new clinical practice guidelines is crucial.
**Methods:** The RANZCP Clinical Practice Guidelines (CPG) for Schizophrenia and Related Disorders were developed using the existing RANZCP and international guidelines, research evidence, and in the absence of clear evidence, expert consensus. The NHMRC levels of evidence for intervention studies were used as a benchmark for each recommendation. A clinical staging model was proposed. There was an increased emphasis on physical health comorbidities, psychological treatments, and vocational recovery. The draft document was subjected to extensive review and revision involving independent psychiatrists, other clinicians and stakeholders, consumer groups, RANZCP committees and reviewers for the ANZJP. The Guidelines are available for open access on the RANZCP website at https://www.ranzcp.org/Publications/Guidelines-and-resources-for-practice.aspx.
**Results:** The Guidelines have been widely cited. The RANZCP has developed a Consumer Guide and Clinical Audit Tools based on the CPG recommendations. The recommendations made in the guidelines have resulted in some controversy – most notably about the use of depot antipsychotics, and antipsychotic medication discontinuation after recovery from first episode psychosis. As with most CPGs, there is no mechanism for ongoing updating of treatment recommendations in response to new evidence, so regular revisions of CPGs will be needed.
**Discussion:** The Guidelines provide a comprehensive summary of the evidence for interventions to treat schizophrenia and related disorders, set out a recommended standard of care to be adopted by clinicians in Australia and New Zealand, and create a benchmark against which individual practice and services can be compared. The debate generated by the publication of the guidelines has highlighted the gap between the recommended standard of care and existing practice, especially as it relates to the physical care and psycho-social interventions offered to people with these conditions.

### T250. CLINICAL CORRELATES OF SUBJECTIVE QUALITY OF LIFE IN INDIVIDUALS WITH AT-RISK MENTAL STATE FOR PSYCHOSIS IN HONG KONG

Irving Teng[1], Wing Chung Chang*[,1], Hoi Ching Lee[1], Cheuk Fei Wong[1], Sui Fung Wo[1], Suet In Chan[1], Sanyin Chiu[1], Lai Ming Hui[1], Kit Wa Chan[1], Ho Ming Lee[1], Yi Nam Suen[1], Eric Chen[1]
*[1]The University of Hong Kong*

**Background:** Subjective quality of life (SQoL) is an important outcome domain in individuals with at-risk mental state (ARMS) for psychosis. In an effort to better understand and maximize SQoL in ARMS populations, an increasing number of research has been conducted to investigate factors determining SQoL. This study aimed to examine clinical, functional and cognitive correlates of SQoL in Chinese young people presenting with ARMS in Hong Kong.
**Methods:** This is a naturalistic prospective study examining the longitudinal course of ARMS and prediction of psychosis in Hong Kong. In total, 110 Chinese participants aged 15 to 40 years presenting with ARMS were recruited from a territory-wide specialized early intervention service for psychosis. ARMS status was verified using Comprehensive Assessment for At-Risk Mental State (CAARMS). Assessments encompassing symptom profiles (Positive and Negative Syndrome Scale, PANSS; Montgomery-Asberg Depression Rating Scale, MADRS; Brief Negative Symptom Scale, BNSS), functioning (Social and Occupational Functioning Rating Scale, SOFAS) and a brief battery of cognitive tests was conducted. A validated Chinese version of SF12 questionnaire was used to measure SQoL. The current analysis focused on data collected at baseline.
**Results:** Of 110 ARMS participants, 48.2% were male. The mean age and educational level of the sample was 20.9 years (S.D.=6.7) and 11.4 years (S.D.=2.6), respectively. Correlation analyses revealed that SF12 mental health score was correlated with MADRS total score, BNSS total score and SOFAS score, while SF12 physical health score was correlated with PANSS positive symptom score only (p<0.05). Multiple linear regression analysis showed that only MADRS total score was independently associated with SF12 mental health score (p<0.001). SQoL measures were not correlated with any cognitive functions.
**Discussion:** Our results were consistent with the literature which indicates that psychological domain of SQoL is significantly related to depressive symptoms in ARMS individuals. Further analysis on the longitudinal data regarding our prospective ARMS cohort will clarify variables predictive of SQoL at follow-up.

### T251. THE STUDY OF QUALITY OF LIFE AND A GLOBAL FUNCTIONING FOR THE SCHIZOPHRENIA PATIENTS IN COMMUNITY BY THEIR RESIDENTIAL ENVIRONMENT

Sang-Yeol Lee*[,1], Young-Joon Kwon[2], Bo-Hyun Yoon[3], Kwanghun Lee[4], Moon Doo Kim[5], Beomwoo Nam[6], Sung-Yong Park[7], Eunsung Lim[8]
*[1]Wonkwnag University School of Medicine and Hospital; [2]Soonchunhyang University Chunan Hospital; [3]Naju National Hospital; [4]College of Medicine, Dongguk University; [5]School of Medicine, Jeju National University; [6]School of Medicine, Konkuk University; [7] Keyo Hospital; [8]Shinsegae Hospital*

**Background:** Mental health is so deeply related to our residential environment that the difficulties of residence could worsen it for schizophrenia patients. Moreover, patients with schizophrenia might induce severe residential problems such as poverty, discrimination, failure to education, frequent migration, homeless, and etc. This study investigated the quality of life and a global functioning for schizophrenia patients in community by their residential environment.
**Methods:** Total 648 patients with schizophrenia living in Jeollabukdo(province) were tested demographic and clinical characteristics. Housing and residential satisfaction were measured by the questionnaires established by Ministry of Land, Transport and Maritime Affairs and modified for this study. Psychiatric and psychological variables were assessed by Global Assessment Function (GAF) and World Health Organization Quality of Life Assessment Instrument Brief Form (WHOQOL - BREF). Correlations among variables were analyzed using frequency analysis and Pearson's product moment correlation coefficient.
**Results:** As the results of correlations between quality of life and housing satisfaction, correlations were shown at a global quality of life (r=0.312, p<.01), physical health (r=0.227, p<.01), psychological domain (r=0.215, p<.01), social relation domain (r=0.170, p<.01), and environmental domain (r=0.372, p<.01). For the correlation between quality of life and residential area satisfaction, a general quality of life (r=0.307, p<.01), physical health (r=0.242, p<.01), psychological domain (r=0.243, p<.01), social relation domain (r=0.169, p<.01), and environmental domain (r=0.306, p<.01) were correlated.
**Discussion:** The correlations among residential environment, quality of life, and a global functioning were significant. Consequently, it is necessary for the government policy that can improve housing and residential environment for the mentally disordered and ultimately contribute to enhance their welfare.

### T252. TREATMENT DELAY AND OUTCOME COMPARISON OF EXTENDED EARLY INTERVENTION SERVICE AND STANDARD PSYCHIATRIC CARE FOR ADULTS PRESENTING WITH FIRST-EPISODE PSYCHOSIS IN HONG KONG

Wing Chung Chang*[,1], Chun Ho[1], Chi Fai Or[1], Tsz Ting Liu[1], Fu Chun Lau[1], On Ki Chu[1], Lai Ming Hui[1], Kit Wa Chan[1], Ho Ming Lee[1], Yi Nam Suen[1], Eric Chen[1]
*[1]The University of Hong Kong*

**Background:** A territory-wide specialized early intervention (EI) service for psychosis (EASY) has been implemented in Hong Kong since 2001, providing 2-year phase-specific early assessment and clinical care to young people aged 15–25 years presenting with first-episode psychosis (FEP). Previous evaluation demonstrated superiority of EASY programme over standard care in outcome improvement in FEP. Recently, EASY has been extended to provide 3-year EI service to FEP patients aged 15 to 64 years. However, effectiveness of EI on adult FEP populations has not been well examined.
**Methods:** This study adopted case versus historical-control design, comparing patients received 3-year EASY treatment (EI group) with those managed by standard psychiatric care (SC group) prior to implementation of EASY extension in terms of treatment delay and outcomes in symptom and functioning. In total, 320 Chinese adult FEP patients aged 26–55 years (160 in EI group, 160 in SC group) were included in the study. Retrospective record review detailing service utilization over 3-year treatment period was conducted. Follow-up interview assessment (on average 48.3 months after service entry) encompassing premorbid adjustment, duration of untreated psychosis (DUP), clinical (Positive and Negative Syndrome Scale, PANSS; Calgary Depression Scale, CDS), functional (Role Functioning Scale, RFS) and treatment profiles was administered. Comparison analyses on DUP and service utilization were based on record review data of 320 patients. Clinical and functional outcome analyses focused on data collected from follow-up interview assessment (251 patients completed follow-up assessment, 130 from EI and 121 from SC groups).
**Results:** EI and SC groups were comparable regarding demographics, premorbid and baseline characteristics, except the use of second-generation antipsychotic (SGA) treatment (EI patients were more likely to receive SGA than SC patients). EI patients had significantly shorter DUP than SC counterparts (p=0.015). Regarding follow-up outcomes, EI patients displayed lower levels of negative (p=0.044) and depressive symptoms (p=0.055), higher scores in RFS immediate social network (p=0.027) and lower rates of service disengagement (p=0.048) than SC patients even when SGA use and DUP were adjusted as covariates in analysis of covariance for comparison. There were no significant group differences in admission and suicide rates.
**Discussion:** Our results indicate that extended EASY service achieve favorable outcomes in adult FEP patients on shortening of treatment delay and improvement in negative symptoms and social functioning, and service disengagement reduction. Further evaluation is required to assess the sustainability of positive effects.

### T253. THE CORRELATION ANALYSIS BETWEEN RENAMING SCHIZOPHRENIA AND VISITING FREQUENCY OF MENTAL HEALTH SERVICES BY BIG DATA ANALYSIS (INTERNET SEARCHES AND NEWSPAPER ARTICLES) IN SOUTH KOREA

Sang Yup Lee[1], Kyung Sue Hong[2], Yeon Ho Joo[3], Shinsuke Koike[4], Yu Sang Lee*[,5], Jun Soo Kwon[6]
*[1]Yonsei University; [2]Sungkyunkwan University School of Medicine, Samsung Medical Center; [3]University of Ulsan College of Medicine; [4]The University of Tokyo; [5]Yongin Mental Hospital; [6]Seoul National University College of Medicine*

**Background:** Korean Neuropsychiatric Association changed the Korean term for schizophrenia from 'split-mind disorder' to 'attunement disorder' in 2012, to dispel the stigma associated with name, and to promote early detection and treatment. Information on the internet affects the public awareness and attitude toward schizophrenia. The main purpose of this study was to investigate the correlation between renaming schizophrenia and the pattern of mental health services utilization by big data analysis of internet (newspaper articles and internet searches) in Korea.

**Methods:** From January 2016 to September 2017, newspaper articles on "attunement disorder" and "split-mind disorder" available on the internet were classified as related with negative images like crime and helpful or positive in dispelling the stigma. The relationship between the number of anti-stigma newspaper articles and newspaper articles of schizophrenia containing both positive and negative images was examined. In addition, using Naver, a major internet search engine in Korea, we investigated the total number of internet searches of both old and new name of schizophrenia by gender differences. Finally, the frequency of the visits of mental health services of patients with schizophrenia was measured using the Korean Healthcare Bigdata Hub (http://opendata.hira.or.kr/home.do#none) for 14 months and the correlation between the frequency of the visits and the above big data was examined. The data were analyzed using the SPSS/WIN 24.0. Pearson correlation coefficients were used to analyze correlations.

**Results:** The amounts of newspaper articles containing anti-stigma of schizophrenia were correlated with the amounts of newspaper articles containing negative images like crime of the new name (attunement disorder) of schizophrenia ($r=0.528$, $p<0.01$), which was greater than the amounts of newspaper articles containing the old name (split-mind disorder) of schizophrenia ($r=0.300$, $p<0.01$). We also found that a strong positive correlation between the number of articles about "attunement disorder" and search frequency about the term on the internet. In addition, the search frequency was more highly related to the number of articles containing negative images of the illness (e.g., related crimes, $r = 0.910$, $p<0.01$) than that of articles providing positive aspects of the illness (e.g., dispelling stigma, $r = 0.423$, $p<0.01$). There was no significant correlation between the number of schizophrenia-related newspaper articles in previous month and the visits of mental health services of patients with schizophrenia in next month. There were no gender differences in internet searches. The correlation between the internet search frequency for "attunement disorder" in the previous month and the visits of the mental health services of patients with schizophrenia ($r = 0.185$, $p>0.05$) in next month was larger than the correlation of "split-mind disorder" searches with mental health services utilization ($r = 0.082$, $p>0.05$).

**Discussion:** "Attunement disorder" rather than "split-mind disorder" was appeared more frequently in newspaper articles of the anti-stigma characteristics. "Attunement disorder" seems to be more useful for anti-stigma campaign. Renaming schizophrenia didn't seem to affect the visiting frequency of mental health services. There was statistical limitation which was originated from the lack of numbers of patient's information. It was because Korean Bigdata Hub provided patients information just for 14 months as monthly data. Also, it should be considered that the time period, the kinds of mental disorders and the search engine we investigated were limited. Future research needs to overcome these limitations.

### T254. IMPACT OF A DROP IN, OPEN ENDED, PSYCHO EDUCATIONAL GROUP ON CLIENT ENGAGEMENT IN AN EARLY PSYCHOSIS INTERVENTION PROGRAM

Crystal Morris*[,1], Elham Sadeh[1], Greg McMillan[1], Sharman Robertson[1]

[1]*The Ottawa Hospital*

**Background:** Participating in groups are a critical part of rehabilitation model in Early Psychosis Intervention. Effectiveness of intervention is dependent on the willingness of an individual to engage and remain engaged throughout the duration of the intervention. Approximately 30% of individuals with a first episode of psychosis disengage from services despite ongoing needs.

On Track is a community based mental health outpatient program specialized in early assessment & treatment of psychosis. Client population includes individuals aged 16 to 35 years residing in the North East of Ontario, Canada.

The program offers psychiatric follow up as well as unique group based interventions. Clients are required to be screened for group suitability & community safety prior to participation. Program evaluation highlighted challenges to engage clients. An open-ended, psycho-educational, drop in group called "The Café" was developed.

**Methods:** Study used a mixed-methods approach to evaluate the impact of the Café sessions on clients & their engagement in the program. Data was collected between December 2016 to June 2017. All clients were diagnosed with primary psychosis disorder. Patient experiences & engagement level were measured using a questionnaire & the Singh-O'Brien Level of Engagement Scale.

**Results:** 43 clients participated in 29 Café sessions in seven months. Average number of participants per session was 7.3 (SD=3.05). Over 72% of clients participated in at least one to five sessions. Average age was 23.9 (SD=0.6). Participants were predominantly male (82%), white (42%), English speaking (93%), living with their parents (76.2%), no employment (69%), with some college or university degree (45.2%). Primary source of income were their families (47.6%). Over 59% of participants were in the program one year or less, 18.2 % & 22.7 % of participants have been in the program for two & three years, respectively.

The engagement score among males did not differ significantly from females ($t(42)=-0.94$, $p=0.35$). There was no significant differences in engagement scores between ethnic groups ($F(4)=2.84$, $p=0.062$). There was a marginally significant difference in engagement score between program duration groups ($F(2)=3.23$, $p=0.051$); the engagement score for clients who have been enrolled in the program for over two years were slightly higher than one year clients.

41% of clients attended Café before participating in any other groups; 4 of them joined other recreational & leisure groups. 3 clients later enrolled in a skill based group. 92% of respondents commented about the benefit of Café to their recovery as being "good" or "better". Qualitative data identified socializing, coping with illness, & drop in nature of the Café as main themes contributing to recovery.

**Discussion:** In an attempt to increase opportunities for engagement, our program developed a drop in Café style group to provide client centered psycho-education group. The result of our intervention showed that the Café provided benefits to client's engagement and recovery. The Café facilitated additional engagement in both recreational and leisure & skill based group streams. The drop in nature of the Café was advantageous to increasing engagement. Participation in the Café resulted in improvements in coping with the illness & socialization. Clients who had been enrolled in our program for 2 years reported higher levels of engagement compared to clients newly enrolled.

Evaluating our intervention through collecting both qualitative & quantitative data provided us with a better understanding of the impact of the open- ended, drop in nature, psycho-education based group. Involvement of both client's and families in the identification of the topics was another advantage of our study.

### T255. WHO PARTICIPATED IN FAMILY WORK IN THE US RAISE-ETP FIRST EPISODE SAMPLE?

Shirley Glynn*[,1], Susan Gingerich[2], Piper Meyer-Kalos[3], Kim Mueser[4], Alec Chan-Golston[1], Catherine Sugar[2], Nina Schooler[5], John Kane[6]

[1]*University of California, Los Angeles;* [2]*Independent Consultant;* [3]*The University of Minnesota;* [4]*Boston University;* [5]*SUNY Downstate Medical Center;* [6]*Northwell Health*

**Background:** The Recovery After an Initial Schizophrenia Episode Early Treatment Program (RAISE-ETP) is a US NIMH-funded 34 site cluster randomized controlled trial which evaluated the benefits of participation in a multicomponent intervention, entitled NAVIGATE, for first episode psychosis (FEP). Previously, participation in NAVIGATE was reported to yield significant participant benefits, compared to customary care (Kane et al, 2016). NAVIGATE included tailored medication, individual resiliency training, family education, and supported education and employment. Here we examine the absolute rate of family engagement in professional support services in the intent to treat sample, as well identify predictors of participation.
**Methods:** A total of 404 individuals between ages 15 and 40 were enrolled. DSM-IV diagnoses of non-affective psychosis were included. All participants had experienced only one episode of psychosis, had been prescribed less than 6 months of lifetime psychotic medication, spoke English, and provided informed consent. Participants were offered a minimum of two years of NAVIGATE or customary care (CC). At baseline, participants provided demographic and clinical history information; they were administered the Heinrichs-Carpenter Quality of Life Scale (QOL) and the Positive and Negative Symptom Scale (PANSS). Site research assistants interviewed participants monthly to capture participation in the four types of NAVIGATE interventions, allowing treatment groups to be compared on receipt of key services.
**Results:** One hundred nineteen of the 404 participants (29.4%) reported their relatives attending five or more family sessions within the first year of randomization (102 families (45.74%) in NAVIGATE; 17 (9.39%) in CC). In a simultaneous logistic regression analysis predicting meeting this five family sessions threshold or not, significant independent predictors (all $p < .05$) included treatment group, consumer negative symptoms, consumer self-reported quality of family relationship, race, and consumer residence. Relatives were more likely to attend family sessions if their loved one was 1) randomized to NAVIGATE, 2) had greater negative symptoms on the PANSS, 3) self-reported as emotionally closer to the family, 4) was Caucasian, and 5) lived with family. Other consumer PANSS and QOL scores, consumer age, ethnicity, health insurance status, cigarette smoking status, and consumers' mother education were not significant independent predictors.
**Discussion:** Although the benefits of family support and education have been highlighted for persons with a recent onset of psychosis, the results here suggest that engaging relatives in these services, at least in the US, can be challenging. Even given a relatively low threshold of attendance at least 5 family sessions in the first year of treatment, the majority of this sample did not meet the criterion, although participation rates were significantly higher in NAVIGATE. This increase likely reflects the effort NAVIGATE teams expended to engage relatives. It is perhaps not surprising that families of consumers who live with them and/or report feeling closer to them are more likely to attend clinic sessions. Interestingly, higher levels of consumer negative, but not positive symptoms, were also associated with greater attendance at family sessions; this finding suggests that living with a consumer who appears unmotivated and withdrawn may be particularly challenging and prompt relatives to seek more assistance. Finally, our data on race suggest, as other have noted, that greater outreach may be needed to engage non-Caucasian families in services.

### Poster Session II

### F1. GENOME-WIDE ASSOCIATION STUDIES SUGGESTED ASSOCIATION BETWEEN DGKB AND ANTIPSYCHOTIC INDUCED WEIGHT GAIN IN EUROPEANS AND AFRICAN AMERICANS

Malgorzata Maciukiewicz[1], Arun Tiwari[2], Vanessa Goncalves[1], Clement Zai[2], Eva Brandl[3], Natalie Freeman[1], Jeffrey Lieberman[4], Herbert Meltzer[5], Christopher Laughlin[6], Erika Nurmi[7], James Kennedy[2], Daniel Mueller*[,8]
*[1]Centre for Addiction and Mental Health; [2]Centre for Addiction and Mental Health, University of Toronto; [3]Charité University Clinic; [4]Columbia University; [5]Northwestern University Feinberg School of Medicine; [6]UCLA Semel Institute for Neuroscience; [7]University of California, Los Angeles; [8]University of Toronto*

**Background:** Schizophrenia (SCZ) is a severe, devastating disorder with a life-time prevalence of 1% irrespective of gender or ethnic group, treated primarily with antipsychotic (AP) medications. Despite clinical efficacy of APs, they are associated with severe side effects including antipsychotic-induced weight gain (AIWG).
**Methods:** We investigated n=201 schizophrenia or schizoaffective disorder patients of European and African American ancestry who were treated mostly with clozapine or olanzapine. Individuals were genotyped on the Infinium Omni2.5 BeadChip. We conducted genome-wide association analysis for AIWG defined primarily as the percentage of weight change from baseline. Additionally, we ran pathway, enrichment, network, and polygenic risk score analyses to investigate top genes using in silico methods.
**Results:** In the mixed sample, we observed genome-wide significant association between the diacylglycerol kinase beta (DGKB) variant (β=0.411; p=3.15 × 10–9) and percentage of weight change. The association remained nominally significant in both Europeans (β=0.271; p=0.002) and African Americans (β=0.579; p=5.73 × 10–5) for the same risk allele. In Europeans, the top variant (β=0.406; p=1.26 × 10–6) was located upstream of the Stanniocalcin 2 (STC2) gene. Bayesian fine mapping suggested the variant nearby SNP upstream of STC2 (p=0.034; PHRED=3.691, posterior prob.=0.496) to be the most significant. We noticed no significant enrichment in metabolic pathways for SNPs, but our top genes (p<5 × 10–5) were enriched in the GWAS catalog for risk of obesity (pmixed=0.018; pEuropeans=0.015) and schizophrenia (pmixed=0.006). Top genes also interacted with known risk factors for obesity (Glucose-6-Phosphate Dehydrogenase (G6PD)) and schizophrenia (NudE Neurodevelopment Protein 1 Like 1 (NDEL1)), and are targeted by microRNAs related to schizophrenia (mir-34a) and obesity (mir-19b). Polygenic risk score analyses did not provide support for major genetic overlap between obesity-related and lipid-associated SNPs and the risk of AIWG.
**Discussion:** Our findings suggested that a variant in DGKB is associated with the percentage of weight gain in both African Americans and Europeans.

### F2. CHILDHOOD TRAUMA AND LACK OF CULTURAL IDENTITY AS RISK FACTORS OF ATTENUATED PSYCHOSIS SYMPTOMS AMONG AFRICAN AMERICAN YOUNG ADULTS

Huijun Li*[,1], Monique Rowe[2]
*[1]Florida A&M University; [2]Jewish Community Center*

**Background:** Schizophrenia spectrum diagnosis is more commonly assigned to African Americans. Failing to understand and appropriately manage cultural differences will have significant mental health consequences for varied racial/ethnic groups in particular (Betancourt, Green, & Carrillo, 2002). The purpose of the present study was to examine risk factors of attenuated psychosis syndrome in a sample of African American young adults, specifically to investigate whether lack of ethnic identity and adverse childhood experiences (ACEs) put an individual at a higher risk of developing attenuated psychotic symptoms.
Adverse Childhood Experiences (ACE) as Risk Factor of APS:
The Comorbidity Survey (NCS) Part 2 data showed that the effects of neglect and sexual abuse, along with physical abuse similarly put a child at risk for psychosis. People who had suffered childhood adversity were 2.8 times more likely to develop psychosis than those who had not. Studies have also begun to look at gender differences in schizophrenia by way of ACEs.
Lack of Ethnic Identity as Risk Factors of APS:
The African worldview reflects psychological communal, spiritual, collective survival thrust as opposed to the European worldview of individualism and materialism. Cultural Misorientation (CM) represents that foreign psychological or psychopathological disposition in the African personality, which allows African Americans to unknowingly value and participate in European cultural indoctrination through the practice of European cultural values, rituals, and customs. The purpose of this study was to explore the roles that CM play on the overall presentation of attenuated psychotic symptoms, by way of ACE exposure.
**Methods:** Participants: Participants included 304 African American college students, 199 (65.46%) women and 105 (34.54%) men from a Historically Black College and University in the southeastern region of the United States. Participants were between 18 and 25 years of age.
**Instruments:** Adverse Childhood Experiences Scale measures the association of multiple types of abuse with different types of health outcomes. Prodromal Questionnaire- Brief (PQ-B) measures the presence of negative symptoms, perceptual abnormalities such as hallucinations, and unusual thought content like delusional ideas and paranoia. Cultural Misorientation - Short Form assesses the condition of cultural misorientation across 6 subscales-- materialism orientation, individualism orientation, alien-self orientation, anti-self orientation, self-destructive orientation, and integration orientation.
**Results:** The Pearson correlation analysis indicated no significant relationship (r = -.073, p = .206) between ACE exposure and APS total scores on PQ-B. However, an unexpected negative significant relationship between childhood abuse exposures and symptom severity was observed (r = -.126*, p = .028), indicating that participants who reported more instances of childhood abuse tended to report less symptom severity. In addition, Cultural Misorientation (CM) was significantly positively correlated to PBQ total scores r = .194**, p = .001) and the severity of those symptoms (r = .171**, p = .003). CM materialism and individualism subscales mediated the relationship between childhood abuse and PQ-B total scores and symptom severity.
**Discussion:** This study provides support that some aspects of cultural misorientation can be detrimental to African Americans. Helping to reduce material and individualistic desires that have become detrimental should also be a central focus of implemented mental health programs.

### F3. A CASE OF LEUKOCYTOSIS ASSOCIATED WITH CLOZAPINE TREATMENT FOR THE MANAGEMENT OF CHRONIC SCHIZOPHRENIA

Min-Kyu Song*[,1], Won-Myong Bahk[2], Young Joon Kwon[3], Bo-Hyun Yoon[4], Sang-Yeol Lee[5], Duk-In Jon[6], Sung-Yong Park[7], Eunsung Lim[8]
*[1]Yesan Mental Health Clinic; [2]Yeouido St. Mary's Hospital, The Catholic University of Korea; [3]College of Medicine, Soonchunhyang University; [4]Naju National Hospital [5]Wonkwang University, School of Medicine; [6]College of Medicine, Hallym University; [7]Keyo Hospital; [8]Shinsegae Hospital*

**Background:** Clozapine is an atypical antipsychotic drug with therapeutic efficacy through serotonin-dopamine antagonism. It has been used for the management of treatment-resistant schizophrenia and reducing the risk of suicidal behavior. In addition to agranulocytosis and leukopenia, clozapine has also been reported to be associated with other types of blood dyscrasias, including leukocytosis.
We wish to report a case of leukocytosis associated with clozapine treatment in a patient of chronic schizophrenia.
**Methods:** Our patient is a 48-year-old woman with a diagnosis of schizophrenia since the age of 22. She has a history of numerous hospitalizations and substantial treatment with conservative antipsychotics.
We evaluated her medical and psychiatric history as well as mental status. Then We assumed that she might have treatment-resistant schizophrenia and accordingly commenced treatment with clozapine. At the time of admission, her WBC count was 8.01 × 109/L and ANC was 5110. Clozapine was started at 3rd hospital day. We stopped aripiprazole and gradually increased clozapine. Only 2mg of lorazepam was used in combination with clozapine. Remission was achieved with 450 mg/day of clozapine. WBC on the 1st day of treatment was 12.41 × 109/L (ANC; 9481) and clozapine was 300mg/day. WBC on the 18th day of treatment was 15.32 × 109/L (ANC; 12501), and at that time, her clozapine dosage was 450mg/day. At this point, her vital sign was within normal range and physical examination did not showed any infectious signs. On the 25th day of treatment, WBC count was 22.1 × 109/L and ANC was 17680. However, no general medical condition to explain the leukocytosis was found.
We concluded that her leukocytosis was linked to clozapine, and decided to taper out clozapine despite there being no medical contraindication. After two weeks from starting blonanserin and olanzapine, WBC count normalized. Fortunately, despite replacing the medication, the remission was maintained.
**Results:** In our case, the increase in white blood cell count with increasing dose of clozapine was evident and did not reveal any other medical cause to explain leukocytosis in the patient. In addition, leukocytosis improved significantly after discontinuing clozapine. Also, there have been reports of cases of leukocytosis associated with clozapine treatment, and the mechanism of leukocytosis has been explained to some extent. Considering these temporal associations, mechanisms, and previous cases, it is reasonable to consider leukocytosis associated with clozapine in this case.
**Discussion:** This case suggests a temporal relationship between the use of clozapine and leukocytosis. It also shows the rapid resolution of leukocytosis after discontinuation of clozapine. The mechanisms of clozapine-induced leukocytosis may be related to changes in plasmatic concentrations of granulocyte colony-stimulating factor, tumor necrosis factor-α, interleukin (IL)-2 and IL-6 cytokines, which could be stimulated by clozapine. In our case, an increase in WBC count was consistent with an increase in clozapine dose, suggesting a dose-dependent effect. However, the appearance of leukocytosis during clozapine treatment does not mean that clozapine should be discontinued. Nevertheless, the WBC continued to increase steadily, and clinicians inevitably replaced the drugs in consideration of the fact that he was not a general hospital but a psychiatric clinic.
In this paper we have reported a patient of chronic schizophrenia who developed leukocytosis during clozapine treatment. It appears that most of clozapine-associated leukocytosis can be benign medical condition. However, clinicians should be aware of the dangers of other blood disorders such as leukocytosis.

### F4. LINKING LIFE EVENTS WITH NEGATIVE AFFECT AND PSYCHOTIC EXPERIENCES IN DAILY LIVES OF YOUTH: STRESS SENSITIVITY AS A PUTATIVE MECHANISM?

Christian Rauschenberg*[,1], Jim van Os[2], Dimitri Cremers[3], Matthieu Goedhart[3], Jan Schieveld[4], Ulrich Reininghaus[1]
*[1]Maastricht University; [2]University Medical Center Utrecht; [3]Tilburg University; [4]Maastricht University Medical Center*

**Background:** Negative life events are associated with a range of mental disorders, including psychosis. However, evidence on underlying mechanisms remain scarce. The current study aimed to investigate whether life events (e.g. intrusive threat, experience of loss, illness) impact on the sensitivity towards stress in daily lives of youth.
**Methods:** The Experience Sampling Method was used to measure momentary stress (i.e. event-related, activity-related, social), negative affect, and psychotic experiences in a sample of 42 help-seeking adolescents and young adults (service user), 17 siblings, and 40 comparison subjects (controls). Life events during lifetime and the previous year as well as depressive, anxiety, and psychotic symptoms were assessed.
**Results:** Stress sensitivity, that is, the associations between momentary stress and (i) negative affect and (ii) psychotic experiences, was modified by lifetime and previous negative life events in service users. While there was strong evidence for increased negative affect and psychotic experiences in service users when high vs. low levels of lifetime exposure to negative life events were compared a pattern of resilience was evident in controls with no marked differences in the magnitude of associations comparing high vs. low exposure levels. However, in controls, exposure to life events during the previous year were also found to impact on the stress sensitivity in daily life. Less consistent findings were observed in siblings.
**Discussion:** Our findings point to the importance of time that has passed between exposure to and impact of life events on stress sensitivity: while the detrimental effects may attenuate in controls over time, service users appeared to be at greater risk of negative long-term effects. Thus, stress sensitivity may constitute an important risk and resilience mechanism through which adverse life events impact on mental health in youth. Targeting stress sensitivity in daily life through ecological momentary interventions, potentially with stronger effects shortly after stress exposure, may represent a promising novel therapeutic approach.

### F5. CLOZAPINE RELATED THROMBOCYTOSIS AND THROMBOCYTOPENIA

Yuree Kang*[,1], Seung-Hyun Shon[1], Woon Yoon[1], Jungsun Lee[1]
*[1]Asan Medical Center*

**Background:** It is well known that clozapine causes hematological side effects such as agranulocytosis, neutropenia, and leukocytosis. But, the results about the effects of clozapine on the number of platelets were not consistent. Although thrombocytopenia or agranulocytosis are regarded as more clinically important side effects, thrombocytosis should be monitored because it may be related with increased the risk of thrombosis and pulmonary embolism. We investigated the effect of clozapine on the number of platelets in schizophrenia patients starting clozapine.
**Methods:** This was a retrospective chart review study using ABLE, an electrical medical record inquiry system of Asan Medical Center. Among individuals who were diagnosed schizophrenia, who applied clozapine more than three months were included as study subjects. Those who were unable to identify the complete blood count (CBC) at the beginning of the clozapine administration and who did not perform more than one CBC at least every 3 months during the observation period were excluded from the study. CBC scores at baseline and at 1, 3, 6, 9, and 12 months after the initiation of medication were obtained, and the mean platelet counts at the initiation and platelet counts at each observation period were compared by paired t-test. The cumulative incidence of thrombocytopenia (<150000 / mm3) and thrombocytosis (> 450000 / mm3) for one year were also calculated.
**Results:** Ninety-six patients were enrolled in this study and 50 and 41 subjects were remained at month 6 and 12, retrospectively. There was a significant mean platelet change only at month 1 (275.292 ± 74.464/mL) compared to the initiation of treatment (255.500 ± 74.464/mL) (t=-3.553, p>0.001). The cumulative incidence rates were 3.13% for thrombocytopenia, 6.25% for thrombocytosis.

**Discussion:** Mandatory hematological monitoring is important in the use of clozapine, and other trajectories as well as the WBC might be of clinical interest.

### F6. PSYCHOLOGICAL RESILIENCE TO SUICIDAL THOUGHTS AND BEHAVIOURS: A QUALITATIVE EXAMINATION OF THE EXPERIENCES OF PEOPLE WITH MENTAL HEALTH PROBLEMS ON THE SCHIZOPHRENIA SPECTRUM

Kamelia Harris*[,1], Patricia Gooding[1], Gillian Haddock[1], Sarah Peters[1]
[1] *University of Manchester*

**Background:** Suicide is one of the leading causes of premature death in schizophrenia. The lifetime risk of death by suicide in people with schizophrenia is around 10 per 100 people. Some people can counter the impact of suicide risk (e.g., life stressors) by incorporating personal skills and experiences. Although exposure to life stressors can have a detrimental effect on mental health, this does not apply to everyone, as some individuals appear protected from the impact of stress. The notion of resilience in mental health has recently gained research attention. Resilience has been defined as an individual's skills or resources that help them manage suicidal thoughts and feelings. Consistent with this definition, it is important to understand the psychological factors that contribute to resilience to the frequency and severity of suicidal thoughts and behaviours. We aimed to examine the experiences of psychological resilience to suicidal thoughts and behaviours of people with schizophrenia-spectrum mental health problems.
**Methods:** We conducted face-to-face interviews with 20 individuals with experiences on the schizophrenia spectrum and lifetime experiences of suicidal thoughts, plans, and behaviours. Participants were recruited from community mental health services in the North-West of England, UK. A topic guide for semi-structured interviews was used to facilitate participants' descriptions of their experiences in their own words. All interviews were audio-recorded, with participants' consent, and transcribed verbatim. Thematic analysis was used to examine the themes and patterns within the data which were important in addressing the research aim.
**Results:** The majority of the participants were white British, single, and living alone. The average age of the sample was 48 years (SD = 14.4; range: 23–75 years) and 50% were female. Several factors were involved in promoting psychological resilience. Participants described strategies that indicated they were being active in maintaining and developing their resilience. These included cognitive reasoning (e.g., rationalising and validating experiences) and active coping strategies (e.g., acceptance of experiences, perseverance). Perceived social support from significant others and mental health professionals (e.g., care coordinators, psychiatrists) also played an important role in bolstering individual resilience.
**Discussion:** This is the first study to showcase the experiences of resilience to suicidal thoughts and behaviours from the unique perspective of individuals with mental health problems on the schizophrenia spectrum and a range of suicidal experiences (e.g., ideation, attempts, plans). The data indicated that resilience to suicidal thoughts and behaviours developed over time, through the experience of managing psychotic symptoms and their deleterious impact on individual wellbeing. People employed different types of coping strategies, depending on the severity of their psychotic symptoms and suicidal feelings. However, if their symptoms became severe, they usually sought support from mental health services. Efforts to develop psychological resilience to suicidal thoughts and behaviours in individuals with schizophrenia are of paramount importance to clinical research and practice. It is important to consider the impact of psychotic symptoms, which were the main precipitating factors of suicidal thoughts and behaviours for most participants, in the development of psychological resilience. Of note, only individuals under the care of mental health services were recruited into the study. Future research should explore the resilience experiences of people not accessing mental health services.

### F7. SEX DIFFERENCES IN THE ASSOCIATION BETWEEN SELF-REPORTS OF CHILDHOOD ADVERSITIES AND SCHIZOTYPAL PERSONALITY TRAITS

Diamantis Toutountzidis*[,1], Tim Gale[1], Karen Irvine[1], Shivani Sharma[1], Keith Laws[1]
[1] *University of Hertfordshire*

**Background:** While it has been repeatedly documented that people with schizophrenia report higher levels of adverse events in childhood (emotional, physical and sexual abuse), this has not been extensively examined in healthy individuals who score highly on schizotypal personality traits. The continuum hypothesis of psychosis and schizophrenia suggests it is important to assess the relationship in those who are healthy but who experience some psychotic-like symptoms. Of course, it is problematic to rely upon the veracity of events that anyone might recall from their childhood, but this is likely to be compounded by the presence of well-documented memory and executive problems, as well as symptoms such as delusional thinking, in some adults with psychosis. One advantage of examining healthy participants is that recall is not affected by the condition itself or memory- and executive-function problems. As there is evidence that the expression of psychotic disorders differ between males and females, the etiological mechanisms and pathways to the development and experience of psychotic symptoms may equally differ. Indeed, sex differences in the association between childhood trauma and psychotic symptoms have been noted. The aim of this present study was to investigate any links between childhood trauma and psychotic-like symptoms in healthy individuals. Based on previous research the expectation is that associations will be found between self-reports of childhood trauma and schizotypal personality traits. These associations would be expected to differ between males and females.
**Methods:** The sample consisted of 320 participants (221 females, 99 males) with a mean age of 28.24 (SD 12.76). Childhood traumatic events were assessed by three sub-scales (Physical Punishment; Emotional Abuse; and Sexual Events) of the Early Trauma Inventory Self Report-Short Form (ETISR-SF; Bremner et al., 2007). Schizotypal personality traits were assessed using the Five Factor Schizotypal Inventory (FFSI; Edmundson et al., 2011). This consists of nine subscales (Interpersonal Suspiciousness; Social Anhedonia; Social Isolation and Withdrawal; Physical Anhedonia; Social Anxiousness; Social Discomfort; Odd and Eccentric; Aberrant Ideas; and Aberrant Perceptions) which were constructed as schizotypic variants of respective facets of the five factor personality model.
**Results:** The relationship between childhood trauma and schizotypy was examined using Spearman's bivariate correlation analyses. Males showed significant positive correlations (ranging from .28 to .39) between Emotional Abuse and seven out of nine schizotypal sub-scales. The other two childhood trauma scales were not associated with any schizotypal sub-scales in males.
Females showed significant positive correlations (ranging from .19 to .34) between Physical Punishment and eight out of nine schizotypal sub-scales. Additionally, females showed significant positive correlations (ranging from .26 to .35) between Emotional Abuse and all schizotypal sub-scales. Sexual Events positively correlated with two schizotypal sub-scales (Aberrant Ideas and Aberrant Perceptions) in females.
**Discussion:** From the results of this study it appears that emotional abuse was linked to the expression of psychotic-like symptoms in both sexes across a wide array of symptomatology. Therefore, any effect of emotional abuse should not be considered sex or symptom specific. By contrast, physical abuse appeared to be sex specific – affecting only females – but not symptom specific. Finally, sexual abuse appeared to have a specific link with disorganized thoughts and hallucination-like experiences in females.

### F8. SEARCHING FOR A STRATIFICATION MARKER FOR ANTIOXIDANT USE IN SCHIZOPHRENIA AND BIPOLAR DISORDER: A META-ANALYSIS OF MRS STUDIES OF ANTERIOR CINGULATE GLUTATHIONE

Avyarthana Dey[1], Alborz Javadzadeh[1], Priyadharshini Sabesan[2], Joaquim Radua[3], Jean Theberge[4], Lena Palaniyappan*,[1]
*[1]University of Western Ontario; [2]London Health Sciences Center; [3]King's College London; [4]St. Joseph's Health Care London*

**Background:** Glutathione [GSH] is a major intracellular antioxidant that disposes peroxides and protects neurons and glial cells from oxidative stress. In both schizophrenia and bipolar disorder, atypical levels of GSH has been demonstrated, particularly in the anterior cingulate cortex (ACC), though no consistent results have emerged due to limitations in sample size. Examining the state of GSH deficit in schizophrenia is a critical step when attempting to correct putative redox imbalance in this illness using agents such as N-Acetyl Cysteine (NAC). We conducted a meta-analysis to investigate the aberrations in GSH levels in the ACC of patients with schizophrenia and bipolar disorder measured using magnetic resonance spectroscopy (MRS).
**Methods:** Medline, Google Scholar, Ovid Online and EMBASE databases were searched for studies published until September 2017. Search terms included magnetic resonance spectroscopy, MRS, schizophrenia, psychosis, psychotic, bipolar disorder, glutathione, GSH. We included all 1H-MRS studies reporting GSH values for patients satisfying DSM or ICD based criteria for a primary psychotic disorder (SCZ) or bipolar disorder (BPAD) in comparison to a healthy controls (HC) group. We screened all identified abstracts, filtered studies that did not satisfy inclusion criteria, hand-searched references and contacted experts to locate further studies. We excluded studies that reported only on comorbid illnesses, did not compare patients and HCs, or failed to report data required to construct effect size metrics. After initial screening, a total of 261 patients and 185 controls were considered for the meta-analysis from the SCZ group; 464 patients and 245 controls were considered for the meta-analysis from the BPAD group. A random-effects, inverse-weighted variance model was used to calculate the pooled effect size. Mean values were extracted and verified independently. Effect sizes were computed based on excel macro, produced by Major Depressive Disorder Neuroimaging Database investigators.
**Results:** Contrary to our expectations, in SCZ, there were no significant differences in ACC GSH in patients compared to HC (RFX p= 0.74; 95% CI, -0.24 to 0.17; FFX p= 0.71; heterogeneity p = 0.58). In BPAD, there were highly significant differences in the ACC GSH, with patients having higher GSH concentrations than HC (RFX: p= 0.0003; 95% CI, 0.14 to 0.5; heterogeneity p=0.70). In the BPAD group, the mean effect size (SMD) was d= 0.32, indicating a small to medium sized difference. A network meta-analysis revealed significantly higher GSH levels in BPAD compared to SCZ (RFX p= 0.01; 95% CI, 0.08 to 0.63; SMD=0.36; heterogeneity p = 0.71). There were several methodological issues in the reported studies. Notably, most acquisitions were not optimized to collect GSH spectra; polymorphisms in the glutamate-cysteine ligase catalytic gene (GCLC) were not quantified in most studies; voxel placements were not standardized across the published studies; majority of patients were medicated, in various stages of illness.
**Discussion:** There are no major differences in concentration of ACC glutathione in the anterior cingulate cortex in patients with schizophrenia, though in bipolar disorder, GSH levels appear elevated. Given that GSH is the most readily accessible cortical redox marker in vivo, current status of MRS literature is insufficient to prepare for stratified therapeutics with antioxidants among patients with schizophrenia. Nevertheless, abnormalities in the redox system may be more pronounced in bipolar disorder compared to schizophrenia, and could serve to guide stratification in samples lacking diagnostic clarity (e.g. in First Episode Psychosis clinics).

### F9. ALTERATIONS OF NEURONAL METABOLISM IN PATIENT SUBGROUPS AT ULTRA-HIGH RISK FOR PSYCHOSIS ACCORDING TO PACE CRITERIA – A 1H/31P-MR-SPECTROSCOPY STUDY

Stefan Smesny*,[1], Stephan Schack[1], Alexander Gussew[1], Kerstin Langbein[1], Juergen Reichenbach[1]
*[1]University Hospital Jena*

**Background:** Glutamatergic dysfunction, deregulated mitochondrial metabolism and alterations of membrane phospholipids have been extensively investigated in schizophrenic illness by using in vivo magnetic resonance spectroscopy (MRS). Findings in the ultra-high risk (UHR) phase of psychotic illness, however, are still rare and inconsistent. Combining both 1H- and 31P-MRS, this study investigates these aspects in the different UHR patient subgroups as defined by PACE (Personal Assessment and Crisis Evaluation) criteria.
**Methods:** We applied 3 T chemical shift imaging (3D 31P-MRS, 2D 1H-MRS) and hippocampal single-voxel MRS in 69 neuroleptic-naïve UHR patients (age: 26.2 ± 6.2y; males 59.4% 41/69, attenuated symptoms (AS) n=50, BLIPS n=5, genetic risk (GR) n=8, AS+GR n=6; transition rate 17.2%, all transitions in the AS or BLIPS group) and 61 healthy controls (age: 25.2 ± 4.8y; males 54.1% 33/61). 11 metabolite markers were investigated (neuronal/mitochondrial metabolism: glutamate (Glu), N-acetyl-aspartate (NAA), phosphocreatine (PCr), and adenosine triphosphate (ATP); phospholipid synthesis: phosphomonoester/-metabolites (PME, Peth, Pch); phospholipids breakdown: phosphodiester/-metabolites (PDE, Gpeth, Gpch); astrocyte activation: myo-Inositol (mI)) in 5 brain regions (dorsolateral prefrontal cortex, DLPFC; dorsomedial prefrontal cortex, DMPFC; dorsal anterior cingulate cortex, dACC; mediodorsal thalamus, Th; and hipoocampus, Hip). Psychopathology was assessed using the CAARMS-Interview as well as PANSS, BPRS-E and SCL-90-R ratings. Statistical analysis included multi-and univariate ANOVA, Kruskal-Wallis-tests and correlation analysis.
**Results:** (i) In all UHR individuals (and also in the AS and BLIPS subgroup), NAA was reduced in the left Th. There was no alteration of Glu. While PCr was increased in the left DLPFC, left dACC (right trend) and in the right Hip, ATP was not different from controls. PME were decreased in the right Hip, PDE did not differ from controls. mI was found increased in the left Hip. (ii) In the GR subgroup PCr was increased in the bilateral Th. The PME metabolite Peth was decreased in the right Th. PDE were increased in the left dACC. mI was increased in the left Th.
**Discussion:** While the observed pattern of metabolite abnormalities in the AS and BLIPS risk group suggests a pathology that affects the left thalamus (NAA decrease), left DLPFC, dACC and bilateral Hip (left: PCr increase, PME decrease; right mI increase), the pathology of the GR group appears more focussed on the bilateral Th (bil. PCr increase, right PME decrease, left mI increase) and left dACC (PDE increase). The results suggest a functional disturbance of networks including the left DLPFC, dACC, bilateral Hip and Th, whereby the latter might be more an expression of a genetic risk profile.

### F10. DIFFERENTIAL EXPRESSION OF MICRORNAS IN CEREBROSPINAL FLUID AND PLASMA SAMPLES IN SCHIZOPHRENIA

Juan Gallego*,[1], Eric Alsop[2], Todd Lencz[3], Kendall Van Keuren-Jensen[2], Anil Malhotra[3]
*[1]Weill Cornell Medical College; [2]The Translational Genomics Research Institute; [3]Zucker Hillside Hospital, Feinstein Institute for Medical Research*

**Background:** Genetic variants in miRNA genes and abnormalities in the concentration of microRNAs (miRNAs) in tissues and biological fluids have recently been associated with a diagnosis of schizophrenia. Most of these studies used post-mortem brain tissue or whole blood as the source of RNA. However, examination of microRNAs in cerebrospinal fluid (CSF) might provide an in vivo biomarker, more accurately reflecting expression level changes in the brain. To date, there are no studies that have investigated miRNA expression in CSF in patients with schizophrenia using small RNA-seq. In the past, our group had the opportunity of investigating the correlation between miRNA profiles in CSF and blood measured using microarray technology. Therefore, to expand our findings and use current cutting-edge technology, we measured miRNA profiles in CSF and plasma using small RNA-seq in a sample of patients with schizophrenia-spectrum disorder (SSD) diagnosis and healthy volunteers.
**Methods:** Twenty-two SSD patients and 17 healthy volunteers underwent a lumbar puncture and a blood draw. 15–25 cc of CSF and 5–10 cc of peripheral blood were obtained from each subject. CSF and peripheral blood samples were centrifuged. CSF and plasma samples were aliquoted into 1 mL cryovials, and stored at -80C degrees. Vesicular RNA was extracted from 1 mL of CSF and plasma samples following the protocol from the Qiagen exoRNA easy kit. The BioScientific NextFlex RNA sequencing kit was used for library construction. Sequencing was done on HiSeq2500. Samples that had at least 50,000 reads going to mature miRNA sequences were included in the analysis. Differential expression analyses were conducted in R using the DESeq2 package in Bioconductor.
**Results:** In the overall sample cohort, most subjects were male (66.7%), not Hispanic (81.0%) and black (48.7%). Mean age was 36.8 years (SD=12.3), There were no differences in age, sex, ethnicity or race between the patient and healthy control groups. In the patient group, 16 (72.7%) had schizophrenia, 5 (22.7%) had schizoaffective disorder and 1 (4.5%) had psychosis not otherwise specified. Differential expression (DE) analyses were conducted for 144 miRNAs in CSF and 354 miRNAs in plasma. After adjusting for multiple comparisons, DE analysis between patients and controls in CSF showed statistically significant higher levels in patients of miR-769-5p, miR-99b-3p, miR-107, miR-451a and miR-708-5. Similar analysis in plasma showed statistically significant higher levels in patients for miR-375, miR-204-5p, miR-942-5p, miR-6734-5p, miR-423-5p and miR-144-5p. Principal component analysis showed a clear separation between CSF and peripheral blood samples. Out of 443 miRNAs used to examine the relationship between CSF and plasma, 205 (46.3%) were detected in both plasma and CSF samples, 88 (19.9%) were detected only in CSF samples while 150 (33.9%) were detected only in plasma samples.
**Discussion:** Five miRNAs were upregulated in CSF samples and six were upregulated in plasma samples of SSD patients compared to healthy volunteers. There was no overlap in the statistically significant upregulated miRNAs between CSF and plasma samples. Therefore, miRNA profiles in CSF and plasma have important quantitative and qualitative differences that may make them excellent, but different, candidate biofluids for biomarker discovery.

### F11. TRANSLATIONAL STUDY OF GRIN1, GRIN2A AND 2B GENE EXPRESSION IN PATIENTS WITH SCHIZOPHRENIA AND ANIMAL MODELS

Loureiro Camila Marcelino[1], Corsi-Zuelli Fabiana[2], Fachim Helene Aparecida[2], Shuhama Rosana[2], Joca Sâmia Regiane Lourenço[3], Menezes Paulo Rossi[2], Del-Ben Cristina Marta[2], Louzada-Junior Paulo[2], Paulo Louzada-Junir*[,1]
*[1]University of São Paulo; [2]Ribeirão Preto Medical School, University of São Paulo; [3]School of Pharmaceutical Sciences, University of São Paulo,*

**Background:** Changes in glutamatergic system, specifically the ionotropic receptor N-methyl-D-aspartate (NMDAR), are involved in psychosis. NMDARs could be composed of two NR1 and two NR2 subunits. NR1 is one obligatory subunit and is the glycine binding site; and NR2 subunit contain the binding site for the neurotransmitter glutamate and have four different subtypes including NR2A-D. NR1 and NR2A-B are essential subunits of NMDAR, which are encoded by genes Grin1, Grin2A and Grin2B, and have been identified as candidate genes for psychiatric disorders. NMDARs dysfunction disrupts neural excitation and to contribute to the altered brain function underlying, especially in schizophrenia and other psychosis. The aims of this work were 1) to evaluate the expression of Grin1, Grin2A and 2B genes by qPCR of patients with first episode of schizophrenia compared with the siblings and controls; 2) to quantify the NR1 and NR2 subunits plasma concentrations by ELISA; 3) to evaluate the Grin1, Grin2A and 2B gene expression by qPCR in peripheral blood and animals brain tissue.
**Methods:** Participants will be 30 patients diagnosed with schizophrenia or schizophreniform disorder, including the shorter illness without substance addiction; those participants with siblings who agreed to participate (n = 30) and 30 controls, matched to patients by sex, age and education. Male Wistar rats were kept isolated (n = 10) or grouped (n = 10) from weaning for 10 weeks. After this period the animals were exposed to the Open Field and soon afterwards they were sacrificed, hippocampus and prefrontal cortex (PFC) were dissected to RNA extraction. RT-PCR was performed using probes and TaqMan mastermix to evaluate the mRNA expression. One-way ANOVA with a Bonferroni correction was used for statistical analysis.
**Results:** Humans: Regarding the glutamatergic system, none of the chosen genes were expressed in the studied sample. Animals: Isolated reared animals presented hyperlocomotion at the two first time bins (0–5 and 5–10 min) in periphery of the arena when compared to the grouped [0–5 min: $p = 0.025$; 5–10 min: $p = 0.002$], respectively. Decreased expression of Grin1 (31%), Grin2A (45%) and Grin2B (52%) were found in PFC of isolated animals when compared to grouped ($p < 0.05$), while no changes were found in the hippocampus.
**Discussion:** Changes in the expression of essential isoforms (NR1 and NR2) that make up NMDAR in PFC suggest abnormalities of glutamatergic neurotransmission in the pathophysiology of schizophrenia, corroborating recent studies. In addition, this study reinforces the validity of the social isolation rearing model from weaning with a better understanding of the mechanisms of NMDAR hypofunction and the influence of the environment on gene expression in this disorder.

### F12. INFLAMMATORY BIOMARKERS AND COGNITION IN FIRST EPISODE PSYCHOSIS: GENDER DIFFERENCES

Bibiana Cabrera Llorca*[,1], Miquel Bioque[1], Gisela Mezquida[1], Ana Gonzalez-Pinto[2], Mara Parellada[3], Julio Bobes[4], Antonio Lobo[5], Borja García-Bueno[6], Karina MacDowell[7], Carla Torrent[1], Pilar Saiz-Martinez[6], Juan Carlos Leza[7], Miquel Bernardo[8]
*[1]Neurosciences Institute, Hospital Clinic of Barcelona; [2]Hospital Universitario Araba, Universidad del País Vasco; [3]Hospital General Universitario Gregorio Marañon, Universidad Complutense de Madrid; [4]University of Oviedo; [5]Instituto Investigación Sanitaria Aragón, University of Zaragoza; [6]Complutense University, Instituto de Investigación Hospital; [7]Universidad Complutense de Madrid; [8]Hospital Clinic Barcelona, Universitat Barcelona*

**Background:** Cognitive impairment is considered a central feature of psychotic disorders, with an important impact on prognosis and functional outcome (Nuechterlein et al., 2011). Among the etiological explanations on psychosis, several hypotheses involving alterations in the immune /

inflammatory system have been proposed and recent research links these inflammatory processes with cognitive function, suggesting that the presence of inflammation is associated with poorer cognitive performance (Ribeiro-Santos et al., 2014, Cabrera et al., 2016). The study of the influence of gender on the possible association between inflammatory biomarkers and cognitive performance may favor the implementation of personalized treatments.
The aim of the study is to investigate the association between inflammatory biomarkers and gender-based cognition in a sample of first psychotic episode (FEP). A total of 92 patients with a FEP, 62 men and 30 women, recruited in five clinical centers were included. A neurocognitive assessment was performed and the expression of pro and anti-inflammatory mediators in peripheral blood mononuclear cells (PBMC) and plasma was measured.
**Methods:** In the neurocognitive evaluation the domains of attention, verbal and working memory and executive function were included. Other and clinical variables included the assessment of psychotic and affective symptoms, age, sex, educational level and socio-economic level. The expression of the proinflammatory mediators (NFκB, iNOS, COX-2, PGE2, NO-2 and TBARS) and anti-inflammatory (15d-PGJ2, PPARγ and IκBα) of the main intracellular inflammatory pathway was measured in PBMC and plasma.
**Results:** A correlation was made between inflammatory biomarkers and neurocognitive performance according to gender, and significant associations were selected to perform a subsequent hierarchical regression analysis. In the final model, only the expression of COX2 was associated with better performance in executive function in males ($B = 0.504$, $p = <0.001$) and the expression of NO2 to worse performance in working memory in women ($B = -0.911$; $p = 0.010$), after controlling the confounding factors. Men and women did not differ in any of the confusing variables.
**Discussion:** The expression of certain pro / anti-inflammatory components could have a differential effect according to the gender of patients with FEP. The expression of COX2 could be beneficial in the case of males, explaining part of the variability in executive function. On the contrary, the expression of NO2 could have a detrimental effect on working memory in women. The establishment of biomarkers linked to gender-based cognition could be useful for monitoring the course of cognitive decline and could guide treatment programs, providing tools to select a personalized approach.

### F13. TOWARDS MOLECULAR INSIGHTS INTO PSYCHIATRIC DISORDERS USING AFFINITY PROTEOMICS

David Just*[,1], Anna Månberg[1], Eva Lindholm Carlström[2], Janet Cunningham[2], Peter Nilsson[1]
[1]*Affinity Proteomics-Science for Life Laboratory, KTH-Royal Insititute of Technology;* [2]*Uppsala University*

**Background:** Numerous studies have shown a correlation between high autoantibody titers and subsequent autoimmune disease in patients with psychiatric disorders compared to healthy individuals. In this study we used a targeted affinity proteomics approach to investigate these autoantibody repertoires. We therefore obtained serum samples from patients diagnosed with various psychiatric disorders and compared these with samples of healthy volunteers. Additionally we used our approach to identify autoantibodies in post mortem brain tissue from patients diagnosed with schizophrenia.
**Methods:** In this study we utilized a well characterized cohort of patients with psychiatric disorder to study the autoantibody repertoire. From this sample set we analysed more than 600 serum in a first discovery approach. Based on the in-house screening and previous external published studies of autoantibodies within psychiatry we selected a set of 231 protein fragments from the Human Protein Atlas with a length of roughly 80 amino acids. With this selected panel we screened additional 130 post mortem brain tissue samples. Autoantibody profiling was performed using suspension bead array technology and IgG reactivity was measured in patients and controls.
**Results:** Our findings could indicate altered immune response in patients with psychiatric disorders compared to healthy controls. In our study we identified potential predictive autoimmune signatures. Those were presented with higher IgG reactivity in patients compared to healthy control samples.
**Discussion:** With our approach we were able to profile autoantibody repertoires in patients with psychiatric disorders. Additionally, we were able to use our approach to profile brain tissue using a multiplexed affinity proteomics approach. By further validating these putative autoimmunity targets, we could gain insights into the autoantigens associated to chronical mental illnesses.

### F14. REDUCED DURATION MISMATCH NEGATIVITY ASSOCIATED WITH DECREASED GLUTAMATE+GLUTAMINE LEVEL IN SUBJECTS AT CLINICAL HIGH-RISK FOR PSYCHOSIS

Yingying Tang*[,1], TianHong Zhang[1], Junjie Wang[1], LiHua Xu[1], Zhenying Qian[1], HuiRu Cui[1], Larry J. Seidman[2], Robert W. McCarley[4], Matcheri S. Keshavan[2], William S. Stone[2], Margaret Niznikiewicz[3], JiJun Wang[1]
[1]*Shanghai Mental Health Center, Shanghai Jiaotong University School of Medicine;* [2]*Harvard Medical School, Beth Israel Deaconess Medical Center;* [3]*Harvard Medical School, Veterans Affairs Boston Healthcare System, Beth Israel Deaconess Medical Center*

**Background:** Abnormal mismatch negativity (MMN), thought to be a putative marker of glutamatergic function, has been reported in non-Asian, first episode schizophrenia and clinical high-risk for psychosis (CHR) individuals as indicative of impairments in pre-attentive processes. However, reports of abnormal MMN in Asian populations are sparse, as well as its relationships to glutamate and γ–aminobutyric acid (GABA) levels in medial prefrontal cortex. The present longitudinal study explored MMN differences between CHR subjects who will and who will not remit, and its relationships with prefrontal glutamate and GABA levels.
**Methods:** All subjects participated in the ShangHai At-Risk for Psychosis (SHARP) program. CHR subjects met the criteria defined by the Chinese version of the Structural Interview for Prodromal Syndromes (SIPS). From the SHARP sample, 76 CHR subjects (41 male, age $18.63 \pm 5.02$ years) and 53 HC (31 male, age $17.72 \pm 3.18$ years) completed both MMN test and proton magnetic resonance spectroscopy (1H MRS) scans using a MEGA-PRESS sequence at their initial visit. CHR subjects were divided into remitted (37) and non-remitted (34) individuals based on their clinical symptoms and functional scores at a one-year follow up. Duration MMN amplitude was measured at electrodes F1/2, Fz, FC1/2, FCz, C1/2 and Cz. Concentrations of glutamate+glutamine (Glx) and GABA in the medial prefrontal cortex (mPFC) were quantified using the LCModel software (version 6.3-0I).
Repeated measures analysis of variance (ANOVA) with group (remitted CHR, non-remitted CHR and HC) as the between-group factor and electrodes (Fz, FCz and Cz) as the within-group factor were performed for the midline sites, and the ANOVA using F1/2, FC1/2 and C1/C2 with laterality (left and right hemisphere) as an additional within-group factor was performed for the lateral sites. Correlations of the dMMN amplitude (averaged over the 9 electrodes) and Glx and GABA concentrations were assessed by Pearson correlation tests for each group.
**Results:** There was a significant main effect of group ($F(2,121)=3.14$, $p<0.05$) for the midline fronto-central dMMN amplitude. Post-hoc tests showed that non-remitted CHR subjects had lower baseline dMMN amplitude ($-4.75 \pm 0.37\mu v$) than HC ($-5.92 \pm 0.30\mu v$, $p<0.05$), whereas dMMN in remitted CHR ($-5.22 \pm 0.36\mu v$, $p=0.41$) was comparable to dMMN in HC. The main effect of group was marginally significant at lateral sites ($F(2,121)=2.83$, $p=0.06$). DMMN amplitude in non-remitted CHR ($-4.67 \pm 0.37\mu v$) tended to be lower than those in HC ($-5.76 \pm 0.29\mu v$, p<0.1), while remitted CHR had dMMN amplitude (-5.11 ± 0.35μv, p=0.47) comparable to HC. There was no significant main effect of laterality or interaction of group × laterality.
In non-remitted CHR subjects, dMMN amplitude was significantly correlated with Glx level (r=-0.47, p<0.01) and with GABA level (r=-0.38, p<0.05) in the mPFC. However, the correlation of dMMN amplitude with Glx or GABA levels was not significant among either HC or remitted CHR.
**Discussion:** In line with previous studies, reduced dMMN amplitude distinguished between remitted and non-remitted CHR subjects, with remitted CHR not different from HCs. Our finding further supports the idea that reduced dMMN amplitude could be a candidate biomarker for predicting outcome in CHR. More importantly, we linked the reduced dMMN amplitude in non-remitted CHR to their Glx and GABA levels in mPFC, the region identified as one of dMMN sources (responsible for attention switching) thus supporting the idea that NMDA-mediated disruptions may play a key role in predicting psychosis and functional outcome.

### F15. DIFFERENTIAL EXPRESSION PATTERNS OF EPIDERMAL GROWTH FACTOR (EGF) AND IMMUNE SYSTEM MARKERS IN DORSOLATERAL PREFRONTAL (BA46) AND ORBITOFRONTAL (BA11) CORTICES IN SCHIZOPHRENIA AND MOOD DISORDER

Tharini Ketharanathan*[,1], Avril Pereira[2], Ian Everall[2], Suresh Sundram[3]
*[1]University of Melbourne; [2]Institute of Psychiatry, Psychology & Neuroscience, King's College London; [3]Monash University*

**Background:** Environmental risk factors that operate at maternal, foetal and post-natal levels, causing immune activation are known risk factors for schizophrenia. How this risk is transduced is unknown but one plausible disease mechanism may be through immune activation perturbing central nervous system growth factor systems, such as the epidermal growth factor (EGF) system critical to neuronal differentiation, maturation and plasticity, altering neurodevelopment. These interactions between EGF and immune systems may involve specific critical brain regions and not others.
**Methods:** The expression of candidate genes from EGF and immune systems and related signalling pathways, including ligands, receptors and intermediary molecules, were examined in post-mortem dorsolateral prefrontal (DLPFC) (n=114 genes) and orbitofrontal cortical (OFC) (n=105 genes) tissues, from schizophrenia and mood disorder patients and healthy controls (n=68), using the Fluidigm Biomark qRT-PCR platform. Data were analysed by ANOVA or corresponding non-parametric test (Kruskal-Wallis) in GraphPad Prism 6/7 statistical software and p values were adjusted for multiple testing (Benjamini-Hochberg).
**Results:** In DLPFC, 68 genes were significantly differently expressed between diagnostic groups. In comparison to healthy controls, 60 genes were differentially expressed in schizophrenia and 14 in the mood disorder group. Collectively these differentially expressed genes belonged predominantly to ERBB signalling and associated MAPK, PI3K and MTOR pathways and with immune pathways involving toll like receptor (TLR), TNF, nuclear factor kappa B (NFB), JAK-STAT, and complement signalling. Although there was some overlap the expression profiles in schizophrenia and mood disorder differed considerably. There were genes (n=15) with significantly lowered expression in both patient groups compared to controls which belonged predominantly to immune pathways such as TNF and TLR. However, 36 genes were differentially expressed between schizophrenia and mood disorder, all of them having lower expression in schizophrenia, predominantly representing pathways PI3K/MTOR, MAPK, TLR and TNF signalling via NFKB. Gene expression in EGF system signalling via MAPK and PI3K pathways, and interleukin signalling via JAK-STAT were significantly lower in schizophrenia than in mood disorder and healthy controls. ErbB4 was the only gene significantly elevated in a patient group (mood disorder) compared to the controls.
In comparison to DLPFC, only five genes out of the 105 examined were differentially expressed between the diagnostic groups in OFC, which belonged to NFB, TLR, JAK-STAT and growth factor signalling pathways. In comparison to healthy controls all were differentially expressed in schizophrenia and three genes in the mood disorder group. The expression of most genes was decreased in the patient groups compared to control subjects in both brain regions.
**Discussion:** We conclude that there is a prominent regional difference in the expression of EGF and immune system markers, identifying the DLPFC as a region of high activity for the interaction between these two systems relative to the OFC. In this region, the differing profiles of gene expression between schizophrenia and mood disorder involved EGF signalling pathways including PI3K/MTOR and MAPK along with immune pathways such as TNF, TLR and JAK-STAT signalling, possibly reflecting variant pathological processes involving immune and EGF system signalling between these sets of disorders.

### F16. GLUTAMATE AND GABA LEVELS IN ANTIPSYCHOTIC-NAÏVE SCHIZOPHRENIA PATIENTS ARE ASSOCIATED WITH TREATMENT OUTCOME AFTER 1.5 AND 6 MONTHS

Kirsten Bojesen*[,1], Brian Broberg[2], Kasper Jessen[3], Anne Sigvard[2], Karen Tangmose[2], Mette Ødegaard Nielsen[4], Egill Rostrup[5], Birte Glenthoj[3]
*[1]Center for Neuropsychiatric Schizophrenia Research (CNSR) and Center for Clinical Intervention and Neuropsychiatric Schizophrenia Research (CINS), Copenhagen University Hospital; [2]Center for Neuropsychiatric Schizophrenia Research; [3]Center for Neuropsychiatric Schizophrenia Research, and Center for Clinical Intervention and Neuropsychiatric Schizophrenia Research, University of Copenhagen; [4]CNSR, Copenhagen University Hospital; [5]Center for Neuropsychiatric Schizophrenia Research, and Center for Clinical Intervention and Neuropsychiatric Schizophrenia Research, University of Copenhagen, Glostrup University Hospital*

**Background:** Higher glutamate levels are found in the anterior cingulate cortex (ACC) of non-responder (NR) patients with schizophrenia in cross-sectional studies. However, it remains unclear if this reflects the pathophysiology of NR patients or the effect of antipsychotics and illness chronicity. Also, no previous study has assessed if levels of GABA in the ACC and glutamate in the thalamus are abnormal in NR patients from illness onset. To investigate this, we examined antipsychotic-naïve schizophrenia (SCZ) patients before and after treatment.
**Methods:** Longitudinal study of 38 initially antipsychotic naïve SCZ patients and 34 matched healthy controls (HC) assessed at baseline, after 1.5 months (NSCZ=29, NHC=33), and 6 months (NSCZ=26, NHC=28) of treatment. Patients were treated with aripiprazole for the first 1.5 months (open label). Hereafter, treatment could be modified. Responders (R) and non-responders (NR) were assessed using the Andreasen criteria. Glutamate spectra in the ACC and left thalamus were acquired with a PRESS sequence, and GABA spectra in the ACC with a MEGA-PRESS on a 3T MR scanner.
**Results:** First, the trajectory of glutamate/Cr and GABA/Cr levels were evaluated in SCZ patients and HC with a linear mixed model. In the left thalamus, a significant time*group interaction was observed (p=0.01) due to higher levels of glutamate/Cr in SCZ patients at baseline (p=0.03), but not after 1.5 and 6 months' treatment as compared with HC. In the ACC, a significant main effect of group was found for both glutamate/Cr (p=0.04) and GABA/Cr (p=0.003) due to lower levels in SCZ patients at all examinations, and the time*group interactions were non-significant.

Secondly, we investigated if baseline levels of glutamate/Cr and GABA/Cr differed in NR patients after 1.5 and 6 months' treatment using ANOVA. In the left thalamus, NR patients after both 1.5 and 6 months had significantly higher baseline glutamate/Cr compared with HC ($P_{1.5months}=0.03$ and $P_{6months}<0.05$), whereas R and HC did not differ. In the ACC, NR after 1.5 months showed a trend for lower GABA/Cr at baseline (p=0.06), and in NR after 6 months baseline GABA/Cr was significantly lower compared with HC (p=0.03), whereas R and HC did not differ. In the ACC, there was no baseline difference in glutamate/Cr of NR patients after 1.5 and 6 months compared with HC.

**Discussion:** The findings indicate increased glutamatergic turnover in the left thalamus and decreased GABAergic neurotransmission in the ACC in the pathophysiology of schizophrenia. Treatment normalises glutamate levels in the left thalamus, but does not affect GABA and glutamate levels in the ACC. Importantly, NR patients are characterized by more pronounced glutamatergic and GABAergic disturbances in the antipsychotic-naïve state. Compounds that modify glutamatergic and GABAergic neurotransmission might have therapeutic potential in this subgroup.

### F17. DIFFERENCES IN INTRACRANIAL VOLUME, IQ AND PSYCHOPATHOLOGY IN YOUNG OFFSPRING OF PATIENTS AFFECTED WITH SCHIZOPHRENIA OR BIPOLAR DISORDER

Neeltje van Haren[1], Setiaman Nikita*[,2], Martijn Koevoets[1], Heleen Baalbergen[1], Matthijs Vink[3], Esther Mesman[1], René Kahn[4], Manon Hillegers[5]

*[1]Brain Center Rudolf Magnus, University Medical Center Utrecht; [2]University Medical Center Utrecht; [3]Utrecht University; [4]Icahn School of Medicine at Mount Sinai; [5]Erasmus Medical Center, Sophia Children's Hospital*

**Background:** Offspring of patients with schizophrenia and bipolar disorder are at increased risk to develop psychopathology. It has been suggested that the development of these disorders may be a result of early neurodevelopmental abnormalities. The intracranial volume (ICV) is a direct marker for neurodevelopment in the early years, as ICV reaches 90% of its full size around the age of five. Interestingly, a smaller ICV is more consistently found in SZ patients, compared to controls, than in bipolar disorder patients. The offspring of these two patients group may provide important information on the putative neurodevelopmental trajectory underlying the development of these disorders. We compared ICV between offspring of at least one parent with SZ (SZo) or BD (BDo) and control offspring (Co) in relation to IQ and the presence of psychopathology.

**Methods:** A large sample of children and adolescents (8–18 years old; 54 SZo, 90 BDo, and 46 Co) was included. T1-weighted (3-Tesla) MRI brain scans were available for 146 participants. Group differences in ICV, global and local brain measures, psychopathology (K-SADS-PL, CBCL/6–18), IQ (WISC-III/WAIS-III), and their interactions were analyzed. FreeSurfer-5.3.0 was used for subcortical and cortical volume, cortical thickness, and cortical surface area estimations. Groups were compared using linear mixed effects modeling, corrected for family dependencies. FDR-correction was applied.

**Results:** Our main finding was that ICV was significantly smaller in SZo, compared to BDo and Co. IQ was significantly lower in both SZo and BDo, relative to Co, but could not explain the smaller ICV in SZo. ICV was also not explained by psychopathology, even though there was no significant difference in 'any psychopathology' between SZo and BDo. There was however some illness specificity as BDo had a higher prevalence of 'any mood disorder' as compared with Co, and SZo had a higher prevalence of major depressive disorder and autism spectrum disorders as compared with BDo and Co. After correcting for ICV, the cortex was significantly thinner in SZo compared to BDo and Co, and BDo had larger lateral ventricles than Co. Without correction for ICV, volumes of the total brain and gray matter were significantly smaller in SZo than in BDo and Co. Cortical white matter volume was significantly smaller in SZo as compared with Co.

**Discussion:** Irrespective of a lower IQ and increased presence of psychopathology in both high-risk offspring groups, abnormal early brain development, expressed as smaller ICV, differentiates offspring of patients with schizophrenia from offspring of patients with bipolar disorder and offspring of healthy parents. This suggest that the risk for schizophrenia is, in contrast to that in bipolar disorder, characterized by stunted brain development.

### F18. IS SCHIZOPHRENIA A MULTI-SYSTEM DISORDER? CONSIDERING NEUROLOGICAL, IMMUNE, CARDIOMETABOLIC, AND ENDOCRINE ALTERATIONS IN FIRST EPISODE PSYCHOSIS

Toby Pillinger*[,1], Enrico D'Ambrosio[1], Rob McCutcheon[1], Oliver Howes[2]

*[1]Institute of Psychiatry, King's College; [2]King's College London, Institute of Psychiatry; Medical Research Council Clinical Sciences Centre, Hammersmith Hospital, Imperial College*

**Background:** People with schizophrenia and related psychotic disorders show abnormalities in several organ systems in addition to the central nervous system (CNS). It is not yet known how the magnitude of disturbance in non-CNS systems compares with the magnitude of CNS disturbance in FEP. Here, we statistically compare effect sizes (ES) for non-CNS and CNS dysfunction in FEP, and consider whether schizophrenia is a multi-system disorder.

**Methods:** Pubmed was systematically searched from 1990 to May 2017 for meta-analyses examining non-CNS dysfunction in FEP, focusing on immune, cardiometabolic, and hypothalamic-pituitary-adrenal (HPA) systems. A parallel search was performed for meta-analyses examining representative CNS dysfunction in FEP, specifically neurophysiological, neurochemical and brain structural alterations. To statistically compare the magnitude of effect sizes (ES) between different CNS and non-CNS systems in FEP, data were extracted from case-control studies making up these meta-analyses, and meta-analyses repeated. For non-CNS parameters, random-effects meta-analyses were performed examining immune (cytokines, CRP, and lymphocytes), cardiometabolic (glucose/insulin and lipids), and HPA parameters (cortisol and prolactin). For CNS parameters, meta-analyses were performed examining brain structural, neurophysiological (Auditory P300 amplitude and latency, duration deviant mismatch negativity), and neurochemical parameters (N-acetylaspartic acid levels). Standardized mean differences between patient and control cohort parameters were used as the ES (Hedges adjusted g). As well as individual meta-analyses being run for each parameter as described, 6 separate sub-group meta-analyses were performed examining data for overall immune, cardiometabolic, HPA, brain structural, neurophysiological, and neurochemical systems. Sub-group summary effect size magnitudes were calculated by running a combined analysis of all studies assigned to a sub-group (e.g. to calculate the summary effect size magnitude for immune alterations, a single analysis was performed that combined IL-1b, sIL-2R, IL-6, TNFa, TGFb, CRP, and lymphocyte count data sets). Summary effect sizes for these 6 individual systems were statistically compared in a fixed effects model using a Wald-type test. This was also used to compare overall summary CNS and non-CNS effect sizes. Antipsychotic naïve sensitivity analyses were performed.

**Results:** Data were extracted for 165 studies comprising a total sample size of 13,440. The summary effect size for immune alterations (g=1.19; CI:0.82–1.56) was significantly greater than brain structural (g=0.40; P<.001) and neurochemical alterations (g=0.43; P<.001), and no different from neurophysiological alterations (g=0.80; P=0.05). The summary effect size for HPA alterations (g=0.68; CI:0.32–1.04) was not significantly different from brain structural (P=.14), neurophysiological (P=.54), and neurochemical (P=.22) alterations. The summary effect size for cardiometabolic alterations (g=0.23; CI:0.15–0.31) was significantly lower than neurochemical (P=.04), neurophysiological (P<.001) and brain structural alterations (P=.001). The overall summary effect sizes for non-CNS (g=0.58; CI:0.44–0.72) and CNS (g=0.50; CI:0.44–0.56) alterations were not significantly different (P=.28).
**Discussion:** These data indicate that there are robust alterations in non-CNS systems in psychosis, and that these are broadly similar in magnitude to a range of CNS alterations, indicating that psychosis involves multiple organ systems to a comparable degree from onset. We consider three models that could account for these findings and discuss implications for future research and treatment.

### F19. TELOMERE SHORTENING IN YOUNG PEOPLE WITH FIRST EPISODE PSYCHOSIS: A 12-MONTH FOLLOW-UP STUDY

David Fraguas*[,1], Sandra Recio[2], Covadonga M. Diaz-Caneja[2], Maria A. Blasco[3], Ana Carolina Moisés[3], Celso Arango[2]
*[1]Hospital General Universitario Gregorio Marañón; [2]Hospital General Universitario Gregorio Marañón, School of Medicine, Universidad Complutense; [3]Spanish National Cancer Research Centre (CNIO)*

**Background:** Short telomere length is a biomarker of cell oxidation and aging. Patients with first-episode psychosis (FEP) have been reported to have shorter telomeres than healthy controls (HC), suggesting that there is a premature and accelerated cellular aging in FEP. However, there are not data on longitudinal changes of telomere length in people with FEP relative to HC. We present preliminary results on 1-year longitudinal changes in peripheral blood mononuclear cells (PBMCs) telomere length and the proportion of PBMCs with short telomeres in young people with FEP and HC.
**Methods:** 16 young patients with FEP (43.8% female, mean age 17.9 years) and 21 young HC (61.9% female, mean age 16.6 years) were enrolled in the study. PBMCs telomere length and the proportion of PBMCs with short telomeres (i.e. <3kb) were determined using high-throughput quantitative fluorescence in situ hybridization (HT Q-FISH) at baseline (16 patients with FEP and 21 HC) and 12-month follow-up (4 patients with FEP and 4 HC).
**Results:** At baseline, we did not find significant differences in telomere length nor in proportion of PBMCs with short telomeres between FEP patients and HC. During the one-year follow-up, we found a significantly greater loss of telomere length (p=0.019; explained variance=69.7%) and a non-significantly trend for greater increase in the proportion of PBMCs with short telomeres (p=0.097; explained variance=45.5%) in patients with FEP than in HC.
**Discussion:** Telomere length changes during the first years of the illness can represent an early marker of accelerated cellular aging in patients with first-episode psychosis.

### F20. SEX-SPECIFIC STRUCTURAL AND FUNCTIONAL CIRCUIT DIFFERENCES IN YOUTH WITH PSYCHOSIS SPECTRUM SYMPTOMS

Grace Jacobs*[,1], Stephanie Ameis[1], Joseph Viviano[2], Erin Dickie[2], Anne Wheeler[3], Sonja Stojanovski[3], Aristotle Voineskos[1]
*[1]University of Toronto, CAMH; [2]CAMH; [3]University of Toronto, Hospital for Sick Children*

**Background:** Functional connectivity differences in the cortico-thalamic-striatal-cortical (CTSC) circuit, as well as altered subcortical region volumes have been observed in schizophrenia. In this study, structural and functional magnetic resonance imaging (MRI) were used in a large child and youth sample aged 11–21 years (n=1134) including children with psychosis spectrum (PS) symptoms (n=312) to further understanding of these biomarkers in youth outside of high risk groups and with a wider range of symptom severity.
**Methods:** Structural subregions of the thalamus and striatum were identified using the segmentation tool MAGeT Brain. Functional subregions were segmented based on functional connectivity with the 7 functional networks identified in Yeo et al, 2011. Average time series from functional subregions were correlated vertex-wide with cortical surfaces and Fisher Z transformed. FSL's PALM was used to examine differences and interactions between PS groups and sex. Age and in scanner motion (mean framewise displacement) were covaried for and a family wise error rate correction was applied. Structural subregion volume differences and interactions between PS groups and sex were investigated statistically using analyses of covariance (ANCOVA) with a false discovery rate of 5% correction for multiple testing. Age, intracranial volume, WRAT score and current medication use were covaried for.
**Results:** Sex-specific differences between PS and non-PS youth in structural subregion volumes were seen in both the striatum and thalamus. There was a persistent pattern of increased volumes in girls with PS symptoms, but decreased volumes in boys with PS symptoms compared to non-PS youth in the bilateral posterior putamen of the striatum (F=9.26, pFDR=0.006), higher order thalamic bilateral pulvinar (F=9.85, pFDR=0.004), left medial dorsal nuclei (F=7.42, pFDR=0.01), as well as first order thalamic left ventral posterior nucleus (F=6.47, pFDR=0.02), medial geniculate nucleus (F=10.03, pFDR=0.004) and bilateral lateral geniculate nuclei (F=5.7, pFDR=0.03). However, both PS girls and boys had increased nucleus accumbens volumes (t=2.66, pFDR=0.02). Decreased functional connectivity was found in PS youth between a striatal subregion in the right posterior putamen (corresponding to the dorsal attention network) and occipital areas (pFWE=0.005). This pattern was found to be driven by differences in specifically PS boys and not PS girls (pFWE=0.004).
**Discussion:** Multiple sex-specific structural differences between PS and non-PS youth were found in striatal and thalamic subregions. Hypoconnectivity between the striatal posterior putamen and occipital regions in PS boys overlap with structural increases in this subcortical volume in PS boys. Finding these early indicators is a key strategy to provide insight into neural mechanisms underlying the development of psychosis with the aim to improve and better target treatments.

### F21. ELECTROPHYSIOLOGICAL PARAMETERS OF SELECTIVE ATTENTION IN ADOLESCENTS WITH A FIRST EPISODE OF PSYCHOSIS: A COMPARISON WITH ADHD

Iris Selten*[,1], Jacob Rydkjaer[2], Anne Katrine Pagsberg[3], Birgitte Fagerlund[2], Birte Glenthoj[4], Jens Richardt Møllegaard Jepsen[4], Bob Oranje[5]
*[1]CINS & CNSR, Mental Health Centre Glostrup, University of Copenhagen; [2]CINS & CNSR, Psychiatric Center Glostrup; [3]Child and Adolescent Mental Health Services; [4]Center for Neuropsychiatric Schizophrenia Research (CNSR) and Center for Clinical Intervention and Neuropsychiatric Schizophrenia Research (CINS), Copenhagen University Hospital; [6]CINS and CNSR, University of Copenhagen/University Medical Center Utrecht*

**Background:** Neuropsychological deficiencies in attentional processes and filtering of information are shown by both patients with schizophrenia and Attention Deficit Hyperactivity Disorder (ADHD). Given that behavioral symptoms differ, differential neurophysiological processes are likely to be underlying each disorder. Deficiencies in early auditory processing measured by event-related potentials (ERPs) such as the P300 amplitude and mismatch negativity are suggested to be biomarkers for schizophrenia. Here we study if these electrophysiological processes are impaired in, and specific for, young individuals with a first episode psychosis (FEP), by directly comparing them with typically developing peers and adolescents with ADHD.
**Methods:** 27 FEP patients, 25 ADHD patients and 45 age and gender matched Healthy Controls (HC), all aged between 12 and 17 years old, were assessed for their N1, N2, P2, P3a, P3b, MMN and PN amplitudes in a selective attention paradigm (auditory oddball task).
**Results:** ADHD patients showed significantly smaller N2 and P3b amplitudes than HC, whereas FEP patients showed intermediate amplitudes. In addition, we found a second order interaction effect, indicating that the HC group had larger P2 amplitudes to attended deviant stimuli than to unattended deviant stimuli, whereas the response to non-attended deviant stimuli did not differ from that to non-attended standard stimuli. The ADHD group did not show this difference, in fact, they showed P2 amplitudes to unattended deviant stimuli that were larger than those to unattended standards. Interestingly, this effect was also found in the FEP group, although this only reached trend level ($p = 0.08$) of statistical significance. Post-hoc dividing the FEP group in patients with and without a diagnosis of schizophrenia, showed the same second order interaction effect in patients without schizophrenia as that in the ADHD subjects, while the interaction effect in patients with schizophrenia was identical to that of the HC. Furthermore, FEP patients without schizophrenia showed significantly smaller P3b amplitudes compared to FEP patients with schizophrenia and HC. Last, FEP patients with schizophrenia showed trend level significant smaller MMN amplitudes than HC ($p = 0.09$). No further significant group differences were found.
**Discussion:** FEP patients without schizophrenia showed impaired neurophysiological functioning compared to their counterparts with schizophrenia, who in turn showed similar performance as healthy controls. Given that use of medication did not differ between FEP patients with and without schizophrenia, our data suggest that other factors may play a role, such as comorbid symptoms. Since we found considerable overlap between FEP patients without schizophrenia and ADHD patients our current data do not support the theory that any of the investigated electrophysiological measures are useful as specific biomarkers for schizophrenia.

### F22. DYSLIPIDEMIA AND INFLAMMATORY MARKERS IN RELATION TO CLINICAL SYMPTOMATOLOGY IN PSYCHOTIC DISORDERS

Sherif M. Gohar*[,1], Ingrid Dieset[1], Nils Eiel Steen[1], Ragni H. Mørch[1], Trude S. Iversen[1], Vidar M. Steen[2], Ole A. Andreassen[1], Ingrid Melle[1]
*[1]NORMENT K.G. Jebsen Centre for Psychosis Research, Oslo University Hospital, Institute of Clinical Medicine, University of Oslo; [2]NORMENT K.G. Jebsen Center for Psychosis ResearchUniversity of Bergen*

**Background:** Although the role of lipid disturbances and inflammation in psychotic disorders have been demonstrated in a substantial body of research, the association between clinical symptomatology and these two important pathways has not been studied in detail. The main aim of this study was to investigate the associations between serum lipid levels [total cholesterol (TC), low density lipoprotein (LDL), triglyceride (TG)]; general or specific inflammatory markers [C-reactive protein (CRP), soluble tumor necrosis factor receptor 1(sTNF-R1), osteoprotegerin (OPG), interleukin 1 receptor antagonist (IL-1Ra)]; and clinical symptoms (positive, negative and depressive) in patients with psychotic disorders.
**Methods:** The sample is consisted of 652 participants divided in two groups: Schizophrenia, schizophreniform and schizoaffective, (schizophrenia group, N = 344); psychosis NOS, psychotic bipolar I, II and NOS, (non-schizophrenia group, N = 308) recruited consecutively between 2003 and 2015 from five major hospitals in Oslo, Norway, as part of Thematically Organized Psychosis (TOP) Study. The Regional Committee for Medical Research Ethics approved the study. Demographic, clinical and medications data were obtained by clinical interviews and from medical records. SCID-I was used for diagnosis in addition to Positive and Negative Syndrome Scale (PANSS) and Calgary Depression Scale for Schizophrenia (CDSS) to assess symptoms severity. Bivariate and multivariate analyses were performed to evaluate associations between symptom profiles, lipid levels and inflammatory markers.
**Results:** Schizophrenia group showed higher levels of TC and LDL compared to non-schizophrenia group after adjusting for age, gender, BMI, smoking, and medications. TC and LDL were positively correlated with depression, whereas TG and LDL were positively correlated with negative symptoms. CRP and OPG were significantly associated with higher levels of TC and LDL. While, sTNF-R1 showed significant positive correlation only with TG. In multivariate regression, higher LDL was significantly associated with higher age, BMI, depressive severity in addition to two inflammatory markers CRP and OPG.
**Discussion:** The findings of this study highlight the importance of understanding the interaction between inflammatory markers and lipid, and their relation to clinical profile especially depression in psychotic disorders

### F23. LOW-GRADE INFLAMMATORY PROFILE IN FIRST-EPISODE PSYCHOSIS: RESULTS FROM THE STREAM STUDY IN BRAZIL

Fabiana Corsi-Zuelli*[,1], Camila Loureiro[1], Fachim Helene Aparecida[1], Rosana Shuhama[1], Menezes Paulo Rossi[1], Paulo Louzada-Junior[1], Del-Ben Cristina Marta[1]
*[1]University of São Paulo, Ribeirão Preto School of Medicine*

**Background:** The inflammatory hypothesis of schizophrenia suggests that abnormalities in inflammatory factors may contribute to the onset of this disease early in adulthood. Nevertheless, no study has investigated possible effects of shared environment by looking at cytokine levels in healthy siblings of first-episode psychosis patients (FEP). The aim of this study was to investigate inflammatory cytokine (IL-6, IL-10, TNF-a) abnormalities in a sizeable epidemiological-based sample of FEP patients, their healthy siblings and population-based controls.
**Methods:** This study is part of the epidemiological investigation "Schizophrenia and Other Psychoses Translational Research: Environment and Molecular Biology" (STREAM), conducted in Ribeirão Preto (São Paulo, Brazil) and surrounding area, which is part of the international consortium "European Network of National Schizophrenia Networks Studying Gene-Environment Interactions" (EU-GEI). We recruited a total sample of 507 participants, composed by 166 FEP patients (64% males; mean age: $30.3 \pm 12.2$), 76 siblings (30.3% males; mean age: $31.5 \pm 11.0$) and 265 population-based controls (50.2% males; mean age: $31.7 \pm 11.2$). Plasma cytokines IL-6, TNF-a and IL-10 were analysed by Multiplex Bead Array (Luminex xMap technology). We performed the multivariate general linear model (GLM) analysis with age as covariate, cytokines as dependent variables and diagnostic group and sex as explanatory variables.
**Results:** We found significant differences between the three groups [$F(6,994) = 10.864$; $p < 0.001$] in the plasma concentration of all the three cytokines ($p < 0.001$), independent of the sex of the participants ($p = 0.115$; interaction diagnosis & sex $p = 0.115$). FEP patients had significantly higher levels of IL-6, TNF-a, and IL-10 when compared to controls ($p < 0.001$ for all). When compared to their siblings, patients had higher levels of both TNF-a and IL-10 ($p < 0.05$ for both), and a trend to IL-6 ($p = 0.058$), whereas siblings did not differ from controls for any of the three cytokines analysed ($p > 0.05$ for all).
**Discussion:** This is the first study conducted in the South hemisphere to demonstrate the low-grade inflammatory profile in FEP patients, compared to their siblings and community-based controls. The fact that IL-6, TNF-a and IL-10 are all higher in the FEP group than their healthy siblings and community-based controls suggests the synergism of individual vulnerability factors in the development of this inflammatory profile.

### F24. OMEGA-6 PUFA METABOLISM DISTURBED IN PHOSPHO- AND SPHINGOLIPIDS IN NEUROLEPTIC-NAÏVE FIRST-EPISODE SCHIZOPHRENIA PATIENTS – A FATTY ACID PROFILING STUDY IN FIVE LIPID FRACTIONS

Kerstin Langbein*[,1], Christian Fleischer[1], Katrin Kuhnt[2], Stefan Smesny[1]
*[1]Jena University Hospital; [2]Friedrich-Schiller-University Jena*

**Background:** Alterations of polyunsaturated fatty acid (PUFA) levels are a well-replicated finding in schizophrenia research. There is however a controversy about the origin of this abnormality and its importance in the pathogenesis of schizophrenic illness. To investigate the influence of nutrition, in this study we investigate different aspects of fatty acid metabolization in a cohort of neuroleptic naïve first-episode schizophrenia patients (FEP) and a group of healthy controls (HC) matched for age and gender.
**Methods:** In 33 FEP (25.8 ± 4.8y, male 60.6% 20/33) and 32 HC (24.9 ± 4.6y, male 53.1% 17/32) fatty acid profile was investigated by gas chromatography in blood plasma lipids of the triacylglycerol (TAG) fraction (closely related to fat consumption within recent days, rich in SFA, MUFA (~50%), and LA (C18:2n6)), the cholesterol ester (CE) fraction (dependent on fat consumption within recent days, rich in LA (C18:2n6)(~51%), SFA and PUFA), and the plasma phospholipid (PL) fraction (reflecting fat consumption of the last weeks to month, rich in SFA (~50%), AA (~7%), and LA (C18:2n6). In erythrocyte membranes (sensitive to fat consumption within weeks to month), fatty acid profile was investigated in phospholipids of the phosphoethanolamin (PE) fraction (rich in PUFA (~45%), AA (C20:4n6) and SFA) and of the sphingomyelin (SM) fraction (rich in long chain SFA (>70%) and MUFA (including NA C24:1c15). Psychopathology was assessed using the PANSS, BPRS-E and SCL-90-R ratings. Statistical analysis included multi- and univariate ANOVA, non-parametric tests and correlation analysis.
**Results:** In the plasma PL fraction and in the lipid fractions of erythrocyte membranes (PE, SM) that are less influenced by recent nutrition, patients showed generally reduced omega-6 PUFA levels, particularly in terms of AA in the SM fraction. While PUFA of the PL and PE fraction were positively correlated in HC, this was not the case in the FEP group.
**Discussion:** Our results support the previous finding of a general omega-6 PUFA deficit in FEP. The decrease points towards an endogenous regulation deficit that is independent of recent nutrition, might affect the metabolism of grey and white matter structural components, and could cause alterations of AA downstream functions. While correlation analysis in HC strongly suggests that nutrition and supplementation have the potential to influence PUFA availability, inner transport and metabolization pathways seem to be disturbed in FEP.

### F25. NEURAPRO REVISITED: INCREASES IN LONG-CHAIN OMEGA-3 FATTY ACIDS IMPROVE FUNCTIONAL AND SYMPTOMATIC OUTCOMES IN ULTRAHIGH RISK PATIENTS

G. Paul Amminger*[,1], Barnaby Nelson[1], Hok Pan Yuen[1], Connie Markulev[1], Miriam R. Schäfer[1], Monika Schlögelhofer[2], Nilufar Mossaheb[2], Stephan Smesny[3], Ian Hickie[4], Gregor Berger[5], Eric Chen[6], Lieuwe de Haan[7], Dorien H. Nieman[7], Merete Nordentoft[8], Anita Riecher-Rössler[9], Swapna Verma[10], Maximus Berger[1], Andrew Thompson[11], Alison Yung[12], Patrick D. McGorry[1]
*[1]Orygen, National Centre of Excellence in Youth Mental Health; [2]Medical University of Vienna; [3]University Hospital, Jena; [4]University of Sydney; [5]Child and Adolescent Psychiatric Service of the Canton of Zurich; [6]University of Hong Kong; [7]Academic Medical Center, Amsterdam; [8]Psychiatric Centre Bispebjerg; [9]Psychiatric University Clinics Basel; [10]Institute of Mental Health, Singapore; [11]Warwick Medical School; [12]Institute of Brain, Behaviour and Mental Health, University of Manchester*

**Background:** The NEURAPRO multicentre randomised controlled trial (RCT) of long-chain polyunsaturated omega-3 fatty acids (ω-3 PUFAs) ('fish oil') in combination with high-quality psychosocial intervention (cognitive behavioural case management [CBCM]) vs. placebo in combination with CBCM in young people at ultrahigh risk (UHR) of psychosis showed that the group allocated to fish oil had no clinical benefits over the placebo group. However, a limitation of the trial was that adherence with the study medication was relatively low. Furthermore, although RCTs are placed at the top of the evidence hierarchy, this methodology has limitations in fish oil RCTs, since the test agent is not only present in the intervention group, but ω-3 fats are present in the diet and in the tissues of all participants. A biomarker analysis of ω-3 changes during the trial can ultimately determine the efficacy of ω-3 supplementation in this trial.
**Methods:** The NEURAPRO study was conducted from March 2010 to September 2014, in 10 specialized early psychosis treatment services in Australia, Asia, and Europe. In this study of 304 young people at UHR for psychotic disorders, 153 (50.3%) were allocated to ω-3 PUFAs and 151 (49.7%) to placebo. In all, 139 (45.7%) were male; mean (SD) age was 19.1 (4.6) years. The primary outcome was transition to psychosis assessed with the Comprehensive Assessment of the At-Risk Mental State. Secondary outcomes were levels of psychopathology and functioning assessed by the Brief Psychiatric Rating Scale (BPRS), the Scale for the Assessment of Negative Symptoms (SANS), the Montgomery Asberg Depression Rating Scale (MADRS), the Young Mania Rating Scale (YMRS), the Social and Occupational Functioning Assessment Scale (SOFAS), and the Global Functioning: Social and Role scales). Levels of ω-3 PUFAs in fish oil, eicosapentaenoic acid (EPA) and docosahexaenoic acid (DHA) (amongst other fatty acids) were measured as percentage of total fatty acids in erythrocytes at baseline and at month 6 (end-of-intervention). We examined changes in cell membrane levels of EPA and DHA, as measures of ω-3 intake independent of source. Data were analysed as a single cohort. Cox proportional hazards models and linear regression analyses were used to examine relationships between the ω-3 index (EPA+DHA) with clinical outcomes at month 6 and 12.
**Results:** When analysed as a single cohort, no association was observed between the ω-3 index and transition to psychosis at any follow-up time point but increase of the ω-3 index was found significantly related with most of the functional and symptomatic measures at month 6 and 12, in linear regression models adjusting for relevant baseline factors (i.e., functioning, psychopathology, ω-3 index and smoking). The models revealed consistent results, with low functioning or high psychopathology at baseline, low levels of ω-3s at baseline and increase of the ω-3 index independently predicting clinical improvements at in this sample.
**Discussion:** In contrast to our RCT analysis, this study using biomarkers shows that increase in erythrocyte ω-3 PUFAs may improve clinical outcomes of UHR patients. The results also imply that people with low DHA and EPA levels may benefit more from supplementation with fish oil. The analysis also highlights shortcomings of the RCT design in situations when the tested intervention is available outside the study.

### F26. THE NATURE OF CLINICAL HIGH-RISK SYMPTOMS: NEW INSIGHTS GAINED FROM AGE EFFECTS

Frauke Schultze-Lutter*[,1], Chantal Michel[2], Stefanie Schmidt[2]
*[1]Heinrich-Heine University Düsseldorf; [2]University Hospital of Child and Adolescent Psychiatry, University of Bern*

**Background:** Early detection of psychosis is an important topic in psychiatry and is involving ever younger patient groups. Yet, developmental issues are still under researched. Thus, we examined risk symptoms and criteria in 8-40-year-olds from the general population.

**Methods:** Clinical high-risk symptoms, i.e. attenuated and transient psycvhotic symptoms (APS, BIPS) as well ascognitive and perceptive basic symptoms (BS), were assessed by well-trained psychologists performed assessments of risk symptoms, using established interviews. Differentiating between perceptive and non-perceptive/cognitive phenomena, impact of age groups on risk symptoms and their clinical significance (current psychosocial functioning deficits or non-psychotic DSM-IV axis-I disorder) was assessed by logistic regression analyses.
**Results:** Altogether, 9.9% of interviewees (N=689) reported APS, and 18.1% BS; 1.3% met APS, 3.3% COPER and 1.2% COGDIS criteria. For APS, an age effect was detected around age 16: compared to 16-40-year-olds, 8-15-year-olds reported more perceptive APS and lesser clinical significance of non-perceptive APS. Similar age effects of BS on prevalence and clinical significance that differed between perceptive and cognitive BS and followed brain maturation patterns were also detected: around age 18 for perceptive and in the early twenties for cognitive BS.
**Discussion:** These findings strongly suggest differential developmental factors affecting prevalence and clinical significance of APS and BS: While neurocognitive maturation might influence the presence of APS, brain maturation seems to influence the presence of BS. These findings emphasize the need to address the differential effects of perceptive and non-perceptive risk phenomena, and their interaction with age, also in terms of conversion to psychosis, in future studies.

### F27. LATENT PROFILES OF DEVELOPMENTAL SCHIZOTYPY IN THE GENERAL POPULATION: ASSOCIATIONS WITH CHILDHOOD TRAUMA AND FAMILIAL MENTAL ILLNESS

Melissa Green*[,1], Richard J. Linscott[2], Kristin R. Laurens[3], Stacy Tzoumakis[4], Kimberlie Dean[4], Johanna C. Badcock[5], Vaughan J. Carr[4]
*[1]University of New South Wales Prince of Wales Hospital; [2]University of Otago; [3]Australian Catholic University; [4]University of New South Wales; [5]University of Western Australia*

**Background:** Latent liability for schizophrenia (schizotypy) is expressed in various combinations of cognitive, psychological, and behavioural characteristics evident in the general population. Historical models propose that distinct classes of individuals expressing different forms of schizotypy may represent manifestations of differential levels of genetic and environmental risk for schizophrenia (or related illness). However, there has been little investigation of developmental models of schizotypy in childhood. Here, we sought to delineate latent profiles of schizotypy among children aged 11–12 years, and to examine associations between emerging schizotypal profiles and parental history of mental illness (as a proxy for genetic risk), early life trauma, and childhood contact with health services for mental illness up to age 13 years.
**Methods:** Latent profiles of schizotypy were distinguished among 22,137 children (mean age=11.9 years) for whom intergenerational records of health service contact for mental illness and child protection reports were linked to the Middle Childhood Survey (MCS) within the NSW Child Development Study.[1] Selected MCS items were used to index schizotypy across six domains (Unusual Experiences, Cognitive Disorganisation, Impulsive Non-conformity, Introversion, Dysphoria and Self-Other disturbance). Using Latent Profile Analyses (LPA), four groups emerged according to patterns of expression across these domains; membership of three putative schizotypy groups were examined in relation to the likelihood of being exposed to childhood maltreatment and parental mental illness, and the child's own mental illness up to age 13 years, relative to the no risk group.
**Results:** Four classes emerged from the LPA: (1) 'schizotypy' (n=1323; 6%); (2) 'dysphoric pseudo-schizotypy' (n=4261, 19%); (3) 'introverted pseudo-schizotypy' (n=4473; 20%) and; (4) 'no psychopathology' (no-risk, n=12,080; 55%). Children in the schizotypy group had the greatest odds of being the subject of a child protection report (OR=2.9, 95% CI 2.6–3.3) and in contact with health services for mental illness by age 13 years (OR=2.7, 95% CI 2.2–3.3), relative to the no-risk group. The odds of child protection reports and childhood mental disorders were smaller, yet significantly increased, among dysphoric pseudo-schizotypy (ORs=1.9 and 1.8, respectively) and introverted pseudo-schizotypy (ORs=1.7 and 1.4, respectively), relative to the no-risk group. Parental mental illness exposure was greatest among the schizotypy (OR=2.3, 95% CI 2.0–2.6) subgroup, and was also increased in dysphoric pseudo-schizotypy (OR=1.6, 95% CI 1.5–1.8) and introverted pseudo-schizotypy (OR=1.4, 95% CI 1.3–1.5), relative to the no-risk group.
**Discussion:** We provide evidence for distinct subtypes of children expressing different forms of schizotypy among a large Australian sample from the general population. The subgroup of children labeled 'schizotypy' (6%) characterized by high levels of cognitive disorganisation, impulsive non-conformity, introversion, and self-other disturbance may be at highest risk for developing schizophrenia or other mental illness in adulthood, and had a greater likelihood of childhood maltreatment and parental mental illness history, than other 'pseudo-schizotypy' groups.
**Reference:**
1. Carr, V.J., Harris, F., Raudino, A., Luo, L., Kariuki, M., Liu, E., Tzoumakis, S., Smith, M., Holbrook, A., Bore, M., Brinkman, S.A., Lenroot, R.K., Dix, K., Dean, K., Laurens, K.R., Green, M.J. (2016) Cohort Profile: The New South Wales Child Development Study (NSW-CDS) – An Australian multi-agency, multi-generational, longitudinal record linkage study. 6:e009023 doi:10.1136/bmjopen-2015–009023.

### F28. PROGRESSIVE POST-ONSET REORGANISATION OF MRI-DERIVED CORTICAL THICKNESS IN ADOLESCENTS WITH SCHIZOPHRENIA

Lena Palaniyappan*[,1], Anthony James[2]
*[1]University of Western Ontario; [2]University of Oxford*

**Background:** Cortical thickness changes continuously throughout healthy adolescence reflecting ongoing maturation. In schizophrenia, distributed abnormalities in cortical maturation are suspected. To study if these distributed changes are a result of a co-ordinated process, we investigated the structural covariance among the longitudinal post-onset thickness changes that occur across various brain regions in adolescent-onset schizophrenia.
**Methods:** 19 healthy adolescents and 18 age-matched patients with early-onset schizophrenia were scanned twice (~2 years' interval). The rate of change in cortical thickness was estimated both at lobar and sulcogyral level. Group level structural covariance was studied using a graph theoretical framework.
**Results:** At baseline, patients had distributed reduction in cortical thickness compared to controls, though this deviation was abolished over the next 2 years. Occipital cortex had a significantly deviant rate of change in patients (0.8% increase per year) compared to controls (2.5% thinning/year). Patients had a significant increase in covariance of right anterior insula and calcarine sulcus with rest of the brain.
**Discussion:** Post-onset structural changes in EOS are not a result of random, mutually independent processes. A spatially interconnected reorganization process, distinct from normal maturational events may underlie these distributed changes.

### F29. HIGH-RISK SYMPTOMS FOR PSYCHOSIS IN ADOLESCENTS AND ITS RELATIONSHIP WITH FAMILY BURDEN

Olga Puig-Navarro*[,1], Elena De la Serna[2], Jordina Tor[3], Anna Sintes[3], Gisela Sugranyes[1], Marina Redondo[1], Marta Pardo[3], Montse Dolz[3], Inmaculada Baeza[1]
*[1]Hospital Clinic of Barcelona; [2]Centro de Investigación Biomédica en Red de Salud Mental; [3]Hospital St Joan de Déu*

**Background:** High-risk symptoms for psychosis (HRS) and substantial functional impairment occurs early in the course of psychosis (Fusar-Poli et al., 2015). Many patients with HRS are adolescents who are still living at home and are highly reliant on their relatives for support. Objectives: (1) To compare the family burden of caregivers of adolescents with HRS with carers of an age and gender matched healthy control group (HC), (2) to examine the relationships between different family burden aspects and high-risk symptoms for psychosis in the HRS sample.
**Methods:** Sample: 68 HRS subjects (15.3 ± 1.7 years, 66% females) and 42 HC subjects (15.5 ± 1.5 years, 66% females) from a prospective longitudinal study including help-seeking subjects who met HRS criteria (Child and Adolescent Psychiatry and Psychology departments of Hospital Clínic and Sant Joan de Déu, Barcelona, Spain). Inclusion criteria: age 10–17 years, meeting criteria for 1) attenuated positive or negative symptoms in the previous 12-months, 2) brief intermittent psychotic symptoms, 3) first or second degree relative with schizophrenia or schizotypical disorder plus impairment of functioning. Exclusion criteria: IQ<70, having a diagnosis of ASD. For HC subjects, exclusion criteria were having 1st or 2nd degree familiar with a psychotic disorder; a diagnosis of ASD and/or IQ<70. Instruments: the Semistructured Interview for Prodromal Syndromes and Scale of Prodromal Symptoms (SIPS/SOPS), the Hamilton Depression Scale and the Young Mania Scale for affective symptoms, a cognitive battery and the Caregiver Burden Inventory (CBI) which is a measure of family burden that has been validated in first-episode patients (McCleery et al., 2007). Caregivers' responses are rated on a Likert scale from 0 (not at all descriptive) to 4 (very descriptive) and distributed in 5 factors: Time-Dependence Burden (T-Db), Developmental Burden (Db), Physical Burden (Pb), Social Burden (Sb), and Emotional Burden (Eb). High scores indicate greater perceived burden.
**Results:** HRS and HC subjects did not significantly differ in age (t=0.68, p=0.497) and sex (X2=0.003, p=0.958). Intellectual quotient was higher in HC (mean=105.4 ± 11.28) than in HRS subjects (98.63 ± 14.27, t=2.53, p=0.013). Mean scores of high-risk symptoms in HRS subjects were higher than in HC subjects (t>-9.35, p<0.001): positive: 9.12 ± 4.76, negative:11.16 ± 5.49, disorganitzation:4.96 ± 3.03, general: 8.22 ± 3.83, and total symptoms:33.24 ± 12.59. HRS subjects had also higher scores in depressive (10.54 ± 7.54, t=-9.75, p<0.001) and manic symptoms (3.61 ± 4.53, t=-5.10, p<0.001). Caregivers of HRS subjects showed higher scores than caregivers of HC in all CBI subscales (t>-5.59, p<0.001; T-Db: 6.36 ± 5.01 vs 1.02 ± 1.60, Db: 7.42 ± 6.51 vs 0.45 ± 1.23, Pb: 7.00 ± 6.13 vs 0.58 ± 1.80, Sb: 4.77 ± 4.66 vs 0.64 ± 1.95, Eb: 4.86 ± 4.64 vs 0.93 ± 2.66). Time-Dependence burden reported by caregivers of HRS patients was significantly correlated with the SOPS total score (r=0.303, p=0.014) and with the negative SOPS subscale score (r=0.308, p=0.012). The relationship between negative SOPS symptoms and time-dependence burden remained after controlling for affective symptoms (F=5.07, p0.028) and intelligence quotient (F=7.27, p=0.009). This factor represents objective aspects of burden arising from demands on the caregiver's time.
**Discussion:** Caregivers of adolescents meeting criteria for HRS showed high perceived burden compared with caregivers of healthy adolescents. Time-dependence burden reported by caregivers was related to negative prodromal symptoms of HRS subjects. These findings highlighted that family burden occurs early in the course of psychosis. Acknowledgments: ISC-III/FIS, FEDER.

### F30. SMARTPHONE APPLICATION "ROBIN": FEASIBILITY, ENGAGEMENT AND SATISFACTION OF A SMARTPHONE APPLICATION APPROACH TO SUPPORT TREATMENT OF (ATTENUATED) PSYCHOTIC SYMPTOMS IN ADOLESCENTS

Nina Traber-Walker*[,1], Sibylle Metzler[1], Miriam Gerstenberg[1], Susanne Walitza[1], Maurizia Franscini[1]
*[1]University Hospital Zurich*

**Background:** There is increasing interest in using mobile technologies such as smartphones application in mental health care. First research results from the use of smartphone applications in the treatment of psychotic disorders are promising. Current analysis showed, that especially young people would be interested in smartphone applications within treatment settings. However, there is a lack of investigations in this population. There is also little known about mobile technologies in the work with attenuated psychotic symptoms. To address these gaps, we developed "Robin", a specific smartphone application to support the therapy of adolescents with attenuated or full-blown psychotic symptoms. The smartphone application targets medication adherence, real-time symptom assessment and provides help coping with symptoms and stressful situations in daily life.
**Methods:** Based on existing literature and our clinical expertise within a specialized outpatient care for adolescents with (attenuated) psychotic symptoms, a first modular version of the app was developed and adapted after first pilot investigations with patients (N=7, Age 14–18) and therapists (N=10). Participants of the pilot investigation completed a questionnaire regarding usability and acceptance of the application. Furthermore, we investigated how the patients used the application in their daily life by analyzing the user data from the application. In September 2017, the development of the smartphone application has been finalized and we have started with a systematic clinical evaluation study for testing the efficiency of the app. The application is only used in combination with psychotherapy in our university hospital for child and adolescent psychiatry.
**Results:** The data from our pilot investigation showed, that "Robin" was accepted by clinicians and patients. All clinicians said they would like to use the application to enrich their therapeutic approaches. All patients in the pilot project used the application in their daily life. Especially modules with information about symptoms and coping strategies were frequently used. Since September 2017, first patients have been included to the systematic evaluation study. In Florence 2018, we will present first data from this study about feasibility, engagement and subjectively perceived benefit of the smartphone application.
**Discussion:** The first feedbacks from the pilot investigation were encouraging. The findings were used to improve and adapt the application. Since September 2017, the application is used in psychotherapy and an evaluation study has started. This is one of the first clinical trials to test the efficacy of a specific application developed for adolescents with psychotic and with attenuated psychotic symptoms.

### F31. POLYGENIC RISK SCORES AND EARLY RISK ENDOPHENOTYPES IN CHILDREN AT GENETIC RISK OF SCHIZOPHRENIA AND BIPOLAR DISORDER: IMPLICATIONS FOR THE DEFINITION OF THE CHILDHOOD RISK STATUS

Thomas Paccalet*[,1], Alexandre Bureau[2], Elsa Gilbert[3], Nicolas Berthelot[4], Pierre Marquet[3], Sébastien Boies[5], Valérie Jomphe[5], Daphné Lussier[5], Michel Maziade[3]
*[1]CIUSSS de la Capitale-Nationale - CERVO Brain Research Center; [2]Laval University; [3]Université Laval & CERVO Brain Research Centre; [4]Université du Québec à Trois-Rivières; [5]CERVO Brain Research Centre*

**Background:** Polygenic risk scores (PRS) of schizophrenia (SZ) or bipolar disorder (BD) are derived from genomewide association studies discriminating unrelated patients from controls. We have recently shown that both the SZ PRS and the BD PRS also distinguished affected patients from their non-affected adult relatives in a familial sample.[1] Furthermore, the association of the SZ PRS with BD subjects and, reciprocally, of the BD PRS with SZ subjects support the shared susceptibility for these diseases.[1] Importantly, new studies suggest that PRS would also distinguish the offspring at genetic risk from controls[2] and may be associated with psychotic-like experiences and negative symptoms in adolescents of the general population.[3] Little is known though about the contribution of the PRS in the risk prediction in children at genetic risk.
Our group and others have shown that the risk trajectory of high-risk children (HR) born to an affected parent can be characterized by their risk endophenotypes, i.e. specific cognitive deficits and psychotic-like or mood-like experiences in childhood that flag the neurodevelopmental origin of the illness. Children at risk accumulate these risk endophenotypes along their developmental trajectory and this aggregation is a predictor of later transition to illness.[4,5]
We hypothesized that since the PRS is a reflection of the genomic liability to illness, it would consequently relate to risk endophenotypes and their aggregation in children at risk. Our objectives were to evaluate i) the power of PRS to discriminate children at risk from healthy controls and, ii) the association of SZ and BP PRS to early risk endophenotypes in these children.
**Methods:** The sample comprised 70 HR from the Eastern Quebec Kindred Study of multigenerational families densely affected by SZ and BD and 894 healthy controls from the CARTaGENE project. Whole genome SNP genotyping was performed from blood samples. Calculation of PRS was made according to our previous report.[1] All HR were characterized using 4 established risk indicators[4]: cognitive impairments, psychotic-like experiences, childhood non-psychotic Axis 1 DSM diagnoses and episodes of poor functioning. Stratification of the HR by the presence of childhood trauma was also performed.
**Results:** PRS distinguished HR from healthy controls (p<.05). Significant associations of SZ PRS and risk endophenotypes were detected for psychotic-like experiences (relative risk RR=1.4, p=.034) and, when stratifying for trauma, for the speed of processing cognitive domain (p=.049). Importantly, PRS was significantly higher in HR who aggregated psychotic-like experiences and axis 1 diagnoses (RR=3, p=.01), and a trend was detected with the aggregation of cognitive deficits, psychotic-like experiences and axis 1 diagnoses (p=.08).
**Discussion:** PRS were associated with individual risk endophenotypes and with the aggregation of risk endophenotypes in children born to an affected parent. These results call for further study on the exact contribution to the childhood risk status of the genomic susceptibility indexed by PRS and the combination of risk endophenotypes. Considering that the clinically high-risk (CHR) status can be defined as a late phase of risk,[6] the accumulation of risk indicators in childhood, including PRS and risk endophenotypes, document this early life period as the optimal timing for early intervention approaches.
**References**:
1. Boies et al., Am J Med Genet B Neuropsychiatr Genet, 2017
2. Fullerton et al., Am J Med Genet B Neuropsychiatr Genet, 2015
3. Jones et al., JAMA Psychiatry, 2016
4. Paccalet et al., Schizophr Res, 2016
5. Maziade, N Eng J Med, 2017
6. Seidman & Nordentoft, Schizophr Bull, 2015

### F32. DIFFERENCES BETWEEN YOUTH AT CLINICAL HIGH-RISK FOR PSYCHOSIS WHO DO NOT TRANSITION TO PSYCHOSIS: THE NORTH AMERICAN PRODROME LONGITUDINAL STUDY (NAPLS-2)

Olga Santesteban-Echarri*[,1], Jacqueline Stowkowy[1], Scott W. Woods[2], Lu Liu[1], Kristin S. Cadenhead[3], Tyrone D. Cannon[2], Barbara A. Cornblatt[4], Thomas H. McGlashan[2], Diana O. Perkins[5], Larry J. Seidman[6], Ming T. Tsuang[7], Elaine F. Walker[8], Carrie E. Bearden[9], Daniel H. Mathalon[10], Jean M. Addington[1]
*[1]Hotchkiss Brain Institute, University of Calgary; [2]Yale University; [3]University of California San Diego; [4]Zucker Hillside Hospital; [5]University of North Carolina, Chapel Hill; [6]Harvard Medical School at Beth Israel Deaconess Medical Center, Massachusetts General Hospital; [7]University of California, San Diego, University of California, La Jolla; [8]Emory University; [9]University of California, Los Angeles; [10]University of California, San Francisco*

**Background:** In the clinical high risk (CHR) for psychosis literature, typically, the focus is on determining the risk of conversion to psychosis. However, between 70% and 85% of youth who meet CHR criteria do not develop psychosis during the follow-up period of the study in which they participate. The aim of this study is to focus on CHR youth who did not transition to psychosis and to determine whether there are differences amongst them.
**Methods:** The North American Prodrome Longitudinal Study (NAPLS-2) is an 8-site prospective, longitudinal study including 764 help-seeking youth, age 12–35, meeting criteria for a psychosis risk syndrome based on the Structured Interview for Psychosis-risk Syndromes (SIPS), and 279 healthy controls (HC). For this analysis, only youth who did not make a transition to psychosis and completed 2 years of follow-up (n=278, 154 males, 124 females; mean age 18.8) were included. At the 24-month final assessment, the sample was divided into 3 groups: 1) those in remission, determined by scores ≤2 on all 5 attenuated psychotic symptoms on The Scale of Psychosis-risk Symptoms (SOPS); 2) symptomatic, determined by still having a rating of 3–5 on any one of the 5 attenuated psychotic symptoms on the SOPS; 3) prodromal progression, determined by continuing to meet the Criteria of Psychosis-risk Syndromes (COPS). The groups were compared at baseline and at 24-month follow-up on: age, gender, the presence of a current and lifetime psychiatric diagnosis, and social and role functioning. The use of antipsychotic medication was examined across all assessments (baseline, 6-, 12-, 18- & 24 months) using Generalized Linear Models to examine differences among the 3 groups.
**Results:** Among the participants, 110 (39.57%) were in-remission, 93 (33.45%) symptomatic, and 75 (26.98%) prodromal progression. At baseline there were no significant differences in age, gender, social and role functioning, or SCID diagnoses except on current PTSD (p=.001) with most cases in the prodromal progression group, and on current anxiety disorder (p=≤.0001) with most cases in the symptomatic group. The prodromal progression had significantly higher ratings on unusual thought content compared to the in-remission group and significantly higher ratings on suspiciousness than the symptomatic group. At 24-month follow-up there were significant differences in negative symptoms (p=≤.0001) between prodromal progression (M=9.19), symptomatic (M=8.84), and in remission (M=5.99) groups; and social functioning (p=≤.005; M=6.56, M=6.68, M=7.20 respectively). Although the in-remission group had the highest ratings on social functioning these were significantly lower in social (M=7.20) and role (M=6.68) functioning than HC (M=8.73, M=8.62 respectively). The groups did not differ on their use of antipsychotics over the course of their 2 years in the study.
**Discussion:** There were very few differences on baseline measures amongst the different two-year outcome groups. At 2 years, even though those in remission had improved social and role functioning relative to the other 2 groups, they still had lower social and role functioning than HC.

### F33. MATERNAL AND PATERNAL CANNABIS USE DURING PREGNANCY AND RISK OF PSYCHOTIC SYMPTOMS IN THE OFFSPRING

Koen Bolhuis*[,1], Steven A. Kushner[1], Manon H.J. Hillegers[2], Henning Tiemeier[1], Hanan El Marroun[1]
*[1]Erasmus Medical Center; [2]Erasmus Medical Center, University Medical Center Utrecht*

**Background:** Cannabis use has repeatedly been associated with psychotic symptoms, with persistent risks beyond the direct effects of exogenous cannabinoids. However, it remains unknown whether cannabis use during pregnancy is a causal risk factor for psychotic symptoms in the offspring, or whether this relationship is explained by shared etiological factors, such as genetic and environmental vulnerabilities. More innovative study designs are needed to address this question. Here, we examined the adverse effects of cannabis exposure during pregnancy on psychotic symptoms in pre-adolescent offspring. Such a method would help causal inference as comparisons can be made between the observed associations of maternal versus paternal cannabis use during pregnancy and the risk of psychotic symptoms in the offspring. If the association between cannabis use and psychotic symptoms is causal, early intra-uterine exposure to cannabis could potentially affect neurodevelopment and, hence, contribute to the pathogenesis of psychotic phenomena in children who have not yet used cannabis themselves.

**Methods:** This study used data from the Generation R Study, a prospective population-based birth cohort from Rotterdam, the Netherlands. Participants were included if data on maternal cannabis use during pregnancy of offspring psychotic-like symptoms at age ten years were available (N = 3692). To determine cannabis exposure, we used prospective maternal self-reports during pregnancy and cannabis metabolite levels from urine. Paternal cannabis use during pregnancy was obtained through maternal report. At age ten years, children were queried regarding psychotic symptoms. Ordinal logistic regression was conducted to investigate whether maternal and paternal cannabis use were associated with offspring psychotic symptoms. In a secondary analysis, a distinction was made between maternal cannabis use exclusively before versus continued maternal cannabis use during pregnancy. All models were adjusted for covariates that were previously associated with cannabis use in this cohort.

**Results:** Maternal cannabis use was associated with an increased risk for psychotic symptoms in their offspring (n = 183, ORadjusted=1.38 [95% CI 1.03–1.85]). Estimates were comparable for cannabis use exclusively before pregnancy versus continued cannabis during pregnancy (cannabis use before pregnancy: n = 98, ORadjusted=1.39 [95% CI 0.94–2.06]; continued cannabis use during pregnancy: n = 85, ORadjusted=1.37 [95% CI 0.90–2.08]). Paternal cannabis use was significantly associated with offspring psychotic symptoms (n = 297, ORadjusted=1.44 [95% CI 1.14–1.82]).

**Discussion:** Using data from a large population-based birth cohort, we demonstrated that maternal and paternal cannabis use were each associated with offspring psychotic symptoms at age ten years, well before the risk period of adolescent cannabis use initiation. Notably, estimates were similar for maternal cannabis use exclusively before pregnancy versus continued cannabis use during pregnancy. Moreover, estimates were comparable for maternal versus paternal cannabis use during pregnancy. This suggests that common etiologies, rather than solely causal intra-uterine mechanisms, underlie the association between parental cannabis use and offspring psychotic symptoms, shedding potential new light on the debated causal path from cannabis use to psychosis. Our findings indicate that diagnostic screening and preventative measures need to be adapted for young people at risk for severe mental illness, and that these programs need to offer a family-focused approach.

### F34. AUDITORY SENSORY GATING IN YOUNG ADOLESCENTS WITH EARLY-ONSET PSYCHOSIS: A COMPARISON WITH ADHD

Cecilie Lemvigh*[,1], Jens Richardt Møllegaard Jepsen[1], Birgitte Fagerlund[1], Anne Katrine Pagsberg[2], Birte Glenthoj[1], Jacob Rydkjaer[1], Bob Oranje[3]

*[1]Center for Neuropsychiatric Schizophrenia Research, Center for Clinical Intervention and Neuropsychiatric Schizophrenia Research, University of Copenhagen; [2]Child and Adolescent Mental Health Center; [3]University Medical Center Utrecht, Holland/Center for Neuropsychiatric Schizophrenia Research, and Center for Clinical Intervention and Neuropsychiatric Schizophrenia Research, University of Copenhagen*

**Background:** Numerous studies have demonstrated impaired sensory gating in schizophrenia and this phenomenon has been proposed as a candidate biomarker for the disorder. Sensory gating is typically assessed during an auditory paired-click test commonly referred to as a P50 suppression paradigm. When two identical stimuli are presented, healthy subjects show a decrease in their neural response to the second stimulus, reflected in a decreased P50 amplitude, whereas schizophrenia patients on average show a much smaller decrease. So far, sensory gating has primarily been investigated in adult patients with schizophrenia, but gating disturbances have also been demonstrated in other illnesses, e.g. in schizotypal personality disorder, albeit less marked. Although the typical age of onset for schizophrenia is late adolescence to early adulthood, a sizable group of patients presents with psychotic symptoms during childhood or early adolescence. Manifestation of psychotic symptoms before the age of 18 is commonly referred to as early-onset psychosis (EOP). Various studies have reported a more severe course of illness and a poorer outcome in EOP compared to the adult-onset form of the disorder. In parallel, we expect more pronounced sensory gating deficits in EOP.

Impaired sensory gating may not be specific to psychosis, but rather a shared disturbance of neuropsychiatric disorders. Although symptoms of attention deficit hyperactivity disorder (ADHD) differ in many ways from those found in schizophrenia, there are common characteristics. Compared to schizophrenia, relatively few studies have investigated sensory gating in ADHD, and some report P50 gating deficits similar to those frequently found in patients with schizophrenia.

**Methods:** We investigated P50 suppression in a large cohort of adolescents (12–17 years old) consisting of patients with either EOP (N=56) or ADHD (N=28) as well as age and gender matched healthy controls (N=72). In our paradigm two identical sounds (clicks) were presented separated by a 500ms interval. The amount of suppression was expressed as the ratio between the P50 amplitude of a subject's response to the first click and his/her amplitude in response to the second click.

**Results:** The EOP patients scored significantly higher on PANSS (positive, negative, general, and total PANSS scores) compared to both ADHD patients and healthy controls. However, there were neither significant group differences in raw P50 amplitude, nor in the gating ratios between young adolescents with EOP, ADHD and healthy controls.

**Discussion:** This is the first study to investigate sensory gating in young adolescents with EOP. We found no P50 suppression deficits in these patients which, given the relatively large sample size in our study, cannot merely be ascribed to power issues. The results are in contrast with the majority of studies investigating sensory gating in schizophrenia and ADHD. However, the results are in agreement with earlier studies from our lab showing evidence of inconsistent P50 suppression deficits in two separate cohorts of adult, antipsychotic naïve, first-episode patients with schizophrenia. Based on our findings, P50 sensory gating cannot differentiate between young adolescents with EOP or ADHD, and deficient P50 suppression does not seem to be a valid biomarker for EOP.

### F35. PRESCRIPTION PATTERN OF ANTIPSYCHOTICS FOR CHILDREN AND ADOLESCENTS WITH SCHIZOPHRENIA IN KOREA BASED ON NATIONWIDE DATA

Kee Jeong Park*[,1], Jung Sun Lee[1], Hyo-Won Kim[1]

*[1]Asan Medical Center, University of Ulsan College of Medicine*

**Background:** This study aimed to analyze the extent and pattern of antipsychotic prescription for Korean children and adolescents with schizophrenia using population-based data.

**Methods:** Our data was retrieved from the Korean National Health Insurance Review & Assessment Service-National Sample for 2013, which was a stratified sampling from the entire population under the Korean national health insurance program. Among 0.2 million children and adolescents aged 6–18 years from data, subjects who had received any antipsychotic medication in the year were investigated for the prescribed medication and concomitant psychotropic medication.
**Results:** A total of 91 children and adolescents (mean age 16.2 ± 2.2 years, 53 boys) received antipsychotic medication. Among the prescriptions, risperidone (n = 59, 35.3%) and aripiprazole (n = 34, 20.4%) were the two most frequently prescribed antipsychotics. Of 91 patients, 80 (87.9%) have prescribed with antipsychotic monopharmacy (mean 134.9 ± 11.1 day), 33 (36.3%) with bipharmacy (mean 136.9 ± 20.2 days), and 12 (13.2%) with polypharmacy (more than three antipsychotics) (mean 32.5 ± 7.3 days) in the year. Mood stabilizers (n=48, 52.7%), and antidepressants (n=35, 38.5%) were co-medicated in the year.
**Discussion:** Our study shows the prescription pattern of antipsychotics for children and adolescents with schizophrenia in Korea.

### F36. SELF-ESTEEM AND SYMPTOMS IN INDIVIDUALS AT CLINICAL HIGH-RISK FOR PSYCHOSIS

Caridad Benavides*[,1], Gary Brucato[2], David Kimhy[1]
[1]*Icahn School of Medicine at Mount Sinai;* [2]*Columbia University*

**Background:** Individuals with psychotic symptoms often report low global self-esteem (GSE).
However, it remains unclear whether the low GSE is linked to the presence of psychotic symptoms or it is present prior to the onset of psychosis. Additionally, the specific subdomains of GSE in these populations are unknown.
**Methods:** To address this question, we conducted a cross-sectional study comparing global and SE elements among individuals at clinical high-risk for psychosis (CHR; n=36), individuals with schizophrenia (SCZ; n=43), and healthy controls (HC; n=40). We then examined among CHR individuals the association between GSE, subdomains, and symptoms.
**Results:** CHR individuals displayed significantly lower GSE compared to HC, at a level comparable to individuals with SCZ. The low GSE was driven primarily by self-perceptions of work and interpersonal relationships abilities, in the sub-domains of provider (r=.53, p<.001), physical appearance (r=.37, p=.026), sense of humor (r=.45, p=.006), intimate relations (r=.45, p=.006), and job competence (r=.39, p=.018). Lower GSE was associated with overall negative (p<0.01) and disorganized (p<0.05) symptoms severity, but not positive ones.
**Discussion:** This investigation demonstrates that low self-esteem, which is prevalent in SCZ, is also present to a comparable degree of severity in CHR individuals. Our findings help to better understand the CHR period and may suggest targets for early psychosocial treatments aimed at improving social and occupational functioning, as such domains appear to play an important role in determining GSE in this population. The examination of different dimensions of self-esteem in the assessment of CHR populations may contribute to a better understanding of the phenomenology of prodromal symptoms, and thus, may have implications for finding successful early therapeutic approaches, rehabilitation, and social integration for a condition that is accompanied by considerable suffering and risk of suicide.

### F37. CACNA1C HYPOFUNCTION IN KETAMINE-ACTIVATED BRAIN NETWORKS IMPAIRS MEMORY CONSOLIDATION AND FUNCTIONAL BRAIN NETWORK CONNECTIVITY

Rebecca Hughes[1], Cosima Willi[1], Jayde Whittingham-Dowd[1], Susan Broughton[1], Greg Bristow[1], Neil Dawson*[,1]
[1]*Lancaster University*

**Background:** The CACNA1C gene, encoding the pore forming subunit of the calcium channel Cav1.2, is an established risk factor for a variety of psychiatric disorders including schizophrenia. The mechanisms through which altered Cav1.2 function increases the risk of developing these disorders is not well understood. Administration of subanaesthetic doses of the NMDA receptor antagonist ketamine provides a translational model of schizophrenia, modifying neuronal activity in brain networks underlying the symptoms of the disorder.
**Methods:** Here we selectively reduce Cav1.2 expression in the brain networks activated by ketamine, using a novel transgenic mouse model (Cav-KHypo mice), and elucidate how this impacts on learning and memory, brain metabolism and functional brain network connectivity.
**Results:** We show that the induction of Cav1.2 hypofunction in schizophrenia-relevant (ketamine-activated) brain networks impairs memory consolidation. In addition, Cav-KHypo mice show schizophrenia-relevant alterations in cerebral metabolism including prefrontal cortex, thalamic and hippocampal hypometabolism. The induction of Cav1.2 hypofunction in ketamine activated brain networks impairs memory consolidation by altering functional connectivity between neural systems underlying this process, including compromised prefrontal-hippocampal and compromised thalamic connectivity.
**Discussion:** The data suggest that reducing CACNA1C expression in brain areas linked to schizophrenia is sufficient to induce disease related neural dysfunction. Furthermore, the data suggest that the regulation of neural system functional connectivity and memory consolidation may be primary mechanisms through which CACNA1C risk genes impact on cognition and increases the risk of developing psychiatric disorders.

### F38. WINTER-LIKE PHOTOPERIOD GESTATION DELETERIOUSLY AFFECT EXPERIENTIAL- BUT NOT EXPRESSIVE-RELATED BEHAVIORS, WHILE ADULT PHENCYCLIDINE TREATMENT INCREASES EXPERIENTIAL BEHAVIORS: RELEVANCE TO SCHIZOPHRENIA

Molly Kwiatkowski[1], Zackary Cope[1], Chuck van de Chapelle[1], Davide Dulcis[1], Susan Powell[1], Jared Young*[,1]
[1]*University of California, San Diego*

**Background:** Patients with schizophrenia exhibit negative symptoms that predict functional outcome. Understanding the mechanisms underlying these symptoms are important for developing novel therapeutic treatments. These negative symptoms can be grouped into experiential- (motivational) or expressive- (social) based behaviors testable in rodents. Increased incidences of schizophrenia have been reported with winter gestation, while antagonizing NMDA receptor function in adulthood has been used to model other symptoms of schizophrenia. These two approaches were used to determine mechanisms that may contribute to negative symptoms in schizophrenia and test potential therapeutics.
**Methods:** Normal dam mice were housed in custom photoperiod chambers for one week under normal photoperiod conditions (12 hour light:12 hour dark) to allow for initial acclimation. Half of the photoperiod chambers were then shifted to a short active (SA) condition (19 hours light: 5 hours dark) with mating triads formed. After a two-week pairing, pregnant dams were single-housed. The resulting pups were reared under these photoperiod conditions until P28, at which time they were weaned by sex into tetrads and moved into a standard vivarium room under normal active (NA) photoperiod. Mice were trained to perform nose-poke responses in a five-choice operant chamber. Adult Long Evans rats were also trained in separate 5-choice chambers and repeatedly treated with phencyclidine (PCP). Motivational behavior was tested on a progressive ratio breakpoint (PRB). Social behavior of mice were also assessed using a 3-chocie social recognition paradigm.
**Results:** In PRB, breakpoint was decreased in WT mice reared in SA vs. NA photoperiod (F(1,66)=4.4, p<0.05). In contrast however, no deficits in social interaction was observed (F<1, ns). Unlike the SA gestation-induced reduction in breakpoint, subchronic PCP increased breakpoint 1 [t(21)=5.3, p<0.0001], 7 [t(21)=2.2, p<0.0001] and 14 days after treatment to rats.

**Discussion:** Winter-like photoperiod births in mice induced psychiatry-relevant amotivation as measured by breakpoint, though did not affect social interaction or recognition. In contrast, subchronic PCP treatment increased breakpoint in rats tested 1 and 7 days after treatment. Hence, neurodevelopmental mechanisms underlying the winter-like gestation likely contribute to experiential- but not expressive-related behaviors, while altered NMDA receptor function are unlikely to contribute to such experiential-related abnormalities. Ongoing work will characterize epigenetic, synaptic, and/or system-level adaptations underlying developmental differences between NA and SA photoperiod born mice as well as defining critical periods throughout gestation and rearing that are driving these effects.

### F39. MATERNAL IMMUNE ACTIVATION MODELS: MIND YOUR CAGING SYSTEMS!

Flavia Müller*[,1], Ulrike Weber-Stadlbauer[1], Urs Meyer[1]
*[1]Institute of Pharmacology and Toxicology, University of Zurich*

**Background:** Rodent models of maternal immune activation (MIA) are increasingly used as experimental tools to study neuronal and behavioral dysfunctions in relation to infection-mediated neurodevelopmental disorders such as schizophrenia and autism. One of the most widely used MIA models is based on gestational administration of poly(I:C) (= polyriboinosinic-polyribocytdilic acid), a synthetic analog of double-stranded RNA that induces a cytokine-associated viral-like acute phase response. The effects of poly(I:C)-induced MIA on phenotypic changes in the offspring are known to be influenced by various factors, including the precise prenatal timing, genetic background, and immune stimulus intensity. Thus far, however, it has been ignored whether differences in laboratory housing systems can similarly affect the outcomes of MIA models. Here, we examined this possibility by comparing poly(I:C)-based MIA in two housing systems that are commonly used in preclinical rodent research, namely the individually ventilated cage (IVC) and open cage (OC) systems.
**Methods:** Pregnant C57BL6/N mice were kept in IVC or OC and treated with a low (1 mg/kg, i.v.) or high (5 mg/kg, i.v.) dose of poly(I:C), or with corresponding vehicle solution (pyrogen-free, sterile 0.9% NaCl; i.v.). MIA or control treatment was induced on gestation day (GD) 9 or 12, and the resulting offspring were raised and maintained in IVC or OC until adulthood for behavioral testing. An additional cohort of dams were used to assess the influence of the different caging systems on poly(I:C)-induced cytokine responses in the maternal plasma, placenta, and fetal brains 1 hr and 6 hrs post-treatment.
**Results:** Maternal administration of poly(I:C) on GD9 caused a dose-dependent increase in spontaneous abortion in IVC but not in OC system, whereas MIA in IVC systems during a later gestational time-point (GD12) did not do so. Maternal and fetal pro-inflammatory cytokine responses to poly(I:C) were markedly higher in animals kept in IVC as compared to OC systems. The efficacy of MIA to induce long-term behavioral deficits was influenced by the different housing conditions, the dosing of poly(I:C), and the precise prenatal timing.
**Discussion:** The present study identified the type of cage system as a novel factor that can confound the outcomes of MIA. Our findings thus urge the need to consider and report the kind of cages used in rodent MIA models. Providing this information seems pivotal to yield robust and reproducible results in these models.

### F40. NEUREXIN-1A (NRNX1A) HYPOFUNCTION INDUCES SCHIZOPHRENIA-RELEVANT DEFICITS IN CEREBRAL METABOLISM, COGNITIVE PROCESSING SPEED AND COGNITIVE FLEXIBILITY

Rebecca Hughes*[,1], Jayde Whittingham-Dowd[1], Greg Bristow[1], Stephen Clapcote[2], Susan Broughton[1], Neil Dawson[1]
*[1]Lancaster University; [2]University of Leeds*

**Background:** Heterozygous deletions in NEUREXIN-1 (NRXN1) substantially increase the risk of developing schizophrenia (SZ) (Rujescu et al., 2009. Hum Mol Genet 18(5):988–96). We currently have little understanding into the mechanisms by which NRXN1 impacts on the brain to increase the risk of developing the disorder, and to which symptom domains NRXN1 may contribute. Patients with schizophrenia show deficits in cognitive processing speed and cognitive flexibility (Sanchez et al., 2009. J Clin Psychiat. 70(6):888–896, Dieci et al., 1997. Schizophr Res. 25(1):33–42). In addition, patients show characteristic alterations in brain function including "hypofrontality"; prefrontal cortex hypometabolism (Hill et al., 2004. Acta Psychiatr Scand. 110(4):243–56). Here we characterise, in a transgenic mouse model, the impact of Nrxn1α hypofunction on cognitive flexibility, processing speed and cerebral metabolism to determine the potential translational relevance of the model to SZ.
**Methods:** Nrxn1α heterozygous (Hz) mice and their wild-type (Wt) littermates, of both sexes, were tested at 3, 6, 9 and 12 months old using a between-groups design. Associative learning and cognitive flexibility (reversal learning and set shifting) were assessed in a two choice odour based set shifting task (adapted from Young et al., 2010. Cog Affect Behav Neurosci. 10(2):243–251). Mice completed a series of testing phases that included two odour discrimination phases (OD1 and OD2), one reversal learning phase (OD2R) and an extra-dimensional shift (EDS), with animals shifting attentional set from odour to location. The criterion to successfully complete each phase was set at 6 consecutive correct choices. The number of trials to reach criterion, percentage correct and average latency for correct choices were recorded. After behavioural testing cerebral metabolism was determined in 49 brain regions using 14C-2-deoxyglucose functional brain imaging (Dawson et al., 2015. Transl Psychiatry. 5(5):e569). Data were analysed using ANOVA and t-test with Bonferroni correction. Significance was set at $p<0.05$.
**Results:** In the associative learning phases of the task (OD1 and OD2) Nrxn1α Hz mice took a similar number of trials as Wt controls to reach criteria. Nrxn1α Hz mice also completed a similar percentage of correct trials during these phases. This suggests that associative learning is not impaired in Nrxn1α Hz mice. However, Nrxn1α Hz mice showed a significant increase in the latency of correct choices in comparison to Wt animals during these phases, supporting significantly decreased processing speed in these animals. We also found that reversal learning (CDR2) was impaired in Nrxn1α Hz mice, evidenced by a significant increase in trials to criterion relative to Wt controls. In the brain imaging study, we found that Nrxn1α Hz mice show significant hypofrontality, with a reduced rate of metabolism in the anterior and medial prelimbic cortex (aPrL, mPrL). By contrast, Nrxn1α heterozygous mice show significant hypermetabolism in the dorsal raphe (DR), ventral tegmental area (VTA) and retrosplenial cortex (RSC).
**Discussion:** Nrxn1α heterozygosity induces SZ-like impairments in cognitive processing speed, cognitive flexibility (reversal learning) and cerebral metabolism, including the induction of hypofrontality. Nrxn1α heterozygosity also alters metabolism in neuromodulatory brain regions, including the serotonergic DR and dopaminergic VTA, which may also contribute to its impact on cognition. These data give new insight into the mechanisms by which NRXN1 heterozygosity increases the risk of developing SZ and suggest that Nrxn1α Hz mice provide a translational tool for drug discovery in relation to the cognitive deficits seen in the disorder.

### F41. SCHIZOPHRENIA-RELEVANT ALTERATIONS IN CEREBRAL METABOLISM, GLUTAMATE AND MONOAMINERGIC NEUROTRANSMITTER SYSTEM FUNCTION IN A MOUSE MODEL OF 16P11.2 DUPLICATION

Greg Bristow*[,1], David Thomson[2], Judith Pratt[3], Brian Morris[4], Neil Dawson[1]

[1]Lancaster University; [2]University of Strathclyde; [3]University of Strathclyde, Strathclyde Institute Pharmacy & Biomedical Science; [4]University of Glasgow

**Background:** Duplication at 16p11.2, affecting approximately 30 genes, has consistently been associated with increased risk of schizophrenia (Psychiatric Genomics Consortium, 2017. Nat Genet 49: 27). We currently have little understanding of how this CNV impacts on brain and neurotransmitter system function. Here we use a mouse model of 16p11.2 duplication (7DUP mice, Horev et al. 2011. PNAS. 108: 17076) to determine the impact of this CNV on cerebral metabolism. In addition, we characterize in vivo glutamate and monoamine neurotransmitter system function by challenging these animals acutely with ketamine and d-amphetamine, respectively.
**Methods:** 7DUP mice and littermate controls were treated with ketamine (25mg/kg), d-amphetamine (5mg/kg), or saline (2ml/kg). n=11 (6 male, 5 female) for each genotype per treatment group. Cerebral metabolism was determined by 14C-2-deoxyglucose functional brain imaging (Dawson et al., 2015.Transl Psychiatry. 5:e569). Data were analysed using repeated measures ANOVA with post-hoc Tukey's HSD.
**Results:** 7DUP mice show significant constitutive hypometabolism in the thalamic reticular nucleus, mesolimbic system, and in neuromodulatory brain regions. Hypometabolism was also seen in the striatum of female, but not in male, 7DUP mice. 7DUP mice were also found to show hypermetabolism in the hippocampus, amygdala, and cerebral cortex. The impact of ketamine on cerebral metabolism is attenuated in 7DUP mice with sex specific effects, being evident in the mesolimbic and neuromodulatory system of males, whereas the attenuation is present in the hippocampus and striatum in female mice. By contrast, 7DUP mice showed an exaggerated response to d-amphetamine. Again, these effects were influenced by sex, with the exaggerated response being significantly more widespread in males than in females.
**Discussion:** 7DUP mice show altered constitutive cerebral metabolism in brain regions implicated in schizophrenia, including hippocampal and temporal cortex hyperactivity. In addition, 7DUP mice demonstrate a reduced response to ketamine, supporting NMDA receptor hypofunction as a result of 16p11.2 duplication. This effect is consistent with the glutamate hypofunction hypothesis of schizophrenia. By contrast, 7DUP mice show an exaggerated response to d-amphetamine, supporting monoamine neurotransmitter system dysfunction as a consequence of 16p11.2 duplication. Intriguingly, each of these effects differs in male and female mice, suggesting that the phenotypic impact of 16p11.2 duplication is influenced by sex. These data provide new insight into the mechanisms through with 16p11.2 duplication increases the risk of developing schizophrenia.

### F42. CHONDROTIN-6 SULFATE CLUSTERS: ASSOCIATION OF SYNAPTIC DOMAINS AND REGULATION OF SYNAPTIC PLASTICITY DURING FEAR LEARNING

Gabriele Chelini*[,1], Cristina Berciu[1], Kanoelani Pilobello[1], Durning Peter[2], Jenkins Rachel[3], Moazzzam Kahn[1], Teniel Ramikie[1], Siva Subramanian[1], Kerry Ressler[1], Charalampos Pantazopoulos[1], Sabina Berretta[1]
[1]Harvard Medical School McLean Hospital; [2]Oberlin College; [3]Lesley University

**Background:** Emerging evidence from our group and others has brought the brain extracellular matrix (ECM) to the forefront of investigations on brain disorders. Our group has shown that organized perisynaptic ECM aggregates, i.e. perineuronal nets (PNNs) are decreased in several brain regions in people with schizophrenia (SZ) and bipolar disorder (BD). PNNs were detected by their expression of specific chondroitin sulfate proteoglycans (CSPGs), main components of the ECM, thought to play a key role in synaptic regulation during development and adulthood. Our studies have also shown that glial cells expressing CSPGs are altered in these disorders, suggesting a link between glial cell and PNN abnormalities. Finally, we have recently shown that novel CSPG structures, bearing a distinct CS-6 sulfation pattern and named CS-6 glial clusters, are decreased in the amygdala of people with SZ and BD. The morphology and function of CS-6 glial clusters is not currently known, but evidence from rodents and on the role of CSPGs in regulating synaptic functions strongly suggest that they may affect synaptic plasticity. We tested this hypothesis using a combination of human postmortem and rodent brain studies.
**Methods:** High Resolution electron microscopy was used to investigate the ultrastructural organization of CS-6 glia clusters. A transgenic mouse model expressing green fluorescent protein in a subset of excitatory pyramidal neurons was used to investigate dendritic spines association with CS-6 glia clusters. Mice were exposed to a single session of auditory fear conditioning for a total of 15 minutes. Animals were euthanized 4 hours after behavioral test. Multiplex immunocytochemistry was used to visualize CS-6 clusters.
**Results:** In human tissue, we show that CS-6 glia clusters are widespread in several brain regions, including the amygdala, entorhinal cortex, thalamus and hippocampus. Ultrastructural results show that CS-6 glia clusters are formed by CS-6 accumulations surrounding several dendrites. CS-6 expression was dected in astrocytes surrounding the dendrites, particularly in astrocytic endfeet enveloping dendritic spines, and within spines postsynaptic densities. Following auditory fear conditioning, marked changes of CS-6 glia clusters were observed in hippocampus regions dentate gyrus (g>1.5) and CA2 (g>1.5) and basolateral amygdala (g>1).
**Discussion:** These findings suggest that CS-6 glia clusters may represent segregated microdomains, dynamically regulated during learning and contributing to the modulation of synaptic regulation machinery. Specifically, we postulate that astrocytes synthesize CS-6 CSPG and secrete it through their endfeet around dendrites, modulating structural plasticity of dendritic spines. These results suggest a relationship between the abnormalities in CSPGs expression and alteration in dendritic spines, two pathological landmarks observed in postmortem brains of people with SZ and BD.

### F43. POTENTIATION OF INHIBITORY NEUROTRANSMISSION IN THE TREATMENT OF RECENT-ONSET SCHIZOPHRENIA BY MODIFICATION OF DEVELOPMENTAL PRUNING OF PREFRONTAL CIRCUITRY

Erin Burke[1], Joanne Wojcik[2], Larry J. Seidman[2], Alan Green[3], Tsung-Ung Wilson Woo*[,4]
[1]Arbour Hospital; [2]Beth Israel Deaconess Medical Center, Harvard Medical School; [3]Dartmouth College; [4]Harvard Medical School/McLean Hospital

**Background:** The overt symptoms and deficits of schizophrenia (SZ) typically emerge during late adolescence and early adulthood, followed by a period of post-onset functional deterioration. This peri-onset period temporally coincides with the final maturation of the prefrontal cortex (PFC), which is characterized by a process of extensive pruning of synaptic connectivities. Developmental maturation of inhibitory neurotransmission may play a key role in regulating the onset and duration of peri-adolescent synaptic pruning. We hypothesize that a deficit in the developmental increase in inhibitory neurotransmission may disturb the PFC synaptic pruning process and hence contribute to the onset and the functional deterioration that is characteristic of the early course of SZ. Enhancement of inhibitory neurotransmission may therefore restore the integrity of PFC neural circuitry, which may then lead to lasting improvements in cognitive deficits and clinical symptoms.

**Methods:** Here, we report preliminary data on the possible efficacy of tiagabine (Gabitril), which is a selective uptake inhibitor of the GABA (gamma-aminobutyric acid) transporter GAT-1, in the treatment of recent-onset schizophrenia. Subjects were randomized to receive either tiagabine or placebo added on to their antipsychotic regimen.
**Results:** Our data suggest that treatment with tiagabine during the early course of the illness can modulate PFC activation, as demonstrated by functional magnetic resonance imaging during working memory, and improve negative symptoms.
**Discussion:** Taken together, the proposed treatment strategy represents an effort to actively translate preclinical findings in SZ research into clinically testable hypotheses. This kind of translational approach, we believe, will ultimately lead to breakthrough in the treatment and possible prevention of SZ.

### F44. AN ADD-ON TRIAL WITH N-ACETYL-CYSTEINE (NAC) IN EARLY PSYCHOSIS PATIENTS: TOWARDS BIOMARKER GUIDED TREATMENT

Philippe Conus*[,1], Margot Fournier[2], Lijing Xin[3], Martine Cleusix[4], Philipp S. Baumann[4], Carina Ferrari[2], Ann Cousins[5], Luis Alameda[1], Mehdi Gholam-Razaee[1], Philippe Golay[2], Raoul Jenni[2], Tsung-Ung Wilson Woo[6], Matcheri Keshavan[7], Chin B. Eap[2], Joanne Wojcik[8], Michel Cuenod[2], Thierry Buclin[2], Rolf Gruetter[9], Larry Seidman[7], Kim Do[2]
*[1]Lausanne University; [2]Lausanne University Hospital; [3]Ecole Polytechnique Fédérale de Lausanne; [4]Centre Hospitalier Universitaire Vaudois, University of Lausanne; [5]Beth Israel Deaconess Medical Center, Harvard Medical School; [6]Harvard Medical School, McLean Hospital; [7]Harvard Medical School; [8]Beth Israel Deaconess Medical Center; [9]Ecole Polytechnique Fédérale de Lausanne, Lausanne University Hospital*

**Background:** Oxidative stress, coupled with dysregulation of inflammation, NMDAR and dopamine, is involved in schizophrenia (SZ) pathophysiology. Earlier add-on clinical trials showed in chronic SZ patients that NAC, a precursor of glutathione (GSH), an important cerebral antioxidant, improved negative symptoms, mismatch negativity and local synchronization. We hypothesized that NAC at an earlier stage of illness would have a greater impact.
**Methods:** Early psychosis patients (EP, less than 5 years of illness, N=63; NAC=32, placebo=31) were supplemented with NAC (2.7g/day, 6 months) in a double-blind randomized placebo-controlled trial. Outcome measures: PANSS and neurocognition (MATRICS Consensus Cognitive Battery; n=36); quantification of medial prefronfal cortex glutathione (GSHmPFC) by 1H-magnetic-resonance-spectroscopy, of white matter diffusion properties estimated by generalized fractional anisotropy (gFA) computed from diffusion spectrum imaging (DSI), of blood cells GSH (GSHBC) and GSH peroxidase activity (GPxBC) at start and end of trial
**Results:** While PANSS negative and positive were not affected by NAC, NAC improved Processing Speed (NAC > Placebo; $F(1, 30)=5.849$, $p=.022$), favoring 2 of 3 processing speed tasks (Trail Making A, $F(1, 30)=4.279$, $p=.048$ & Verbal Fluency, $F(1, 30)=5.749$, $p=.023$). GSHmPFC (+23%, p=0.005) and GSHBC (+19%, p=0.05) were increased following NAC treatment. In patients with high-baseline GPxBC (>22.3U/gHb), subgroup explorations revealed an improvement of PANSS positive compared to placebo (p=0.02). The change of PANSS positive correlated negatively with that of GPxBC activity, showing that the improvement paralleled the restoration of redox status. NAC group showed 11% increase in fornix white matter integrity as measured by gFA, correlating with an increase in GSHmPFC over the 6-months period.
**Discussion:** This is the first clinical trial assessing the impact of NAC treatment in a sample of EP and the potential predictive role of peripheral biomarkers of redox dysregulation. The hypothesis that NAC would be beneficial to negative symptoms in EP was not confirmed in this small sample, most likely in reason of their very low level at baseline. The NAC induced GSHmPFC increase demonstrates its target engagement. NAC improved Processing Speed showing a therapeutic enhancement of cognitive functions. Most importantly, NAC improved fornix integrity, in association with brain GSH elevation, demonstrating for the first time that a redox regulator can enhance structural connectivity. Peripheral redox status allows identifying a subgroup of patients with improved positive symptoms. Future biomarker guided antioxidant interventions in larger EP samples should replicate these findings.

### F45. THE EFFICACY AND SAFETY OF BLONASERIN AFTER SWITCHING FROM OTHER ATYPICAL ANTIPSYCHOTICS IN SCHIZOPHRENIC INPATIENTS: AN OPEN-LABEL, MULTI-CENTER TRIAL

Bo-Hyun Yoon*[,1], Won-Myong Bahk[2], Young Joon Kwon[3], Sang-Yeol Lee[4], Kwanghun Lee[5], Moon Doo Kim[6], Sung-Yong Park[7], Min-Kyu Song[8]
*[1]Naju National Hospital; [2]St. Mary's Hospital, The Catholic University of Korea, [3]College of Medicine, Soonchunhyang University; [4]Wonkwang University, School of Medicine; [5]College of Medicine, Dongguk University; [6]School of Medicine, Jeju National University [7]Keyo Hospital; [8]Yesan Mental Health Clinic*

**Background:** The aim of this study was to investigate the efficacy and safety of blonanserin treatment after switching from other atypical antipsychotics in schizophrenic inpatients who showed inadequate efficacy and poor tolerability.
**Methods:** A total of 63 schizophrenic inpatients (inadequate response group=45 and poor tolerability group=18) were included in this study. They were already treated with atypical antipsychotics except blonanserin and not favored due to inadequate responses or intolerable adverse effects. Blonanserin was administered during 12 weeks after switching from their previous antispsychotics. Treatment response was evaluated with Brief Psychiatric Rating Scale (BPRS) and CGI-S, and safety profile were measured with Abnormal Involuntary Movement Scale (AIMS), Simpson-Angus Extrapyramidal Side effects Scale (SAR)S and Barnes Akathisia Rating Scale (BARS). Drug Attitude Inventory (DAI-10) and Subjective Well-being Under Neuroleptic Treatment (SWN) were used for subjective estimates. Assessments were done at baseline, 1, 2, 4, 8 and 12 weeks after blonanserin treatment. Repeated measures of ANOVA were done to analyze the group (inadequate vs. intolerable group) and time effects.
**Results:** CGI and BPRS were showed significant treatment responses after switching to Blonaserin. Time effects were significant at 2, 4, 8, 12 weeks after switching and group by time effect were also significant at that time. Mean changes of AIMS, SARS and BARS scores were not significant throughout test trial. Although SWN was significantly improved after switching to Blonaserin, it was not found significant group by time effect.
**Discussion:** The results suggest that blonanserin may be effective and well tolerable in schizophrenic patients who showed inadequate treatment response or poor tolerability.

### F46. LUMATEPERONE (ITI-007): FAVORABLE SAFETY PROFILE IN AN OPEN LABEL SAFETY SWITCHING STUDY FROM STANDARD-OF-CARE ANTIPSYCHOTIC THERAPY IN PATIENTS WITH SCHIZOPHRENIA

Robert Davis[1], Alex Dmitrienko[2], Steven Glass[1], Susan Kozauer[1], Jelena Saillard[1], Michal Weingart[1], Andrew Satlin[1], Sharon Mates[1], Christoph Correll[3], Kimberly Vanover*[,1]

[1]Intra-Celluar Therapies, Inc.; [2]Mediana Research; [3]The Zucker Hillside Hospital, Hofstra North Shore LIJ School of Medicine

**Background:** Lumateperone (ITI-007) is a first-in-class investigational agent in development for the treatment of schizophrenia. Acting synergistically through serotonergic, dopaminergic and glutamatergic systems, lumateperone represents a new approach to the treatment of schizophrenia and other neuropsychiatric disorders. Lumateperone is a potent antagonist at 5-HT2A receptors and exhibits serotonin reuptake inhibition. Lumateperone also binds to dopamine D1 and D2 receptors acting as a mesolimbic/mesocortical dopamine phosphoprotein modulator (DPPM) with pre-synaptic partial agonism and post-synaptic antagonism at D2 receptors and as an indirect glutamatergic (GluN2B) phosphoprotein modulator with D1-dependent enhancement of both NMDA and AMPA currents via the mTOR protein pathway. Lumateperone demonstrated antipsychotic efficacy in two well-controlled clinical trials and was found to be well tolerated with a safety profile similar to placebo in all trials conducted to date.
**Methods:** In an open-label safety study, 302 patients with schizophrenia were switched from standard-of-care (SOC) antipsychotic therapy to 6 weeks treatment with lumateperone (ITI-007 60 mg, equivalent to 42 mg active base) QPM with no dose titration, then switched back to SOC for 2 weeks. The primary objective was to determine the safety of lumateperone, assessed by adverse events, body weight, 12-lead electrocardiograms, vital signs, clinical laboratory tests, motor assessments, and the Columbia-Suicide Severity Rating Scale. The secondary objectives were to determine the effectiveness of lumateperone to improve psychopathology as measured by the PANSS, social functioning as measured by the PANSS Pro-Social Factor and the Personal and Social Performance Scale (PSP), and depression as measured by the Calgary Depression Scale for Schizophrenia.
**Results:** Lumateperone was generally well-tolerated with a favorable safety profile. There was no drug related serious adverse event. In comparison to treatment with SOC antipsychotics at baseline, mean body weight decreased with lumateperone treatment. Lumateperone also demonstrated a favorable cardiometabolic and endocrine safety profile. Mean levels of cholesterol, triglycerides and prolactin improved with lumateperone treatment and worsened again when patients returned to SOC. The cardiovascular safety of lumateperone was favorable including no QTc interval prolongation. While efficacy data in an open-label study should be interpreted cautiously due to the absence of a parallel control group, improvements were observed in change from baseline of the PANSS total scores. Improvements were also seen in the Positive symptom subscale score, General Psychopathology subscale score, Marder Negative Factor score, and Prosocial Factor score as well as in the PSP scale. Greater improvements were observed in subgroups of patients with elevated symptomatology such as those with comorbid symptoms of depression and those with prominent negative symptoms.
**Discussion:** Lumateperone represents a novel approach to the treatment of schizophrenia with a favorable safety profile. The lack of metabolic, motor and cardiovascular safety issues presents a safety profile differentiated from standard-of-care antipsychotic therapy. Patients with stable symptoms on other antipsychotics may further improve when switched to lumateperone, with no dose titration needed. These data may warrant further investigation in placebo-controlled trials in patients with prominent negative symptoms and, separately, in patients with comorbid depression to demonstrate efficacy in these populations.

### F47. COGNITIVE REMEDIATION AND PHYSICAL EXERCISE IN MULTI-EPISODE SCHIZOPHRENIA: STUDY PROTOCOL FOR A RANDOMISED CONTROLLED TRIAL

Nuria Pujol*[,1], Víctor Pérez-Solà[2], Romina Cortizo[2], Lourdes Ayllon[3], Teresa Salvador[3], Daniel Moreno[3], Ferran Català[3], Jacobo Chamorro[3], Silvia Oller[2], Javier Polo-Velasco[3], Adelina Abellanas[3], Cristobal Diez-Aja[3], Anna Mane[2]
[1]Universitat De Barcelona; [2]Institute of Neuropsychiatry and Addiction of the Barcelona MAR Health Park, Spain Hospital del Mar Medical Research Institute, Ciber Salud Mental Group (CIBERSAM); [3]Institute of Neuropsychiatry and Addiction of the Barcelona MAR Health Park

**Background:** Cognitive remediation (CR) and physical exercise have separately shown promising results in schizophrenia cognitive improvement, despite this, the impact on daily functionality is still limited. Physical exercise increases Brain Derived Neurotrophic Factor (BDNF) levels, promoting neuronal and cognitive plasticity, which can maximize the impact of CR. We are conducting a randomised controlled trial to determine the efficacy of an intensive program that combines CR and physical exercise on cognition and related outcomes for patients with schizophrenia. In addition, we investigate functional and structural brain effects of this intervention and its association to BDNF.
**Methods:** This study protocol describes a randomized controlled trial in which 74 patients are randomly assigned to either CR and physical exercise or CR and health promotion. The interventions are 12-week long and consist of three weekly sessions (90 min of CR and 40 min of either aerobic exercise or health promotion). To be included in the study, patients must be diagnosed with schizophrenia or schizoaffective disorder, aged 28–60 years, and do low physical activity, as measured by International Physical Activity Questionnaire, IPAQ. Exclusion Criteria for participation in the study are the presence of neurological or substance use disorders, IQ < 70 and somatic illnesses that contraindicate physical exercise. Healthy control participants (n=18) are screened for the presence of lifetime Axis I psychotic disorders and for the presence of a first-degree relative with schizophrenia. Primary outcome measures are cognitive performance, functional outcome, negative symptoms, BDNF levels and neuroimaging measures. Secondary outcome measures are quality of life and metabolic parameters. All measures are blindly assessed at baseline, at 3 months follow up and at 15 months follow up.
This trial was approved by the Comité Ètic d'Investigació Clínica de l'Hospital del Mar (CEIC) 2015/6209/I
**Results:** This poster is a study protocol. We will correct data from now on.
**Discussion:** The results of this trial will provide valuable information about whether cognitive remediation efficacy for patients with schizophrenia can be enhanced by aerobic exercise-induced BDNF upregulation.
TRIAL REGISTRATION:
The trial is registered at www.clinicaltrials.gov (NCT02864576)

### F48. RANDOMISED CONTROLLED TRIAL OF SOCIAL COGNITION INTERACTION TRAINING

Frances Dark*[,1], James Scott[2], Andrea Baker[3], Stephen Parker[4], Anne Gordon[5], Ellie Newman[1], Victoria Gore-Jones[1], Sukanta Saha[6], Carmen Lim[6], David Penn[7]
[1]Metro South Health Services; [2]The University of Queensland, Queensland Centre for Mental Health Research, Metro North Mental Health Service; [3]Queensland Centre for Mental Health Research; [4]Bayside Community Care Unit Redland Bay; [5]Metro North Mental Health Service; [6]Queensland Centre for Mental Health Research, Queensland Brain Institute; [7]University of North Carolina, Australian Catholic University

**Background:** Enthusiasm for the importance of social cognition in schizophrenia has grown as research has revealed that it is more strongly related to functional outcomes than neurocognition. A promising therapy developed by Roberts and Penn is Social Cognitive Intervention Training (SCIT). This therapy is comprised of three phases (i.e., Introduction & Emotions, Figuring out Situations, Checking it out) administered in a group format.
**Objective:** To evaluate the efficacy of Social Cognition and Interaction Training (SCIT) in improving social cognitive and social functioning deficits of patients with schizophrenia spectrum disorder compared with standard of care, Befriending Therapy (BT).
**Methods:** A 10-week, single-blind, randomized controlled trial (RCT) of SCIT and BT was carried out in 120 patients with schizophrenia spectrum disorder. Primary outcome measure is the total score on the Bell Lysaker Emotion Recognition Task (BLERT) at 12 weeks. Mixed Model for Repeated Measures was used to analyse change in BLERT score from baseline to 3-month follow-up between SCIT and BT groups. Secondary measures of the study are improvements on the Social Functioning Scale, [1] [1] [1] Hinting Task, Social Skills Performance Assessment, Internal, Personal and Situational Attributions Questionnaire, and Meta Cognition Questionnaire.
**Results:** Among 120 patients, the mean age (SD) was 36.8 years (10.4) and 71.7% were males. Of these, 59 were randomized to the BT group and 61 to the SCIT group. The mean age of participants was 36.8 years. 85.8% were receiving government benefit and 50% lived in supported housing. 71.7% were males. Pre/Post data will be presented on the 91 participants who completed the study. Results examining the primary outcome measure found there was insufficient evidence to conclude that the SCIT group was significantly different compared to BT group in terms of emotion recognition (BLERT scores) (SCIT vs BT change: 0.437, 95% CI: -0.14 to 1.01; P = 0.136). There was an overall effect of time where both treatments showed a steady improvement over time from baseline to endpoint and the effect was maintained at the three-month follow-up. There was no significant time x treatment group interaction which indicated that there was no difference in patterns of change in the treatment group over time. Data on secondary outcomes is currently being analysed.
**Discussion:** In this medium sized RCT of social cognition interaction therapy that used an active control, (BT) and standardised measure of emotional recognition, (BLERT) we found no significant difference between the interventions in our primary outcome measure of emotional recognition. Improvement in emotional perception has been found in the majority of studies of social cognitive interventions for schizophrenia. More specifically our results differ to those of Hasson-Ohayon who found significant improvement in emotion recognition in a RCT of SCIT with social mentoring compared with social mentoring alone in people diagnosed with schizophrenia, schizoaffective disorder, depression or bipolar disorder (Hasson-Ohayon 2014). This study is the largest RCT of SCIT to find a negative result in regards to emotion perception.

### F49. EFFECTIVENESS OF THE META-COGNITIVE TRAINING IN PEOPLE WITH FIRST-EPISODE PSYCHOSIS: DOES GENDER MATTER?

Susana Ochoa*[,1], Miriam Salas[1], Raquel Lopez-Carrilero[1], Esther Pousa[2], Ana Barajas[3], Eva Grasa[4], Maria Luissa Barrigón[5], Esther Lorente[6], Jordi Cid[7], Fermin González[8], Isabel Ruiz[9], Irene Birulés[1]
*[1]Parc Sanitari Sant Joan de Deu; [2]Parc de Salut Mar; [3]Les Corts; [4]Hospital Sant Pau; [5]Servicio de Psiquiatria y Psicologia Clinica; [6]Hospital Clínico de Valencia; [7]Institut de Psiquiatria Girona; [8]University of Jaen; [9]University of Málaga*

**Background:** The Meta-Cognitive Training (MCT) is a psychological intervention that combines psychoeducational components and cognitive behavioural therapies with a meta-cognitive approach. The MCT (Moritz and Woodward, 2007) focuses on different cognitive biases, theory of mind and attribution bias, as well as on the predictive value of depressed mood and low self-esteem on paranoid ideation. The MCT has result effective for people with schizophrenia in order to improve symptoms, cognitive insight, jumping to conclusions, memory and quality of life. However, less information is regarding the effectiveness of MCT in first episode psychosis. On the other hand, gender has an important role in psychosis, nevertheless the effect of gender in the effectiveness of MCT has not been proved.
The aim of the study is to assess the effectiveness of MCT regarding symptoms and cognitive insight in people with first episode psychosis, considering the effect of gender.
**Methods:** A multicenter, randomized, controlled clinical trial was performed. A total of 122 patients were randomized to an MCT or a psychoeducational intervention. The sample was composed of people with a recent onset of psychosis, recruited from 9 public centers in Spain. The treatment consisted of 8 weekly sessions for both groups. Patients were assessed at three time-points: baseline, post-treatment, and at six months of follow-up. The evaluator was blinded to the condition of the patient. Symptoms were assessed with the PANSS and cognitive insight with the BCIS. A regression model for repeated measures was performed with the SPSS by gender.
**Results:** The sample was composed by 85 men and 37 women, although 53 men and 21 women completed the treatment and the follow-up.
Both psychoeducational and MCT group improved in positive symptoms at post-treatment and follow-up ($p<0.05$-0.001) with higher effect sizes in the MCT group (0.53 versus 0.38). Regarding negative symptoms the MCT group improved in the follow-up ($p<0.001$) and general symptoms MCT improved in the post-treatment and follow-up ($p<0.001$). Cognitive insight was higher in people who attended the MCT, in self-certainty in the post-treatment ($p<0.05$), self-reflectiveness in the follow-up ($p<0.05$) and the composite index in both assessments ($p<0.05$).
Considering the results by gender, men of both groups improve more in positive, negative and disorganized symptoms of the PANSS ($p<0.001$-0.046) while women improve in positive symptoms. A tendency of interaction between group and affective symptoms was found only in women ($p=0.062$), improving more women of the MCT group. Regarding cognitive insight, women of the MCT group improve more in self certainty and total BCIS compared with the psychoeducational group ($p<0.001$–0.022).
**Discussion:** MCT could be an effective psychological intervention for people with a recent-onset of psychosis for the improvement of cognitive insight and psychotic symptoms. It seems that women could benefit more from the MCT intervention than men in reduction of affective symptoms and in the improvement of cognitive insight.

### F50. METACOGNITIVE REFLECTION AND INSIGHT THERAPY: A MULTICENTER RANDOMIZED CONTROLLED TRIAL

Steven de Jong*[,1], Rozanne van Donkersgoed[2], Marieke Timmerman[2], Marije aan het Rot[2], Lex Wunderink[3], Johan Arends[4], Mark van der Gaag[5], Andre Aleman[2], Paul Lysaker[6], Marieke Pijnenborg[2]
*[1]University of Amsterdam; [2]Rijksuniversiteit Groningen; [3]GGZ Friesland; [4]GGZ Drenthe; [5]VU University; [6]Roudeboush VA Medical Center*

**Background:** Difficulties in metacognition, or the ability to think about thinking and feeling, form an impediment to daily life functioning for persons with a psychotic disorder. In the past years, our research team has undertaken a multicenter, randomized controlled trial to investigate the efficacy of a new intervention designed to assist persons with a psychotic disorder to improve their metacognitive functioning (Metacognitive Reflection and Insight Therapy; MERIT).
**Methods:** After training thirteen therapists from seven mental healthcare institutes in the Netherlands, participants (n=70) with a DSM-IV-TR diagnosis of schizophrenia or schizoaffective disorder were included and randomized into either the MERIT condition or a treatment-as-usual condition. Persons randomized into the MERIT condition received 40 sessions of metacognitive psychotherapy, while persons in the control condition received care as usual. Measures of primary outcome (metacognition), secondary outcomes (empathy, depression, insight, stigma, social functioning, symptoms and quality of life) and control variables (neurocognition, premorbid IQ) were collected at baseline (pre), directly after therapy end (post) and at 6-month follow-up. After the follow-up measurement, research assistants were unblinded in order to conduct an interview with the participants regarding their experience of the therapy.
**Results:** Multilevel intention-to-treat and sensitivity analyses demonstrated that in both groups metacognition had improved, with no significant differences between the groups ($\chi2$ (1)=0.435, p=.51). At 6-month follow-up, however, participants in the MERIT condition demonstrated they had continued to improve on metacognition, while scores from the control condition dipped back down ($\chi2$ (1)=3.763, p=.05). Gains mainly seemed to be on metacognitive Self-Reflectivity ($\chi2$ (1)=10.295, p=.001). No effects were found on secondary measures in either condition.
**Discussion:** During this presentation, we will discuss our findings and the therapy protocol, including a discussion of the clinical relevance of the current intervention, analysis of post-therapy interviews surrounding the participant's experiences of the therapy, as well as practical limitations that were encountered during this five-year trial.
**Note:** S. de Jong (speaker) and R.J.M van Donkersgoed are early career scientists, expected to defend their dissertations in 2018.

### F51. INTEGRATED COGNITIVE REMEDIATION THERAPY PREVENTS RELAPSES IN SCHIZOPHRENIA OUTPATIENTS DURING 8-YEARS FOLLOW-UP

Daniel Mueller*[,1], Conny Steichen[1], Annina R. Reymond[1], Volker Roder[1]
[1]*University Hospital of Psychiatry and Psychotherapy, Bern*

**Background:** Relapse prevention is a major aim of any treatment for schizophrenia patients. In general, recent meta-analyses showed that one third of schizophrenia patients relapse in the first year after treatment, which corresponds with rehospitalization. Since years, study data support evidence for successful relapse prevention of psycho-educative and family therapy approaches in combination with pharmacological treatment. So far little is known about the impact of Cognitive Remediation Therapy (CRT) on relapse prevention.
**Methods:** The purpose of this RCT was to investigate whether additional CRT could prevent relapses compared to treatment as usual (TAU) defined as pharmacological and other psychosocial treatments. The CRT approach of choice was the Integrated Neurocognitive Therapy (INT) developed in our lab. INT is a group approach consisting of 4 modules including interventions on all the neuro- and social cognitive domains, defined by the MATRICS initiative, as well as educational, emotion regulation and stress reduction tasks. In this international multicenter study, a total of 156 stabilized schizophrenia outpatients, diagnosed with DSM-IV, participated. From this sample, 71 participants of two out of eight centers could be observed during a follow-up of 1, 5 and 8 years, regarding number of relapses and days of rehospitalization. Relapses were defined as increased symptoms followed by rehospitalization.
**Results:** One year after therapy, no marked differences between INT and TAU groups in relapse rates were evident. But during 5- and 8-year follow-up, 78% and 83% of TAU patients relapsed compared to 48% and 52% of INT patients suggesting a significant benefit of INT. TAU patients suffered from more than 2 relapses after 5 years and 2.5 relapses after 8 years. In comparison, INT patients showed 0.9 relapses after 5 and 1.4 relapses after 8 years. After the 5 years follow-up there was a highly significant difference between INT and TAU, and after the 8-years a statistical tendency favoring INT could be found. Regarding the days of hospitalization, TAU patients presented a mean value of 8 days during 1 year after treatment, 90 days after 5 years and 105 days after 8 years compared to INT patients with 1.2 days after 1 year, 19 days after 5 years and 35 days after 8 years. The comparison after 1 year was close to significant, the other ones were clearly significant favoring again the INT intervention.
**Discussion:** These data on INT intervention support evidence for an impact of CRT on relapse prevention in a 1, 5 and 8 years follow-up. However, the identification of mechanisms of change within INT treatment needs further research.

### F52. EFFICACY AND SAFETY OF LURASIDONE IN ADOLESCENTS WITH SCHIZOPHRENIA: INTERIM ANALYSIS OF A 24-MONTH, OPEN-LABEL EXTENSION STUDY

Robert Goldman[1], Michael Tocco*[,1], Andrei Pikalov[1], Josephine Cucchiaro[2], Ling Deng[1], Antony Loebel[1]
[1]*Sunovion Pharmaceuticals, Inc.*

**Background:** Long-term efficacy and safety data from prospective studies in adolescents with schizophrenia are limited. Lurasidone is an atypical antipsychotic that has demonstrated efficacy in the treatment of schizophrenia in both adults and adolescents. The aim of the current open-label trial was to obtain long-term data on the safety and effectiveness of lurasidone in adolescents with schizophrenia.
**Methods:** Patients ages 13–17 with schizophrenia were randomized to 6 weeks of double-blind (DB) treatment with lurasidone 40 mg/d, 80 mg/d or placebo. Patients who completed this study were eligible to enroll in a 2-year, open-label (OL), flexible-dose (20–80 mg/d) extension study in which patients were continued on lurasidone, or switched from placebo to lurasidone. These data are the results of an interim analysis of the 2-year study. Effectiveness measures included the Positive and Negative Syndrome Scale (PANSS) total score (responder criteria, ≥20% reduction from double-blind baseline).
**Results:** A total of 271 patients completed 6 weeks of double-blind treatment and entered the 2-year extension study. At the time of the interim analysis, 132 patients had completed 52 weeks of treatment (24 patients were 2-year study completers; 96 patients were still ongoing; and 12 patients had discontinued after 52 weeks); 57 patients were still ongoing in the first 1-year of treatment; and 82 patients terminated prior to week 52 (28 patients due to withdrawal of consent; 23 due to adverse events; 9 due to lack of efficacy; and 22 for other reasons). Mean PANSS total score at double-blind baseline was 93.5. Overall mean change from double-blind to open-label baseline (after 6 weeks of treatment) was -17.5 (for patients assigned to lurasidone vs. placebo in the initial 6-week study, mean change was: -19.8 vs. -12.9). Overall mean change from double-blind baseline in the PANSS total score at weeks 28 (n=215), 52 (n=133), 76 (n=86), and 104 (n=24) was -29.2, -34.0, -35.0, and -34.1, respectively. Responder rates at week 52 and week 104 were 91.7% and 100%, respectively. During open-label treatment, the most common adverse events were headache (21.8%), nausea (11.8%), and anxiety (11.8%); 6.6% of patients reported an adverse event as severe. Median change in laboratory parameters from double-blind baseline to weeks 52 and 104, respectively, were: total cholesterol, -2.0 and -5.0 mg/dL; triglycerides, +3.5 and +3.0 mg/dL; hemoglobin A1c, 0.0 and 0.1%; prolactin in female, +0.5 and -0.5 ng/mL and males, +0.15 and +3.5 ng/mL; and mean change from DB baseline in weight at weeks 52 and 104 were 3.8 and 7.2 kg, vs. an expected weight gain of 3.3 and 5.1 kg, based on the gender-and-age specific CDC growth chart.
**Discussion:** In adolescents with schizophrenia, long-term treatment with lurasidone was associated with continued improvement in symptoms of schizophrenia. After one year of lurasidone treatment, minimal effects were observed on body weight, lipids, and glycemic indices.

### F53. DOSE REDUCTION OF HIGH-DOSE FIRST-GENERATION ANTIPSYCHOTICS OR SWITCH TO ZIPRASIDONE IN LONG-STAY PATIENTS WITH SCHIZOPHRENIA: A 1-YEAR DOUBLE-BLIND RANDOMIZED CLINICAL TRIAL

Jan Bogers*[,1], Raphael Schulte[2], Broekman Theo[3], Lieuwe de Haan[4]

[1]MHO Rivierduinen; [2]MHO North Holland North; [3]Bureau Beta; [4]Academic Medical Center, University of Amsterdam

**Background:** Long-stay patients with severe schizophrenia are frequently treated with high doses of first-generation antipsychotics (FGA). Dose reduction or switching to ziprasidone may reduce the severity of negative symptoms or side effects.

**Methods:** In a randomized double-blind trial, we compared the effect of FGA dose reduction (to equivalent of 5 mg/day haloperidol) (n=24) or switching to ziprasidone 160 mg/day (n=24). Negative symptoms after 1 year of treatment were primary outcome measure. Treatment failure was defined as a prolonged (>4 weeks) or repeated relapse.

**Results:** Negative symptoms did not change significantly during dose reduction nor was there a significant difference between treatments. Neurological side effects diminished in both conditions. Positive symptoms, excited symptoms, and emotional distress worsened over time with ziprasidone, resulting in a significant difference in favour of FGA dose reduction. More patients in the ziprasidone condition (46%) than in the FGA condition (21%) relapsed. Although some recovered within 4 weeks, treatment failed in 25% of the patients in the ziprasidone condition and in 17% of the patients in the FGA condition (non-significant differences). In about 80% of patients, doses could be reduced without a prolonged increase in symptom severity.

**Discussion:** In long-stay patients with severe schizophrenia, reducing high doses of FGA to a dose equivalent of 5 mg/day haloperidol or switching to ziprasidone did not improve negative symptoms. Reducing antipsychotic doses was feasible in most patients, although the risk of relapse is substantial. Neither FGA dose reduction nor ziprasidone seems an adequate alternative to clozapine for treatment-resistant schizophrenia.

### F54. PHARMACOLOGICAL ENHANCEMENT OF COGNITION AND SOCIAL COGNITION IN THE PSYCHOSIS SPECTRUM

M. Mercedes Perez-Rodriguez*[,1], Daniel Rosell[1], Scott Moeller[1], Prantik Kundu[1], Margaret McCLure[1], Erin Hazlett[1], Antonia New[1]
[1]Icahn School of Medicine at Mount Sinai

**Background:** Abnormalities in cognition and social cognition represent a core feature of the schizophrenia spectrum disorders. Schizotypal personality disorder (SPD) is a milder disorder within the schizophrenia spectrum, characterized by attenuated, schizophrenia-like traits without overt psychosis.

Study 1: Working memory impairments are a core cognitive deficit in schizophrenia and SPD. The dopamine D1 receptor is a promising target to enhance working memory. We aimed to test the effect of the D1 agonist dihydrexidine (DAR-0100A) to enhance working memory in patients with SPD.

Study 2: Oxytocin modulates social cognition. However, oxytocin's effect on social cognitive errors in the schizophrenia spectrum remains unexplored. We aimed to: 1) characterize social cognitive (mentalizing) errors in SPD patients and test their relationship with positive and negative symptoms of psychosis; 2) test the effect of intranasal oxytocin on mentalizing errors.

**Methods:** Study 1: We performed a randomized, double blind, placebo-controlled trial of DAR-0100A (15 mg/150 ml of normal saline i.v. over 30 min) in medication-free SPD patients (n=16). Study 2: Subjects: 15 SPD patients, 15 healthy controls [HC], and 15 psychiatric controls (PC). Intervention: intranasal oxytocin 24/40IU/placebo. Measures: Movie for the Assessment of Social Cognition (MASC), a naturalistic video task measuring mentalizing accuracy, "no mentalizing" errors, "hypomentalizing" errors and "hypermentalizing" errors. The "hyper-hypomentalizing ratio" can be computed to capture the predominant mentalizing tendency; PANSS; Schizotypal Personality Questionnaire, SPQ. Mentalizing measures were compared across groups (SPD, HC, PC), and treatments (oxytocin 24IU/40IU vs placebo) using ANOVA. Pearson correlations assessed the relationship between social cognition and symptoms.

**Results:** Study 1: Treatment with dihydrexidine (DAR-0100A) was associated with significantly improved working memory performance relative to placebo, with a very large effect size (Cohen's d=1.14). Study 2: SPD patients had lower mentalizing accuracy (F=10.11;df=1;p=0.003), made more "No mentalizing" or "hypomentalizing" errors (F=12.92;df=1;p=0.001), and had lower hyper-hypomentalizing ratios than HCs (F=2.84; df=1;p=0.099,trend level). In a subset of patients –including 8 SPD-, a single dose of intranasal oxytocin significantly increased the hyper-hypomentalizing ratio (F=6.84, df=1,p=0.019) and increased visual attention to social cues.

"No mentalizing" and "hypomentalizing" errors were significantly correlated with negative symptoms. "Hypermentalizing" errors were significantly correlated with positive symptoms and the "ideas of reference" and "suspiciousness" SPQ subscales.

**Discussion:** Study 1: These preliminary findings lend further clinical support to the potential of D1 receptor agonists to treat schizophrenia-spectrum working memory impairments.

Study 2: As hypothesized, SPD patients had impaired, less accurate social cognition, and made more "no mentalizing" and "hypomentalizing" errors, correlated with negative symptoms. Conversely, "hypermentalizing errors" were correlated with positive symptoms. Oxytocin increased the tendency to hypermentalize. This effect may normalize the abnormalities found at baseline in SPD patients. These results support the role of social cognitive impairments as an underlying factor of positive and negative symptoms of psychosis, with specific associations with paranoid and delusional traits. Our results also suggest that intranasal oxytocin modulates social cognitive errors in the psychosis spectrum.

### F55. EFFICACY OF COMPUTER ASSISTED COGNITIVE REMEDIATION IN MID-AGED AND OLDER INPATIENTS WITH CHRONIC SCHIZOPHRENIA IN KOREA

Kee-Hong Choi[1], Jinsook Kang[1], Sun-Min Kim[1], Seung-Hwan Lee[2], Seon-Cheol Park[3], Won-Hye Lee[4], Sun Choi[5], Kiho Park*[,1], Tae-Yeon Hwang[4]
[1]Korea University; [2]Inje University College of Medicine, Ilsan Paik Hospital; [3]Inje University College of Medicine and Haeundae Paik Hospital; [4]National Center for Mental Health; [5]Yong In Mental Hospital

**Background:** Accumulating evidence indicates that cognitive remediation(CR) is effective for improving various cognitive deficits in adult patients with schizophrenia. Although reports of brain plasticity in older adults and the service needs of chronic mid-aged and older patients with schizophrenia are increasing, very few randomized controlled trials of CR have been conducted in mid-aged and older inpatients with schizophrenia. We investigated the efficacy of individualized CR on the cognitive impairments of mid-aged and older inpatients with schizophrenia within the context of comprehensive psychiatric rehabilitation(PR) by comparing the results obtained with PR only and treatment as usual(TAU).

**Methods:** Fifty-seven mid-aged and older individuals with schizophrenia (age mean: 50.07 sd: 6.01) and mild to moderate cognitive deficits were enrolled. All participants stayed in long-stay closed ward hospital. Thirty-eight who were undergoing PR were randomly assigned to CR + PR (N = 19) or PR-only (N = 19) groups. For PR groups (CR+PR group and PR only group) received comprehensive inpatient PR, including optimal pharmacotherapy, vocational rehabilitation, social skills training, daily living skills training, illness management, independent living skills training, and patient empowerment program. For CR+PR group, CR treatment was provided adjunctive to their PR program described above. CR treatment consisted of 24 sessions that occurred twice a week for 1 h/session for over 3 months. The PSSCogRehab software program which was translated in Korea and Lumosity cognitive enhancement game were utilized for CR training. Participants in PR-only group were also received same psychiatric rehabilitation program, that specific training on neurocognitive functioning was not included. Nineteen participants who were undergoing TAU without CR or PR were evaluated pre- and post-treatment.

**Results:** No group differences were found in key demographical variables, premorbid IQ, psychiatric characteristics or baseline neurocognitive functioning at the pre-treatment. CR was easily provided and well received (drop-out rates = 5.3%) by mid-aged and older psychiatric inpatients. the CR + PR group showed greater post-treatment performance on both WCST total errors and WCST %CL compared with the PR-only group (WCST total errors: mean difference = 12.28, p = 0.026; WCST %CL: mean difference = 16.47, p = 0.017) and TAU group (WCST total errors: mean difference = 14.00, p = 0.015; WCST %CL: mean difference = 16.24, p = 0.023). However, no group differences were found on WCST total errors and WCST %CL between the PR-only and TAU groups. No group differences were found for processing speed, attention, verbal working memory or cognitive flexibility.

**Discussion:** The results of the current study partially supported our primary hypothesis. Specifically, compared with the PR-only and TAU groups, the CR + PR group showed greater improvement in executive functioning. Importantly, the reliable change index(RCI) indicated that more participants in the CR + PR group had clinically meaningful improvements in LM and executive functioning compared with participants in the PR-only and TAU groups. These results suggested that CR improved some cognitive deficits in mid-aged and older long-stay inpatients with schizophrenia and that it was effective as an adjunctive treatment to the usual PR services provided in inpatient settings.

### F56. IMPACT OF SIDE EFFECTS DUE TO SECOND-GENERATION ANTIPSYCHOTICS ON THE FUNCTIONING OF PATIENTS WITH SCHIZOPHRENIA: AN OBSERVATIONAL, PATIENT CENTERED, WEB SURVEY

Rajiv Tandon*[,1], Catherine Weiss[2], William Lenderking[3], Owen Cooper[3], Huda Shalhoub[3], Leah Kleinman[3], Jun Chen[3], Ann Hartry[4], Mallik Green[2], Stine R. Meehan[5], Laëtitia Bouérat Douvold[5]

*[1]University of Florida; [2]Otsuka Pharmaceutical Development & Commercialization, Inc.; [3]Evidera; [4]Lundbeck LLC; [5]H. Lundbeck A/S*

**Background:** In patients with schizophrenia, antipsychotic medications, including second-generation antipsychotics, may cause many side-effects (SE) often leading to treatment discontinuation, and possible relapse as a consequence. The impact of treatments on patient-centered outcomes such as health-related quality of life (HRQOL) is less well understood. Even less well understood is the impact of side effects on patient-centered outcomes such as daily functioning and HRQOL. Therefore, the study's primary goal was to gain a deeper understanding of the impacts of SEs of second generation antipsychotics on patients' day to day functioning.

**Methods:** A cross-sectional, web-based, patient-reported survey was fielded in the United States between July and November 2017. The final survey included patient socio-demographics, a quality of life measure (Quality of Life Enjoyment and Satisfaction Questionnaire Short Form, Q-LES-Q-SF), questions on treatment satisfaction, SEs experienced (Glasgow Antipsychotic Side-Effect Scale, GASS), and questions about the impact of SEs on functioning and emotions. Patients were recruited through patient advocacy and support groups, and medical research panels. Patient inclusion criteria: Self-reported schizophrenia diagnosis; 18 to 65 years old; stable for at least one month at time of screening; prescribed a second-generation antipsychotic medication for 1–12 months; the final sample consisted of those individuals who reported experiencing one or more side-effects based on the GASS.

**Results:** The total sample (n=180) had a mean age of 35 (range 18–61) years old, of which 58.3% were females. Approximately a quarter (27.8%) of the sample had a college degree or higher; 69.4% identified as White, followed by 16.7% Black/ African American, and 6.1% Native Hawaiian/ Pacific Islanders. Most prevalent SEs reported on the GASS were 'difficulty sleeping' (81.1%), 'feeling sleepy during the day' (77.2%), 'dry mouth' (70.6%), and 'feeling restless (60.6%). The SEs most commonly reported as distressing, for those patients experiencing that SE, were difficulty passing urine (23.3%), and feeling drugged/like a zombie (19.4%). The minimum impact from SEs on daily functioning was 53.2 on a 0–100 Visual Analogue Scale (higher number reflects more negative impact on daily functioning; 0=no impact and 100=very highly impacted). Across the SEs further probed about, the most severe impact was on one's 'ability to get or do a job'; specifically, for the SEs 'shaky hands or arms' the mean impact was 76.1, followed by 69.8 for restlessness. 'Problems enjoying sex' had the greatest effect on one's 'intimate relationships' (mean 74.8), and feeling drugged/ like a zombie had the greatest effect on one's 'ability to concentrate' (mean 70.2).

**Discussion:** The study indicates the importance of incorporating the patient with schizophrenia's perspective when assessing SE experiences and impact on functioning due to second generation antipsychotic agents. Findings suggest that both activating SEs (restlessness) and sedating SEs (feeling drugged and sleepiness) have pronounced undesirable impact on daily patient functioning.

### F57. CORRELATION FACTORS OF ABNORMAL MENSES IN SCHIZOPHRENIA TREATMENT WITH RISPERIDONE

Fang Dong*[,1], Qijing Bo[1], Chuanyue Wang[1]

*[1]Beijing Anding Hospital of Capital Medical University*

**Background:** A significant percentage of women taking antipsychotic medication may be suffering from abnormal menses during their treatment, which influences both fertility and adherence to medication. It is particularly common in patients prescripted with risperidone. This study aimed to identify the risk factors for abnormal menses in female individuals with schizophrenia during risperidone treatment, especially the relationship between abnormal menses and the dose or the length of the medicine.

**Methods:** This study used a retrospective data. 202 female patients diagnosed with schizophrenia using risperidone were screened. Doses and length of treatment with risperidone were various. 38 were excluded for their menstrual irregularities before treatment, in which 4 amenorrhea and 15 menopause. 164 female patients included, but 3 of them absent of data. 161 female patients included in analyses at last.

**Results:** Of the 161 patients, 119 were eumenorrhea up to our analyses, and other 42 abnormal menses, including 23 menstrual irregularities, 8 amenorrhea and 11 oligomenorrhea. There was no statistical difference in age (32.0 ± 8.6 vs. 31.4 ± 10.1) (years), education (12.2 ± 2.3 vs. 12.6 ± 2.2) (years), age at onset 26.7 ± 8.0 vs. 24.8 ± 8.4) (years), duration of illness (5.8 ± 5.2 vs. 7.0 ± 7.7) (years), PANSS total score (37.2 ± 8.8 vs. 38.1 ± 7.0) between normal group and abnormal group. There was also no statistical difference in risperidone dose at baseline (4.3 ± 0.7 vs. 4.3 ± 0.5) (mg/d), total treatment in this episode (5.3 ± 4.7 vs. 5.4 ± 5.4) (months), overall length of risperidone treatment in this episode (86.7 ± 62.0 vs. 98.6 ± 73.5) (days), length of risperidone treatment at optimal therapeutic dose (63.0 ± 64.5 vs. 51.3 ± 26.7) (days).

**Discussion:** Some research suggests antipsychotic-induced abnormal menses is related to medication-induced high prolactinemia level and low estradiol level pretreatment. But few study reports the relationship between abnormal menses and the dose or the length of the medicine. This study got negative results, which suggest the occurrence of abnormal menses widely depend on individual quality rather than the length and the dose of the antipsychotic. But there are some limits in the study. First, the dosage range among these subjects were relatively narrow. And then, the length of risperidone treatment is generally short. In the next step of research, we will improve these two points.

### F58. COMMUNITY-BASED MULTI-SITE RANDOMIZED CONTROLLED TRIAL OF BEHAVIORAL ACTIVATION FOR NEGATIVE SYMPTOMS OF INDIVIDUALS WITH CHRONIC SCHIZOPHRENIA

Eunbyeol Lee*[,1], Yun-Ji Cha[1], Ji-Hye Oh[2], Na-Ri Hwang[2], Kee-Hong Choi[1], Ho-Jun Seo[2]
*[1]Korea University; [2]The Catholic University of Korea*

**Background:** As existing treatments for negative symptoms in schizophrenia have limited empirical support, development of effective treatments for negative symptoms in schizophrenia is in urgent need. Behavioral activation (BA), which is an evidence-based treatment for depression, is a promising candidate treatment for negative symptoms as it proved its feasibility and preliminary efficacy in non-randomized controlled trial for community dwelling individuals with chronic schizophrenia (Choi et al., 2015; Mairs et al., 2011). The primary purpose of the current study was to investigate whether BA would improve negative symptoms as compared with treatment as usual (TAU) for community dwelling individuals with chronic schizophrenia in a multi-site randomized controlled trial. In addition, we explored whether BA would improve other psychiatric symptoms, quality of life and neuro-cognitive functioning.
**Methods:** For multi-site trials, mental health professionals were trained with BA manual (Choi et al., 2015) and their fidelity was checked by the authors. BA was delivered in a group format once a week for 10 weeks. Participants aged 18 years or older were recruited from community mental health centers and day hospitals in Seoul and Gyeonggi-do area. A total of seventy-two patients with negative symptoms of schizophrenia were randomly assigned into either BA+TAU or TAU. As a primary outcome, negative symptoms were measured using clinical interviews (e.g., BNSS, CAINS, PANSS negative symptoms factor) and self-report questionnaires (i.e., MAP-SR) before and after the 10-week treatments. The secondary outcome measures included other psychiatric symptoms, quality of life, and neuro-cognitive assessment.
**Results:** BA was well accepted by community dwelling individuals with chronic schizophrenia (drop-out rates of BA+TAU and TAU, 10% and 14%, respectively). Intention-to-treat analyses indicated that compared to TAU condition, BA+TAU group showed greater improvement in negative symptoms, as measured by CAINS, BNSS, and PANSS negative symptom factor (Time*Group interaction effects, F=7.476, p<.01 for CAINS total; F=5.663, p<.05 for BNSS total; F=6.092, p<.05 for PANSS negative symptoms factor). In addition, the results indicated group differences in favor of BA+TAU on the PANSS general psychopathology factor and the Quality of Life, but not neurocognitive functioning (Time*Group interaction effects, F=5.660, p<.05 for PANSS general psychopathology factor; F=7.541, p<.01 for QOL total).
**Discussion:** The results of the current study demonstrate the feasibility and the efficacy of BA+TAU for negative symptoms of community dwelling individuals with chronic schizophrenia as compared to TAU when delivered by BA trained mental health professionals. Thus, it is speculated that BA is an effective adjunct psychosocial approach to usual comprehensive psychiatric rehabilitation for negative symptoms. Since the current study is ongoing and follow-up data will be available by the time of presentation at SIRS 2018, it will be examined whether benefits of BA would be maintained 3 months after the termination of 10-week BA treatment.

### F59. VISUALIZING MENTAL REPRESENTATION OF TRUSTWORTHY FACES IN SCHIZOPHRENIA

Neeltje Van Haren*[,1], Loek Brinkman[2], Henk Aarts[2], Ron Dotsch[2]
*[1]University Medical Centre Utrecht, Brain Centre Rudolf Magnus; [2]Utrecht University*

**Background:** The ability to perceive, recognize and process own and others' emotions is crucial for efficient and effective social communication. Many different tasks have been used to investigate impairments herein in patients with schizophrenia. Evidence suggests that perception, discrimination and recognition of affective facial expressions are impaired in schizophrenia patients. Importantly, not everyone may interpret the same facial expression similarly. People match their internal representation of specific facial expressions to perceived faces and variation in these internal representations may result in a distortion of social reality. The impairments in face and/or emotion processing and the bias toward a more negative experience may be causally related to degradation of the internal representation itself or to disturbances in the higher-order evaluation of visual input against functionally intact internal representations. In an attempt to develop ways of visualizing an individuals' internal representation of an emotional face on a computer screen, we set out to visualize the representation of a male and a female face.
**Methods:** We use a data-driven technique, i.e. reverse correlation image classification (RCIC), which makes it possible to visualize internal representations of faces on computer screens. Participants judge noisy images of faces that are created by superimposing random noise on a single constant base face. The random noise distorts the base face at the pixel level, generating facial variation across stimuli that is fully unconstrained and unaffected by researchers' a priori expectations. The participants' responses to a large number of faces are used to model the facial information that was idiosyncratically diagnostic for the judgments. This analysis yields a classification image (CI) for each participant, which visualizes the facial characteristics that drive judgments of emotional expressions (i.e., their internal representation).
We introduce an objective metric, i.e. infoVal, using gender as proof-of-principle. infoVal quantifies the probability that an observed CI was not generated by a random process and is equivalent to a modified z score. First, we test the association between infoVal and more common markers of data quality, i.e. the subjective recognizability, objective discriminability and test-retest reliability of CIs (convergent validity). Second, we use RCIC to investigate and reconstruct the mental representation of trustworthiness as expressed on the face in 32 patients with schizophrenia and 39 controls.
**Results:** Subjective ratings showed that male and female CIs were more strongly associated with masculinity and femininity, respectively, when infoVal scores where high (p<.001). Second, infoVal scores were highly correlated with test-retest reliability, i.e., higher scores corresponded with higher test-retest reliability (p<1x10-13).
Preliminary analyses of the RCIC task on the internal representation of trustworthy and untrustworthy faces showed that both patients and controls are capable of performing the task adequately. Data-driven multidimensional scaling of the classification images implicate 3 clusters of images, reflecting untrustworthy, neutral, and trustworthy faces. These first analyses suggest that there is no evidence for differences in internal representation of (un)trustworthy faces between patients and controls.
**Discussion:** We showed how infoVal scores can facilitate the interpretation of CIs. This opens the way to comprehensively investigate internal representations of emotional faces in patients with schizophrenia.

### F60. INFLAMMATORY MARKERS AND COGNITIVE PERFORMANCE IN PATIENTS WITH SCHIZOPHRENIA TREATED WITH LURASIDONE

Andrei Pikalov*[,1], Brian Miller[2], Cynthia Siu[3], Michael Tocco[1], Joyce Tsai[1], Philip Harvey[4], Antony Loebel[1]

[1]Sunovion Pharmaceuticals, Inc.; [2]Medical College of Georgia, Georgia Regents University; [3]COS and Associates Ltd., Central District; [5]Leonard M. Miller School of Medicine, University of Miami

**Background:** Recent studies have linked inflammation, obesity, and lipid dysregulation with cognitive impairment, a core feature of schizophrenia. Elevated C-reactive protein concentration has been shown to be a reliable biomarker for inflammatory states. We conducted an exploratory analysis to investigate the potential influence of inflammation, obesity and lipid metabolism on changes in symptom severity and cognitive performance in patients with schizophrenia treated with lurasidone.
**Methods:** Patients with acute exacerbation of schizophrenia were treated with one of two fixed doses of lurasidone (80 or 160 mg/day), placebo, or 600 mg/day quetiapine XR in a 6-week double-blind study. A wide-range CRP (wr-CRP) assay (equivalent to high sensitivity CRP assay) was used to assess levels of inflammation. CRP was evaluated as a logarithm transformed (log) continuous variable and as a categorical variable divided into low (≤ 2 mg/L), medium (> 2 mg/L and ≤ 5 mg/L) and high (> 5 mg/L) subgroups. Cognitive function was assessed with the CogState computerized cognitive battery at baseline and week 6 endpoint. Nonparametric bootstrap resampling method was applied to estimate the main and interactive effects of CRP on ranked cognitive scores.
**Results:** Elevated level of wr-CRP (log) was associated with cognitive impairment at study baseline (P < 0.05), with significantly lower cognitive performance in the subgroup with high wr-CRP (> 5 mg/L) compared to those with low wr- CRP (< 2 mg/L) at study baseline (P < 0.05). Higher level of CRP (log) was also associated with significantly greater symptom severity as assessed by PANSS score, as well as higher BMI/body weight, and lower levels of high-density lipoprotein (HDL) and high hemoglobin A1c (HbA1c) at study baseline (P < 0.05). No significant associations were found for wr-CRP (log) with low-density lipoprotein (LDL) and total cholesterol at study baseline. High wr-CRP level (> 5 mg/L) at study baseline predicted less improvement of cognitive composite score at week 6 endpoint for all treatment groups, compared to those with low to medium wr-CRP levels (< 5 mg/L).
The joint effect of wr-CRP (log) and HDL or HOMA-IR on moderating procognitive effects of lurasidone was significant (P<0.05), with greater lurasidone (vs. placebo) effect size in patients with either low wr-CRP and high HDL concentration or lower levels of both wr-CRP and HOMA-IR. Lurasidone treatment was associated with significant reduction in symptom severity as assessed by PANSS, CGI-S, and MADRS change scores from baseline to week 6, independent of wr-CRP, HDL and HOMA-IR levels at study baseline. Lurasidone had no significant effect on change in wr-CRP level from baseline to week 6 endpoint.
**Discussion:** Our findings from this exploratory analysis of a placebo-controlled trial in patients with schizophrenia suggest that the joint effects of low wr-CRP level combined with either high HDL or low HOMA-IR can predict cognitive improvement in patients treated with lurasidone (vs. placebo). These findings suggest that inflammation and its interactive effects with insulin resistance and lipid parameters in patients with schizophrenia might impact cognition and response to treatment with antipsychotics.

### F61. THE RELATIONSHIP OF AGE AND SYMPTOMS WITH COGNITIVE PLANNING IN SCHIZOPHRENIA

Dimitrios Kontis*,[1], Alexandra Giannakopoulou[1], Eirini Theochari[1], Angeliki Andreopoulou[1], Spyridoula Vassilouli[1], Dimitra Giannakopoulou[1], Eleni Siettou[1], Eleftheria Tsaltas[2]
[1]Psychiatric Hospital of Attica; [2]Athens University Medical School

**Background:** The relationship of age and symptoms with the performance on non-verbal cognitive planning tasks in schizophrenia could be useful for the development of cognitive remediation programmes.
**Methods:** During a cross-sectional study, 97 medicated and stabilized patients with chronic schizophrenia (61 males and 36 females, mean age=43.74 years, standard deviation-SD=11.59), which were consecutively referred to our Unit, were assessed using the Stockings of Cambridge (SOC) task of the Cambridge Neuropsychological Test Automated Battery (CANTAB) and the Positive and Negative Syndrome Scale (PANSS). Linear regression analyses were conducted in order to investigate the correlations of symptoms and age with SOC performance.
**Results:** Age and PANSS total scores negatively correlated with optimal SOC solutions (problems solved in minimum moves) (age: B=-0.05, 95% CI=-0.089, -0–012, df=86, t=-2.599, p=0.011, symptoms: B=-0.047, 95%CI=-0.071, -0.024, df=86, t=-3.982, p<0.001). The effects of total symptoms were driven by positive (B=-0.149, 95%CI=-0.229, -0.068, df=86 t=-3.672, p<0.001), negative (B=-0.087, 95%CI=-0.150, -0.023, df=86, t=-2.717, p=0.008) and general psychopathology symptoms (B=-0.065, 95%CI=-0.108, -0.023, df=86, t=-3.045, p=0.03). PANSS total scores positively correlated with mean excess moves in 2- (B=0.007, 95%CI=0.002, 0.012, df=86, t=2.656, p=0.009), 3- (B=0.014, 95%CI=0.005, 0.023, df=86, t=2.951, p=0.004) and 5-move (B=0.026, 95%CI=0.008, 0.044, df=86, t=2.923, p=0.004) problems and age only in 4- (B=0.026, 95%CI=0.006, 0.046, df=86, t=2.571, p=0.012) and 5-move (B=0.032, 95%CI=0.002, 0.061, df=86, t=2.152, p=0.034) problems. We could not find any association between PANSS scores and age with initial or subsequent thinking times during the SOC task.
**Discussion:** Cognitive planning deficits in schizophrenia are associated with patients' symptoms and age. Whereas the effect of symptoms appears to be independent of task difficulty, the age effect emerges when the planning tasks become more complex. The role of drugs remains to be examined in future analyses.

### F62. RISKY DECISION-MAKING PERFORMANCE IN PATIENTS WITH EARLY SCHIZOPHRENIA-SPECTRUM DISORDER

Sin Ki Luk[1], Tatia M.C. Lee[1], Eric YH Chen[1], Wing Chung Chang*,[1]
[1]The University of Hong Kong

**Background:** Dysfunction in risky decision-making has been regarded as one potential contributing factor to functional impairment exhibited in patients with schizophrenia. Literature has revealed suboptimal risky decision-making in chronic schizophrenia patients. However, abnormality in risky decision-making has not been investigated in the early stage of illness. This study aimed to examine whether early schizophrenia patients displayed aberrant risky decision-making using two well-validated paradigms including Balloon Analogue Risk Task (BART; Lejuez et al., 2002) and Risky-Gains Task (RGT; Paulus et al., 2003).
**Methods:** Thirty-three clinically-stable patients diagnosed with DSM-V schizophrenia-spectrum disorder (including schizophrenia, schizoaffective disorder or schizophreniform disorder) were recruited from specialized early intervention service for psychosis in Hong Kong. A group of healthy controls (n=32), matched with age, gender and educational levels, was enrolled for comparison. All participants were evaluated with a brief battery of cognitive assessment and two computerized risky decision-making tasks. Symptom assessment was also conducted for patients.
**Results:** In both BART and RGT, patients with early schizophrenia-spectrum disorder performed worse than healthy controls regarding total points gained and reaction time. In BART, patients had significantly lower adjusted scores (F(1,63)=7.8, p<0.05) and lower balloon exploration rates than controls (F(1,63)=11.5, p<0.001), indicating that patients exhibited a tendency toward risk-aversion. In GRT, three-way analysis of variance revealed significant group x response interaction (F(1,63)=7.8, p<0.05), with post-hoc independent t-test showing that patients significantly preferred safe over risky options than controls (t=2.6, p<0.05). There were no significant correlations of risky decision-making parameters with symptom ratings and cognitive functions.

**Discussion:** We extend previous findings of chronic samples to patients with early schizophrenia-spectrum disorder and indicate that suboptimal risky decision-making with risk-aversion preference has also been observed in the early course of illness. Further research is warranted to clarify the longitudinal change of aberrant risk-aversive behavioral patterns and its relationship with prospective functional and symptom outcomes.

### F63. COGNITIVE CORRELATES OF THE NEGATIVE SYMPTOMS EXPRESSIVE AND EXPERIENTIAL DEFICIT FACTORS IN PSYCHOSIS

Serge Sevy[1], Anzalee Khan[1], Jean-Pierre Lindenmayer*[,2], Susan McGurk[3], Benedicto Paker[1], Isidora Ljuri[1], Mohan Parak[1], Abraham Goldring[1]
*[1]Manhattan Psychiatric Center; [2]New York University; [3]Boston University*

**Background:** Primary negative symptoms of schizophrenia contribute heavily to functional disability. Treatment of these symptoms continues to be a major unmet need, even when positive symptoms are controlled. Recent factor analyses of negative symptoms using the PANSS and other symptom assessments in patients with schizophrenia have identified two factors of negative symptoms: expressive and experiential deficits. These two factors most likely have very different clinical, neurocognitive and neurobiological correlates. This study examines the clinical and cognitive correlates associated with expressive and experiential deficits in a large cohort of patients with psychosis before and after computerized cognitive remediation.

**Methods:** This is a secondary data analysis of subjects enrolled in a cognitive remediation program for 12 weeks. One hundred fifty-one subjects age 18 - 55 with a DSM IV-TR diagnosis of schizophrenia, schizoaffective disorder or bipolar disorder were enrolled. Assessments of demographic, psychopathology (PANSS), cognition (MCBB), and daily living skills (UPSA-Brief) were conducted at baseline and endpoint. Exploratory (EFA) and confirmatory (CFA) factor analyses of PANSS items as well as Pearson's correlations between factors, demographics, MCCB, and UPSA-Brief scores were examined at baseline and endpoint.

**Results:** EFA baseline PANSS data resulted in the five-factor model of the PANSS with seven items attributed to the Negative Symptom Factor (NSF; N1, blunted affect; N2, emotional withdrawal; N3, poor rapport; N4, passive social withdrawal; N6, lack of spontaneity and flow of conversation; G7, motor retardation; and G16, active social avoidance). CFA of the NSF revealed a two-factor model consisting of an Expressive Deficit (N1, N3, N6, G7), and an Experiential Deficit (N2, N4, and G16). Difference tests comparing the one-factor and two-factor models found that the two-factor model exhibited significantly better fit than the one-factor model ($\chi 2 = 67.117$, df = 1, $p \leq 0.001$; CFI = 0.92; Tucker–Lewis index TLI = 0.91; root mean square error of approximation RMSEA = 0.040; and Goodness of Fit index GFI = 0.93). There were significant correlations between the Expressive Deficit factor score and cognition: TMT- A (r=-0.259, p=0.001), BACS Symbol coding (r=-0.287, p=0.001), Category Fluency (r=-0.342, p=0.001), Hopkins Verbal Learning Test – revised (HTLV-R) (r=-0.236, p=0.05), Letter Number Sequencing (r=-0.256, P=0.001), and NAB Mazes (r=-0.409, p=0.001). The Expressive Deficit factor was also significantly correlated with the neurocognitive domains of Processing Speed (r=-0.352, p=0.001) and Reasoning/Problem Solving (r=-0.338, p=0.001). There were no significant correlations between either factor and UPSA-Brief or the MCCB cognitive composite. There were no significant correlations for change from baseline to endpoint in negative symptoms.

**Discussion:** Our results support the negative symptom two-factor model of Expressive Deficit and Experiential Deficit domains. Only the Expressive Deficit factor was associated with baseline deficits in Working Memory, Processing Speed, Reasoning/Problem Solving and Verbal Learning. The association of the Expressive Deficit factor with significant cognitive impairments supports a more profound neurobiological dysfunction in contrast to the Experiential Deficit factor and may represent an important treatment challenge. The relevance of these findings for the treatment of negative symptoms in schizophrenia will be discussed.

### F64. DISRUPTION IN WORKING MEMORY GATING OBSERVED IN SCHIZOPHRENIA

Rachael Blackman*[,1], Noah Philip[1], David Badre[2]
*[1]Alpert Medical School, Brown University; [2]Brown University*

**Background:** Working memory and cognitive control deficits are hallmarks of schizophrenia. It is not known specifically how gating mechanisms that regulate memory may be disrupted in schizophrenia. Gating mechanisms determine what task-relevant information is selected into working memory while distractors are left out (input gating) and which items stored in working memory are selected for the rule or goal at hand (output gating). The current study investigated whether patients are able to perform the same cognitive control task that is able to dissociate input and output gating processes in a general population, and explored whether schizophrenia patients inappropriately use suboptimal cognitive control strategies (e.g. output gating when input gating can be used).

**Methods:** Patients (n=5) with schizophrenia or schizoaffective disorder were recruited from the Providence VAMC. Participants completed a computer-based cognitive control task. In this task, participants remembered a target item from a sequence of two items in order to select a response. A context (rule) cued which item was relevant to remember, and was presented first in the stimulus sequence (context first) or last (context last). On selective trials, one item in the trial was relevant. On global trials, both items in the trial were relevant.

**Results:** Patients were able to complete the task with minor modifications to adjust for ability to understand the task rules. Preliminary results of reaction time data suggest that patients were challenged at increased cognitive load. Patients performed poorly on trials where participants could use only an input gating strategy (selective first). Preliminary data also suggest that performance in patients tended to be slightly worse for selective first trials where the distractor was presented before the relevant item (i.e. on trials where input gating would be required to keep the distractor out of working memory).

**Discussion:** The current study supports the feasibility of using the cognitive control task selected to investigate gating mechanisms in the schizophrenia patient population. Preliminary data suggest disruption in the ability for patients to optimally use gating strategies and handle cognitive load. Future research will seek to reproduce these preliminary results in a larger sample, as well as compare patient performance to an age-matched control population directly. By understanding how gating mechanisms are disrupted in the patient population, we may be able to better develop therapeutic interventions such as cognitive training strategies to treat cognitive dysfunction in schizophrenia patients.

### F65. NETWORK ANALYSIS OF EMPATHY, SCHIZOTYPY AND AFFECTIVE STATES IN A COLLEGE SAMPLE

Yi Wang*[,1], Wen-hua Liu[2], Hai-song Shi[3], Raymond C. K. Chan[1]
*[1]Institute of Psychology, Chinese Academy of Sciences; [2]School of Health Management, Guangzhou Medical University; [3]North China Electric Power University,*

**Background:** Although the deficits of empathy in schizophrenia spectrum disorders has been recognized in previous studies, little is known about the associations between empathy and schizotypal traits. In this study, we examined the associations among empathy, schizotypy and affective states using the psychological network analysis in a college sample to better understand the social cognition deficits in schizophrenia.
**Methods:** College students (n=1486; male = 574, female = 912; mean age=18.8 years; SD=0.85) were recruited and all of them finished self-reported questionnaire capturing empathy (Interpersonal Reactivity Index, IRI; four subscales: perspective taking, empathic concern, fantasy, personal distress), schizotypy (Wisconsin Psychosis Proneness Scales, including social anhedonia, physical anhedonia, magical ideation and perceptual aberration scales) and affective states (Depression, Anxiety and Stress Scale, 21 items). There were significant sex differences on IRI (female > male for all four subscales, ps < 0.01), DASS depression (male > female, p < 0.01) and schizotypal traits (male > female, ps < 0.05) Psychological networks were constructed taking the subscales of measures as nodes and the edges representing the partial correlation between each pair of nodes controlling all other nodes were estimated using Gaussian graphical model in male and female sample, respectively. Also, the centrality indices, including strength, closeness and betweenness were calculated to identify the central nodes in the network.
**Results:** In males, cognitive empathy (perspective taking and fantasy) showed strong connections with physical anhedonia, while affective empathy (empathic concern) connected with social anhedonia and stress. Personal distress connected with magical ideation and anxiety; fantasy connected with magical ideation. Regarding the centrality, perceptual aberration had the strongest strength, followed by stress; social anhedonia had the highest closeness and betweenness. In females, cognitive empathy (perspective taking and fantasy) showed strong connection with physical anhedonia, affective empathy (empathic concern) connected with social anhedonia. Personal distress connected with anxiety; fantasy connected with magical ideation. Stress showed strongest strength, followed by anxiety and magical ideation; anxiety had highest betweenness; fantasy had highest closeness followed by social anhedonia.
**Discussion:** In the present study, we found that cognitive empathy was strongly connected with physical anhedonia, while affective empathy connected with social anhedonia, regardless of sex. In addition, our findings suggested different network interactions among empathy, schizotypal traits and affective states between males and females. The perceptual aberration and social anhedonia play a central role in the network of males while stress and anxiety are important in females.

### F66. DO CLINICAL VARIABLES DURING THE EARLY ILLNESS PERIOD PREDICT THE COGNITIVE COURSE IN EARLY-ONSET SCHIZOPHRENIA?

Charlotte Teigset*,[1], Christine Mohn[1], Bjorn Rund[1]
[1]*University of Oslo*

**Background:** Early-onset schizophrenia (EOS) affects approximately 5% of the schizophrenia population, and reflects increased disease severity, with a worse clinical course and outcome. Because of extensive brain maturation in the adolescence, the EOS patients provide unique neurodevelopmental data that may contribute to a better understanding of schizophrenia at all ages. Cognitive dysfunction is a central feature of schizophrenia, and is assumed to be more pronounced in EOS than in later onset illness. Previously, we have reported a deteriorated, but stable cognitive course in EOS,[1] and examined the relationship between cognition and symptoms (submitted).[2] While both cognition and clinical variables have been subject to comprehensive research in schizophrenia, the interaction between the two has gained less attention, especially in EOS. An essential question now, is to what extent the longitudinal course of cognition is influenced by clinical variables in the early illness period.
**Methods:** Thirty-one EOS patients and 73 controls (age 12–18) were assessed on clinical variables at baseline (PANSS, duration of untreated psychosis [DUP], hospitalizations, suicide attempts and remission). Neuropsychological assessments with the MATRICS Consensus Cognitive Battery (MCCB) were conducted at baseline, after both one and two years, and composite scores of total performance were calculated. The analyses were performed with a linear mixed model.
**Results:** In the present study, both PANSS-general and suicide attempt history at baseline were identified as risk factors of longitudinal cognitive function. We did not detect a relationship between DUP, remission, positive/negative symptoms and hospitalizations on the one hand, and long-term cognition on the other.
**Discussion:** Some baseline characteristics (psychotic symptoms, DUP, remission and hospitalization) had no influence on cognition within the first two years of illness. In contrast, we found that a higher amount of general symptoms (PANSS) and a history of suicide attempts at baseline significantly predicted a deteriorated longitudinal composite score in EOS. This may imply that cognitive deterioration is influenced by a strong affective response to the illness, rather than a result of irrational or psychotic symptoms in and of themselves. Our findings indicate that higher scores of general symptoms, as well as suicide attempt history, predict a deteriorated cognitive course, and should be subject to specific attention in the evaluation and treatment of patients with early-onset psychosis.
**References:**
1. Juuhl-Langseth, M., Holmen, A., Thormodsen, R., Oie, M., & Rund, B. R. (2014). Relative stability of neurocognitive deficits in early onset schizophrenia spectrum patients. Schizophrenia Research, 156(2–3), 241–247. doi:10.1016/j.schres.2014.04.014
2. Teigset, C., Mohn, C., Brunborg, C., Holmen, A., Juuhl-Langseth, M., Rund, B. (2018). Do clinical characteristics predict the cognitive course in early-onset schizophrenia-spectrum disorders?

### F67. NEUROCOGNITION IN 7-YEAR-OLD CHILDREN OF PARENTS WITH SCHIZOPHRENIA OR BIPOLAR DISORDER

Nicoline Hemager*,[1], Kerstin Plessen[2], Anne Amalie Thorup[3], Camilla J. Christiani[4], Ditte Ellersgaard[3], Katrine Søborg Spang[4], Birgitte Klee Burton[3], Aja Greve[5], Ditte L. Gantriis[6], Anne Søndergaard[3], Maja Gregersen[3], Ole Mors[5], Merete Nordentoft[3], Jens Richardt Møllegaard Jepsen[3]
[1]*Mental Health Centre Copenhagen, Child and Adolescent Mental Health Centre, University Hospital Lausanne;* [3]*Child and Adolescent Mental Health Center, Capital Region of Denmark;* [4]*Research Unit at Mental Health Center, The Lundbeck Foundation Initiative for Integrative Psychiatric Research (iPSYCH);* [5]*Aarhus University Hospital;* [6]*Aarhus University Hospital, The Lundbeck Foundation Initiative for Integrative Psychiatric Research (iPSYCH).*

**Background:** Children of parents with schizophrenia or bipolar disorder display neurocognitive deficits. However, studies of schizophrenia offspring and bipolar offspring at the same age are lacking. The objective was to compare neurocognitive abilities in 7-year-old children of parents with schizophrenia or bipolar disorder with neurocognitive abilities in children of parents without these disorders.
**Methods:** In this nationwide cohort study we assessed 522 7-year-old children (schizophrenia offspring: N=202, bipolar offspring: N=120, and controls=200) with a detailed and well validated neurocognitive test battery. We compared the neurocognitive test scores of the three study groups.
**Results:** Children of parents with schizophrenia showed neurocognitive deficits, whereas children of parents with bipolar disorder displayed neurocognitive abilities comparable to the control group.

**Discussion:** Neurocognitive deficits are numerous in 7-year-old children of parents with schizophrenia, which supports the neurodevelopmental model of schizophrenia. Unimpaired neurocognitive abilities in children of parents with bipolar disorder indicate different neurodevelopmental manifestations in these high risk populations at this early age. Our results call for early identification of schizophrenia offspring with cognitive dysfunctions.

### F68. PREMORBID IQ, EDUCATIONAL LEVEL AND JUMPING TO CONCLUSIONS AS PREDICTORS OF CLINICAL OUTCOME AT FIRST ONSET OF PSYCHOSIS OVER THE NEXT 4 YEARS: THE GAP STUDY

Victoria Rodriguez*,[1], Olesya Ajnakina[1], Simona Stilo[1], Valeria Mondelli[1], Tiago Reis Marques[1], Antonella Trotta[1], Giada Tripoli[1], Diego Quattrone[1], Marco Colizzi[1], Poonam Sood[1], Ben Wiffen[1], Paola Dazzan[1], Evangelos Vassos[1], Marta Di Forti[1], Anthony David[1], Robin Murray[1]
[1]*Institute of Psychiatry, Psychology & Neuroscience, King's College London*

**Background:** Cognition and more recently social cognition, have been shown to be a strong predictor of clinical and functional outcome in psychosis. Jumping to Conclusions (JTC), which is defined as the proneness to require less information before forming beliefs or making a decision, has been related to the formation and maintenance of delusions. However, its relevance to longer-term outcome is unclear. On the other hand, there is evidence in the literature to suggest differences of patterns in clinical outcome and service based ethnicity. Using data from the GAP case-control study of first-episode psychosis (FEP), we set out to test whether the premorbid IQ, educational level and presence of JTC would predict poor clinical outcome at 4 year controlling for ethnicity.
**Methods:** 431 FEP patients were assessed with the positive and negative syndrome scale (PANSS) and Global Assessment of Functioning (GAF). Premorbid IQ was measured by the National Adult Reading Test (NART) scale, probabilistic reasoning "Beads" task was applied and educational levels were recorded alongside with socio-occupational variables at the time of recruitment. Follow-up data over an average period of 4 years were obtained from the electronic psychiatric clinical records in the South London and Maudsley NHS Foundation Trust (SLaM); including items concerning clinical course and outcomes (remission, intervention of police, use of involuntary treatment – the Mental Health Act (MHA) -, and inpatient days). We build different regression models using separately premorbid IQ, education level and JTC as predictors for each clinical outcome, both unadjusted and adjusted by ethnicity, age and gender.
**Results:** Higher educational level was predictor of clinical remission [adjusted OR=1.9, 95% confidence interval (CI) 1.2–3, p=0.005]. FEP who presented JTC at baseline were more likely during the follow up period to be detained under the MHA [adjusted OR=11.23, 95% confidence interval (CI) 2.64–47.76, p=0.001], require intervention by the police (adjusted OR=10.76, 95% CI 2.4–48.26, p=0.002) and have longer admissions (adjusted IRR=4.04, 95% CI 1.43–11.36, p=0.008). We couldn't find any predictor effect for clinical outcome for premorbid IQ. The association with level of education and JTC was not accounted for by socio-demographic variables including ethnicity.
**Discussion:** Although we did not find association with premorbid IQ, educational level as indirect proxy of neurocognition showed a predictor effect for clinical remission. JTC in FEP is associated with serious subsequent consequences in terms of social disturbance and a poor therapeutic alliance. Our findings raise the question of whether the implementation of specific interventions to reduce JTC, such as Metacognition Training, may be a useful addition in early psychosis intervention programs.

### F69. MUSCARINIC M1 RECEPTOR SEQUENCE VARIATION AND GENERAL COGNITION

Sean Carruthers*,[1], Kiymet Bozaoglu[2], Caroline Gurvich[3], Philip Sumner[4], Eric Tan[5], Elizabeth Thomas[5], Susan Rossell[6]
[1]*Swinburne University;* [2]*Bruce Lefroy Centre for Genetic Health Research, Murdoch Children's Research Institute, University of Melbourne;* [3]*Monash Alfred Psychiatry Research Centre, The Alfred Hospital and Monash University;* [4]*Swinburne University, Monash Alfred Psychiatry Research Centre, The Alfred Hospital;* [5]*Monash Alfred Psychiatry Research Centre, Brain and Psychological Sciences Research Centre, Swinburne University;* [6]*Monash Alfred Psychiatry Research Centre, The Alfred Hospital and Monash University, Central Clinical School, Brain and Psychological Sciences Research Centre, Swinburne University of Technology, The University of Melbourne and St. Vincent's Hospital*

**Background:** It has been reported that individuals with schizophrenia who are homozygous at the c.267C > A single nucleotide polymorphism (rs2067477) within the cholinergic muscarinic M1 receptor gene exhibit impaired Wisconsin Card Sorting Test (WCST) performance compared to those who are heterozygous. This investigation sought to examine the influence rs2067477 genotype variation has on general cognitive function.
**Methods:** 87 individuals with schizophrenia/schizoaffective disorder (Sz/SAD) and 224 healthy controls (HC) completed the MATRICS Consensus Cognitive Battery and D-KEFS Stroop to determine whether rs2067477 genotype variation influenced cognition.
**Results:** No significant differences in MCCB domain scores or D-KEFS Stroop were found across genotype in both a patient-only sample and a combined patient-healthy control sample
**Discussion:** Despite rs2067477 genotype variation being shown to influence executive functioning, specifically performance on the WCST in individual with schizophrenia, no such association could be detected across a number of general cognitive domains or on an alternative measure of shifting/cognitive flexibility.

### F70. COMPUTERIZED SOCIAL COGNITIVE TRAINING (SCT) IMPROVES COGNITION AND RESTORES FUNCTIONAL CONNECTIVITY IN RECENT ONSET PSYCHOSIS: AN INTERIM REPORT

Shalaila Haas*,[1], Nikolaos Koutsouleris[2], Anne Ruef[2], Bruno Biagianti[3], Joseph Kambeitz[2], Dominic Dwyer[2], Ifrah Khanyaree[2], Rachele Sanfelici[2], Lana Kambeitz-Ilankovic[2]
[1]*International Max Planck Research School in Translational Psychiatry;* [2]*Ludwig-Maximilian-University;* [3]*University of California, San Francisco*

**Background:** Neurocognitive impairments are a core and enduring feature of psychosis that continue to persist despite pharmacological interventions. Neurocognitive interventions have emerged as a supplementary treatment option to improve cognition in early psychosis patients. Recently, focus has shifted to using social cognitive training (SCT) as evidence suggests that targeting social cognition may lead to improvements not only in cognition but also in real-world functioning (Horan et al., 2011). This improvement is thought to be mediated by restoration of functional brain activity in patients undergoing neurocognitive interventions, especially associated with medial prefrontal cortex (Hooker et al., 2014). In this study, we report our interim findings of the effects of a 10-hour SCT on cognition and resting-state functional connectivity (rsFC). Our hypothesis was that training would improve cognition and normalize functional connectivity.

**Methods:** In this randomized-controlled study, one recent onset psychosis (ROP) patient arm (n=18) underwent a 6-week (10-hour) computerized SCT (Brain HQ, Posit Science, https://www.brainhq.com/), while another naturalistic arm (n=18) received treatment as usual (TAU). Both treatment arms were assessed on a battery of neurocognitive tests and underwent a multimodal imaging protocol, including a 10 min resting-state fMRI, at two timepoints (baseline, T0; follow-up, FU). Seed-based voxel-wise rsFC was performed and individual-level rsFC correlation maps were calculated between the bilateral medial prefrontal cortex (mPFC) and the whole brain.
**Results:** The SCT group showed significant improvements in the domain of spatial working memory (p<0.05), processing speed (p<0.05) and resilience to both immediate and delayed memory decline over 6 weeks (p<0.05), as compared to TAU. Comparison of FC between the two measurement time points, suggested increased FC between mPFC and left inferior temporal gyrus (ITG), as well as increased FC between mPFC and left somatosensory area in ROP patients that underwent SCT relative to TAU.
**Discussion:** We have shown improvements in processing speed, verbal memory, and spatial working memory that agree with previous studies using computerized cognitive interventions. Moreover, the improvement in the spatial working memory domain was significant in the most demanding test condition with 10 elements - indicative of benefits from the fine-tuning of higher-level executive functions. The neuroimaging results also suggested that the improvements may have been mediated by the improvement of FC in regions typically associated with social cognition and facial recognition (Adolphs et al., 2009). These results are in line with recent studies investigating not only the feasibility of SCT as an intervention, but also the effects on cognition and underlying neural alterations resulting from intensive computerized neurocognitive interventions (Hooker et al., 2012; Nahum et al., 2014; Subramaniam et al., 2014). Future studies using machine learning methods will be necessary to determine functional biomarkers in order to personalize SCT at the individual-level.

### F71. THE STRUCTURE OF NEUROCOGNITION ACROSS CHILDHOOD AND ADULTHOOD IN YOUNG PEOPLE WITH PSYCHOSIS.

Josephine Mollon*[,1], Anthony David[2], Stanley Zammit[3], Glyn Lewis[4], Avi Reichenberg[5]
*[1]Yale University; [2]Institute of Psychiatry, King's College London; [3]Cardiff University & University of Bristol; [4]University College London; [5]Icahn School of Medicine at Mount Sinai*

**Background:** There is substantial evidence for connection abnormalities in the brains of schizophrenia patients. However, little is known about the structure of cognitive functioning across the psychosis spectrum.
**Methods:** We used data from the Avon Longitudinal Study of Parents and Children (ALSPAC). Data from all individuals who underwent cognitive testing at age 8 and psychiatric assessment at 18 years were used to examine network structure of cognition in childhood (age 8). A subsample of individuals who underwent further cognitive testing at age 20 was used to examine change in cognitive network structure between childhood (age 8) and adulthood (age 20). Networks comprised nodes (cognitive tests) joined together by edges (partial correlations). Organization of subnetworks by cognitive domains (verbal, perceptual, working memory and processing speed) and measures indicating 1) important cognitive tests or hubs, 2) network integration and 3) network density, were examined. Participants with non-affective psychotic disorder, affective psychotic disorder, psychotic experiences and depression were compared to controls.
**Results:** In childhood, affective and non-affective psychosis groups showed disruption to cognitive subnetworks and hubs, as well as greater network connectivity (β=0.44, p<.001, β=0.16, p<.001), dysconnectivity (β=−0.47, p<.001, β=−0.19, p=.002), integration (β=−12.7, p<.001, β=−10.2, p<.001) and density (β=0.49, p<.001, β=0.17, p<.001). The psychotic experiences group showed intact subnetworks and hubs, but increased network integration (β=−5.5, p<.001) and density (β=0.02, p=.04). The depression group also showed intact subnetworks and hubs, but increased integration (β=−5.9, p<.001). Between childhood and adulthood increasing density was seen in the psychotic experiences group (β=0.09, p=.04), and the depression group showed increasing integration (β=−3.15, p=.04). Controls showed increasing reliance on the working memory hub between childhood and adulthood, while all other groups remained reliant on attention and visuospatial abilities.
**Discussion:** Overall, individuals with psychotic disorder showed substantial qualitative and quantitative differences in cognitive network structure. Individuals with psychotic experiences and depression showed more subtle deviations. Abnormalities in cognitive network structure were seen even in the absence of cognitive impairment, suggesting the importance of looking beyond deficits to how performance is achieved.

### F72. NEUROCOGNITION AND ADAPTIVE FUNCTIONING IN THE 22Q11.2 DELETION SYNDROME MODEL OF SCHIZOPHRENIA

Fiksinski Ania*[,1], Elemi Breetvelt[2], Jacob Vorstman[3], Eva Chow[2], Erin Lee[2], Lisa Palmer[2], Erik Boot[2], Nancy Butcher[2], Rene Kahn[1], Anne Bassett[2]
*[1]UMC Utrecht; [2]University of Toronto; [3]UMC Utrecht, University of Toronto*

**Background:** Identifying factors that influence functional outcome is an important goal in schizophrenia research. These factors, including overall cognitive functioning (IQ) and more specific domains of neurocognitive functioning, may not only aid in identifying those individuals at greatest risk for poor functional outcome but could inform potentially targetable treatment objectives. The 22q11.2 deletion syndrome (22q11DS) is a unique genetic model with high risk (20–25%) for schizophrenia. This study aimed to identify potentially targetable domains of neurocognitive functioning associated with functional outcome in adults with 22q11DS.
**Methods:** Using data available from a comprehensive battery of 15 neurocognitive tests for 99 adults with 22q11DS (n=43 with schizophrenia) we derived four domains of neurocognition (Verbal memory, Visual memory, Motor functioning, and Executive performance) using a principal component analysis. To investigate the association of these domains with adaptive functioning, we used Vineland Adaptive Behavior Scales (VABS) data available for 84 subjects in a logistic regression model that accounted for the effects of schizophrenia status and overall intellectual level.
**Results:** The regression model explained 46.8% of the variance in overall functional outcome (p < 0.0001) and 47.7% of the variance on the daily living skills subdomain (p < 0.0001). Executive performance was significantly associated with subsequent functional outcome (p = 0.046); age and schizophrenia were also significant factors. VABS adaptive functioning scale scores were higher in those with better performance on Executive domain tests, no psychotic illness, and older age. The effects of Executive Performance on functioning did not significantly differ between those with and without psychotic illness.
**Discussion:** The significant relationship between Executive Performance and functional outcome is a novel addition to our understanding of cognitive factors that may contribute to the variability in functional outcome in schizophrenia high-risk groups. The results provide impetus for further studies of Executive Performance as a potential target of early intervention strategies to mitigate risk for schizophrenia and functional deterioration.

### F73. COGNITIVE CLUSTERING IN SCHIZOPHRENIA PATIENTS, THEIR FIRST-DEGREE RELATIVES AND HEALTHY SUBJECTS IS ASSOCIATED WITH ANTERIOR CINGULATE CORTEX VOLUME

Kazutaka Ohi*[,1], Takamitsu Shimada[1], Kiyotaka Nemoto[2], Yuzuru Kataoka[1], Toshiki Yasuyama[1], Kohei Kimura[1], Hiroaki Okubo[1], Takashi Uehara[1], Yasuhiro Kawasaki[1]
*[1]Kanazawa Medical University; [2]Institute of Clinical Medicine, University of Tsukuba*

**Background:** Cognitive impairments are a core feature in schizophrenia patients and are also observed in first-degree relatives of the schizophrenia patients. However, substantial variability in the impairments exists within and among schizophrenia patients, first-degree relatives and healthy controls. A cluster-analytic approach can group individuals based on profiles of traits and create more homogeneous groupings than predefined categories.
**Methods:** Here, we investigated differences in the Brief Assessment of Cognition in Schizophrenia (BACS) neuropsychological battery (six subscales) among 81 schizophrenia patients, 20 unaffected first-degree relatives and 25 healthy controls. To identify three homogeneous and meaningful cognitive groups regardless of categorical diagnoses (schizophrenia patients, first-degree relatives and healthy controls), cognitive clustering was performed using a k-means clustering analysis approach, and differences in the BACS subscales (verbal memory, digit sequencing, token motor, verbal fluency, symbol coding and Tower of London) among the cognitive cluster groups were investigated. Finally, the effects of diagnosis and cognition on brain volumes were examined.
**Results:** As expected, there were significant differences in the five BACS subscales among the diagnostic groups (verbal memory, $F_{2,123}=20.6$, $P=1.90 \times 10^{-8}$; digit sequencing, $F_{2,123}=8.0$, $P=5.65 \times 10^{-4}$; token motor, $F_{2,123}=16.0$, $P=6.92 \times 10^{-7}$; verbal fluency, $F_{2,123}=14.8$, $P=1.79 \times 10^{-6}$ and symbol coding, $F_{2,123}=28.8$, $P=5.64 \times 10^{-11}$). The cluster-analytic approach generated three meaningful subgroups: (i) neuropsychologically normal (Cluster 1, N=36), (ii) intermediate impaired (Cluster 2, N=60) and (iii) widespread impaired (Cluster 3, N=30). The cognitive subgroups were mainly affected by the clinical diagnosis ($\chi^2=46.7$, $P=5.33 \times 10^{-10}$), and significant differences in all BACS subscales among clusters were found (verbal memory, $F_{2,123}=64.1$, $P=8.49 \times 10^{-20}$; digit sequencing, $F_{2,123}=35.7$, $P=5.89 \times 10^{-13}$; token motor, $F_{2,123}=71.7$, $P=2.29 \times 10^{-21}$; verbal fluency, $F_{2,123}=84.2$, $P=9.05 \times 10^{-24}$; symbol coding, $F_{2,123}=115.6$, $P=5.70 \times 10^{-29}$ and Tower of London, $F_{2,123}=6.9$, $P=1.43 \times 10^{-3}$). The effects of the diagnosis (SCZ<FR<HC) and cognitive clusters (Clusters 3<2<1) on brain volumes overlapped in the frontal, temporal and limbic regions. Frontal and temporal volumes were mainly affected by the diagnosis, whereas the anterior cingulate cortex volumes were affected by the additive effects of diagnosis and cognition (FWE-corrected $P<0.05$, x, y, z=1.5, 40.5, 19.5, T=5.49).
**Discussion:** We investigated the cognitive heterogeneity and cognitive continuum among schizophrenia patients, first-degree relatives and healthy controls. The cognitive clustering approach without using clinical diagnoses successfully produced more homogeneous cognitive clusters: a neuropsychologically normal, an intermediately impaired and a globally impaired cognitive cluster. Clinical diagnoses (healthy controls, first-degree relatives and schizophrenia patients) were not evenly distributed into the three clusters; i.e., these clusters were mainly affected by clinical diagnoses. Both diagnoses and cognitive clusters were associated with decreased anterior cingulate cortex volumes. Our findings demonstrate a cognitive continuum among schizophrenia patients, first-degree relatives and healthy controls and support the hypothesis that cognitive impairments and the related anterior cingulate cortex volumes would be useful intermediate phenotypes in the pathophysiology of schizophrenia.

### F74. CLINICAL SYMPTOMS AND NOT OBJECTIVE COGNITIVE PERFORMANCE DRIVE SUBJECTIVE COGNITIVE COMPLAINTS IN PATIENTS WITH SCHIZOPHRENIA

Jack Cotter*[,1], Kiri Granger[1], John Evenden[1], Jennifer Barnett[1], Michael Sand[2]
*[1]Cambridge Cognition; [2]Boehringer Ingelheim*

**Background:** The United States Food and Drug Administration (FDA) recommends that drugs that exert a pro-cognitive effect should be accompanied by measurable improvements in 'real-world' functioning. Patients with schizophrenia typically exhibit substantial impairments across a wide range of cognitive domains, representing an important target for therapeutic intervention. This has led to the development of several instruments specifically for use in this population that seek to capture the subjective experience and impact of cognitive dysfunction on daily living for use as co-primary endpoints in clinical trials. However, it remains unclear to what extent these accurately reflect objective cognitive performance.
**Methods:** We conducted a secondary analysis of data from 413 patients with schizophrenia who participated in a multi-national randomized, double-blind, placebo-controlled trial. During the trial, participants completed two different neurocognitive test batteries, the Cambridge Neuropsychological Test Automated Battery (CANTAB) and the MATRICS Consensus Cognitive Battery (MCCB). They also completed two measures of subjective cognitive performance, the Schizophrenia Cognition Rating Scale (SCoRS) and the Patient Reported Experience of Cognitive Impairment in Schizophrenia (PRECIS). The severity of patient's symptoms was also assessed at each time point using the Positive and Negative Syndrome Scale (PANSS). All assessments were conducted at baseline and at a 12-week follow-up assessment (end of treatment). We examined the associations between these variables using correlational and multivariable linear regression analyses.
**Results:** Scores on each of the subjective measures of cognition were weakly correlated in the expected direction with objective measures of cognitive performance across both the CANTAB and MCCB tasks. Scores on each of the subjective cognition measures were more strongly associated with severity of symptoms as assessed using the PANSS. Multivariable regression analyses suggested that clinical symptoms accounted for significantly greater variance in subjective cognition scores than either objective cognitive performance or demographic factors.
**Discussion:** Subjective appraisals of cognition are poor predictors of objective cognitive performance in patients with schizophrenia. The burden reported by patients on these instruments appears to be more closely associated with the severity of their clinical symptoms. This has important implications for the use of these measures as co-primary endpoints in clinical trials assessing pro-cognitive drug effects.

### F75. MEDICATION ADHERENCE AND DISCONTINUATION IN PATIENTS WITH SCHIZOPHRENIA TREATED WITH ARIPIPRAZOLE ONCE-MONTHLY LONG-ACTING INJECTABLE VERSUS THOSE TREATED WITH ORAL ANTIPSYCHOTICS

Mallik Greene[1], Tingjian Yan[2], Eunice Chang[2], Ann Hartry*[,3], Beth Pulasky[1]
*[1]Otsuka Pharmaceutical Development & Commercialization, Inc. (OPDC); [2]Partnership for Health Analytic Research, LLC; [3]Lundbeck*

**Background:** Adherence to antipsychotic treatment is essential in treating schizophrenia symptoms and in preventing costly relapse. This study aimed to compare medication adherence and discontinuation in patients with schizophrenia treated with aripiprazole once-monthly long-acting injectable antipsychotic (LAI; AOM 400) to those who changed to a different oral antipsychotic.
**Methods:** This retrospective cohort analysis used the Truven Health Analytics MarketScan® Medicaid, commercial, and supplemental Medicare claims databases. In patients ≥18 years old with schizophrenia, two mutually exclusive cohorts were created: the AOM 400 cohort, patients who initiated AOM 400 between 01/01/2013-06/30/2015 (the ID period); and the oral cohort, patients who changed to a different oral antipsychotic during the ID period. AOM 400 or new oral therapy initiation was the index date. Patients were followed for ≥1 year. Primary outcome measures were adherence (proportion of days covered [PDC]) during 1-year post-index and index medication discontinuation (gap ≥60 days) during entire follow-up. Cox regression and linear regression models were used to estimate risk of discontinuation and PDC, respectively, adjusting for demographic and clinical characteristics, insurance type, baseline medication, and baseline ED visits or hospitalizations.
**Results:** The study sample consisted of 408 (10.8%) AOM 400 patients and 3,361 (89.2%) oral antipsychotic patients. AOM 400 patients had better medication adherence (adjusted mean PDC: 57.0% vs. 47.6%, $p<0.001$) than the oral cohort. Sixty-three percent of AOM 400 patients were partially (PDC 40%-79%) to fully adherent (PDC >80%) vs. 51.1% of oral antipsychotic patients ($p<0.001$). AOM 400 patients also had a lower medication discontinuation rate (75.2% vs. 85.0%; $p<0.001$) within 1 year. Median time to discontinuation of AOM 400 was 193 days vs. 89 days for oral antipsychotics ($p<0.001$). In the Cox model, oral antipsychotic patients discontinued their index treatment at a higher rate than AOM 400 patients (hazard ratio: 1.45; $p<0.001$).
**Discussion:** This real-world study suggests that patients with schizophrenia initiating AOM 400 had better medication adherence and lower discontinuation risk than patients who changed to different oral antipsychotics.

### F76. CHILDHOOD TRAUMA RELATED TO ABNORMAL SOCIAL COGNITION IN SCHIZOPHRENIA

Aldara Alvarez Astorga*[,1], Alba Lubeiro Juárez[2], Eva Sotelo[1], Mercedes Vaquero[1], Vicente Molina Rodriguez[2]
[1]*University Hospital of Valladolid;* [2]*University of Valladolid*

**Background:** Childhood trauma has been proposed as a risk factor for schizophrenia. Moreover, it has been related to brain abnormalities associated with cognitive functions, including social cognition. Alterations in mentalizing skills are found in both schizophrenia patients and individuals exposed to childhood trauma. We hypothesize that childhood trauma might be related to emotional processing deficits in psychotic patients.
**Methods:** The present study is ongoing. To date, we have assessed social cognition and childhood trauma in 30 patients with schizophrenia. Social cognition is quantified using Mayer, Salovey and Caruso emotional intelligence test (MSCEIT) with five different categories: i) emotional perception, ii) emotional facilitation, iii) emotional comprehension, iii) emotional management and iv) emotional intellectual quotient dimensions. Early trauma data is collected using Childhood Trauma Questionnaire (CTQ), which yields physical, emotional, sexual abuse and neglect scores. We have assessed the correlation coefficients (Spearman´s rho) between childhood trauma and social cognition scores.
**Results:** According to our preliminary analyses, there are significant inverse correlation coefficients in the patients group between emotional neglect and total trauma scores and, on the other hand, social cognition scores for the facilitation, comprehension, management and emotional intellectual quotient dimensions. Thus, patients with higher scores reflecting more severe emotional neglect and total trauma performed lower in social cognition tests.
**Discussion:** Childhood trauma experiences may contribute to social cognition deficits in schizophrenia.

### F77. OXYTOCIN ENHANCES VISUAL ATTENTION TO FACIAL STIMULI IN PATIENTS WITH SCHIZOPHRENIA: EVIDENCE FROM AN EYE-TRACKING STUDY

Lilla Porffy*[,1], Rebekah Wigton[2], Antoine Coutrot[3], Dan Joyce[1], Isabelle Mareschal[4], Sukhi Shergill[1]
[1]*Institute of Psychiatry, Psychology & Neuroscience;* [2]*Beth Israel Deaconess Medical Center;* [3]*University College London;* [4]*Queen Mary University of London*

**Background:** Deficits in social cognition often develop during the prodromal stages of psychosis, remain stable over the course of the illness, and have a dramatic impact on daily functioning (Fett et al., 2011). Social cue processing, particularly face perception, plays a critical role in social cognitive functioning. Patients with schizophrenia struggle to extract information from faces and interpret facial expressions (Kohler et al., 2010). These deficits may be explained by restricted visual attention. Indeed, eye-tracking studies have demonstrated that people with schizophrenia show reduced exploratory behaviour (i.e. reduced number of fixations and longer fixation durations) in response to facial stimuli compared to healthy controls (e.g. Manor et al., 1999). Oxytocin has been demonstrated to exert pro-social effects on behaviour and modulate eye gaze during perception of faces. In the present study, we tested whether the neuropeptide, oxytocin, has a compensatory effect on visual processing of human faces.
**Methods:** Twenty right-handed male subjects with schizophrenia ($n = 16$) or schizoaffective disorder ($n = 4$) were administered intranasal oxytocin 40UI or placebo in a double-blind, placebo-controlled, cross-over fashion during two visits separated by 7 days. Participants engaged in a free-viewing eye-tracking task, during which they were looking at 6 facial images of two Caucasian men displaying angry, happy, and neutral facial expressions, and 6 control images in a random order. Eye-tracking measures including 1) total number of fixations, 2) dispersion, 3) saccade amplitude, and 4) mean duration of fixations were captured using the EyeLink 1000 system (SR Research Ltd, Ottawa, Ontario, Canada). Four separate 2 x 4 repeated-measures analysis of variance (ANOVA) were carried out to evaluate the within-subject effects of treatment, stimuli, and the interactions between stimuli and treatment ($p < .05$, two-tailed).
**Results:** We found a main effect of treatment ($F_{1,17} = 16.139$, $p = .001$), but not a main effect of stimuli ($F_{3,51} = 1.479$, $p > .231$) on total number of fixations. There was a main effect of treatment on duration of fixation, ($F_{1,13} = 5.455$, $p = .036$) but not a main effect of stimuli ($F_{3,39} = 1.267$, $p = .299$). For dispersion, there was a significant main effect of stimuli ($F_{3,51} = 3.424$, $p = .024$) but no main effect of treatment ($F_{1,17} = 3.170$, $p = .093$). Analysis of saccade amplitudes revealed no main effect of treatment ($F_{1,17} = 2.666$, $p = .121$) or stimuli ($F_{3,51} = 0.289$, $p = .833$). None of the interactions reached significance.
**Discussion:** To our knowledge, this is the first study to explore the effects of oxytocin on eye movements in individuals with schizophrenia. We found that oxytocin increased exploratory viewing behaviour in response to affective facial stimuli by significantly increasing the total number and duration of fixations compared to placebo. While previous findings regarding oxytocin have been inconsistent, our findings are in line with research showing that the intranasal administration of 40UI oxytocin may improve social cognitive deficits in schizophrenia (e.g. Davis et al., 2013). Future experiments may wish to explore the correlation between eye movement changes induced by oxytocin and facial affect recognition in larger samples.

### F78. OVERCOMING A BOTTOM-UP ATTENTIONAL BIAS BY PROVIDING TOP-DOWN INFORMATION DURING WORKING MEMORY ENCODING IN SCHIZOPHRENIA

Catherine Barnes*[,1], Lara Rösler[2], Michael Schaum[1], Deliah Macht[1], Benjamin Peters[3], Jutta Mayer[1], Michael Wibral[1], Andreas Reif[1], Robert Bittner[1]
*[1]Goethe University Hospital Frankfurt; [2]University of Würzburg; [3]Institute for Medical Psychology, Goethe University Hospital Frankfurt*

**Background:** Cognitive impairments including deficits in working memory are commonly observed in schizophrenia. A bottom-up attentional bias has been suggested for encoding visually salient yet irrelevant information. To date it is not known if this bias persists when additional top-down information in the form of a predictive cue is provided. We were motivated to clarify this issue.
**Methods:** 40 patients with schizophrenia were measured and matched with 40 healthy control participants. During a change detection task four Gabor patches (two flickering and two non-flickering) with varying orientations were shown and participants had to memorize the orientations of the Gabor patches. A colored fixation cross was displayed before the stimuli either cueing two (predictive cue) or all four (non-predictive cue) Gabor patch locations resulting in a 2 x 2 design of four conditions with the factors salience (flickering vs. non-flickering) and cue (predictive cue vs. non-predictive cue). During retrieval a single Gabor patch was displayed, and participants reported if the orientation was the same or had changed in that location. At the beginning of each block participants were instructed to either encode the flickering or non-flickering patches (targets) whose location could either be cued or uncued. In 80 % of trials, a target was probed during retrieval.
**Results:** Patients encoded less information than healthy controls in all four conditions. Both healthy controls and patients encoded more visually salient information than non-salient information, and performance was near chance level during non-target trials. Patients encoded significantly more information when a predictive cue was provided before encoding visually non-salient information.
**Discussion:** Patients were able to overcome their bottom-up attentional bias of encoding visually salient irrelevant information when provided with top-down information. These findings are in line with previous reports of a bottom-up attentional bias during working memory encoding in schizophrenia. We propose that this bias can be overcome by providing additional top-down information.

### F79. ATTRIBUTION OF INTENTIONS IN PATIENTS WITH SCHIZOPHRENIA SPECTRUM DISORDERS WITH PERSECUTORY DELUSIONS

Michal Hajdúk*[,1], Lucia Pavelková[1], Peter Ohrablo[2], Veronika Petrušová[2], Anton Heretik[1], Ľubica Forgáčová[2]
*[1]Comenius University; [2]Slovak Medical University UHB*

**Background:** Social cognitive deficits are considered hallmark features of schizophrenia spectrum disorders. Consistent patterns of relationships have been established between theory of mind impairment and severity of negative symptoms. Some studies have suggested that patients, specifically those with persecutory delusion, can over attribute intentions. Difficulties in theory of mind in patients with schizophrenia can vary between hypo and hyper – mentalization depending on the level of symptoms. The aim of the study was to test model which proposed hypo -mentalization vs. hyper -mentalization deficit in patients with schizophrenia spectrum disorders with persecutory delusions.
**Methods:** 40 patients diagnosed with schizophrenia spectrum disorder, 19 patients with anxiety, affective and personality disorders without persecutory delusions, and 28 healthy controls were enrolled in the study. Diagnoses were established according to ICD-10 criteria. Animation Task was used for theory of mind assessment. Task consists of 12 videos (moving triangles) with three types of stimuli (random, goal-directed and theory of mind – condition). Stimuli were presented in fixed, random order before symptom assessment. Participants were asked to describe content of videos, and the degree of intentionality and appropriateness was evaluated by two raters according to task`s manual. Mutual rating of raters was used in the present analysis. Brief Psychiatric Rating Scale was used for assessment of symptoms severity.
**Results:** A repeated measures ANOVA with stimuli type as within-factor and group as between-factor revealed significant effect of Stimuli type (F= 171.585, p < .001), and interaction of factors (F = 5.401, p = .001) on rating of intentionality. Group effect was not significant (F= .836, p = .437). Patients with schizophrenia had significantly lower ratings of intentionality in theory of mind condition, specifically. A second repeated measures ANOVA analyzed differences in levels of appropriateness. Results revealed significant effect of stimuli type (F= 12.698, p < .001), group (F= 6.966, p = .002) and interaction of factors (F = 3.211, p = .020). Responses of patients with schizophrenia were less appropriate than controls in goal-directed and theory of mind condition compared to the random condition. Severity of negative symptoms was associate with lower level of intentionality in random condition. Hostility and suspiciousness were associated with higher level of intentionality in goal directed (rs=.330, p=.037) and theory of mind conditions (rs=.348, p=.028). Severity of suspiciousness was moderately to strongly associated with appropriateness of descriptions in all conditions (rs from -.423 to -.517).
**Discussion:** Results of study highlighted importance of distinguishing between hyper- and hypo-mentalization in patients with schizophrenia as specific impairments were associated with positive and negative symptoms, respectively. Over attribution of intentions to random movement was moderately associated with paranoid symptoms. Patients provided less appropriate descriptions which was associated with higher level of suspiciousness. Implications for development, maintenance treatment of persecutory delusions will be discussed.
Research was supported by Psychiatric Society of Slovak Medical Agency – grant no: 02/2015

### F80. COGNITIVE TRAJECTORIES OVER 6 YEARS IN FIRST-EPISODE SCHIZOPHRENIA AND HEALTHY CONTROLS – A PROSPECTIVE LONGITUDINAL MULTI-ASSESSMENT STUDY

Susie Fu*[,1], Nikolai Czajkowski[1], Anne-Kari Torgalsboen[1]
*[1]University of Oslo*

**Background:** Patients with first-episode schizophrenia (FES) have consistently showed impaired cognitive functioning compared to healthy controls across a broad array of cognitive domains. After psychosis onset the cognitive performance in FES seems to remain stable or even improve over time. Many earlier studies, however, did not include healthy control groups which made it unclear whether cognitive changes were due to genuine improvements or other arbitrary factors. Thus, the development of individual cognitive domains over time is not yet fully examined.
**Methods:** The present study has a multi-assessment design, and includes data from eight follow-ups over six years. For the patient group, assessments were conducted yearly, apart from the first year where assessments were conducted every six months. Healthy controls were assessed at baseline, after two years and after six years. A total of 28 FES-patients and 28 healthy controls participated in the study, with 79 % of patients retained at the 6-year follow-up. Cognition was assessed with MATRICS Consensus Cognitive Battery. Data were analyzed with linear multilevel models.

**Results:** FES-patients scored lower than the control group across all cognitive domains at baseline. Over six years, the cognitive trajectories of visual learning seem to remain stable for both groups, while FES-patients showed slight improvements in attention (β = 1.34, SE = .18, p < .001), verbal learning (β = .65, SE = .29, p < .031), processing speed (β = .69, SE = .35, p < .051), reasoning/ problem solving (β = 1.68, SE = .27 p < .001), working memory (β = .89, SE = .27, p < .002) and social cognition (β = .93, SE = .30, p < .003). Most of these cognitive trajectories start to improve within the first year of illness and continues throughout the six year period. The improvement in processing speed (β = .18, SE = .48, p > .05), verbal learning (β = .56, SE = .59, p > .05) and social cognition (β = .82, SE = .59, p > .05) seem to be larger for FES-patients compared to controls, but these differences were not significant. The patient group's improvement in reasoning/ problem solving (β = 1.31, SE = .51, p < .05) was significantly larger that the control group, but they showed smaller improvement in working memory (β = -1.03, SE = .51, p < .05).
**Discussion:** Our results show that improvements are already discernable after 6 months following illness outbreak. There are different trajectories for different cognitive domains. Moreover, two cognitive domain trajectories were significantly different between control group and FES-patients. This points to the importance of assessing cognitive development over many years with multiple assessments when exploring cognitive impairments in schizophrenia. From a clinical perspective, this may speak in favor of a targeted rehabilitation of different cognitive domains.

### F81. AGE OF ONSET OF CANNABIS USE AND COGNITIVE FUNCTION IN FIRST EPISODE NON-AFFECTIVE PSYCHOSIS PATIENTS: 3-YEAR FOLLOW-UP OUTCOME

Esther Setién-Suero*[,1], Diana Tordesillas-Gutierrez[2], Benedicto Crespo-Facorro[2], Rosa Ayesa-Arriola[2]
[1]*Centro de Investigación Biomedica en Red de Salud Mental (CIBERSAM);* [2]*University Hospital Marques de Valdecilla -IDIVAL, University of Cantabria, CIBERSAM*

**Background:** In recent years, the effect of cannabis use on cognitive functions in patients with psychosis has been widely studied, but results are somewhat contradictory. On the other hand, it has also been studied the relevance of the age of onset of consume, suggesting that the early age of onset of consumption may be related to a greater cognitive impairment.
**Methods:** 349 patients with a first episode of non-affective psychosis were studied. Patients were classified in cannabis users and non-users. Users were divided according to their age at the beginning of use of cannabis in: early-onset (age<16) and late-onset (≥16 years-old). Differences between groups at baseline were studied on sociodemographic, clinical and cognitive variables. The groups were longitudinally (3-year) compared on cognitive variables.
**Results:** Out of the 349 patients included in this study, 38.7% (N=135) were cannabis users, of them 39.3% (N=53) started consuming before 16 years of age and 60.7% (N=82) did so at age 16 of after. No differences were found between early-onset and late-onset groups on cognitive domains. However, cannabis users (early and late) showed significantly worse performance in processing speed than non-users. Longitudinal analises revealed that the groups of early-onset, late-onset and non-users of cannabis, had different evolution in processing speed domain and in the global cognitive functioning.
**Discussion:** The main findings of this study were that, although there were differences between patients who used cannabis and those who did not, minimal differences aroused between the early-onset and late-onset cannabis users. With respect to longitudinal analyses, we must be careful with their interpretation, since although a priori we found a significant group by time interaction (early-onset, late-onset, and non-users) in some domain, when the cannabis use at 3-year follow-up was considered, results did not show any significance, this reveals that cannabis users (early-onset and late-onset) and non-cannabis users did not differ in the degree of change in their cognitive functions, regardless of whether or not the patients had maintained consumption during the first 3-year of disease progression.

### F82. COGNITIVE BIASES IN PATIENTS WITH SCHIZOPHRENIA AND HIGH SCHOOL STUDENTS: ASSOCIATION WITH PSYCHOPATHOLOGICAL SYMPTOMS

Francesc Estrada*[,1], Maribel Ahuir[1], Josep Maria Crosas[1], Claudia Vilaplana[1], Alba González-Fernández[1], Meritxell Tost[1], Irina Olasz[1], Nora Mesa[1], Estefania Gago[1], Montserrat Pàmias[1], José Antonio Monreal[1], Diego Palao[1], Javier Labad[1]
[1]*Parc Taulí Sabadell*

**Background:** Some cognitive biases, mainly the "jumping to conclusions" and attributional styles, play a key role in the formation and maintenance of delusions. Other thinking errors include dichotomous thinking, emotionally based reasoning, and catastrophising. The aim of our study was to assess the relationship between cognitive biases and psychopathological symptoms (positive, negative, depressive) in a clinical sample of patients with schizophrenia and a population sample of high school students.
**Methods:** The clinical sample included 35 patients with schizophrenia (35.6 ± 10.8 years, 40% women) attending to the Department of Mental Health from Parc Taulí Hospital Universitari (Sabadell, Spain) and 45 high school students (16.6 ± 0.9 years) located in the same province.
Cognitive biases were assessed with the Cognitive Biases Questionnaire, that covers 5 types of biases (intentionalising [I]; catastrophizing [C]; dichotomous thinking [DT]; jumping to conclusions [JTC]; and emotional reasoning [ER]) and also gives a total score. Psychopathological symptoms in patients were assessed with the Positive and Negative Syndrome Scale (PANSS) and the Calgary Depression Scale for Schizophrenia (CDSS). Psychopathological symptoms in high school students were assessed with the Community Assessment of Psychic Experiences (CAPE).
Statistical analyses were performed with SPSS version 21.0. CBQ scores between groups were compared with Student's T-test. The association between dimensions of the CBQ and scores of psychopathological scales was tested with Spearman's correlations. Significance level was set at p<0.05 (two-tailed).
**Results:** CBQ total scores did not differed between patients with schizophrenia (45.3 ± 8.2) and high school students (44.2 ± 6.7). No significant differences between groups were found in any of the five cognitive biases.
When exploring the relationship between cognitive biases and psychopathological symptoms in patients with schizophrenia, total CBQ scores were associated with CDSS scores (r= 0.65, p<0.001). In relation to particular cognitive biases, depressive symptoms were associated with all cognitive biases (I: r= 0.43, p= 0.017; C: r= 0.62, p<0.001; DT: r= 0.42, p= 0.020; JTC: r= 0.46, p= 0.012; ER: r= 0.57, p= 0.001), positive symptoms with ER (r= 0.43, p= 0.009) and general psychopathology symptoms of the PANSS with C (r= 0.34, p= 0.044), DT (r= 0.35, p= 0.041) and ER (r= 0.45, p= 0.007).
In high school students, CBQ total scores were associated with positive (r= 0.43, p= 0.003) and depressive (r= 0.35, p= 0.020) symptoms. In relation to particular cognitive biases, depressive symptoms were associated with DT (r= 0.47, p= 0.001) whereas positive symptoms were associated with DT (r= 0.31, p= 0.036) and ER (r= 0.30, p= 0.047).
**Discussion:** Although we did not find significant differences in the presence of cognitive biases when comparing two different samples, similar associations were found when exploring the relationship between cognitive biases and psychopathology symptoms. Our results are in accordance previous studies reporting the role of some cognitive biases on the risk of developing psychotic symptoms. On the other hand, a clear association between cognitive biases was found for depressive symptoms in both patients with schizophrenia and high school students. Our study highlights the importance of identifying and treating cognitive biases with appropriate therapies (e.g. metacognitive training) for improving the outcome of psychoses in both patients and people at risk for developing a psychotic disorder in the future.

### F83. INVESTIGATING THE RELATIONSHIP BETWEEN NEGATIVE SYMPTOM PROFILE AND COGNITIVE FUNCTION IN SCHIZOPHRENIA

Caitlin Yolland*[,1], Wei Lin Toh[2], Eric Tan[3], Caroline Gurvich[3], Erica Neill[4], Susan Rossell[5]
*[1]Swinburne University of Technology; [2]Monash Alfred Psychiatry Research Centre; [3]Brain and Psychological Sciences Research Centre, Swinburne University, Monash Alfred Psychiatry Research Centre, The Alfred Hospital and Monash University; [5]University of Melbourne*

**Background:** Negative symptoms are core to schizophrenia. Understanding the complex way specific symptom profiles may affect cognition independent of a diagnosis of schizophrenia per se will allow for an improved understanding of the disorder, and specific subtypes as well as potential treatment targets therein.
**Methods:** The neurocognitive profiles of 132 patients with schizophrenia/schizoaffective disorder and 189 healthy controls were examined using the MATRICS Consensus Cognitive Battery. Patients were grouped as either having a negative symptom profile or no negative symptoms using the PANSS. Healthy controls were grouped as high or low schizotypy on the negative symptom analogue subscale from the O-LIFE.
**Results:** There was a significant effect of negative symptom profile on the processing speed domain, the participants with negative symptoms performed significantly worse than those with no negative symptoms, after controlling for premorbid IQ, $F(1,129)=4.30$, $p<0.05$. The same relationship with speed of processing was found when investigating high vs low schizotypal aspects of negative symptoms in an equivalent analysis of healthy controls, with those scoring highly on negative symptoms performing significantly worse, after premorbid IQ was controlled for, $F(1,186)=6.24$, $p<0.05$.
**Discussion:** The processing speed domain seems significantly impacted by negative symptom profile in both schizophrenia patients and healthy controls. The speed of processing deficits does not seem to be presenting a bottom up influence on higher order cognitive tasks, as no group differences were observed on reasoning and problem solving tasks. In conclusion, these findings indicate that the negative symptom cluster contributes to this specific cognitive impairment independently of the disorder.

### F84. ASSOCIATIONS BETWEEN INTELLIGENCE, VERBAL WORKING MEMORY AND PROCESSING SPEED IN PARENTS WITH SCHIZOPHRENIA OR BIPOLAR DISORDER AND THEIR 7-YEAR OLD OFFSPRING

Aja Greve*[,1], Jens Richardt Møllegaard Jepsen[2], Erik Lykke Mortensen[3], Rudolf Uher[4], Lynn Mackenzie[4], Leslie Foldager[5], Ditte Gantriis[1], Birgitte Klee Burton[6], Ditte Ellersgaard[7], Camilla Jerlang Christiani[7], Katrine Spang[7], Nicoline Hemager[7], Maria Toft Henriksen[1], Kate Kold Zahle[1], Henriette Stadsgaard[1], Anne Thorup[7], Merete Nordentoft[7], Kerstin Plessen[7], Ole Mors[1], Vibeke Bliksted[1]
*[1]Aarhus University Hospital; [2]Copenhagen University Hospital; [3]University of Copenhagen; [4]Dalhousie University, Halifax; [5]Aarhus University; [6]Child and Adolescent Mental Health Centre, Mental Health Services Capital Region; [7]Mental Health Centre Copenhagen, Copenhagen University Hospital, Mental Health Services Capital Region*

**Background:** Neurocognitive phenotypes may contribute to understanding the pathway leading from genes to psychopathology. We aimed to investigate associations of intelligence, processing speed and verbal working memory between parents and offspring in families with schizophrenia, bipolar disorder and controls.
**Methods:** Data are from The Danish High Risk and Resilience Study – VIA7, a population-based nationwide cohort identified through Danish Registries. Participants are 522 children aged 7 with no, one, or two parents with schizophrenia or bipolar disorder and biological parents. Control children were matched to children from the schizophrenia group (age, gender, and municipality). Children at familial risk of bipolar disorder were comparison group. Child assessors were blinded to risk status. Main Outcomes were intelligence measured with Reynolds Intellectual Screening Test (RIST), verbal working memory assessed with letter number sequencing (LNS) and processing speed assessed with Coding (WISC-IV/WAIS-IV).
**Results:** We examined 434 index parents (151 schizophrenia, 100 bipolar disorder and 183 controls, mean (SD) age 39.7 (5.7), 264 (61%) females), 443 co-parents (mean (SD) age 40.1 (5.4), 210 (47%) females) and 489 children (mean (SD) age 7.8 (0.2), 231 (47%) females). Children´s intelligence was associated with index parents' intelligence ($B = 0.40$, 95% CI: 0.28;0.52, $p < 0.001$) and co-parents' intelligence ($B = 0.16$, 95% CI: 0.03;0.28, $p = 0.012$). Children's processing speed was associated with index parents' processing speed ($B = 0.07$, 95% CI: 0.02;0.12, $p = 0.007$), co-parents' processing speed ($B = 0.09$, 95% CI: 0.04;0.15, $p < 0.001$), group (schizophrenia: $B = -1.92$, 95% CI: -3.63;-0.21, $p = 0.028$) and gender of child (male: $B = -4.55$, 95% CI: -4.98; -2.12, $p < 0.001$). Children's working memory was associated with index parents' LNS score ($B=0.25$, 95% CI: 0.13;0.37, $p < 0.001$), co-parents' working memory ($B = 0.23$, 95% CI: 0.09;0.37, $p = 0.001$), group (schizophrenia: $B = -1.02$, 95% CI: -0.89;-0.16, $p = 0.020$) and gender of child (male: $B = -0.85$, 95% CI: -1.60;-0.11, $p = 0.025$).
**Discussion:** Findings showed associations of neurocognitive phenotypes between parents and offspring. These associations do not differ markedly between schizophrenia, bipolar disorder and controls.

### F85. THE RELATIONSHIP BETWEEN JUMPING TO CONCLUSIONS AND NEUROPSYCHOLOGICAL FUNCTIONING IN SCHIZOPHRENIA

Martyna Krężołek*[,1], Renata Pionke[2], Beata Banaszak[3], Andrzej Kokoszka[1], Łukasz Gawęda[4]
*[1]Medical University of Warsaw; [2]Institute of Psychology, Polish Academy of Sciences; [3]Bielański Hospital; [4]Medical University of Warsaw, University Medical Center Hamburg Eppendorf*

**Background:** Patients with schizophrenia display a tendency to jump to conclusions (JTC), although the cognitive mechanisms of JTC remain unknown. The main aim of our study was to investigate the relationship between the subjective and objective measure of JTC and neuropsychological functioning in a sample of people with schizophrenia.
**Methods:** A total of 85 patients diagnosed (44 females, mean age 42.20, SD=11.42) with schizophrenia were assessed with neuropsychological tests, including executive functions, verbal memory, working memory, processing speed and attention. JTC was assessed with the Fish Task (probability 80:20 and 60:40) and a self-report scale (DACOBS). Symptom severity was assessed with the PANSS. The relationship between JTC and neuropsychological functioning was investigated with correlation and regression analyses.
**Results:** JTC measured by the 60:40 Fish Task version showed a greater number of moderate associations with neuropsychological variables as compared with the task's 80:20 version. Self-reported JTC turned out to be negatively correlated with CVLT and the Backward Digit Span. The regression analyses model, when controlling for duration of illness, age and symptoms, showed that neuropsychological variables associated with the tests, i.e. CVLT and Forward Digit Span, were specifically related to JTC measured by Fish Task 60:40. These variables turned out to be insignificant predictors of JTC 80:20 and JTC as the DACOBS subscale. JTC measured using Fish Task (60:40) was correlated only with disorganization.

**Discussion:** The results from the present study suggest that the relationship between decision making during the reasoning task and neuropsychological functioning is modulated by task demands. In particular, verbal working memory deficits are implicated in more hasty decision making when the task is demanding.

### F86. SOCIAL COGNITION IN HOMICIDE OFFENDERS WITH SCHIZOPHRENIA

Katharina Engelstad*[,1], Bjørn Rishovd Rund[2], Anne-Kari Torgalsboen[2], Anja Vaskinn[3]
*[1]Vestre Viken Hospital Trust; [2]University of Oslo; [3]Oslo University Hospital*

**Background:** Schizophrenia is characterized by impairments in social and non-social cognition that act as strong predictors of the poor outcome of the disorder, including violence. In fact, it has been suggested that the inclusion of (social) cognition in risk assessment tools may increase their accuracy. A premise for this line of thinking is that individuals with schizophrenia and a history of violent offences should present with a different social cognitive profile than individuals with schizophrenia without such a history. In this study, we compare social and non-social cognition in homicide offenders with schizophrenia (HOS) and in individuals with schizophrenia without a history of interpersonal violence (non-HOS).

**Methods:** Twenty-six HOS and 28 non-HOS were included. They underwent a comprehensive clinical and neuropsychological assessment protocol where the MATRICS Consensus Cognitive Battery (MCCB) and four tests of social cognition were applied. Facial emotion perception was assessed with Pictures of Facial Affect (PFA) where portrait photographs of people expressing one of six emotions are presented. Emotion perception from bodies was measured with Emotion in Biological Motion (EmoBio), a point-light task consisting of short movie clips where lighted dots, indicative of one of four emotions, move across the computer screen. Theory of mind (ToM) was indexed by two tests. The Hinting Task consists of ten short stories where a hint is dropped. The ecologically valid Movie for the Assessment of Social Cognition (MASC) shows four characters who meet for dinner. The movie is stopped several times, and the test taker is instructed to answer questions about the thoughts, intentions and emotions of a given character. This yields scores for cognitive and affective ToM. In addition, the multiple-choice response format provides information on overmentalizing and undermentalizing errors.

**Results:** Preliminary analyses, using independent samples t-tests, showed significant differences for the overall scores on the EmoBio and MASC tests, with non-HOS outperforming HOS participants. Follow-up analyses for the EmoBio test revealed no statistically significant differences for any specific emotion, although the group difference for the recognition of happy, fearful or neutral body movements approached a medium effect size. On the MASC test, HOS presented with reduced cognitive and affective ToM, compared to non-HOS, and also committed more undermentalizing errors. These effect sizes were large.

**Discussion:** Our preliminary analyses show that homicide offenders with schizophrenia have reduced emotion perception and theory of mind compared to individuals with schizophrenia without a history of interpersonal violence. This lends support to the idea that social cognitive deficits can be a risk factor for interpersonal violence in persons with a diagnosis of schizophrenia.

### F87. SERUM PROLACTIN LEVELS AND COGNITIVE OUTCOME IN FIRST EPISODE PSYCHOSIS: A PROSPECTIVE 1-YEAR FOLLOW-UP STUDY

Meritxell Tost Bonet*[,1], Itziar Montalvo[1], Francesc Estrada[1], Maria Isabel Ahuir Perez[1], Ángel Cabezas[2], Montse Paños[1], Montserrat Sole[2], José Antonio Monreal[1], vanessa Sanchez-gistau[2], Elisabet Vilella[2], Diego Palao[1], Javier Labad[1]
*[1]Parc Taulí Hospital Universitari, I3PT, Universitat Autònoma de Barcelona; [2]Early Intervention Service, Hospital Universitari Institut Pere Mata, IISPV, Universitat Rovira i Virgili*

**Background:** Recent studies in patients with psychotic disorders or prolactinomas suggest that increased prolactin levels may have negative effects on cognition. Most previous studies including patients with psychotic disorders are cross-sectional, and longitudinal studies are lacking. We aimed to conduct an observational, prospective study to explore whether prolactin levels during the first year of treatment are associated with changes in cognitive tasks. Our hypothesis would be that those patients with increased prolactin levels would show a poorer cognitive outcome than those patients with lower prolactin levels.

**Methods:** We studied 60 patients (24.5 ± 6.8 years; 36% women) with a first episode psychosis (FEP) attending the Early Psychosis Programme from two institutions (Parc Taulí Hospital Universitari, Sabadell, Spain; Hospital Universitari Institut Pere Mata). Ethical approval was obtained from the local Ethics Committees of both institutions. Clinical diagnoses for a FEP were generated with the OPCRIT checklist v.4.0 after a semistructured interview by a psychiatrist. The MATRICS Consensus Cognitive Battery (MCCB) was administered to explore neuropsychological functioning at two-time points (baseline, 1 year). The MCCB contains 10 tests to measure cognitive performance in 7 cognitive domains. Three fasting blood samples (baseline, 6 months, 12 months) were obtained in the morning between 8:30 h and 9:30 h in resting conditions, to determine unstimulated plasma prolactin. Serum prolactin levels were determined with immunoassays standardized against the 3rd International Reference Standard 84/500. Statistical analyses were performed with SPSS version 21.0. As prolactin levels might be increased by stress, particularly at the onset of the FEP, we calculated mean prolactin levels over the follow-up period taking into account prolactin values at 6 and 12 months. A general linear model for repeated measures was performed in order to test whether longitudinal changes in cognitive tasks differed by mean prolactin levels. All analyses were adjusted for gender. A p value $< 0.05$ (two-tailed) was considered to be significant. For descriptive purposes, patients in the fourth quartile for serum prolactin (>47.8 ng/ml for men; >54.3 ng/ml for women) where compared with those with lower prolactin levels.

**Results:** Patients improved in all 10 MCCB cognitive tasks one year later ($p<0.05$ for all tasks). When exploring the interaction between time x prolactin in the GLM analyses, significant interactions were found for three cognitive tasks related with processing speed (BACS-SC [F= 5.9, p= 0.018]; Fluency [F= 5.6, p= 0.022]) and reasoning and problem solving (NAB mazes [F= 4.8, p= 0.033]). Percent change over the follow-up period in these cognitive tasks was greater for patients with lower prolactin levels, when compared to those in the highest quartile: BACS-SC (11.8% vs 0.6%), Fluency (8.5% vs -7.4%) and NAB mazes (10.7% vs -1.1%).

**Discussion:** Our study is in accordance with previous cross-sectional studies reporting a negative effect of prolactin levels on cognition and adds new information with a prospective design. A limitation of our study is that patients were treated with different antipsychotics based on the clinical routine practice. Antipsychotic-induced hyperprolactinaemia, which is caused by tuberoinfundibular blockade of D2 receptors, may be reflecting the blockade of D2 receptors in other dopaminergic pathways (striatum and mesocortical pathways) that may also affect cognitive abilities.

Further clinical trials are needed to reduce the heterogeneity of the treatment effects and to confirm the potential negative effects of prolactin on cognitive abilities.

### F88. MANIPULATION OF THE GUT MICROBIOTA WITH A PREBIOTIC IN SCHIZOPHRENIA: A DOUBLE-BLINDED RANDOMIZED PLACEBO-CONTROLLED CROSS-OVER STUDY

Amy Kao*[,1], Phil Burnet[1], Belinda Lennox[1]
[1]*University of Oxford*

**Background:** Neurocognitive impairment is increasingly recognized as a fundamental symptomatology in schizophrenia with more than 80% of patients exhibiting significant deficits, even at first episode of illness. Current pharmacotherapies do not alleviate cognitive symptoms, and often cause severe metabolic dysfunction and weight gain that readily become major health concerns. Identifying adjunctive interventions that improve cognition without disrupting the therapeutic actions of anti-psychotic medications, and can mitigate the metabolic side-effects of these drugs, would be highly beneficial to patients and improve prognosis. We have recently demonstrated that the manipulation of the rat gut microbiota with a prebiotic (dietary fibre that grows beneficial enteric bacteria) improves cognitive flexibility and prevents olanzapine-mediated weight gain. We therefore aim to explore whether these actions of the prebiotic translate to medicated stable patients with schizophrenia.
**Methods:** A total of 40 patients with psychosis aged 18–65 will be enrolled in a 24-week maltodextrin-controlled cross-over experimental medicine study. Participants will receive either a 12-week treatment with a prebiotic (active compound) followed by a 12-week maltodextrin supplement (placebo), or in the reverse order. The order of supplements that participants receive is randomized. The primary outcome is to examine the influence of prebiotic supplementation on neurocognitive functioning, which is measured using a tablet-based neuropsychometric test battery. We will also examine the impact on clinical metabolic measures such as total weight and visceral adiposity. The concentration of immune-related serum proteins as well as neuroendocrine hormones will be evaluated. All measurements will take place at baseline, at 12-week cross-over, and at the end of the 24-week study. A within-subjects repeated measures analysis will be performed, and co-variates (gender, weight, medication) identified. This trial is registered with ClinicalTrials.gov, identifier number NCT03153046.
**Results:** We have currently screened 36 patients, of whom 30 were eligible for the study (67% male). At baseline, the average age of all recruited was 36.41 ± 11.42. The overall cognitive score was -2.03 ± 0.53 where the subtests included verbal memory (29.09 ± 8.75), digit sequencing (15.41 ± 2.77), token motor (55.74 ± 24.14), semantic fluency (8.83 ± 3.46), letter fluency (11.39 ± 3.65), symbol coding (35.61 ± 9.22) and tower of London (15.1 ± 4.06). There was no difference in overall cognitive between male (14.29 ± 2.46) and female (17.22 ± 2.30) patients. However, long-term associative learning as measured by the digit sequencing subtest appeared to show a significant difference between male (14.29 ± 2.46) and female (17.22 ± 2.33; p=0.008) participants. No significant differences between clinical metabolic measures were observed in baseline BMI (32.25 ± 6.79) and abdominal obesity as measured by hip-to-waist ratio (0.94 ± 0.11). The serum concentrations of immune and endocrine markers will also be presented.
**Discussion:** This investigation, to our knowledge, is the first clinical study to provide medicated schizophrenia patients with a prebiotic, as a potential means of improving cognition and managing secondary metabolic dysfunction. Although a potential link between commensal enteric bacteria and schizophrenia have been suggested, earlier work with probiotics (live cultures) did not support this association. However, since the current study uses a prebiotic that proliferates multiple species of gut bacteria, it will provide more robust data that will support, or refute, the validity of manipulating the gut microbiome in the treatment of schizophrenia.

### F89. COGNITIVE IMPAIRMENT WORSENING ACROSS AFFECTIVE TO PSYCHOSIS SPECTRUM: A COHORT STUDY OF UNIPOLAR/BIPOLAR DEPRESSION AND BIPOLAR SCHIZOAFFECTIVE DISORDERS

Martin Roy*[,1], Marie-Pierre Strippoli[2], Caroline Vandeleur[2], Elsa Gilbert[3], Enrique Castelao[2], Jean-Michel Aubry[4], Pierre Marquet[3], Martin Preisig[2]
[1]*CERVO Brain Research Centre;* [2]*University Hospital of Lausanne;* [3]*Université Laval, CERVO Brain Research Centre;* [4]*University Hospital of Geneva*

**Background:** Bipolar (BPD), schizoaffective bipolar (SAM) and major depressive disorders (MDD) reveal large heterogeneity in terms of symptom expression, course and treatment response. This heterogeneity could be the source of a large variance of cognitive performance observed in these subjects. The aim of the present analyses was to compare the cognitive performance of patients with BPD, SAM, MDD and medical controls with adjustment for a comprehensive array of potential confounders. To go a step further we will simultaneously test the effects of multiple clinical characteristics including lifetime history and duration of psychotic symptoms, manic/hypomanic and depressive episodes, age of onset of disorder, current GAF score, time since remission of the last episode and presence of a depressive episode at the time of the assessment on the cognitive performance.
**Methods:** Data stemmed from the Lausanne-Geneva Family and High-Risk study. Patients with BPD (n=62), SAM (n=22) and MDD (n=51) were interviewed every three years over a mean duration of follow-up of 12 years. All patients were assessed clinically with the semi-structured Diagnostic Interview for Genetic Studies (DIGS). The cognitive assessment was made with the MATRICS and the Victoria Stroop Test.
**Results:** The global cognitive index (excluding Stroop result) shows that SAM subgroup had the lowest global score with 40.6 (SD=8.5), BPD 47.4 (SD=7.8) and MDD 49.7 (SD=8.7). A multiple linear regression accounting for several confounders such as comorbid psychiatric disorders and medication confirms that only SAM and BPD are statistically different from controls (p<0.001 and p<0.01 respectively). MDD did not differ from controls (p>0.05). Overall, patients with BPD or SAM but not with MDD showed poorer cognitive performance than controls in terms of the global score and speed of processing, verbal learning, working memory, visual learning, attention/vigilance and inhibition.
**Discussion:** Our data confirm cognitive impairment in patients with BPD or SAM compared to controls after adjustment for a comprehensive array of potential confounder variables. We were able to evaluate the specificity of cognitive performance of psychotic, maniac and depressive dimensions of the major mood disorders within the same sample. Furthermore, these data stress that the presence of the "schizo dimension" concomitant to mania and depression contributes to worsening the cognitive performance in an additive manner.

### F90. SOCIAL INFERENCE AND BELIEFS DIFFER IN INDIVIDUALS WITH SUBCLINICAL PERSECUTORY DELUSIONAL TENDENCIES

Katharina Wellstein*[,1], Andreea Diaconescu[2], Martin Bischof[3], Annia Rüesch Ranganadan[2], Johannes Ullrich[4], Klaas Enno Stephan[1]
[1]*ETH Zurich;* [2]*University of Zurich, ETH Zurich;* [3]*Psychiatric University Hospital Zurich;* [4]*University of Zurich*

**Background:** It has long been suspected that abnormalities in social inference (e.g., about the intentions of others) play a key role for persecutory delusions. In this study, we examined the association between subclinical persecutory delusions (PD) and social inference, testing the prediction that proneness to PD is related to altered social inference and beliefs.
**Methods:** We included 148 participants who scored on opposite ends of Freeman's Paranoia Checklist (PCL). High scorers and low scorers were thus assigned to two respective participant groups, which were matched according to age, education in years, and gender. Participants performed a probabilistic advice-taking task with a dynamically changing social context (volatility) under one of two experimental frames. Our design was thus 2x2 factorial (high vs. low delusional tendencies, dispositional vs. situational frame). In the task, participants had to integrate two types of cues simultaneously in order to make informed predictions, namely a social cue (advice provided by an adviser) and a non-social cue (probabilities given via pie-chart). In addition, the experimental frames differentially emphasized possible reasons behind unhelpful advice and either highlighted (i) the adviser's possible intentions (dispositional frame) or (ii) the rules of the game (situational frame). Task structure was identical across frames. When integrating the framing information, participants were expected to take advice into account more in the situational frame than in the dispositional frame, since the latter induces some mistrust due to highlighting the adviser's intentions.
**Results:** The behavioral data showed significant group-by-frame interactions (F=5.7381, p<0.05), indicating that in the situational frame high PCL scorers took advice less into account than low scorers. This reduced adaptation to the frame was particularly visible after the experience of volatility. Additionally, high PCL scorers believed significantly more frequently that incorrect advice was delivered intentionally (F=16.369, p<0.001) and that such malevolent behavior was directed towards them personally (p<0.05). High scorers also reported attributing unhelpful advice more to the adviser (F=8.047, p<0.01) instead of the rules of the game, compared to low scorers. The high scorers in the PCL reported higher negative, positive, and depressive symptoms on the CAPE compared to low scorers (p<0.001) but did not differ regarding cognitive performance in the Brief Neurocognitive Assessment (BNA).
**Discussion:** Overall, our results suggest that social inference in individuals with subclinical PD tendencies is less sensitive to differences in social context and shaped by negative beliefs about the intentions of others. These findings may help future attempts of identifying at risk mental state individuals and understanding maladaptive behavior in schizophrenia.

### F91. ASSOCIATION BETWEEN SYMPTOM DIMENSIONS AND EXECUTIVE FUNCTION IN SCHIZOPHRENIA

Ana Luiza Franco*[,1], Bruno Bertolucci Ortiz[1], Felipe Ferreira[2], Ana Olívia Fonseca[1], Arthur de Almeida Berberian[1], Deyvis Rocha[1], Cristiano Noto[1], Rodrigo Bressan[1], Ary Gadelha[1]
*[1]Universidade Federal de São Paulo (UNIFESP)*

**Background:** Impaired executive function is a core cognitive deficit in schizophrenia and strongly associated with functional outcomes. Understand the relationship between clinical symptoms and executive function may help the clinician to better manage the cognitive impairment and inform prognosis. The main objective of the present study was to investigate the association between symptom dimensions and executive function in schizophrenia.
**Methods:** One-hundred and two patients with schizophrenia were recruited from the schizophrenia outpatient clinic from Universidade Federal de São Paulo (PROESQ/UNIFESP). Diagnosis was confirmed through the Structured Clinical Interview for DSM-IV (SCID-I) and dimensional psychopathology was assessed by the Positive and Negative Syndrome Scale (PANSS). The PANSS items were grouped in five factors: positive, negative, disorganized/cognitive, mood/depression and excitement/hostility factors. The cognitive battery included the following tests: Plus–Minus Task, Number–Letter Task, Trail Making Test - Part B, Keep Track Task, Letter Memory Task, Visual Working Memory Test – MTV, Stroop Test, Semantic Generation Task and The Tower of London Test – TOL. All tasks were computerized and assessed by the software Cronos. A single latent variable for executive function was derived through Confirmatory factor analysis and yield good model fits (CFI: 0.997; TLI: 0.996; RMSEA: 0.017; SRMR: 0.041).
**Results:** When the factors were entered individually, negative (df=121, r=0,35, p <0.001) and disorganized (df= 121; r=-0.48, p <0.001) factors were significant predictors of EF. In a multivariate regression analysis, including all the factors and correcting for age, gender and duration of illness, only the disorganized factor remained significant ($r^2$=0,21,p<0.001).
**Discussion:** The disorganized factor was the symptomatic dimension more strongly associated with EF. The potential use of disorganized dimension as indicator of poor executive function and related outcomes, i.e., treatment resistant schizophrenia, should be further investigated.

### F92. COMPARISONS BETWEEN CANNABIS USERS AND NON-USERS PATIENTS WITH FIRST-EPISODE PSYCHOSIS IN NEUROCOGNITIVE FUNCTIONING: A META-ANALYSIS

Teresa Sanchez-Gutierrez*[,1], Belén Fernandez-Castilla[2], Sara Barbeito[1], Juan Antonio Becerra[1], Ana Calvo[1]
*[1]Universidad Internacioal de La Rioja (UNIR); [2]KU Leuven, University of Leuven*

**Background:** Patients with first episode psychosis (FEP) frequently report cannabis use although its effects on cognitive functioning are still unclear. Several studies suggest a decrease in the executive function, verbal memory and working memory of FEP cannabis users (González-Pinto et al., 2016; Mata et al. 2008) while other studies show improvements in the neurocognitive function of this group (Setién-Suero et al., 2017, Cuhna et al., 2013, Leeson et al., 2012, Yücel et al., 2012; Rodríguez-Sánchez et al., 2010) or even absence of neurocognitive differences between FEP cannabis users and non-users (Burgra et al., 2013). This meta-analysis aims to explore the magnitude of effect of cannabis use on neurocognition in patients with FEP.
**Methods:** Articles for consideration were identified through extensive literature searches using online databases, which included PubMed, Medline and PsychInfo. The search was limited to English language articles. The used keywords were: "first episode psychosis" OR, "neurocognition and cannabis", in combination with a number of neuropsychology-related terms including "neurocog*" and "neuropsycholog*". Given that other substances including alcohol, cocaine, and stimulants are associated with altered cognitive performance, studies in which participants met for poly-substance use disorders, even if there was preferential use towards cannabis, were excluded. Eight studies from 2008 to 2017 met inclusion criteria from a total sample of 16 initial studies. Five hundred and eighteen of these participants were cannabis users with FEP, and 639 were patients with no cannabis use. A total of 58 effect sizes of neuropsychological test variables were categorized into 4 cognitive domains (premorbid IQ, executive functioning, working memory and verbal memory and learning). Age of first cannabis use, duration of cannabis use, percentage of males and age were abstracted and assembled as moderator variables. Standardized mean differences were computed for each cognitive domain between cannabis-using patients and patients with no history of cannabis use. Negative effect sizes would display better cognitive functioning of non-cannabis users. We employed a meta-analytic three level model to combine effect sizes across studies.
**Results:** Effect sizes were not significantly different from zero in any of the neurocognitive domains when FEP cannabis users and non-users patients were compared [working memory (d= -0.03, SE=0.15, CI = -0.33–0.26, p=0.83), executive function (d= 0,14, SE=0.16, CI = -0.17–0.45, p=0.37), verbal memory and learning (d= 0.04, SE=0.15, CI = -0.25–0.33, p=0.27) and premorbid IQ (d= 0.06, SE=0.09, CI = -0.24–0.12, p=0.50)]. Only one moderator variable resulted significant in the executive function denoting superior performance in FEP cannabis-using patients as they were older.

**Discussion:** Cannabis use is not related to an ameliorated or improved neurocognitive functioning in patients with a first episode psychosis. This is consistent with previous studies which showed absence of differences in the neurocognitive functioning between FEP cannabis users and non-users (Burgra et al., 2013). However, it has been demonstrated that continued cannabis intake worsens cognitive performance although some of the FEP patients had better premorbid capacities (González-Pinto, 2016). Moreover, the doses and the different types of cannabis preparations may interfere the present results. Meta-analysis on longitudinal studies which include these potential moderator variables may be performed in the future.

### F93. SUBCLINICAL PSYCHOSIS COMPONENTS MAKE DIRECT AND INDIRECT CONTRIBUTIONS TO ACTIVE SUICIDE IDEATION IN ADOLESCENTS

Richard Linscott*[,1], Theresa Parker[1]
[1]*University of Otago*

**Background:** Subclinical psychosis predicts concurrent and future suicidal ideation and attempts. A key account of this relationship is that it is spurious—that suicidality and subclinical psychosis are both products of a common confounding factor such as environmental risk exposures (e.g., neglect or abuse) or the burden of general psychopathology. This account is unsatisfactory for several reasons, including that subclinical psychosis may be especially predictive of more lethal forms of suicidal behaviour. Moreover, few have considered the relationship in light of contemporary accounts of suicide. Therefore, we sought to better understand the link between subclinical psychosis and suicidality using a contemporary ideation–action framework in which perceived burden and thwarted belonging are distinguished as proximal pathways to suicidal ideation. We tested whether this framework fully mediates the relationship of subclinical psychosis with suicidal ideation, consistent with a common confounding factor account.
**Methods:** Randomly sampled 15- to 18-year-olds from a socio-economically representative high school were invited to participate anonymously. Of those invited (n = 300), 59% provided informed consent and completed self-report measures of positive, negative, and disorganised components of subclinical psychosis (Schizotypal Personality Questionnaire [SPQ]), thwarted belonging and perceived burden (Interpersonal Needs Questionnaire), and passive and active suicidal ideation (Beck Scale for Suicide Ideation [BSS]). Participants were classified using BSS responses as non-ideators, passive ideators, or active ideators. In regression modelling (maximum likelihood estimation with bias-corrected bootstrapping), direct and indirect effects of SPQ components on ideator classifications were obtained. Mediators were perceived burden, thwarted belonging, and their interaction term. Sex and migrant status were entered as covariates.
**Results:** Of those with complete data (n = 156), 69.9% were non-ideators, 12.8% were passive ideators, and 17.3% were active ideators. In bivariate analyses, SPQ positive scores predicted passive ideation ($r = .24$, $p < .001$) but negative ($r = .13$, $p > .05$) and disorganised scores ($r = .14$, $p > .05$) did not. In contrast, active ideation was strongly predicted by negative ($r = .39$, $p < .001$) and disorganised scores ($r = .34$, $p < .001$) and less strongly predicted by positive scores ($r = .19$, $p < .05$). Mediation models predicted passive (R2 = .29, $p < .05$) and active ideation (R2 = .65, $p < .001$). Passive ideation was sensitive only to indirect effects of SPQ scores: negative ($\beta = .14$, $p < .01$) and disorganised SPQ scores ($\beta = .11$, $p < .05$) were mediated by perceived burden. For active ideation, negative ($\beta = .17$, $p < .05$) and disorganised scores ($\beta = .14$, $p < .05$) had similar indirect effects mediated by perceived burden but there were also direct effects of positive ($\beta = -.44$, $p < .01$) and negative SPQ scores ($\beta = .37$, $p < .05$). Thwarted belonging did not mediate the effects of SPQ scores on ideator status.
**Discussion:** A contemporary ideation–action model of suicide did not fully account for the relationship between subclinical psychosis and suicidal ideation. Instead, some components of subclinical psychosis directly influenced suicidal ideation status: Positive subclinical psychosis components protected against active suicidal ideation whereas negative components increased the risk of active ideation. Negative and disorganised components of subclinical psychosis also increased the risk of ideation by increasing the perception of self-hate and liability on others. Subclinical psychosis makes a unique contribution to the prediction of suicidal ideation.

### F94. A PREVALENCE PILOT STUDY FOR OBSTRUCTIVE SLEEP APNEA SYNDROME IN CLOZAPINE USERS

M.F. Knevel[1], L.B. Luteijn*[,2], Jan Bogers[3], Jean-Paul Selten[4]
[1]*GGZ Rivierduinen;* [2]*GGZ Midden Holland;* [3]*Rivierduinen Centre for Mental Health, Dutch Clozapine Collaboration Group;* [4]*Maastricht University, Rivierduinen Mental Health Care Institute*

**Background:** Obstructive Sleep Apnea Syndrome (OSAS) is a frequent and common disorder. Estimated 50.000 persons in the Netherlands suffer from this disorder. Clozapine is known for its efficacy in treatment resistant schizophrenia. Frequent side effects of clozapine are weight gain, fatigue, sleepiness and metabolic syndrome. Similar symptoms occur in the course a OSAS. The Dutch pharmacovigilance centre LAREB (LAREB 2012) proposed an association between OSAS and clozapine usage, independent of confounding factors as obesity, smoking and glucose intolerance. Although clozapine is much used in the treatment of schizophrenia, OSAS prevalence studies in the clozapine treatment group are scarce. Research is needed to elucidate the relationship between clozapine use and OSAS. Identifying OSAS and treatment with continuous positive airway pressure (CPAP) could possibly (Galletly te al, 2016), through reduction in cardiovascular risk factors, have a favorable effect on mortality and possibly have a positive effect on daytime sleepiness, fatigue and daytime functioning.
Primary goal of this study is discovering the prevalence of OSAS in clozapine using schizophrenic spectrum disorder patients. The secondary goal is discovering how willing schizophrenic spectrum disorder patient are in undergoing a polysomnography.
**Hypothesis:** Many patients with schizophrenia spectrum disorder have multiple OSAS risk factors: obesity, presence of metabolic syndrome, frequent usage of benzodiazepines, male sex, older age. OSAS prevalence is estimated to be much higher than in the general population because of these risk factors. Atypical antipsychotics are an independent risk factor for the development of OSAS. Polysomnographically diagnosed OSAS will even be higher in the clozapine treatment group estimated to be present in 30% percent of the patients.
**Methods:** Research design: prospective observational and cross-sectional study in a group of stable adult patients with DSM IV schizophrenia spectrum disorder treated with clozapine in an outpatient community mental health service. Estimated study group consists of 30–50 patients. Exclusion criteria: unwillingness to undergo a polysomnography, inability to give informed consent, insufficient understanding of the Dutch language, severe cardiac failure, a history a cerebrovascular accidents and alcohol abuse.
Method: screening on the presence of OSAS symptoms and risk factors associated with OSAS through: Epworth Sleepiness Scale for daytime sleepiness (Johns, 1991), STOP-BANG Questionaire ((SBQ: Chung 2012) when there is a high risk for OSAS followed by an ambulatory polysomnography including heart rate/ECG, respiratory measures with nasal flow canule and thermistor flow inductive respiratory movements, oximetry, and snoaring noises through sensory measurements (AASAM, 2009). OSAS is considered to be present in the presence of daytime sleepiness and if the Apnea Hypopnea Index (AHI) is larger than 5.0 obstructive or mixed type respiratory events per hour (AASM, 2009: Berry et. al, 2015: NVALT & CBO, 2009).
Statistical analysis: polysomnography: descriptive and univariate analysis. Presence of OSAS will be dichomotized (1 =OSAS present; 0 = OSAS absent) Summation of the amount of positive results will be presented as percentage of the total study population. Chi- squared test for considering of the height of the results on the ESS test and the STOP-Bang test and the prevalence of OSAS. Statistical significance: $p < 0.05$.

Prevalence: percentage of patients in the clozapine treatment group with OSAS.
**Results:** No results at the moment of poster submission. In April 2018 results will be presented.
**Discussion:** Will be presented in April 2018.

### F95. ASSESSING MANIC SYMPTOMS IN PATIENTS WITH SCHIZOPHRENIA USING THE YOUNG MANIA RATING SCALE

Tae Yong Kim*[,1], Seon-Cheol Park[2], Joonho Choi[3], Han-Yong Jung[4], Joo Eon Park[5]
*[1]VHS Medical Center; [2]Inje University College of Medicine and Haeundae Paik Hospital; [3]College of Medicine, Hanyang University; [4]Soonchunhyang University Bucheon Hospital[5]Keyo Hospital*

**Background:** Van Os and Kapur have proposed that the discrete categorical dichotomy of schizophrenia versus bipolar disorder be changed to a dimensional conceptualization. It is also known that manic symptoms can contribute to the clinical course and prognosis of schizophrenia. Hence, a domain for mania has been included in the Clinician-Rated Dimensions of Psychosis Symptom Severity (CRDPSS) in the Diagnostic and Statistical Manual of Mental Disorders, 5th edition (DSM-5). However, the psychometric properties of the Young Mania Rating Scale (YMRS) have been little studied in subjects with schizophrenia.
**Methods:** One hundred and sixty-six inpatients with schizophrenia (diagnosed with DSM-5,2 age ≥ 18 years and ≤ 65 years, and length of hospital stay ≥ 2 weeks) were enrolled from two mental hospitals in Korea. The Institutional Review Board approved the study protocol, and informed consent was given by all study subjects before the start of the study. The Korean version of the YMRS was used to evaluate the severity of manic symptoms. In addition, the domain for mania in the CRDPSS was used to evaluate presence or absence of manic symptoms (0–1, absence; 2–4, presence).
**Results:** The average age and age-at-onset of the subjects were 46.5 (SD = 11.2) and 25.2 (SD = 13.2) years, respectively. Half were men (51.5%), and most were unmarried (79.1%), religiously affiliated (61.5%) and educated below high school graduate level (73.0%). The mean chlorpromazine equivalent dose of prescribed antipsychotics was 921.1 (SD = 952.0) mg. The mean total score on the YMRS was 7.3 (SD = 6.9) and the mean item scores were: 0.2 (SD = 0.4) for elevated mood, 0.1 (SD = 0.4) for increased motor activity, 0.1 (SD = 0.4) for sexual interest, 0.1 (SD = 0.4) for sleep, 0.4 (SD = 0.8) for irritability, 0.6 (SD = 1.2) for speech, 0.8 (SD = 1.1) for language, 2.0 (SD = 3.3) for content, 0.2 (SD = 0.7) for aggressive behavior, 1.0 (SD = 1.0) for appearance, and 1.8 (SD = 1.7) for insight. The Cronbach α for the 11 YMRS items was 0.66, which is considered an acceptable level of internal consistency. Moreover, only 4% (n = 7) of the 166 subjects had manic symptoms as assessed by the mania domain in the CRDPSS. A receiver operating characteristic curve (ROC) showed that the optimal cut-off score distinguishing schizophrenia patients with and without manic symptoms was 10 with a sensitivity of 88.3% and specificity of 75.6% (area under curve = 0.803, P = 0.012).
**Discussion:** Since a 10 point total score on the YMRS represents a mild level of Clinical Global Impression (CGI) severity of mania, we may conclude that our threshold on the YMRS for identifying manic symptoms in patients with schizophrenia is reasonable. Hence it may be useful to investigate the evaluation of manic symptoms in patients with schizophrenia from the perspective of deconstructing psychoses.

### F96. AGE AND GENDER DETERMINED DIFFERENCES IN THE ONSET OF CHRONIC PHYSICAL MULTIMORBIDITIES AMONG PATIENTS WITH SCHIZOPHRENIA OR DEPRESSION AND THE GENERAL POPULATION

Ivona Šimunović Filipčić*[,1], Ena Ivezić[2], Željko Milovac[2], Ines Kašpar[2], Sandra Kocijan Lovko[2], Lada Goršić[2], Tomislav Gajšak[2], Strahimir Sučić[2], Antonija Slaviček Sučić[2], Majda Grah[2], Nikolina Tunjić Vukadinović[2], Žarko Bajić[2], Marijana Braš[1], Igor Filipčić[2]
*[1]University Hospital Center Zagreb; [2]Psychiatric Hospital "Sveti Ivan"*

**Background:** The links between schizophrenia (SCH) or major depressive disorder (MDD) and chronic physical multimorbidities (CPM) are well established. Patients diagnosed with these disorders have a higher prevalence of CPM than the general population (GEP). However, our knowledge of age and gender determined differences in the development of CPM between SCH, MDD, and GEP remains fragmented and inconsistent. This exploratory study intended to compare the onset of CPM in female and male SCH and MDD patients, and the general population (GEP).
**Methods:** This nested, single-centered, cross-sectional study was performed during 2016 at Psychiatric hospital Sveti Ivan, Zagreb-Croatia. Data were collected for a consecutive sample of 136 patients diagnosed with SCH, 290 diagnosed with MDD, and 861 participants from the general population of the city of Zagreb and Zagreb County. The primary outcome was the prevalence of CPM. The secondary outcome was the prevalence of CPM in the youngest age group ≤35 years.
**Results:** After adjustment for gender and education, the prevalence of CPM was significantly different between patients with SCH or MDD and GEP ($p<0.001$). In the oldest age group (≥65 years) the difference was not significant anymore. In the youngest age group, the prevalence was highest in SCH patients (33%) followed by MDD (26%) and GEP (15%) indicating the early onset of CPM in severe mental illness. In the male participants <35 years old, there were no significant differences in the prevalence of CPM between SCH (25%), MDD (23%) and GEP (15%) ($p=0.411$). However, in the female participants <35 years old the difference was significant and clinically relevant ($p=0.006$). Prevalence of CPM in female participants was 50% in SCH, 33% in MDD and 14% in GEP.
**Discussion:** This study finding indicated the earlier onset of CPM in SCH and MDD patients than in GEP. This difference is primarily caused by the high prevalence of CPM in young female patients diagnosed with SCH. More prevalent physical morbidity points to a substantial disadvantage of female SCH patients early in the course of the illness. Understanding the nature and biological basis regarding the risk and outcome of CPM might help to identify new therapeutic targets, allow more individualized treatment, and facilitate better risk prediction and application of healthcare resources.

### F97. CHRONIC PHYSICAL MULTIMORBIDITIES, GENDER DISPARITIES AND TREATMENT OUTCOME IN SCHIZOPHRENIA

Igor Filipčić*[,1], Ivona Šimunović Filipčić[2], Ena Ivezić[1], Sanja Devčić[1], Davor Bodor[1], Branka Restek Petrović[1], Ivana Bakija[1], Paola Presečki[1], Silvana Jelavić[1], Nino Mimica[1], Katarina Matić[1], Dina Librenjak[1], Nenad Jakšić[2], Žarko Bajić[1]
*[1]Psychiatric Hospital Sveti Ivan; [2]University Hospital Center Zagreb*

**Background:** Increased physical morbidity in patients with schizophrenia (SCH) is well established. However, our knowledge on the role of gender in chronic physical multimorbidities (CPM) remains limited, and the evidence about the effect of CPM on SCH treatment outcome is sparse. The present study explored the gender-dependent differences in the prevalence, and age of onset of CPM between SCH and the general population (GEP), as well as the effect of CPM on hospital readmission in patients with SCH.

**Methods:** This cross-sectional study was nested within the larger frame of a prospective cohort study conducted at Psychiatric Hospital "Sveti Ivan", Croatia. Data were collected for a consecutive sample of 136 (49 female and 87 male) patients diagnosed with SCH (ICD-10) and 861 (467 female and 394 male) participants from the general population. The primary outcome was the prevalence of CPM. A secondary outcome was the number of psychiatric readmissions since diagnosis.

**Results:** In the total sample we observed the significant difference in CPM prevalence between SCH and GEP in the youngest age group, <35 years old (p=0.006). Among the male participants <35 years old, there were no significant differences in the prevalence of CPM between SCH (25%) and GEP (15%) (p=0.216). However, among the female participants <35 years old, the difference was significant and clinically relevant (p=0.002). Prevalence of CPM was 50% in SCH patients, and 14% in GEP. After the adjustment for age, sex, a number of psychiatric comorbidities and duration of SCH, the number of physical illness comorbidities was significantly associated with the number of previous psychiatric hospital readmission. (multivariate, robust regression; B=0.98; β=0.24; p=0.022). Approximately, the number of rehospitalizations increases for one with each chronic physical illness.

**Discussion:** This study identified gender differences in the prevalence of CPM in SCH patients, and the significant association of CPM with psychiatric hospital readmission. Higher physical morbidity points to a substantial disadvantage of female patients early in the course of illness. Understanding the nature and biological basis of gender-determined differences in risk and outcome of CPM might help to identify new therapeutic targets, allow more individualized treatment, and facilitate better risk prediction and application of healthcare resources.

### F98. HYPOVITAMINOSIS D IN SCHIZOPHRENIA: ASSOCIATED CARDIOVASCULAR RISK

Rahma Nefzi*[,1], Amine Larnaout[1], Hanen Ben Ammar[1], Emira Khelifa[1], Amina Aissa[1], Zouhaier El Hechmi

[1]*Razi Hospital*

**Background:** Vitamin D modulate the course of many neurologic diseases and conditions. Moreover, the prevalence of vitamin D deficiency might be higher in psychiatric patients, in particular with schizophrenia.

Likewise, there is an inverse relationship between vitamin D levels and several cardiovascular risk factors, including the metabolic syndrome, that patients with schizophrenia are predisposed to develop. It is within this framework that this study aims to explore the relationship between vitamin D levels in a cohort of Tunisian patients with schizophrenia and to determine the cardiovascular risk according to whether they had hypovitaminosis D or not.

**Methods:** A cross-sectional and retrospective descriptive study was conducted at the "F" psychiatry department at the Razi Hospital, Manouba over a twelve-month period from June 1st, 2015 to May 31st, 2016, including 80 patients with schizophrenia in period of clinical remission. The evaluation focused on anthropometric parameters and cardiovascular risk factors. A dosage of vitamin D was performed.

**Results:** The patients had an average age of 42.5 years and 70% were male. 25 patients had metabolic syndrome. 49% of patients had vitamin D insufficiency and 51% had vitamin D deficiency. Vitamin D levels had not been affected by the clinical characteristics of the disease. However, there was no significant association between vitamin D levels and metabolic syndrome. A significant negative correlation was found between the total sum of the various cardiovascular risk factors and the vitamin D deficiency (p <0.001).

**Discussion:** In our study, all patients had vitamin D levels below the recommended levels. 25 patients (31%) met the criteria for metabolic syndrome. All our patients had at least one cardiovascular risk factor. The majority (33% and 27%) had respectively three or four FRCV. 10% had more than five concurrent FRCVs. This result has been described in many studies. Indeed, in patients with schizophrenia, the cardiometabolic risk seems to increase continuously. Several European studies have reported a prevalence of metabolic syndrome ranging from 28% to 37% in patients with schizophrenia. Higher rates of 43% and 46% were reported respectively in the United States and Canada. Moreover, with schizophrenia have an increased risk of sudden death and are 2 to 4 times more likely to die prematurely compared to the general population. These results have been explained with a multicausal model focusing on genetics, lifestyle, smoking, diet and sedentary behavior as well as by the side effects of antipsychotics known to induce weight gain and aggravate symptoms. risk factors for cardiometabolic disease, although studies in naïve patients reflect various abnormalities early on. However, several studies confirm that certain metabolic abnormalities may occur in schizophrenic patients naive to any antipsychotic treatment.

This result is consistent with current literature data that highlight increased metabolic and cardiovascular risk in vitamin D deficiency. Indeed, in the general population, vitamin D deficiency is an important risk factor for cardiometabolic disease. The majority of cohort studies have reported an increase in the incidence of cardiovascular disease in people with low vitamin D levels.

### F99. FIRST EPISODE PSYCHOSIS PATIENTS WHO USED CANNABIS DEVELOP THEIR ILLNESS AT A SIGNIFICANTLY YOUNGER AGE THAN THOSE WHO NEVER USED CONSISTENTLY ACROSS EUROPE AND BRAZIL

Caterina La Cascia[1], Laura Ferraro*[,2], Fabio Seminerio[2], Giada Tripoli[3], Lucia Sideli[2], Diego Quattrone[3], Marta Di Forti[3], Daniele La Barbera[2], Robin Murray[3]

[1]*Ricercatore Universitario;* [2]*University of Palermo;* [3]*Institute of Psychiatry, King's College London*

**Background:** Patients presenting to psychiatric services with their first episode of psychosis (FEP) report higher rates of previous cannabis use than the general population (Donoghue et al., 2011; Myles, Myles and Large, 2016). Evidence suggested that patients suffering from psychosis with a history of cannabis use have an earlier age of onset of psychosis (AOP) than those who never used it (Di Forti et al., 2013).

We aim to investigate if the reported association between use of cannabis and AOP is consistent across different countries, once having taken into account different patterns of cannabis use (i.e. frequency of use and age at first use).

**Methods:** We analysed data on patterns of lifetime cannabis use and AOP from FEP=1,149 (61.7% males) from 5 European countries and Brazil part of the European network of national schizophrenia networks studying European Gene-Environment-Interaction (EUGEI) study.

Patients met ICD-10 criteria for psychosis, ascertained by using OPCRIT (McGuffin et al., 1991).

The CEQmv (Di Forti et al., 2009) further modified for the EUGEI study, was used to collect data on lifetime frequency of cannabis use (never used/ used at least once but less than daily/ everyday use) and age at first use in years (then dichotomized according to mean age at first use ≤15 years or ≥16 years).

We used two ANOVAs: age of onset was used as the outcome variable and frequency of cannabis use and age of first use were respectively entered as independent predictors, along with country, gender and self-ascribed ethnicity.

**Results:** 63.3% of our sample used cannabis at least once in lifetime. Among those who used cannabis in their lifetime, mean age at first use was 16.8 years (sd=4.6) and median age was 16 years, 42.3% tried first time cannabis at 15 years or before, 57.7% at 16 years or older.

Patients who smoked cannabis on a recreational basis (mean age 29.0; contrast=5.8, CI 95% 4.3, 7.2, p<0.001) and on a daily basis (mean age 26.6; contrast=2.4, CI 95% 0.9, 3.9, p=0.001) had lower age of onset than not users patients (mean age 34.8) across all countries, once have taken into account gender and ethnicity

Only, those who started using cannabis ≤15 years had an earlier age of onset (25.5 years) than those who started at their 16 years or later (29.5 years), (F(1,683)=37.3, p<0.001). This relationship was the same across different countries (p=0.968), and independently influenced by ethnicity (F(5, 683)=2.3, p=0.03) but not by gender (p=0.057).

**Discussion:** Our results suggest a generalizable across country and specific effect of frequency of use and early age at first cannabis use on significantly anticipate age of psychosis onset in First episode Psychosis patients.

### F100. FACTOR STRUCTURE OF THE CANNABIS EXPERIENCES QUESTIONNAIRE IN A FIRST-EPISODE PSYCHOSIS SAMPLE

Michael Birnbaum[1], Luca Pauselli*[,2], Sean Cleary[3], Claire Ramsey Wan[4], Michael Compton[5]
*[1]North Shore-LIJ; [2]College of Physicians and Surgeons, Columbia University, New York State Psychiatric Institute; [3]The George Washington University; [4]Cambridge Health Alliance; [5]College of Physicians and Surgeons, Columbia University*

**Background:** The Cannabis Experiences Questionnaire (CEQ) was developed to measure the subjective experiences of cannabis use both during and after intoxication. Despite the need to better understand the nature of the complex and significant relationship between cannabis use and early psychosis, this questionnaire has rarely been used in individuals with first-episode psychosis.

**Methods:** We conducted a set of factor analyses using CEQ data from 194 first-episode psychosis patients who used cannabis, in order to uncover the underlying factor structure of the questionnaire and thus the overarching types of psychological experiences during/after using cannabis in young people with psychotic disorders.

**Results:** Confirmatory factor analyses were performed on the 2 full-scale CEQ factor structures identified in the literature and neither model fit the data within acceptable levels. Using all 56 CEQ items, an exploratory factor analysis (EFA) model was fit with an oblique rotation. Models with 3, 4, and 5 factors were further explored to identify underlying factors. The final 4-factor EFA model provided the best fit. It included 47 items (3 items had multiple loadings and 6 items did not load on any factor), with names given, based on item composition, as follows: Factor 1 (Distortions of Reality and Self-Perception) included 18 items ($\alpha = 0.89$), Factor 2 (Euphoria Effects) included 16 items ($\alpha = 0.89$), Factor 3 (Slowing and Amotivational Effects) included 7 items ($\alpha = 0.81$), and Factor 4 (Anxiety and Paranoia Effects) included 6 items ($\alpha = 0.79$).

**Discussion:** Our derived factor structure differed from those stemming from previous EFAs using different samples (eg, healthy individuals with varying degrees of schizotypy). The inconsistency might be best explained by the different populations sampled, ranging from healthy individuals who have smoked cannabis at least once to individuals with schizophrenia who smoked it regularly. Specifically, differences could be related to variations in how cannabis affects healthy individuals as well as those with schizotypy, as opposed to those with emerging or frank psychosis. Elucidating the underlying factor structure of the CEQ in first-episode psychosis samples could help researchers move towards a deeper understanding of the types of experiences associated with cannabis intoxication among young adults with first-episode psychosis and could inform the development of programs designed to reduce use, improve the course of illness, and possibly delay or prevent the onset of psychotic symptoms in those at risk.

### F101. CANNABIS USE AND HEPATIC STEATOSIS IN PSYCHOSIS: RESULTS FROM A 3-YEAR LONGITUDINAL STUDY

Javier Vázquez-Bourgon[1], Lucía Sanchez Blanco[2], Ruth Landera Rodriguez[2], Esther Setién Suero[3], Rodrigo Romero Jiménez[2], Diana Tordesillas-Gutiérrez*[,4], Rosa Ayesa Arriola[5], Benedicto Crespo-Facorro[5]
*[1]University Hospital Marques de Valdecilla - IDIVAL, University of Cantabria, CIBERSAM; [2]University Hospital Marqués de Valdecilla-IDIVAL; [3]Centro de Investigación Biomédica en Red de Salud Mental (CIBERSAM), Instituto de Salud Carlos III; [4]Valdecilla Biomedical Research Institute IDIVAL; [5]University Hospital Marqués de Valdecilla-IDIVAL, University of Cantabria, Centro de Investigación Biomédica en Red de Salud Mental (CIBERSAM), Instituto de Salud Carlos III*

**Background:** Metabolic alterations are common in patients suffering from psychosis. The rise in glycemic lipids may be related to and observed increased in the prevalence of hepatic steatosis measured by the Fatty Liver Index. However, we have recently reported a probable protective effect of cannabis smoking on weight gain and related metabolic alterations in a sample of patients drug-naïve suffering from a first episode of psychosis. We aimed to explore the effect of cannabis smoking on hepatic steatosis in a sample of first-episode non-affective psychosis patients.

**Methods:** Anthropometric measurements, glycemic and lipid parameters, and liver steatosis index (FLI), were obtained at baseline and after 3 years of having initiated treatment. Patients were divided into two groups depending on self-reported cannabis use (cannabis users and non-users).

**Results:** Cannabis users presented at baseline lower FLI (F=4.26, p=0.040) than non-users. These differences were also observed after 3 years of treatment (F=6.61, p=0.011).

**Discussion:** Our results support the hypothesis that cannabis has a protective effect against hepatic steatosis. However, before being transferred to clinical practice, this study should be replicated, using larger samples.

### F102. CHANGE IN PATTERNS OF CANNABIS AND OTHER SUBSTANCE USE OVER TIME IN EARLY PSYCHOSIS- EXAMINING THE EFFECT OF DEVELOPMENT OF PSYCHOSIS

Musa Sami*[,1], Michael Lynskey[1], Sagnik Bhattacharyya[1]
*[1]King's College London*

**Background:** Understanding how the onset of psychosis affects patterns of substance use would inform the development of effective interventions. To date no study has compared substance misuse patterns over a life-course to a comparable control group to determine how patients who develop psychosis modify substance misuse patterns.

**Methods:** In a well-characterised clinical cohort of patients with psychotic disorders (n=257) we compared frequency of use of most common substances before and after development of psychotic disorder using a within subjects design. Using a between-subjects design we compared patients who had ever used cannabis (n=194) to a control non-clinical cohort of cannabis users (n=1055) over comparable periods in life, accounting for the effects of age, gender, other substance use and location.

**Results:** Patients reduced frequency of consumption of cannabis, alcohol, cocaine and ecstasy (p<=0.001, all comparisons) but not tobacco or crack cocaine. Since adolescence, compared to controls, patients were more likely to reduce cannabis frequency (OR 2.3, p<0.001) and less likely to have increased cannabis frequency (OR 0.2, p<0.001). Patients with psychosis were more likely to have used heavily earlier, with a greater proportion using cannabis more than once weekly, using more potent forms of cannabis, and reporting solitary use up to age 16. However, with regards to current use, controls were currently using more heavily across these parameters than patients.
**Discussion:** Patients who develop psychosis decrease substance consumption after adolescence compared to other non-psychotic substance users. A history of heavy early use of cannabis may be a tractable target for intervention.

### F103. A META-ANALYSIS OF PERSONALITY TRAITS IN DUAL DIAGNOSIS PSYCHOSIS AND SUBSTANCE USE DISORDER

Hyeonju Oh*[,1], Seon-Kyeong Jang[2], Kee-Hong Choi[1]
*[1]Korea University; [2]University of Minnesota, Twin Cities*

**Background:** The comorbidity of substance use and psychotic disorders has been constantly explored in psychosis literature due to its markedly high prevalence and its contribution in debilitating outcomes in various aspects (e.g., lower therapeutic compliance, higher rate of relapse, longer hospitalization, homelessness, and poorer overall functioning). One promising conceptualization of substance use disorder in schizophrenia is understanding shared personality traits such as impulsivity. Thus far, empirical evidences indicated several personality traits as candidate such as impulsivity, neuroticism and anhedonia. However, the accumulated empirical data remains inconsistent across studies. Current study aimed to aggregate data regarding trait personality features in facet level utilizing four-factor UPPS model (Whiteside & Lynam, 2001), four-factor Hierarchical Structural Model (Markon, Krueger, & Watson, 2005), and anhedonia scale for investigating personality traits of impulsivity, neuroticism and anhedonia.
**Methods:** A systematic literature research was conducted using PubMed, Google Scholar, Scopus, and ProQuest, dissertation database in Proquest, and hand searches from reference lists of identified articles. Our analysis covers all studies published up to May 2017. The electronic database research resulted in an initial pool of 110 studies in total, and 12 studies remains for current meta-analysis after screening by inclusion/exclusion criteria and removing duplicate studies. Two authors (SKJ and HJO) independently coded data, and all authors cross-checked to ensure the reliability of coded data to reach consensus. Effect-size estimates were calculated based on means and standard deviations of psychotic disorder only group (PSD) and dual diagnosis group (DD) on personality scales using R package metafor. Specifically, Hedges' g was derived with a random-effect model, allowing unbiased effect sizes adjusted for different sample sizes that helps to infer population-level estimates. To examine potential confounding factors, independent two-sample t-tests were conducted between DD and PSD group for any significant differences in demographic and clinical characteristics. Fail-Safe N test was utilized to address publication bias of the current meta-analysis, and inconsistency of data was assessed using heterogeneity measure (I2). The methodological quality assessment of included studies was created by using an adapted version of Agency for Healthcare Research and Quality (AHRQ) tool for observational studies.
**Results:** There were no statistically significant baseline differences in demographic and clinical characteristics between DD and PSD group. The results indicate that DD patients exhibited significantly higher scores on negative urgency, low premeditation, and sensation seeking compared to PSD within the UPPS model. However, low perseveration did not differ between two groups. Within the HS model, unconscientious disinhibition was significantly higher in DD compared to PSD, but not negative affect, disagreeable disinhibition, and positive affect. Lastly, trait anhedonia score was not significantly different between groups.
**Discussion:** Despite a limited number of available studies, our meta-analysis allowed to specify the personality trait in facet level of dual diagnosis patients compared to patients without substance use disorder. We conclude that the personality trait of DD patients may lead to the employment of urgent emotional regulation strategies (i.e. substance use) to alleviate negative emotion. More effective emotion regulations strategies (e.g., mindfulness, emotion tolerance skills, etc) might need to be integrated for treatment for DD patients.

### F104. A STANDARDISED EMPIRICAL INVESTIGATION OF THE CLINICAL PROFILE OF PSYCHOSIS FOLLOWING TRAUMATIC BRAIN INJURY (PFTBI)

Rachel Batty*[,1], Neil Thomas[1], Andrew Francis[2], Malcolm Hopwood[3], Jennie Ponsford[4], Susan Rossell[2]
*[1]Swinburne University of Technology; [2]RMIT University; [3]University of Melbourne; [4]Monash-Epworth Rehabilitation Research Centre*

**Background:** Persons who experience symptoms of psychosis following a brain injury live with a complex dual diagnosis that is often accompanied by substantial distress and disability. However, a comprehensive examination of the clinical presentation of PFTBI using standardised clinical measures has not been reported in the literature. This information is vital for accurate diagnosis and effective treatment.
**Methods:** Patients with PFTBI (n =10) and schizophrenia (n =98) participated in a comprehensive clinical assessment that included the Structured Clinical Interview for DSM-IV-TR Axis I Disorders (SCID-I/P), the Positive and Negative Syndrome Scale (PANSS), the Scale for the Assessment of Positive Symptoms (SAPS), the Scale for the Assessment of Negative Symptoms (SANS), and the Thought Language and Communication Index (TLC).
**Results:** The clinical profiles of the PFTBI group met symptom/course criteria for: schizophrenia (n = 6), schizoaffective (n = 2), schizophreniform (n = 1), and paranoid psychosis (n = 1). PFTBI patients had a significantly reduced PANSS negative score relative to schizophrenia patients. No other significant differences between PFTBI and schizophrenia clinical profiles were found.
**Discussion:** The clinical profile of PFTBI appears to be comparable to schizophrenia/ schizoaffective disorder except with respect to negative psychotic symptoms. Reduced negative symptoms in PFTBI have previously been reported in a small number of case studies, and thus warrant further investigation as a diagnostic distinction in this patient group.

### F105. MEASURING EMPATHY IN SCHIZOPHRENIA: THE EMPATHIC ACCURACY TASK AND ITS CORRELATION WITH OTHER EMPATHY MEASURES

Rozanne van Donkersgoed[1], Bouwina Sportel*[,2], Steven De Jong[3], Marije aan het Rot[3], Alexander Wunderink[4], Paul Lysaker[5], Ilanit Hasson-Ohayon[6], Andre Aleman[7], Marieke (Gerdina) Pijnenborg[3]
*[1]GGZ Friesland, Rijksuniversiteit Groningen; [2]Department of Psychotic Disorders Assen,[3]Rijksuniversiteit Groningen; [4]Friesland Mental Health Services; [5]Roudebush VA Medical Center, Indiana University School of Medicine; [6]Bar-Ilan University; [7]University Medical Center Groningen*

**Background:** Empathy is a complex interpersonal process thought to be impaired in individuals with schizophrenia spectrum disorders. Past studies have mainly used questionnaires or performance-based tasks with static cues to measure cognitive and affective empathy. In contrast, we used an Empathic Accuracy Task (EAT) designed to capture the more dynamic aspects of empathy by using video clips in which perceivers continuously judge emotionally charged stories of various targets. We compared individuals with schizophrenia to healthy controls on the

EAT and assessed correlations among the EAT and three other commonly used empathy tasks.
**Methods:** Patients (n=92) and healthy controls (n=42) matched for age and education, completed the EAT, the Interpersonal Reactivity Index, the Questionnaire of Cognitive and Affective Empathy and the Faux Pas task. Differences between groups were analyzed and correlations were calculated between empathy measurement instruments.
**Results:** The groups differed in EAT performance, with controls outperforming patients. A moderating effect was found for the emotional expressivity of the target: while both patients and controls scored low when judging targets with low expressivity, controls performed better than patients with more expressive targets. Though there were also group differences on the cognitive and affective empathy questionnaires (with lower scores for patients in comparison to controls), EAT performance did not correlate with questionnaire scores. Reduced empathy performance did not seem to be part of a generalized cognitive deficit, as differences between patients and controls on general cognition was not significant.
**Discussion:** Individuals with schizophrenia benefit less from the emotional expressivity of other people than controls, which contributes to their impaired empathic accuracy. The lack of correlation between the EAT and the questionnaires suggests a distinction between self-report empathy and actual empathy performance. To explore empathic difficulties in real life, it is important to use instruments that take the interpersonal perspective into account.

### F106. STATE AND TRAIT RELATED NATURE OF INSIGHT IMPAIRMENT IN SCHIZOPHRENIA

Lebogang Phahladira*[,1], Bonginkosi Chiliza[2], Laila Asmal[1], Sanja Kilian[1], Freda Scheffler[1], Robin Emsley[1]
*[1]Stellenbosch University; [2]University of KwaZulu Natal*

**Background:** Impairment of insight is a prominent feature of schizophrenia and is associated with poor adherence and poor outcomes. While many studies have investigated the nature of insight impairment in schizophrenia, few have charted its course longitudinally. In this study we investigated changes in different components of insight during the first 12 months of antipsychotic treatment.
**Methods:** The sample comprised 107 never or minimally treated patients with a first episode of schizophrenia, schizophreiform or schizoaffective disorder. They were treated according to a fixed protocol with flupenthixol decanoate. Insight was assessed with the self-rating Birchwood Insight Scale and the investigator rated global insight item of the PANSS scale.Psychopathology was assessed with the PANSS and CDSS. Cognitive performance was assessed with the MATRICS. We performed evaluations at baseline, month 6 and month 12. Linear mixed effects mixed models for repeated measures were conducted to assess changes over time, adjusting for age, gender and educational status.
**Results:** There were no significant changes in the BIS subscales of symptom awareness, awareness of illness or total BIS score. The need for treatment subscale improved slightly (p=0.02) while the PANSS global insight improved considerably (p<0.0001). Degree of insight impairment was only weakly correlated with psychopathology and cognition
**Discussion:** Insight impairment is common in schizophrenia and displays trait-like rather than state-like features. These findings have important clinical implications.

### F107. CSF ABNORMALITIES IN SCHIZOPHRENIA AND DEPRESSION: PRELIMINARY RESULTS FROM A LARGE SCALE COHORT

Tatiana Oviedo Salcedo*[,1], Anna Gagsteiger[1], Lot de Witte[2], Tania Kümpfel[1], René Kahn[2], Peter Falkai[1], Peter Eichhorn[1], Jurjen Luykx[2], Alkomiet Hasan[1]
*[1]Ludwig Maximilian University; [2]University Medical Center Utrecht, Brain Center Rudolf Magnus*

**Background:** CSF abnormalities and a neuroinflammatory pathophysiology have been discussed for affective and non-affective psychosis for more than 30-years. Recent studies pointed towards a specific phenotype of autoimmune-antibody mediated psychosis, but evidence is still sparse. Especially CSF data investigating autoimmune antibodies in large-scale CSF cohorts of affective and non-affective psychoses are lacking.
**Methods:** We analyzed a retrospective naturalistic cohort of 592 patients with A) schizophrenia-spectrum disorders (N = 330) or B) depressive disorders (N = 262) who underwent a lumbar puncture as part of the clinical routine in the Department of Psychiatry and Psychotherapy at the Ludwig-Maximilians University Munich between July 2012 and May 2017. We used a predefined systematic algorithm for the database search in the clinical documentation system and data was extracted by TO and AG. The study was approved by the local ethics committee.
**Results:** We identified 592 patients with standard CSF parameters. Schizophrenia spectrum patients did not differ from depressive patients with regard to the white blood cell count (cells/µl) (p = 0.774) or albumin quotient (p = 0.663). The general prevalence of oligoclonal bands did not differ between groups (schizophrenia: 37.0%, depression: 37.8%; p = 0.838). However, schizophrenia patients showed higher frequencies for intrathecal oligoclonal bands (32% of all oligoclonal bands) compared to depressive patients (19.1% of all oligoclonal bands. (p = 0.034). 124 schizophrenia-spectrum patients (54 first-episode patients) received CSF analyses for neural antibodies. None of the patients showed positive CSF results in any of the tested autoimmune-encephalitis panel (NMDA(N=119), AMPA-1(N=114), AMPA-2(N=114), CASPR(N=111), LGI-1(N=110) and GABA-B(N=112)-Antibodies) in CSF. The results for the intracellular onconeuronal and synaptic antibodies were also negative (Amphyphysin(N=93), Yo(N=58), Hu(N=94), Ri(N=94), CV2(N=93), Ma1(N=93) and Ma2(N=93)-Antibodies). Three of these patients with negative CSF titers did have low-titer neuronal antibodies in serum: CASPR-2-AB: 1:10, CASPR-2-AB: 1:50, Yo-AB: low band-intensity. 36 depression patients were also tested for autoimmune antibodies and again no positive reports could be identified in CSF.
**Discussion:** This is the first analyses of autoimmune antibodies in first-episode and recurrent schizophrenia and depressive mood disorder showing no positive CSF titers. However, schizophrenia patients have a higher prevalence of intrathecal oligoclonal bands compared to affective patients pointing towards more immunological disturbances in this population. The here presented analyses are exploratory and need to undergo confirmatory analyses and quality control.

### F108. PSYCHOTIC EXPERIENCES IN A NORWEGIAN SAMPLE - TENTATIVE RESULTS OF A QUESTIONNAIRE VALIDATION

Isabella Kusztrits*[,1], Frank Larøi[2], Julien Laloyaux[2], Lynn Marquardt[1], Igne Sinkeviciute[3], Eirik Kjelby[4], Erik Johnsen[5], Josef Bless[6], Kristiina Kompus[6], Kenneth Hugdahl[5], Marco Hirnstein[1]
*[1]University of Bergen; [2]University of Bergen, NORMENT Center for the Study of Mental Disorders, University of Liège; [3]Haukeland University Hospital, NORMENT Center for the Study of Mental Disorders; [4]University Hospital; [5]Haukeland University Hospital, University of Bergen, University of Oslo; [6]University of Bergen, NORMENT Center for the Study of Mental Disorders*

**Background:** Auditory verbal hallucinations are a major symptom in schizophrenia but also affect patients with other diagnoses and healthy people without any pathology. This applies also to delusions and hallucinations of other sensory modalities. Since most questionnaires that assess hallucinations focus on one particular disorder, the Questionnaire for Psychotic Experiences (QPE) was created to provide an instrument that is applicable independently of clinical status.

The QPE is a semi-structured interview consisting of 50 items that are categorized into four subscales (visual, auditory, other hallucinations and delusions). Each subscale starts with a screening question to indicate if a symptom is present, followed by questions regarding specific characteristics. We translated the QPE into Norwegian (bokmål) and distributed the screening questions online with the aims (1) to validate the Norwegian version of the screener QPE and (2) to assess the prevalence of hallucinations and delusions in the Norwegian population independently of the clinical status.

**Methods:** We conducted an online survey using a test/re-test design, which comprised the 13 screening questions of the QPE as well as demographic and clinical questions. Seven days after initial completion of the QPE participants received a link for the second round. For test/re-test reliability, we calculated concordance rates (i.e., percentage rates of how many participants gave the same response at the first and second measurement). Internal consistency is indicated with Cronbach's alpha. Finally, we calculated a principal component analysis (PCA) for the QPE items to identify the QPE's item structure. The study was approved by the regional ethics committee.

**Results:** Until now, 407 individuals (304 females, 103 males) with an age range of 18 to 78 (mean = 32.7) participated in the first part of the online survey, of whom 185 also took part in the re-test.

Twenty-eight % of all participants had at least one psychiatric diagnosis. Among the healthy participants alone, 35% reported auditory hallucinations, 26% visual hallucinations, 40% tactile hallucinations and 28% olfactory hallucinations. Around 68% of all healthy participants reported at least one delusional experience.

Cronbach's alpha across all 13 items for the entire sample was 0.772 in the first round and 0.765 in the second round. Test/re-test reliability was between 79% and 99%. The PCA, also based on the entire sample, revealed one dimension, with high loadings especially on delusion-related questions (range: 0.488–0.697).

**Discussion:** The distribution of different modalities of hallucinations and delusions in the healthy sample suggests that psychotic experiences are not necessarily connected to diagnoses. This finding is in accordance with other studies and supports the hypothesis that psychotic experiences are independent of the clinical status.

The Cronbach's alpha suggests a good internal consistency at both time points, which stays stable over time and the test/re-test reliability shows a high accordance between the answers of round one and two. The PCA implies that the QPE screener is best characterized with a unidimensional structure, indicating that there is substantial overlap between hallucinations and delusions, even though factor loadings are particularly high for delusions. We conclude that the Norwegian version of the screener QPE is a viable tool for assessing psychotic experiences across both psychiatric and healthy populations.

### F109. BOUNDARIES BETWEEN DEFICIT AND NONDEFICIT SCHIZOPHRENIA: LONG TERM STABILITY AND OUTCOMES

Gustavo Mustafé*[,1], Diego Mendes[1], Thaís Martins[1], Thalita Fernandes[1], Clarissa Dantas[1]

[1]*Medical School, State University of Campinas*

**Background:** Negative symptoms of schizophrenia are admittedly associated with a poorer outcome regarding aspects such as functionality, quality of life and cognitive performance. Patients with prominent, persistent and primary negative symptoms have been considered to manifest a putative subtype called "Deficit schizophrenia" (DS). However, the boundaries of deficit and nondeficit forms were put in question since the publication of a study that considered separately a group of patients with persistent negative symptoms whose primary nature could not be asserted, the "ambiguous nondeficit" group, who would be otherwise categorized as nondefict according to the gold standard instrument: the Schedule for the Assessment of the Deficit Syndrome (SDS). Those patients presented psychopathological features, quality of life, insight and cognitive function quite different from the nondeficit group, and closer to the deficit group. The objectives of the present study are to investigate the stability of the categorization regarding the presence of DS in three groups: "deficit"(DS), "nondeficit" (ND) and "ambiguous nondeficit" (SND) over a long term follow-up and to evaluate clinical outcomes in the different groups.

**Methods:** We will contact 85 patients with schizophrenia, considered clinically stable in the previous year, who participated in a study about the DS in 2009/2010. Back then, they were recruited in two sites: an outpatient service of a university general hospital (49 patients) and a community-based mental health service (36 patients). Patients will be assessed with the same instruments adopted in the first study: a questionnaire for clinical and demographic information; BPRS, SAPS, SANS, Calgary Depression Scale, the SDS, QLS, and a battery of neurocognitive tests. We started the recruitment by the patients originally treated in the outpatient clinic.

**Results:** Here we present partial results. Of the 49 patients, 5 refused to participate in the follow-up study, 3 died prematurely, and 1 had the diagnostic changed for bipolar disorder. Assessment interval was 6.9 years ± 0.5 Among the 20 reassessed patients, mean age at baseline was 36.9 ± 8.9 years, mean duration of mental illness was 16 ± 10.1 years, and 75% were men. They had in mean, 10.7 ± 3.3 years of education, only 20% had any work activity, 15% were married and 55% had a low socioeconomic position. These demographic aspects slightly worsened: only 15% had an occupation at follow-up, and 60% fell in the lower socioeconomic position. Regarding the SDS classification, 4 of 9 ND patients at the baseline were reclassified as DS; 1 of 7 DS was reclassified as ND, the other 6 remained DS; from the AND, 3 were considered DS and 1 ND, from a total of 4. At the end, there were 13 DS and 7 ND, while at the baseline they were: 7 DS, 9 ND and 4 AND. Concerning psychopathology, 80% of the patients had an increase in SANS and the most expressive increase was in nondeficit group (an average of 5.4 points), although the average in DS group remained the higher (18.9 points). Still, SAPS and Calgary remained low in all three subgroups, with a mean of 6.20 and 2.20 points, respectively. As to medication, 70% of the baseline were in use of Clozapine (67% of ND, 57% of DS and 100% of the AND group) and that total number remained the same during the follow up.

**Discussion:** Our preliminary results are derived from a small sample. Although we cannot draw definite conclusion, these outcomes suggest trends that are worth observing: the worsening of negative symptoms among patients and the tendency of conversion to DS group, especially among the "ambiguous" group. This advocates against the dichotomous division of deficit and nondeficit schizophrenia and speaks in favor of a dimensional understanding of negative symptoms.

### F110. THE BRIEF NEGATIVE SYMPTOM SCALE (BNSS): VALIDATION IN A MULTICENTER BRAZILIAN STUDY

Heydrich Medeiros[1], Helio Elkis*[,2], Selene Vasconcelos[1], Diana Martins[1], Raissa Alexandria[3], Ana Albuquerque[3], Rosana Freitas[4], Maria Scardoelli[4], Elaine Di Sarno[4], Isabel Napolitano[4], Graça Oliveira[4], Adriana Vizzotto[4], Antônio Peregrino[3], Murilo Lima[3]

[1]*Federal University of Paraíba;* [2]*University of Sao Paulo Medical School;* [3]*University of Pernambuco;* [4]*University of São Paulo*

**Background:** Negative symptoms are a core feature of schizophrenia. The Brief Negative Symptom Scale (BNSS) is a new scale developed to assess negative symptoms in schizophrenia.

**Methods:** The present study aimed to examine the construct validity of BNSS, by using convergent and divergent validities as well as factor analysis, in a Brazilian sample of 111 outpatients diagnosed with schizophrenia by DSM-5. Patients were evaluated by the Brazilian version of the BNSS and positive and negative subscales of the Positive and Negative Syndrome Scale (PANSS)
**Results:** Assessment of patients by both instruments revealed an either an excellent internal consistency (Cronbach's alpha = 0.938) or inter-rater reliability (ICC = 0.92), as well as a strong correlation between BNSS and negative PANSS (r = 0.866) and a weak correlation of the instrument with the positive PANSS (r = 0.292) thus characterizing adequate convergent and discriminant validities, respectively. The exploratory factor analysis identified two distinct factors, namely, motivation/pleasure and emotional expressivity, accounting for 68.63% of the total variance.
**Discussion:** The study shows that the Brazilian version of the BNSS has adequate psychometric properties and it is a reliable instrument for the assessment of negative symptoms in schizophrenia, either for clinical practice or research.

### F111. ELECTROPHYSIOLOGICAL CORRELATES OF AVOLITION-APATHY DOMAIN IN SCHIZOPHRENIA

Giulia Giordano*[,1], Thomas Koenig[2], Armida Mucci[1], Annarita Vignapiano[1], Antonella Amodio[1], Giorgio Di Lorenzo[3], Cinzia Niolu[3], Mario Altamura[4], Antonello Bellomo[4], Silvana Galderisi[1]
*[1]University of Campania "Luigi Vanvitelli"; [2]Translational Research Center, University Hospital of Psychiatry Bern; [3]University of Rome Tor Vergata; [4]University of Foggia*

**Background:** Negative symptoms represent a core feature of schizophrenia. They have been associated to poor functional outcome, worse quality of life and poor response to pharmacological treatment. Several factor analytic studies have reported that negative symptoms can be divided into two domains referred to as Avolition-apathy which includes Avolition, Anhedonia and Asociality and the Expressive deficit domain, which includes Alogia and Blunted affect.
Avolition-apathy has been associated to a dysfunction of brain circuits involved in motivation, in particular to those related to the ability to anticipate pleasure and learn from rewards. It is highly controversial whether Avolition-apathy and all subcomponent symptoms share the same neurobiological underpinnings.
Our study, using brain electrical microstates (MS) which reflect global, subsecond patterns of functional connectivity, had two primary aims: 1) to identify differences between healthy controls (HC) and clinically stable people with schizophrenia (SCZ) in brain electrical microstate parameters and 2) to investigate the associations of the microstate parameters with the Avolition-apathy domain and its subcomponent symptoms.
**Methods:** We analyzed multichannel resting EEGs in 142 SCZ and in 64 HC, recruited within the add-on EEG study of the Italian Network for Research on Psychoses. The microstate analysis was performed using an in-house plugin for Brain Vision Analyzer. Based on the microstate map templates from a large normative study, each moment of the ongoing EEGs was assigned to one of four microstates (MS) classes (MS-A, MS-B, MS-C, MS-D). Microstates were then quantified in terms of relative time contribution, duration and occurrence. Negative symptoms were assessed using the Brief Negative Symptoms Scale (BNSS): Avolition-apathy was obteined by summing the scores on the subscales Anhedonia (consummatory and anticipatory anhedonia), Avolition and Asociality; Expressive deficit was computed by summing the scores on the subscales Blunted Affect and Alogia.
Analysis of variance (ANOVA) was used to test group differences on MS parameters. Pearson's r coefficients were computed to investigate the correlations of MS parameters with the negative symptom domains and subcomponent symptoms.
**Results:** There was no significant group difference in sex (p=0.073) and age (p=0.547) between SCZ and HC. SCZ, in comparison to HC, showed increased contribution (p=0.009) and duration (p=0.016) of MS-C.
As regard to negative symptoms, the total score of the BNSS was positively correlated with the contribution of MS-A (r= 0.19, p<0.03). Avolition-apathy domain (r=0.22, p<0.01), anticipatory anhedonia (r=0.20, p=0.02), avolition (r=0.20, p=0.02) and asociality (r=0.25, p=0.003), but not consummatory anhedonia (r=0.13, p=0.13), were positively correlated with the contribution of MS-A. There was no correlation between Expressive deficit and MS-A parameters.
**Discussion:** Our findings, in line with previous studies, showed an increased contribution of MS-C in SCZ. MS-C was not associated with clinical features, thus probably representing a trait marker of the disease. In addition, our results support different neurophysiological correlates of the two negative symptom domains and suggest that only anticipatory anhedonia, but not consummatory anhedonia, might be linked to the Avolition-apathy domain. These findings are in line with studies reporting an intact ability to experience in the moment pleasure and an impairment in pleasure anticipation (anticipatory anhedonia) in people with schizophrenia.

### F112. LESS SYMPTOMS IN SCHIZOPHRENIA – A RISK FACTOR FOR IMPAIRED INSIGHT OF FUNCTIONING?

Olsson Anna-Karin*[,1], Maivor Olsson-Tall[2], Hawar Moradi[2], Fredrik Hjärthag[1], Lars Helldin[3]
*[1]Karlstad University [2]NU Health Care Hospital; [3]Health Care Hospital, and Karlstad University*

**Background:** People with schizophrenia demonstrate deficits in insight and the ability to self-evaluate their functioning. Research about patients' ability to recognize their psychotic symptoms is well established, but recent findings show that there are still unexplored fields regarding how patients perceive their level of functioning A previous study showed that patients who overestimate their functioning, also consistently get high scores in interview-based assessment regarding real-world functional performance. The possible consequences of patients' ability to correctly estimate their function need to be further investigated. The aim of the present study was to examine how the perception of one's own capacity relate to symptoms in patients with schizophrenia spectrum disorders.
**Methods:** Data collection took place within the ongoing project Clinical Long-term Investigation of Psychosis in Sweden (CLIPS), which examines psychiatric outpatients. In this study, 222 patients with schizophrenia participated. They were divided into four groups based on their results on the UPSA-B and their self-perceived function; two groups with ordinary function (accurate estimators and under -estimators) and two groups with low function (accurate estimators and over-estimators). The groups were compared regarding psychiatric symptoms, examined using the Positive and Negative Syndrome Scale (PANSS). Non-parametric statistics were used to analyze differences in their symptoms.
**Results:** There were statistically significant differences in the total score of PANSS across the four groups of function. The following analyses showed significant differences in the negative and general domain. Results from the post hoc examination revealed identical patterns in these two symptom domains. The group with Low function accurate estimators have significantly more severe symptoms compared to the other three groups.

**Discussion:** The result in the present study showed that patients with low function who overestimate their function have less or the same level of symptoms as patients in the two groups with ordinary functioning. In further studies it is important to investigate if this actually is a result of lower symptom level or if it is due to the impaired insight. This is important since the result in the present study mirror previous results where patients who overestimate a low function also, by clinicians, will be perceived as patients with a higher capacity and less difficulties.

### F113. IMPACT OF NEIGHBORHOOD CHARACTERISTICS ON PARANOIA, LONELINESS, AND PERCEIVED REJECTION IN A TRANSDIAGNOSTIC SAMPLE WITH PSYCHOSIS

Christina Savage*[,1], Cristina Garcia[1], LeeAnn Shan[1], Alexandra Andrea[1], Melanie Bennett[2], Jack Blanchard[1]
*[1]University of Maryland; [2]University of Maryland School of Medicine*

**Background:** Paranoia is unsubstantiated thinking that others want to cause harm, and it exists on a spectrum ranging from suspicion to delusions both in the general population and in individuals with psychosis (Freeman et al., 2011; Freeman, 2016). Paranoia is interpersonal in nature, and research has shown that individuals with paranoid delusions use person attributions more than depressed or healthy controls during a task with interpersonal vignettes (Bentall, Kaney, & Dewey, 1991). Therefore, it is important to explore how other interpersonal or social factors may affect paranoia. Neighborhood characteristics, such as reduced social cohesion and crime, perceived rejection, and loneliness have been associated with paranoia (Lamster, Nittel, Rief, Mehl, & Lincoln, 2017; Newbury et al., 2017; Wickham, Taylor, Shevlin, & Bentall, 2014). However, it is still unclear whether these factors affect paranoia independently or have a more additive influence. Some researchers have proposed that perceived rejection and loneliness reduce community engagement and the size of social networks in this population (Cechnicki & Wojciechowska, 2008; Kidd et al., 2016), but others have not supported these findings (Trémeau et al., 2016). The current study will try to clarify the literature by exploring the associations between neighborhood characteristics, social factors (namely, perceived rejection and loneliness), social network, and paranoia.
**Methods:** The current study will examine how paranoia correlates with neighborhood characteristics, loneliness, perceived rejection, and social network size in a transdiagnostic sample with psychosis. We will utilize the Neighborhood Environment Scale (Mujahid et al., 2007) to assess social cohesion, safety, violence, and activities with neighbors within participants' residencies. We will use the Paranoid Thought Scales (Green et al., 2008) to assess paranoid ideation and the Adult Social Relationship Scales (Cyranowski et al., 2013) to assess perceived rejection and loneliness over the past month. In addition, we will use the Social Network Index (Cohen et al., 1997) to investigate the correlation between participants' social network and paranoia, social rejection, and loneliness.
**Results:** Preliminary results (N = 13) indicate a significant correlation between paranoia and perception of neighborhood social cohesion ($r = -0.57$, $p < 0.05$). In addition, loneliness ($r = 0.60$, $p < 0.05$) and perceived social rejection ($r = 0.52$, $p < 0.05$) were the largest correlates of paranoia. We will conduct formal analyses with a larger N to further explore these and other associations.
**Discussion:** Discussion will be included in the poster after more data has been collected.

### F114. DISORGANIZATION AND COGNITIVE DYSFUNCTIONS IN SCHIZOPHRENIA: A STUDY OF RESTING STATE EEG

Ananrita Vignapiano*[,1], Thomas Koenig[2], Armida Mucci[1], Giulia Maria Giordano[1], Antonella Amodio[1],

Giorgio Di Lorenzo[4], Cinzia Niolu[3], Mario Altamura[4], Antonello Bellomo[4], Silvana Galderisi[1]
*[1]University of Campania "L. Vanvitelli"; [2]Translational Research Center, University Hospital of Psychiatry Bern; [3]University of Rome 'Tor Vergata'; [4]Psychiatry Unit, University of Foggia*

**Background:** A disorganization factor was found by several factor-analytic studies of schizophrenia symptoms. This factor does not appear to be affected by age, severity of other symptoms and chronicity of illness. A greater severity of disorganization is associated with poor functioning. Despite the general similarity of different factorial model, there is no consensus about which symptoms have to be included in the disorganization factor. Using the Positive and Negative Syndrome Scale (PANSS), Conceptual disorganization' (P2), 'Difficulty in abstract thinking' (N5) and 'Poor attention' (G11) were core features of the disorganization factor.
The overlap of these items with neurocognitive functions is still debated. However, the heterogeneity of this factor and its neurobiological basis should be further investigated.
In the context of the multicenter study of the Italian Network for Research on Psychoses, the main aims of our study were to investigate electrophysiological and neurocognitive correlates of the disorganization factor, and to assess if each PANSS item, loading on the disorganization factor, could be underpinned by similar electrophysiological or cognitive alterations.
**Methods:** Resting state EEGs were recorded for 5 minutes in 145 stabilized subjects with schizophrenia (SCZ) and 69 matched healthy controls (HC). The disorganization factor was evaluated using three PANSS items: P2, N5, and G11 (4).
Neurocognitive functions were assessed using the MATRICS Consensus Cognitive Battery (MCCB). Spectral amplitude was quantified in nine frequency bands. All statistical analyses of the scalp multichannel spectral amplitude (SAmp) data were performed using RAGU software.
Statistical comparisons between the SAmp maps of SCZ and HC were assessed by topographic analyses of variance (TANOVA). In SCZ, topographic analyses of covariance (TANCOVA) evaluated correlations between SAmp and disorganization, its constituent items and MCCB domains. Furthermore, Pearson's correlations were performed between disorganization and its constituent items and MCCB neurocognitive domains.
**Results:** TANOVA, comparing the group SAmp maps revealed increased Delta, Theta, and Beta1 and decreased Alpha2 SAmp in SCZ.
In the SCZ group, disorganization was significantly correlated to the Alpha1 SAmp. This relation was negative and most pronounced at occipital sites. At the items level, only N5 showed the same negative correlation at occipital sites.
MCCB neurocognitive composite score was associated with disorganization factor, and its constituent items P2 and N5. No significant correlation between Alpha1 SAmp and MCCB cognitive domains was observed.
**Discussion:** Our findings illustrate the heterogeneity of disorganization dimension and a partial overlap with neurocognitive domains. 'Difficulty in abstract thinking' showed a unique association with Alpha1 activity, which is thought to be involved in the construction of conceptual maps.
Furthermore, the observed association of Alpha1 with 'Difficulty in abstract thinking' suggests that some aspects of disorganization could be underpinned by the impairment of basic neurobiological functions that are only partially evaluated using MCCB.

### F115. INSIGHT AND MANIC SYMPTOMS IN PATIENTS WITH CHRONIC SCHIZOPHRENIA IN THE KOREAN COMMUNITY

Duk-In Jon*[,1], Bo-Hyun Yoon[2], Sang-Yeol Lee[3], Kwanghun Lee[4], Won-Myong Bahk[5], Beomwoo Nam[6], Sung-Yong Park[7], Min-Kyu Song[8]
*[1]Hallym University Sacred Heart Hospital; [2]Naju National Hospital; [3]Wonkwnag University School of Medicine and Hospital; [4]Dongguk University Hospital; [5]Yeouido St. Mary's Hospital, The*

*Catholic University of Korea; [6]Konkuk University; [7]Keyo Hospital; [8]Yesan Mental Health Clinic*

**Background:** Many studies have highlighted the similarity of the symptoms between bipolar disorders and schizophrenia. Moreover, there are no pathognomonic symptoms that can differentiate these two disorders, and 9% of schizophrenia patients have experienced a manic syndrome in their lifetime. Insight about their symptoms and illness is very important factor for the differential diagnosis and the management in schizophrenia. To examine the relationship among the insight, the psychotic and manic symptoms, and clinical variables in patients with chronic schizophrenia.
**Methods:** Seventy-four participants (male 44, female 30) with chronic schizophrenia in community mental health facilities have been evaluated with the Scale to assess Unawareness of Mental Disorder (SUMD), the Mood Disorder Questionnaire (MDQ), and the Brief Psychiatric Rating Scale (BPRS).
**Results:** The mean number of previous admission was 3.85. Their drug adherence was favorable (6.73 day/week). Mean CGI-S score was 3.8. Thirty-five percent of subject were MDQ positive (cutoff point = 7 or more). Among SUMD, "awareness of effect of medication" showed significant negative correlation (r = -0.33) with total MDQ score not with total BPRS score. The negative correlation was more obvious in participants with negative MDQ (total MDQ score 6 or less, r = -0.31). Several MDQ items (irritability, r = -0.25; decreased sleep, r = -0.27; thought racing, r = -0.28; and easy distractibility, r = -0.40) negatively correlated with "awareness of effect of medication". In contrast, only one item (guilt feeling, r = -0.27) of BPRS revealed this correlation. Individual items in MDQ and BPRS rarely correlated with each other. Total MDQ score was not correlated with duration of illness and medication adherence.
**Discussion:** Manic symptoms were frequently detected even in schizophrenia as reported in previous studies. This made it difficult to differentially diagnose the disorder using only the total MDQ score. There was possible relationship between these manic symptoms and their insight. Identifying manic symptoms in schizophrenia would be considerable in clinical setting.

### F116. CAN INSIGHT LEAD TO REMISSION - FOR PATIENTS WITH SCHIZOPHRENIA?

Maivor Olsson-Tall*[,1], Fredrik Hjärthag[2], Anna-Karin Olsson[1], Madeleine Johansson[1], Hawar Moradi[1], Lars Helldin[1]
*[1]NU Health Care Hospital; [2]Karlstad University*

**Background:** It is important to have extensive knowledge of the patients with schizophrenia, to provide the right support in outpatient care to create a good situation for the patient and prevent hospitalization. Lack of insight regarding the illness and symptoms might impair the patient's ability to understand the illness, the treatment and relapses into psychotic episodes. Symptomatic remission is a well-established goal for treatment. If the core-symptoms do not affect their functions and the status is stable for at least six months, the patient is in remission. Previous research has shown an increased remission status from approximately 30% to more than 50% after using standardized remission criteria for patients with schizophrenia in Sweden. This study aims to investigate the relationship between both insight of symptoms and illness insight with cross-sectional remission.
**Methods:** This is a cross-sectional study and the participants consisted of totally 289 patients with schizophrenia diagnosis. Of the participants 111 were women and 178 were men, with a mean age of 47 years (19–83 years old). Using semi-structured interviews and evidence-based assessment scales Remission Scale - Symptom (RS-S) and Psychosis Evaluation Tool for Common use by Caregivers (PECC) the data is collected. Cross tabulations were used to compare the distribution of the variables and the Pearson Chi-Square Tests for examine significant association.
**Results:** Insight of symptom: The results show that from the patients who are not in remission, 69.5% are missing insight to symptoms, while 30.5% are having insight of symptoms. When it comes to the patients within remission, 59.0% have an insight of symptoms, while 41.0% are missing this sort of insight. The findings in the analysis with Chi-Square Tests examine independence indicated significant association between insight of symptoms and remission status (1, x2 = 22.17), p = <0,001, phi = -0.28.
Insight of illness: During the analysis of insight of illness, the results show that from the patients who are in remission, 61.9% possess insight of illness, compared to 38.1% of the patients lacking insight of illness. Concerning the insight of illness for the patients who are not in remission, 68.2% lack insight of illness, while 31.8% possess insight of illness. The Chi-Square Tests examine independence indicated significant association between insight of illness and remission status (1, x2 = 24.28), p = <0.001, phi = -0.29.
**Discussion:** The results show that there is a relationship between insight of symptoms and illness with the cross-sectional symptomatic remission. However, the question is still open if remission is a consequence of insight or if the insight changes over time according to the activity of the illness. By following patients over time and monitoring the activity of symptoms including the state of remission and insight, it will probably be visualized if changes occur related to each other or independently. Also, whether the main focus for success is pharmaceutical treatment aiming for maximal symptom reduction or psychoeducational treatment to develop patients' ability to understand their illness. Finally, whether insight after being established is a state or a treatment phenomenon? Further research to explore this issue is needed.

### F117. SCHIZOTYPY PERSONALITY TRAITS RELATED TO PSYCHOLOGICAL FUNCTIONING AND INTERNALIZED STIGMA

Olivia Altamirano*[,1], Amy Weisman de Mamani[1]
*[1]University of Miami*

**Background:** People diagnosed with schizophrenia spectrum illnesses report higher levels of internalized stigma in comparison to other mental health diagnoses (Holzinger, Beck, Munk, Weithaas, & Angermeyer, 2003). Studies have shown high overlap between depression and symptoms of schizotypy in nonclinical adolescents (Fonseca-Pedrero, Paino, Lemos-Giráldez, & Muñiz, 2011), but the role that stigma plays in this relationship has yet to be examined. This is of importance because it can be targeted (Rüsch, Angermeyer, & Corrigan, 2005) and prior literature has found that awareness campaigns to reduce stigma can improve psychological functioning (Mittal, Sullivan, Chekuri, Allee, & Corrigan, 2012). Based on previous literature, we predict that schizotypal personality traits will be related to symptoms of depression, anxiety and stress (DASS), and that these will both be related to internalized stigma.
**Methods:** The current study is a sample of 494 college students who completed surveys to assess for schizotypal personality traits (SPQ; Raine, 1991), depression, anxiety and stress (Depression Anxiety Stress Scales DASS; Lovibond & Lovibond, 1995), and internalized stigma (ISMI; Boyd, Otilingam, & DeForge, 2014).
**Results:** Correlation coefficients indicated that higher endorsement of schizotypal personality was associated with greater DASS scores (r=.645; p<.01) and internalized stigma (r=.406; p<01). A multiple regression was conducted regressing SPQ on ISMI and DASS, F (2,491) = 183.949, p<.01, R2=.428. Controlling for ISMI scores, DASS was predictive of higher schizotypal personality ratings (Beta=.586; p<01). Controlling for DASS, ISMI scores were also predictive of schizotypal personality (Beta=.123; p=.002).
**Discussion:** As hypothesized, schizotypy, DASS, and internalized stigma were all positively associated. Internalized stigma could lead to symptoms of depression, anxiety and stress as well as schizotypy. It is possible that internalized stigma plays its own unique role in the onset of schyzotypy. This study is limited by the self-report and cross-sectional nature. Longitudinal studies are necessary to further assess causality in these variables. Screening for schizotypal personality traits when patients present for symptoms of depression and anxiety could be useful in early intervention efforts. Moreover, campaigns that target mental illness stigma could aid in improving psychological functioning, and in reducing schizotypal personality traits.

### F118. ARCHITECTURE OF PSYCHOSIS SYMPTOMS AND NEURAL PREDICTORS OF CONVERSION AMONG CLINICAL HIGH RISK INDIVIDUALS WITH AUTISM SPECTRUM DISORDER

Jennifer Foss-Feig*[,1], Eva Velthorst[1], Sylvia Guillory[1], Holly Hamilton[2], Brian Roach[3], Peter Bachman[4], Aysenil Belger[5], Ricardo Carrion[6], Erica Duncan[7], Jason Johannesen[8], Gregory Light[9], Margaret Niznikiewicz[10], Jean Addington[11], Kristin Cadenhead[9], Tyrone Cannon[12], Barbara Cornblatt[6], Thomas McGlashan[12], Diana Perkins[5], Larry Seidman[13], Ming Tsuang[10], Elaine Walker[14], Scott Woods[12], Carrie Bearden[15], Daniel Mathalon[2]

*[1]Icahn School of Medicine at Mount Sinai; [2]University of California, San Francisco, San Francisco VA Health Care System; [3]Northern California Institute for Research and Education; [4]University of Pittsburgh; [5]University of North Carolina; [6]Zucker Hillside Hospital; [7]Emory University, Atlanta VA Medical Center; [8]Yale University, VA Connecticut Healthcare System; [9]University of California, San Diego; [10]Harvard Medical School/BHCS; [11]University of Calgary; [12]Yale University; [13]Harvard Medical School; [14]Emory University; [15]University of California, Los Angeles*

**Background:** Individuals with autism spectrum disorders (ASD) have symptoms, including social and sensory deficits, and neurobiological alterations that overlap with schizophrenia. Though there is evidence of high rates of psychosis symptoms in ASD, little is known about psychosis prodrome in ASD, or about predictors of psychosis conversion in this population. In this study, we leverage data from clinical high risk (CHR) patients from the NAPLS2 consortium to examine: a) baseline differences in psychosis symptoms and social functioning, b) relative risk of conversion, and c) whether neural response to sensory stimuli yields differential predictors of conversion in CHR individuals with and without ASD (CHR/ASD+; CHR/ASD-).

**Methods:** Clinical, electrophysiological, and 24-month follow-up data were available for 305 individuals (14 CHR/ASD+; 291 CHR/ASD-). We examined baseline differences on the SOPS, GFS, and TASIT. Conversion risk was computed with the Cannon conversion calculator, and conversion was defined as SOPS>6 at 2-year outcome. P300 event-related potentials (ERP) were extracted from ongoing EEG collected at baseline in response to Target and Novel auditory and visual stimuli, each presented on 10% of trials within streams of 80% standard stimuli in the same modality.

**Results:** In line with our expectations, CHR/ASD+ had worse functioning than CHR/ASD- on the GF-Social scale (t=-4.2, p<.01) and TASIT total score (t=-2.9, p=<.01), but groups did not differ in their psychotic symptoms on the SOPS (Positive: p=.72; Negative: p=.13; Disorganization: p=.13; General: p=.86). Groups did not differ in the rate at which they converted to psychosis (CHR/ASD+: 15.4%; CHR/ASD-: 11.1%; p=.50), and the Cannon risk score was equally predictive of 2-year conversion across groups (p=.39). EEG data revealed dissociable profiles regarding neural response to sensory stimuli in those who did versus did not convert to psychosis, depending on ASD status. P300 response over central electrodes to Novel visual stimuli was weaker in CHR- converters (n=71) than CHR-non-converters (n=220), but stronger in CHR/ASD+ converters (n=4) than CHR/ASD+ non-converters (n=10) (Novel Stimuli: Modality by ASD interaction, F=5.66, p=.02; Modality by ASD by Converter Interaction, F=3.57, p=.06). For both auditory and visual Target stimuli, P300 response over parietal electrodes did not differ between CHR/ASD- converters and non-converters; however, whereas CHR/ASD+ individuals who did not convert had amplitudes similar to all CHR/ASD- individuals, CHR/ASD+ converters had substantially greater auditory and visual P300 amplitudes (Target Stimuli: ASD by Converter interaction, F=12.12, p=.001).

**Discussion:** Individuals with ASD and CHR have greater social deficits than the general CHR population, but show similar psychotic symptoms and have similar risk for conversion to psychosis. Neural response to sensory stimuli is important for understanding risk for conversion, and differs among CHR individuals dependent on whether they have ASD. In particular, whereas all CHR individuals who do not convert share a common pattern of attenuated ERP amplitudes reflecting attention allocation to target and novel auditory and visual stimuli, CHR/ASD+ who convert have a unique pattern of globally heightened P300 responses to infrequent novel and target stimuli. These findings have two important implications: 1) individuals with ASD do convert to psychosis and have similar CHR symptom and risk profiles to non-ASD CHR patients clinically; 2) in CHR individuals with ASD in particular, examining neural markers of attention allocation to sensory stimuli may reveal important predictive clues about risk for conversion.

### F119. MULTILEVEL ANALYSIS IMPROVES THE MODEL FIT OF THE DIMENSIONAL STRUCTURE OF THE PANSS IN PATIENTS WITH SCHIZOPHRENIA

Cinthia Higuchi[1], Hugo Cogo-Moreira[1], Bruno Bertolucci*[,1], Christoph U. Correll[2], Cristiano Noto[1], Quirino Cordeiro[3], Rosana Freitas[4], Hélio Elkis[4], Sintia I. Belangero[1], Rodrigo A. Bressan[1], Ary Gadelha[1]

*[1]Universidade Federal de São Paulo; [2]The Zucker Hillside Hospital, Hofstra North Shore LIJ School of Medicine; [3]Faculdade de Ciências Médicas da Santa Casa de São Paulo; [4]Universidade de Sao Paolo*

**Background:** Principal component analyses (PCA) studies show that schizophrenia symptoms are usually grouped into five domains. However, to infer a latent dimensional structure, confirmatory factor analysis (CFA) is more appropriate than PCA. Most CFA studies addressing the five-factor model yielded poor fit indices. One single study achieved a good fit using a multilevel CFA structure with the interviewers as level. Other possible reasons for sample heterogeneity and subsequent poor model adjustments, such as differences in patients' clinical profiles across clinical units and clinical staging, were not measured in this study. We aimed to replicate the effect of the CFA multilevel analyses and evaluate the possible influence of other heterogeneity sources as levels, i.e., clinical staging, on the Positive and Negative Syndrome Scale (PANSS) five-factor structure.

**Methods:** 700 patients with schizophrenia at four different centers had their PANSS analyzed. A Confirmatory Factor Analysis (CFA) was conducted using the following fit index: Comparative Fit Index (CFI) and Non-Normed Fit Index (NNFI) >0.95, the Root Mean Square Errors of Approximation (RMSEA) <0.06, and Weighted Root Mean Square Residual (WRMR) <1.0. Thereafter, we performed multilevel analyses considering the following levels: i) centers, ii) interviewers and iii) clinical staging for schizophrenia (first episode, treatment-resistant schizophrenia and non-treatment resistant schizophrenia).

**Results:** The mean (SD) age was 34.9 (10.3) years, mean age of onset was 21.7 (7.5), mean duration of illness means was 13.2 (9.7) years, and 64.3% of the sample was male. The CFA model without multilevel analyses yielded poor fit indices: RMSEA = 0.102 (90% CI: 0.097 – 0.107; Cfit was <0.001), CFI = 0.921 and NNFI = 0.906 and WRMR = 1.952. When the multilevel analysis was applied, all models reached an acceptable fit: i) centers: RMSEA = 0.044 (90% CI: 0.038 – 0.049; CFit = 0.964), CFI = 0.981, NNFI = 0.977, and WRMR = 1.860; ii) interviewers: RMSEA = 0.047 (90% CI: 0.041 – 0.053; CFit = 0.765), CFI = 0.947, NNFI = 0.938, and

WRMR = 1.531; iii) clinical stage: RMSEA = 0.052 (90% CI: 0.046 – 0.058; CFit = 0.274), CFI = 0.988, NNFI = 0.985, and WRMR = 2.433.
**Discussion:** Good CFA model fits were only achieved when the multilevel structure was applied. Besides the bias generated by data collection (i.e., local of data collection and raters), the clinical staging is a potential source of variability to consider in schizophrenia dimensional structure. As dimensional approaches gain relevance to reduce heterogeneity in schizophrenia and to investigate their biological substrates, reliable methods to address latent dimensions are required.

### F120. USING DIGITAL MEDIA ADVERTISING IN EARLY PSYCHOSIS INTERVENTION

Michael Birnbaum*,[1], Chantel Garrett[2], Amit Baumel[1], Asra Rizvi[1], Whitney Muscat[1], John Kane[1]
[1]*Northwell Health;* [2]*Strong 365 (One Mind)*

**Background:** Identifying and engaging youth with early stage psychotic disorders in order to facilitate timely treatment initiation remains a major public health challenge. While advertisers routinely use the internet to directly target consumers, limited efforts have focused on applying available technology to proactively encourage help seeking in the mental health community. This study explores how one might take advantage of Google Adwords in order to reach prospective patients with early psychosis.
**Methods:** A landing page was developed with the primary goal of encouraging help seeking individuals in New York City to contact their local early psychosis intervention clinic. In order to provide the best opportunity to reach the intended audience, Google AdWords was utilized linking over 2,000 manually selected search terms to strategically placed landing page advertisements. The campaign ran for 14 weeks between April 11th and July 18th 2016 with a total budget of $1427.
**Results:** The ads appeared 191,313 times and were clicked on 4,350 times at a per-click cost of $.33. Many users took additional help seeking steps including obtaining psychosis specific information/education (n=1,918 / 44%), completing a psychosis self-screener (n=671 / 15%) and contacting the Early Treatment Program (n=57 / 1%).
**Discussion:** Digital ads appear to be a reasonable and cost effective method to reach individuals who are searching for behavioral health information online. More research is needed to better understand the many complex steps between online search inquiries and making first clinical contact.

### F121. DOES RELAPSE CONTRIBUTE TO TREATMENT RESISTANCE? ANTIPSYCHOTIC RESPONSE IN FIRST- VS. SECOND-EPISODE SCHIZOPHRENIA

Hiroyoshi Takeuchi*,[1], Cynthia Siu[2], Gary Remington[3], Gagan Fervaha[4], George Foussias[3], Robert Zipursky[5], Ofer Agid[3]
[1]*Keio University School of Medicine;* [2]*COS and Associates Ltd.;* [3]*Centre for Addiction and Mental Health (CAMH), University of Toronto;* [4]*Queen's University Faculty of Medicine;* [5]*McMaster University,*

**Background:** The objective of this study was to compare trajectories of antipsychotic response before and after relapse following response from a first episode of schizophrenia or schizoaffective disorder.
**Methods:** The current analysis included patients with a diagnosis of first-episode schizophrenia or schizoaffective disorder who met the following criteria: (1) referral to the First-Episode Psychosis Program between 2003 and 2013; (2) treatment with an oral second-generation antipsychotic according to a standardized treatment algorithm; (3) positive symptom remission; (4) subsequent relapse (i.e., second episode) in association with non-adherence; and (4) reintroduction of antipsychotic treatment. The following outcomes were used as an index of antipsychotic treatment response: change in the Brief Psychiatric Rating Scale (BPRS) total score and number of patients who achieved positive symptom remission, including 20% and 50% response improvement.
**Results:** A total of 130 patients were included in the analyses. All patients took the same antipsychotic in both episodes. Antipsychotic doses in the second episode were significantly higher than those in the first episode (P=0.03). There were significant episode-by-time interactions for all outcomes of antipsychotic treatment response over 1 year (all Ps<0.001) in favor of the first episode compared to the second episode. Results remained unchanged after adjusting for antipsychotic dose.
**Discussion:** The present findings suggest that antipsychotic treatment response is reduced or delayed in the face of relapse following effective treatment of the first episode of schizophrenia.

### F122. CLINICAL CHARACTERISTICS OF LATE-ONSET SCHIZOPHRENIA IN COMPARISON WITH EARLY-ONSET SCHIZOPHRENIA: ONE YEAR FOLLOW-UP STUDY

Jung Suk Lee*,[1], Mi-Ae Ko[1], Seon-Koo Lee[2]
[1]*NHIS Ilsan Hospital;* [2]*Bundang Jesaeng Hospital*

**Background:** Late-onset schizophrenia (LOS) differs from early-onset schizophrenia (EOS) in several ways including predominance of women, better premorbid social adjustment and lower severity of positive/negative symptoms. However, no studies evaluated the longitudinal course of LOS. This study aimed to investigate the clinical characteristics of LOS in comparison with EOS and the longitudinal course of clinical symptoms and functioning in LOS.
**Methods:** By reviewing medical records, we assessed demographic data, clinical characteristics, and general functioning of 20 LOS (5 males) and 44 EOS (16 males) who admitted to National Health Insurance Service Ilsan Hospital. LOS and EOS were defined according to age at first onset: ≥40 years (LOS) and <40 years (EOS). The level of clinical symptoms were rated using the Positive and Negative Syndrome Scale (PANSS), and general functioning was evaluated using the General Assessment of Functioning (GAF).
**Results:** There was no significant difference in gender between LOS and EOS. The mean ages of onset were 45.4 ± 3.97 (LOS) and 28.4 ± 6.69 (EOS) years. Significantly more LOS patients (90.0%) had a marital history including divorce than EOS (56.8%). There were no differences between LOS and EOS in the positive, negative, and general scores of PANSS measured at admission and 1 year after. LOS patients had significantly higher score of PANSS N2 item (Emotional withdrawal) both at admission (LOS: 4.00 ± 1.34; EOS: 3.43 ± 1.52) and 1 year after (LOS: 3.50 ± 1.00; EOS: 2.91 ± 1.05) than EOS. There were negative correlations between GAF (1 year after) and N2 item score (at admission: r= –0.45, p=0.04; 1 year after: r= –0.85, p<0.001) in the LOS group, but no significant correlation exists in the EOS group.
**Discussion:** Consistent with previous studies, our study suggested that LOS patients had better premorbid social functioning because marital history can be regarded as index of premorbid social adjustment. However, on the contrary to previous findings, LOS patients had more severe emotional withdrawal and it was related to worse functioning. This finding may be due to cultural specificity in Korea; thus, further studies with larger samples are needed for confirmation.

### F123. BELIEFS ABOUT THEIR VOICES AND DEGREE OF RESILIENCE IN PERSONS WITH AUDITORY VERBAL HALLUCINATIONS WITH AND WITHOUT NEED FOR CARE

Julien Laloyaux*,[1], Alberto Collazzoni[2], Isabella Kusztrits[3], Marco Hirnstein[3], Kristiina Kompus[4], Josef J. Bless[4], Frank Larøi[1]

[1]University of Bergen, NORMENT – Norwegian Center of Excellence for Mental Disorders Research, University of Oslo, University of Liège; [2]University of California, Los Angeles; [3]University of Bergen; [4]University of Bergen; NORMENT – Norwegian Center of Excellence for Mental Disorders Research, University of Oslo

**Background:** Auditory Verbal Hallucinations (AVH) are prevalent in many psychopathologies but are also experienced by a minority of the healthy general population. There is cumulative evidence that the beliefs people hold about their voices (e.g., power) are strongly related to the impact of the voices (e.g., depression, anxiety) and to the coping strategies that they adopt (e.g., resistance, engagement). To date, research on resilience has identified many factors that promote wellbeing and that protect people from developing psychopathologies despite exposure to health or psychological adversities. However, no previous studies have examined resilience in people who experience AVH with and without need for care, and neither have the relations between resilience and beliefs about the voices been examined.
**Methods:** Fifty persons who report hearing voices frequently were recruited online. Based on the presence of a psychiatric diagnosis, the use of antipsychotic medication, and on the consultation of a psychiatrist, they were then classified as being Healthy Voice-Hearers (HVH) or Patients (P). One hundred and nineteen healthy participants who have never experienced hearing voices were also recruited as a control group (CTRL). All participants completed the Resilience Scale for Adults. In addition, the HVH and P groups completed questionnaires that assess the beliefs they hold about their voices (the revised Beliefs About Voices Questionnaire) and voice characteristics (frequency and emotional content).
**Results:** The data collection is currently underway, and thus the following results are preliminary. Kruskal-Wallis ANOVAs revealed significant differences between the three groups (HVH, P, CTRL) on several resilience factors. In particular, post-hoc analyses demonstrated that the CTRL and HVH groups were more resilient than the P group for the perception of self and of future. In addition, the HVH group was found to be more resilient than the P group in terms of social competence. Finally, for social factors (social resources and family cohesion), results showed that the CTRL group was more resilient than the P group. However, the HVH group was not significantly different from the P and the CTRL groups. Concerning voice characteristics, Mann-Whitney tests revealed that, compared to the P, the HVH perceived their voices as being less omnipotent and malevolent, less negative and more positive, and showed less resistance against the voices. Finally, correlational analyses (Spearman) demonstrated that better resilience (and in particular the individual factors such as the perception of self and of the future, and social competence) was related to fewer negative beliefs about the voices, less resistance, lower voice frequency, and less negative and more positive emotional content.
**Discussion:** The present study showed that people who experience AVH without need for care have a different pattern of resilience compared to patients with AVH, and to healthy controls without AVH. In particular, the HVHs did not differ from the CTRL on the personal factors of resilience and did not differ from the patients in terms of social factors. In addition, better resilience (and especially the personal factors) was found to be related to fewer negative beliefs about the voices, better coping strategies, lower voice frequency, and less negative and more positive emotional content. Taken together, these results show that resilience – and in particular, the personal factors – may be an important variable influencing the need for care in people experiencing AVH. The present study has important theoretical and clinical implications, in particular, suggesting that the personal factors of resilience may be a treatment target in order to diminish the impact of voices.

### F124. SEX DIFFERENCES IN OUTCOME IN FIRST EPISODE PSYCHOSIS PATIENTS: A 10-YEAR FOLLOW-UP STUDY

Rosa Ayesa-Arriola*,[1], Esther Setién-Suero[2], Diana Tordesillas-Gutierrez[3], Benedicto Crespo-Facorro[4]
[1]Marqués de Valdecilla University Hospital, IDIVAL, School of Medicine, University of Cantabria; [2]Centro de Investigación Biomedica en Red de Salud Mental (CIBERSAM); [3]University Hospital Marques de Valdecilla -IDIVAL; University of Cantabria; CIBERSAM; [4]Marqués de Valdecilla University Hospital, IDIVAL, Instituto de Investigación Valdecilla

**Background:** Specialized early intervention programs are efficient in treating patients with a first episode of psychosis (FEP) at least after 2 years. However, few studies have examined long-term outcomes, and particularly prognostic implications of the sex of FEP patients.
**Methods:** We aimed to investigate long-term neuropsychological and functional outcomes in female and male 10 years after the first presentation of a non-affective psychotic episode. One hundred sixty-five FEP patients, 73 women and 92 men were assessed for sociodemographic, clinical and neuropsychological information.
**Results:** Differences in outcome between female and male based on baseline, 1-year, 3-year and 10-year follow-up information were substantial, showing women better outcomes on several variables. Schizophrenia diagnosis was significantly more frequent in men (82% vs. 62%; $p = 0.01$). Women were more likely than men married (45% vs. 24%; $p = 0.01$) and having children (41% vs. 13%); $p < 0.001$). Significant differences arose for social function ($F= 5.469$; $p = 0.022$) and processing speed ($F = 12.66$; $p < 0.001$). There was also some weak evidence (albeit not quite statistically significant at $p < 0.05$) for negative symptoms and global neurocognitive function.
**Discussion:** Women who suffered a first episode of psychosis have better functional and neurocognitive outcomes compared to men. This differential outcome profile is important for clinicians to consider sex specific therapeutic approaches.

### F125. POOR OUTCOME SCHIZOPHRENIA (KRAEPELINIAN SUB-TYPE): SOCIAL COGNITIVE AND NEURODEVELOPMENTAL MARKERS

Emmanuel Chevallier*,[1], Audrey Tanguy[2], Marie-Odile Krebs[3], Serge Mitelman[4], Monte Buchsbaum[5], Marie-Cécile Bralet[6]
[1]FJ6 CHI Clermont de l'Oise; [2]AP-HP Paris; [3]SHU Ste Anne, Paris; [4]Icahn School of Medicine; [5]NeuroPet Center, UCSD; [6]Unité CRISALID CHI Clermont de l'Oise

**Background:** Poor outcome schizophrenia represents a public health challenge and it asks questions about neurodevelopmental mechanisms by its own. The kraepelinian schizophrenia sub-type, defined by Keefe's criteria (1987), refers to a very poor prognosis sub-group (severe dysfunction in self-care) on the basis of the longitudinal course of the illness. Studies on kraepelinian sub-group show differences with good outcome patients regarding pre-morbid functioning, negative and disorganized symptoms, impaired performance on specific social cognitive and motor deficits (visual-motor processing, abstraction/flexibility, fine motor dexterity) (Albus and al., 1996; Bralet and al., 2006). Neurological soft signs (NSS) refer to subtle neurological abnormalities comprising deficits in sensory integration, motor coordination and sequencing of complex motor acts. Previous studies showed that NSS scores are correlated to schizophrenia, specifically among patients with poor premorbid functioning and with severe negative and disorganization symptoms. NSS could be a neurodevelopmental marker interesting to detect patients with a risk of poor prognosis. Deficits in theory of mind is correlated with disorganization and poor prognosis. The aim of our study was to explore the association between NSS, Theory of Mind and kraepelinian sub-type in order to understand better the etiopathogenic mechanisms underlying the kraepelinian sub-type.
**Methods:** In 2016, we recruited 2 samples of 25 schizophrenic patients, kraepelinians and no-kraepelinians, matched on sex, ages (+/- 5 years) and duration of illness (+/- 5 years) from the psychiatric departments in

Picardie area (France), according to DSM-IV-TR criteria and using Keefe's criteria. Several socio-demographical, pharmacological, clinical, cognitive and NNS (with the 3 subscores, sensory integration, motor integration and motor coordination) (Krebs and al., 2000) were collected for each patient. To compare the 2 sub-groups we used bi-variate analysis and multivariate regression analysis (p< 0,05)
**Results:** Results showed a worse significant NSS score among kraepelinian patients: total score, sensory integration, motor integration, motor coordination, p< 0,0001; there was no link with treatment (equ mg/day chlorpromazine). As well kraepelinians show worse significantly performance at the eye gaze test p<0,001. Multivariate analysis showed that kraepelinian sub-type is more explained significantly by eyes-test, motor integration and disorganization dimension
**Discussion:** Poor prognosis schizophrenia refers to specific and complex neurodevelopmental mechanisms which could be markers of a poor outcome. We must confirm these results in a larger prospective cohort from UHR and first episode assessing some specific neurodevelopmental markers using NSS and cognitive assessment. As well some specific biological markers and genes implicating in neurodevelopment and glutamatergic system could be studied in these patients. Focusing on these specific markers could contribute to define innovating combinating therapeutic strategies (pharmacological, cognitive remediation and social skills) to avoid poor prognosis.

### F126. PATHWAYS FROM SPEECH ILLUSIONS TO PSYCHOTIC SYMPTOMS IN SUBJECTS AT ULTRA-HIGH RISK FOR PSYCHOSIS: COMBINING AN EXPERIMENTAL PARADIGM OF ABERRANT EXPERIENCES WITH NETWORK ANALYSIS

Adela-Maria Isvoranu[1], Lindylou Boyette*[,1], Frederike Schirmbeck[1], Eva Velthorst[2], Claudia Simons[3], Denny Borsboom[1], Lieuwe De Haan[4]
*[1]University of Amsterdam; [2]Icahn School of Medicine at Mount Sinai; [3]GGzE / Maastricht University; [4]AMC-Academisch Psychiatrisch Centrum*

**Background:** One of the oldest and most influential theories of psychosis formation states that delusions arise in an attempt to explain unusual experiences, including perceptual aberrations. The White Noise Task by Galdos et al (2011) was developed as an experimental task to assess the tendency to attribute meaning to random perceptual stimuli: speech illusions in white noise. Studies to date have demonstrated that speech illusions as assessed with the White Noise Task are associated with a composite measure of positive symptoms in patients with psychotic disorders (Galdos et al, 2011; Catalan et al, 2014). However, findings in non-clinical samples have been inconsistent: one study found an association with a composite measure of subclinical positive symptoms, including support for a relation with familial psychosis liability (Galdos et al, 2011), whereas other studies did not find any association in non-clinical samples or only partly (Catalan et al, 2014; Rimvall et al, 2016; Pries et al, 2017). The current study aims to further examine whether speech illusions as assessed with the White Noise Task are indicative of psychosis liability and to explore specific symptomatic pathways.
**Methods:** We conducted symptom-based network analyses in Ultra-High Risk (UHR) subjects participating in the European network of national networks studying gene-environment interactions in schizophrenia project (EU-GEI, 2014; www.eu-gei.eu). Psychotic symptoms were assessed with the Brief Psychiatric Rating Scale (BPRS). Transition to clinical psychosis was assessed with the Comprehensive Assessment of At Risk Mental State (CAARMS). We used a conservative measure of speech illusions, as described in Catalan et al (2014).
**Results:** The current sample consisted of 339 UHR subjects, of which 9.1% (N=31) experienced speech illusions. Preliminary network analyses in cross-sectional baseline data showed potential pathways from speech illusions to delusional ideation, through hallucinatory experiences. We also found evidence of prospective relations between speech illusions at baseline and transition to clinical psychosis. Pathways ran via baseline psychotic symptoms and affective symptoms, as well as a 'direct' pathway.
**Discussion:** As far as we are aware, this is the first study combining an experimental measure of aberrant experiences with symptom-based network analysis. Although the current reported findings are preliminary and exploratory, they tentatively support a relation between speech illusions as assessed with the White Noise Task and psychosis liability. This relation may be dependent on sample composition, and not generalizable to the general population as a whole. Future studies may benefit from focusing on more detailed trajectories of both susceptibility to speech illusions and course of (sub)clinical psychotic symptom severity in subjects with increased risk for psychosis, with use of more frequent, short assessment periods and inclusion of environmental risk factors for transition to clinical disorder.

### F127. GLOBAL RECOVERY IN A FIRST EPISODE PSYCHOSIS PROGRAM IN SOUTH AMERICA

Barbara Iruretagoyena*[,1], Nicolas Crossley[1], Juan Undurraga[2], Carmen Castañeda[2], Carlos Gallardo[2], Felipe Mancilla[2], Alfonso Gonzalez[2]
*[1]P. Universidad Catolica de Chile; [2]Early Intervention Program, J. Horwitz Psychiatric Institute*

**Background:** Metanalisis show that global recovery, (a state of clinical and social well functioning) is achieved by 13.5% patients (25%-75% quartiles 8.1–20%) (Jääskeläinen, 2013) diagnosed with schizophrenia. It has also been suggested that recovery is higher in low or lower middle-income countries compared to high and upper middle income countries. However, this is only based in a few studies. We here looked at the number of patients with first episode psychosis that met recovery criteria based on both clinical and social domains in a South American early intervention sample. We also examined whether recovery was associated with factors such as diagnoses, sex, education, substance use and duration of untreated psychosis.
**Methods:** This is a cross-sectionall study in an outpatient First Episode Psychosis program in Chile. We gathered information on different aspects of the patients, including sociodemographic, clinical, functional and metabolic status. FAST (Functional Assessment Short Test) and SS-DSM5 (Symptom Severity Scale of the DSM5 for Schizophrenia) were applied to patients. Global recovery was defined as the presence for at least 6 months of: 1. Working or studying. 2. SS-DSM5 scale with no dimension with score over two. 3. FAST with score under 21 (which correlates with GAF > 61). The group who met recovery criteria (improvement in both clinical and social domains) was identified, and correlation and regression analysis were performed to explore the association between global recovery and selected variables.
**Results:** We included 80 patients in this study. Overall, 20% met global recovery criteria. Patients who did not accomplish recovery did so because of being unemployed (80.6%), not studying (79.7%), or scoring above threshold in SS-DSM5 cognitive (54.7%) and negative (54.7%) symptom domains. Univariate correlation analyses showed a significant association of global recovery with recreational drug use, diagnoses, and duration of untreated psychosis (all corrected for multiple comparisons). After multiple regression analysis including these variables, age and gender, the only one associated with recovery was shorter duration of untreated psychosis (p=0.02) OR 0.616 (IC95% 0.409–0.925).
**Discussion:** The number of patients achieving global recovery is consistent with the one reported for schizophrenia in previous meta-analysis and with studies on recovery after first episode psychosis (16.6% (25%-75% quartiles

9–20.4)) (Jääskeläinen, 2013). Negative and cognitive symptoms frequently impair patient recovery. On the other hand the duration of untreated psychosis shows itself as one of the most important characteristics related with functional prognoses.

### F128. THE AGE OF ONSET OF SCHIZOPHRENIA SPECTRUM DISORDERS

Jouko Miettunen*[,1], Johanna Immonen[1], John McGrath[2], Matti Isohanni[1], Erika Jääskeläinen[1]
*[1]University of Oulu; [2]University of Queensland, Queensland Brain Institute*

**Background:** This study characterizes the age of onset of schizophrenia spectrum disorders and summarizes findings regarding a range of clinical and social outcomes, cognition, brain structure, and mortality.
**Methods:** The review is based on series of systematic and nonsystematic literature searches. We included original articles and systematic reviews looking associations between age of onset and incidence, risk factors, suicides, brain structure and cognition.
**Results:** The peak age of onset for schizophrenia spectrum disorders is between 20 to 29 years, in where the incidence estimate was among males 4.15 and among females 1.71 per 10,000 person-years. Male gender has been linked with earlier onset age, although among those with family history and cannabis use corresponding gender difference do not exist. Early onset schizophrenia has been linked e.g. with higher familial risk, poor premorbid social adjustment and cannabis use. In adult samples, earlier age of onset associated with worse outcome, regarding hospitalisations, negative symptoms, relapses, social and occupational functioning, and global outcome. Also in childhood and adolescence schizophrenia, earlier onset has been linked with more severe outcomes. Early age of onset has been linked also with larger cognitive deficits and brain alterations. In the few existing studies, later AOO has been linked with a higher suicide rate. In all, the current study found various differences between patients with different age of onset. However, the studies on age of onset are relative heterogeneous on methodology and have given varying results. More good quality studies are needed including patients without restriction due to the onset age.
**Discussion:** Age of onset is an important characteristic of schizophrenia that could help when examining the origin, genetic mechanism and care of schizophrenia. Understanding factors that influence age of onset in schizophrenia may offer clues to prevent or delay the onset of this debilitating group of disorders.

### F129. COMBINED PATTERNS OF TOBACCO AND CANNABIS USE IN ADOLESCENCE AND THEIR ASSOCIATION WITH PSYCHOTIC EXPERIENCES: A LONGITUDINAL ANALYSIS

Stanley Zammit*[,1], Hannah Jones[2], Suzanne Gage[3], Jon Heron[2], Matthew Hickman[2], Glyn Lewis[4], Marcus Munafo[2]
*[1]Cardiff University; [2]University of Bristol; [3]University of Liverpool; [4]University College London*

**Background:** There has been increasing concern about potentially causal effects of tobacco use on psychosis, but epidemiological studies have been less robust in attempts to minimise effects of confounding than studies of cannabis use have been. We therefore aim to examine the association of patterns of cigarette and cannabis use with preceding and subsequent psychotic experiences, and compare patterns of confounding across these patterns.
**Methods:** We analysed repeated measures of cigarette and cannabis use during adolescence in a sample of 5,300 individuals in the Avon Longitudinal Study of Parents and Children birth cohort who had at least 3 measures of cigarette and cannabis use between ages 14–19 years. Cigarette and cannabis use data were summarised using longitudinal latent class analysis to identify longitudinal classes of substance use, and associations between classes and psychotic experiences at 18 years were assessed.
**Results:** Prior to adjusting for a range of potential confounders, there was strong evidence that early-onset cigarette-only use (4.3%), early-onset cannabis use (3.2%), and late-onset cannabis use (11.9%), but not later-onset cigarette-only use (14.8%) latent classes were associated with increased psychotic experiences compared to non-users (65.9%) (omnibus $P<0.001$). After adjusting for confounders, the association for early-onset cigarette-only use attenuated substantially (unadjusted odds ratio (OR) = 3.03, 95%CI 1.13, 8.14; adjusted OR = 1.78, 95%CI 0.54, 5.88), whereas those for early-onset (adjusted OR = 3.70, 95%CI 1.66, 8.25) and late-onset (adjusted OR = 2.97, 95%CI 1.63, 5.40) cannabis use were unchanged.
**Discussion:** Our findings indicate that whilst individuals who use either cannabis or cigarettes during adolescence have an increased risk of developing subsequent psychotic experiences, the epidemiological evidence for this being causal is substantively more robust for cannabis than it is for tobacco

### F130. INCREASED RISK OF PSYCHOTIC DISORDERS IN AFRICAN MIGRANTS TO AUSTRALIA

Linglee Downey[1], Scott Eaton[1], Kristen Thien[1], Melissa Bardell-Williams[1], Meghan Bowtell[1], Aswin Ratheesh[1], Patrick D. McGorry[1], Brian O'Donoghue*[,1]
*[1]Orygen, the National Centre of Excellence in Youth Mental Health*

**Background:** Certain migrants groups are at an increased risk of psychosis compared to the native-born population, however these findings relate to certain countries, mainly in Europe and America where the research has been conducted. It is not yet known whether migrants to Australia are at an increased risk for developing a psychotic disorder. This study aimed to determine whether first-generation migrants in a geographically defined catchment area in Melbourne have an increased risk of developing a psychotic disorder.
**Methods:** This study included an all young people aged between 15 and 24 residing in a geographically defined catchment area of north western Melbourne who presented to the Early Psychosis Prevention and Intervention Centre (EPPIC) between 01.01.11 and 31.12.13. Data pertaining to the at risk population was obtained from the Australian 2011 Census and incidence rates ratios were calculated.
**Results:** A total of 527 individuals with FEP were included, 393 were Australian-born (74.6%) and 134 (25.4%) were overseas-born. First generation migrants from Kenya (IRR=9.81), Ethiopia (IRR=5.17), Somalia (IRR=3.78), and Sudan (IRR=3.57), had significantly increased risk of having a psychotic disorder. Conversely, first generation migrants from India and China had significantly decreased risk of having psychosis.
**Discussion:** First-generation migrants from East Africa and the Horn of Africa have significantly high rates of psychosis and they may have experienced factors pre-, during, and post-migration, predisposing them to psychosis

### F131. CHILDHOOD ADVERSITIES IN PEOPLE AT ULTRA-HIGH RISK (UHR) FOR PSYCHOSIS: SYSTEMATIC REVIEW & META-ANALYSIS

Oon Him Peh[1], Attilio Rapisarda*[,1], Jimmy Lee[1]
*[1]Institute of Mental Health*

**Background:** Childhood adversities such as childhood abuse, bullying victimisation, and parental separation have been found to be associated with many psychiatric illnesses, including psychosis. A large body of research has been conducted on individuals at ultra-high risk (UHR) for psychosis, or clinical high risk (CHR)

for psychosis. This review aims to quantitatively summarise (i) the associations between childhood adversities and the UHR state, and (ii) how these adversities may be linked with a higher risk of transition to psychosis (TTP).

**Methods:** We conducted systematic searches based on PubMed, EMBASE, and PsycINFO databases. We adopted search terms aimed at retrieving studies related to: (1) populations which were at UHR of psychosis, (2) exposure to childhood adversities, and (3) schizophrenia. Studies were eligible as long as they reported information on any form of childhood adversities and recruited participants at UHR of psychosis. Studies that only investigated the level of psychotic symptoms in a cohort or among schizophrenia patients were excluded.

Whenever possible, we conducted meta-analyses to compare, across UHR and healthy individuals: (a) the levels of childhood trauma exposure, (b) childhood bullying victimisation, and (c) parental separation or loss. We conducted a second set of meta-analyses to investigate the effect of childhood trauma on TTP. Whenever allowed by provision of detailed information, we also conducted separate meta-analytic computations for each reported subtype of childhood adversity and trauma. All analyses were conducted in Review Manager 5.3, using inverse variance or Mantel-Haenszel methods (random effects model).

**Results:** The systematic searches yielded 13 case-control, cross-sectional, and prospective studies from 27 publications, which recorded exposure to childhood adversities among UHR individuals: five of these studies employed longitudinal designs to investigate the conversion rate among UHR. Meta-analytic calculations revealed that, as compared to healthy controls, UHR individuals reported more severe childhood trauma (Random effects Hedges' g = 1.38; 95% CI: 0.92–1.84, Z = 5.92, p < .001), were 5.5 times and 2.5 times more likely to report emotional abuse (OR = 5.54, 95% CI = 1.13–27.20, p = .03) and physical abuse (OR = 2.53, 95% CI = 0.73 - 8.76, p = .14) respectively. UHR individuals were 3.1 times as likely to report bullying victimisation (OR = 3.09, 95% CI = 2.23 - 4.30; Z = 6.72, p < .001). However, childhood trauma exposure in general was not significantly associated with psychotic conversion (HR = 1.01, 95% CI: 0.99 - 1.03; Z = 1.51, p = .13), suggesting perhaps that this risk is either mediated by other risk factors or that most specific traumatic experiences may contribute to an enhanced risk of conversion among UHR individuals.

**Discussion:** To date, this is the first meta-analysis that quantitatively summarises the associations between childhood adversities and TTP, and between specific abuse subtypes and the UHR state or TPP. Overall, our findings support the association between childhood adversities (trauma and bullying) and the UHR state; however, these adversities alone may not be sufficient to cause a UHR individual to develop frank psychosis. Most studies did not adjust for potentially confounding variables such as cannabis use, gender, education level, age, comorbid psychiatric disorders and other unmeasured variables such as socioeconomic status, urbanicity, genetic risk, and PTSD symptoms. The current review supports the need to screen for childhood adversities among the UHR population and to provide treatment accordingly, which may improve patients' engagement with their treatments and result in better clinical outcomes.

### F132. IDENTIFICATION OF PATIENTS WITH RECENT ONSET PSYCHOSIS IN KWAZULU NATAL, SOUTH AFRICA: A PILOT STUDY WITH TRADITIONAL HEALTH PRACTITIONERS AND DIAGNOSTIC INSTRUMENTS

Wim Veling*[,1], Jonathan Burns[2], Hans W. Hoek[3], Ezra Susser[4]
*[1]University Medical Center Groningen; [2]University of Exeter; [3]University of Groningen; [4]Columbia University*

**Background:** There is considerable variation in epidemiology and clinical course of psychotic disorders across social and geographical contexts. To date, very little data is available of low- and middle-income countries (LMICs). Obtaining valuable evidence from under-represented regions such as Sub-Saharan Africa holds the promise of advancing our knowledge and understanding of psychosis and will provide a strong basis for redressing inequities in service provision for people with psychotic disorders living in LMICs. Many patients in these countries remain undetected and untreated, partly due to lack of formal health care facilities. This study in rural South Africa aimed to investigate if it is possible to identify patients with recent onset psychosis in collaboration with traditional health practitioners (THPs).

**Methods:** We developed a strategy to engage with THPs. Key to the collaboration between psychiatry, THPs and the local community, was the building of trust by recognizing and acknowledging local authorities, mutual respect for health constructs, taking time to find common ground, and adaptation of the procedures to sociocultural norms. Fifty THPs agreed to collaborate and were asked to refer help-seeking clients with recent onset psychosis to the study. At referral, the THPs rated probability of psychosis ("maybe disturbed" or "disturbed"). A two-step diagnostic procedure was conducted, including the self-report Community Assessment of Psychic Experiences (CAPE) as screening instrument, and a semi-structured interview using the Schedules for Clinical Assessment in Neuropsychiatry (SCAN). Accuracy of THP referrals, and test characteristics of the THP rating and the CAPE were calculated.

**Results:** In six months, 149 help-seeking clients were referred by THPs, of which 44 (29.5%) received a SCAN DSM-IV diagnosis of psychotic disorder. The positive predictive value of a THP "disturbed" rating was 53.8%. Test characteristics of the CAPE were poor.

**Discussion:** This pilot study in rural South Africa found that it is possible to identify patients with recent onset psychosis in collaboration with THPs. THPs not only grasped the concept of psychosis, they recognized "being disturbed" as a condition that is often difficult to treat and for which collaboration with psychiatric mental health care might be beneficial. By contrast, the CAPE performed poorly as a screening instrument. Collaboration with THPs is a promising approach to improve detection of patients with psychosis in LMIC.

### F133. ARE WE UNDERESTIMATING THE INCIDENCE OF PSYCHOTIC DISORDER? ESTIMATES FROM POPULATION-BASED HEALTH ADMINISTRATIVE DATA FROM ONTARIO, CANADA

Kelly Anderson*[,1], Ross Norman[1], Arlene MacDougall[1], Jordan Edwards[1], Lena Palaniyappan[1], Cindy Lau[2], Paul Kurdyak[2]
*[1]Western University; [2]Institute for Clinical Evaluative Sciences*

**Background:** Recent incidence estimates from population-based health administrative data in Ontario suggest an incidence rate of non-affective psychosis of 55.6 per 100,000 person-years in the general population. However, early psychosis intervention (EPI) programs across the province estimate that the treated incidence of first-episode psychosis is in the range of 12 to 13 per 100,000 per year, which corresponds to frequently cited estimates of the incidence of schizophrenia. This discrepancy between population-based estimates of incidence and the treated incidence reported by EPI programs suggests that there may be additional cases of psychotic disorder receiving services elsewhere in the health care system. Our objective was to estimate the incidence of non-affective psychosis in the catchment area of an EPI program, and compare this estimate to the EPI-treated incidence of psychotic disorder.

**Methods:** We constructed a retrospective cohort of incident cases of non-affective psychosis in the catchment area from 1997 to 2015 using linked population-based health administrative data. Cases were identified by the presence either one hospitalization with a primary discharge diagnosis of non-affective psychosis, or two outpatient physician billings with a diagnosis of non-affective psychosis occurring within a 12-month period. We estimated cumulative incidence proportions of non-affective psychoses for the total sample meeting our case definition using denominator data obtained from the census. Using admission ratios from the EPI program (# admitted/# referred), we correct our population-based incidence estimate to yield an estimated "true incidence" of non-affective psychosis.

**Results:** Reslts: Our case definition identified 2,864 cases of incident non-affective psychosis over the 17-year time-period. We estimate that the “true incidence” of non-affective psychosis in the program catchment area is more than twice as high as the EPI-treated incidence estimates (final numbers forthcoming).
**Discussion:** Our findings suggest that incidence estimates obtained using case ascertainment strategies limited to specialized psychiatric services may substantially underestimate the incidence of non-affective psychotic disorders, relative to population-based estimates. We need accurate information on the epidemiology of psychotic disorders to allow service planners and administrators to more effectively resource EPI services and evaluate their coverage.

### F134. MATERNAL PRENATAL C-REACTIVE PROTEIN AND ADOLESCENT NEURODEVELOPMENTAL OUTCOMES IN THE NORTHERN FINLAND BIRTH COHORT 1986

Hugh Ramsay*[,1], Golam Khandaker[2], Heljä-Marja Surcel[3], Juha Veijola[4]
*[1]University of Oulu, Royal College of Surgeons in Ireland; [2]University of Cambridge; [3]National Institute for Health and Welfare; [4]University of Oulu, Oulu University Hospital*

**Background:** In utero exposure to infections is associated with adverse neurocognitive outcomes in the offspring. Elevated maternal prenatal serum inflammatory markers, such as C-reactive protein (CRP), have been associated with increased risks of neurodevelopmental disorders, including schizophrenia, later in life. The objective of this study is to investigate the associations between elevated serum concentrations of CRP in early gestation, prospectively assayed in maternal sera, and adolescent psychotic experiences and academic performance. We hypothesised that elevated maternal CRP is associated with adolescent psychotic experiences and poorer academic performance.
**Methods:** Using data from the Northern Finland Birth Cohort 1986 (NFBC1986), a prospective birth cohort including data since before birth, we examined the association between maternal CRP levels in early gestation (N=7,600) and adolescent psychotic experiences (n=396/5,071) and poorer academic performance (n=2,324/6,770), controlling for sex and maternal education level using multivariable regression analysis. Prior to analyses we determined there was sufficient power to detect small associations (OR>1.68) for these variables.
**Results:** After controlling for sex and maternal education, those in the highest tertile of prenatal maternal CRP had increased odds of auditory hallucinations (on the PROD-screen) at age 16 years (adjusted OR=1.38, 95% CI: 1.09, 1.74), and poorer school academic performance (beta=-0.08, 95% CI: -0.14, -0.03).
**Discussion:** Maternal prenatal immune activation is associated with neurodevelopmental outcomes in adolescent offspring. This work extends previous findings regarding prenatal/childhood immune activation and clinical psychiatric diagnoses in adult offspring.

### F135. BODY MASS INDEX TRAJECTORIES IN CHILDHOOD AND RISK FOR NON-AFFECTIVE PSYCHOSIS – A GENERAL POPULATION COHORT STUDY

Elina Sormunen*[,1], Maiju Saarinen[2], Raimo K.R. Salokangas[1], Jorma Viikari[2], Olli Raitakari[2], Jarmo Hietala[2]
*[1]University of Turku; [2]University of Turku, Turku University Hospital*

**Background:** It is well known that underweight in adolescence and early adulthood predicts later schizophrenia.[1] Some studies have shown an association between future schizophrenia or psychosis and underweight in children, starting at the age of 7.[2] There are very few previous studies concerning underweight in early childhood and the risk of psychosis as well as other psychiatric outcomes. Our aim was to study whether deviation from normal weight, i.e. underweight or overweight, in early childhood and adolescence predicts later development of non-affective psychosis. And if so, whether the mechanism is specific to psychosis or also predicts other psychiatric disorders.
**Methods:** The participants were derived from a general population cohort study ‘Cardiovascular Risk of Young Finns’, which was started in 1980 with 3596 children and adolescents participating from six different age groups (3–18 years), with a continued follow-up. BMI was recorded before the first hospitalization due to a psychiatric disorder (≤18 years of age) and categorized as underweight, normal weight or overweight using the BMI classification for children and adolescents provided by Cole et al.[3,4] All psychiatric diagnoses of the participants were acquired from the Finnish Hospital Discharge Register. We formed DSM-IV diagnostic groups of non-affective psychosis (n = 70, including a schizophrenia subgroup, n = 41), personality disorders (n = 44), affective disorders (mood- and anxiety disorders, n = 115), and substance-related disorders (n = 53). Participants in the diagnostic groups were compared with subjects with no psychiatric diagnoses (n = 3313). Sex, age, low birth weight and mother’s mental disorders were used as potential confounders in the analyses.
**Results:** Underweight, but not overweight, during the age of 3 to 18 years independently predicted later development of non-affective psychosis. Underweight in childhood and/or adolescence increased the risk of psychosis over two-fold (relative risk (RR) [95% CI] 2.31 [1.2–4.4]). Results were similar for schizophrenia; underweight was associated with nearly 2.5-fold risk of schizophrenia (RR 2.44 [1.03–5.8]). Underweight or overweight in childhood and adolescence was not significantly associated with any other studied psychiatric disorder with a more severe clinical phenotype that required hospital treatment.
**Discussion:** Underweight in childhood and adolescence is an independent risk factor for later non-affective psychosis. The mechanism behind underweight in premorbid phase of psychosis is not known but e.g. low level of insulin-like growth factor-I (IGF-I) may be involved. These results support the hypothesis of schizophrenia as a neurodevelopmental disorder with somatic aspects appearing already in early childhood and psychosis as a late stage of illness.
**References:**
1.Weiser M, Knobler H, Lubin G, et al. Body mass index and future schizophrenia in Israeli male adolescents. J Clin Psychiatry. 2004;65(11):1546–1549. doi:10.4088/JCP.v65n1117.
2.Wahlbeck K, Forsén T, Osmond C, Barker DJP, Eriksson JG. Association of Schizophrenia With Low Maternal Body Mass Index, Small Size at Birth, and Thinness During Childhood. Arch Gen Psychiatry. 2001;58(1):48. doi:10.1001/archpsyc.58.1.48.
3.Cole TJ. Establishing a standard definition for child overweight and obesity worldwide: international survey. Bmj. 2000;320(7244):1240-1240. doi:10.1136/bmj.320.7244.1240.
4.Cole TJ, Flegal KM, Nicholls D, Jackson AA. Body mass index cut offs to define thinness in children and adolescents: international survey. Bmj. 2007;335(7612):194-194. doi:10.1136/bmj.39238.399444.55.

### F136. PARSING DUP TO REFINE EARLY DETECTION: QUANTILE REGRESSION OF RESULTS FROM THE SCANDINAVIAN TIPS STUDY

Maria Ferrara*[,1], Sinan Guloksuz[2], Shadie Burke[3], Fangyong Li[3], Svein Friis[4], Wenche ten Velden Hegelstad[5], Inge Joa[5], Jan Olav Johannessen[5], Ingrid Melle[6], Erik Simonsen[7], Vinod Srihari[3]
*[1]Yale University, AUSL Modena; [2]Academic Hospital Maastricht; [3]Yale University; [4]Oslo University Hospital, Institute of Clinical Medicine, University of Oslo; [5]TIPS – Centre for Clinical*

Research in Psychosis, Stavanger University Hospital; [6]Institute of Clinical Medicine, University of Oslo, Oslo University Hospital, NORMENT KG Jebsen Centre for Psychosis Research, Oslo University Hospital; [7]University of Copenhagen

**Background:** Prolonged duration of untreated psychosis (DUP) is associated with poor outcome. The Scandinavian TIPS study deployed an early detection (ED) campaign to halve DUP. However, while reducing DUP will improve outcomes for most patients, there are some for whom prolonged DUP is a byproduct of an insidiously illness rather than a modifiable prognostic factor. It is also unclear whether the success of an ED program relies on targeting those with longer or shorter DUP. Previously, we demonstrated that quantile regression (QR) can both manage skewed distributions and allow analysis for meaningful heterogeneity in DUP. The current study aims to investigate the utility of QR to analyze the impact of an ED campaign across the DUP distribution, using data from the TIPS study. We hypothesized the effectiveness of TIPS's ED campaign will vary across different quantiles of DUP.
**Methods:** Between 1997 and 2000, a comprehensive early detection (ED) program with public information campaigns and low-threshold psychosis detection teams was established in one health-care area (ED-area), but not in a comparable area (No-ED area). Users with DSM IV non-organic non-affective first episode psychosis were consecutively recruited. Demographic, social and clinical characteristics of people enrolled in an ED area were compared to those coming from a No-ED area. Quantile regression can model the relationship between conditional quantiles of response and independent variables. Unlike ordinary least-squares regression that focuses on conditional mean response, QR estimates the heterogeneous effects of ED across different quantiles of DUP, rather than presuming a uniform mean effect. It is particularly useful when the differential effect of predictors on lower or upper quantile of outcome are of interest. In this study, we examined the impact of ED across the entire quantiles of DUP, particularly on Q1, Q2, Q3, dividing data into four quartiles. A post hoc analysis of the effect of gender, marital status, premorbid adjustment social level, and social cluster on quartiles of DUP was also conducted.
**Results:** The total sample included 301 subjects, of which 161 belonged to an early detection (ED) area. If compared to users from No-ED area, ED users were younger (mean age 25 vs 31), and mainly unmarried (80% vs 62%).
QR highlighted that ED had no effect on the first quartile (Q1) of DUP, with very short DUP, even in the No-ED area. ED was significantly associated with a reduction in the second quartile of DUP (median) (11 weeks reduction, $p<0.001$), and the third quartile of DUP (Q3) (41 weeks reduction, $p=0.01$). The effect of ED was significantly stronger on last quartile than Q1($p=0.01$) and Q2 ($p=0.04$) suggesting a stronger effect of campaign on people with longer DUP.
After controlling for age and marital status, the ED campaign's effect on Q3 of DUP significantly differed by gender: only male users in the ED group showed a significant reduction in this quartile of DUP (coefficient [SE] at Q3=−46.6; $P = .01$), suggesting an interaction between gender and ED campaigns in reducing DUP.
**Discussion:** Quantile regression represents a powerful tool reveal the different effects of an ED campaign across DUP distribution. The upper tail of DUP benefited the most from ED program: users with longer DUP might be hesitant to engage into treatment because of a longstanding active psychosis or failed attempts in the community to receive care. Very short DUP, highly associated with rapidly escalating symptoms, was not affected by the campaign. This could represent a subgroup of patients for which no specific ED efforts are needed or further reductions may need strategies targeting prodromal signs. These findings have been fertile ground for generating hypotheses that could lead to targeted ED efforts.

### F137. INFECTIONS OF THE CENTRAL NERVOUS SYSTEM AS A RISK FACTOR FOR MENTAL DISORDERS AND COGNITIVE IMPAIRMENT: A NATIONWIDE REGISTER-BASED STUDY

Emilie Pedersen*[,1], Ole Köhler-Forsberg[1], Merete Nordentoft[1], Liselotte Petersen[2], Michael Eriksen Benros[1]
*[1]Mental Health Centre Copenhagen, University of Copenhagen; [2]National Centre for Register-based Research, Aarhus University*

**Background:** Infections and inflammatory diseases have long been suggested as risk factors for cognitive decline and mental disorders, most notably schizophrenia and affective disorders. However, largescale studies have been lacking. This study aims to investigate the association between specific CNS-infections and the risk of developing mental disorders, and whether the causal agent of the CNS-infection has an effect on the association. Furthermore, this study will investigate the possible effect of CNS-infections on cognition in the largest study to date.
**Methods:** We will utilize the unique personal registration number to link nationwide Danish registers in order to identify all individuals born in Denmark between January 1, 1977, and December 31, 2010, with follow-up from birth. We will investigate the association between CNS-infections with the risk of 1) developing mental disorders and 2) affected cognition (defined as the highest completed level of education, completion of the 9th grade and grade average score at the end of the 9th grade). Further analyses will estimate the risk within every psychiatric diagnostic category based on the International Classification of Diseases, 10th edition (ICD-10), e.g. organic mental disorders (ICD-10: F00-09), substance abuse disorders (ICD-10: F10-19) and schizophrenia spectrum disorders (ICD-10: F20-29). The risk related to the different pathogens causing the CNS-infection will also be investigated. Data will be analysed using survival analysis to approximate relative risks estimated by Poisson regression, and will be adjusted for age, sex, calendar year, first-born status, parental history of mental disorders and educational level of the parents.
**Results:** All analyses are expected to be completed no later than February 2018 and ready to be presented at the conference in April 2018.
**Discussion:** This population-based cohort study will be the largest to date investigating the association between CNS-infections and mental disorders, and whether there's a difference in risk depending on the pathogen responsible for the CNS-infection. Additionally, it will be one of the largest studies investigating the effect of CNS-infections on cognition. It will add important knowledge to our understanding of the association between CNS-infections and mental disorders, and between CNS-infections and cognition.

### F138. INVESTIGATING A CAUSAL ASSOCIATION BETWEEN NEUROTICISM AND SCHIZOPHRENIA USING TWO-SAMPLE MENDELIAN RANDOMIZATION

Hannah Jones*[,1], George Davey Smith[1], Michael C. O'Donovan[2], Michael J. Owen[2], James Walters[2], Stanley Zammit[3]
*[1]University of Bristol; [2]Cardiff University; [3]University of Bristol, Cardiff University*

**Background:** Anxiety is a prominent feature of schizophrenia, present in the prodromal phase of the illness. There is strong evidence that the personality trait neuroticism, an underlying factor strongly associated with anxiety, is genetically correlated with schizophrenia, implying that neuroticism and schizophrenia share genetic risk factors in common. However, a genetic correlation may also suggest a possible role of neuroticism in the pathogenesis of schizophrenia. We therefore performed a Mendelian randomization (MR) analysis using publicly available data to investigate the potential casual association between neuroticism and schizophrenia.
**Methods:** We performed bi-directional two-sample MR between neuroticism and schizophrenia using the most recent publically available summary-level genome-wide data. Single nucleotide polymorphisms (SNPs) associated with neuroticism ($p \leq 1e-5$) and schizophrenia ($p \leq 5e-8$) were combined using an inverse-variance-weighted (IVW) multiplicative random effects approach. Impact of potential MR assumption violations were explored using weighted median, weighted mode and MR Egger methods. All analyses were performed using the TwoSampleMR R package.

**Results:** The IVW MR method provided strong evidence of a casual effect of genetically instrumented neuroticism on risk of schizophrenia ($p < 0.001$). This causal association was also evident when using the median weighted approach ($p = 0.004$) but evidence was weaker when using the weighted mode ($p = 0.719$) and MR Egger approaches ($p = 0.439$). The MR Egger intercept provided weak evidence of presence of horizontal pleiotropy ($p = 0.067$), however, the I2GX statistic indicated potential violation of the no measurement error MR assumption. There was also evidence of a causal effect of schizophrenia on neuroticism (IVW $p = 0.001$, weighted median $p = 0.017$, weighted mode $p = 0.018$) however, again, the I2GX statistic indicated potential violation of the no measurement error MR assumption.
**Discussion:** Assuming certain MR assumptions are met, our results provide evidence of a bi-directional causal association between neuroticism and schizophrenia suggesting a genetic overlap rather than a uni-directional casual association, however, the impact of feedback loops between exposure and outcome cannot be addressed. Although there was evidence of horizontal pleiotropy between neuroticism and schizophrenia, evidence of violation of the no measurement error indicates that the MR Egger results should be interpreted with caution.

### F139. INCLINATION OF STIGMA TOWARD SCHIZOPHRENIA, ATTENUATED PSYCHOSIS, PSYCHOTIC-LIKE EXPERIENCES AND DEPRESSION AMONG DIFFERENT SUBPOPULATIONS IN TAIWAN

Chen-Chung Liu*[,1], Yen-Chin Wang[2]
[1]*National Taiwan University Hospital and College of Medicine, National Taiwan University;* [2]*National Taiwan University Hospital*

**Background:** Stigma toward mental illness may lead to delayed detection, impaired treatment adherence, and poorer recovery. The fear of being labeled as at risk of psychosis can be barriers to early identification of putative prodromal subjects. Compounded by an intriguing feature, psychotic-like experience with no apparent impact on functioning, we wonder if people will generalize their prejudice and discrimination towards schizophrenia to subjects with subthreshold psychotic symptoms.
**Methods:** A cross-sectional survey using a structuralized questionnaire modified from a previous study conducted by the Hong Kong University Early Psychosis research team was employed. Participants were recruited from various channels; including laypersons in general population invited after their attending talks about mental health topics, patients with mental illness and their key caregivers of 2 hospitals, and mental health professionals. The key component of this questionnaire is comprised by 4 case vignettes describing the symptoms and disabilities of patients with attenuated psychosis syndrome (APS), schizophrenia, depression, and psychotic-like experiences (PLE), respectively; followed by 2 sets of questions using 4-point Likert scale with 19 and 21 items for each set to measure social distance as a proxy for discrimination and prejudices. Basic demographic information, including age, gender, education level, current occupation, and previous contact with persons with mental illness were also collected.
**Results:** A total of 354 subjects completed this survey, including 239 lay publics, 32 psychiatric patients, 29 patient's main caregivers, and 54 mental health professionals. Stigmas, especially prejudice, toward PLE are significantly higher in the patient group compared to the other 3 groups. Prejudice, but not discrimination, toward depression is significantly lower in professionals group. Stigmas toward schizophrenia and APS are in general not significantly different among groups, although the general public showed marginally higher scores in discrimination compared to the patient group. In each individual group, the patterns of attitude reflecting discrimination and prejudice are almost identical; that is, highest toward schizophrenia, followed by APS and depression (almost equivalent to each other), and lowest toward PLE, except the patient group which failed to reveal significant differences in ratings of 4 clinical case vignettes.
**Discussion:** APS seems to be a clinical entity too new to be judged. The majority of participants, except the patient group, reported a similar gradient of stigma toward different clinical severities. Such a result is consistent with previous studies that APS shared similar level of stigma with depression, not as high stigma as towards schizophrenia, but higher than PLE. Interestingly, patients with psychosis might have assimilated PLE to symptoms of schizophrenia based on their personal experiences, so they might have overrated the severity of subjects with PLE.

### F140. HOMICIDES OF PHYSICIANS AND MENTAL HEALTH WORKERS

Michael Knable*[,1]
[1]*Sylvan C. Herman Foundation*

**Background:** Violence towards mental health care workers, and towards physicians in general, is a common occupational hazard. The goal of this work was to determine to what extent violence escalates to actual homicides, both for mental health workers and for physicians in general. Characteristics of the victims, the perpetrators and of the methods of homicide were examined in order to formulate recommendations for violence assessment and safety measures in healthcare settings.
**Methods:** A systematic search for accounts describing homicides of mental health workers between 1981 and 2014, and for physicians between 1981 and 2017 was conducted. Cases of homicides committed by patients, family members of patients, and co-workers of the victims were included. Cases of homicide that occurred in correctional setting, or in agencies not focused on health care (such as child protective services) were excluded. News outlet accounts, internet sources, and the medical literature was searched for details of these cases. Data that were extracted included demographic details on victims and perpetrators, scene and method of homicide, presence of psychiatric diagnoses and prior treatment, and disposition of the perpetrators.
**Results:** Results obtained for mental health workers has been published previously1. Thirty-three homicides of mental health workers were found and examined. Young women caseworkers who were unaccompanied during visits to residential treatment facilities were the most common victims. Men with a diagnosis of schizophrenia were the most common perpetrators. The most likely method of homicide was gunshot. Perpetrators often had a prior history of violence, criminal charges, involuntary hospitalization and nonadherence to medications. Thirty cases of homicides of physicians were found and examined. Psychiatry was the single most likely specialty of the victims (37%). Most homicides occurred in physician offices (33%). The most common psychiatric diagnosis of the perpetrators was schizophrenia (17%), but many other diagnoses were identified, and 33% of perpetrators could not be assigned a diagnosis. The most common method of homicide was again by gunshot.
**Discussion:** Homicides of mental health workers, and of physicians generally, are rare events that emerge from a background of common aggression and violence in healthcare settings. Many of these homicides may have been preventable. Strategies to identify violence risk and to train acute care staff in possible prevention measures, as well as some policy and training measures will be discussed.

### F141. DUTY TO WARN FOR POTENTIAL RISK OF PSYCHOLOGICAL HARM: A CASE REPORT

Naista Zhand*[,1], David Attwood[1]
[1]*University of Ottawa Faculty of Medicine*

**Background:** Under the current ethical and legal standards physicians are expected to breach confidentiality when a third party is at risk. However, the law has mainly focused on risk of bodily harm and there is no legal guidance on physicians' responsibilty when it comes to risk of psychological harm to a potential targeted victim
**Methods:** This case report illustrates a clinical dilemma on duty to warn for mental health professionals.
**Results:** N/A
**Discussion:** This case report illustrates an unconventional perspective on a psychiatrist's duty to warn: consideration of risk of psychological harm to the potential target of their patient. Psychological trauma can have a potential negative impact on victims, affecting their mental health and well being as well as their physical health. The ethicolegal dilemma discussed in this case has implications for policies related to the care of psychiatric patients.

### F142. THE USE OF NEUROIMAGING MARKERS IN STRATIFIED DIAGNOSIS AND THERAPY OF SCHIZOPHRENIC AND AFFECTIVE DISORDERS

Oliver Gruber*,[1]
[1]*University Clinic Heidelberg*

**Background:** Neuroimaging techniques have been developed as important tools to investigate brain dysfunctions that underlie mental disorders. In particular, modern functional magnetic resonance imaging (fMRI) holds the promise to provide neurofunctional biomarkers for improved diagnosis, prognosis, and optimized treatment of schizophrenic and affective disorders.
**Methods:** Neurofunctional connectivity MRI using advanced experimental paradigms permits targeted investigation of the functional integrity of brain systems involved in the pathomechanisms of schizophrenic and affective disorders. From these investigations, pathophysiologically relevant neuroimaging biomarkers can be derived for differential diagnosis and tailored treatment selection.
**Results:** Possible neuroimaging biomarkers will be presented for the prediction of development and clinical course of schizophrenic and affective disorders as well as for the prediction of individual treatment responses. Further, recent neuroimaging findings on possible pathophysiological subtypes of schizophrenic and affective disorders will be discussed.
**Discussion:** These findings from functional neuroimaging studies may help to foster the development of precision medicine in psychiatry.

### F143. PREDICTORS OF RELAPSE: PATIENT, DISEASE, COGNITIVE, AND FUNCTIONAL CHARACTERISTICS WITH COMT GENE VAL158MET POLYMORPHISM IN A 2-YEAR FOLLOW-UP

Anzalee Khan*,[1], Jean-Pierre Lindenmayer[2], Isidora Ljuri[1], Veronica Ozog[3], Amod Thanju[1]
[1]*Nathan S. Kline Institute for Psychiatric Research;* [2]*New York University;* [3]*Teachers College Columbia University*

**Background:** Schizophrenia is a severe and chronic mental illness characterized by continual relapses that may require hospitalization, changes in medications, arrests, emergency room hospitalizations, self-harm or suicidal behavior. Research has shown that costs associated with treatment received following relapse may constitute the largest share of treatment costs psychiatric illnesses. Although, demographic and clinical characteristics associated with relapse have been examined in previous research, information about potential predictors of relapse are limited. The aim of this study was to evaluate the effect of patient and disease characteristics, cognitive, functioning, and COMT gene polymorphism (rs4680) on relapse during 2-year following completion of an inpatient rehabilitation and cognitive treatment.
**Methods:** Data were taken from a COMT genotype and response to cognitive remediation study of schizophrenia in the United States conducted between 07/2005 and 10/2015 for inpatients with schizophrenia who were also participating in psychiatric rehabilitation. Patients with and without relapse 2 years following completion of the study were compared on clinical, demographic, cognitive, functional and COMT genotype characteristics. The COMT gene rs4680 polymorphism was genotyped using a DNA sequence detection system. Relapse or events identified as treatment failures include: arrest, psychiatric re-hospitalization, suicide, discontinuation of antipsychotic treatment due to inadequate efficacy, treatment supplementation with another antipsychotic due to inadequate efficacy, discontinuation of antipsychotic treatment due to safety or tolerability, or increase in the level of psychiatric services. Baseline (end of study, start of 2-year follow-up) predictors of subsequent relapse were also assessed. Univariate Analsyis and Cox's regression was used to examine the effect of potential predictors on outcome.
**Results:** Of 140 subjects with eligible data, 91 (65.00%) relapsed during the 2-year follow-up period. Patients who relapsed were younger (< 45 years), higher number of previous hospitalizations, shorter chronicity of illness (< 10 years), PANSS baseline score of >4 on the core PANSS items (conceptual disorganization, hallucinatory behavior, suspiciousness, unusual thought content), higher negative symptom factor, substance use, PSP score of < 60 and lower MCCB composite T score (> 2 SD below the mean). Univariate analysis shows that COMT rs4680 gene variants were different between relapse and stable groups. The COMT rs4680 gene had an interaction with PANSS baseline core item scores and MCCB composite score. Number of previous antipsychotic trials did not predict relapse.
**Discussion:** There is a high relapse rate within 2 years in chronic schizophrenia. Behavioral symptoms, aided by genetic and environmental factors common to this population (homelessness, unemployment, and social isolation) frequently lead to treatment failure. Knowing potential triggers of relapse can help in developing resources for this population to reduce treatment failures and associated costs.

### F144. MUSCARINIC M1 RECEPTOR SIGNALLING UNDERLYING COGNITION IN PSYCHOTIC DISORDERS

Therese van Amelsvoort*,[1], Geor Bakker[1], Claudia Vingerhoets[1], Barbara J. Sahakian[2], Oswald Bloemen[3], Matthan Caan[4], Jan Booij[4]
[1]*Maastricht University;* [2]*University of Cambridge;* [3]*GGz Centraal, Maastricht University;* [4]*Academic Medical Centre*

**Background:** Antipsychotic treatment has failed to improve cognitive deficits associated with psychotic disorders. This has led to an increased interest to revisit earlier implications from post-mortem studies that lowered muscarinic M1 receptor signaling may underlie these symptoms. This receptor is highly expressed in important regions for cognition such as the dorsolateral prefrontal cortex (DLPFC) and hippocampus, and administration of anti-muscarinic agents gives induce cognitive deficits in healthy volunteers. Administration of xanomeline, a M1/4 agonist in patients with schizophrenia resulted in improved learning and memory scores and decreased psychotic symptom severity. Therefore, the current study sought to examine alterations in muscarinic M1 receptor signaling in relation to cognitive functioning in medication free subjects with psychotic disorders and matched controls.
**Methods:** Muscarinic M1 binding potential (BPND) was measured using single photon emission computed tomography (SPECT) with the M1 selective radiopharmaceutical 123I-iododexetimide in the DLPFC and hippocampus in the psychotic group. Pharmacological functional magnetic resonance imaging (phMRI) with the M1 antagonist biperiden was used to assess differences in functional response on the paired associate learning task (PAL) and emotion recognition task (ERT) adapted for fMRI from The Cambridge Neuropsychological Test Automated Battery in all subjects. The PAL task assessed encoding phase (learning) and retrieval (memory) of figure-place associations and the ERT task social cognition, both highly predictive of functional outcome. Cluster significance was set at Z>2.3, with cluster threshold correction at p<0.05.

**Results:** The current study included 26 (mean age: 27.68; 19male/7 female) subjects with a psychotic disorder and 29 (mean age 25.63; 20 male/9 female) matched controls. Subjects with psychotic disorders recalled less figure place associations than controls (t=2.9, p=0.005) and were worse in recognizing different intensities of disgust emotions (t=2.26, p=0.03). Psychotic subjects showed a blunted response in functional reactivity to biperiden in the bilateral superior and medial frontal gyri with decreasing intensity of disgust facial expressions compared to controls, this blunted response was greatest in those with lower M1 BPND in the DLPFC. During encoding processes, psychotic subjects also showed differential reactivity to biperiden in the left middle frontal gyrus, insula, and caudate nucleus showing hypoactivation compared to controls. Greater hypoactivation was significantly associated with lower hippocampal M1 BPND. For retrieval both groups showed lowered activation under biperiden in the inferior frontal gyrus, but psychotic subjects failed to show increased activation with increasing cognitive load in the placebo condition, like the controls. Lower hippocampal M1 BPND in psychotic subjects was associated with lower activation of this region.

**Discussion:** Results show preliminary evidence for altered M1 signaling of prefrontal areas in psychotic disorders underlying social cognition and learning and memory processes. Additionally, results show an important role for the M1 receptor in the DLPFC and hippocampus in altered fronto-striatal activation underlying encoding processes. Lower hippocampal M1 BPND is related to more severe alterations in underlying functional activation in encoding and retrieval processes. Results further support the need for development of therapeutic strategies that focus on the M1 receptor to improve cognitive functioning and functional outcome in psychosis.

### F145. WHAT ARE THE MAIN BRAIN CHANGES IN FMRI AFTER TREATMENT IN FIRST EPISODE PSYCHOSIS? A SYSTEMATIC REVIEW

Carlos Gonzalez[1], Olga Sparano[2], Pau Soldevila-Matias[1], Gracian Garcia-Marti[3], Luis Marti-Bonmati[4], Benedicto Crespo-Facorro[5], Julio Sanjuan*[,6]

[1]*Clinic Hospital INCLIVA;* [2]*Ribera Hospital;* [3]*Quiron Hospital;* [4]*La Fe Hospital, Quiron Hospital;* [5]*Marques de Valdecilla Hospital, Santander;* [6] *University of Valencia*

**Background:** There are many studies using structural MRI to explore the longitudinal course of F Episode Psychosis (FEP).[2] On the other hand, there is a lack of functional MRI studies examining the longitudinal course of FEP. The aim of this work is to make a literature systematic review of these studies, to summarize the knowledge about longitudinal course of functional brain activity in FEP.

**Methods:** We followed the PRISMA guidelines for conducting systematic reviews and combined the use of electronic and manual systematic search methods, in the principal databases (MedLine, PubMed and Web of Science) using the query "longitudinal" AND "fMRI" AND "first episode psychosis" OR "first episode schizophrenia". This search included (PERIODO). The inclusion criteria were: a) FEP diagnose; b) at least 2 functional MRI scans (pre-post); c) both task and resting-state scans were included. The exclusion criteria were: a) chronic patients in the studied sample; b) structural imaging results; c) just 1 fMRI scan; d) reviews and metaanalysis.

**Results:** 202 records were identified through database searching. A total of 10 articles were selected. From them, a total of 276 FES patients were examined by fMRI. In all of these studies patients were diagnosed by structured interviews according to DSM-IV-TR or ICD-10 criteria. The average age of the FES sample was 26.64 years old. Nine of the 10 studies involved 2 scans with a mean interval between them of 7 months. Six of the 10 studies did the first scan without any antipsychotic treatment, but all of them had medication at follow-up scan. Most of the studies used a region of interest (ROI) approach, and examined the role mainly of these areas: limbic system, hippocampus, striatum and prefrontal cortex.

Five of the studies used a resting-state paradigm. The other 5 works implemented some cognitive or emotional task using some visual stimuli.

Attending to the imaging results at baseline, most of studies found an hypoactivation of several brain areas, specially the limbic areas, like thalamus, amygdala and hippocampus. There are some other areas less activated compared to controls, including striatum, anterior cingulated cortex, orbitofrontal cortex, temporal gyrus and cerebellum posterior lobe.

At follow-up, almost all studies reported normalization of the hypoactivation levels found at baseline in the same regions. When the results at baseline were an increased activation, it also normalized at follow-up. There is only one study reporting an increased activity at baseline comparing to healthy volunteers which is still increased at follow-up scans.

**Discussion:** There are very few studies exploring fMRI longitudinal course of FEP patients.

Our results in FEP are similar with other recent reviews in chronic schizophrenia samples,[1] finding normalization (increase) of brain activity after antipsychotic treatment.

There are only visual or resting-state paradigms during scanning, which could explain some of the results. More investigations, involving other paradigms and related with psychopathological changes are needed, to test how the brain of the FEP patients change over time.

**References:**

1. Kani, A. S. (2017). Converging effects of diverse treatment modalities on frontal cortex in schizophrenia: A review of longitudinal functional magnetic resonance imaging studies. Journal of Psychiatric Research, 84, 256–276. J.JPSYCHIRES.2016.10.012

2. Gong, Q. (2016). A selective review of cerebral abnormalities in patients with first-episode schizophrenia before and after treatment. American Journal of Psychiatry, 173(3), 232–243.

### F146. S-KETAMINE-INDUCED NMDA RECEPTOR BLOCKADE DURING NATURAL SPEECH PRODUCTION AND ITS IMPLICATIONS FOR FORMAL THOUGHT DISORDER IN SCHIZOPHRENIA: A PHARMACO-FMRI STUDY

Tilo Kircher*[,1], Arne Nagels[1]

[1]*Marburg University*

**Background:** FTD is a dimensional, phenomenologically defined construct, which can clinically be subdivided into positive (pFTD) versus negative (nFTD) as well as objective versus subjective symptom clusters. Structural and functional changes in the lateral temporal language areas have been related to formal thought disorder (FTD) in schizophrenia. Continuous, natural speech production activates the right lateral temporal lobe in schizophrenia, as opposed to the left in healthy subjects. Positive and negative FTD can be elicited in healthy subjects by glutamatergic NMDA blockade with ketamine. It is unclear, whether the glutamate system is related to the reversed hemispheric lateralization during speaking in patients.

**Methods:** In a double-blind, cross over, placebo-controlled study, 15 healthy, male, right-handed volunteers overtly described 7 pictures for 3 minutes each, while BOLD signal changes were acquired with fMRI. As a measure of linguistic demand, the number of words within 20-second epochs was correlated with BOLD responses.

**Results:** Participants developed S-ketamine-induced psychotic symptoms, particularly positive FTD. Ketamine vs. placebo was associated with enhanced neural responses in the right middle and inferior temporal gyri.

**Discussion:** Similar to a previous fMRI study in schizophrenia patients vs. healthy controls applying the same design, S-ketamine reversed functional lateralization during speech production in healthy subjects. Results demonstrate an association between glutamatergic imbalance, dysactivations in lateral temporal brain areas, and FTD symptom formation. Left superior temporal gyrus (STG) cortical volume is decreased in schizophrenia patients (SZ) with pFTD in structural magnetic resonance imaging (sMRI) studies and shows reversed activation in functional MRI (fMRI) experiments during speech production. pFTDs are related to synaptic rarefication in the glutamate system of the superior and middle lateral temporal cortices.

**References**:

1. Nagels A, Cabanis M, Oppel A, Kirner-Veselinovic A, Schales C, Kircher T. Neuropsychopharmacology. 2017 Nov 6. doi: 10.1038/npp.2017.270.

2. Kircher T, Liddle PF, Brammer MJ, Williams SC, Murray RM, McGuire PK. Reversed lateralization of temporal activation during speech production in thought disordered patients with schizophrenia. Psychol Med. 2002 Apr;32(3):439–49.

3. Kircher T, Bröhl H, Meier F, Engelen J. Formal Thought Disorders (FTD): From Phenomenology to Neurobiology. Lancet Psychiatry, in press.

### F147. RESTING STATE NETWORKS ALTERATION IN PANTOTHENATE-KINASE ASSOCIATED NEURODEGENERATION (PKAN)

Gianluca Mingoia*[,1], Peter Stoeter[2], Rea Rodriguez-Raecke[3]
*[1]RWTH Aachen University; [2]CEDIMAT; [3], Uniklinik RWTH Aachen*

**Background:** While functional MRI and PET studies have shown altered task-related brain activity in PKAN, we want to find such differences also in the resting state (RS).

Here we used ICA based analysis to investigate RS fMRI data to compare connectivity of 11 well known networks (Auditory, Cerebellum, Default Mode Network (DMN), Exectutive Control, Fronto-parietal 1, Fronto-parietal 2, Salience, Sensorimotor, Visual1, Visual2, Visual3 network) between patients with PKAN and healthy controls suggesting deficits in related neuropsychological functions.

**Methods:** We obtained RS fMRI series (3T, 3x3x3mm resolution, 45 slices, TR 2s, 300 volumes) in 17 PKAN patients but 3 were discarded because of excessive movement, (mean age 17.2a±7.1) on stable medication and 15 healthy controls (22.5a±8.3).

Subjects were asked to lie in the scanner keeping eyes closed with no further specific instructions. Data were pre-processed; we applied FSL MELODIC (pICA) yielding IC, we used FIX to auto-classify ICA components which represent artifacts and an automated routine to select for each subject the component matching the anatomical definition of resting state networks.

SPM12 was used for second level analysis, we used two sample t-test to compare networks functional connectivity between groups. In addition, we used multiple regression to correlate RS networks activity components with Dystonia score.

**Results:** Our method reliably identified all networks in every control and patients. We found significant differences in the anatomical pattern of areas. Patients showed decreased functional connectivity in comparison to healthy controls in portions of Fronto-parietal 1, Fronto-parietal 2 and Visual1 networks; in addition, patients showed increased functional connectivity in comparison to healthy controls in portions of Cerebellum, DMN, Executive Control, Salience and Visual1 networks. Finally, significant correlation was found between dystonia score and functional connectivity of Cerebellum, Fronto-parietal1, Fronto-parietal2, Salience, Sensorimotor and Visual2 networks.

**Discussion:** Well known resting state networks were reliable identified from RS fMRI in PKAN patients. The differences in anatomical distribution point to possible alterations in functional connectivity in PKAN, which suggests disruption in cerebellum, DMN, fronto-parietal, salience and visual activity. Correlations with dystonia suggest a direct relation to motor items, which would support a clinical significance of altered RS networks activity.

### F148. A PILOT STUDY OF [11C] (R)-MEQAA PET BRAIN IMAGING ANALYSIS OF ALPHA 7 NICOTINIC ACETYLCHOLINE RECEPTORS AVAILABILITY IN SCHIZOPHRENIA

Tomoyasu Wakuda*[,1], Masamichi Yokokura[1], Kyoko Nakaizumi[1], Yasuhiko Kato[1], Yosuke Kameno[1], Masami Futatsubashi[2], Etsuji Yoshikawa[2], Yasuhiro Magata[1], Yasuomi Ouchi[1], Hidenori Yamasue[1], Nori Takei[3]
*[1]Hamamatsu University School of Medicine; [2]Hamamatsu Photonics KK; [3]Hamamatsu University School of Medicine, Institute of Psychiatry*

**Background:** A growing body of evidence suggests that the aberrant cholinergic system may underlie the pathophysiology in schizophrenia. Nicotinic acetylcholine receptor (nAChR) subtype α7 (henceforth 'α7 nAChR') is located in presynaptic and postsynaptic constructs in the cerebral cortex and considered to play a key role in the regulation of learning and memory. Additionally, α7 nAChR is deemed to exert neuroprotective effects. Therefore, α7 nAChR is one of the potent therapeutic targets for negative symptoms and cognitive impairment in schizophrenia. In effect, several randomised trials to assess the efficacy and safety of α7 nAChR agonists are currently underway.

There is some evidence in support of aberrant α7 nAChR in schizophrenia. In postmortem studies, protein levels of α7 nAChR in the frontal cortex (Guan et al., 1999) have been reported to be decreased in patients with schizophrenia. However, the availability of α7 nAChR in individuals with schizophrenia has yet to be examined in vivo. In this pilot study, we aim to clarify availability of α7 nAChR in the brains of patients with schizophrenia using positron emission tomography (PET) with a ligand of [11C] (R)-2-methylamino-benzoic acid 1-aza-bicyclo[2.2.2]oct-3-yl ester ([11C] (R)-MeQAA).

**Methods:** All participants provided informed consent. Inclusion criteria included diagnosis of schizophrenia according to the Diagnostic and Statistical Manual of Mental Disorders-Fifth Edition (DSM-5, 2013). Patients were excluded if they had (1) full IQ under 69 measured with the Wechsler Adult Intelligent Scale-III; (2) current or past history of tobacco smoking; (3) history of neurological disorder or structural brain abnormality; (4) use of benzodiazepines, antidepressants, or anticholinergics in the past 6 months; and (5) substance abuse. Although scanning drug-free or drug-naïve patients for investigation is optimal, it is extremely difficult to attain this. Consequently, participants with schizophrenia comprised medicated cases.

We evaluated the availability of α7 nAChR by estimating non-displaceable binding potential (BPND) of the tracer using PET with [11C] (R)-MeQAA, a selective PET tracer for α7 nAChR. Four patients with schizophrenia (age: range 27–39; m/f: 2/2) and 5 age-matched healthy adults (age: range 22–32; m/f: 2/3) underwent the PET scan. The level of BPND in patients with schizophrenia was compared with that for control participants by applying regions of interest (ROIs) approach. In this pilot study, we opted for 4 cortical areas, the superior frontal, middle frontal, parietal, and temporal cortices, for ROIs. This study was approved by the Hamamatsu University School of Medicine Ethics Committee.

**Results:** We found the levels of [11C] (R)-MeQAA BPND significantly lower in the middle frontal cortex (p = 0.036) in patients with schizophrenia. Additionally, there was a trend towards a decreased level of BPND in the temporal cortex (p = 0.067) and parietal cortex (p = 0.087) in the brains of schizophrenia patients, although it failed to reach statistical significance. There was no difference in the superior frontal cortex.
**Discussion:** To our knowledge, this represents the first demonstration of anomalies in the acetylcholinergic system in the in vivo brains of schizophrenia patients. However, this is regarded as a pilot study, and further recruitment of schizophrenia patients with a recent onset and minimal use of antipsychotic medication, followed by scanning and data analyses, will be continued.

### F149. NEUROBIOLOGY OF SELF-AGENCY DURING REALITY MONITORING AND SPEECH FEEDBACK MONITORING: IMPLICATIONS FOR TREATMENT DEVELOPMENT IN SCHIZOPHRENIA

Karuna Subramaniam*[,1], Leighton Hinkley[1], Hardik Kothare[1], Danielle Mizuiri[1], Sophia Vinogradov[2], John Houde[1], Srikantan Nagarajan[1]
*[1]Univeristy of California, San Francisco; [2]University of Minnesota*

**Background:** Self-agency is the experience of being the agent of one's own thoughts and motor actions. The intact experience of self-agency is necessary for successful interactions with the outside world (i.e., reality monitoring). Reality monitoring is the ability to distinguish internally self-generated information from outside reality (externally-derived information). We found that healthy control (HC) participants recruit medial prefrontal cortex (mPFC) during encoding of self-generated information, which is also activated during accurate retrieval of self-generated information. By contrast, patients with schizophrenia (SZ) have specific self-agency impairments and do not show mPFC activation during encoding or retrieval of self-generated information. These findings indicate that SZ may rely more on environmental externally-derived information, rather than on internal self-generated information, to guide reality monitoring. Here, we relate the experience of self-agency during a lower-level speech feedback monitoring (i.e., monitoring what we hear ourselves say) to our higher-level cognitive reality monitoring task. We examine whether the sense of self-agency during speech feedback monitoring and reality monitoring are driven by the same fundamental mechanism that we hypothesize underlies the capacity to experience self-agency—the ability to make reliable predictions about the outcomes of self-generated actions.
**Methods:** During speech feedback monitoring we assess self-agency by altering environmental auditory feedback so that participants listen to a perturbed version of their own speech. When subjects hear minimal perturbations in their auditory feedback while speaking, they make corrective responses, indicating that they judge the perturbations as errors in their speech output. These corrective responses are modulated by subjects' reliance on internal predictions about the outcome of their speech output; the more subjects rely on their internal predictions, i.e. their sense of self-agency, the less they rely on external auditory feedback, resulting in smaller corrective responses. Thus, subjects who produced smaller corrective responses manifested an enhanced sense of self-agency in that they relied more on their internal predictions to generate their own actions (i.e., their speech output).
**Results:** We found that self-agency judgments in the reality-monitoring task were higher in people who had smaller corrective responses (p=.05) during minimal speech perturbations of their auditory feedback. These results provide support for a unitary process for the experience of self-agency resulting from the ability to reliably predict the outcomes of self-generated actions, that governs low-level speech control and higher level reality monitoring.
**Discussion:** These findings have important therapeutic implications in SZ, suggesting that the more participants rely on internal predictions to guide their actions, the smaller their corrective responses in their speech output, and the more likely they are to make correct judgments of self-agency during reality monitoring. In conclusion, these findings, therefore, indicate that reality monitoring and speech monitoring paradigms provide quick and robust markers of the experience of self-agency, indicating which subjects followed their internal predictions to guide their own actions. Together, these findings have important therapeutic implications for potentiating improvements in self-agency judgments not only in HC, but in patients with schizophrenia who suffer from critical self-agency impairments.

### F150. OVERESTIMATING ENVIRONMENTAL VOLATILITY INCREASES SWITCHING BEHAVIOR AND IS LINKED TO ACTIVATION OF DORSOLATERAL PREFRONTAL CORTEX IN SCHIZOPHRENIA

Lorenz Deserno[1], Rebecca Boehme[2], Christoph Mathys[3], Teresa Katthagen[4], Jakob Kaminski[4], Klaas Enno Stephan[5], Andreas Heinz[4], Florian Schlagenhauf*[,4]
*[1]Max Planck Institute for Human Cognitive and Brain Sciences; [2]Center for Social and Affective Neuroscience, Linköping University; [3]Scuola Internazionale Superiore di Studi Avanzati (SISSA) [4]Charité – Universitätsmedizin Berlin; [5]University of Zurich & ETH Zurich*

**Background:** Reward-based decision-making is impaired in schizophrenia, as reflected by increased switching between choices. The underlying cognitive mechanisms and associated neural signatures remain unknown. Reinforcement learning (RL) and hierarchical Bayesian learning account for this behavior in different ways. We hypothesized that enhanced switching during flexible reward-based decision-making in schizophrenia relates to higher-order beliefs about environmental volatility and examined the associated neural signatures.
**Methods:** 46 medicated schizophrenia patients and 43 controls underwent a reward-based decision-making task requiring flexible behavior to changing action-outcome contingencies during functional Magnetic Resonance Imaging (fMRI). Computational modeling of behavior was performed, including RL and the Hierarchical Gaussian Filter (HGF). The estimated learning trajectories informed the analysis of fMRI data.
**Results:** A three-level HGF accounted best for the observed choice data and revealed a heightened prior belief about environmental volatility and a stronger influence of volatility on lower-level learning of action-outcome contingencies in schizophrenia. This finding was replicated in an independent sample of unmedicated patients. Beliefs about environmental volatility were reflected by higher activity in dorsolateral prefrontal cortex (dlPFC) of patients compared to controls.
**Discussion:** This study suggests a mechanistic explanation for instable behavior in schizophrenia: patients inferred the environment as being too volatile and thus overestimated environmental changes, leading to maladaptive choice switching. Our data suggest enhanced dlPFC activity related to beliefs about environmental volatility as a neural learning signature of instable behavior. Such detailed 'computational phenotyping' may provide useful information to dissect clinical heterogeneity and could improve prediction of outcome.

### F151. A SYSTEMATIC REVIEW OF TASK-BASED FUNCTIONAL NEUROIMAGING STUDIES IN THOUGHT DISORDER

Philip Sumner*[,1], Imogen Bell[1], Susan Rossell[2]
*[1]Swinburne University of Technology; [2]Monash Alfred Psychiatry Research Centre, The Alfred Hospital and Monash University, Central Clinical School, Swinburne University of Technology, The University of Melbourne, St. Vincent's Hospital*

**Background:** Thought disorder (TD) is an important symptom that demonstrates familial aggregation, predicts conversion to psychosis in those at risk, and predicts the duration and rate of hospitalisation in those with psychosis. However, the aetiology of TD is debated, with theoretical accounts revolving around executive, language, and semantic impairments. The aim of the current systematic review was to synthesise the research that has investigated TD using task-based functional neuroimaging techniques to target executive, language, or semantic functions.
**Methods:** The literature search was conducted in accordance with the Preferred Reporting Items for Systematic Reviews and Meta-Analyses (PRISMA) statement. The databases: PubMed, Scopus, and Web of Science were used to locate relevant literature from January 1990 to August 2016. The search strategy was broad and inclusive to capture exploratory and secondary TD-related analyses.
**Results:** The search yielded 5821 records, from which 37 pertinent studies were identified. Functional correlates of TD included the superior and middle temporal, fusiform, and inferior frontal gyri bilaterally, as well as the left and right cingulate cortex, the right caudate nucleus, and the cerebellum. TD-related increases and decreases in activation were both evident in most of these regions. However, the specificity of these correlates from general clinical and cognitive influences, as well as the relationships between task-based function and behavioural performance, are currently unknown.
**Discussion:** The cortical regions implicated overlap with those thought to contribute to language and semantic systems. Cortico-striatal circuitry may additionally play a role in some aspects of TD through aberrant salience representation and inappropriate attentional prioritisation. To advance the field further, greater integration across structural, functional, and behavioural measures is required, in addition to non-unitary considerations of TD and more thorough investigations of component cognitive processes.

### F152. N-ACETYL-CYSTEINE SUPPLEMENTATION IMPROVES FUNCTIONAL CONNECTIVITY IN THE CINGULATE CORTEX IN EARLY PSYCHOSIS

Emeline Mullier[1], Timo Roine[2], Alessandra Griffa[3], Philipp S. Baumann[2], Paul Klauser*[,1], Philippe Conus[2], Kim Q. Do[1], Patric Hagmann[1]
*[1]Lausanne University Hospital; [2]Centre Hospitalier Universitaire Vaudois (CHUV), University of Lausanne (UNIL); [3]The Dutch Connectome Lab*

**Background:** In schizophrenic patients, increasing evidence suggests that oxidative stress is involved on the disease pathophysiology. Estimation of the level of glutathione (GSH), main actor of the brain redox dysregulation, has revealed a decreased GSH levels in early psychosis patients (EPP). N-Acetyl-Cysteine (NAC) is an antioxidant and precursor of GSH, almost devoid of side effects. In chronic patients, add-on of NAC improved negative symptoms and reduced side effects of antipsychotics. In a recent double-blind randomized placebo-controlled trial, early psychosis patients received NAC supplementation. Whether NAC treatment leads to improvement in FC in EPP is unknown. Given the known connectivity disruptions in schizophrenia and given that GSH levels correlated with FC within the cingulate cortex, we investigate the effect of NAC supplementation during 6 months in EPP on cingulum cortex FC.
**Methods:** 20 EPP which constitutes a subgroup of the double-blind randomized placebo-controlled trial by [7] were scanned. T1-weighted volumes (1 mm in-plane, 1.2 mm slice thickness, TR/TE/TI 2300/2.98/900ms) and resting-state fMRI (gradient-echo-EPI sequence, 3.3x3.3x3.3 mm3, TR/TE 1920/30ms) recording were acquired on a Siemens-3T scanner in EPP who received either NAC (n=9) or placebo (n=11) as add-on treatment at baseline (T1) and follow-up (T2) after 6 months. A control group (n=93) was scanned in the same conditions. Data were processed with CMTK using a 68 regions parcellation according to a state-of-the-art pipeline. Time series were averaged over the Freesurfer regions and Pearson's correlation was used as FC measure. We investigated FC changes occurring in the cingular cortices between T1 and T2 for NAC and placebo EPP and between EPP and CTRL by looking at the FC strength of these regions. Moreover, we assessed global efficiency and edge betweenness centrality (BC) for the whole brain network.
**Results:** FC between caudal and isthmus cingulate cortices increases significantly after 6 months for patients who received NAC supplementation (p=0.01) but not for patients who received the placebo. FC of these regions was also higher in patients after 6 months of NAC supplementation (but not in placebo or baseline patients). Regarding the local network measures, BC differences are observed for the same regions. Indeed, edge BC increases between caudal and isthmus cingulate regions after NAC supplementation compared to placebo (p=0.015). At the nodal level, the increase of BC can be mainly ascribed to the isthmus cingulate cortex (p=0.01).
**Discussion:** NAC supplementation has an impact on FC between caudal and isthmus cingulate regions in our dataset. NAC increases FC strength between these two regions. This strength is significantly higher than for matched control subjects. NAC has a stimulating effect on FC in regions along the cingulum bundle. This FC change could be partially explained by the changes in BC. The edge BC of the caudal and isthmus cingulate connection is increased for NAC subjects: a highest number of shortest paths go through this connection. It shows a growing importance of this edge in connecting the frontal and posterior regions of the brain network. A higher number of subjects would allow to confirming the robustness of these findings. This is a first study assessing the impact of NAC treatment on FC in a sample of EPP. After 6 months, NAC improves FC in caudal anterior and isthmus cingulate cortices. This FC increase is characterized by a higher BC in these brain areas, improving the connection between the frontal and posterior regions along the cingulum bundle. These results suggest that FC along the cingulum may be a biomarker for NAC treatment response.

### F153. NEUROMAGNETIC MISMATCH NEGATIVITY IN CLINICAL HIGH RISK AND FIRST-EPISODE PSYCHOSIS INDIVIDUALS

Emmi Mikanmaa*[,1], Marc Recasens[1], Hanna Thuné[1], Tineke Grent-'t-Jong[1], Andrew Gumley[1], Matthias Schwannauer[2], Stephen Lawrie[3], Ruchika Gajwani[1], Joachim Gross[1], Peter Uhlhaas[1]
*[1]University of Glasgow; [2]University of Edinburgh; [3]Royal Edinburgh Hospital*

**Background:** Auditory mismatch negativity (MMN) and its magnetic counterpart (MMNm) are event-related potentials/fields elicited by violations of previously established auditory regularities (Näätänen et al. 2007). Reduced MMN amplitude is a robust finding in both medicated and unmedicated schizophrenia (ScZ) patients (for a review, see Umbricht & Krljes, 2005), potentially indicating impaired predictive processes (Sauer et al. 2017). Furthermore, several studies have examined MMN responses in individuals at clinical high risk for psychosis (CHR) and have found MMN deficits to be present already in this population, indicating that MMN is compromised before psychosis onset and could represent a marker of risk for psychosis development. However, not all studies have reported significant differences in MMN amplitude between clinical high risk and healthy individuals and there is evidence to suggest that MMN deficits are primarily observed in CHR individuals who transition to psychosis (Bodatsch et al., 2015).

**Methods:** The purpose of the present study is to investigate MEG recorded MMNm amplitude responses to both duration deviants and sound omissions in n = 90 CHR-individuals who were screened with the Schizophrenia Proneness Instrument and the Comprehensive Assessment of At Risk Mental States. N = 50 healthy controls served as a comparison group. We employed an auditory oddball paradigm in which three different sequences of auditory stimuli were presented binaurally at ~ 70 dB (150 ms SOA, 700 – 100 ms ISI). The standard sequence contained five identical tones (80 ms) and the deviant sequence contained four identical tones and one duration deviant tone (40 ms). In addition, an omission sequence which contained only four identical tones was included to examine auditory predictions. Participants were instructed to focus their attention away from the sounds and to perform a simple visual detection task. We analyzed MMNm responses to unpredictable deviant and omitted sounds in bsensor and source space. The linearly constrained minimum variance (LCMV) beamformer was used to identify the generators of the MMNm responses and to compute artefact-free source-level time-courses.

**Results:** Consistent with previous studies revealing MMN sources in auditory cortical areas, bilateral MMNm sources were localized in auditory cortices for both duration deviants and sound omissions. Our data show attenuated MMNm responses elicited by duration deviant sounds in CHR individuals compared to healthy controls.

**Discussion:** Taken together, the study findings suggest that reduced MMNm amplitude is present before psychosis onset and reflects aberrant predictive processing rather than deficient stimulus-specific adaptation. Clinical follow-up measurements will be obtained to determine whether MMNm amplitudes are a potential biomarker for predicting onset of psychosis.

### F154. ABERRANT SALIENCE NETWORK FUNCTIONAL CONNECTIVITY IN AUDITORY VERBAL HALLUCINATIONS: A FIRST EPISODE PSYCHOSIS SAMPLE

Paris Alexandros Lalousis*[,1], Pavan Mallikarjun[1], Thomas Frederick Dunne[1], Kareen Heinze[1], Renate Reniers[1], Matthew R. Broome[2], Baldeep Farmah[3], Femi Oyebode[1], Stephen Wood[4], Rachel Upthegrove[1]

*[1]University of Birmingham; [2]University of Oxford; [3]Forward Thinking Birmingham; [4]Orygen, the National Centre of Excellence in Youth Mental Health*

**Background:** Auditory verbal hallucinations (AVH) often lead to distress and functional disability, and are frequently associated with psychotic illness. Theories of abnormal integration have been proposed to explain symptoms of schizophrenia, including delusions and hallucinations, with a central abnormality being aberrant activity in intrinsic brain networks such as the default mode network (DMN) or the salience network (SN). Previous investigations of patients with schizophrenia assessing functional connectivity (FC) have used a seed-based functional connectivity approach (sb-FC), with seed placement in brain areas responsible for auditory processing, language, and memory; the striatum, and in areas of DMN. These have generated some conflicting results, possibly because of the varying seed placement. The aim of the current study was to address these confounding factors by investigating the intrinsic FC in first episode psychosis (FEP) patients with AVH using within-sample AVH symptom capture seeds. It was hypothesised that patients would show aberrant resting state FC between areas of the DMN and SN and these areas.

**Methods:** Eighteen FEP individuals and 20 healthy controls were recruited. All the participants underwent resting-state functional Magnetic Resonance Imaging (rs-fMRI). The Data Processing Assistant for Resting-State fMRI Advanced Edition (DPARSFA) V3.1 (http://rfmri.org/DPARSF) (Yan & Zang, 2010) and the statistical parametric mapping software 8 (SPM8) (SPM, Friston, The Wellcome Department of Cognitive Neurology, London, Uk; http://www.fil.ion.ucl.ac.uk/spm) were used to preprocess and analyze the data.

**Results:** Patients showed increased FC between left insula and bilateral cerebellum, and angular gyrus; and increased FC between left claustrum and left cerebellum and postcentral gyrus. There was reduced FC in FEP patients with AVH between left claustrum and left insula compared to HC. The FC between left insula and left claustrum seeds for patients and HC is shown separately in supplementary information. There were no significant correlations between DUP, dose of antipsychotic medications, and severity of hallucinations and the mean coefficients of clusters that were significantly different between FEP patients and HC.

**Discussion:** FEP patients showed increased functional connectivity between left insula and bilateral cerebellum and angular gyrus; and increased functional connectivity between left claustrum and left cerebellum and postcentral gyrus. We also found reduced functional connectivity between left claustrum and left insula in FEP patients compared to HC. It is possible the pathology of AVH is primarily located in the insula and angular gyrus. However, given our results of both the left insula seed in patients and HC shows connectivity with right insula and anterior cingulate cortex (key regions of SN) and literature from patients with chronic AVH, the suggestion may be that resting state dysconnectivity within the DMN and SN are implicated in the generation of AVH, which during the experience itself will further involve temporal and auditory networks. Furthermore, decreased intrinsic functional connectivity between the claustrum and the insula may lead to compensatory over activity in parts of the auditory network including areas involved in DMN, auditory processing, language and memory, leading to the complex and individual content of AVH when they occur.

### F155. THE NEUROPHYSIOLOGICAL AND BEHAVIOURAL EFFECTS OF TRANSCRANIAL DIRECT CURRENT STIMULATION ON WORKING MEMORY AND EXECUTIVE FUNCTIONING IN SCHIZOPHRENIA

Natasza Orlov*[,1], Owen O'Daly[1], Derek Tracy[1], John Rothwell[2], Sukhi Shergill[3]

*[1]King's College London; [2]University College London; [3]Cognition Schizophrenia and Imaging (CSI) Lab*

**Background:** Individuals with schizophrenia typically suffer a range of cognitive deficits, including in executive functioning (EF) and working memory (WM) [1,2]. Such difficulties are strongly predictive of functional outcomes, but there is a lack of effective therapeutic interventions [3].

Transcranial direct current stimulation (tDCS) is a novel neuromodulatory technique with emerging evidence of potential pro-cognitive applications; however there has been a dearth of understanding of mechanistic effects of this intervention [4].

The aim of this study was to evaluate the neurophysiological effects of tDCS during WM and EF assessment in individuals with schizophrenia.

**Methods:** We utilized functional magnetic resonance imaging (fMRI) to evaluate the impact of tDCS on WM and EF in individuals with schizophrenia, randomized to receive either 'real' or 'sham' (placebo tDCS). Participants completed a WM (blocked 0–3 back) and an EF (color-word Stroop) during 30 min of 2mA tDCS applied to Broadman area 10/46 (anode 35cm2); with cathode placed on right supraorbita. Sham stimulation was applied for 30 sec. tDCS was applied during fMRI (online) [5].

In addition to whole brain, we also conducted task relevant region of interest analysis (ROI) to compare mean frontal and prefrontal (Broadman are 10/46 mask) and anterior cingulate cortex (ACC) activity between the two groups during tDCS. All analyses were restricted a p-value of 0.05, following family wise-error correction (FWE).

**Results:** There were no between-group differences in socio-demographic or clinical characteristics (Tab 1.) Participants did not differ on WM task performance during online tDCS (Tab 2). However, there were significant between-group differences in manipulation of information with the real tDCS performing significantly better than sham, controlled for baseline (b=0.68, CI 0.14 - 1.21; p=0.044) after consolidation [6].
During WM the ROI analysis demonstrated increased activation underneath the site of the anode in the medial frontal gyrus in the real tDCS group, as compared to sham. There was a positive correlation between with consolidated performance and the activity in the medial frontal gyrus.
Further, tDCS demonstrated significantly reduced activation in the left cerebellum.
During EF task, increased performance was associated with decreased activity in the ACC [5].
**Discussion:** This is the first tDCS study to examine the brain activity during WM and EF assessment in individuals with schizophrenia using fMRI This data suggests that biasing the membrane potential of neuronal populations in the frontal cortex seems to improve their response to other inputs i.e. decreased BOLD activation in the WM and EF network. Although the mechanism of action of tDCS is not clear yet [6], one may speculate that if the BOLD response represents synaptic activity [7], including input from other areas, then tDCS might increase the probability that a synaptic input will generate a response in an output neuron, without the need of any additional energy expended by the cell. tDCS offers a potential new therapeutic approach to the treatment of cognitive deficits.

### F156. LONGITUDINAL WORKING MEMORY FUNCTIONAL DYSCONNECTIVITY REFLECTS HETEROGENEITY IN INDIVIDUALS AT ULTRA HIGH RISK FOR PSYCHOSIS

Siwei Liu*[,1], Jimmy Lee[2], Jesisca Tandi[3], Chenhao Wang[3], Joseph K.W. Lim[3], Newfei Ho[2], Joann Poh[3], R. Alison Adcock[4], Richard Keefe[4], Stephen Wood[5], Ranga Krishnan[3], Michael Chee[3], Juan Zhou[3]
*[1]Duke-NUS Medical School; [2]Institute of Mental Health, Singapore; [3]Centre for Cognitive Neuroscience, Duke-NUS Medical School; [4]Duke University; [5] University of Birmingham*

**Background:** Variation in trajectories of Ultra high-risk (UHR) psychosis mental state posts challenge to schizophrenia prevention or onset delay intervention. Our previous work described the heterogeneity at this prodromal stage of schizophrenia in both brain structure changes and resting-state functional network differences. Functional dysconnectivity can be one of the altered brain substrates underlying clinical symptoms. Lower resting-state functional connectivity (FC) within the salience network (SN) in schizophrenia was detectable at the UHR stage. FC between the fronto-parietal network (FPN) and the SN was disrupted when network integration fell apart in the UHR state.
FPN and SN are important for working memory (WM), which is largely compromised in schizophrenia and lesser in UHR group. Our previous work showed that WM task-related activation in the FPN and SN differed between the UHR and controls. Importantly, such differences varied with WM demands. Evidence has demonstrated that compared to resting-state FC, task-based FC may better predict behavioral performance. However, the WM-related FC in UHR group and its longitudinal changes are still largely unknown.
To cover the gap, we sought to examine the heterogeneity in the WM task-related FC changes in a group of UHR participants over time. We expected WM-related FC would link to individual differences in clinical trajectories.
**Methods:** Based on the longitudinal changes of UHR state within 2 years, participants were divided into 3 groups: 42 controls, 34 UHR remitters (UHR-R) and 42 UHR non-remitters (UHR-NR). We acquired fMRI (TR/TE = 2000/30 ms, 3 x 3 x 4 mm3, 28 slices) when participants performed WM task at different WM demands, varying from information maintenance alone (low) to requiring both maintenance and manipulation (high). We used seed-based approach (gPPI) to compare task-related FC of the FPN and the SN among groups. Voxel-wise FC with six seeds (bilateral anterior insula, parietal, and dorsal lateral prefrontal cortex, identified based on task activation) was regressed on WM demands and groups, controlling for age, gender, education, ethnicity, handedness and task accuracy. Linear mixed modeling methods were used to test longitudinal FC changes and the association between FC and clinical syndromes.
**Results:** Task performance was worse at high WM demand as expected, but no significant difference was found between groups or over time. Compared to controls, higher FC between the FPN (superior parietal gyrus) and the SN (insula) at low demand was observed in the UHR-NR group at baseline. Within the SN, WM-demand related FC between right insula and thalamus varied among 3 groups: low FC at low demand and high FC at high demand in controls; high FC at low demand but low FC at high demand in the UHR-R group. In contrast, UHR-NR group had high thalamus-insula FC in both WM demands.
Moreover, longitudinal FC increase only occurred within the SN at high WM demand in the UHR-R group, while other task-related baseline group differences of FC remained stable over time. Importantly, the rate of intra-SN increase of FC over time at higher WM demand was associated with decline of the positive psychosis syndromes in the UHR-R group.
**Discussion:** In support of the functional dysconnectivity hypothesis, our study indicated that UHR state was accompanied by altered brain FC during WM task performance. In contrast to lower SN FC at rest, UHR state showed SN hyper-connectivity in task with low WM demand, suggesting the importance of studying UHR both at resting and in task. Importantly, intra-SN FC increase at high WM demand was linked to positive psychosis syndrome reduction in remitters, while no FC changes if UHR state persisted. We argued that task FC could reflect the clinical heterogeneity of the UHR group.

### F157. HIERARCHICAL PREDICTION ERRORS DURING AUDITORY MISMATCH UNDER PHARMACOLOGICAL MANIPULATIONS: A COMPUTATIONAL SINGLE-TRIAL EEG ANALYSIS

Lilian Weber*[,1], Andreea Diaconescu[2], Sara Tomiello[2], Dario Schöbi[2], Sandra Iglesias[2], Christoph Mathys[3], Helene Haker[2], Gabor Stefanics[2], André Schmidt[4], Michael Kometer[5], Franz X Vollenweider[5], Klaas Enno Stephan[2]
*[1]ETH Zurich; [2]University of Zurich, ETH Zurich; [3]SISSA; [4]University of Basel (UPK); [5]University Hospital of Psychiatry, University of Zurich*

**Background:** A central theme of contemporary neuroscience is the notion that the brain embodies a generative model of its sensory inputs to infer on the underlying environmental causes, and that it uses hierarchical prediction errors (PEs) to continuously update this model. In two pharmacological EEG studies, we investigate trial-wise hierarchical PEs during the auditory mismatch negativity (MMN), an electrophysiological response to unexpected events, which depends on NMDA-receptor mediated plasticity and has repeatedly been shown to be reduced in schizophrenia.
**Methods:** Study1: Reanalysis of 64 channel EEG data from a previously published MMN study (Schmidt et al., 2012) using a placebo-controlled, within-subject design (N=19) to examine the effect of S-ketamine. Study2: 64 channel EEG data recorded during MMN (between subjects, double-blind, placebo-controlled design, N=73), to examine the effects of amisulpride and biperiden. Using the Hierarchical Gaussian Filter, a Bayesian learning model, we extracted trial-by-trial PE estimates on two hierarchical levels. These served as regressors in a GLM of trial-wise EEG signals at the sensor level.

**Results:** We find strong correlations of EEG with both PEs in both samples: lower-level PEs show effects early on (Study1: 133ms post-stimulus, Study2: 177ms), higher-level PEs later (Study1: 240ms, Study2: 450ms). The temporal order of these signatures thus mimics the hierarchical relationship of the PEs, as proposed by our computational model, where lower level beliefs need to be updated before learning can ensue on higher levels. Ketamine significantly reduced the representation of the higher-level PE in Study1. (Study2 has not been unblinded.)
**Discussion:** These studies present first evidence for hierarchical PEs during MMN and demonstrate that single-trial analyses guided by a computational model can distinguish different types (levels) of PEs, which are differentially linked to neuromodulators of demonstrated relevance for schizophrenia. Our analysis approach thus provides better mechanistic interpretability of pharmacological MMN studies, which will hopefully support the development of computational assays for diagnosis and treatment predictions in schizophrenia.

### F158. FUNCTIONAL CONNECTIVITY DIVERSITY OF THE INSULA CORTEX IN SCHIZOPHRENIA: SUBREGIONS OR CONTINUA?

Ye Tian*[,1], Chad Bousman[2], Chenxing Liu[3], Christos Pantelis[3], Andrew Zalesky[3]
*[1]The University of Melbourne; [2]University of Calgary; [3]The University of Melbourne, Melbourne Neuropsychiatry Centre*

**Background:** The function, cytoarchitecture and connectivity of the insula cortex are diverse. Cluster analyses have been applied to functional magnetic resonance imaging (MRI) connectivity data to parse this diversity and subdivide the insula into discrete subregions. However, the number of subregions comprising the insula remains vexed and whether these putative subregions are disturbed in neuropsychiatric illness are unknown. The present study aimed to (i) rigorously evaluate the number of subregions (if any) into which the insula can be subdivided based on topographic variation in whole-brain patterns of insula functional connectivity; and, (ii) establish whether the connectional topography of the insula is altered in schizophrenia.
**Methods:** Two alternative models explaining the heterogeneity of insula connectivity were tested: (Model i) insula comprising discrete subregions, each associated with a distinct connectivity fingerprint; and, (Model ii) connectivity varying as a continuum across insula, without marked boundaries. Cluster analysis was used to delineate discrete subregions, and a novel gradient-based method was developed to evaluate whether connectivity varied continuously across the insula. These models were tested in a sample of individuals with schizophrenia (N=49), healthy comparison individuals (N=52) and an independent validation cohort from the Human Connectome Project (N=50).
**Results:** Cluster analyses indicated that the insula comprised anterior and posterior subregions, with significantly less differentiation in connectivity patterns between these two clusters in the schizophrenia group (right: P=.0038; left: P=.002). The anterior insula was more strongly connected to the sensory-motor, occipital/parietal cortex and posterior lobe of cerebellum in the schizophrenia group, whereas the connectivity between the posterior insula and prefrontal cortex and thalamus was stronger in the patients (PFWE<.05). The dysconnectivity between anterior insula and anterior cingulate cortex was correlated with the severity of emotion withdrawal (Jonckeere-Terpstra test; JT=-3.74, P<.001). Most importantly however, for the majority of individuals in both datasets, the degree of cluster separation between insula subregions identified with cluster analyses was not significantly improved compared to clusters delineated in null data that was generated from white matter, where no clusters were expected. Modeling patterns of insula connectivity as continua of variation across a rostrocaudal axis was found to provide a more parsimonious model than using distinct subregions segregated by sharp boundaries. The variation in connectivity across this rostrocaudal axis was significantly reduced in schizophrenia patients (P=0.02).
**Discussion:** This is the first study that comprehensively investigate the potential differences in connectional pathology of insula between its anterior and posterior aspects. We conclude that the connectional diversity of the insula inferred from resting-state functional connectivity should be conceptualized as continua of variation, rather than discrete subregions. We posit that the reduced differentiation between the anterior and posterior insula in schizophrenia may impact on the ability in discriminating self-generated from externally-generated sensory information, possibly contributing to hallucinations in the disorder.

### F159. NEUROMAGNETIC 40 HZ AUDITORY STEADY STATE RESPONSES AND AUDITORY CORTICAL GABA AND GLX IN CLINICAL HIGH RISK AND FIRST EPISODE OF PSYCHOSIS INDIVIDUALS

Hanna Thuné*[,1], Tineke Grent-'t-Jong[1], Emmi Mikanmaa[1], Marc Recasens[1], Frances Crabbe[1], Jozien Goense[1], Andrew Gumley[1], Matthias Schwannauer[2], Stephen Lawrie[3], Ruchika Gajwani[1], Joachim Gross[1], Peter Uhlhaas[1]
*[1]University of Glasgow; [2]University of Edinburgh; [3]Royal Edinburgh Hospital*

**Background:** Robust impairments in the power and phase of 40 Hz auditory steady state responses (ASSR) have been reported in chronic schizophrenia patients. This could reflect changes in the balance between inhibitory GABAergic and excitatory glutamatergic neurotransmission in auditory cortex. However, the direct link between the ASSR and alterations in these neurotransmitter systems has not been systematically explored. Furthermore, it remains unclear whether 40 Hz ASSR impairments are present in early and at-risk stages of psychosis. The current study aims to explore the 40 Hz ASSR in first-episode of psychosis patients and individuals at clinical high risk (CHR) of psychosis, and the possible relationship of deficits in gamma-band entrainment to a dysfunctional excitation inhibition balance, as reflected by alterations in cortical GABA and glutamate.
**Methods:** Data from 80 CHR, 11 FEP and 40 age-matched healthy control participants were collected as part of the MRC-funded Youth Mental Health Risk and Resilience study. MEG data were recorded on a 4D Neuroimaging Magnes 3600 Whole Head 248 Channel system, while participants were passively presented with a series of 1000 Hz carrier tones amplitude modulated at 40 Hz. Data were analysed at sensor and source-level in the frequency-domain for spectral power and intertrial phase-coherence (ITPC). For source-reconstruction, an eLoreta source-analysis algorithms was employed. Auditory regions of interest (ROIs) were defined using 98 nodes defined from the AAL-atlas. Levels of right auditory GABA and Glx (glutamate + glutamine) were measured using 1H-MRS at 3T and 2*2*2 cm voxels and were estimated relative to water. GABA levels were further corrected for grey and white matter and cerebrospinal fluid levels within the voxel. Finally, 40 Hz ASSR power in right auditory cortex was explored in relation to neurotransmitter levels in the same region.
**Results:** Across groups, the ASSR stimulus activated temporal regions, including bilateral heschl's gyrus and superior temporal cortex. A significant effect of hemisphere was found, reflecting higher 40 Hz ASSR power in the right hemisphere across groups. CHR and FEP participants showed attenuated 40 Hz ASSR power and ITPC compared to healthy control participants in right temporal regions, but an increase in spectral power in the left hemisphere. A moderate positive correlation was found between right auditory GABA and 40 Hz ASSR power in the right superior temporal gyrus in CHR, but not in controls.
**Discussion:** These results provide a link between MRS measures of GABA and 40 Hz ASSR power impairments in CHR individuals. Furthermore, these preliminary findings indicate that slight alterations in the 40 Hz

ASSR are present in FEP patients and may arise already in the CHR stage, prior to the onset of psychosis.

### F160. CLASSIFYING SCHIZOPHRENIA BY PATTERNS OF BOLD FLUCTUATIONS USING MULTIVARIATE PATTERN RECOGNITION ANALYSIS

Jing Shang*[,1], Christian Sorg[1], Josef G. Bäuml[1], Joseph Kambeitz[1], Felix Brandl[1], Nikolaos Koutsouleris[1]
[1]*LMU*

**Background:** Schizophrenia is characterized by changes in both ongoing blood oxygenation level dependent (BOLD) signal fluctuations of resting-state fMRI and their coherence in terms of functional connectivity. The current study asks the question whether individualized patterns of BOLD fluctuations are able to classify schizophrenia patients from healthy controls.
**Methods:** To investigate this question, 61 schizophrenia (SZ) patients and 73 healthy controls (HC) were obtained from a Mind Research Network COBRE dataset available via COINS (http://coins.mrn.org/dx). The amplitude of low-frequency fluctuations (ALFF) was used as main outcome measure reflecting BOLD fluctuations. Multivariate pattern classification framework based on support-vector machines (SVM) was used to generate and validate ALFF patterns for group separation.
**Results:** ALFF based classifiers were able to distinguish between SZ patients and HC with 76.9% accuracies (balanced accuracy 76.5%, specificity 80.8%, sensitivity 72.1%, Area Under the Curve: 0.78). Decreased ALFF highly predictive for SZ was located in bilateral somatomotor cortex, cuneus and orbitofrontal cortex. Increased ALFF highly predictive for SZ was located in the thalamus, dorsomedial prefrontal cortex, and precuneus.
**Discussion:** Conclusions: Our results provide evidence for BOLD-fluctuation pattern could be treated as reliable feature to identify individual patients with schizophrenia from healthy controls. Multivariate pattern analysis such as support vector machine may reliably detect signatures of schizophrenia.

### F161. BRAIN VOLUME CHANGE IN PATIENTS WITH SCHIZOPHRENIA AND ITS RELATION WITH ANTIPSYCHOTIC DRUG USE

Fatma Kiras*[,1], Mustafa Yildiz[1], Yonca Anık[1]
[1]*Kocaeli Üniversitesi*

**Background:** Schizophrenia is a chronic severe mental illness affecting 0.4% of the society with its early age of onset (generally between 15–25 years) which can be seen in people from all social strata, has recurrent episodes or relapses, and causes disability by deteriorating interpersonal and occupational functioning. Critical behavioral findings accelerated the efforts on understanding the neurobiology of this complex disorder. Although the nature of progressive structural brain change is not understood completely, identifying the role of antipsychotic drugs is important to comprehend how much of the progressive brain volume change is the result of schizophrenia. The purpose of the study is to perform the volumetric brain magnetic resonance imaging (MRI) from patients with schizophrenia, to repeat the MRI after one year regular follow-up, to investigate whether brain volume-brain structures' volume change by time and reveal its relationship with the type and dosage of antipsychotic drug (APD). This study is considered that would contribute to the discussions about the disease process of schizophrenia and the effect of APD use on structural changes in brain.
**Methods:** Fifty-two patients diagnosed with schizophrenia according to DSM IV was participated to the study. Patients are followed for approximately 14 months. In the first interview, patients were applied to a sociodemographic form, The Structured Clinical Interview for DSM-IV (SCID-I), Positive and Negative Syndrome Scale (PANSS), Global Assessment of Functioning (GAF), Clinical Global Impression (CGI). After the first interviews, the patients who performed brain MR were arranged control meetings by the same doctor regularly (monthly, bimonthly, or when needed) until the second MR. In control meetings, patients were applied to clinical interview form, PANSS, GAF, and CGI. Drugs were recorded regularly by making sure that they took the drugs precisely with the help of their families. Chlorpromazine equivalent doses of APDs were calculated as milligram (mg) and turned into dose-year unit. Second MRIs were performed approximately after 14 months. In SPM program, paired sample t test was used to compare two MRIs and see whether gray matter volume was changed regardless the type of drugs the person used. Also, ANOVA was used to identify the relationship between the type of the drug taken between two MRIs and the volume change in gray matter by choosing the drug type as covariant. SPSS 22 was used to examine the relationship between the sociodemographic features of the patients and their brain volume findings.
**Results:** From the patients participated in our study, 19 of them were female and 33 were male, mean age was 36.5, the level of education was 11 years, the mean onset of the disease was 24 and duration of the illness was 11 years. On the first MRI analyses, gray matter was negatively correlated with the age of the patients (r= -0,473, p<0,001) and the duration of the illness (r=-0,316, p<0,05), and positively correlated with the onset of the disease (r= 0,281, p<0,05) and the level of education (r= 0,321, p<0,05); while white matter positively correlated only with the level of education (r= 0,321, p<0,05). Throughout 14 months, no significant difference was found in brain volume change analysis with 95% confidence. Gray matter was not significantly correlated with APD dose, atypical antipsychotic drug (AAPD) use, and typical antipsychotic drug (TAPD) use.
**Discussion:** It can be concluded that throughout 14 months period, brain volume of the patients with schizophrenia was not change significantly and the type and dose of APD used during this process did not have any effects on volume change.

### F162. MORE EFFORT, BUT LESS EFFICIENCY - WORKING MEMORY IN SCHIZOPHRENIA: AN INVESTIGATION INTO CAUSE FOR DIVERGENT RESULTS

Pavan Mallikarjun*[,1], Sabrina Meecham[2], Rachel Upthegrove[1], Paris Alexandros Lalousis[1], Anna Diukova[3], Dorothee Auer[4], Peter Liddle[4]
[1]*University of Birmingham;* [2]*Birbeck, University of London;* [3]*Cardiff University;* [4]*University of Nottingham*

**Background:** Functional brain imaging studies of working memory in people with schizophrenia have reported divergent reports with some studies reporting hypofrontal activation while report hypofrontal activation. The inconsistencies in the literature have been postulated to be related to task requirements or poor task performance in schizophrenia. An alternative hypothesis proposed is that different regions within the PFC show either hypo or hyperactivity, depending on their location. In view of the inconsistencies in previous research, the aim of the current research is to examine abnormalities in a cohort of partially remitted people with schizophrenia and matched controls
**Methods:** Twenty people with schizophrenia and 20 matched healthy controls (matched on age, gender and parental social and economic status) were selected from the Nottingham area to undergo a functional magnetic resonance imaging (fMRI) scan while undergoing two levels of the visual n-back task (zero-back and two back). The behavioural results (latency and accuracy) on the nback task was compared using repeated measures analysis. The MR images were reoriented, realigned, co-registered to the T1-weighted anatomical image, and normalised to the MNI space. Normalised images were resliced to a voxel size of 3 mm3, smoothed with an 8 mm full-width half-maximum Gaussian kernel using SPM 5. The GLM approach implemented in SPM 5 was utilized for fMRI data modeling and analyses. Each epoch type (zero-back, two-back and rest) was modeled in a boxcar design, using a canonical hemodynamic response function and its temporal derivative. The single group second level analysis consisted of voxelwise one-sample t-tests with significance threshold set at voxel level $p < .05$, false discovery rate (FDR) corrected for multiple comparisons across the entire brain.

The between-group comparison employed voxelwise two sample t-tests with the significance criterion based on the spatial extent of suprathreshold voxel clusters, a method proposed by (Friston et al., 1994). The criterion for inclusion of a voxel in a cluster was set at $p < .05$ (uncorrected) and a cluster was considered as significant at level of $p < .05$ (corrected).

**Results:** Patients exhibited relatively high general function and mild symptoms, but had significantly longer reaction time and decline in performance. Both controls and patients activated similar set of brain regions similar to Central executive network and deactivated brain areas comprising the default mode network. Most noteworthy, we did not find evidence for hypofrontality (reduced activity in DLPFC in patients compared to controls) in this study. Compared to controls, patients showed areas of greater activation in left postcentral gyrus, anterior and posterior medial frontal gyrus and left superior temporal gyrus.

**Discussion:** Both controls and patients showed activation in widespread brain areas consistent with the CEN, and deactivation in areas consistent with DMN regions. There was no evidence of hypofrontality in this sample of remitted people with schizophrenia. Patients showed activation patterns on the zeroback task similar to the activation patterns for controls on the two back task, suggesting that patients showed increased brain activation on a task with lesser requirements, though behavioural results suggest inefficiency despite the increased effort.

### F163. STRUCTURAL AND FUNCTIONAL ALTERATIONS IN THE BRAIN DURING WORKING MEMORY IN MEDICATION-NAÏVE PATIENTS AT CLINICAL HIGH-RISK FOR PSYCHOSIS

Kolbjørn Brønnick[1], Jens Gisselgård[2], Inge Joa[1], Aase Ingvild[1], Robert Jørgensen[1], Jan Olav Johannessen*[,1]

*[1]TIPS – Centre for Clinical Research in Psychosis, Stavanger University Hospital; [2]Stavanger University Hospital*

**Background:** The pre-TIPS study in 1994–95 showed that the duration of untreated psychosis (DUP) was long in our region with a mean value of 2.1 years, and median 26 weeks. This set the stage for the TIPS-study (1997–2000), reducing DUP through information campaigns targeted to the general population and other referral agents (GP's, schools and others) in Rogaland County (Norway). The information campaigns were launched together with a low threshold organization with direct access to an early detection team, for a diagnostic interview and help. The hypothesis was that this could change help seeking behavior, awareness towards psychosis and thus reduce the DUP. The information campaigns and the early detection team were introduced in an early detection(ED) area (Rogaland county, Norway) comparing DUP with two usual-detection control sites in Oslo (Norway) and Roskilde (Denmark). As a result, DUP in the early detection area was reduced from 26 weeks median to 4 weeks median. Symptom and function advantages of early detection and DUP reduction have been demonstrated as being significant throughout the follow-up period. Social and functional outcome have been increasingly emphasized as being key parameters, as these contribute to both quality of life and to financial costs in society. The TIPS program continues to include and follow patients; since the mid 2000's also young people with ultra-high risk states (Prevention of Psychosis; POP) or substance induced psychoses.

Previous ultra-high risk studies suggest that psychotic disorders are associated with structural and functional abnormalities within the frontoparietal circuits and that medication status is a potential confounding factor. We investigated neural correlates to ultra-high risk (UHR) for psychosis in medication-naïve patients.

**Methods:** 41 CHR patients and 37 healthy controls were examined with 1.5 Tesla MRI, yielding functional scans while performing an N-back task and structural T1-weighted brain images. Functional and structural data underwent automated preprocessing steps in SPM and Freesurfer, correspondingly. The groups were compared employing mass-univariate strategy within the generalized linear modelling framework.

**Results:** UHR demonstrated reduced suppression of the medial temporal lobe (MTL) regions during n-back task. We also found that, consistent with previous findings, UHR subjects demonstrated thinning in prefrontal, cingulate, insular and inferior temporal areas, as well as reduced hippocampal volumes.

**Discussion:** The present findings add to the growing evidence of specific structural and functional abnormalities in the brain as potential neuroimaging markers of psychosis vulnerability.

### F164. VENTRAL AND DORSAL STRIATAL DYSFUNCTION DURING REWARD ANTICIPATION IS ASSOCIATED WITH NEGATIVE SYMPTOMS IN PATIENTS WITH SCHIZOPHRENIA AND HEALTHY INDIVIDUALS

Marta Stepien[1], Andrei Manoliu[1], Roman Kubli[1], Karoline Schneider[1], Philippe N. Tobler[2], Erich Seifritz[1], Marcus Herdener[1], Stefan Kaiser[3], Matthias Kirschner*[,1]

*[1]Psychiatric Hospital, University of Zurich; [2]Laboratory for Social and Neural Systems Research, University of Zurich; [3]Geneva University Hospitals*

**Background:** Negative symptoms are a core feature of schizophrenia and also found in healthy individuals in subclinical forms. According to the current literature the two negative symptom domains, apathy and diminished expression may have different underlying neural mechanisms. Previous observations suggest that striatal dysfunction is associated with apathy in schizophrenia. However, it is unclear whether apathy is specifically related to ventral or dorsal striatal alterations. Here, we investigated striatal dysfunction in patients with schizophrenia and a nonclinical population, to determine whether it is a relevant neural correlate for apathy.

**Methods:** Chronic schizophrenia patients (n= 16) and healthy controls (n=23) underwent an event- related functional MRI, while performing a variant of the Monetary Incentive Delay Task. The two negative symptom domains were assessed in both groups using the Brief Negative Symptoms Scale.

**Results:** In schizophrenia patients, we saw a strong negative correlation between apathy and ventral and dorsal striatal activation during reward anticipation. In contrast, there was no correlation with diminished expression. In healthy controls, global negative symptoms were correlated with decreased dorsal striatal activity.

**Discussion:** This study replicates our previous findings of a correlation between ventral striatal activity and apathy but not diminished expression in chronic schizophrenia patients. The association between apathy and reduced dorsal striatal activity suggests that impaired action-outcome selection is involved in the pathophysiology of motivational deficits. Finally, our findings in healthy controls support the idea that striatal alterations are a plausible neural correlate for negative symptoms in both a clinical and a subclinical context.

### F165. OBSTRUCTIVE SLEEP APNOEA IS COMMON IN SCHIZOPHRENIA AND RESPONDS WELL TO TREATMENT – A NOVEL AND PRACTICAL MEANS TO IMPROVE COGNITION AND METABOLIC HEALTH?

Cherrie Galletly*[,1], Hannah Myles[1], Andrew Vincent[2], Nicholas Myles[3], Robert Adams[1], Madhu Chandratilleke[4], Dennis Liu[1], Jeremy Mercer[4], Andrew Vakulin[4], Gary Wittert[2]

[1]University of Adelaide; [2]Freemasons Foundation Centre for Men's Health; [3]Royal Adelaide Hospital; [4]Adelaide Institute for Sleep Health: A Flinders Centre for Research Excellence, Flinders University

**Background:** Obstructive sleep apnoea (OSA) is characterised by repeated collapse of the upper airway during sleep, causing hypoxia, frequent arousals and disruption to sleep architecture. OSA is more likely in people who are obese, smoke tobacco, and use alcohol and sedating medications – all these factors are more common in schizophrenia. OSA is likely to be underdiagnosed in schizophrenia as symptoms such as non-restorative sleep, depression and daytime somnolence may be attributed to chronic mental illness. OSA in the normal population is associated with cognitive deficits and poor cardiovascular health, both of which are common in schizophrenia, so comorbid OSA in schizophrenia may be exacerbating these problems.
Treatment of OSA with continuous positive airway pressure (CPAP) reduces daytime sleepiness, and improves quality of life, cognitive function, and cardiovascular risk factors. There are no published studies of CPAP treatment of OSA in schizophrenia, so it is not known whether these benefits also occur in the patient population.
**Methods:** Previous research into OSA in schizophrenia has utilised subjective screening instruments and there are no large studies using polysomnography (PSG), the gold standard method to diagnose OSA. We undertook home sleep studies using polysomnography in 30 people with schizophrenia, treated with clozapine. Participants cooperated well and all studies were of good quality.
We treated 6 participants with severe OSA with CPAP. Treatment adherence was good with mean CPAP usage of of 7.7 hours/night.
**Results:** We found that 14/30 (40%) of our participants with schizophrenia had OSA and 8/30 (27%) had severe OSA; twice the prevalence of severe OSA in the general population.
After six months CPAP treatment there was significant improvement in cognition, especially verbal memory, working memory and motor skills. Average weight loss was 7.2kg (SD 9k) with a 12mmHg (SD 18) reduction in systolic blood pressure. Normal sleep architecture was restored: on average the percentage of the night spent in restorative slow wave sleep increased from 4.8% to 31.6%, and the percentage in REM sleep from an average of 4.1% to 31.4%. The mean percentage of the night spent in a hypoxic state with oxygen saturation less than 90% reduced from an average of 27.6% to 2%.
**Discussion:** Improved awareness of the high prevalence of OSA in schizophrenia and access to diagnostic screening by home PSG should ensure this important comorbid condition is not missed. CPAP treatment for OSA in people with schizophrenia is feasible and has the potential to improve both cognition and cardiovascular health, resulting in better functioning and reduced cardiovascular morbidity.

### F166. LOW-GRADE INFLAMMATION IN FIRST-EPISODE PSYCHOSIS IS DETERMINED BY WAIST CIRCUMFERENCE INCREASE

Jaakko Keinänen*,[1], Outi Mantere[2], Tuula Kieseppä[3], Maija Lindgren[4], Teemu Mäntylä[4], Eva Rikandi[4], Jouko Sundvall[4], Minna Torniainen-Holm[4], Jaana Suvisaari[4]
[1]University of Helsinki; [2]McGill University; [3]University of Helsinki, Helsinki University Hospital; [4]National Institute for Health and Welfare

**Background:** There is evidence of low-grade inflammation in psychosis, as measured by the high-sensitivity C-reactive protein (hs-CRP). Significant weight gain is common during the first months of antipsychotic treatment. In the general population, overweight and obesity often lead to systemic low-grade inflammation. Lifestyle factors, such as smoking, can contribute to the pro-inflammatory changes. The metabolic changes in people with first-episode psychosis (FEP) taking place after the onset of psychosis can be especially harmful as these individuals are typically young and without major somatic illnesses. We aimed to study how the low-grade inflammation, measured by hs-CRP, develops in FEP and to clarify the effect of waist circumference increase in the inflammation.
**Methods:** The Helsinki Early Psychosis Study recruited FEP patients (age 18 to 40 years) attaining their first treatment for psychosis from the catchment area of the Helsinki University Hospital. We recruited 95 FEP patients and 62 controls. The inclusion criterion for the study was receiving a score of at least 4 in Unusual thought content or Hallucinations in the Brief Psychiatric Rating Scale - Extended (BPRS-E). Diagnoses of psychotic disorders according to the DSM-IV criteria were later verified using the Structured Diagnostic Interview for DSM-IV and reviewing all medical records. Substance-induced psychotic disorders and psychotic disorders due to a general medical condition were excluded. We measured the changes in hs-CRP, weight, waist circumference, glucose metabolism and lipids at baseline and at follow-ups of 2 and 12 months. We used linear mixed effects models to analyze the relationship between hs-CRP and waist circumference. In the model, we included a random intercept for each patient and, as fixed effects, we entered sex, time (days from baseline measurement), waist circumference and antipsychotic use at each assessment point, and baseline cigarette smoking.
**Results:** At baseline, FEP patients (mean age 26.1 years) had higher insulin resistance, total and LDL cholesterol, apolipoprotein B and triglyceride levels than controls. However, baseline weight and waist circumference, hs-CRP, fasting glucose and HDL cholesterol were similar between patients and controls. A robust change in anthropometric measures and inflammation was evident among patients by 12 months. Hs-CRP was significantly higher in patients at 12-month follow up than at baseline (baseline hs-CRP 0.67 mg/l, IQR 0.33–2.54; 12-month 1.73 mg/l, IQR 0.49–4.21; Wilcoxon signed-rank $p = 0.007$). When at the baseline the prevalence of overweight or obesity was 30% (28/94) in patients with FEP, by 12 months the prevalence was 59% (35/59) (McNemar´s test $p < 0.001$). The proportion of patients gaining ≥ 7 % of baseline weight was 68 % (40/59). The median weight gain among patients was 9.6 kg (IQR 1.5–13.6 kg), and the waist circumference increase 6.0 cm (IQR 2.0–13.0 cm). In the mixed effects model waist circumference ($p < 0.0001$) and sex ($p = 0.014$) were significantly associated with hs-CRP level.
**Discussion:** We detected a significant elevation in hs-CRP in people with FEP during the first treatment year. The rise in hs-CRP was determined by waist circumference increase. Patients with FEP are in a marked risk of developing abdominal obesity and subsequent low-grade inflammation during the first year of treatment. Prevention of the early metabolic changes in first-episode psychosis is important, as abdominal obesity and inflammation are associated with increased risk of cardiovascular events and mortality.

### F167. ACCESS, UNDERSTAND, APPRAISE AND APPLY TO / OF HEALTH INFORMATION AND HEALTH LITERACY IN INDIVIDUALS AT-RISK FOR PSYCHOSIS: A SYSTEMATIC REVIEW

Mauro Seves*,[1], Theresa Haidl[1], Susanne Eggers[1], Ayda Rostamzadeh[1], Anna Genske[2], Saskia Jünger[2], Christiane Woopen[2], Frank Jessen[1], Stephan Ruhrmann[1]
[1]University of Cologne; [2]Institute for the History of Medicine and Medical Ethics, University of Cologne

**Background:** Numerous studies suggest that health literacy (HL) plays a crucial role in maintaining and improving individual health. Furthermore, empirical findings highlight the relation between levels of a person's HL and clinical outcomes. So far, there are no reviews, which investigate HL in individuals at-risk for psychosis. The aim of the current review is to assess how individuals at risk of developing a first episode of psychosis gain access to, understand, evaluate and apply risk-related health information.
**Methods:** A mixed-methods approach was used to analyze and synthesize a variety of study types including qualitative and quantitative studies. Search strategy, screening and data selection have been carried out according to the PRISMA criteria. The systematic search was applied on peer-reviewed literature in PUBMED, Cochrane Library, PsycINFO and Web of Science. Studies were included if participants met clinical high risk criteria (CHR), including the basic symptom criterion (BS) and the ultra-high risk (UHR) criteria. The UHR criteria comprise the attenuated psychotic symptom criterion (APS), the brief limited psychotic symptom criterion (BLIPS) and the genetic risk and functional decline criterion (GRDP) Furthermore, studies must have used validated HL measures or any operationalization of the HL's subdimensions (access, understanding, appraisal, decision-making or action) as a primary outcome. A third inclusion criterion comprised that the concept of HL or one of the four dimensions was mentioned in title or abstract. Data extraction and synthesis was implemented according to existing recommendations for appraising evidence from different study types. The quality of the included studies was evaluated and related to the study results.
**Results:** The search string returned 10587 papers. After data extraction 15 quantitative as well as 4 qualitative studies and 3 reviews were included. The Quality assessment evaluated 12 publications as "good", 9 as "fair" and one paper as "poor". Only one of the studies assessed HL with as primary outcome. In the other studies, the five different subdimensions of HL were investigated as a secondary outcome respectively mentioned in the paper. "Gaining Access" was examined in 18 of the 22 studies. "Understanding" has been assessed in 7 publications. "Appraise" was examined in 9 studies. "Apply decision making" and "Apply health behavior" were investigated in 1 of 8 studies. Since none of the included publications operationalized neither HL nor the subdimensions of HL with a validated measure, no explicit influencing factors could be found.
**Discussion:** Quantitative and qualitative evidence indicates that subjects at-risk for psychosis describe a lack of understanding about their state and fear stigmatization that might lead to dysfunctional coping strategies, such as ignoring and hiding symptoms. Affected subjects are eager to be informed about their condition and describe favoured channels for obtaining information. The internet, family members, school personnel and GP's play a crucial role in gain access to, understand, evaluate and apply risk-related health information. The results clearly highlight that more research should be dedicated to HL in individuals at risk of developing a psychosis. Further studies should explore the relation between HL and clinical outcomes in this target population by assessing the underlining constructs with validated tools.

### F168. PSYCHOTIC EXPERIENCE AND ADOLESCENT BRAIN TRAJECTORY: EVIDENCE FOR STRUCTURAL ALTERATIONS IN DOPAMINERGIC REGIONS

Andre Zugman*[,1], Ary Gadelha[1], Felipe Argolo[1], Pedro Pan[1], Luis Augusto Rohde[1], Euripedes C. Miguel[1], Rodrigo Bressan[1], Andrea Parolin Jackowski[1]
[1]*Federal University of Sao Paulo*

**Background:** Psychotic Experiences (PE) are often reported by children and adolescents and have a bidirectional association with mental disorders, both increasing subsequent risk for mental disorders and being more frequently reported among subjects with a current psychiatric diagnosis. The brain developmental trajectories associated with PE in children and adolescents and how PE may evolve to a full blown mental disorder remain largely unknown. We assessed PE effect on subcortical and cortical measures over a 3-year follow-up in a community cohort of children and adolescents.
**Methods:** This study is part of the Brazilian High-risk Cohort, a multi-site longitudinal study. A total of 2,512 youths (6–12 years old, mean age at baseline 9.7 years, SD = 1.92; 53,1% male) completed the baseline assessment and 2,012, the 3-year follow-up (T1). PE were assessed at the two timepoints with The Community Assessment of Psychic Experience (CAPE). A confirmatory factor analysis (CFA) was used to generate a latent score for PE for baseline and follow-up, yielding good model fits.
A subset of the sample (n=809) was scanned in a 1.5T scanner on either baseline or follow-up resulting in 1183 MRI scans. Structural images were processed using Freesurfer. Subcortical volumes for the amygdala, hippocampus, caudate, putamen and pallidum were entered as a dependent variable in a linear mixed effects model (lme) with age, sex, CAPE and CAPE by age interaction as fixed effects and site and subject as random effects. The same model was applied in a mass-univariate analysis for cortical thickness measure (CT). A smoothing kernel (FWHM = 10 mm) was applied to CT before statistical testing. False Discovery Rate was used to control for multiple comparisons in the mass-univariate analysis with a $p<0.05$ threshold.
**Results:** CAPE was significantly related to the right putamen (Beta: -0.30 p:0.03), right caudate (Beta: -0.32 p: 0.03) and left caudate (Beta: -0.32 p:0.02). The age by CAPE interaction was significant for the three regions (right putamen Beta: 0.001 p:0.04, right caudate Beta:0.002 p: 0.03 and left caudate Beta: 0.001 p:0.03).
CAPE and CAPE by age interaction terms showed no effect on cortical thickness after correction for multiple comparisons.
**Discussion:** PE report was associated with lower subcortical volumes of the caudate and putamen, regions of the striatum. The striatum receives important dopaminergic neurotransmission and have been previously implicated in the pathogenesis of schizophrenia. In line with the hypothesis in schizophrenia, the PE experienced by children and adolescents may relate to a dopaminergic imbalance, possibly due to developmental structure alterations of the striatum and its connections. Interestingly, neither the hippocampus nor the amygdala were related to PE. Taken together with the lack of findings related to cortical thickness, the results presented here suggests a probable dopaminergic role on PE in young people with no current psychotic disorder.

### F169. BRAIN CONNECTIVITY DURING PSYCHOLOGICAL STRESS IN PATIENTS WITH SCHIZOPHRENIA

Mariana Castro*[,1], Gabriela De Pino[1], Hernán Bocaccio[1], Stella Sanchez[1], Lucas Drucaroff[1], Elsa Costanzo[1], Agustina Wainsztein[1], Salvador M. Guinjoan[1], Mirta F. Villarreal[1]
[1]*CONICET - UBA - FLENI*

**Background:** It is commonly accepted that in most patients with schizophrenia external factors act on genetic predisposition to produce active psychotic symptoms. In fact, we showed that patients with schizophrenia have an abnormal brain activation and peripheral autonomic response to psychological stress. We sought to characterize the brain connectivity networks of such response in schizophrenia.
**Methods:** We studied the pattern of brain connectivity in relation to mental arithmetic stress paradigm in 21 patients and 21 healthy subjects aged 18 to 50 years, using 3T-fMRI. A period of 6 minutes of resting state acquisition (PRE) were followed by a block design with three 1-minute CONTROL task (one digit sum), 1 minute STRESS task (two digit subtraction) and 1 minute rest after task (POST). Pairwise Pearson correlations were calculated between 90 regions of interest. Data were analyzed with MATLAB and SPSS software.
**Results:** Patients with schizophrenia showed a lower connectivity network between fronto-temporal limbic areas compared with control subjects during control and stress task.

Moreover, we observed a great variability of link density during resting state in patients but not in controls, and it diminishes in response to task.

**Discussion:** Patients present abnormalities in networks related to stress response showing an alteration in fronto-temporal connectivity, and a poor and random modulation of these networks at rest. Current and previous findings suggest abnormal fronto-temporal connectivity that ultimately would lead to psychotic symptoms emergency in response to an environmental stressor and, even, could be related to hypervigilance and misattribution feeding into the paranoid cognition characteristic of patients with schizophrenia.

### F170. SCHIZOPHRENIA POLYGENIC RISK SCORE ASSOCIATED WITH LEFT TEMPORAL GYRIFICATION

Igor Nenadic*[,1], Stephanie Gräger[2], Swapnil Awasthi[3], Kerstin Langbein[2], Maren Dietzek[2], Bianca Besteher[2], Stephan Ripka[4], Markus M. Noethen[5], Heinrich Sauer[2], Christian Gaser[2], Franziska Degenhardt[5]
*[1]Philipps-Universität Marburg - UKGM; [2]Jena University Hospital; [3]MIT; [4]MIT / Charite; [5]Bonn University*

**Background:** Brain structural changes in schizophrenia are thought to arise in part from genetic liability, as shown in studies of twins and siblings. Polygenic risk scores (PGRS) derived from large-scale genome-wide association studies (GWAS) have allowed to use measures of genetic liability calculated from large numbers of individual single nucleotide polymorphisms (SNPs). Initial studies on PGRS and structural imaging have, however, failed to provide clear associations. We used three separate measures of brain morphometry (voxel-based morphometry, cortical thickness, and gyrification) in a sample of healthy subjects to associate them with PGRS for schizophrenia in order to test the hypothesis that gyrification, a putative indicator of early brain development.

**Methods:** We analysed high-resolution MRI scans (3 Tesla, T1-weighted MPRAGE, 1x1x1mm resolution) from n=153 healthy subjects with not current or previous psychiatric condition recruited from the local community. DNA from each subject was analysed using the PsychChip, and polygenic risk scores were calculated for schizophrenia, as well as bipolar disorder and major depression (for assessment of relative specificity of the schizophrenia PGRS). MRI data were pre-processed with the CAT12 toolbox (dbm.neuro.uni-jena.de/cat12) for analysis using a) voxel-based morphometry (VBM), b) cortical thickness, and c) gyrification (calculated using the absolute mean curvature approach (Luders et al., NeuroImage 2006). We initially used $p<0.001$ uncorr. on the peak-level and performed correction for multiple-comparisons on the cluster level.

**Results:** We found a negative correlation of the schizophrenia polygenic risk score with gyrification in the left anterior superior cortex (i.e. the higher risk score loading the lower local gyrification), which was significant at the cluster-level for FWE correction ($p<0.047$). There was not such significant finding for positive correlations, nor for any of the VBM or cortical thickness analyses. Also, there was not significant association (positive or negative) with major depression or bipolar disorder PGRS in any of the three morphometry analyses.

**Discussion:** Our findings suggest that SNP-based genetic risk for schizophrenia is associated with left temporal gyrification, a putative indicator of early brain development, which again might be affected by multiple schizophrenia risk genes regulating cortical formation and connectivity. Furthermore, our findings are consistent with the notion of specificity for both morphometric marker (i.e. gyrification, but not VBM or cortical thickness) as well as diagnosis (with negative findings for major depressive and bipolar disorder risk scores). PGRS might impact on early developmental markers of brain structure (and possibly function), rather than overall liability-related variance.

### F171. ALTERED DIFFUSIVITY IN THE BRAIN OF PATIENTS WITH SCHIZOPHRENIA: A DIFFUSION WEIGHTED MAGNETIC RESONANCE IMAGING STUDIES WITH PUBLIC NEUROIMAGING DATA

Young Tak Jo[1], Yeon Ho Joo*[,1], Harin Kim[2], Seung-hyun Shon[1], Woon Yoon[1], Sung Woo Joo[3], Jungsun Lee[1]
*[1]Asan Medical Center, University of Ulsan College of Medicine; [2]Korean Armed Forces Capital Hospital; [3]Republic of Korea Marine Corps*

**Background:** In recent decades, numerous in vivo brain imaging studies utilizing diffusion weighted MRI (dMRI) technique have focused on altered diffusivity in brains of patients with schizophrenia. However, the literature has not reached at consistent consensus despite a few interesting and promising results. In this study, we investigated whether or not various measures of dMRI (FA, AD, RD, and TR) are altered in patients with schizophrenia by comparing them in both patients and healthy controls with public neuroimaging data from SchizConnect (http://schizconnect.org).

**Methods:** The final data set was consisted of 121 schizophrenia patients and 119 healthy controls. After verifying 161 anatomical regions of interest (ROIs), we estimated the mean value and standard deviation of fractional anisotropy (FA), axial diffusivity (AD), radial diffusivity (RD), and trace (TR) in each ROI among the healthy controls. After that, we calculated the Z-score of each single ROI in every individual brain of both patients and healthy controls. The Z-score information of each person is then integrated into two location-independent measures. One is the total number of "abnormal" lesions, in which the absolute Z-score is above the cut-off value estimated by the Bonferroni correction, and the other is the largest absolute Z-score. After all, by using Welch two-sample t-test, we compared these two measures between the groups of patients and healthy controls.

**Results:** The number of abnormal lesions was notably increased in patients group, in terms of RD ($p=0.01063$) and TR ($p=0.009329$). Meanwhile, no statistically significant differences related to FA and AD were observed. On the other hand, it was found that the largest absolute Z-score was elevated in patients group, in terms of AD ($p=0.03371$), RD ($p=0.0001762$), and TR ($p<0.00001$). Otherwise, no significant differences related to FA were observed.

**Discussion:** In this study, we found a few remarkable differences of familiar measures, especially TR, between brains of patients with schizophrenia and healthy controls. This suggests that there should be some subtle changes in the brains of patients with schizophrenia, including microstructural destruction.

### F172. INDIVIDUAL PREDICTION OF RISK IN ADOLESCENT OFFSPRING OF PARENTS WITH SCHIZOPHRENIA OR BIPOLAR DISORDER: A MACHINE LEARNING NEUROIMAGING STUDY WITH A CROSS-STAGE VALIDATION

Hugo Schnack*[,1], Julia Binnewies[1], Nikita Setiaman[1], Woutje Berdowski[1], Neeltje van Haren[1], René Kahn[1], Manon Hillegers[1]
*[1]UMC Utrecht*

**Background:** Schizophrenia (SZ) and bipolar disorder (BD) are severe psychiatric disorders that are not easily distinguishable based on clinical measures. Offspring of patients with SZ or BD have a tenfold increased risk of developing the disorder as well as an increased risk for other severe mental disorders. Reliable identification of these subjects might allow for early recognition and intervention, which have been shown to be beneficial for treatment outcome and may even prevent transition to illness. Based on abundant evidence that SZ and BD are associated with structural brain abnormalities, we investigated whether MRI brain-scans can be used to detect individual risk of developing SZ or BD in adolescents.

**Methods:** Structural MRI brain-scans were acquired in adolescent offspring (8–19 year) of parents with schizophrenia (oSZ;N=50), bipolar disorder (oBD;N=82), and without a mood or psychotic DSM-IV disorder (oHC;N=53), as part of the Dutch Bipolar and Schizophrenia Offspring Study (DBSOS). Support vector machine (SVM) models were trained on the gray matter tissue density maps to predict to which offspring class (oHC/oBD/oSZ) an individual belonged. Prediction accuracy was assessed using cross-validation. To validate our prediction models, we applied them to the tissue maps from subjects from a sample of unrelated HC/BD/SZ adults. Secondly, validated prediction models built from the adult subjects' MRI scans were applied to the tissue maps of the adolescents to predict illness class (HC/BD/SZ).

**Results:** The offspring-based model separated oHC/oSZ individuals with 77% accuracy (p<0.001), oHC/oBD with 68% accuracy (p<0.001), and oBD/oSZ with 64% accuracy (p<0.01). The adult-based models could separate the patients' offspring from the healthy offspring with 66–70% accuracy, but oBD from oSZ with lower accuracy (59%). In addition, the offspring models could separate adult patients from control subjects with comparable accuracy (66–68%) and separate the two patient groups with moderate accuracy (69%).

**Discussion:** The familial high-risk adolescents could be separated from controls with moderate to high accuracy (up to 77%), based on their MRI-scans. Moreover, the brain tissue patterns based on risk (adolescents) or illness (adults) were able to predict (risk) class in the other stage group. These results show (1) that high-risk individuals already show brain abnormalities, and (2) display similarities with abnormalities in ill adults, and (3) which can be used to detect (risk of) the disorder at the individual level. This suggests that MRI-scans, after further improvement and independent validation, may be of added value in the risk profiling of BD and SZ.

### F173. PITCH AND DURATION MISMATCH NEGATIVITY, AUDITORY CORTEX GRAY MATTER, AND PRODROMAL ROLE FUNCTIONING IN THE FIRST EPISODE SCHIZOPHRENIA SPECTRUM

Dean Salisbury*[,1], Anna Shafer[2], Brian Coffman[2], Timothy Murphy[2]

*[1]University of Pittsburgh School of Medicine; [2]University of Pittsburgh*

**Background:** Primary auditory cortex, contained within Heschl's gyrus, is implicated auditory processing deficits and auditory verbal hallucinations in schizophrenia. Previously we showed a pathological correlation between the magnitude of the pitch-deviant mismatch negativity (pMMN) response during a passive auditory task and reductions in gray matter volume in Heschl's gyrus in subjects with first hospitalized for schizophrenia. The aim of this study was to replicate this finding, examine duration-deviant mismatch negativity (dMMN) and gray matter correlations, and to examine pre-psychosis role functioning, in a first episode psychosis sample within the schizophrenia-spectrum.

**Methods:** Participants included 40 first episode schizophrenia subjects (FESz) and 40 healthy controls (HC) matched for age, parental socioeconomic status, IQ, sex, and handedness. For MMN extracted from the EEG, standard tones were presented repeatedly (1 kHz, 75 dB, 50 ms pips, 5 ms rise/fall times, 330 ms SOA) with an occasional pitch deviant (1.2 kHz, 10% of trials) or duration deviant (100 ms, 10% of trials) interspersed. pMMN and dMMN were measured from subtraction waveforms as the average voltage within a 100-ms group averaged peak window at Fz. Role functioning was measured with the Cornblatt Global Functioning: Role scale. A subset of 28 FESz and 28 matched HC underwent structural MRI. High-resolution T1-weighted structural MRI data (3T) were acquired for each subject. Freesurfer was used to segment white matter, gray matter, and pial surfaces. Left and right Heschl's gyri were manually edited regions of interest, and gray matter volumes determined.

**Results:** Despite a lack of pMMN or dMMN reduction at the group level in FESz, both measures were pathologically correlated with role functioning in the year prior to hospitalization. In FESz, smaller pMMN at Fz was associated with poorer role functioning in the year prior to psychosis (rho= -.35, p =.03). Similar associations were observed for dMMN (rho= -.41, p <.01). Furthermore, in the subset of FESz with sMRI, smaller pMMN at Fz was associated with less total gray matter volume in left Heschl's gyrus (TGMV) (rho= -.40, p =.03) but not right. Similar associations were observed for dMMN (rho= -.47, p .01). As well, role functioning and auditory cortex gray matter volumes were not correlated in FESz. There were no significant correlations within HC.

**Discussion:** Although pMMN and dMMN are not reduced at the group level, the size of both are associated with impaired functioning prior to psychosis and reduced gray matter volume of left hemisphere Heschl's gyrus, containing primary and secondary auditory cortices. Thus, pMMN and dMMN although not sufficient as biomarkers of disease presence, are suitable as reliable biomarkers of disease progression. Presumably, poorer role functioning and less gray matter reflect more of the pre-psychosis progressive pathological process thought to occur in the prodromal phase of psychosis. Hence, pMMN and dMMN are likely to serve as sensitive and robust outcome measures for therapeutic interventions and to guide treatment strategies in the prodrome and during early psychosis.

### F174. OBESITY AND BRAIN INTEGRITY IN SCHIZOPHRENIA AND BIPOLAR DISORDER: DIVERGENT PATTERNS OF WHITE MATTER MICROSTRUCTURE DAMAGE IN A TRANSDIAGNOSTIC APPROACH

Ramiro Reckziegel[1], Raffael Massuda[2], Leticia Czepielewski[3], Monise Costanzi[4], Rodrigo Spinz[3], Isadora Remus[3], Clarissa Gama*[,3]

*[1]Hospital de Clínicas de Porto Alegre - HCPA; [2]Universidade Federal do Parana - Brazil; [3] Federal University of Rio Grande do Sul; [4]Hospital Presidente Vargas - Brazil*

**Background:** Obesity is associated with both structural and functional changes of the central nervous system, and is frequent in psychiatry settings. The increased prevalence of obesity in schizophrenia (SCZ) and bipolar disorder (BD) is associated with illness severity, functioning impairment and cognitive deficits. It cannot be attributed to biases inherent in treatment-seeking samples, given that this association is detectable even in drug-naïve patients. Diffusion tensor imaging (DTI) analyses of major brain fibers in both disorders show shared abnormalities of white matter. DTI has been employed as a highly sensitive tool to investigate microstructural changes in white matter structure. While gray matter alterations in obesity point to a consistent reduction with increasing body mass index (BMI), volumetric changes in white matter are more complex and less conclusive. Fractional anisotropy (FA) is the most commonly used parameter as it is the best estimate of fiber integrity as well as axonal and myelin degeneration, and has been reported an association with BMI in depressed BD patients, but not explored in SCZ nor in comparison with a control group (CTR). The aim of this study was to analyze the relationship between obesity and brain alterations assessed by DTI in SCZ, BD and CTR.

**Methods:** In one-hundred fifty (N=150) individuals (SCZ:49; BD:35; CTR:66) were administered clinical rating scales, collected sociodemographic data and submitted to magnetic resonance imaging (MRI) acquisition in a 1.5 T machine. Linear regression models were performed independently for each group in order to test the relationship of BMI on each brain fiber FA, using gender, age and years of disease for the patients as covariates.
**Results:** The mean BMI was different among groups (F(143)6.533; P= .002), higher in BD group (BD 29.69 ± 6.55; CTR: 25.54 ± 4.25; SCZ: 26.42 ± 6.02). In BD, the model that predicted FA in the left cingulate gyrus endings was significant controlling for covariates (F(4,21)= 3.273; p = .031; Adj. $R^2$= .384), with a main effect of BMI (t=-2.870; p= .009; β=- .531). For SCZ and CTR groups, we did not find significant models to predict brain fiber FAs from BMI controlling for covariates.
**Discussion:** BMI was associated with reduced FA in cingulate gyrus in BD, implying that obesity may play a role in microstructure damage in the limbic system. These findings are in consonance with the literature and may be related with processing of emotional and cognitive responses disrupted in BD. Conversely, it did not predicted FA in SCZ or CTR connection bundles, possibly because of the lower BMI levels in these groups. Also, we were not able to control for treatment adherence, a variable correlated with both white matter integrity and weight gain. At last, obesity appears to be correlated with white matter microstructure in a heterogeneous and disease specific course depending on the underlying psychopathology, showing association with impairment in BD but not SCZ and CTR. Further studies are needed to explore the role of treatment in the interpretation of these findings.

### F175. NEUROLOGICAL SOFT SIGNS (NSS) AND BRAIN MORPHOLOGY IN PATIENTS WITH CHRONIC SCHIZOPHRENIA AND HEALTHY CONTROLS

Christina Herold*[,1], Marc Lässer[2], Ulrich Seidl[3], Dusan Hirjak[4], Philipp Thomann[1], Marco Essig[5], Johannes Schröder[1]
*[1]University of Heidelberg; [2]Rehabilitation Hospital Zihlschlacht; [3]Center for Mental Health; [4]Central Institute of Mental Health, Medical Faculty Mannheim; [5]German Cancer Research Center*

**Background:** Subtle abnormalities in sensory integration, motor coordination and sequencing of complex motor acts or neurological soft signs (NSS) are a characteristic phenomenon in patients with schizophrenia at any stage of the illness. Previous MRI studies in schizophrenia have shown that NSS are associated with abnormal cortical, thalamic and cerebellar structure. However, these studies mainly focused on first-episode or recent onset schizophrenia and the respective correlates between brain structure and NSS in patients with a chronic course of the disorder remain rather unclear.
**Methods:** 49 middle-aged patients with chronic schizophrenia with a mean duration of illness of 20.3 ± 14.0 years and 29 healthy subjects matched for age and sex were included. NSS were examined on the Heidelberg Scale and correlated to grey matter by using whole brain high resolution magnetic resonance imaging (3 Tesla) with SPM12 analyses.
**Results:** As expected, NSS in patients were significantly elevated in contrast to healthy controls, a finding, which not only applied to NSS sumscore, but also to the respective subscores motor coordination, sensory integration, complex motor tasks, right/left and spatial orientation and hard signs (p≤0.001).
Patients and healthy controls differed referring to right inferior frontal gyrus and left parahippocampal gyrus, with patients showing significantly reduced gray matter volumes, respectively. Within the patient group NSS total score was significantly correlated to reduced grey matter in right occipital lobe, left parahippocampal gyrus, left superior temporal gyrus, left thalamus (medial dorsal nucleus) and left posterior lobe of the cerebellum (declive). The respective findings remained significant after FDR correction for multiple comparisons (k=100 voxels). These results were confirmed when chlorpromazine (CPZ)-equivalents were introduced as additional covariate; moreover, no significant correlates arose between NSS and CPZ-equivalents. In the control group, VBM revealed that higher NSS total scores were significantly correlated with volume of right lentiform nucleus (medial globus pallidus).
**Discussion:** Our study leads further support to the model of 'cognitive dysmetria' with a disrupted cortico-cerebellar-thalamic-cortical circuit in schizophrenia. This interpretation is also maintained by a different correlational pattern in our control group. Furthermore, the middle temporal/parahippocampal region may correspond to reduced mnestic functions, which are – besides elevated NSS – consistently reported to be impaired in patients with a chronic course of the disorder.

### F176. CLINICAL CORRELATES OF CORTICAL STRUCTURE IN ANTIPSYCHOTIC-NAÏVE SCHIZOPHRENIA PATIENTS BEFORE AND AFTER SIX-WEEK TREATMENT WITH A DOPAMINE D2/3 RECEPTOR ANTAGONIST

Kasper Jessen*[,1], Egill Rostrup[1], Rene C. W. Mandl[2], Mette Ø. Nielsen[1], Nikolaj Bak[1], Birgitte Fagerlund[1], Birte Y. Glenthøj[1], Bjørn H. Ebdrup[1]
*[1]Center for Clinical Intervention and Neuropsychiatric Schizophrenia Research, Mental Health Centre Glostrup, University of Copenhagen; [2]Brain Center Rudolf Magnus, University Medical Center Utrecht*

**Background:** Schizophrenia has been associated with changes in both cortical thickness and surface area, but antipsychotic exposure, illness progression, and substance use may confound the observations. We investigated cortical thickness and surface area, as well as mean curvature, before and after antipsychotic monotherapy with a relatively selective dopamine D2/3 antagonist (amisulpride), in antipsychotic-naïve schizophrenia patients.
**Methods:** Fifty-six patients and 59 matched healthy controls (HC) underwent T1-weighted 3T magnetic resonance imaging. Forty-one patients and 51 HCs were re-scanned. FreeSurfer-processed baseline values and symmetrized percentage changes (SPC) in cortical structures were analysed using univariate ANOVA. Clinical measures comprised psychopathology ratings, assessment of functioning, and tests of premorbid and current intelligence.
**Results:** At baseline, groups did not differ in cortical thickness or surface area, while left curvature was higher in patients (p=0.015). In the complete sample, a higher curvature was associated with lower premorbid- (p=0.009) and current intelligence (p<0.001). Also, a lower surface area was associated with lower premorbid intelligence (p=0.014) and, in patients, a higher PANSS total (p=0.037). Lifetime substance use was associated with reduced cortical thickness (p=0.043). After six weeks, groups did not differ in overall change in cortical structures. Amisulpride dose (275.0 mg/day) did not correlate with cortical structures (p>0.43). A decrease in cortical thickness SPC was associated with less symptom improvement (p=0.002) and increase in surface area SPC was associated with improvement in GAF (p=0.047).
**Discussion:** Our results indicate that schizophrenia may be associated with subtle aberrations in cortical structures and these changes appear clinically relevant. Mean curvature holds promise as a sensitive supplement to cortical thickness and surface area, to detect complex structural brain abnormalities.

### F177. THALAMIC SUBREGION RESTING STATE CONNECTIVITY IN SCHIZOPHRENIA MEASURED AT 7T

Jun Hua[1], Ann Choe[2], Nicholas Blair[2], Anita Barber[1], Allison Brandt[2], James Pekar[1], Peter van Zijl[1], Christopher Ross[2], Issel Lim[1], Feng Xu[1], Russell Margolis*[,2]

[1]Johns Hopkins University, Kennedy Krieger Institute; [2]Johns Hopkins University

**Background:** The thalamus, a critical node in which multiple cerebral circuits converge, is organized into multiple subnuclei, classified as either first-order (receiving peripheral sensory input) or higher-order (receiving input primarily from the cortex). Higher-order nuclei are of particular salience in psychotic disorders, as they appear to control cortico-cortical information transmission, possibly through regulation of neuronal synchrony. Substantial evidence has demonstrated abnormalities of thalamo-cortical connectivity in schizophrenia, generally with hyperconnectivity with sensorimotor and temporal cortices and hypoconnectivity with frontal regions. We took advantage of the spatial resolution of 7T (voxels of 3.375 mm3 vs 27 mm3 at 3T) to preliminarily assess resting state connectivity between specific thalamic nuclei and cortical regions in schizophrenia.
**Methods:** Resting state fMRI scans were obtained for 14 SCZ patients (mean age 39.5, mean disease duration 18.8 years) and 14 matched controls (smoking, age, sex) using a Phillps 7T imaging system, with GRE EPI (TR/TE/FA=2000/22ms/60º, voxel=2.5mm iso, 54 slices, 7min. Data analysis was carried out with SPM8 / Matlab6. Preprocessing included realignment, slice time correction, co-registration, segmentation, normalization; nuisance removal (CompCor), regression of global mean and motion parameters; spatially smoothing (5mm kernel) and temporal filtering (0.01-0.1Hz). Seed-based analysis was carried out using thalamic sub-regions as described in the Oxford Thalamic Connectivity Atlas (seven regions based on anatomic connectivity rather than histology), and whole brain connectivity maps (z values) to each seed were calculated. Second-level t-tests were performed to examine differential connectivity between SCZ patients and controls (thresholded at a voxel-level of $p<.001$ and multiple-comparisons corrected at a cluster-level threshold of $p<.05$). Effect size was estimated with Cohen's d. The IBASPM116 atlas was used to identify anatomical regions within the significant clusters.
**Results:** Both reduced and enhanced functional connectivity between the thalamus and multiple brain regions were observed. Statistically significant differences between scz and controls were detected in 47 regions, with particularly strong differences between scz and control in thalamo-temporal cortex connectivity, consistent with previous results at 3T. The same analysis was performed but with seeds placed in each of the seven thalamic subregions defined by the Oxford thalamic connectivity atlas. Enhanced connectivity was observed between all thalamic sub-regions and the motor cortex. Enhanced connectivity to the temporal cortex was detected in several thalamic sub-regions, but not sub-region 7, which has the highest anatomical connection probability in controls. Reduced functional connectivity in SCZ was detected between thalamic sub-regions 4, 6, and 7, and prefrontal and cingulate cortex.
**Discussion:** Our results provide preliminary evidence of changes in resting state thalamo-cortical connectivity in schizophrenia that are specific to particular thalamic subregions. If confirmed by larger scale studies, identifying altered functional connectivity patterns of specific thalamic subnuclei may provide important clues about the pathogenic process in schizophrenia, with the potential of serving as biomarkers for use in therapeutic development.

### F178. NEUROANATOMICAL PROFILES OF TREATMENT-RESISTANCE IN PATIENTS WITH SCHIZOPHRENIA

Eric Plitman*,[1], Yusuke Iwata[1], Shinichiro Nakajima[1], Jun Ku Chung[1], Raihaan Patel[2], Fernando Caravaggio[1], Julia Kim[1], Vincenzo De Luca[1], Sofia Chavez[1], Gary Remington[1], M. Mallar Chakravarty[2], Philip Gerretsen[1], Ariel Graff-Guerrero[1]
[1]Centre for Addiction and Mental Health, University of Toronto; [2]Douglas Mental Health University Institute, McGill University

**Background:** About 20 to 35% of patients with schizophrenia show partial or no response to standard first-line antipsychotic treatment and are thus believed to have treatment-resistant schizophrenia (TRS). Notably, the antipsychotic clozapine (CLZ) has been reported to have superior efficacy compared to other agents for the treatment of TRS. However, a subset of patients still do not respond to CLZ treatment and are thus considered to have ultra-treatment-resistant schizophrenia (UTRS). Overall, the pathophysiology associated with UTRS appears to be different than TRS, yet both remain elusive. In light of the unknown factors underlying UTRS and TRS, along with the widely reported structural alterations that exist in patients with schizophrenia, our study aimed to examine subcortical structure volumes and cortical thickness in patients with UTRS, patients with TRS responding to CLZ (henceforth, TRS), patients responding to a first-line antipsychotic (treatment non-resistant schizophrenia (TnRS)), and healthy controls (HC). We hypothesized that deficits in subcortical structure volumes and cortical thickness would exist within the UTRS group compared to other groups.
**Methods:** As of December 2017, the sample consisted of a total of 94 participants, including 24 patients with UTRS, 24 patients with TRS, 21 patients with TnRS, and 25 HCs. Participants underwent a 3-dimensional T1-weighted scan in a 3T MRI machine. The MAGeT-Brain segmentation algorithm was used to acquire volumes of the thalamus, striatum, globus pallidus, hippocampus, and amygdala. Cortical thickness was estimated using the CIVET processing pipeline. Total brain volume was obtained using the BEaST method. Group comparisons were performed using analyses of covariance and post-hoc comparisons.
**Results:** Group volumetric differences were identified bilaterally within the thalamus, striatum, and globus pallidus ($p<0.01$). Post-hoc investigations revealed that bilateral thalamic volumes were smaller in the UTRS group compared to the HC group ($p<0.01$), bilateral striatal volumes were larger in the TnRS group compared to the UTRS and HC groups ($p<0.01$), and bilateral globus pallidus volumes were larger in the TnRS group compared to the HC group ($p<0.01$). No differences in hippocampal, amygdala, or total brain volume were observed. At a 5% false discovery rate, widespread cortical thinning was identified in both the UTRS and TRS groups compared to the TnRS and HC groups; this effect was stronger and more diffuse in the UTRS group.
**Discussion:** Our findings suggest that thalamic volume deficits might be a distinct feature of UTRS. Contrastingly, striatal and globus pallidus volume enlargement may be associated with first-line antipsychotic response or treatment. Cortical thinning is apparent in both the UTRS and TRS groups. In many cases, structural compromise appears to follow a continuum of response, whereby deficits are most severe in UTRS patients, followed by TRS patients, who are followed by TnRS patients and HCs.

### F179. NEURAL CORRELATES IN MUSICAL DEFICITS IN SCHIZOPHRENIA

Ken Sawada*,[1], Sanae Hatada[2], Ryoshuke Fujito[3], William Honer[4]
[1]Kochi Medical School; [2]Tosa Rehabilitation College; [3]Fujito Hospital; [4]University of British Columbia

**Background:** Several studies have shown that patients with schizophrenia have low musical ability that correlates with poor cognitive functions and severe negative symptoms. Recently, using surface-based analysis, we reported that thinner cortical thickness in the left temporal, parietal, and inferior frontal regions is associated with lower musical ability in schizophrenia. Interestingly, thicker cortical thickness in the left supramarginal region correlates with lower musical ability in controls. Musical deficits such as congenital amusia was shown to contribute to white and grey matter pathophysiology in schizophrenia. We, therefore, sought to investigate diffusion tensor images (DTI) of patients with schizophrenia using an automated probabilistic tractography algorithm.

**Methods:** Twenty-two right-handed patients with schizophrenia (12 males and 10 females, mean age = 45.9 years) and 20 right-handed healthy control subjects (13 males and 7 females, mean age = 42.8 years) consented to participate in this study. We measured musical ability, cognitive functions, and clinical assessments using the Montreal Battery for Evaluation of Amusia (MBEA), Brief Assessment of Cognition in Schizophrenia (BACS), and Positive and Negative Syndrome Scale (PANSS), respectively. We employed automatic probabilistic tractography DTI analysis using TRActs Constrained by UnderLying Anatomy (TRACULA) available in the Freesurfer software for the reconstruction of major tract bundles.
**Results:** Whole-tract diffusion characteristics in patients with schizophrenia and controls were significantly different. Fractional anisotropy (FA) was lower for patients with schizophrenia compared to controls in the left superior longitudinal fasciculus - parietal endings (slfp) ($p < 0.001$), left cingulum - angular bundle (cab) ($p < 0.001$), and corpus callosum - forceps minor (fminor) ($p < 0.001$). We found significant correlation between musical abilities and FA alterations in slfp in both controls and patients with schizophrenia. While lower musical ability corresponds to lower FA in slfp of controls ($r = -0.572$, $p = 0.013$), it is associated with higher FA in the slfp of patients with schizophrenia ($r = 0.515$, $p = 0.021$).
**Discussion:** This study shows that TRACULA can be used for the detection of decrements in several DTI tracts including the left slfp, left cab, and fminor in patients with schizophrenia. It revealed that while lower musical ability correlates with lower FA values in the left slfp in controls, it is associated with higher FA values in the same region in patients with schizophrenia. This contradictory finding in controls and patients with schizophrenia with regard to white matter pathology may reflect left supramarginal region malfunction resulting in cortical pathology in patients with schizophrenia. The data suggest that patients with schizophrenia may be more susceptible to changes in cortical thickness in the supramarginal region, and white matter alteration in the left slfp. Further study is needed to confirm the results. The characteristics of grey and white matter in the left parietal region which are relevant to musical ability may provide insight into pathological progression in patients with schizophrenia.

### F180. CANNABINOID 1 RECEPTOR AVAILABILITY & MEMORY FUNCTION IN FIRST EPISODE PSYCHOSIS: A MULTI-MODAL PET-FMRI STUDY

Faith Borgan*[,1], Mattia Veronese[2], Owen O'Daly[1], Tiago Reis Marques[2], Maria Rogdaki[2], Oliver Howes[2]
*[1]King's College London; [2]Psychiatric Imaging Group, MRC Clinical Sciences Centre, Institute of Clinical Science, Imperial College London*

**Background:** Although memory deficits are a core stable feature of schizophrenia, the neurobiology of these deficits remain poorly understood and unaddressed by current treatments. Converging lines of evidence show that the cannabinoid 1 receptor modulates memory function by altering mitochondrial function as well as synaptic transmission and plasticity. We aimed to investigate the association between memory function and cannabinoid 1 receptor availability, for the first time as far as we're aware in vivo. We also aimed to investigate the cannabinoid 1 receptor, for the first time as far as we're aware in first episode psychosis in order to identify if memory function is linked to a cannabinoid 1 receptor dysregulation.
**Methods:** Sixty-seven volunteers including 32 first episode psychosis patients (28 un-medicated, 4 medicated) and 35 matched healthy volunteers completed the Sternberg working memory paradigm during a functional magnetic resonance imaging (fMRI) scan. A subset of these volunteers including 20 healthy volunteers and 20 first episode psychosis patients (17 un-medicated, 3 medicated) also underwent a dynamic positron emission tomography (PET) scan using a cannabinoid 1 receptor selective radiotracer [11C]MePPEP with arterial blood sampling.
**Results:** Relative to healthy volunteers, first episode psychosis patients showed a significantly lower availability of cannabinoid 1 receptors in the hippocampus (Hedge's g=0.6) but showed greater bilateral hippocampal (left: pFWE=0.001; right: pFWE=0.002) and parahippocampal (left: pFWE=0.005; right: pFWE=0.014). functional activation during memory encoding. Healthy volunteers showed an association between CB1R availability in the hippocampus and mean functional activation in the parahippocampal gyrus during memory encoding (R=.567, p=0.027) but this association was not demonstrated by patients (R=.027, p=0.474). Relative to healthy volunteers, first episode patients also showed a significantly lower availability of cannabinoid 1 receptors in the anterior cingulate (Hedge's g=0.7) which was positively correlated with cognitive performance on the Wechsler Adult Intelligent Scale digit symbol coding test (R=.519, p=0.006) but inversely associated with the severity of delusional symptoms measured using the Positive and Negative Syndrome Scale (R=-.570, p=0.033).
**Discussion:** We demonstrate for the first time as far as we're aware that cannabinoid 1 receptor availability is linked to the neural correlates of memory encoding in healthy volunteers. We also demonstrate that first episode psychosis patients altered hippocampal functional activation during a memory task in the context of a hippocampal dysregulation in the cannabinoid 1 receptor. These findings extend an existing body of literature highlighting the role of the hippocampus in the pathophysiology of psychosis. These findings have implications for the understanding and treatment of memory deficits in schizophrenia.

### F181. CHANGE IN PREFRONTAL-LIMBIC MORPHOLOGY AND COGNITION IN DRUG-NAÏVE FIRST-EPISODE PSYCHOSIS PATIENTS FOLLOWING ATYPICAL ANTIPSYCHOTIC TREATMENT: A BRIEF LONGITUDINAL STUDY

Melissa Woodward*[,1], Geoffrey Smith[1], William Honer[1], Vina Goghari[2], Lili Kopala[1], Kristina Gicas[1], Allen Thornton[3], Wayne Su[1], William MacEwan[1], Donna Lang[1]
*[1]University of British Columbia; [2]University of Toronto; [3]Simon Fraser University*

**Background:** Atypical antipsychotics are thought to normalize structural morphology in subcortical regions, however their effect on cortical volume remains equivocal.1,2 Studying the impact of atypical antipsychotic treatment on cortical structure in drug-naïve first-episode psychosis (FEP) patients is an opportunity to elucidate the effects of illness chronicity and treatment. Previous work has indicated the potential for short-term atypical antipsychotic treatment to increase cortical thickness in FEP patients, particularly the rostral and caudal middle frontal cortices.3 Both entorhinal and orbitofrontal cortices are decreased in patients with schizophrenia and impairment in prefrontal-limbic circuitry has been linked with cognitive impairment in patients.4,5 We examined the ability of an eight-week atypical antipsychotic treatment to increase entorhinal cortex (ERC) and orbitofrontal cortex (OFC) volume and thickness and improve symptom severity in drug-naïve FEP patients.
**Methods:** Twenty-three FEP patients treated with risperidone or quetiapine and 28 healthy volunteers completed structural 3T magnetic resonance imaging, neurocognitive testing and clinical assessments at baseline, four weeks and eight weeks. Volumetric segmentation of the cortical regions of interest was performed with Freesurfer 5.3 software. Baseline and eight-week follow-up assessments were used to calculate change scores for clinical, cognitive and structural variables to compare between groups. Change in volume, clinical and cognitive scores were analyzed with ANCOVA with age, antipsychotic dose and total brain volume entered as covariates.

**Results:** FEP patients and healthy volunteers did not differ significantly in volume or thickness for both ERC and OFC regions at baseline. FEP patients demonstrated a significant increase in OFC volume (F(22,1)=5.23, p=0.34) and an increase in ERC thickness (F(22,1)=12.80, p=0.002) following treatment. Healthy volunteers had an increase in ERC volume (F(27, 1)=4.99, p=0.35). Cognitive switching, an indicator of executive functioning, was not predicted by our brain measures of interest at baseline. Following treatment, increased OFC volume predicted a worse performance on the cognitive switching task for patients (β(22,1)=0.770, p=0.017) but a better score for healthy volunteers (β(23,1)=-0.712, p=0.044). Symptom severity scores were not significantly related to our brain regions of interest.
**Discussion:** FEP patients have increase entorhinal cortical thickness and orbitofrontal cortical volume following an 8-week treatment of atypical antipsychotics. Increased OFC volume is associated with a decreased proficiency at cognitive switching for FEP patients but an increased proficiency for healthy volunteers. This difference may be due to underlying neurodevelopmental differences between psychosis patients and healthy controls and improvement in neurocognitive tasks may occur given a longer duration of antipsychotic treatment. These findings demonstrate the impact of atypical antipsychotics on cortical morphology in key regions associated with psychosis-spectrum disorders.

### F182. SYMPTOM-RELATED STRUCTURAL BRAIN PATTERN IN PATIENTS WITH SCHIZOPHRENIA-A PARTIAL LEAST SQUARE ANALYSIS

Susanna Muckenhuber-Sternbauer*[,1], Anne Ruef[2], Peter Falkai[2], Dominic Dwyer[2], Nikolaos Koutsouleris[2]
*[1]Ludwig-Maximilians-University, University Psychiatric Hospital; [2]Ludwig-Maximilians-University*

**Background:** Multivariate neuroimaging studies of schizophrenia have revealed a generalizable neuroanatomical signature of the illness which however does not fully explain the variance of ist clinical phenotyps. A potential strategy to improve the mapping between the psychopathology and brain pathology of the disorder is to decipher the dictionary of symptom pattern and their neuroanatomical fingerprints. If successful, such a strategy could support a biologically informed revision of the taxonomy of psychosis.
**Methods:** 176 patients with first episode to chronic stages of schizophrenia were assessed using the Positive and Negative Syndrome Scale (PANSS) and scanned using T1-weighted magnetic resonance imaging (MRI). The patients`PANSS scores, sociodemographic data and disease course variables, as well as their grey matter volume maps (GMV) entered a multivariate Partial Least Square (PLS) analysis that decomposed unique patterns of brain-behavior covariance between these data domains into latent variales (LV). We tested the LVs for significance using nonparametric- permutation and bootstrap resampling techniques.
**Results:** Three LVs showed significant brain-behavioral constellations. The first pattern linked hippocampal and medial frontal cortex volume with negative symptoms, age and age of onset. The second pattern consisted of opposite correlation between positive and negative symptoms associated with positive loadings in the subcortical structures such as the thalamus, the caudate nucleus and negative loadings in the auditory, insular and medial prefrontal cortices. The third LV presented a pattern involving negative symptoms, illness duration and age of onset as well as volume reductions in the anterior insular and orbitofrotal cortices.
**Discussion:** Our results indicate that the heterogeneity of schizophrenia can be decomposed into clinically relevant brain-behavioral phenotyps of the disorder, suggesting a biologically-informed and disease stage-sensitive stratification of schizophrenic patients. This might provide a neurobiological basis for future stratified investigations of treatment effects and prognosis both in early and advanced stages of schizophrenia.

### F183. FUSIFORM GYRUS ABNORMALITIES RELATED TO VERBAL INTELLIGENCE AND NEGATIVE SYMPTOMS IN FIRST-EPISODE PSYCHOSIS

Sang-Hyuk Lee[1], Jeong Hoon Kim[2], Sra Jung*[,1], Arira Lee[1]
*[1]CHA Bundang Medical Center, CHA University; [2]Bundang Jesaeng Hospital*

**Background:** Researches have shown that verbal intelligence of first-episode psychosis (FEP) can be deteriorated. Only a few studies have investigated about neural correlates of verbal intelligence in FEP. Fusiform gyrus is often referred to the “visual word form area (VWFA)”, also known to be systematically activated by reading. The object of this study is to explore the relationship between the volume of language processing regions and the verbal IQ (VIQ) in FEP.
**Methods:** 102 patients with FEP and 54 HC were enrolled this study. All subjects were right-handed. All patients were interviewed and diagnosed with the diagnostic criteria in Structured Clinical Interview for DSM-5 and examined by means of MRI at 3 Tesla at base line. Schizophrenia patients were measured their IQ through Korean-Wechsler Adult Intelligence Scale (K-WAIS). Positive and Negative Syndrome Scale (PANSS) were administered at baseline and 8 weeks after antipsychotics administration for patients. We used the FreeSurfer software package for estimating the volume of language processing area including pars triangularis, pars opercularis, insula, Heschl’s gyrus, Planum temporale and fusiform gyrus of left dominant hemisphere.
**Results:** Compared to the HCs, first-episode psychosis patients showed volume reductions in fusiform gyrus (p=0.000) and pars opercularis (p=0.006) of left hemisphere among language related regions after Bonferoni correction. We found that only the volume of fusiform gyrus of left hemisphere showed significant positive correlation with VIQ (r=0.30, p=0.026) and also showed positive correlation with its subscales (vocabulary subscale (p=0.042), arithmetic WAIS subscale (p=0.012), similarities subscale (p=0.034)). In addition, the volume of fusiform gyrus of left hemisphere were significantly negative correlated with the score of PANSS Negative subscale at 8 weeks (p=0.016) after antipsychotics administration.
**Discussion:** These findings suggest that the fusiform gyrus can be related to the verbal intelligence in first-episode psychosis patients and it may be associated with the severity of negative symptoms after treatment.

### F184. TESTING THE GABA-GLUTAMATE HYPOTHESIS FOR SCHIZOPHRENIA IN RELATION TO AUDITORY HALLUCINATIONS - PRELIMINARY RESULTS

Helene Hjelmervik*[,1], Alexander R. Craven[2], Erik Johnsen[3], Kristiina Kompus[1], Rune A. Kroken[3], Else-Marie Løberg[3], Kenneth Hugdahl[3]
*[1]University of Bergen; [2]University of Bergen, NORMENT Center of Excellence, University of Oslo; [3]Haukeland University Hospital, NORMENT Center of Excellence, University of Oslo; University of Bergen*

**Background:** The gamma-aminobutyric acid (GABA)-glutamate hypothesis of schizophrenia suggests a neurotransmitter imbalance – reduced GABA and increased glutamate - which causes disruption of the modulation between inhibitory GABAergic interneurons and excitatory glutamatergic neurons. In the left superior temporal gyrus (STG) both hyperactivation and increased glutamate levels have previously been associated with auditory hallucinations in schizophrenia patients. However, the STG GABA-glutamate imbalance by simultaneously measuring GABA and glutamate in the same subjects has not previously been tested, and was therefore the aim of the present study. We hypothesized reduced GABA and increased glutamate in the patients relative to controls. Furthermore, reduced GABA and elevated glutamate in STG should be related to severity of auditory hallucinations in these patients.

**Methods:** The current study tested 23 schizophrenia patients (18 hallucinating and 5 non-hallucinating) and 53 healthy controls. The sample included both female and male participants above the age of 18. All patients were on medication, and they were tested at different times relative to the treatment onset. Magnetic resonance spectroscopy (MRS) was used to acquire data from voxels in the right and left superior temporal gyrus (Heschl's gyri) with a 3T GE 750 Discovery MR scanner. PRESS and MEGA-PRESS sequences were applied to measure glutamate and GABA, respectively. Voxel tissue water was used as reference for glutamate and GABA. Scores on the Positive and Negative Syndrome Scale (PANSS) were also collected, and used to differentiate hallucinating from non-hallucinating patients.

**Results:** Separate 2(Group) x 2(Hemisphere) ANOVAs were estimated for GABA and glutamate. No main or interaction effects came out significant for GABA (All $F(1,73)<2.7$, $p>0.1$, $\eta 2<0.03$). The analysis for glutamate resulted in a main effect of Hemisphere ($F(1,74)=24$, $p<0.001$, $\eta 2=0.25$) in which the right STG showed overall higher concentrations than the left STG. In addition, an interaction effect between Group and Hemisphere was found ($F(1,74)=5.22$, $p=0.03$, $\eta 2=0.07$). Bonferroni Post-hoc analysis showed significantly elevated glutamate levels in patients relative to controls in the right STG only ($p=0.005$). Furthermore, a multiple regression analysis was estimated between severity of hallucinations (PANSS P3 item) at the time of testing, and GABA and glutamate values in left and right STG. Although the overall model fit was non-significant, an approximate significant correlation was found between hallucination severity and left STG glutamate levels ($\beta=0.36$, $t=2.1$, $SE=0.17$, $p=0.05$).

**Discussion:** The present study found higher glutamate levels in schizophrenia patients relative to healthy controls in the right STG. In spite of no overall differences in glutamate in the left STG, as initially hypothesized, glutamate levels in this region was found to predict severity of auditory hallucinations. One could speculate that the additional neuronal activity associated with auditory hallucinations elevate glutamate to 'normal levels' corresponding to that of healthy controls. The increased glutamate levels in the right STG seems (linearly) unrelated to auditory hallucinations and future analysis should test whether other symptoms are related to this finding. Overall, the study found only limited support for the GABA-glutamate hypothesis; In spite of increased glutamate in one of the regions, GABA was not found to be reduced in patients and was unrelated to auditory hallucinations.

### F185. BRAIN STRUCTURAL PATTERNS DIFFERENTIATE EARLY- AND LATE-ONSET CANNABIS USE IN PSYCHOTIC PATIENTS: PRELIMINARY RESULTS FROM THE PRONIA STUDY

Nora Penzel*[,1], Peter Falkai[1], Nikolaos Koutsouleris[1], Joseph Kambeitz[1]
*[1]Ludwig Maximilian University*

**Background:** Cannabis use is considered to be one of the most important environmental risk factors for developing psychosis. Previous research indicates that consumption which is initiated during adolescence is associated with a higher risk of adverse effects. It has been proposed that in this period cannabis use may be more harmful due to the disruptive impact on the endocannabinoid system which is critically involved in brain development. Multiple studies indicate structural brain alterations in patients with schizophrenia as well as in cannabis users. However, the effect of cannabis use on brain development in patients with psychosis is currently only poorly understood. Thus, in the current analysis we employed a multivariate approach to investigate the hypothesis that early cannabis use might be associated with marked alterations in brain structure that are distinct from alterations in late-users.

**Methods:** n=39 patients with recent onset psychosis (ROP) and cannabis abuse of the PRONIA sample took part in a structural MRI (sMRI) scan and were clinically assessed with respect to their cannabis use characteristics and psychotic symptoms using the positive and negative symptoms scale (PANSS). Patients were grouped into early-users (n=21, onset before age of 18) and late-users (n=18, onset after age of 18). Multivariate pattern classification was performed on the basis of sMRI data to differentiate early- and late-users.

**Results:** Early- and late-users did not differ with respect to age, gender or amount of current cannabis use. Early-users showed significantly higher PANSS scores compared to late-users ($p < 0.05$). Structural MRI allowed the differentiation between early- and late-users with 72 % (81 % of the early-users, 61 % of the late-users).

**Discussion:** The current results indicate a distinction between psychotic patients with cannabis abuse who started to consume before the age of 18 and those who did later in life. The groups could be distinguished by means both of their clinical data, i.e., more severe psychotic symptoms in the early-users, and of their neuroanatomical data. These findings are in line with former literature, indicating that cannabis use during the period of adolescence is associated with persistent and more severe negative outcomes than use which is initiated in the adulthood. However, due to the relatively low sample size, these results serve only as preliminary results and need further investigation.

### F186. TAPETUM ABNORMALITIES IN FIRST-EPISODE PSYCHOSIS AND RELATIONSHIP TO SYMPTOM SEVERITY AND DURATION OF UNTREATED PSYCHOSIS

Sun Woo Lee[1], Arira Lee[1], Kang-Soo Lee[1], Jeong Hoon Kim[2], Sang-Hyuk Lee*[,1]
*[1]CHA Bundang Medical Center, CHA University; [2]Bundang Jesaeng Hospital*

**Background:** Diffusion tensor imaging (DTI) studies have shown white-matter (WM) abnormalities in most corpus callosum segments in first-episode psychosis (FEP) patients. However, the tapetum, one of its sub-segments, is not fully studied until now. This study focuses on tapetum and its relationship with psychotic disorders using DTI with symptom severities and duration of untreated psychosis (DUP).

**Methods:** Ninety-five FEP patients and thirty healthy controls (HCs) were enrolled. Tapetum, region of interest was extracted using 3D slicer and tract-based spatial statistics (TBSS) were used for DTI analysis. All patients were assessed DUP and clinical symptoms by Scale for the Assessment of Positive Symptoms (SAPS), Scale for the Assessment of Negative Symptoms (SANS) at baseline.

**Results:** TBSS revealed that fractional anisotropy (FA) values of tapetum in FEP is significantly decreased compared to HCs ($p<0.01$, FWE-corrected). Exploratory correlational analysis revealed significant negative correlations between FA values of left tapetum and baseline SAPS total scores ($r =-0.278$, $p<0.05$), bizarre behavior subscale scores ($r =-0.310$, $p<0.01$), positive formal thought disorder subscale scores ($r =-0.348$, $p<0.01$), inappropriate affect subscale scores ($r =-0.315$, $p<0.01$) and SANS avolition-apathy subscale scores ($r =-0.257$, $p<0.05$). Also FA values of left tapetum was negatively correlated with DUP ($r =-0.426$, $p<0.00$).

**Discussion:** FEP patients show a significant reduction in WM integrity of left tapetum, also show correlation with clinical symptoms and DUP. These results suggested that left tapetum may play a role in symptom severities and prognosis in FEP.

### F187. TBSS ANALYSIS OF WHITE MATTER ALTERATIONS IN SCHIZOPHRENIA PATIENTS VS. HEALTHY CONTROLS – RELATION TO AUDITORY VERBAL HALLUCINATIONS

Justyna Beresniewicz*[,1], Rene Westerhausen[2], Erik Johnsen[1], Kenneth Hugdahl[4], Kristiina Comps[1]

[1]University of Bergen; [2]University of Oslo, University of Bergen; [3]University of Bergen, University of Oslo, Haukeland University Hospital

**Background:** Recent Magnetic Resonance Imaging (MRI) studies on schizophrenia suggest that auditory verbal hallucinations (AVH) might be caused by alterations in connectivity of frontal and temporoparietal language-related areas.[3] as well as in connectivity of the default mode network (DMN).[1] Therefore, diffusion tensor imaging (DTI) of white matter fiber tracts, subserving anatomical connections between distant and proximal brain regions, could offer complementary information for understanding the anatomical underpinnings of AVH.
**Methods:** Tract-based spatial statistics (TBSS) allows voxel-wise analysis of multi-subject diffusion data based on fractional anisotropy (FA), assessing microstructural properties of white matter tracts. This study investigates brain white matter tracts in 85 schizophrenia patients and 111 healthy, matched controls using TBSS analysis. Patients were grouped into subgroups (hallucinating and non-hallucinating) based on the Positive and Negative Syndrome Scale (PANSS), with a cut-off PANSS P3 >= 3 in a 12 months period. Additionally, a comparison between the whole patient sample and controls was performed. The whole-brain analysis was performed with permutation analysis of linear models (PALM).[2] Two-tailed t-test was used for group comparison of the patients and controls and a 2 x 2 factorial design was used for hemispheric comparison between patients and controls.
**Results:** TBSS results shows significantly lower fractional anisotropy in the right inferior longitudinal fasciculus in schizophrenia patients in comparison to healthy controls (FDR correction $p < 0.05$). However, after subtracting non-hallucinating patients from the group this effect was no longer present. Hemispheric comparison between patients and healthy controls revealed wide- spread FA reduction in several white matter pathways such as: the corpus callosum (genu and body), cingulum cortex, inferior longitudinal fasciculus, superior corona radiata, anterior thalamic radiation (FDR correction $p < 0.05$). This effect was also present after excluding the non-hallucinating patients from the sample.
**Discussion:** The present findings indicate reduced white matter integrity in schizophrenia patients compared with controls in the inferior longitudinal fasciculus. However, this observation is most likely not related to hallucination proneness since it was not present when comparing the hallucinating patient subgroup with the control group. Hemispheric comparison between patients and controls in both the whole data sample as well as hallucinating group showed significant differences in several white matter pathways. This could indicate that schizophrenia patients (with or without AVH) have altered FA differences between the hemispheres. More research is needed to further understand the implications of these findings.
**References:**
1. Kim, D., Manoach, D., Mathalon, D., Turner, J., Mannell, M., Brown, G.,… Calhoun, V. (2009, November). Dysregulation of working memory and default-mode networks in schizophrenia using independent component analysis, an fBIRN and MCIC study. Human Brain Mapping, 3795–811.
2. Winkler, A. M., Ridgway, G. R., Webster, M. A., Smith, S. M., & Nichols, T. E. (2014). Permutation inference for the general linear model. Elsevier, 381–397.
3. Zhang, L., Li, B., Wang, H., Li, L., Liao, Q., Liu, Y., . . . Tan, Q. (2017). Decreased middle temporal gyrus connectivity in the language network in schizophrenia patients with auditory verbal hallucinations. Neurscience Letters, 177–182.

### F188. THALAMIC MICROSTRUCTURE IN UNAFFECTED RELATIVES OF SCHIZOPHRENIA

Wu Jeong Hwang*[,1], Kang Ik Cho[2], Yoo Bin Kwak[1], Tae Young Lee[3], Jun Soo Kwon[3]
*[1]Seoul National University; [2]Seoul National University, Medical Research Center; [3]Seoul National University College of Medicine*

**Background:** Family, twin, adoption and candidate gene studies all support a genetic component for psychotic disorders. A considerable evidence suggests that the thalamus is abnormal in schizophrenia. The thalamus has a heterogeneous structure with its nucleus having distinct inputs and outputs. Disrupted thalamo-cortical connectivity, in particular, is considered as a core psychopathology in patients with schizophrenia. The disruption is also observed in subjects at clinical-high risk for psychosis. However, using the conventional magnetic resonance imaging methods, it had been difficult to investigate the subtle structural changes that may be present in the thalamus. Furthermore, despite the numerous reports of thalamic abnormalities in schizophrenia, the genetic aspect of the thalamic microstructure has not been thoroughly investigated.
**Methods:** To examine the microstructure of the thalamus, a total of 34 unaffected relatives of schizophrenia (UR) and 33 healthy control subjects underwent diffusion-weighted and diffusion kurtosis magnetic resonance imaging. Using the probabilistic tractography the projections from the thalamus to the lateral and medial prefrontal cortices, lateral and medial temporal cortices, occipital cortex, somatosensory cortex, parietal cortex and orbitofrontal cortex were analyzed. Then, the thalamus was segmented by the projections and the microstructures of those segmented regions were compared between the groups. The mean kurtosis values of the segmented regions were analyzed by analysis of covariance with age and sex as covariates and the results were adjusted with Bonferroni correction.
**Results:** There was no statistical difference in the mean kurtosis values of the left and right thalamic regions projecting to any of the investigated regions between the UR and healthy controls.
**Discussion:** Our findings, via diffusion kurtosis imaging, show preserved microstructural integrity of the thalamus in UR and that this imaging technique may be less well suited to detect thalamic abnormalities in them.

### F189. PERSONALIZED MEDICINE: ESTIMATING THE IMPACT OF GENOTYPES ON ANTIPSYCHOTIC EFFICACY USING A QUANTITATIVE SYSTEMS PHARMACOLOGY APPROACH

Hugo Geerts*[,1], Athan Spiros[1]
*[1]In Silico Biosciences*

**Background:** CNS disorders are lagging behind other indications such as oncology in implementing genotype-dependent treatment algorithms for personalized medicine. This is due to the limited knowledge about the interaction of the relevant biology and the drug's pharmacology
**Methods:** We applied a mechanism-based computer model of a cortico-striatal-thalamocortical loop of the dorsal motor circuit that has been calibrated with clinical data on antipsychotic treatment in schizophrenia patients (Spiros, Roberts et al. 2017). The Quantitative Systems Pharmacology (QSP) model is based on the appropriate connections between basal ganglia regions and consists of 220 neurons (8 different cell types), 3500 synapses and implementations of 32 CNS active targets, based on their unique locations and coupling with intracellular pathways. COMTVal156Met, 5-HTTLPR rs 23351 s/L and D2DRTaq1A1 genotypes are implemented using human imaging data in non-medicated human volunteers
**Results:** The dose-dependent antipsychotic effect for risperidone, aripiprazole and paliperidone is sensitive to the COMT genotype with the MM genotype having the greatest difference with the wild-type. Interestingly the 5-HTTLPR genotype interacts with the COMT genotype: this difference is positive for 5-HTTLPRss and negative for 5-HTTLPR LL. Olanzapine, quetiapine, clozapine and haloperidol are affected much less. The D2DRTaq1A allele interacts in a complex way with the COMT genotype with haloperidol, aripiprazole and risperidone and with the 5-HTLLPR genotype for haloperidol, aripiprazole, risperidone and paliperidone. These effects are anticipated to be detectable in clinical settings.

**Discussion:** The QSP platform predicts strong and complex genotype-dependent interactions with aripiprazole, risperidone, haloperidol and paliperidone and to a much smaller degree with olanzapine, quetiapine and clozapine. These predictions could in principle be verified in clinical setting and could lead to rational personalized treatment guidance

### F190. EFFECT OF SELECTED GENE VARIANTS ON THE RELATIONSHIP BETWEEN EARLY CANNABIS USE AND AGE OF ONSET OF PSYCHOSIS

Rohit Lodhi[1], Yabing Wang[1], Gina McIntyre[1], David Rossolatos[1], Sudhakar Sivapalan[1], Beatriz Henriques[1], Brodie Heywood[1], Candice Crocker[2], Scot Purdon[1], Kathy Aitchison[1], Philip Tibbo*[,3]

*[1]University of Alberta; [2]Dalhousie University, Nova Scotia Health Authority; [3]Capital District Health Authority*

**Background:** Cannabis use, particularly regular use in adolescence, is associated with an increased risk of developing psychosis earlier. An earlier age of onset negates the protective effects of more mature psychosocial and individual variables, thus the potential for worse outcomes. Genetic variations in this relationship are important to understand as this will allow not only a better understanding of the biological interaction of cannabis and psychosis, but would inform future genetic approaches to risk identification as well. We uniquely examined the mediation of this association (gene x cannabis associations in age of onset of psychosis (AoP)) in 3 genetic variants which, while each have been examined separately, not in combination in the same population. We examined: 1) COMT Val158Met (rs4680) 2) BDNF Val66Met (rs6265) and 3) the AKT1 variant rs2494732.
**Methods:** 168 subjects with a diagnosis of psychosis were recruited from 2 sites in Canada, Edmonton Alberta and Halifax Nova Scotia. Cannabis use data (age at first and regular use) were collected using an electronic self-report survey (to address potential minimization of use to a researcher) and saliva samples were used for genotyping. Kaplan-Meier and Cox regression analyses were used to study the gene – cannabis effects.
**Results:** In those who had used cannabis, first use of cannabis prior to 20 years of age was associated with earlier AoP ($p = .005$). In those who used cannabis before age 20, rs4680 had a trend level association with AoP (log rank test: $p=0.0617$). A trend effect for an rs6265 x gender interaction ($HR = 2.08$, $p = 0.067$) on AoP, controlling for regular cannabis use was also observed. No association was observed between rs2494732 and rs6265 - rs2494732 interaction, and AoP.
**Discussion:** The trends in our associations are in keeping with previous literature, however our gender analyses underscores the importance of examining sex and gender as we further move towards risk identification for the cannabis and psychosis interaction. Our results also suggest that not all genetic variants associated with psychosis are involved with the association between cannabis and AoP. While our results offer support for future research in this area, larger sample sizes are required to test the gene-cannabis-AoP relationship.

### F191. THE GENETICS OF DRUG-RELATED MOVEMENT DISORDERS AN UMBRELLA REVIEW OF META-ANALYSES

Nadine van der Burg[1], P. Roberto Bakker*[,2], Jim Van Os[3], Peter Van Harten[2], Asmar Al Hadithy[4]

*[1]GGZ Centraal; [2]Maastricht University; [3]Maastricht University Medical Centre; [4]Parnassia Group*

**Background:** Treatment with antipsychotics can provoke drug-related movement disorders (DRMD) (also known as extrapyramidal symptoms (EPS)), i.e. tardive dyskinesia (TD), parkinsonism, akathisia and tardive dystonia. DRMD remain a cause for concern in the treatment of patients with psychotic disorders, especially because the DRMD can become irreversible (Correll and Schenk, 2008). There are lower percentages in younger patients (32%) (Mentzel 2017), but the prevalence is substantial in chronic patients (68%) (Bakker 2011), with around a quarter of chronic patients showing two different types of DRMD (Bakker 2011). DRMD can cause severe impairment in quality of life (Fujimaki 2012. In addition, a meta-analysis (Ballesteros 2000) and two recent studies showed a higher mortality in patients with tardive dyskinesia (Chong 2009, Dean and Thuras 2009). It is therefore important to find ways of preventing DRMD. Pharmacogenetic studies may identify genetic risk factors, which underlie individual vulnerability for DRMD in response to antipsychotics (Reynolds 2007; Ohmori 2003; Lerer 2002), in theory paving the way for individually tailored medication prescriptions (Lerer and Segman 2006). To date, many different papers have been written on the subject and they have presented inconsistent results.
The aim of this umbrella review is to provide clinicians and patients with evidence-based information regarding the genes that are thought to be associated with DRMD and to use this umbrella review on the genetics of DRMD as a basis for recommendations for future prevention programs and research.
**Methods:** To identify all relevant meta-analyses a Medline, Embase, and Psychinfo literature search was performed. Titles and abstract were screened using predetermined criteria by two independent authors. The methodological quality of included meta-analyses was assessed by two overview authors using 'assessment of multiple systematic reviews' (AMSTAR) critical appraisal checklist. Reference lists of included papers and those of reviews were cross-checked and no new publications were found.
**Results:** The search yielded 14 meta-analysis studies and consensus was obtained. The DRD3, DRD2, CYP2D6, 5-HT2A, COMT and MnSOD genes all contain variants that increase the odds ratio of TD. However meta-analyses showed diminishing significance over time and meta-analyses on the same subject were difficult to compare due to differences in patient population and methods used.
**Discussion:** For now it appears that TD is a complex disease with multiple genes that are involved in its phenotype and more studies (eg. Genome wide associatio studies), on a larger scale, are required to develop a genetic test kit to predict the chance of TD. To achieve this multiple research groups need to work together, a DRMD genetic database needs to be in place to overcome publication bias and results need to be stratified by patient characteristics.
The result could be test that undoubtedly will be of great clinical value in treating patients and by prospectively preventing debilitating DRMD.

### F192. SYSTEMATIC META-ANALYSIS IDENTIFIES FIVE NOVEL ASSOCIATION LOCI FOR SCHIZOPHRENIA

Chenxing Liu*[,1], Tetsu Kanazawa[2], Ye Tian[1], Suriati Saini[1], Christos Pantelis[1], Ian Everall[3], Chad Bousman[4]

*[1]University of Melbourne; [2]Osaka Medical College; [3]Institute of Psychiatry, Psychology & Neuroscience, King's College London; [4]University of Calgary*

**Background:** Schizophrenia is a highly heritable psychiatric disorder. In the past 30 years, thousands of case-control and family-designed association studies have examined candidate genes for schizophrenia. To assist the field in interpreting this large volume of gene-association studies, the online SzGene database was created and included meta analyses for 287 polymorphisms at the time of its final update in 2010. However, since then more than one-thousand new gene-association studies in schizophrenia have been conducted. As such, we have conducted an updated systematic review and meta-analysis of all gene-association studies in schizophrenia published before March 2017.

**Methods:** Studies published between January 2010 and March 2017 were identified using a customized querying strategy. Identified gene-association studies were included in the review and meta-analysis if they: 1) were case-control or family-designed studies with polymorphisms detected in schizophrenia or schizoaffective patients, 2) provide sufficient data to perform meta-analysis, and 3) were not genome-wide association studies (GWAS). Random-effects meta-analysis was performed on polymorphisms with four or more independent studies.

**Results:** Raw data of 1711 studies included in the SzGene database were integrated with 1368 studies identified from our systematic literature search. Random-effects meta-analysis were applied to 540 polymorphisms with at least four studies and 89 of these polymorphisms were nominally associated with SZ (unadjusted $p < 0.05$). After bonferroni correction, 12 polymorphisms remained statistically significant, including five associations not reported in the most recent Psychiatric Genomics Consortium schizophrenia GWAS: 1) rs11098403, p = 1.45e-10, odds ratio = 0.65; 2) rs12807809, p = 7.04e-06, odds ratio = 0.91; 3) rs910694 in PDE4B: p = 1.05e-05, odds ratio = 0.77; 4) rs1801133 in MTHFR: p = 2.99e-05, odds ratio = 1.13 and 5) rs1602565: p = 7.73e-05, odds ratio = 1.11.

**Discussion:** Our results provide a comprehensive and up-to-date review of candidate gene association studies in schizophrenia. Findings complement results from GWASs in schizophrenia but also expand the current list of candidate loci for schizophrenia.

### F193. DYSREGULATION OF RETINOID SIGNALLING IN SCHIZOPHRENIA OBSERVED IN WHOLE GENOME SEQUENCE ANALYSIS

William Reay*,[1], Joshua Atkins[1], Chantel Fitzsimmons[1], Melissa Green[2], Vaughn Carr[2], Murray Cairns[1]
*[1]University of Newcastle; [2]School of Psychiatry, University of New South Wales*

**Background:** Retinoic acid (RA) is intrinsically linked to neurodevelopment and has been implicated in schizophrenia (SZ). This is supported by preliminary trials of a retinoid receptor agonist, Bexarotene, as an adjuvant and the association of five common variants in proximity to members of this pathway at genome wide significance. In addition to these high frequency variants with small effect size, we suspect that this pathway is also affected by rare variants with much higher impact. We aimed to examine the burden of variants in retinoid loci in schizophrenia along with its potential consequences for clinical practice.

**Methods:** Whole genome sequencing was performed on SZ cases (N=331) and non-psychiatric controls (N=167). Cases were further clustered by cognitive measures to derive the cognitive deficit (CD; N=166) and cognitively spared (CS; N=165) subtypes. Disease and subtype associated genomic variation was then analysed in a panel of 129 genes selected for involvement in RA biology (Molecular Signatures Database). Rare variation was aggregated at the gene level using the optimal unified sequence kernel association test (SKAT-O). Clinical metadata was further examined for each case with a rare putative high impact loss of function variant in a RA panel gene predicted using SnpEff. The rare variant burden on target genes of RA receptor binding in SZ was investigated by logistic regression of variants mapped to consensus 5 base pair spaced direct repeat (DR5) retinoic acid response element (RARE) motifs.

**Results:** Gene level rare variant association uncovered suggestive associations with SZ ($P < 0.01$) for three retinoid genes – RBP3, ADH1C and RPE65. In addition, a stronger signal was detected overall for CD cases implicating four additional genes including the RA receptors RARB (P = 1.1 x 10–3) and RARG (P = 9.2 x 10–3). SZ patients with a rare high impact genotype predicted in a RA panel gene were more likely to have serious symptomology as defined by a global assessment of functioning (GAF) score below 50, P = 7.1 x 10–3 (Two-Tailed Fisher's Exact Test). We also found evidence of an increased burden of rare variants within predicted DR5-RARE in SZ (P = 0.017, odds ratio [OR] = 1.094, 95% confidence interval [CI] = 1.023- 1.186), however, there was no significant difference between the cognitive subtypes (CD/CS, P = 0.8, OR = 1.002, 95% CI = 0.961–1.045).

**Discussion:** Our findings suggest that RA mediated control of gene expression is heterogeneously disrupted in SZ by rare variants in DR5-RARE motifs. Strong signals in the context of our sample size further support the possibility of enrichment of rare loci in genes involved with RA biology, particularly in CD cases with impaired cognition. Moreover, we identified a subset of patients with likely high effect size genotypes in the RA pathway. Future work will examine whether these high-risk patients would benefit from retinoid based pharmacological intervention.

### F194. ASSOCIATION STUDY BETWEEN TREATMENT RESPONSE OF AMISULPRIDE AND DOPAMINE D3 RECEPTOR GENE POLYMORPHISMS

Kwanghun Lee*,[1], Won-Myong Bahk[2], Bo-Hyun Yoon[3], Duk-In Jon[4], Sang-Yeol Lee[5], Moon Doo Kim[6], Beomwoo Nam[7], Min-Kyu Song[8]
*[1]Dongguk University Hospital; [2]Yeouido St. Mary's Hospital, The Catholic University of Korea; [3]Naju National Hospital; [4]College of Medicine, Hallym University; [5]Wonkwang University, School of Medicine; [6]School of Medicine, Jeju National University; [7]School of Medicine, Konkuk University; [8]Yesan Mental Health Clinic*

**Background:** The aim of this study is to evaluate the association between rs6280 and rs905568 genetic polymorphism of DRD3 gene and the treatment response of amisulpride.

**Methods:** After six weeks treatment of amisulpride, 125 schizophrenia patients were interviewed based on the Positive and Negative Syndrome Scale (PANSS) and the Clinical Global Impression-Severity (CGI-S). The genotyping for rs6280 and rs905568 was performed using TaqMan single nucleotide polymorphism (SNP) genotyping assay.

**Results:** There was no significant difference in the frequency of genotype and allele of rs6280 between the responders and non-responders based on the total, positive, and general score of PANSS and CGI-S score. However, there was a significant association between this SNP and treatment response in the negative score of PANSS ($\chi 2 = 5.23$, $p = 0.022$). There was no significant association between rs905568 and the response in positive, negative, general, and total PANSS score and CGI-S score.

**Discussion:** This is the first positive association study between DRD3 gene and the treatment response of negative symptoms to amisulpride in Korean schizophrenia patients. A larger scale research on more SNP of the DRD3 gene will make a progress in the study of pharmacogenetics on the treatment response of the amisulpride.

### F195. ENRICHMENT OF PATHOGENIC VARIANTS ASSOCIATED WITH TREATABLE GENETIC DISEASES IN LARGE SCHIZOPHRENIA, BIPOLAR AND DEPRESSION COHORTS

Venuja Sriretnakumar[1], Ricardo Harripaul[1], John Vincent[2], James Kennedy[2], Joyce So*,[3]
*[1]University of Toronto; [2]Centre for Addiction and Mental Health; [3]University Health Network and Mount Sinai Hospital*

**Background:** Genetic diseases are individually rare but collectively common. Many genetic conditions can mimic mental health disorders, with psychiatric symptoms that are difficult to treat with regular medications. Treatment of the underlying genetic disease can cure the associated psychiatric symptoms or help regular medications work better. Discovery of rare genetic diseases in psychiatric patients would reveal specific treatment options, and give information about the chances of other family members being affected. In this study, we test the hypothesis that psychiatric populations are enriched for pathogenic variants associated with selected treatable inborn errors of metabolism (IEMs).
**Methods:** Using targeted next-generation sequencing, we screened schizophrenia (n=1132), bipolar (n=719) and major depressive disorder (n=195) patients for variants in genes associated with Niemann-Pick disease type C (NPC), Wilson disease (WD), homocystinuria (HOM) and acute intermittent porphyria (AIP), and compared the frequency of known and predicted pathogenic variants found to 123 136 samples from the gnomAD consortium.
**Results:** Our study is the first to explore the prevalence of NPC, WD, HOM and AIP gene variants in well-defined psychiatric cohorts. Among 2046 cases (male, n=1106; female, n=940), carrier rates of 0·93%, 0·98% and 0·20% for NPC, WD and HOM were seen, respectively. The carrier rate for NPC was marginally enriched in the SCZ cohort (1·15%) compared to general (95% CI, 0·007 – 0·021; p=0·084) and comparison (95% CI, 1·967 – 5·272; p=5·16e-05) populations. AIP affected rate of 0·29% was observed across the entire psychiatric cohort relative to the general (95% CI, 0·001 – 0·006; p=3·47e-13) and comparison (95% CI, 1·572 – 10·044; p=0·012) populations, an almost 300x enrichment in comparison to what is expected in the general population.
**Discussion:** An enrichment of known and predicted pathogenic variants associated with NPC and AIP was found in the psychiatric cohort, especially in SCZ patients. The results of this proof-of-principle study support that rare genetic disease variants, such as those associated with treatable IEMs, may contribute to the pathogenesis and treatment responsiveness of psychiatric disorders. Discovering genetic diseases in psychiatric patients will shift how health care is delivered to these vulnerable patients by addressing underlying conditions rather than masking symptoms with medications, and has the potential to especially help patients who don't respond to regular psychotropic medications. Further studies screening large psychiatric cohorts for pathogenic variants in a large panel of treatable IEM genes will reveal the full impact of such disorders for psychiatric patients.

### F196. DIFFERENTIAL EFFECTS OF MGLU5 RECEPTOR BLOCKADE ON BEHAVIOR, SCHIZOPHRENIA-RELEVANT GENE EXPRESSION AND NEURONAL ACTIVATION PATTERNS FROM DEVELOPMENT TO AGING MICE

Dragos Inta[1], Alessia Luoni[2], Marco A. Riva[3], Peter Gass*[,1]
*[1]Central Institute of Mental Health; [2]University of Milan; [3]Universita' degli Studi di Milano*

**Background:** The glutamate system is implicated both in schizophrenia and mood disorders. Mice lacking metabotropic mGlu5 receptors (mGluR5 KO) display schizophrenia-like abnormalities. Additionally, mGluR5 antagonists represent promising alternative anxiolytics/antidepressants. However, the underlying age-specific molecular/cellular mechanisms are only partially understood. We aimed at identifying molecular alterations associated with a genetically induced mGluR5 deletion, which results in a schizophrenia-like phenotype. Additionally, we investigated age-specific effects of mGluR5 antagonists on emotional behaviour and c-fos activation.
**Methods:** For analysis of mRNA and protein levels we performed Real-time RT-PCR and Western blot investigations in the hippocampus and prefrontal/frontal cortex (PFC/FC) of mice with a genetic deletion of the metabotropic glutamate receptor 5 (mGlu5), addressing key components of the GABAergic and glutamatergic systems. Additionally, we used classical behavioral tests for determining anxiety- and depression-like changes triggered by the mGluR5 antagonist 2-Methyl-6-(phenylethynyl)pyridine (MPEP). Finally, we used profiling of c-Fos expression, as marker of neuronal activity, induced by MPEP from postnatal day 16 (P16) to adulthood (P90).
**Results:** mGlu5 knockout (KO) mice showed a significant reduction of reelin, GAD65, GAD67 and parvalbumin mRNA levels, which is specific for the PFC/FC, and that is paralleled by a significant reduction of protein levels in male KO mice. We also analysed the main NMDA and AMPA receptor subunits, namely GluN1, GluN2A, GluN2B and GluA1, and observed that mGlu5 deletion determined a significant reduction of their mRNA levels, also within the hippocampus, with differences between the two genders. We measured age-specific alterations in emotional behaviour of mGluR5 KO mice, with marked increase of anxiety during aging. There was a remarkably conserved activation of the paraventricular nucleus of the hypothalamus, implicated in stress regulation, by MPEP at all investigated ages, whereas the extended amygdala was specifically activated in adulthood only.
**Discussion:** Our data suggest that neurochemical abnormalities impinging the glutamatergic and GABAergic systems may be responsible for the behavioral phenotype associated with mGlu5 KO animals and point to the close interaction of these molecular players for the development of neuropsychiatric disorders such as schizophrenia. These data could contribute to a better understanding of the involvement of mGlu5 alterations in the molecular imbalance between excitation and inhibition underlying the emergence of a schizophrenic-like phenotype and to understand the potential of mGlu5 modulators in reversing the deficits characterizing the schizophrenic pathology.

### F197. PROMOTING MYELIN REPAIR RESCUES MICE FROM SCHIZOPHRENIA-LIKE BEHAVIOR INDUCED BY SOCIAL ISOLATION

Lan Xiao*[,1]
*[1]Third Military Medical University*

**Background:** Although pathological and genetic evidence suggest that oligodendrocyte (OL) or myelin deficits are associated with schizophrenia, the contribution of OL/myelin deficits to its etiology has not been clearly dissected, because OL/myelin abnormalities may be a concomitant phenomenon during the pathogenesis of schizophrenia.
**Methods:** Using olig2 ablation specifically in OLs (olig2 CKO) mice, we detected myelin development status and animal behaviors under normal condition or subjected to social isolation. We also examined the therapeutic effect of FDA-approved compounds, like quetiapine (an APD) or clemastine (a histamine antagonist) on animal behaviors.
**Results:** Our results demonstrated that deleting of olig2 leaded to impaired development of OLs and myelin deficit from postnatal day14 (P14) to P56, preferentially in cerebral cortex, and these young adult Olig2 KO mice showed anxiety-like behavior, motor skill learning deficit and cognitive deficit. Moreover, Olig2 CKO mice exhibited earlier social avoidance behavior than the WT littermates under prolonged social isolation, indicating that myelin deficit may enhance risk of schizophrenia upon environmental stress attacking. Interestingly, enhancing oligodendrocyte generation and myelin repair by quetiapine or clemastine successfully reversed the above phenotype.
**Discussion:** Taking together, promoting myelin repair may present a new therapeutic strategy against schizophrenia.

### F198. EFFECTS OF CANNABINOIDS ON A HUMAN OLIGODENDROCYTE CULTURE: IMPLICATIONS FOR SCHIZOPHRENIA

Valéria Almeida*[,1], Gabriela Seabra[1], Jose Alexandre Crippa[2], Jaime Eduardo Hallak[3], Antonio W. Zuardi[3], Daniel Martins-De-Souza[1]

[1]University of Campinas (UNICAMP); [2]University of São Paulo; [3]University of São Paulo, National Institute for Translational Medicine

**Background:** Preclinical and clinical studies have suggested the involvement of the endocannabinoid system in schizophrenia pathobiology. In addition, in vitro studies have shown the molecular pathways and biological processes associated with cannabinoids' effects in some cell types, such as glial cell cultures. Thus, the effects of cannabinoid drugs on these cells may contribute to our knowledge about the pathobiology of schizophrenia. Specifically, oligodendrocytes are associated with white matter deficits in schizophrenia. The modulation of their function, survival, and differentiation can result in new approaches to treat schizophrenia's white matter-associated deficits. Here we have investigated the effects of cannabidiol (CBD) on a human oligodendrocyte culture (MO3.13). In another experiment, we pretreated the MO3.13 with MK801, an in vitro model of study schizophrenia proposed by our group, in terms of protein expression.
**Methods:** MO3.13 oligodendrocytes were treated with CBD (1μM), or MK801 (50 μM) followed by CBD (1 μM). After 8 hours, proteins were extracted from these cells, digested, and processed in a state-of-the-art LC-MS/MS system. Quantitative proteomics approaches were then employed in a label-free fashion. Differentially expressed proteins were analyzed using systems biology in silico tools.
**Results:** Analyses identified that several proteins were up- or down-regulated in response to CBD treatment. These proteins were analyzed in terms of biological processes, pathways, and functions. CBD affected the expression of 136 proteins. Some proteins such as the transient receptor potential channel, microtubule-associated proteins, Rho GTPase activating proteins (21 and 23), and the calcium channel, voltage-dependent T type alpha 1H subunit, among others possibly involved in myelination process, were increased by CBD. Additionally, the MK801-treatment decreased proteins of cytoskeleton, microtubule and RHO GTPases activate KTN1. MK801 also increased proteins involved in glycolysis and eukaryotic translation initiation and CBD attenuated these changes.
**Discussion:** Studies have shown the effects of CBD on the treatment of schizophrenia; but the mechanisms involved in its antipsychotic properties are not fully understood. Herein, we observed that CBD modulated the expression of proteins that can be implicated in schizophrenia pathobiology. For instance, MAPs functions are related to cytoskeleton organization, differentiation, and migration of oligodendrocytes. Studies have shown a decrease of MAPs in schizophrenia patients; thus, increasing MAP2 and MAP4 by CBD may be an interesting mechanism to treat and prevent cytoskeleton impairments in oligodendrocytes and neurons in schizophrenia. Moreover, CBD increased the voltage gated channel that is involved in cannabinoid retrograde signaling and glutamate and GABAergic neurotransmission. CACNA1H modulates Ca2+ levels and the synaptic vesicle cycle. To note, we also found effects of CBD on pathways and biological processes involved with schizophrenia pathobiology, such as glucose metabolism, axon guidance, and inflammation mediated by cytokine signaling. In relation to MK801-treatement, we observed that affected proteins involved in glycolysis and CBD attenuated this change, like antipsychotics (as demonstrated in Cassoli et al., 2016). Moreover, MK801-treatment affected the RHO GTPases family that has been implicated in schizophrenia, and CBD increased these proteins. In summary, these proteomic findings may provide an integrated picture of the role of endocannabinoid signaling in oligodendrocyte cells and possible implications for schizophrenia's pathobiology.

### F199. O-GLCNAC DYSREGULATION IN SCHIZOPHRENIA CORTEX

Toni Mueller*,[1], Anita Pinner[1], James Meador-Woodruff[1]
[1]University of Alabama at Birmingham

**Background:** O-linked β-linked N-acetylglucosamine (O-GlcNAc) is a post-translational glycosylation modification with ubiquitous functions in cell biology. The attachment of GlcNAc to serine or threonine (S/T) residues transiently adorns thousands of nuclear, cytosolic, and mitochondrial proteins and modulates protein function and localization via dynamic cooperation with kinase and phosphatase enzymes. O-GlcNAc transferase (OGT) exists in complex with S/T phosphatase subunits PP1β and PP1γ and, along with the activity of O-GlcNAcase (OGA), facilitates rapid cycling between O-GlcNAcylation and phosphorylation states to serve as an "on/off switch" for substrate activation. Altered levels of OGT, OGA, and/or O-GlcNAc have been shown to influence many pathways pertinent to schizophrenia (SZ) pathophysiology. Notably, elevated O-GlcNAc and enhanced O-GlcNAcylation mediate glucose tolerance and insulin resistance which can lead to diabetes, an illness often found comorbid with SZ. Elevated O-GlcNAcylation can also produce mitochondrial abnormalities consistent with those identified in SZ. In an exploratory study of glycosylation enzyme transcript expression, our lab found OGT mRNA levels 253% higher in SZ than non-psychiatrically ill comparison (COMP) subjects ($p < 0.0001$). Based on this evidence, we hypothesized that OGT protein levels or the ratio of OGT:OGA enzymes are elevated in SZ brain.
**Methods:** Expression of OGT and OGA were measured using western blots of superior temporal gyrus (STG; Brodmann Area 22) homogenates from sex- and age-matched pairs of SZ and COMP subjects ($N = 17$). Standard immunoblotting methods and commercially available antibodies were used to detect the targets of interest and protein levels were normalized to intra-lane valosin containing protein (VCP) expression; VCP expression has previously been found to be unchanged in SZ STG.
**Results:** In the current study, we found OGA protein levels reduced 18% in SZ ($p < 0.01$) and SZ subjects demonstrate a trend toward increased ratios of OGT:OGA ($p = 0.05$). OGT was not different between groups ($p = 0.77$).
**Discussion:** Our current results partially support our original hypothesis that an altered ratio of OGT:OGA may contribute to abnormalities of O-GlcNAcylation and consequent cellular metabolic abnormalities in SZ. A trend toward increased OGT:OGA along with decreased expression of OGA would produce the same functional outcome as the originally predicted OGT increase: upregulation of protein O-GlcNAcylation. Given that the mRNA study used samples of dorsolateral prefrontal cortex (DLPFC) while our protein-level measures were from STG, it is not inconceivable that potential O-GlcNAc dysregulation could arise from upregulated OGT in one brain region, but downregulated OGA in another. To elaborate on these findings, we will investigate OGT and OGA expression in the DLPFC and will assess total O-GlcNAcylation in both brain regions to determine functional consequences of altered enzyme expression.

### F200. ELUCIDATING THE ROLE OF CILIA IN NEUROPSYCHIATRIC DISEASES THROUGH INTERACTOME ANALYSIS

Madhavi Ganapathiraju*,[1], Srilakshmi Chaparala[1], Cecilia Lo[1]
[1]University of Pittsburgh

**Background:** Cilia are microtubule-based organelles present on the surface of many eukaryotic cell types critical for tissue homeostasis and proper organ development. Ciliary dysfunction underlies a growing list of human diseases and disorders collectively called ciliopathies such as Bardet-Biedl syndrome (BBS), Joubert syndrome, Meckel–Gruber syndrome and primary ciliary dyskinesia. Many ciliary proteins are associated with neuronal function consistent with neuronal developmental delays, cognitive, learning, and memory deficits observed in several ciliopathies, suggesting that ciliary dysfunction may contribute to pathogenesis of neuronal diseases and that an understanding of how ciliary proteins function together as a system would provide much needed mechanistic insights into their molecular etiologies.
**Methods:** We constructed protein-protein interaction (PPI) networks of genes associated with cilia and those associated with 7 neuropsychiatric diseases: schizophrenia, attention deficit hyperactivity disorder, major depressive disorder, bipolar disorder, autism spectrum disorder, Alzheimer's disease and Parkinson's disease. The interactome is constructed with experimentally determined PPIs from BioGRID and HPRD databases and novel PPIs predicted using our High-confidence PPI Prediction (HiPPIP) model. We previously presented Schizophrenia Interactome constructed using HiPPIP andalso showed that novel PPIs are highly accurate based on computational and experimental validations. We validated additional PPIs of cilia interactome here. We computed how closely connected cilia is to genes associated with neuropsychiatric diseases, through interactome and pathway analysis. Additionally, we analyzed drugs that proteins in the cilia interactome, and found that majority of these drugs are nervous system associated drugs.

**Results:** The ciliary protein interactome consists of 165 ciliary proteins with 1,011 known PPIs and 765 novel PPIs. We found the overlap between cilia and neuropsychiatric interactomes to be statistically highly significant. For e.g., cilia interactome has an overlap of 125 genes with schizophrenia interactome of which 26 are novel interactors of cilia, and has significant overlap with pathways relevant to schizophrenia. About 184 genes in the cilia interactome are targeted by 548 FDA approved drugs, of which 103 are used to treat nervous system diseases.

**Discussion:** Ciliary genes like DRD1 and DRD2 are implicated in neurotransmission and associated with schizophrenia. DRD1 has 4 novel interactors and DRD2 has 12 novel interactors that may have significant role in the pathology of mental disorders. Neuronal pathways associated with cilia interactome with high statistical significance such as dopamine signaling, eNOS signaling, synaptic long-term potentiation pathways are known to be associated schizophrenia. Wnt signaling and PCP signaling are also known to be associated with cilia mediated neurodevelopmental signaling, defects in these pathways contributing to schizophrenia. Novel interactions for cilia proteins validated by experiments have functional significance in association with cilia and neuronal disorders. For e.g., IFT88, a cilia protein required for cilia assembly, is critical for SHH signaling, cell cycle regulation and cerebellar development and is also associated with schizophrenia and bipolar disorder. CACNA1I is predicted to interact with DNAL4 and MKS1, both involved in transport of proteins required for ciliogenesis. GWAS studies show that CACNA1I is associated with schizophrenia. Taken together, the cilia interactome presented here provides novel insights into the relationship between ciliary protein function and neuropsychiatric diseases.

### F201. KINASE NETWORK DYSREGULATION IN SCHIZOPHRENIA: IMPLICATIONS FOR NEW TREATMENT STRATEGIES

Eduard Bentea*[,1], Erica Depasquale[2], Jarek Meller[2], Zhexing Wen[3], Robert E. McCullumsmith[1]
*[1]University of Cincinnati; [2]Cincinnati Children's Hospital Medical Center; [3]Emory University School of Medicine*

**Background:** Disrupted-in-schizophrenia 1 (DISC1) is one of the most substantiated genetic risk factors for schizophrenia (SZ). A large array of animal studies supports an etiopathogenic role of DISC1, by linking it with regulation of processes such as synapse formation and neuronal development. However, much less is known regarding the involvement of DISC1 in human neurons. Induced pluripotent stem cells (iPSCs) generated from patients carrying the disease have emerged as powerful tools to study cellular dysfunction in a disease-relevant context. In this study, we investigated serine/threonine kinase networks in a human iPSC model of DISC1-related SZ.

**Methods:** PamChip arrays evaluate kinase activity by measuring phosphorylation levels of a series of immobilized peptide sequences during exposure to kinases in the sample. We employed PamChip arrays to map the serine/threonine sub-kinome of neuronally differentiated iPSCs generated from a patient with SZ presenting the frame-shift DISC1 mutation (D2-1), an unaffected family member without the mutation (C3-1), as well as of isogenic iPSC lines in which the mutation was either corrected in D2-1 (resulting in the cell line D2-R), or introduced in C3-1 (resulting in the cell line C3-M). Using a bioinformatics workflow that identifies kinase hits using a random sampling model, we identified kinases that emerged as common hits after comparing D2-1 with D2-R (changed after rescuing the mutation in the patient cell line) and C3-M with C3-1 (changed after introducing the mutation in the control cell line). We used the resulting kinase network to identify pathways, perturbagens, and drugs related to the disease phenotype.

**Results:** By comparing D2-1 to D2-R, 9 peptide sequences were identified to be differentially phosphorylated at a +/- 1.15 fold-change level. After assigning upstream kinases to these peptides and generating the random sampling model, we identified 3 kinase subfamilies which were over-represented in D2-1 vs. D2-R: TAO, KHS and 5' adenosine monophosphate-activated protein kinase (AMPK). By comparing C3-M to C3-1, we could identify 13 peptide sequences differentially phosphorylated at a +/- 1.15 fold-change level. Mapping these sequences to upstream kinases and running the random sampling model, led to the identification of 9 kinase subfamilies over-represented in C3-M vs. C3-1: AMPK, TAO, BUD32, WNK, KHS, RAD53, CK1, NEK and MLK. By overlapping the results, we could identify a set of 3 kinase subfamilies (TAO, KHS, and AMPK) commonly changed between the two methods of comparison. Ingenuity pathway analysis identified post-translational modification, cell signaling, cell morphology, cell cycle, and cellular assembly and organization, as the top functions of the DISC1 kinase network.

**Discussion:** Kinases are potent modulators of intracellular signaling that control patterns of gene expression, cytoskeletal dynamics, function of neurotransmitter systems and cellular metabolism, which may be of relevance to the etiopathogenesis of mental disorders, such as SZ. Herein, we characterized the serine/threonine sub-kinome of neuronally differentiated iPSCs from a patient with SZ presenting with a 4-bp deletion in DISC1. Using gene editing we created isogenic cell lines to either rescue the mutation in the patient cell line, or introduce the mutation in iPSCs obtained from an unaffected family member, to strengthen causality for the DISC1 mutation. This approach led to the identification of 3 kinase subfamilies as common hits of the DISC1 phenotype: TAO, KHS, and AMPK. Our unbiased approach led to the novel identification of kinases implicated in DISC1-related SZ. Further validation of these findings may open new avenues for treating this highly disabling neuropsychiatric disorder.

### F202. ABNORMAL REMODELING PROCESSING IN NEURAL GPI-APS SECRETORY PATHWAY IN SCHIZOPHRENIA

Pitna Kim*[,1], James Meador-Woodruff[1]
*[1]University of Alabama at Birmingham*

**Background:** Abnormalities in post translational modifications (PTMs) such as glycosylation have become targets of schizophrenia (SCZ) research and are implicated in the neuropathophysiology of this illness. Glycosylphosphatidylinositol (GPI) attachment to proteins and glycoproteins events; proteins essential to cellular function, including neurotransmitter rreceptors, adhesion molecules, and enzymes, are all modified by GPI. Biosynthesis of GPI -APs occurs in the endoplasmic reticulum (ER). Once GPI-APs are synthesized, they are transported from the ER to the cell surface through the Golgi apparatus. Inositol deacylation of the GPI-APs by is common PTMs. The GPI-anchored proteins (GPI-APs) play an essential role in many biological PGAP1 is required for efficient export from the ER and acts a molecular mechanism for quality control of GPI-APs. The p24 complex binds specifically to GPI-APs and plays a role in their selective trafficking by sensing the status of the GPI anchor and to promote efficient ER exit of remodeled GPI-APs. In this study, we identified abnormalities of proteins associated with the ER exit of GPI-APs in SCZ. To address mechanisms of GPI-APs ER exit, we measured expression of proteins of the GPI-APs ER exit and targeting pathway. We also measured expression of GPI-APs which have been previously implicated in SCZ, including GPC1, NCAM, MDGA2 and EPHA1.

**Methods:** We assessed the total expression and subcellular localization of proteins involved in ER export processing of GPI-APs from the DLPFC of 15 matched pairs of SCZ and comparison subjects. Specifically, we measured levels of PGAP1 and Tmp21 (p24). Additionally, we performed a Triton X-114 phase separation to distinguish between membrane-associated and cytosolic forms of protein substrates. We confirmed the sensitivity of each target GPI-AP to phosphatidylinositol-specific phospholipase C (PI-PLC), an enzyme that specifically cleaves GPI from GPI-APs.
**Results:** We found a significant decrease in p24 in total tissue homogenates and PGAP1 in an ER enriched fraction from subjects with SCZ. We also identified diminished sensitivity of the GPI-APs, GPC1 and NCAM, to PI-PLC treatment in SCZ.
**Discussion:** Decreased PGAP1 in an ER enriched fraction in consistent with reduced inositol deacylation and potential dysfunction as the gatekeeper of GPI-AP ER exit in SCZ. This also suggests that the GPI-anchor is not correctly modified. Decreased p24 levels suggest downregulation of transport between the Golgi and the ER in SCZ. Additionally, we observed unchanged total level of GPI-APs in Triton X-114 phase separation, but a significant decrease in the amount of NCAM and GPC1 that was sensitive to PI-PLC in SCZ. This finding may be consistent with abnormal GPI modification of these two candidate proteins. Together, these findings suggest dysregulation of the GPI-APs remodeling system in SCZ, which may impact the structure of the GPI-anchor for SCZ-relevant proteins like NCAM and GPC1.

### F203. A META-ANALYSIS OF MINOR PHYSICAL ANOMALIES IN FIRST-DEGREE UNAFFECTED RELATIVES OF PATIENTS WITH SCHIZOPHRENIA

Ozge Akgul*,[1], Emre Bora[2], Berna Binnur Akdede[2], Köksal Alptekin[1]
*[1]Dokuz Eylul University; [2]Dokuz Eylül University School of Medicine*

**Background:** Neurodevelopmental abnormalities are common in schizophrenia. Minor physical anomalies (MPAs) are associated with abnormalities in neural development. Previous studies clearly demonstrated that MPAs are significantly increased in schizophrenia. However, the available evidence in unaffected relatives of patients with schizophrenia is contradictory.
**Methods:** A literature search was conducted between 1 JAN 1980 and SEP 2017 in PUBMED and SCOPUS. Random-effects model was used. Heterogeneity was tested with Q test and I2. The meta-analysis was conducted using OpenMetaAnalyst software.
**Results:** 16 studies were included in the meta-analysis. MPAs were significantly more common unaffected first-degree relatives of patients with schizophrenia (d=0.56, CI=0.40–0.73, p<0.001). There was a significant heterogeneity in distribution of effect sizes (Q=42.2, p<0.001). The level of this heterogeneity was medium in range (I2=64 %). In meta-regression analyses, demographic variables were not significantly related with magnitude of the effect size.
**Discussion:** MPAs are associated with risk of schizophrenia. However, the level of heterogeneity suggests that risk of psychosis is associated with neurodevelopmental abnormalities in some but not all individuals. Findings also emphasize that resilience factors might be protecting many neurodevelopmentally impaired relatives of schizophrenia against having a full-blown psychotic disorder.

### F204. THE DANISH HIGH-RISK AND RESILIENCE STUDY - VIA 7 - A PROSPECTIVE COHORTE STUDY OF 522 7 YEARS OLD CHILDREN BORN TO PARENTS DIAGNOSED WITH SCHIZOPHRENIA OR BIPOLAR DISORDER - RESULTS ON PSYCHOPATHOLOGY, COGNITION AND LIVING CONDITIONS

Anne Amalie Thorup*,[1], Nicoline Hemager[2], Ditte V. Ellersgaard[2], Camilla Jerlang Christiani[2], Birgitte Klee Burton[2], Katrine S. Spang[2], Maja Gregersen[1], Anne Søndergaard[2], Ditte L. Gantriis[3], Aja Greve[3], Jens Richardt Jepsen[4], Ole Mors[3], Kerstin von Plessen[5], Merete Nordentoft[2]
*[1]Child and Adolescent Mental Health Center Capital Region of Denmark; [2]Mental Health Center, Capital Region of Denmark, The Lundbeck Foundation Initiative for Integrative Psychiatric Research (iPSYCH); [3]Aarhus University Hospital, The Lundbeck Foundation Initiative for Integrative Psychiatric Research (iPSYCH); [4]Center for Neuropsychiatric Schizophrenia Research, Child and Adolescent Mental Health Center, Mental Health Services, Capital Region of Denmark; [5]University Hospital Lausanne*

**Background:** For decades familial high-risk studies have informed us about genetic and environmental risk factors for schizophrenia and recently also bipolar disorder. Familial high-risk studies are important and relevant and may represent a possible shortcut to learning more about early markers of illness, mental vulnerability and resilience.
**Methods:** The Danish High Risk and Resilience Study – VIA 7 is a prospective cohort study of 522 7-year old children, 202 of them born to at least one parent diagnosed with schizophrenia in the Danish registries, 120 of them born to a least one parent diagnosed with bipolar disorder and 200 of them born to parents without any of these diagnoses. A comprehensive battery has been used combining assessments from several domains for both parents and children.
**Results:** Results show that children born to parents with schizophrenia or bipolar disorder have higher frequencies of early mental problems. Further there are marked differences between the three groups concerning neuro cognition, motor functioning and living conditions including socioeconomic status, early risk factors and home environment - all factors that are known to be important with regard to healthy child development.
**Discussion:** First results from the VIA 7-study indicate that many children and families have unmet needs and problems. Perspectives are two-fold: we aim to follow the cohort and conduct a new assessment before puberty (at age 11). Simultaneously, we are evolving an early, integrated, specialized and family based intervention, called VIA Family, to prevent or ameliorate development of severe mental illness in individuals born to parents with schizophrenia or bipolar disorder.

### F205. OLFACTORY IDENTIFICATION IN 7-YEAR OLD CHILDREN AT FAMILIAL RISK TO DEVELOP SCHIZOPHRENIA

Anna Hester Ver Loren van Themaat*,[1], Jens Richardt Møllegaard Jepsen[2], Camilla Christiani[3], Merete Nordentoft[3]
*[1]University of Copenhagen; [2]Mental Health Services – Capital Region of Denmark, Child and Adolescent Mental Health Centre, The Lundbeck Foundation Initiative for Integrative Psychiatric Research, Center for Clinical Intervention and Neuropsychiatric Schizophrenia Research; [3]Mental Health Centre Copenhagen*

**Background:** Olfactory dysfunction has repeatedly been observed in individuals diagnosed with schizophrenia. The most stable and consistent finding on the behavioral level is that of smell identification deficits. However, the nature of olfactory identification abnormalities seems to extend to structural abnormalities in the underlying neurobiology of the olfactory system. Furthermore, smell identification deficits are also documented in first-episode patients and non-psychotic first-degree relatives of schizophrenia patients. Family members of schizophrenia patients also show structural abnormalities of the olfactory system, suggesting that these may serve as an endophenotype for the development of schizophrenia.

Only a few studies examined the olfactory identification ability in adolescents at-risk for schizophrenia and suggested smell identification deficits as a risk marker for schizophrenia. These studies included adolescents at clinical as well as at genetic risk for schizophrenia. None of these studies focused on children at genetic risk for schizophrenia. Therefore, we investigated the olfactory identification ability in children of parents with schizophrenia in comparison to children of parents without a psychotic disorder. As we are also interested in the specificity of the olfactory impairments to schizophrenia, we included children of parents with bipolar disorder. We hypothesize that children at genetic risk for schizophrenia would have the most severe smell identification deficits and that children of bipolar disorder patients would have less severe deficits than the at-risk for schizophrenia group but more severe than the group of children without a psychotic parent.
**Methods:** Participants - The olfactory identification ability was assessed in 202 children of schizophrenia patients ('children at familial risk for schizophrenia') in relation to that of 200 children of parents without a psychotic disorder ('controls'). In addition, we also assessed the B-SIT in 120 children of bipolar disorder patients ('children at familial risk for bipolar disorder'). All children were 7 years of age at the time of assessment and they were part of the Danish High Risk and Resilience Study – VIA7.
Brief Smell Identification Test - The Brief Smell Identification Test (B-SIT) contains 12 items that need to be scratched and sniffed. The test has excellent reliability (> 0.80) and demonstrates agreement for abnormal olfaction comparing B-SIT with the San Diego Odor Identification Test (SDOIT). A maximum score of 12 reflects intact olfactory identification functioning. B-SIT has been conducted in patients with neurodegenerative disorders (Parkinson's disease and Alzheimer's disease) and can be used for individuals above 5 years of age.
Statistics - We will use analysis of covariance (ANCOVA) for analysis of the B-SIT total scores with 'diagnosis of parent' as the independent variable and age and sex as covariates for the three groups.
**Results:** Analyses will be performed within the next 3 months so can be presented in April 2018.
**Discussion:** Conclusion and discussion cannot be drawn at this time.

### F206. A TRANSLATIONAL STUDY OF BEHAVIOR, BRAIN STRUCTURE AND GENE PATHWAY IN ERBB4 KNOCKOUT MICE AND FIRST-EPISODE TREATMENT-NAÏVE PATIENTS WITH SCHIZOPHRENIA

Chengcheng Zhang*[,1], Peiyan Ni[2], Tao Li[2]
*[1]West China Hospital Sichuan University; [2]Mental Health Center, West China Hospital of Sichuan University*

**Background:** The current study was to explore how disruption of specific molecular circuits in the cerebral cortex may cause large-scale brain structure deficits and behavior changes via a translational study in conditional Erbb4 mutant mice and patients with schizophrenia.
**Methods:** We conducted prepulse inhibition (PPI) and brain structural and diffusion magnetic resonance imaging (MRI) scans in 27 mice with ErbB4 knockout in parvalbumin (PV) interneurons and 23 age, sex-matched controls. Real-time quantitative polymerase chain reaction was used to assess the levels of five GABA-related transcripts in brain regions. We also measured structural and diffusion MRI and cumulative contribution of risk alleles in the GABA pathway genes using polygenic risk scores (PRS) in first-episode treatment-naïve schizophrenic patients (N=117) and age, sex-matched healthy controls (N=86).
**Results:** ErbB4 knockout mice displayed behavioral deficit of PPI, as well as gray and white matter impairment in right sensorimotor cortical-striatal networks. We found significant correlations between gray matter volumes (GMVs) of the somatosensory cortex and PPI as well as GAD1 mRNA expression in controls but not in knockout mice. These findings were confirmed in a human sample where we observed significantly decreased gray and white matter impairment in sensorimotor cortical-striatal networks in schizophrenics. The PRS of GABA-pathway genes also displayed a negative correlation with the GMVs of the somatosensory cortex in patients.
**Discussion:** Our study identified ErbB4 ablation induced prepulse inhibition deficits and GABAergic dysregulation in sensorimotor cortical-lateral striatal networks. We propose that ErbB4 signaling participates in sensorimotor gating dysfunction in schizophrenia by getting involved in somatosensory cortex deficits and GABAergic dysfunction.

### F207. SCHIZOTYPY AND SENSORY GATING: A 6-MONTH-OLD EEG STUDY

Eleanor Smith*[,1], Trevor Crawford[1], Megan Thomas[2], Vincent Reid[1]
*[1]Lancaster University; [2]Blackpool Victoria Teaching Hospital NHS Foundation Trust*

**Background:** Schizotypal traits are present in the general population and are distributed along a continuum, with the clinical disorder schizophrenia found at its extremity (Claridge, 1997). Schizotypy is a dimension of personality within the general population, which has been found to be elevated among schizophrenia-spectrum patients (Brosey and Woodward, 2015) and their first-degree relatives (Morenzo-Izco et al., 2015). One hypothesis to account for the sensory deficits observed across the spectrum suggests a difficulty in the inhibition of irrelevant sensory input, such as the secondary beep in the paired-click paradigm.
Sensory gating describes the pre-attentional habituation of responses to repeated sensory input, for example, auditory tones. This gating mechanism is used to distinguish between important and irrelevant information (Hall et al., 2011) and is typically explored using the paired-click paradigm and analysed using the P50 event-related potential component. This can be observed approximately 50-milliseconds following the presentation of an auditory stimulus and is a highly established biological trait of schizophrenia, with abnormalities displayed in the P50 component all throughout the schizophrenia-spectrum.
**Methods:** This research aimed to observe whether the 6-month-old offspring of mothers with schizotypic traits display abnormalities in the P50 event-related component when explored using the paired-click paradigm. The paired-click paradigm was used to highlight the sensory-gating abilities of fifty-three 6-month-old infants during 15-minutes of continuous sleep. The mothers of the infants completed the Short Form of the Oxford and Liverpool Inventory of Feelings and Experiences, which was used to determine their personality dimension scores, and identify schizotypic traits. Participants were categorized into one of three groups: infants of controls mothers, infants of intermediate mothers, and infants of schizotypic mothers.
**Results:** It was predicted that the 6-month-old infants of mothers who demonstrate schizotypy scores would demonstrate different amplitudes compared to those of control mothers. This research found a significant generalized difference between the P50 component for the paired-clicks in the right hemisphere of the brain ($F(1,51)=5.34$, $p=.025$), and a significant latency effect was observed in the frontal regions ($F(1,51)=5.41$, $p=.024$). A significant between-subjects effect was observed centrally ($F(2,50)=3.71$, $p=.031$); suggesting there are significant differences between the ways each group distinguished the paired-clicks. Infants of schizotypic mothers showed an increase in activation compared to other groups. An interaction was observed in the left hemisphere between the paired-clicks and each identifiable group ($F(2,50) = 3.45$, $p = .039$). In addition to the P50 a significant slow wave effect was also observed across the left ($F(1,51)=8.38$, $p=.006$) and right ($F(1,51)=7.81$, $p=.007$) posterior regions; a latency effect in the left ($F(1,51)=5.47$, $p=.023$), and a distinction in mean amplitude in the right ($F(1,51)=7.25$, $p=.010$).
**Discussion:** Schizotypy is viewed as a risk factor for schizophrenia, which is present in the general population, and is present on the schizophrenia-spectrum. The 6-month-old infants of mothers showed an increase in activity centrally, demonstrating that the infants' P50 amplitudes were influenced by their mothers' schizotypy status. This finding is consistent with the developmental hypothesis of schizophrenia, however longitudinal studies will be required to determine whether a sensory gating deficit is a valid predictor of the later onset of schizophrenia.

### F208. COGNITION, POSITIVE SYMPTOMS, AND INTERNET USE FOR MENTAL HEALTH IN PEOPLE WITH PSYCHOSIS

Kristi-Ann Villagonzalo[1], Chelsea Arnold[1], Fiona Foley[1], Denny Meyer[1], John Farhall[2], Susan Rossell *,[1], Neil Thomas[1]
[1]*Swinburne University of Technology;* [2]*La Trobe University*

**Background:** People with severe mental illness are increasingly using digital resources for mental health, including social media and online interventions. However, individuals' ability to engage with or benefit from such resources may be impaired by deficits in cognition and insight, and experiences of psychotic symptoms, including paranoia about cyber-security or motives of others in online social interactions. This study aimed to explore the association between cognition, positive symptoms, and internet use for mental health information in adults with psychosis.
**Methods:** This study used baseline data collected as part of a broader research program investigating a digital recovery-focused intervention for psychosis. Participants completed a questionnaire on their existing internet use, both in general and for mental health information, and a range of cognitive and functioning measures. Cognitive variables included premorbid IQ, estimated using the Wechsler Test of Adult Reading, and composite scores for processing speed, working memory, and executive functioning. The Positive and Negative Syndrome Scale was also administered, with five items used to examine the relationship between mental health-related internet use and psychopathology: Delusions, Grandiosity, Suspiciousness & Persecution, Unusual Thought Content, and Lack of Judgment & Insight. Logistic regressions were used to identify unique predictors of internet use for mental health information, controlling for age and frequency of general internet use.
**Results:** 179 adults with psychosis (mean age = 39.82 years; range = 18–65; SD = 11.0) took part in this study, of whom 157 (87.7%) were regular internet users. Of these, 107 (68.2%) reported regularly using the internet for mental health information, with 33 (20.9%) doing so daily, 28 (17.7%) weekly, and 46 (29.3%) monthly or less. General websites were most commonly used for this purpose (n = 92; 58.6%), followed by video streaming sites (n = 62; 39.5%), social networking sites (n = 52; 33.2%), and forums (n = 34; 21.7%). When age and frequency of general internet use were controlled for, use of any type of website for mental health information was predicted by lower scores on Grandiosity (Exp(B) = .675, 95% CI = .513, .886, p = .005); mental health-related social media use was significantly predicted by lower estimated premorbid IQ (Exp(B) = .964, 95% CI = .937, .991, p = .010); lower scores on Unusual Thought Content predicted use of both video networking sites (Exp(B) = .629, 95% CI = .403, .981, p = 041) and forums (Exp(B) = .576, 95% CI = .379, .876, p = .010) for mental health information; while use of general websites for mental health information was not uniquely predicted by any cognitive or symptom variables.
**Discussion:** While internet use for mental health information is now common among people with severe mental illness, the presence of psychotic symptoms may inhibit such information-seeking behaviour, particularly when using interactive websites such as video streaming sites and forums. Cognitive functioning may also affect how online sources of mental health information are selected. However, using general websites for mental health information is common regardless of cognition and symptom severity, with implications for how such resources should be designed.

### F209. TRANSCRANIAL DIRECT CURRENT STIMULATION (TDCS) IN A NON-CLINICAL POPULATION AS A MODEL FOR TREATMENT OF AUDITORY VERBAL HALLUCINATIONS IN SCHIZOPHRENIA

Marquardt Lynn*,[1], Isabella Kusztrits[1], Alex Craven[1], Kenneth Hugdahl[2], Marco Hirnstein[1]
[1]*University of Bergen;* [2]*University of Bergen, NORMENT Centre of Excellence, University of Oslo, Haukeland University Hospital*

**Background:** We used transcranial direct current stimulation (tDCS) in a non-clinical population to simulate a model of tDCS-treatment for auditory verbal hallucinations (AVH) in schizophrenia. In tDCS, a low current is induced via two electrodes attached to the scalp. The anode and cathode typically up- and downregulate neuronal activity, respectively. It was suggested that AVH arise due to two main neuronal pathways: hyper-activity in the language areas in the temporo-parietal cortex and hypo-activity of the cognitive control areas in the dorsolateral prefrontal cortex. Accordingly, it was further hypothesized that by reducing activity in the temporo-parietal cortex through cathodal tDCS and simultaneously increasing neuronal activity in the dorsolateral prefrontal cortex with anodal tDCS, AVH could be reduced. Patients with schizophrenia, particularly those with AVH show additionally deficits on language and cognitive control tasks, which are known to draw on temporo-parietal and dorsolateral prefrontal cortex regions, respectively. In order to test the model we thus reversed the electrode montage in non-clinical participants and tested whether they would show similar deficits as schizophrenia patients. In addition, the healthy participants underwent magnetic resonance spectroscopy (MRS) to test whether, in accordance with the model, glutamate levels increase under the anode, and decrease under the cathode area.
**Methods:** Eighteen participants were recruited in a convenience sample (7 male/ 11 female) with a mean age of 26 years. They were tested twice with a mean interval of 8 days. In one session they received real 2mA tDCS for 20 min, while in an MRI scanner (GE 750, 3T). The other session was a sham stimulation control. The order of real/sham stimulation was counterbalanced and stimulation was double-blind. In each session, MRS was measured using a PRESS sequence (TE=35ms, 1500ms) before and after stimulation. MRS data were acquired from two voxels, one in the left dorsolateral prefrontal cortex (22ml) and one in the left temporo parietal cortex (25ml), right underneath anode and cathode electrodes, respectively. MRS data were analyzed using LCModel software; water-scaled estimates for glutamate and glutamine combined (Glx) are reported herein, with N-Acetylaspartate (NAA) and creatine (Cre) inspected to ensure stability of the Glx measure. Glx levels were subjected to a 2x2x2 ANOVA with the within-participant factors Stimulation (real vs sham), Stimulation area (dorsolateral prefrontal cortex versus temporo-parietal cortex), and Time (Pre and Post stimulation).
**Results:** Two datasets where excluded from analysis due to poor spectral quality. As expected, NAA (F1,16=.809, p=.382) and Cre (F1,16=.005, p=.944) did not show significant changes. There was a trend for Glx to be higher during real as compared to sham stimulation (main effect Stimulation F1,16=3.867, p=.067) and for Glx to be higher after than before stimulation (main effect Time F1,16=1.396, p=.078).
**Discussion:** Glx was increased during real compared to sham tDCS, and before compared to after stimulation. This could indicate that tDCS overall changes neuronal firing thresholds. However, we did not observe the expected three-way interaction of reduced glutamate levels in the dorsolateral prefrontal cortex and increased glutamate levels in the temporo-parietal cortex. This could be due to the relatively small sample. However, the present data analysis is preliminary and we aim to report findings for a larger dataset.

### F210. EXAMINING PATTERNS AND PREDICTORS OF INDIVIDUAL RESPONSE TO COGNITIVE REMEDIATION THERAPY

Maree Reser*[,1], Susan Rossell[1]
[1]*Swinburne University of Technology*

**Background:** People with serious mental health problems, particularly those with a diagnosis of schizophrenia and related disorders, often report difficulties in concentration, attention and memory. These cognitive problems can make day-to-day functioning more difficult, and are one of the strongest predictors of future persisting disability. Cognitive remediation therapy (CRT) has been shown to be moderately effective in helping people to strengthen such cognitive skills as processing speed, attention, working memory and auditory and visual memory. However, the same evidence also suggests that there is significant variability in response, with around 40–60% of CRT participants not benefiting. Traditional group analysis often masks such variability. To maximise the effectiveness of CRT, it is important to identify individual patterns of cognitive response and factors that predict such patterns.
**Methods:** Twenty-two community-based individuals (12 male) with a mean age of 38.14 years (SD 9.85), diagnosed with schizophrenia (n = 15), schizoaffective disorder (n = 6) or schizophreniform (n = 1), completed a minimum 24-session cognitive remediation intervention in a single arm trial using Posit Science's visual intensive program. Measures of premorbid and current IQ were administered at baseline and blood or saliva to perform genetic analysis was collected. The MCCB was administered pre- and post-intervention to evaluate cognitive response to CRT. To determine individual change at a MCCB cognitive domain and composite level, reliable change indices were calculated at the 95% confidence interval, adjusted for practice effects. Individuals were categorised according to whether there was a) no evidence of change, b) reliable change in at least one domain with maintenance across other domains, or c) evidence of a decline in cognitive functioning or a mix of decline and improvement. Correlates of group membership and individual patterns of response were examined.
**Results:** Of the 22 participants, 11 experienced reliable change in at least one cognitive domain, 8 participants experienced no change, and 3 participants experienced either a decline (n = 1) or a mix of improvement and decline across distinct cognitive domains. Improvements were seen in attention/vigilance (18%, n = 4), processing speed (14%, n = 3), cognitive composite (14%, n = 3), reasoning and problem solving (9%, n = 2), working memory (9%, n = 2), verbal learning (5%, n = 1), visual learning (5%, n = 1), and social cognition (5%, n = 1). For a majority of improvers (n = 10), change was limited to a single domain.
**Discussion:** Cognitive remediation has the potential to strengthen cognitive skills that underpin improvements in functioning. In line with other studies, using a stringent measure of reliable change, 50% of study participants showed improvement in at least one cognitive domain. Given the small sample size further analysis will examine select cognitive, genetic and learning potential correlates (variables selected for this analysis to be drawn from a recent systematic review from this team) with individual patterns of cognitive response to CRT.

### F211. FINDING AND FIXING ATTENTIONAL DYSFUNCTION IN SCHIZOPHRENIA

Robert Reinhart*[,1]
[1]*Boston University*

**Background:** Schizophrenia is the most debilitating health problem that exists, and its cognitive impairments are the greatest predictor of disability. Since the earliest clinical descriptions of the illness, abnormalities of attention have been at the core of the cognitive symptoms of schizophrenia. Theories of attentional dysfunction in schizophrenia propose that the deficits arise from either an inability to maintain working memory representations that guide attention, or difficulty focusing lower-level visual attention mechanisms. However, these theoretical accounts neglect the role of long-term memory representations in controlling attention.
**Methods:** To test competing accounts of the etiology of cognitive deficits in schizophrenia, we devised a cued visual search task that allowed us to examine the integrity of the memory mechanisms that control attention and the lower-level mechanisms for focusing attention on visual inputs in patients with schizophrenia and demographically matched controls. In this task, a target object was cued at the beginning of each trial. The task-relevant cue signaled the identity of the target that could appear in the search array presented a second later. Then the target remained the same for three to seven consecutive trials (length of run randomized) before it was changed to a different object. While patients and controls were repeatedly searching for the same target object, we used electrophysiological measurements of brain activity to directly measure how they were focusing attention on the search targets, as well as how they recruited working memory and long-term memory representations to control attention as they searched for the task-relevant targets. In a second experiment, we used a causal manipulation of brain activity to provide converging evidence for our hypotheses regarding the locus of the attentional deficits in schizophrenia and to determine whether it is possible to improve attention in patients. This experiment was a double-blind, sham-controlled, within-subjects design using transcranial direct-current stimulation (tDCS) and established electroencephalographic (EEG) signatures of working memory, long-term memory, and attention.
**Results:** Here, we show that the control of perceptual attention is impaired in people with schizophrenia, and that this impairment is driven by an inability to shift attentional control from working memory to long-term memory across practice. Contrary to predictions of the dominant models, this attentional impairment is observed in the face of exuberant neural activity indexing working memory and completely normal activity indexing the focusing of visual attention. Next, we provide converging evidence for the locus of attentional impairments in long-term memory by showing that noninvasive electrical stimulation of medial frontal cortex rectifies long-term memory related neural signatures and normalizes the ability of patients to find targets in complex visual scenes.
**Discussion:** The findings challenge existing views of the locus of dysfunction underlying attentional impairments in schizophrenia. Moreover, the results highlight the crucial importance of long-term memory systems in controlling attention and associated abnormalities in the hippocampus and other brain areas in schizophrenia.

### F212. IMPROVEMENT IN COGNITIVE BIASES AFTER GROUP PSYCHOEDUCATION AND METACOGNITIVE TRAINING IN RECENT ONSET PSYCHOSIS

Maria Isabel Ahuir Perez*[,1], Ángel Cabezas[2], Maria José Miñano[1], Maria José Algora[2], Francesc Estrada[1], Montserrat Sole[2], Juan David Barbero[1], Itziar Montalvo[1], Alfonso Gutierrez-Zotes[3], Vanessa Sanchez-Gistau[4], José Antonio Monreal[1], Elisabet Vilella[2], Diego Palao[1], Javier Labad[1]
[1]*Parc Taulí Hospital Universitari, I3PT, Universitat Autònoma de Barcelona;* [2]*Early Intervention Service, Hospital Universitari Institut Pere Mata, IISPV, Universitat Rovira i Virgili;* [3]*Hospital Universitari Institut Pere Mata, IISPV, Universitat Rovira i Virgili;*

[4]Hospital Univeristari institut Pere Mata, IISP, Universitat Rovira i Virgili

**Background:** Group psychotherapeutic treatments can improve negative symptoms and social functioning deficits in the treatment of schizophrenia. These treatments may include different modalities including group cognitive behavioral therapy, psychoeducation and metacognitive training (MCT). MCT is effective for preventing delusions by modifying the cognitive biases most related to psychosis. Our primary goal was to address whether cognitive biases improve more specifically with MCT when compared to psychoeducation in a sample of patients with recent onset psychosis.
**Methods:** Design: a multicenter randomized, pilot clinical trial was performed, in which one group received psychoeducation and the other MCT.
**Sample:** 49 patients aged between 18–35 years and with a diagnosis of psychotic disorder according to DSM-IV-TR criteria and less than 3 years of duration of illness. All patients were recruited at two Early Psychosis Programmes in Spain (ParcTaulí Hospital Universitari, Sabadell; Hospital UniversitariInstitut Pere Mata, Reus). Ethical approval was obtained from the local Ethics Committees of both institutions.
**Outcomes:** Patients were evaluated at baseline and at the end of each intervention. The primary outcome was cognitive biases, assessed with Cognitive Biases Questionnaire for Psychosis (CBQ). Secondary outcomes included cognitive insight, psychopathological symptoms (positive, negative, depressive) and psychosocial functioning.
**Interventions:** The interventions consisted of 8 weekly group sessions of MCT (developed at the University of Hamburg-Eppendorf by Steffen Moritz) or psychoeducation. MCT program included sessions dealing with attributional style, jumping to conclusions, changing beliefs, empathy, memory, and depression and self-esteem. The psychoeducational program included sessions addressing aspects related to psychotic illness (psychotic symptoms, risk factors of relapse, stress management, psychopharmacological treatment, substance use, physical health and social skills).
**Statistical analysis:** A general linear model for repeated measures was performed in order to compare the longitudinal effect of the intervention and to test whether changes in outcome variables differed by treatment group. All analyses were adjusted for gender. A p value $< 0.05$ (two-tailed) was considered to be significant.
**Results:** Of all 49 patients, 38 (77.6%) completed at least 50% of the sessions, and were included in the final analyses. 21 received psychoeducation and 16 MCT.
Cognitive biases improved significantly in both psychoeducation ($43.8 \pm 11.2$ vs $40.8 \pm 10.4$) and MCT groups ($44.2 \pm 7.6$ vs $39.6 \pm 5.0$). The time effect was significant ($F= 18.9$, $p<0.001$) without a different pattern in the change of CBQ scores between groups (interaction time x group, $F= 0.63$, $p= 0.431$). An improvement in negative symptoms was also observed after receiving both treatments, without significant differences between groups. No significant changes over time were observed in positive symptoms, depressive symptoms or psychosocial functioning.
**Discussion:** Both group psychoeducation and MCT improve cognitive biases in recent onset psychosis. Our study does not support a superiority of one intervention over the other in terms of improving cognitive biases.

### F213. THE EFFECTS OF GROUP INTEGRATIVE ARTS THERAPY BASED ON SOCIAL SKILL TRAINING ON THE SOCIAL ADAPTIVE FUNCTION, EMPOWERMENT AND SUBJECTIVE WELL-BEING IN INPATIENTS WITH CHRONIC SCHIZOPHRENIA

Eunsung Lim*[,1], Sang-Yeol Lee[2], Won-Myong Bahk[3], Bo-Hyun Yoon[4], Duk-In Jon[5], Moon Doo Kim[6], Sung-Yong Park[7], Min-Kyu Song[8]

*[1]Shinsegae Hospital; [2]Wonkwang University, School of Medicine; [3]Yeouido St. Mary's Hospital, The Catholic University of Korea; [4]Naju National Hospital; [5]College of Medicine, Hallym University; [6]School of Medicine, Jeju National University; [7]Keyo Hospital; [8]Yesan Mental Health Clinic*

**Background:** The object of this study was to investigate the effects of group integrative arts therapy based on social skill training on communication, social adaptive function, and subjective well-being in inpatients with chronic schizophrenia.
**Methods:** Among the 125 patients who had been hospitalized in the H mental hospital in S city after being diagnosed with schizophrenia by psychiatrists according to DSM-IV, 72 patients were selected by inclusion criteria and 48 patients were randomly assigned into an experimental group(n=16), comparative group(n=16), and control group(n=16). During this study, 4 patients from each group dropped out. The final subjects of each groups were 12 patients. The experimental group followed a 60 minutes long social skill training based on group integrative arts therapy program for twice a week and 20 times in total. The Comparative group followed a social skill training program only for 60 minutes twice a week for 20 times in total. The control group received no treatment. To assess the social adaptive function, empowerment, subjective well-being of the subjects, Communication Competence Scale(CCS), Empowerment Scale(ES) and Korean Modification of Subjective Well-Being Scale(KmSWN) were used as subjective measuring. Assertiveness Observation Evaluation Scale(AOES), Social Adaptive Functioning Scale(SAFS), and Nurses' Observation Scale of Inpatient Evaluation-30(NOSIE-30) were also used as objective measuring that were rated by nurses or social workers at the mental hospital. One-way ANOVA and Chi-Square Test were performed to check differences among groups homogeneity. Mixed ANOVA and Sheffe test were used to find the effect of group integrative arts therapy in the differences among groups.
**Results:** First, there was no statistically significant difference except nonverbal communication of CCS among three groups in homogeneity test of sociodemographic and clinical variables.
2nd, the group integrative arts therapy based on social skill training was found to significantly increase the communication of experimental group more than comparative group, and that of comparative group more than the control group.
3rd, the group integrative arts therapy based on social skill training was found to significantly increase the assertiveness of the experimental group and comparative group more than control groups.
4th, the group integrative arts therapy based on social skill training was found to significantly increase the social adaptive functioning of the experimental group more than comparative group, and that of the comparative group more than the groups.
5th, the group integrative arts therapy based on social skill training was found to significantly increase the NOSIE-30 of the experimental group and the comparative group more than control group. NOSIE-positive and irritability of NOSIE-30 in the comparative group was increased more than those of the experimental and the control groups.
6th, the group integrative arts therapy based on social skill training was found to significantly increase the empowerment of the experimental group more than that of the comparative and the control group.
7th, the group integrative arts therapy based on social skill training was found not to significantly increase the subjective well-being in all of the experimental, comparative and control groups.
**Discussion:** The group integrative arts therapy based on social skill training is found to significantly enhance the social adaptive function and empowerment of inpatients with chronic schizophrenia than social skill training. These results suggest that group integrative arts therapy could be utilized as effective mental rehabilitation intervention program for inpatients with chronic schizophrenia.

### F214. PSYCHOLOGICAL INTERVENTIONS FOR POSITIVE SYMPTOMS IN SCHIZOPHRENIA: A NETWORK META-ANALYSIS

Irene Bighelli*[,1], Stefan Leucht[1]
[1]*Technische Universität München*

**Background:** There is rising awareness about the need of multi-disciplinary approaches integrating psychological treatments for schizophrenia, but a comprehensive evidence base on their relative efficacy is lacking. Conventional pairwise meta-analyses cannot provide a hierarchy based on the randomised evidence. We aimed to integrate the available evidence to create hierarchies of the comparative efficacy, acceptability and tolerability of psychological interventions for schizophrenia.
**Methods:** We performed a network meta-analysis (which uses both direct and indirect comparisons) of randomized controlled trials on psychological treatments aimed at positive symptoms in the acute treatment of schizophrenia, compared with another psychological intervention or with a no treatment condition (waiting list, treatment as usual). We excluded trials done in patients with predominant negative symptoms, concomitant psychiatric disorders or medical illnesses, and those done in first episode or stable patients. Published and unpublished studies were sought through database searches, trial registries and websites. Study selection and data extraction were conducted by at least two independent reviewers. Our primary outcome is the change in positive symptoms on a validated rating scale. Secondary outcomes include number of dropouts, overall and negative symptoms of schizophrenia, response, relapse, adherence, depression, quality of life, functioning and adverse events. Analyses were conducted in R within a frequentist framework. The risk of bias in studies has been evaluated using the Cochrane Risk of Bias tool and the credibility of the evidence will be evaluated using an adaptation of the GRADE framework to NMA, recommended by the Cochrane guidance. Subgroup and sensitivity analyses will be conducted to assess the robustness of the findings. The protocol of this review has been registered in Prospero (registration number: CRD42017067795).
**Results:** After screening 20196 references for title and abstract and 2555 full text articles, we identified 58 suitable trials, for a total of 3956 participants. Regarding primary outcome positive symptoms, only Cognitive Behavioural Therapy was significantly more effective than treatment as usual, with a standardised mean difference of -0.59 [95% credible interval -1.03; -0.16].
The standardised mean differences with 95% credible intervals for other interventions were: Acceptance and Commitment Therapy -0.07 [-2.12; 1.98], Cognitive Therapy -0.18 [-1.92; 1.55], Hallucination Treatment -0.69 [-2.40; 1.01], Metacognitive Therapy -0.26 [-1.16; 0.64], Mindfulness -0.26 [-2.14; 1.62], with heterogeneity $tau^2 = 0.6942$.
Data analyses on other outcomes are ongoing.
**Discussion:** We are going to investigate the possible sources of heterogeneity with the pre-planned subgroup analyses: number of sessions, study duration, individual versus group setting, expertise of the therapist and baseline severity.
A network meta-analysis is the only methodology that allows the production of hierarchies of interventions for treatment of schizophrenia. Such hierarchies, saying which treatment is likely to be the best, the second best and so on, are essential for guideline development. The results of this study are therefore likely to provide knowledge of great impact for treatment decisions.
This project has received funding from the European Union's Horizon 2020 research and innovation programme under the Marie Skłodowska-Curie grant agreement No 701717.

### F215. EARLY SIGNS ACTION PLAN: EXPERIENCES OF RELATIVES

Nils Sjöström*[,1], David Berg[2], Sofia Johansson[1], Eva Andreasson[1], Marda Waern[2]
[1]*Sahlgrenska University Hospital;* [2]*Sahlgrenska University Hospital, University of Gothenburg*

**Background:** The shift towards person-centered care is ongoing within healthcare today. The Early Signs Action Plan was developed to facilitate participation of patients and their next-of-kin in outpatient psychiatric services specialized in the treatment of persons with schizophrenia and similar disorders. The aim was to investigate relatives' experiences regarding the activation of the action plan for their next-of-kin.
**Methods:** The study is a qualitative interview study using a semi-structured interview guide. The interviews are conducted with relatives (anticipated N=10) to outpatients whose Early Signs Action Plan has been activated. Interviews are digitally recorded and transcribed verbatim. The material is analyzed with qualitative content analysis
**Results:** Preliminary analysis based on the first six interviews suggests that relatives experience increased involvement in services as well as improved relations with care staff. Relatives felt a greater sense of security as they were more knowledgeable, and activation of the plan resulted in a more immediate response from service providers. However, some respondents expressed communication problems with staff, pointing to a need for improved flow of information and increased understanding of the situation. Some expressed a feeling of uncertainty related to lack of feedback from staff, as well as lack of continuity and limited inclusion in the care process. Results from the entire study will be presented
**Discussion:** Early Signs Action Plan may constitute a useful tool for the involvement of relatives in psychiatric services. However, relatives pointed out several areas for improvement

### F216. SLEEP QUALITY AND CLINICAL IMPROVEMENT IN FIRST EPISODE PSYCHOSIS

L. Prayosha Villa[1], Lyndsay Schmidt[1], Tyler M. Moore[1], Monica Calkins*[,2], Christian G. Kohler[1]
[1]*University of Pennsylvania;* [2]*University of Pennsylvania School of Medicine*

**Background:** Sleep disturbance is a common feature in early psychosis. Sleep quality has shown to be associated with both symptom severity and clinical improvement in persons with chronic illness.
Understanding the influence of sleep quality in early psychosis can be beneficial in determining interventions for coordinated specialty care (CSC). Using patients from a CSC intervention program for first episode psychosis, we investigated the association between subjective sleep quality with clinical response and clinical symptom correlates.
**Methods:** Participants were consecutive patients admitted between March 2015 to June 2017 who underwent coordinated specialty care at Penn PERC (Psychosis Evaluation and Recovery Center). Eligible participants were young persons ages 16–35 years who had experienced onset of psychosis within 3 years prior to intake and who underwent 2-years of CSC for early psychosis, including cognitive therapy for psychosis recovery (CT-R), medication management, family education and occupational support. Standardized self and observer based rating scales evaluating sleep quality (PSQI) and other clinical symptoms, e.g., anxiety (BAI), depression (BDI) and affective states (PANAS), and clinical improvement (CGI-I) were administered at intake, after 3 months, 6 months and subsequently every 6 months of CSC. Participants provided informed consent. 48 participants completed assessment at 2-time points between intake and 2–4 months later and 38 underwent assessment at 3 time points, including 6–7 months following intake. Correlational analyses were performed on PSQI change (slope) over 3 assessments and change in BAI, BDI, PANAS-negative, PANAS-positive. Analysis were further stratified by improvement – CGI-I <2 (much improvement) (n=17) and CGI >3 (little/no improvement) (n=21).
**Results:** Of 48 patients, average age at intake was 22 years (Male:Female=40:8; Caucasian:African-American/Other=28:20). Primary analyses of sleep quality and clinical improvement included participants with three PSQI rating timepoints over 6–7 months of CSC (n=38). Overall PSQI ratings did not change significantly over time. BAI and BDI-II scores significantly decreased over time, indicating subjective clinical improvement with treatment. There was a trend for positive correlations among PSQI, and BAI and BDI-II scores. When stratified by improvement, those rated 'much improved' group greater reduction of PSQI scores.
**Discussion:** We found that improved sleep quality was present in participants who experienced much global clinical improvement over 6 months of CSC. In addition, better sleep quality correlated with reduced depression and anxiety symptoms. Though these findings do not address direction of causality, our findings indicate that improving sleep quality should be a specific focus in treatment of early psychosis. Further analysis will be conducted to investigate the relationship between sleep and clinical improvement using other clinical measures, such as symptom severity, and the dataset will be expanded to include data through the end of 2017.

### F217. BASIC SELF-DISTURBANCE IN ADOLESCENCE AND SCHIZOPHRENIA-SPECTRUM DISORDERS IN YOUNG ADULTHOOD: A 7-YEAR FOLLOW-UP STUDY AMONG TREATMENT-SEEKING ADOLESCENTS

Dan Koren*[,1], Yair Tzivoni[1], Liat Schalit[1], Noa Reznik[1], Merav Adres[1], Josef Parnas[2]
*[1]University of Haifa; [2]University of Copenhagen*

**Background:** Phenomenological research indicates that disturbance of the basic sense of self may be a core phenotypic marker of schizophrenia spectrum disorders. Basic self-disturbance refers to a disruption of the sense of first-person perspective and self-presence that is associated with a variety of anomalous subjective experiences. Recent cross-sectional and prospective pilot studies provided preliminary support for the notion that SD may provide a means of further "closing in" on individuals truly at high-risk for psychosis, particularly of schizophrenia spectrum disorders (SSD). The goal of this study was to replicate and extend these pilot findings by examining the long-term persistence of SD and the degree to which their level in adolescence predicts SSD seven years later in young adulthood.
**Methods:** The 7-year stability of SD and their association with later in life SSD were explored in a sample of 40 young adults. SD was assessed with the Examination of Anomalous Self-Experience (EASE), prodromal symptoms and syndromes were assessed with the Structured Interview for Prodromal Syndromes (SIPS), present and lifetime diagnoses of schizophrenia-spectrum and other co-morbid disorders were assessed with the Kiddie Schedule for Affective Disorders and Schizophrenia (K-SADS) in adolescence and the Operational Criteria (OPCRIT) checklist for psychotic and affective illness in young adulthood, level of distress with the Mood and Anxiety States Questionnaire (MASQ), and psychosocial functioning with the Strength and Difficulties Questionnaire (SDQ).
**Results:** Forty young adults (Mean age=23.7, S.D.=1.3) out of the 82 who had participated seven years earlier in a study on the association between SD and attenuated psychosis symptoms (APS) were available and agreed to participate in the 1-year follow-up (Mean=1.4, S.D.=0.8). There were no significant differences between those who were available and those who lost for the follow-up assessment on any of the major socio-demographic or clinical variables at baseline. Eight (20%) of the 40 participants in the present study met diagnostic criteria for an SSD (2 Schizophrenia, three non-organic psychotic disorder, and three schizotypal personality disorder). The total EASE score was slightly higher in young adulthood compared to seven years earlier. However, this can reflect a difference in the administration method of the EASE between the two occasions. Consistent with our first hypothesis, the correlation between the total EASE score at baseline and 7-year follow-up was moderate and significant (r=0.59, p<.001). Similarly, consistent with our second hypothesis, SD at baseline was a significant predictor of an SSD diagnosis in young adulthood.
**Discussion:** These results provide further support for the temporal stability of SD over time. Also, they provide further support for the notion that SD is a phenotypic indicator of risk for SSD.

### F218. REAL-TIME ASSESSMENT OF AUDITORY HALLUCINATIONS USING A SMARTPHONE APPLICATION; RESULTS FROM A PILOT STUDY

Josef Bless*[,1], Runar Smelror[2], Ingrid Agartz[2], Kenneth Hugdahl[3]
*[1]University of Bergen; [2]Norwegian Center of Excellence for Mental Disorders Research, University of Oslo, Diakonhjemmet Hospital; [3]University of Bergen, Norwegian Center of Excellence for Mental Disorders Research, University of Oslo, Haukeland University Hospital*

**Background:** A challenge in current research on auditory hallucinations (AHs) is that the assessment of symptom dimensions largely depends on structured interview scales, such as the PANSS, PSYRATS etc. In order to collect more ecologically valid data, we developed a smartphone app that can be used by patients to report on their experience in real-time, i.e. when the voices are actually present. The aim of this study was to investigate feasibility of the app and whether it can provide new phenomenological information on the temporal fluctuations of AHs in adolescent patients with early-onset schizophrenia (EOS).
**Methods:** Using the experience sampling method, one adolescent EOS patient used the app for a period of 16 days, during which the patient received random reminders five times per day, to answer questions on five dimensions relevant to AHs: Control (no – full), Content (negative – positive), Localization (outside head – inside head), Intensity (yelling – whispering), and Influence (not troublesome – very troublesome). The answers were registered on visual analog scales (VASs) implemented in the app.
**Results:** The patient responded to the notifications in 87% of the cases and in addition completed the questions 15 times on own initiative. In 73% of all responses, the patient indicated to experience AHs at the time of response. The results from the VASs showed that AH-dimensions are not stable but fluctuate over time. Several AVH-dimensions were significantly correlated ($p < .01$) with each other: Influence correlated with Content ($r = -.71$), Intensity ($r = -.37$), and Control ($r = -.76$), whereas Content correlated with Intensity ($r = .39$) and Control ($r = .57$). showed several correlations a negative correlation with content of however, only localization (voices coming from outside - inside the head) correlated significantly with the number of days in use. In addition, the participant reported more internal voices over the course of 16 days ($p < .01$; $r = .36$) and later hours of the day ($p < .05$; $r = .22$).
**Discussion:** The app captures the ebb-and-flow of AVHs and provides a unique profile of symptom severity and interrelationship between AVH-dimensions. Such information has potential relevance for patient-tailored intervention.

### F219. NOVEL OBJECTIVE ASSESSMENT OF ACTIVITY ENGAGEMENT IN SCHIZOPHRENIA USING WIRELESS MOTION CAPTURE

Ishraq Siddiqui*[,1], Gary Remington[1], Gagan Fervaha[1], Paul Fletcher[1], Aristotle Voineskos[1], Sarah Saperia[1], Konstantine Zakzanis[2], George Foussias[1]
*[1]Centre for Addiction and Mental Health; [2]University of Toronto Scarborough*

**Background:** Amotivation and reduced engagement in goal-directed activities are prominent features of schizophrenia. Previous investigations of patients' engagement in activities have largely relied on accounts of daily living activities rather than objective task-based measures. The current study used wireless motion capture in an open-field setting to evaluate activity preference when individuals are provided an explicit choice between an active engagement option versus a passive engagement option.
**Methods:** Twenty stable adult outpatients with schizophrenia and twenty matched healthy controls completed the Activity Preference Task, in which participants play a physical motion-based video game (active engagement) or watch a film (passive engagement) for fifteen minutes. No incentive was associated with either activity, and participants could engage in either activity at any time. Duration of engagement on the active option and number of switches between activity options were computed as the primary task outcome measures using objective motion data. Participants' behaviour during active engagement was further quantified by computation of physical intensity (average hand speed) and persistence (tendency for sustained continuous engagement). Clinical assessments of positive and negative symptoms, apathy, cognition, depression, medication side-effects, motor ability, and community functioning were also administered.
**Results:** Schizophrenia participants' duration, intensity, and persistence of active engagement were correlated with apathy ($|\rho|=0.72–0.79$, $p<0.01$) and community functioning ($\rho=0.50–0.67$, $p<0.05$). Although no significant group differences were detected in the individual comparisons of task measures, exploratory cluster analysis based on the two primary task measures identified three clusters of individuals with distinct profiles of engagement intensity ($F(2,36)=9.141$, $p<0.001$) and persistence ($F(2,36)=13.954$, $p<0.001$), and clinical apathy ($F(2,37)=4.183$, $p=0.023$). Further, there were significant diagnostic group by cluster assignment interaction effects for engagement intensity ($F(2,33)=4.551$, $p=0.018$) and apathy ($F(2,34)=3.445$, $p=0.043$) that highlighted substantial behavioural heterogeneity specific to schizophrenia; these interaction effects appeared to be driven primarily by a subgroup of patients who exhibited reduced engagement and increased apathy compared to individuals in other clusters as well as within-cluster healthy control counterparts.
**Discussion:** The Activity Preference Task provides a means of quantifying activity engagement in schizophrenia, which may be particularly valuable given the lack of objective assessments that measure non-incentivized, intrinsically motivated behaviours. Our initial findings suggest that patients with schizophrenia as a group are equally inclined as healthy individuals towards actively engaging activities when presented an explicit choice, but provision of such opportunities may be insufficient for amotivated patients to initiate and maintain engagement in functional behaviours.

### F220. THREAT ANTICIPATION AND NEGATIVE AFFECT IN EARLY PSYCHOSIS

UnYoung Chavez-Baldini*[,1], Inez Myin-Germeys[2], Charlotte Gayer-Anderson[3], Matthew Kempton[3], Lucia Valmaggia[3], Philip McGuire[3], Til Wykes[3], Craig Morgan[3], Ulrich Reininghaus[4]
[1]*Academic Hospital Maastricht* [2]*KU Leuven;* [3]*Institute of Psychiatry, Psychology & Neuroscience, King's College London;* [4]*Institute of Psychiatry Psychology and Neuroscience, King's College London, South London, Maudsley NHS Foundation Trust;* [5]*Maastricht University*

**Background:** Increasingly, evidence points to the involvement of cognitive and affective processes in psychotic disorders. To determine the interplay of mechanisms involved in the development and maintenance of psychosis, these pathways must be studied in different stages of psychosis, such as early psychosis. Previous research, however, mostly uses cross-sectional data, and there remains a need to extend research to include timeseries and longitudinal models to investigate the direction of the relationship between these processes and psychotic experiences.
**Methods:** Lagged multilevel moderated mediation models were used to analyze the experience sampling method (ESM) data of 53 controls, 46 participants with at-risk mental state (ARMS) for psychosis, and 51 participants with first-episode psychosis (FEP) to investigate the direction of effect between threat anticipation, negative affect, and psychotic experiences. Furthermore, specific affect symptoms (i.e., anxiety and insecurity, separately) and psychotic experiences (i.e., paranoia and visual and auditory hallucinations, separately) were analyzed.
**Results:** The effect of threat anticipation (t0) on psychotic experiences (t1) was mediated by negative affect for ARMS participants and controls. Threat anticipation (t0) had a direct effect on psychotic experiences (t1) and psychotic experiences (t0) had a direct effect on threat anticipation (t1) for FEP participants. The relationship between threat anticipation (t0) and paranoia (t1) was mediated by anxiety for FEP participants and controls and mediated by insecurity for ARMS participants. Threat anticipation (t0) had a direct effect on auditory and visual hallucinations (t1) for FEP participants, and there was a direct effect of visual hallucinations (t0) on threat anticipation (t1) for ARMS participants.
**Discussion:** The findings demonstrate that threat anticipation leads to psychotic experiences, including paranoia and hallucinations, and affective disturbances mediate some of the relationships. However, there was inadequate evidence for psychotic experiences, paranoia, and hallucinations leading to threat anticipation. Together, these results provide insight into the direction of cognitive and affective processes that develop and maintain psychotic experiences in early psychosis.

### F221. SELECTIVE ATTENTION BIAS FOR FEAR STIMULI AND HALLUCINATION IN PATIENTS WITH SCHIZOPHRENIA: A PRELIMINARY STUDY

Jiyun Yum[1], Han-Suk Kim[1], Ho Jun Seo*[,1]
[1]*The Catholic University of Korea*

**Background:** Several studies have shown the association between affective dysregulation and severity of psychotic symptoms in schizophrenic patients. Attentional biases, which operate automatically to favor the processing of emotionally negative information in early stages of information processing, are known to play a causal role in the etiology of anxiety and other negative affective states. This study was conducted to evaluate the association between selective attention bias for fear stimuli and psychotic symptoms in patients with schizophrenia.
**Methods:** A total of 66 patients with schizophrenia were included in the study. Attentional biases were measured with the dot-probe task with facial expression of neutral and fear emotional. To measure the psychotic features of the participants, the Positive and Negative Symptom Scale (PANSS), Psychotic Symptom Rating Scale (PSYRATS), the Scale to Assess Unawareness of Mental Disorder (SUMD), and Clinical Global Impression–Severity scale (CGI-S) were used.
**Results:** Attentional vigilance scores were calculated by subtracting the median RT in congruent trials (dot at the position of the fear face) from the median RT in incongruent trials (dot at the position of the neutral face) Attentional vigilance scores was moderately correlated with the hallucination subscale of PSYTATS ($r=0.268$, $p=0.029$) in the participants. No correlation was found between selective attention bias and the scores of PANSS, PSYTATS-delusion, SUMD, and CGI-S. When the participants were divided into biased and non-biased groups by the attentional vigilance scores of +40 msec, no significant difference was found in the clinical measures. However, a statistical trend was found in hallucination severities between the biased and non-biased groups ($p=0.092$).
**Discussion:** As a pilot study, the results suggest that the emotional information processing might affect the subjective severity of psychotic features in schizophrenia. Further study would be needed to clarify this association.

### F222. CONCRETE THINKING PATTERN IN DAILY DECISION-MAKING PROCESS OF PATIENTS WITH SCHIZOPHRENIA: THROUGH EYE-TRACKING

Soo-Jeong Kim*[,1], Yeon-Ju Hong[1], Min-Woo Kim[1], Young-Hoon Jung[1], Jae-Jin Kim[1]
[1] *Yonsei University College of Medicine*

**Background:** Concrete thinking is one of the common clinical features of patients with schizophrenia, especially chronic and symptomatically severe patients. Until now, neuropsychological tests or proverb tests have been used to measure the difficulty of abstract thinking in patients with schizophrenia, but these methods have been difficult to know how concrete thinking patterns affect daily living of schizophrenia patients. In this study, we constructed a task that supposes the purchase of clothes in order to realize a situation close to the decision making of everyday life and tried to visualize the thinking process through eye-tracking method.
**Methods:** Each twenty of healthy controls and schizophrenia patients performed the task of purchasing clothes. In serial, subjects are asked whether the clothes are fancy, suitable for themselves, affordable and whether they would buy the clothes. The eye gaze information was obtained by using the eye tracking system of the SensoMotoric Instrument, and the fixation time in the face and the clothes areas was measured. The changes of the fixation time ratio of the face to the clothes between the matching and the purchasing decision blocks for each group were compaired using repeated-measure ANOVA and the change of the ratio in patients with schizophrenia was analyzed with the PANSS concrete thinking score using generalized estimating equations. To investigate the relationship between the fixation time ratio and the answer of the purchasing block, two-way ANOVA with post hoc independent T-test was used.
**Results:** There was a significant interaction effect between the groups on the change of the fixation time ratio($p<0.001$). In the patient group, the fixation time ratio decreased (matching:$0.474 \pm 0.044$, purchasing:$0.206 \pm 0.069$) while the fixation time ratio increased in the control group (preference:$0.233 \pm 0.041$, purchase decision:$0.463 \pm 0.064$). To investigate whether the change of the fixation time ratio is affected by the degree of concrete thinking, the odds ratio of the concrete thinking score to the fixation time ratio was calculated as 0.825(95% CI; 0.684–0.992). The higher the concrete thinking score, the greater the decrease in the fixation time ratio was. Comparing the relationship between the answers in the purchasing block and the fixation time ratio, the interaction of fixation time ratios for each group was significant($p=0.031$). In the post-hoc independent t-test, the fixation time ratio was significantly different for the control group($p<0.001$); $0.167 \pm 0.124$ with purchase and $0.566 \pm 0.073$ without the purchase. For the patient group, there was no significant difference in the fixation time ratios by the answer.
**Discussion:** The patients with schizophrenia relatively concentrated more on the face in the matching block, where the question included both "you" referring to the self and the clothes. In the purchasing block, where question did not contain "you", they focused less on the face. The concrete thinking scores had a significant influence on the change of the fixation time ratio in the patients with schizophrenia, confirming the possibility that the concrete thinking contributed to the difference of gaze information patterns between the two groups. In the final purchasing block, the differences in fixation time ratios by the answer were observed only in the control group. If the price is higher than expected in the previous block, it is estimated that the negative emotion is triggered and schizophrenia patients could easily depend on concrete thinking rather than consolidating the information from the previous blocks and making a re-judgment. It is necessary for the further study to devise a task which can measure emotional reaction and also closely related with the decision of everyday life.

### F223. COMPARATIVE STUDY OF HEART RATE VARIABILITY AND EMOTIONAL RESPONSE TO POSITIVE AND NEGATIVE AUDIOVISUAL STIMULATION IN PATIENTS WITH CHRONIC SCHIZOPHRENIA AND HEALTHY CONTROL

Sang-Yeol Lee*[,1], Won-Myong Bahk[2], Young-Joon Kwon[3], Duk-In Jon[4], Moon Doo Kim[5], Beomboo Nam[6], Sung-Yong Park[7], Min-Kyu Song[8]
[1] *Wonkwnag University School of Medicine and Hospital;* [2] *Yeouido St. Mary's Hospital, The Catholic University of Korea;* [3]*Soonchunhyang University Chunan Hospital;* [4]*College of Medicine, Hallym University;* [5]*School of Medicine, Jeju National University;* [6]*School of Medicine, Konbuk University;* [7]*Keyo Hospital;* [8] *Yesan Mental Health Clinic*

**Background:** This study was to investigate the Heart Rate Variability(HRV) and emotional response to positive and negative audiovisual stimulation in patients with chronic schizophrenia and healthy control group
**Methods:** Among 253 chronic schizophrenic patients admitted in 00 Hospital in 00 city by psychiatrist, 104 patients were informed about this research and consented. Those who met this study criteria were randomly selected. 35 healthy control consisted of peoples that did not have past and present history of mental and physical illness. Positive and negative affect and HRV were compared between chronic schizophrenia and healthy control groups, and positive and negative affect and HRV to positive and negative audiovisual stimulation were measured according to planed research process. Positive and negative audiovisual stimulation was defined by an art therapy professionalist and a psychiatrist as 10 positive and negative pictures. 3 positive and negative musics were shown to two groups for 4 minutes simultaneously. Positive and negative audiovisual stimulation were shown to two groups during 1-week intermission. HRV was measured with Ubpulse H3, an equipment for autonomic nervous system test made by Laxtha company and also analyzed by frequency domain analysis. Emotional Empathy Scale(EES) and Positive Affect and Negative Affect Schedule (PANAS) of two groups were measured at baseline and after positive and negative audiovisual stimulation. Global Assessment of Functioning Scale(GAF) and Positive and Negative Syndrome Scale(PANSS) of chronic schizophrenia group were measured by a psychiatrist.
**Results:** 1) Positive affect of patients group were significantly lower than control group, negative affect of patients group were significantly higher than control group. Low Frequency(LF), High Frequency(HF), and Total Power(TP) of HRV in patients group were significantly lowered than control group at baseline.
2) 7 subscales of emotional empathy scale were lowered in patients group compared to control group.
3) Positive affect of patients group was significantly less increased compared to the control group after positive audiovisual stimulation, negative affect of patients group was significantly less decreased to the control group after positive audiovisual stimulation.
4) Positive affect of patients group was increased after negative audiovisual stimulation, but positive affect of control group was significantly decreased compared to the patients group after negative audiovisual stimulation. There was no significant difference in negative affect between two groups after audiovisual stimulation.
5) LF of patients group was significantly higher than control group after positive audiovisual stimulation, HF and TP of patients group were significantly lowered than control group after positive audiovisual stimulation.
6) LF of patients group was significantly higher than control group after negative audiovisual stimulation, HF and PT of patients were significantly lowered than control group after negative audiovisaul stimulation.
**Discussion:** Patients with schizophrenia showed lower positive affect, higher negative affect, and lowered HRV parameters compared to the control group. They also showed lower empathy ability and inappropriate and non-contexual response. Schizophrenic patients represented hypersensitive sympathetic nervous system activity and lowered parasympathetic nervous system activity to the audiovisual stimulation. These results suggested that schizophrenic patients would show higher negative affect, less adaptive autonomic nervous system and hypersensitive or sharp to audiovisual stimulation, and decreased relaxation ability after stimulation. Audiovisual stimulation in integrative arts therapy program for schizophrenia might have avoid overactive sympathetic stimulation and recommend activate parasympathetic stimulation. Integrative art therapy for schizophrenia must be sufficiently relaxed, empathetic, and promote positive affect during therapeutic process.

### F224. UTILITY OF SALIVA FOR MONITORING OF CLOZAPINE LEVELS

Jimmy Lee*[,1], Jie Yin Yee[1], Yuen Mei See[1], Charmaine Tang[1], Boon Tat Ng[1], Gary Remington[2], Balram Chowbay[3]
*[1]Institute of Mental Health, Singapore; [2]Centre for Addiction and Mental Health (CAMH), University of Toronto; [3]National Cancer Centre, Singapore*

**Background:** Clozapine has specific indication for use in Treatment Resistant Schizophrenia (TRS) with guidelines for therapeutic drug monitoring. Though a plasma level > 350 ng/ml has been cited as the therapeutic threshold, many other studies have shown poor or inconsistent associations between blood levels of clozapine and side effects. Clozapine is about 95% bound to plasma proteins with a small biologically active fraction. Saliva presents as a promising alternative for therapeutic drug monitoring where clozapine levels would be at equilibrium with free unbound clozapine in the plasma. This provides the added advantage of a potentially stronger relationship with efficacy and adverse effects when compared to plasma levels because of saliva's closer representation of biologically-active clozapine. Salivary collection is also non-invasive and can be sampled serially for more precise evaluation of intra-individual variations. In the present investigation, we set out to evaluate the agreement and comparative clinical utility between plasma and salivary clozapine levels.
**Methods:** 53 participants with schizophrenia and on stable doses of clozapine for at least 2 weeks were recruited for the study. Participants had to undergo a clinical interview and the SCID, PANSS, plus side effect scales were administered. Symptomatic remission status was defined using the symptom criteria proposed by Andreasen et al (2005). A fasting sample of venous blood and salivary sample were collected at the same time. Assays for clozapine and norclozapine were performed using high performance liquid chromatography. A total of 106 saliva and plasma samples have been analysed.
**Results:** Our results showed strong correlations between plasma and salivary levels of clozapine ($r=0.61$, $P<0.05$) and norclozapine ($r=0.63$, $P<0.05$). Twenty (37.7%) participants achieved symptomatic remission at the time of recruitment. Non-remitters had a significantly higher level of plasma clozapine. Thirty-one (93.9%) participants in the non-remitter group have plasma clozapine levels greater than 350ng/ml.
**Discussion:** Our study shows potential for salivary samples to be an alternate non-invasive source for therapeutic drug monitoring of clozapine. This will be useful in serial monitoring of clozapine levels to evaluate treatment adherence and fluctuating pharmacokinetic profiles. There is a significant proportion of patients who do not achieve symptomatic remission on clozapine, which highlights a pressing need to identify new pharmacological agents or modalities of treatment.

### F225. LEVODOPA AUGMENTATION OF ANTIPSYCHOTICS FOR THE TREATMENT OF NEGATIVE SYMPTOMS IN SCHIZOPHRENIA

George Foussias*[,1], Naren Rao[2], Gagan Fervaha[1], Carol Borlido[1], Lillian Haber[1], Hiroyoshi Takeuchi[3], Romina Mizrahi[1], Gary Remington[4]
*[1]Centre for Addiction and Mental Health; [2]National Institute of Mental Health and Neurosciences, Bangalore [3]Keio University, School of Medicine; [4]Centre for Addiction and Mental Health, University of Toronto*

**Background:** Negative symptoms (i.e., motivation deficits and diminished emotional expression) are prevalent in schizophrenia and consistently linked with functional impairment for affected individuals. Despite advances in psychopharmacology for schizophrenia, there remain no effective treatments for these negative symptoms. Older literature, however, suggests that levodopa in conjunction with antipsychotic treatment can have beneficial effects for patients with schizophrenia. While supporting the safety and potential efficacy of dopamine augmentation, these studies did not evaluate effects within specific symptom domains, particularly negative symptoms. This open-label pilot study was conducted to evaluate the preliminary efficacy and safety of levodopa augmentation of antipsychotics for the treatment of negative symptoms.
**Methods:** Ten stable outpatients with schizophrenia between the ages of 18 and 60 were enrolled. All were treated with stable atypical antipsychotic monotherapy for at least eight weeks, and had a minimum total score of 30 on the Scale for the Assessment of Negative Symptoms (SANS). Participants were treated with adjunctive open-label levodopa/carbidopa, with dose titration to levodopa 300mg TID over 38 days as tolerated, and dose maintenance for the remainder of the eight-week trial. Baseline assessments consisted of the SANS, Scale for the Assessment of Positive Symptoms (SAPS), Brief Psychiatric Rating Scale (BPRS), Clinical Global Impression (CGI), Calgary Depression Scale for Schizophrenia (CDSS), MATRICS Consensus Cognitive Battery (MCCB), and Quality of Life Scale (QLS) for community functioning. Treatment side effects were evaluated with the Simpson Angus Scale (SAS), Barnes Akathisia Rating Scale (BARS), Abnormal Involuntary Movement Scale (AIMS), UKU side effect rating scale (UKU), Liverpool University Neuroleptic Side Effect Rating Scale (LUNSERS), Yale-Brown Obsessive Compulsive Scale (YBOCS), and Barratt Impulsiveness Scale (BIS). Participants were reassessed on measures of psychosis and side effects weekly for the first four weeks, and every two weeks thereafter. The SANS and CGI were repeated at weeks 4 and 8, with the MCCB and QLS re-administered at week 8. Statistical analyses included the intent-to-treat sample (i.e., participants who received at least one dose of study medication) and consisted of linear mixed models for change in SANS total score (our primary outcome), and change in other clinical and side effects measures as a result of treatment.
**Results:** Enrolled participants (eight male and two female) had a mean age of 37.2 years. Seven participants completed the study, with three participants dropping out. The mean final dose of levodopa for study completers was 835.7 mg (SD 170.1). Levodopa augmentation resulted in a significant improvement in negative symptoms, with a mean SANS reduction for study completers of 15.3 (SD 5.7). This equated to a mean SANS improvement of 25.5% (range 17% to 57%), with 43% of study completers experiencing > 20% improvement in SANS score. Notably, significant improvement in negative symptoms emerged after the first four weeks of treatment. There was no significant change in positive symptoms, nor other clinical outcomes or side effect measures.
**Discussion:** Our findings suggest that levodopa augmentation of antipsychotics may be an effective and well-tolerated treatment for negative symptoms in schizophrenia. These findings, however, need replication in larger randomized controlled trials. With the dearth of available treatments for negative symptoms, levodopa augmentation may represent a novel pharmacologic strategy to address this critical unmet therapeutic need for schizophrenia.

### F226. CLINICAL FACTORS ASSOCIATED WITH CONTINUATION OF LONG-ACTING INJECTABLE ANTIPSYCHOTIC MEDICATION: RETROSPECTIVE CHART REVIEW

Shi Hyun Kang*[,1], Jong Il Lee[1]
*[1]Seoul National Hospital*

**Background:** Long-acting injectable (LAI) antipsychotic medications provide a potential solution to the poor adherence to oral therapies in schizophrenia. However, not all patients with schizophrenia seem to have therapeutic benefits using LAI antipsychotics.
The objectives of this study were to investigate clinical factors in patients with schizophrenia who have continued the LAI antipsychotics compared with the patients who have discontinued the LAI antipsychotics.
**Methods:** Data were collected by retrospective chart review of all 150 patients prescribing LAI therapy during 2005–2012 in a mental hospital. The data including age at onset, age at starting LAI antipsychotics, duration of illness at starting LAI antipsychotics, total number of admission before starting LAI antipsychotics were gathered. The subjects were classified into three group; 1) the continuation group (n=27, 18.0%), 2) the discontinuation group (n=57, 38.0%), and 3) the follow-up loss group (n=66, 44%). The ANOVA were used to compare the clinical variables among 3 groups. The stepwise multiple linear regression analyses were conducted to evaluate the association between the duration of LAI medications and clinical variables.
**Results:** There were significant differences among three groups in age (52.9 ± 9.1, 44.8 ± 12.9, 49.5 ± 12.0, p=0.009), age at onset (30.7 ± 10.4, 24.6 ± 9.9, 26.2 ± 8.6, p=0.027) and age at starting LAI medications (44.1 ± 8.9, 37.0 ± 12.4, 40.8 ± 11.2, p=0.021). In regression analyses, the duration of LAI medications were significantly associated with age (β=1.727, p<0.001), age at starting LAI medications (β=-1.489, p<0.001), and the total number of admission before starting LAI medications (β=-0.177, p=0.008).
**Discussion:** The continuation of LAI medications was associated with age, age at starting LAI medications and the number of admission before starting LAI medications.

### F227. PSYCHOLOGICAL TRAUMA OCCURRING DURING ADOLESCENCE IS ASSOCIATED WITH AN INCREASED RISK OF GREATER WAIST CIRCUMFERENCE IN EARLY PSYCHOSIS PATIENTS INDEPENDENTLY OF MEDICATION

Luis Alameda*[,1], Axel Levier[2], Philippe Golay[3], Mehdi Gholam-Rezaee[3], Frederik Vandenberghe[2], Aurélie Delacretaz[2], Anaïs Glatard[2], Céline Dubath[2], Kim Q. Do[3], Chin B. Eap[3], Philippe Conus[4]
*[1]Institute of Psychiatry, Psychology & Neuroscience, King's College London; [2]Lausanne University Hospital, Hospital of Cery; [3]Lausanne University Hospital; [4]Centre Hospitalier Universitaire Vaudois (CHUV), University of Lausanne (UNIL)*

**Background:** The high prevalence of obesity in patients suffering from psychosis is a major concern as it dramatically increases the mortality rates of such patients in the long term. The mechanisms by which these patients develop overweight are poorly understood. It has been suggested that exposure to Childhood Trauma (CT) may play a role in the risk for obesity; however, whether this is the case for Early Psychosis (EP) patients and independently of the impact of medication has yet to be investigated. In addition, it is unknown whether the age at the time of exposure to CT can modulate the link between CT and obesity in EP patients.
**Methods:** 136 EP patients aged 18–35 were recruited from the Treatment and Early Intervention in Psychosis Program (TIPP-Lausanne). Body Mass Index (BMI), Weight Gain (WG) and Waist Circumference (WC) were measured and monitored prospectively after psychotropic prescription during a follow-up period of 1 year (patients were assessed at baseline, after 1, 2, 3, 6 months and 1 year of antipsychotic treatment). Patients were classified into Early-Trauma if they had faced at least one experience of abuse (physical, sexual, or emotional) or neglect (physical or emotional) before age 12, and Late-Trauma if the exposure had occurred between ages 12 and 16. Linear Mixed effect models with a random intercept were used to investigate the impact of Trauma (early or late) on the metabolic parameters longitudinally, Marko Chain Monte Carlo (MCMC) method was used to adjust these models with sufficiently large number of MCMC iterations. Models were adjusted for age, socioeconomic status, baseline BMI, medication intake prior to the first assessment and during the treatment phase, and by the diagnosis of depression.
**Results:** Patients were more likely to have a diagnosis of Schizophrenia (61%; N=83), they had a mean age of 26 at the time of first assessment, and exposure to 1 or more forms of traumatic experiences before 16 years of age was present in 32% of the sample. No differences between groups were found at baseline in terms of BMI or WC. Late-Trauma patients, when compared to Non-Trauma patients showed greater WCs during the follow-up (p=0.012). No differences between Early or Late-Trauma patients and Non-Trauma patients were found in any of the other outcome measures during the follow up. Baseline BMI and treatment duration were significantly associated with the level of BMI and WC during the follow up. None of the other potential confounding factors were significantly associated with the outcome measures during the follow up.
**Discussion:** Exposition to trauma during adolescence in EP patients is associated with a higher risk of greater WC during the early phase of the disease, independently of the medication intake, depression and other confounding factors. Specific preventive measures should be addressed in these patients in order to reduce the risk of obesity. Depending on the timing of traumatic exposure, different developmental mechanisms may underlie this differential possible impact on WC. Further studies on interactions between central consequences of traumatism and metabolic syndrome are warranted.

### F228. EFFECTIVENESS OF PALIPERIDONE PALMITATE VS OTHER LONG-ACTING INJECTABLE (LAI) ANTIPSYCHOTICS – AN ELECTRONIC CASE REGISTER STUDY

Rashmi Patel*[,1], Edward Chesney[1], Matthew Taylor[1], David Taylor[2], Philip McGuire[1]
*[1]King's College London; [2]Maudsley Hospital and Institute of Pharmaceutical Science, King's College London*

**Background:** Paliperidone palmitate is a 2nd generation long-acting injectable (LAI) antipsychotic which is increasingly prescribed for patients with chronic schizophrenia. However, it is more expensive than 1st generation LAI antipsychotics and little is known about its effectiveness in a real world clinical setting. We sought to address this issue by analyzing a large electronic case register of patients with schizophrenia treated with LAI antipsychotics.
**Methods:** Data were obtained from 1,281 patients in the South London and Maudsley NHS Foundation Trust (SLaM) Biomedical Research Centre (BRC) Case Register who were treated with an LAI antipsychotic between 1st April 2011 and 31st January 2015. The number of days spent as a psychiatric inpatient and the number of admissions to a psychiatric hospital were extracted using the Clinical Record Interactive Search tool (CRIS) and analyzed in each of the 3 years before and after LAI prescription using multivariable regression.
**Results:** Patients who received paliperidone palmitate (n=430; 33.6%) spent more time in hospital (β coefficient 12.3 days, 95% CI 2.3 to 19.2, p=0.001) and were admitted to hospital more frequently (IRR 1.44, 95% CI 1.29 to 1.61, p<0.001) in the year prior to treatment than those treated with other LAI antipsychotics (n=851, 66.4%). However, there were no significant differences between paliperidone and the other LAI antipsychotics in the 3 years after initiation with respect to the number of days spent in hospital (β coefficient 5.4 days, 95% CI -57.3 to 68.2, p=0.86) or frequency of hospital admissions (Incidence rate ratio 1.07, 95% CI 0.62 to 1.83, p=0.82).
**Discussion:** Paliperidone palmitate was more likely to be prescribed in patients with more severe illness, as indicated by a history of more frequent and lengthy hospital admissions prior to initiation. The absence of differences in outcomes after initiation indicates that the effectiveness of paliperidone palmitate was similar to that of other LAI antipsychotics. However, paliperidone palmitate may be better tolerated than other 1st generation LAI antipsychotics with a lower rate of discontinuation. These findings merit consideration in relation to the high cost of paliperidone palmitate compared to other LAI antipsychotics.

### F229. THE BIOLOGICAL UNDERPINNINGS OF TREATMENT RESPONSE IN DELUSIONAL DISORDER: A SYSTEMATIC REVIEW OF QUALITATIVE EVIDENCE-TO-DATE

Alexandre González-Rodríguez*[,1], Francesc Estrada[2], Itziar Montalvo[1], José Antonio Monreal[3], Diego Palao[3], Javier Labad[3]

*[1]Parc Tauli University Hospital, Institut d'Investigació i Innovació Parc Taulí (I3PT); [2]Parc Tauli University Hospital; [3]Parc Tauli University Hospital, Institut d'Investigació i Innovació Parc Taulí (I3PT), Autonomous University of Barcelona (UAB)*

**Background:** The dopamine hypothesis of schizophrenia has been extensively proposed as a neurobiological mechanism that explains the relationship between schizophrenic symptoms and hyperdopaminergic states. This hypothesis is supported by direct and indirect evidence, and it mainly postulates that antipsychotics act blocking dopamine receptors. When focusing on delusional disorder patients, especially delusional disorder somatic type, a great effort towards the search for a biological basis of treatment response has been recently demonstrated. Thus, the main goal of this systematic review was to examine the evidence explaining the biological underpinnings of treatment response in delusional disorder.

**Methods:** A systematic review was performed using Pubmed, Scopus and PsycINFO databases (from 1990 to October 2017), according to the Preferred Reporting Items for Systematic Reviews and Meta-Analyses (PRISMA) statement. The following search terms were used: [('treat*' OR 'therap*' OR 'biol*') AND ('delusional disorder')]. This systematic computerized search was completed by additional studies hand-checked through reference lists from the included studies and review articles. Studies were only included if the met our inclusion criteria: (a) the International Classification of Diseases (ICD) or Diagnostic and Statistical Manual of Mental Disorders (DSM) diagnosis for delusional disorder, (b) be published in peer-reviewed journals, (c) in English, German or Spanish, (d) and reporting a hypothesis for the biological basis of treatment response in delusional disorder, irrespective of method and study design. Exclusion criteria were: (a) studies including organic delusional disorder or (b) somatic delusions secondary to other psychiatric diagnoses. The literature search strategy, data extraction and synthesis was conducted independently by two authors (A.G.R, F.E.). When disagreement, it was solved by consensus.

**Results:** A total of 59 articles were identified, of which 12 met our inclusion criteria. Four hypotheses were addressed: (1) Dopaminergic dysfunction (n=4): ziprasidone-induced supersensitivity psychosis by chronic blockade of D2 Dopamine Receptor (DRD2) (n=1); pretreatment levels of plasma homovallinic acid (pHVA) (n=1); dopamine transporter (DAT) dysfunction (n=1) and effectiveness of aripiprazole (DRD2 agonist) (n=1). (2) Serotonergic dysfunction (n=6): drug occupancy in 5-HT1A and 5-HT2A receptors (n=3) and efficacy of 5-HT2 antagonists (n=3). Brain dysfunction (n=7): hypoperfusion in cerebral blood flow in temporal and parietal lobes, left side (n=5), right side (n=1) and lack of basal ganglia and subcortical gray matter lesions (n=1). Genetic evidence (n=1): implications of DRD2 Ser311Cys, DRD3 Ser9Gly and TH VNTR polymorphisms.

**Discussion:** The strongests biological contributors for treatment response in delusional disorder seem to be those implicating monoaminergic systems, particularly dopamine and serotonergic neurotransmitters. Although the low level of evidence, the serotonergic dysfunction may be associated with response rates, especially in delusional disorder somatic type. The link between genetic variants of dopamine receptors and neuroimaging findings in delusional disorder may open new avenues for the search of the biological underpinnings of treatment response. The evidence for an integrated model involving dopamine and serotonin systems bears further investigations.

### F230. COMPARISON OF PALIPERIDONE PALMITATE 3-MONTH AND PALIPERIDONE PALMITATE 1-MONTH FORMULATION FOR NEGATIVE SYMPTOMS IN SCHIZOPHRENIA: A PHASE 3 NON-INFERIORITY STUDY

Maju Mathews[1], Srihari Gopal*[,1], Arun Singh[1], Jagadish Gogate[2], Edward Kim[2], Katalin Pungor[2]

*[1]Janssen Research & Development, LLC; [2]Janssen Scientific Affairs, LLC*

**Background:** Negative symptoms of schizophrenia are key predictors of long-term disability. It is important to understand whether treatment with long-acting injectable antipsychotics can improve negative symptom psychopathology. Paliperidone palmitate 3-month formulation (PP3M) provides a sustained release of paliperidone, permitting a significantly extended dosing interval of only 4 doses per year in patients with schizophrenia. The efficacy of PP3M as assessed by relapse rate is comparable to the paliperidone palmitate 1-month formulation (PP1M). The purpose of this post-hoc analysis was to compare the improvement in negative symptoms in patients treated with PP1M and PP3M.

**Methods:** Data from a randomized, double-blind (DB), parallel-group, multicenter, phase 3 study in patients with schizophrenia were analyzed. Patients aged 18 to 70 years with schizophrenia (DSM-IV-TR) and a total Positive and Negative Syndrome Scale (PANSS) score of 70–120 at screening were enrolled. After screening (3 weeks), patients entered a 17-week open-label (OL) phase, to receive PP1M (day 1 [150 mg eq. deltoid], day 8 [100 mg eq. deltoid], weeks 5, 9 and 13 [50, 75, 100, or 150 mg eq., deltoid/gluteal]) and entered a 48-week DB phase and were randomized (1:1) to receive fixed doses of either PP1M (50, 75, 100, or 150 mg eq., stabilized in OL) or PP3M (175, 263, 350, or 525 mg eq.) in deltoid or gluteal muscle until they relapsed or withdrew from study. The PANSS total scores with emphasis on 7-item negative subscale scores for PP1M vs PP3M were assessed.

**Results:** Of 1429 patients enrolled, 1016 were randomized to receive PP3M (n=504) or PP1M (n=512) in DB phase. Majority of patients were men and white (both 55%), with a mean (SD) age of 38.4 (11.86) years. At baseline, the mean (SE) negative subscale total was 23.2 (0.12), indicating a moderate to severe level of negative symptoms. Negative subscale and negative symptoms factor scores showed continuous improvements throughout the OL and double-blind phases of the study - mean (SD) at OL baseline and DB endpoint for total negative subscale score and symptom factor score were 23.2 (4.60) and 22.3 (4.87), and 15.9 (4.99) and 14.9 (4.81), both R2:0.16, respectively. The mean (SD) PANSS negative subscale score changes from DB baseline for PP1M vs PP3M were similar over time (mean change from baseline to DB endpoint was -1.4 (3.67), R2:0.06 vs -1.4 (3.63), R2:0.05).

**Discussion:** Development of an LAI antipsychotic with less frequent dosing than those currently available would be of potential advantage to patients, caregivers, and prescribers. PP3M and PP1M demonstrated consistent and similar efficacy in patients with moderate to severe negative symptoms of schizophrenia over the observed timepoints, including impact on patients with predominantly negative symptoms. Longer continuous treatment with PP3M showed greater benefit. This indicates that long-acting therapies are associated with continued improvement in negative symptoms over time. Treatment with LAIs for longer than a year was associated with the greatest improvements in negative symptoms.

### F231. GYM RATS: EXERCISE REVERSES COGNITIVE IMPAIRMENT IN THE PHENCYCLIDINE RAT MODEL OF SCHIZOPHRENIA

Lisa Heaney*,[1], Joanna Neill[1], Antonio Gonzales[2], Alison Yung[1], Stuart Allan[1], Michael Harte[1]
*[1]University of Manchester; [2]University of Bath*

**Background:** The cognitive deficits of schizophrenia have been identified as an unmet clinical need. They are predictive of functional outcome [Green et al., 2000] and quality of life [Fujii et al., 2004], yet there are no treatments able to normalise cognition in schizophrenia. There is increasing evidence that exercise is helpful for these symptoms [Geyer et al., 2012], but the systems involved remain enigmatic. Animal models can be used to scrutinise both the behavioural and biological effects of exercise. The sub-chronic phencyclidine (PCP) rat model for schizophrenia is a well-established and widely utilised model that is used to investigate schizophrenia-like cognitive deficits [Neill et al., 201]. This two-part study investigates whether voluntary wheel running is able to reverse cognitive impairment in the sub-chronic PCP rat model for schizophrenia, and how long the effect of exercise lasts.
**Methods:** Female Lister Hooded rats (n=80) were pseudo-randomised into four groups: vehicle-control; vehicle-exercise; PCP-control and PCP-exercise (n=20 per group). Rats were treated either with saline (vehicle) or PCP (2mg/kg, i.p. bi-daily, followed by a seven-day wash-out period). Vehicle and PCP exercise groups had access to a wheel for 1 hour a day, 5 days a week, for 6 weeks. The vehicle and PCP control groups were treated in the same way, but the wheels were locked. Rats were tested in the novel object recognition (NOR) memory paradigm pre-exercise (time point 1, T1) post-exercise (time point 2, T2), after two weeks rest (time point 3, T3) and four weeks rest (time point 4, T4). Half of the animals from each group (n=10 per group) were sacrificed post exercise (T2), and the remaining animals were sacrificed after 4 weeks rest. For each animal, 1 brain hemisphere was collected for protein analysis and 1 hemisphere was fixed for immunohistochemistry. Behavioural data were analysed using two-way ANOVA and post-hoc t-tests.
**Results:** Pre-exercise (T1), in the retention phase both vehicle groups were spent more time exploring the novel over the familiar object, an effect that was not seen in the PCP groups. Post-exercise (T2 & T3), in the retention phase both vehicle groups and the PCP exercise group spent more time exploring the novel over the familiar object, an effect that was not seen in the PCP-control group. Post-exercise (T4) in the retention phase both vehicle groups were spent more time exploring the novel over the familiar object, an effect that was not seen in the PCP groups.
**Discussion:** Exercise is able to rescue the NOR cognitive deficit seen in the sub-chronic rat model for schizophrenia. This corresponds with human studies reporting positive effects of exercise in patients with schizophrenia and provides a potential tool to thoroughly investigate the pro-cognitive effects of exercise The benefits of the exercise intervention were observed 2 weeks post-exercise with the deficits returning in the PCP treated animals when they were tested 4 weeks post-exercise. Post-mortem analysis is underway to determine the potential mechanisms by which exercise improves cognitive impairment.

### F232. A PHASE 3 STUDY TO DETERMINE THE ANTIPSYCHOTIC EFFICACY AND SAFETY OF ALKS 3831 IN ADULT PATIENTS WITH ACUTE EXACERBATION OF SCHIZOPHRENIA

David McDonnell*,[1], Steven G. Potkin[2], Adam Simmons[1], Ying Jiang[1], Lauren DiPetrillo[1], Bernard Silverman[1]
*[1]Alkermes, Inc.; [2]University of California, Irvine*

**Background:** ALKS 3831, currently under development for the treatment of schizophrenia, is composed of a flexible dose of olanzapine (OLZ) and a fixed dose of 10 mg of samidorphan. In a Phase 2 study, ALKS 3831 mitigated OLZ-associated weight gain and exhibited antipsychotic efficacy similar to OLZ alone. This Phase 3 study assessed antipsychotic efficacy and safety of ALKS 3831 in patients with acute exacerbation of schizophrenia.
**Methods:** This was an international (USA, Ukraine, Serbia, and Bulgaria), 4-week, randomised, double-blind, active and placebo (PBO)-controlled study of ALKS 3831 in patients with acute exacerbation of schizophrenia (ClinicalTrials.gov: NCT02634346). Eligible patients (N=403) were randomised 1:1:1 to receive either ALKS 3831, OLZ, or PBO. Patients were treated in an inpatient setting for the first 2 weeks of the study and could be treated as inpatients or outpatients for the remaining 2 weeks. Patients were excluded if they received OLZ within 6 months prior to screening. Antipsychotic efficacy was assessed using the Positive and Negative Syndrome Scale (PANSS), Clinical Global Impression–Severity (CGI-S), and CGI–Improvement (CGI-I) scales. Safety and tolerability were assessed as adverse events (AEs).
**Results:** Of 401 patients randomised and dosed to ALKS 3831, OLZ, and PBO, 91%, 89%, and 83% of patients, respectively, completed treatment. The most common reason for discontinuation was withdrawal by patient (6% in both the ALKS 3831 and PBO groups, and 7% in the OLZ group). Baseline characteristics were generally similar between groups; however, baseline mean body-mass index was higher in the OLZ group than in the ALKS 3831 group. Baseline mean ± standard deviation scores were 101.7 ± 11.9 for PANSS total score and 5.1 ± 0.7 for CGI-S score. The mean OLZ dose was 18.4 mg/day in both active treatment arms. Least squares (LS) mean difference ± standard error (SE) versus PBO from baseline to Week 4 in PANSS total score was –6.4 ± 1.8 (P<.001) for the ALKS 3831 group and –5.3 ± 1.8 (P=.004) for the OLZ group. LS mean difference ± SE vs PBO from baseline to Week 4 in CGI-S score was −0.38 ± 0.12 (P=.002) for the ALKS 3831 group and −0.44 ± 0.12 (P<.001) for the OLZ group. The percentage of patients with an improvement in PANSS response (≥30% improvement from baseline) at Week 4 was 60%, 54%, and 38% in the ALKS 3831, OLZ, and PBO groups, respectively. The percentage of patients with an improvement in CGI-I response (score of ≤2) at Week 4 was 58%, 51%, and 33% in the ALKS 3831, OLZ, and PBO groups, respectively. Discontinuation due to AEs was low in all groups. Common AEs (≥5%) included weight gain, somnolence, dry mouth, anxiety, headache, and schizophrenia.
**Discussion:** ALKS 3831 demonstrated greater antipsychotic efficacy than PBO, as measured by the PANSS and CGI-S scale, and was similar to the active control, OLZ. The safety profile of ALKS 3831 was similar to OLZ.

### F233. NEGATIVE SYMPTOMS ARE INDEPENDENT MODERATOR FACTORS OF TREATMENT RESISTANT SCHIZOPHRENIA EFFECTS ON MULTIPLE CLINICAL, PSYCHOPATHOLOGICAL, COGNITIVE AND PSYCHOSOCIAL VARIABLES

Felice Iasevoli*,[1], Benedetta Altavilla[1], Camilla Avagliano[1], Annarita Barone[1], Luigi D'Ambrosio[1], Marta Matrone[1], Danilo Notar Francesco[1], Eugenio Razzino[1], Andrea de Bartolomeis[1]
*[1]University of Naples "Federico II"*

**Background:** Negative symptoms (NSs) are more severe in Treatment Resistant Schizophrenia (TRS) than Antipsychotic Responder Schizophrenia (ARS) patients. NSs are predictors of outcomes of neurological soft signs and functional capacity in TRS but not in ARS patients. The scope of this work is to clarify whether NSs effects are integral to or independent from the TRS diagnosis in our sample of patients.
**Methods:** 70 out of 206 eligible putative TRS and ARS patients were included (enrollment still ongoing). Patients were tested by the Neurological

Evaluation Scale (NES); the CGI-S; the PANSS; the Heinrichs' Quality of Life Scale (QLS); the UCSD Performance-Based Skills Assessment (UPSA); the Personal and Social Performance (PSP) scale and Specific Level of Functioning (SLOF). Patients were subdivided in NSHigh (severe NSs) and NSLow (mild NSs) based on ROC curve-derived cut-off.
**Results:** At the Student's t test, NSHigh had significantly lower scores than NSLow patients on: Verbal Fluency; QLS score; PSP score; UPSA Financial, Communication, and Family Skills; UPSA total score; all SLOF areas (except Area4). NSHigh patients had significantly higher scores than NSLow patients on CGI-S; PANSS Positive and General Psychopathology Subscale scores; and NES score.
Distribution of NS patients was significantly different between TRS/ARS diagnostic groups, as NSHigh patients were significantly more frequent in the TRS group (Pearson chi square: □1=5.51, p=.001). Notably, mean PANSS Negative Subscale scores were significantly higher in TRS compared to ARS patients (Student's t: F1,58=2.84, p=.006).
Since multiple variables found to be significantly different in NSHigh vs. NSLow patients were also significantly different between TRS and ARS patients, the question arises whether the significant differences found between diagnostic groups may depend on the higher percentage of patients with more severe NSs in the TRS group. Therefore, a two-way ANOVA was carried out with dichotomous NS and Diagnosis variables as the independent variables. Outcomes on multiple clinical variables were significantly different among groups. A NS*Diagnosis interaction effect was found for NES score (F1,58=4.32, p=.042, Visuospatial Memory, UPSA Transportation skills, and SLOF Area1. In all these cases, NSHigh/TRS patients performed significantly worse than the other patient groups; in the case of NES score, NSHigh/TRS patients score significantly higher than the other groups. Independent effect of either NSs or Diagnosis were also found for multiple variables, suggesting that NSs and Diagnosis may interact but their effects are not completely overlapping. To have a more deepen comprehension of NS effects on diagnosis, we carried out a moderator regression analysis and an ANCOVA analysis that further confirmed the finding that NSs' mediate Diagnosis effects on a number of clinical outcomes.
Given that NSs largely affect clinical variables, we asked which distinct symptom may exert the greater impact on each of these variables. Therefore, we carried out a including the seven PANSS Negative Subscale items as the independent variables. The items that explained the highest variance in clinical variables were mostly Stereotyped Thinking (N7), Passive Social Withdrawal (N4), and Difficulty in Abstract Thinking (N5).
**Discussion:** These data suggest that NSs are both independent determinants and moderators of TRS/ARS diagnosis effect on multiple psychopathology, cognitive, and psychosocial factors. More impaired functions attributed to non-response to antipsychotics may depend on more severe NSs. However, only a subset of NSs appears to exert this action, possibly related to the multidimensional construct of these symptoms.

### F234. TYPICAL AND ATYPICAL ANTIPSYCHOTICS' D2R AFFINITY AND DOSES INFLUENCES POSTSYNAPTIC DENSITY BY MODULATING THE SPATIAL EXPRESSION OF HOMER1A A GENE HIGHLY IMPLICATED IN SYNAPTIC PLASTICITY AND PSYCHOSIS

Avagliano Camilla*[,1], Elisabetta Buonaguro[1], Carmine Tomasetti[1], Federica Marmo[1], Licia Vellucci[1], Felice Iasevoli[1], Andrea de Bartolomeis[1]
*[1]University School of Medicine Federico II*

**Background:** Post-synaptic density (PSD) is an ultra-specialized structure of excitatory synapses composed by a large variety of molecules (scaffolding proteins, glutamate receptors, cytoskeleton proteins). PSD has been implicated in synaptic plasticity, memory formation and in the pathophysiology of psychiatric disorders by extensive GWA studies. The immediate early gene Homer1a is part of this complex molecular machinery for signaling transmission and its expression is modulated by antipsychotics (APDs). Here we show a comparative analysis of Homer1a expression data by first and second-generation APDs, in order to correlate it to their receptor profile.
**Methods:** We analyzed Homer1a expression induced by APDs at various doses in Sprague-Dawley rat forebrain, collecting data from multiple In Situ Hybridization experiments carried out in our laboratory in standard controlled conditions. Homer1a expression levels were normalized as the ratio of the corresponding mean vehicle value in each region. Normalized expression levels were quantitatively compared by ANOVA and Tukey's post-hoc test (p<.05) and grouped in four classes: no induction; light induction; moderate induction; high induction.
**Results:** In the striatum, sertindole did not induce Homer1a expression. Quetiapine and amisulpride were observed to trigger light induction of the gene. Clozapine triggered a light-moderate induction. Moderate induction was found by olanzapine and aripiprazole, while high induction was found by ziprasidone, asenapine, and haloperidol, especially in caudate-putamen regions.
In the cortex, Homer1a mRNA was not induced by sertindole, 4mg/kg ziprasidone, haloperidol (0.25 and 0.5mg/kg). Haloperidol 0.8mg/kg, 15mg/kg quetiapine, 10mg/kg and 35mg/kg amisulpride triggered light induction. Moderate induction was found for 30mg/kg quetiapine, olanzapine, clozapine, 10mg/kg ziprasidone and for asenapine at all doses tested. Notably, both clozapine and 10mg/kg ziprasidone induced the highest levels of Homer1a mRNA in the insular cortex.
**Discussion:** A strong correlation with D2 receptor blockade and the extent of Homer1a expression in striatum, but not in the cortex, was found. However, other molecular mechanisms (e.g. D1 receptor activation in striatum; 5-HT2A receptor blockade in the cortex) may contribute to affect its expression levels.

### F235. DIFFERENTIAL EFFECTS OF ANTIPSYCHOTICS ON NEUROINFLAMMATION AND ENERGY SENSING IN A HYPOTHALAMIC CELL LINE

Chantel Kowalchuk*[,1], Pruntha Kanagasundaram[1], Gary Remington[1], Denise Belsham[2], Margaret Hahn[1]
*[1]Centre for Addiction and Mental Health, University of Toronto; [2]University of Toronto*

**Background:** Antipsychotics (AP)s are the cornerstone of treatment for schizophrenia but cause serious metabolic side-effects. The hypothalamus is the primary brain region responsible for whole body energy regulation and disruptions in energy sensing (e.g. insulin signaling) and inflammation in this brain region have been implicated in the development of peripheral insulin resistance and obesity. Thus, it is possible that hypothalamic inflammation and disturbed energy sensing could be involved in AP-induced metabolic disturbances. Data in relation to AP-associated changes in inflammatory markers in schizophrenia has been inconsistent, owing in part to confounds of illness-related factors (e.g. diet, smoking) and secondary effects of weight gain. To our knowledge, direct effects of APs on hypothalamic cells in relation to insulin signaling and inflammation have not been examined.
**Methods:** To examine direct, molecular effects of APs in the hypothalamus, an immortalized rat hypothalamic cell line, rHypoE-19, was treated with olanzapine (dose range between 0.25–100 uM), clozapine (2.5–100 uM) or aripiprazole (5–20 uM). Western blotting was used to detect changes in the energy sensing protein AMPK, components of the insulin signaling pathway (AKT, GSK3B), and components of the mitogen activated-protein kinase (MAPK) pathway (ERK1/2, JNK, p38), the latter which are linked to inflammation. Quantitative real-time PCR was performed to determine changes in the mRNA expression of interleukin (IL)-6, IL-10 and brain derived neurotrophic factor (BDNF).

**Results:** Both olanzapine (100 uM) and clozapine (100 uM) significantly increased pERK1/2 and pJNK protein expression, while aripiprazole (20 uM) only increased pJNK. Clozapine (100 uM) and aripiprazole (5 and 20 uM) significantly increased AMPK phosphorylation and inhibited insulin-induced phosphorylation of AKT. Olanzapine (100 uM) treatment caused a significant increase in IL-6 while aripiprazole (20 uM) significantly decreased IL-10. Olanzapine (100 uM) and aripiprazole (20 uM) increased BDNF expression.
**Discussion:** All the APs studied upregulated pJNK, along with olanzapine-associated increases in IL-6, and aripiprazole-associated decreases in IL-10, together suggesting AP-mediated upregulation of pro-inflammatory pathways in rHypoE-19 neurons. Aripiprazole and clozapine (but not olanzapine) inhibited insulin-stimulated AKT, suggesting impaired hypothalamic insulin action by some, but not all, APs. Clozapine additionally increased AMPK phosphorylation (activation), an orexigenic energy sensor, which would also be expected to disrupt energy homeostasis. Conversely, olanzapine and aripiprazole increased BDNF, a factor linked to the underlying etiology of schizophrenia, suggesting BDNF upregulation may be a mechanism of therapeutic action. Taken together, our findings suggest differential and pleotropic effects of APs on neuroinflammation and energy sensing in the hypothalamus, which do not necessarily align consistently with known metabolic liability of these agents (i.e. clozapine = olanzapine > aripiprazole). Our data warrants further exploration into the mechanism of these effects, including replication of these effects in an in vivo model.

### F236. CLONIDINE NORMALIZES MMN IN SCHIZOPHRENIA PATIENTS ON STABLE MEDICATION

Caitlyn Kruiper*[,1], Birte Glenthoj[2], Bob Oranje[1]
*[1]Center for Neuropsychiatric Schizophrenia Research (CNSR) and Center for Clinical Intervention and Neuropsychiatric Schizophrenia Research (CINS), Mental Health Center Glostrup, University of Copenhagen, University Medical Center Utrecht, Utrecht University; [2]Center for Neuropsychiatric Schizophrenia Research (CNSR) Center for Clinical Intervention and Neuropsychiatric Schizophrenia Research (CINS), Copenhagen University Hospital, Psychiatric Center Glostrup*

**Background:** Schizophrenia is a severe brain disorder with profound deficits in prefrontal cortical cognitive functioning. These cognitive deficits form a core feature in schizophrenia for which treatment has been proven to be clinically challenging. One of the key neurotransmitters involved in cognitive functioning is noradrenaline. Previous research has demonstrated disrupted noradrenergic activity in schizophrenia while several studies report improvements in prefrontal cognitive functioning by a selective α2-agonist. Clonidine is such a selective α2A-agonist and previous research in our lab has demonstrated that a (range of) single dosage(s) of clonidine normalize(s) sensory gating in chronically ill, yet stably medicated patients with schizophrenia. Currently, we investigated if clonidine also normalizes Mismatch Negativity (MMN) deficits in this same group of patients. This is important, since reports have shown that MMN amplitude is associated with negative symptoms and cognitive functioning in schizophrenia.
**Methods:** In a pseudo-randomized, double-blind, placebo-controlled experiment twenty chronically ill, yet stably medicated, male patients with schizophrenia were tested with the MMN paradigm from the Copenhagen Psychophysiological Test-Battery (CPTB) on 5 different occasions, each separated by a week. Four hours prior to testing patients were randomized administered either a placebo (non psycho-active compound) or a single dose of 25, 50, 75 or 150 µg of clonidine (Catapresan) on top of their usual medication on each occasion, in such a way that each patient received every dose once. Patients were matched on age and gender with 20 healthy controls (HC), who did not receive any treatment. The MMN paradigm consisted of 1800 stimuli with 4 types of stimuli: 1 standard (1000 Hz, 50 ms) presented 82% of the time and 3 types of deviants, based on either frequency (FreqMMN: 1200 Hz, 50 ms), duration (DurMMN: 1000 Hz, 100 ms) or their combination (FreqDurMMN: 1200 Hz, 100 ms) each with a probability of 6%. All stimuli had an intensity of 75dB and were presented with an interstimulus interval randomized between 300 and 500 ms. Subjects were requested to ignore all stimuli and were therefore watching a (muted) nature documentary.
**Results:** In the placebo condition, patients had significantly reduced DurMMN and FreqDurMMN amplitude compared to HC, whereas FreqMMN did not show significant group differences. Furthermore, all doses of clonidine normalized FreqDurMMN amplitude in such a way that it was not significantly different anymore from the HC, while DurMMN only normalized with the highest dose (150ug).
**Discussion:** Our results indicate disrupted MMN amplitude in chronically ill patients with schizophrenia in spite of the fact that they were stable on their medical treatment. In addition, our data provide evidence that a single dose of clonidine is able to normalize MMN amplitude in these patients. Furthermore, patients could not distinguish between the placebo and the treatment conditions or reported any side effects of these low doses of clonidine. Together with our previous reports indicating normalized sensory and sensorimotor gating in these patients following administration of clonidine, our results could be of potential high clinical relevance in the treatment of schizophrenia. Future studies should therefore focus on longer trial periods to investigate if clonidine, besides normalizing MMN amplitude and sensory(motor) gating, can also ameliorate negative symptoms and cognitive functioning in schizophrenia.

### F237. DOPAMINERGIC EFFECTS ON HIERARCHICAL PREDICTION ERRORS AND CONNECTIVITY DURING SOCIAL LEARNING

Daniel Hauke*[,1], Andreea Diaconescu[2], Katharina Wellstein[2], Sara Tomiello[2], Lionel Rigoux[3], Jakob Heinzle[2], Klaas Enno Stephan[2]
*[1]University of Basel, UPK; [2]University of Zurich, ETH Zurich; [3]Max Planck Institute for Metabolism Research*

**Background:** Persecutory delusions (PD) constitute core symptoms in psychosis that may emerge from aberrant learning and inference about others' intentions. Computational assays that use generative models of electrophysiological data to probe this learning process and its underlying neuronal mechanisms, in particular the effects of dopamine (DA) on synaptic plasticity, could provide mechanistic insights into the emergence of PD in psychosis. More importantly, they could enable prediction of individual treatment response to DA antagonists and thus help to address an important problem of clinical management of psychosis.
**Methods:** We tested 137 healthy volunteers (mean age: 22 ± 3) in a double-blind, placebo-controlled, between subject pharmacological study: placebo (n = 47), DA precursor L-Dopa (n = 45), and DA receptor antagonist amisulpride (n = 45). Electroencephalography was recorded using a 128-channel Brain-Vision system.
Participants performed a social learning task that required learning about an adviser's intentions and how they changed over time. Subsequently, we modeled participants' behavior with the hierarchical Gaussian filter (HGF), a model in which learning is driven by hierarchical prediction error (PE) updates: At the first level, positive PEs indicate that advice was better than expected (advice PE: aPE). At the second level, a positive PE signals that the adviser's intentions were less stable than predicted (volatility PE: vPE). Using the trial-wise estimates from the HGF, we performed single-trial EEG analyses of PE activity at sensor and source levels. We also examined DA effects on effective connectivity with dynamical causal modelling (DCM). To this end, we divided event-related potentials (ERPs) according to PE magnitudes into 2 bins corresponding to positive and negative PEs.

**Results:** At the sensor-level, we identified distinct temporal profiles of hierarchical PEs (peak effects: aPE at 112ms, vPE at 276ms). In source space, three sources showed significant effects for both PEs: anterior temporoparietal junction (TPJ), dorsal middle cingulate cortex (MCC) and supplementary motor cortex (SMA).
To identify the connections that convey PEs, we compared two DCM families that allowed input to different nodes of the network, and different modulatory effects of PE magnitude. The family with input entering the SMA and propagating via MCC to TPJ explained aPE-evoked activity best, whereas the family with input into the TPJ and propagating in the opposite direction best described the effects of vPE-evoked activity. Bayesian model selection identified the winning model for aPE effects; this model proposed PE magnitude modulations of input gain and effective connectivity from TPJ to MCC, and MCC to SMA. Conversely, a model with connectivity modulation from MCC to TPJ best described the effects of vPE. Second, we investigated the impact of dopaminergic perturbations of the network by comparing DCM parameters of the winning models across pharmacological groups. Post hoc t-tests revealed that DA impacted on aPE-induced perturbations only, which is in line with previous findings that aPEs are represented in dopaminergic regions while vPEs are likely encoded by activity in cholinergic regions. Specifically, DA modulated TPJ-MCC and SMA-MCC connectivity.
**Discussion:** Model-based analysis of EEG data in a social learning task detects DA effects on connectivity, even when behavior (accuracy, reaction time) was not affected by the drugs. Currently, we are extending our computational approach to first-episode schizophrenia patients, where we hope to use parameters from neuronal and behavioral models to predict individual treatment response.

### F238. COMPETENCE-PERFORMANCE DISCREPANCY IN SOCIAL FUNCTIONING IN PATIENTS WITH SCHIZOPHRENIA: THE IMPACT OF SOCIAL ANXIETY

Takahiro Nemoto*[,1], Takashi Uchino[1], Sayaka Aikawa[1], Tomoyuki Funatogawa[1], Hiroshi Matsumoto[1], Taiju Yamaguchi[1], Naoyuki Katagiri[1], Naohisa Tsujino[1], Kei Sakuma[1], Masafumi Mizuno[1]
*[1]Toho University School of Medicine*

**Background:** Social functioning deficits are of critical importance in patients with schizophrenia, because they affect the long-term outcomes and quality of life (QOL) of the patients. Two aspects of social functioning, namely, competence (ability to perform skilled activities, that is, what one can do) and performance (actual performance of skilled activities, that is, what one actually does) are considered to have a significant influence on how well the patients can live independently in the community. Although the two aspects are usually thought to go hand in hand, discrepancy between the two is often observed in patients with schizophrenia in clinical practice. Some patients are not able to function in the community to the best of their ability; some patients appear to get along everyday living better than they would be expected to. The aim of the present study was to identify factors influencing the occurrence of such discrepancy of social functioning in patients with schizophrenia.
**Methods:** A total of 205 stable outpatients with schizophrenia aged 40 years old or under were recruited at the Toho University Omori Medical Center, Tokyo. Of the 205 patients, 100 were male (48.8%) and 105 (51.2%) were female. The mean age of the participants was 29.3 years and the mean estimated premorbid IQ was 100.8. The mean age at disease onset was 22.0 years old, and the mean duration of illness at the start of the study was 6.7 years. The social functioning, psychiatric symptoms, social anxiety, cognitive function, and QOL of the participants were assessed. The patients were divided into 4 groups by the cutoff points for competence and performance calculated using a comprehensive dataset of the Social Functioning Scale (SFS) obtained from multiple facilities.
**Results:** The subjects were divided according to their level of competence and performance as follows: good competence and good performance (CP) group, 108 (52.7%) patients; good competence but poor performance (Cp) group, 40 (19.5%) patients; poor competence but good performance (cP) group, 13 (6.3%) patients; poor competence and poor performance (cp) group, 44 (21.5%) patients. Among the 4 groups, the objects of particular interest in this study were the differences between CP and Cp groups and between the cP and cp groups. One-way ANOVA revealed significant differences among the groups in the scores on the Positive and Negative Syndrome Scale (PANSS), Liebowitz Social Anxiety Scale (LSAS), Global Assessment of Functioning Scale (GAF), World Health Organization-Quality of Life 26 (WHOQOL26), and Social Functioning Scale (SFS). Post-hoc comparisons revealed that the PANSS negative symptoms and general psychopathology scores, GAF score, WHOQOL26 score, and SFS total score were significantly worse in the Cp group than in the CP group, and that the LSAS score, GAF score, WHOQOL26 score, and SFS total score were significantly better in the cP group than in the cp group.
**Discussion:** In patients who are capable of living well in the community but do not perform well, negative symptoms may be involved in this discrepancy of social functioning. Patients who are able to maintain themselves well despite their poor social competence appear to have milder social anxiety symptoms as compared to patients who are neither competent nor capable of performing well in terms of social functioning in the community. Suitable and personalized approaches based on the patients' profile of dysfunction would seem to be indispensable for the recovery of such patients.

### F239. THE ROLE OF DANCE/MOVEMENT THERAPY IN THE TREATMENT OF NEGATIVE SYNDROME AND PSYCHOSOCIAL FUNCTIONING OF PATIENTS WITH SCHIZOPHRENIA: RESULT FROM A PILOT MIXED METHODS INTERVENTION STUDY WITH EXPLANATORY INTENT

Karolina Bryl*[,1]
*[1]Drexel University, College of Nursing and Health Professions*

**Background:** Optimizing psychosocial functioning and overall well-being by reducing the severity of negative symptoms are important outcomes for individuals with schizophrenia. Movement-based therapeutic approaches are uniquely capable of addressing the non-verbal nature of negative symptoms. Dance/Movement Therapy (DMT), a promising treatment for mental health conditions such as schizophrenia, has been found to reduce the occurrence and severity of negative symptoms and to have a positive impact on the psychosocial functioning. Although preliminary findings suggest DMT as a treatment intervention, limited research and inconclusive findings preclude generalizations and more research is needed. We aimed to examine the treatment effects of a 10-week (20 sessions) group DMT treatment program.
**Methods:** We employed a mixed methods intervention design with explanatory intent, in which a randomized controlled trial is followed by semi-structured exit interviews. Thirty-one severely ill individuals diagnosed with schizophrenia participated in the RCT that used a two-arm parallel group design to assess and show the difference between patients receiving standard care (SC) and patients receiving standard care plus DMT on measures of negative symptoms (as primary outcome; PANSS, BNSS) and psychosocial functioning (as secondary outcomes; WHO-DAS 2.0, SDS). Quantitative measures were taken pre and post- intervention. Participants who participated in a minimum of 50% of DMT sessions (n=15) were invited to an exit interview. This criterion was also used to analyze quantitative data, leaving n=28 for quantitative analysis.

**Results:** All participants in both groups (n=31) completed the study. Because of such a small sample size (n=28) and a pilot nature of the study we were restricted to use descriptive statistics.

The quantitative data suggest that DMT and SC were not equally effective in enhancing primary outcomes. Analysis of the PANSS mean score changes showed a slight increase in the negative symptom in the DMT from 28.33 ± 4.76 to 29.00 ± 4.10, and slight decrease in the SC from 28.92 ± 5.72 to 27.08 ± 5.64. BNSS scores indicate that both groups improved. SC participants reported grater reduction on BNSS overal score from 53.31 ± 11.48 to 47.77 ± 8.10 in comparison to DMT from 53.07 ± 7.27 to 51.93 ± 6.18. However, DMT participants reported reduction of symptoms in distress, antisocial activity, avolition and verbal expression.

Analysis of WHO-DAS suggests that DMT was effective in reduction of disability severity compared to SC. DMT participants reported grater improvement in cognition, mobility, self-care, and getting along. Both groups reported reduction of the impact of difficulties on daily functioning on SDS, however DMT participants reported a greater reduction in days during which they were completely unable to perform or had to limit their usual activities or work due to symptoms. In the SC, the results suggest a reduction in the number of days lost and days of lower productivity.

Qualitative findings identified participants' experiences and the most important themes related to benefits of the DMT intervention: enhanced activation, motivation, socialization, and self-awareness.

**Discussion:** Results of this study contribute to knowledge about body-based interventions for schizophrenia and indicate that DMT had an effect on participants psychosocial functioning and coping with negative symptoms. Integration of quantitative and qualitative data provides a wider perspective by gaining a better understanding of the treatment outcomes and explaining inconclusive results. Findings of this study set the stage for larger fully powered research, examining intervention methods and procedures, as well as treatment effects, more thoroughly.

### F240. MULTI-MODAL PREDICTION OF GLOBAL FUNCTION FROM NEUROCOGNITIVE AND NEUROIMAGING MEASURES: OUTCOMES FROM THE PRONIA STUDY

John Gillam[1], Dominic Dwyer*[,2], Anne Ruef[2], Nikolaos Koutsouleris[2], Stephen Wood[3]

*[1]University of Melbourne; [2]Ludwig-Maximilian-University; [3]Orygen, the National Centre of Excellence in Youth Mental Health*

**Background:** In order to extract the most powerful predictive models from data collected within the PRONIA study, diverse information sources must be combined. PRONIA aims to combine information from a range of study sites across Europe as well as from a diverse range of information sources. For each subject, neurocognitive, neuroimaging and clinically observed data has been collected that is intended to provide the basis for the development of predictive models for use in individualised diagnosis and prediction. However, as yet it is unclear as to which elements (or combination) of the measured data provide optimal predictive capacity and which features will generalize best.

**Methods:** In order to combine data from a diverse range of sources a number of approaches may be considered. While it is initially attractive to concatenate the features gathered from each modality, this approach is problematic in two ways. Not only do the appropriate pre-processing steps differ between modalities, but the high dimensionality of imaging data (in comparison to neurocognitive measures) may alter the way each modality contributes to the decision function during learning. Instead, we investigate more simplistic learning approaches in an initial step that produces a single outcome for each modality considered. In a second step these outcomes are combined to generate a final estimate of the target class.

In this investigation neurocognitive and neuroimaging data, collected as part of the PRONIA study, were considered as features for prediction of clinically observed global function, measured at the same time-point. Each neurocognitive test, applied as part of the PRONIA battery, was considered as an independent modality, as were each of a range of MRI-based neuroimaging measures (from structural, functional and diffusion imaging). Support Vector Classification (SVC) was conducted for each modality, with the target class defined as a score of 65 or less on the Global Assessment of Function. Both linear classification and the use of radial basis functions were explored within the initial modality-independent learning phase as well as during modality fusion as part of the second learning phase. Repeated, nested, cross-validation was employed in both stages in order ensure robust estimates of generalisation.

**Results:** Because each modality is reduced to a single measure in the first stage, each can contribute on an equal basis to the predictive outcome in the second while allowing inter-modality interaction. While SVC models do not naturally provide probabilistic outcomes, the distance of each point to the separating hyperplane can be scaled to represent the relative class probabilities. Predictions obtained at the first stage not only provide for the second phase of learning, but also provide a means to assess each modality for predictive accuracy. Correlations between the predictions from each mode provide information as to which combination of data may contribute constructively to the final outcome while learning approaches within the second phase can also be used to identify the most useful predictors.

**Discussion:** The two-stage learning framework provides a useful approach to learning that allows assessment of each separate data stream as well as the fused-prediction outcome. The contribution of each data stream to the final prediction may be explored while interactions between data streams can also be contextualised. However, more subtle interactions between data, particularly at the initial input stage, may be difficult to observe and so the extension of this approach to more structured data-fusion and is considered.

### F241. DEVELOPMENT AND VALIDATION OF THE SOCIAL FUNCTIONING ASSESSMENT SCALE (SFAS) FOR PEOPLE WITH SCHIZOPHRENIA IN TURKEY

Mustafa Yildiz*[,1], Fatma Kiras[1], Aysel İncedere[1], Duygu Esen[1], Mehmet Buğrahan Gürcan[1], Betül Abut[1], Kübra İpçi[1], Güzide Örüm[2], Ümit Tural[1], Güliz Özgen[2]

*[1]Kocaeli Universitesi; [2]Bakirkoy Ruh Sagligi ve Hastaliklari Hastanesi*

**Background:** Social functioning is generally defined as having profound and qualitative interpersonal relations, and meeting the expectations and defined roles in society. Determining the level of the social functioning is important especially for people with schizophrenia that proceed with disabilities. Main dimensions of the social functioning are 1) Self-care, 2) Independent living, 3) Interpersonal relationships (family, friends, neighbors, etc.), 4) Leisure time and recreation, and 5) Occupational activities like school or job. The purpose of this study was to develop a culturally-sensitive, user-friendly scale that could assess the social functioning of the people with schizophrenia.

**Methods:** After examining the studies assessed social functioning in people with mental illnesses, an original 50-item scale was formed. Habits of 425 people living in the community was examined with this form so as identifying the prevalence and frequency of behavior patterns related with social functioning in Turkish community. Regarding the findings of that study, 28-item scale was formed that assess the social functioning of the patients. New form was given to 25 patients, and items which was difficult to comprehend were reevaluated and the scale was finalized as Social Functioning Assessment Scale. One hundred and thirty outpatients with schizophrenia or schizoaffective disorder were given a sociodemographic form, Social Functioning Assessment Scale (SFAS), Clinical Global Impression-Severity (CGI-S), and Global Assessment of Functioning (GAF). At the same time, Social Functioning Scale (SFS) and SFAS was given to the relatives of the patients who live together. For reliability analyses; internal consistency coefficient, item-total correlation, and split-half reliability was assessed. For validity analyses; explanatory factor analysis, and convergent validity were examined via Spearman correlation.

**Results:** The data from 104 patients with schizophrenia and 26 with schizoaffective disorder whose 75% were males, 69% were single, mean age was 37, the level of education was 10 years was examined. The average onset of the illness was 23 years, and the duration of illness was 14 years. Cronbach's alpha coefficient for SFAS total score was .83, and for factors were between .69 and .77. Split-half reliability coefficient of SFAS was .73. There was a satisfactory correlation between SFAS filled by patients and by relatives (r=.60, p<0.001). For factor analysis, Kaiser-Meyer-Olkin value was .78, and Barlett test was significant (p<0.001). In explanatory factor analysis, SFAS was found to be compose of three factors (self-care, interpersonal relationships and recreation, independent living) and that they can explain 45% of the total variance. Nine items were omitted because of having lower factor value than .40. Self-care factor had 7-item, interpersonal relationships and recreation factor had 7 items and independent living factor had 4 items. Occupational life could not get in any of factors; however, since it was very important for social functioning, it was added to the scale as fourth factor.

SFAS total score was correlated with PANSS negative subscale (r=-.35, p<0,001), PANSS-total (r=-.29, p<0,001), CGI-S (r=-.33, p<0,001), GAF (r=.28, p<0,001) and SFS total score (r=.52, p<0,001).

**Discussion:** Regarding the findings of the study, SFAS was considered a culturally-sensitive, easy-to-use, and valid instrument that objectively assesses the social functioning of the patients with schizophrenia in Turkey.

### F242. CHILDHOOD ADVERSITY AND PSYCHOTIC EXPERIENCES IN THE GENERAL POPULATION: WHAT IS THE PREDICTIVE ROLE OF RESILIENCE, COPING STYLE AND SOCIAL SUPPORT?

David Mongan*[,1], Ciaran Shannon[2], Donncha Hanna[3], Adrian Boyd[4], Ciaran Mulholland[2]

*[1]Belfast Health and Social Care Trust; [2]Northern Health and Social Care Trust/ Queens University Belfast; [3]Queens University Belfast; [4]Northern Health and Social Care Trust*

**Background:** A history of childhood adversity is known to be associated with psychotic disorder as well as subclinical psychotic-like experiences. This study aimed to examine the relationship between specific types of childhood adversity and psychotic-like experiences in a general population sample, and to determine the predictive role of psychological resilience, coping style and perceived social support.

**Methods:** An online survey was conducted with a US-based general population sample of 748 participants (aged 18 – 35 years) using Amazon's Mechanical Turk (an online crowd-sourcing service). Participants completed the following validated measures: the Adverse Childhood Experiences Questionnaire (ACE-Q) as a measure of childhood adversities, the Prodromal Questionnaire (PQ-16) as a measure of psychotic experiences, the Brief Resilience Scale (BRS) measuring level of psychological resilience, the Brief COPE Scale as a measure of predominant coping style, the Multidimensional Scale of Perceived Social Support and the Neighbourhood Cohesion Scale. A series of backwards stepwise hierarchical regression analyses was employed to determine predictors of PQ-16 score.

**Results:** Participants reported an average of 2.99 attenuated psychotic symptoms (from a total of 16 on the PQ-16), and an average of 2.77 childhood adversities (from a total of 10 on the ACE-Q). In the final regression model, which explained 33% of the variance in PQ-16 score, the specific types of childhood adversity which significantly predicted PQ-16 score were verbal abuse, sexual abuse and physical neglect. Level of resilience and coping via emotional support were significant negative predictive factors of PQ-16 score. The coping styles of self-distraction, denial, substance use, venting, religion and self-blame were significant positive predictors of PQ-16 score. Perceived social support and neighbourhood cohesion were not significant predictors.

**Discussion:** The results of this study add support to the relationship between history of childhood adversity and psychotic-like experiences in the general population. Our data suggest that a differential effect exists dependent on the specific type of adversity (the strongest observed effect was for physical neglect). These findings highlight the need for routine clinical enquiry regarding childhood trauma for patients experiencing attenuated psychotic symptoms. We also found that psychological resilience and coping style were important predictive factors in this relationship (whilst perceived social support and neighbourhood cohesion were not). These may represent possible avenues for psychosocial augmentative interventions in the early stages of the psychosis continuum.

### F243. INFLUENCE OF METACOGNITION AND IRRATIONAL BELIEFS ON SOCIAL FUNCTIONING IN PSYCHOSIS OF RECENT ONSET

Helena García-Mieres*[,1], Raquel López-Carrilero[2], Jordi Cid[3], Esther Pousa[4], Ana Barajas[5], Eva Grasa[6], Maria Luisa Barrigon[7], Isabel Ruiz-Delgado[8], Ana de Apraiz[2], Fermín González-Higueras[9], Esther Lorente Rovira[10], the Spanish Metacognition Study Group, Susana Ochoa[2]

*[1]Universitat de Barcelona; [2]Parc Sanitari Sant Joan de Deu; [3]Salut Mental Girona; [4]Institut de Neuropsiquiatria i Addiccions, Hospital del Mar; [5]Centro de Higiene Mental Les Corts, Research Unit; [6]IIB-Hospital Santa Creu I Sant Pau; [7]Fundación Jiménez Díaz Hospital; [8]Servicio Andaluz Salud Málaga; [10]Servicio Andaluz de Salud; [10]ADIRM*

**Background:** Social functioning is affected in early psychosis stages. This affection has multiple domains, such as vocational functioning or performance of independent living skills. These different domains are also linked; so elucidating differential or generalized determinants on specific areas and global outcomes is thus a critical step in case conceptualization and the development planning of effective early interventions. The aim of this study was to test the influence of specific domains of metacognition in different and global areas of social functioning.

**Methods:** A cross-sectional study was performed based on baseline data from a main multicenter clinical trial. The sample was composed of 122 patients with psychosis of recent onset treated at one of the nine participating mental health centers from diverse regions of Spain. The order of assessment was a sociodemographic questionnaire, the Positive and Negative Syndrome Scale (PANSS), the Social Functioning Scale (SFS), the Hinting Task (Theory of Mind, ToM), the Beck Cognitive Insight Scale (BCIS), the Internal, Personal and Situational Attributions Questionnaire (IPSAQ), the Irrational Belief Test (TCI) and the Emotional Recognition Test Faces. Pearson correlations and multiple regression analysis were performed.

**Results:** In the first models, results showed that social engagement/withdrawal was explained by Helplessness (9.2% of the variance). Interpersonal communication was explained by Emotional Irresponsibility, internal attribution of negative events, affective JTC and emotion recognition (17.5% of variance). Independence-competence was explained by Helplessness, Emotional Irresponsibility and ToM (16% of variance). Independence-performance was explained by Helplessness (8.2% of variance). Employment/occupation was explained Emotional Irresponsibility (12.4% of variance). Prosocial Activities was explained by Helplessness and

Emotional Irresponsibility (14.4% of variance). Finally, the total score of the SFS was explained by Helplessness and self-reflectiveness (16% of variance). Subsequently, in a second analysis, negative symptoms emerged as a significant mediator for most domains of social functioning.
**Discussion:** In our results, two kind of irrational beliefs, one of the main axes of cognitive therapy, emerged as relevant for social functioning in psychosis of recent onset. However, classic social cognition and metacognition measures were less significant, only ToM and self-reflecteness influenced some aspects of social functioning. Further analysis of determinants of social functioning in psychosis should explore the role of irrational beliefs and consider them for treatment strategy, along social cognition and negative symptoms.

### F244. CHILDHOOD PSYCHOTIC EXPERIENCES ARE ASSOCIATED WITH PERSISTENTLY POORER FUNCTIONING INTO YOUNG ADULTHOOD: A 9-YEAR FOLLOW-UP STUDY

Dónal Campbell*[,1], Colm Healy[1], Mary Cannon[1]
[1]*Royal College of Surgeons in Ireland*

**Background:** Psychotic experiences (PEs) are relatively common in childhood and early adolescence, being present in 17% of children aged 9 to 12 (Kelleher et al., 2012). Research suggests that young people who experience PEs are more vulnerable to psychopathology later in life, despite PEs being transient in 78.7% of cases (Zammit et al., 2013). While childhood PEs are associated with poorer functioning (Kelleher et al., 2015), it has not yet been established whether the impact of PEs on functioning persists into later life.
**Methods:** 52 participants from a prospective cohort study (retention rate: 60.4%) of Irish young people were included on the basis that they had completed a clinical interview at all three data-collection time points (T1 mean age: 11.69; T2: 15.80; T3 18.80). Following each interview, participants were scored on the Global Assessment of Functioning (GAF) scale, and given a Current (C-GAF) score and a Most Severe Past (MSP-GAF) score. Fixed-effects repeated-measures models were used to compare the scores of those with a history of PEs at T1 (n=18) to those without (n=34), accounting for age, gender, and childhood functioning. Secondary analyses investigated whether differences in functioning were evident in those who reported transient PEs (only at T1; n=12).
**Results:** Overall, participants who had reported childhood PEs (T1) received significantly lower C-GAF scores ($F = 31.553$, $p < .001$) and MSP-GAF scores ($F = 79.377$, $p < .001$) than those without PEs. Simple effects analysis indicated that deficits in the PE group were evident at each time point for both C-GAF scores (T1: $p = .001$; T2: $p <.001$; T3: $p = .002$) and MSP-GAF scores (T1: $p < .001$; T2: $p = .001$; T3: $p < .001$), indicating poorer functioning from childhood, through adolescence, into early adulthood. There was no significant effect of the co-variates.
When the analysis was restricted to a comparison of participants who reported PEs at T1 only (i.e. transient PEs) and those with no history of PEs, the PE group had poorer functioning scoring than their peers across the three time points (C-GAF: $F = 17.709$, $p < .001$; MSP-GAF: $F= 32.247$, $p < .001$).
**Discussion:** The analysis provides longitudinal evidence that the presentation of PEs is associated with persistent poor global functioning throughout adolescence and into early adulthood, even when the phenomena are transient. PEs appear to be a marker for vulnerability that extends beyond mental disorder. These results tentatively suggest a causal link between PEs and poorer functioning later in life, as the difference in functioning between the groups in early adulthood was still evident after accounting for childhood functioning. Moreover, the disparity between the groups is clinically relevant, with the PE group scoring one to two categories lower than their peers on the GAF scale even into early adulthood. Childhood PEs are an excellent prognostic marker for future functioning and providing targeted early intervention for these individuals may reduce the likelihood of developing a significant clinical disorder later in life.

### F245. COGNITIVE RESERVE DIFFERENCE IN AFFECTIVE AND NONAFFECTIVE PSYCHOSIS

Silvia Amoretti*[,1], Bibiana Cabrera Llorca[2], Gisela Mezquida[2], Manuel J. Cuesta[3], Mara Parellada[4], Ana Gonzalez-Pinto[5], Iluminada Corripio[6], Eduard Vieta[7], Miquel Bernardo[1]
[1]*Hospital Clinic of Barcelona, University of Barcelona;* [2]*Hospital Clinic Barcelona, Institut Clinic Neurociencies;* [3]*Complejo Hospitalario de Navarra, IdiSNA, Navarra Institute for Health Research;* [4]*Hospital General Universitario Gregorio Marañon, Universidad Complutense de;* [5]*Hospital Universitario Araba, Universidad del País Vasco;* [6]*Hospital Santa Creu i St. Pau;* [7]*Institute of Neuroscience, Hospital Clinic, University of Barcelona*

**Background:** The cognitive reserve (CR) refers to the capacity of an adult brain to cope with pathology in order to minimize the symptoms (Stern, 2002). Recent studies have shown that CR is associated with clinical, functional and cognitive outcomes in patients with severe mental illness (de la Serna et al., 2013; Forcada et al., 2015; Anaya et al., 2016; Amoretti et al. ., 2016; Grande et al., 2017). Higher CR has been related to a later onset of psychosis, greater adherence and fewer psychotic symptoms (Barnett et al., 2006). However, there are no studies that evaluate longitudinally the role of CR depending on the diagnosis.
The objective is to analyze the impact of CR according to the diagnosis and to study whether having a high CR may be associated with better clinical, functional and cognitive outcomes.
**Methods:** We gathered all the relevant clinical and sociodemographic data. All subjects were assessed clinically, neuropsychologically and functionally at baseline and after a two-year follow-up. To assess CR, three proxies have been integrated: premorbid IQ, years of education-occupation and leisure activities. To determine whether the level of CR was associated with clinical, functional and neuropsychological outcomes and whether it was different between diagnoses, a multivariate analysis of variance was used.
**Results:** 285 DSM-IV patients with first episode of psychosis (FEP) were enrolled. The sample was divided into affective and non-affective groups.
In the non-affective group, those with high CR are older and had a better socioeconomic status, better functioning and cognitive performance and lower symptoms, as well as a shorter duration of untreated psychosis (DUP) and a later age of onset. After 2 years of follow-up, they showed significant differences in all the cognitive domains evaluated, except for the executive functions.
In the affective group, the patients with high and low CR showed differences in positive and manic symptoms, as well as in verbal memory at baseline. At 2 years of follow-up the differences were observed in functionality, positive and negative symptoms and in verbal memory. There were no significant differences in terms of age, gender, DUP, or age of onset, although significant differences were found in socioeconomic level ($p = 0.038$).
**Discussion:** Higher CR can result in better recovery and functioning and in higher cognitive performance in patients with a FEP. Therefore, we propose that early interventions focused on the promotion of neuropsychological abilities and CR could reduce the harmful impact of this disease.
However, it is necessary that these interventions should be personalized taking into account that CR plays a differential role according to the diagnosis.

### F246. A SYSTEMATIC REVIEW COMPARING THE NEURAL CORRELATES OF EMPATHY ASSOCIATED WITH THE ONSET AND PROGRESSION OF SCHIZOPHRENIA

Meneshka Ponnampalam*[,1]
[1]*The University of Birmingham*

**Background:** Empathic deficits present in nearly all Schizophrenia patients (SCZ). These result from impairments in various social cognitive tasks, often leading to social isolation and withdrawal. There is evidence that empathy deficits occur before illness-onset in those at ‘ultra-high risk’ of psychosis (UHR) and those with a ‘first-episode of psychosis’ (FEP). Empathy defects are associated with neurological abnormalities, which have been studied separately in UHR, FEP and SCZ populations. This review aims to gain further insight into neurological changes associated with illness progression, by comparing brain changes associated with empathy across UHR, FEP and SCZ populations.

**Methods:** Studies considering functional activity, connectivity and structural changes in UHR, FEP and SCZ populations were systematically reviewed. Data from 26 studies was used.

**Results:** All three subgroups showed abnormal patterns of activation and connectivity across a range of regions, particularly in the frontal, limbic and temporal areas. Structural abnormalities appeared as widespread grey matter loss, largely in the temporal lobe, across all three participant groups. Notably, impaired empathic behavioural responses were found in FEP and SCZ subjects only, despite abnormal brain patterns in all three groups.

**Discussion:** Our findings suggest that abnormal connectivity, structure and activation of the frontal, limbic and temporal areas contribute significantly to empathy deficits, and also worsen with illness progression. However, the multifaceted nature of empathy means that behavioural impairments likely result from a combination of disruptions of the frontal, limbic and temporal areas as well as many other neural networks involved in social information processing.

### F247. INTERNALIZED STIGMA HAS A STRONGER RELATIONSHIP WITH INTRINSIC MOTIVATION COMPARED TO AMOTIVATION IN EARLY PHASE AND PROLONGED SCHIZOPHRENIA

Ruth Firmin*[,1], Lauren Luther[2], Paul Lysaker[3], Jennifer Vohs[4]
*[1]UCLA; [2]Indiana University, Purdue University; [3]Roudebush VA Medical Center, Indiana University School of Medicine; [4]Indiana University School of Medicine*

**Background:** Motivation deficits predict decreased functioning in schizophrenia. Recent work suggests deficits reflect challenges in separate domains: intrinsic motivation (one’s internal drive to engage in a behavior out of enjoyment or interest) and amotivation (one’s broader decrease in motivated behavior linked to avolition and anhedonia). Internalized stigma is another determinant of functioning for people with schizophrenia that may impact motivation. However, little is known about these relationships, including which aspects of motivation it may impact nor when these links emerge. Identifying the link between these constructs may help to identify whether internalized stigma may be a novel treatment target to facilitate improvements in motivation.

**Methods:** Forty adults with early phase schizophrenia and 66 adults with prolonged schizophrenia completed measures of internalized stigma, intrinsic motivation, and amotivation. Pearson’s correlations were examined followed by Fischer’s r-to-z transformations to compare differences in the magnitude of associations between internalized stigma and intrinsic motivation and internalized stigma and amotivation among the first episode and prolonged samples. Next, we conducted stepwise regressions to examine whether internalized stigma was associated with intrinsic motivation above and beyond associations with amotivation in each sample.

**Results:** In the early phase sample, the association between internalized stigma was greater with intrinsic motivation ($r=-0.48$, $p=.00$) compared to amotivation ($r=0.27$, $p=0.10$). Associations with internalized stigma in the prolonged sample were also greater with intrinsic motivation ($r=-0.30$, $p=0.02$) versus amotivation ($r=0.19$, $p=0.12$). The magnitude of the associations between internalized stigma and intrinsic motivation ($z=1.03$, $p=0.15$) and between internalized stigma and amotivation ($z=0.41$, $p = 0.34$) did not significantly differ when comparing phase of illness. Regression analyses indicated that, controlling for amotivation, internalized stigma predicted intrinsic motivation in both the prolonged sample ($R2=0.09$, $F(1,64) =6.18$, $p=0.02$) and the early phase schizophrenia sample ($R2=0.23$, $F(1,37)=10.98$, $p=.00$).

**Discussion:** Results suggest internalized stigma has a stronger relationship with intrinsic motivation separate from, and above and beyond, its association with amotivation. Findings support models of intrinsic and amotivation being distinct domains. Links between internalized stigma and motivation appear to emerge and persist from the early stages of schizophrenia, suggesting that targeting stigma in early intervention services may help to improve intrinsic motivation in people with schizophrenia.

### F248. COMMUNICATIVE-PRAGMATIC IMPAIRMENT IN SCHIZOPHRENIA: THE ROLE OF EXECUTIVE FUNCTION AND THEORY OF MIND

Alberto Parola*[,1], Laura Berardinelli[2], Francesca M. Bosco[3]
*[1]Center for Cognitive Science, University of Turin; [2]A.S.L. “Città di Torino”; [3]University of Turin, Institute of Neurosciences of Turin*

**Background:** Individuals with schizophrenia frequently exhibit a wide range of communicative-pragmatic disorders. Previous studies reported deicits in the comprehension of non-literal and figurative forms of language, such as indirect speech acts, deceit, irony, metaphors and idioms, as well as deficits in conversational and narrative skills. Moreover, schizophrenia is often associated with impairment in cognitive functions, such as Executive Functions (EF) and Theory of Mind (ToM). Few studies examined at the same time the role that ToM and EF can play in the comprehension of different communicative acts, such as sincere communicative acts, deceit and irony. Thus, the relation between ToM, EF and pragmatic ability in schizophrenia is still not completely clear. The aim of this study is to evaluate the relationship between the ability to manage different communicative pragmatic phenomena (i.e., sincere, deceitful and ironic communicative acts), and ToM and EF.

**Methods:** 26 individuals with schizophrenia and 26 matched controls took part in the study. We evaluated communicative pragmatic-ability using the lingusitic and extralinguistic scales of the Assement Battery for Communication (ABaCo). We assessed EF - working memory, inhibition and cognitive flexibility-, ToM and background cognitive functions - general intelligence, selective attention and speed processing - using a battery of standardized neuropsychological tests.

**Results:** To investigate the presence of significant differences in communicative-pragmatic performance between patients and controls, we performed a 2x3 ANOVA with participant (individuals with schizophrenia, healthy control) as between-subjects factor, and the type of pragmatic phenomena (sincere, deceitful and ironic) as within-subjects factor. For each of the ABaCo subscales, we found a main effect of participant ($.0001 < p. < .001$), showing that experimental group performed significantly worse than control group. We also found a linear trend in pragmatic performance ($.0001 < p. < .008$), that revealed a linear decrease in scores depending on the pragmatic phenomenon investigated: sincere communicative acts were the easiest to understand, followed by deceit and irony. To evaluate the role of cognitive and ToM tasks on pragmatic performance in patients, we performed a regression analysis. We included relevant predictors in the model, i.e. cognitive background factors, EF and ToM. We found that the only significant predictor was ToM, that contributed to increase the quote of explained variance in the comprehension and production of linguistic sincere communicative acts ($p = .005$) and linguistic deceit ($p. = .009$).

**Discussion:** Results showed that individuals with schizophrenia performed poorly in the comprehension and production of different kinds of pragmatic phenomena, i.e. sincere, deceitful and ironic communicative acts. This result confirms that communicative-pragmatic impairment is a core deficit in schizophrenia. In addition, we found an association between ToM and comprehension and production of sincere and deceitful communicative acts, while no association between irony and ToM was found. The results of the present investigation confirm the role that ToM can play in managing sincere and deceitful communicative acts, while do not seem to support previous evidences indicating ToM as the main factors in explaining irony understanding.

### F249. FAMILY BURDEN IN THE US RAISE-ETP PROGRAM: TREATMENT EFFECTS AND PREDICTORS

Shirley Glynn*,[1], Susan Gingerich[2], Piper Meyer-Kalos[3], Kim Mueser[4], Alec Chan-Golston[1], Catherine Sugar[1], Nina Schooler[5], Robert Rosenheck[6], John Kane[7]
*[1]UCLA; [2]Independent Consultant; [3]The University of Minnesota; [4]Boston University; [5]SUNY Downstate Medical Center [6]Yale University; [7]Northwell Health*

**Background:** The Recovery After an Initial Schizophrenia Episode Early Treatment Program (RAISE-ETP) is a US NIMH-funded 34 site cluster randomized controlled trial evaluating the benefits of participation in a multicomponent intervention for first episode psychosis (FEP). Previously, participation in the RAISE-ETP comprehensive specialty care (CSC) program, entitled NAVIGATE, was reported to yield significant participant clinical and functional improvements, compared to customary care (Kane et al, 2016). NAVIGATE included tailored medication, individual resiliency training, family education, and supported education and employment. Family burden has been identified as a key factor in FEP, with high levels of distress often found in relatives. Here, we look at the presence and predictors of family burden in relatives in the RAISE-ETP sample over the two years of study participation.
**Methods:** A total of 404 individuals between ages 15 and 40 were enrolled. DSM-IV diagnoses of non-affective psychosis were included. All participants had experienced only one episode of psychosis, had been prescribed less than 6 months of lifetime psychotic medication, spoke English, and provided informed consent. Participants were offered a minimum of two years of CSC or customary care. At baseline, participants provided demographic and clinical history information; they were administered the Heinrichs-Carpenter Quality of Life Scale (QOL) and the Positive and Negative Symptom Scale (PANSS) regularly throughout the study. Each participant was asked to nominate a family member for administration of the Burden Assessment Scale (BAS) throughout the study. The BAS yields a total score, as well as subscales assessing disrupted activities, personal distress, guilt, time perspective, and worry.
**Results:** Fifty-seven percent of the participants nominated a relative who was assessed with the BAS. Interestingly, the only statistically significant independent predictors of baseline family burden were relatives' reports of their loved ones' dependence and lack of help with chores; no consumer demographic, PANSS, or QOL variables were identified. BAS total scores improved significantly in both conditions, but significantly more in NAVIGATE. Consumer report of better family relationship quality on the QOL was associated with significantly less family burden on the BAS over time, but neither PANSS positive, negative or symptom total, total QOL, nor participation in specific CSC psychosocial components mediated the observed BAS total burden reductions. With regard to the BAS burden components, there was a main effect of improvement over time on family disrupted activities, guilt, time perspective, and worry, with disrupted activities, personal distress, and guilt all evidencing a time by group interaction favoring greater reductions in NAVIGATE.
**Discussion:** As anticipated, family burden is widely evidenced in the relatives of US FEP consumers who are new to treatment. This burden does not appear to reflect unique consumer characteristics. There appears to be a reduction in family burden during the loved one's FEP treatment, with that reduction enhanced when the consumer is participating in a more intensive CSC program. Interestingly, while many potential intervening variables were tested as mediators of the CSC impact on family burden, none were identified. The overall pattern of results suggests that it maybe the very fact of a loved one being enrolled in a treatment program, especially if it is a comprehensive FEP intervention, rather than engagement in specific program components or consumer improvements, that are associated with reductions in family burden over the first year of treatment.

### F250. DEPRESSION IN SCHIZOPHRENIA: CORRELATIONS WITH OBJECTIVE AND SUBJECTIVE QUALITY OF LIFE OUTCOMES

Philip Harvey*,[1], Kimberly Vanover[2], Robert Davis[2], David Penn[3], Amy Pinkham[4]
*[1]Leonard M. Miller School of Medicine, University of Miami; [2]Intra-Cellular Therapies, Inc.; [3]University of North Carolina; [4]University of Texas at Dallas*

**Background:** Schizophrenia is increasing recognized to be associated with symptoms of depression. As many as 40% of people with schizophrenia (SCZ) have a fully syndromal major depressive episode at some time in their lives and the mean severity of depression in unselected samples is often in the mildly to moderately depressed range (mean BDI score of 11–16). While patients who report no depression have been found to report very low levels of subjective distress, a comprehensive study of the subjective quality of life correlates of depression in schizophrenia has not been performed. Further, given the impact of depression on interpersonal functioning, an assessment of the relationships between depression and social cognition is warranted.
**Methods:** Two samples of patients with SCZ (n's= 179 and 218) were compared to samples of HC (n's= 104 and 154) and were examined with self-reported measures of depression (Beck Depression Inventory-II; BDI), social cognition, and everyday functioning and performed a total of 14 different social cognition performance-based tests. Some of these tests measured attribution bias (AIHQ), while others measured interpersonal sensitivity (PADS, PID5) while others were performance based tests of emotion recognition and perception as well as social inference and theory of mind. Participants were also examined for their speed of completion of the tasks and their confidence in their accuracy. Patients were also clinically rated with the PANSS.
**Results:** In both samples, SCZ patients were more depressed than HC (15,15, vs. 6 and 6). In both samples of SCZ, BDI scores were correlated with clinical ratings of depression (PANSS item 6: r's=.60 and .61. Performance on tests of emotion recognition and perception, social inference, and theory of mind were not correlated with BDI in either sample. In both samples, higher BDI were correlated with self-reports of more impaired everyday functioning, lower subjective impressions of social cognitive competence, and greater feelings of interpersonal sensitivity, combined with the impression that others were mistreating them. Depression in HC, but not patients, was associated with lower confidence while performing social cognitive tests and depression in SCZ, but not HC, was associated with slower performance on these same tests.
**Discussion:** Depressed mood impacts self assessment of abilities and global world views in very similar ways in HC and people with SCZ. These impressions are not due to objective impairments in performance that are associated with depression. In contrast, objective performance on social cognitive tests, like previous studies of the relationship of neurocognition and functional and depression, shows remarkably little overlap with subjective depression. Although the similarity of the relationships between depression, interpersonal sensitivity, and subjective quality of life are similar in HC and SCZ, the more severe depression on the part of the SCZ populations suggests that this is an area of considerable importance for clinical intervention with either pharmacological or psychotherapeutic interventions.

### F251. REVISITING THE RELATIONSHIP BETWEEN NEUROCOGNITION AND SUBJECTIVE QUALITY OF LIFE IN SCHIZOPHRENIA

Eric Tan*[,1], Stuart Lee[2], Susan Rossell[3]
[1]*Swinburne University;* [2]*Monash Alfred Psychiatry Research Centre, The Alfred Hospital and Monash University, Central Clinical School;* [3]*Monash Alfred Psychiatry Research Centre, The Alfred Hospital and Monash University, Central Clinical School, Swinburne University of Technology, The University of Melbourne, St. Vincent's Hospital*

**Background:** Neurocognitive impairments are a major feature of schizophrenia and present long-term challenges to the quality of life (QOL) of patients. Their contribution to a patient's life satisfaction (subjective QOL; sQOL) has been much investigated, however, results have been equivocal and often non-significant. This contrasts with relatively more evidence for the neurocognition-objective QOL (oQOL) relationship. Previous work has also not investigated any differences in the subjective QOL associations between lower-order (e.g. processing speed, attention) and higher order (e.g. executive function) cognitive abilities. This study sought to better characterise the neurocognition-sQOL relationship through 3 separate analyses in clinical and healthy control samples: 1) examining correlational relationships between oQOL and sQOL and both lower-order and executive cognitive skills; 2) examining if lower-order or executive cognitive skills moderate the relationship between oQOL and sQOL; and 3) examining if the relationship between sQOL and both lower-order and executive cognitive skills differs between groups.
**Methods:** Data from 57 schizophrenia/schizoaffective disorder patients (age: M=43.40, SD=10.85) and 48 healthy controls (age: M=39.82, SD=13.89) was analysed. QOL was assessed using the Lehman's Quality of Life Interview. Lower-order cognitive skills were assessed using 9 tasks: Trail Making Test-A, symbol coding, animal fluency, spatial span, letter-number span, continuous performance test, Hopkins verbal learning test, brief visuospatial memory test and digit span. Executive function was measured via: Mazes, MSCEIT-ME, DKEFS Colour-Word Interference Test (Inhibition and Switching) and letter fluency. All task scores were converted to z-scores and composites were calculated to represent lower-order cognition and executive function.
**Results:** In line with the literature, the results revealed significant correlations between oQOL and sQOL but no associations between sQOL and either cognition measure in both groups (Analysis 1). In Analysis 2, neither lower-order nor executive cognitive skills moderated the relationship between oQOL and sQOL in either patients or controls. In Analysis 3, group membership moderated the relationship between executive function and sQOL ($p=.037$), with a positive relationship for controls but negative relationship for patients. Group did not moderate the relationship between lower-order cognition and sQOL ($p=.16$).
**Discussion:** The relationship between cognition and sQOL appears to be more related to higher-order abilities relating to idea generation, inhibition and reasoning than lower-level functions. Additionally, directional group differences in this relationship may reflect better executive functions leading to lower sQOL assessment in patients and thus lower ratings compared to controls who predictably rate higher.

### F252. SERVICE PROVISION FOR ULTRA-HIGH RISK FOR PSYCHOSIS: IMPLEMENTATION OF CLINICAL GUIDELINES IN ENGLAND

Helen Stain*[,1], Lauren Mawn[2], Stephanie Common[3], Marie Pilton[4], Andrew Thompson[5]
[1]*Leeds Trinity University;* [2]*School of Psychology, Newcastle University;* [3]*Tees Esk Wear Valleys NHS Foundation Trust;* [4]*Newcastle Upon Tyne Hospitals NHS Foundation Trust;* [5]*Warwick Medical School, University of Warwick*

**Background:** Evidence from meta-analyses of randomised clinical trials show interventions for young people at ultra high risk (UHR) of developing psychosis are effective both clinically and economically. While research evidence has begun to be integrated into clinical guidelines, there is a lack of research on the implementation of these guidelines. This paper examines service provision for UHR individuals in accordance with current clinical guidelines within the National Health Service (NHS) in England.
**Methods:** A self-report online survey was completed by clinical leaders of Early Intervention in Psychosis (EIP) teams (N=50) within the NHS across the UK.
**Results:** Of the 50 EIP teams responding (from 30 NHS Trusts), 53% reported inclusion of the UHR group in their service mandate, with age range predominantly 14–35 years (81%) and service provided for at least 12 months (53%). Provision of services according to NICE clinical guidelines showed 50% of services offered cognitive behavioural therapy (CBT) for psychosis, and 42% offered family intervention. Contrary to guidelines, 50% of services offered antipsychotic medication. Around half of services provided training in assessment by CAARMS, psychoeducation, CBT for psychosis, family work and treatment for anxiety and depression.
**Discussion:** Despite clear evidence for the benefit of early intervention in this population, current provision for UHR within EIP services in England does not match clinical guidelines. While some argue this is due to a lack of allocated funding, it is important to note the similar variable adherence to clinical guidelines in the treatment of people with established schizophrenia.

### F253. A FIDELITY TOOL FOR THE AUSTRALIAN EARLY PSYCHOSIS SYSTEM

Eoin Killackey*[,1], Kristi van der EL[2], Heather Stavely[1]
[1]*Orygen, The National Centre of Excellence in Youth Mental Health;* [2]*Orygen, The National Centre of Excellence in Youth Mental Health, The University of Melbourne*

**Background:** In the 2010 Australian Federal Budget funding was committed to establish an early psychosis service system based on the EPPIC model of early intervention for psychosis that was developed in Melbourne, Australia. This system was established by 2016. As part of the development of the system a fidelity scale was developed to measure the adherence to the model of the sites delivering the early psychosis services.
**Methods:** The EPPIC Model Integrity Tool (EMIT) was developed as part of the national reforms around early psychosis. The EMIT is an 80 item assessment tool that maps onto the 16 core components of the EPPIC model. The tool is used by attending the sites and speaking with staff and young people as well as accessing documents, policies and high level data around client flow through the service. The first two rounds of fidelity assessment were conducted in July/August and October/November 2017
**Results:** Results of the first two rounds of fidelity testing demonstrate a level of fidelity to the model consistent with expectations of each site in relation to their phase of establishment. Data show that there are some components of the EPPIC model that are better implemented than others. These data will give an initial snapshot of the adherence of the sites to the EPPIC model. Also presented will be the means by which reporting back from the assessments to the sites will facilitate closer adherence to the model.
**Discussion:** Model fidelity is an increasingly recognised way to ensure that programs based on evidence continue to deliver high quality outcomes, and avoid drifting away from the model. This presentation will demonstrate the outcomes from the first two rounds of application of the EMIT and ways in which fidelity testing can help services to improve their support of young people with early psychosis.

### F254. EXPANDING THE REACH OF NAVIGATE CSC PROGRAMS ACROSS THE U.S.: WHAT DO WE KNOW?

Piper Meyer-Kalos*[,1], Shaunequa James[1], Jillian Wright-Martin[1], Susan Gingerich[2], Shirley Glynn[3]
[1]*The University of Minnesota;* [2]*Independent Consultant;* [3]*UCLA*

**Background:** The Recovery After Initial Schizophrenia Episode-Early Treatment Program (RAISE-ETP) study was a landmark investigation whose positive results led to increased funding and support to build first episode psychosis programs across the US. Every state in the country received dedicated funding to implement a coordinated specialty care (CSC) program designed to identify and treat persons with first episode psychosis within the context of the nation's multi-payer health system. Since the funding began in 2014, numerous CSC programs have been developed but little is known about which models of treatment providers are implementing and the success of these programs. The research here presents data from a survey focusing on providing feedback from the first episode psychosis programs in the US implementing NAVIGATE, the CSC program utilized in RAISE-ETP. The survey targets the program directors in the NAVIGATE programs; the aims of the survey include 1) to describe the program characteristics of NAVIGATE teams in the US and 2) to better understand how NAVIGATE programs are identifying and enrolling people into their services. Capturing local data on CSC team composition and case identification strategies is particularly critical in multi-payer systems lacking guidance and oversight from a national health system.
**Methods:** An online survey is being conducted to assess the implementation of NAVIGATE programs in the US and evaluate the procedures that the program director utilizes to identify and enroll NAVIGATE participants in services. Program directors from NAVIGATE programs are being identified and contacted to participate by national trainers to join a national database of first episode programs. Program data collected includes information about the location of the program, staff in the different NAVIGATE team roles (prescriber, individual clinician, family clinician, and employment/education specialists, as well as optional roles such as peer advocate and case manager), program enrollment criteria, number of participants screened and enrolled, and rates of planned and unplanned discharge. In addition, program directors are asked questions to report community based strategies to identify participants and screening procedures to enroll participants. Data analysis will focus on presenting the demographic and clinical characteristics of the programs. Common themes will be ascertained, including barriers and facilitators to identifying and enrolling participants with first episode psychosis. Helpful recommendations provided by the project directors on identifying and screening participants will be synthesized and reported.
**Results:** There are approximately 30 NAVIGATE programs in 14 states in the US. Results will highlight the dissemination of NAVIGATE in the US and implementation of these programs across a wide range of different communities. We will describe the dissemination of NAVIGATE across the US and similarities and differences across NAVIGATE programs. Results also will provide feedback on the challenges and helpful strategies that program directors have used to engage people in treatment.
**Discussion:** The findings from this survey will be the first to provide an overview of the implementation of the NAVIGATE program in the US. The results will provide an overview of the dissemination of the NAVIGATE program, the only CSC program evaluated in a national US trial. Recommendations could help inform the ongoing development and dissemination of coordinated specialty care programs.

### F255. FACTORS RESPONSIBLE FOR DELAY IN TREATMENT SEEKING IN PATIENTS WITH PSYCHOSIS- A QUALITATIVE STUDY FROM CENTRAL INDIA

Mamidipalli Spoorthy*[,1]
[1]*All India Institute of Medical Sciences, Raipur*

**Background:** Delay in treatment seeking in psychoses is not only influenced by stigma, societal attitudes, unawareness, under-diagnosis but also is coloured by the socio-cultural background of the patient. Finding out these reasons for delay in treatment can help both the patient and the family members by reducing the morbidity & burden associated with untreated psychosis.
**Methods:** This is a hospital based cross-sectional study, conducted Raipur. Included are purposive sample of 25 family members & patients with a diagnosis of schizophrenia, schizoaffective disorder, or psychotic disorder-not otherwise specified - using the Mini International Neuropsychiatric Interview-Plus version, aged 18–60 years, who are able to understand and speak Hindi, and in regular contact with the patient. DUP is defined as the number of months from the onset of positive psychotic symptoms until start of proper treatment. Semi-structured interview was conducted by using open ended questions to assess the factors responsible for treatment delay and verbatims were recorded.
Qualitative analysis
We used content analysis for the purpose of this study. Each investigator generated separate categories and themes after reading the transcripts word by word. Theme generation was continued till theoretical saturation emerged and. Categories and themes identified by both the investigators in common were used in the results as it would increase their validity.
**Results:** 1. Socio-demographic profile
64% of patients were diagnosed with Schizophrenia and the rest were diagnosed with Psychosis NOS. Mean total duration of untreated psychosis was 15 months. Relation of family members with the patient was like parents (48%), spouse (24%), siblings (12%), children (8%), uncle/aunt (4%), grand-parents (4%).
2. Results of qualitative analysis
Based upon the content analysis technique used, we have generated certain categories of factors responsible for treatment delay and generated themes in each category.

A. Illness related factors
  a. Unawareness of illness
  b. Explanatory models of illness
    i. Supernatural causation of illness
    ii. Biological causation of illness
  c. Stigma associated with illness
B. Patient related factors
  a. Underlying pre-morbid personality
  b. Symptoms at the onset
  c. Onset along with life events
  d. Poor insight/uncooperative patient
  e. Impaired functioning
C. Family related factors
  a. Shared societal beliefs
  b. Cultural constraints
  c. Lack of support from significant others or poor social support
D. Treatment related factors
  a. Poor knowledge of general physicians about psychiatric disorders and poor referral
  b. Misconceptions about the effects of medication
E. Others
  a. Financial constraints

**Discussion:** The most common cause of delay is unawareness about the illness apart from the supernatural causation. To our knowledge this is the first study where we found that if the patient's personality presents in an exaggerated way, or patient's psychopathology is in line with the socio-cultural background, it might lead to delay. Though the findings about patient's poor insight, uncooperativeness, negative symptoms, absence of violence, financial burden, stigma, lack of social support was proved by many studies, preserved functioning is our novel finding.
Though these themes seem to be separate, they are interdependent and interact in a complex way leading to the delay in treatment seeking. Interventions focused at each and every step need to be devised in further studies in order to overcome these barriers.

### F256. CAN WE REDUCE THE DURATION OF UNTREATED PSYCHOSIS?

Dominic Oliver*[,1], Cathy Davies[1], Georgia Crossland[1], Steffiany Lim[1], George Gifford[1], Philip McGuire[1], Paolo Fusar-Poli[1]
[1]*Institute of Psychiatry Psychology and Neuroscience, King's College London*

**Background:** Reduction of duration of untreated psychosis (DUP) is the key strategy of early interventions for improving the outcomes of first episode psychosis. Although several controlled interventional studies have been conducted with the aim of reducing DUP, the results are highly inconsistent and conflicting.
**Methods:** The current study systematically searched Web of Science and Ovid for English original articles investigating interventions adopted to reduce DUP, compared to a control intervention, up to 6th April 2017. 16 controlled interventional studies were retrieved, including 1964 patients in the intervention arm and 1358 in the control arm. The controlled interventions studies were characterised by: standalone first episode psychosis services, standalone clinical high risk services, community interventions, healthcare professional training and multifocus interventions. Random effects meta-analyses were conducted.
**Results:** There was no summary evidence that available interventions are successful in reducing DUP during the first episode of psychosis (Hedges' g = -0.12, 95%CIs -0.25 to 0.01). Subgroup analyses showed no differences within each subgroup, with the exception of clinical high risk services (Hedges' g = -0.386, 95%CI -0.726 to -0.045). . There was substantial heterogeneity (I2 = 66.4%), most of which was accounted by different definitions of DUP onset (R2=0.88).
**Discussion:** These negative findings may reflect a parcelled research base in the area, lack of prospective randomized controlled trials (only two randomised cluster designed studies were present) and small sample sizes. Psychometric standardisation of DUP definition, improvement of study design and implementation of preventative strategies seem the most promising avenues for reducing DUP and improving outcomes of first-episode psychosis.

### Poster Session III

### S1. THE ASSOCIATION BETWEEN WAR-RELATED STRESS, PTSD SYMPTOMS, AND SUB-CLINICAL PSYCHOSIS: A CROSS-CULTURAL POPULATION-BASED STUDY AMONG PALESTINIAN AND ISRAELI YOUNG ADULTS

Amjad Mussa[1], Meirav Blizovski[1], Dan Koren*,[1]
[1]*University of Haifa*

**Background:** Sub-clinical or attenuated psychosis symptoms (APS) in the general population has become a focus of considerable research interest over the past two decades, as they appear to index an increased risk for psychotic outcomes. Recent data from several community-based studies around the world provide convincing support for an association of APS with traumatic stress that is likely moderated by familial genetic risk and gender. However, relatively little is known about the degree to which APS are associated with terror/war-related stress. Moreover, relatively little is known about the degree to which cultural/religious factors moderate this association. Hence, the overarching goal of this study was to address this lacuna in the literature by examining the relationship between exposure to terror/war-related events, PTSD symptoms and familial genetic risk among Palestinian and Israeli youth.
**Methods:** Exposure to terror/war-related trauma, presence and severity of PTSD symptoms, perceived ability to cope with trauma, familial genetic-risk, and APS were assessed in a representative sample of 530 Israeli and 1100 Palestinian (451 from Israel, 264 from the West Bank, and 385 from the Gaza Strip) young adults with a mean age of 36.7 (SD=8.4). PTSD symptoms were assessed with the Post-traumatic Disorder Scale (PDS), perceived ability to cope with trauma with the Perceived Ability To Cope With Trauma Scale (PACT), and APS with the Community Assessments of Psychic Experiences (CAPE).
**Results:** As hypothesized, there was a significant three-way interaction effect of exposure to terror-war-related trauma, religion, and familial genetic-risk on APS. The highest level of APS was among Palestinians who live in the Gaza strip, with no significant differences between Jews and Palestinians who live in Israel or in the West Bank. Also, consistent with our hypotheses, the three-way association between exposure to trauma, familial genetic risk and religion was mediated by PTSD symptoms and perceived ability to cope with trauma.
**Discussion:** These findings provide further support for the link between exposure to trauma, familial genetic-risk, and APS. Also, it provides further support for the mediating role that PTSD symptoms play in this link. Finally, it suggests that religious background moderates the link between exposure to trauma and APS.

### S2. CHILDHOOD TRAUMA IS ASSOCIATED WITH SEVERITY OF AT-RISK MENTAL STATE AND PSYCHOSIS IN UHR INDIVIDUALS AND PATIENTS WITH SCHIZOPHRENIA

Attilio Rapisarda*,[1], Oon Him Peh[1], Kang Sim[1], Jimmy Lee[1]
[1]*Institute of Mental Health*

**Background:** Experiences of abuse during childhood are highly prevalent in psychiatric populations and are associated with a higher risk of developing psychosis during adolescence and young adulthood. This study explored the association between childhood trauma, psychotic symptoms and comorbid symptomatology in Ultra-High Risk individuals and schizophrenia patients.
**Methods:** 59 patients diagnosed with schizophrenia (mean age= 33), 164 individuals at Ultra-High Risk for psychosis (UHR) (mean age= 22) and 60 healthy individuals (mean age= 28) were recruited from the clinic and the community and assessed using the Structured Clinical Interview for DSM-IV (SCID), Comprehensive Assessment of At-Risk Mental State (CAARMS), Global Assessment of Functioning (GAF) and Childhood Trauma Questionnaire. Patients and UHR were also assessed on the Positive and Negative Syndrome Scale (PANSS) and Beck Anxiety Inventory (BAI). A Kruskal-Wallis one-way analysis was performed to detect significant differences in the severity of reported trauma across groups. Multiple regression analyses were computed on Blom-ranked, normalized scores to measure the association between reported trauma and symptomatology in the UHR participants and patients.
**Results:** Significant differences in severity of reported childhood traumatic experiences were found across the three groups for emotional abuse (p= .000), emotional neglect (p= .000), physical neglect (p= .000), physical abuse (p= .001) and sexual abuse (p= .007). The severity of traumatic experiences reported by patients and UHR individuals was consistently higher compared with controls, with effect sizes ranging from a minimum of Cohen's d= 0.54 (sexual abuse) to a maximum of Cohen's d= 0.97 (emotional abuse).
In UHR, higher CTQ total scores, reflecting more severe overall abuse, were associated with more severe prodromal psychotic symptoms ($\beta$= .259, p= .001). Also, more severe anxiety symptoms were associated with more severe emotional ($\beta$= .365, p= .000), physical ($\beta$= .283, p= .000), and sexual abuse ($\beta$= .214, p= .006).
In UHR and patients, higher PANSS negative sub-scale scores were associated with higher levels of emotional ($\beta$= .196, p= .012) and physical neglect ($\beta$= .175, p= .026).
**Discussion:** Our findings support previous evidence on the higher prevalence of different types of abuse experienced during childhood by schizophrenia patients and individuals at risk for psychosis. Among the different types of abuse investigated, severe emotional abuse is most strongly associated with severe prodromal symptomatology and anxiety experienced during young adulthood by UHR individuals.

### S3. CHILDHOOD TRAUMA AND COGNITIVE FUNCTIONING IN SCHIZOPHRENIA SPECTRUM DISORDERS: EFFECT OF FREQUENCY AND TYPE OF CHILDHOOD TRAUMA

Nina Morkved*,[1], Erik Johnsen[2], Rune Kroken[2], Rolf Gjestad[3], Dagfinn Winje[4], Jens Thimm[5], Farivar Fathian[6], Else-Marie Løberg[2]
[1]*Mosjoen District Psychiatric Centre, Helgeland Hospital;* [2]*Haukeland University Hospital, University of Bergen;* [3]*Haukeland University Hospital;* [4]*University of Bergen;* [5]*University of Tromsø;* [6]*NKS Olaviken Gerontopsychiatric Hospital*

**Background:** Cognitive impairment is a core feature of schizophrenia spectrum disorders (SSDs). Exposure to childhood trauma (CT), defined as physical, sexual and emotional abuse, and physical and emotional neglect, has been associated with SSDs across study designs and populations. Possibly, there is a relationship between exposure to CT and cognitive impairment in individuals with SSDs. Research has shown that a history of CT may be related to decline in cognitive performance in the general population, as well as in SSDs, whereas other studies have failed to find evidence for an association between CT and cognitive impairments in patients with SSDs. Findings on the relation between CT and cognitive impairment in individuals with SSDs is not conclusive, and a minority of the studies to date have examined the effects of frequency and severity of CT subtypes in SSDs and the relation to cognitive abilities. We hypothesize that there will be a negative relationship between the frequency and severity of CT and cognitive functioning, possibly in a dose dependent matter. CT subtypes may influence this relationship.

**Methods:** The present study is part of the Bergen Psychosis project 2 (BP2), Haukeland University Hospital, Norway. Patients were recruited at the Medical University in Innsbruck, Innsbruck, Austria; Stavanger University Hospital, Stavanger, Norway; and Haukeland University Hospital, Bergen, Norway, and gave informed consent to participate. To be included, patients had to meet ICD-10 criteria for SSDs (F20-F29), be > 16 years of age, and score ≥ 4 on at least one of the psychosis items on the Positive and Negative Syndrome Scale (PANSS). Childhood trauma (physical, emotional, sexual abuse, and physical, emotional neglect) was measured by the Childhood Trauma Questionnaire Short-Form (CTQ-SF). Cognitive functioning was examined by means of a comprehensive neuropsychological test battery. The following cognitive domains were assessed: verbal and visuospatial abilities, learning, memory, attention and working memory, executive functioning, and processing speed. The assessments were completed within three months of inclusion to the study.
**Results:** The relationship between the frequency and severity of CT and cognition will be examined, in addition to the possible influence of CT subtypes. Preliminary findings will be reported.
**Discussion:** The clinical implications of our findings will be discussed.

### S4. ASYMMETRIC DRUG-INDUCED PARKINSONISM IS RELATED TO PSYCHOPATHOLOGY

Lydia Pieters*[,1], P. Roberto Bakker[2], Peter N. Van Harten[2]
[1]*Psychiatric Center GGz Centraal;* [2]*Maastricht University, Psychiatric Centre GGz Centraal*

**Background:** Drug-Induced Parkinsonism (DIP) is the most common movement disorder induced by antipsychotics. The prevalence of DIP in chronic psychiatric populations ranges between 17 and 72% (1–3). Although, DIP is mostly symmetric, asymmetric DIP is reported in 18 to 54% of the patients. (4). There are no studies to the clinical relevance of asymmetric DIP. We investigated the prevalence of motor asymmetry in DIP and its relationship to the severity of psychopathology in a prospective study.
**Methods:** In a cohort study of 207 long-stay psychiatric inpatients the prevalence of DIP was assessed at least two times (mean follow-up 1.1 year) in each patient (5). DIP was assessed with the Unified Parkinson Disease Rating Scale (UPDRS) and the prevalence of persistent DIP was 56.2%. Patients with at least one time parkinsonism in the upper/lower limb(s) were included for analyses. Asymmetry of parkinsonism was calculated with the symmetry index (Figure 1). A cut-off value of ≥ 0,20 was used for the definition of asymmetric DIP. Multilevel mixed models were built to explore the relationship between asymmetry in DIP and the severity of psychopathology, measured on the Clinical Global Impression-Schizophrenia scale severity index (CGI-SCH SI).
**Results:** In a cohort study of 207 long-stay psychiatric inpatients the prevalence of DIP was assessed at least two times (mean follow-up 1.1 year) in each patient (5). DIP was assessed with the Unified Parkinson Disease Rating Scale (UPDRS) and the prevalence of persistent DIP was 56.2%. Patients with at least one time parkinsonism in the upper/lower limb(s) were included for analyses. Asymmetry of parkinsonism was calculated with the symmetry index (Figure 1). A cut-off value of ≥ 0,20 was used for the definition of asymmetric DIP. Multilevel mixed models were built to explore the relationship between asymmetry in DIP and the severity of psychopathology, measured on the Clinical Global Impression-Schizophrenia scale severity index (CGI-SCH SI).
**Discussion:** DIP is asymmetric in 1 of 5 patients. Therefore, the clinical rule that Parkinson's disease always starts asymmetrically and such may be helpful to differentiate between Parkinson's disease and DIP is not valid. Asymmetric presentation of DIP is of clinical relevance as it is related to the severity of psychopathology. Asymmetric DIP may alert the clinician of more severe psychopathology. Replication is indicated to examine the robustness of the relationship.

**References:**
1. Modestin J, Wehrli MV, Stephan PL, Agarwalla P. Evolution of neuroleptic-induced extrapyramidal syndromes under long-term neuroleptic treatment. Schizophr Res. 2008; 100(1–3): 97–107.
2. Janno S, Holi M, Tuisku K, Wahlbeck K. Prevalence of Neuroleptic-Induced Movement Disorders in Chronic Schizophrenia Inpatients. Am J Psychiatry. 2004; 161(1): 160–163.
3. Harten PN Van, Matroos GE, Hoek HW. The prevalence of tardive dystonia, tardive dyskinesia, parkinsonism and akathisia. The Curaçao Extrapyramidal Syndromes Study I. Schizophr Res. 1996; 19(2): 195–203.
4. Shin H, Chung J. Drug-Induced Parkinsonism. J Clin Neurol. 2012; 8: 15–21.
5. Bakker PR, de Groot IW, van Os J, van Harten PN. Long-Stay Psychiatric Patients: A Prospective Study Revealing Persistent Antipsychotic-Induced Movement Disorder. PLoS One. 2011; 6(10): 1–6.

### S5. EFFECTS OF EARLY LIFE ADVERSITY ON IMMUNE FUNCTION AND COGNITIVE PERFORMANCE IN YOUTHS WITH AND WITHOUT EXPERIENCE OF PSYCHOTIC SYMPTOMS

Jessica Holland*[,1], Maria Dauvermann[1], Derek Morris[1], Stan Zammit[2], Golam Khandaker[3], Gary Donohoe[1]
[1]*National University of Ireland, Galway;* [2]*University of Bristol;* [3]*Cambridge University*

**Background:** Early life adversity (ELA), including physical abuse or neglect and emotional abuse or neglect, is a significant risk factor for schizophrenia. Changes in cognitive function, and in particular social cognition, are also associated with this disorder. In psychosis, ELA and cognitive deficits have, separately, been associated with an increased immune response. In this study we sought to determine whether ELA's might affect cognitive performance and if so, whether these affects were mediated via an impact on immune response.
**Methods:** We investigated the relationship between ELA, immune response and cognition in the Avon Longitudinal study of parents and children (ALSPAC; n~5,000). ELA was defined in terms of the experience of physical abuse or neglect, emotional abuse or neglect, witnessing domestic violence, and harsh parenting before the age of 5 years. Social cognition was defined in terms of performance on theory of mind while general cognitive ability was defined in terms of IQ. Immune function was measured using C-reactive protein and Interleukin-6. Analysis was run both for the full sample and for individuals presenting with a history of psychotic symptoms at age 12.
**Results:** Early life adversity was associated with poorer performance on a range of both general and social cognitive measures. Increased immune activation was associated with cognitive performance, but was not observed to mediate the effects of ELA on cognition. Comparable findings were observed in children presenting with and without psychotic symptoms.
**Discussion:** While increased immune response has been associated with both early life adversity and cognitive impairment, this response was not observed to mediate the relationship between these two variables. Alternative hypothesis for the mechanism by which ELA may result in poorer cognitive performance, including attachment related effects, will be discussed.

### S6. EARLY LIFE ADVERSITIES AND SOCIAL COGNITIVE DYSFUNCTION IN SCHIZOPHRENIA AND OTHER MAJOR PSYCHIATRIC DISORDERS: A SYSTEMATIC REVIEW

Karolina Rokita*[,1], Maria M. Dauvermann[2], Gary Donohoe[2]
[1]*Cognitive Genetics and Cognitive Therapy Group, National University of Ireland, Galway;* [2]*National University of Ireland, Galway*

**Background:** Early life adversity has been identified as a potentially causal factor in the development of mental disorders. Little is known, however, about the association between various types of early life adversities and social cognitive function in adults with major psychiatric disorders, such as schizophrenia, borderline personality disorder, bipolar disorder and major depressive disorder. We conducted a systematic review aimed at elucidating possible underlying cognitive mechanisms that may form the pathway between early life adversities and social cognitive dysfunction.
**Methods:** Relevant studies were identified via electronic and manual searches of the literature, and included peer reviewed English language articles published up to May 2017. Quality of individual articles was assessed using the quality evaluation scale.
**Results:** A total of 15 studies were included in the systematic review with the quality assessment scores ranging from 2 to 5 (out of 6). The majority of the studies demonstrated that various types of early life adversities, specifically physical neglect, emotional and sexual abuse and insecure attachment, are significantly associated with social cognitive function.
**Discussion:** Presented in the context of an attachment model, we conclude that childhood adversity results in poor internal working models, selective attention towards emotional stimuli and greater difficulties with emotional self-regulation. The importance of these findings for development of interventions which diminish the adverse effects of childhood maltreatment on social cognition is discussed.

### S7. NEOSENSITIZATION TO MULTIPLE DRUGS FOLLOWING DRUG REACTION WITH EOSINOPHILIA AND SYSTEMIC SYMPTOMS SYNDROME (DRESS)

Moon Doo Kim*[,1], Won-Myong Bahk[2], Young Joon Kwon[3], Bo-Hyun Yoon[4], Kwanghun Lee[5], Beomwoo Nam[6], Eunsung Lim[7], Min-Kyu Song[8]
*[1]School of Medicine, Jeju National University; [2]Yeouido St. Mary's Hospital, The Catholic University of Korea; [3]College of Medicine, Soonchunhyang University; [4]Naju National Hospital; [5]College of Medicine, Dongguk University; [6]School of Medicine, Konkuk University; [7]Shinsegae Hospital; [8]Yesan Mental Health Clinic*

**Background:** Drug Reaction with Eosinophilia and Systemic Symptoms (DRESS) syndrome is associated with severe skin eruptions, fever, hematological abnormalities, and multi-organ involvement. Although aromatic anticonvulsant drugs have been frequently associated with the manifestation of DRESS syndrome, its induction following treatment with non-aromatic anticonvulsants, such as valproate, has rarely been reported. Moreover, there are limited data regarding the development of neosensitization related to chemically unrelated drugs following an episode of DRESS syndrome.
**Methods:** Here, a case of neosensitization to multiple drugs is described. The present case report describes a female patient who experienced neosensitization to amoxicillin, olanzapine, and quetiapine following the manifestation of DRESS syndrome induced by valproate.
**Results:** A 50-year-old woman with a 15-year history of schizophrenia was being treated with lithium (1200 mg) and quetiapine (600 mg) about 1 month, but due to high lithium serum concentrations, the lithium was changed to valproate (600 mg). Seven days later, the patient developed a whole-body skin rash, facial edema, and hyperthermia. Laboratory tests revealed an abnormal white cell count (25.2 × 103 /μL with 6% eosinophils) and aspartate transaminase (AST) and alanine transaminase (ALT) concentrations of 2729 IU/L and 2749IU/L, respectively. At that time, the patient had no any other medical history including drug allergy. A diagnosis of DRESS syndrome due to valproate treatment was established by a consulting dermatologist. As a result, all medicines were discontinued because of severe hepatitis, and intravenous methylprednisolone (60 mg per day) was administered for 1 week. The skin rash, fever, and liver dysfunction progressively disappeared. After discharge, the patient was treated with quetiapine (200 mg). However, she became lost to follow up after 6 months. Approximately 3 years later, the patient was admitted to a local hospital for psychotic symptoms aggravation because she was not taken antipsychotics for 3 years. She treated with lithium (900 mg), sulpiride (600 mg), risperidone (2 mg), and quetiapine (100 mg) for 2 weeks. Additionally, the patient initiated treatment with amoxicillin for acute tonsillitis. On the first day of amoxicillin intake, she developed fever, diffuse erythematous macules on her trunk, and facial edema, and she was transferred to a general hospital via the emergency department. To control her psychotic symptoms she is prescribed olanzapine, haloperidol and quetiapine step by step but all these medications develop fever, skin rash and abnormal AST/ALT. Finally she was given amisulpiride which had not been previously prescribed. Within 2 months, the patient's psychotic symptoms had gradually decreased and ultimately remitted.
**Discussion:** To our knowledge, this is the first case report of neosensitization to multiple drugs after valproate-induced DRESS syndrome. A thorough search of Pubmed was performed to identify similar cases, which confirmed that no cases of hypersensitivity to amoxicillin or neosensitization to multiple drugs after a valproate-related DRESS episode have been reported. Furthermore, only two studies have reported possible neosensitization to amoxicillin following DRESS episodes induced by carbamazepine, and only one case reported neosensitization to amoxicillin following a DRESS episode induced by allopurinol.

### S8. RELATIONSHIP BETWEEN ALLOSTATIC LOAD AND POOR FUNCTIONAL CAPACITY IN YOUTH AT ULTRA-HIGH RISK FOR PSYCHOSIS

Maximus Berger[1], Suzie Lavoie[2], Patrick D. McGorry[3], Barnaby Nelson[3], Connie Markulev[3], Hok Pan Yuen[2], Miriam Schaefer[3], Zoltan Sarnyai[1], G. Paul Amminger*[,3]
*[1]James Cook University; [2]Orygen, The National Centre of Excellence in Youth Mental Health, University of Melbourne; [3]Orygen, The National Centre of Excellence in Youth Mental Health*

**Background:** Current pathophysiological models of psychotic disorders suggest that stress contributes to the aetiology and trajectory of the disorder. Allostatic load (AL), a multisystem index of immune, neuroendocrine and metabolic dysregulation, is thought to represent the cumulative biological impact of stress. Two recent studies suggest that AL is elevated in patients with first-episode psychosis and related to psychotic symptoms and poor social and occupational functioning. Here, we investigate the relationship between AL and clinical outcomes in individuals at ultra-high risk for psychosis.
**Methods:** AL was measured in a sub-group of participants of the NEURAPRO study, a multicentre randomized-controlled trial of omega-3 polyunsaturated fatty acids versus placebo in people aged 13 - 40 at UHR for psychosis. A total of 106 participants who underwent additional biomarker analysis were included in the present study. Biomarkers for the AL index were selected based on (1) representation of several physiological systems including the cardiovascular, neuroendocrine, immune, and metabolic systems, (2) use in previous AL research, and (3) associations with disease risk. We adopted a scaled AL algorithm whereby each marker proportionally contributes to the overall AL index. Clinical outcomes were assessed 3 and 12 months after study intake using the Social and Occupational Functioning Assessment Scale (SOFAS), the Brief Psychiatric Rating Scale (BPRS), the Scale for the Assessment of Negative Symptoms (SANS), the Montgomery-Asberg Depression Rating Scale (MADRS) and the Young Mania Rating Scale (YMRS). We hypothesised that AL would be (1) associated with higher symptoms scores and reduced functioning at baseline and (2) related to more severe symptoms and reduced functioning at the 3 and 12 month assessments. These hypotheses were tested by calculating Pearson correlation coefficients and by using linear regression modelling, respectively.

**Results:** No significant correlations of AL with any of the psychometric scales are observed at study intake. AL at baseline was associated with lower SOFAS scores at 3 months (B=-1.436, p=0.042) but not at 12 months (B=-1.096, p=0.297). No prospective associations of AL were found with any of the other psychometric measures (all p>0.05).
**Discussion:** Our data support the notion that multisystem dysregulation, indexed as AL, may be a potential predictor of early treatment response and warrants further investigation. These observations are consistent with recent research demonstrating elevated AL in patients with psychotic disorders that is related to reduced functioning.

### S9. RESTING EEG CHANGES IN SCHIZOPHRENIA

Leonhardt Unruh[1], Rick Adams*[,2], Alan Anticevic[3], L. Elliot Hong[4]
*[1]University of Göttingen; [2]University College London; [3]Yale University; [4]University of Maryland*

**Background:** Numerous previous studies have found increased power in low frequencies in resting EEG data in subjects with a diagnosis of schizophrenia (Scz). Low frequency power in δ is usually localised to frontal channels, whereas increases in θ power can be more widespread (Boutros et al., 2008). Given the low spatial resolution of EEG data, we used a spatial filter (the surface Laplacian) to investigate whether differences in resting EEG frequency band power in Scz are truly distributed throughout the cortex, or whether they localise to more focal areas.
**Methods:** 64 channel EEG data were recorded for 5 minutes in both eyes closed and eyes open conditions in 103 medicated Scz (M=71, F=32, mean(std) age=40(14.3) yrs) and 104 controls (M=64, F=30, mean(std) age=40(13.8) yrs). The data were epoched and epochs with artefacts were detected by their amplitude, variance or kurtosis and removed. The power spectral density at each channel was computed using Welch's method, and the surface Laplacian (using the spherical spline method of Perrin et al. (1987, 1989)) was applied to reduce volume conduction effects and improve topographical localization. Power was analysed separately for five frequency bands: δ (2–4 Hz), θ (4–8 Hz), α (8–12 Hz), β (15–30 Hz) and γ (30–50 Hz). Permutation testing (20 runs of 1000 group label permutations) was performed with correction for multiple comparisons based on clusters of channels of significantly different (p<0.05 uncorrected) power.
**Results:** Scz and controls showed group differences in θ power (mean(std) over all channels, eyes open condition: Scz=5.7(10)x104 μVmm-2 and controls=3.2(5.9)x104 μVmm-2, t test, t(181) = 2.0, p = 0.047; and at trend level significance in the eyes closed condition: Scz=6.0(9.6)x104 μVmm-2 and controls=3.8(6.2)x104 μVmm-2, t test, t(181) = 1.8, p = 0.067), but no group differences in the other four frequency bands (all p >0.05). Scz had greater θ power in both eyes open and eyes closed conditions using cluster-based permutation tests (both p<0.05). The median cluster sizes (25% quantile, 75% quantile) with elevated θ power in Scz were of 53 (51, 55) channels in the eyes open and 50.5 (48, 51.5) in the eyes closed condition.
**Discussion:** These initial results support previous findings of widespread increased θ band power in Scz (e.g. Narayanan et al., 2014). These θ differences appear to be manifest in the majority of the cortex, rather than being localised to one particular area.

### S10. ASTROGLIAL PATHOLOGY IN SCHIZOPHRENIA: A META-ANALYSIS OF MRS STUDIES OF ANTERIOR CINGULATE MYOINOSITOL

Avyarthana Dey[1], Priyadharshini Sabesan[2], Jean Theberge[3], Joaquim Radua[4], Lena Palaniyappan*[,1]
*[1]University of Western Ontario; [2]London Health Sciences Center; [3]St. Joseph's Health Care London; [4]IoPPN, King's College London*

**Background:** Several lines of evidence support a role for astroglial pathology in schizophrenia. 1H-MRS does not specifically differentiate between brain cell types; nevertheless, given that myo-inositol (mIns) is particularly abundant in astroglia rather than neuron and microglial cells, it can be considered an astroglial marker. mIns levels in the brain can decrease after brain injury with an efflux of mIns from astrocytes occurring as an osmoregulatory response. Many small sized studies have reported on mIns concentration in schizophrenia, but to date these have not been pooled to estimate a collective effect size. Examining the state of mIns deficit is a critical step to delineate the role of astroglial cells in schizophrenia. We conducted a meta-analysis to investigate the aberrations in myo-inositol levels in the ACC of patients with schizophrenia and measured using magnetic resonance spectroscopy (MRS).
**Methods:** Medline, Google Scholar, Ovid Online and EMBASE databases were searched for studies published until September 2017. Search terms included full forms and variations of magnetic resonance spectroscopy, MRS, schizophrenia, psychosis, myo-inositol, inositol, Ins, mI, mIns. We included all 1H-MRS studies reporting mIns values for patients satisfying DSM or ICD based criteria for a primary psychotic disorder (SCZ) in comparison to a healthy controls (HC) group. We screened all identified abstracts, filtered studies that did not satisfy inclusion criteria, hand-searched references and contacted experts to locate further studies. 9 studies were identified that included 223 patients in SCZ group and 231 HCs. We excluded studies that reported only on comorbid illnesses, did not compare patients and HCs, or failed to report data required to construct effect size metrics. A random-effects and fixed-effects, inverse-weighted variance model was used to calculate the pooled effect size. Mean values were extracted and verified independently and effect sizes were computed based on Excel Macro produced by Major Depressive Disorder Neuroimaging Database (MaND) investigators.
**Results:** Contrary to our expectations, in SCZ, there were no significant differences in ACC mIns in patients compared to HC (RFX=0.359, p= 0.057; 95% CI, -0.728 to 0.011; heterogeneity p = 0.0004). In the SCZ group, the mean effect size (Cohen's d) was d= 0.39, indicating a medium sized difference. There were several methodological issues in the reported studies. Notably, most studies reported on mIns spectrum only when seeking differences in other metabolites; voxel placements were not standardized across the published studies; majority of patients were medicated, in various stages of illness. There was no statistical evidence for a publication bias (p=0.8).
**Discussion:** There is a medium effect-size, albeit statistically insignificant, reduction in the concentration of mIns in the anterior cingulate cortex in patients with schizophrenia. Given that mIns is the most readily accessible cortical marker in vivo for astroglial activity, it may be feasible to use MRS to stratify patients with astroglial abnormality from those without such an abnormality in schizophrenia.

### S11. BRAIN ABNORMALITIES ASSOCIATED WITH DISEASE-SPECIFIC AND GENETIC RISK FACTORS FOR SCHIZOPHRENIA

Vina Goghari*[,1]
*[1]University of Toronto*

**Background:** Schizophrenia is a severe disorder affecting approximately 1% of the population. The disorder is associated with symptoms such as false perceptions and beliefs and disturbances in affect and language production. In 2004 the total direct healthcare and non-healthcare cost in Canada was estimated at $2 billion, with an additional productivity loss estimated at $5 billion.
**Methods:** I will present a program of research into disease-specific and genetic risk factors associated with structural and functional brain abnormalities, including morphology (amount of grey matter), structural connectivity (amount of white matter integrity), and brain functioning (amount of brain activity) in individuals with schizophrenia, their family members, and community controls using magnetic resonance imaging. As healthy relatives share genes with their affected family member, but do not share the disease process, abnormalities present in relatives are likely associated with the genes for schizophrenia.
**Results:** Evidence was found for disease-specific, genetic risk and compensatory brain mechanisms associated with schizophrenia that were complementary between the results from brain morphology, structural connectivity, and brain functioning.
**Discussion:** Isolating the biological and genetic basis of these deficits could ultimately aid in developing novel psychosocial and pharmacological treatments to facilitate improved day-to-day functioning in schizophrenia.

### S12. THE 'AUTOANTIBODYOME' IN PSYCHOSIS: A PILOT STUDY AND BLUEPRINT FOR BIOMARKER DISCOVERY

Thomas Pollak*,[1], Cassandra DeMarshall[2], Abhirup Sarkar[2], Timothy Nicholson[1], Philip McGuire[1], James Stone[1], Anthony David[1], Robin Murray[1], Robert Nagele[2]
*[1]Institute of Psychiatry, Psychology & Neuroscience, King's College London; [2]Rowan University School of Osteopathic Medicine*

**Background:** Recent studies seeking to describe the prevalence and significance of autoantibodies in psychotic disorders can be characterized as 'top-down' in theoretical approach; that is, autoantibodies to specific (usually CNS) antigens are sought based on a) the known function of the antigen (e.g. NMDAR) and its putative role in psychosis or b) the clinical observation that these autoantibodies can cause psychosis as part of a more complex neuropsychiatric presentation (e.g. autoimmune encephalitis). No candidate autoantibodies with a clear diagnostic or prognostic role have been definitively established.
We sought to take an alternative, 'bottom-up' approach to the significance of autoantibodies in psychosis that remains agnostic as to the potential significance of any one antigen. Every individual harbours autoantibodies directed against many thousands of self-antigens and the vast majority are not disease-causing. Indeed production of autoantibodies may represent a physiological, antigen-specific 'debris-clearing' response to tissue destruction or damage. It follows that the autoantibody profile of any individual reflects any pathological process that is ongoing in that individual and can thus serve as a 'readout' of the disease state in question.
**Methods:** Sera from 20 patients with a first episode of psychosis (FEP) (males: n=16; mean age: 29.35 s.d.: 7.07) from the Genetics and Psychosis (GAP) study and 20 matched controls were analysed, using Invitrogen's ProtoArray v5.1 Human Protein Microarrays, for the presence of IgG to 9486 unique human protein antigens which had been expressed as GST fusion proteins in insect cells, purified and spotted onto slides. Following application of a fluorescent secondary IgG, reactivity patterns were automatically read using a fluorescence scanner. Samples were split into testing and training sets, and the top 50 most differentially expressed and differentially depleted antibodies were then chosen as biomarkers.
**Results:** The top 50 expressed biomarkers from the training set correctly identified 100% of psychosis subjects from the testing set, and 80% of healthy controls (OOB estimate of error rate 10%). When training and testing sets were swapped, biomarker overlap was 46% and 90% of psychosis subjects and 90% of controls were correctly identified (OOB estimate of error rate 10%). The top 50 depleted biomarkers from the training set correctly identified 90% of psychosis subjects from the testing set, and 90% of healthy controls (OOB estimate of error rate 10%). When training and testing sets were swapped, depletion biomarker overlap was 2% and 70% of psychosis subjects and 40% of controls were correctly identified (OOB estimate of error rate 45%).
**Discussion:** The autoantibodyome in FEP differs from that of healthy individuals. In this novel pilot study, a panel of 50 differentially expressed autoantibodies allowed confident discrimination between patients and controls, potentially paving the way for development of antibody-based diagnostics for psychosis using a simple blood test and fewer autoantibodies. Depletion biomarkers, thought to represent antibodies selectively depleted from the blood due to target binding in tissues, had less replicability and utility than expression biomarkers, which may offer insights into the active role of the adaptive immune system in psychosis. Further work will attempt to validate this approach in larger samples, using psychiatric disease controls. This autoantibodyomic approach may also show promise for the identification of predictive and prognostic biomarkers in psychotic disorders.

### S13. DO PATIENTS WITH RECENT-ONSET DEPRESSION DIFFER CLINICALLY AND NEUROBIOLOGICALLY FROM DEPRESSED PATIENTS WITH A CLINICAL HIGH-RISK STATE FOR PSYCHOSIS?

Rachel Upthegrove[1], Renate Reniers[1], Pavan Mallikarjun[1], Eva Meisenzahl[2], Katharine Chisholm[1], Stefan Borgwardt[3], Stephan Ruhrmann[4], Raimo Salokangas[5], Paolo Brambilla[6], Stephen Wood[7], Nikolaos Koutsouleris[2], Paris Alexandros Lalousis*,[1]
*[1]University of Birmingham; [2]Ludwig Maximilians University; [3]University of Basel; [4]University Hospital, University of Cologne; [5]University of Turku; [6]University of Milan; [7]Orygen, the National Centre of Excellence in Youth Mental Health*

**Background:** Major depressive disorder (MDD) is one of the most common mental disorders, with a lifetime prevalence of 14.6%. The impact of depression is considerable; poor social and economic functioning and significant life limitations [1]. Depression is also the most common co-morbidity seen with other mental disorders. The prevalence of depressive disorder in schizophrenia has been reported to be around 40% [2]. When examining very early phases of illness, in groups identified as at clinical high risk (CHR) for psychosis, high rates of 'co-morbid' axis one diagnoses are reported, with over 50% reaching criteria for a depressive disorder. Those people with schizophrenia send depression are significantly more likely to relapse, to be a safety concern (be arrested, victimized or suicidal), have greater substance-related problems and poorer recovery [2]. In addition, depression has been linked to increased risk of transition from CHR to FEP, suggesting that in this group depression also indicates a poorer outcome [3]. Currently, the diagnosis of depression is based on the phenomenological evaluation of symptoms and behavior. However, there remains significant debate around the heterogeneity of depressive symptoms and their function as prognostic indicators [4]. Neuroimaging holds "diagnostic potential" for depression [5]. However, studies show that brain alterations are often small and reliability is difficult, and there has been no neuroimaging investigation of depression as a co-morbid diagnosis. We aim to further understand the symptom profile of depression in emerging mental disorders, including in the clinical high risk group (CHR) and recent onset psychosis (ROP) as compared to those with recent onset depression (ROD). This has important implications for the accurate identification of a potentially malleable target for treatment, and indeed development of novel therapeutic options. We also aim to explore the ability of brain imagining (structural MRI) to add accuracy to the classification prediction models
**Methods:** Data from the PRONIA study, an EUFP7 funded 8 center study recruiting ROD, CHR and ROP participants will be presented. Analysis will include demographic information and BDI-II (Beck Depression Inventory), CAARMS (Comprehensive Assessment of the At Risk Mental State), SANS (Scale for the assessment of negative symptoms) total score PANSS (Positive and negative Symptom Score) and SPI-A together with structural MRI imaging. We will report descriptive detail from the PRONIA discovery sample (n716), machine learning classification with Neurominer® and VBM analysis of sMRI scans across groups.
**Results:** Data from BDI-II symptom endorsement suggests a 'classical depression phenotype' corresponding to Becks 'cognitive triad'; "life is pointless, future hopeless, self as worthless" may separate depression in ROD from ROP, with other symptoms potentially able to separate ROP

from ROD. In classification, a 65% sensitivity and specificity are found. Data will also be presented on the CHR group and their alignment, together with VBM analysis for structural MRI examining correlates with highly weighted classifying symptoms in and across all three groups.

**Discussion:** When given early in the course of illness, interventions have the greatest potential impact, and characterization and accurate diagnosis of depression in emerging mental disorders is an important goal. This study suggests it may be possible to accurately identify depression in different diagnostic categories, including major depressive disorder, psychosis and clinical high risk, and that neuroimaging holds potential to add to diagnostic accuracy in complex co-morbid disorders.

### S14. DNA METHYLATION CHANGES IN GABAERGIC AND GLUTAMATERGIC MARKERS IN EARLY SCHIZOPHRENIA

Helene Fachim*[,1], Camila Loureiro[2], Fabiana Corsi-Zueli[2], Paulo Rossi Menezes[2], Paulo Louzada Jr[2], Caroline Dalton[1], Cristina Marta Del-Ben[2], Gavin Reynolds[1]
[1]*Sheffield Hallam University;* [2]*University of Sao Paulo*

**Background:** GABAergic and glutamatergic systems play an important role in the neurobiology of schizophrenia, and changes in their markers are reported in both postmortem human brain and in animal models. Recent studies have demonstrated that abnormalities in DNA methylation may underlie the alterations in various indicators of GABAergic and glutamatergic functions in schizophrenia. As our group previously found decreased NR2 protein plasma levels and downregulation of parvalbumin (PVALB) mRNA in first episode of psychosis (FEP) patients, we hypothesised that changes in DNA methylation may be responsible for these indicators of glutamatergic and GABAergic deficits in FEP patients.

**Methods:** Blood samples were collected from patients in FEP (n = 35) after their first contact with the mental health assistance, siblings (n = 21) and population-based controls (n = 35). Bisulfite conversion and pyrosequencing were used to determine methylation levels in 4 CpG sites in promoter sequence of PVALB and 5 CpG sites at GRIN2B (gene which encodes NR2).

**Results:** We found hypermethylation at a CpG site within the PVALB promoter sequence in patients and their siblings compared to population-based control group (p< 0.001) while overall hypomethylation was found in the 5 CpGs analysed within GRIN2B promoter sequence (p < 0.01).

**Discussion:** Our PVALB findings are consistent with our previous studies showing that PVALB promoter methylation is elevated in schizophrenia and, additionally this is the first evidence showing changes in GRIN2B promoter methylation in psychosis. These results together suggest that these epigenetic findings may relate to the reduction of protein expression of indicators of glutamate and GABA systems seen in this disease.

### S15. ABNORMAL EYE TRACKING IN PATIENTS WITH SCHIZOPHRENIA UNDER THE SOCIAL SCENE

Qijing Bo*[,1], Xianbin Li[1], Wenlong Jiang[1], Changming Wang[1], Chuanyue Wang[1]
[1]*Beijing Anding Hospital, Capital Medical University*

**Background:** To investigate whether the eye movement pattern is different between facial emotion recognition and real social scene emotion recognition, and which can better reflect the social function and the clinical symptoms using a novel theme identification task.

**Methods:** Total 29 patients with schizophrenia and 31 healthy controls completed the theme identification task, in which subjects selected which word, out of positive, neutral and negative, described the theme of a picture under facial emotion recognition and real social scene emotion recognition. Positive and negative syndrome scale (PANSS) and social function in psychosis inpatients (SSPI) were used to assess the symptom and social function.

**Results:** The schizophrenia's eye movement paradigms under both facial emotion and social scene show decreased number of fixation (t=-3.49, P=0.00; t=-3.62, P=0.00), decreased number of saccades (t=-3.15, P=0.00; t=-3.72, P=0.00), decreased scan path length (t=-2.23, P=0.03; t=-4.18, P=0.03), decreased fixation number in interest area (t=3.01, P=0.00; t=-3.24, P=0.00). Different from facial emotion cognition, the eye movement under social scene cognition showed lower percentage of fixation number in interest area than that in healthy subjects (P=0.01), furthermore, the length of scan path under the negative social scene pictures was associated with the total score of SSPI (r=-0.38, P=0.04), the PANSS total score (r=-0.46, P=0.01), the positive symptoms score (r=-0.39, P=0.04), the general score (r=-0.50, P=0.01).

**Discussion:** The patients showed more abnormal eye tracking indicators under social scene than facial emotion. Under negative emotion social scene, the length of scan path related to social function and clinical symptoms, it may be a potential indicator to evaluate social function and degree of disease.

### S16. GLUTAMATERGIC NEUROMETABOLITE LEVELS IN PATIENTS WITH TREATMENT-RESISTANT SCHIZOPHRENIA: A CROSS-SECTIONAL 3T PROTON MRS STUDY

Yusuke Iwata*[,1], Shinichiro Nakajima[2], Eric Plitman[2], Jun Ku Chung[2], Fernando Caravaggio[2], Julia Kim[1], Eric Brown[1], Nathan Chan[1], Parita Shah[1], Sofia Chavez[1], Philip Gerretsen[2], Masaru Mimura[3], Gary Remington[1], Ariel Graff-Guerrero[2]
[1]*Centre for Addiction and Mental Health;* [2]*Centre for Addiction and Mental Health, University of Toronto;* [3]*Keio University School of Medicine*

**Background:** In terms of response to antipsychotic treatment, patients with schizophrenia can be classified into three groups; (1) treatment-resistant patients who are clozapine (CLZ)-resistant (ultra treatment-resistant schizophrenia [UTRS]), (2) treatment-resistant patients who are CLZ-responsive (TRS), and (3) patients who respond to non-CLZ antipsychotics (treatment non-resistant schizophrenia [TnRS]). The aim of this study was to examine glutamatergic neurometabolite levels in these three patient groups, along with healthy controls (HCs), using proton magnetic resonance spectroscopy (1H-MRS).

**Methods:** Glutamate (Glu) and glutamate+glutamine (Glx) levels were assessed in the associative striatum (Str), anterior cingulate cortex (ACC), and dorsolateral prefrontal cortex (DLPFC) using 3T 1H-MRS (PRESS, TE=35ms). Neurometabolite levels were corrected for cerebrospinal fluid proportion.

**Results:** A total of 100 participants (26 UTRS, 27 TRS, 21 TnRS, and 26 HCs) were included in this study. Patients with UTRS showed higher Glx levels in the ACC compared to HCs (p=0.038). When patients with UTRS and TRS were combined into one group, this subset of patients showed higher Glu and Glx levels in the ACC compared to HCs (p=0.028 and p=0.023, respectively). There were no significant group differences in the Str or DLPFC.

**Discussion:** Previous findings reporting higher glutamatergic levels in the ACC of patients with TRS may be mainly influenced by patients with CLZ non-responder. Higher ACC glutamatergic neurometabolite level may be a biological trait of resistance to the first-line antipsychotic treatment that is retained even after CLZ administration.

### S17. ZNF804A GENE AND CANNABIS USE: INTERACTION ON THE RISK FOR PSYCHOSIS IN A NON-CLINICAL SAMPLE

Jordi Soler Garcia*[,1], Barbara Arias[2], Jorge Moya[3], Manuel Ignacio Ibañez[4], Generós Ortet[4], Lourdes Fañanás[2], Mar Fatjó-Vilas[2]
*[1]University of Barcelona, Biomedicine Institute of the University of Barcelona (IBUB); [2]University of Barcelona, Biomedicine Institute of the University of Barcelona (IBUB), Centre for Biomedical Research Network on Mental Health (CIBERSAM); [3]University of Lleida, Centre for Biomedical Research Network on Mental Health (CIBERSAM); [4]Universitat Jaume I, Centre for Biomedical Research Network on Mental Health (CIBERSAM)*

**Background:** The ZNF804A gene and cannabis use are risk factors for psychosis, both of which have also been associated with schizotypic traits. This study aimed to test whether ZNF804A (rs1344706) modulates the relation between cannabis use and schizotypy levels in a general population sample.
**Methods:** The sample consisted of 389 Spanish non-clinical subjects (43% males, mean age=21.1(2.19)). Schizotypy was evaluated with the three factors of the Schizotypal Personality Questionnaire-Brief (SPQ-B): Cognitive-Perceptual (CP), Interpersonal (I) and Disorganized (D). Subjects were classified as cannabis users or non-users. Multiple linear regressions were conducted to test the effect of genetic and environment factors and their interaction on SPQ-B scores. Sex and anxiety scores (evaluated with SCL) were included as covariates.
**Results:** The analyses showed a significant linear relationship between the ZNF804A and SPQ-I: homozygotes AA showed higher scores (p=0.001). An interaction between cannabis use and rs1344706 on SPQ-CP was observed: among individuals AA, cannabis users presented higher scores than non-users, while among individuals CC, cannabis users presented lower scores compared to non-users (p=0.005).
**Discussion:** These results add evidence on that the ZNF804A modulates schizotypy and suggest that schizotypy levels are influenced by an interaction between the exposure to cannabis and the ZNF804A genotype.

### S18. PREDICTING PROGNOSIS IN PATIENTS WITH FIRST EPISODE PSYCHOSIS USING AUDITORY P300: A 1-YEAR FOLLOW-UP STUDY

Kim Minah*[,1], Lho Kyung-jin[1], Lee Tak Hyung[2], Kwak Yoo Bin[2], Kwon Jun Soo[1]
*[1]Seoul National University College of Medicine; [2]Seoul National University College of Natural Science*

**Background:** Although early intervention is crucial for favorable outcome in patients with schizophrenia, development of biomarkers for predicting prognosis of psychotic disorder still requires more research. This study aims to investigate whether auditory P300 predict prognosis in patients with first episode psychosis (FEP) during 1-year of follow-up period.
**Methods:** Twenty-four patients with FEP were examined with auditory P300 at baseline, and their clinical status were re-assessed after 1 year. Multiple regression analysis was used to identify factors predictive of prognosis in FEP patients during the follow-up period.
**Results:** In the multiple regression analysis, P300 amplitude at CPz significantly predicted later improvement of Positive and Negative Syndrome Scale (PANSS) total, positive and general sub-scores. Improvement of Global Assessment of Functioning (GAF) and Brief Psychiatric Rating Scale (BPRS) scores were predicted by baseline P300 amplitude at CPz.
**Discussion:** P300 may be a possible predictor of improvement in symptoms and functional status, as well as overall psychiatric status in patients with FEP. Future study with larger sample and longer follow-up period is needed to confirm the findings of the current study.

### S19. EVIDENCE OF THE LIPID PARADOX IN PSYCHOSIS: A META-ANALYSIS OF CHOLESTEROL AND TRIGLYCERIDE LEVELS IN FIRST EPISODE PSYCHOSIS

Toby Pillinger*[,1], Katherine Beck[2], Brendon Stubbs[1], Oliver Howes[3]
*[1]Institute of Psychiatry, King's College; [2]King's College London, MRC Clinical Sciences Centre; [3]Institute of Psychiatry, King's College London, Hammersmith Hospital, Imperial College London*

**Background:** Untreated, active rheumatoid arthritis is associated with reduced total and lowdensity lipoprotein (LDL) cholesterol. Despite this apparently favorable lipid profile, these patients are at elevated risk of cardiovascular disease, with the association therefore referred to as the 'lipid paradox'. The mechanism underlying low total and LDL cholesterol in rheumatoid arthritis is considered to be driven by inflammatory cytokines. IL-6 reduces lipid levels in animal models, and treatment of rheumatoid arthritis with anti-IL-6 and anti-TNFalpha monoclonal antibodies is associated with an increase in total and LDL cholesterol. First episode psychosis (FEP) is associated with elevated levels of these same inflammatory cytokines, thus a similar mechanism underlying lipid abnormalities may exist. This study set out to clarify the lipid status of antipsychotic naive/minimally treated FEP, testing the hypothesis that if psychosis is deemed to be an inflammatory condition, then a serological metabolic signature characterized by reduced total and LDL cholesterol should be observed.
**Methods:** A meta-analysis of studies examining lipid parameters in individuals with FEP and no or minimal antipsychotic exposure versus a healthy control group was performed. Studies reported fasting total cholesterol, low-density lipoprotein (LDL) cholesterol, high-density lipoprotein (HDL) cholesterol, triglycerides and leptin levels.
**Results:** Of 2070 citations retrieved, 20 case–control studies met inclusion criteria including 1167 patients and 1184 controls. Total cholesterol and LDL cholesterol levels were significantly decreased in patients compared with controls, corresponding to an absolute reduction of 0.26 mmol/L (p = 0.005) and 0.15 mmol/L (p = 0.001) respectively. These findings remained in BMI-matched sensitivity analyses. Triglyceride levels were significantly increased in the patient group, corresponding to an absolute increase of 0.08 mmol/L (p = 0.02). However, HDL cholesterol and leptin levels were not altered in patients compared with controls.
**Discussion:** Total and LDL cholesterol levels are reduced in FEP, findings which persist in BMI-matched sensitivity analyses. This metabolic signature, combined with elevated insulin resistance that we have previously demonstrated in FEP, mirrors metabolic outcomes observed in pro-inflammatory conditions such as rheumatoid arthritis. FEP is associated with raised levels of multiple pro-inflammatory mediators, which include the adipocytokines IL6 and TNFalpha. IL6 is associated with a reduction in cholesterol in pre-clinical models, thus the mechanism underlying low cholesterol in FEP could be driven by a pro-inflammatory state, and the lipid paradox of rheumatoid arthritis may be present in FEP. These findings also indicate that hypercholesterolemia in patients with chronic schizophrenia is secondary and potentially modifiable. In contrast, triglycerides are elevated in FEP. Hypertriglyceridemia is a feature of type 2 diabetes mellitus, therefore this finding adds to the evidence for glucose dysregulation in this cohort.

### S20. PARAHIPPOCAMPAL THICKNESS PREDICTS TREATMENT IMPROVEMENT IN EARLY AND CHRONIC SCHIZOPHRENIA

Philipp Homan*[,1], Miklos Argyelan[2], Delbert Robinson[2], Todd Lencz[2], Anil Malhotra[2]
*[1]Feinstein Institute for Medical Research; [2]Zucker Hillside Hospital, Feinstein Institute for Medical Research*

**Background:** Despite recent advances, there is still a major need for prediction of treatment success in schizophrenia. Cortical thickness measures are relatively easy to obtain and may provide biomarker candidates. Here, we tested a set of candidate brain regions as predictors of treatment response in first episode schizophrenia and in two independent schizophrenia cohorts. Regions included the precuneus, inferior parietal gyrus, superior temporal gyrus, parahippocampal gyrus, anterior cingulate, inferior frontal gyrus, insula, lateral and medial orbitofrontal cortex, and occipital cortex.
**Methods:** In the discovery cohort, we used the whole sample of patients to estimate individual response slopes using Empirical Bayes, 36 of which had cortical thickness measurements at baseline. Patients were scanned aprior to treatment with either risperidone or aripiprazole. Symptoms were assessed with the Brief Psychiatric Rating Scale at baseline and over the course of up to 52 weeks. Cortical thickness in regions of interest were examined via magnetic resonance imaging and used as predictors of individual treatment response, defined as individual response slope.
**Results:** Parahippocampal thickness at baseline predicted the individual response to treatment ($P < 0.05$, Bonferroni-corrected). This was replicated in two independent schizophrenia cohorts including a recent onset cohort ($N = 33$) and a sample of chronic schizophrenia patients ($N = 52$), respectively. The overall effect was quantified with an internal meta-analysis ($\beta = 0.4$, 95% CI [0.24; 0.56]; $z = 4.84$, $P < 0.001$).
**Discussion:** Parahippocampal thickness may be a promising marker of treatment success both at the early and the chronic stage of schizophrenia.

### S21. EVENT-RELATED REPETITIVE TMS TO RIGHT POSTERIOR STS (BUT NOT OCCIPITAL FACE AREA) IN HEALTHY VOLUNTEERS (HV) BRIEFLY RECAPITULATES FACE EMOTION RECOGNITION (FER) DEFICITS OF SCHIZOPHRENIA (SZ)

Cheryl Corcoran*[,1], Jack Grinband[2], Jaimie Gowatsky[3], Casimir Klim[3], Matthew Hoptman[4], Daniel Javitt[5]
*[1]Icahn School of Medicine at Mount Sinai; [2]Columbia University, NYSPI; [3]NYSPI; [4]Nathan Kline Institute; [5]Columbia University, Nathan Kline Institute*

**Background:** Profound FER deficits exist in Sz, causing social disability, though can be partly remediated with computer-based training. Neurostimulation might augment remediation if critical nodes were identified. We aimed to 1) briefly recapitulate FER deficits of Sz in HV using rTMS to rpSTS, 2) identify connectivity patterns of rpSTS regressed by FER, and 3) apply TMS to rpSTS with fMRI as readout.
**Methods:** 1) Nine healthy volunteers had rTMS (10 Hz; 500 msec; 110% RMT) to rpSTS or rOFA (counterbalanced; 10/10 system overlay with standard MRI) concurrent (1/3 trials) with stimuli (http://faces.mpdl.mpg.de/) for emotion or gender identification (button press). 14 Sz patients completed these tasks without TMS. 2) Whole-brain resting-connectivity analyses, seeded by rpSTS, was applied in 27 Sz and 35 HV who also completed the UPenn FER task. 3) BOLD fMRI was obtained in 4 HV pre- and post-TMS to rpSTS (1 Hz; 20 minutes).
**Results:** 1) In HV, rTMS to rpSTS only (not OFA) significantly slowed reaction time for FER only (not gender identification): overall F test for logRT ($p=.001$) with post-hoc rpSTS vs.OFA ($p=.005$) and rpSTS vs. non-stim trials ($p=.004$). rpSTS recapitulated slowed RT ad lower FER accuracy of Sz. 2) In both HV and Sz, rpSTS had significant resting connectivity with V1 ($p= .00013$), positively modulated by FER accuracy. 3) Analyses are ongoing.
**Discussion:** rpSTS is a critical node in the FER circuit with connectivity to primary visual cortex modulated by FER, whose disruption recapitulates FER deficits, making it a candidate target for remediatory neurostimulation.

### S22. A TRANSDIAGNOSTIC NEUROANATOMICAL SIGNATURE OF PSYCHIATRIC ILLNESS

Gong Qiyong[1], Cristina Scarpazza*[,2], Jing Dai[3], Manxi He[3], Xin Xu[3], Yan Shi[3], Baiwan Zhou[1], Sandra Vreira[2], Eamon McCrory[4], Cheng Yang[1], Feifei Zang[1], Su Lui[1], Andrea Mechelli[2]
*[1]Huaxi MR Research Center (HMRRC), West China Hospital of Sichuan University; [2]Institute of Psychiatry, Psychology and Neuroscience, King's College London; [3]Chengdu Mental Health Center; [4]University College London*

**Background:** Despite an increasing research and clinical focus on trans-diagnostic approaches to mental health, it remains unclear whether different diagnostic categories share a common neuronatomical basis. The current investigation sought to investigate whether a shared (trans-diagnostic) set of structural alterations characterized schizophrenia, depression, post-traumatic stress disorder and obsessive-compulsive disorder, and determine whether any such alterations reflected markers of psychiatric illness or pre-existing familial vulnerability.
**Methods:** A total of 404 patients with a psychiatric diagnosis were recruited (psychosis, n=129; unipolar depression, n=92; post-traumatic stress disorder, n=91; obsessive compulsive disorder, n=92) alongside 201 healthy controls and 20 unaffected first-degree relatives. We collected structural Magnetic Resonance Imaging scans from each participant using the same 3.0 Tesla scanner and acquisition sequence, and tested for trans-diagnostic alterations using Voxel-Based Morphometry.
**Results:** We report that the four psychiatric groups were all characterized by significantly greater gray matter volume in the bilateral putamen relative to healthy controls (right putamen: x=32 y=6 z=-2; z-score: 5.97; p-value<0.001 after FWE-correction; left putamen: x=-30 y=5 z=-7; z-score: 4.97; p-value=0.001 after FWE-correction). Furthermore, the volume of this region was positively correlated with severity of symptoms across groups, so that a higher gray matter volume in the putamen was associated with higher severity of symptoms (partial correlation: the results are age and gender corrected; right putamen: r=0.313, p<0.001; left putamen: =0.326, p<0.001). Bilateral putamen enlargement was also evident in unaffected relatives compared to healthy controls (right putamen: x=32 y=-6 z=2; t-value: 8.13, p-value<0.001 after FWE-correction; left putamen: x=-32 y=-8 z=2; z-score:9.39, p-value<0.001 after FWE-correction).
**Discussion:** These findings indicate that increased putamen volume may reflect a trans-diagnostic marker of underlying familial vulnerability to psychopathology. This raises the prospect that this region could be used to assess degree of familiar vulnerability to psychopathology above and beyond traditional diagnostic boundaries. Our investigation provides support to emerging conceptualisations of psychiatric illness, in which each disorder is best understood as a combination of diagnosis-specific features and a trans-diagnostic factor reflecting general psychopathology.

### S23. INTRODUCING COMPASS: COMPARING BRAIN ACTIVITY ACROSS PATIENTS WITH DIFFERENTIAL TREATMENT RESPONSE IN SCHIZOPHRENIA – AN OBSERVATIONAL STUDY

Sandra Iglesias*[,1], Jakob Siemerkus[1], Martin Bischof[2], Sara Tomiello[3], Dario Schöbi[3], Lilian Weber[3], Jakob Heinzle[3], Julian Möller[4], Stephan Egger[2], Wolfgang Gerke[6], Markus Baumgartner[6], Wolfram Kawohl[7], Stefan Borgwardt[4], Stefan Kaiser[8], Helene Haker[3], Klaas Enno Stephan[3]
*[1]University of Zurich & Swiss Federal Institute of Technology (ETH Zurich); [2]University of Zurich; [3]ETH Zurich; [4]University of Basel; [6]Clienia Schlössli AG, Oetwil am See; [7]Psychiatrische Dienste Aargau AG; [8]Hôpitaux Universitaires de Genève*

**Background:** Present pharmacological treatment approaches in schizophrenia rest on "neuroleptic" drugs, all of which act as antagonists at dopamine D2/D3 receptors but additionally display major variability in their binding capacity to neurotransmitter receptors (Van Os & Kapur 2009). At present, the choice of any particular drug does not rest on any principled criteria: Once individual treatment has been started, therapeutic efficacy is monitored clinically, and a switch to a different drug is initiated when clear improvements remain absent after a few weeks. It is presently not possible to predict in advance which patients will respond well to a particular drug and who will experience little or no benefit (Case et al. 2011; Kapur et al. 2012).

For instance, clozapine and olanzapine are often prescribed after other antipsychotics have shown to be ineffective in patients with schizophrenia or related disorders due to their pronounced side-effects. Both drugs, clozapine and olanzapine, share certain pharmacodynamic properties with comparatively low affinity towards dopamine D2-receptors, but very high affinity towards muscarinic receptors – a unique constellation that distinguishes them from other common antipsychotics. Importantly, previous studies have shown that a subgroup of schizophrenia patients might particularly benefit from these properties (Raedler et al. 2003, Scarr et al. 2009).

Here, we present an ongoing observational study (COMPASS) which builds on these observations and addresses the question whether functional readouts of dopaminergic and muscarinic systems in individual patients could enable personalised treatment predictions. Guided by the dysconnection hypothesis of schizophrenia (Stephan et al., 2009), which postulates aberrant interactions between NMDA receptors and neuromodulators like dopamine/acetylcholine, the COMPASS study adopts a neuromodeling approach. The focus is on EEG/fMRI paradigms and computational models with empirically demonstrated sensitivity for altered function of NMDA, dopamine and muscarinic receptors, respectively.

**Methods:** To detect even small effect sizes, the study aims to recruit N=120 patients with schizophrenia who begin treatment with, switch to, or augment medication with olanzapine or clozapine. If possible, a replication sample (an additional N=120) will be recruited, too. Patients will be examined +/- 96h relative to treatment onset. Data acquisition encompasses the following measurements: Clinical interview, EEG (working memory, reward learning under volatility, auditory MMN under volatility, "resting"-state), MRI (optional; fMRI during auditory MMN under volatility, "resting"-state, and structural imaging), blood samples (genetic and biochemical analyses). After 2 and 8 weeks a clinical follow-up is conducted.

**Results:** The study is ongoing.

**Discussion:** The EEG/fMRI data will be analysed by computational models that infer functional states of glutamatergic, dopaminergic, and cholinergic systems (for review, Stephan et al. 2015). Model parameter estimates will serve as features in machine learning analyses of treatment prediction (Brodersen et al. 2014).

If successful, this proof-of-concept study will lead to clinically useful tests for predicting the efficacy of clozapine/olanzapine prior to or during very early treatment. This could have a significant impact on clinical management as it would enable predicting, at an early stage, the therapeutic benefit for individual patients. Our neuromodeling approach to individual predictions may thus provide a principled basis for treatment decisions, help spare side-effects and enable informed switches in treatment strategy.

### S24. IS IT FEASIBLE TO PREDICT LONG-TERM METABOLIC OUTCOMES IN PSYCHOSIS USING BIOLOGICAL PROFILING AT BASELINE?

Conrad Iyegbe*[,1], Lauren Allen[2], John Lally[2], Marta DiForti[3], Robin Murray[2], Fiona Gaughran[2]

*[1]Department of Psychological Medicine; [2]Department of Psychosis Studies; [3]Centre for Social, Genetic and Developmental Psychiatry*

**Background:** Antipsychotic medications are widely prescribed for the treatment of psychotic disorders but carry a variable propensity to increase weight. Thus metabolic dysfunction is the primary cause of premature death in psychosis patients. A system-based approach to understanding the molecular mechanisms behind metabolic dysfunction can potentially provide scope for tailored interventions and alternative treatment pathways that avert such risks on an individual basis. The aim of this study is to identify transcriptomic predictors of high Body Mass Index (BMI) and blood glucose in first episode and chronic psychosis patients.

**Methods:** 100 first-episode and 100 chronic cases of psychosis meeting ICD-10 criteria (F20-29 and F30-33) were recruited as part of 2 independent studies from 3 NHS Trusts: South London and Maudsley (SLAM), Oxleas and Sussex. Cases were ethnically mixed and aged between 18–65. All participants gave informed consent for biological sampling and a range of physical health assessments. Blood glucose was measure using HbA1c while height and weight data were also taken and used to calculate BMI. For FEP subjects biological measures were taken at baseline, 3 months and 12 months post recruitment. RNA samples were collected at the baseline timepoint via PAXgene blood tubes and interrogates, using the Illumina HumanHT-12.v4 beadchip array. Samples were run at the National Institute for Health Research's (NIHR) Biomedical Research Centre for Mental Health (BRC-MH) at the Institute of Psychiatry, Psychology and Neuroscience. A total of 4756 probes passed a stringent quality control across the 200 samples.

**Results:** Quantitative data on BMI and hba1c levels were used to assess the predictive efficacy of variables grouped by source (ie. clinical, demographic, technical and transcriptomic features) in first episode psychosis patients. All the predictor categories were included in the initial model, although individual categories were then dropped one at a time. This leave-one-out strategy allowed the direction, impact and relative contribution of the different feature classes to be assessed. Gene-expression and clinical features were consistently associated with the lowest Mean Squared Error after 100 iterations of K-fold cross-validation and after 11 different values of the alpha parameter across 500 imputed datasets. Hba1c or BMI was used as the clinical predictor, depending on whether Hba1c or BMI was used as the target variable. Unattributed surrogate variables derived from surrogate variable analysis (n=6) were analysed within the technical feature set. Having established that gene expression has inherent value as a predictor of metabolic status the same analytical steps were repeated for the discretised versions of these traits (ie. diabetes and obesity). Top-ranking gene transcripts were compared between the quantitative and discretised models. Rank lists of transcripts were subsetted to allow the power distribution across ordered transcripts to be profiled.

**Discussion:** The top performing transcripts identified are undergoing validation analysis in the chronic sample. Results will be conveyed in terms of sensitivity and false positive rates (ie. the area under the Receiver Operating Characteristic curve). We will undertake further validation through trajectory analysis of gene-expression profiles in followed-up patients.

### S25. COGNITIVE REMEDIATION THERAPY AND ITS EFFECTS ON BDNF SERUM LEVELS

Rafael Penades*[,1], Irene López-Vílchez[2], Rosa Catalán[2], Barbara Arias[3], Clemente García-Rizo[4], Guillem Masana[4], Miquel Bernardo[2]

*[1]Hospital Clinic Barcelona, Universitat Barcelona, IDIBAPS, CIBERSAM; [2]Hospital Clínic Barcelona, Universitat Barcelona; [3]Universitat Barcelona; [4]Hospital Clínic Barcelona*

**Background:** Brain-derived neurotrophic factor (BDNF) has been proposed as a biomarker of schizophrenia and, more specifically, as a biomarker of cognitive recovery. Unfortunately, it has only been tested once with cognitive remediation treatment (CRT).

**Methods:** A randomized and controlled trial (NCT02341131) with 70 schizophrenia outpatients and 15 healthy volunteers was conducted. The participants with schizophrenia were randomly assigned to either CRT or the control group. All the participants were assessed in terms of cognition, quality of life, and their serum BDNF levels at both baseline and after the intervention. Additionally, comparisons of the effects of the different genotypes of the Val66Met polymorphism at the BDNF gene on the outcome variables were also performed.

**Results:** The patients in the CRT group presented with improvements in cognition. However, no significant changes were detected in the serum levels of BDNF. Interestingly, we found a significant positive interaction effect between the serum BDNF levels and the different BDNF genotypes. The Val/Val group showed significantly higher serum levels after the CRT treatment.

**Discussion:** The replication of the previous finding of increased serum BDNF levels after cognitive remediation in clinically stable individuals with schizophrenia was not achieved. However, our data indicated that genetic variability may be mediating serum BDNF activity in the context of CRT. All in all, the current consideration of BDNF as a biomarker of cognitive recovery in schizophrenia is promising but still premature.

### S26. HERITABILITY OF SOCIAL MISTRUST IN CHILD AND ADOLESCENT NON-CLINICAL SAMPLES: A HEALTHY TWINS STUDY

Han-yu Zhou[1], Keri Wong[2], Li-juan Shi[3], Xi-long Cui[4], Yun Qian[5], Ya-song Du[5], Simon S.Y. Lui[6], Xue-rong Luo[5], Eric F.C. Cheung[6], Anna Docherty[7], Raymond Chan*[,1]
*[1]Institute of Psychology; [2]University of Cambridge; [3]Hunan University of Science and Technology; [4]Central South University; [5]Shanghai Mental Health Centre; [6]Castle Peak Hospital; [7]VIPBG*

**Background:** Paranoia, or excessive suspiciousness of others, has been one of the core psychotic symptoms of schizophrenia. Recent studies have extended the study of psychotic symptoms in clinical groups to psychotic-like experiences in the general population. Few studies have systematically examined the prevalence of paranoid thinking or its attenuated form, social mistrust, in young children in the community. The present study examined the Social Mistrust Scale (SMS) and utilized it to examine the structure, prevalence, and heritability of social mistrust in a large sample of Chinese children and adolescents.

**Methods:** We administered the SMS to 1047 pairs of healthy twins aged 8 to 14 years and conducted structural equation modelling (SEM) to assess the structure of the SMS. Heritability of social mistrust was estimated in a subsample of twins (n=959 pairs). Finally, we examined administered the SMS to 32 adolescents with childhood-onset schizophrenia and 34 healthy controls to examine the convergent validity between the SMS and the Positive and Negative Syndrome Scale (PANSS).

**Results:** The SEM showed a three-factor structure for social mistrust (home, school, and general mistrust). Social mistrust was moderately heritable (39%, 95% CI [21%-59%]) with context-dependent sex differences. The SMS exhibited good discriminant validity in distinguishing adolescents with childhood-onset schizophrenia from healthy controls (AUC=0.80), and good convergent validity with the Positive and Negative Syndrome Scale (rs = 0.33–0.45).

**Discussion:** Taken together, the present findings showed a stable latent structure of the SMS in a large-scale non-clinical sample of children and adolescents. We found a moderate heritability estimate for social mistrust (39%) in a large healthy-twin sample. In addition, significant gender differences were found, where home mistrust was heritable for males (58%) but not for females, and school mistrust was heritable for females (54%) but not for males. Finally, we also demonstrated that the SMS possesses good discriminate validity in identifying adolescents with childhood-onset schizophrenia from healthy controls and convergent validity with standardized clinical measures of schizophrenia symptoms.

### S27. RELIABILITY OF SCHIZOPHRENIA DIAGNOSES IN CHILDREN AND ADOLESCENTS IN DENMARK

Ditte Lammers Vernal*[,1], Anne Dorte Stenstrøm[2], Nina Staal[3], Anne Marie Raabjerg Christensen[3], Christine Ebbesen[4], Anne Katrine Pagsberg[3], Christoph Correll[5], René Ernst Nielsen[1], Marlene Briciet Lauritsen[6]
*[1]Aalborg University Hospital; [2]University Clinic Odense, University of Southern Denmark; [3]Child and Adolescent Mental Health Center, Mental Health Services, Capital Region of Denmark; [4]Center for Child and Adolescent Psychiatry, Aarhus University Hospital, Central Denmark Region; [5]The Zucker Hillside Hospital, Northwell Health; [6]Aalborg University Hospital, North Denmark Region*

**Background:** Schizophrenia in children and adolescents are diagnosed using the same criteria as for adults, but the assessment may be more complex due to both developmental issues, premorbid difficulties and a less elaborated symptomatic presentation. There is a great scarcity of studies looking into validity of schizophrenia in children and adolescents.

**Methods:** We aimed to assess 1) the concordance and validity of schizophrenia register diagnoses among children and adolescents (early onset schizophrenia=EOS) in the Danish Psychiatric Central Research Register (DPCRR), and 2) the validity of clinical record schizophrenia diagnoses. Furthermore, to extract data from psychiatric records with confirmed schizophrenia in order to describe premorbid characteristics, history and symptomatology.

Psychiatric records from 200 patients with a first-time diagnosis of schizophrenia (F20.x) <18 years between 1994 and 2009 in the DPCRR was randomly selected for the study. The psychiatric records were evaluated by experienced clinicians according to ICD-10 criteria, using a predefined checklist. All records were assessed by two raters and inter-rater reliability was assessed.

**Results:** We were able to retrieve 178 of the 200 psychiatric records. The mean age of patients was 15.2 years, and 56.2% were male. The register-based and clinical diagnosis matched in 158 cases. In the 10.2% registration errors, the records reported schizophrenia as a rule-out tentative diagnosis in the majority of cases. Among the 158 psychiatric records with a clinical diagnosis of schizophrenia, the raters confirmed 132 records (83.5%) as schizophrenia and a total of 145 records (91.8%) as in the schizophrenia-spectrum. Interrater reliability was substantial with Cohen's kappa >0.78–0.83. Compared to diagnoses made in outpatient settings, EOS diagnoses during hospitalizations had fewer registration errors and a higher validity between raters' diagnosis and clinical diagnosis.

Among the cases with EOS confirmed by raters, 85.8% had family history of mental disorders, 93.1% had experienced adverse life events during childhood with 46.9% having experienced trauma. Hallucinations were present in 76.9%, negative symptoms in 57.4% and formal thought disorder symptoms in 34%. Catatonic symptoms were described in 4.7% cases.

**Discussion:** To our knowledge, the study is the largest to date investigating validity of schizophrenia diagnosed in children and adolescents in clinical settings. The study confirms assessment of schizophrenia in children and adolescents to be complex, especially in outpatient settings. All evaluations by raters were conducted by use of psychiatric records retrospectively. As the diagnoses were made 8 - 24 years ago, it is believed to be the best method, however, the possibility exists that some cases were not confirmed due to lack of adequate description of psychopathology in the records. Furthermore, the raters were not blinded to the diagnoses, as only patients with a register diagnosis of schizophrenia were included.

### S28. THE ROLE OF COPING IN THE ASSOCIATION BETWEEN SUBCLINICAL PSYCHOTIC EXPERIENCES AND DAILY FUNCTIONING: EVIDENCE FROM TWO INDEPENDENT ADOLESCENT SAMPLES FROM THE GENERAL POPULATION

Katharine Chisholm*[,1], Johanna Wigman[2], Danielle Hallett[1], Tamara Woodall[1], Simone Mahfouda[3], Renate Reniers[1], Eoin Killackey[4], Alison Yung[5], Stephen Wood[4], Ashleigh Lin[6]
[1]*University of Birmingham;* [2]*University of Groningen, University Medical Center Groningen, University Center for Psychiatry;* [3]*Telethon Kids Institute, The University of Western Australia;* [4]*Orygen, The National Centre of Excellence in Youth Mental Health;* [5]*Institute of Brain Behaviour and Mental Health, University of Manchester;* [6]*Telethon Kids Institute*

**Background:** Subclinical psychotic experiences (attenuated, brief, or limited psychotic-like experiences) are present in approximately 5% of adults and 7.5% of adolescents from the general population. Whilst the majority of these experiences are transitory, individuals who report subclinical psychotic experiences are at greater risk developing psychotic spectrum disorders, as well as other adverse outcomes. It is now well established that there is an inverse association between psychosocial functioning and (subthreshold) psychotic experiences in both clinical and non-clinical populations, however the mechanisms which drive this association are unclear. Adolescents with subclinical psychotic experiences are more likely to use maladaptive coping strategies and less likely to use adaptive ones. Given that coping styles are potentially modifiable, clarifying how coping may mediate the association between subclinical psychotic experiences and functioning could provide an important avenue for psychosocial intervention. In the current study we aimed to determine whether the association between subclinical psychotic experiences and psychosocial functioning is mediated by coping style. We conducted a within study replication in two large adolescent samples from the general populations of Australia and the United Kingdom.
**Methods:** 723 adolescents from Melbourne, Australia, and 239 adolescents from Birmingham, UK, took part in the study. Subclinical positive psychotic experiences were measured using the Community Assessment of Psychic Experiences (CAPE). The Coping Inventory for Stressful Situations (CISS) assessed three different coping styles; task oriented (adaptive) styles and emotion and avoidance oriented (maladaptive) styles. Functioning was measured via the Multidimensional Assessment of Functioning Scale (MAFS), which assesses general, family, and peer functioning. Mediation analysis was conducted using the PROCESS macro for SPSS.
**Results:** Subclinical psychotic experiences were strongly associated with reduced general and family functioning, and to a lesser extent with reduced peer functioning. Higher subclinical psychotic experiences were associated with lower task (adaptive) and avoidance (maladaptive) oriented coping and increased emotion (maladaptive) oriented coping. Task and emotion oriented coping were found to significantly mediate the relationship between subclinical psychotic experiences and all three types of functioning in both the Melbourne and the Birmingham samples. Avoidance oriented coping was found to significantly mediate subclinical psychotic experiences and peer functioning in the Melbourne sample only. Avoidance oriented coping was not found to mediate subclinical psychotic experiences with general or peer functioning in either sample.
**Discussion:** Given that 17% of children and 7.5% of adolescents experience subclinical psychotic experiences and that these experiences are associated with reduced functioning, high levels of distress, and suicidal ideation, introducing classroom based learning about coping strategies in schools may encourage the adoption of more positive coping strategies earlier. Additionally, the findings of the present study have important clinical treatment implications, as they suggest that techniques which increase levels of adaptive coping and reduce levels of maladaptive coping (in particular emotion-oriented styles) may help to break the cycle between subclinical psychotic experiences, functional decline, and eventual need for care.

### S29. CONCORDANCE BETWEEN SELF-REPORT AT INTERVIEW-BASED RATINGS OF PSYCHOTIC EXPERIENCES IN PRE-ADOLESCENCE AND ASSOCIATED PSYCHOPATHOLOGY

Martin K. Rimvall*[,1], Steffie Gundersen[1], Lars Clemmensen[1], Charlotte Ulrikka Rask[2], Anne Mette Skovgaard[3], Frank Verhulst[1], Jim van Os[4], Pia Jeppesen[1]
[1]*Child and Adolescent Mental Health Center, Mental Health Services, The Capital Region of Denmark;* [2]*Aarhus University Hospital;* [3]*University of Copenhagen;* [4]*Maastricht University Medical Centre*

**Background:** Self-report measures are often used to assess psychotic experiences (PE) in large-scale epidemiological samples, and have contributed substantially to our knowledge on PE. However, different self-report PE (PE-S) measures have yielded particularly wide-spread prevalence-estimates of PE ranging from 21–66 % in 7–13 year old children, whereas interview based measures of PE (PE-I) vary less (10 -23 %). Especially PE-S have been criticized for being over-inclusive and not capturing the essence of low-grade psychosis. The current study is the first large-scale study to examine the psychometric properties of a PE-S measure in children, and the first to compare the clinical correlates of PE-S and PE-I in the same sample.
**Methods:** As part of the general population Copenhagen Child Cohort 2000 studies, 1571 children aged 11–12 years were independently assessed for both PE-I and PE-S. PE-I were assessed by trained professionals with 22 items on hallucinations and delusions from the Kiddie Schedule for Affective Disorders and Schizophrenia present and life-time version (Kiddie-SADS-PL). PE-S were assessed by 10 questions covering hallucinations, delusions and subjective thought disturbances ever in life, forming a new section of the diagnostic interview, the Development and Well Being Questionnaire (DAWBA).
We assessed the psychometric properties of PE-S, using PE-I as the "gold-standard". We analyzed the association between PE-S and emotional and neurodevelopmental DSM-IV disorders of the child as well as a history of psychotic disorders in 1st degree family members. Both have previously been examined in previous studies of the current cohort, and were significantly associated with PE-I.
**Results:** The prevalence of PE-S was higher compared to PE-I, 28.1% and 10.2% respectively. The predictive values of any type of PE-S for any PE-I were: sensitivity = 73.8%, specificity = 77.1%, positive predictive value = 26.8% and negative predictive value = 96.3%.
The association between PE and mental health disorders and a family history of psychotic disorders yielded slightly lower odds ratios (OR) for PE-S compared to PE-I. However, the associations remained statistically significant and had overlapping confidence intervals: For any emotional or neurodevelopmental DSM-IV disorder: PE-I OR 3.3 (CI95% 2.3–4.8) and PE-S OR 2.7 (CI95% 2.1–3.6), for a 1st degree family history of psychotic disorder; PE-I OR 4.9 (CI95% 1.9–12.5) and PE-S OR 2.6 (CI95% 1.1–6.3).
**Discussion:** PE-S were almost 3 times more likely to be reported, compared to observer-rated PE-I. However, the associations with unfavorable clinical correlates were only slightly attenuated for PE-S when compared to PE-I. The study confirmed that PE-S are clinically relevant, and the DAWBA-section proved valuable as a screening tool for PE in the pre-adolescent general population.

### S30. UNDERSTANDING THE NATURE OF CHILDHOOD SUSPICIOUSNESS: A QUALITATIVE STUDY

Keri Wong*[,1]
[1]*University of Cambridge*

**Background:** Paranoia exists on a continuum of severity in adult patient populations and more recently it has been found to exist in children and adolescents in the general population. Childhood paranoia, assessed by the Social Mistrust Scale (SMS), has been found to be related to both internalising and externalising problem behaviours (Wong, Freeman & Hughes, 2014); however, the nature of why children's suspicions are related to psychosocial functioning remains unexamined. The current qualitative study addresses this gap by following up the original 2014 sample to examining the nature of children's suspicions using thematic analysis. By giving voice to children and adolescents, I will discuss: 1) children's definition of trust and mistrust more broadly 2) the common themes generated from interview questions about children's suspicions in relation to baseline self-reported levels of suspiciousness on the Social Mistrust Scale (SMS) and 3) other developmental psychosocial factors contributing to childhood suspicions. This study is also the first study to address whether or not children's suspicions are valid, or grounded in reality, using interviewer ratings and child self-report measures of mistrust.
**Methods:** 118 trusting and persistently mistrustful children from the UK (n=40) and Hong Kong (n=78) were matched and followed-up at 6 and 12 months based on their self-reported levels of suspiciousness on the Social Mistrust Scale. Correlations and kappas were conducted to assess the stability and convergent validity between assessments. Thematic analysis was conducted on 95 (80%) randomly selected semi-structured interviews about mistrust. The coding scheme generated from this analysis was further tested on the remaining transcripts for discriminant validity.
**Results:** Children's definition of trust was consistent with existing developmental literature. Commonly discussed topics related to mistrust, particularly school mistrust, included (i) experiences of bullying, concerns with popularity and the consequences of being targeted, (ii) emotional worries, anxieties and feelings of hostility, spying, and teasing, and (iii) coping mechanisms that maintained children's avoidant behaviours. Consistent with the threat anticipation cognitive model of delusions (Freeman et al., 2007), persistently mistrustful children reported frequent peer victimization and hostile attributional bias. Instances of unfounded paranoia were rare but not absent. There was moderate convergent validity between interviewer ratings and the SMS ($k=.49$, $p<.001$). The coding scheme discriminated trusting and mistrustful children accurately.
**Discussion:** Interviews with trusting and persistently mistrustful children are necessary in verifying unfounded childhood suspicions. Complementing self-report measures of suspiciousness, thematic codes from this study have the potential to screen for persistent and strongly held suspicions that may develop into delusions later in life.

### S31. BASIC SENSE OF SELF IN YOUTH AT HIGH RISK FOR DEVELOPING SCHIZOPHRENIA

Hadar Hazan*[,1], Richard Linscott[1], Elaine Reese[1]
[1]*University of Otago*

**Background:** Phenomenological researchers argue that schizophrenia is first and foremost a disorder of the basic sense of self (also known as ipsity, minimal or core self), that is, of the immediate, pre-reflective, embodied sense of being immersed in the world. According to the self-disorder model, impairment of the basic sense of self precedes clinical symptoms and is independent of them. Therefore, we postulated that youth at high psychometric risk for developing schizophrenia would present an impairment in their basic sense of self, as measured by levels of ego strength, basic symptoms, and pronoun usage.
**Methods:** Eighty undergraduate students aged 19–22 years ($M = 20.83$ years, $SD = 1.28$ years) completed the Schizotypal Personality Questionnaire (SPQ), Ego Strengths Questionnaire (ESQ), a self-report version of Schizophrenia Proneness Instrument (SPI-A), and four written narratives about personal and fictional experiences. Based on the SPQ scores, participants were allocated to either control (at or below the 84th percentile on all three subscales) or study group (above the 90th percentile on at least one subscales). To obtain the linguistic dimension of the pronouns usage in the written narratives, the essays were subjected to Linguistic Inquiry and Word Count (LIWC).
**Results:** Compared to the control group, the high-risk group presented lower levels of ego strength, higher levels of basic symptoms, and used more personal pronouns and the they pronoun in narratives. Self-report on the SPI-A and ESQ correlated significantly with the objective lexical pattern of pronoun use: Lower ego strength correlated with greater use of they and more self-reported basic symptoms correlated with greater use of pronouns overall, personal pronouns, and the pronouns she and they. Ego strength had the most predictive power for group membership
**Discussion:** In line with the hypotheses, there were significant differences between the schizotypy and the control groups in objective and subjective measures of basic sense of self. Subjective measures indicated a lower level of ego strength and higher levels of basic symptoms for the schizotypy group, as compared with the control group. Objective measures revealed a different lexical pattern with higher use of third-person and personal pronouns for the schizotypy group, as compared with the control group. Subjective (basic symptoms and ego strength) and objective measures significantly correlated with each other (pronoun use). Nevertheless, it is only the level of cognitive-perceptive disturbances that best predicted membership of the schizotypy group. Taken together, these results indicate a weak sense of basic self, namely a self-disorder, in a nonclinical population. Detection of self-disorder in the premorbid and prodrome stages of schizophrenia, paired with a suitable intervention, can help to prevent or at least minimize, the eruption of its active stage. In the future, it needs to be determined how these measures of self-disorder in non-clinical population can predict transition to schizophrenia and to other psychotic disorders. Furthermore, it would be valuable to test the distributions of measures of self-disorder in younger population from a more diverse background, such as high school students from a different socio-economic background.
Lastly, it is possible to conclude that some impairment in the basic sense of self does exist in schizotypy. This is apparent across the measures of self-disorder and suggests that there is a core feature that distinguishes schizotypes from non-schizotypes.

### S32. ANXIETY IN THE DEVELOPMENT OF PSYCHOTIC EXPERIENCES IN CHILDREN AND ADOLESCENTS: A SYSTEMATIC REVIEW

Jessica Bird*[,1], Felicity Waite[1], Daniel Freeman[1]
[1]*University of Oxford*

**Background:** Although traditionally separated in psychiatry, the overlap between psychosis and neurosis is becoming clear. It has been argued that emotional processes are not only a core component of psychotic experiences (PEs), but also have a causal role in symptom development. In particular, increasing evidence has highlighted anxiety may be especially important in the emergence of psychotic experiences. As the time when psychotic symptoms first emerge, understanding how anxiety influences their development in childhood and adolescence will inform early intervention. In this systematic review, we investigate the role of anxiety across the spectra of psychotic experiences in children and young people. We examine the available evidence whilst aiming to answer the questions of whether anxiety and psychotic experiences co-occur in young people and if anxiety plays a contributory causal role.
**Methods:** A systematic literature search was conducted on PsychINFO, Medline, EMBASE, and Scopus (updated October 2017). Papers published in peer-reviewed journals were identified that investigated the relationship between anxiety and PEs in children and adolescents within both non-clinical and clinical populations. Twenty-three papers met inclusion criteria. The RTI item bank assessment framework for observational studies was used to evaluate the precision and risk of bias of each individual study.
**Results:** The findings of this review demonstrated clear evidence for a consistent relationship between anxiety and psychotic experiences in non-clinical populations of young people, which generally displayed the highest quality of evidence. Although of overall lower quality, this relationship was also evident in clinical populations of young people with mental health problems other than psychosis. Evidence for the role of anxiety in those at clinical risk high for psychosis or diagnosed with psychosis was less conclusive, with very few studies, of variable quality, identified. These studies provided mixed findings, with some evidence for a co-occurrence between anxiety and psychosis risk/disorder, but no clear evidence of a causal role for anxiety in the transition to psychotic disorder. The size of the associations with anxiety appeared to vary by psychotic experience, with moderate evidence for hallucinations and little evidence grandiosity or anhedonia. Paranoia has the strongest evidence for a relationship with anxiety. Most studies were cross-sectional with few longitudinal studies preventing clear conclusions on a causal role of anxiety.
**Discussion:** Evidence suggests that anxiety and psychotic experiences may co-occur in young people. This relationship appears to be transdiagnostic and potentially specific to individual psychotic experiences. As a result, more studies focusing on specific psychotic symptoms and psychological mechanisms are needed. However, research must move beyond cross-sectional associations and test the potential contributory causal role of anxiety using prospective, experimental, and interventionist designs. Despite the unknown direction of causality, the notable associations of psychotic experiences and anxiety in child mental health services indicate that this relationship may be of clinical importance. Establishing higher quality research in clinical settings is essential. Testing the effect of treating anxiety on the severity of psychotic symptoms will inform early intervention.

### S33. ETRO - A PROSPECTIVE FOLLOW-UP STUDY OF THE COMBINED TREATMENT APPROACH “ROBIN” FOR ADOLESCENTS WITH HIGH RISK FOR DEVELOPING A PSYCHOTIC DISORDER: THERAPY MODULES ENHANCED BY A SMARTPHONE APPLICATION

Maurizia Franscini*[,1], Nina Traber-Walker[1], Miriam Gerstenberg[1], Sibylle Metzler[1], Susanne Walitza[1]
*[1]Psychiatric University Hospital Zurich*

**Background:** The most promising strategy in targeted prevention of psychotic disorders is to treat the at risk-symptoms in the pre-psychotic period. Although high risk-symptoms for psychotic disorder are common in adolescence and associated with a marked reduction in functioning, the evidence base required to guide effective interventions for adolescents at risk and even first-episode psychosis is limited. The clinicians from the early intervention center in Zurich have developed the treatment approach “Robin” (standardized manual and smartphone App) for adolescents with high risk for developing a psychotic disorder. The manual is targeting at risk-symptoms, comorbid disorders, improvement of quality of life and daily functioning. The therapy modules are based on evidence based treatment strategies in adults with a high risk and recommendations for adolescents with first episodes of psychosis. It follows the guidelines on early intervention in clinical psychosis high risk states of the European Association for Psychiatry. The intervention also includes a smartphone application for supporting the patients between sessions. This application targets real-time symptom assessment, medication adherence, and provides coping strategies for dealing with symptoms of psychosis and daily life hurdles.
**Methods:** The clinical intervention study ETRo is designed as a naturalistic controlled trial. The goal is to compare efficacy of a 16-week intervention in patients with at-risk symptoms (age range 13–18) with an active control group (treatment as usual). Power calculations conducted in collaboration with a statistician determined the recruitment goal of 30 participants in the treatment condition. Participants from a former early recognition study (N=62, Age: 13–18 years, Ø 15.06) are included for the control condition. For the intervention condition, help seeking adolescents with APS-Symptoms, aged 13–18, are being recruited during a three year time period. Within this prospective study, at-risk symptoms and data for comorbid symptoms, functioning, self-efficacy, and quality of life are collected at six time points (baseline, during the treatment period, immediately after intervention and 6, 12 and 24 months later).
**Results:** Since August 2017, first participants have been included and their treatment has started. In Florence, we will present our first results. This will include implementation of the treatment program and first findings of treatment period.
**Discussion:** Even though young patients with at-risk symptoms may profit best of specialised treatment approaches, little is known about age-appropriate treatment strategies in this vulnerable age group. This is one of the first controlled trials to test the efficacy of a specific treatment program for minor patients with attenuated psychotic symptoms.

### S34. DEVELOPMENTAL TRAJECTORIES OF PSYCHOTIC EXPERIENCES AND THEORY OF MIND IN 11-YEAR-OLD OFFSPRING OF PARENTS WITH SCHIZOPHRENIA OR BIPOLAR DISORDER

Maja Gregersen*[,1], Lars Clemmensen[2], Anne Søndergaard[3], Camilla Jerlang Christiani[3], Nicoline Hemager[4], Ditte Ellersgaard[3], Katrine Søborg Spang[5], Aja Greve[6], Ditte Lou Gantriis[6], Christina Bruun Knudsen[6], Anna Krogh Andreassen[6], Lotte Veddum[6], Henriette Brockdorff Stadsgaard[6], Vibeke Fuglsang Bliksted[6], Ole Mors[6], Kerstin J. Plessen[7], Merete Nordentoft[8], Jens Richardt Møllegaard Jepsen[9], Anne A.E. Thorup[5]
*[1]Mental Health Services, Capital Region of Denmark, Mental Health Centre Copenhagen; [2]Mental Health Services, Capital Region of Denmark, Child and Adolescent Mental Health Centre; [3]Mental Health Services, Capital Region of Denmark, Mental Health Centre Copenhagen, The Lundbeck Foundation Initiative for Integrative Psychiatric Research; [4]Mental Health Services, Capital Region of Denmark, Mental Health Centre Copenhagen, Child and Adolescent Mental Health Centre, The Lundbeck Foundation Initiative for Integrative Psychiatric Research; [5]Mental Health Services, Capital Region of Denmark, Child and Adolescent Mental Health Centre, The Lundbeck Foundation Initiative for Integrative Psychiatric Research; [6]The Lundbeck Foundation Initiative for Integrative Psychiatric Research, Aarhus University Hospital; [7]Mental Health Services, Capital Region of Denmark, Child and Adolescent Mental Health Centre, The Lundbeck Foundation Initiative for Integrative Psychiatric Research, University of Copenhagen; [8]Mental Health Services, Capital Region of Denmark, Mental Health Centre Copenhagen, The Lundbeck Foundation Initiative for Integrative Psychiatric Research, University of Copenhagen; [9]Mental Health Services, Capital Region of Denmark, Child and Adolescent Mental Health Centre, The Lundbeck Foundation Initiative for Integrative Psychiatric Research, Center for Clinical Intervention and Neuropsychiatric Schizophrenia Research*

**Background:** The study is a part of the Danish High Risk and Resilience Study, Via 11 and aims to explore the developmental trajectories of psychotic experiences (PEs) and theory of mind in children born to parents with schizophrenia or bipolar disorder. In a cross sectional perspective we also aim to explore possible associations between PEs and social cognitive deficits, particularly hyper-theory-of-mind. We also wish to explore the significance of other potential risk factors for PEs such as cognitive biases, adverse life events, and insecure attachment styles. Earlier studies have shown that PEs during childhood are predictive of later psychotic disorders, especially if they persist over time. We expect the possible risk factors to have a cumulative effect.

**Methods:** The Danish High Risk and Resilience Study, Via 11, is the first follow-up of a cohort of 522 children and their parents. The cohort consists of children where one or both parents have been diagnosed with a schizophrenia spectrum disorder (N=202), children where one or both parents have been diagnosed with bipolar affective disorder (N=120) and children where neither of the parents have been diagnosed with these disorders (N=200). The children and their parents were assessed with a comprehensive assessment battery e.g. social- and neurocognitive tests and diagnostic interviews when the children were seven years old, and they will now be re-assessed for the first time at age 11. Data for this study is currently being collected as a part of the Via 11. Psychotic experiences will be assessed on the Scale of Prodromal Symptoms based on K-SADS interviews and with the Magical Thinking Questionnaire. Social cognitive skills will be assessed with Frith-Happé Animated Triangles and Theory-of-Mind Storybook Frederik. Cognitive bias i.e. jumping to conclusions will be assessed with the Beads task. Adverse life events will be assessed with the K-SADS interviews, the Child Trauma Screening Questionnaire, and with a questionnaire about bullying based on the Olweus Bully/Victim Questionnaire. Measures of neurocognitive and attentional deficits will also be included. Child attachment style was assessed with the Story Stem Assessment Protocol and emotion recognition with the ERT from Cantab.

**Hypotheses:**

-Age seven: Children in the two high risk groups will be have higher rates of insecure or disorganized attachment styles compared with children in the control group. We expect insecure and disorganized attachment to be associated with poorer social cognition (theory of mind and emotion recognition) and with worse general psychopathology and PEs.

-Age 11: We expect children born to parents with schizophrenia spectrum disorders to report higher frequencies of PEs than children born to parents without these disorders. We expect children with PEs to have higher levels of general psychopathology and poorer levels of daily functioning than children without PEs. We expect children in the two high risk groups to have poorer theory of mind than children in the control group.

-Age 11: We expect PEs to be associated with poor social cognition, particularly hyper theory-of-mind, higher rates of cognitive bias, adverse life events, neurocognitive and attentional impairments, and to be predicted by insecure and disorganized attachment styles.

**Results:** The data collection started in March 2017. Results from the 11-year-follow-up are expected in 2020.

**Discussion:** Examining PEs over time during childhood is important since it may improve our ability to identify children who are at a particularly high risk of developing psychotic disorders and other psychopathology later in life and thus to identify a particularly vulnerable subgroup towards whom early interventions should be targeted.

### S35. NEURORADIOLOGICAL FINDINGS IN CHILD AND ADOLESCENT PATIENTS WITH PSYCHOTIC DISORDERS

Justo Pinzón Espinosa*[,1], Adriana Fortea[1], Laura Espinosa[1], Anna Giménez[1], Gerard Anmella[1], Immaculada Baeza[2], Gisela Sugranyes[2]

*[1]Hospital Clinic of Barcelona; [2]Hospital Clínic of Barcelona, IDIBAPS*

**Background:** A 22 - 31% prevalence of abnormal radiological findings (RF) has been reported among patients with first episode of psychosis (FEP), ranging from clinically non-significant findings to overt neurological pathology. While one study (Borgwardt et al., 2006) found a higher proportion of RF in adult subjects at high risk for psychosis (35%) and FEP patients (40%) than in patients with depression (18%) or healthy controls (12%), another found a similar increase in RF in patients with affective and psychotic disorders (Landin et al, 2016). This suggests that macroscopic brain anomalies may be characteristic of at least a subset of patients in the early stages of psychosis, and these RF may not be specific to schizophrenia, but also to psychosis with affective symptoms.

To this day, all published research studies have been done in primarily adult samples. Psychosis with onset before age 18 may be associated with more salient biological features linked with greater genetic load and neurodevelopmental antecedents (Arango, 2014).

**Aims:** To assess the prevalence of neuroradiological abnormalities in a population with early-onset psychosis (EOP) in comparison to a sample of community controls, and to evaluate the association of these findings with the type of psychotic disorder of the patients.

**Methods:** Design: Naturalistic, observational, retrospective, single-center controlled study.

A chart review of individuals admitted to the Inpatient unit of the Dept. of Child and Adolescent Psychiatry and Psychology from January 2013 to December 2016 was done. Patients were 6 to 17 years old, fulfilled DSM-IV-TR criteria for a psychotic disorder (PD), and had a radiology report of a brain MRI. The community control (CC) group had a similar age and gender distribution and no current diagnosis of any psychotic disorder. Any neurological or severe medical condition or head trauma with loss of consciousness were exclusion criteria for both groups.

Sociodemographic, clinical, and radiological variables were recorded for both groups. Given the association of abnormal RF with prematurity, perinatal complications and neurodevelopmental disorders, these data were collected and sorted dichotomously.

Descriptive statistical analysis consisted of a means and standard deviation for quantitative variables and percentages for qualitative variables. Between-group differences were calculated with chi-square test or Fisher's test using IBM SPSS v23.

**Results:** A total of 191 individuals were included (127 PD vs 64 CC, mean ages: 14.7 ± 1.8 vs 13.8 ± 2.3, t=3.0, p=.01; %females: 55.9.0% vs 60.9%, $\chi 2$=.44, p=.50). Main diagnoses in PD were psychosis not otherwise specified (PNOS) (59.1%), schizoaffective disorder (SAD) (12.6%), schizophrenia (SCZ) (11.0%), bipolar disorder (BD) (8.7%) and major depression with psychotic features (MDD) (8.7%).

The PD group presented with a significantly higher prevalence of qualitative neuroimaging abnormalities in comparison to CC (21.3% (n=27) vs 6.2% (n=4), $\chi 2$=7.1, p=.008). These included arachnoid cysts, dilated perivascular space or white matter intensity anomalies. The prevalence of abnormal RF was 25.3% in PNOS, 21.4% in SCZ, 18.2% in MDD 12.5% in SAD and 9.1% in BD.

**Discussion:** A significantly higher prevalence of RF was found in youth with both affective and non-affective psychosis compared to similar-aged controls, concurring with some (Borgwardt et al., 2006; Landin et al., 2016), yet not with other (Sommer et al., 2013) observations in adult samples. These findings may reflect an impact of subtle biological alterations associated with psychosis on brain development, which may be more salient in early-onset cases. Our data highlight the need to continue assessing the significance of abnormal RF in patients with EOP

### S36. DIFFERENTIAL ENCODING OF SENSITIZATION AND CROSS SENSITIZATION TO PSYCHOSTIMULANTS AND ANTIPSYCHOTICS IN NUCLEUS ACCUMBENS D1- AND D2-RECEPTOR EXPRESSING MEDIUM SPINY NEURONS

Davide Amato*[,1], Jasper Heinsbroek[1], Peter W. Kalivas[1]

*[1]Medical University of South Carolina*

**Background:** Nearly half of all individuals diagnosed with schizophrenia abuse addictive substances such as cocaine. Currently, the neurobiological mechanisms in patients with schizophrenia that lead to cocaine abuse are unknown. A possible explanation for the co-morbidity between schizophrenia and addiction is that the rewarding properties of cocaine reverse the diminished motivational drive caused by chronic antipsychotic regimen. Moreover, chronic antipsychotic treatment can sensitize and amplify cocaine rewarding effects and exacerbate psychoses.

**Methods:** The rewarding properties of cocaine are attributed to the differential effects of dopamine on D1 and D2 receptor-expressing medium spiny neurons (MSNs) in the nucleus accumbens (NAc). Using in vivo Ca2+ miniature microscopic imaging, we characterize the role of D1 and D2 MSN in mono- and a cross- sensitization paradigms. D1- and D2-Cre mice were injected with a Cre dependent calcium indicator (gCaMP6f) and implanted with a gradient index (GRIN) lens above the nucleus accumbens and calcium activity was recorded using a head mounted miniature microscope. Cocaine sensitization was measured after a classic repeated cocaine regiment and antipsychotic and psychostimulant cross-sensitization was measured by a single cocaine injection after chronic pre-treatment with haloperidol.

**Results:** We found that both D1-MSN and D2-MSN populations are modulated by initial cocaine experience and further modulated during the expression of cocaine sensitization. A subpopulation of D1-MSN displayed initial activation, but reduced activity during the expression of sensitization. By contrast, the majority of D2-MSNs were suppressed by initial cocaine experience, but became active during the expression of sensitization. Furthermore, activity of D1- and D2-MSNs bidirectionally related with the observed behavioral responses to cocaine. Cross-sensitization following haloperidol treatment led to increased behavioral responses to psychostimulants. Current experiments are set out to investigate the neuronal responses of D1 and D2-MSN during cross sensitization between haloperidol and cocaine.

**Discussion:** Cocaine sensitization leads to differential neuronal responses in D1- and D2-MSN and these responses are differentially correlated with the magnitude of the sensitized behavioral response. These results reveal important new insights in the neurobiological processes in the nucleus accumbens that underlie psychostimulant sensitization and provide an important new model for studying the pharmacology of antipsychotic effects on striatal function and its potential role in increasing the susceptibility of schizophrenic patients to developing drug addiction.

### S37. STATE-DEPENDENT EFFECTS OF D2 PARTIAL AGONIST ARIPIPRAZOLE ON DOPAMINE NEURON ACTIVITY IN THE MAM NEURODEVELOPMENTAL MODEL OF SCHIZOPHRENIA

Susan Sonnenschein*[,1], Kathryn Gill[1], Anthony Grace[1]
[1]*University of Pittsburgh*

**Background:** Aripiprazole is an antipsychotic drug characterized by partial agonist activity at D2 receptors that impacts both hyperdopaminergic and hypodopaminergic states. It is unclear whether aripiprazole reduces dopamine neuron activity via inhibition or by excitation-induced depolarization block, the latter being characteristic of D2 antagonist administration, and how aripiprazole interacts with D2 antagonist-induced reduction in dopamine neuron activity.

**Methods:** Adult offspring of saline and MAM-treated rats received aripiprazole (10 mg/kg), or vehicle, p.o. and dopamine neuron activity was examined 2h following acute treatment, or after 1d or 7d withdrawal from 21d repeated treatment. Dopamine neuron activity in the VTA was measured using in vivo extracellular recordings from anesthetized rats. After electrophysiological sampling, apomorphine (200 µg/kg i.p. or 20 µg/kg i.v.) was administered, followed by resampling the VTA to test for the presence of depolarization block. Additional recordings were conducted in MAM rats 1 h following acute haloperidol treatment (0.6 mg/kg, i.p). After electrophysiological sampling, aripiprazole (1mg/kg, i.p.) was administered to examine its effect on haloperidol-induced depolarization block.

**Results:** Both acute and repeated administration of aripiprazole reversed the increased number of spontaneously active dopamine neurons in MAM rats without impacting control rats. The reduction in dopamine neuron activity persisted after 7d withdrawal from repeated aripiprazole treatment and was not impacted by administration of apomorphine. In contrast, aripiprazole increased dopamine neuron activity in haloperidol-treated MAM rats.

**Discussion:** This study establishes that aripiprazole rapidly reduces hyperdopaminergic activity in MAM rats, without impacting dopamine neuron population activity in normal rats. The reduction is not due to depolarization block and persists 1 week following withdrawal from repeated treatment. Aripiprazole also removes haloperidol-induced depolarization block in MAM rats, which may underlie the acute psychotic symptoms observed clinically following the switch from D2 antagonist to aripiprazole treatment.

### S38. CHARACTERISING THE COGNITIVE CONSEQUENCES OF DISRUPTED BDNF-TRKB SIGNALLING AT PARVALBUMIN-EXPRESSING INTERNEURONS

Adrienne Grech*[,1], Xin Du[1], Rachel Hill[1]
[1]*Monash University*

**Background:** Schizophrenia is a debilitating syndrome characterised by three main symptom categories: positive, negative and cognitive. Cognitive symptoms emerge first, and currently do not have appropriate treatments, despite being a strong predictor of the severity and progress of the illness. Cognitive deficits are thought to be partly attributed to impaired synchronization of gamma frequency oscillatory activity. Gamma oscillations are generated by a subclass of GABAergic interneuron that expresses the calcium binding protein, parvalbumin (PV). PV-interneurons are supported by Brain Derived Neurotrophic Factor (BDNF) and recent evidence has found that cessation of BDNF support in PV- interneurons impairs gamma oscillations. All of these factors have been demonstrated to have a role in cognitive processing, but their dynamic relationship is not completely understood.

**Methods:** The aims of this study were: 1) To generate transgenic mice where 50% of BDNF receptor (TrkB) gene is excised from PV-expressing neurons using the cre-lox recombination system and 2) To investigate the cognitive and behavioural consequences of disrupted BDNF signalling at inhibitory PV-expressing interneurons. Male and female mice underwent a battery of tests including: Y-Maze, Novel Object Recognition Task (NORT), Elevated Plus Maze, Locomotor and Cheeseboard Maze.

**Results:** Sex-specific spatial memory impairments were found in PV-Cre x TrkB floxed mice with only males showing no preference for the novel arm in the Y-maze paradigm. Furthermore, male PV-Cre x TrkB floxed mice displayed a lack of cognitive flexibility in the cheeseboard maze for long term spatial memory. No significant differences were observed in measures of anxiety and activity, indicating that these were not confounding variables for cognitive measures.

**Discussion:** This mouse line has not been cognitively characterised before and the results are of major interest. Subtle changes to cognition were observed and were sex-dependent. Interestingly, only males were observed to have changes in cognition, in line with human data. Human males with schizophrenia tend to exhibit more severe cognitive symptoms. Overall, the evidence from this study supports a role for BDNF-TrkB signalling at PV interneurons in regulating spatial memory performance. Future work will be investigating spatial search strategies of the Cheeseboard Maze, in order to elucidate further any cognitive differences between the genotypes. Additionally, future work will aim to specifically disrupt BDNF-TrkB signalling in the hippocampus and/or prefrontal cortex, as these two areas are highly implicated in both cognition and schizophrenia. It would also be of interest to use this genotype in a two-hit model, to further investigate the interaction of multiple factors and their impact on cognitive functions.

### S39. GPR139 AN OPHAN GPCR AFFECTING NEGATIVE DOMAINS OF SCHIZOPHRENIA

Joy Atienza*,[1], Holly Reichard[1], Victoria Mulligan[1], Jackie Cilia[1], Holger Monenschein[1], Deanna Collia[1], Jim Ray[1], Gavin Kilpatrick[1], Nicola Brice[1], Mark Carlton[1], Steve Hitchcock[1], Ged Corbett[1]
[1]*Takeda*

**Background:** Individuals with schizophrenia fail to appropriately use negative feedback to guide learning. These learning deficits are thought to arise from abnormalities in midbrain dopamine activity. The habenula is a well conserved paired structure that sits in the midline, adjacent to the third ventricle, and dorsal and posterior to the thalamus. Classic studies have shown that it is part of the reward pathway and functions with dopamine neurons in the ventral tegmental area (VTA) to mediate reward related signals, specifically aversive and negative stimulus (Hikosaka, 2010). Several studies point to pathology in the habenula as contributing to schizophrenia (Sandyk, 1992; Caputo et al., 1998; Shepard et al., 2006). Despite the many studies on the habenula, the precise function of the habenula remains unclear. Here we describe functional consequences of regulation of GPR139 an orphan GPCR that is specifically expressed in the CNS and enriched in the habenula (Matsuo et al., 2005) in mouse models of domain of schizophrenia.
**Methods:** Specific expression of mouse GPR139 in the habenula was evaluated using bacterial artificial chromosome translating ribosome affinity purification (bacTRAP) and confirmed by immunohistochemistry. GPR139-/- mice were generated by removal of a 736bp region encoding the seven transmembrane domain (7TM) domain. Knockout animals were maintained inbred on a 129/SvEv background and evaluated in behavioral models that reflect aspects of negative symptoms. High throughput screen (HTS) for small molecules was performed. We expressed full length GPR139 into CHO cells and screened a 600K library using a calcium assay to identify small molecule agonist. Hits were identified and physical properties optimized to produce a molecule suitable for in vivo evaluation. GPR139 agonist was testedin vivo in social interaction in Poly(I:C) and cognitive test in subchronic PCP, models of schizophrenia.
**Results:** Using bacTRAP we observed enriched expression of GPR139 in substance P positive- cells of the medial habenula. This expression was confirmed with immunohistochemistry. To determine if GPR139 regulates the known role of the habenula in learning, motivation, and social behaviour, GPR139 -/- animals were generated and phenotyped. These animals appeared normal and performed comparably to wild-type animals in a range of standard tasks. However, they were significantly impaired in models that reflect aspects of negative symptoms such as progressive ratio (Killeen et al., 2009; Heath et al., 2016), a measure of motivation and nest-building (Pedersen et al., 2014; Nakajima et al., 2013), a model of self-neglect. Furthermore, these animals showed deficits in novel object recognition model of working memory (Antunes and Biala, 2012). The small molecule agonist was observed to reverse deficits in models of schizophrenia including cognition in a subchronic PCP induced attentional set-shifting paradigm (Birrell and Brown, 2000) and social interaction in the Poly(I:C) maternal model (Meyer et al., 2008; Bitanihirwe et al., 2010).
**Discussion:** These findings identify an orphan receptor that plays an important role in habenular function. Modulation of this receptor may provide an opportunity to address an important unmet need in schizophrenia.

### S40. MEDIAL SEPTUM ACTIVATION PRODUCES OPPOSITE EFFECTS ON DOPAMINE NEURON ACTIVITY IN THE VENTRAL TEGMENTAL AREA AND SUBSTANTIA NIGRA PARS COMPACTA IN MAM VERSUS NORMAL RATS

David Bortz*,[1], Anthony Grace[1]
[1]*University of Pittsburgh*

**Background:** Disruptions in dopamine (DA) signaling are central to the pathophysiology of several major psychiatric disorders, including schizophrenia. Thus, discovery of novel therapeutic approaches that normalize DA signaling is a major focus of research. One pathway that is critical for DA regulation originates from the ventral subiculum (vSub) of the hippocampus and controls ventral tegmental area (VTA) DA neuron activity. A potent regulator of hippocampal function is the medial septum (MS); a sub-region of the basal forebrain that widely innervates the hippocampus, including the vSub, via cholinergic, GABAergic, and glutamatergic projections, drives hippocampal theta rhythms, and affects goal-directed learning and memory. Despite this, it has never been determined if the MS is an afferent regulator of the midbrain DA system, and therefore may be a novel therapeutic treatment target for DA-related disorders.
**Methods:** Effects of MS activation (NMDA, 0.75 µg/ 0.2 µL) were examined in intact and methylazoxymethanol acetate- treated (MAM) male Sprague-Dawley rats using anesthetized single unit DA recordings in the VTA and substantia nigra pars compacta (SNc) and locomotor behavior in an open field following systemic amphetamine (0.75 mg/kg).
**Results:** MS activation produced a prolonged 71% increase in the number of spontaneously active DA cells in the VTA, and an opposing 40% decrease in the number of active DA cells in the SNc, compared to vehicle infusions. These effects were mediated by the vSub and ventral pallidum as local infusion of TTX (1 µM /0.5 µL) and bicuculline (1 ng/ 0.5 µL), respectively, reversed DA population activity changes in both regions. MS activation also decreased the locomotor response to amphetamine (49% reduction in distance traveled during peak ambulation compared to vehicle). MS activation-induced changes in both DA population activity and amphetamine-induced hyperlocomotion were selectively mediated by different neurotransmitter populations from MS to vSub as infusion of scopolamine (8 µg/1.0 µL) into the vSub selectively prevented DA population activity changes in the VTA. In contrast, infusion of bicuculline (12.5 ng/0.5 µL) selectively prevented DA population activity changes in the SNc and the decrease in amphetamine-induced hyperlocomotion. In MAM rats, MS activation produced opposite effects on DA population activity versus controls as it decreased VTA DA activity by 51% and increased SNc DA activity by 47%. This was accompanied by a similar reversal in amphetamine-induced hyperlocomotion, with MS activation increasing locomotion in MAM animals (113% increase in distance traveled during peak ambulation compared to control animals). The reversal in behavioral output is likely due to disrupted GABAergic projections from MS to vSub as bicuculline infusion into vSub prevented the increase in locomotor behavior.
**Discussion:** These data indicate that the MS differentially regulates both VTA and SNc DA neuron activity and behavioral output, but via distinct pathways, and that this regulation is disrupted in a well-validated animal model of schizophrenia. Therefore, this suggests that in normal animals the MS might activate the VTA to increase information processing while delaying action via SNc inhibition. In contrast, the inhibition of VTA and activation of SNc in MAM rats might promote a rapid response without adequate processing of information.

### S41. SHORT- AND LONG-TERM OUTCOMES OF VITAMIN D SUPPLEMENTATION IN CLOZAPINE TREATED CHRONIC SCHIZOPHRENIA PATIENTS

Amir Krivoy*,[1], Roy Onn[1], Yael Vilner[1], Zvika Friedman[1], Abraham Weizman[1]
[1]*Geha Metal Health Center*

**Background:** Vitamin D deficiency is highly prevalent in patients with psychosis. While accumulating data suggests that vitamin D deficiency may be involved in the clinical and metabolic outcomes of schizophrenia, there are no vitamin D supplementation studies in this context. Objective: To assess the short- and long-term impact of vitamin D supplementation on psychiatric and metabolic status in chronic clozapine-treated schizophrenia patients.

**Methods:** Following a first phase of eight-week, randomized, double blind, placebo-controlled clinical trial, in which schizophrenia patients who had been maintained on clozapine treatment for at least 18 weeks with total PANSS scores >70 and with low levels of vitamin D (<75 nmol/L) were recruited, a second phase, post-RCT, was performed. In the RCT patients were randomly allocated to either weekly oral drops of vitamin D (14,000 IU) or placebo and subsequently assessed at two-week intervals regarding psychosis severity, mood, cognition and metabolic status. In the post-RCt phase, all participants were assessed at 24 weeks' time point, while being prescribed 800 IU vitamin D daily supplementation in an open-label design.

**Results:** Twenty-four patients were randomly assigned to vitamin D and the other 23 patients to the placebo arm. No between-group differences were found in baseline measures. After eight weeks, the vitamin D group had higher increase in vitamin D levels (31.4 vs -0.4 nM, p<0.0001). There was no significant effect of vitamin D on PANSS score, depression or metabolic parameters. The vitamin D group, however, showed a positive small effect on cognitive function (effect size=0.17). On the long-term follow up, 37 patients completed the 24-week assessment. Mean vitamin D levels did not change from baseline (57.56 nM to 57.28 nM) and no association was found between this change and the changes in psychiatric, metabolic and cognitive measures. The only significant inverse association was found between vitamin D levels and HDL (p=0.007).

**Discussion:** This is the first study to assess the outcomes of vitamin D as an adjunct to clozapine in chronic schizophrenia patients, in the short and long term. The increase in vitamin D levels on the short-term was associated with a small positive effect on cognition, without any effect on psychosis, mood or metabolic status. There were no significant effects of vitamin D supplementation on the assessed measures in the long-term either.

### S42. KETAMINE DYSREGULATES TASK-RELATED NEURAL OSCILLATIONS IN THALAMO-CORTICAL CIRCUITS: IMPLICATIONS FOR PATHOPHYSIOLOGICAL THEORIES OF VISUAL-PERCEPTUAL DEFICITS IN SCHIZOPHRENIA

Tineke Grent-'t-Jong*[,1], Davide Rivolta[2], Joachim Gross[1], Ruchika Gajwani[3], Stephen Lawrie[4], Matthias Schwannauer[4], Tonio Heidegger[5], Michael Wibral[5], Wolf Singer[6], Andreas Sauer[6], Bertram Scheller[5], Peter Uhlhaas[1]

*[1]University of Glasgow, Institute of Neuroscience and Psychology; [2]School of Psychology, University of East London; [3]Institute of Health and Wellbeing, University of Glasgow; [4]University of Edinburgh; [5]Goethe University; [6]Max-Planck Institute for Brain Research*

**Background:** Hypofunction of the N-methyl-D-aspartate receptor (NMDA-R) has been implicated as a possible mechanism underlying cognitive deficits and aberrant neuronal dynamics in schizophrenia (ScZ).

**Methods:** In a single-blind cross-over design, 14 participants received either a subanaesthetic dose of S-ketamine (0.006 mg/Kg) or saline while Magnetoencephalographic (MEG) data were recorded during a visual task. In addition, MEG-data were obtained in a sample of unmedicated first-episode psychosis (FEP) patients (n = 10) and in patients with chronic ScZ (n = 16). MEG-data were analyzed at source-level in the 1–90 Hz frequency range in occipital and thalamic regions of interest (ROIs). In addition, directed functional connectivity analysis was performed using Granger Causality (GC). Psychopathology was assessed by the Positive and Negative Syndrome Scale (PANSS).

**Results:** Behavioral impairments were accompanied by increased amplitude and frequency of gamma-power (63–80 Hz) in occipital regions during Ketamine administration, while low-frequency (~5–30 Hz) activity was upregulated. Moreover, Ketamine disrupted feedforward (FF) and feedback (FB) signaling at high and low frequencies leading to hypoconnectivity in thalamo-cortical (TC) networks. In contrast, FEP and chronic ScZ patients showed a different pattern of MEG-activity, characterized by decreased task-induced high-gamma band oscillations and increased FF/FB-mediated GC-connectivity.

**Discussion:** The effects of Ketamine on high-frequency oscillations and their connectivity profile are not consistent with observations in FEP and chronic ScZ-patients. Accordingly, the current data have implications for theories of cognitive dysfunctions and circuit impairments in the disorder, suggesting that the effects of acute NMDA-R hypofunction are not consistent with impairments in visuo-perceptual oscillations in ScZ-patients.

### S43. A PROOF-OF-MECHANISM STUDY OF THE PDE10 INHIBITOR RG7203 IN PATIENTS WITH PROBING REWARD FUNCTIONS WITH IMAGING AND BEHAVIORAL APPROACHES

Daniel Umbricht*[,1], Jürgen Dukart[1], Markus Abt[1], Paul Tamburri[1], Chris Chatham[1], Michael Frank[2], Anne Collins[2], David Walling[3], Rick Mofsen[4], Daniel Gruener[4], Lev Gertsik[8], Jeffrey Sevigny[3]

*[1]F. Hoffman - La Roche Ltd.; [2]Brown University; [3]CNS Network, LLC; [4]St. Louis Clinical Trials; [5]CCT*

**Background:** The enzyme phosphodiesterase 10A (PDE10A) is highly expressed in the striatum where it modulates both dopamine D2 and D1 dependent signaling. Its inhibition leads to a suppression of D2 mediated signaling –similar to effects of D2 antagonists - and an enhancement of D1 dependent signaling. D1-dependent signaling has been implicated in reward based learning. Its deficient activation may be a key factor underlying deficient reward functions including reward anticipation and reward based learning that have been implicated as major drivers of negative symptoms of schizophrenia. Therefore, inhibition of PDE10 could be a way to ameliorate such deficits and consequently negative symptoms. In healthy volunteers the PDE10 inhibitor RG7203 indeed enhanced performance in tasks that probed reward functioning suggestive of its potential utility to treat negative symptoms in schizophrenia. We therefore tested the hypothesis that it should enhance imaging and behavioral markers of reward functions in patients with moderate negative symptoms in order to establish mechanistic proof of its utility as treatment of negative symptoms.

**Methods:** In a three-way cross-over study we investigated the effects of two doses of RG7203 (5 mg and 15 mg) and placebo given as adjunctive treatment to stable background antipsychotic treatment on reward functioning and reward-based effortful behavior using the monetary incentive delay (MID) task during fMRI and the effort choice task in patients with chronic schizophrenia and moderate levels of negative symptoms (PANSS negative symptom factor score ≥ 18 points). Each treatment period lasted three weeks followed by a 2 week washout period. fMRI and behavioral tasks were administered at the end of each treatment period. Key outcome measures were the differential BOLD during reward anticipation and overall BOLD activity during the MID task and the percentage of high-effort high-reward choices when the probability of reward was 100% during the effort choice task.

**Results:** Thirty-three patients with schizophrenia (30 male; 21 B, 9 W, 3 A; mean age 36.6 ± 7 y; PANSS NSFS = 22.8 (±1.4) at screening) were recruited at three study centers in the US. Twenty-four subjects finished the entire study. RG7203 at 5 mg significantly increased differential BOLD activity during reward anticipation in the MID task. However, this enhancement occurred in the context of a significant decrease of BOLD activity across all conditions during the MID task under treatment with RG7203. RG7203 significantly worsened reward-based effortful behavior in the effort choice task (the high-effort high-reward choice: 67% for both doses of RG7203 versus 73% for placebo). Multiple regression revealed that the decrease in effortful behavior was significantly related to the decrease in overall BOLD

activity during the MID task and not related to the difference of BOLD activity during reward anticipation versus the control condition.
**Discussion:** In contrast to our expectation and previous results in healthy volunteers, RG7203 worsened indices of reward functions which we hypothesize may be due to a further enhancement of D2 antagonistic activity. The results do not support the utility of a PDE10 inhibitor as adjunctive treatment for negative symptoms in patients with schizophrenia. Given the previous observation that RG7203 enhanced reward functions in healthy volunteers who were not treated with D2 antagonist, the results of our study point to potentially deleterious effects of D2 blockade on reward functions and by extension on negative symptoms of schizophrenia. They raise the question if the presence of D2 antagonistic treatment curtails the potential effects of any adjunctive treatment for negative symptoms.

### S44. LUMATEPERONE (ITI-007) FOR THE TREATMENT OF SCHIZOPHRENIA: PLACEBO-CONTROLLED CLINICAL TRIALS AND AN OPEN-LABEL SAFETY SWITCHING STUDY

Kimberly Vanover*[,1], Alex Dmitrienko[2], Steven Glass[1], Susan Kozauer[1], Jelena Saillard[1], Michal Weingart[1], Andrew Satlin[1], Sharon Mates[1], Christoph Correll[3], Robert Davis[1]
*[1]Intra-Cellular Therapies, Inc.; [2]Mediana Research; [3]The Zucker Hillside Hospital, Hofstra North Shore LIJ School of Medicine*

**Background:** Lumateperone (ITI-007) is a first-in-class investigational agent in development for the treatment of schizophrenia. Acting synergistically through serotonergic, dopaminergic and glutamatergic systems, lumateperone represents a new approach to the treatment of schizophrenia and other neuropsychiatric disorders. Lumateperone is a potent antagonist at 5-HT2A receptors and exhibits serotonin reuptake inhibition. Lumateperone also binds to dopamine D1 and D2 receptors acting as a mesolimbic/mesocortical dopamine phosphoprotein modulator (DPPM) with pre-synaptic partial agonism and post-synaptic antagonism at D2 receptors and as an indirect glutamatergic (GluN2B) phosphoprotein modulator with D1-dependent enhancement of both NMDA and AMPA currents via the mTOR protein pathway.
**Methods:** Lumateperone was evaluated in 3 controlled clinical trials to evaluate efficacy in patients with acute schizophrenia. In Study ITI-007-005, 335 patients were randomized equally across 4 treatment arms: one of two doses of lumateperone, risperidone (active control) or placebo QAM for 4 weeks. In Study ITI-007-301, 450 patients were randomized equally across 3 treatment arms: one of two doses of lumateperone or placebo QAM for 4 weeks. In Study ITI-007-302, 696 patients were randomized equally across 4 treatment arms: one of two doses of lumateperone, risperidone (active control) or placebo QAM for 6 weeks. In all 3 studies, the primary endpoint was change from baseline on the Positive and Negative Syndrome Scale (PANSS) total score compared to placebo. Also, a 6-week open-label safety switching study was conducted. In this ITI-007-303 study 302 patients with stable schizophrenia were switched from standard-of-care (SOC) antipsychotics and treated for 6 weeks with lumateperone QPM outpatient and then switched back to SOC for 2 weeks.
**Results:** Two of the 3 randomized studies were positive. In Studies ITI-007-005 and ITI-007-301, lumateperone (60 mg ITI-007, equivalent to 42 mg active base) met the primary endpoint with statistically significant superior efficacy over placebo at Day 28 as measured by the PANSS total score (Study ITI-007-005 $p=0.017$; Study ITI-007-301 $p=0.022$). In Study ITI-007-302, neither dose of lumateperone separated from placebo on the primary endpoint in the intent-to-treat population; a high placebo response was observed in this study. Across all 3 efficacy trials, lumateperone improved symptoms of schizophrenia with the same trajectory and same magnitude of improvement from baseline on the PANSS total score.
Lumateperone was well-tolerated with a favorable safety profile in all studies. In the two studies with risperidone included as an active control, lumateperone was statistically significantly better than risperidone on key safety and tolerability measures including prolactin, glucose, lipids and weight. In the open-label safety switching study statistically significant improvements from SOC were observed in body weight, cardiometabolic and endocrine parameters worsened again when switched back to SOC medication. In this open-label study, symptoms of schizophrenia generally remained stable or improved. Greater improvements were observed in subgroups of patients with elevated symptomatology such as those with comorbid symptoms of depression and those with prominent negative symptoms.
**Discussion:** Lumateperone represents a novel approach to the treatment of schizophrenia with a favorable safety profile in clinical trials. The lack of metabolic, motor and cardiovascular safety issues presents a safety profile differentiated from standard-of-care antipsychotic therapy.

### S45. MENTALIZATION BASED TREATMENT FOR NON-AFFECTIVE PSYCHOTIC DISORDER

Jonas Weijers*[,1], Corine ten Kate[2], Elisabeth Eurelings-Bontekoe[3], Wolfgang Viechtbauer[4], Jean-Paul Selten[5]
*[1]GGZ Leiden; [2]GGZ Rivierduinen; [3]Leiden University; [4]Maastricht University; [5]Maastricht University, Rivierduinen Mental Health Care Institute*

**Background:** Deficits in mentalizing – i.e. the ability to understand one's own and another's behavior in terms of mental states such as beliefs, feelings and intentions – have been widely observed in patients with non-affective psychotic disorder (NAPD). In turn, robust evidence has shown these impairments to be related to social dysfunction and negative symptoms. However, few treatments have been developed to effectively treat impaired mentalizing, in spite of its increased recognition as an important treatment target. Mentalization based treatment (MBT) is a psychodynamic therapy rooted in attachment theory, originally developed and empirically found to be effective in treating borderline personality disorder. MBT for psychotic disorder aims to improve social functioning in NAPD patients by targeting impaired mentalizing.
**Methods:** The study is a multicenter, rater-blinded, randomized controlled trial. Ninety patients, who were diagnosed with NAPD by a psychiatrist and whose diagnosis was confirmed by researchers with the CASH, were recruited from community treatment teams in the Netherlands. They were randomly allocated to either treatment as usual plus MBT or to treatment as usual only. MBT consisted of 18 months of group therapy (one hour weekly) and individual therapy (30 minutes per two weeks). Patients had a mean age of 31.48 years (SD = 8.87) and a mean duration since onset of psychosis of 5.53 years (SD = 3.65). The primary outcome variable was social functioning (measured with the Social Functioning Scale). Other outcome variables were positive, negative, depressive, and anxious symptoms, as well as insight (PANSS), quality of life (MANSA), substance abuse, social stress reactivity (Experience Sampling Method), and mentalizing capacity (Social Cognition and Object-Relations System; Hinting Task).
**Results:** This will be the first presentation of our trial results.
**Discussion:** The clinical implications of the results and limitations of the trial will be discussed. Theoretical considerations suggest that mentalization-based treatment could be an effective treatment for social dysfunction and impaired mentalizing in NAPD. If Mentalization-based treatment for psychotic disorders proves to be effective at improving social functioning and mentalizing, it may provide a valuable addition to treatment as usual.

### S46. THE VALIDITY AND SENSITIVITY OF PANSS-6 IN TREATMENT-RESISTANT SCHIZOPHRENIA

Soren Dinesen Ostergaard*[,1], Leslie Foldager[2], Ole Mors[1], Per Bech[3], Christoph U. Correll[4]

[1]Aarhus University Hospital; [2]Aarhus University; [3]Psychiatric Centre North Zealand, Copenhagen University Hospital; [4]The Zucker Hillside Hospital, Northwell Health

**Background:** A six-item version (PANSS-6: P1=Delusions, P2=Conceptual disorganization, P3=Hallucinations, N1=Blunted Affect, N4=Social withdrawal, N6=Lack of spontaneity/flow of conversation) of the 30-item Positive and Negative Syndrome Scale (PANSS-30) has shown promise in the measurement of symptom severity in acutely exacerbated- and chronic schizophrenia, but its validity in treatment-resistant schizophrenia remains unknown. Therefore, we tested the validity and sensitivity of PANSS-6 based on data from the clozapine phase of the Clinical Antipsychotic Trials of Intervention Effectiveness (CATIE) study.
**Methods:** I) The scalability of PANSS-6 and PANSS-30 (i.e., whether all items provide unique information regarding syndrome severity) was tested by means of item response theory analysis ad modum Rasch; II) The correlation between PANSS-6 and PANSS-30 total scores was investigated by means of Spearman correlation analysis; III) The accuracy of PANSS-6 in identifying symptom remission was tested by comparing remission on PANSS-6 (score of ≤3 on each of the six PANSS-6 items) with remission according to the Andreasen criteria (score of ≤3 on the 8 PANSS items considered in the Andreasen criteria); and IV) The antipsychotic effect of clozapine was compared to that of olanzapine, risperidone and quetiapine using the "speed of change" on PANSS-6 and PANSS-30 (change in total score per day) as outcomes.
**Results:** We found that I) only PANSS-6 and not PANSS-30 was scalable; II) The correlation between PANSS-6 and PANSS-30 total scores was high (Spearman coefficient: 0.85), III) PANSS-6 did accurately classify syndrome remission as defined by the Andreasen criteria, and IV) The only antipsychotic that resulted in improvement (speed of change significantly lower than 0 during the first three months of treatment) was clozapine, both when using PANSS-6 (speed of change: -0.072 points/day; 95%CI: -0.121, -0.024) and when using PANSS-30 (speed of change: -0.201 points/day; 95%CI: -0.400, -0.002) as outcome measures.
**Discussion:** These findings suggest that PANSS-6 validly measures severity, remission and antipsychotic efficacy in treatment-resistant schizophrenia.

### S47. ADD-ON SPIRONOLACTONE FOR THE TREATMENT OF SCHIZOPHRENIA (SPIRO TREAT)

Astrid Roeh*,[1], Stefan Leucht[2], Peter Falkai[1], Berthold Langguth[3], Irina Papazova[1], Alkomiet Hasan[1]
[1]Ludwig Maximilian University; [2]Technical University of Munich; [3]University of Regensburg

**Background:** Patients with schizophrenia often display three main types of symptoms: positive symptoms (e.g. auditory hallucinations or delusions), negative symptoms (e.g. blunted affect, lack or decline in speech, social withdrawal) and cognitive symptoms (e.g. impairment of working memory and declarative memory). Treatment of positive symptoms with available antipsychotics is well established, while therapy options for cognitive and negative symptoms are lacking. Neurobiological studies identified an enhanced NRG1-ERBB4 signaling as a risk pathway in schizophrenia. Spironolactone was found to function as an inhibitor of the ERBB4 receptor. In Nrg1 type III transgenic mice, spironolactone treatment led to an improvement of schizophrenia-like symptoms (Wehr et al. 2017). This is the first study to investigate an add-on spironolactone treatment in schizophrenia patients for the treatment of cognitive deficits.
**Methods:** This is a multicenter, randomized, double-blind, parallel (3-groups), longitudinal pilot study including 3 x 27 (81) patients with a clinically stable schizophrenia. Patients are randomized in three groups: one group receives add-on spironolactone 100 mg for three weeks (intervention I), one group receives add-on spironolactone 200 mg for three weeks (intervention II) and one group receives add-on placebo for three weeks (control group). The primary endpoint is the modification of the working memory in dependence to the intervention as investigated with the n-back task (0-, 1- and 2-back). Secondary endpoints include: modification of other cognitive functions (e.g. declarative memory and attention), psychopathology (e.g. PANSS and CDSS), overall level of functioning via GAF and severity of disease via CGI, changes in the number of patients in remission using the Andreasen Criteria, modifications of the inhibitory cortical function in the context of the prepuls paradigm with TMS, evaluating possible dose-differences, spironolactone effects on mRNA levels in peripheral blood lymphocytes (PBMC measures). Statistical analysis of the primary endpoint will be based on the intention-to-treat (ITT) population including all randomised patients using a mixed model ANOVA.
**Results:** The first patient was recruited in July 2015. Currently 45 patients are included in the trial. The overall tolerance of the medication was satisfactory with 48 reported AE (adverse events). In 3 cases, the causality of the medication was definite, in 4 cases probable and in 5 cases possible. The other cases were probably or definitely not related to the study medication. SAE (serious adverse events) were not reported. The current state of research will be discussed on the conference.
**Discussion:** This is the first study to describe the effects of an add-on treatment of spironolactone in patients with a clinically stable schizophrenia. Also, it is the first study with a direct target of a biochemically disturbed signaling pathway in schizophrenia patients with a possibly new treatment option in this severe disease.
Funded by Stanley Foundation: (13T-004). EUDRACT: 2014-001968-35, WHO-Clinical-Trials: http://apps.who.int/trialsearch/Trial2.aspx?TrialID=EUCTR2014-001968-35-DE
**Reference:**
1. Wehr, M.C., Hinrichs, W., Brzózka, M.M., Unterbarnscheidt, T., Herholt, A., Wintgens, J.P., Papiol, S., Soto-Bernardini, M.C., Kravchenko, M., Zhang, M., et al. (2017). Spironolactone is an antagonist of NRG1-ERBB4 signaling and schizophrenia-relevant endophenotypes in mice. EMBO Mol. Med.

### S48. INTER-RATER RELIABILITY OF PANSS-6 SCHIZOPHRENIA SEVERITY RATINGS OBTAINED USING THE SIMPLIFIED NEGATIVE AND POSITIVE SYMPTOMS INTERVIEW (SNAPSI)

Pernille Kølbæk*,[1], Per Bech[2], Ole Mors[3], Christoph U. Correll[4], Søren D. Østergaard[3]
[1]Aarhus University Hospital; [2]Mental Health Centre North Zealand, University of Copenhagen; [3]Aarhus University Hospital, Aarhus University; [4]The Zucker Hillside Hospital

**Background:** Schizophrenia is a severe mental disorder requiring multimodal treatment. Monitoring the severity of schizophrenia during treatment is essential to a successful outcome. The most widely used measure of the severity of schizophrenia is the 30-item Positive And Negative Syndrome Scale (PANSS-30) obtained by the Structured Clinical Interview, SCI-PANSS, which takes approximately an hour to administer. This is too long for routine clinical use. Recently, our group extracted a 6-item scale (PANSS-6), which has shown promising psychometric properties. The scale consists of the following items: P1 - Delusions, P2 - Conceptual disorganization, P3 - Hallucinatory behavior, N1 - Blunted Affect, N4 - Passive/apathetic social withdrawal and N6 - Lack of spontaneity & flow of conversation. For now, it remains unknown whether it is possible to obtain sufficient information for PANSS-6 rating via a short and focused interview. Recently, our group developed an interview, the Simplified Negative and Positive Symptoms Interview (SNAPSI), which enables PANSS-6 rating. Field-testing at hospitals in the United States and Denmark has shown that the patient section of SNAPSI can be completed in approximately 15–25 minutes by raters who are unfamiliar with the interview and involving patients hearing the questions for the first time. The aim of the present study was to test the inter-rater reliability of PANSS-6 ratings obtained using the SNAPSI.
**Methods:** The team of raters (five medical doctors and two psychologists) attended training sessions prior to the inter-rater reliability test. At the training sessions one rater interviewed a patient with schizophrenia using the SNAPSI, while all raters conducted PANSS-6 ratings independently. After each interview the PANSS-6 ratings were discussed until an agreement was reached. Each rater participated in at least six SNAPSI/PANSS-6 training ratings.
For the inter-rater reliability test, a total of 12 patients with a primary diagnosis of schizophrenia, currently undergoing in- or outpatient treatment at the Department for Psychosis, Aarhus University Hospital – Denmark, will be recruited. The team of raters will perform a total of at least 50 PANSS-6 ratings via SNAPSI. All raters will conduct the SNAPSI at least once. As a measure of inter-rater reliability, we will calculate the Intraclass Correlation Coefficient based on the 50 PANSS-6 ratings.
**Results:** The results of the inter-rater reliability test will be available in January 2018 and presented at the SIRS 2018 conference.
**Discussion:** If the results of the inter-rater reliability test are satisfactory, we will conduct a clinical validation of PANSS-6. In this study we will test whether PANSS-6 ratings obtained using the SNAPSI correspond to PANSS-6 ratings extracted from independent PANSS-30 ratings obtained using the SCI-PANSS. If this is the case, PANSS-6 ratings obtained using the SNAPSI will facilitate valid measurement-based care of schizophrenia in clinical practice.

### S49. EFFICACY OF HIGH-FREQUENCY REPETITIVE TRANSCRANIAL MAGNETIC STIMULATION ON PANSS FACTORS IN SCHIZOPHRENIA WITH PREDOMINANT NEGATIVE SYMPTOMS – RESULTS FROM AN EXPLORATORY RE-ANALYSIS

Maximilian Hansbauer*,[1], Thomas Wobrock[2], Birgit Kunze[2], Berthold Langguth[3], Michael Landgrebe[3], Joachim Cordes[4], Wolfgang Wölwer[4], Georg Winterer[5], Wolfgang Gaebel[4], Göran Hajak[6], Christian Ohmann[7], Pablo Verde[4], Marcella Rietschel[8], Raees Ahmed[2], William Honer[9], Berend Malchow[1], Wolfgang Strube[1], Thomas Schneider-Axmann[1], Peter Falkai[1], Alkomiet Hasan[1]
*[1]Ludwig Maximilian University; [2]Universität Göttingen; [3]University of Regensburg; [4]Heinrich-Heine University [5]Charite – University Medicine Berlin; [6]Sozialstiftung Bamberg; [7]European Clinical Research Network (ECRIN); [8]Central Institute of Mental Health; [9]University of British Columbia*

**Background:** Repetitive transcranial magnetic stimulation (rTMS) applied to the left frontal lobe is discussed to be a promising add-on treatment for negative symptoms in schizophrenia. The Positive and Negative Syndrome Scale (PANSS) has been used as outcome parameter in several previous rTMS trials, but studies focusing on PANSS factor analyses are lacking.
For this purpose, we used the available PANSS data of the 'rTMS for the Treatment of Negative Symptoms in Schizophrenia' (RESIS) trial to calculate different literature-based PANSS factors and to re-evaluate the impact of rTMS on negative symptoms in this trial.
**Methods:** In an exploratory re-analysis of published data from the RESIS study (Wobrock et al. 2015), we tested the impact of rTMS applied to the left dorsolateral prefrontal cortex on two PANSS factors for negative symptoms in psychotic disorders as well as on a PANSS five-factor consensus model intending to show that active rTMS treatment improves PANSS negative symptom subscores.
**Results:** In accordance to the original analysis, all PANSS factors showed an improvement over time in the active and, to a considerable extent, also in the sham rTMS group. However, comparing the data before and directly after the rTMS intervention, the PANSS excitement factor improved in the active rTMS group significantly more than in the sham group, but this finding did not persist if follow-up data were taken into account. These additional analyses extend the previously reported RESIS trial results showing unspecific improvements in the PANSS positive subscale in the active rTMS group.
Our PANSS factor-based approach to investigate the impact of prefrontal rTMS on different negative symptom domains confirmed no overall beneficial effect of the active compared to sham rTMS.
**Discussion:** This secondary analysis of the RESIS trials has several limitations. First of all, the analysis of the primary endpoint was negative [24] and all subsequent secondary analyses showing a positive effect of the intervention (here: change in PANSS excitement factor) are of limited statistical power and therefore subject to uncertainty. On the other hand, our analyses confirm the negative finding of the original publication extends this finding to a broader negative symptom definition. Moreover, the new analysis provides a possible, but hypothetical explanation for the previously described effect of active rTMS on PANSS positive subscale. Of course, many other PANSS factor models are available and in pharmacological research the Marder factors [23, 35] have particular significance. However, the here used five-factor consensus model [21] includes the Marder factor results and our negative symptom factors overlaps with those factors. Another limitation is that it may be possible that our sham stimulation (coil tilted over one wing at an angle of 45°[24]) may still have been slightly biologically active as discussed elsewhere [24].

### S50. EMPLOYING TEXT-MESSAGES TO IMPROVE MOTIVATION: MOBILE ENHANCEMENT OF MOTIVATION IN SCHIZOPHRENIA

Lauren Luther*,[1], Melanie Watkins[1], Annalee Johnson-Kwochka[1], Michelle Salyers[1]
*[1]Indiana University-Purdue University*

**Background:** Motivation deficits are among the strongest determinants of reduced functioning and quality of life in people with schizophrenia. Mobile interventions are a promising approach to improving these deficits because they can provide frequent cues and reinforcements to support goal-directed behavior in daily life. The objective of this study is to assess the initial feasibility/acceptability and effectiveness of Mobile Enhancement of Motivation in Schizophrenia (MEMS), a personalized mobile text message intervention, compared to a goal-setting alone intervention.
**Methods:** Fifty-six participants with a schizophrenia-spectrum disorder have been enrolled in this ongoing controlled pilot study. Twenty-seven participants have been randomized to MEMS, while 29 participants have been randomized to the goal-setting alone condition. Participants in both groups set individualized recovery goals to complete over an 8-week period. Those in the MEMS group also receive three sets of personalized, interactive text messages each weekday to reinforce and cue goal completion. Blinded assessments are conducted before and after the 8-week period and include validated measures of motivation, quality of life, and functioning. Goal attainment and self-reported satisfaction with MEMS are also assessed.
**Results:** To date, 36 participants ($n = 18$ in each group) have completed both baseline and follow-up assessments. Initial results suggest that relative to the goal-setting alone group, the MEMS group demonstrated significantly greater improvements in clinician-rated motivation ($F(1, 33) = 7.14$, $p = .01$; between-group $d = .89$). Specifically, the MEMS group demonstrated significantly higher clinician-rated motivation after 8 weeks (within-group $d = .62$), while clinician-rated motivation remained the same in the goal-setting alone group (within-group $d = -.02$). Across both groups, participants also significantly improved on clinician-rated functioning over time ($t(35) = -2.56$, $p = .02$, $d = .43$), but there was no difference between the two groups ($F(1, 33) = .01$, $p = .94$; between-group $d = .03$). No improvement on self-reported quality of life was observed in either group or across the full sample. The MEMS group reported strong satisfaction with the text-messages. Recruitment has been completed, and analyses from the full sample will be ready to present at the meeting.
**Discussion:** Initial results indicate that MEMS is acceptable and may successfully improve motivation in people with schizophrenia-spectrum disorders. However, additional analyses with the full sample are needed to more rigorously test the feasibility and effectiveness of MEMS.

### S51. MOTIVATIONAL ENHANCEMENT IMPROVES TREATMENT OUTCOMES OF MOBILE-BASED COGNITIVE REMEDIATION IN INDIVIDUALS WITH SCHIZOPHRENIA

Eun Jin Kim*,[1], YongChun Bahk[1], Kee-Hong Choi[1]
[1]*Korea University*

**Background:** Cognitive impairment is a core feature of schizophrenia, which limits functions of individuals with schizophrenia and negatively influences their quality of life (Green, 1993; Green et al, 2000; Heaton et al., 2001; Heinrichs, 1998). While pharmacological treatment is known to have a limited effect on impaired cognition in schizophrenia (Marder, 2006; Rund and Borg, 1999; Elie et al., 2010), a majority of literature has concluded that cognitive remediation(CR) produces small to moderate improvements (McGurk et al., 2007; Wykes et al., 2011). As the smartphone user population continues to increase, the effectiveness of CR based on mobile devices have started to be studied. While CR is effective in improving cognitive deficit, treatment adherence and engagement of participants in the real world setting is known to be poor compared to laboratory setting. Thus, in the current randomized controlled study, we aimed to investigate whether motivational intervention would enhance motivation, treatment adherence and neurocognitive function of individuals with schizophrenia.
**Methods:** All subjects participated in a group-based CR using mobile application (mCR) twice a week for five weeks, and were given opportunity to practice voluntarily outside the treatment sessions. While CR only group participated in usual CR with Q&A sessions, experimental group participated CR sessions integrated with motivational intervention. For motivational enhancement (ME), we employed principles (e.g., goal setting, linking of CR with life goals, etc) of the bridging group (Medalia, Revheim, & Herlands, 2009) along with key aspects of motivational interviewing (e.g., open end questions, affirmation, reflect, and summary). We hypothesized that compared to CR only group, CR+ME group would show higher levels of intrinsic motivation, attendance rate and extra voluntary training hours, and greater improvement in cognitive functions.
**Results:** We are undergoing the current project, and a total of 14 participants were randomly assigned to either CR+ME (n=8) or CR only (n=5). Among 14 participants, two participants dropped out (n=1 experimental group and n=1 control group).
Independent sample t-test were used to compare scores of demographics and clinical characteristics between groups, and no differences were found except for the PANSS excitement subscale ($t = 2.91$, $P < .05$) at the time of pre-treatment. Due to a small sample, we conducted paired sample t-tests to examine whether there was a significant difference between the pre and post-test for two groups, respectively. The paired t-test revealed improvements in coding, TMT-B, logical memory I and K-AVLT immediate recall performances of CR+ME ($t=-2.92$, $p < .01$; $t=-3.65$, $p < .05$; $t=-3.20$, $p < .05$; $t=-2.89$, $p < .05$), but not CR only. In addition, there were pre and post-treatment differences in motivation variables (MSQ) for CR+ME. Comparing task related motivational level of first session to the final 10th session, CR+ME showed increased identified regulation(IR) score of MSQ and decreased external regulation(ER) score (IR=22.3(3.2), 23.5(3.7); ER= 10.67 (3.88), 6.67 (3.08)).
**Discussion:** We conclude that ME is promising to further enhance neurocognitive and motivational outcomes of mCR. The data collection process is expected to be completed in late January 2018, and the results will be accordingly updated by the time of presentation at SIRS 2018. Limitations and future directions will be discussed.

### S52. WORKING MECHANISMS OF VIRTUAL REALITY BASED CBT FOR PARANOIA: A RANDOMIZED CONTROLLED TRIAL EXAMINING COGNITIVE BIASES, SCHEMATIC BELIEFS AND SAFETY BEHAVIOR

Chris Geraets*,[1], Marije Van Beilen[1], Roos Pot-Kolder[2], Mark Van der Gaag[2], Wim Veling[1]
[1]*University Medical Center Groningen;* [2]*VU Amsterdam*

**Background:** Recently, the efficacy of a novel virtual reality based cognitive behavior therapy (VR-CBT) for paranoia was demonstrated. Cognitive biases, cognitive limitations, negative schematic beliefs and safety behavior have been associated with paranoid ideations and delusions. It is unknown whether VR-CBT affects these associated factors, and how changes in these factors relate to changes in paranoid ideation.
**Methods:** In this multi-center randomized controlled trial patients with a psychotic disorder and paranoia were randomized to VR-CBT (n = 58) or treatment as usual (TAU; n = 58). VR-CBT consisted of maximally sixteen 60-minute individual therapy sessions. Paranoia, safety behavior, schematic beliefs, cognitive biases and limitations were assessed at baseline, post-treatment (at three months) and follow-up (at six months). Mixed model analyses were conducted to study treatment effects. Mediation analyses were performed to explore putative working mechanisms by which VR-CBT reduced paranoia.
**Results:** VR-CBT, but not TAU, led to reductions in jumping to conclusions, attention for threat bias and social cognition problems. Schematic beliefs remained unaffected. The effect of VR-CBT on paranoia was mediated by reductions in safety behavior and social cognition problems.
**Discussion:** VR-CBT affects multiple mechanisms that are associated with paranoid ideation. Although maintaining factors of paranoia are likely to influence each other, targeting safety behavior and social cognitive problems seems effective in breaking the vicious circle of paranoia.

### S53. COMPARISON OF RALOXIFENE AND ISRADIPINE AS AN ADJUNCTIVE TREATMENT IN COGNITIVE DEFICITS OF PATIENTS WITH SCHIZOPHRENIA

Bita Vahdani*,[1], Alireza Armani kian[1], Abdolreza Esmaeilzadeh[1], Saeedeh Zenoozian[1], Vida Yousefi[1], Saeedeh Mazloomzadeh[1]
[1]*Zanjan University of Medical Sciences*

**Background:** Cognitive impairment is the most important feature of schizophrenia that leads to severe social and functional disability. Improving neurocognitive physiopathologic aspect of schizophrenia is a current challenge to identify the pathway to develop goal directed clinical interventions in practice. In the current study we investigated the effect of raloxifine as a selective estrogen modulator and isradipine as a voltage gated L type calcium channel blocker on the enhancement of schizophrenic patients' cognitive deficits.
**Methods:** We designed a double blind randomized, parallel, placebo controlled clinical trials. 60 patients with schizophrenia randomized in 3 specific groups. The first group received isradipine 5 mg, the second raloxifine 60 mg and the third placebo for 6 consequent weeks, in the same shape capsules, 2 times a day, alongside treatment with the conventional antipsychotics. The initial and final lab tests, ECG, as well as cognitive tests in specific domains such as attention, processing speed, executive function and verbal memory were carried out.
**Results:** Our findings, revealed a remarkable association between adjunctive treatment of raloxifine in verbal memory deficits. moreover, isradipine treatment indicated significant improvement relative to placebo in verbal memory as well as attention dysfunction in some variables of the Stroop test. However, no effect was observed in processing speed and executive function deficits.
**Discussion:** The study provides the first evidence to our knowledge, which isradipine as a novel therapy was associated with improvement in verbal memory and attention, both related to hippocampal and cerebellar activity. Overall, further investigation is necessary to determine the various ways of the both drugs performance in the brain.

### S54. THE ROLE OF THE CLINICAL PHARMACIST IN DRUG EDUCATION FOR INCREASING COMPLIANCE WITH DRUG THERAPY IN THE PERIOD OF DISCHARGE WITH THE DIAGNOSIS OF SCHIZOPHRENIA SPECTRUM DISORDERS

Nadir Yalcin*,[1], Ayce Celiker[1], Seref Can Gurel[1], Sertac Ak[1], Mumin Kazim Yazici[1]
[1]*Hacettepe University*

**Background:** The inability to achieve full compliance with drug treatment during the post-discharge period with exacerbations in the illness in patients with schizophrenia and other psychotic disorders is a major problem for the patients themselves, their families, and the healthcare staff in psychiatry.
**Methods:** In this prospective study, it was aimed to evaluate whether the written and verbal drug education (drug color and shape, interactions, side effects, etc.) given by the clinical pharmacist during the discharge period had an effect on drug compliance. Between 1st September 2016 and 12th June 2017, 40 patients diagnosed with schizophrenia, schizoaffective disorder, schizotypal personality disorder or acute schizophrenia-like psychotic disorder according to ICD-10 diagnostic criteria who were admitted to Hacettepe University Faculty of Medicine, Department of Psychiatry Inpatient Service, were involved in this study. A number of scales were used to evaluate the severity of illness, drug side effects and drug compliance respectively; PANSS; UKU, SAS, BARS, AIMS; MARS and ROMI. It has been emphasized during discharge to the patients by the clinical pharmacist that how important administering the prescribed medicines regularly and as directed. Six to 8 weeks after discharge, the patients were invited to be reevaluated using the scales applied during admission.
**Results:** There was a statistically significant increase in compliance with treatment as quantitatively assessed by the MARS after drug education ($p<0.001$). There was no significant correlation between compliance and gender, age, tobacco/alcohol use or marital status. At the same time, a significant correlation between severity of akathisia obtained through BARS and a decrease in MARS scores representing the level of compliance was observed (r: -0.367; $p<0.05$). A decrease in the baseline MARS score was related to an increase in the total number of hospitalizations (r: -0.325; $p<0.05$) and the number of psychotropic drugs used (r: -0.316; $p<0.05$). When the factors that may affect compliance were examined by multiple regression analysis, akathisia was found to have the highest impact on compliance (ß: -0.389, r2: -0.002, F: 0.750).
**Discussion:** These results support the literature in terms of the importance of the impact of side effects on compliance. As a result of the study, it was seen that drug counseling services given by clinical pharmacists can effectively be employed in psychiatric care, for the rational use of medicines. It appears that it is necessary to take advantage of drug counseling on drug use and to develop strategies to improve drug compliance in psychiatry.

### S55. MECHANISTIC BASIS OF FRONTO-TEMPORAL TRANSCRANIAL DIRECT CURRENT STIMULATION ON AUDITORY VERBAL HALLUCINATION IN SCHIZOPHRENIA: A MEDIATION ANALYSIS OF COROLLARY DISCHARGE

Anushree Bose*,[1], Hema Nawani[1], Sri Mahavir Agarwal[1], Venkataram Shivakumar[1], Janardhanan C. Narayanaswamy[1], Devvarta Kumar[1], Ganesan Venkatasubramanian[1]
[1]*National Institute of Mental Health and Neurosciences (NIMHANS)*

**Background:** Corollary discharge (CD), ubiquitous throughout the animal kingdom, refers to suppression of sensory consequences arising from self-generated actions. Complex motor acts like covert/overt speech are associated with corollary discharge that helps in ascertaining agency. Auditory verbal hallucinations (AVH) are hypothesized to originate due to failure of corollary discharge in auditory processing system. Transcranial Direct Current Stimulation (tDCS), as an add-on treatment, has been reported to significantly reduce severity of persistent AVH in schizophrenia patients. In this study, we describe mediation analysis findings that strongly support a role for amelioration of corollary discharge deficits as a mechanistic basis for tDCS effects on AVH in schizophrenia.
**Methods:** 27 DSM-IV-TR Schizophrenia patients (SCZ) with persistent AVH despite adequate antipsychotic treatment and 27 healthy controls (HC) underwent neurophysiological assessment for CD. In an event-related potential task, N1 component that reflects cortical responsiveness of auditory cortex to sounds, was elicited and examined in two conditions – i) Talk (with online auricular feedback of self-spoken speech sounds) and ii) Listen (passive playback of recorded self-spoken speech sounds). Corollary discharge index (CDI) was calculated by subtracting Listen condition N1 amplitude from Talk condition N1 amplitude (at FCz). Among these 27 patients, 13 patients participated in a randomized, double-blind, sham-controlled study examining the effect of add-on tDCS on AVH and CDI [5 consecutive days, twice-daily, 20-minute sessions; 2mA; anode: left dorsolateral prefrontal cortex; cathode: left temporo-parietal junction]. Mediation analysis was modelled with tDCS type (Verum vs. Sham) as independent variable, percent change in auditory hallucination rating scale score (AHRS) as dependent variable and percent change in CDI as the mediator. As recommended for small samples, bootstrap estimation approach with 5000 samples was used to examine the indirect effect of independent variable on dependent variable through proposed mediator for significance.
**Results:** SCZ (Mean±SD: $-0.67 \pm 1.93$) had significantly deficient CDI than HC ($1.36 \pm 2.18$) (t=3.62; p=0.001). Verum tDCS ($32.24 \pm 16.48$) resulted in greater percentage reduction in AHRS than sham ($4.79 \pm 8.84$) (t=3.64, p=0.004). There was a significant increase in CDI (t=2.48; p=0.03) with verum ($0.85 \pm 1.08$) but not sham ($-0.55 \pm 0.98$) tDCS. Percent change in CDI positively correlated with percent change in AHRS from pre-RCT to post-RCT time-point for the entire sample (N=13; $\rho=0.55$, p=0.05). Regression analysis showed that tDCS type (verum/sham) was a significant predictor of percent change in AHRS ($\beta=-27.46$, p=0.003) as well as percent change in CDI ($\beta=-1.40$, p=0.033). Percent change in CDI was a significant predictor of percent change in AHRS ($\beta=8.87$, p=0.014). When controlled for percent change in CDI, tDCS type ceased to be a significant predictor of percent change in AHRS ($\beta=-15.0$, p=0.063). The predictors accounted for approximately 75% of the variance (R2=0.756, $p<0.001$). Bootstrap estimation results indicate the coefficient of indirect effect to be significant, $\beta=-12.46$, SE=6.92, 95% CI=-31.20, -2.79, and significantly different from zero at $p<0.05$ (two tailed).
**Discussion:** Fronto-temporal tDCS reduces severity of auditory verbal hallucination in schizophrenia possibly through correction of the deficient corollary discharge. Fronto-temporal network is crucial to self-tagging component of auditory processing and has conspicuous implications for auditory verbal hallucination pathophysiology.
Trial No. CTRI/2014/12/005307 (Clinical Trials Registry–India)

### S56. PHOENIX GROUP, A PROJECT TO PREVENT RELAPSES IN SCHIZOPHRENIA

Marcelo Chiramberro*,[1], Tuula Kieseppä[2], Asko Wegelius[1], Tuomo Töhönen[1], Jenna Ilvonen[1], Heli Väisänen[1], Katja Sarkkinen[1], Jenni Tikkala[1], Marita Leiponen[1], Markku Ruppa[1], Matias Hyytiäinen[1], Ira Hynninen-Sundelin[1], Esa Anttonen[1]
[1]*Peijas Hospital, University of Helsinki;* [2]*Helsinki University Hospital*

**Background:** Schizophrenia is a syndrome of variable and highly disruptive psychopathology that infers emotion, perception, and several aspects of behavior. Relapses caused by noncompliance are common and may lead to hospitalizations. In many ways they increase social disability and health care costs.
The project's philosophy was based on the motivation of the patients to continue their treatment, improve their quality of life, and reduce disease relapses, which in turn reduces public health care costs.
**Methods:** We enrolled 10 schizophrenia and schizoaffective patients with a poor treatment adhesion in a pilot group. All the patients were at the acute psychosis ward. The patient's average hospitalization days were 180 days/year, and they were highly noncompliant with the open ward treatment accompanied with assertive community treatment model. Seven patients had depot injections and three patients had oral antipsychotic medication.
During the hospital treatment the patients started in a group, which continued after the discharge. Weekly group meetings with different activities were organized at the hospital by the ward personnel. The doctor, nurses, a psychologist and an occupational therapist participated the group with different combinations and in a nonhierarchical manner. Psychoeducation was used to increase the knowledge and coping with the disorder. Functional group activities like cooking, arts and visits to different places were organized.
The clinical parameters including the work status, relapse rates and hospitalization days were evaluated at every 6-months during 18 months. For the clinical measurements we used the Brief Psychiatric Rating Scale (BPRS) and 15D Health Related Quality of Life instrument.
**Results:** At 18 months follow-up, eight of 10 group members had not needed hospitalization at all, one needed hospitalization of 15 days and another 20 days. Both of them were in voluntary treatment. During the pilot stage, two patients got jobs.
At five year follow-up, five of 10 initial patients were full-time employed persons. None have needed hospitalization after 18 months.
**Discussion:** The intensive group focused on noncompliant patients, organized by the hospital ward, where these patients had been recurrently treated, improved substantially the patients' commitment to the treatment and decreased rehospitalizations. The non-hierarchical group operating in the interface of the hospital and open ward was able to cause a significant reduction of general health costs and an improvement in the quality of life of these patients. They were reintegrated into society, and the stigma and marginalization associated with psychoses decreased while the self-esteem improved. The patients were able to create friendships with others in the same situations. They helped one another, and thus also improved their own self-help capacity. These elements prevent social isolation, treatment nonadherence and functional deterioration, which also would be a risk for increased violence and suicide.
The group meetings began during the hospital treatment, and the patients intensively continued in the familial group after the discharge. However, the goal for these patients is gradually to leave the group and attend other open-ward, occupational and social activities.
**Conclusions:** Through psycho-educational interventions combined with pharmacological and psychological treatments, Phoenix group, a patient orientated, peer supporting and hospital wall-breaking method obtains results clearly observable that we can warmly recommend.

### S57. TREATMENT SATISFACTION IN ACUTE PHASE PSYCHOSIS: COMPARISON BETWEEN ANTIPSYCHOTIC NAÏVE AND PREVIOUSLY MEDICATED PATIENTS

Lena Stabell*[,1], Rolf Gjestad[1], Rune Kroken[2], Else-Marie Løberg[1], Hugo A Jørgensen[3], Erik Johnsen[2]
*[1]Haukeland University Hospital; [2]Haukeland University Hospital, University of Bergen; [3]University of Bergen*

**Background:** Patient satisfaction is a complex phenomenon, and relations between patient satisfaction, symptom load, insight, side effects and depression have been shown in patients treated for psychosis. However, few studies on patient satisfaction in psychosis discriminate between persons previously treated with antipsychotic drugs and those who are antipsychotic drug naïve.
Our aim was to test whether predictors for treatment satisfaction differ between previously medicated with antipsychotics (PM) and those who were antipsychotic naïve (AN) at admission.
**Methods:** In total 226 consecutive patients were included when admitted to hospital due to symptoms of active psychosis (≥4 on in one or more items of PANSS positive subscale), and were candidates for oral antipsychotic medication. At baseline PANSS, CDSS and Clinical Global Impression-Severity of Illness scale (CGI-S) were conducted. A total of 104 patients were assessed at discharge or follow up after a maximum of 11 weeks (mean 28.5 days, SD 14.1). In addition to the baseline assessments, patient satisfaction was assessed by the UKU Consumer Satisfaction Rating scale and the UKU Side Effect Rating Scale. For statistics structural equation modelling was performed to test multi sample growth models.
**Results:** Patients assessed at baseline were found to be statistically similar to those also assessed at discharge/follow-up, with the exception of a slightly higher PANSS negative subscale score in those assessed at baseline only (independent samples t-test: $p = 0.023$, mean difference 2.3, 95% confidence interval of the mean difference 0.3–4.3).
There was a general improvement in function between baseline and follow-up, reflected in the CGI-S score reduction from 5.17 (SD .619) to 3.70 (SD 1.09) and symptom reduction in PANSS positive: mean change - 6.68 (SD 5.00).
For PM patients (N =55), satisfaction was predicted by level of insight ($b = -2.21$, $\beta = -0.42$, $p = 0.000$), and positive symptom reduction ($b = -0.56$, $\beta = -0.39$, $p = 0.012$). The most satisfied patients had high level of insight at baseline, and the steepest decline in positive symptoms.
For AN patients (N =49), satisfaction was predicted by level and change of insight (respectively: $b= -2.21$, $\beta = -0.46$, $p = 0.000$; $b = -1.53$, $\beta = -0.32$, $p = 0.032$), change in depression ($b = -0.37$, $\beta = -0.26$, $p = 0.025$), and side effects ($b = -0.15$, $\beta = -0.30$, $p = 0.033$). The most satisfied patients had high level of insight at baseline, most improvement in insight, the steepest decline in the CDSS score, and the lowest level of side effects.
**Discussion:** The consecutive inclusions of patients make these finding relevant and generalizable to everyday practice in similar clinical settings.
Our findings suggest that reducing positive symptoms and side effects are important, but not solely sufficient to enhance patient satisfaction, and that differences exist among drug naïve and previously medicated patients. Improving insight and reducing depression are key processes to enhance satisfaction, particularly for antipsychotic naïve patients. Being drug naïve may be considered a proxy for First Episode Psychosis (FEP). Symptoms of depression seem to be particularly prevalent in FEP patients (1), and might dominate the experienced distress relative to symptoms of psychosis in these patients. Furthermore, FEP patients are more sensitive to the side effects of antipsychotic drugs. Another key target for improving satisfaction is to keep antipsychotic drug treatment at the lowest effective doses, as most side effects are dose-related.
References
1. Cotton SM, Lambert M, Schimmelmann BG, Mackinnon A, Gleeson JFM, Berk M, et al. Depressive symptoms in first episode schizophrenia spectrum disorder. Schizophrenia research. 2012;134(1):20–6.

### S58. A RANDOMIZED CONTROLLED TRIAL COMPARING VIRTUAL REALITY THERAPY TO COGNITIVE BEHAVIORAL THERAPY IN SCHIZOPHRENIA WITH TREATMENT REFRACTORY HALLUCINATIONS: PRELIMINARY RESULTS

Laura Dellazizzo[1], Stéphane Potvin[1], Kieron O'Connor[1], Alexandre Dumais*[,2]
*[1]University of Montreal; [2]Institute Philippe Pinel of Montreal*

**Background:** While many pharmacological and psychosocial interventions are available, many treatment-resistant schizophrenia patients continue to suffer from persistent psychotic symptoms, mainly auditory verbal hallucinations (AVH). Recently, a psychological therapy using computerized technology has shown large therapeutic effects on AVH severity by enabling patients to engage in a dialogue with a representation of their distressing voices. These very hopeful results have been extended by our team using immersive 3D virtual reality. The results of VR therapy (VRT) in our pilot trial involving 15 schizophrenia patients with refractory AVH were clinically promising for the severity and distress related to hallucinations, illness symptomatology, depressive symptoms and quality of life. Notably, clinical improvements of our pilot remained significant at our 3-month follow-up. Such findings suggest that VRT seems to be a highly promising intervention for refractory AVH.
**Methods:** To further research in this field, the primary goal of this randomized-controlled trial is to show that VRT is superior to a widely utilized psychotherapy, that is Cognitive behavioral therapy (CBT), for the treatment of persistent auditory verbal hallucinations in schizophrenia. Our secondary goal is to examine the effects of these interventions on beliefs about voices, illness symptomatology, mood symptoms (anxiety and depression), self-esteem, level of functioning and quality of life.
This is a single-blinded randomized-controlled, single-site parallel study of VRT versus CBT. Each treatment group will include 52 randomized participants (assuming 20% attrition) of over 18 years of age hearing persecutory voices and suffering from treatment resistant schizophrenia or schizoaffective disorder. Diagnoses will be established with the Structured Interview for DSM-V. Patients will be included if they have been hearing persecutor voices that did not respond to ≥2 antipsychotic trials.
VRT comprises of 9 weekly sessions: 1 avatar creation session and 8 therapeutic sessions, where the patients are confronted to their reproduced hallucinatory experience and are encouraged to enter in a dialogue with their virtual persecutor. CBT includes 9 weekly sessions consisting of learning modules and task assignments. Subjects will be evaluated at baseline and post-treatment to assess primary (auditory verbal hallucinations as measured with the Psychotic Symptoms Rating Scale) and secondary outcomes. Mixed Anova analyses will be performed to measure and compare the effects of both interventions.
**Results:** Presently, 37 patients have been recruited and 9 have abandoned the study. Our preliminary results on 28 patients show that there is no significant difference between the treatment conditions for all our measures. As expected, more participants will be required to show the superiority of VRT over CBT. However, when performing separate ANOVA analyses for each condition, VRT shows significant improvements of auditory verbal hallucinations severity after the treatment (on our primary outcome) contrarily to CBT. VRT also produced significant decreases on the beliefs that voices are omnipotent and malevolent, on psychotic symptomatology, depressive symptoms and an increase on quality of life. CBT obtained no significant improvements.
**Discussion:** While limited by the small number of patients, such findings are nonetheless already supporting the hypothesis of the superiority of VRT on auditory verbal hallucinations. As expected, a moderate effect is found for our adapted short CBT for psychosis, though not significant at this point. The current trial will contribute to the validation of a novel innovative approach answering a fundamental clinical need.

### S59. CHILDHOOD TRAUMA IS ASSOCIATED WITH SOCIAL COGNITION AND SCHIZOTYPAL PERSONALITY TRAITS IN PSYCHOTIC AND HEALTHY POPULATIONS

Yann Quide[1], Sarah Cohen-Woods[2], Nicole O'Reilly[1], Vaughan Carr[1], Bernet Elzinga[3], Melissa Green*[,4]
*[1]University of New South Wales; [2]Flinders University; [3]Leiden University; [4]University of New South Wales, Prince of Wales Hospital*

**Background:** Childhood trauma is a transdiagnostic risk factor for adult psychiatric disorders, including schizophrenia and bipolar-I disorder. Recent meta-analytic and epidemiological studies suggest a 3-fold increase in risk for psychotic symptoms in adulthood, following childhood trauma exposure. However, associations between trauma exposure and schizotypal personality traits, as well as cognitive and social cognitive abilities, have been less well studied in clinical populations spanning the psychotic-mood spectrum.
**Methods:** Participants were 79 schizophrenia cases, 84 bipolar disorder cases, and 75 healthy control participants who completed the Childhood Trauma Questionnaire (CTQ), the Schizotypal Personality Questionnaire (SPQ), and a standard battery of cognitive tests (to measure executive functions, working memory, attention, immediate and delayed memory), as well as social cognitive tests of facial emotion processing (the Ekman 60 faces task) and Theory-of-Mind (The Awareness of Social Inference Test; TASIT). The CTQ measures childhood trauma exposure on 5 domains (physical abuse, emotional abuse, sexual abuse, physical neglect, emotional neglect); clinically significant levels of childhood trauma exposure on at least one domain (according to specified thresholds for each domain) were evident in 54 schizophrenia cases, 55 bipolar disorder cases, and 26 healthy individuals. Trauma-exposed and non-exposed groups were compared on schizotypal personality features (referred to as 'schizotypy'), cognitive and social cognitive abilities.
**Results:** In both the clinical groups and healthy controls, trauma-exposed participants reported higher levels of schizotypy, especially suspiciousness, relative to non-exposed individuals; this was revealed in the context of higher overall schizotypy levels in both schizophrenia and bipolar disorder, relative to healthy controls. Similarly, while the schizophrenia group showed lower social cognitive and cognitive performances relative to both the bipolar disorder and healthy control groups, trauma-exposed individuals showed deficits in social cognitive, but not general cognitive abilities, regardless of case versus control status.
**Discussion:** These findings suggest that childhood trauma exposure has long-term effects on schizotypy, especially suspiciousness, and complex social cognitive abilities in both healthy and psychotic populations. However, there was no interaction of clinical group with trauma exposure in relation to schizotypal personality dimensions, and the influence of early life trauma on cognitive functions was not distinguishable from the effects of psychotic illness in adulthood. It is possible that traumagenic processes contribute to paranoid ideation and social cognitive disturbances that contribute to psychosis-proneness in the general population, consistent with historical models of schizotypy as latent liability for schizophrenia and related psychotic disorders.

### S60. SPANISH ADAPTATION AND VALIDATION OF THE SFRT-2 IN PATIENTS WITH SCHIZOPHRENIA AND HEALTHY CONTROLS

Ainara Gómez-Gastiasoro[1], Javier Peña[1], Leire Zubiaurre-Elorza[1], Naroa Ibarretxe-Bilbao[1], Natalia Ojeda*[,1]
*[1]University of Deusto*

**Background:** Social cognition (SC) impairment is common among patients with schizophrenia. This multidimensional construct comprises four domains: a) theory of mind (ToM); b) social perception (SP); c) attributional style (AS); and, d) emotion perception (EP). Especially within SC subdomains, SP, along with neurocognition, seems to be highly related to functional outcome in this population. However, nowadays and to our knowledge, only one measure of SP is available in Spanish and none of the existing SP measures have been adapted to native Spanish-speaking population. The scarce number of SP tests available, highlights the need of reliable instruments in Spanish. Therefore, the aim of the present study was to adapt and validate the SP assessment tool "Situational Feature Recognition Test 2" (SFRT-2) into native Spanish-speaking patients with schizophrenia and healthy controls (HC).

**Methods:** The SFRT-2 was translated and retro-translated into Spanish. After that, one hundred and one patients with schizophrenia and 100 HC were assessed in order to obtain psychometric properties of the test. First, reliability of the SFRT-2 was studied with Cronbach's alpha coefficients for actions hits, actions false positives, goals hits and goals false positives separately, in both patients and HC. Second, in patients' group, concurrent validity was calculated using Spearman's correlations in order to assess the relationship between SFRT hits and false positives scores and other SC measures such as ToM, EP, AS and global SC. Third, divergent validity was assessed in patients' group by means of Spearman's correlations in order to study the relationship between SFRT-2 and a neurocognition composite score. Finally, discriminant validity of SFRT-2 actions and objectives hits and false positives was obtained comparing schizophrenia and HC groups by means of Receiver Operating Characteristic (ROC) curve analysis. Percentiles for the SFRT-2 scores were also calculated and shown in order to facilitate clinical assessment of SP.
**Results:** Regarding reliability of the test, internal consistency indexes of the SFRT-2 hits and false positives ranged from $\alpha = .66$ to $\alpha = .90$ in both groups, with higher indexes corresponding to patients' group. Concerning convergent and divergent validity, SFRT-2 significantly correlated with other measures of SC, especially with ToM (SFRT-2 hits: $r = .46$, $p < .01$), and also, but to a lesser extent, with neurocognition composite score (SFRT-2 hits: $r = .33$, $p < .01$). Receiver Operating Characteristic (ROC) curve analysis showed that SFRT-2 hits and false positives discriminate well between patients with schizophrenia and HC, being false positives the indexes which best discriminated between both groups (actions false positives: AUC = .74, $p < .001$; objectives false positives: AUC = .78, $p < .001$).
**Discussion:** Spanish adaptation and validation of the SFRT-2 showed good psychometric properties in both patients with schizophrenia and HC. In addition, reliability of the instrument seemed to be especially high among patients with schizophrenia. To our knowledge, this is the first adaptation and validation of an existing SP measure into native Spanish-speaking patients with schizophrenia. Given the good psychometric properties obtained by the Spanish adaptation, results further support the use of the SFRT-2 as an adequate measure to assess SP in patients with schizophrenia in both research and clinical practice. To that aim, SFRT-2 percentile scores for Spanish population were also provided in order to contribute to the appropriate detection of SP impairment in Spanish-speaking patients with schizophrenia.

### S61. THE ASSOCIATION OF VERBAL LEARNING DEFICITS WITH AGE AND SYMPTOMS IN SCHIZOPHRENIA

Dimitrios Kontis*[,1], Alexandra Giannakopoulou[1], Eirini Theochari[1], Angeliki Andreopoulou[1], Spyridoula Vassilouli[1], Dimitra Giannakopoulou[1], Eleni Siettou[1], Eleftheria Tsaltas[2]
*[1]Psychiatric Hospital of Attica; [2]Athens University Medical School*

**Background:** The relationship of age and symptoms with the performance on verbal learning and memory tasks in schizophrenia could provide useful information for optimizing and individualizing the efforts to remediate the cognitive impairments of patients.
**Methods:** During a cross-sectional study, 97 medicated and stabilized patients with chronic schizophrenia (61 males and 36 females, mean age=43.74 years, standard deviation-SD=11.59), which were consecutively referred to our Unit, were assessed using the Hopkins Verbal Learning Test (HVLT) and the Positive and Negative Syndrome Scale (PANSS). A linear regression analysis was conducted in order to investigate the effect of symptoms and age on HVLT performance.
**Results:** Increased age and total PANSS symptoms were associated with worse total recall (raw scores) (B=-0.109. 95% confidence interval-C.I.-=-0.18, -0.038, t=-3.038, df=90 p=0.003 and B=-0.053, 95%CI=-0.097, -0.008, t=-2.356, df=90, p=0.021, respectively). The effect of symptoms on HVLT total recall was significant for positive (B=-0.166, 95%CI=-0.316, -0.015, t=-2.189, df=90, p=0.031), negative (B=-0.167, 95%CI=-0.279, -0.054, t=-2.949, df=90, p=0.004), but not for general psychopathology symptoms (B=-0.05, 95%CI=-0.129, 0.03, t=-1.247, df=90, p=0.216). Further analyses revealed the significant negative correlations of total symptoms with the performance in immediate recall during the first HVLT trial (B=-0.021, 95% CI=-0.036, -0.005, df=89, p=0.011), and age during the second (B=-0.046, 95%CI=-0.076,-0.017, p=0.003) and third (B=-0.048, 95%CI=-0.083, -0.014, df=89, p=0.007) HVLT immediate recall trials. Both total symptoms and age were significantly negatively correlated with the performance in recognition discrimination (raw scores) (symptoms: B=-0.199, 95%CI=-0.363, -0.035, df=87, t=-2.415, p=0.017 and age: B=-0.357, 95%CI=-0.617, -0.098, df=87, t=-2.737, p=0.008). We failed to find any significant correlation between either age or symptoms with delayed recall.
**Discussion:** Age and symptoms are associated with immediate verbal learning and memory impairments but not with deficits in verbal delayed recall in schizophrenia. The effects of medication remain to be explored in future analyses. Cognitive remediation programmes against verbal learning deficits in individuals with schizophrenia should take into account their age as well as their symptomatology.

### S62. SUBMISSION WITHDRAWN

### S63. WHICH CLINICAL AND COGNITIVE FACTORS ARE RELATED WITH CHANGES IN JUMPING TO CONCLUSIONS IN FIRST-EPISODE PSYCHOSIS?

Susana Ochoa*[,1], Raquel Lopez-Carrilero[1], Eva Grasa[2], Maria Luisa Barrigón[3], EShter Pousa[4], Ana Barajas[5], Esther Lorente[6], Jordi Cid[7], Fermin González[8], Isabel Ruiz[9], Trinidad Pélaez[1]
*[1]Parc Sanitari Sant Joan de Deu; [2]Parc de Salut Marc; [3]Servicio de Psiquiatria y Psicologia Clinica; [4]Hospital del Mar; [5]Les Corts; [7]Hospital Clínic Valencia; [8]Institut de Psiquiatria de Girona, University of Jaen; [9]University of Málaga*

**Background:** The data gathering bias of jumping to conclusions (JTC) consists in a tendency to take a decision without sufficient information. There is evidence that suggests that the JTC bias does not improve (So et al., 2010), however other authors suggest that some psychological interventions such as Metacognitive Training have demonstrated that JTC can be reduced (Aghotor et al., 2010; Moritz et al., 2014; Pankowski et al., 2016; Ochoa et al., 2017). Nevertheless, any study has assessed the clinical and cognitive factor that are related with the improvement of this bias in schizophrenia or first episode psychosis.
The aim of the study is to assess which clinical and cognitive factors are related with the improvement of the JTC after a psychological intervention (Meta-Cognitive or psychoeducational group).
**Methods:** A total of 113 people were assessed with the beads task in two moments: basal and after 3 months. The sample was composed of people with a recent onset of psychosis, recruited from 9 public centers in Spain. Symptoms were assessed with the PANSS and the Psyrats; insight was assessed with the SUMD and the BCIS, and a neuropsychological battery including TMTA and TMTB, digits, WSCT and IQ was used.
**Results:** A total of 28 (24.8%) patients performed JTC in the basal assessment; of them 18 improved JTC after the interventions and 10 remains performing JTC. People who improved JTC presented higher levels of insight (p=0.032), better neuropsychological functioning in TMTA (p=0.011), Digits (p=0.033) and IQ (p=0.014). Moreover, people who improved JTC presented a tendency to score lower in hallucinations (p=0.097), and better in the WSCT (p=0.065) and TMTB (p=0.076).

**Discussion:** Some clinical and cognitive characteristics facilitated that people improved JTC bias. People with a better insight and better scores in attention, memory and IQ have more probabilities to improve JTC after a psychological intervention. These variables should be controlled in the interventions with the idea of better address these.

### S64. EXECUTIVE FUNCTION OF CHRONIC SCHIZOPHRENIA PATIENTS IN A SEVEN-YEAR FOLLOW-UP

Thalita Fernandes[1], Thais Martins[1], Gustavo Mustafé[1], Diego Mendes[1], Luiz Fernando Pegoraro[1], Clarissa De Rosalmei Dantas*,[1]
[1]*University of Campinas*

**Background:** Cognitive deficits in schizophrenia are generalized, but memory and executive dysfunction represent the more robust impairments and are strongly associated with adverse social and occupational outcomes. Those deficits are present in all phases of the disease, but their course, particularly in the chronic phase, is less clear. The aim of this study is to investigate changes in the performance of chronic schizophrenia patients in tests of executive function over a seven-year test-retest period.
**Methods:** We will contact 85 patients with schizophrenia, considered clinically stable in previous year, who participated in a study about the deficit syndrome of schizophrenia in 2009–2010. Back then, they were recruited in two sites: an outpatient service of a general hospital (49 patients) and a community-based mental health service for patients with severe mental illness (36 patients), both in Campinas, Brazil. Patients will be reassessed with the same instruments adopted in the first study: SAPS; SANS; Calgary Depression Scale (CDS); Quality of Life Scale (QLS) and a battery of tests comprising Verbal Fluency Tasks; Digit Span Forward (DSF) and Backward (DSB) and Trail Making Tests (TMT) A and B. Additionally, we included three instruments: PSP, for social functioning; Wisconsin Card Sorting Test (WCST) and London Tower Test (LTT), for executive functions. The Wilcoxon test was used to compare executive performance at baseline and at follow-up. Linear regression was used to test associations between variables. We started the recruitment by the patients originally treated in the outpatient clinic.
**Results:** We present in this poster partial results. Among the 20 patients reinterviewed the mean age at baseline was 36.9 ± 8.9 years, mean duration of mental illness was 16 ± 10.1 years, 75% were men. They had in mean, 10.7 ± 3.3 years of education, only 20% had any work activity, and 15% were married. Mean length of test-retest interval was 6.9 years (minimum 6 and maximum 7.7). At follow-up, 4 patients had improved their education, but only 3 (15%) had any work activity. Up to now 19 patients completed the cognitive reassessment. We performed a principal components factor analysis (PCA) including DSB, TMT-B and VFT for both baseline and follow-up assessments. PCA yielded a single factor for the set of tests in both assessments, which we named Executive Factor, accounting for 57% of variance in baseline and 51.38% in the follow-up assessment. Factor scores were calculated and then compared: 7 patients had higher scores on Executive Factor in the follow-up and 10 had worse scores but differences were not significant. In the linear regression analysis, we did not find significant associations between performance in executive functions in the follow-up assessment and clinical and psychopathological variables neither at the baseline nor at the follow-up assessment. In the Wisconsin test, approximately 60% of the patients managed to form only up to 01 category, which is considered a bad performance. The mean score in LTT was 57.3 ± 10.6 for movements and 170.4 ± 125.2 for time.
**Discussion:** The results presented are partial, obtained with a provisional small sample size but they show some interesting trends. In general, there was a group tendency for a slightly worse performance after 7 years of the baseline assessment, but we could identify two groups of patients who differ from that general tendency: one with marked deterioration and one with improvement of executive performance over time. If those initial findings are to be confirmed, our next step will be to investigate characteristics associated to improvement or deterioration of executive performance. That sort of information is of great relevance in the pursuit of recovery for schizophrenia patients.

### S65. INDEPENDENT COMPUTERISED COGNITIVE REMEDIATION FOR PSYCHOSIS: AN INVESTIGATION OF PATIENT EXPERIENCES

April Hargreaves*,[1], Niamh Daly-Ryan[1], Rachael Dillon[1], Gary Donohoe[2]
[1]*Trinity College Dublin;* [2]*NUI Galway*

**Background:** Cognitive remediation (CR) is an effective therapy, shown to improve cognitive performance and functioning in patients with psychosis. We recently conducted a randomised control trial (RCT) demonstrating the effectiveness of a new computer based therapy, conducted with little therapist support. This study aims to assess the subjective experience of the participants in this trial.
**Methods:** Twenty people with psychosis conducted a post-RCT questionnaire facilitated interview, which assessed their satisfaction with CR. Thematic analysis was then employed to identify common themes.
**Results:** Three broad themes were identified, with participants reporting a predominantly positive experience of taking part in the therapy. In particular, participants reported improved cognition, improved positive self-regard, a development of life skills and a transfer of benefits to everyday life. Whilst there were reports of the therapy being difficult and tiring, patients expressed a positive attitude towards their therapist and a reluctance to see the therapy come to a close.
**Discussion:** It is acceptable and beneficial for patients with psychosis to undertake independent CR therapy with reduced therapist contact.

### S66. THEORY OF MIND IN A FIRST-EPISODE PSYCHOSIS POPULATION USING THE HINTING TASK

Maija Lindgren*,[1], Minna Torniainen-Holm[1], Inkeri Heiskanen[2], Greta Voutilainen[3], Ulla Pulkkinen[4], Tuukka Mehtälä[5], Markus Jokela[6], Tuula Kieseppä[7], Jaana Suvisaari[1], Sebastian Therman[1]
[1]*National Institute for Health and Welfare;* [2]*Helsinki Deaconess Institute;* [3]*Social Services and Health Care, City of Helsinki, Aurora Hospital;* [4]*National Institute for Health and Welfare, Royal College of Surgeons in Ireland;* [5]*Hospital District of Helsinki and Uusimaa, Hyvinkää Hospital Area;* [6]*Helsinki University;* [7]*National Institute for Health and Welfare, University of Helsinki and Helsinki University Hospital*

**Background:** Impaired social functioning is one of the most apparent features of psychotic disorders. Deficits in social cognition may explain impaired functioning even more than the other cognitive deficits related to psychosis. Of the areas of social cognition, especially relevant to psychosis appear to be deficiencies in theory of mind (ToM), the ability to perceive and interpret the mental states of others, or "mentalizing". Currently, it is unclear to what extent general cognitive deficits explain impairment in ToM.
We wanted to explore 1) the possible difference in ToM between first-episode psychosis (FEP) patients and controls, 2) whether diagnosis group (schizophrenia vs. other psychotic disorders) and level of functioning are associated with ToM, and 3) to what extent these differences are explained by general cognitive performance.
**Methods:** This study examined ToM in young adults with FEP (n=66). Of those, 25 had schizophrenia and the rest were diagnosed with other psychotic disorders. Age- and gender-matched controls were identified from the Population Information System (n=62). The participants were administered a broad neuropsychological assessment, part of which was the Hinting Task assessing ToM. With 10 short discussions in everyday situations, the Hinting Task assesses the ability to conclude, from indirect speech, what another person really means.

A factor score of the Hinting Task was formed, taking into account the varying difficulty level and relevance of the 10 items. To investigate the association between ToM and general cognitive functions, we summarized non-social cognitive performance constructing a "g factor", which was used as an overall index of general cognitive performance.

**Results:** The internal consistency of the Hinting Task calculated from the dichotomized data was modest, with McDonald's categorical omega estimated at .57 (95 % CI .36, .71). In the single dimensional factor solution, items 8 and 9 had the weakest loadings and item 10 the strongest. Items 9 and 10 of the Hinting Task were the easiest, and items 1 and 8 were the most difficult.

Participants with FEP (mean score 16.0) performed worse than controls (mean score 17.4) on the Hinting Task (Cohen's d=0.50 calculated from factor scores). However, the difference between FEP and control groups was no longer significant when general cognition was controlled for. 75 % of the variance between the groups was explained by general cognitive deficits, especially impaired processing speed (WAIS-III Digit Symbol) and episodic memory (WMS-III Logical Memory).

When the FEP group was divided according to diagnosis, those with schizophrenia scored lower on the Hinting Task than the other psychosis patients. The ToM difference between individuals with schizophrenia and controls (Cohen's d=1.1) remained significant even when general cognitive performance was controlled for. In contrast, those with other psychotic disorders than schizophrenia did not differ from controls.

ToM performance of the best functioning patient group (20 patients who had a GAF score ≥50, Hinting Task mean score 17.8) did not differ from that of the control group.

**Discussion:** Based on this study, and supporting previous findings (Bora & Pantelis, Schizophr. Res. 2013), deficits in ToM were already present in early psychosis. They were largely overlapping with deficits in general cognitive processes. However, and in line with previous meta-analytical findings (Sprong et al., Br. J. Psychiatry 2007), in a subgroup of the FEP patients with schizophrenia, impairments in ToM remained after controlling for overall cognitive functioning. In conclusion, specific deficits in ToM could be found in schizophrenia, independent from general cognitive deficits.

### S67. INTEGRATION OF SENSORY AND SOCIAL INFORMATION DURING DECISION MAKING IN SCHIZOPHRENIA

Arndis Simonsen*[,1], Riccardo Fusaroli[2], Vibeke Bliksted[3], Ole Mors[4], Andreas Roepstorff[2], Daniel Campbell-Meiklejohn[5]

*[1]The Lundbeck Foundation Initiative for Integrative Psychiatric Research, Aarhus University; [2]Aarhus University; [3]Aarhus University Hospital; [4]The Lundbeck Foundation Initiative for Integrative Psychiatric Research, Aarhus University Hospital; [5]University of Sussex*

**Background:** Previous findings suggest that schizophrenia is associated with abnormalities in information integration. The recent Bayesian model of circular inference attempts to characterize the information integration style in schizophrenia (Jardri et al. (2017). Nat Commun, 8:14218). The model suggests that during information integration of prior beliefs and sensory evidence, patients tend to put too much weight on the sensory evidence and to take it into account multiple times (over-count) compared to healthy individuals. An imbalance in excitatory/inhibitory regulation of hierarchical neural processing has been suggested to cause of this phenomenon (Jardri et al., 2017). Here, we investigated whether circular inference could be extended to describe the integration of sensory information and socially acquired information and whether the specific information integration style could contribute to the characteristic symptomatology and the social impairments seen in the disorder.

**Methods:** Thirty-five patients with schizophrenia or schizo-affective disorder and 40 matched healthy controls performed the task. Participants had to guess the color (red or green) of the next marble to be drawn from a hidden urn based on information from their own sample (eight marbles) and the choices and confidence (high or low) of four other people. We fitted and comparatively assessed four multilevel Bayesian models (generalized linear model, simple Bayes, weighted Bayes, circular inference) describing how patients and controls integrated information. Positive and negative symptom severity and social functioning were also assessed.

**Results:** The circular inference model best described the information integration in both patients and controls (WAIC weight = 1). Patients tended to over-weigh ($\beta$ = 0.48, 95% CI: 0.30; 0.62) and over-count ($\beta$ = 0.30, 95% CI: 0.12; 0.47) sensory information and under-weigh ($\beta$ = -0.29, 95% CI: -0.42; -0.13) and under-count ($\beta$ = -0.23, 95% CI: -0.35; -0.06) social information compared to controls. Crucially, this varied with symptomatology: the higher the symptom severity, the more over-counting and the higher the weight on sensory information and the more under-counting and the less weight on social information. More weight on social information was associated with higher level of functioning in the patients ($\beta$ = 0.88, 95% CI: 0.01; 3.73).

**Discussion:** All participants integrated social and sensory information in a non-linear fashion. Patients displayed a distinctive tendency to rely more and less discriminatively on sensory than on social information. This information integration style may contribute to the characteristic symptoms and the social impairments in schizophrenia. Further exploration of this potential causal role is warranted.

### S68. SYMPTOMS, NEUROCOGNITION, SOCIAL COGNITION AND METACOGNITION IN SCHIZOPHRENIA: A NETWORK ANALYSIS

Ilanit Hasson-Ohayon*[,1], Gil Goldzweig[2], Adi Lavie[1], Lauren Luther[3], Paul Lysaker[3]

*[1]Bar-Ilan University; [2]School of Behavioral Sciences, The Academic College of Tel-Aviv; [3]Roudebush VA Medical Center and the Indiana University School of Medicine*

**Background:** Schizophrenia is associated with broad range of phenomena which affect function and represent significant barriers to recovery. These include semi-independent forms of psychopathology, disturbances in neurocognition, social cognition and metacognition. The current study explores the paths through which these constructs affect each other and whether some of these phenomena play a relatively more or less central role than others as they interact. Answers to these questions seem essential to choosing which of a dizzying array of problems should be targeted by treatment.

**Methods:** Data was collected from 81 adult outpatients with schizophrenia or schizoaffective disorder, recruited at a Veterans' Affairs Medical Center and a community mental health center in Indiana, USA. Network analysis which explored the relative relationships of five groups of symptoms (positive, negative, disorganization, hostility and emotional discomfort), six domains of neurocognition, four domains of social cognition and four domains of metacognition with one another was conducted. The analysis produces the following centrality measures: 1) strength of items within a network according to their sum weighted connections; 2) closeness between items that reflect the distance from a particular item to all others; 3) betweenness which reflect the number of times that an item appears on the shortest path between two other items.

**Results:** A clear differentiation between metacognition, social cognition, neurocognition and symptoms was observed. The only outliers were social cognition attribution, which was close to the symptoms area, and the cognitive symptoms factor that was found close to the neuro-cognition area. The social cognition was found in an "intermediate" area between the metacognition and neurocognition. Metacognition variables were the closest to the symptoms variables. The strongest nodes are: metacognition-self reflectivity, theory of mind measures of social cognition and visual memory. The nodes with the highest closeness measure were self-reflectivity sub-scale of metacognition and theory of mind of social cognition. The node with the highest betweenness measure was metacognition self-reflectivity.

**Discussion:** The centrality of the self-experience in schizophrenia is emphasized in phenomenological, theoretical as well as empirical literature and can be traced back to earlier writing on schizophrenia. Accordingly, a sense of barren or diminished self, problems in self-reflection and self-clarity as well as difficulties in agency and ownership over one's thoughts, feelings and sensations which is necessary for creating meaning were reported and discussed. The current study adds to this body of literature the finding that in a network which includes symptoms, social cognition, neuro cognition and metacognition variables, self-reflection is standing out as being a central connector that has the strongest relationship with other variables. As such it impacts all the network, and interventions targeting metacognitive self-reflection are expected to have secondary effects on additional constructs in the network- i.e additional elements of metacognition, social cognition, neurocognition and symptoms.

### S69. A CASE STUDY OF CLOZAPINE AND COGNITION: FRIEND OR FOE?

Gisela Mezquida*[,1], George Savulich[2], Samuel Atkinson[3], Miguel Bernardo[4], Emilio Fernandez-Egea[5]
[1]*Hosp. Clinic Barcel, Institut Clínic Neuro;* [2]*University of Cambridge;* [3]*Cambridgeshire and Peterborough NHS Foundation Trust;* [4]*Hospital Clínic, Universitat De Barcelona/IDIBAPS/CIBERSAM;* [5]*University of Cambridge, Cambridgeshire and Peterborough NHS Foundation Trust, CIBERSAM*

**Background:** Cognitive dysfunction is as a hallmark feature of schizophrenia. Antipsychotic medication is effective for treating the positive symptoms of psychosis, but their potential for therapeutic effects on cognition continues to divide researchers.

Improvements in clinical symptoms can occur independently of cognitive functioning, whereby typical antipsychotics improve different clinical domains, but have little or no efficacy for improving primary cognitive functions. However, it has been suggested that the second-generation antipsychotic clozapine can improve cognition in schizophrenia. Unusual cases, such as remitted patients who decide to stop taking clozapine, thus represent a unique opportunity to understand the effect of antipsychotic medication on cognition, as described in the case study below.

**Methods:** A 38-year old man suffered severe psychotic episodes at age 19, leading to a diagnosis of treatment-resistant schizophrenia. Clozapine was initiated at age 21, with excellent treatment response and complete remission of positive, negative and depressive symptoms. For the following 16 years, he had a full-time paid job, lived independently and had an ample social circle. For the last five years, he was stable and compliant taking 175 mg of clozapine, with clozapine levels in sub-therapeutic range (between 0.17 and 0.26 mg/L). He had no metabolic syndrome and no oversedation, but complained of hypersalivation and persistent memory problems. He decided, against advice, that medication was no longer needed and requested support for waning off clozapine.

A five-month program was subsequently implemented, with 25 mg reductions of clozapine every six weeks. Four enhanced assessments were scheduled after two weeks on stable doses of 125, 75, 25 and 0 mg, respectively, which included the Brief Assessment of Cognition in Schizophrenia (BACS) and the Clinical Global Impression-Schizophrenia scale (CGI-SCH).

**Results:** At each level of clozapine dose, the patient continued to be stable. However, the patient showed impaired cognitive performance at the highest dose of clozapine titration (see T-scores from Table and figure), with improvements then shown at each level of dose reduction (75 and 25 mg). The final assessment was done after 6 weeks of not taking medication. At this time, he was admitted to hospital for psychotic relapse. Unfortunately, the patient refused to re-initiate clozapine and has remained floridly psychotic for 18 months.

Our case suggests that cognitive impairment is dose-dependent with clozapine. As presented, the t-score of the patient at 125 mg was worse than normative data reported for healthy controls (z-score=0.50) and patients with schizophrenia treated with other first-or second generation antipsychotics (z-score=-1.42). However, cognitive performance of the patient was most notably impaired when medication-free.

**Discussion:** The longterm effects of high-dose antipsychotic medication on cognition in patients with schizophrenia are largely unknown. It is also possible that changes in cognitive states are either domain-specific with clozapine. Nonetheless, daily antipsychotic dose and polypharmacy have been shown to predict poor cognitive functioning.

The present case is also a reminder that the appropriate long-term maintenance dose of clozapine is poorly understood. As this case suggests, the current recommended therapeutic levels of clozapine (0.35 to 0.50 mg/L) might be unnecessarily high for patients in full remission. Controlled studies are therefore needed to evaluate the potential for cognitive improvements in schizophrenia as a function of reduced clozapine dose, to ensure the optimal delivery needed not just for good clinical but also good cognitive outcome.

### S70. ABERRANT SALIENCE: A COMPARISON OF DIFFERENT MEASURES IN ANXIETY AND SCHIZOPHRENIA

Suzanne Neumann*[,1], Richard Linscott[1]
[1]*University of Otago*

**Background:** Aberrant salience is thought to play a role in the development of the symptoms of schizophrenia, but the hypothesis lacks consistent support. Previous research found no relationship in a population sample between two measures of aberrant salience: the self-report Aberrant Salience Inventory (ASI) and the computerised Salience Attribution Task (SAT), which measures implicit (behavioural) and explicit (self-report) aberrant and adaptive salience. We compared the ASI and SAT in individuals with schizophrenia, with anxiety, and with no mental disorder (unaffected).

**Methods:** Individuals with schizophrenia ($n = 30$), anxiety ($n = 33$), or unaffected ($n = 30$) completed the ASI and the SAT.

**Results:** ASI scores were higher in the schizophrenia group than anxiety ($t(90) = 2.72$, $p < .01$) and unaffected groups ($t (90) = 5.29$, $p < .001$) and higher in the anxiety than unaffected group ($t(90) = 2.69$, $p < .01$). SAT explicit adaptive salience scores were lower in the schizophrenia group than the anxiety ($t(90) = -3.79$, $p < .001$) and unaffected groups ($t(90)= -3.86$, $p < .001$). The schizophrenia group also had higher SAT implicit aberrant salience than the anxiety group ($t(90) = 2.57$, $p < .05$) but not the unaffected group ($t(90) = 3.75$, $p = .08$); there was no difference between anxiety and control groups ($t(90)$ -0.76, $p = .45$). Group did not affect SAT explicit aberrant salience ($F(2,91) = 0.47$, $p =.63$) or implicit adaptive salience ($F(90) = 0.62$, $p = .54$). We found no correlation between the ASI and the SAT (all $\tau < .218$, $p > .05$).

**Discussion:** Higher ASI scores were associated with, but not unique to, schizophrenia. Reduced SAT explicit adaptive salience was associated with schizophrenia, while SAT implicit aberrant salience scores differed between psychopathologies. Consistent with previous findings, there was no relationship between the ASI and the SAT. The ASI is designed to measure a trait associated with schizophrenia. Conversely, the SAT implicit aberrant salience measures response latency to irrelevant stimuli. The lack of relationship between ASI and SAT may, therefore, be due to construct divergence.

### S71. ABERRANT TIMING AND SALIENCE NETWORK IN SCHIZOPHRENIA: FINDINGS FROM A META-ANALYSIS

Irene Alústiza*[,1], María Sol Garcés[1], Marta Ortuño[1], Patricio Molero[1], Felipe Ortuño[1]
*[1]Clinica Universidad de Navarra*

**Background:** Schizophrenia (SZ) affects several domains of cognitive function. Abnormal time and novelty processing, which is related to change detection, has been reported in this disorder. Timing and oddball tasks can be used to assess change detection in perceptual processes. We hypothesize that an impaired timing network underlies disruptive cognitive functioning in SZ, such as saliency detection. Therefore, timing dysfunction might be a primary cognitive deficit in this disorder.
To address this issue, our aim was to elucidate the neural areas underlying target detection and timing in SZ, as well as to determine whether the timing dysfunctional activity pattern showed by SZ patients matches the pattern involved in attention salience processing. The final purpose of our study was to identify the brain structures activated during both timing and oddball tasks in patients with SZ, as compared to healthy controls (HC).
**Methods:** We conducted two independent comprehensive literature searches of whole-brain functional magnetic resonance imaging (fMRI) studies that compared patients with SZ and HC using oddball and timing tasks. The searches were conducted with PubMed engine up to November 2017. Keywords used in the first search were: "schizophrenia" plus "functional magnetic resonance imaging" or "fMRI" plus "timing" or "time perception" or "time estimation". In the second search keywords used were: "schizophrenia" plus "functional magnetic resonance imaging" or "fMRI" plus "event-related", plus "oddball".
We excluded studies that 1) used a region-of-interest approach; 2) did not report peak coordinates for the relevant contrast; 3) used different statistical thresholds in different regions of the brain; 4) used techniques other than fMRI; 5) were based on Independent Component Analysis; 6) were case reports, qualitative studies, reviews or meta-analyses.
We ran two independent signed differential mapping (SDM) meta-analyses of fMRI studies conducting comparisons between HC and patients with SZ: one reporting brain activation patterns during an oddball task, and a second one using timing tasks. We carried out a final multimodal meta-analysis to combine the findings from the two previous SDM meta-analyses. The aim of this multimodal analysis was to detect brain regions that are activated or deactivated by both timing and oddball tasks in SZ.
**Results:** Our initial search returned 173 papers, but application of inclusion criteria reduced this number to 8. Among them, 3 studied timing (which included a total of 53 SZ patients and 60 HC) and 5 examined oddball paradigm (which included a total of 100 SZ patients and 122 HC).
Relative to HC, patients with SZ showed significantly hypoactivation in right striatum, right middle frontal gyrus (BA 9 and 45), and right median cingulate / paracingulate gyri (BA 32) during timing tasks. For oddball tasks, even if they showed significantly decreased activation in right inferior parietal gyri (BA 40) and corpus callosum, they also exhibited hyperactivation or failure of deactivation in left superior frontal gyrus, and dorsolateral (BA 9). Finally, overlapping was found in regions that were hypoactivated and hyperactivated by oddball tasks in SZ patients relative to HC.
**Discussion:** Our results show that there is a common dysfunctional participation of frontal, cingulate, striatum, and parietal regions in SZ during both timing and oddball tasks. These findings suggest that a deficient timing network underlies attentional salience. However, these results are preliminary and further studies may be conducted to address the specific role of timing on cognition.

### S72. PRO-SOCIAL PROSPECTIVE MEMORY PERFORMANCE IS ASSOCIATED WITH PLASMA OXYTOCIN LEVEL IN SEXUALLY DIMORPHIC WAY IN FIRST-DEGREE RELATIVES OF SCHIZOPHRENIA

Fuchun Zhou*[,1], Lan Xiong[2], Ping Zhou[3], Yu-Tao Xiang[4], Chuan-Yue Wang[1]
*[1]Beijing Anding Hospital, Capital Medical University; [2]Université de Montréal; [3]Shenzhen Kangning Hospital; [4]University of Macau*

**Background:** Prospective memory (PM) in real world is mostly societal and it often involves activities in which the individual has to remember to do something for others, which is also called pro-social PM. The neuropeptide oxytocin has been implicated in social cognition and social interaction in a number of studies. The aim of the present study was to investigate the correlation between pro-social PM performance and plasma oxytocin level in first-episode schizophrenic patients (FES), first-degree relatives (FDRs) of schizophrenia, and healthy controls (HCs). In addition, we also tried to explore the sexually dimorphic feature in the interactions of abovementioned factors.
**Methods:** Forty-six FES patients, 41 non-psychotic FDRs of patients with chronic schizophrenia (unrelated to the FES group) and 54 HCs were studied. Pro-social time-based prospective memory (TBPM) and event-based prospective memory (EBPM) performance were assessed with the Chinese version of the Cambridge Prospective Memory Test (C-CAMPROMPT). A serial of tests reflecting retrospective memory and executive functions were also administrated. Plasma oxytocin levels were determined by radioimmunoassay using a RIA kit.
**Results:** (1) There were significant differences in performance between FES, FDRs, and HCs with respect both TBPM and EBPM, even after controlling for age, sex and education level by analysis of covariance (ANCOVA). We found significant group*sex interaction only regarding TBPM (F(2, 133)=4.8, p=0.01). Female HCs performed significantly poorer than male HCs on TBPM (11.1 ± 5.5 vs 14.4 ± 4.8, t=-2.3, p=0.026). However, this sexually dimorphic trend was not seen in either FES (9.0 ± 5.1 vs 7.5 ± 5.1, t=1.0, p=0.34) or FDRs (12.3 ± 3.5 vs 11.1 ± 4.4, t=1.0, p=0.3). (2) A significant group*sex interaction was also revealed with regard to plasma oxytocin level (F(2, 134)=4.1, p=0.018). In HCs, females exhibited significantly higher plasma oxytocin level than males (62.2 ± 28.3 pg/ml vs 44.4 ± 20.9 pg/ml, t=2.5, p=0.015). But this sexually dimorphic feature did not appear in FES (58.3 ± 20.1pg/ml vs 59.6 ± 19.4 pg/ml, t=-0.2, p=0.822) or FDRs (60.4 ± 19.2 pg/ml vs 74.0 ± 46.0 pg/ml, t=-1.2, p=0.230). In addition, there was a significant difference in plasma oxytocin level between the three groups only in male. Post-hoc analyses suggest male FDRs exhibited significant higher plasma oxytocin level than male HCs. (3) After controlling age and education level, partial correlation analysis indicated higher plasma oxytocin levels to be significantly associated with higher TBPM scores in FDRs (r=0.39, p=0.015). In order to determine the origin of this correlation, we further conducted 2 separate partial correlation analyses in males and females, still with age and education level as controlled variables. The correlation between plasma oxytocin level and TBPM remained significant in male FDRs (r=0.5, p=0.021) but disappeared in female FDRs (r=0.2, p=0.434).
**Discussion:** In the present study, we found the pre-existing sex-specific patterns (as in HCs) of plasma oxytocin level and TBPM were substantially disrupted in FES and FDRs. Moreover, a significant association between plasma oxytocin levels and PM was only found in FDRs, and only male FDRs contribute to this significant association, suggesting oxytocin may play an important role regulating pro-social PM in FDRs in sexually dimorphic way.
Longitudinal studies with larger sample size and measurement of oxytocin receptor function and genetic variations should be conducted in the future.

### S73. EFFECT OF LURASIDONE ON COGNITION IN ADOLESCENTS WITH SCHIZOPHRENIA: INTERIM ANALYSIS OF A 2-YEAR OPEN-LABEL EXTENSION STUDY

Philip D. Harvey[1], Robert Goldman[2], Michael Tocco*,[2], Ling Deng[2], Josephine Cucchiaro[2], Antony Loebel[2]
*[1]University of Miami Miller School of Medicine; [2]Sunovion Pharmaceuticals, Inc.*

**Background:** The onset of schizophrenia typically occurs during adolescence or early adulthood and is characterized by severe symptomatology and lifelong functional impairment. Cognitive dysfunction is common in schizophrenia and is associated with significant impairment in functioning. Very little is known about the long-term effects of antipsychotic treatment on cognition in adolescents with schizophrenia. To this extent, cognitive data are presented from an interim analysis of an ongoing 2-year long-term safety study of lurasidone in the treatment of children and adolescents with schizophrenia.
**Methods:** Patients aged 13–17 years with schizophrenia who completed 6 weeks of double-blind (DB), placebo-controlled treatment with lurasidone were enrolled in a 2-year, open-label (OL) study in which patients were continued on lurasidone or switched from placebo to lurasidone. Cognitive function was assessed with the Brief CogState battery, which evaluates four cognitive domains: processing speed, attention/vigilance, visual learning, and working memory. Based on normative data, an overall cognitive composite Z-score was calculated as the average of the standardized Z-scores for each of the four cognitive domains. These results are based on an interim analysis of the 2-year data.
**Results:** A total of 271 patients completed 6 weeks of double-blind treatment and entered the 2-year extension study. At the time of the interim analysis, 132 patients had completed 52 weeks of treatment (24 patients were 2-year study completers; 96 patients were still ongoing; and 12 patients had discontinued after 52 weeks); 57 patients were still ongoing in the first 1-year of treatment; and 82 patients terminated prior to week 52. The cognitive composite Z-score showed impairment at double-blind baseline (-1.09). Mean change in Z-score, from DB baseline to OL weeks 0 (OL-baseline), 28, 52, and 104, respectively, were observed for the cognitive composite (+0.04, +0.16, +0.30, +0.57), and for the CogState domains processing speed (-0.08, +0.02, +0.16, +0.68), attention/vigilance (0.00, 0.00, +0.05, +0.38), visual learning (+0.19, +0.45, +0.75, +1.07), working memory accuracy (+0.18, +0.24, +0.73, +0.30), working memory speed (+0.06, +0.23, +0.28, +0.15).
**Discussion:** In this study of adolescents with schizophrenia, lurasidone was not associated with cognitive impairment after up to 104 weeks of treatment. Larger sample sizes are needed to confirm the robustness of the improvement was observed in selected cognitive domain scores, most notably visual learning and processing speed.

### S74. EXPLORING PARTICIPANT-LEVEL TRAJECTORIES OF COGNITIVE PERFORMANCE AMONG PATIENTS WITH SCHIZOPHRENIA IN A MULTI-NATIONAL TRIAL

Kiri Granger*,[1], Jack Cotter[1], Elizabeth Baker[1], John Evenden[1], Jennifer Barnett[1], Michael Sand[2]
*[1]Cambridge Cognition; [2]Boehringer Ingelheim Pharma GmbH & Co. KG*

**Background:** It remains unclear whether the lack of clinical trial success and drug approval for cognitive impairment associated with schizophrenia (CIAS) is due to compounds being ineffective, or whether trial methodology itself has been a limiting factor in successfully demonstrating the efficacy of these agents. Schizophrenia is a heterogeneous disorder and whilst cognitive deficits are a core feature, the profile and degree of neuropsychological impairment can vary across patients. Though most individuals with schizophrenia exhibit some general cognitive impairment compared to antecedent expectations, such as premorbid intelligence, up to a quarter display cognitive performance in the 'normal' range. This may pose a problem for pro-cognitive drug trials in this population given that it potentially inflates baseline scores and reduces the scope to see improvement between treatment and placebo groups. In order to examine this potential issue, we investigated participant-level trajectories of cognitive performance among patients with schizophrenia enrolled in a multi-national, phase II clinical trial.
**Methods:** We conducted a post-hoc analysis of existing trial data from 463 patients with schizophrenia who participated in a randomized, double-blind, placebo-controlled trial. Patients met established diagnosis for schizophrenia (DSM-5), were clinically stable (non-acute) and had no more than moderate severity ratings on the Positive and Negative Syndrome Scale (PANSS). During the trial, participants completed two different neurocognitive test batteries, the Cambridge Neuropsychological Test Automated Battery (CANTAB) and the MATRICS Consensus Cognitive Battery (MCCB), at 4 separate time points (screening, baseline, week 6 & week 12). Participant data were pooled across placebo and treatment groups to explore trajectories of cognitive performance, at the participant-level, across the course of the study.
**Results:** Linear mixed model analyses revealed that participants who performed within the 'normal range' at screening on cognitive tasks as measured by CANTAB, continued to perform well at baseline, week 6 and week 12, showing no significant change in their performance. By contrast, participants who performed below the normal range at screening, showed a significant improvement in their test performance across the remainder of the study. When compared in the context of MCCB, those participants who performed a standard deviation (SD) above the MCCB normative mean at screening, were also the participants who performed within the normal range on CANTAB. Approximately 25% of the overall sample were performing within a clinically normal cognitive range at screening.
**Discussion:** Substantial variability was evident in cognitive performance among the current sample of patients with schizophrenia. We identified a subsample of patients whose performance fell within a clinically normal range. Cognitive improvement was observed only in those who exhibited a deficit at screening, bringing into question whether the inclusion of unimpaired patients in clinical trials increases the risk of ceiling effects and minimizes chance to see change. Further analyses will determine the interaction between different cognitive trajectories and the treatment arms included in this trial to explore whether there are individuals with a particular cognitive profile who are most likely to respond to treatment. This has potentially important methodological implications in the search to find a drug to treat CIAS.

### S75. MALADAPTIVE SOCIAL-EVALUATIVE AND SELF-BELIEFS INFORMING THE RELATION BETWEEN PARANOIA AND SOCIAL ANXIETY

Alexandra Andrea*,[1], LeeAnn Shan[1], Cristina Garcia[1], Christina Savage[1], Melanie Bennett[2], Jack Blanchard[1]
*[1]University of Maryland; [2]University of Maryland School of Medicine*

**Background:** Paranoid delusions are reported among those with positive symptoms of schizophrenia (Bentall et al., 2009), those at high-risk for the development of psychosis (Addington et al., 2015; Salokangas et al., 2016), and in the general population (i.e., Freeman et al., 2005). Paranoia is related to functional impairment in multiple domains (i.e. Pinkham et al., 2016; McGurk et al., 2013). Given the presence of paranoia at both clinical and non-clinical levels, paranoia may be best conceptualized dimensionally. Further, robust relations between paranoia and social anxiety suggest that the two may exist together on one spectrum, with paranoia reflecting the most extreme end of this continuum (i.e. Gilbert, Boxall, Cheung, & Irons, 2005; Lim, Rodebaugh, Zyphur & Gleeson, 2016).

Evidence suggests that several factors may influence the development of paranoia, including social cognitive biases such as diminished trust, increased hostility, and increased tendency to blame others (Pinkham et al., 2014). These types of social cognitive biases do not seem to be unique to paranoia and are also observed among those with social anxiety (i.e., Green & Phillips, 2004). Additional research suggests that paranoia and social anxiety may share other relevant attributes, such as need for approval from others (Rector, 2004), desire for closeness (Lim, Rodebaugh, Zyphur & Gleeson, 2016), and worry about and expectation of social rejection (Freeman, 2014). Despite literature to support overlap between paranoia and social anxiety, there is less research examining whether core beliefs of social anxiety contribute to the development and maintenance of paranoia. Specifically, core beliefs of social anxiety include (1) conditional beliefs: negative self-appraisals dependent on performance (2) unconditional beliefs: negative self-appraisals independent of behavior, and (3) high-standard beliefs: the attribution of self-worth based on performance (Wong, Moulds & Rapee, 2014). There is a need for research to determine the association between paranoia and beliefs related to social anxiety.
The aim of the current study is to identify whether cognitions thought to be central to social anxiety are also related to paranoia in a sample of those with psychosis. We hypothesize that (1) social anxiety and paranoia will be related, and (2) heightened maladaptive self-beliefs of social anxiety will be associated with increased paranoia.
**Methods:** We will use the Social Interaction Anxiety Scale (Heimberg, Mueller, Holt, Hope & Liebowitz, 1992) to measure social anxiety symptomatology. We will use the Self-Beliefs Related to Social Anxiety Scale (Wong, Moulds & Rapee, 2014) including three subscales: conditional beliefs, unconditional beliefs, and high standard beliefs, to quantify cognitions of social anxiety. To measure paranoia, we will use the Green Paranoid Thoughts Scale (Green et al., 2008).
**Results:** Preliminary analyses ($N = 14$) indicate that the social anxiety is robustly related to paranoia ($r = 0.59$, $p < 0.05$). Unconditional beliefs were related to paranoid thoughts ($r = 0.62$, $p < 0.05$) and paranoia was moderately correlated to conditional beliefs ($r = 0.35$, $p = 0.24$) and total self-beliefs related to social anxiety scores ($r = 0.39$, $p = 0.19$), but these relations were not statistically significant. Additional data will be available at the time of presentation.
**Discussion:** Results of this research will both inform the development of paranoia and allow for a better understanding of the relation between paranoia and social anxiety. Specifically, cognitive mechanisms underlying their relation will be illuminated.

### S76. A BEHAVIOURAL ECONOMIC ANALYSIS OF EFFORT-RELATED CHOICE IN SCHIZOPHRENIA

Gagan Fervaha*[,1], Ofer Agid[2], George Foussias[2], Hiroyoshi Takeuchi[2], Konstantine Zakzanis[3], Ariel Graff-Guerrero[2], Gary Remington[2]
*[1]Queen's University Faculty of Medicine; [2]Centre for Addiction and Mental Health (CAMH), University of Toronto; [3]University of Toronto*

**Background:** Motivational deficits are prevalent feature of schizophrenia, which have been tightly linked to real-world outcomes. Abnormalities in effort cost computations have been proposed as a candidate mechanism underlying these deficits. In the present study, we sought to employ behavioural economic analyses to further understand cost-benefit decision making abnormalities in schizophrenia.
**Methods:** 58 young adults with schizophrenia and 58 matched controls participated in this study. Participants completed an effort-based decision-making task in which they made decisions to expend physical effort in exchange for monetary rewards. From participants choice behaviour, we computed indifference values (i.e. the reward value at which participants would be indifferent to expending effort vs not) for each individual participant. Other computational parameters were also computed such as choice consistency and subjective reward valuation.
**Results:** Patents and controls did not differ in their subjective valuation of reward. On the decision-making task, patients made more inconsistent choices relative to controls. In both univariate and multivariate analyses controlling for potential confounders, patients had higher indifference values meaning that patients required more money in the exchange of their effort. Among patients, higher indifference values were associated with more severe clinical motivational deficits.
**Discussion:** Patients had multiple abnormalities related to their decisions to expend effort for reward. Choices were more chaotic and reward value was discounted by effort at a steeper rate in patients. These results point toward an abnormality in the computation of effort costs or in the integration of these costs with value signals.

### S77. JUMPING TO CONCLUSIONS AND FACIAL EMOTION RECOGNITION IMPAIRMENT IN FIRST EPISODE PSYCHOSIS ACROSS EUROPE

Giada Tripoli*[,1], Diego Quattrone[1], Charlotte Gayer-Anderson[1], Victoria Rodriguez[1], Natashia Benzian-Olsson[1], Laura Ferraro[2], Caterina La Cascia[2], Crocettarachele Sartorio[2], Lucia Sideli[2], Fabio Seminerio[2], Daniele La Barbera[2], Craig Morgan[1], Pak Sham[3], Marta Di Forti[1], Robin Murray[1]
*[1]Institute of Psychiatry, Psychology & Neuroscience, King's College London; [2]University of Palermo; [3]The University of Hong Kong*

**Background:** Jumping to conclusions (JTC) is a well-established reasoning and data gathering bias found in patients with psychosis even at illness onset (First Episode Psychosis, FEP). Preliminary work in this field focused primarily on the association with delusions, although jumping to conclusions has also been found in non-deluded schizophrenia patients after remission, and in individual with at risk mental state.
Moreover, psychotic patients tend to show impairments in social cognition, struggling in identifying, processing and interpreting social clues. Deficits in facial emotion recognition (FER) – a key component of the construct – represent a well-replicated finding in schizophrenia. Furthermore, deficits in global facial affect recognition have been found in FEP with the same severity as at further stages, especially for anger recognition. The present study aims to measure JTC and FER bias in a sample of FEP recruited across 5 European countries, compared with healthy controls.
**Methods:** Data on JTC (Beads task 60:40), FER (Degraded Facial Recognition task – DFAR) and socio-demographics have been analysed in a sample of 643 FEP and 1019 population controls recruited as part as the EU-GEI study across UK, Netherlands, France, Spain, and Italy.
IQ scores were used to exclude cases and controls with current IQ<70 (N=171) from JTC analysis and a score <41 (N=384) on the Benton Facial Recognition test for the analysis on DFAR. Logistic regression model was applied to predict case/control status using 1) JTC and 2) DFAR as predictive variables controlling for age, gender and country.
**Results:** We showed that the presence of JTC bias varies across different countries both in cases ($\chi2=23.77$ $p<0.001$) and controls groups ($\chi2=14.01$ $p=0.007$).
Logistic regression analyses revealed JTC to be a significant predictor of case/control status (Adj OR=1.88 CI 95%=1.43–2.29 $p<0.001$).
As well as JTC, FER differed over Europe in both groups (FEP, total: $F=17.37$, $p<0.001$; neutral: $F=12.4$, $p<0.001$; happy: $F=25.62$, $p<0.001$; frightened: $F=8.78$, $p<0.001$; angry: $F=5.48$, $p<0.001$. Controls, total: $F=23.06$, $p<0.001$; neutral: $F=21.72$, $p<0.001$; happy: $F=21.74$, $p<0.001$; frightened: $F=14.14$, $p<0.001$; angry: $F=12.49$, $p<0.001$).
Logistic regression analyses revealed all DFAR scores, except for happy emotions, to be negatively associated with case/control status (total: B=-.0182 $p=0.001$; neutral: B=-.054 $p=0.003$; happy: B=-.0196 $p=0.2$; frightened: B= -.065 $p<0.001$; angry: B=-.030 $p=0.04$).

**Discussion:** This study supports the evidence that 1) FEP patients are more likely to present JTC and FER impairments than controls; 2) cognition and social cognition might represent transcultural features of psychotic disorders.

### S78. EXAMINING SEMANTIC AND EPISODIC MEMORY IN SCHIZOPHRENIA USING THE HOPKINS VERBAL LEARNING TASK

Erica Neill*[,1], Susan Rossell[2]
*[1]University of Melbourne; [2]Swinburne University*

**Background:** Schizophrenia is associated with deficits in both episodic and semantic memory however, our understanding of how the deficits in each system independently contribute to overall memory performance is poorly understood.
The Hopkins Verbal Learning Task (HVLT) is a memory task using a single word list. To perform the task successfully, participants need to use both episodic and semantic abilities. Both episodic and semantic clustering scores can be calculated which provide nuanced information about the memory encoding and retrieval techniques used by those performing the task.
**Methods:** Sixty schizophrenia patients and sixty healthy controls were compared in their performance on the HVLT. In addition to analysing immediate recall, learning slope, delayed recall and recognition, semantic and episodic clustering were also compared. Further, given the link between thought disorder and semantic function, this symptom was correlated with memory performance measures.
**Results:** The schizophrenia group demonstrated worse performance across learning trials, delayed recall, and recognition indicating a generalised memory problem. Clustering scores were used to probe into semantic and episodic function specifically. The schizophrenia group demonstrated normal episodic clustering in the face of significantly impaired semantic clustering. Further, semantic clustering performance positively correlated with all general memory measures whilst episodic clustering did not. Finally, thought disorder did not correlate with any HVLT performance measure apart from semantic clustering.
**Discussion:** It is difficult to tease apart the contributions of semantic and episodic memory impairments to poor overall memory function in schizophrenia. In this study, we have first demonstrated intact episodic clustering in the face of impaired semantic clustering. Then, by correlating semantic and episodic clustering scores with general memory performance measures, we were able to demonstrate that semantic memory performance is more significantly related to overall memory performance than episodic performance. Finally, this result supports the specificity of the relationship between thought disorder and semantic memory impairment.

### S79. ENHANCING WORKING MEMORY IN SCHIZOPHRENIA USING 1MA AND 2MA TRANSCRANIAL DIRECT STIMULATION TO THE LEFT DORSOLATERAL PREFRONTAL CORTEX

Irina Papazova*[,1], Wolfgang Strube[1], Benedikt Becker[1], Bettina Henning[1], Tobias Schwippel[2], Andreas Fallgatter[3], Frank Padberg[1], Ulrich Palm[1], Peter Falkai[1], Christian Plewnia[2], Alkomiet Hasan[1]
*[1]Ludwig Maximilian University Munich; [2]University Hospital Tuebingen; [3]University of Tuebingen*

**Background:** Cognitive impairment is a key symptom of schizophrenia, causing patients' occupational disability and worsening their life quality. Yet, the treatment options are still scarce. Recent research suggests that transcranial direct stimulation (tDCS) to the dorsolateral prefrontal cortex (DLPFC) could enhance a crucial cognitive process such as working memory. Here, for the first time we examined the effects of tDCS on simultaneous working memory performance in schizophrenia patients in regard of stimulation intensity and cognitive load.
**Methods:** Forty schizophrenia patients (N = 40) participated in two separate double-blind, sham-controlled experiments, both consisting of a pre-stimulation baseline, an active anodal and a sham tDCS single-session. Stimulation application was conducted to the F3 (anode) and to the right deltoid muscle (cathode) for 21 min. In Experiment 1 (N = 20) patients received tDCS at 1 mA and Experiment 2 (N = 20) – at 2 mA. In total, 120 experimental sessions were performed. Working memory was measured during stimulation using a verbal n-back task with three cognitive loads - 1-back, 2-back, 3-back. Applying the Signal Detection Theory, we estimated the discriminability index d prime, which together with reaction times served as study outcomes. Using several RM-ANOVAs we compared working memory performance during sham and active tDCS across all cognitive loads for each experiment. In a subsequent mixed-model RM-ANOVA, we pooled data from both experiments and analyzed differences in working memory performance in regard of stimulation intensity.
**Results:** Data analysis showed significant greater d prime values during active tDCS than during sham tDCS only in Experiment 1 ($F1, 19 = 4.48$, $p = .048$). In Experiment 2, there was a numeric improvement of d prime during tDCS that however did not reach significance ($F1, 19 = 2.31$, $p = .145$). The subsequent mixed-model RM-ANOVA revealed a significant overall effect of brain stimulation, prompting higher d prime values ($F1, 38 = 6.05$, $p = .019$), but no main of stimulation intensity ($p = .392$). Analysis on reaction times revealed no significant results.
**Discussion:** This is the first study comparing the online effects of 1mA and 2mA tDCS on working memory in schizophrenia patients. In line with previous research, tDCS improved working memory functioning in schizophrenia. However, this enhancement did not differ between stimulation intensities, implying that tDCS effects on cognition could be dose independent. Overall, our results provide further evidence that tDCS may be an effective and feasible intervention for cognitive impairment in schizophrenia and underline the need for future research on the specific stimulation parameters.

### S80. NEUROCOGNITIVE FUNCTIONING IN YOUTH AT RISK OF SERIOUS MENTAL ILLNESS

Sylvia Romanowska*[,1], Glenda MacQueen[1], Benjamin I. Goldstein[2], JianLi Wang[3], Sidney H. Kennedy[4], Signe Bray[1], Catherine Lebel[1], Jean Addington[1]
*[1]Hotchkiss Brain Institute, University of Calgary; [2]University of Toronto; [3]Institute of Mental Health Research, University of Ottawa; [4]University Health Network, Toronto*

**Background:** Neurocognitive deficits are associated with many serious mental illnesses (SMI), including schizophrenia, bipolar disorder, and major depressive disorder, and have been found to negatively impact social and occupational outcomes, clinical prognosis, and overall quality of life. These deficits have also been observed in people in earlier phases of schizophrenia, specifically in young people at clinical high risk (CHR) of psychosis. In these youth, neurocognitive deficits present at a level intermediate to healthy controls and those with early psychosis, indicating that mild impairments in neurocognitive functioning may be early markers of illness development. It is possible that neurocognitive deficits may be present in young people at risk of a range of SMI beyond the psychosis-spectrum, including affective and anxiety disorders. The aim of this study was to compare neurocognitive functioning in a sample of youth at risk of SMI across the different clinical stages described by McGorry and colleagues and compare them to healthy controls (HCs). It was hypothesized that participants in the later stages of risk, characterized by the presence of subthreshold psychiatric symptoms or attenuated syndromes, would exhibit impairments in neurocognitive performance compared to HCs and asymptomatic youth at familial high risk.

**Methods:** This was an observational, cross-sectional study of 243 male and female individuals between the ages of 12–26. The sample consists of participants in the Canadian Psychiatric Risk and Outcome Study (PROCAN) and included: asymptomatic participants at familial high risk for SMI (Stage 0; n=41); youth with early mood or anxiety symptoms (Stage 1a; n=52); youth with attenuated psychotic or affective syndromes and distress (Stages 1b; n=108); and HCs (n=42). The neurocognitive battery included the WRAT-4 reading task, WASI Vocabulary and WASI Matrix Reasoning tasks, and the Measurement and Treatment Research to Improve Cognition in Schizophrenia (MATRICS) Consensus Cognitive Battery (MCCB). All neurocognitive tasks were administered at baseline. Group differences in neurocognitive performance were analyzed using MANCOVA/ANCOVA analyses. Covariates included age and sex.
**Results:** Subjects in Stage 0 and Stage 1a did not significantly differ from any group. Subjects in Stage 1b (attenuated syndromes) had significantly lower neurocognitive scores in the domains of speed of processing, working memory, attention/vigilance and reasoning and problem solving, and on composite scores of neurocognitive performance and full-scale IQ compared to HCs. A secondary analysis demonstrated that subjects in Stage 1b who met CHR status according to Criteria of Psychosis-risk Syndromes (n=83) had lower scores in the domains of working memory, verbal learning, and reasoning and problem solving and on the overall composite score than the other participants in Stage 1b who did not meet CHR criteria.
**Discussion:** This study provides evidence for a growing literature which suggests that neurocognitive deficits may be markers of susceptibility for SMI development. It also increases what is known about neurocognitive performance associated with different stages of risk for SMI. Identification of such impairments could aid with detection of early mental health problems prior to illness onset.

### S81. NEUROCOGNITIVE FUNCTIONING IN SCHIZOPHRENIA AND BIPOLAR DISORDER DURING THE REMISSION AND THE PSYCHOTIC STATES

Deniz Ceylan*[,1], Berna Binnur Akdede[2], Emre Bora[2], Ceren Hıdıroğlu[2], Zeliha Tunca[2], Köksal Alptekin[2], Ayşegül Özerdem[2]
*[1]Izmir University of Economics; [2]Dokuz Eylül University, School of Medicine*

**Background:** Previous literature comparing cognitive functioning between bipolar disorder (BD) and schizophrenia (Sch), particularly focused on remitted patients with BD (i.e. euthymics) and clinically stable patients with Sch; and suggested milder cognitive impairment in BD in comparison to Sch. Acute psychotic symptoms may lead poorer cognitive functioning in both disorders. Limited evidence suggests milder deficits in psychotic mania than in acute psychosis in Sch. We aimed to compare cognitive functioning in Sch and BD during the remission and the psychotic states.
**Methods:** Several domains of cognitive functioning were compared among patients with BD who had a history of psychosis [32 with a current psychotic manic episode, 44 in euthymia for at least 6 months] and patients with Sch [41 with psychotic symptoms, 39 remitted according to Andreassen et al. criteria (2006)] in comparison to 55 healthy controls (HC). Participants performed a cognitive battery including Wisconsin Card Sorting, Rey Auditory Verbal Learning, Stroop, Auditory Consonant Trigram, Trail Making, Digit Span, Controlled Word Association, Category Fluency and Digit Symbol tests.
Principal components analyses were performed to extract the 'global cognition' factor and for dimensionality reduction to identify neurocognitive domains among patients with BD and Sch. The optimum number of cognitive components was identified by inspecting the scree plot. Each factor score was assessed for normality by calculating tests of skewness and kurtosis. The factor scores were compared between patients with Sch and patients with BD using two-way analyses of variance (ANCOVA) adjusting for age. Pairwise comparisons with Bonferroni corrections were used for post-hoc analysis.
**Results:** Mean age, sex ratio levels of education were similar among patients with BD, patients with Sch and HCs. Principal components analyses revealed a global cognition factor that explains 52.6% of variance and a subsequent PCA revealed 5 factor domains including processing speed, verbal memory, visual memory, working memory and planning. Both patients with BD and patients with Sch have significantly poorer global cognition ($p<0.001$; $p<0.001$), processing speed ($p<0.001$; $p<0.001$); verbal memory ($p<0.001$; $p=0.007$); visual memory ($p=0.033$; $p=0.016$) and planning ($p=0.011$; $p=0.006$) than HCs. Patients with BD presented higher scores in global cognition, processing speed ($p=0.010$) and verbal memory ($p=0.011$) than patients with Sch ($p<0.001$). Global cognition and processing speed domains differ among groups with respect to both diagnosis [$F=18.466$, $p<0.001$; $F=7.864$, $p=0.006$] and state [$F=8.910$, $p=0.001$; $F=3.958$, $p=0.048$]. Processing speed, but not other components, displayed a significant interaction between diagnosis and state [$F=14.808$, $p<0.001$)].
**Discussion:** Deficits in global cognition was milder in BD, than those in Sch in both the remission and the psychotic states. Although diagnosis seems to be the major factor affecting the cognitive performance, our data present significant interactions of diagnosis and state in processing speed.

### S82. GOAL MANAGEMENT TRAINING OF EXECUTIVE FUNCTIONS FOR PATIENTS WITH SCHIZOPHRENIA OR HIGH RISK OF SCHIZOPHRENIA: BASELINE CHARACTERISTICS AND PRELIMINARY RESULTS FROM AN RCT

Ingvild Haugen*[,1], Elisabeth Haug[1], Torill Ueland[2], Jan Stubberud[3], Merete Øie[1]
*[1]Innlandet Hospital Trust; [2]Oslo University Hospital; [3]Lovisenberg Diaconal Hospital, University of Oslo*

**Background:** About 85% of patients with schizophrenia have cognitive impairments, executive functions being particularly affected. Executive dysfunctions are important predictors of functional outcomes. Unlike psychotic symptoms, cognitive deficits do not improve during periods of remission and change only minimally with antipsychotic medications. Thus, effective interventions aimed at improving executive functions in this population are needed.
One of the most validated interventions for executive dysfunction is Goal Management Training (GMT). GMT is a compensatory intervention that relies on metacognitive strategies for improving participants' ability to organize and achieve goals in everyday life. GMT has received empirical support in studies of other populations, such as people with neurological conditions and in healthy elderly. To our knowledge no previous studies have investigated the effect of group-based GMT in patients with schizophrenia spectrum disorders or with high risk of schizophrenia. Thus, this is the main objective of the study. Baseline characteristis and preliminary results from the first patients will be presented.
**Methods:** Participants (16–67 years, males and females, IQ >70) with executive dysfunction, will be recruited among patients referred for treatment at Innlandet Hospital Trust in Norway from 2017 to 2020. The study aims to include patients treated for psychotic disorder for less than 5 years and new patients who either have symptoms that meet the DSM-IV criteria for a diagnosis of broad schizophrenia spectrum disorder or who are considered at high risk of psychosis. We aim to recruit one hundred participants for the current randomized controlled trial (RCT), with efficacy of GMT (n = 50) being compared with results of subjects in a wait-list condition (WL, n = 50).
Measurements include self-report of executive function, emotional health, and social- and everyday function. Informant reports of executive function will also be collected. Furthermore, neuropsychological tests designed specifically to measure areas of executive function will be utilized, as well as role-playing tasks thought to have good ecological validity. Symptoms of psychosis will also be assessed. GMT will be administered in 9 (twice weekly) x 2 hour sessions in accordance with the GMT research protocol. A general linear model with repeated measures analysis of variance (RM ANOVA) will be used to examine differential group treatment effects. A 2 x 3 mixed-design will be applied, with Group (GMT, WL) as between-subjects factor, and Session (baseline [T1], post-intervention [T2], and 6 months follow-up [T3]) as within-subjects factor. Interpretation of the strength of experimental effects will be provided with effect size statistics.
**Results:** Baseline characteristics and preliminary results from the first participants will be presented.
**Discussion:** Based on findings from previous GMT-studies, we hypothesize that post-intervention changes will be reflected in improved scores on self-reported and/or objective measures of executive functions (particularly in the areas of planning and attentional control) compared to patients in WL. We also expect that GMT participants will improve their goal attainment in everyday life and social functioning after the intervention. Additionally, we expect post-intervention changes to be reflected in improved scores on measures of emotional health.

### S83. THE IMPACT OF COENZYME Q10 ON THE COGNITIVE DEFICITS AND SYMPTOMS OF SCHIZOPHRENIA: PROTOCOL AND BASELINE DATA OF A RANDOMISED, PLACEBO-CONTROLLED STUDY

Áine Maguire*[,1], April Hargreaves[1], Michael Gill[1]
[1]*Trinity College Dublin*

**Background:** CoQ10 is a vital component of mitochondrial function and metabolism, and its deficiency creates greater vulnerability to disease due to impaired mitochondrial energy generation and cellular antioxidant capacity. CoQ10 functions as an electron carrier within the mitochondrial electron transport chain during cellular respiration. Schizophrenia is a disorder with documented CoQ10 deficiency and mitochondrial dysfunction, and cellular respiration and mitochondrial network dynamics can be impaired due to altered complex 1 activity in the disorder. Key features of schizophrenia such as depression, fatigue and cognitive impairment have been independently associated with mitochondrial dysfunction and increased oxidative stress. In bipolar disorder, fibromyalgia, chronic fatigue syndrome and multiple sclerosis these symptoms have been effectively reduced through CoQ10 supplementation. We assess the impact of CoQ10 supplementation in individuals with a diagnosis of schizophrenia through a double-blind, randomised, placebo-controlled study.
**Methods:** Approximately 300 participants with a DSM-IV diagnosis of schizophrenia or schizoaffective disorder, with no neurological or psychiatric co-morbidity will be recruited to this study. Participants will be randomised to take 100 mg dose capsules of CoQ10 or placebo three times daily for six months, and undergo neuropsychological and cognitive testing at three time points (baseline, midpoint, six months post-randomisation). Changes in participants' global cognitive function, sustained attention, working memory, processing speed, negative symptoms, levels of depression and anxiety, fatigue, blood pressure, quality of life and functional status following CoQ10 supplementation will be assessed. Blood samples are also taken at each assessment session to assess baseline and changes in levels of plasma CoQ10 and mitochondrial function via lactate analysis.
**Results:** Currently baseline data is available for 42 participants (mean age = 50.2, SD=10.7). All participants either have a clinical diagnosis of schizophrenia (n=34) or schizoaffective disorder (n=8). The mean estimated IQ of the group is 92.4 (SD=20.5), and participants have a median of 13 years in education. Thirty-nine percent of participants reported mild to severe levels of depression and twenty-three percent reported moderate or severe levels of anxiety. Seventy-three percent of participants reported good to very good quality of life. FACIT-fatigue scores were negatively correlated with both depression and anxiety scores, such that greater fatigue levels were associated with higher levels of depression (r=-.484, p<0.01) and anxiety (r=-.539, p<0.01).
**Discussion:** CoQ10 is a mitochondrial agent that plays a fundamental role in energy production and mitochondrial function. The available baseline data suggest a relationship between fatigue and depression and anxiety levels in individuals with a diagnosis of schizophrenia. CoQ10 supplementation has the potential to affect these symptoms, through CoQ10's ability to restore electron flow in the electron transfer chain and increase mitochondrial antioxidant capacity. The study commenced in November 2016 and patient enrolment and assessment is ongoing. Updated baseline information will be presented including further cognitive assessments. To minimise risk of bias while recruitment and assessments are ongoing, unblinding and outcome analysis will not be conducted at time of presentation.

### S84. NEUROPSYCHOLOGICAL FUNCTIONING AS A PREDICTOR OF PSYCHOLOGICAL RESILIENCE: PRELIMINARY RESULTS FROM THE PRONIA STUDY

Alexandra Stainton*[,1], Katharine Chisholm[1], Rachel Upthegrove[1], Stephen Wood[2], PRONIA Study Group
[1]*University of Birmingham;* [2]*Orygen, the National Centre of Excellence in Youth Mental Health*

**Background:** Resilience provides a new understanding of the highly variable trajectories of mental illness, and has consistently been linked with improved mental health outcomes. Resilience is largely defined as the presence of additional factors which overcome a specific risk for mental illness, leading to ultimately more positive outcomes than expected given said risk. Previous research in the area has focused on identifying psychological factors which may be associated with resilience. Moving forwards, it is essential that researchers investigate how resilience may function in different domains. The aim for the present research was to conduct a preliminary investigation into the possible role of neuropsychological performance in resilience using data from the PRONIA study.
**Methods:** Participants were individuals aged 15–40 who were recruited into the PRONIA study. Total scores for the Resilience Scale for Adults (RSA), assessing self-report psychological resilience, were available for 587 participants. The sample included individuals with first-episode psychosis (N=113), first-episode depression (N=118), individuals at ultra-high risk for psychosis (N=109), and healthy controls (N=247). Participants also completed a comprehensive neuropsychological test battery which assessed performance in the following domains: IQ, executive functioning (EF), processing speed (PS), sustained attention, working memory, visual memory, social cognition, motivational salience, and verbal learning and memory.
**Results:** A stepwise multiple linear regression was used to identify which of the neuropsychological domains would best predict RSA total score. The final model significantly predicted RSA total score, explaining 4% of the variance in these scores, $F(2, 512) = 12.37$, $p < 0.001$. The model indicated that higher RSA total was associated with PS ($\beta$=3.35, p=.032) and EF ($\beta$=4.15, p=0.046). EF provided the highest relative contribution in the model, with every 1 point increase resulting in 4.15 standard deviation increase in RSA total.
**Discussion:** The present results suggest that neuropsychological performance has a small, but significant relationship with psychological resilience. The two neuropsychological domains which best predicted this outcome were PS and EF. Resilience has been argued to be a highly dynamic process, by which individuals must utilise assets and resources to their benefit. Furthermore, the effectiveness of such factors will vary across time and circumstance, adding to the flexibility required to navigate this process. These results support this conceptualisation of resilience, as EF is thought to involve the organisation and execution of complex thoughts and behaviour. Processing speed has also been found to affect other cognitive functions such as reasoning. These neuropsychological processes may aid an individual's ability to utilise protective factors to their benefit during a period of adversity or risk. These results are preliminary, and future research should look to replicate and extend this research to form a multi-modal model of resilience. A deeper understanding of the mechanisms underlying this process can then inform future intervention strategies.

### S85. THE EFFECT OF LONG-TERM SOCIAL DEPRIVATION ON EFFORT ALLOCATION PATTERN IN PATIENTS WITH SCHIZOPHRENIA

Min-yi Chu*[,1], Hui-xin Hu[1], Hua Ni[2], Wei-hong Lu[3], Simon S.Y. Lui[4], Eric F.C. Cheung[4], Zhen-hui Yi[3], Raymond Chan[6]
*[1]Shanghai Jiao Tong University School of Medicine; [2]Shanghai Xuhui Mental Centre; [3]Shanghai Mental Health Centre, Shanghai Jiao Tong University School of Medicine; [4]Castle Peak Hospital; [6]Chinese Academy of Sciences*

**Background:** Motivational deficit is a common feature of negative symptoms in patients with schizophrenia. Patients with schizophrenia are impaired in goal-directed behaviour and effort allocation decision-making to pursue a potential reward. On the other hand, limited work has suggested that schizophrenia patients who experienced long-term social deprivation showed more severe negative symptoms. However, it is not yet fully clear the long-lasting impact of long-term social deprivation on motivation in these patients. The current study aimed to investigate the effect of long-term social deprivation on effort allocation pattern in patients with schizophrenia.
**Methods:** We recruited 21 patients with schizophrenia institutionalized for more than 15 years and 20 patients with schizophrenia dwelling in the community and 24 healthy controls for this study. We administered the Effort-Expenditure for Rewards Task (EEfRT) to capture reward-based motivational salience, which requires participants make decision to choose a hard or easy task based on reward probability and magnitude. Moreover, a set of self-reported checklists including the Chapman Psychosis Proneness Scales, the Temporal Experience of Pleasure Scale, the Anticipatory and Consummatory Interpersonal Pleasure Scale and the Emotional Expressivity Scale were also administered to all the participants. For patients with schizophrenia, they also received rating score on the Positive and Negative Syndrome Scale (PANSS) and the Scale for the Assessment of Negative Symptoms (SANS).
**Results:** Institutionalized patients had exhibited significantly more prominent negative symptoms, especially in alogia subscale, attention subscale, and a trend of statistical significance in anhedonia subscale of SANSS. The two clinical groups did not differ in positive symptoms subscale and general psychopathology symptoms subscale of PANSS. Findings from one-way ANOVA analysis showed that both institutionalized patients and community-dwelling patients with schizophrenia did not differ from healthy controls in experiential pleasure and emotion expression. For performance in the EEFRT, amotivation was only observed in institutionalized patients with schizophrenia, they were significantly less likely to expend effort to pursue a potential reward than healthy controls in both medium (50%) probability and high (80%) probability level. Hence, as the reward probability increased, unlike healthy controls, institutionalized patients could not increase their hard task choices.
**Discussion:** Institutionalized patients with schizophrenia exhibited significantly more motivational deficits than healthy controls, and such impairment was not observed in community-dwelling patients. However, both institutionalized patients and community-dwelling patients with schizophrenia showed no deficits in self-reported scales measuring pleasure experience and expression. These findings further revealed that long-term social deprivation may be a vital contributor to severe motivation deficits of patients with schizophrenia.

### S86. EXAMINING REASONING BIASES IN SCHIZOPHRENIA USING A MODIFIED "JUMPING TO CONCLUSIONS" TASK

Hans Klein*[,1], Amy Pinkham[1]
*[1]University of Texas at Dallas*

**Background:** The Jumping To Conclusion (JTC) bias has been extensively studied in relation to schizophrenia and persecutory delusions. It is suggested that performance on the traditional JTC task relates to a pervasive bias to make decisions quickly, contributing to delusion formation. However, the mechanisms underlying performance on this task, as well as the relationship between the JTC bias and other reasoning biases implicated in delusional ideation, is not fully understood. We examined the relationship between several biases believed to be involved in delusion formation and maintenance to further clarify potential co-occurrences of these biases and their relation to delusional ideation.
**Methods:** In order to assess the co-occurrence of reasoning biases in decision making, we modified the traditional JTC task in order to assess a number of previously identified biases that may be implicated in delusion formation and maintenance. 46 participants with schizophrenia and 46 healthy controls completed two versions of the modified task utilizing neutral (blue and red beads in a jar) and salient (negative and positive comments in a list) stimuli, both with 60:40 ratios.
**Results:** 2 x 2 mixed ANOVAs were performed on each of the modified variables using group [patients vs. controls] as a between subjects variable and task type [neutral vs. salient] as a within subject variable. We replicated previous findings of main effects of a JTC bias for group, $F(1, 90) = 4.149$, $p = .045$, $\eta 2p = .044$, and task type, $F(1, 90) = 4.724$, $p = .032$, $\eta 2p = .050$ such that patients showed a greater JTC bias, and in both groups, the JTC bias was more pronounced for the salient task. However, a main effect of group was also evident for number of illogical judgments, $F(1, 90) = 11.596$, $p = .001$, $\eta 2p = .114$, indicating that patients showed greater difficulty in probabilistic reasoning. When controlling for probabilistic reasoning ability, the group main effect for the JTC bias disappeared, $F(1,89) =0.169$, $p = 0.682$, $\eta 2p = 0.002$. None of our modified variables significantly correlated with symptom severity within our patient population.
**Discussion:** While we were not able to correlate our modified variables with symptoms of schizophrenia, we were able to observe a pattern of group differences that may help further understand decision-making processes in individuals with schizophrenia. Our findings that faulty probability assessment accounts for the JTC bias indicates that the traditional JTC bias task may not represent an inherent hasty decision making bias, but rather an inability to fully understand and execute the stated goals of the task. These results call into question the current understanding of the JTC bias and the independence of this bias apart from the cognitive demands of the task.

### S87. THE INITIAL CHANGE IN THE SERUM LEVEL OF C-REACTIVE PROTEIN IN ACUTE PSYCHOSIS IS ASSOCIATED WITH COGNITIVE PERFORMANCE IN LATER PHASES

Farivar Fathian*[,1], Else-Marie Løberg[2], Rolf Gjestad[3], Vidar Martin Steen[4], Rune Kroken[2], Hugo A. Jørgensen[4], Erik Johnsen[2]
*[1]NKS Olaviken Gerontopsychiatric Hospital; [2]University of Bergen, Haukeland University Hospital; [3]Haukeland University Hospital; [4]University of Bergen*

**Background:** Inflammatory processes have been implicated in the pathophysiology of schizophrenia and related psychosis and could be particularly relevant to the associated cognitive deficits. The C-reactive protein (CRP) serves as a general marker of inflammation, and inverse relationships between CRP levels and cognitive performance in acute psychosis have been demonstrated. Here we aimed to investigate how the serum level and initial change of CRP in acutely admitted patients with psychosis were correlated with cognitive performance during a 6-months follow-up period.
**Methods:** The study is part of a pragmatic, randomized trial comparing four different second-generation antipsychotic drugs, and consists of 208 acute phase patients recruited at admittance for psychosis (schizophrenia, schizoaffective disorder, acute and transient psychotic disorder, delusional disorder, drug-induced psychosis, bipolar disorder except for manic psychosis, or major depressive disorder with psychotic features). The present study reports data for all treatment groups collectively, and does not compare treatment groups.
Measurements of CRP and cognitive performance were conducted at baseline (T1) and after an average of 4 weeks after inclusion (T2). Cognition was assessed after 3 months (T3) and 6 months (T4) of follow-up.
**Results:** Global cognition improved during the follow-up period of 6 months, especially in the T1–T2 interval. The different cognitive subdomains showed different time-dependent profiles of improvement, with memory and attention improving significantly also in the later phases. Reduction of the CRP level during the initial follow-up interval (T1–T2) was associated with increased overall cognitive performance in the T2–T4 interval, but not in the T1–T2 interval. For the cognitive subdomains, we found an inverse association between change in CRP level and learning (T1–T2 interval), verbal abilities (T2–T4 interval), and attention (T2–T3 interval).
**Discussion:** The main finding of the present study was that the global cognitive performance continued to improve from the initial phase (baseline to 4 weeks) of acute psychosis to the later phase (4 weeks–6 months), and was predicted by the change in CRP level that was observed during the initial phase (baseline–4 weeks) of the treatment. These findings might indicate a prolonged effect of inflammatory processes on cognition in acute psychosis, stretching beyond the initial phase.
There is substantial evidence that inflammatory processes are involved in the cognitive performance in psychosis. CRP levels have been associated with cognitive impairment in patients with schizophrenia.
**Conclusions:** These findings indicate that initial changes in the serum level of CRP in the acute phase of psychosis may predict cognitive functioning in later phases of the disease.

### S88. GRANDIOSE IDEAS IN SCHIZOPHRENIA: THE ROLE OF OPTIMISM BIAS AND HALLUCINATIONS

Catherine Bortolon*[,1], Delphine Capdevielle[2], Hanan Yazbek[2], Joanna Norton[3], Stéphane Raffard[1]
*[1]University of Montpellier; [2]Hospital La Colombière, CHU Montpellier; [3]INSERM*

**Background:** Grandiose delusions (GDs) are defined as false beliefs about having an inflated worth, power, or a special identity which are firmly sustained despite undeniable evidence to the contrary. GDs have received little attention although it is the second most commonly encountered delusional beliefs (Stompe, et al., 2006). Consequently, there is no intervention program targeting GD and only few studies have explored the psychological processes underpinning GDs. Considering that delusions have an origin in autobiographical memory (Berna et al., 2017) and thus also in how individuals project themselves into the future, the aim of this present study was to explore the role of future projection, sensibility to reward and punishment, and anticipatory pleasure in GDs in schizophrenia (SZ) disorder.
**Methods:** 133 SZ patients completed measures of positive and negative symptoms, sensibility to reward and punishment, anticipatory pleasure, depression, and an experimental task in which individuals were asked to project themselves into positive, negative and neutral future situations and evaluate to what extent they believed the situation would happen in the future.
**Results:** For the first set of analyses (Independent samples test), the sample was divided into two groups according to GD scores. Patients with higher GD scores (PANSS 5 score > 3; High GG M = 4; Low GD M = 1) obtained higher scores on sensibility to reward and self-future projection into positive situations as well as on positive symptoms. No significant differences were found regarding the other measures. Secondly, positive symptoms, sensibility to reward and positive future expectations were entered in the regression analysis. Results showed that hallucinations (B = 0.359, p = 0.0001) and positive future expectations (B = 0.216, p = 0.011) were significantly associated with GD (R2 = 0.317, p = 0.009).
**Discussion:** This present study showed that sensibility to reward and especially higher optimism bias for the future may be important psychological processes associated with GDs in SZ patients. Optimism bias for the future may play a role in amplifying and reinforcing elated mood built upon preexisting inflated (or accurate) perceptions of self (Freeman & Garety, 2003). Together with cognitive bias such as jumping to conclusions (Garety et al., 2012), they may provide evidence for the plausibility of GDs. Concerning the association between GDs and hallucinations, it is possible that patients experiencing hallucinations may interpret this phenomenon as a kind of special ability or power, resulting in turn in GDs maintenance.
**References:**
1. Berna, F., Evrard, R., Coutelle, R., Kobayashi, H., Laprévote, V., &Danion, J. M. (2017). Characteristics of memories of delusion-like experiences within the psychosis continuum: Pilot studies providing new insight on the relationship between self and delusions. Journal of behavior therapy and experimental psychiatry, 56, 33–41.
2. Freeman, D., & Garety, P. A. (2003). Connecting neurosis and psychosis: the direct influence of emotion on delusions and hallucinations. Behaviour research and therapy, 41(8), 923–947.
3. Garety, P. A., Gittins, M., Jolley, S., Bebbington, P., Dunn, G., Kuipers, E., … & Freeman, D. (2012). Differences in cognitive and emotional processes between persecutory and grandiose delusions.Schizophrenia bulletin, 39(3), 629–639;
4. Stompe, T., Karakula, H., Rudalevičiene, P., Okribelashvili, N., Chaudhry, H. R., Idemudia,E. E., et al. (2006). The pathoplastic effect of culture on psychotic symptoms inschizophrenia.World Cultural Psychiatry Research Review, 157−163.

### S89. THE IMPACT OF COGNITIVE REMEDIATION ON COGNITIVE AND PSYCHOSOCIAL OUTCOMES IN SCHIZOPHRENIA AND THE ROLE OF INTRINSIC MOTIVATION

Shayden Bryce*[,1], Jennie Ponsford[2], Stuart Lee[3], Eric Tan[4], Sean Carruthers[5], Richard Lawrence[1], Susan Rossell[6]
*[1]Monash University; [2]Monash-Epworth Rehabilitation Research Centre; [3]Monash Alfred Psychiatry Research Centre, The Alfred Hospital and Monash University, Central Clinical School; [4]Swinburne University, Monash Alfred Psychiatry Research Centre, The Alfred Hospital and Monash University; [5]Swinburne University and Monash Alfred Psychiatry Research Centre, The Alfred Hospital; [6]Swinburne University, Monash Alfred Psychiatry Research Centre, St. Vincents Mental Health*

**Background:** Cognitive remediation (CR) therapies are upheld as promising methods of reducing cognitive impairment in schizophrenia. However, controlled trials with blind assessors and active comparison conditions are lacking, along with evidence of generalization of CR to everyday function and self-efficacy. In addition, the role of patient-specific factors such as motivation in predicting adherence and training outcomes has not been investigated. This assessor-blinded, randomized controlled trial compared the impact of 'drill-and-strategy' CR with a computer game (CG) control delivered in a group-setting on cognitive function, independent living skills and self-efficacy, and examined the impact of intrinsic motivation on group attendance and treatment response.

**Methods:** Fifty-six people with schizophrenia or schizoaffective disorder were randomized into CR or CG, and offered 20 one-hour sessions over 10 weeks. Measures of cognition (MATRICS consensus cognitive battery), psychopathology (Positive and Negative Syndrome Scale), self-efficacy (Revised Self Efficacy Scale) and independent living skills (Independent Living Skills Survey) were administered at baseline, end-group and three-months post-group. Intrinsic motivation (Intrinsic Motivation Inventory-Schizophrenia Research) was measured in-session at baseline and end-group.

**Results:** Primary analysis was conducted for participants who completed end-therapy assessment (CR=22; Control=21). Linear mixed-effect analysis found a significant interaction effect for cognition (p=.028). Pairwise comparisons revealed that cognition was better at end-group and three-month follow-up than baseline for CR completers, with no differences between timepoints for controls. Three-quarters (77%) of CR completers showed a reliable improvement in at least one cognitive domain. A significant time effect was also evident for self-efficacy (p=.028), with the combined groups showing higher self-efficacy at end-group than baseline. No changes in independent living skills were observed. Early reports of program value predicted session attendance above baseline cognitive and clinical symptoms. Enhanced program interest and value over time increased the likelihood of reliable cognitive improvement.

**Discussion:** Drill-and-strategy CR, delivered as a stand-alone treatment in a group setting, may improve cognition in schizophrenia when compared to active controls. Enhancing motivation may increase the likelihood of achieving meaningful cognitive improvements. This type of CR, however, may not translate to independent living domains, even if enhanced cognition and confidence in completing everyday behaviors is achieved. Independent living skills may need to be targeted directly to achieve meaningful changes in this domain.

### S90. IMPLICIT PROCESSING OF BODILY EMOTIONS IN SCHIZOPHRENIA

Michal Hajdúk*[,1], Hans Klein[2], Emily Bass[2], Amy Pinkham[3]
*[1]Comenius University; [2]University of Texas at Dallas; [3]University of Texas at Dallas, University of Texas Southwestern Medical School*

**Background:** Explicit emotion recognition from faces is severely impaired in patients with schizophrenia; however, implicit processing of facial emotion appears to be intact and comparable to healthy control individuals (Shasteen et al., 2016). Social cues are not restricted to facial expressions, and body posture makes substantial contributions to nonverbal communication (de Gelder, 2006). The role of body perception in social processing among individuals with schizophrenia has not yet been studied. The aim of the present study was to evaluate whether intact implicit processing of emotion in patients with schizophrenia spectrum disorders extends to body cues devoid of facial information.

**Methods:** Fifty-three patients diagnosed with schizophrenia spectrum disorders and 34 matched healthy controls completed the Affect Misattribution Task, a paradigm in which affective responses to primes are projected onto neutral targets. Primes consisted of pictures from The Bodily Expressive Action Stimulus Test and included 24 images each of bodies expressing happy, angry, and neutral expressions. Participants were asked to indicate whether neutral targets (i.e., Chinese symbols) were more or less threatening than average symbol. Scores on the Paranoia Scale and PANSS Suspiciousness item were used for measuring levels of paranoid ideation.

**Results:** A repeated measures ANOVA with prime type as the within-subjects factor and group membership as the between-subjects factor revealed significant main effects for prime type ($F(2,170)= 16.722$, $p < .001$, $\eta 2 = .164$) and group ($F(1,85)= 5.704$, $p = .019$, $\eta 2 = .063$) such that patients identified more targets as threatening and, across both groups more targets were identified as threatening in the anger condition relative to the neutral and positive condition. The interaction was not significant ($F(2,170) =.142$, $p = .868$, $\eta 2 =.002$). In patients, the total number of threatening responses was positively correlated with self-reported paranoid ideation measured by the Paranoia Scale ($r = .391$, $p = .004$). However, the association between PANSS Suspiciousness ratings and number of threatening responses was not significant ($r = .168$, $p = .229$).

**Discussion:** In both groups, angry bodies were rated as more threatening than neutral and happy expressions, which suggest that patients have intact implicit processing of emotions from body cues. This parallels previous findings of intact implicit processing in facial emotion perception. Patients also tended to rate stimuli as more threatening on average, which may be partially explained by higher levels of paranoid ideation in this group. Results will be discussed in relationships to threat processing theories.

Research was supported by Slovak Research and Development Agency no: APVV-15–0686 and internal funding provided by The University of Texas at Dallas.

### S91. CLINICAL CHARACTERISTICS OF FORMAL THOUGHT DISORDER IN SCHIZOPHRENIA

Joonho Choi*[,1], Han-Yong Jung[2], Joo Eon Park[3], Hoseon Lee[4], Seon-Cheol Park[5]
*[1]Hanyang University Guri Hospital; [2]Soonchunhyang University Bucheon Hospital; [3]Keyo Hospital; [4]St. Andrew Hospital; [5]Inje University College of Medicine and Haeundae Paik Hospital*

**Background:** Our study aimed to present the distinctive correlates of formal thought disorder in patients with schizophrenia, using the Clinical Language Disorder Rating Scale (CLANG).

**Methods:** We compared the formal thought disorder and other clinical characteristics between schizophrenia patients with (n = 82) and without (n = 80) formal thought disorder. Psychometric scales including the CLANG, Brief Psychiatric Rating Scale (BPRS), Young Mania Rating Scale (YMRS), Calgery Depression Scale for Schizophrenia (CDSS) and Word Fluency Test (WFT) were used.

**Results:** After adjusting the effects of age, sex and total scores on the BPRS, YMRS and WFT, the subjects with disorganized speech presented significantly higher score on the poverty of contents of abnormal syntax ($F = 7.08$, $P = 0.01$), lack of semantic association ($F = 8.02$, $P =0.01$), disclosure failure ($F = 60.97$, $P < 0.001$), pragmatics disorder ($F = 11.94$, $P = 0.01$), dysarthria ($F = 13.61$, $P < 0.001$), and paraphasic error ($F = 8.25$, $P = 0.01$) items than those without formal thought disorder. With defining the mentioned item scores as covariates, binary logistic regression model predicted that disclosure failure (adjusted odds ratio [aOR] = 5.88, $P < 0.001$) and pragmatics disorder (aOR = 2.17, $P = 0.04$) were distinctive correlates of formal thought disorder in patients with schizophrenia.

**Discussion:** Disclosure failure and pragmatics disorder might be used as the distinctive indexes for formal thought disorder in patients with schizophrenia.

### S92. DISTINCT RISK FACTORS FOR OBSESSIVE AND COMPULSIVE SYMPTOMS IN SCHIZOPHRENIA

Emilio Fernandez-Egea*[,1], Yulia Worbe[2], Miguel Bernardo[3], Trevor W Robbins[1]
*[1]University of Cambridge; [2]Saint-Antoine Hospital, Paris; [3]Barcelona Clinic Schizophrenia Unit. Hospital Clinic Barcelona*

**Background:** Obsessive-compulsive disorder (OCD) is common in schizophrenia patients treated with antipsy-chotics with significant anti-serotoninergic action. Clozapine was the first drug reported and is still the medication more frequently associated with OCD, along with olanzapine. The aim of this study was to study the OCD prevalence, clinical profile and associated severity factors, using electronic records of a large cohort of clozapine-medicated schizophrenic patients. Patients were routinely screened for OCD using standardised scales as well as relevant clinical, psychometric and demographic data.
**Methods:** The electronic records of a large cohort of clozapine-medicated schizophrenia patients routinely screened for OCD using standard measures were used. The Obsessive Compulsive Inventory Revised version (OCI-R) was available from 118 cases and a 21 points cut-off threshold for OCD was defined.
**Results:** OCD prevalence was 47% and significantly higher in patients on several medications including clozapine than on clozapine monotherapy (64% vs 31%; p=.001). Two OCI-R factors had significantly higher scores in these patients and were associated with distinct risk factors: checking behaviour (mean=5.1; SD=3.6), which correlated with length on clozapine treatment (r=.21; p=.026), and obsessing factor (mean=4.8; SD=3.6), which correlated with psychosis severity (r=.59; p=.001). However, these factors along with total OCI-R, did not correlate with either clozapine dose or plasma levels, after correcting for psychosis severity.
**Discussion:** We propose an imbalance between impaired goal-directed behaviour and habit formation in favour of the latter in clozapine-OCD as a potential theoretical framework for our results. Compulsion in clozapine-medicated schizophrenia patients could be understood as a long-term by-product of the psychosis (even after remission) perpetuated by clozapine's potent antiserotoninergic properties. Screening for OCD in clozapine patients, and probably in those treated with structurally similar drugs like olanzapine, should be widely adopted by clinicians.

### S93. DIETARY PATTERNS AND PHYSICAL ACTIVITY IN PEOPLE WITH SCHIZOPHRENIA

Ane Jakobsen*,[1]
[1]*Mental Health Center Copenhagen*

**Background:** People with severe mental disorders die 10–25 years earlier than people in the Western background population, mainly due to lifestyle related diseases, with cardiovascular disease (CVD) being the most frequent cause of death. Major contributors to this excess morbidity and mortality are unhealthy lifestyle factors including tobacco smoking, unhealthy eating habits and lower levels of physical activity. The aim of this study was to investigate the dietary habits and levels of physical activity in people with schizophrenia spectrum disorders and overweight and to compare the results with the current recommendations and with results from the general Danish population.
**Methods:** We interviewed a sample of 428 people with schizophrenia spectrum disorders and increased waist circumference enrolled in the CHANGE trial using a Food Frequency Questionaire (FFQ) and a 24 hours recall interview, a Physical Activity Scale (PAS), scale for sssessment of positive and negative symptoms (SAPS and SANS, respectively), Brief Assessment of Cognition in Schizophrenia (BACS) and Global Assessment of Functioning (GAF). We compared with information on dietary intake and physical activity in the general Danish population from the Danish National Survey of Dietary Habits and Physical Activity in 2011–2013 (DANSDA).
**Results:** The CHANGE participants reported a very low energy intake and their distribution of nutrients (i.e. fat, protein and carbohydrates) harmonized with the recommendations from the Danish Health Authorities, and were similar to the latest report on the dietary habits in the Danish general population. However, the intake of saturated fat, sugar and alcohol exceed the recommended amounts and the corresponding intake in the general population. The intake of fiber, vegetables and fruit and fish were insufficient and also less than in the general population. The overall estimated quality of the dietary habits was poor, only 10.7% of the participants had healthy dietary patterns, and the quality was poorer than in the general population. Even with a very liberal definition of the term "homecooked", only 62% of the participants had taken any part in the preparation of their food. The level of physical activity was low and only one fifth of the participants complied with the recommendations of min. 30 minutes daily moderate-to-vigorous activity. Half of the CHANGE participants were smokers, compared to 17% in the general population. Negative symptoms were significantly associated with poorer dietary quality and less physical activity, whereas no such significant associations were found for cognition, positive symptoms or antipsychotic medication.
**Discussion:** Even when accounting for some error from recall - and social desirability bias, the findings point in the direction that the average energy intake in obese people with schizophrenia spectrum disorders is not exceeding that of the general population, and that overweight may to some degree be a result of physical inactivity and metabolic adverse effects of antipsychotic medication. The physical activity level is low and the rate of tobacco smoking is high, and our results suggest that negative symptoms play a significant role. Future research should focus on bringing about lifestyle changes in this fragile population in order to reduce the excess risk of CVD and mortality.

### S94. INTEGRATED DIABETES MANAGEMENT FOR INDIVIDUALS WITH SERIOUS MENTAL ILLNESS

Kristina Cieslak*,[1], Corinne Cather[1], Sarah Maclaurin[1], Deborah Wexler[1], Anne Thorndike[1], Trina Chang[1], Gladys Pachas[1], Mike Vilme[1], Oliver Freudenreich[1], Anne Eden Evins[1]
[1]*Massachusetts General Hospital*

**Background:** Premature mortality due to cardiovascular disease in those with schizophrenia is the largest lifespan disparity in the US and is growing; adults in the US with schizophrenia die on average 28 years earlier than those in the general population. An estimated one in five people with severe mental illness (SMI) has diabetes; lifetime rates of diabetes among those with SMI are two to three times higher than for those in the general population. Contributing factors to this astonishingly high rate of diabetes include effects of antipsychotic medication, unhealthy lifestyle, and likely factors related to schizophrenia itself. High rates of tobacco dependence and poor understanding of diabetes management combine to cause to the extraordinarily high morbidity and mortality associated with diabetes in those with SMI. There exists a significant gap in the literature for theory and evidence-based interventions to improve the ability of those with SMI to manage their diabetes.
**Methods:** We have developed a 16-week tailored behavioral and educational group intervention for individuals with schizophrenia and diabetes, utilizing the concept of 'reverse integrated care,' bringing medical intervention into the community mental health setting. Core features of this intervention include motivational interviewing, basic education, and problem-solving. The primary outcome of this study is glycemic control, as measured by hemoglobin A1C (HbA1C). Secondary outcomes include lipid panel, measures of diabetes knowledge and self-management, blood pressure, weight, BMI, and step count.
**Results:** Thirty individuals were consented and randomized to a two-period crossover design consisting of a 16-week group intervention and a 16-week observation period. Average HbA1c at baseline=7.5, range=5.9–13.4. Seventeen individuals successfully completed the intervention. An average 0.59-point reduction in HbA1c was observed from baseline to the end of the 16-week active intervention (t=1.99, DF=17, p=0.063). A marginally significant weight reduction was observed from baseline to week 16 in the active condition of 5.3 pounds (t=2.07, DF=17, p=0.054). Ten participants lost greater than five pounds. Significant changes were observed in increased average step count of 3189 steps/day (t=2.25, DF=17, p=0.038), and improved scores on diet (t=2.84, DF=17, p=0.01), exercise (t=2.24, DF=17, p=0.039), and foot care (t=2.99, DF=17, p=0.01) diabetes self-care measures. Promising decreases were seen in systolic blood pressure – those with baseline >130 systolic blood pressure reducing from an average of 138 to 125; diastolic blood pressure – those with baseline >90 reduced from an average of 93 to 80; a 10-point average reduction in total cholesterol (t=-1.13, DF=17, p=0.27), and 50-point average reduction in triglycerides (t=-1.29, DF=17, p=0.21). A continued decrease was observed for A1C, weight, and triglycerides in the first active intervention group 16-weeks post-completion, suggesting sustainability of gains made during the intervention.

**Discussion:** There is a pressing need to address the morbidity and premature mortality related to modifiable health behaviors in this underserved population, yet individuals with SMI and diabetes are much less likely to be identified or to receive recommended diabetes care and monitoring. We hope to further establish and refine a standard of care diabetes education curriculum, tailored for individuals with SMI, a population with high prevalence of diabetes but low rates of diabetes diagnosis, education, and treatment. Results from year one demonstrate this program to be easily implementable, well-accepted, socially relevant and effective.

### S95. PREVALENCE AND CLINICAL CORRELATES OF COMORBID OBSESSIVE-COMPULSIVE DISORDER IN PATIENTS WITH SCHIZOPHRENIA

Lee Bora*[,1], Kyung Sue Hong[2], Ji Hyun Baek[2], So Yung Yang[2]
*[1]Samsung Medical Center; [2]Sungkyunkwan University School of Medicine, Samsung Medical Center*

**Background:** Obsessive compulsive symptoms (OCS) commonly occur in the course of schizophrenia. However, reported rates of comorbid obsessive compulsive disorder (OCD) in schizophrenia were highly variable among studies. In addition, influences of OCS on the symptomatology and functioning of schizophrenia have not been fully explored. The aim of this study was to investigate the clinic-based prevalence rate of OCD in schizophrenia patients, and to evaluated clinical correlates of the comorbidity.

**Methods:** Patients with schizophrenia (n=320) were recruited and lifetime clinical characteristics were evaluated comprehensively. Patients having comorbid OCD (OCD group, n=66) and those without OCD (the non-OCD group, n=254) were compared in terms of clinical characteristics and cognitive functioning.

**Results:** OCD was found in 20.6% of the subjects. Earlier age at onset, male gender, and higher level of education were associated with comorbid OCD. In terms of individual symptoms and symptom dimensions, 'anxiety (p=0.009) and 'depression (p=0.001)' were more frequently observed in the OCD group than in the non-OCD group. The prodromal impairment was higher in the non-OCD group (p=0.016). The OCD group showed better performance in working memory domain (p= 0.003), and other cognitive domains did not show any significant group difference.

**Discussion:** The prevalence rate of OCD in the current subjects was within the range of previously reported comorbidity rates in schizophrenia patients from other populations. Association of OCS with anxiety and depressive symptoms seems to be a common finding which was also reported in previous studies of schizophrenia and bipolar disorders. Regarding cognitive functions, inconsistent results including the current report have been generated suggesting heterogeneous developmental mechanisms of OCS in schizophrenia.

### S96. CARDIOVASCULAR DISEASE RISK IN PATIENTS WITH SCHIZOPHRENIA AND BIPOLAR DISORDER

Linn Rødevand*[,1], Nils Eiel Steen[1], Daniel Quintana[1], Elina Johanna Reponen[1], Ragni Helene Mørch[1], Synve Hoffart Lunding[1], Trude Iversen[1], Trine V. Lagerberg[1], Ingrid Melle[1], Ole A. Andreassen[1]
*[1]NORMENT & K.G. Jebsen Centre for Psychosis Research, Oslo University Hospital, University of Oslo*

**Background:** Patients with schizophrenia and bipolar disorder have markedly reduced life expectancy compared to the general population (15–20 years). A major contributor of excess death is cardiovascular disease (CVD) [1]. During the last decade, there have been several public health campaigns for health promotion and disease prevention, and tobacco legislation has become stricter. These strategies appear to have been effective in improving the health of the general Norwegian population [2]. It is unknown whether the elevated CVD risk in patients with schizophrenia and bipolar disorder has sustained in spite of these health promotion approaches. Here we investigate the development of CVD risk factors in a large representative sample of Norwegian patients with schizophrenia and bipolar disorder between 2002 and 2017. More specifically, we explored whether the CVD risk level was similar in a cohort from 2006 and a second cohort from 2017.

**Methods:** Cross sectional analysis was performed among DSM-IV diagnosed patients included from 2002–2005 (cohort 1) and from 2006–2017 (cohort 2), respectively. Cohort 1 consisted of 161 patients with schizophrenia and 109 patients with bipolar disorder, and cohort 2 consisted of 623 patients with schizophrenia and 387 patients with bipolar disorder. Comparisons were made between cohorts regarding demographic variables, psychiatric symptoms, tobacco use, body mass index, waist circumference, total cholesterol, high-density lipoprotein (HDL) cholesterol, low-density lipoprotein (LDL) cholesterol, triglycerides, fasting glucose, systolic blood pressure, and diastolic blood pressure. ANCOVA was used for these analyses, with adjustments for age, duration of the disorder, and duration of psychopharmacological treatment.

**Results:** Mean age was significantly higher for cohort 1 (35.1 years vs. 31.2 years, $p < .001$). There was no statistically significant difference in any of the other demographic variables or symptoms. Among patients with schizophrenia, there was no significant difference in the prevalence of CVD risk factors except from glucose being slightly increased in patients included in cohort 2 ($p = .047$). Among patients with bipolar disorder, there was a significant reduction in the level of total cholesterol, LDL, systolic, and diastolic blood pressure in cohort 2 (all p values < .01). These differences remained statistically significant after adjusting for age, duration of the disorder, and duration of psychopharmacological treatment.

**Discussion:** Despite major advances in health promotion and disease prevention during the past decade, the level of CVD risk factors has remained high in patients with schizophrenia. While the level of some CVD risk factors improved in patients with bipolar disorder, they are still at increased risk of CVD. Thus, patients with severe mental disorders, especially schizophrenia, do not appear to have benefited from recent health promotion measures. Our findings also highlight the need for more effective interventions to reduce the risk of CVD in individuals with severe mental disorders, which may reduce the gap in life expectancy compared to the general population.

**References:**

1. Nordentoft M, Wahlbeck K, Hallgren J, et al. Excess mortality, causes of death and life expectancy in 270,770 patients with recent onset of mental disorders in Denmark, Finland and Sweden. PLoS One 2013;8(1):e55176. doi: 10.1371/journal.pone.0055176
2. Mannsverk J, Wilsgaard T, Mathiesen EB, et al. Trends in Modifiable Risk Factors Are Associated With Declining Incidence of Hospitalized and Nonhospitalized Acute Coronary Heart Disease in a Population. Circulation 2016;133(1):74–81. doi: 10.1161/circulationaha.115.016960

### S97. SOCIAL ANXIETY IN SCHIZOPHRENIA: THE IMPACT OF HALLUCINATIONS AND SELF-ESTEEM SUPPORT

Laura Faith*[,1], Elise Racette[1], Amber Grove[1], Melisa Rempfer[1]
*[1] University of Missouri–Kansas City*

**Background:** Social anxiety is an underreported concern in schizophrenia (SCZ). Prevalence rates in the general population range from 0.5–7% (APA, 2013), but are higher in SCZ, and estimated to be 11–36% (Mazeh et al., 2009; Pallanti et al., 2004). Yet, research is limited with no established social anxiety treatments. Social anxiety is associated with decreased quality of life (Hansson, 2006), low self-esteem (Gumley et al., 2005), and increased psychopathology (Vrbova et al., 2017). Lysaker and Hammersley (2006) found that people with delusions and impairment in flexibility had the highest levels of social anxiety compared to those with fewer symptoms. Additionally, Lysaker et al. (2010) found that people with both high paranoia and theory of mind had higher social anxiety compared to those with lower levels of either paranoia or theory of mind. Taken together, this research suggests that symptoms may increase social anxiety, but other factors may inhibit their impact. The current study aims to add to this literature by exploring how different levels of hallucinations and self-esteem support affect social anxiety in SCZ.

**Methods:** Outpatients with SCZ (N=50) participated in the current study. Participants were 76% male with a mean age of 42.50. Participants were African-American (n=27; 54%), Caucasian (n=11; 22%), multi-racial (n=5; 10%), Asian (n=4, 8%), or Hispanic (n=3; 6%). Social fear, social avoidance, and overall social anxiety was measured with the Liebowitz Social Anxiety Scale (LSAS; Liebowitz, 1987). Self-esteem support (SeS) was measured with a subscale taken from the Interpersonal Support Evaluation List (ISEL; Cohen & Hoberman, 1983). SeS is the appraisal of the self compared with others and other's opinions of the self. Hallucinations (HA) were scored with the observer-rated Scale for Assessment of Positive Symptoms (SAPS; Andreasen, 1983). Participants were classified as having hallucinations if their SAPS global hallucinations were rated moderate to severe. This was chosen a priori as it reflects a level of clear hallucinations that may bother the person to some extent, as defined within the SAPS. Participants were classified as having either high or low SeS based on a mean split of the distribution of scores. Once participants were classified, we planned to compare groups on levels of social anxiety. This method was modified from previous research reporting similar groupings of symptoms and their relationship to social anxiety (Lysaker & Hammersley, 2006).

**Results:** Four groups resulted after including the dichotomized variables with the following proportions: low SeS/no HA (n=6; 12.5%), low SeS/HA (n=11, 22.9%), high SeS/no HA (n=13; 27.1%), and high SeS/HA (n=18, 37.5%). A one-way ANOVA was conducted to analyze the differences between groups. Post-Hoc analyses revealed the following differences. The HA/low SeS group had higher social anxiety than in the no HA/high SeS group (p=.030) and no HA/low SeS group (p=.039). The HA/low SeS group had higher social fear (p=.017) and social avoidance (p=.013) than in the no HA/high SeS group. There was a trending difference revealing that participants in the HA/low SeS group had higher social avoidance than in the HA/high SeS group (p=.056). There was a trending difference revealing that the HA/low SeS group had greater overall social anxiety than those in the HA/high SeS group (p=.064).

**Discussion:** These results present preliminary findings on social anxiety in people with different levels of HA and SeS. We found that people with low SeS and HA had significantly higher levels of social anxiety, social fear, and social avoidance than participants with only one of neither of these symptoms. These results will be discussed further to highlight implications to treatment and comorbidities in SCZ.

### S98. THE RELATIONSHIP BETWEEN CARDIOVASCULAR RISK FACTORS AND COGNITIVE IMPAIRMENT IN PEOPLE WITH SCHIZOPHRENIA: A SYSTEMATIC REVIEW AND META-ANALYSIS

Katsuhiko Hagi*[,1], Tadashi Nosaka[1], Christoph Correll[2]

[1]*Dainippon Sumitomo Pharma;* [2]*Hofstra Northwell School of Medicine*

**Background:** Schizophrenia is associated with cardiovascular abnormalities, including diabetes mellitus (DM), metabolic syndrome (MetS), obesity, hypertension and dyslipidemia. Since cardiovascular risk factors can worsen cognition in the general population, they may also contribute to cognitive impairment in schizophrenia.

**Methods:** Performing an electronic search in Embase/Scopus/MEDLINE/PubMed/Cochrane library/www.clinicaltrials.gov), we meta-analyzed cross-sectional studies comparing neurocognitive functioning in schizophrenia patients with versus without cardiovascular risk factors. Global cognition and Attention/Vigilance, Reasoning/Problem Solving Speed of Processing Verbal Learning, Visual Learning and Working Memory were analyzed.

**Results:** Data from 22 trials (n=9,579, DM=8 studies, MetS=9 studies, obesity=8 studies, overweight=8 studies, arterial hypertension=5 studies, dyslipidemia=4 studies) were meta-analyzed.

Significantly greater global cognitive deficits in schizophrenia were associated with presence of DM (n=2,976, Hedges' g=0.322; 95%CI=0.227–0.417, p<0.001), MetS (n=2,269, Hedges' g=0.409; 95%CI=0.166–0.652, p=0.001) and hypertension (n=1,899, Hedges' g=0.210; 95%CI=0.110–0.311, p<0.001), but not with obesity (n=2,779, Hedges' g=0.695, 95%CI=-0.320, 1.709, p=0.180), overweight (n=2,825, Hedges' g=0.406; 95%CI=-0.445, 1.257, p=0.350), or dyslipidemia (n=1,761, Hedges' g=-0.055; 95%CI=-0.162, 0.051, p=309). Among 6 analyzed cognitive domains, DM (Hedges' g=0.23–0.40) and hypertension (Hedges' g=0.15–0.27) were each associated with significantly greater cognitive dysfunction in 4 domains, MetS with 3 (Hedges' g=0.16–0.42), obesity (Hedges' g=0.35) and overweight (Hedges' g=0.24) with one, and dyslipidemia with none.

**Discussion:** DM, MetS and hypertension are associated with significant global cognitive impairment in schizophrenia. The same cardiovascular risk factors and, less so, obesity and overweight, are associated with worse performance in specific cognitive domains. Research is needed to determine to what degree improving cardiovascular risk factors also improves cognitive impairment in schizophrenia.

### S99. CANNABIS USE, PSYCHOTIC-LIKE EXPERIENCES AND ABERRANT SALIENCE IN A SAMPLE OF BELGIAN STUDENTS

Francesco Bernardini*[,1], Chiara Gobbicchi[2], Luigi Attademo[3], Severin Puchalski[1], Patrizia Moretti[2], Alfonso Tortorella[2], Gwenole Loas[4]

[1]*Free University of Brussels;* [2]*Università degli Studi di Perugia;* [3]*ASP Basilicata;* [4]*Erasme Hospital*

**Background:** Cannabis is the most popular illicit drug in the western world and its use seems to be strongly associated with an increased risk of developing schizophrenia and other psychotic disorders. Its use can induce transient psychotic symptoms in healthy individuals and increase rate of subclinical psychotic symptoms in the general population. Subclinical psychotic experiences (also called Psychotic Like Experiences: PLEs), such as magical thinking, paranoid ideation or hallucinations, could be considered as a phenotype qualitatively similar to the symptomatology of psychotic disorders but quantitatively less severe in terms of intensity, frequency and impairment. They are fairly common in the general population and usually transitory and self-limiting but they could become abnormally persistent and evolve to a full-blown psychotic disorder, especially if combined with certain environmental risk factors, such as trauma, urbanicity, cannabis use. PLEs may be considered as an early marker of a latent psychosis vulnerability and the frequently good outcome of subclinical psychosis can be turned in negative outcomes by the association with environmental risk factors, such as cannabis use. We focus our attention on aberrant salience, a peculiar psychotic experience, frequently reported during the prodromal phase that precede the onset of full-blown psychotic illness. Aberrant salience is the unusual or incorrect assignment of salience or significance to innocuous stimuli; it has been hypothesized to be an important mechanism in the development of psychosis.
**Methods:** Undergraduate students of ULB (Universitè Libre de Bruxelles) and INSAS (Institut national supérieur des arts du spectacle) of Brussels (Belgium) were invited to participate to the study.
A self-report questionnaire, investigating socio-demographic characteristics, and cannabis use was administered, evaluating lifetime and current cannabis use.
Aberrant Salience Inventory (ASI) is a 29 item Yes–No questionnaire developed to evaluate aberrant salience. French version of Community Assessment of Psychic Experiences (CAPE), was used to evaluate dimensions of psychosis and PLEs. CAPE is a 42-item, self-report questionnaire, developed to measure the lifetime prevalence of PLEs in the general population. The questionnaire assesses three symptom dimensions (positive, depressive and negative symptoms). All statistical analysis was carried out with Statistical Package for Social Sciences, Version 20.0. We evaluated individual correlations between years of cannabis use and days of cannabis use in the last month with the tools scores. We also explored correlation of ASI score with different CAPE scores and different types of PLEs. Correlations were carried out by using the nonparametric Spearman correlation test.
**Results:** The final sample was of 257 participants. 46,3% of subjects reported a lifetime cannabis use and 35.0% reported a current cannabis use (last 30 days). Compared with non-users, cannabis users showed significant higher ASI scores and also higher positive and negative dimensions CAPE scores. No significant association was found between cannabis consumption and the depressive dimension of CAPE. Years of cannabis use and frequency of use in the last 30 days showed a small positive correlation with ASI score; also, weaker positive correlations with CAPE positive and negative dimensions scores were observed.
**Discussion:** To some extent, our results support the evidences that cannabis use is associated with an increased rate of psychotic experiences in individuals without clinical form of psychosis. Future prospective longitudinal studies are required to better investigate the meaning of the association between cannabis use and PLEs.

### S100. EFFECTS OF CANNABIS USE ON BODY MASS, FASTING GLUCOSE AND LIPIDS DURING THE FIRST 12 MONTHS OF TREATMENT IN SCHIZOPHRENIA SPECTRUM DISORDERS

Frederika Scheffler*[,1], Sanja Kilian[1], Bonga Chiliza[1], Laila Asmal[1], Lebogang Phahladira[1], Stefan du Plessis[1], Martin Kidd[1], Robin Murray[2], Marta Di Forti[2], Soraya Seedat[1], Robin Emsley[1]
*[1]Stellenbosch University; [4]Institute of Psychiatry, King's College, London*

**Background:** Acute cannabis use stimulates appetite, while general population studies suggest that chronic use is associated with reduced risk of obesity and other cardiometabolic risk factors.
**Methods:** In this study, we investigated changes in body mass index (BMI), fasting blood glucose and lipids, and rates of metabolic syndrome risk factors in cannabis users vs. non-users in 109 minimally treated patients with first-episode schizophrenia, schizophreniform or schizo-affective disorder who were treated according to a standardized treatment regime with depot antipsychotic medication over 12 months. Participants underwent repeated urine toxicology tests for cannabis and those testing positive at any time during the study (n=40), were compared with those who tested negative at all time points (n=69).
**Results:** There was a significant group*time interaction effect (p=0.002) with the cannabis negative group showing a greater increase in BMI than the cannabis positive group, after adjusting for age, sex, methamphetamine use and modal dose of antipsychotic. There were no group*time interaction effects for fasting blood glucose or lipids. Post hoc tests indicated significant increases in fasting blood glucose and triglycerides and a decrease in high-density lipoprotein cholesterol for the cannabis negative group, with no significant changes in the cannabis positive group. Rates of metabolic syndrome did not differ significantly between groups. However, more cannabis negative patients had elevated waist-circumference at endpoint (p=0.003).
**Discussion:** Although other indirect effects such as dietary neglect and smoking may be contributory and could explain our findings, it may be that chronic cannabis use directly suppresses appetite, thereby preventing weight gain in users.

### S101. CLINICAL FACTORS ASSOCIATED WITH CANNABIS USE IN A CHILEAN SAMPLE OF FIRST EPISODE PSYCHOSIS PATIENTS

Barbara Iruretagoyena*[,1], Nicolas Crossley[1], Alfonso Gonzalez-Valderrama[2], Cristian Mena[2], Carmen Castañeda[2], Camila Diaz[2], Juan Undurraga[2]
*[1]Universidad Católica de Chile; [2]J. Horwitz Psychiatric Institute*

**Background:** Cannabis has been associated with higher risk to develop psychosis and worse long-term outcomes. Cannabis use in 2016 is estimated to be 11.3% in Chile in 2016 (SENDA 2016). As in many other countries, cannabis use has steadily increased during last 20 years, across all socioeconomic groups, but especially in men and in the group aged 12 to 25 years old. At the same time, 5.7% subjects declared using high potency cannabis. We here examined the frequency of cannabis use in patients with a first episode psychosis in Chile. We also sought to identify etiologic factors associated with cannabis use, as well as its impact on clinical and functional status.
**Methods:** We performed a cross-sectional study on patients from an outpatient Early Intervention in Psychosis unit in Chile. Data included sociodemographic characteristics, cannabis and other substance use, and standardized clinical and functional status. FAST (Functional Assessment Short Test), SS-DSM5 (Symptom Severity Scale of the DSM5 for Schizophrenia), CUPIT (Cannabis use Problem Identification Scale) and MG (Morinsky Green Adherence Questionnaire) were applied to the participants.
**Results:** We included 80 patients, of which 23.8% used cannabis during the previous year. 47.3% of cannabis users had used cannabis with high THC concentration. 63.2% of consumers had a moderate-high score in the CUPIT scale, indicating a high prevalence of risk consumption and use disorder. Regarding variables related with cannabis use, correlation analysis showed a significant relationship with alcohol use ($p<0.001$), drug use ($p<0.001$) and duration of untreated psychosis (p=0.039, all corrected for multiple analysis.). Multivariate regression analyses including these variables along with gender and age, only showed relation with drug use (p=0.031, OR 7.94 (1.21–51.91).
Regarding cannabis use and clinical and functional outcomes, correlation analysis showed association with adherence problems as reported by physician (p= 0.026) and the Morinsky Green Adherence Questionnaire (p= 0.031). Results showed an OR of 5.23 (1.38–19.76) for adherence problems and OR 0.2 (0.05–0.75) for Morinsky Green Adherence Questionnaire reporting good adherence. There was no effect in treatment resistance, FAST score, SS-DSM5 global, cognitive or negative score.
**Discussion:** The percentage of cannabis use in this first episode psychosis sample is high, with a large subgroup using high potency THC cannabis. Cannabis use was associated to other drug use and to treatment adherence problems reported by physician and patient. This shows the importance of substance use treatment in first episode programs.

### S102. CANNABIS MAY PROTECT AGAINST CERTAIN DISORDERS OF THE DIGESTIVE ORGANS IN PATIENTS WITH SCHIZOPHRENIA BUT NOT IN HEALTHY CONTROLS

Julie Aamand Olesen[1], Christine Merrild Posselt[2], Merete Nordentoft[3], Carsten Hjorthoej*[,1]
[1]*Copenhagen University Hospital, Mental Health Center Copenhagen;* [2]*The Parker Institute;* [3]*Mental Health Centre Copenhagen, The Lundbeck Foundation Initiative for Integrative Psychiatric Research (iPSYCH)*

**Background:** Cannabis use disorder increases both overall mortality and several cause-specific mortalities. However, an unexpected finding has been that, in patients with schizophrenia, cannabis use disorders were associated with a decreased risk of death from causes related to the digestive organs. Further indications of potentially beneficial effects of cannabis were supported by a 2016 systematic review showing protective effects of cannabis in patients with established gastrointestinal disorders. Further, schizophrenia, either in itself or through the use of antipsychotics, may interact with cannabis in various organs, potentially leading to different associations. For these reasons, we aimed to investigate the associations between cannabis and later development of disorders of the digestive organs, both in patients with schizophrenia and in healthy controls.
**Methods:** We used the nationwide Danish registers in a prospective cohort study. All individuals born since 1955 in Denmark and diagnosed with schizophrenia were included, and matched to approximately ten controls on age and sex to healthy controls. Cannabis use disorders and alcohol and other substance use disorders (SUD) were identified through combinations of several health registers, as were the outcomes. We investigated the following outcomes: Any disorder of the digestive organs, cancers of the digestive organs, non-cancer disorders of the digestive organs, inflammatory bowel disease, disorders of the gut-brain axis, non-cancer disorders of the pancreas, non-cancer disorders of the liver, and a category of disorders of the digestive organs considered to be severe. For each analysis, people with a corresponding digestive-organ diagnosis before either the date of schizophrenia or the controls' corresponding match-date were excluded.
**Results:** The number of patients with schizophrenia varied between 17,718 and 22,636 in different analyses. After adjusting for other SUDs, cannabis use disorders showed protective effects in patients with schizophrenia against disorders of the gut-brain axis (HR=0.78, 95% CI 0.68–0.91, p=0.001), and serious disorders of the digestive organs (HR=0.81, 95% CI 0.69–0.96, p=0.02). Non-significant protective effects were also apparent for non-cancer disorders of the digestive organs (HR=0.93, 95% CI 0.85–1.02, p=0.11), inflammatory bowel disease (HR=0.67, 95% CI 0.42–1.08, p=0.10), and non-cancer disorders of the liver (HR=0.80, 95% CI 0.61–1.05, p=0.12). These protective effects were never shown in healthy controls, where cannabis use disorders generally were not associated in any direction with digestive-organ disorders in the fully adjusted analyses.
**Discussion:** Cannabis use disorders may show protective effects against certain disorders of the digestive organs in patients with schizophrenia. The explanations may be causal, which would however require further explanations as to why the same effects are not seen in healthy controls. This may be due to use of antipsychotic medication interacting with cannabis, or due to higher levels of cannabis consumption in cases with schizophrenia than in controls. The effect may also be in part explained by comorbid use of tobacco, as nicotine is also known to interact with the gastrointestinal system.

### S103. IS CANNABIS A RISK FACTOR FOR SUICIDE ATTEMPTS IN MEN AND WOMEN WITH PSYCHOTIC ILLNESS?

Anna Waterreus*[,1], Patricia Di Prinzio[1], Johanna Badcock[1], Mat Martin-Iverson[1], Assen Jablensky[1], Vera Morgan[1]
[1]*University of Western Australia*

**Background:** A growing body of evidence supports the association between cannabis use and an increased risk of suicidal behaviour in the general population. However, studies that have examined the relationship between cannabis use and suicide in people with a psychotic disorder report divergent findings. Further research is needed to help clarify the relationship between cannabis use and suicidal behaviour in men and women with psychotic illness.
**Aim:** To examine whether past-year cannabis use by men and women with psychotic disorders was associated with an increased risk of suicide attempt, and what others factors were associated with suicide attempt, stratified by sex.
**Methods:** Data from 1065 men and 725 women interviewed in the second Australian national survey of psychosis (SHIP) were analysed using multiple logistic regression to model separately, for each sex, the impact of daily, casual or no past-year cannabis use and other risk factors, on a past 12-month suicide attempt.
**Results:** In the 12 months prior to interview, 168 (9.4%) participants attempted suicide. Almost one quarter (23.1%) of women using cannabis on a daily basis attempted suicide compared to 15.2% of casual users and 10.2% of non-users. In contrast, the proportion of men attempting suicide across daily, casual and cannabis non-users was 10.8%, 9.1%, and 6.1% respectively. Unadjusted analyses showed daily cannabis users of both sexes had significantly increased odds of attempting suicide compared to non-users (men OR: 1.85, 95% CI: 1.02–3.35, women OR: 2.64, 95% CI: 1.39–5.00). This relationship remained, but was no longer significant, after adjusting for other covariates. Other factors associated with a significantly increased odds of a suicide attempt were: feeling isolated and lonely for men, and homelessness and hallucinations for women. Depression had the strongest association with attempting suicide for both sexes. Analysis examining whether the influence of cannabis use on suicide attempt differed according to age group (18–34 years or 35–64 years) indicated daily cannabis was associated with higher odds of attempting suicide in older men compared to non-users (OR: 2.80 95% CI: 1.10–7.13); this was not found in younger men or women.
**Discussion:** This study highlights the high rates of suicide attempt in people with psychotic illness (9.4% in contrast to 2.4% for the Australian general population), the increased risk of a suicide attempt associated with cannabis use, particularly for older men, and how risk factors differ between men and women. However, it also raises a number of questions regarding what are the possible mechanisms underpinning a relationship between cannabis use and suicidal behaviour, in particular, whether cannabis use has an influence on specific biological pathways, which may also explain the observed differences between men and women. With a number of countries considering legalising cannabis use, it is important for researchers to continue to clarify what impact cannabis use has on people with psychotic illness.

### S104. THE RELIABILITY AND VALIDITY OF THE CORE SCHIZOPHRENIA SYMPTOMS SCALE OF THE STANDARD FOR CLINICIANS' INTERVIEW IN PSYCHIATRY

Ahmed Aboraya*[,1]
[1]*William R. Sharpe Jr. Hospital*

**Background:** Core schizophrenia symptoms (CSS) include delusions, hallucinations and disorganization and have been included in the diagnostic criteria of schizophrenia since Kraeplin (Kendler 2016). More than 40 subtypes of delusions, hallucinations and disorganization have been described as symptoms and signs of schizophrenia. There is a need to derive a short list of the core symptoms and signs of schizophrenia that are reliable, valid and useful for clinicians in clinical settings and clinical research.
**Methods:** The Standard for Clinicians' Interview in Psychiatry (SCIP) is a new diagnostic interview designed to be used by clinicians (psychiatrists and experienced mental health professionals) in clinical settings and clinical research. The SCIP is a valid and reliable tool and was tested in an international multisite study in three countries (USA, Canada and Egypt) between 2000 and 2012 (Aboraya, El-Missiry et al. 2014, Aboraya 2015, Aboraya 2016, Aboraya, Nasrallah et al. 2016). A total of 700 patients were interviewed at William R. Sharpe Jr. Hospital in Weston, West Virginia (670 patients) and Chestnut Ridge Center in Morgantown, West Virginia (30 patients). Mean patient age was 34, 59% male, 95% White and 34% had less than 12 years of education. The SCIP includes 38 items covering subtypes of delusions, hallucinations and disorganization. The 38 items were shortened by removing items with low prevalence, low sensitivity or low item-rest correlation (< 0.4). The reliability and validity of the remaining items was recalculated with repetitive iterations. The final model was developed with input from experts. The result is the Core Schizophrenia Symptoms (CSS) Scale which has 18 items: 6 items measuring hallucinations, 8 items measuring delusions and 4 items measuring disorganization. The items were scored with binary and Likert-type scales ranging from 0 to 3. The reliability of the CSS scale was measured using the kappa coefficient for inter-rater reliability of the CSS individual items and Cronbach's alpha for internal consistency of the CSS dimension. The validity of the CSS scale was assessed using Receiver Operating Characteristic (ROC) curves to determine the best clinical cut-off point for the CSS scale that maximizes sensitivity and specificity of the scale against the SCIP diagnosis of schizophrenia (the reference standard).
**Results:** Table (1) shows stable kappa values and standard error of 15 CSS items. Nine items have good reliability (kappa > 0.7), three items have fair reliability (kappa values range from 0.5 to 0.7) and three items have poor reliability (kappa < 0.5). Table (2) shows the internal consistency of the CSS dimension using Cronbach's alpha and one-sided 95% confidence interval. The Cronbach's alpha is 0.8317, indicating excellent internal consistency. Table (3) shows the sensitivity and specificity of the Core Schizophrenia Symptoms (CSS) scale. At a cut-off of one or more positive items, sensitivity is 95.06% and specificity is 88.94%; at a cut-off of two or more positive items, sensitivity is 90.12% and specificity is 89.39%.
**Discussion:** The Core Schizophrenia Symptoms (CSS) Scale is reliable at the level of individual items and at the dimensional level. In addition, the CSS scale is a valid scale that differentiates between schizophrenia and non-schizophrenia cases in a clinical population.

### S105. VALIDATING THE PREDICTIVE ACCURACY OF THE NAPLS-2 PSYCHOSIS RISK CALCULATOR IN A CLINICAL HIGH-RISK SAMPLE FROM THE SHARP (SHANGHAI AT RISK FOR PSYCHOSIS) PROGRAM

TianHong Zhang*,[1], HuiJun Li[2], LiHua Xu[1], YingYing Tang[1], HuiRu Cui[1], Junjie Wang[1], Chunbo Li[1], Kristen Woodberry[3], Daniel I. Shapiro[3], Margaret Niznikiewicz[3], Martha E. Shenton[4], Matcheri S. Keshavan[5], William S. Stone[3], JiJun Wang[1], Robert W. McCarley[6], Larry J. Seidman[7]
*[1]Shanghai Mental Health Center, Shanghai Jiao Tong University School of Medicine; [2]Florida A & M University; [3]Beth Israel Deaconess Medical Center, Harvard Medical Center; [4]Brigham and Women's Hospital, Harvard Medical School, Veterans Affairs Boston Healthcare System; [5]Massachusetts Mental Health Center, Beth Israel Deaconess Medical Center, Harvard Medical School; [6]Harvard Medical School, Veterans Affairs Boston Healthcare System; [7]Harvard Medical School*

**Background:** The present study aims to validate the predictive accuracy of the NAPLS-2 psychosis risk calculator in a clinical high-risk (CHR) sample from the SHARP (ShangHai At Risk for Psychosis) program in Shanghai, China using comparable inclusion/exclusion criteria and assessments.
**Methods:** Three hundred CHR individuals were identified by the Chinese version of the Structured Interview for Prodromal Symptoms. Of these, 228 (76.0%) completed neuro-cognitive assessments at baseline and 199 (66.3%) had at least a one-year follow-up assessment. The latter group was used in risk calculation. Six key predictors (baseline age, unusual thoughts and suspiciousness, symbol coding and verbal learning test performance, functional decline and family history of psychosis) were entered into the NAPLS-2 model to generate a psychosis risk estimate for each case. The area under the receiver operating characteristic curve (AUC) was used to test the effectiveness of this discrimination.
**Results:** The NAPLS risk calculator showed moderate discrimination of subsequent transition to psychosis in the SHARP sample with an AUC of 0.631 ($p = 0.007$). Whether discriminating either transition or poor treatment/clinical outcomes, the AUC of the model increased to 0.754 ($p < 0.001$). A risk estimate of 30% or higher had moderate sensitivity (53%) and excellent specificity (86%) for prediction of poor treatment/clinical outcome.
**Discussion:** The NAPLS-2 risk calculator largely generalizes to a Shanghai CHR sample but is meaningfully improved when predicting an individual's poor clinical outcome as well as conversion. Our findings provide a critical step in the implementation of CHR risk calculation in China.

### S106. SUBMISSION WITHDRAWN

### S107. HEALTHCARE UTILIZATION AND COST IN SCHIZOPHRENIA AND BIPOLAR DISORDER: REAL-WORLD EVIDENCE FROM US CLAIMS DATABASES

Mallik Greene[1], Tingjian Yan[2], Eunice Chang[2], Ann Hartry*,[3], Jennifer Munday[4], Michael S. Broder[4]
*[1]Otsuka Pharmaceutical Development & Commercialization, Inc.; [2]Partnership for Health Analytic Research, LLC; [3]Lundbeck; [4]Phar, LLC*

**Background:** Schizophrenia (SCZ) and bipolar disorder (BD) are distinct psychiatric disorders, but patients may be diagnosed with both. The objective of this study was to explore healthcare resource utilization (HCRU) and cost in patients with claims-based diagnoses of SCZ, type 1 BD (BD-I), and both in a real-world setting.
**Methods:** This retrospective study used (1/1/12–6/30/16) Truven MarketScan® Commercial, Medicaid, and Medicare Supplemental databases. SCZ was defined as 1 inpatient or 2 outpatient claims for SCZ; BD-I was defined analogously. Three mutually exclusive groups were included: 1) SCZ alone: new episode with SCZ (e.g., met the claims-based diagnostic criteria for SCZ, but not for BD-I), 2) BD-I alone: new episode with BD-I (e.g., met the claims-based diagnostic criteria for BD-I, but not for SCZ), and 3) a diagnosis of both SCZ and BD-I: new episodes with both SCZ and BD-I (e.g., met the claims-based diagnostic criteria for both SCZ and BD-I). Descriptive statistics were reported; costs were adjusted to 2016 US$.
**Results:** Of the 63,725 patients in the final sample, 11.5% had SCZ alone, 80.8% had BD-I alone, and 7.7% had a diagnosis of both SCZ and BD. In the year following diagnosis, the group having a diagnosis of both SCZ and BD-I had the highest all-cause hospitalization rates (67.4% versus 39.5% in SCZ alone and 33.7% in BD-I alone) and the highest mean (SD) number of emergency room visits [3.44 (7.1] versus 1.39 (3.5) in SCZ alone and 1.29 (3.2) in BD-I alone]. All-cause total healthcare costs were highest in the group having a diagnosis of both SCZ and BD-I [mean (SD): $51,085 (62,759)], followed by the SCZ alone group [$34,204 (52,995)], and the BD-I alone group [$26,393 (48,294)].

**Discussion:** Patients with a diagnosis of both SCZ and BD-I had higher HCRU and cost than patients with either diagnosis alone. Physicians who recognize these diagnostically challenging patients may be able to effect improved treatment early in the disease process.

### S108. ASSOCIATIONS BETWEEN GLOBAL BRAIN MEASURES AND STATE- AND TRAIT- RELATED SYMPTOM EXPRESSION OF SCHIZOPHRENIA

Laila Asmal[1], Stéfan Du Plessis*[,1], Bonginkosi Chiliza[1], Sanja Kilian[1], Riaan Olivier[1], Freda Scheffler[1], Lebogang Phahladira[1], Paola Dazzan[2], Robin Emsley[1]
*[1]Stellenbosch University; [2]Institute of Psychiatry, Psychology and Neuroscience, King's College London*

**Background:** The course of schizophrenia is characterised by episodes of psychotic symptoms and enduring deficits of negative symptoms, cognition and functioning. We investigated the relationship between global brain measures and trait-related symptoms (endpoint scores), and global brain measures and state-related symptoms (change scores).
**Methods:** We examined global cortical, subcortical and white matter volume, and global cortical thickness in 54 first-episode schizophrenia patients at baseline. We performed clinical, cognitive, and neurological assessments at baseline and twelve month follow-up. We used hierarchical multiple regression to predict baseline brain measures.
**Results:** State-related clinical predictors accounted for 8% of variance in white matter volume, trait-related clinical predictors accounted for 7% of variance in subcortical volume. Trait-related cognitive scores accounted for 15% of variance in subcortical volume and 13% of variance in cortical volume. Baseline subcortical gray matter volume was significantly associated with sensory integration (0.02) and verbal learning (0.04) trait scores, cortical volume with verbal learning (0.04) trait scores, cortical thickness with social and occupational functioning (0.03) trait scores, and white matter volume with motor coordination (0.007) state scores.
**Discussion:** Impaired verbal learning may be the cognitive domain that is particularly trait-related, and possibly closest to the neurodevelopmental deficit underlying schizophrenia. State and trait components of neurological soft signs may be differentially related to brain structure. Mediators of the relationship between trait functional deficits and cortical thickness needs consideration.

### S109. SYMPTOM NETWORK MODELS OF PSYCHOSIS

Adela-Maria Isvoranu*[,1]
*[1]University of Amsterdam*

**Background:** Disorders within the psychosis spectrum are highly heterogeneous and multifactorial (Weinberger & Harrison, 2010). However, in spite of decades of research, causes of psychosis are still uncertain (e.g., Tandon et al., 2008). In an attempt to overcome these shortcomings, recent years have seen a rise in the modeling of psychotic disorders as networks of interacting symptoms (Borsboom, 2017). The centerpiece of network modeling lies in the idea that symptoms are active causal agents in producing disorder states, and that the study of their causal interaction is central to progress in understanding and treating mental disorders (Isvoranu et al., submitted). This presentation aims to introduce the network approach to mental disorders in the context of psychotic symptomatology.
**Methods:** The network approach is a novel psychometric framework based on a dynamical systems perspective. In network models, mental disorders such as schizophrenia are no longer conceptualized as common causes of symptoms, but as conditions that arise from the interaction between symptoms. The pattern on interactions can be visualized in a network structure, in which variables (e.g., symptoms, environmental factors, genetic factors) are represented as nodes and the presence of an edge between any two nodes implies the existence of a statistical association, which does not vanish upon controlling for all of the other nodes in the network (Isvoranu et al., 2016). This talk will include two examples of network models. First, using general population data a network model for the relation between three environmental risk factors (cannabis use, developmental trauma, and urban environment), dimensional measures of psychopathology and a composite measure of psychosis is constructed (Isvoranu et al., 2016). Second, using the GROUP dataset (Korver et al., 2012) which includes patients, siblings of patients, parents and controls, a network model is constructed for the relation between a polygenic risk score for psychosis liability and symptoms of psychotic disorders.
**Results:** The results of the first study indicate specific paths between environmental factors and symptoms, most often involving cannabis use (Isvoranu et al., 2016). In addition, the analysis suggests that symptom networks are more strongly connected for people exposed to environmental risk factors, indicating that environmental exposure may lead to less resilient symptom networks. The second study indicates that genetic vulnerability assessed via a polygenic risk score is associated with several individual psychotic symptoms – especially positive psychotic symptoms – suggesting that part of the missing heritability problem may be lie in the psychometric conceptualization of psychosis.
**Discussion:** Psychotic disorders feature a multitude of symptoms and problems, which lead to an inherent heterogeneity of psychosis. Current (psychometric) conceptualizations of pathology cannot fully encompass the complexity of these problems – this yields to the need of developing tools that could aid our understanding of psychiatric disorders and could ultimately be implemented in clinical practice. Network modeling may provide such a tool. It is unlikely that there is such a thing as "one-size fits all treatment" for psychosis spectrum disorders, and intervention planning may require personalized network modelling (Isvoranu et al., submitted). In the coming years we are likely to learn the extent to which the network approach could aid research and clinicians.

### S110. THE CLINICAL IMPLICATION OF CLINICIAN-RATED DIMENSIONS OF PSYCHOSIS SYMPTOM SEVERITY (CRDPSS) FOR DIAGNOSIS BY DSM-5

Beomwoo Nam*[,1], Won-Myong Bahk[2], Sang-Yeol Lee[3], Kwanghun Lee[4], Duk-In Jon[5], Eunsung Lim[6], Sung-Yong Park[7], Min-Kyu Song[8], Seongwoo Jo[1], Youngsoon Jeon[1]
*[1]Konkuk University; [2]Yeouido St. Mary's Hospital, The Catholic University of Korea; [3]Wonkwang University, School of Medicine; [4]College of Medicine, Dongguk University; [5]College of Medicine, Hallym University; [6]Shinsegae Hospital; [7]Keyo Hospital; [8]Yesan Mental Health Clinic*

**Background:** The most recently published the 5th edition of the DSM proposed a dimensional approach with continuous of schizophrenia and other psychoses. The newly proposed Clinician-Rated Dimensions of Psychosis Symptom Severity (CRDPSS) in the DSM was recommended to be evaluated in all disorders with psychotic symptoms in eight dimensions; Hallucinations, Delusions, Disorganized speech, Abnormal psychomotor behavior, Negative symptoms, Impaired cognition, Depression, Mania. The purpose of this study is to examine if Clinician-Rated Dimensions of Psychosis Symptom Severity (CRDPSS) can usefully be used for the Non-Affective Psychoses (NP) and Affective Psychoses (AP).
**Methods:** Participants in the study were 175 diagnosed with Schizophrenia, or Schizophreniform Disorder, Schizoaffective Disorder, mood disorder with psychotic symptoms (Major Depressive Disorder, Bipolar Disorder) based on DSM-5 diagnostic criteria and were assigned to either the NP (n = 154) or AP (n = 21) group. CRDPSS was performed jointly by a psychiatrist and a psychiatric resident to assess the severity of the psychotic symptoms of all the participants. And WHO Disability Assessment Schedule 2.0 (WHODAS 2.0) was responded to all participants. Independent T-test was conducted to determine whether there was a difference in CRDPSS profile and WHODAS 2.0 scores between the two groups. In addition, a linear discriminant analysis was performed to determine whether the CRDPSS profile can discriminate between the two groups.
**Results:** Demographics and WHODAS 2.0 had no statistically significant differences between the two groups. On the other hand, Patients in the NP group had higher Hallucination ($p < .05$) and Negative symptoms ($p < .001$), however, lower Mania ($p < .001$). As a result of constructing a linear discriminant function for NP and AP, the correct classification rate of CRDPSS to discriminate between two groups was 84%.
**Discussion:** The results of this study are the first to distinct effectively that Non-Affective psychoses and Affective psychoses by CRDPSS profile. There was no difference in the level of functional disability between groups NP and AP, but only CRDPSS profile could discriminate both groups. Hallucinations, Negative symptoms, and Mania were the major contributors to the distinction between the two groups. This is consistent with the previous studies that these are important in distinguishing Schizophrenia and Bipolar Disorder from each other. CRDPSS provides a new perspective that can be viewed from an integrated perspective, the NP and AP. Regarding the result of this study that it is more important to identify the score profile than the combined score of CRDPSS, because patients exhibit very heterogeneous profile of symptoms.

### S111. ARE SCHIZOPHRENIA AND SCHIZOAFFECTIVE DISORDER SEPARABLE?

Walter Heinrichs[1], Leah Hartman[1], Farena Pinnock*[,1], Farzaneh Mashhadi[1], Sarah Ciantar[1]
*[1]York University*

**Background:** Resolving the definition, heterogeneity and validity of schizophrenia-spectrum disorders remains a challenge, including the distinctiveness of schizophrenia and schizoaffective disorder. Here we report clinical, cognitive and structural brain imaging data with special reference to social processing in corresponding patient groups and non-psychiatric control participants. The study question was: to what extent do these data support schizophrenia and schizoaffective disorder as separable biobehavioural syndromes of psychotic illness?
**Methods:** DSM-V criteria were applied to an outpatient sample, yielding n=44 with schizophrenia and n=29 with schizoaffective disorder. In addition to demographic data, symptom severity was measured in both patient groups with the Positive and Negative Syndrome Scale (PANSS). Overall cognition was measured with the MATRICS Consensus Cognitive Battery (MCCB) composite and social cognition with Theory of Mind, emotion perception and attribution bias tasks. Cortical thickness in regions associated with the social brain network was measured with a 3T General Electric MRI short bore scanner, with parcellations obtained using methods described by Destrieux et al. (2010) in Freesurfer. Non-psychiatric control participants (n=63) were studied with cognitive, social cognitive and MRI measures for comparison.
**Results:** Study groups did not differ in age, educational achievement, proportion of males or prevalence of English as the preferred language. Patient groups did not differ in symptom severity (PANSS) or anti-psychotic medication (1st versus 2nd generation), but did differ significantly in terms of independent living, with schizoaffective patients significantly more independent than schizophrenia patients. The composite MCCB index and theory of mind task revealed significant differences between controls and patient groups, but no differences between patient groups. Schizophrenia patients differed significantly from both schizoaffective and control participants on the emotion perception task. There were no group differences in attribution bias. Multivariate analysis of variance (MANOVA) revealed that cortical thickness values in the social network were significantly lower in patient groups relative to controls for 14 regions. There were no schizophrenia vs schizoaffective group differences following correction. However, 9 regions were significantly reduced in schizophrenia patients relative to controls and 5 regions in schizoaffective patients relative to controls. Cingulate gyrus and superior temporal sulcus regional differences remained significant following correction.
**Discussion:** Although schizophrenia and schizoaffective disorder continue to be recognized as distinct syndromes in some diagnostic systems (e.g. DMS V), the validity of the distinction remains in question. Apart from functional independence, which may in part be an artifact of the diagnostic criteria, and aspects of emotion perception, we found no evidence to support longstanding conjectures that these syndromes are distinct, at least not in terms of the clinical, cognitive, social cognitive and social brain network-associated measures used in this study.

### S112. RELATION BETWEEN EARLY-ONSET PSYCHOSIS AND FORMAL THOUGHT DISORDER IN SCHIZOPHRENIA

Koksal Alptekin*[,1], Simge Altinok[1], Halis Ulas[1], Banu Degirmencioglu[1], Berna Akded[1]
*[1]Dokuz Eylul University*

**Background:** Formal thought disorder is one of the fundamental features of schizophrenia. Early onset schizophrenia (EOS) is strongly related to poor prognosis and illness outcomes. The aim of this study is to investigate the relation of EOS and formal thought disorder in schizophrenia.
**Methods:** This research was a retrospective study. Data regarding the patients with schizophrenia were obtained from two separate studies conducted at Dokuz Eylul University.
Thought disorder scores were compared between 32 patients with early onset schizophrenia (EOS; age ≤ 18) and 120 patients with adult onset schizophrenia (AOS; age > 18). Also, we looked at the effect of Duration of Untreated Psychosis (DUP). We further categorized these two sets as short DUP (short DUP; ≤ 6 month) and long DUP (long DUP; > 6 month) groups.
**Results:** Schizophrenia patients with early onset showed significantly higher scores compared to adult onset schizophrenia patients with regards to poverty of speech ($U= 1525.50$; $p = .037$) and peculiar sentences ($U= 1613.50$; $p = .043$).
**Discussion:** Early onset schizophrenia patients had significant formal thought disorder abnormalities. Formal thought disorder may have some developmental characteristics which indicate an important dimension related to prognosis and outcome of schizophrenia. Also, DUP may have potential effect over formal thought disorder.

### S113. THE ASSOCIATION BETWEEN SCHIZOTYPAL COMPONENTS AND CONSPIRACIST BELIEFS THROUGH COGNITIVE MEDIATORS

David Barron*[,1], Adrian Furnham[2], Laura Weis[2], Kevin Morgan[3], Tony Towell[3], Viren Swami[4]
*[1]Perdana University; [2]University College London; [3]University of Westminster; [4]Anglia Ruskin University*

**Background:** Belief in conspiracy theories (i.e., a subset of false narratives in which the ultimate cause of an event is believed to be due to a malevolent plot by multiple actors working together) is a widespread and stable aspect of contemporary public opinion. Given such findings, researchers have sought to understand the factors that make someone more or less likely to adopt conspiracist beliefs. More specifically, scholars have focused primarily on social and differential aspects, as well as possible psychopathological elements. These endeavours have led to reports of significant associations between schizotypal facets (odd or magical thinking and, to a lesser extent, ideas of references) and the endorsement of conspiracist beliefs. However, one limitation of extant findings is the assumption that the aforementioned relationships are ultimately direct; that is, schizotypal facets are directly associated with conspiracist beliefs, rather than influenced by mediating processes. To overcome this limitation, the present study sought to replicate previous findings by confirming the relationships between components of schizotypy and conspiracist beliefs. Second, this study examined the mediating influence of cognitive processes on this relationship.

**Methods:** An international online sample of 411 women and men completed measures of schizotypal components (i.e., odd beliefs or magical thinking and ideas of reference), conspiracist beliefs, and cognitive processes (i.e., need for cognition, analytic thinking, and cognitive insight).

**Results:** Through path analysis, results indicated associations between both schizotypal facets and conspiracist beliefs in the present sample. Further, there was evidence for the association between analytic thinking and conspiracist beliefs, and between cognitive insight and conspiracist beliefs. Indeed, cognitive insight was found to mediate the association between odd beliefs or magical thinking and ideas of reference with conspiracist beliefs. In addition, analytic thinking provided a mediating link to conspiracy ideation for odd beliefs or magical thinking, this was not found with ideas of reference. Despite an association between odd beliefs or magical thinking and need for cognition, this did not extend to conspiracist beliefs.

**Discussion:** In summary, the results of this study supported the association between schizotypal components and conspiracist beliefs. However, they also extend previous research by suggesting that cognitive processes mediate this link. That is, although a direct link between these variables may be tenable, it is also important to consider the possible ways in which schizotypy influences cognitive processes, which in turn have an effect on conspiracist beliefs. From a practical point-of-view, this highlights possible intervention routes for reducing conspiracist beliefs, either by targeting schizotypal traits indirectly or cognitive factors directly. While this research addresses schizotypy, patients with psychotic disorders and those with an at-risk mental state have also been shown to have reasoning biases. Therefore, future research, in relation to the clinical spectrum, should consider not only reasoning biases, but an outcome of conspiracy beliefs.

### S114. ASSOCIATION BETWEEN APATHY AND DEPRESSION: SECONDARY OR REFLECTING UNDERLYING COMMON FEATURES?

Ann Faerden*[,1], Siv Hege Lyngstad[1], Ingrid Melle[1]
[1]*Oslo University Hospital*

**Background:** Negative symptoms and depression have in many studies been found to be moderately associated, and when present, it reflects negative symptoms of a secondary nature. Primary negative symptoms are thought to be intrinsic to schizophrenia, while secondary to be caused by depression, positive symptoms and medication side effects. Instability in above associations over time is also considered to reflect a secondary origin. Despite the clinical importance of secondary negative symptoms little research has focused on what is their underlying nature. Especially apathy and depression have common clinical features and are often found to correlate. Apathy and depression are self-perceived states and self-reports could add to our understanding. Studying the underlying themes of associations between apathy and depression as well as stability over time could therefore add to the current understanding of the primary or secondary nature of negative symptoms.

**Methods:** Eighty-four first episode psychosis patients from TOP/NORMENT study in Oslo, Norway were assessed at baseline and 1-year follow-up with the Calgary Depression Scale (CDSS), Apathy Evaluation Scale (AES), both self-report (AES-S) and clinician (AES-C), and the Positive and Negative Symptoms Scale (PANSS). Correlation with total scale, individual scale items and linear regression was used to study associations and explained variance over time. Results were repeated controlling for positive symptoms and excluding those with high level of depression.

**Results:** CDSS and AES correlated at the 0.4 to 0.5 levels at both baseline and follow up, regardless of AES-S or AES-C. Hopelessness and feeling of depression were the CDSS items with stable and concurrent correlation strength to AES-S and AES-C. For CDSS, we found correlation of equal strength and stability to the AES-S- and AES-C items of getting things done during the day, spending time on interests, getting excited and taking initiative. Same significant correlations to CDSS were found for PANSS amotivation factor, but not for PANSS expressive factor. Controlling for PANSS positive symptoms did not change results, and excluding those with high levels of depression only mildly changed results.

**Discussion:** This study shows a significant correlation between apathy and depression that is stable over time for the full scale and also at the item level, regardless of self-reporting or clinician assessed apathy. Underlying themes of the concurrent correlation reflect lack of initiative and hopelessness and are in line with the defeatist beliefs found to correlate with negative symptoms, mediate between motivation and reduced effort and have recently been a target for cognitive remediation therapy. This study does not give an answer to a primary or secondary origin of apathy, but the stability points more to an underlying common nature than one being the cause of the other.

### S115. SYMPTOM DOMAINS IN SCHIZOPHRENIA AND GENE POLYMORPHISMS

Vjekoslav Peitl*[,1], Branka Vidrih[1], Draženka Ostojić[2], Tanja Franćeski[3], Milena Peitl[4], Dalibor Karlović[1]
[1]*University Hospital Center Sestre Milosrdnice, Catholic University of Croatia;* [2]*Psychiatric Clinic Vrapče;* [3]*University Hospital Centre Sestre milosrdnice;* [4]*University Hospital Centre Zagreb*

**Background:** Dopamine and serotonin neurotransmission relies mostly on the action of four factors: serotonin and dopamine transporters (SERT and DAT) and enzymes monoaminooxidase A (MAO-A) and catechol-O-mehtyltransferase (COMT). The goal of this research was to closely examine schizophrenia symptom domains in relation to the investigated polymorphisms.

**Methods:** Study group was composed of 300 schizophrenic patients. Severity of schizophrenia was assessed by the Positive and negative syndrome scale (PANSS), depressive symptoms were assessed with Calgary depression scale for schizophrenia (CDSS). SERT (5-HTTLPR), DAT (VNTR), COMT (Val158Met) and MAO-A (VNTR) gene polymorphisms were analyzed. Schizophrenia symptom dimensions were determined with multivariate statistical methods, while logistic regression and ANOVA were used to investigate the influence of a genotype on a symptom domain.

**Results:** Factor analysis of PANSS scale retained all 30 items and identified 5 separate factors (aggressive/impulsive, affective/depressive, cognitive, negative and positive symptoms). Analysis of CDSS scale revealed 2 separate factors (depression and suicidality).

Statistically significant PANSS variables were those of aggressive/impulsive and negative symptoms, while suicidality was the only significant CDSS variable.

**Discussion:** Our PANSS scale factor analysis established 5 distinct factors. Previous factor analyses provided from 3, up to 7 different factors, but mostly 5 distinct ones: negative symptoms, positive symptoms, depressive symptoms, excitement and disorganization. That factor distribution corresponds to our findings in terms of identified number of factors, but seems to differ in terms of item distribution within those factors.

When testing the influence of investigated gene polymorphisms on the variable of total PANSS score and five distinct factors we did not establish significant findings regarding four variables: total PANSS score, positive, cognitive and affective/depressive symptoms. While that is in line with majority of other investigations, SERT promoter polymorphism and COMT Val158Met gene polymorphism have been previously associated with depressive and positive symptoms. SERT and MAO-A polymorphisms separately had a significant effect on the variable of aggressive/impulsive symptoms, which has not been reported earlier. Furthermore, significant influence of COMT gene polymorphism was established for the variable of negative symptoms, which is a confirmation of some earlier reports, although there have been contrary findings. Previous reports of CDSS scale factor structure are limited to data from its initial validation to the few recent findings of its three-factor structure (depression, cognition and melancholy). We identified 2 separate factors using factor analysis, "depression" (which included seven out of nine items) and "suicidality". To the best of our knowledge this is the first investigation of the putative association between any of the four investigated polymorphisms and depressive symptoms of schizophrenia measured by the CDSS scale. Ultimately, we did not establish a significant association of investigated gene polymorphisms and total CDSS score, as well as the "depression" factor. However, there was a significant association between the "suicidality" factor and SERT and MAO-A gene polymorphisms, as well as their interaction. The fact that the significant association was established for only one of the two obtained CDSS factors suggests that the association is subtle and, at least partially, explains rarely reported associations between investigated gene polymorphisms and schizophrenia symptom domains, which is especially true for depressive symptomatology.

### S116. THE IMPACT OF PSYCHOTIC EXPERIENCES IN THE EARLY STAGES OF MENTAL HEALTH PROBLEMS IN YOUNG PEOPLE

Kareen Heinze*[,1], Ashleigh Lin[2], Barnaby Nelson[3], Renate Reniers[1], Rachel Upthegrove[1], Latoya Clarke[4], Ayesha Roche[5], Lowrie Angelique, Stephen Wood[3]

*[1]University of Birmingham; [2]Telethon Kids Institute; [3]Orygen, The National Centre of Excellence in Youth Mental Health; [4]University of Warwick; [5]University of Sheffield*

**Background:** Anxiety and depressive symptoms and psychotic experiences constitute common features of emerging mental disorders in young people. Psychotic experiences and the ultra-high risk (UHR) state for psychosis appear to have a particular importance for clinical presentation, progression of symptomatology, quality of life and functioning, but the impact of psychotic experiences in individuals seeking help at non-UHR services, compared to UHR services, is under-researched.

**Methods:** 69 young people (Mage ± SD at baseline = 20.8 ± 2.6, range 16–26 years, 48 females) presenting to mental health services were grouped according to UHR and non-UHR status. They were assessed at baseline for psychotic experiences, anxiety and depressive symptoms, psychological distress, psychosocial functioning and quality of life. They were followed up at three, six, and 12 months. Data were analysed using mixed and general linear modelling.

**Results:** UHR individuals reported higher levels of depressive symptoms and lower levels of role functioning at baseline compared to non-UHR individuals. Significant differences were evident over time for psychological distress and quality of life, with greater impairment developing in those at UHR. No robust differences were reported for anxiety symptoms or social functioning.

**Discussion:** Psychotic experiences appear to be particularly associated with depressive symptoms and psychological distress, impaired role functioning and quality of life in help-seeking young people in the medium-term. It is therefore important to pay special attention to psychotic experiences in the early stages of mental health problems even if psychotic symptoms are not the main motivation for help-seeking.

### S117. MODELLING THE RELATIONSHIP BETWEEN INSIGHT, PSYCHOPATHOLOGY AND GENDER IN SCHIZOPHRENIA USING STRUCTURAL EQUATIONS

Jesus Cobo*[,1], Javier Labad[2], Esther Pousa[3], Lourdes Nieto[4], Susana Ochoa[5], Judith Usall[5], Iris Baños[5], Beatriz González[6], Carmina Massons[7], Isabel Ruiz[8], Ada Ruiz[3]

*[1]Corporació Sanitària Parc Taulí; [2]Corporació Sanitària Parc Taulí, Hospital Universitari – UAB - CIBERSAM; [3]Institut de Neuropsiquiatria i Addiccions, Hospital del Mar; [4]Instituto Nacional de Psiquiatría Ramón de la Fuente Muñiz; [5]Parc Sanitari San Joan de Dèu - CIBERSAM; [6]Hospital Benito Menni; [7]Corporació Sanitària Parc Taulí, Hospital Universitari; [8]Universitat Autònoma de Barcelona*

**Background:** Studies about the problem of the lack of awareness of the illness in psychosis have a long trajectory of research (Amador and David, 2004). Previous reported articles exert their insidious negative effects in the evolution and managing of the illness both by the patients and professionals. Lack of insight have been related to a generally poor prognosis of schizophrenia, predisposing to non-adherence with antipsychotic and other negatives influences on outcomes. In the last years, previous analysis from the Insight Barcelona Research Group tried to develop a deeper view of insight dimensions in schizophrenia (Pousa et al., 2017).

The impact of gender in schizophrenia and first-episode psychosis has been studied extensively in recent decades (Riecher-Rössler and Häfner, 2000; Ochoa et al., 2011). Previous studies about the role of gender in the deficit of insight in psychosis showed inconclusive results. These contradictory findings may be related to differences in the study populations as well as to the use of different instruments to assess insight and other related variables. In these sense, our group presented a previous analysis focused on insight and gender (Cobo et al., 2016) into specific psychotic symptoms. Using the Spanish complete version of the Scale of Unawareness of Mental Disorder - SUMD (Ruiz et al., 2008) and the Five-PANSS Lidenmayer's Factors (1995) - Positive, Negative, Cognitive, Depressive and Excitement. No gender differences in the three main dimensions of insight in psychosis were found, neither in awareness and attribution of symptoms when assessed globally. However, gender differences appear in awareness and attribution of particular symptoms when assessed separately, with women showing higher levels of unawareness and misattribution than men. On the other hand, a different pattern of clinical, sociodemographic and functional variables seem to affect insight in men and women differently.

**Aim:** The aim of this study was to modelling the influence of psychopathological factors in the deficit of insight in psychosis, taking in account the difference of gender as a relevant variable.

**Methods:** A multicenter sample of 305 patients with schizophrenia who agreed to participate was evaluated in four centers of the metropolitan area of Barcelona (Catalonia). Psychopathological assessment was performed using the Five-PANSS Wallwork's Factors (2012). Insight and its dimensions were assessed by means of the SUMD. Structural Equation Models (SEM) was used to fix the model in the total sample and by gender.

**Results:** SEM models for the sample showed a moderate fix capacity. Insight SUMD Dimensions models in the schizophrenia sub-sample were related significantly to Positive, Excited and Depressed PANSS Wallwork's Factors. Higher scores on Positive and Excited PANSS Wallwork's Factors reduce de insight scores. Conversely, higher scores in Depressed PANSS Wallwork Factor were associated to greater awareness. In women affected of schizophrenia, SEM models fixed poorly and the lack of association for any isolated PANSS factor, probably due to the size of the subsample.

**Discussion:** There is a lack of studies in the area. In the previous SEM model of Xavier et al. (2017), disorganized symptoms had the strongest effect on insight. In addition, both in our study and in the study of Xavier et al. (2017), negative symptoms have no significative effect on either illness insight or treatment insight. In our opinion, the possible explanation of the principal differences between the studies is related to the instruments utilized and the sample characteristics. Our data also support gender differences in the influence of psychopatology factors on the insight dimensions.

### S118. CAN THE POSITIVE AND NEGATIVE SYNDROME SCALE (PANSS) DIFFERENTIATE REFRACTORY FROM NON-REFRACTORY SCHIZOPHRENIA? A FACTOR ANALYTIC INVESTIGATION BASED ON DATA FROM THE PATTERN COHORT STUDY

Rosana de Freitas*[,1], Bernardo dos Santos[1], Carlo Altamura[2], Corrado Bernasconi[3], Ricardo Corral[4], Jonathan Evans[5], Ashok Malla[6], Marie-Odile Krebs[7], Anna-Lena Nordstroem[3], Mathias Zink[8], Josep Maria Haro[9], Helio Elkis[1]
*[1]Universidade de São Paulo; [2]University of Milan, Fondazione IRCCS Ca' Granda, Ospedale Maggiore Policlinico; [3]F. Hoffmann-La Roche Ltd; [4]Fundación para el Estudio y Tratamiento de las Enfermedades Mentales (FETEM); [5]University of Bristol; [6]McGill University; [7]INSERM, Paris Descartes University, Sainte-Anne Hospital, Service Hospitalo-Universitaire, Paris; [8]Central Institute of Mental Health; [9]Parc Sanitari Sant Joan de Déu, CIBERSAM, Universitat de Barcelona*

**Background:** Treatment Resistant Schizophrenia (TRS) and Non-Treatment Resistant Schizophrenia (NTRS) may represent different biological subtypes of schizophrenia but there are few studies which investigated the distinction between these groups in terms of psychopathology. In the present study, we used both Exploratory (EFA) and Confirmatory (CFA) Factor Analyses to investigate symptom dimensions in TRS in comparison with NTRS using the Positive and Negative Syndrome Scale (PANSS).
**Methods:** Data from 1429 patients who participated in the PATTERN study a Non- Intervention Prospective Study of Patients with Persistent Symptoms of Schizophrenia) was used. TRS was defined by proxy, based on the use of clozapine (TRS) whereas NTRS used non-clozapine antipsychotics (NTRS). EFA methods included the extraction of principal components and the Varimax rotation. The number of factors was chosen based on the Kaiser criterion. Factors items were considered valid when loadings were greater or equal to 0.5. The fit to the data was evaluated by CFA in comparison with well established PANSS models using fit indexes such as: NNFI (Non-Normed Fit Index), NFI (Normed fit Index), CFI (Comparative Fit Index), RMEA (Root-Mean-Square Error of Approximation). SPSS 23.0 and R version 3.2.2 were used for statistical analyses.
**Results:** Demographic data showed that, when compared with NTRS, patients with TRS showed an earlier age of onset, a longer duration of illness, higher PANSS positive scores, a higher duration of persistent positive and negative symptoms. There were no differences between groups in terms of the duration of untreated psychosis. The EFA yielded almost the same five-factor structure in both groups namely Negative, Positive, Affective, Disorganized/Cognitive and Excitation factors. CFA showed that both models do not fit completely to the data when compared with well known PANSS factor analytical models.
**Discussion:** Data from a large cross-national sample of 1429 patients of the Pattern study showed that TRS and NTRS patients have an almost identical factor structure when evaluated by the PANSS. These results are similar to a previous study with a smaller sample which has evaluated the dimensions of the PANSS in patients with refractory schizophrenia.

### S119. MULTICULTURAL IDENTITY INTEGRATION AND SCHIZOTYPAL PERSONALITY DISORDER

Daisy Lopez*[,1], Amy Weisman de Mamani[1]
*[1]University of Miami*

**Background:** Schizotypal personality disorder (SPD) is often misdiagnosed and understudied. Moreover, when diagnosed correctly, SPD can be difficult to treat and is associated with significant functional impairment. Furthermore, SPD falls under a schizophrenia-spectrum phenotype and can aid in better understanding the trajectory, risk factors, and treatment for psychotic disorders. Given the lack of research on SPD and the underutilization of mental health services by ethnic minorities, this population may be at increased risk for poor outcomes (Delphin-Rittmon, et al., 2015). Yet, few studies assess cultural factors that may account for differences among minorities with psychotic related disorders or SPD. Multicultural identity integration (MII) may offer insights into the presentation of mental illness among ethnic minorities. According to Amoit et al.'s (2007) cognitive-developmental model of social identity configuration, there are four multiple identity configurations. The present study assessed three of the four—categorization, in which individuals identify with one of their cultural groups over others; compartmentalization, in which individuals preserve multiple, separate identities within themselves; and integration, where individuals merge their multiple cultural identities. Research finds that individuals who integrate their culture identities have better mental health outcomes, such as risk for depression, whereas those that do not integrate either culture and compartmentalize their identities, that is, maintain separate identities, have the worst outcomes (Nguyen & Benet-Martinez, 2013). We propose that individuals struggling to integrate identities and instead categorize or compartmentalize them will display higher symptom endorsement of SPD.
**Methods:** Participants included 261 ethnic minority students from the University of Miami. Students completed measures of schizotypy (Schizotypal Personality Questionnaire; Raine, 1991) and multicultural identities within the self (The Multicultural Identity Integration Scale; Yampolsky et al., 2013). All scales demonstrated good-to-excellent reliability.
**Results:** When correlating SPD symptoms to the three forms of identity integration, we found a significant correlation with categorization ($r = .14$, $p = .02$) and compartmentalization ($r = .20$, $p < .01$), however the correlation was non-significant with integration ($r = .07$, $p = .30$). When conducting a linear regression using levels of MII to predict SPD, increased levels of categorization ($\beta = .42$) and compartmentalization ($\beta = 1.53$) were associated with greater endorsement of SPD symptoms ($F(2,258) = 5.49$, $R2 = .04$, $p < .01$).
**Discussion:** As hypothesized, increased categorization and compartmentalization of multiple cultural identities were associated with greater endorsement of SPD symptoms. Poor adjustment to a new culture and consequential integration of multiple identities may place individuals at risk for developing early symptoms of SPD. However, integration of identities was not significantly related to endorsement of SPD. Therefore, it seems that although poorer integration of identities may serve as a risk factor, greater integration may not necessarily serve as a protective factor. This study is limited by the constricted age range and SES inherent in a college sample. Gathering more information on immigration status, years in the US, etc. may be helpful in highlighting nuances within the data. Interventions targeting individuals with low identity integration may be beneficial to individuals at risk of developing SPD. This is especially true given the real-world functional impairment similar to schizophrenia found among those with SPD.

### S120. FACTORS ASSOCIATED WITH SUICIDE ATTEMPTS AMONG PATIENTS WITH SCHIZOPHRENIA

Rahma Nefzi*[,1], Amine Larnaout[1], Hanen Ben Ammar[1], Emira Khelifa[1], Amina Aissa[1], Zouhaier EL Hechmi[1]
*[1]Razi Hospital*

**Background:** Suicide is the leading cause of surmortality among patients with schizophrenia.
Despite efficient antipsychotic treatments, suicide rates reaches up to 15% of death causes and near half of the patients had at least once attempted suicide. Thereby, the early identification of clinical profiles and risk factors is important for the development of management strategies.
**Methods:** A cross-sectional and retrospective descriptive study was conducted at the "F" psychiatry department at the Razi Hospital, Manouba including 56 patients with schizophrenia in period of clinical stability. The evaluation focused on sociodemographic and clinical characteristics (using the positive and negative syndrome scale (PANSS); The Calgary Depression Scale for Schizophrenia (CDSS); The Global Assessment of Functioning (GAF); The Clinical Global Impression (CGI) rating scales). Personal history of suicidal attempts was assessed.
**Results:** In this study, fourteen patients with schizophrenia (25%) never attempted suicide. 58 % (N=32) committed one or two suicide attempts. Only 8.5% (N= 5) had 3 and 4 attempts each. Number of suicide attempts was negatively correlated with the age of onset (p=0.024, r=-0.442) and the GAF score (p= 0.002, r=- 0.483). An association was found between the personal history of suicidal attempts and the existence of a triggering factor of the onset (p=0.03). A positive correlation was found with the number of hospitalization (p=0.14, r=0.663), with the PANSS items: delusions (p=0.41, r=0.358), hallucinations (p=0.12, r=0.402), Suspiciousness/persecution (p=0.35, r= 0.342) and Somatic concern (p=0.048, r=0.322); with the CDSS guilty ideas of references (p=0.008, r=0.426) and with the CGI efficacy index (p=0.32, r=0.348).
**Discussion:** The rates of suicide are the highest among patients with schizophrenia. Previous studies have estimated the prevalence of suicide attempts in individuals with schizophrenia up to 50%.
The increase in suicide attempts is associated with depressive symptoms which are very common within schizophrenia. The risk of suicide is not constant during the evolution of schizophrenia: it is the highest during the first years. A meta-analysis of 29 studies (Hawton et al., 2005) related that risk factors for suicide was higher in schizophrenic Caucasian men, those who live alone, who have recently experienced a loss, who have a family history of depression, who are more educated and have a higher IQ. Alteration of the abnormality the serotonin system may provide a biologic base to this phenomenon. It is essential to try to early detect and carefully assess the demographic and clinical profiles of patients with high risk of suicide.

### S121. JUMPING TO CONCLUSIONS, SOCIAL COGNITION AND METACOGNITION IN PEOPLE WITH A RECENT-ONSET OF PSYCHOSIS

Raquel López-Carrilero*[,1], Luciana Díaz[2], Esther Pousa[3], Eva Grasa[4], Ana Barajas[5], Maria Luisa Barrigon[6], Fermin Gonzalez Higueras[7], Esther Lorente[8], Jordi Cid[9], Isabel Ruiz[10], Irene Birules[11], Susana Ochoa[1]
*[1]Parc Sanitari Sant Joan de Deu; [2]Hospital Moyano; [3]Parc Salut Mar; [4]IIB-Hospital Santa Creu I Sant Pau; [5]Centro de Higiene Mental Les Corts, Research Unit; [6]Fundación Jiménez Díaz Hospital; [7]Servicio Andaluz de Salud Jaen; [8]Hospital Clínico Universitario Valencia; [9]Salut Mental i Addiccions IAS Girona; [10]Servicio Andaluz Salud Málaga; [11]Parc Sanitari Sant Joan de Déu*

**Background:** The reasoning bias of jumping to conclusions (JTC) consists of a tendency to have an impaired decision process in which assumptions are made having little information. JTC is one of the most widely studied cognitive biases in psychosis (Freeman, 2007; Garety and Freeman, 1999; Moritz and Woodward, 2005) due to its higher prevalence in people with psychosis in comparison to healthy participants as found by So et al. (2016) in her meta-analyses and by Dudley et al. (2016) in his systematic review and meta-analysis. Social cognition(sc) is the best defined as a set of neurocognitive processes related to understanding, recognizing, processing, and appropriately using social stimuli in one´s environment (Adolphs, 1999). The domains are: emotion processing, theory of mind, attributional style and social perception.
**Methods:** One hundred and twenty patients with a recent onset of a psychotic disorder were assessed. Jumping to conclusions (JTC) was assessed with the beads task in which the subject must take a decision regarding the probability of the extracted bead belonging to one of two jars. In task 1 a jar is presented with a ratio of 85% black beads and 15% orange beats and another jar with inverse proportion. Task 2 is the same but the probability is 60%-40%. Task 3 has the same probability 60%-40& but instead of beads, the jars contain negative and positive adjectives. JTC was considered as taking a decision after extracting one or two beads (Brett-Jones et al. 1987). A battery of questionnaires regarding social cognition was included: The Hinting Task, was used to assess ToM. (Corcoran et al. 1995; Gil et al. 2012); Emotional perception was assessed with the Emotional Recognition Test Faces (Baron-Cohen et al. 1997), and the attributional style was assessed with the Internal, Personal and Situational Attributions Questionnaire (IPSAQ; Kinderman & Bentall, 1996). Irrational beliefs were assessed with the irrational Belief Test (TCI; Calvete & Cardeñoso, 2001). The scale is composed of ten subscales: needing acceptance from others, high expectations, guilt, intolerance to frustration, worry and anxiety, emotional irresponsibility, avoidance of problems, dependence, helplessness, and perfectionism.
**Results:** Patients who performed JTC in Task 1 scored higher levels of worry and anxiety (p=0.026), perfectionism (p=0.01) and internal attribution for negative events (p=0.034) and lower in externalizing bias (p=0.042) and emotional recognition (p=0.042). Patients who performed JTC in Task 2 scored higher levels of worry and anxiety (p=0.016) and lower in emotional recognition (p=0.031). And finally, patients who performed JTC in Task 3 scored higher in internal attribution for negative events (p=0.029) and worry and anxiety (p=0.002) whereas that lower in situational attribution for negative events (p=0.004) and emotional recognition (p=0.017). ToM is not related with any of the JTC tasks.
**Discussion:** JTC is related to some aspects of social cognition, specifically with attributional style and emotional recognition. Moreover, JTC is related with perfectionism and worry and anxiety irrational beliefs.

### S122. 3-YEAR NEGATIVE SYMPTOM TRAJECTORY AND ITS RELATIONSHIP WITH SYMPTOM AND FUNCTIONAL OUTCOMES IN FIRST-EPISODE NON-AFFECTIVE PSYCHOSIS: A PROSPECTIVE 13-YEAR FOLLOW-UP STUDY

Wui Hang Ho[1], Wing Chung Chang*[,1], Yee Man Tang[1], Lai Ming Hui[1], Kit Wa Chan[1], Ho Ming Lee[1], Yi Nam Suen[1], Eric Chen[1]
*[1]The University of Hong Kong*

**Background:** Negative symptoms are a core feature of schizophrenia and are a major determinant of functional impairment. Few studies have been conducted to examine patterns of longitudinal course of negative symptoms in the early stage of illness. Differential relationships of negative symptom trajectories with long-term clinical and functional outcomes remain to be clarified. This study aimed to investigate patterns of negative symptom trajectories over 3 years, utilizing latent class growth analysis (LCGA), in patients presenting with first-episode non-affective psychosis. Predictive capacity of symptom trajectories on 13-year functional and negative symptom outcomes was also examined.
**Methods:** One hundred thirty-six Chinese patients aged 18–55 years presenting with DSM-IV first-episode schizophrenia, schizoaffective disorder, schizophreniform disorder, brief psychotic disorder or delusional disorder were assessed at clinical stabilization for first psychotic episode (baseline), 1, 2, 3 and 13 years of follow-up. Assessments encompassing premorbid adjustment, baseline symptom and cognitive profiles and functional levels were conducted. Negative symptoms were measured by High Royds Evaluation of Negativity (HEN) Scale. Individual class membership of negative symptoms derived from LCGA was based on HEN ratings at baseline, 1, 2 and 3-year follow-up.
**Results:** Three distinct negative symptom trajectories were identified including low-stable (59.6%, n=81), moderate-stable (29.4%, n=40) and high-increasing (11.0%, n=15) trajectories. Multinomial regression analysis revealed that poorer premorbid adjustment, lower baseline cognitive composite scores and more severe baseline depression predicted high-increasing trajectory membership (Nagelkerke pseudo R2=0.339, Model $\chi 2$=277.96, p<0.01). At 13 years, 88 patients (64.7%) completed follow-up assessment, with attrition analysis indicating lack of significant differences in demographic, premorbid and baseline characteristics between completers and non-completers. Analysis of covariance (controlling for premorbid adjustment, baseline cognition and depression) followed by post-hoc comparison analyses found that high-increasing trajectory was significantly associated with poorer global functional outcome and higher negative symptom levels at 13-year follow-up.
**Discussion:** Our results indicate that 11% of first-episode non-affective psychosis patients displayed persistently high levels of negative symptoms with gradual symptom worsening over 3-year follow-up. This trajectory membership was predictive of poorer negative symptom and functional outcomes 13 years after presentation. High-increasing negative symptom trajectory identified in the initial 3 years of treatment for first-episode psychosis may represent a subgroup of patients having markedly elevated risk of developing deficit syndrome in the later course of illness.

### S123. TREATMENT RESISTANT SCHIZOPHRENIA AND GYRIFICATION-BASED CONNECTOME

Olesya Ajnakina*[,1], Tushar Das[2], John Lally[1], Marta Di Forti[1], Robin Murray[1], Lena Palaniyappan[2]
*[1]Institute of Psychiatry, King's College London; [2]University of Western Ontario*

**Background:** Treatment-resistant schizophrenia (TRS) is a major cause of disability and functional impairment worldwide. Approximately 30% of patients with schizophrenia will develop TRS at some point during their illness course. Despite the staggering financial and emotional costs associated with TRS, this severe disorder is poorly understood. The pathophysiological basis of TRS is posited in part to have neurodevelopmental roots. If early brain development (<2 years of age) influences TRS, then cortical gyrification, which is often complete by 2 years of life, could be abnormal in TRS when compared to non-TRS subjects. Subtle but diffuse pathological changes that occur during early development are postulated to disrupt the maturational relationship (covariance) among brain regions, even if no localised morphological changes are seen in adult life. The disrupted structural covariance resulting from diffuse developmental dyscoordination in early life can be quantified using gyrification-based connectomes obtained using graph theory. We applied this method to baseline MRI data collected during first contact with mental health services for psychosis to predict the emergence of TRS in the next 5 years.
**Methods:** 70 patients with first episode schizophrenia spectrum disorder who presented to mental health services between 2005 and 2010 were followed up for 5 years using electronic case notes. Psychopathology was assessed at baseline with the Positive and Negative Syndrome Scale (PANSS) and symptom dimensions were derived using Wallwork's model. TRS was defined according to Health and Clinical Excellence guidelines. Structural MRI images were obtained at baseline, with minimal exposure to antipsychotics (<3 months). Local gyrification indices were computed using Schaer's method for 68 contiguous cortical regions (34 in each hemisphere) using Freesurfer's Desikan atlas. After adjusting for age, gender and intracranial volume, group-based structural covariance was estimated (68x68 correlation indices) and each subject's contribution to the covariance was quantified using a jack-knife procedure, providing one distance matrix for each subject. These matrices were used to construct distance-based gyrification connectomes using Graph Analysis Toolbox. We used a functional data analysis approach across a range of cost-thresholds to reduce multiple testing when comparing TRS and non-TRS groups.
**Results:** 17 (24.3%) of patients with first episode schizophrenia spectrum disorder met criteria for TRS at the end of the 5 years of follow up; 53 (75.7%) were non-TRS. TRS subjects had a significant reduction in small-worldness compared to non-TRS group (Hedges's g=2.09, p<0.001) and reduced clustering coefficient (Hedges's g=1.07, p<0.001) with increased path length (Hedges's g=-2.17, p<0.001).The positive symptoms were positively correlated (after adjusting for age, gender and TRS status) with higher small-worldness (r=0.414, p=0.001) suggesting that a predominantly hyperdopaminergic status that induces positive symptoms may relate to preserved small-worldness seen in non-TRS individuals, while subtle developmental changes resulting in reduced small-worldness may underlie TRS.
**Discussion:** These changes suggest that in the presence of TRS, the cortex-wide covariance in folding patterns become less organized, with reduced regional segregation as well as reduced overall integration of the morphological connectome. Such an effect may result from weakening of the tensions that arise from inter-regional connectivity in the neonatal brain. The emergence of TRS may be characterised by a neurodevelopmentally driven abnormality in structural organisation of the human cortex in those who develop schizophrenia.

### S124. RECOVERY TRAJECTORIES IN FIRST EPISODE PSYCHOSIS PATIENTS: THE ROLE OF TIMING

Rosa Ayesa-Arriola*[,1], Jose Maria Pelayo-Teran[2], Esther Setien-Suero[3], Rocio Perez-Iglesias[4], Benedicto Crespo-Facorro[3]
*[1]Marqués de Valdecilla University Hospital, IDIVAL, School of Medicine, University of Cantabria; [2]Servicio de Psiquiatría. Hospital El Bierzo SACYL Ponferrada, Castilla y León; [3]University Hospital Marques de Valdecilla -IDIVAL, University of Cantabria, CIBERSAM; [4]IoPPN, King's College London*

**Background:** Prevent symptom relapse and promote functional recovery are the two main goals of early intervention services in first episode of psychosis. Identify patterns of recovery will be important in developing and implementing targeted recovery focused interventions. The goal of this study was to explore trajectories of recovery following a first episode of psychosis.
**Methods:** A sample of 373 FEP patients was followed over 3 years. Recovery profiles in terms of symptomatic and functional remission were explored. Relapses during follow-up were considered.
**Results:** Four recovery trajectories were identified: good stable (26%), good unstable (21%), poor unstable (10%), poor stable (43%). Those who met criteria for good stable recovery more likely have less severe baseline negative symptoms (OR= 2.092; 95% CI = 0.99–4.419) and not be diagnosed with schizophrenia (OR= 2.242; 95% CI = 1.015–4.954); short DUP (OR= 2.152; 95% CI = 0.879–5.27) and low premorbid IQ (OR = 2.281; 95% CI = 0.954–5.457) increased the likelihood of good unstable recovery; less severe baseline negative symptoms (OR= 3.851; 95% CI = 1.422–10.435) and single status (OR= 4.307; 95% CI = 1.014–18.293) increased the likelihood of a poor unstable recovery when these three trajectories were compared with a poor stable recovery. Poor unstable trajectory was significantly associated with a high relapse rate (73%).
**Discussion:** Our results shed light on identifying different recovery profiles in FEP. Despite evidence for early intervention effectiveness, we should explore ways to prevent relapse and improve long-term recovery, particularly attending the role of timing in the design of interventions.

### S125. THE DYNAMIC RELATIONSHIP BETWEEN INSIGHT AND SUICIDAL BEHAVIOUR IN FIRST EPISODE PSYCHOSIS PATIENTS OVER 3-YEAR FOLLOW-UP

Rosa Ayesa-Arriola*[,1], Jose Maria Pelayo-Teran[2], Javier-David Lopez-Morinigo[3], Manuel Canal-Rivero[4], Esther Setien-Suero[5], Manuel J. Cuesta[6], Anthony David[3], Benedicto Crespo-Facorro[5]
*[1]Marqués de Valdecilla University Hospital, IDIVAL, School of Medicine, University of Cantabria; [2]Servicio de Psiquiatría, Hospital El Bierzo, SACYL, Ponferrada, Castilla y León; [3]Institute of Psychiatry, Psychology and Neuroscience, King's College London; [4]University Hospital Virgen del Rocio, Autonomus University of Barcelona; [5]University Hospital Marques de Valdecilla -IDIVAL, University of Cantabria, CIBERSAM; [6]Complejo Hospitalario de Navarra, IdiSNA, Navarra Institute for Health Research*

**Background:** Studies have established the high risk of suicide in first episode psychosis (FEP). Between 15%-26% of FEP patients attempt suicide at least once before their first contact with psychiatric services and 2–5% die from suicide. Also, many patients with schizophrenia spectrum disorders lack insight into having a mental disorder. However, the relationship between insight changes and suicidal behaviour in FEP remains poorly understood.
**Methods:** Information about suicidal behaviour was available on a cohort of 397 FEP patients. Three dimensions of insight (into mental illness, the need for treatment, and the social consequences) were measured at: baseline, 1 and 3 years after the initiation of treatment. Survival analyses examined time to suicidal behavior in relation to i) insight at baseline, ii) the closest insight measure to the suicide attempt, and iii) changes in insight during the follow-up.
**Results:** No associations were found between baseline insight dimensions and time to suicidal behaviour. However, poor insight at the evaluation closest to the suicide attempt was associated with an increased risk of suicide. Stability of insight did not affect the risk of suicidal behaviour, while changes in either direction were linked with an increased risk of suicidal behaviour, particularly worsening insight.
**Discussion:** Insight in psychosis is a dynamic concept and we demonstrated the relationship between insight and suicide risk to be equally dynamic. Poor insight seems to increase the risk, especially when insight levels change. Repeated insight assessment to detect change from early psychosis may play a role in suicide prevention.

### S126. GOOD OUTCOME IN INDIVIDUALS AT ULTRA-HIGH RISK (UHR) OF DEVELOPING PSYCHOSIS: A DELPHI STUDY

Natalia Petros*[,1], Andrea Mechelli[1], Paolo Fusar-Poli[2], Sandra Vieira[1], Emma Rowland[3], Philip McGuire[4]
*[1]Institute of Psychiatry, Psychology and Neuroscience, King's College London; [2] Institute of Psychiatry Psychology and Neuroscience, King's College London, University of Pavia; [3]King's College London*

**Background:** Long-term outcomes for individuals at risk of developing psychosis are heterogeneous; some develop a psychotic disorder, others continue to experience attenuated psychotic symptoms (APS) and some experience clinical remission and functional recovery. Existing UHR literature is primarily vulnerability- and disease-focused. In recent years, there has been a gradual shift in research to focus on more favourable outcomes, yet despite positive findings, very few UHR studies have directly investigated or even reported good outcomes in this population. Perhaps one major obstacle for this research is the lack of a sound definition of what constitutes a 'good' outcome for UHR individuals. The current study uses the Delphi method to systematically reach a consensus definition, amongst UHR clinical and research experts, of good outcome in this clinical population. To our knowledge, this is the first UHR-focused study to utilize the Delphi method.
**Methods:** A three-round online Delphi study was conducted and n=135 UHR-expert clinicians and researchers drawn from multiple continents were invited to take part. In Round 1, participants were asked to: i) select from a list of items those which they considered most important to the definition of good outcome in UHR and ii) make suggestions on ways in which good outcome could be determined in a standardised way. In Round 2, participants were asked rate the importance of each item to good outcome in UHR individuals, on a 5-point Likert scale. According to the proportion of participants who rated the items as 'essential' or 'important', items were: i) accepted as part of the consensus and included as a standard if rated by ≥80% or more of the group, ii) re-introduced in the third round and participants were given the opportunity to re-rate them if rated by between 50–79% of the group or iii) excluded if rated by less than 50% of participants.
**Results:** Forty-six (34.1%) participants responded to the first round of the Delphi process, 39 (84.7% retention rate) responded to Round 2, and 30 (76.9% retention rate) to Round 3. Of the 46 UHR-experts, 20 were psychiatrists, 17 were psychologists, 8 were researchers/lecturers and 1 was a social worker. Fifteen items were endorsed by ≥80% of the expert-participants as 'essential' or 'important' for defining UHR-specific good outcome at one-year follow-up. Items fell into one of the following categories: functioning, symptoms, other clinically relevant factors or personal wellbeing. 'Daily functional capacity' and and 'self-reported improvement in mental health' were rated as 'essential' to defining good outcome for an UHR individual at one-year, by 100% of the Delphi sample. A reduction in the distress associated with APS was deemed 'important' by 92.1% of the sample, more so than the complete remission of APS. Many similar items were rated with the same level of endorsement for the question on outcome at five-year follow-up. Twenty-one protective factors reached ≥80% endorsement for being essential or important for good outcome in UHR individuals and fell into at least one of the following categories: community support; mental health services support; cognitive factors; personal wellbeing; social network/support; substance use/abuse; daily living factors; and premorbid factors.
**Discussion:** This three-phase Delphi study achieved consensus on the core features of good outcome at one-year and five-years in the UHR population. The items that form this definition could be used in future research and clinically, to evaluate treatment and outcome of UHR individuals. They can also be of value to the development of intervention frameworks. Further studies involving other stakeholder groups, particularly individuals considered to be at risk of developing psychosis, are needed.

### S127. "HOW COULD THIS HAPPEN?": PSYCHOSIS OR DEPRESSION AS A FACTOR IN DEATH BY SUICIDE

James Overholser*[,1], Alison Athey[1], Eleanor Beale[1], Lesa Dieter[1], Craig Stockmeier[2]
*[1]Case Western Reserve University; [2]University of Mississippi Medical Center*

**Background:** Numerous factors play a role in the path of self-destruction that ends in suicide. The risk of suicidal behavior is increased when a depressed patient is also struggling with psychotic symptoms. Likewise, among adults with a psychotic disorder, suicide risk is related to depression, hopelessness, low self-esteem, social isolation and stressful life events. The present study was designed to examine the differential impact of major depression versus psychotic thinking on suicide risk in adults.
**Methods:** Subjects:
The present study evaluated 104 adults who had died by suicide. Among these suicidal adults, 81 met diagnostic criteria for a Major Depressive

Disorder at the time of their death, 10 met criteria for a psychotic disorder and 13 met criteria for the presence of both depression and psychosis.
Measures:
Structured Clinical Interview for DSM-Disorders (SCID: First et al., 1994) used informant interviews to evaluate the presence of major mental illness in the adult who died by suicide. Interviews were conducted with family members, gathering detailed information about the duration and severity of major mental illness.
Suicidal Actions Checklist (Dejong, Overholser, & Stockmeier, 2010) was used to gather information about recent stressors, previous suicide attempts and past hospitalizations for psychiatric problems. Prior research (Dejong & Overholser, 2009) has documented an adequate level of agreement for the Suicidal Actions Checklist when collected from suicide attempters as compared to family member informants.
Procedures:
Assessment procedures followed the guidelines for psychological autopsy research (Hawton et al., 1998), whereby family members were interviewed two months after the death of their loved one. In order to determine the most accurate diagnosis for each case, all records were reviewed by a psychiatrist, a clinical psychologist, a social worker, and a neuroscientist.
**Results:** Because of the small number of patients with a psychotic disorder (with or without depression), these cases were combined into one group, and several non-significant trends are reported. As compared to suicide completers with a depressive disorder, the psychotic cases were more likely to be younger ($t = 2.18$, $p < .05$), unmarried ($\chi 2 = 3.13$, $p < .08$), unemployed at the time of their death ($\chi 2 = 9.75$, $p < .01$), and more likely to meet criteria for cannabis abuse ($\chi 2 = 3.75$, $p < .06$). Suicide completers with nonpsychotic depression were more likely to meet criteria for a comorbid diagnosis of alcohol abuse ($\chi 2 = 4.36$, $p < .05$) and often had alcohol in their system at the time of their death ($\chi 2 = 4.35$, $p < .04$). Despite the higher rate of personality disorders among the depressed completers ($\chi 2 = 3.14$, $p < .08$), the psychotic cases of suicide were more likely to have a chronic course to their symptoms ($\chi 2 = 3.11$, $p < .08$) with a history of prior psychiatric hospitalization ($\chi 2 = 18.22$, $p < .01$). Although not significant, when the three groups were examined separately, the depressed psychotic cases were more likely to have attempted suicide prior to the actual death by suicide (62%) compared to the depressed non-psychotic patients (37%) as well as the psychotic non-depressed cases (40%). Qualitative analyses will examine various patterns that may have direct links to suicidal urges.
**Discussion:** Adults with a psychotic disorder have a more chronic condition that appears difficult to treat and tends to impair work or home functioning. The psychotic patients were less likely to rely on alcohol to lower their inhibitions about committing a suicidal act, suggesting other factors need to be addressed in prevention efforts. Patients may allow their psychotic thinking to guide their behavior, and when combined with depression can result in self-destructive actions.

### S128. CORRELATION OF DURATION OF UNTREATED PSYCHOSIS WITH TREATMENT RESPONSE ON THE SYMPTOM DIMENSIONS OF SCHIZOPHRENIA

Beatriz Yukie Elias[1], Daniela Koga Tonsig[1], Bruno Bertolucci*[,1], Cristiano Noto[1], Daniel Azevedo Cavalcante[1], Quirino Cordeiro[2], Rodrigo Bressan[1], Ary Gadelha[1]
*[1]Federal University of Sao Paolo; [2]Santa Casa de misericordia de São Paulo*

**Background:** Longer duration of untreated psychosis (DUP) predicts worse response to treatment and functional outcomes in first episode of schizophrenia (FES). Longer DUP also seem to particulary affect the severity of negative symptoms, but most studies enrolled previously medicated patients and did not focus on differential effects on schizophrenia symptomatic dimensions. This study investigates how DUP influences the five dimensions of symptoms of schizophrenia on antipsychotic naïve FEP patients before and after two months of treatment.
**Methods:** Drug-naïve patients at FES (n = 97) were recruited from the Inpatient Psychiatric Unit of Santa Casa de Misericórdia de São Paulo (Sao Paulo, Brazil), between 2011 and 2016. Subjects were assessed at hospital admission and after two months of follow up. All patients were treated with antipsychotics after the diagnosis was confirmed with the Structured Clinical Interview for DSM-IV (SCID-I). The Positive and Negative Syndrome Scale (PANSS) was administered at baseline and after two months of treatment. The PANSS items were grouped in five factors: positive, negative, disorganized/cognitive, mood/depression and excitement/hostility factors. The factors percentage reduction from baseline after treatment were correlated with the DUP, controlled for sex, age, years of education.
**Results:** The mean years of education of the sample was 9.2 (± 2.6 SD), mean age was 24.9 (± 7.0 SD), 62.9% were male and 42.7% were unemployed or had stopped their studies because of symptoms. Pearson correlation coefficients of the factors with DUP were: Positive = - 0.311 ($p < 0.001$); Negative= -0.340 ($p < 0.001$); Disorganized = -0.188 ($p = 0.033$); Hostility = -0.201 ($p= 0.023$); Depression = 0.030 ($p = 0.389$).
**Discussion:** Shorter DUP enhanced the early response to treatment in the positive, negative, disorganized and hostility dimensions. In line with the literature, our findings support that reducing the DUP may be one of the few interventions for a more favorable response to treatment on negative symptoms.

### S129. DOES TREATMENT RESISTANT SCHIZOPHRENIA PRESENT A CHARACTERISTIC SYMPTOMATIC SIGNATURE?

Bruno Bertolucci*[,1], José Cássio Pitta[1], Cinthia Higuchi[1], Cristiano Noto[1], Deyvis Rocha[1], Dan Joyce[2], Christoph Correll[3], Rodrigo Bressan[1], Ary Gadelha[1]
*[1]UNIFESP; [2]Institute of Psychiatry, King's College; [3]The Zucker Hillside Hospital, Hofstra North Shore LIJ School of Medicine*

**Background:** Treatment-resistant schizophrenia (TRS) may underlie a specific biological signature among patients with schizophrenia. The main lines of evidence suggest a glutamatergic rather than dopaminergic dysfunction in TRS, with lower levels of striatal dopamine and higher levels of glutamate in anterior cingulate. Whether this biological signature relates to a distinct symptomatic profile remains unclear. Our objective is to define a symptom profile of patients with TRS.
**Methods:** We used two samples of patients with schizophrenia. First, we followed a discovery sample of inpatients (n=203) to prospectively identify TRS predictors, then we tested the predictors in a replication sample of outpatients (n=207). The samples were collected independently. All patients were assessed with the Positive and Negative Syndrome Scale (PANSS), the Clinical Global Impressions-Severity Scale (CGI-S) and the Global Assessment of Functioning Scale (GAF). Diagnosis was confirmed using the Structured Clinical Interview for DSM-IV Axis I Disorders (SCID-I). TRS was defined according the criteria of the Schizophrenia Algorithm of the International Psychopharmacology Algorithm Project (IPAP). Initially, we tested if patients with disorganized subtype were more likely to be TRS, and grouped the patients into disorganized or non-disorganized schizophrenia according to SCID-I. Then, we checked which PANSS items at the baseline predicted TRS at the follow-up through multiple logistic regression analyses. A receiver operating characteristic (ROC) curve with the best items was performed at the follow-up.
**Results:** TRS was more common in disorganized schizophrenia in the inpatient sample (73.8% vs 22.4%, $P < 0.001$) and in the outpatient sample (68.2% vs 28.2%, $P < 0.001$) in comparison to non-disorganized schizophrenia. They also presented worse scores on PANSS, CGI-S and GAF ($P < 0.001$). In the second step, three PANSS items, P2 (conceptual disorganization), N5 (difficulty in abstract thinking) and G9 (unusual thought content), predicted TRS with 78.4% accuracy ($P = 0.011$, $P = 0.010$ and

P <0.001). The ROC analysis using the sum of PE+N5G+G9 predicted TRS with a sensitivity of 72.3%, and a specificity of 82.4%. In the outpatient sample, logistic regression analysis of the model P2+N5+G9 discriminated TRS with 69.3% accuracy (P <0.001).
**Discussion:** Non-paranoid clinical presentations, specially disorganized characteristics, may consist in clinical markers of TRS. Further Cross-validation of such clinical findings and biological features may improve prediction of TRS

### S130. INCIDENCE OF FIRST EPISODE OF PSYCHOSIS IN AN AUSTRALIAN COHORT AND ASSOCIATIONS WITH NEIGHBOURHOOD CHARACTERISTICS

Scott Eaton[1], Linglee Downey[1], Kristen Thien[1], Meghan Bowtell[1], Melissa Bardell-Williams[1], Aswin Ratheesh[1], Patrick McGorry[1], Brian O'Donoghue*[,1]
[1]*Orygen, the National Centre of Excellence in Youth Mental Health*

**Background:** The incidence of psychotic disorders varies between geographical areas and is associated with neighbourhood characteristics. However, the research to date has been mainly confined to Northern European and North American populations. This study will determine whether the incidence of first episode psychosis (FEP) is associated with neighbourhood characteristics, specifically social deprivation, unemployment, social fragmentation and social capital.
**Methods:** This study was conducted at the Early Psychosis Prevention and Intervention Centre (EPPIC) which provides specialist treatment to all young people aged 15–24 diagnosed with a FEP residing in a defined geographical catchment area within western and northwestern Melbourne. Census data was used to code postcodes for neighbourhood characteristics and determine the at-risk population of people aged 15–24 living within the catchment area. Incidence rate ratios were calculated.
**Results:** 527 young people treated for a FEP over a three-year period met inclusion criteria. This represents an annual incidence rate of 105.34 per 100,000 persons aged 15–24 per year. There was an increased incidence of FEP in neighbourhoods of greatest social deprivation (IRR=1.60, p=0.003), highest unemployment (IRR=1.67, p=0.001), least social capital (IRR=1.32, p=0.06) and above average social fragmentation (IRR=1.57, p=0.005). All these associations were stronger for non-affective psychoses and absent for affective psychoses. There was variation between sexes, with association only present for social fragmentation in women and social deprivation in men.
**Discussion:** This study demonstrates that the incidence of psychotic disorders varies according to neighbourhood characteristics, with higher rates in neighbourhoods with higher inequality. Services in each area should be resourced appropriately to ensure that the expected incidence can be effectively managed.

### S131. PERCEPTUAL ABNORMALITIES AND RELIGIOSITY IN ULTRA HIGH-RISK FOR PSYCHOSIS (UHR) INDIVIDUALS IN A LATIN AMERICAN POPULATIONAL SAMPLE RESULTS FROM THE SAO PAULO SSAPP COHORT

Alexandre Loch*[,1], Camille Chianca[1], Elder Lanzani Freitas[1], Julio Cesar Andrade[1], Tania Maria Alves[1], Mauricio Henriques Serpa[1], Lucas Hortêncio[1], Martinus Theodorus van de Bilt[1], Wagner Gattaz[1], Wulf Rössler[3]
[1]*University of Sao Paulo;* [3]*Charité, University of Zurich, ZInEP, University of Sao Paulo*

**Background:** In the last decades, the ultra-high risk for psychosis (UHR) status has been studied to prevent people from developing full-blown psychosis. To better understand this condition, and to better predict conversion, the relationship between UHR and several biological and environmental markers have been assessed. Nevertheless, most UHR studies come from developed countries, constituting a gap in the literature regarding culture-related environmental factors. This study aims to study religiosity, a peculiar cultural constituent of Latin-American societies, in populational samples of UHR individuals and controls from the city of Sao Paulo, Brazil.
**Methods:** These are partial results from the cohort project SSAPP (Subclinical Symptoms and Prodromal Psychosis).
Over 2500 individuals aged between 18 and 30 years were asked to participate in the research in a household survey. The Prodromal Questionnaire was used, a screening instrument for UHR constituted of 92 yes-or-no items divided into 4 domains; positive, negative, disorganization, and general symptoms. Those with 18 points or more in the positive subscale were asked to come to the Institute of Psychiatry to undergo blood testing, neuropsychological evaluation, to complete several self-filling questionnaires, and to undergo a clinical interview with an experienced psychiatrist with the SIPS (Structured Interview for Prodromal Syndromes). Functioning was assessed with the Global Assessment of Functioning (GAF), and religiosity was evaluated with the DUREL (Duke religiosity Index), which measures organizational and non-organizational religious activity, and intrinsic religiosity.
Total sample for this study was constituted of 60 UHR individuals and 91 controls.
The scores on the 5 positive symptoms items in the SIPS, the three religiosity dimensions, and the GAF were correlated, in controls and in UHR individuals.
All variables had a non-normal distribution (Kruskal-Wallis p<0.001), so Spearman's test was used. Generalized linear model was used between the resulting significantly associated variables.
SPSS 23 for Mac was used for the analysis.
**Results:** Level of organizational and non-organizational religious activity did not differ between samples, but controls had significantly higher levels of intrinsic religiosity than UHR individuals (p=0.04).
None of the religiosity measures were related to positive symptom items for controls. For UHR individuals, P4 (perceptual abnormalities) was positively related to organizational religiosity (p=0.001).
GAF was not related to any P item in controls, but they inversely correlated to P4 (perceptual abnormalities, p=0.033) and P5 (disorganized communication, p=0.031) in UHR individuals. GAF was not correlated to religiosity, neither in UHR nor in controls.
Generalized linear model using P4 as dependent variable and GAF and Organizational religiosity as independent variable showed that organizational religiosity determined P4 score rather than GAF.
**Discussion:** Results indicate that a higher score on perceptual abnormalities was related to a higher attendance in churches/temples in UHR individuals in our sample. Our study sheds light to an important aspect of Latin American cultures, namely the relationship between exceptional experiences and religion in lay people. In developing countries with a lack of mental health services churches might act as important gatekeepers for UHR individuals. Since an expected inverted correlation between GAF and religiosity was not found, we might hypothesize that religion might have been used to cope with subclinical symptoms. Longitudinal data would be required to test this hypothesis.

### S132. A NORMATIVE CHART FOR THE TRAJECTORY OF COGNITIVE FUNCTIONING IN INDIVIDUALS AT HIGH RISK FOR SCHIZOPHRENIA: LONGITUDINAL FINDINGS FROM THE INTERNATIONAL BRAIN AND BEHAVIOR CONSORTIUM ON 22Q11.2 DELETION SYNDROME

Fiksinski Ania*[,1], Jacob Vorstman[2], Anne Bassett[3], Elemi Breetvelt[3]
[1]*UMC Utrecht;* [2]*UMC Utrecht, University of Toronto;* [3]*University of Toronto*

**Background:** In schizophrenia, a decline in cognitive functioning often precedes the onset of the first psychotic episode by many years. We have previously shown this to be the case for individuals with 22q11.2 deletion syndrome (22q11DS), a genetic variant associated with a 20–25% risk of developing schizophrenia. We also observed that, regardless of the subsequent development of psychosis, individuals with 22q11DS show, on average a modest decline in IQ between 8 and 24 years and that in those who develop a psychotic disorder this decline follows a steeper trajectory. The tendency for a modest cognitive decline may represent a cognitive phenotype that is specific to 22q11DS, and possibly, an endophenotype for schizophrenia in this population. In order to assess this, we constructed a normative chart for cognitive development over time in individuals with 22q11DS.
**Methods:** We made use of the International Brain and Behavior Consortium on 22q11DS (IBBC) database that includes cross-sectional and longitudinal cognitive data from 1871 individuals with 22q11DS (mean age 15.7, SD 7.4, years; n = 330 (17.6%) with schizophrenia). All IQ measurements were obtained by qualified personnel using Wechsler tests, including WPPSI, WISC and WAIS, depending on the participants' ages. We used all available IQ data points obtained in the age range 6 - 40 years to construct normative charts for Full Scale IQ (FSIQ), Verbal IQ (VIQ) and Performance IQ (PIQ), after a comprehensive quality control procedure to ensure reliable and comparable IQ data across all international sites. We used a polynomial regression model, similar to what is used in standardized international growth charts for height and weight, to create the normative chart.
**Results:** The 4th order polynomial regression provided a good fit for the IQ data from our sample, allowing for the observed larger variability in very young age groups. On average, between the ages of 6 and 40 years, the 22q11DS population showed a gradual, modest decline in FSIQ, VIQ and PIQ. Consistent with our previous results, individuals who went on to develop schizophrenia showed a steeper decline, representing a deviation from their expected trajectory, than those who had no documented psychotic illness.
**Discussion:** This study is, to our knowledge, the first to demonstrate that a normative chart for cognitive development through to adulthood can be reliably constructed for a genetically selected high-risk population. This normative chart can be readily applied both in clinical care and in research and may serve as an example for constructing similar normative charts in other high-risk groups and/or genetic disorders.

### S133. THE RELATIONSHIP BETWEEN EXPOSURE TO TRAUMA DURING CHILDHOOD AND ADOLESCENCE AND PSYCHOTIC EXPERIENCES AT 18 YEARS OLD IN A NON-CLINICAL POPULATION

Jazz Croft*[,1], Stanley Zammit[2], Jon Heron[1], Christoph Teufel[3]
[1]*University of Bristol;* [2]*University of Bristol, Cardiff University;* [3]*Cardiff University*

**Background:** Childhood trauma (CT) is an established risk factor for the onset and development of psychotic experiences (PEs), with some evidence that interpersonal traumas being more strongly associated with risk of PEs than accidental injury or adversities such as parental divorce. However, it is not clear whether specific types of interpersonal trauma, such as emotional neglect or sexual abuse, are more strongly associated with risk of PEs than others, or whether there are critical or sensitive periods within which exposure to these carries the greatest risk.
**Methods:** Our study aimed to extend this literature by comparing the presence of multiple exposures to identify the most important types of trauma exposure and examining the association of trauma exposure with PEs throughout childhood in a large sample. We investigated whether different trauma exposures at different points of development from 0–17 years old are associated with PEs in The Avon Longitudinal Study of Parents and Children (ALSPAC) birth cohort. The measures of different types of trauma (physical abuse, emotional abuse, bullying, domestic violence, sexual abuse, emotional neglect) at different time-points (0–5 years old, 5–11 years old, 11–17 years old) were derived from questions asked throughout the longitudinal study. PEs were assessed by the face-to-face, semi-structured, Psychosis-Like Symptoms interview (PLIKSi) at 18 years old. The primary outcome was suspected or definite psychotic experiences (PEs). Multivariable logistic regression was used to examine the effects of exposure to specific types of trauma, and of exposure to trauma across specific age periods, before and after adjusting for the presence of other traumatic exposures and for confounders, including measures of socioeconomic measures (parental income, maternal education, crowded living conditions at birth), sex, measures of parental mental health, parental drug use, parental trouble with the law, IQ and genetic risk for schizophrenia, bipolar disorder and major depressive disorder. The robustness of these findings will be explored by using penalised-regression and MI methods to test specific competing hypotheses and address potential bias due to missing data.
**Results:** All trauma types exposed to between 0–17 years of age were strongly associated with PEs (adjusted ORs 1.64 – 2.13). When controlling for the presence of other trauma exposures and confounders, exposure to domestic violence at 0–5 (adjusted OR 1.67, 95% CI 1.24 – 2.26), bullying at 5–11 (adjusted OR 1.83, 95% CI 1.34, 2.48) and sexual abuse, emotional neglect and bullying at 11–17 (adjusted ORs 2.04 – 2.39) were the traumas most strongly associated with PEs ($p<.001$). Whilst exposure to any trauma at each of the age-periods was associated with PEs (adjusted ORs 1.55–2.11), the strongest association was for exposure at ages 11–17 years. The cumulative risk of reporting multiple types trauma exposures at each time-point further increased the risk of reporting PEs. Adjusting for confounders did not alter the association between trauma exposures and PEs.
**Discussion:** Previous studies have shown that different types of trauma during childhood increase the risk of PEs. Our results show that the association between trauma and PEs is not explained by a broad range of potential confounders, including genetic risk for schizophrenia, supporting a causal effect of trauma exposure on psychosis. Our findings do not support the presence of a critical period of risk, but indicate that exposure to trauma during any period of childhood or adolescent development increases risk of psychotic experiences.

### S134. THE INCIDENCE OF PSYCHOSIS IN OLDER PEOPLE: A SWEDISH POPULATION-BASED COHORT STUDY

Jean Stafford*[,1], Robert Howard[1], Christina Dalman[2], James Kirkbride[1]
[1]*University College London;* [2]*Karolinska Institutet*

**Background:** People aged 65 and above have consistently been omitted from research on the epidemiology of psychotic disorders. Correspondingly, little is known about the incidence of very late-onset schizophrenia-like psychosis (VLOSLP). We aimed to characterise the incidence of VLOSLP in a Swedish population cohort, including how incidence varied by age, sex, migration, deprivation, traumatic life events, and social isolation.
**Methods:** We conducted a Swedish population-based cohort study to examine the incidence of VLOSLP by potential environmental risk factors. The cohort, born in 1920–1949 and living in Sweden, were followed up from age 60 until the end of follow-up (30th December 2011), emigration, death, or diagnosis with a non-affective psychotic disorder. We used Cox regression to obtain hazard ratios and 95% confidence intervals for VLOSLP by age, sex, migration, disposable income at age 60, and the experience of the death of a partner or child, adjusting for potential confounders.
**Results:** In a cohort of 2,955,796 people, we identified 14,825 cases with VLOSLP, with an overall incidence rate of 38.1 (95% CI: 37.5 – 38.7) per 100,000 person-years at-risk. Rates were higher amongst migrants from North America (HR=1.4, 95% CI=1.0–1.9), Europe (HR=1.5, 95% CI=1.4–1.6), Russian-Baltic regions (HR=1.7, 95% CI=1.4–2) and Africa

(HR=2.0, 95% CI=1.4–2.7) compared to Swedish-born, with a lower rate in migrants from the Middle East (HR=0.7, 95% CI=0.5–1.0). Rates were higher in those with the lowest income (HR=3.1, 95% CI=2.9–3.3), who experienced the death of a partner (HR=1.1, 95% CI=1.0–1.2), death of a child in infancy (HR=1.2, 95% CI=1.0–1.5), and those without a partner (HR=1.8, 95% CI=1.8–1.9) or children (HR=2.6, 95% CI=2.5–2.7).
**Discussion:** In this large, national cohort study we identified several potential risk factors for developing psychosis later in life, including migration, deprivation, social isolation and traumatic life events. This may have important implications for our understanding of the aetiology of VLOSLP and could help to inform public mental health and service planning.

### S135. THE PSYCHOSIS CONTINUUM IN ELDERLY NON DEMENTED PERSONS: EVIDENCE DERIVED FROM PSYCHOTIC SYMPTOMS IN OLDER PEOPLE WITHOUT DEMENTIA FROM A BRAZILIAN COMMUNITY-BASED SAMPLE: A SEVEN YEARS' FOLLOW-UP

Walter Soares*[,1], Eriton dos Santos[1], Cassio Bottino[1], Helio Elkis[1]
[1]*University of São Paulo*

**Background:** The idea of psychosis as a dichotomous entity is generally accepted, but general population studies samples have shown individuals reporting psychotic symptoms that do not fulfill clinical criteria for any disorder. Literature suggests that psychosis phenotype is almost 50 times higher than the dichotomous psychosis concept and are more frequent in persons with lower age, lower level of education or quality of life. The prevalence of hallucinations and delusions is higher than the prevalence of psychotic clinical disorders thus suggesting evidence of a psychosis continuum, which is not adequately appreciated in the literature, particularly in elderly populations.
The purpose of the present study was to evaluate a cohort of an elderly population during a seven year follow up study aiming to determine the incidence of psychotic symptoms and their correlations with somatic and cognitive clinical aspects.
**Methods:** This is cohort study of a community-based sample of elderly subjects. Patients were evaluated by standard clinical interviews, clinical and cognitive status including the Mini-Mental State Evaluation (MMSE). At study entry in 2004, the sample was composed of 1,125 individuals aged 60 years and older. Of this total, 547 subjects were re-evaluated in 2011 and submitted to the original study protocol. Of these, 199 showed no psychotic symptoms at phase I, while 64 already had psychotic symptoms in 2004.
**Results:** The incidence of at least one psychotic symptom in the 7 years period was 8.0% and 1.0%. Visual/tactile hallucinations were the most frequent (4.5%), followed by persecutory delusions (3.0%) and auditory hallucinations (2.5%). Individuals that reported persecutory delusions had the lowest MMSE mean score (19.00). Epilepsy was a predictive variable for auditory and visual/tactile hallucinations (OR: 7.75 and 15.83); lower MMSE (OR: 0.72) and reported depression (OR: 6.48) were predictive for persecutory delusions. Visual/tactile hallucinations were predictive of cognitive impairment conversion (OR: 5.66). A total of 57.8% of individuals with psychotic symptoms developed cognitive impairment after 7 years.
**Discussion:** The presence (incidence) of subclinical psychotic symptoms in an elderly sample of a developing country like Brazil as well as the conversion rate to cognitive impairment were higher than reported in other developed countries. Visual/tactile hallucination had a crucial position in this context, was the most frequent symptom reported and was the only psychotic symptom which could predict cognitive impairment after a period of 7 years. A significant relationship was found between the incidence of psychotic symptoms and low MMSE scores, as well as clinical comorbidities such as epilepsy and reported depression. These findings provide evidence for the psychosis continuum hypothesis among elders and contribute to elucidate risk factors for these symptoms expression and its relation to cognitive impairment conversion.
This is part of a Ph.D. thesis and the correspondent article was published in PLoS One journal (doi: 10.1371/journal.pone.0178471).

### S136. A NOVEL APPROACH FOR DEVELOPING PREDICTION MODEL OF TRANSITION TO PSYCHOSIS: DYNAMIC PREDICTION USING JOINT MODELLING

Hok Pan Yuen*[,1], Andrew Mackinnon[2], Jessica Hartmann[1], Paul Amminger[1], Connie Markulev[1], Suzie Lavoie[1], Miriam Schafer[3], Andrea Polari[1], Nilufar Mossaheb[4], Monika Schlogelhofer[5], Stefan Smesny[5], Ian Hickie[6], Gregor Berger[7], Eric Chen[8], Lieuwe de Hann[9], Dorien Nieman[9], Merete Nordentoft[10], Anita Riecher-Rössler[11], Swapna Verma[12], Andrew Thompson[13], Alison Yung[14], Patrick McGorry[1], Barnaby Nelson[1]
[1]*Orygen, The National Centre of Excellence in Youth Mental Health, Centre for Youth Mental Health, The University of Melbourne;* [2]*Centre for Mental Health, The University of Melbourne, Black Dog Institute and University of New South Wales;* [3]*Orygen, the National Centre of Excellence in Youth Mental Health;* [4]*Medical University of Vienna;* [5]*University Hospital Jena;* [6]*Brain & Mind Research Institute, University of Sydney;* [7]*Child and Adolescent Psychiatric Service of the Canton of Zurich;* [8]*University of Hong Kong;* [9]*Academic Medical Center, Amsterdam;* [10]*Mental Health Centre Copenhagen, Mental Health Services in the Capital Region, Copenhagen University Hospital;* [11]*Psychiatric University Clinics Basel;* [12]*Institute of Mental Health, Singapore;* [13]*Warwick Medical School, University of Warwick, North Warwickshire Early Intervention in Psychosis Service, Coventry and Warwickshire NHS Partnership Trust;* [14]*Institute of Brain, Behaviour and Mental Health, University of Manchester, Greater Manchester West NHS Mental Health Foundation Trust*

**Background:** Ever since the establishment of strategies for identifying people at ultra-high risk (UHR) of developing psychosis about twenty years ago, much research has been conducted in seeking risk factors and in developing prediction models for predicting which UHR individuals will actually make a transition to psychosis. The goal is to provide specific interventions to those of high susceptibility. Such research almost invariably uses fixed predictor variables, typically variables assessed at baseline, i.e. service entry. Interest has now emerged to investigate whether the dynamic nature of psychopathology can be used to improve prediction of the onset of psychosis. As studies on UHR individuals usually require follow-up of participants over time, the longitudinal nature of these studies provides the opportunity to capture the dynamic characteristics of psychopathology by conducting multiple assessments across the study period. The idea is that prediction can be updated continuously as more information about changes in patients' conditions are obtained. Over the past two decades, statistical methodology that can combine the time-to-transition aspect and the longitudinal aspect of UHR studies into one model has emerged. The methodology is called joint modelling.
**Methods:** The aim is to describe the joint modelling methodology and to demonstrate how joint modelling can be used to develop a prediction model for transition to psychosis. The data from the NEURAPRO Study was used for the demonstration. This study was a multi-centre placebo-controlled randomized trial of the effect of omega-3 polyunsaturated fatty acids on transition risk in UHR individuals. The sample size was 304. Study assessments were conducted monthly during the first 6 months and then at months 9 and 12. There were in total 40 known transitions.

**Results:** Compared with the conventional approach of using only fixed predictors, joint modelling prediction models showed significantly better sensitivity, specificity and likelihood ratios.
**Discussion:** Joint modelling is a useful statistical tool which can improve the prediction of the onset of psychosis and has the potential in guiding the provision of timely and personalized treatment to patients concerned.

### S137. DO HALLUCINATIONS PREDICT THE TRANSITION FROM SUICIDAL THOUGHTS TO ATTEMPTS? RESULTS FROM AN AUSTRALIAN LONGITUDINAL COHORT STUDY

Emily Hielscher*[,1], Jordan DeVylder[2], Melissa Connell[1], Penelope Hasking[3], Graham Martin[1], James Scott[1]
[1]*The University of Queensland;* [2]*Fordham University;* [3]*Curtin University*

**Background:** Although suicidal ideation is a well-documented risk factor for suicidal behaviour, the majority of those with suicidal thoughts do not go on to make an attempt. Therefore, it is important to improve prediction of which individuals are more likely to act on their suicidal thoughts, as highlighted in Klonsky and May's (2015) ideation-to-action framework. Auditory hallucinations (AH) and psychological distress (PD) are strongly associated with both suicidal thoughts and behaviour, but their role in the ideation-to-attempt transition has not been investigated in a longitudinal dataset.
**Methods:** Participants were from an Australian longitudinal cohort of 1793 adolescents (12–17 years). Suicidal thoughts and behaviours were measured using the Self-Harm Behaviour Questionnaire. The Diagnostic Interview Schedule for Children was used to assess AH. PD was categorised using the General Health Questionnaire (GHQ) clinical cut-off. Those reporting suicidal ideation were stratified into four groups: (i) Those who did not have PD or AH (reference group), (ii) AH only, (iii) PD only, and (iv) PD and AH. Using logistic regression, we examined associations between baseline suicidal ideation, and incident suicide attempts during the 12-month follow-up, stratified by the four comparison groups. All analyses were adjusted for age and sex.
**Results:** AH were strongly and independently associated with baseline suicidal ideation (OR=3.84; 95%CI=2.46–6.02) and suicide attempts in the following 12 months (OR=3.21; 95%CI=1.18–8.76). Among adolescents with baseline suicidal ideation (n=235; 13.1%), 14 or 6.0% attempted suicide at follow-up. Those with AH only were not at significantly increased risk of transition from suicidal thoughts to attempts (OR=2.97; 95%CI=0.26–34.59). Similarly, adolescents with PD only did not have a significant increase in transition from ideation to attempts (OR=4.48; 95%CI=0.91–22.14). Adolescents who had both PD and AH had an eight-fold increased risk (OR=8.42; 95%CI=1.46–48.67) of acting on their suicidal thoughts.
**Discussion:** Adolescents with both PD and AH had the greatest likelihood of acting on their suicidal thoughts. AH alone did not significantly predict the transition from suicidal thoughts to attempts despite high odds ratios, possibly due to the low prevalence of suicide attempts among ideators and consequently limited statistical power. Future studies examining for negative and distressing content of hallucinations may assist in explaining their role in the ideation-to-attempt transition. Screening adolescents who are distressed and have hallucinations may assist with predicting those at greatest risk of future suicide attempts.

### S138. AN INVESTIGATION INTO THE ASSOCIATION BETWEEN EXPOSURE TO PRENATAL STRESS AND RISK OF PSYCHOSIS IN OFFSPRING

Ross Brannigan*[,1], Antti Tanskanen[2], Matti O. Hunnunen[3], Mary Cannon[1], Finbarr Leacy[1], Mary Clarke[1]
[1]*Royal College of Surgeons in Ireland;* [2]*Karolinska Institutet;* [3]*National Institute for Health and Welfare*

**Background:** Existing literature suggests that prenatal stress may be a risk factor for offspring psychiatric disorders. For example, large ecological studies have found that those exposed to stressors during gestation, such as war and famine, have a twofold increase risk of schizophrenia, as well as an increased risk for other affective disorders. Similarly, it was found that exposure to stressful events during pregnancy, such as the death of a relative during first trimester, increases the odds of the offspring developing schizophrenia in adulthood. In this study, our aim was to assess in a birth cohort, whether those who were exposed to prenatal stress were at higher odds for developing psychosis and other psychiatric disorders.
**Methods:** Using the Helsinki temperament cohort, a yearlong birth cohort with data collected from pregnancy onwards, logistic regressions were run examining perceived prenatal stress as a risk factor for psychosis and other psychiatric disorders. The exposure (prenatal stress) was measured using prenatal questionnaires which were given to pregnant women at antenatal clinic visits if birth was expected between 1st July 1975 and 30th June 1976. Psychiatric outcomes were assessed using linkage between the Finnish population register and the Finnish hospital discharge register in 2005.
**Results:** In total, 3660 pregnant women submitted at least one prenatal questionnaire with the mean number of prenatal questionnaires submitted per woman being 6. At the point of register access, 226 individuals had either an ICD 8, 9 or 10 diagnoses, 72 diagnosed with a psychosis disorder. It was found that those exposed to prenatal stress were at a greater risk of developing psychosis (OR = 1.54, 95% CI = 0.78 – 3.05).
**Discussion:** Our findings are in line with the current literature indicating a higher risk of psychosis among those exposed to perceived prenatal stress.

### S139. INVESTIGATING THE GENETIC ARCHITECTURE OF GENERAL AND SPECIFIC PSYCHOPATHOLOGY IN ADOLESCENCE USING SCHIZOPHRENIA POLYGENIC SCORES

Hannah Jones*[,1], Jon Heron[1], Gemma Hammerton[1], Jan Stochl[2], Peter B. Jones[2], Mary Cannon[3], George Davey Smith[1], Peter Holmans[4], Glyn Lewis[5], David EJ. Linden[4], Michael C. O'Donovan[4], Michael J. Owen[4], James Walters[4], Stanley Zammit[6]
[1]*University of Bristol;* [2]*University of Cambridge;* [3]*Royal College of Surgeons in Ireland;* [4]*Cardiff University;* [5]*University College London;* [6]*University of Bristol, Cardiff University*

**Background:** Whilst associations between polygenic risk scores (PRSs) for schizophrenia and various phenotypic outcomes have been reported, an understanding of developmental pathways can only be gained by modelling comorbidity across psychopathology, something no studies have done to date. We examine how genetic risk for schizophrenia relates to a broad range of adolescent psychopathology using a latent modelling approach, and compare this to genetic risk for other psychiatric disorders, to gain a more comprehensive understanding of development pathways at this age.
**Methods:** PRSs for schizophrenia, major depressive disorder, neuroticism and bipolar disorder were generated for individuals in the Avon Longitudinal Study of Parents and Children (ALSPAC) birth cohort. Multivariate linear regression was used to examine relationships of these PRSs with psychopathology factors modelled within i) a correlated factors structure, and ii) a bifactor structure.
**Results:** The schizophrenia PRS was associated with an increase in factors describing psychotic experiences, negative dimension, depression, and anxiety, but once modelling a general psychopathology factor specific effects above this persisted only for the negative dimension. Similar factor relationships were observed for the neuroticism PRS, with a (weak) specific effect only for anxiety once modelling general psychopathology.
**Discussion:** Psychopathology during adolescence can be described by a general psychopathology construct that captures common variance as well as by specific constructs capturing remaining non-shared variance. Schizophrenia risk genetic variants identified through genome-wide association studies mainly index negative rather than positive symptom psychopathology during adolescence. This has potentially important implications both for research and risk prediction in high-risk samples.

### S140. VOICE-SELECTIVE FORWARD MODEL ABNORMALITIES IN NONCLINICAL VOICE HEARERS

Ana Pinheiro*[,1], Michael Schwartze[2], Sonja Kotz[2]
*[1]University of Lisbon; [2]Maastricht University*

**Background:** Auditory verbal hallucinations (AVH) are one of the cardinal symptoms of psychosis but they are also present in 6–13% of individuals in the general population. Impaired predictive internal forward modelling has been proposed to underlie the experience of AVH in psychotic patients, but it remains unclear whether similar abnormalities are also present in nonclinical voice hearers. The current study sought to answer the question of whether and how hallucination predisposition modulates sensory prediction of tones and voices using event-related potentials (ERP) of the electroencephalogram (EEG).
**Methods:** Participants with low (n=15) and high (n=17) hallucination predisposition, classified based on their Launay-Slade Hallucination Scale (LSHS) scores, were tested in an auditory task involving presentation of self-triggered and externally triggered tonal or own voice stimuli.
**Results:** Participants with low and high hallucination predisposition displayed comparable N1 suppression effects to self-triggered tones (no significant group effect – $p>.05$) but the latter displayed enhanced N1 (group x condition x ROI interaction - $F(4, 120)=7.971$, $p<.001$) and reduced P2 (group x condition x ROI interaction - $F(4, 120)=5.626$, $p<.001$) responses to their self-triggered voice. Further, pre-stimulus alpha power was enhanced for self-triggered voices compared to tones in individuals with high hallucination predisposition (group x stimulus type interaction - $F(1, 30)=4.479$, $p=.043$). Anomalies in forward modelling were specifically associated with LSHS auditory hallucination scores ($r=-471$, $p=.003$).
**Discussion:** Together, these findings suggest that altered forward modelling of one's own voice is core to AVH. These results also provide partial support for the continuum model of psychosis, suggesting that psychotic symptoms form a continuum in the general population. A voice-specific, rather than a generalized, forward model dysfunction may explain why hallucinated voices are the most common type of auditory hallucinations.

### S141. TRANSGENIC OVEREXPRESSION OF THE TYPE III ISOFORM OF NEUREGULIN 1 IN MICE INDUCES ABNORMALITIES ON AUDITORY EVENT RELATED EEG BIOMARKERS RELATED TO SCHIZOPHRENIA

Holger Rosenbrock*[,1], Wiebke Nissen[2], Roberto Arban[2], Rossner Moritz[3], Schwab Markus[4], Cornelia Dorner-Ciossek[2], Niklas Schülert[2]
*[1]Boehringer Ingelheim Pharma GmbH & Co KG; [2]CNS Diseases Research, Boehringer Ingelheim Pharma GmbH & Co KG; [3]Ludwig-Maximilians-Universität München; [4]Hannover Medical School*

**Background:** Genetic, post-mortem and preclinical studies in transgenic mice repeatedly implicate neuregulin 1 (NRG1) as a critical component in the pathophysiology of schizophrenia. Its predominant neuronal receptor, ErbB4, is primarily expressed in fast-spiking interneurons enabling the maintenance of normal excitatory/inhibitory balance (E/I balance) of neuronal networks. Changes in E/I balance can be assessed in-vivo via special electroencephalography (EEG) techniques and have become an important preclinical and clinical readout to investigate the underlying mechanisms of psychiatric disorders. In fact, patients with schizophrenia show aberrant processing of sensory information leading to deficits in auditory event-related potentials (AERP), the detection of deviant auditory stimuli (mismatch negativity, MMN) and the 40Hz auditory steady-state response (ASSR) as well as to increased basal gamma oscillation.
Patients with Schizophrenia carrying NRG1 HapICE risk alleles appear to overproduce the NRG1 type III isoform in their brain. In the transgenic mouse, NRG1 type III overexpression (HANI mice; Velanac et al., 2012) results in altered synaptic activity and in behavioural changes like reduced prepulse inhibition and impaired cognition compatible with a schizophrenia-related phenotype (Agarwal et al, 2014). In the present study, the potential disruption of the E/I balance in HANI mice has been investigate via EEG recording.
**Methods:** Superficial electrodes were implanted above the auditory cortex and the frontal cortex. We used a novel wireless neurologger system for the recording of EEG data in awake freely moving mice. Data analysis was performed with commercially available software which is also used in clinical setting.
**Results:** Overexpression of NRG1 abolished MMN, significantly increased the P1 and N1 amplitude of AERP, increased basal gamma oscillation and reduced phase-lock coherence in the 40 Hz ASSR compared to the wildtype littermates.
**Discussion:** In this study we showed for the first time that overexpression of NRG1 leads to deficits in event-related EEG biomarkers supporting the notion that the NRG1-ErbB4 pathway is involved in maintaining the E/I balance, sensory stimulus processing and ultimately cognitive function. Our results indicate that the NRG1 III tg mouse model represents a tool with high translational potential to investigate pathological mechanisms related to schizophrenia.

### S142. RESTING STATE NETWORKS ALTERATION IN BIPOLAR DEPRESSION

Gianluca Mingoia*[,1], Igor Nenadic[2]
*[1]Aachen University; [2]Jena University Hospital*

**Background:** While functional MRI and PET studies have shown altered task-related brain activity in bipolar depression, recent studies suggest that such differences might also be found in the resting state (RS). Here we used ICA based analysis to investigate RS fMRI data to compare connectivity of 11 well known networks (Auditory, Cerebellum, DMN, Exectutive Control, Fronto-parietal 1, Fronto-parietal 2, Salience, Sensorimotor, Visual1, Visual2, Visual3 network) between patients with bipolar depression and healthy controls suggesting deficits in related neuropsychological functions.
**Methods:** We obtained RS fMRI series (3T, 3x3x3mm resolution, 45 slices, TR 2.55s, 210 volumes) in 22 bipolar patients (mean age 38.4a±11.3), on stable medication and 22 matched healthy controls (36.8a±11.7).
Subjects were asked to lie in the scanner keeping eyes closed with no further specific instructions. Data were pre-processed; we applied FSL MELODIC (pICA) yielding IC, we used FIX to auto-classify ICA components which represent artifacts and an automated routine to select for each subject the component matching the anatomical definition of resting state networks.
SPM12 was used for second level analysis, we used two sample t-test to compare networks functional connectivity between groups.
**Results:** Our method reliably identified all networks in every controls and patients. We found significant differences in the anatomical pattern of areas. Patients showed decreased functional connectivity in comparison to healthy controls in portions Cerebellum, DMN, Fronto-parietal1,

Fronto-parietal2, Visual1, Visual2 and Visual3 networks; in addition, patients showed increased functional connectivity in comparison to healthy controls in portions of Cerebellum Frontoparietal1 networks.
The power spectrum of the bipolar patients and healthy control time courses don't differ significantly in any of the brain networks, but there is a slight difference between the average slope between bipolar and healthy subject, Total Av. Bip = -0.88743 and Total Av. HC = -0.90282.
**Discussion:** Well-known resting state networks were reliable identified from RS fMRI in Bipolar depression patients. The differences in anatomical distribution point to possible alterations in functional connectivity in Bipolar depression, which suggests disruption in cerebellum, DMN, fronto-parietal and visual neuropsychological related activity.

### S143. NEURAL CORRELATES OF INTENTION AND BELIEF INFERENCE RELATIVE TO EMOTION ATTRIBUTION TO OTHERS IN SCHIZOPHRENIA AND PSYCHOSIS PRONENESS: ACTIVATION LIKELIHOOD ESTIMATION META-ANALYSIS

Ksenija Vucurovic*[,1], Stéphanie Caillies[2], Arthur Kaladjian[1]
*[1]Laboratory Cognition, Health and Society, University of Reims Champagne Ardenne; [2]Laboratory Cognition, Health and Society, University of Reims Champagne Ardenne, University Hospital in Reims*

**Background:** Social cognition can be briefly defined as the ability to interact with and understand others. It involves several cognitive processes that are considered as critical for an adapted social functioning. In patients with schizophrenia (SCZ) and subjects prone to psychosis (PP), a number of studies have revealed impairments in the abilities to infer beliefs, intentions or emotions of others. Cognitive tasks specifically addressing these abilities have also revealed abnormal neural processing in these subjects. However, these studies have not yet been compared in order to identify shared or distinct functional brain networks underlying these processes in the two subject groups.
Therefore, we aimed to determine whether the neurofunctional correlates of intention/belief attribution are distinct from those of emotional inference in SCZ and PP compared to healthy controls (HC). We further attempted to identify neuroimaging markers of psychosis endophenotype in mentalizing tasks. Finally, we examined shared and distinct brain regions involved in intention/belief attribution relative to emotional inference in SCZ.
**Methods:** Using a neural coordinate-based Activation Likelihood Estimation (ALE) meta-analysis, we investigated differences in activation patterns between intention/belief and emotion attributions to others in SCZ and PP relative to HC.
**Results:** We selected 33 studies after a systematic review of the literature. Inferring intentions/beliefs in SCZ patients correlated with decreased functional activation in the medial prefrontal cortex (mPFC) and left posterior temporoparietal junction (TPJ). In PP subjects, precuneus, posterior cingulate gyrus, middle and superior temporal gyri displayed additional under-activation pattern, while posterior cingulate, right TPJ, left lateral PFC and insula were over-activated. In patients with SCZ thalamus and striatum, right dorsolateral PFC, right insula, and right transverse temporal gyrus were under-activated during emotion attribution to others, while left ventrolateral PFC, left insula, right lingual gyrus and areas in the cerebellum were over-activated. Finally, in PP subjects, right TPJ was under-activated while left parahippocampal, middle and superior temporal gyri, were over-activated during affective mentalizing. Conjunction analyses demonstrated under-activation in left rostral mPFC and left fusiform gyri in both SCZ and PP relative to HC during intention/belief inference tasks.
**Discussion:** Our results suggest abnormal neural functioning in fast emotional appraisal and subsequent cognitive modulation during emotion perspective taking in SCZ. In PP, abnormal activation was observed only in cortical regions well known as recruited in emotional top-down regulation. When there is no emotional content in perspective taking like in intention/belief attribution to others, two core regions appeared as under-activated in SCZ and PP, namely left rostral portion of mPFC and, to a lesser extent, the left fusiform gyrus, suggesting that these two regions play a role in top-down modulation of cognitive mentalizing and could be neuroimaging markers of psychosis endophenotype. Thus, abnormal functioning in these specific brain areas could be a valuable predictor for developing schizophrenia in at-risk subjects. Moreover, these brain regions could be targeted by non-invasive neuromodulation techniques in order to restore cognitive function.

### S144. THE ASSOCIATION BETWEEN BRAIN ACTIVITY IN THE PREFRONTAL CORTEX AND DEMOGRAPHIC VARIABLES: A LARGE-SAMPLE FUNCTIONAL NEAR-INFRARED SPECTROSCOPY STUDY

Shinsuke Koike*[,1], Eisuke Sakakibara[1], Yoshihiro Satomura[1], Hanako Sakurada[1], Mika Yamagishi[1], Jun Matsuoka[1], Naohiro Okada[1], Kiyoto Kasai[1]
*[1]University of Tokyo*

**Background:** A functional near-infrared spectroscopy (fNIRS) has an advantage of easy measurement of the activity in the surface of the cortex with a naturalistic position. Therefore, fNIRS has been used as an aid for differential diagnosis of depressive symptoms as a clinical application in Japan. However, the fNIRS diagnosis system is not considered gender, age, and task performance which could be associated with brain activity. We previously reported that the fNIRS brain activity was associated with gender, age, cognitive performance, age at onset, and clinical stages of psychosis. Therefore, we intend to explore the association between fNIRS brain activity in the prefrontal cortex and demographic variables using a large sample size.
**Methods:** Of 163 patients with schizophrenia and 470 healthy controls who were measured using a fNIRS instrument from April 2004 to April 2016, 224 measurements from 152 patients and 475 from 386 controls were analyzed after exclusion by the criteria. We analyzed the intensity and timing of brain activity during the letter version of a verbal fluency task in the subregion of the prefrontal cortex.
The associations between brain activity and demographic variables were tested using general linear mixed models with the main effect of gender, age, group and interaction by group as fixed effects, and measurement time and interval by participant as random effects. We compared the models including all possible combination of the fixed effects. Then we further tested the association between brain activity and measurement time, measurement interval, task performance, sleepness, premorbid IQ, handedness, and education year by adding the main effect of each variable and interaction by group into the best-fitted model.
**Results:** Model comparison showed that the best fitted and reliable model included the main effects of gender, age, and group for the intensity of brain activity in the prefrontal cortex. The intensity was smaller when female, older, and schizophrenia group. The best model for the timing included main effects of group and task performance, showing the timing was earlier when control group and better task performance.
**Discussion:** To the best of our knowledge, this is the first study which investigated the association between brain activity and demographic variables in a large sample set assessed by the same instrument and task. In future, the improvement of the clinical application fNIRS system adding to demographic variables is needed.

### S145. ANTIPSYCHOTIC DISCONTINUATION IN FIRST EPISODE PSYCHOSIS: [18F]DOPA AND [11C]RACLOPRIDE PET STUDY

Euitae Kim*,[1]
[1]*Seoul National University Bundang Hospital*

**Background:** Recent meta-analysis revealed that elevated presynaptic striatal dopaminergic function is a robust feature of psychosis like schizophrenia. Considering increased dopaminergic capacity in psychotic disorders, it is not surprising that antipsychotic drugs, which primarily block dopaminergic neurotransmission, are mostly effective in the treatment of psychosis. However, it remains obscure what would happen to presynaptic dopaminergic function with antipsychotic treatment. This is an important issue addressing whether the current antipsychotic drugs are correcting the primary dopaminergic abnormality or not. In addition, the issue can give a clue regarding the mechanism of relapse in psychotic disorders.
**Methods:** We measured presynaptic dopamine capacity using [18F]DOPA PET before and after the antipsychotic discontinuation in first episode psychosis. The binding potentials of [11C]raclopride were also measured after the discontinuation. Healthy controls had [18F]DOPA and [11C]raclopride scans at the corresponding date.
First episode psychosis patients were carefully monitored in the aspects of symptomatic aggravations.
**Results:** The presynaptic dopamine capacity and the density of dopamine receptors showed significant group effect and the interaction between group and time ($p<0.005$)
**Discussion:** Dopaminergic function seems to play a critical role in relapse of first episode psychosis.

### S146. EFFECT OF CLOZAPINE ON REGIONAL CEREBRAL BLOOD FLOW IN TREATMENT-RESISTANT SCHIZOPHRENIA

Kyra-Verena Sendt*,[1], Grant McQueen[1], Amy Gillespie[1], James MacCabe[1], Fernando Zelaya[1], Alice Egerton[1]
[1]*Institute of Psychiatry, Psychology & Neuroscience, King's College London*

**Background:** Approximately one-third of schizophrenia patients will not respond adequately to conventional antipsychotic treatment; termed treatment-resistant schizophrenia (TRS). The only antipsychotic recommended for this group is clozapine, which may have unique efficacy in improving residual symptoms. The biological mechanisms underlying its efficacy are poorly understood. Previous studies have examined the effects of clozapine on regional cerebral blood flow (rCBF) using radiotracer approaches in relatively small samples of patients, showing, in particular, frontal and limbic perfusion changes1,2,3. In this study, we evaluate the effects of clozapine on rCBF, measured with a non-invasive MRI technique - pulsed continuous arterial spin labelling (pCASL) - which does not require radiotracer injection, as part of an ongoing study to identify neuroimaging predictors and mediators of clozapine response.
**Methods:** Participants ≥18 years of age with TRS were recruited at the Institute of Psychiatry, Psychology & Neuroscience, Kings College London (UK). TRS status was ascertained by the documented failure to respond to at least two different antipsychotic trials of adequate length. Participants were either clozapine-naïve or had not taken clozapine for at least three months prior to the baseline MRI scan. After baseline MRI, clozapine was administered as part of routine clinical care for 12 weeks, after which a second MRI scan was performed. Symptomatic response was defined as a reduction of 20% of the Positive and Negative Syndrome Scale (PANSS)4 score and non-response was defined as <20% decrease in PANSS score.
pCASL data was acquired on a General Electric 3 Tesla MR-750 MR scanner. Arterial blood was labelled using a long, adiabatic (1.8 seconds) radio frequency pulse. After a post-labelling delay of 2.025s, perfusion images were acquired with a 3D Fast Spin Echo spiral multi-shot readout (TE 32ms/TR = 5500ms; ETL = 64). Cerebral blood flow (CBF) maps were computed with a spatial resolution of 2x2x3mm, in a total acquisition time of less than 6min. CBF maps were pre-processed using the Automatic Software for ASL processing (ASAP) toolbox5. Changes in rCBF after 12 weeks of clozapine were analysed in a full factorial ANOVA design, using SPM 12 (www.fil.ion.ucl.ac.uk/spm). Clusters of significant CBF changes were assessed at $p<0.05$ after Family-Wise Error correction for cluster extent, using a cluster-forming threshold of $T>2.74$.
**Results:** This is an interim analysis of 24 patients who completed both scans. Contrasts were examined at a whole brain, assumption-free voxel-wise analysis, restricted to grey matter and co-varied for global perfusion. Clozapine administration significantly decreased perfusion in the medial frontal gyrus. There was also a significant response x time interaction, centred in the left posterior cerebellum and extending to the bilateral visual cortex and right precuneus.
**Discussion:** These interim results indicate that pCASL may be able to identify brain regions in which activity is modulated by clozapine administration as well as areas that may mediate symptomatic improvement. A key question for future analyses will be the degree to which rCBF may predict symptomatic response to clozapine, as the ability to predict a good likelihood of response could enable earlier clozapine initiation.

**References:**
1. Lahti AC et al. Biol Psychiatry 2003; 53: 601–8.
2. Potkin SG et al. Mol Psychiatry 2003; 8: 109–13.
3. Molina V et al. Prog Neuropsychopharmacol Biol Psychiatry 2008; 32: 948–54.
4. Kay SR et al. Psychiatry Res 1988; 23: 99–110.
5. Mato Abad VM et al. Magn Reson Imaging 2016; 34: 334–44.

### S147. ASSOCIATION BETWEEN SOCIAL ANHEDONIA AND TOPOLOGICAL PROFILE OF BRAIN NETWORK IN SCHIZOPHRENIA

Zhi Li*,[1], Raymond Chan[1]
[1]*Institute of Psychology, Chinese Academy of Sciences*

**Background:** As a refractory negative symptom, social anhedonia is prevalent in people with schizophrenia spectrum disorders. Furthermore, schizophrenia is conclusively correlated with disorganized functional brain network reflected by topological profile. However, studies on the relationship between social anhedonia and topological properties of functional brain network in schizophrenia were limited to a large extent. In the present study we explored the neurofunctional mechanism of social anhedonia in schizophrenia from the perspective of topological profile of functional brain network.
**Methods:** Six-minute resting-state fMRI images were acquired from 65 patients with schizophrenia in a 3T SIMENS scanner. Topological properties of functional brain network derived from the resting-state fMRI image, including clustering coefficient, global efficiency and small-worldness were calculated. The social anhedonia of each participant was measured with the Chapman Social Anhedonia Scale. Due to the wide-range of duration of illness in patients with schizophrenia, we included the duration of illness, social anhedonia and their interaction into a generalized linear model to predict the three topological properties, with gender, age and education years as covariates.

**Results:** We found that the clustering coefficient of brain functional network in schizophrenia increased (p = 0.032, beta = 0.000194 at the minimum network sparsity 35% in which all the nodes were fully connected), whereas the global efficiency decreased (p = 0.005, beta = -0.000022), as the progression of schizophrenia. Although the main effect of social anhedonia in predicting both the clustering efficient and the global efficiency were not significant, its interaction with the duration of illness was significant (p = 0.021, beta = 0.000038 for the clustering coefficient; p = 0.023, beta = -0.000003 for the global efficiency).
**Discussion:** With the development of schizophrenia, the increase of clustering coefficient and decrease of global efficiency of functional brain network may reflect the pathophysiology of schizophrenia since the onset of illness. Social anhedonia plays as a mediator between the altered topological profile of brain network and the progression of schizophrenia.

### S148. DIFFERENTIAL NEURAL REWARD MECHANISMS IN TREATMENT RESPONSIVE AND TREATMENT RESISTANT SCHIZOPHRENIA

Lucy Vanes*[,1], Elias Mouchlianitis[1], Tracy Collier[1], Bruno Averbeck[2], Sukhi Shergill[3]
[1]*Institute of Psychiatry Psychology and Neuroscience, King's College London;* [2]*National Institute of Mental Health;* [3]*Cognition Schizophrenia and Imaging (CSI) Lab*

**Background:** The significant proportion of schizophrenia patients refractory to treatment targeting the dopamine system suggests that more than one mechanism may cause psychotic symptoms. Reinforcement learning tasks have frequently been employed in schizophrenia to assess dopaminergic functioning and reward processing, but studies have not directly compared groups of treatment-refractory and non-refractory patients.
**Methods:** In the current functional magnetic resonance imaging study 21 patients with treatment resistant schizophrenia (TRS), 21 patients with non-treatment resistant schizophrenia (NTR), and 24 healthy controls (HC) performed a probabilistic reinforcement learning task, utilising emotionally valenced face stimuli which elicit a social bias toward happy faces. Behavior was characterized with a reinforcement learning model. Trial-wise reward prediction error (RPE) signaling and the differential impact of emotional bias on these reward signals were compared between groups.
**Results:** Patients showed impaired reinforcement learning relative to controls, while all groups demonstrated an emotional bias favouring selection of the happy faces. The pattern of RPE signaling was similar in HC and TRS groups, whereas NTR patients showed significant attenuation of RPE-related activation. The TRS patients differed from the NTR patients in the relationship between emotional bias and subcortical RPE signal during negative feedback.
**Discussion:** TRS can be dissociated from NTR on the basis of a different neural mechanism underlying their symptoms. The data support the hypothesis that a favourable response to antipsychotic treatment may be contingent on dopaminergic dysfunction, characterized by aberrant RPE signaling, whereas treatment resistance may be characterized by an abnormality in distinct cognitive mechanisms interacting with this response.

### S149. EFFECTS OF INTRANASAL OXYTOCIN ON RESTING CEREBRAL BLOOD FLOW IN PEOPLE AT ULTRA-HIGH RISK FOR PSYCHOSIS

Cathy Davies*[,1], Grazia Rutigliano[1], Marco Cappucciati[1], Andrea De Micheli[1], Valentina Ramella-Cravaro[1], Umberto Provenzani[1], Andre Schmidt[1], Yannis Paloyelis[1], Philip McGuire[1], Paolo Fusar-poli[1]
[1]*Institute of Psychiatry, King's College London*

**Background:** Recent research suggests that individuals at ultra-high risk for psychosis (UHR) show altered resting cerebral blood flow (rCBF) in key regions linked to psychosis pathophysiology: the hippocampus, midbrain, and basal ganglia. Greater perturbations in basal ganglia rCBF were correlated with positive psychotic symptoms, while remission from the UHR state was associated with a longitudinal normalization of hippocampal rCBF. Oxytocin -a neuropeptide with potential anxiolytic and prosocial properties- is currently under investigation as a novel therapeutic for a number of neuropsychiatric disorders. Previous work conducted in healthy males demonstrated that a single acute dose of intranasal oxytocin had marked effects on rCBF across all of the aforementioned regions (hippocampus, basal ganglia, midbrain), as well as the amygdala, anterior cingulate cortex and cerebellum - regions where neurofunctional alterations have been previously reported in UHR groups. Despite these findings, no studies have yet examined the effects of intranasal oxytocin on resting perfusion in UHR individuals.
**Methods:** In a double-blind, placebo-controlled, crossover design, 30 UHR males underwent two MRI scans at 3 Tesla, once after 40IU intranasal oxytocin and once after matched placebo (one-week wash-out). Arterial spin labeling (ASL) was used to measure rCBF starting approximately 22 minutes post-intranasal administration. The severity of attenuated psychotic symptoms was assessed using the Comprehensive Assessment of At-Risk Mental States (CAARMS). Measures of social cognition, emotional processing and level of functioning were also acquired. We hypothesized that relative to placebo, a single acute dose of intranasal oxytocin would modulate rCBF in the hippocampus, basal ganglia and midbrain, and that this effect would be greater in those with more severe baseline deficits in social and emotional functioning.
**Results:** Data analysis is currently ongoing and the results will be presented at the conference.
**Discussion:** These results will provide physiological evidence for a potential first-in-class intervention for UHR patients. Given the current lack of evidence for effective treatments in this patient group, better understanding of the neural correlates of the high-risk state and the physiological basis for the effects of novel therapeutics is desperately warranted.

### S150. DOPAMINE SYNTHESIS CAPACITY IN ANTIPSYCHOTIC NAÏVE FIRST EPISODE PSYCHOTIC PATIENTS

Anne Sigvard*[,1], Karen Tangmose[1], Kirsten Borup Bojesen[1], Kasper Jessen[1], Mette Ødegaard Nielsen[1], Dan Fuglø[2], Lars Thorbjørn Jensen[2], Egill Rostrup[1], Birte Glenthøj[1]
[1]*Center for Clinical Intervention and Neuropsychiatric Schizophrenia Research, Mental Health Centre Glostrup, University of Copenhagen;* [2]*Herlev Hospital, University of Copenhagen*

**Background:** Insufficient response to antipsychotics constitutes a challenge in the treatment of patients suffering from schizophrenia. Treatment resistances have been linked to a normal striatal dopamine system. We aim to stratify antipsychotic-naïve first-episode patients based on striatal dopamine synthesis capacity (DSC) measured with positron emission tomography (PET). We hypothesize that patients who respond to treatment have an increased DSC at baseline compared to non-responders and healthy controls (HC).
**Methods:** The current data have been collected as a part of a multimodal first episode study. Patients are examined before and after 6 weeks treatment with flexible doses of Aripiprazole.
PET: Dynamic scans are performed in an integrated PET-CT scanner using the tracer 3,4-dihydroxy-6-[18F]fluoro-L-phenylalanine (18F-FDOPA). Duration of scanning is two times one hour, with half an hour break.

Arterial blood samples are collected during the scanning sessions and provide information on the ratio between intact 18F-FDOPA and the primary metabolite. The input functions used for this data analysis are image derived based on the largest cerebral vessels and metabolite corrected.
Regions of interest (ROIs) are manually drawn around the cerebellum and semi automatically around the basal ganglia. In this preliminary work, DSC values are based on Ki parameters obtained from slopes on Patlak plots.
**Results:** DSC has been measured at baseline on 16 patients (mean age 22.6 years, 5 males) and 18 HC (mean age 21.7 years, 9 males), with no significant difference in age or gender between groups.
At baseline patients had a PANSS total score of 74 (SD 9.6) and GAF total score of 35 (SD 5.2).
No significant difference in Ki at baseline was shown between patients and HC. Clinical follow-up data was available on 12 patients. They received a mean Aripiprazole-dose of 9.3 (SD 3.9). Paired t-test at showed a significant effect of treatment with a follow up PANSS total of 55 (SD 11.9, p<0.001) and GAF total of 49 (SD 11.2, p=0.002).
Six patients were characterized as responders, and six as non-responders using the Nancy Andreasen remission criteria. There were no baseline differences in PANSS or GAF scores between responders and non-responders. Nor did the dose of Aripiprazole differ between these groups. There was however significant difference in the GAF total score at follow up (p =0.001), as GAF was 59 (SD 9.3) for responders and 39 (SD 5.3) for non-responders.
Mean Ki-values at baseline was 0.79 (SD 0.2) for non-responders, 0.88 (SD 0.2) for responders and 0.91 (SD 0.2) for HC. One way ANOVA showed no significant group difference.
**Discussion:** Although not significant, we found a slightly lower Ki-value at baseline for non-responders compared to baseline Ki-values for responders and HC in these preliminary analyses. This was unexpected, but should be taken with precaution, as the results represent work in progress.
Inclusion of subjects and data-analyses is ongoing, and data analysis will be more extensive in spring 2018, especially regarding the methodology:
The current image derived input function suffers from partial volume effects (PVE). To account for PVE and other factors the image data will be co-registered with T1-weighted MRI data and normalized to standard space in order to use a standard anatomical atlas to help define the ROIs. Finally, as mentioned earlier, arterial samples are collected and we plan to use arterial input functions to correct for the complicated kinetics of the tracer. To improve the Ki estimation we will use the metabolite corrected arterial plasma curve as input function and compare these results with the current method.

### S151. SUBMISSION WITHDRAWN

### S152. CANNABIDIOL INDUCED MODULATION OF MEDIOTEMPORAL ACTIVITY DURING A VERBAL MEMORY TASK IN FIRST-EPISODE PSYCHOSIS

Aisling O'Neill*[,1], Robin Wilson[1], Grace Blest-Hopley[1], Luciano Annibale[1], Marco Colizzi[1], Sagnik Bhattacharyya[1]
[1]*Institute of Psychiatry, Psychology & Neuroscience, King's College London*

**Background:** Global neurocognitive impairments are a central feature of psychosis. Deficits in verbal memory in particular are the most consistently reported of these impairments from the first-episode of psychosis (FEP). Neuroimaging studies in psychosis have largely identified reductions in neural activation during various memory and learning related tasks, particularly in the medial temporal lobe, compared to healthy controls. Tetrahydrocannabinol (THC) and cannabidiol (CBD), both components of the cannabis plant that act through the endocannabinoid (eCB) system in the brain, have been found to induce direct and opposite neural effects during similar tasks in healthy samples, when compared to each other. Additionally, CBD has been shown to have antipsychotic properties, and may suppress THC induced psychotic symptoms and their directly associated functional abnormalities in healthy individuals. Thus far, the effects of CBD on the neural substrates implicated in memory and learning, and those underlying psychotic symptoms in FEP cohorts is unknown.
**Methods:** 17 FEP patients were initially recruited to the study. A double-blind, randomized, placebo controlled, repeated measures, within subject cross over design, with at least a one-week washout period between scans was employed. Participants were given identical capsules of either CBD (600mg), or placebo (PLB), then scanned using a block design fMRI paradigm, while performing a verbal paired associate learning task. 13 participants completed scanning, and were included in the analysis of the data. An ROI mask of the hippocampus, striatum, and parahippocampal gyrus was used in the data analysis, and all results were thresholded for less than one false positive over the whole map.
**Results:** A CBD related decrease in activity was observed in the left hippocampus (p = 0.0024) and the right parahippocampal gyrus (p = 0.0024) during the recall condition, within the FEP group. No significant differences between PLB and CBD functional activity were observed during the encoding condition. No significant differences were observed between FEP participant performances on the CBD and PLB study days.
**Discussion:** These findings provide robust evidence of the modulatory effect of an acute dose of CBD on the neural substrates underlying learning and memory, supporting a role for the eCB system in the abnormalities observed in psychosis, and its potential as a target for treatment.

### S153. WHERE IS THE ABNORMAL BRAIN ACTIVITY IN FIRST EPISODE PSYCHOSIS?

Soldevila-Matias Pau*[,1], González-Vivas Carlos[2], García-Martí Gracián[3], Soprano-Ros Olga[4], Martí-Bonmatí Luis[5], Crespo-Facorro Benedicto[6], Sanjuán Julio[7]
[1]*Research Institute of Clinic University Hospital of Valencia (INCLIVA);* [2]*Clinic University Hospital;* [3]*Center for Networking Biomedical Research in Mental Health (CIBERSAM), Quirónsalud Hospital;* [4]*La Ribera University Hospital;* [5]*Quirónsalud Hospital;* [6]*Center for Networking Biomedical Research in Mental Health (CIBERSAM), University Hospital Marqués de Valdecilla, IFIMAV, University of Cantabria;* [7]*Research Institute of Clinic University Hospital of Valencia (INCLIVA), Center for Networking Biomedical Research in Mental Health (CIBERSAM), Clinic University Hospital, University of Valencia School of Medicine.*

**Background:** Recent review about functional magnetic resonance imaging (fMRI) in first episode psychosis (FEP) concluded that there is an abnormal connectivity involving the frontal temporal pathway similar to found in chronic schizophrenia (Mwansisya et al., 2017). Besides, thalamic circuits were also altered in chronic schizophrenia patients (Li et al., 2017). The present work gives a wider review of studies using functional magnetic resonance imaging techniques (fMRI) on first-episode psychotic patients, specifically focus on the main areas involved.
**Methods:** The review was made in accordance with the PRISMA guidelines (Moher et al., 2009). For each study, the following factors were extracted: anatomical location of the main finding and type of functional abnormality (hypo and hyperactivation). A total of 3 different databases (Pubmed, Web of Knowledge, PsycInfo) were reviewed. Thirty-five of 643 (from 2000 to 31st October 2017) neuroimaging papers were analyzed.
**Results:** We found that the dorsolateral prefrontal cortex (DLPFC) showed 52% of activity abnormalities (55% was hypoactivation). Temporal lobe showed 51% of functional activity altered (61% was hypoactivity). The ventrolateral prefrontal cortex (VLPFC) presented 28% of aberrant fMRI (75% was hyperactivity). Thalamus presented alteration activity with 20% (71% was hypoactivity). The Cingulate was also altered during activation with 20% of patients (85% was hypoactivity). Finally, functional alterations of the Amygdala were present in 14% of the selected patients (80% was hypoactivation).

**Discussion:** This larger review suggests that there are several possible areas apart from fronto temporal pathways (Mwansisya et al., 2017), that have to be taken into account at the early course of psychosis, such as lymbic system, thalamo-cortical networks and cingulate. These functional activation abnormalities seem to be different to the reported in the previous review. The different results seem to be clearly influenced by the kind of paradigm. Moreover, our finding is not in concordance with the suggestion that thalamic alterations became only prominent at the chronic phase of psychosis (Li et al., 2017).

**References:**

1. Moher, D et al., Preferred reporting items for systematic reviews and meta-analyses: The Prisma statement. PLoS Med, 2009, 6, 1000097.
2. Mwansisya, T et al., Task and resting-state fMRi studies in first-episode schizophrenia: A systematic review. Schizophrenia Research article in press, 2017.
3. Li, T et al., Brain-wide analysis of functional connectivity in first episode and chronic stages of schizophrenia: Schizophrenia Bulletin 2016, 43, 436–448.

### S154. THE ROLE OF DOPAMINE IN PROCESSING THE MEANINGFUL INFORMATION OF OBSERVATIONS, AND IMPLICATIONS FOR THE ABERRANT SALIENCE HYPOTHESIS OF SCHIZOPHRENIA

Matthew Nour*[,1], Tarik Dahoun[2], Philipp Schwartenbeck[3], Rick Adams[4], Thomas FitzGerald[5], Christopher Coello[6], Matthew Wall[7], Raymond Dolan[3], Oliver Howes[8]
*[1]King's College London, Institute of Psychiatry; [2]Imperial College London, Institute of Clinical Sciences; [3]University College London, Wellcome Trust Centre for Neuroimaging and Max Planck UCL Centre for Computational Psychiatry and Ageing Research; [4]University College London, Institute of Cognitive Neuroscience and Division of Psychiatry; [5]University of East Anglia; [6]University of Oslo, Institute of Basic Medical Sciences; [7]Imanova Centre for Imaging Sciences, Hammersmith Hospital; [8]King's College London, Institute of Psychiatry, Imperial College London, Institute of Clinical Sciences, MRC London Institute of Medical Sciences*

**Background:** The aberrant salience hypothesis of schizophrenia proposes that symptoms such as paranoia arise when behavioural salience is attributed to neutral stimuli. Mesolimbic dopamine dysfunction is thought to be central to this mechanism; building on findings that activity in this pathway conveys a (signed) reward prediction error signal. Given that many psychotic symptoms are not explicitly related to reward learning, it is relevant that recent studies in rodents have demonstrated a role for midbrain dopamine neurons in value-neutral associative learning. Direct evidence for this role in humans, however, is lacking.

In this study we asked whether the mesolimbic dopamine circuit is involved in encoding the value-neutral meaningful information of observations, using a model-based functional magnetic resonance imaging (fMRI) task and dopamine positron emission tomography (PET). We define 'meaningful information' as the degree to which an observation results in a belief-update to an agent's internal model of the environment (Kullback-Leibler divergence from prior to posterior beliefs; 'Bayesian surprise').

**Methods:** Participants were tasked to infer the current (hidden) state of the environment, using partially-informative observations at each trial, and then report their belief at the end of each trial. Participant beliefs were modelled using a Hidden Markov Model of the task and iterative application of Bayes' rule, allowing us to quantify the Bayesian surprise (meaningful information content) associated with a trial observation. Crucially, our task de-correlated Bayesian surprise from both the pure sensory unexpectedness of an observation (unexpected but meaningless information) and its signed reward prediction error. 39 healthy participants (22M, mean age 26y) performed 180 task trials within an fMRI scanner. 36 participants also had a [11C]-(+)-4-propyl-9-hydroxy-naphthoxazine (PHNO) PET scan to quantify dopamine-2/3 receptor (D2/3R) availability. 17 participants additionally had a second PET scan 3hrs post 0.5mg/kg oral dexamphetamine, to quantify striatal dopamine release capacity. Neuroimaging analyses were restricted to the bilateral substantia nigra/ventral tegmental area (SN/VTA) and ventral striatum (VS).

**Results:** Our computational model closely predicted participant behaviour ($R2= .67$), and there was a negative correlation between subclinical paranoia and the degree to which participant behaviour approximated normative Bayesian performance (rho = -.60, $P<0.001$). Neuronal activation encoding the meaningful information content of an observation (Bayesian surprise) was present in SN/VTA and VS (both P(peak)<0.05, SVC), whereas no such encoding was present for sensory unexpectedness or reward-prediction error. Crucially, activation encoding Bayesian surprise was inversely correlated with D2/3R availability in the SN/VTA (rho = -.43, P=0.009), consistent with a tonic inhibitory role for midbrain D2/3Rs. Moreover, activation encoding Bayesian surprise was inversely related to dopamine release capacity in the VS (rho = -.66, P=0.005), indicating that subjects with high dopamine release capacity showed blunted striatal activation in response to belief-changing information, as is also found in schizophrenia.

**Discussion:** We provide direct evidence in humans that a mesolimbic dopamine circuit is involved in encoding the meaningful information content of observations, distinct from its involvement in processing signed reward prediction error. These results implicate dopamine in a wider range of function than reward learning, including updating a predictive associative model of the world, and are therefore relevant for the aberrant salience hypothesis of schizophrenia.

### S155. SENSORY ATTENUATION DURING AUDITORY PROCESSING IN PARTICIPANTS AT CLINICAL-HIGH RISK FOR PSYCHOSIS: EVIDENCE FROM MAGNETOENCEPHALOGRAPHY

Lingling Hua*[,1], Marc Recasens[2], Tineke Grent-'t-Jong[3], Emmi Mikanmaa[2], Hanna Thuné[2], Andrew Gumley[2], Matthis Schwannauer[4], Stephen Lawire[4], Ruchika Gajwan Gajwan[2], Joachim Gross[2], Peter Uhlhaas[2]
*[1]School of Psychology; [2]University of Glasgow; [3]University of Glasgow, Institute of Neuroscience and Psychology, Centre for Cognitive NeuroImaging, School of Psychology; [4]University of Edinburgh*

**Background:** The ability to predict the sensory feedback of self-generated stimuli against incoming sensory information is of importance to distinguish internal from external stimuli and is associated with sensory attenuation. Furthermore, it has been proposed that deficits in sensory attenuation could contribute to clinical symptoms of schizophrenia, including hallucinations and delusions, involving potential deficits in corollary discharge. The current study examined the hypothesis whether sensory attenuation is present in participants at clinical high–risk (CHR) for psychosis.

**Methods:** Sixty-four CHR-participants and 32 healthy controls were presented with auditory stimuli during two experimental conditions: 1) In a passive condition, participants were presented with ripple sounds (40HZ with 2000ms duration, 83db) and responded to flat sounds (1000HZ with 2000ms duration,83db) with the right-index finger and 2) In an active condition, the ripple sounds were elicited by a button press with the right-index finger every 4s. MEG-data were acquired with a 248-magnetometers whole-head MEG system (MAGNES 3600 WH, 4-D Neuroimaging) at a sampling rate of 1017Hz. We focussed on the M100 response during the passive and active conditions at sensor- and source-level. A LCMV beamforming approach was employed for source reconstruction and virtual channels in primary auditory cortex and the left superior temporal cortex were used further analysis of sensory attenuation effects. Condition and group effects were tested with a cluster-based nonparametric test implemented in Fieldtrip with a window of interest for the M100 component between 120ms-150ms.

**Results:** There was a significant decrease (P =0.009) in the amplitude of M100 component in the active vs. passive conditions across groups at both sensor- and source-level. Interaction-effects revealed that that sensory attenuation was significantly reduced in auditory cortices in the CHR group vs controls (P=0.032).

**Discussion:** The current results highlight that sensory attenuation can be studied with ASSR-paradigms and that both primary auditory and superior temporal cortices underlie this effect. Moreover, our current findings suggest that sensory attenuation is impaired in CHR-participants, suggesting the possibility of impaired corollary discharge processes as a potential biomarker for the early diagnosis and detection of schizophrenia.

### S156. FRONTO-STRIATAL FUNCTIONAL CONNECTIVITY AND STRIATAL DOPAMINE CAPACITY IN TREATMENT-RESPONSIVE AND REFRACTORY SCHIZOPHRENIA

Seoyoung Kim*[,1], Wi Hoon Jung[2], Euitae Kim[1], Jun Soo Kwon[3]
*[1]Seoul National University Bundang Hospital; [2]Korea University; [3]Seoul National University College of Medicine*

**Background:** Schizophrenia is thought to be a heterogeneous disorder and evidences reflect categorically distinct subtypes according to the antipsychotic treatment response. Altered frontostriatal functional connectivity (FC) in schizophrenia and its correlation with antipsychotic treatment response also suggests divergence of underlying pathophysiologic mechanism. Meanwhile, the observations that prefrontal activity correlates with striatal dopaminergic function, leads to the hypothesis that the disrupted frontostriatal FC would be related with altered dopaminergic pathway in schizophrenia. The aim of this study was to investigate the relationship between frontostriatal FC and striatal dopaminergic activity in patients with schizophrenia according to the responsiveness to first-line antipsychotic drug.

**Methods:** 24 symptomatically stable schizophrenia patients were recruited from Seoul National University Hospital, 12 of which responded to first-line antipsychotic drugs (first-line AP group) and 12 stable under clozapine (clozapine group), along with 12 matched health controls. All participants underwent resting-state functional MRI and [F18]DOPA positron emission tomography.

**Results:** There were no significant difference in the total PANSS score between the first-line AP group and the clozapine group (mean difference=0.67, s.e.=3.21, df=33, p=1.000). Voxel-based analysis found significant negative correlation between frontal FC to the left associative striatum and the kicer in the corresponding region was found in first-line AP group but not in clozapine group or healthy control. Additional region of interest analysis confirmed the result (control group: R2=0.032, p=0.572; first-line AP group: R2=0.551, p=0.005; clozapine group: R2=0.108, p=0.297).

**Discussion:** Different patterns of relationship between striatal dopamine capacity and frontostriatal FC observed in this study indicates different pathophysiology underlying schizophrenia according to antipsychotics treatment-responsiveness. Results should be reconfirmed in prospective manner with larger sample size in future studies.

### S157. NEURAL CORRELATES OF SMOOTH PURSUIT EYE MOVEMENTS IN POSITIVE AND NEGATIVE SCHIZOTYPY

Eliana Faiola*[,1], Maria F. Urquijo[2], Inga Meyhöfer[1], Anna Kasparbauer[1], Maria Steffens[1], Michael Wagner[1], Nikolaos Koutsouleris[2], Ulrich Ettinger[1]
*[1]University of Bonn; [2]Ludwig Maximilian University Munich*

**Background:** Both schizophrenia patients and highly schizotypal individuals are known to perform worse in smooth pursuit eye movements (SPEM) as compared to healthy controls with low levels of schizotypy. However, little is known about the neural correlates of SPEM deficits in subjects with high levels of schizotypy. In a previous study, individuals with high total schizotypy levels showed reduced activation in motion processing and other visual areas during SPEM than low schizotypal controls. Interestingly, reduced activation in these areas is also observed in schizophrenia patients. This suggests that schizotypy and schizophrenia overlap not only at the behavioral, but also at the neural level. In the present study, we followed up on these intriguing results by differentiating between negative and positive schizotypal groups. We expected to find lower SPEM performance in both highly negative and highly positive schizotypes (HNS and HPS) as compared to low schizotypal controls (LS). Moreover, in the schizotypy groups, activation in the SPEM network was expected to be reduced in a similar way as previously reported for schizophrenia patients and highly schizotypal individuals.

**Methods:** In this ongoing, bi-center study, 88 healthy subjects (28 HNS, 23 HPS, 37 LS) underwent functional magnetic resonance imaging (fMRI) at 3T during a smooth pursuit task with concurrent oculographic measurement. Sinusoidal targets with frequencies of 0.2 Hz and 0.4 Hz were presented in a block design, with pursuit blocks alternating with blocks of fixation.

**Results:** At the level of performance, we found an interaction between target frequency and group for the root mean square error (RMSE) of eye position (p = .026). This result indicates greater performance detriments from low to high frequency in HPS, as compared to the other two groups. At the neural level, overall activations during pursuit across the entire sample were found in brain regions known to be part of the pursuit network, i.e. frontal and supplementary eye fields, lateral geniculate nucleus, and visual cortex including V5. However, none of these regions displayed activation differences between groups. With multiple regression analyses for each of the groups, we investigated associations between cluster peak voxel activations and performance. For the low target frequency, a negative association was found between visual area V5 in the left hemisphere and the total saccade frequency for HPS ($\beta = -.462$, p = .03). For the high target frequency, activation in left V5 was negatively associated with RMSE in the LS group ($\beta = -.411$, p = .01).

**Discussion:** We replicated previous findings of reduced SPEM performance in highly schizotypal individuals. In addition, a negative association between activation in area V5 and RMSE was only found among LS, but not for the two schizotypy groups. This is in line with previous findings of SPEM impairments in schizophrenia patients being associated to alterations in motion sensitive area V5 and underlines the importance of motion processing for SPEM deficits in the schizophrenia spectrum. However, we also found an association between activation in V5 and total saccade rate during pursuit in HPS, suggesting that V5 abnormalities may be restricted to negative schizotypy. Given the relatively small number of participants in this analysis, we expect to find broader and clearer group differences of neural mechanisms during pursuit with larger sample sizes.

### S158. REWARD ALTERATIONS IN ANTIPSYCHOTIC NAÏVE FIRST-EPISODE-PSYCHOSIS PATIENTS BEFORE AND AFTER TREATMENT WITH A PARTIAL DOPAMINE AGONIST

Karen Tangmose*[,1], Mette Odegård Nielsen[2], Anne Sigvard[2], Kasper Jessen[2], Kirsten Bojesen[2], Marie Bjerregaard[2], Egill Rostrup[2], Birte Glenthoj[2]
*[1]Center for Neuropsychiatric Schizophrenia Research; [2]Center for Neuropsychiatric Schizophrenia Research, CNSR, and Center for Clinical Intervention and Neuropsychiatric Schizophrenia Research, CINS, Mental Health Center Glostrup, University of Copenhagen*

**Background:** Alterations of the brain reward system is a common finding in patients with psychoses and it may be affected by antipsychotic medication. There are however only few longitudinal studies on medication effect and the effect of a partial dopamine agonist have not previously been examined in patients. The aim of the present study is to explore reward abnormalities in first episode psychotic patients and matched heathy controls (HC) before and after treatment with a partial dopamine agonist (aripiprazole), and relate the findings to dopamine synthesis capacity (F-DOPA-PET), glutamate and GABA levels in the brain (MRS at 3T) and treatment outcome. Here we present preliminary baseline and follow up analyses on functional magnetic resonance imaging (fMRI) only.
**Methods:** The project is a part of a multimodal prospective cohort study. Reward related brain activity was examined with fMRI using a variant of the Monetary Incentive Delay Task before and after 6 weeks, where patients were treated with individual doses of aripiprazole. Psychopathology was measured with the Positive and Negative Syndrome Scale (PANSS). Whole brain voxel-wise group comparison was performed at baseline and follow up using two sample t-test with a corrected cluster significant threshold of P=0.05. Likewise, the effect of time and group time interaction was analyzed voxel-wise.
**Results:** Inclusion is ongoing and data have been analyzed for 19 patients, age 22.9(4.6), 9 males (47%) and 24 HC, age 22.1(2.7), 11 males (46%). Mean medication dose was 11.7 (6) mg aripiprazole at follow up.
Psychopathology: At baseline patients were moderately ill with a mean PANSS total score of 69 (14). Paired t-test showed a significant reduction over time for PANSS total score to 57 (12) ($P<0.001$), with significant improvements in PANSS positive, PANSS negative and PANSS general scores (all $p<0.05$).
fMRI: There were no group differences at baseline.
At follow up, patients had an increased signal in medial frontal cortex and Anterior Cingulate Cortex (ACC) compared to HC during anticipation of monetary gain. During outcome evaluation, patients likewise had an increased signal in right striatum and paracingulate gyrus in the win contrast, increased signal in left ventral part of striatum and ACC in the lose contrast, and increased signal in right striatum and ACC in the miss contrast compared to HC. There was only a significant effect of time in patients in the anticipation to win contrast and no significant group time interaction.
**Discussion:** The data represent work in progress and should be taken with precaution. The group-differences at follow up which were not found at baseline may suggest that treatment with a partial dopamine agonist lead to alterations of reward processing in patients. This is further supported by the effect of time in patients in the anticipation to win contrast. The data collection is still ongoing, and we expect to increase the size of the cohort and plan to relate the findings to measures of dopamine, GABA, glutamate and psychopathology.

### S159. REDUCED PROCESSING SPEED IN SCHIZOPHRENIA IS MEDIATED BY WHITE MATTER INTEGRITY

Saetbyeol Cha*[,1], Woon Yoon[1], Seung-Hyun Shon[1], Jungsun Lee[1]
*[1]Asan Medical Center*

**Background:** Meta-analysis suggest that processing speed deficit is the largest single cognitive impairment in schizophrenia. Processing speed predicts functional outcome and indicates a vulnerability marker for schizophrenia. Several authors have proposed that abnormalities in white matter is related to reduced processing speed in schizophrenia. The purpose of this research was to investigate the relationship between processing speed and structural properties of white matter pathways in schizophrenia and healthy controls.
**Methods:** The data using this study were from the SchizConnect. Participants included 64 patients with schizophrenia and 71 healthy controls. Diffusion tensor imaging(DTI) method was used to measure fractional anisotropy along white matter tracts. Group differences in white matter integrity-inferred from fractional anisotropy (FA), processing speed, verbal memory were examined. Mediation analysis were applied to inspect the relationship between FA and cognitive performance.
**Results:** Participants with schizophrenia had significantly reduced processing speed, verbal memory deficits, and whole-brain fractional anisotropy deficit. There were significant group differences in white matter integrity of the left thalamus occipital, right extreme capsule, and right thalamus occipital. FA in left thalamus occipital and right extreme capsule mediated group differences in processing speed, but not other cognitive domains.
**Discussion:** Study findings indicate that mediation effect of processing speed is regional tract-specific. These finding suggest that the structural integrity of white matter tracts associated with left thalamus occipital, right extreme capsule is closely related to reduced processing speed in schizophrenia, but not verbal memory and verbal learning.

### S160. INTERACTIONS BETWEEN BOTTOM-UP AND TOP-DOWN ATTENTION DURING WORKING MEMORY ENCODING: EVALUATION OF AN FMRI PARADIGM FOR THE STUDY OF COGNITIVE DYSFUNCTION IN SCHIZOPHRENIA

Mishal Qubad*[,1], Catherine Barnes[2], Lara Rösler[3], Michael Schaum[2], Benjamin Peters[2], Michael Wibral[2], Andreas Reif[2], Robert Bittner[2]
*[1]University Hospital Frankfurt, Goethe University; [2]Goethe University Hospital Frankfurt; [3]University of Würzburg Germany*

**Background:** Patients with schizophrenia suffer from profound impairments of working memory and selective attention. These cognitive domains show a considerable overlap on both the behavioral and neurophysiological level. Importantly, selective attention appears to be crucial for the selection of information to be encoded into working memory. A number of studies have demonstrated that the efficiency of this “gatekeeper” function influences working memory performance. Furthermore, behavioural evidence indicates, that patients with schizophrenia have a specific deficit when required to suppress irrelevant but highly salient visual information during working memory encoding. Therefore, elucidating the neurophysiological mechanisms underlying the “gatekeeper” function of selective attention for working memory is highly relevant for understanding this deficit in schizophrenia. The aim of the current study was to investigate the neurophysiological correlates of encoding either salient or non-salient information in the presence of distractors of opposite saliency using functional magnetic resonance imaging (fMRI). Furthermore, we wanted to study the impact of additional top-down information guiding the selection of task relevant information.
**Methods:** 35 healthy volunteers underwent fMRI in a 3 T Siemens Trio scanner. During a change detection task four Gabor patches (two flickering and two non-flickering) with varying orientations were shown and participants had to memorise the orientations of the Gabor patches. A colored fixation cross was displayed before the stimuli either cueing two (predictive cue) or four (non-predictive cue) Gabor patch locations resulting in a 2 x 2 design of four conditions with the factors salience (flickering vs. non-flickering)

and cue (predictive cue vs. non-predictive cue). During retrieval a single Gabor patch was displayed, and participants reported if the orientation was the same or had changed in that location. At the beginning of each block participants were instructed to either encode the flickering or non-flickering patches (targets) whose location could either be cued or uncued. In 80 % of trials, a target was probed during retrieval. Data analysis in Brain Voyager included standard data preprocessing. Additionally, a multiscale curvature driven cortex based alignment procedure was used to minimise macro-anatomical variability between subjects. Subsequently, functional data were analysed using a random-effects multi-subject general linear model ($p<0.05$, FDR corrected). Functional connectivity analysis was performed using Granger Causality Mapping.

**Results:** Participants were able to preferentially encode task-relevant information in all four conditions. During encoding, they showed activation in a distributed network of fronto-parietal and visual areas. For salient compared to non-salient distractors, we observed increased functional connectivity between attention-related areas and extrastriate visual cortex. This difference was more pronounced for trials with a predictive compared to non-predictive cue.

**Discussion:** We were able to map the cerebral networks responsible for determining the contents of working memory. The observed patterns of connectivity indicate that core regions of the frontal-parietal network involved in both working memory and selective attention play a crucial role in the filtering of information by modulating the processing of information in visual areas. Our current findings provide the basis for studying the neurophysiological underpinnings of the interaction between impairments of working memory and selective attention in schizophrenia.

### S161. FUNCTIONAL BRAIN NETWORKS INVOLVED IN ATTENTIONAL BIASING IN SCHIZOPHRENIA

Paul Metzak*,[1], Todd Woodward[2]
*[1]University of Calgary; [2]University of British Columbia*

**Background:** Although the symptomatology in schizophrenia is variable, many of the cognitive deficits that are associated with the illness, including impairments in attention, working memory, verbal learning and executive functions, persist over time from the prodrome to the chronic phase. One of the cognitive domains showing pronounced deficits is executive function, which is the ability to adaptively adjust behavior in the face of changing environmental demands. Attentional biasing is one aspect of executive function that attentuates conflict between competing stimuli (or competing features of a stimulus) via the top-down regulation of attention. The goal of this study was to use functional magnetic resonance imaging (fMRI) to isolate the brain activity related to differences in levels of attentional biasing in schizophrenia patients, where these levels were varied from trial-to-trial by manipulating the number of relevant stimulus dimensions.

**Methods:** Participants - Twenty-three schizophrenia patients and twenty-one healthy volunteers, matched on age and gender, were recruited from the Vancouver area.

Task – The task involved performing three discrete tasks in alternation: judging whether shapes are blue or red, judging whether numbers are odd or even, and judging whether letters are uppercase or lowercase. Each stimulus contained either one dimension that cued a task in the task set (e.g. the numeral '2' in white ink), two dimensions (e.g. the numeral '2' in blue ink), or three dimensions, such that all three tasks in the set are cued (e.g. the word 'TWO' written in blue ink). Each stimulus was presented in the center of the screen and the judgment to be performed was cued with a single word followed by a question mark.

**Results:** The fMRI data was analyzed using Constrained Principal Component Analysis, which identifies brain networks common to all participants and indexes the activity of each network for each participant. Three components were extracted for further examination.

Component 1 displayed activations located in the visual cortices, parietal lobes, primary motor areas, supplementary motor area (SMA), dorsal anterior cingulate cortex (dAcc), and cerebellum. The statistical analysis indicated that this component was reliable but did not differentiate between patients and volunteers.

Component 2 displayed activations in the occipital lobes, dAcc, SMA, parietal lobes and primary motor areas, and deactivations in the medial prefrontal cortices and the posterior cingulate/precuneus. The statistical analysis indicated that the activity in this component was reliable, and became stronger as stimulus dimensions increased. However, the patients did not increase activity to the same degree as the volunteers in the most challenging condition.

Component 3 displayed activations in the occipital lobes, hippocampi, and left parietal and primary motor areas as well as deactivations in superior and middle frontal gyri. The statistical analysis indicated that this component was reliable, but activity levels did not differentiate between patients and volunteers.

**Discussion:** The results indicate that patients and volunteers activated the same networks while performing the attentional biasing task. However, the statistical analysis of Component 2 suggests that patients display an inefficient pattern of brain activity, such that they have higher levels of activity than volunteers when little attentional biasing is required and significantly lower levels of activity than volunteers when high levels of attentional biasing was required. This pattern of results is suggestive of inefficient neural activity, particularly at higher levels of task difficulty, a finding which has previously been described in the schizophrenia literature.

### S162. IMPACT OF THE PRESENCE OF A PEER WORKER IN AN EARLY INTERVENTION UNIT FOR YOUNG ADULTS WITH MENTAL ILLNESS (JADE)

Maryse Badan Ba*,[1], Elisabeth Sturm[2], Laurent Bediat[1], Logos Curtis[1]
*[1]University Hospital of Geneva; [2]Re-pairs Association*

**Background:** A current trend in health care and in particular mental health care is to reduce the divide between patients and their community, which is encouraging new practices as well as new health care professions. The concept of a peer worker, a previous mental health care user, is revealing itself to be complementary to that of other health care workers as well as effective (Davidson et al., 2012). One aspect of the peer worker given his or her previous experience is as an intermediary for communication. In mental health care units such as ours (Geneva based JADE program for early intervention in mental health) the introduction of a peer worker as a new concept can lead to many benefits but also carries questions and uncertainties.

**Methods:** In order to assess the impact of a peer worker's presence in our unit over a period of 2 months, we submitted questionnaires to patients and staff. We present results from questionnaires from 7 patients and 15 staff. In order to further explore the subjective appreciation of this integration, we included open ended questions to also assess constructive suggestions from patients and staff.

**Results:** Data collection is in progress.

**Discussion:** The impact of the presence of peer-worker in our mental health care unit will be discussed.

### S163. FEASIBILITY STUDY: MEASURES OF SLEEP AND PHYSICAL ACTIVITY IN PEOPLE WITH SCHIZOPHRENIA

Alexandra Berry*,[1], Richard Drake[1], Roger Webb[1], Darren Ashcroft[1], Matthew Carr[1], Alison Yung[1]
*[1]University of Manchester*

**Background:** People with schizophrenia and related psychotic illnesses have poor physical health and are at an increased risk of developing long-term physical health conditions such as diabetes and heart disease. While this may be due to unhealthy lifestyles, such as lack of physical activity, circadian rhythm problems may also play a part. It is therefore important to be able to measure physical activity and sleep patterns in schizophrenia. This study aims to assess for feasibility by comparing ActiGraph accelerometer data, mobile phone app data and questionnaire data.
**Methods:** A cross-sectional comparison of different assessment methods of sleep and general activity was used. Assessment methods included:

a) ActiGraph wGT3X-BT accelerometers worn on the waist and wrist for 7 days.
b) Lenovo A Plus smartphone apps 'SleepBot' and 'Google Fit' installed for the purposes of gathering data on sleep and physical activity patterns for 7 days.
c) Simple Physical Activity Questionnaire taken at baseline and on day 7.

At the seven-day assessment participants were interviewed using a topic guide covering their experiences. This explored the feasibility and acceptability of the measures and possible barriers for implementation.
**Results:** 14 out of a planned 30 participants who met DSM IV-R criteria for schizophrenia spectrum psychoses have been recruited across Greater Manchester from wards and in the community. All participants were retained for the 7-day study duration. Preliminary assessment has shown concordance between the different measures. 3 out of the 14 participants engaged in vigorous physical activity during the 7 days. All 14 participants spent more than 50% of their time sedentary during the 7 days. Participants showed fragmented sleep with a high number of awakenings.
**Discussion:** Using mobile phones and accelerometers are inexpensive and unobtrusive methods for measuring sleep and physical activity. These measures are feasible and acceptable to people with schizophrenia and are therefore suitable for implementation in routine clinical care. The measures can also be used by service users themselves to enhance their ability to monitor their own physical health. Such self-management and monitoring may encourage goal setting and improve autonomy, which have been found to be associated with increased levels of physical activity.

### S164. "AT-RISK MENTAL STATES" PROGRAM IN LAUSANNE: INFLUENCE OF RECRUITMENT STRATEGIES ON THE RATE OF FALSE POSITIVES

Alessandra Solida*,[1], Martine Cleusix[1], Carline Zorzi[1], Carina Ferrari[1], Kim Q. Do[1], Philippe Conus[1]
[1]*Lausanne University Hospital (CHUV)- CH*

**Background:** Various strategies have been proposed to improve recruitment of "at risk mental state" patients; they may have an impact on the type of patients who reach such programs. We describe the clinical program for "at-risk" patients implemented in 2014 in Lausanne and the characteristics of referrals over the years.
**Methods:** Help seeking patients aged 14 to 35 were initially referred by health care providers for a specialized evaluation in case of suspicion of a potential "prodromal psychotic state" and more recently selected by PQ-16 (Ising et al. 2012) (cut-off: 6/16).
At-Risk Mental State (ARMS) was defined according to the Basic Symptoms criterion (COPER-COGDIS criteria) from the Schizophrenia Proneness Instrument – Adult version (SPI-A) and to the Clinical High Risk criteria of the Structured Interview for Prodromal Syndromes (SIPS). ARMS patients underwent an extensive clinical evaluation (including Mini-SCID, SOFAS, MARDS, Yung Mania Scale, etc.) and were followed-up every 6 months over 3 years.
**Results:** Within a catchment area of 260 000 inhabitants, 110 patients have been referred to our center since 2014 and 100 completed the investigation. 29 (29%) fulfilled ARMS criteria, 52 (52%) didn't and 19 (19%) were already psychotic.
The proportion of true ARMS patients decreased progressively over the years from 45% in 2014 and 2015, to only 22 and 13.9 % in 2016 and 2017.
In our sample of help-seekers, the group of patients ARMS- negative received mostly a schizophrenia spectrum diagnosis (26/52 patients, 50%), associated with low psychosocial functioning, even when not in the precise range of at-risk criteria.
**Discussion:** The global prevalence (29%) of ARMS patients in our sample over the 4 years is marginally lower than previous reports on similar tertiary centers, which ranges from 33 to 51 % (Kline E., 2014). Our lower prevalence of ARMS patients within the sample may be linked to the limited resources we had to conduct an information strategy and our focus on psychologists and psychiatrists working at our department. The introduction in 2016 of more intense screening strategy based on the use of the PQ-16 lead to an increase in referral numbers but decreased the rate of ARMS among referred patients.
Our results confirm the influence of the recruitment strategy and information campaigns on the prevalence of at-risk patients within a population of help-seekers. The prevalence of schizophrenia spectrum diagnosis in our group of patients ARMS-negative also suggests that a larger "vulnerability" model for psychosis, more sensitive to functioning and negative symptoms and not narrowed on the focus of the risk of imminent acute psychosis, may better fit patients' needs.

### S165. ALTERED ASSOCIATION BETWEEN PARIETAL GRAY-MATTER VOLUME AND DISSOCIATIVE SYMPTOMS IN SCHIZOPHRENIA: A VOXEL-BASED MORPHOMETRY STUDY

Huai-Hsuan Tseng*,[1], Chui-de Chiu[2], Kao Chin Chen[1], I Hui Lee[1], Po See Chen[1], Yen Kuang Yang[1]
[1]*National Cheng Kung University;* [2]*The Chinese University of Hong Kong*

**Background:** The presence of dissociative symptoms has been constantly reported in patients with schizophrenia. Dissociative-like experience is also part of the prodromal symptoms in those who have higher risk for psychosis. While the underlying neurobiological causes of dissociative symptoms in patients with schizophrenia remains unclear, a history of trauma seems to be related to their dissociative symptoms, as is seen in dissociative disorders. The traumatic experience has been linked to volumetric alterations in patients with schizophrenia. The current study aimed to explore the associations between past traumatic experience, brain volume alteration and the presence of dissociative symptoms in patient with schizophrenia.
**Methods:** We employed voxel-based morphometry (VBM) to compare the distributions of gray matter volumes (GMV) in 20 patients with schizophrenia (SCZ, 10 Male) and 26 age- and sex-matched healthy volunteers (HV, 11 male). All participants underwent high resolution T1-weighted anatomical images on a 3T MRI system. Past traumatic experience was examined by Brief Betrayal Trauma Survey (BBTS), and the dissociative symptoms were measured by Traumatic Dissociation Scale (TDS).
**Results:** We found a significant GMV reduction in right thalamus area in SCZ relative to HV group (p=0.01, whole-brain FWE corrected). The GMV in thalamus was negatively associated with high-betrayal traumatic experience in SCZ group (r=-0.48, p=0.033), but not in HV (r=-0.08, p=0.71). While examining the association between GMV and dissociative experience, a significant group by dissociation interaction was observed in the left superior parietal lobule/angular gyrus (SPL/AG) was observed (p=0.024, whole-brain cluster corrected), where negative correlations was observed in HV (r=-0.62, p=0.001) but positive correlations were observed in SCZ group (r=0.67, p=0.001). In SCZ group, both traumatic experience and the left SPL/AG GMV significantly predicted the dissociative experience (p=0.001 and p=0.011, respectively; R2

increased from 0.56 to 0.70 by adding the left SPL/AG GMV into the prediction model).

**Discussion:** In line with previous literature, we observed a decreased thalamic GMV in SCZ group, which is uniquely associated with their high-betrayal traumatic experience. Superior parietal lobe and angular gyrus has been reported to involved in dissociative experiences in psychiatric illnesses, and modulates sensory, cognitive and self processing in schizophrenia. The positive association we observed between SPL/AG GMV and dissociation experience in SCZ suggests its crucial role in dissociative psychopathology observed in schizophrenia spectrum disorder. Future studies focusing on functional and connectivity alterations of superior parietal/angular area in SCZ and its contribution to dissociative symptoms is warranted.

### S166. SUBMISSION WITHDRAWN.

### S167. ANATOMICAL CONNECTIVITY OF THE VISUOSPATIAL ATTENTIONAL NETWORK IN SCHIZOPHRENIA: A DTI-BASED TRACTOGRAPHY STUDY

Leroux Elise*[,1], Poirel Nicolas[2], Sonia Dollfus[3]
*[1]Service de Psychiatry Chu de Caen; [2]Université Paris Descartes, Normandie Univ, UNICAEN, Institut Universitaire de France (IUF); [3]Centre Hospitalier Universitaire de Caen, Centre Esquirol*

**Background:** In healthy controls (HCs), the visuospatial attentional network consists of fronto-parietal bundles distributed across both hemispheres, but the anatomical organization of this network remains largely unknown in patients with schizophrenia (SZPs). Using diffusion tensor imaging (DTI)-based tractography, we investigated both white matter integrity and the volume of visuospatial attentional pathways in the right and left hemispheres (RH and LH), as well as their structural asymmetry in SZPs and HCs and hypothesized that SZPs would have WM pathway alterations and abnormal structural asymmetry.

**Methods:** This study included 34 SZPs and 69 HCs. Integrity parameters (fractional anisotropy [FA], radial and mean diffusivity [RD and MD]) and volume were calculated in each fasciculus of this network: the three branches of the superior longitudinal fasciculus (SLFI–III), the inferior longitudinal fasciculus, and the inferior fronto-occipital fasciculus in the RH and LH.

**Results:** In SLFII and SLFIII, compared to RH and LH values for SZPs, HCs presented increased FA and/or decreased RD/MD in RH and/or LH. Both SZPs and HCs presented increased FA and decreased RD/MD in the RH compared to the LH in the SLFIII, whereas only HCs had this pattern in the SLFII. Volumes did not differ between groups.

**Discussion:** To our knowledge, this study is the first to describe the structural hemispheric lateralization/organization of the visuospatial attentional network in SZPs. Our main findings are disrupted structural connectivity in the SLFII associated with abnormal anatomical asymmetry in patients, which could be a substrate of attentional deficits.

### S168. AUDITORY TRANSCALLOSAL FIBERS AND AUDITORY HALLUCINATIONS

Dean Salisbury*[,1], Yiming Wang[2], Brian Coffman[2]
*[1]University of Pittsburgh School of Medicine; [2]University of Pittsburgh*

**Background:** Auditory verbal hallucinations (AVH) are one of the most common symptoms in schizophrenia. Connectivity between left and right auditory cortex may be related to AVH. The aim of this study was to examine transcallosal auditory cortex connectivity in first-episode schizophrenia patients (FESz) who experience AVH.

**Methods:** Diffusion spectrum imaging (DSI) data were obtained from 29 FESz and 23 healthy controls (HCs). Of the 29 FESz participants, 15 were AVH-, with a score of 0 for auditory hallucinations, voices commenting, and voices conversing measured with the Scale for the Assessment of Positive Symptoms (SAPS), and 14 were AVH+, with a score of 2 or greater on at least one of these questions. The three groups (AVH+, AVH-, and healthy controls) were matched for age, parental socioeconomic status, years of education, IQ, gender, and handedness. A deterministic fiber tracking algorithm was used to identify the transcallosal auditory white matter tract, which was identified as the 1000 fibers passing through the posterior third of the corpus callosum and ending bilaterally in Brodmann's area 22, Heschl's gyrus, or planum temporale. Transcallosal auditory cortex connectivity was compared between groups for tract volume, generalized Fractional Anisotropy (gFA), and isotropy.

**Results:** MANOVA revealed a significant difference in connectivity between groups ($F(6, 94) = 2.34$, $p = .038$) that was driven by group differences in tract volume ($F(2, 49) = 3.46$, $p = .039$) and gFA ($F(2, 49) = 4.77$, $p = .013$)). Within FESz, AVH severity significantly correlated with auditory cortex transcallosal gFA ($r = -.44$, $p = .013$). Pairwise t-tests indicated lower gFA and greater tract volume for AVH+ vs AVH- (p's < .05). HCs had a trend towards greater gFA ($p = .068$) vs AVH+ and tract volume ($p = .063$) vs AVH-. All other comparisons were nonsignificant ($p > .1$).

**Discussion:** These findings suggest that structural connectivity differences may underlie AVH in schizophrenia, even early in disease course. FESz participants with AVH have less efficient transcallosal auditory connectivity compared to those without AVH. The reduced gFA in FESz correlated with hallucination severity, suggesting that inefficient coordination of left and right hemisphere auditory processing, crucial for language, was impaired in the disorder. The transcallosal structural integrity and connectivity may indicate a subtype characterized by AVH. Current work is determining the extent to which this fiber deficit is common across Kraepelinian diagnostic categories of psychosis (e.g., bipolar disorder and depression with psychotic features).

### S169. MICROGLIAL ACTIVATION AND MORPHOLOGICAL BRAIN ALTERATIONS IN PSYCHOSIS AND PSYCHOSIS RISK

Sina Hafizi*[,1], Elisa Guma[2], Alex Koppel[3], Tania Da Silva[1], Michael Kiang[1], Sylvain Houle[3], Alan Wilson[3], Pablo Rusjan[3], Mallar Chakravarty[4], Romina Mizrahi[3]
*[1]Centre for Addiction and Mental Health, University of Toronto; [2]Douglas Mental Health University Institute; [3]CAMH; [4]McGill University, Douglas Mental Health University Institute*

**Background:** Abnormal brain structural alterations and microglial activation are implicated in the pathophysiology of psychosis. However, the connection between these two pathologies is not yet well understood. Although previous studies suggested a link between the level of proinflammatory cytokines and abnormalities in brain structure in patients with schizophrenia, there is no in-vivo study investigating whether microglial activation is also linked to morphological brain alterations previously reported in individuals with psychosis and psychosis risk.

**Methods:** In order to address the current gap in the literature, we investigated microglial activation and structural brain abnormalities in key brain regions affected in psychosis (i.e. hippocampus and dorsolateral prefrontal cortex) of a large group of participants (N = 90) including 35 individuals at clinical high risk (CHR) for psychosis, 27 first-episode psychosis (mostly antipsychotic naïve) patients, and 28 healthy volunteers. All the participants underwent a [18F]FEPPA positron emission tomography (PET)

targeting mitochondrial 18 kDa translocator protein (TSPO) to determine microglial activation and a T1 MRI scan to study structural brain characteristics including brain volume, cortical thickness, and hippocampal shape.
**Results:** Using a vertex-wise analysis, we observed a significant microglial activation-by-diagnostic group interaction in morphological measures across the left hippocampus. We observed associations between microglial activation and outward and inward morphological alterations in the dorsal and ventro-medial portions of the left hippocampus, respectively. These associations were only observed in first-episode psychosis group. There was no association between [18F]FEPPA binding and other structural brain characteristics.
**Discussion:** Our results, for the first time, suggest a connection between microglial activation and morphological alterations in hippocampus of first-episode psychosis.

### S170. AMYGDALA SUBNUCLEI VOLUMES IN FIRST-EPISODE PSYCHOSIS: ASSOCIATION WITH CHILDHOOD ADVERSITY

Reetta-Liina Armio*,[1], Heikki Laurikainen[1], Raimo Salokangas[1], Lauri Tuominen[2], Jarmo Hietala[1]
*[1]University of Turku; [2]Harvard University, University of Turku*

**Background:** The amygdala volume is reduced already in the first episode of psychosis. The amygdala is a key region in emotional processing, and its volume reduction has been associated with severity of childhood adversity in psychotic patients. Since the amygdala is comprised of separate subnuclei with distinct anatomy and function we wanted to study whether these effects are present in some subnuclei more than others in first episode of psychosis.
**Methods:** We studied amygdala subnuclei volumes in 68 first-episode psychosis (FEP) patients (mean age = 27.1 ± 6.2, 35 females) and 65 healthy controls (mean 28.9 ± 6.5, 33 females) randomly selected from the general population. Subjects underwent a T1-weighted MRI with 1mm isotropic resolution (Philips Ingenuity 3T). The subnuclei volumes were generated with a new automated algorithm in FreeSurfer. Childhood adversity was measured using the Trauma and Distress Scale Scores (TADS). Baseline group differences in the amygdala subnuclei volumes were tested using repeated measures general linear model. The analyses were restricted to the four largest subnuclei: the lateral, basal, accessory basal, and the corticoamygdaloid transition area with volumes > 100 mm3. There were no differences between hemispheres nor group by hemisphere interactions so left and right hemispheres were averaged. All group comparisons were corrected for age, sex, and total intracranial volume. Association between the volumes and the TADS scores in the FEP group were also corrected for cumulative exposure to antipsychotic medication.
**Results:** We found that amygdala subnuclei were smaller in the FEP patients than in the the controls with regional specifity (subnucleus ROI*Group $p = 0.015$). In the FEP, the most robust reductions were in the lateral nucleus (Bonferroni corrected $p = 0.036$, $\beta = -64.15$). No statistically significant difference was observed in the basal nucleus, the accessory basal nucleus or the corticoamygdaloid transition area. The FEP patients had in average higher TADS total score (19.00 ± 13.56) compared to the HC (7.68 ± 7.07) ($p < 0.001$, $t = 5.84$).
We found that particularly the TADS physical abuse score (FEP(n)=63, HC(n)=59) associated significantly differently with some subnuclei in patients and control group (ROI*Group*Physical abuse $p = 0.016$). The difference was significant only in the lateral nucleus (Group*Physical abuse $p = 0.048$, $\beta = -34.97$). However, there was an overall nonsignificant trend of the negative association between lateral nucleus volume and all TADS scores in the FEP. Similar trend was not seen in the controls.
**Discussion:** We show that the amygdala subnuclei are differently affected already in the first episode of psychosis. Compared to the controls, the FEP patients had smaller lateral nucleus volume, but not basal, accessory basal nucleus or corticoamygdaloid transition area. The lateral nucleus volume was also negatively associated with childhood traumatic experiences, particularly physical abuse in the FEP patients. These findings suggest the involvement of the lateral nucleus of amygdala in the association between childhood traumatic experiences and psychotic disorders. This is well in agreement with studies suggesting that the lateral nucleus of the amygdala is associated with fear learning, recovery from fear and regulation of fear expression.

### S171. ALTERED WHITE MATTER CONNECTIVITY IN PATIENTS WITH SCHIZOPHRENIA USING PUBLIC NEUROIMAGING DATA FROM SCHIZCONNECT

Sung Woo Joo[1], Woon Yoon[2], Seung-Hyun Shon[2], Saetbyeol Chat[2], JungSun Lee*,[3]
*[1]University of Ulsan College of Medicine, Asan Medical Center; [2]Asan Medical Center; [3]University of Ulsan College of Medicine*

**Background:** Several studies have produced a large body of evidence for white matter abnormalities related to schizophrenia. The literature has yet to achieve a state of consistency and reproducibility, and reported low integrity of white matter tracts vary between studies. Whole brain image study with large sample size is needed to address this issue. We investigated white matter integrity in connections between regions of interests (ROI) in the same hemisphere in patients with schizophrenia and healthy controls with public neuroimaging data from SchizConnect (http://schizconnect.org).
**Methods:** A final data set was consisted of 129 healthy controls and 122 schizophrenia patients. For each diffusion weighted image (DWI), a two-tensor full-brain tractography was performed, and DWI images were parcellated by processing and registering the T1 images with FreeSurfer and the Advanced Normalization Tools. We extracted a total of 36 tracts in the both hemisphere connecting ROIs in the same hemisphere with white matter query language. We compared means of diffusion measures between patients and controls, and evaluated correlations with Letter-number sequencing (LNS) test, Vocabulary test, letter fluency test, category fluency test, and trails A of the Trail Making Test (TMT). The Benjamini-Hochberg procedure with false discovery rate (FDR) of 0.05 was used to correct for multiple comparisons.
**Results:** We found a significant RD and TR increase of the left thalamo-occipital tracts and the right uncinate fascicle (UF), and a significant RD increase of the right middle longitudinal fascicle (MDLF), and the right superior longitudinal fascicle (SLF) ii in schizophrenia. There were correlations between the TR in the left thalamo-occipital tracts and letter fluency test, and the RD in the right SLF ii and LNS test, which did not survive after correction for multiple comparisons.
**Discussion:** These results indicate widespread abnormalities of white matter fiber tracts in schizophrenia, contributing to the pathophysiology of schizophrenia.

### S172. BRAIN METABOLITES AND THE RELATION WITH COGNITION AND PSYCHOTIC SYMPTOMS IN MEDICATION-FREE PSYCHOSIS AND CONTROLS: A PHARMACOLOGICAL MAGNETIC RESONANCE SPECTROSCOPY STUDY

Claudia Vingerhoets*,[1], Geor Bakker[1], Marieke van der Pluijm[2], Oswald Bloemen[3], Liesbeth Reneman[2], Matthan Caan[2], Jan Booij[4], Therese van Amelsvoort[1]
*[1]Maastricht University; [2]Academic Medical Center; [3]GGz Centraal,Maastricht University; [4]Academic Medical Center, University of Amsterdam*

**Background:** Psychotic disorders are complex neuropsychiatric disorders characterized by positive, negative and cognitive symptoms. Over the recent years, several neurotransmitter systems and neurometabolites have been related to psychotic disorders but the exact underlying neurobiological mechanisms are still not well understood. One neurotransmitter system that has been increasingly related to psychosis is the cholinergic muscarinic system. Increased choline concentrations and reduced muscarinic M1 receptor expression have been reported in schizophrenia. Therefore, the present study investigated brain metabolite concentrations, their responsivity to M1 receptor blockage, and their relation to cognitive, positive and negative symptoms in psychosis.
**Methods:** 31 medication-free subjects with a psychotic disorder (mean age 27 years) and 31 gender, age and IQ-matched healthy control subjects (mean age 25 years) were enrolled in the study. 1H-proton magnetic resonance spectroscopy (1H-MRS, PRESS) was used to measure brain metabolites in the anterior cingulate cortex (ACC) and striatum. Metabolites measured included choline (Cho), glutamate (Glu), glutamine (Gln), GLX, myoinositol (MI), N-acetylaspartate (NAA) and gluthatione (GSH) (metabolite to creatine ratios were analyzed). All subjects were measured twice: once after placebo and once after a pharmacological challenge (4 mg. biperiden, a M1 receptor antagonist). The order of drug – challenge was counterbalanced. In addition, cognitive function was assessed using the Cambridge Neuropsychological Test Automated Battery (CANTAB) and psychotic symptom severity was assessed with the Positive and Negative Syndrome Scale (PANSS) to examine the relation between brain metabolites and cognition and psychosis symptoms.
**Results:** No significant differences were found in both ACC and striatal brain metabolite levels between subjects with a psychotic disorder and controls after placebo. Moreover, M1 blockade did not significantly affect brain metabolite levels in these regions and no group x challenge interaction effects were found. In addition, in both groups, no correlation was found between cognitive functioning and any of the brain metabolites. In subjects with a psychotic disorder, a positive correlation was found between striatal choline levels (after placebo) and negative symptom severity ($p = 0.024$).
**Discussion:** These results suggest that there are no differences in ACC and striatal brain metabolites between medication-free subjects with a psychotic disorder and healthy controls and that these metabolites are not influences by acute muscarinic M1 receptor antagonism. The significant correlation between striatal choline and negative symptom severity in the psychosis group could indicate that the cholinergic system is involved in negative symptom pathology. This is the first study that examined the influence of M1 receptor blockade on brain metabolites and therefore these results warrant replication.

### S173. GREY MATTER VOLUME DEFFICITS IN PATIENTS WITH A FIRST EPISODE NON-AFFECTIVE PSYCHOSIS AND SUICIDE RELATED BEHAVIOUR

Diana Tordesillas-Gutiérrez*[,1], Manuel Canal-Rivero[2], Esther Setien-Suero[3], Rosa Ayesa-Arriola[3], Manuel Delgado-Alvarado[2], Benedicto Crespo-Facorro[3]
*[1]Neuroimaging Unit, Technological Facilities, Valdecilla Biomedical Research Institute IDIVAL; [2]Neuroimage Unit, Technological Facilities, IDIVAL; [3]University Hospital Marques de Valdecilla -IDIVAL; University of Cantabria, CIBERSAM*

**Background:** Suicide represents the main cause of premature dead in first episode psychosis (FEP) patients. However, our understanding of suicidal behaviour in this population is limited. During the last decade, several works have related suicidal behaviour in FEP patients with structural abnormalities in frontal and temporal areas as well as specific structures such as hippocampus, insula and amygdala. The main aim of this work was to analyse the possible structural brain abnormalities associated with suicide-related-behaviour in a large sample of FEP patients.
**Methods:** We use a voxel-based morphometry (VBM) analysis in 146 FEP individuals: 24 FEP with and 122 without suicidal behaviour. All images were taken in the same 3T Phillips scanner. The CAT 12 toolbox, which is implemented in SPM12 was used for VBM analysis of the data. A two-sample t-test was set with sex, age, handedness, total intracraneal volume and global disability score as nuisance covariables. We applied threshold-free cluster enhancement (TFCE) with 5000 permutations and corrected for multiple comparisons (FWE) at $p<0.05$.
**Results:** A gradual reduction of grey matter volume related to presence of suicide-related-behaviour was found in frontal area, specifically in superior frontal gyrus, middle frontal gyrus, precentral gyrus, inferior frontal gyrus and orbital gyrus. In addition, significant reduction was found in middle temporal gyrus as well as in posterior cingulate gyrus and precuneus.
**Discussion:** Our results are in line with previous works which related suicidal behaviours with reduced frontal regions. Frontal areas are involved in: i) cognitive analysis; ii) foresight and weighing consequences of behaviour; iii) considering future and making predictions; iv) impulse control; v) delaying gratification; vi) inhibiting inappropriate behaviour; vii) initiating appropriate behaviour. Reestructuraria esta frase asi: On the other hand, precuneus is involved in: i) episode memories; ii) reflective self-awareness; iii) executive function; and iv) it is activated during judgements. Finally, cingulate gyrus has been strongly associated with emotional responses to pain, regulation of aggressive behaviour and decision making. Finally, middle temporal gyrus appears to play an important role in retrieving semantic information.
This study provides some insights about brain abnormalities associated with suicide-related-behaviours in FEP patients. In particular, the areas reported in this study are related with important functions such as impulsivity, emotional processing information, responses to pain and aggressiveness which are strongly associated with suicide-related-behaviours. Further studies are necessary to replicate the relevance of these structures in suicidal behaviour in FEP patients.

### S174. ABNORMAL SOCIAL COGNITION RELATED TO STRUCTURAL DISCONNECTIVITY IN SCHIZOPHRENIA

Molina Vicente*[,1], Alba Lubeiro Juárez[1], Eva Sotelo[2], Mercedes Vaquero[2], Rodrigo de Luis-García[1]
*[1]University of Valladolid; [2]University Hospital of Valladolid*

**Background:** Social cognition impairments are found in schizophrenia patients and hamper their ability to form social relationships. The biological underpinnings of this social cognition impairment are poorly investigated. We hypothesize that structural disconnectivity, which is replicated in schizophrenia, might has a relevant role in social cognition.
**Methods:** The study we present here is under development. We have assessed social cognition using the Mayer, Salovey and Caruso emotional intelligence test (MSCEIT) in 30 patients with schizophrenia and 20 healthy controls. Structural connectivity is assessed with anatomical and Diffusion weighted (DWI) images acquired in a 3 Tesla MRI system. Anatomical and DWI images are processed to obtain fractional anisotropy (FA) values in the tracts connecting prefrontal cortex with anterior cingulate, superior temporal gyrus, insula and superior parietal cortex. The following statistics are assessed i) the differences in MSCEIT scores between patients and controls, ii) the differences in FA values between groups, iii) the relation between MSCEIT punctuation and FA values.

**Results:** In our preliminary analyses, patients show significantly lower MSCEIT scores. Furthermore, MSCEIT scores are directly related to FA values in the tracts connecting prefrontal cortex to anterior cingulate and superior temporal gyrus in the patients.
**Discussion:** Social cognition impairments seem to be associated with altered structural connectivity in the patients.

### S175. AMOTIVATION IS ASSOCIATED WITH SMALLER VENTRAL STRIATUM VOLUMES IN OLDER PATIENTS WITH SCHIZOPHRENIA

Fernando Caravaggio*[,1], Gagan Fervaha[2], Yusuke Iwata[3], Eric Plitman[3], Jun Ku Chung[3], Shinichiro Nakajima[1], Wanna Mar[3], Philip Gerretsen[1], Julia Kim[3], Mallar Chakravarty[4], Benoit Mulsant[1], Bruce Pollock[1], David Mamo[1], Gary Remington[3], Ariel Graff-Guerrero[3]
*[1]Centre for Addiction and Mental Health, University of Toronto; [2]Queen's University Faculty of Medicine; [3]Centre for Addiction and Mental Health; [4]McGill University*

**Background:** Motivational deficits are prevalent in patients with schizophrenia, persist despite antipsychotic treatment, and predict long-term outcomes. Evidence suggests that patients with greater amotivation have smaller ventral striatum (VS) volumes. We wished to replicate this finding in a sample of older, chronically medicated patients with schizophrenia. Using structural imaging and positron emission tomography, we examined whether amotivation uniquely predicted VS volumes beyond the effects of striatal dopamine D2/3 receptor (D2/3R) blockade by antipsychotics.
**Methods:** Data from 41 older schizophrenia patients (mean age: $60.2 \pm 6.7$; 11 female) were reanalysed from previously published imaging data. We constructed multivariate linear stepwise regression models with VS volumes as the dependent variable and various sociodemographic and clinical variables as the initial predictors: age, gender, total brain volume, and antipsychotic striatal D2/3R occupancy. Amotivation was included as a subsequent step to determine any unique relationships with VS volumes beyond the contribution of the covariates. In a reduced sample (n = 36), general cognition was also included as a covariate.
**Results:** Amotivation uniquely explained 8% and 6% of the variance in right and left VS volumes, respectively (right: $\beta = -.38$, $t = -2.48$, $P = .01$; left: $\beta = -.31$, $t = -2.17$, $P = .03$). Considering cognition, amotivation levels uniquely explained 9% of the variance in right VS volumes ($\beta = -.43$, $t = -0.26$, $P = .03$).
**Discussion:** We replicate and extend the finding of reduced VS volumes with greater amotivation. We demonstrate this relationship uniquely beyond the potential contributions of striatal D2/3R blockade by antipsychotics. Elucidating the structural correlates of amotivation in schizophrenia may help develop treatments for this presently irremediable deficit.

### S176. SYSTEMATIC REVIEW AND META-ANALYSIS OF MAGNETIC RESONANCE IMAGING FINDINGS IN 22Q11.2 DELETION SYNDROME

Guido Maria Lattanzi*[,1], Cristina Scarpazza[2], Fabio Di Fabio[3], Philip McGuire[1], Giuseppe Sartori[4], Simon B. Eickhoff[5], Stefania Tognin[1]
*[1]Institute of Psychiatry, Psychology & Neuroscience, King's College London; [2]Institute of Psychiatry, Psychology & Neuroscience, King's College London, University of Padua; [3]Sapienza University of Rome; [4]University of Padua; [5]Institute for Systems Neuroscience Heinrich-Heine University Düsseldorf*

**Background:** Since the 22q11.2 Deletion Syndrome (22q11.2 DS) is the most important genetic model for psychotic disorders, an increasing interest in its brain structural and functional abnormalities has arisen in the last decade. However, studies so far have reported inconsistent findings. Therefore, the aims of the present study are 1) to systematic review the literature on structural and functional brain abnormalities associated to 22q11.2 DS and 2) to identify the most consistently reported abnormalities through a meta-analysis of structural (sMRI) and functional magnetic resonance imaging (fMRI) studies.
**Methods:** The following electronic databases were systematically searched: PubMed, ETHOS, Kings Open Portal, EMBASE, MEDLINE, PsycINFO and CINHAL. Studies were included if they presented original data, were written in English, had a sample size larger than 5, had a healthy control comparison group, and if they reported results from a whole brain analysis. As we were interested in identifying abnormalities in both brain structure and function, in the systematic review we included studies that used different imaging techniques (i.e. sMRI, fMRI and diffusion tensor imaging (DTI)). The meta-analysis was performed with studies reporting results in standardised-space coordinates (e.g. Talairach), using the Activation Likelihood Estimation (ALE) method. Results were corrected at cluster level with family wise error correction (p=0.01) and 1000 permutations.
**Results:** Seventy-three original articles were included in the systematic review, 25 of these were also included in the meta-analysis. Forty-two sMRI, 23 fMRI and 11 DTI articles were retrieved. Only one study performed a direct comparison between 22q11.2 DS individuals with and without psychosis.
The systematic review revealed that the most affected areas were the frontal middle gyri bilaterally, the posterior cingulum bilaterally, the right cuneus, the precuneus bilaterally, the right superior temporal gyrus, the left parietal inferior gyrus and the left side of the cerebellum.
The meta-analysis revealed consistent abnormalities in a cluster located in the inferior parietal lobe (4936 voxels, peak of activation in the coordinate -44 -52 48) and extending to the superior temporal gyrus, supramarginal gyrus and precuneus. A second cluster of consistent activation is found in the posterior cingulate cortex (3104 voxels, peak of activation in the coordinate 6 -50 16).
**Discussion:** The systematic review revealed widespread abnormalities throughout the brain, mainly within areas involved in visual and speech processing, language, and within association areas. The meta-analysis of structural and functional studies revealed consistent abnormalities in the inferior parietal lobe, an area consistently found affected in psychosis.
Only few studies on 22q11.2 DS individuals with psychosis were available and most studies included young individuals (mean age 15.12) rather than adults. 22q11.2 DS is one of the most compelling genetic models of schizophrenia, however most imaging studies do not provide clinical data on psychotic symptoms. This could partially be explained by the relatively low mean age of the overall sample; some participants could have been too young to manifest psychotic symptoms. Finally, the present study does not allow to make inferences on brain changes overtime as longitudinal studies were scarce.
Future studies should adopt a longitudinal design and investigate brain abnormalities in adults with 22q11.2 DS displaying symptoms of psychosis. This would help to clarify the brain structural and functional features associated with this particular form of psychosis and their longitudinal course.

### S177. FRONTAL CORTICAL PLASTICITY IN SCHIZOPHRENIA PATIENTS EXAMINED BY LTP-INDUCING ANODAL TDCS AND REPETITIVE EEG

Benjamin Pross*[,1], Melina Siamouli[1], Oliver Pogarell[1], Peter Falkai[1], Alkomiet Hasan[1], Wolfgang Strube[1]
*[1]Ludwig Maximilians University Munich*

**Background:** Frontal cortical deficits have repeatedly been shown to be relevant in the development of psychiatric disorders and are supposed to evoke characteristic psychiatric and cognitive symptoms in schizophrenia. It is assumed that plasticity and connectivity impairments following non-invasive brain stimulation, which are observed as common patterns in the motor system of schizophrenic patients, are as well present in frontal cortical areas and cause the mentioned dysfunctions. Until now experimental evidence is lacking substantiating that this hypothesis is correct and both cortical regions show similar patterns of deficits. Hence, this study aimed to assess the plasticity and connectivity in the frontal cortex of schizophrenia patients.
**Methods:** We applied anodal transcranial direct current stimulation (a-tDCS) to evoke long-term potentiation (LTP)-like plasticity in the dorsolateral prefrontal cortex (DLPFC). This non-invasive brain stimulation has been demonstrated to evoke plasticity in frontal cortical regions. As tDCS modulates cortical activity we employed electroencephalography (EEG) measurements to trace potential deficits in patients with schizophrenia compared to healthy participants. In total 20 schizophrenia patients and 20 age, gender and handedness matched healthy controls received 13Min of a-tDCS (1mA). EEG was measured before and after plasticity induction (up to 50 minutes) to record neuronal changes in excitability and plasticity.
**Results:** First analyses obtained a significant EEG alpha-activity change after LTP application in the frontal cortex of schizophrenia patients. This effect remained stable up to 50 minutes following a-tDCS stimulation.
**Discussion:** We were able to show for the first time that anodal tDCS is capable of inducing stable EEG alpha-activity changes in the frontal cortex of schizophrenia patients. Future analyses will focus on differences to healthy participants, which we hypothesize to show similar but stronger patterns of activity changes after a-tDCS stimulation.

### S178. ALTERED GYRIFICATION IN THE SCHIZOPHRENIA SPECTRUM

Daiki Sasabayashi*[,1], Yoichiro Takayanagi[1], Tsutomu Takahashi[1], Yusuke Takegoshi[2], Tokio Matsuda[2], Atsushi Furuichi[1], Mikio Kido[1], Yumiko Nishikawa[1], Mihoko Nakamura[1], Kyo Noguchi[1], Michio Suzuki[1]
[1]*University of Toyama Graduate School of Medicine and Pharmaceutical Sciences;* [2]*University of Toyama*

**Background:** Increased gyrification in diverse cortical areas has been reported in patients with schizophrenia, which is considered to reflect deviations in early neurodevelopment. Schizotypal personality disorder (SPD) is thought to be a prototypic disorder within the schizophrenia spectrum, which shares biological and psychological commonalities with schizophrenia as a neurobiological basis for vulnerability factors. However, to the best of our knowledge, no magnetic resonance imaging (MRI) studies have investigated the gyrification pattern in SPD.
**Methods:** T1-weighted structural MRI scans were obtained by 1.5-T scanner from 101 patients with schizophrenia, 46 patients with SPD, and 77 age- and gender- matched healthy control subjects. Using FreeSurfer software (version 5.3.), the local gyrification indices (LGIs) of entire cortex were obtained with the method of Schaer and colleagues. Clinical symptoms of the patients were rated with the Scale for the Assessment of Positive Symptoms (SAPS) and the Scale for the Assessment of Negative Symptoms (SANS) at the time of scanning. A general linear model controlling for age, gender, medication dose, and duration of medication was used to compare the LGIs across the groups and to conduct vertex-by-vertex whole brain LGI correlation analyses with clinical variables. This study was approved by the Committee on the Medical Ethics of Toyama University based on the declaration of Helsinki. After a complete description of the study was provided, written informed consent was obtained from all subjects.
**Results:** Compared with the controls, the patients with schizophrenia showed significantly higher LGI in widespread cortical areas including the bilateral frontal, parietal, and occipital regions. The patients with SPD demonstrated significantly higher LGI in the bilateral frontal and left parietal regions compared with the controls. Compared with the patients with SPD, the patients with schizophrenia showed significantly higher LGI in the left occipital and right frontal regions. Both SAPS and SANS total scores were positively correlated with LGI in the bilateral temporal regions in patients with schizophrenia, and were negatively correlated with LGI in the bilateral occipital regions in patients with SPD.
**Discussion:** Increased LGI in the bilateral frontal regions may be the common morphological substrates for the schizophrenia spectrum, possibly representing vulnerability to schizophrenia. In addition, increased LGI in the left occipital and right frontal regions preferentially observed in schizophrenia may have a critical role in manifestation of florid psychotic symptoms.

### S179. PROGNOSTIC UTILITY OF MULTIVARIATE MORPHOMETRY IN SCHIZOPHRENIA

Mingli Li*[,1], Xiaojing Li[1], Deng Wei[1], Yinfei Li[1], Liansheng Zhao[1], Xiaohong Ma[1], Yingcheng Wang[1], Hua Yu[1], Ya-jing Meng[1], Qiang Wang[1], Lena Palaniyappan[2], Tao Li[1]
[1]*Mental Health Center, West China Hospital of Sichuan University;* [2]*University of Western Ontario*

**Background:** Groups of spatially distributed regions show shared variance in morphometric properties (e.g. grey matter volume) among subjects, thus forming independent morphometric 'sources' or covariance-based networks. Source based morphometry is a multivariate approach that is based on independent component analysis, and accounts for the inter-relationahsip among different brain regions while filtering out noisy artefactual effects of mass univariate voxel-based approaches. We have previously demonstrated that with multivariate SBM, it is possible to identify the structural basis of subtle psychopathological features such as formal thought disorder, whose anatomical correlates have been hitherto elusive. In the current study, we use multivariate SBM to identify the morphometric sources in drug-naïve first episode subjects that show progressive changes that predict symptom change over 1 year.
**Methods:** 63 first-episode, drug-naive patients with schizophrenia underwent brain magnetic resonance imaging scans at baseline (T0) and rescanned after 1 year follow-up (T1). Positive and Negative Syndrome Scale (PANSS) was used to assess their psychopathology. Source based morphometry (SBM) was performed to analyze the gray matter volume (GMV), paired T contrasts for loading coefficients of GMV were constructed to detect the components that showed a significant effect of time. The change in PANSS scores between baseline and 1 year was expressed as a ratio of the scores at baseline - adjusted change scores for positive symptoms (POS%), negative symptoms (NEG%) and disorganization symptoms (DISORG%), with each domain score derived using van der Gaag's 5-factor approach. Multiple regression analysis was conducted to predict the percentage change scores in each domain using the T0 and T1 loading coefficients of components showing time effect with age, gender and cumulative antipsychotic dose as covariates.
**Results:** Of the 30 spatial components of gray matter identified by SBM, loading coefficients of anterior cingulate cortex (ACC), anterior insula (AI) & inferior frontal gyrus (IFG), superior temporal gyrus (STG), middle temporal gyrus (MTG) and dorsal lateral prefrontal cortex (DLPFC) reduced with time in patients. The lower volume of AI & IFG at baseline and at 1 year related to poor improvement in positive and disorganization symptoms; lower volume of STG & MTG at baseline and 1 year predicted poor improvement in negative symptoms.
**Discussion:** The baseline distribution of GM in AI & IFG, STG and MTG are predictive of the course of illness. The relationship between GM sources and symptom severity continues even after 1 year of naturalistic exposure to antipsychotic treatment. If judiciously combined with other available predictors of prognosis, source-based morphometric analysis can aid meaningful prognostication in schizophrenia.

### S180. MICROSTRUCTURE COMPLEXITY OF THE THALAMUS IN SCHIZOPHRENIA: A NODDI STUDY

Yasser Alemán-Gómez[1], Timo Roine[1], Elena Najdenovska[1], Philippe Golay[1], Zita Rovó[1], Raoul Jenni[1], Martine Cleusix[1], Philippe Conus[1], Kim Q. Do Do[1], Pascal Steullet[1], Meritxell Bach Cuadra[1], Philipp Baumann*,[2]
*[1] Centre Hospitalier Universitaire Vaudois (CHUV) and University of Lausanne (UNIL); [2]DP-CHUV (TIPP)*

**Background:** Schizophrenia is a neurodevelopmental disease arising from complex interactions between genetic and environmental factors that cause disconnectivity within core brain networks including the thalamus. The thalamus has a central role in the pathophysiology of schizophrenia, however to what extent and how it is affected at the microstructural level is still a matter of debate. In the current study, we apply the Neurite Orientation Dispersion and Density Imaging (NODDI) [1], a recently developed MRI technique, which allows the estimation of the microstructural complexity of dendrites and axons in vivo.
**Methods:** Twenty-three patients with schizophrenia (SCHZ) were recruited from the Service of General Psychiatry (Lausanne University Hospital, Switzerland) (40.18 ± 9.2yo; 18/5 males/females) and 27 healthy controls (HC) (37.7 ± 7.95yo; 18/9 males/females). Magnetization-Prepared Rapid Acquisition Gradient Echo (MPRAGE) and a diffusion spectrum imaging (DSI) was performed on a 3-Tesla scanner (MAGNETOM Trio a Tim system, Siemens, Germany). Thalamus segmentation was performed on the MPRAGE sequence with an in house-pipeline using Freesurfer v5.0.0 for segmentation which was then refined to remove voxels within the ventricles and/or overlapping the internal capsule [2]. Orientation Dispersion Index (ODI), Intracellular Volume Fraction (ICVF) and, Isotropic Volume Fraction (ISOVF) were estimated based on the DSI sequence with NODDI [1]. General Linear Models (GLM) were estimated with outcome measures (ICVF, ISOVF, ODI) as dependent variables, group membership as a fixed factor (HC vs. SCHZ) and age and gender as potential covariates.
References:
1Battistella G, Najdenovska E, Maeder P, et al. Robust thalamic nuclei segmentation method based on local diffusion magnetic resonance properties. Brain Struct Funct 2016. DOI:10.1007/s00429-016-1336-4.
2Zhang H, Schneider T, Wheeler-kingshott CA, Alexander DC. NeuroImage NODDI : Practical in vivo neurite orientation dispersion and density imaging of the human brain. Neuroimage 2012; 61: 1000–16.
**Results:** Mean ODI was significantly increased in schizophrenia patients compared to controls in the right thalamus (F(1,48)=5.032, p= .030, np2 = .095) and in the left thalamus (F(1,48)=4.500, p= .039, np2 = .086). When controlled for age and gender, the difference remained significant for the right thalamus (F(1, 46) = 4.197, p = .046, np2 = .084) but reduced to trend level for the left thalamus (F(1, 46) = 4.029, p = .051, np2 = .081). There were no significant differences on the other measures (ICVF, ISOVF).
**Discussion:** Our results show that the thalamus is affected in patients with SCHZ at the microstructural level. The observed increase in ODI, which estimates the dispersion of neurite orientations, suggests disrupted neurite organization in patients as compared to HC.

### S181. THE STATE OR TRAIT COMPONENT OF DOPAMINE AND GLUTAMATE DYSFUNCTION IN THE RISK FOR PSYCHOSIS: AN IN VIVO MULTIMODAL IMAGING STUDY OF INDIVIDUALS WITH 22Q11.2 DELETION

Maria Rogdaki*,[1], Mattia Veronese[2], Pamela Hathway[3], Sameer Jauhar[4], Maria Gudbrandsen[4], Eileen Daly[4], Oliver Howes[5]
*[1]MRC London Institute of Medical Sciences, Imperial College; [2]Center for Neuroimaging Science, Institute of Psychiatry, Psychology and Neuroscience, King's College London; [3]Imperial College; [4]Institute of Psychiatry, Psychology & Neuroscience, King's College London; [5]MRC London Institute of Medical Sciences, King's College London*

**Background:** Dopaminergic and glutamatergic dysregulation are among the leading hypotheses for schizophrenia. Previous in vivo imaging studies have shown that increased striatal presynaptic dopamine synthesis capacity (DSC) predates the onset of psychosis and is associated with symptom severity. Recent meta-analysis in magnetic resonance spectroscopy studies in schizophrenia reported increased levels of glutamate and glutamine (Glx) in basal ganglia and medial frontal cortex in clinical high-risk groups for psychosis. Despite the evidence of alterations in both dopamine and glutamate in schizophrenia, the degree to which these alterations are trait markers linked to genetic risk for psychosis or reflects state changes is not clear from previous studies. Over the last fifteen years, it has been well established that 22q11 deletion is one of the most important genetic risk factors for the development of schizophrenia. Individuals with 22q11 deletion are at increased genetic risk for psychosis, reaching a prevalence 30% for psychotic disorder. The aims of our study were to investigate dopaminergic and glutamatergic function in individuals with 22q11.2 deletion.
**Methods:** Participants underwent 18F DOPA PET, MRI, as well as clinical measures.21 individuals with 22q11 deletion (14 females and 7 males, age (mean, SD): 26.1(7.72)) and 26 healthy volunteers (15 females and 11 males, age (mean, SD): 26.12(4.28)) took part in the 18F DOPA PET. Standardised ROIs was defined in the striatum, including limbic, associative and sensorimotor sub-regions, and the reference region, defined according to previous study. The ROI atlas was normalised to each individual PET dynamic image. A Patlak analysis was applied to calculate influx constants (Ki values) for the whole striatal ROI relative to uptake in the cerebellar reference region (Kicer [min-1]).
In addition, 17 individuals with 22q11 deletion (11 females and 6 males, age (mean, SD): 26.39(7.7)) and 30 healthy controls (17 females and 13 males, age (mean, SD): 27.17(4.8)) had MRI. 1H-MRS voxels were placed on the anterior cingulate cortex and left striatum. Spectra were analyzed using LC Model version 6.3-1L. Poorly fitted metabolite peaks (Cramer–Rao minimum variance bounds >20% as reported by LC Model) were excluded from further analysis.
**Results:** DSC in the whole striatum was significantly increased in the individuals with 22q11 deletion compared to healthy controls (mean (Kicer [min-1] =0.0143; SD=0.001; mean Kicer [min-1] = 0.0127; SD=0.001, respectively; effect size (Cohen's d= 1.47, p< 0.000). In addition, no difference was found between groups in Glx levels in anterior cingulate cortex (individuals with 22q11 deletion; mean=19.85; SD=3.17, healthy controls; mean= 21.04; SD=3.91) and left striatum (individuals with 22q11 deletion; mean=11.45; SD=2.88, healthy controls; mean=11.6;SD=2.74)(t(44) =-1.065; p=0.29, t(42)=-0.172;p=0.86, respectively).Psychopathology scales were not correlated with either dopaminergic or glutamatergic function in the group of 22q11 deletion.
**Discussion:** Our findings provide evidence that dopamine synthesis capacity has a strong trait component and glutamatergic dysfunction may be most likely associated with disease status. Future studies with longitudinal design are warranted to further investigate the role of dopamine and glutamate in individuals with 22q11 deletion. Moreover, there is a clear need for studies utilizing higher field resonance strength and appropriate radiotracers to investigate glutamatergic function in this population. These will be crucial steps towards the development of new treatments, that can be applied in early stages of illness.

### S182. CHANGE IN CORTICAL MORPHOMETRY IN INDIVIDUALS WITH PERSISTING PSYCHOTIC EXPERIENCES: A LONGITUDINAL PILOT STUDY

Leon Fonville*[,1], Mark Drakesmith[2], Avi Reichenberg[3], Derek Jones[2], David Linden[4], Anthony David[5]
*[1]University of Westminster; [2]Cardiff University Brain Research Imaging Centre; [3]Icahn School of Medicine at Mount Sinai; [4]MRC Centre for Neuropsychiatric Genetics & Genomics, Cardiff University School of Medicine; [5]Institute of Psychiatry, King's College London*

**Background:** There is an increasing interest in the presence of psychotic symptoms in the general population that do not meet the threshold for psychotic disorder. Such psychotic experiences (PEs) are more prevalent than conditions such as schizophrenia and often manifest during childhood and adolescence. PEs are a risk factor for psychotic disorders and a range of adverse psychosocial outcomes. PEs can become abnormally persistent with a corresponding increase in psychiatric morbidity. Previous research has highlighted subtle deviations in brain structure associated with the presence and persistence of PEs, but longitudinal assessments are needed to gauge if these deviations continue, perhaps as part of an atypical neurodevelopmental trajectory.
**Methods:** Longitudinal imaging data, taken at ages 20 and 25, were available for 25 young adults who were part an MRI nested case control study within the Avon Longitudinal Study of Parents and Children. They were selected originally on the basis of presence or absence of PEs. Cortical surface data were analysed using Freesurfer. Presence of PEs was assessed at ages 18, 20, and 25 and we defined persistence as endorsing PEs at multiple time-points. Average cortical volume and thickness were extracted from each brain parcellation. We additionally calculated fractal dimensionality (FD) of each parcellation using a box-count algorithm to capture shape complexity. We compared the rate of change between healthy controls (HC) and those with persistent PEs in each parcellation and used permutation testing to control for multiple comparisons.
**Results:** Both HC and PEs showed the expected age-related net loss in brain volume; an increase in white matter volume offset by a greater reduction in grey matter. We identified greater volume loss in PEs in the left parietal lobe and further examination of local volume highlighted additional changes. PEs were associated with a greater rate of volume loss in the anterior cingulate, postcentral, and lingual gyrus in the left hemisphere and in the right inferior parietal lobule and pars orbitalis. Thinning of the left inferior parietal lobule was greater in those with PEs. There was further converging evidence of focal abnormalities in the left postcentral gyrus in terms of reductions in cortical thickness and FD in PEs. Similarly, we found reduced FD relative to HC in the left rostral anterior cingulate. There was additional evidence for reductions in FD in the left hemisphere in the cuneus, isthmus cingulate, and middle frontal gyrus.
**Discussion:** Our findings highlight a deviation from typical age-related changes in brain volume in individuals with persisting manifestations of PEs. Though these changes could reflect an acceleration of the typical volume loss that is seen with aging, there are several points of evidence against this. We found no differences in global volume changes and only the left parietal lobule was found to show a greater volume loss in PEs. On a local scale, findings seemed to mostly converge on parietal and cingulate regions in the left hemisphere with some evidence of aberrations in frontal regions. These pilot data are, uniquely, unconfounded by illness and treatment related factors and highlight the continued need for longitudinal assessments of brain structure in relation to PEs; there is an increasing risk of transitioning to a psychotic disorder with persistence of PEs and our findings may reflect the neuroanatomical basis for an anomalous developmental trajectory related to psychotic disorders.

### S183. ABNORMALITIES OF FRONTO-SUBCORTICAL PATHWAYS IN SCHIZOPHRENIA AND THE DIFFERENTIAL IMPACTS OF ANTIPSYCHOTIC TREATMENT: A DTI-BASED TRACTOGRAPHY STUDY

Anaïs Vandevelde*[,1], Elise Leroux[1], Maxime Tréhout[1], Sonia Dollfus[1]
*[1]Normandie University*

**Background:** The fronto-striato-thalamic circuitry is a key network associated with several symptoms observed in patients with schizophrenia (SZPs). In this study, we use diffusion tensor imaging (DTI) to investigate the integrity of white matter (WM) pathways involved in this network in SZPs relative to healthy controls (HCs). We also evaluate the differential impact of chronic exposure to clozapine as well as other atypical and typical antipsychotics on fasciculi integrity in this network in schizophrenia.
**Methods:** 63 HCs and 41 SZPs were included in this study. Of the SZPs, 16 were treated with clozapine (SZPsC), 17 with atypical antipsychotics (SZPsA), and 8 with typical antipsychotics (SZPsT). We reconstructed three tracts belonging to the fronto-striato-thalamic network in the left hemisphere using tractography: one fronto-subcortical tract (FSC), one prefronto-subcortical tract (PFSC), and one prefronto-frontal tract (PFF). Diffusion parameters were individually extracted in each tract.
**Results:** SZPs exhibited lower integrity in both the FSC and PFSC relative to HCs, and SZPsT patients showed altered integrity compared to SZPsC patients. There were no WM integrity differences in the PFF between SZP groups or between SZPs and HCs.
**Discussion:** These results suggest that SZPs exhibit structural connectivity abnormalites in the prefronto-fronto-subcortical network that are specifically and differentially impacted by the type of antipsychotic treatment. Additional studies are needed to separate the contributions of clozapine-mediated neuroprotection, neurotoxicity related to typical antipsychotics, and the illness itself to the observed differences.

### S184. MACHINE LEARNING REVEALS DEVIANCE IN NEUROANATOMICAL MATURITY PREDICTIVE OF FUTURE PSYCHOSIS IN YOUTH AT CLINICAL HIGH RISK

Yoonho Chung*[,1], Jean Addington[2], Carrie Bearden[3], Kristen Cadenhead[4], Barbara Cornblatt[5], Daniel Mathalon[6], Thomas McGlashan[1], Diana Perkins[7], Larry Seidman[8], Ming Tsuang[4], Elaine Walker[9], Scott Woods[1], Sarah McEwen[3], Theo van Erp[10], Tyrone Cannon[1]
*[1]Yale University; [2]University of Calgary; [3]University of California, Los Angeles; [4]University of California, San Diego; [5]The Zucker Hillside Hospital; [6]University of California, San Francisco; [7]University of North Carolina; [8]Harvard Medical School; [9]Emory University; [10]University of California, Irvine*

**Background:** Both early (pre- and perinatal) and late (adolescent) neurodevelopmental disturbances are hypothesized to contribute to the pathophysiology of schizophrenia. Disturbances originating earlier in life (e.g., resulting from the interplay of genetic factors and obstetric complications) would be expected to affect brain integrity from birth onwards and could therefore help to explain cases with subtle deficits in premorbid functioning during childhood and earlier ages at onset of full psychosis (i.e., early to mid-teens). In contrast, disturbances that emerge during late adolescence and early adulthood (e.g., via abnormal neuromaturational events and/or environmental factors) could help to explain cases with normal premorbid psychological health and a more acute onset of psychotic symptoms and functional impairment in the late teens and early twenties. However, it is yet unclear whether neuroanatomical data among individuals at clinical high risk (CHR) for psychosis can be modeled to detect early versus late neurodevelopmental influences that is predictive of future psychosis onset. Therefore, in this study, we investigated whether the timing of the appearance or course of the deviation from normal brain maturation, as determined using a machine learning algorithm trained on structural MRI data to estimate age, is potentially relevant to the early versus late neurodevelopmental framework among CHR individuals.
**Methods:** A neuroanatomical-based age prediction model was trained using a supervised machine learning technique with T1 MRI scans from 953 typically developing healthy controls (HC) from the Pediatric Imaging, Neurocognition, and Genetics study (PING) study. The trained model was then applied to 109 HCs and 275 CHR, including 39 converters (CHR-C), from the North American Prodrome Longitudinal Study (NAPLS2) and 14 cases of first episode psychosis patients (FE) for external validation and clinical application. Discrepancy between neuroanatomical-based estimated age and chronological age was computed for each individual (i.e., brain age gap) and compared across clinical groups.
**Results:** The PING-derived model for estimating age accurately predicted NAPLS HC subjects' chronological ages, explaining 51% of the variance ($P < 0.001$) in chronological age, with a mean absolute error of 1.41 years, providing evidence of independent external validation. CHR subjects and FE adolescents showed a significantly greater overestimated gap between model-predicted age and chronological age compared with HC ($Ps < 0.01$). This effect was significantly moderated by chronological age, with neuroanatomical-based estimated age systematically overestimating CHR cases aged 12–17 years, but not among those aged 18–21 years. In the ROC analysis, brain age gap was a significant predictor of conversion to psychosis with an area under the curve of 0.63 ($P < 0.05$) among younger adolescents. In addition, increased deviation of brain age gap predicted pattern of stably low functioning over time ($P < 0.05$) among CHR individuals. In contrast, previously reported evidence of an accelerated reduction in cortical thickness among CHR-C was found to apply only to those cases who were 18 years or older.
**Discussion:** These results are consistent with the view that both early and later neurodevelopmental disturbances contribute to the onset and course of schizophrenia, with the two sets of influences having differing implications for the intercepts and trajectories in structural brain parameters as a function of age. The results also suggest that baseline neuroanatomical measures are likely to be useful in prediction of psychosis especially (or only) among CHR cases who are below 18 years of age at the time of ascertainment.

### S185. DTNBP1 IS ASSOCIATED WITH THE AGE AT ONSET OF KOREAN PATIENTS WITH SCHIZOPHRENIA

Kyuyoung Lee*[,1], Eun-jeong Joo[2]
[1]*Eulji General Hospital, Eulji University School of Medicine;* [2]*Eulji General Hospital*

**Background:** The dysbindin gene (DTNBP1) is located in chromosome 6p22.3, one of the regions of positive linkage for schizophrenia. In particular, dysbindin protein has been found to play a role in the glutatmate neural transmission in the brain. A strong genetic association between DTNBP1 and schizophrenia has been replicated through many recent studies. However, we have not replicated positive association between DTNBP1 and schizophrenia in our Korean sample. Because schizophrenia has been regarded as a disease with a quite heterogeneous origin and evolvement, it is useful to categorize patients with schizophrenia into relatively homogeneous subsets based on clinical characteristics including age at onset (AAO). We investigated the association between DTNBP1 and AAO of schizophrenia.
**Methods:** We assessed age at first occurrence of positive psychotic symptoms of 197 patients with schizophrenia with DSM IV diagnosis, which was re-evaluated by Korean version of Diagnostic Interview for Genetic Study. Five SNPs, SNPA, P1763, P1320, P1635 and P1655 of DTNBP1 were genotyped and genetic association analyses were performed using the PLINK program.
**Results:** In SNPA, patients with AT (N=10) showed significant earlier AAO than those with AA (N=187) ($p<0.0001$). The patients with heterozygote for SNP P1763 (TG, N=40) or P1320 (CT, N=41) also showed significant earlier AAO than those with homozygote ($P<0.0001$, $P<0.0001$, respectively). In addition, haplotype of all SNPs (SNPA-P1320-P1635-P1655-P1763) analysis showed significant association with AAO ($p=0.000953$).
**Discussion:** In conclusion, although we were unable to support an association between DTNBP1 and schizophrenia, DTNBP1 might play a role in disease modifying. However, considering the several limitations of this study, further research involving different polymorphisms in DTNBP1 and various clinical subsets with sufficient numbers will be required to evaluate the contribution of DTNBP1 to schizophrenia.

### S186. KCNH2 POLYMORPHISM ASSOCIATED TO ALTERED EEG FUNCTIONAL NETWORK MODULATION IN SCHIZOPHRENIA

Alba Lubeiro Juárez*[,1], Vicente Molina Rodriguez[1], Maria Guardiola[2], Javier Gómez Pilar[1], Mar Fatjó-Vilas[3]
[1]*University of Valladolid;* [2]*FIDMAG Sisters Hospitallers Research Foundation;* [3]*University of Barcelona, Biomedicine Institute of the University of Barcelona (IBUB), Centre for Biomedical Research Network on Mental Health (CIBERSAM)*

**Background:** The rs3800779 polymorphism at KCNH2 gene, which encodes for a Voltage-Gated Potassium Channel Subunit, has been associated with the risk for schizophrenia (SZ) and with changes in the expression of a brain isoform with specific electrophysiological characteristics (Huffaker et al., 2009). It has been hypothesized that the KCNH2 gene variability could be involved in SZ by means of modulating the neuronal excitability. Graph-theory parameters applied to electroencephalographic (EEG) activity are useful to assess functional connectivity in the brain and have shown altered patterns of global connectivity in schizophrenia. We aimed to investigate whether KCNH2 contributes to functional connectivity alterations replicated in SZ patients.
**Methods:** EEG data were acquired during the performance of an odd-ball task in 50 schizophrenia patients and 101 matched healthy controls. The rs3800779 at KCNH2 was genotyped. From the EEG activity, the Small World index (SW, a measure of network efficiency) was calculated as the coefficient between clustering coefficient (CLC, a measure of network segregation) and path length (PL, a measure of network integration). SW was calculated in two temporal windows with respect to the target tone (pre-stimulus and response). Functional SW modulation (SWm) was calculated as the difference in SW between pre-stimulus and response windows. Finally, the association between KCNH2 polymorphism and functional connectivity modulation was assessed.
**Results:** Patients carrying the A allele (AA or AC, n=25) showed smaller SW modulation in comparison with patients with the CC genotype (n=25) ($t=-2.84$, $df=48$, $p=0.007$). Moreover, patients carrying the A allele showed smaller SW modulation than healthy controls with the A allele (n=45) ($t=-3.41$, $df=68$, $p=0.001$) or without the A allele (n=56) ($t=-3.87$, $df=79$ $p<0.001$). There were no significant SW modulation differences between healthy controls carrying or not the A allele. Patients with the AA/AC genotype showed an inverse SW modulation pattern (decreased SW at response) in comparison with patients without the A allele and controls (increased SW at response).
**Discussion:** Our data indicate that, within SZ patients, the A allele is associated with smaller SW modulation and lower SW values at response, which might be interpreted as an altered ability to coordinate the activity of neural assemblies during cognitive tasks (Basar et al 2016). Although replication analyses are needed, our findings suggest that genetic variation at KCNH2 might contribute to the efficiency of brain functional networks in schizophrenia patients.

**References:**

1. Basar, E., Golbasi, B.T., Tulay, E., Aydin, S., Basar-Eroglu, C., 2016. Best method for analysis of brain oscillations in healthy subjects and neuropsychiatric diseases. Int J Psychophysiol 103, 22–42.
2. \Huffaker, S.J., Chen, J., Nicodemus, K.K., Sambataro, F., Yang, F., Mattay, V., Lipska, B.K., Hyde, T.M., Song, J., Rujescu, D., Giegling, I., Mayilyan, K., Proust, M.J., Soghoyan, A., Caforio, G., Callicott, J.H., Bertolino, A., Meyer-Lindenberg, A., Chang, J., Ji, Y., Egan, M.F., Goldberg, T.E., Kleinman, J.E., Lu, B., Weinberger, D.R., 2009. A primate-specific, brain isoform of KCNH2 affects cortical physiology, cognition, neuronal repolarization and risk of schizophrenia. Nature medicine 15(5), 509–518.

### S187. SEARCHING FOR BRAIN CO-EXPRESSION MODULES THAT CONTRIBUTE DISPROPORTIONATELY TO THE COMMON POLYGENIC RISK FOR SCHIZOPHRENIA

Javier Costas*[,1], Mario Paramo[1], Manuel Arrojo[1]
[1]*Instituto de Investigacion Sanitaria de Santiago de Compostela-SERGAS*

**Background:** Genomic research has revealed that schizophrenia is a highly polygenic disease. Recent estimates indicate that at least 71% of genomic segments of 1 Mb include one or more risk loci for schizophrenia (Loh et al., Nature Genet 2015). This extremely high polygenicity represents a challenge to decipher the biological basis of schizophrenia, as it is expected that any set of SNPs with enough size will be associated with the disorder. Among the different gene sets available for study (such as those from Gene Ontology, KEGG pathway, Reactome pathways or protein protein interaction datasets), those based on brain co-expression networks represent putative functional relationships in the relevant tissue. The aim of this work was to identify brain co-expression networks that contribute disproportionately to the common polygenic risk for schizophrenia to get more insight on schizophrenia etiopathology.

**Methods:** We analyzed a case -control dataset consisting of 582 schizophrenia patients from Galicia, NW Spain, and 591 ancestrally matched controls, genotyped with the Illumina PsychArray. Using as discovery sample the summary results from the largest GWAS of schizophrenia to date (Psychiatric Genomics Consortium, SCZ2), we generated polygenic risk scores (PRS) in our sample based on SNPs located at genes belonging to brain co-expression modules determined by the CommonMind Consortium (Fromer et al., Nature Neurosci 2016). PRS were generated using the clumping procedure of PLINK, considering several different thresholds to select SNPs from the discovery sample. In order to test if any specific module increased risk to schizophrenia more than expected by their size, we generated up to 10,000 random permutations of the same number of SNPs, matched by frequency, distance to nearest gene, number of SNPs in LD and gene density, using SNPsnap.

**Results:** As expected, most modules with enough number of independent SNPs belonging to them showed a significant increase in Nagelkerke's R2 in our case-control sample after the addition of the module-specific PRS in a logistic regression model. Our permutation strategy revealed that most modules did not show an excess of risk, measured by increase in Nagelkerke's R2, in comparison to equal number of SNPs with similar characteristics. But one module, M2c from Fromer et al., remained highly significant after multiple tests' correction. Reactome pathways analysis revealed an over-representation of genes involved in "Neuronal System" and "Axon guidance" among genes from this module. Using the same protocol, we detected that the 84 genes from the neuronal system pathway at this module, representing less than 6% of the genes from the module, explained a higher level of risk than expected. "Voltage-gated Potassium channels" and "Neurexins and neuroligins" are overrepresented among the Neuronal System genes from module M2c.

**Discussion:** Here, we show that, in spite of the high polygenicity of schizophrenia, it is possible to identify gene sets contributing disproportionately to total risk, as it was the case for the M2c module from Fromer et al. These authors have previously reported that the M2c module was enriched in GWAS signals, as well as CNVs and rare variants associated with schizophrenia. Therefore, this module shows a disproportionately contribution to schizophrenia risk.

Study supported by Grant PI14/01020 from Instituto de Salud Carlos III, Ministry of Health, Spanish Government.

### S188. DYSREGULATION OF CIRCULAR RNA EXPRESSION IN SCHIZOPHRENIA OBSERVED IN POSTMORTEM DORSOLATERAL PREFRONTAL CORTEX

Ebrahim Mahmoudi*[,1], Chantel Fitzsimmons[1], Michael Geaghan[1], Murray Cairns[1]
[1]*School of Biomedical Sciences & Pharmacy, University of Newcastle*

**Background:** The last few years has witnessed the emergence of a novel class of long non-coding RNA known as circular RNA (circRNA). These molecules are characterised by their circularity formed through the back splicing of 3' and 5' ends of transcript segments produced by one or more of its exons. CircRNAs function as transcriptional modulators, microRNA regulators, as well as template for translation. In our current study, we profiled circRNA expression in post-mortem brain samples from Schizophrenia (SZ) and control subjects using next-generation sequencing technology to discover the association of these novel RNA molecules with the pathogenesis of SZ.

**Methods:** Total RNA from cerebral cortex (BA46) of 17 SZ patients and 18 healthy controls were subjected to ribosomal RNA depletion and then RNase R treatment to further deplete linear RNA and enrich for exonuclease resistant circRNA transcripts. Sequencing libraries were constructed using Illumina TruSeq RNA Library Prep Kit (LT) (150 cycles) and sequenced by an Illumina NexSeq500. Sequencing data was analysed by the CIRCexplorer2 pipeline to identify circRNA transcripts. To validate the sequencing findings, real-time PCR was performed using outward primers sets designed to specifically amplify circular transcripts.

**Results:** We discovered a large number of distinct circRNAs (95,212), many of which were highly expressed throughout the cohort. Surprisingly, a large proportion (52%) of the identified circRNAs sequences were novel or not previously reported. Differential expression analysis suggested that there was substantial alteration in circRNA expression in SZ. More than two thirds of these molecules displayed decreased expression, whereas the remainder were upregulated. Functional annotation of the host genes was significantly enrichment for terms-related to neurobiology and neurocognitive impairment including clusters such as neurogenesis, differentiation and synapse. Many of these circRNAs were also predicted to interact with miRNAs, supporting a potential miRNA sponging function for these circRNA.

**Discussion:** RNA sequencing in the human postmortem DLPFC revealed dysregulation of circRNA expression in schizophrenia. This alteration was characterized by a substantial decrease in circRNA expression in the disorder. Bioinformatic predictions of circRNA interaction suggest they function as miRNA regulators and may have a broader role in etiology or pathophysiology of the disorder.

### S189. CARVING A MORE SPECIFIC SUBTYPE OF SCHIZOPHRENIA FOR GENETIC STUDIES: SPORADIC SCHIZOAFFECTIVE BIPOLAR TYPE

Nicolaas van der Merwe*[,1], Maria Karayiorgou[2], René Ehlers[1], Johannes Roos[1]
*[1]University of Pretoria; [2]Columbia University New York*

**Background:** Schizophrenia is a heterogeneous group of disorders. The familial-sporadic distinction has been considered under a range of genetic models. Research supports a strong association of de novo copy mutations with sporadic schizophrenia. The aim of the study was to determine a more homogenous phenotype for genetic research via comparison of various clinical and socio-demographic variables in familial and sporadic schizophrenia.
**Methods:** A cross-sectional observational design was used. This study included 384 participants with schizophrenia/schizoaffective disorder from an Afrikaner founder population in South Africa that previously participated in genetic research. A comprehensive data capturing sheet was completed from a pre-existing database that contains information obtained from the Diagnostic Interview for Genetic Studies, chronological clinical summary reports and additional sources of information. The study protocol was approved by the Research Ethics Committee from the Faculty of Health Sciences at the University of Pretoria. Logit models were fitted using the backward elimination procedure to investigate relationships where the dependent variable was binary. Odds ratio estimates were calculated for independent variables that were significant in the model. The Kruskal-Wallis test was conducted to compare the means of groups. For cases where there were significant differences a post-hoc test with a Bonferroni correction was done to determine which groups differ significantly.
**Results:** There were 214 familial and 170 sporadic subjects. 279 had a diagnosis of schizophrenia, 66 schizoaffective, bipolar type and 39 schizoaffective disorder, depressive type. 242 were male and 142 female. The age at onset of the primary psychiatric diagnosis, season of birth, co-morbid diagnoses, symptomatology, suicidality history and marital status weren't significantly different when considering the combined schizophrenia/schizoaffective disorder group and its relationship to familiality. Early deviant behaviour was however decreased in the sporadic group. These findings were replicated when analysing schizophrenia independently from schizoaffective disorder. The sporadic schizoaffective disorder, bipolar type did however have a significantly lower age at onset (mean 20.18 versus 25.07 years), 8.8 times more hallucinations, 6.6 times more odd behaviour before the age of 10 and were 2.8 times more likely to be single. The bipolar type also had 2.9 times more suicide attempts as opposed to ideation. This finding wasn't statistically significant. The sporadic schizoaffective group overall was 2.2 times more likely to abuse substances. The depressive type didn't differ significantly with regards to age at onset, season of birth, co-morbidities, early deviant behaviour, symptomatology, suicidality or marital status.
**Discussion:** The combined schizophrenia/schizoaffective group didn't differ significantly, nor did schizophrenia and schizoaffective disorder depressive type when analysed independently. The sporadic schizoaffective bipolar type differed significantly on multiple important variables that suggest a poorer prognosis and increased disease severity. The sporadic schizoaffective bipolar type forms a more homogenous group, with genetic studies hinting at relatively specific genetic risk factors. Studies elucidating the genetic architecture of this group could prove invaluable in clarifying the aetiology of schizophrenia.

### S190. SCHIZOTYPY & SUICIDALITY: A MENDELIAN RANDOMISATION ANALYSIS

Kirstie O'Hare*[,1], Richard Linscott[1], Tony Merriman[1]
*[1]University of Otago*

**Background:** Subclinical psychotic symptoms, known as schizotypy, predict concurrent and future suicidal ideation and acts. Some suggest this relationship reflects the influence of shared environmental risk factors, or that schizotypy and suicidality are both non-specific indicators of severity of psychopathology. However, this artefactual explanation does not account for the heritability of environmental risk factors, the link between schizotypy and more severe expressions of suicidality, or contemporary theories of suicidality.
**Methods:** We tested whether schizotypy has a direct (causal) effect on the development of suicidal thoughts using a Mendelian randomisation analysis to avoid problems of reverse causality, confounding, and measurement error associated with traditional observational studies. In Mendelian randomisation analyses, genetic variants are used as a proxy measure for a phenotype in order to make causal inferences about the effect of the phenotype on an outcome. We used a schizophrenia gene risk score (GRS), a measure of schizophrenia liability, as a proxy measure for schizotypy. Participants ($n = 4767$) were part of the Philadelphia Neurodevelopmental Cohort, a publicly available resource designed to assess behavioural and biological factors contributing to mental illness in young adults (aged 8–21). Regression analyses were used to test relationships.
**Results:** Schizotypy was found to be a strong predictor of both passive ($OR = 1.84$, $p < .001$) and active ($OR = 2.69$, $p < .001$) suicidal ideation. No relationship was found between the schizophrenia GRS and schizotypy when analysed in separate ethnic groups to adjust for population stratification (European American; $B = .18$, $p = .708$, African American; $B = .086$, $p = .303$). No relationship was found between the schizophrenia GRS and passive ($OR = .97$, $p = .721$) or active ($OR = .99$, $p = .778$) suicidal ideation.
**Discussion:** The hypothesis that there is a causal relationship between schizotypy and suicidality was not supported, though it is unclear if this is due to the schizophrenia GRS being a poor proxy for schizotypy, or a true absence of a causal relationship. Understanding causal risk factors for suicidality is a key area of research and future research should continue to address this using genetically-sensitive designs.

### S191. INVESTIGATION OF THE PREVALENCE OF COPY NUMBER VARIANT SYNDROMES IN A LARGE SCHIZOPHRENIA COHORT

Venuja Sriretnakumar*[,1], Clement Zai[2], James Kennedy[3], Joyce So[4]
*[1]University of Toronto; [2]Centre for Addiction and Mental Health; [3]CAMH, University of Toronto; [4]University Health Network, University of Toronto*

**Background:** Many rare genetic syndromes are known to phenotypically manifest with psychiatric symptoms that can be indistinguishable from primary psychiatric disorders. While the majority of ongoing research in psychiatric genetics has been dedicated to the identification and characterization of genes involved in primary psychiatric disorders, there has been a lack of research to determine the extent to which rare genetic variants contribute to the overall psychiatric disease load. In our study, we aim to investigate the prevalence of clinically well-characterized pathogenic copy number variant (CNV) syndromes that are associated with neuropsychiatric phenotypes in a large schizophrenia patient cohort.
**Methods:** DNA from 348 schizophrenia patients recruited at the Centre for Addiction and Mental Health (CAMH) (Toronto, Canada) was run on the Affymetrix SNP Array 6. 0. CNVs were called using two algorithms (Canary Software and PennCNV) for deletions >200 kb and duplications >500 kb. CNVs called by both algorithms were included in further analysis. All CNVs were individually assessed to determine overlap with known, clinically well-characterized CNV syndromes with the use of the UCSC Genome Browser, DECIPHER GRCh37, and GeneReviews® databases.
**Results:** A total of 861 deletions and 171 duplications were called on 348 schizophrenia patients. In-depth analysis revealed a total of 16 schizophrenia patients with significant deletions. Microdeletions associated with known syndromes that were identified include: 16p11.2-p12.2 (n=1),

16p13.11 (n=3), 17p11.2 (n=2), 22q11.2 (n=5), 1p36 (n=4), and 5q35.3 (n=1). Analysis for pathogenic microduplications is ongoing.

**Discussion:** We observed a greater than expected number of syndromic microdeletions amongst the schizophrenia cohort (16/348, 4.6%), particularly CNVs already hypothesized or known to be associated with neurodevelopmental disorders. Screening for these rare genetic disorders could lead to better understanding of the pathophysiology of psychiatric disorders, as well as the prevalence of these syndromic CNVs within various psychiatric population subtypes. Correctly identifying syndromic CNVs within psychiatric populations can improve patient prognosis. Further analyses will be undertaken to define specific genes contained within the implicated CNV regions to better characterize potential genetic effects on the phenotypic presentation of SCZ patients.

### S192. AKT-MTOR SIGNALING PATHWAY IS DOWNREGULATED IN SCHIZOPHRENIA

Radhika Chadha*,[1], James Meador-Woodruff[1]
[1]*The University of Alabama at Birmingham*

**Background:** Cognitive deficits are observed in many schizophrenia (SZ) patients. The AKT-mTOR pathway is an important signaling cascade associated with long term plasticity and thus may contribute to cognitive dysfunction. This pathway is tightly regulated by differential phosphorylation of key proteins. AKT is a serine-threonine kinase which regulates critical cellular functions like cell survival, proliferation and growth. Prior literature suggests reduced expression of AKT in SZ. mTOR is a kinase that forms 2 distinct complexes- mTORC1 and mTORC2. mTORC1 consists of mTOR, Raptor, GβL, PRAS40 and Deptor proteins. It plays an important role in actin dynamics and acts downstream of AKT. mTORC2 consists of mTOR, Rictor, GβL, Protor, mSin1 and Deptor proteins. It facilitates ribosome biogenesis and protein translation and acts upstream of AKT. Abnormalities in the mTOR complexes can contribute to dysregulated protein synthesis, which has been implicated in SZ. Alterations in the AKT-mTOR cascade, including abnormal phosphorylation of AKT and expression of mTOR complex components, have been suggested as potential mechanisms underlying SZ pathophysiology. Therefore, we hypothesized that protein levels and/or phosphorylation status of key molecules in the AKT-mTOR pathway are altered in SZ.

**Methods:** We used post mortem dorsolateral prefrontal cortex (DLPFC) from 22 matched pairs of SZ and comparison subjects for this study. Using western blot analysis, we measured protein levels of AKT, mTOR, GβL, Raptor, phosphorylated AKT (at S473 & T308) and phosphorylated mTOR (at S2448 & S2481).

**Results:** We found decreased levels of AKT, phosphorylated AKT (at both S473 and T308) and GβL. We also found that the ratio of phosphorylated mTOR (at S2448) to total mTOR was decreased.

**Discussion:** AKT requires phosphorylation at both S473 and T308 for complete activation. It can further regulate the formation of mTORC1 through Rheb. AKT is phosphorylated at S473 by active mTORC2. mTOR phosphorylation at S2448 is required for its activation in both complexes. Our findings that total AKT and its phosphorylated forms are decreased in conjunction with reduced expression of GβL and the ratio of phosphorylated mTOR (at S2448) to total mTOR suggest that the AKT-mTOR signaling pathway is downregulated in SZ DLPFC. Given the importance of this pathway in synaptic plasticity via its regulation of protein translation and cytoskeletal organization, these abnormalities may represent a mechanism underlying cognitive dysfunction in SZ. Future studies will investigate the expression levels of proteins in mTOR complexes and will determine the integrity of mTORC1 and mTORC2 complex formation in SZ.

### S193. EX VIVO SIGNATURE OF PSYCHOSIS AND TREATMENT RESPONSE IN PATIENT-DERIVED NEURONS

Rakesh Karmacharya*,[1], Bradley Watmuff[1], Annie Kathuria[1], Bangyan Liu[1]
[1]*Harvard University*

**Background:** Postmortem studies in schizophrenia show well-replicated neuronal differences in the prefrontal cortex (PFC), specifically showing lower dendritic spine density in upper-layer cortical pyramidal neurons. Animal models that recapitulate features of psychosis also show lower dendritic spine density and synapse number in the PFC, with a more pronounced effect in upper-layer cortical neurons. Furthermore, the decrease in dendritic spines and synapses in animal models have been shown to be reversible with antipsychotic treatment. Results from postmortem brains, animal models and in vitro rodent cultures provide a strong impetus to test the hypothesis that dendritic spine biology plays an important role in the biology of schizophrenia and in mediating the effects of antipsychotic medications.

**Methods:** To extend these findings, we studied cortical neurons generated from subjects with schizophrenia. We reprogrammed induced pluripotent stem cells (iPSCs) from human subjects with schizophrenia and from matched healthy controls. We differentiated human iPSCs along the forebrain lineage to generate mature cortical neurons. We developed a robust experimental approach to delineate and quantify spines in the dendrites as well as methods to outline and measure the spines in order to classify the different spine types. We also developed methodology for functional characterization of individual neurons using calcium imaging in the cortical neuron cultures.

**Results:** We found that cortical neurons generated from the iPSCs of schizophrenia patients had a lower density of dendritic spines when compared to cortical neurons generated from the iPSCs of healthy control subjects. We also delineated the different composition of spine types in cortical neurons from schizophrenia patients when compared to those from healthy control subjects. In cortical neurons from schizophrenia subjects, we found that clozapine exposure in vitro leads to a robust increase in dendritic spine density.

**Discussion:** We found that cortical neurons from iPSCs of schizophrenia subjects recapitulate the dendritic spine differences reported in postmortem brains of schizophrenia subjects. Moreover, we found that human cortical neurons from schizophrenia subjects show increased dendritic spine density when exposed in vitro to clozapine. The ability to delineate cellular features related to disease biology in iPSC-derived neurons opens the door to understand the pathophysiology of schizophrenia and lay the foundations for the development of novel therapeutics.

### S194. INVESTIGATING PERIPHERAL MICRORNA-MRNA INTERACTIONS IN SCHIZOPHRENIA

Michael Geaghan*,[1], Murray Cairns[1]
[1]*University of Newcastle*

**Background:** Schizophrenia is a severe neuropsychiatric disorder, characterised by positive and negative symptoms, and cognitive deficits. High throughput technologies such as microarrays, and more recently next-generation sequencing have identified numerous genetic variants and transcriptional signatures associated with schizophrenia. Over the last decade, microRNAs (miRNAs) have been found differentially expressed in both peripheral and post-mortem grey matter tissue in schizophrenia, and three genome-wide significant schizophrenia-associated variants occur within two miRNA loci – MIR137 and MIR548AJ2. These small, non-coding RNAs are potent regulators of translation, can target a wide variety of transcripts, and are therefore of particular interest in polygenic disorders such as schizophrenia.
**Methods:** We obtained total RNA isolated from peripheral blood mononuclear cell (PBMC) samples from the Australian Schizophrenia Research Bank (ASRB). The samples included 36 individuals with schizophrenia and 15 healthy controls. We utilised small RNA and mRNA sequencing technology to examine both the miRNA and mRNA expression profiles of these sample. Raw reads were aligned to the human genome (hg38), annotated, counted and analysed for differential expression using an open source software pipeline. Correlations between miRNA and mRNA expression were found and matched to predicted TargetScan miRNA-mRNA interactions using the miRComb R package. Ingenuity Pathway Analysis and Gene Set Enrichment Analysis were performed to identify pathways and gene ontologies enriched for differentially expressed genes.
**Results:** 35 miRNAs and 97 genes were differentially expressed (FDR<0.1); most miRNAs (21 out of 35) were downregulated, while the vast majority of mRNAs (80 out of 97) were upregulated. When males and females were analysed separately, we found 14 miRNAs and 365 genes differentially expressed in males, while females only showed 7 miRNAs and 1 gene (NRCAM – neuronal cell adhesion molecule) differentially expressed. Several miRNAs in males were found to significantly correlate with differentially expressed genes, including miR-1271-5p with schizophrenia candidate gene DGCR2. Furthermore, many differentially expressed genes and miRNAs have previously been linked to schizophrenia and neuronal function. Among males, several immune- and inflammation pathways were enriched for differentially expressed genes. Interestingly, while upregulated genes were enriched for immune-related gene ontologies, downregulated genes were enriched for gene ontologies relating to development.
**Discussion:** These results contribute to a growing body of evidence that suggest peripheral miRNA and mRNA expression is altered in schizophrenia. We identify a general downregulation of miRNAs and upregulation of mRNAs in peripheral tissue in schizophrenia. Several significant correlations between miRNAs and mRNAs previously linked to schizophrenia and brain function suggest potential miRNA-mRNA interactions that may be significant for disease pathophysiology.

### S195. ELECTRORETINOGRAPHIC INDICES OF PHOTORECEPTOR, BIPOLAR, AND GANGLION CELL FUNCTIONING DIFFERENTIATE PEOPLE WITH SCHIZOPHRENIA FROM THOSE WITH MAJOR DEPRESSION AND HEALTHY CONTROLS

Docia Demmin*[,1], Matthew Roché[2], Roni Netser[1], Steven Silverstein[1]
*[1]Rutgers University; [2]New Jersey City University*

**Background:** The retina is part of the CNS and provides a window into brain structure and function that has been useful in examining schizophrenia and other psychiatric disorders.
**Methods:** In this ongoing study, we are using flash electroretinography (fERG) to compare retinal cell functioning in schizophrenia (n = 25) and major depressive disorder (MDD; n = 18, to date), both relative to psychiatrically healthy controls (n = 25). Data were averaged over both eyes and collected under both light- and dark-adapted conditions. The primary variables of interest were a-wave activity (reflecting photoreceptor response), b-wave activity (reflecting primarily bipolar cell activity), and photopic negative response (PhNR; reflecting ganglion cell activity).
**Results:** On light-adapted (photopic) tests, schizophrenia patients demonstrated significantly weaker cone and bipolar cell responses than the MDD and healthy control groups (ds = .76 to 1.25). On dark-adapted (scotopic) tests, all groups demonstrated a linear increase in photoreceptor and bipolar cell response with increases in stimulus intensity, but the rate of response gain per unit of intensity increase was significantly weaker for schizophrenia patients than for the other groups (ds = .84 to 1.11). Significant group differences were also found in PhNR amplitude, with the schizophrenia group demonstrating a weaker PhNR (measured at 72 ms post-stimulus presentation) as compared to the healthy control group (d = .50). In the MDD group, the minimum PhNR amplitude occurred significantly earlier than in either of the other two groups (ds =.80 to .92).
**Discussion:** These data confirm abnormal retinal cell functioning in schizophrenia patients receiving treatment. Our finding of normal retinal waveform amplitudes in MDD is consistent with a prior report of normalized fERG amplitudes after antidepressant treatment (Fornaro et al., 2011, J Affective Disorders), but inconsistent with another report showing abnormal values in treated MDD patients (Hébert et al., 2017, Prog Neuropsychopharmacol Biol Psychiatry). Our finding of enhanced PhNR implicit time in MDD is consistent, however, with evidence of enhanced amplitudes in untreated MDD patients in Fornaro et al. (2011). Further studies, using a range of fERG parameter values, are necessary to determine trait and state effects on retinal function in MDD, and which of these effects may overlap or contrast with what is observed in schizophrenia.

### S196. ASSOCIATION BETWEEN INTRACELLULAR INFECTIOUS AGENTS AND SCHIZOPHRENIA IN KOREA

Young-Joon Kwon*[,1], Won-Myong Bahk[2], Bo-Hyun Yoon[3], Duk-In Jon[4], MoonDoo Kim[5], Beomwoo Nam[6], Eunsung Lim[7], Sung-Yong Park[8]
*[1]Soonchunhyang University Chunan Hospital; [2]Yeouido St. Mary's Hospital, The Catholic University of Korea; [3]Naju National Hospital; [4]Hallym University; [5]Jeju National University; [6]Konkuk University; [7]Shinsegae Hospital; [8]Keyo Hospital*

**Background:** A number of studies have reported association between Toxoplasma gondii (T. gondii) and Chlamydia infection and the risk of schizophrenia. The aim of the present study was to compare the prevalence of T. gondii and Chlamydia infection between the schizophrenia and normal control subjects and to compare the clinical features between seropositive and seronegative Korean schizophrenia patients.
**Methods:** The rate of serum reactivity to T. gondii, Chlamydia trachomatis (C. trachomatis), Chlamydia pneumonia in 96 schizophrenia and 50 control subjects was investigated using enzyme-linked immunosorbent assay and indirect fluorescent antibody technique. The clinical symptoms of the schizophrenia patients were scored with Positive and Negative Syndrome Scale and a comparative analysis was carried out.
**Results:** A significant positive association between immunoglobulin G (IgG) antibodies to T. gondii and C. trachomatis in schizophrenia was found, and the odds ratio of schizophrenia associated with IgG antibody was found to be 3.22 and 2.86, respectively. The Toxoplasma-seropositive schizophrenia patient had higher score on the negative subscale N1 and N7 and general psychopathology subscale G13, while C. trachomatis-seropositive schizophrenia patient had higher score on the general psychopathology subscale G10.
**Discussion:** The results from the present study suggest significant association between T. gondii, C. trachomatis infection and schizophrenia. In future, further studies are needed to elucidate the correlation between the two types of infection and schizophrenia.

### S197. OBSTETRIC COMPLICATIONS, NEUROCOGNITION, AND SCHIZOPHRENIA

Bjorn Rund*[,1], Charlotte Teigset[1], Christine Mohn[1]
*[1]University of Oslo*

**Background:** Schizophrenia is a disorder with a heterogeneous genetic and neurobiological background that influences early brain development. The symptoms is the behavioural outcome of deviations in early neurodevelopment, including prenatal insults such as obstetric complications (OC). OC have been linked to an increased risk for schizophrenia in offspring, especially in early-onset schizophrenia (EOS). Extensive cognitive deficits occur in EOS, whereof executive function is one of the best documented. Cognitive dysfunction reflects underlying abnormalities in the brain neurodevelopment, and is considered to be an intermediate variable between OC and schizophrenia. Our research group (Teigset et al, 2016) is the only study that has investigated the relationship between OC and cognition in EOS. This study aimed to examine the frequency of OC in EOS compared to controls, and also investigate the relationship between OC and neurocognitive dysfunction. In the present presentation we will focus upon executive function and report the findings when comparing the same sample of patients and controls as in the Teigset et al study.
**Methods:** Nineteen EOS patients and 53 healthy controls were tested with the MATRICS Consensus Cognitive Battery(MCCB), and two tests for assessment of executive functioning. The selected subtests for measuring executive function were the D-KEFS Color Word Interference Test (Stroop) and the Wisconsin Card Sorting Test. WCST assesses perseverative responses and failure to maintain set, and the Stroop assesses time in seconds for completing the Inhibition and Switching conditions. The cognitive measures were combined with data from the Norwegian Birth Registry (NMBR). Information on OC was collected from the NMBR containing information about all births in Norway, including information about maternal health before and during pregnancy, and any complications arising during pregnancy or birth. The registry includes information about medication during pregnancy, labor interventions, birth complications, maternal complications after birth, whether this was a live birth, any diagnoses in the child or evidence of congenital abnormalities.
**Results:** Group differences in OC were studied with Student's t-tests and Chi-square tests. The association between OC and cognitive function were studied using linear regression analyses. The results indicated no group differences in OC in EOS and healthy controls. However, a shorter gestational length in the EOS group led to significant decreases in the overall neurocognitive composite score (MCCB), in processing speed and in the two executive function tasks.
**Discussion:** Our findings indicate that a shorter gestational length did not increase the risk for developing EOS, but was significantly associated with the cognitive difficulties in this group. In particular, executive functioning were affected, a finding in line with those of Brown et al (2009), showing that prenatal infections were associated with impaired executive function. Interestingly, reductions in neurocognitive performance among those exposed to OC was less extensive in the healthy control group with the same labor-conditions, which may indicate a greater effect of OC on neuropsychological development in schizophrenia. In conclusion, gestational length does not increase the risk for developing EOS, but significantly affects the cognitive difficulties - particularly executive function - seen among cases.
**References:**
1. Brown et al. (2009) Am J Psych, 166: 683–690.
2. Teigset et al. (2016). Psych Res, 244: 78–85.

### S198. PRE-ADOLESCENT BRAIN STRUCTURE: THE INTERPLAY BETWEEN GENETIC VULNERABILITY FOR SCHIZOPHRENIA AND CORTISOL LEVELS

Koen Bolhuis*,[1], Philip R. Jansen[2], Hanan El Marroun[1], Manon H.J. Hillegers[3], Henning Tiemeier[1], Steven A. Kushner[1]
[1]*Erasmus Medical Center;* [2]*Erasmus Medical Center, Vrije Universiteit Amsterdam;* [3]*Erasmus Medical Center, University Medical Center Utrecht*

**Background:** Schizophrenia is a highly heritable disease, mediated through a combination of common and rare genetic variants. In addition to genetic risk, several putative environmental risk factors have been studied in the context of schizophrenia. It has been suggested that the genetic effects on the etiology of schizophrenia may be of limited explanatory power if not viewed in the context of interaction with environmental stressors. Here, in a pre-adolescent population-based sample, we used polygenic risk scores from a case-control discovery sample of the Psychiatric Genomics Consortium as indicators of genetic vulnerability to schizophrenia. In addition, hair cortisol levels were obtained as a naturalistic quantitative metric of long-term physiological stress. We examined whether cortisol levels moderated the relationship between schizophrenia polygenic risks scores and pre-adolescent brain structure.
**Methods:** This study was embedded in the Generation R Study, a prospective birth cohort from the Netherlands. Polygenic risk scores for schizophrenia were calculated in children of European ancestry only. P-value thresholds for inclusion of genetic variants in the polygenic risk score varied between $P < 0.001$ and $P < 1$. Hair cortisol was collected when the children were approximately 6 years old. At age 9 years, children underwent a magnetic resonance imaging (MRI) procedure to assess volumetric brain measures. After genetic and neuroimaging quality control procedures, the final sample consisted of 522 participants. Linear regression models were conducted to examine the associations between schizophrenia polygenic risk scores, hair cortisol levels, and brain volumes. All analyses were adjusted for age, sex, hair color, hair product use and four genetic principal components.
**Results:** Schizophrenia polygenic risk scores were not associated with hair cortisol levels (P-value threshold [PT] < 0.001, $\beta = -0.03$, 95% confidence interval [CI] -0.11 – 0.05, $P = 0.441$), cortical grey matter volume (PT < 0.001, $\beta = -0.03$, 95% CI -0.11 – 0.05, $P = 0.457$) or cerebral white matter volume (PT < 0.001, $\beta = -0.04$, 95% CI -0.12 – 0.04, $P = 0.356$). Higher schizophrenia polygenic risk scores were associated with lower total ventricle volume (PT <0.001, $\beta = -0.10$, 95% CI -0.19 – -0.02, $P = 0.022$), including when mutually adjusted for total brain volume. Notably however, hair cortisol exhibited a positive interaction with schizophrenia risk scores in predicting total ventricle volume (PT < 0.001, $\beta = 0.10$, 95% CI 0.01 – 0.18, $P = 0.027$), i.e. higher schizophrenia polygenic risk score and higher hair cortisol levels were associated with increased ventricle volumes. Hair cortisol was not independently associated with total ventricle volume (PT < 0.001, $\beta = -0.05$, 95% CI -0.14 – 0.03, $P = 0.215$).
**Discussion:** Elevated hair cortisol levels, a biological index of long-term stress, moderated the association between genetic vulnerability to schizophrenia and total cerebral ventricle volume among pre-adolescents. These findings underscore the importance of the interplay between genes and environment in shaping brain development. Using a polygenic risk score for schizophrenia, we provide novel, albeit preliminary, evidence for gene-environment interaction between genetic risk for schizophrenia and environmental stressors in the general pre-adolescent population. This may help to elucidate underlying etiologies and possible early interventions for the psychosis spectrum.

### S199. ENHANCED OLFACTORY IDENTIFICATION IN ADOLESCENTS WITH PSYCHOTIC EXPERIENCES

Eleanor Carey*,[1], Niamh Dooley[1], Erik O'Hanlon[1], Ian Kelleher[1], Mary Cannon[1]
[1]*Royal College of Surgeons in Ireland*

**Background:** The olfactory system has a widely distributed anatomical network reaching both cortical and subcortical structures (Milardi et al., 2017). Olfactory dysfunction has been associated with schizophrenia (Moberg & Turetsky, 2003), where deficits in odour identification (Seidman et al., 1997), odor detection threshold sensitivity (Serby et al., 1990) and odour memory (Wu et al., 1993) can be seen early in the course of the disorder and persist with illness duration (Rupp, 2010). This olfactory dysfunction has been correlated with cognitive deficits, including language (Corcoran et al., 2005), verbal and non-verbal memory (Moberg et al., 2006) and tests of emotional recognition (Goudsmit et al., 2003) in schizophrenia. Neuroanatomically, areas of the temporal lobe, including the superior temporal gyrus, hippocampus and amygdala have all been implicated in the dysfunction. It is not known whether olfactory changes can be detected in the broader extended psychosis phenotype.
**Methods:** The current research focuses on a community-based sample of young adolescents aged 11–13 (N= 140) recruited from schools in the Dublin and Kildare areas of Ireland, These adolescents were assessed for psychotic symptoms using the psychosis section of the Schedule for Affective Disorders and Schizophrenia and also completed the Brief Smell Identification Test (BSIT), derived from the University of Pennsylvania Smell Identification Test (UPSIT),as part of a neuropsychological assessment. The BSIT is a self-administered 12-item test of olfactory functioning, containing common odours (eg lemon, chocolate, and smoke) and participants were required to choose one of four multiple-choice answers.
**Results:** Performance on the BSIT was compared between participants who reported any psychotic experiences (PE) (N=71), and those who did not (N=69). We examined total score and scores for individual smell types, using ANOVA, and co-varied for age and gender.
**Results:** A significant group difference was found when both age and gender were co-varied for, in which the PE group performed significantly better on the BSIT (F=5.56, p= 0.02), and one of the individual twelve odours ("paint- thinner") of the BSIT also was significantly better identified by the PE group (F=7.53, p=0.007). When examined in terms of correlations with psychotic symptoms, BSIT scores were found to significantly correlate with Grandiosity, one of the eight categories of the Adolescent Psychotic-Like Symptoms Screener (APSS) (p=0.004).
**Discussion:** Moberg et al. (2013) report that positive symptoms of psychosis may be moderating our results, where it was hypothesized that this early olfactory hypersensitivity is due to increased vigilance of external and internal stimuli. Corcoran et al. (2005) similarly found that the presence of features associated with mania, such as grandiosity, was associated with better outcomes in smell identification tests.
We hope to extend our analysis to search for neuroanatomical and neuropsychological correlates of these olfactory performance scores in young people with and without psychotic symptoms, as well as looking more in-depth at positive symptoms reported by the PE group and their possible associations with this increased hypersensitivity in olfactory identification. The current research is hoped to capture some of the earliest olfactory changes associated with psychosis vulnerability as a possible biomarker. The study employs a novel participant cohort whom are considered an extended psychosis phenotype and at this point only exhibit psychotic symptoms, but remain at an increased risk of the development of psychosis in adulthood.

### S200. HYPOVITAMINOSIS D IN SCHIZOPHRENIA: PREVALENCE AND ASSOCIATED CLINICAL CHARACTERISTICS

Rahma Nefzi*[,1], Amine Larnaout[1], Hanen Ben Ammar[1], Emira Khelifa[1], Amina Aissa[1], Zouhaier El Hechmi[1]
[1]*Razi Hospital*

**Background:** Schizophrenia is an invalid and severe neurodevelopmental disorder. The implication of vitamin D in the etiopathogenesis of schizophrenia shows through the activation of cellular and inflammatory pathways. It is especially vitamin D deficiency that has been associated with schizophrenia. It is within this framework that this study aims to explore the relationship between vitamin D levels and the clinical characteristics in a cohort of Tunisian patients with schizophrenia.
**Methods:** A cross-sectional and retrospective descriptive study was conducted at the "F" psychiatry department at the Razi Hospital, Manouba over a twelve-month period from June 1st, 2015 to May 31st, 2016, including 80 patients with schizophrenia in period of clinical remission. The evaluation focused on sociodemographic and clinical characteristics. A dosage of vitamin D was performed.
**Results:** The patients had an average age of 42.5 years and 70% were male. The average vitamine D level was 10,57ng/ml ±5,9. 49% of patients had vitamin D insufficiency (between 10 and 30 ng/ml) and 51% had vitamin D deficiency (<10 ng/ml). Vitamin D levels had not been affected by the clinical characteristics of the disease. A negative correlation with the total score of the negative scale (p <0.001) as well as with the severity item of the clinical global impression scale (p = 0.01) were found.
**Discussion:** A large number of research studies in immunogenetics and molecular biology have highlighted the involvement of vitamin D in the etiopathogenesis of schizophrenia through its role in the ontogenesis of dopaminergic systems and also through its intervention in the processes of neuro-protection, immunomodulation and the reduction of oxidative stress. In addition, it has been established that people with psychotic disorders have a high prevalence of vitamin D deficiency, but the correlates and relevance of this deficiency remain unclear.

### S201. RELATION BETWEEN PSYCHOTROPIC DRUGS AND SEIZURE THRESHOLD IN ELECTROCONVULSIVE THERAPY

Seung Hyun Kim*[,1], Jung-Seo Yi[2], Su-Hyuk Chi[3], Hyun-Ghang Jeong[3]
[1]*Korea University, Guro Hospital College of Medicine;* [2]*Hallym University College of Medicine, Kangnam Sacred Heart Hospital;* [3]*Korea University, Guro Hospital*

**Background:** Electroconvulsive therapy (ECT) is the most popular way to stimulate brain for achieving therapeutic effects. The therapeutic effect of ECT results from the induction of a generalized seizure. The minimal amount of electrical energy needed to induce seizures is known as the seizure threshold (ST). It is commonly believed that treatment efficacy is related to stimulus dose relative to ST, but higher stimuli usually also increase unwanted side effects. Therefore, ST is an important issue in conducting ECT. Most patients including schizophrenics undergoing ECT take concomitant psychotropic drugs, but little information is available on how these drugs affect ST. Our study aimed to analyze the relationship between ST and psychotropic drugs in patients treated with ECT.
**Methods:** We retrospectively reviewed the medical charts of 43 patients who received ECT at Korea University Guro Hospital between February 2009 and June 2015. Patients with a history of seizure disorders or other medical emergent conditions were excluded. A total of fifty-eight subjects received ECT during the study period. Patients were excluded if treatment was aborted due to side effects or any other reasons before the 10th session (n=12) because we intended to investigate the ST shift during the course of consecutive ECT sessions. We included 43 subjects in the final data analysis. Patients' psychiatric disorders were diagnosed according to the Diagnostic and Statistical Manual of Mental Disorders (DSM-IV-R) by at least two experienced psychiatrists. ECT was administrated with concurrent antipsychotics and antidepressants.67.4 percent of subjects were diagnosed as schizophrenia and 20.9 percent of subjects were diagnosed as major depressive disorder. We used stepwise multivariate correlation analyses for examining the associations between ST and psychotropic drugs. Data are presented as initial ST, the difference in ST between the first and 10th sessions (ΔST10th), and the mean difference in ST between the first and last sessions (mean ΔSTlast). We used chlorpromazine-equivalent dose for antipsychotics and fluoxetine-equivalent dose for antidepressants.

**Results:** Of the 43 patients included in the study, 20 were male, and the other 23 were female. The mean age of all participants was 41.44 years (SD=15.89). Patients were taking the following antipsychotics and antidepressants: clozapine (n=6), amisulpride (n=9), aripiprazole (n=5), olanzapine (n=18), risperidone (n=1), quetiapine (n=20), haloperidol (n=1), paliperidone (n=5), chlorpromazine (n=1), blonanserin (n=1), escitalopram (n=7), sertraline (n=1), mirtazapine (n=2), duloxetine (n=1), venlafaxine (n=3), amitriptyline (n=1), trazodone (n=1), bupropion (n=1). Participants took an average of 1.91 (SD=1.02, range 0–5) different psychotropic drugs during ECT. The mean number of types of antipsychotics and antidepressants used were 1.53 (SD=0.74, range 0–3) and 0.37 (SD=0.76, range 0–4), respectively. Multivariate regression analyses showed positive correlations between initial ST and the total chlorpromazine-equivalent dose of antipsychotics ($\beta$ = 0.363, $p < 0.05$). The total fluoxetine-equivalent dose of antidepressants was positively correlated to ΔST10th ($\beta$ = 0.486, $p < 0.05$) and mean ΔSTlast ($\beta$ = 0.472, $p < 0.01$).
**Discussion:** Our study elucidated possible effects of psychotropic drugs on ST in patients undergoing ECT. We revealed that larger doses of antipsychotics are associated with higher initial ST, whereas higher doses of antidepressants are associated with stronger shifts of ST during the course of treatment. We believe that our findings provide a basis for creating safer and more efficient ECT protocols.

### S202. EFFICACY OF LONG-TERM RESIDENTIAL TREATMENT FOR PERSISTENT MENTAL ILLNESS

Michael Knable*,[1]
[1]*Sylvan C. Herman Foundation*

**Background:** In the United States, the number of public and private psychiatric hospital beds has steadily declined in recent years, despite the lack of intensive intermediate care alternatives in the community. The design and implementation of intensive residential treatment programs are not currently guided by controlled studies, but these studies are necessary to determine the clinical and economic utility of such programs. We present clinical and outcome data on an initial sample of patients treated over the last 5 years.
**Methods:** Naturalistic, non-controlled assessment of symptomatic and functional outcome in an initial sample of young adults with persistent mental illnesses treated in a community-based residential program. Patients were treated with an individualized combination of modalities such as Illness Education and Management, Supported Employment, Individual, Group and Family Psychotherapies and Psychopharmacology. Standard clinical rating scales were used during the period of treatment and all discharged patients were contacted on an annual basis in order to complete a survey of clinical outcome.
**Results:** 101 patients had been admitted and treated since the facility opened in October 2011. Median age of the patients was 25 years, mean illness duration was 12.6 years, and the mean number of prior hospitalizations was 6.5. Diagnostic distribution was: 36.7% psychotic disorders, 27.7% unipolar mood disorders, 19.8% bipolar mood disorders, 7.9% autism spectrum disorders, and 7.9% post-traumatic stress disorder or other anxiety conditions. 37% of residents met criteria for personality disorders, the majority of which was borderline personality disorder. 42% of residents also met criteria for a substance use disorder in the year prior to admission. Ratings on the Multnomah Community Ability Scale improved by 16%, ratings on the Brief Psychiatric Rating Scale declined by 20% and ratings on the Montgomery-Asberg Depression Rating Scale declined by 37%. The average survey response rate after discharge was 59%. With regard to community engagement: 40.3% of current residents and 35.1% of discharged residents were competitively employed. 16.7% of current residents and 17.8% of discharged residents worked as volunteers, and 23.3% of current residents and 26.3% of discharged residents were attending school. A survey of dispositions revealed that: 49.7% of discharged residents were living independently, 14.9% were living with family, 2.2% were homeless, and 5.4% had died from suicide. The hospitalization rate declined from 0.84/year to 0.57/year before and after discharge.
**Discussion:** Long-term residential treatment for young adults with persistent mental illness results in improved symptomatic recovery, independent living, increased employment rates, and reduced hospitalizations.

### S203. COMPENSATORY COGNITIVE APPROACHES TO IMPROVING FUNCTIONING IN PSYCHOSIS: SYSTEMATIC REVIEW AND META-ANALYSIS

Kelly Allott*,[1], Kristi van-der-EL[2], Emma Parrish[3], Chris Bowie[4], Sean Kidd[5], Susan McGurk[6], Sarah Hetrick[7], Shayden Bryce[8], Matthew Hamilton[2], Eoin Killackey[1], Dawn Velligan[9]
[1]*Orygen, The National Centre of Excellence in Youth Mental Health;* [2]*Orygen, The National Centre of Excellence in Youth Mental Health, The University of Melbourne;* [3]*Harvard Medical School at Beth Israel Deaconess Medical Center;* [4]*Queen's University;* [5]*University of Toronto;* [6]*Boston University;* [7]*University of Auckland and Centre for Youth Mental Health, University of Melbourne;* [8]*Monash University;* [9]*University of Texas Health Science Center at San Antonio, School of Medicine*

**Background:** Cognitive impairments in domains such as attention, memory, processing speed and executive functions are a central feature of psychotic disorders that have significant negative consequences for daily functioning, including activities of daily living, social and vocational roles. Compensatory approaches aim to minimise the impact of cognitive impairment on daily functioning through the use of aids or strategies to reduce cognitive load, in much the same way as glasses reduce the impact of vision impairment. The primary treatment target is real world community functioning and functional capacity, rather than cognition. There is now a need to synthesise the available evidence in this field so that treatment recommendations and future research directions can be better informed. A large body of research into compensatory approaches to cognition in psychosis exists, but this has never been comprehensively synthesised. The aim of this systematic review and meta-analysis is to examine the effects of compensatory approaches for cognitive deficits in psychotic disorders on i) functional outcomes and ii) other outcomes such as symptoms and quality of life.
**Methods:** A systematic review and meta-analysis was conducted according to PRISMA guidelines. PsycINFO and MEDLINE electronic databases were searched from inception to October 2017 using multiple terms for 'psychosis', 'cognition' and 'compensatory'. All papers retrieved from this search were double-screened and final inclusion/exclusion was determine by consensus. Data were double-extracted and risk of bias rated by two independent authors. Meta-analysis only included randomised-controlled trials. Standardised Mean Differences (SMD) were calculated to produce a single summary estimate using the random-effects model with 95% Confidence Intervals using Comprehensive Meta-Analysis (CMA) software. When means or standard deviations were not reported in the original articles, SMDs were calculated from data provided by the study authors.
**Results:** 2192 articles were identified via electronic and manual searches. Forty-two papers describing 40 independent studies were included in the review: case studies (n=4), case series (n=2), uncontrolled single arm pilot studies (n=5), within-subjects designs (n=1), quasi-randomised trials (n=2), and randomised controlled trials (n=26). The types of compensatory interventions included environmental adaptation and supports, internal and external self-management strategies, and errorless learning. Compensatory interventions were associated with improvements in global functioning post intervention (N=1,449; SMD=0.506; 95%CI=0.347, 0.665; p<.001). Improvements in global symptoms (N=849; SMD=-0.297; 95%CI=-0.484,-0.111;p=.002)andpositivesymptoms(N=784;SMD=-0.227; 95%CI=-0.416, -0.038; p=.018) were also found. Compensatory interventions were not associated with improvements in negative symptoms (N=736; SMD=-0.162; 95%CI=-0.382, 0.058; p=.150). The heterogeneity of findings was low.

**Discussion:** Compensatory approaches are effective for improving functioning in psychosis, with a medium effect size. General symptoms and positive symptoms appear to benefit from compensatory approaches, but compensatory approaches are not effective for improving negative symptoms. Future analyses will examine the durability of effects, effects of study quality and moderating factors such as pure vs. partially compensatory, treatment intensity/length, mode of delivery (group vs. individual), baseline functioning level and age of participants.

### S204. NUTRITIONAL DEFICIENCIES AND CLINICAL CORRELATES IN FIRST-EPISODE PSYCHOSIS: A SYSTEMATIC REVIEW AND META-ANALYSIS

Joseph Firth*[,1], Rebekah Carney[2], Scott Teasdale[3], Brendon Stubbs[4], Philip Ward[5], Michael Berk[6], Jerome Sarris[7]
*[1]University of Western Sydney; [2]University of Manchester; [3]UNSW and SESLHD; [4]Institute of Psychiatry, Psychology and Neuroscience King's College London; [5]UNSW; [6]Deakin University, School of Medicine; [7]NICM, University of Western Sydney*

**Background:** Diet is increasingly recognised as a modifiable factor influencing the onset and outcomes of psychiatric disorders. Previous meta-analyses of blood nutrient levels in schizophrenia have already shown significant reductions in various individual vitamins/minerals. However, studies to date have largely focused on individual nutrients, and only considered nutrient status in patients with long-term schizophrenia. Meta-analytic evaluation of the evidence for nutrient deficits in first-episode psychosis (FEP) is completely absent. Therefore, we conducted a systematic review of all published studies comparing blood levels of vitamins and/or mineral in FEP to healthy control samples; and applied meta-analytic techniques to determine the prevalence and extent of deficiencies across the full spectrum of nutrients examined in this population to date.

**Methods:** We searched electronic databases from inception to July 2017 for all studies examining blood levels (i.e. serum, plasma or whole blood) of nutrient levels in people with FEP compared to healthy controls. Our systematic search identified 28 eligible studies, examining blood levels of 16 different nutrients (six vitamins, ten dietary minerals) across 2,612 individuals: 1,221 patients with FEP and 1,391 control subjects. Random effects meta-analyses compared nutrient levels in FEP to healthy controls. Clinical correlates of nutritional status in patient samples were systematically reviewed.

**Results:** Random effects meta-analyses found that people with FEP had large, significant reductions in blood levels of vitamin B9 (i.e. folate) compared to healthy controls(N=6, n=827, g=-0.624, 95% C.I.=-1.176 to -0.072, p=0.027). Significant reductions were also found for vitamin D (N=7, n=906, g=-1.055, 95% C.I.=-1.99 to -0.119, p=0.027) and, among fewer studies, vitamin C (N=2, n=96, g=-2.207, 95% C.I.=-3.71 to -0.71, p=0.004). No differences were found for other vitamins or minerals. Systematic synthesis of clinical correlates showed that reductions in both folate and vitamin D held significant relationships with greater psychiatric symptoms in FEP.

**Discussion:** This is the first meta-analysis to examine the prevalence, extent and clinical correlates of nutritional deficiencies in FEP to date. The deficits in vitamin D and folate which have previously been observed in long-term schizophrenia appear to exist from illness onset, even prior to antipsychotic treatment, and are associated with more severe symptoms. The extent and importance of these deficiencies suggests that routine screening for vitamin D and folate deficiencies should be considered in early intervention services. Furthermore, since our previous meta-analyses have shown that high-dose b-vitamin supplementation can reduce symptoms in long-term schizophrenia, this should now be investigated in FEP. The potential physical and psychological benefits of vitamin D supplementation in early psychosis should also be explored. However, further research is needed to establish casual and mechanistic relationships between vitamin deficiencies, poor diet and the onset and outcomes of psychotic disorders.

### S205. TRANSCRANIAL DIRECT CURRENT STIMULATION FOR SEVERE, PERSISTENT, TREATMENT-REFRACTORY AUDITORY HALLUCINATIONS IN SCHIZOPHRENIA

Parth Goyal*[,1], Lakhan Kataria[1], Chittaranjan Andrade[2], Priyal Desai[1]
*[1]SBKS MI&RC; [2]NIMHANS, Bengaluru*

**Background:** Up to 25% of schizophrenia patients continue to experience distressing auditory hallucinations despite best efforts at treatment with antipsychotic drugs. Transcranial direct current stimulation (tDCS) has been suggested to rapidly attenuate such persistent hallucinations.

**Methods:** We treated 23 schizophrenia patients with persistent, antipsychotic-refractory auditory hallucinations using tDCS in a single-group, open-label design. tDCS was administered at 2 mA current intensity for 20 min, twice-daily and 4 h apart, across 5 consecutive days; the anode was placed over the the left dorsolateral prefrontal cortex and the cathode over the left temporoparietal junction. Ongoing antipsychotic medications were continued unchanged. Patients were assessed using the Auditory Hallucinations Rating Scale (AHRS) at treatment endpoint and at 1- and 3-month follow up. Response was defined as 50% or greater attenuation in AHRS scores.

**Results:** All patients completed the study. tDCS resulted in substantial improvement. Mean (standard deviation) AHRS scores dropped from 29.0(8.3) at baseline to 4.4(5.6) at treatment endpoint; these values were 9.3(9.3) and 7.8(8.4) at 1- and 3-months follow up. The response rate was 91.3%, 69.6%, and 82.6% at the 3 posttreatment assessment points, respectively. Complete remission of hallucinations (AHRS=0) was observed in 61%, 44%, and 44% at the 3 posttreatment assessment points. tDCS was very well tolerated and adverse effects were minimal.

**Discussion:** tDCS is effective and well tolerated in schizophrenia patients with persistent, antipsychotic-refractory auditory hallucinations. In most patients, the benefits last for up to 3 months or longer.

### S206. KNOWLEDGE ABOUT CAUSES OF RELAPSE DURING PSYCHOEDUCATION IN PATIENTS LIVING WITH SCHIZOPHRENIA- A QUALITATIVE ANALYSIS

Anoop Gupta*[,1], Mamidipalli Spoorthy[2], Bina Gurung[1], Robin Jha[1], Bishnu Acharya[1]
*[1]National Medical College; [2]All India Institute of Medical Sciences, Raipur*

**Background:** Evidence till date shows different reasons of relapse in schizophrenia around the world. However, there was almost no reliable data from Nepal. We want to report a thematic study based on the reports of 12 patients living with schizophrenia and their family members. These patients were approached during psychoeducation group sessions.

**Methods:** Twelve patients with a diagnosis of schizophrenia as per Diagnostic and Statistical Manual of mental disorders-5 criteria, who were accompanied by their family members were selected. A minimum duration of illness of 5 years was required as inclusion criteria. Semi structured interviews were conducted with patient and family members separately in 1–2 sessions. Questions were mainly related to their knowledge about causes of relapse in patients in their perspective. Interviews were recorded and transcripts were generated. All the transcripts were read separately by the 3 investigators and common themes agreed upon by all the investigators were generated. We used content analysis for the purpose of the study. A total of 36 sessions psychoeducation were taken in in-patients from National Medical College, Birgunj, Nepal. Eight out of 12 patients were males. The group therapy was psycho-education oriented and based on NIMHANS manual for family-based intervention in schizophrenia. We included those patients who were admitted and improving as per PANSS score (more than 50% of the score at admission).

**Results:** The patients' family members told that these sessions were useful because their issues were discussed and addressed and simpler terms were used during the process. The patients showed ability to participate and understand the proceedings though not always. Two of the patients had sub-normal intelligence and so they were not benefited more than being heard about their sufferings. Their family members reported a better understanding of the illness and non-pharmacological approach for these patients after the sessions. Participants were encouraged to make notes out of the discussions in the sessions but few of them did so.

Following themes emerged after the analysis of transcribed verbatim from the patients and family members.

Themes generated from patient's versions:

1. Residual negative/depressive symptoms
2. Critical comments from family members
3. Adverse effects of medications
4. Improper education about the duration of treatment

Themes generated from family member's versions:

5. Lack of awareness about the illness
6. Belief in super natural causes
7. Affordability issues
8. Poor insight about the illness
9. Poor compliance to medications
10. Stress

**Discussion:** Conclusion: Educating our patients can be tiring and mundane during regular out-patient department. However, the psychoeducation sessions are very important part of the treatment. During that process we should anticipate the possible causes of relapse and educate the same for better outcome.

### S207. TDCS AS FUTURE TREATMENT OPTION FOR SCHIZOPHRENIA PATIENTS - A NEUROPHYSIOLOGICAL INVESTIGATION OF INDUCED PLASTICITY OVER MOTOR AND PREFRONTAL CORTEX USING SLORETA

Nina Filipova*,[1], Alkomiet Hasan[1], Daniel Keeser[1], Wolfgang Strube[1]

[1]*Ludwig-Maximilians-University of Munich*

**Background:** Transcranial direct current stimulation (tDCS) is a non-invasive, plasticity-inducing brain stimulation technique that can induce long-lasting excitability changes in the motor cortex and has been discussed as an alternative treatment option for patients with schizophrenia. Therefore, the aim of the present study was to detect electrophysiological correlates after motor cortical and prefrontal tDCS in order to improve the understanding of tDCS-mechanisms. Anodal and cathodal tDCS was applied over motor cortex (M1) and dorsolateral prefrontal cortex (DLPFC), which is known as one major region of interest considering neurobiology of psychosis. Thus, we looked for tDCS-induced source-localized activity changes in resting EEG by using sLORETA (standardized low-resolution brain electromagnetic tomography) and compared the effects of motor and prefrontal cortex.

**Methods:** A total of 20 healthy volunteers were examined within five sessions (within-subject design). Anodal tDCS (1mA, 13 minutes) and cathodal tDCS (1mA, 9 min) were applied over M1 and respectively DLPFC. In addition, there was a sham tDCS of DLPFC. Transcranial magnetic stimulation (TMS) was performed before and after motor cortical tDCS in order to generate motor evoked potentials (MEP) as periphery indicators of motor cortical plasticity. A 6-minute resting EEG was performed before and after each tDCS treatment. EEG data was then investigated by sLORETA for source-localized brain activity changes.

**Results:** After tDCS over M1, the expected increase of MEP amplitude after anodal tDCS and reduction after cathodal tDCS could be measured. Following anodal tDCS over M1 an increased activity was found in the area of precuneus in EEG frequency band alpha. After cathodal tDCS over M1 an activity decrease was seen in frequency band alpha, beta and total power, which could be localized in insula and temporal gyrus.

After anodal as well as cathodal tDCS over DLPFC decreased activities could be measured in most frequency bands (e.g. delta, theta, alpha, beta, total power). Most of these changes were found in frontal lobe, anterior cingulate or insula. Unexpectedly there were also significant changes after sham tDCS in all frequency bands. However, these were measured mostly in right-sided temporal lobe, which could be due to jaw muscle artefacts.

**Discussion:** The polarity-specific tDCS effects, which can be demonstrated in motor cortex, cannot be seen in prefrontal cortex; instead we detected a polarity-independent frontal modulation. This lack of prefrontal polarity specificity may be explained by a more complex mode of action in frontal cortex. This is consistent with the variable results of prefrontal tDCS in other publications. As the effects from motor cortical studies cannot easily be transferred to the frontal system in healthy subjects, one could speculate that in schizophrenic patients the responses to prefrontal tDCS might be even more difficult to predict. Further investigations are required to evaluate the heterogeneity of source-localized tDCS-effects and to understand prefrontal mechanisms, so that frontal tDCS may be used as a future treatment in schizophrenia patients.

### S208. PREDICTORS OF RESPONSE TO COGNITIVE REMEDIATION THERAPY: SYSTEMATIC REVIEW OF LITERATURE

Maree Reser*,[1], Reneta Slikboer[1], Susan Rossell[1]

[1]*Swinburne University of Technology*

**Background:** Impaired cognitive functioning is considered a core aspect of schizophrenia and is associated with poorer functional outcomes. Proving only marginally responsive to pharmacological interventions, there has been an acceleration of research investigating the efficacy of cognitive remediation therapy (CRT) in ameliorating cognitive deficits. While small to moderate effect sizes have been reported, closer examination suggests 40–60% of participants fail to realise a benefit. To improve both efficacy and effectiveness, better understanding of the factors that predict cognitive response to CRT is needed. To date, no systematic review of the evidence base has been conducted. We aimed to address that gap by providing a synthesis of predictor variables, whether they were moderators, mediators or predictors, of cognitive response to CRT.

**Methods:** An electronic database search was conducted across Scopus, Web of Science and PsychINFO databases and the Cochrane Collaboration Controlled Trials Register for all years until 30/09/2017. Reference lists of published meta-analyses and review articles were hand searched. Eligibility assessment was performed independently in an unblinded standardised manner by two reviewers. Studies that included a CRT arm, had a majority (≥70%) schizophrenia / schizoaffective disorder participants, had at least one training-distinct pre-post measure of cognition and at least one predictor of cognitive outcome were included. Studies that incorporated social cognitive training and/or adjunctive rehabilitation were excluded. A box-score analysis of predictor variables was conducted and the quality of the predictor evidence was assessed.
**Results:** Of 417 records extracted, 37 articles considering 1,499 overlapping CRT participants (2,423 full sample) were included in the final synthesis. On average, participants were in their mid-thirties, majority male, with approximately 12 years education. CRT trial arm size averaged 41.64 participants. Overall, 72 distinct predictors of cognitive response were identified, with an average 4.89 predictor variables considered across an average 3.95 cognitive domains per article. Of these, 42 were analysed once and 10 twice. Discussion focused on the 20 predictors examined a minimum 3 times. Highlighting the exploratory nature of predictor research to-date, few studies were theory driven or evidence based and fewer still were undertaken with a priori hypotheses. Only a handful of analyses included tests of interaction.
**Discussion:** Few of the currently examined predictors of cognitive response to CRT are significant when examined as a systematic review. The influence of age was the most frequently examined predictor, with a majority of articles finding no association. The strongest category level trend was found in baseline cognition, especially in reasoning and problem solving and working memory domains, which was more strongly predictive of within domain improvement. Training task improvement or "learning potential" was the most notable cross-domain predictor of cognitive outcome, though this was limited to three articles and warrants further investigation. It remains unclear why up to 60% of participants do not receive benefit from CRT. There is a need to look beyond the usual candidates, to pool data both to support methodologically robust, large-scale investigations and to better account for cross-population variability.

### S209. PATIENT EXPERIENCES OF THE EARLY SIGNS ACTION PLAN

Nils Sjöström*,[1], Maria Genberg[1], Alexander Marteleur[2], Eva Andreasson[1], Margda Waern[3]
*[1]Sahlgrenska University Hospital; [2]Region Västra Götaland; [3]Sahlgrenska University Hospital, University of Gothenburg*

**Background:** Coercive psychiatric care in Sweden has been criticized by the UN and alternative therapies are called for upon from Swedish politicians. The Early Signs Action Plan was developed to reduce force and promote cooperation between patients and their healthcare providers.
**Aim:** Describe the patients' experience of care when the Early Signs Action Plan is activated in connection with an exacerbation of psychotic illness.
**Methods:** Qualitative research study. Semi-structured interviews (anticipated N=10) will be conducted with patients for whom Early Signs Action Plans were activated. Interviews are recorded and transcribed verbatim. Content analysis is used to analyze the data.
**Results:** Preliminary results from the first five interviews suggest that the action plan facilitates shared decision making and encourages safety measures, and compulsory inpatient care can thus be avoided. The results from the entire study will be presented at the Conference.
**Discussion:** Preliminary findings suggest that the Early Signs Action Plan seemed to be a useful tool to im-prove patient participation and reduce the need for compulsory inpatient care when exacerbations occur.

### S210. MULTIDISCIPLINARY LIFESTYLE-ENHANCING TREATMENT FOR INPATIENTS WITH SEVERE MENTAL ILLNESS (MULTI-STUDY): EFFECTS ON PHYSICAL HEALTH, PSYCHOTIC SYMPTOMS, QUALITY OF LIFE AND FUNCTIONING

Jeroen Deenik*,[1], Diederik Tenback[1], Ingrid Hendriksen[2], Erwin Tak[3], Peter van Harten[1]
*[1]GGz Centraal, Amersfoort; [2]LivIng Active, Santpoort-Zuid; [3]Tak advies en onderzoek, Leiden*

**Background:** Patients with severe mental illness (SMI) are characterized by an unhealthy lifestyle, which contributes to the widening mortality gap with the general population [1] Changing high levels of sedentary behaviour (SB) and lack of physical activity (PA) is very challenging [2–4].
Effective interventions improving lifestyle in inpatients are still limited, while of all patients with SMI, the hospitalized do have the worst health status.
We implemented a MUltidisciplinary Lifestyle Enhancing Treatment for Inpatients with SMI (MULTI), mainly including a daily structure, tailored sports- and work-related activities, attention to dietary habits, psycho-education and participation of staff. It involved a culture change which was implemented based on a 'change-from-within'-principle, using multidisciplinary* cooperation within the current context and resources of inpatient mental healthcare.
* Psychiatrists, activity coordinators, nurse practitioners, dietician and nurses, some of them trained as lifestyle coach.
**Aim:** Evaluate changes in physical and mental health and functioning after 18 months compared to treatment as usual (TAU).
**Methods:** Observational controlled design including long-term hospitalised inpatients with SMI. We used data from routine screening and a previous cross-sectional study (2013), supplemented by a repeated accelerometer measurement (2015). Patients were included if they received no other intervention related to lifestyle within 18 months after the start of MULTI and if baseline accelerometer data was available. Patients were excluded from analysis if they had a lack of data after 18 months because they (1) were deceased, (2) moved or were discharged from the hospital or (3) had insufficient follow-up accelerometer data.
**Measures:**
Accelerometer-measured physical activity (PA) [ActiGraph GT3X+]
Metabolic health [weight, abdominal girth, blood-pressure and -levels and metabolic syndrome criteria]
Psychotic symptoms [PANSS-r]
Quality of life (QoL) [EQ-5D & WHOQoL-Bref]
Psychosocial functioning [HoNOS]
**Analysis:** hierarchical multilevel regression using change-scores, correcting for baseline outcome-value, age, diagnosis and baseline illness-severity.
**Results:** We had sufficient data of 65 patients receiving MULTI and 49 within TAU.
Significant ($p < 0.05$) improvements in total PA (B = 0.5), moderate-to-vigorous PA (B = 1.8%), weight (B = -4.2kg), abdominal girth (B = -3.5cm), systolic blood-pressure (B = -8.0mmHg), HDL-cholesterol (B = 0.1mmol/l) and psychosocial functioning on sums score (B = -3.6), impairment (B = -0.7) and social problems (B = -3.0). No improvements were observed in PA/metabolic health within TAU. Patients receiving MULTI had higher odds to recover from ≥1 metabolic syndrome criterion (OR = 2.06). There was no significant effect on psychotic symptoms. QoL improved significantly in both groups.
**Discussion:** Striking results for clinical practice, as much effort and attempts on lifestyle within inpatients with SMI failed to achieve desired improvements, especially in longer term.
A turnaround in inpatient mental healthcare: the negative trend of deterioration within these patients can be stopped, relevant parameters can even be positively reversed and negative effects are absent.
TAU does not improve physical health A sustainable solution towards a healthier lifestyle in inpatients with SMI at our fingertips, as MULTI was implemented using current context and resources.

### S211. SCHIZOTYPY IN PATIENTS FROM A CLINICAL HIGH RISK SERVICE: TRAIT OR STATE?

Frauke Schultze-Lutter*,[1], Rahel Flückiger[2], Chantal Michel[2], Daniela Hubl[3]

[1]Heinrich-Heine University Düsseldorf; [2]University Hospital of Child and Adolescent Psychiatry and Psychotherapy, University of Bern; [3]University Hospital of Psychiatry, University of Bern

**Background:** Schizotypy is considered to assess psychosis-proneness in terms of a rather stable (personality) trait. For the psychometric assessment of schizotypal traits, the Wisconsin Schizotypy Scales (WSS) are widely used. They consist of four subscales, Physical Anhedonia, Social Anhedonia, Perceptual Aberration and Magical Ideation. The latter 2 positive scales were reported to load on the same factor as delusion- and perception-related attenuated positive symptoms that are used to define a clinical high risk (CHR) state. Results from non-clinical samples showed relatively good invariance of the WSS across time. Yet, it is unknown, if a CHR state influences report on WSS and if the stability of schizotypy measures is equally good in a clinical sample.
**Methods:** This was examined in naturalistic follow-up data of an early detection of psychosis service in Switzerland (N=30 at the time of writing). At baseline (t0), the mean age of the sample was 19 ± 5 years and 45% were male and the mean follow-up duration was 16 ± 10 months (range 5–42 months).
**Results:** Analyses indicated a change in risk status at first follow-up (t1) in 59% (42% decrease, 17% increase of risk), yet Friedman tests revealed no significant differences in WSS mean sum scores for each subscale between t0 and t1: Physical Anhedonia 16.74 vs. 15.23 ($\chi 2(1)=2.133$, p=.144), Social Anhedonia 13.81 vs. 12.61 (Chi2(1)=3.0, p=.083), Perceptual Aberration 5.81 vs. 5.19 (Chi2(1)=2.286, p=.131), Magical Ideation 6.48 vs. 6.19 (Chi2(1)=0, p=1.0).
**Discussion:** These preliminary results indicate that, even in the presence of significant changes in CHR symptomatology, schizotypy scores seem to be relatively stable over time and therefore strengthen the assumption of schizotypy as a trait marker.

### S212. CONVERSING WITH PEOPLE WITH THOUGHT DISORDER

Cherrie Galletly*[,1], David Ash[2], Shaun Sweeney[3], Jonathan Crichton[4]
[1]The University of Adelaide; [2]The Adelaide Clinic; [3]Northern Adelaide Local Health Network; [4]University of South Australia

**Background:** Thought disorder is a common symptom in psychotic disorders such as schizophrenia. In the research and training literature, thought disorder is assumed to prevent any useful conversation (Galletly & Crichton, 2011). It is depicted as something to be sampled and analysed, rather than as a factor that modifies, but does not prevent, meaningful communication. Psychiatrists routinely interact with their thought disordered patients as part of day-to-day clinical care. The study investigates the skills and strategies used by psychiatrists in clinical interviews with patients with thought disorder. The importance of this study is that identification and detailed description of these specific interview techniques will enable them to be used in the training of psychiatrists and other mental health clinicians.
**Methods:** Twenty-four routine interviews between inpatients with thought disorder and their treating psychiatrists were recorded and transcribed. All participants gave written informed consent and this study was approved by the institutional ethics committees.
The transcripts were examined by the research team (an applied linguist, two psychiatrists and mental health social worker). Excerpts were subsequently presented at two workshops, attended by psychiatrists, trainee psychiatrists and junior medical staff. Participants were asked to identify and describe the techniques used by the psychiatrists in the course of their interviews with thought disordered patients.
**Results:** The interviews were generally quite brief (mean duration 19.24 (SD 7) minutes), and had many characteristics in common. The tone was conversational, with a normal turn-taking structure, few repairs and an easy flow despite the often-disjointed content. The psychiatrists were not confrontational or judgemental. During the first half of the interview, there was often a period of delusional, thought disordered discourse, in which psychiatrists engaged through close attention to the patient's language, navigating this while commenting and sometimes asking questions. The purpose seemed to be to build rapport by ensuring the patient felt they had been listened to, and this period of relatively uninterrupted speech provided the psychiatrist with the opportunity to assess the patient's mental state. Rather than comment on the content, psychiatrists often engaged with the feelings (e.g. 'stressed") that arose in response to the psychotic experiences described by patients.
It was clear that both parties contributed to the agenda for the conversation. The patients were active participants, often with specific questions or concerns, and these were answered carefully and respectfully regardless of content. Psychiatrists observed that they were also powerless about some matters of particular concern to patients (e.g. the no-smoking policy). Discharge planning was a substantial component of the interviews, as patients wished to leave hospital as soon as possible, and psychiatrists have institutional pressures to reduce the length of stay. The psychiatrists generally assessed insight, but did not undertake formal risk assessments. They provided explanations of the patient's legal status, current treatments, and planned treatments (such as ECT).
**Discussion:** Thought disorder does not exist in isolation, as a phenomenon to be sampled; rather it modifies but does not prevent meaningful conversation and exchange of ideas and information. Experienced psychiatrists are able to undertake meaningful, useful interviews with people with thought disorder. The skills involved have not been described previously. These findings h can be used to develop training resources for mental health clinicians who will be working with people with psychotic disorders.

### S213. CAN PATIENTS WITH TREATMENT RESISTANT SCHIZOPHRENIA RELIABLY REPORT NEGATIVE SYMPTOMS? A PILOT STUDY USING THE SELF-EVALUATION OF NEGATIVE SYMPTOMS SCALE

Jean-Pierre Lindenmayer*[,1], Abraham Goldring[2], Amanda Hefner[2], Anzalee Khan[2], Amod Thanju[2]
[1]New York University; [2]Manhattan Psychiatric Center

**Background:** The Self-Evaluation of Negative Symptoms (SNS), a 20-item self-rating scale,was developed to assess the subjective experience of negative symptoms by schizophrenia patients. The reliability and validity of the translated French version of the SNS was examined in a sample of outpatients in an US site with schizophrenia and schizoaffective disorders (Dollfus et al., 2015). The author found that the SNS had good psychometric properties and demonstrated that the patients' ratings were highly correlated with observer ratings, which contradicts the expected lack of reliability of patient reported symptoms in patients with schizophrenia. However, the patients included in the study were stable outpatients with high levels of functioning as compared to lower functioning patients. It remains to be explored whether patients with lower levels of functioning are equally able to identify their negative symptoms in a reliable fashion. The aim of the present study was to first evaluate the reliability of the novel tool of self-evaluation of Negative Symptoms (SNS) and to examine its correlation with observer ratings of negative symptoms in a sample of inpatients with ICD 10 schizophrenia or schizo-affective disorder who function at a low level of overall cognition. It was our goal to examine if chronic, low functioning patients are able to complete the instrument without assistance, providing clinically meaningful information with respect to their own perception of negative symptoms.
**Methods:** Patients who met DSM-5 criteria for schizophrenia or schizoaffective disorder were included in the study. All patients will provide written informed consent. Patients were administered the SNS assessment at two time points, separated by 1 one week, followed by other concurrent evaluations: the 16-Item Negative Symptom Assessment (NSA-16), a validated clinical assessment for negative symptoms, the CGI-S, WRAT, BACS, and the CDSS. To examine the internal consistency of the SNS. Cronbach's alpha was calculated for the 20 items and the 5 sub scores at both times. Correlation analyses were performed to examine the convergent validity of the SNS with the observer rated negative symptom scale. Convergent and discriminant validities were tested with Pearson's correlations. The test-retest reliability of the SNS will be tested by intraclass correlation coefficients (ICCs).

**Results:** Fourteen patients with schizophrenia or schizoaffective disorder according to DSM-5, and a mean age of 43.00 (12.48) were evaluated. 66.67% of subjects were African American. Cronbach's coefficient of the SNS ($\alpha = 0.791$) showed good internal consistency. The SNS did not show significant correlations with the NSA-16 ($r = 0.207$, $p = 0.497$), the NSA global score ($r = 0.390$, $p = 0.296$), nor the Clinician Global Impression on the severity of negative symptoms ($r = -0.264$, $p = 0.383$). SNS scores did not correlate with level of insight as measured by the SUMD ($r = -0.51$, $p = 0.870$), Motor Functioning deficits as measured by the SAS ($r = 0.227$, $p = 0.456$). The intrasubject reliability of the SNS revealed good intraclass correlation coefficients (ICC = 0.780). Test-Retest was significant at 0.791, $p = 0.004$, with a significant change at $t(12) = 3.923$, $p = 0.002$.

**Discussion:** Our pilot study suggests that the agreement between self-rating and observer-rating of negative symptoms in patients with treatment resistant schizophrenia is rather low as. Patients also evaluated the severity of their negative symptoms rather differently. Reasons for this discrepancy will be discussed, in particular, in the context of low levels of illness insight as well as the psychometric qualities if the SNS.

### S214. USING ONLINE-SCREENING TO DETECT PARTICIPANTS AT CLINICAL HIGH-RISK FOR PSYCHOSIS

Mhairi McDonald[1], Andrew Gumley[2], Stephen Lawrie[3], Matthias Schwannauer[3], Ruchika Gajwani[2], Joachim Gross[1], Peter Uhlhaas*[,4]

*[1]Institute of Neuroscience and Psychology, University of Glasgow; [2]Institute of Health and Wellbeing, University of Glasgow; [3]University of Edinburgh; [4]Institute of Neuroscience & Psychology, University of Glasgow*

**Background:** Identification of participants at clinical high-risk (CHR) for the development of psychosis is an important objective of current preventive efforts in mental health research. However, the utility of using web-based screening approaches to detect CHR-participants at the population-level has not been investigated.

**Methods:** We tested a web-based screening approach to identify CHR-individuals. Potential participants were invited to a website via email-invitations, flyers and invitation letters involving both the general population and mental health services. 2121 participants completed the 16-item version of the prodromal questionnaire (PQ-16) and a 9-item questionnaire of perceptual and cognitive aberrations (PCA) for the assessment of Basic Symptoms (BS) online.

**Results:** 54% of participants met a-priori cut-off criteria for the PQ and 72 % for PCA-items online. 969 participants were invited for a clinical interview and n = 277 interviews were conducted (response rate: 29%) using the Comprehensive Assessment of At-Risk Mental State (CAARMS) and the Schizophrenia Proneness Interview, Adult Version (SPI-A). N = 88 CHR-participants and n = 8 first-episode psychosis (FEP) were detected. ROC-curve analysis revealed good to moderate sensitivity and specificity for predicting CHR-status based on online-results for both UHR- and BS-criteria (Sensitivity/Specificity: PQ-16 = 76%/50.4%; PCA = 89%/19.7%). CHR-participants were characterized by similar levels of functioning and neurocognitive deficits as clinically identified CHR-groups.

**Discussion:** These data provide evidence for the possibility to identify CHR-participants through population-based web-screening. This could be an important strategy for early intervention and diagnosis of psychotic disorders.

### S215. THE APROSODY OF SCHIZOPHRENIA: COMPUTATIONALLY DERIVED ACOUSTIC PHONETIC UNDERPINNINGS OF MONOTONE SPEECH

Michael Compton[1], Luca Pauselli*[,2], Anya Lunden[3], Sean Cleary[4], Yazeed Alolayan[5], Brooke Halpern[6], Beth Broussard[6], Anthony Crisafio[4], Leslie Capulong[6], Pierfrancesco Balducci[7], Francesco Bernardini[7], Michael Covington[8]

*[1]College of Physicians and Surgeons, Columbia University; [2]College of Physicians and Surgeons, Columbia University, New York State Psychiatric Institute; [3]College of William and Mary; [4]The George Washington University; [5]Case Western Reserve University; [6]Lenox Hill Hospital; [7]Università Degli Studi Di Perugia; [8]University of Georgia*

**Background:** Acoustic phonetics methods are useful in examining some symptoms of schizophrenia; we used such methods to understand the underpinnings of aprosody. We hypothesized that compared to controls and patients without clinically rated aprosody, patients with aprosody would exhibit reduced variability in: pitch, jaw/mouth opening and tongue height (formant F1), tongue front/back position and/or lip rounding (F2), and intensity/loudness.

**Methods:** Audio-recorded speech was obtained from 98 patients (including 25 with clinically rated aprosody and 29 without) and 102 unaffected controls using five tasks: one pertaining to describing a drawing (Task 1), two based on spontaneous speech elicited through a question (Tasks 2 and 3), and two based on reading prose (Tasks 4 and 5). We compared the three groups (patients with aprosody, patients without aprosody, and controls) in terms of variation in pitch, formants F1 and F2, and intensity/loudness.

**Results:** Phonetic values were generally highly correlated across the five speech tasks. Regarding pitch variation, in unadjusted tests, patients with aprosody differed significantly from controls in Tasks 3 and 4; for Task 5, the difference was statistically significant in both unadjusted tests and those adjusted for sociodemographics. For the standard deviation (SD) of F1, the expected pattern was observed in the two reading tasks in adjusted tests (lower values for patients with aprosody, intermediate values for patients without aprosody and higher values for controls). Regarding SD of F2, patients with aprosody had lower values than controls in unadjusted tests across all tasks; in adjusted tests the expected pattern was observed in the two spontaneous speech tasks. Comparisons of variation in intensity/loudness, despite a much smaller sample size of participants with data on this variable, showed the expected pattern in adjusted tests.

**Discussion:** Although values of each individual parameter across the five tasks tend to be highly correlated, it appears that different types of prompts for obtaining audio-recorded speech may in fact produce some differences across phonetic parameters. For example, whereas loudness appeared to be blunted equally across all of our tasks, variation in both pitch and F1 were blunted most obviously in the reading tasks, and reduced variation in F2 was most apparent in the two spontaneous speech tasks. Small sample size, no measures of negative symptoms in healthy controls and not controlling for patients' medications are the main limitations of this work. Nonetheless, findings could represent a step toward developing new methods for measuring and tracking the severity of this specific negative symptom using acoustic phonetics parameters. Such work is relevant to other psychiatric and neurological disorders.

### S216. REDUCED EMOTIONAL FACIAL EXPRESSION IN SCHIZOPHRENIA – A PROBE INTO THE PHENOMENOLOGY AND RELEVANCE OF EXPRESSIVE NEGATIVE SYMPTOMS

Marcel Riehle*[,1], Stephanie Mehl[2], Tania M. Lincoln[1]
*[1]University of Hamburg; [2]Frankfurt University of Applied Sciences*

**Background:** Emotional facial expressions are vital communicative signals and a lack thereof should interfere with successful social interaction. That people with schizophrenia lack emotional facial expression, mostly irrespective of antipsychotic medication, is not only a well-known notion but also backed up by ample evidence. However, a closer look reveals a more complicated picture that implies that maybe only those with expressive negative symptoms (ENS; i.e. blunted affect and alogia) but not those without ENS show reduced facial expressiveness. Furthermore, while the reduction has been found consistently for positive facial expressions, the evidence for negative facial expressions has been mixed. Finally, the social consequences of the reduction are mostly unknown and thus whether or not the reduction actually interferes with social interactions. To address these questions, we tested for the symptom-specificity of reduced positive and negative facial expression (phenomenology) and their social relevance in patients with schizophrenia with versus without ENS.
**Methods:** The frequency of positive and negative facial expressions in an affiliative role-play were assessed with the Facial Expression Coding System (FACES) in people with schizophrenia with (n = 18) and without ENS (n = 30) and in healthy controls (n = 39). Based on observing the role-play, independent raters also rated their willingness for future interactions with each participant. The presence of ENS was assessed via the Positive and Negative Syndrome Scale (PANSS).
**Results:** Patients with schizophrenia and ENS did not differ on positive symptoms and depression or on chlorpromazine equivalent medication dosage from those without ENS. The analysis of the frequency of facial expressions revealed that patients with ENS showed reduced levels of positive facial expressions both compared to those without ENS (d = -0.82) and to controls (d = -1.21). Both patient groups (with and without ENS) showed equally reduced negative facial expressions compared to controls (ds = -0.99 and -0.86). Raters also indicated less willingness for future interactions with patients with ENS than without ENS (d = -0.92). This difference was significantly mediated by the reduced positive facial expressions.
**Discussion:** The findings offer new insights into the phenomenology and the relevance of reduced emotional facial expression in schizophrenia. Our study indicates that the moderate to large mean differences that have been reported in earlier studies comparing samples with more broadly defined schizophrenia to healthy controls could mainly be driven by a reduction in facial expressions that is relatively specific to those with ENS. However, some aspects of reduced facial expression may nevertheless be genuine to more broadly defined schizophrenia given that we found patients with schizophrenia both with and without ENS to exhibit reduced levels of negative facial expressions. Finally, we found that the reduction of the positive facial expressions explained why raters were more willing to interact with those without ENS than with those with ENS. This further highlights the relevance of ENS by showing that they interfere with successful social interaction and go along with immediate social costs.

### S217. SELF-DISTURBANCES AND DIAGNOSTIC STABILITY IN FIRST EPISODE PSYCHOSIS: A SEVEN YEAR FOLLOW-UP STUDY

Ingrid Svendsen*[,1], Merete Øie[1], Paul Møller[2], Barnaby Nelson[3], Ingrid Melle[4], Elisabeth Haug[1]
*[1]Innlandet Hospital Trust, Norway; [2]Vestre Viken Hospital Trust, Norway; [3]Orygen Youth Health Research Centre; [4]NORMENT Centre for Psychosis Research, University of Oslo*

**Background:** Self-disturbances are considered core features of schizophrenia spectrum disorders, and are present in the prodromal, the early psychotic and in the chronic phase. Self-disturbances are also present at first treatment in some patients with psychotic disorders outside of the schizophrenia spectrum. There is limited knowledge about the stability of self-disturbances over time. The aim is to explore the stability of self-disturbances in a seven year follow-up of first episode patients and to examine the association between self-disturbances at start of treatment and diagnostic changes at follow-up.
**Methods:** Longitudinal study of 56 patients recruited at their first treatment for an affective or non-affective psychotic disorder. Self-disturbances were assessed by the Examination of Anomalous Self-Experience (EASE), while diagnostic categories, symptom severity, and functioning were assessed with standard clinical instruments. At baseline we registered life-time experiences of self-disturbances. At follow-up we focused on self-disturbances experienced the last two years
**Results:** At follow-up 35 patients were diagnosed with schizophrenia or a schizoaffective disorder (schizophrenia) and 21 with a bipolar, psychotic disorder or delusional disorder (non-schizophrenia). The level of self-disturbances was significant lower at follow-up than at baseline in patients with schizophrenia. Patients with schizophrenia had significantly higher levels of self-disturbances both at baseline and at follow up than patients in the non-schizophrenia group, who showed stable low levels of self-disturbances. In the schizophrenia group the EASE domain “Cognition and stream of consciousness”, was the most stable. There were no changes into or out of the schizophrenia group. The four patients in the non-schizophrenia group with relatively high EASE total scores at baseline (≥ 15) did not convert to schizophrenia at follow-up, as hypothesized. No patients in the non-schizophrenia group who increased their EASE score from baseline to follow-up converted to the schizophrenia group.
**Discussion:** EASE domain “Cognition and stream of consciousness”, have previously been described as some of the first self-disturbances appearing in the prodromal phase and are also found to be the most predictive of transition to full-threshold psychosis in an Ultra High Risk group. The results from the present study show that these phenomena are also the most stable over time. We did not find that patients outside the schizophrenia group, converted to schizophrenia, neither among those who had high level of self-disturbances at baseline nor those who had increased levels of self-disturbances at follow-up. The current study was conducted in rural areas with considerable distances to the specialized psychiatric health services, and consequently with long duration of untreated psychosis. The observed diagnostic stability is thus to be expected if symptomatic developments relevant for diagnosis take place early in the first episode, in this case before the first treatment contact.

### S218. INSIGHT AND SUBJECTIVITY

Clara Monserrat*[,1], Dolores Sanchez-Cabezudo[2], Carles Garcia-Ribera[3], Carmina Massons[4], Lourdes Nieto[5], Esther Pousa[3], Jesús Cobo[4], Ada Ruiz[1], Susana Ochoa[6], Judith Usall[6]
*[1]Hospital del Mar de Barcelona; [2]Hôpital Philippe Pinel, Amiens; [3]Hospital de Sant Pau de Barcelona; [4]Corporació Sanitària i Universitaria Parc Taulí, Sabadell; [5]Instituto Nacional de Psiquiatría Ramón de la Fuente Muñiz, México D. F.; [6]Sant Joan de Déu de Barcelona*

**Background:** The awareness of mental disorder or insight refers to the ability to perceive the disorder itself and the symptoms, the effects of the treatment and the social consequences of the disorder; and also the ability to attribute the symptoms to a mental disorder. Lack of insight is frequent in schizophrenia and is associated with a low adherence to the treatment and to a worse evolution. A greater insight has been associated with a lower psychopathological severity and with higher levels of depression.
On the other hand, subjective insight refers not only to what happens to the patient but also to how he feels and to the perception of the changes that he undergoes during the psychotic experience. The subjective perception of change is a position that can easily lead to connect with painful and depressive feelings, so it can be assumed that subjective insight could be related more consistently with the depressive symptoms than the clinical insight.
**Methods:** Observational cross-sectional study of a group of 114 schizophrenia patients treated in the psychiatry devices of the Parc de Salut Mar and Parc Taulí Instruments: SUMD, Markova and Berrios Scale and Calgary scale for depression in psychosis.
**Results:** Subjective insight is significantly correlated with Lindenmayer's depressive factor and depression level measured by a Calgary scale.
Clinical insight correlates with positive and excitatory symptoms. The time of evolution explains the non-awareness of the social consequences of the disease.
**Discussion:** The subjective insight into schizophrenia is mainly related to the depressive symptoms. The clinical insight into schizophrenia is related to positive symptoms.

### S219. RISK FACTORS FOR LOW BONE MINERAL DENSITY IN PATIENTS TAKING ANTIPSYCHOTICS

Min Jhon[1], Ji-Eun Hong[2], Cheol Park[3], Ju-Yeon Lee[4], Anna Jo[1], Jae-Min Kim[5], Il-Seon Shin[5], Lana Williams[6], Michael Berk[7], Jin-Sang Yoon[5], Sung-Wan Kim*,[5]
*[1]Chonnam National University Hospital; [2]Gwangju Mental Health Commission; [3]Gwagnju Veterans Hospital; [4]Chonnam National University Medical School, Gwangju Bukgu Community Mental Health Center; [5]Chonnam National University Medical School; [6]Deakin University, IMPACT Strategic Research Centre, School of Medicine; [7]Deakin University, School of Medicine*

**Background:** The aim of this study is to explore potentially modifiable risk factors for low bone mineral density (BMD) in adults with psychotic disorders. Furthermore, we sought to identify gender-specific risk factors.
**Methods:** The study included 285 community-dwelling patients with psychotic disorders. Dual-energy x-ray absorptiometry was used to measure BMD. Laboratory examinations included vitamin D and prolactin levels. Low BMD was defined as<1 standard deviation below the mean for young adults. Clinical characteristics associated with low BMD were identified with logistic regression analysis in total population and each gender.
**Results:** Fifty-eight (20.4%) subjects had low BMD. Low BMD was more common in men and in patients with low body mass indices (BMIs), as well as in those with shorter treatment durations, those on Medicaid, and patients using serotonergic antidepressants. Logistic regression analysis revealed that low BMD was negatively associated with BMI and treatment duration and positively with gender (male) and serotonergic antidepressants use in the overall population. In men, low BMD was associated with treatment duration and BMI; in women, low BMD was associated with BMI, prolactin level, vitamin D, and serotonergic antidepressant use.
**Discussion:** Low BMI was risk factor for reduced BMD in both genders. Shorter treatment duration was associated with low BMD in men, whereas higher prolactin levels, lower vitamin D, and the use of serotonergic antidepressants were associated with low BMD in women. Psychotropic agents should be prescribed mindful of their effects on bone, as use of these medications is a modifiable risk factor for osteoporosis in women with psychotic disorders.

### S220. BLONANSERIN AUGMENTATION IN PATIENTS WITH SCHIZOPHRENIA – WHO IS BENEFITED FROM BLONANSERIN AUGMENTATION? AN OPEN-LABEL, PROSPECTIVE, MULTI-CENTER STUDY

Won-Myong Bahk*,[1], Young Joon Kwon[2], Bo-Hyun Yoon[3], Sang-Yeol Lee[4], Kwanghun Lee[5], Duk-In Jon[6], Moon Doo Kim[7], Eunsung Lim[8]
*[1]Catholic University of Korea; [2]College of Medicine, Soonchunhyang University; [3]Naju National Hospital; [4]Wonkwang University, School of Medicine; [5]College of Medicine, Dongguk University; [6]College of Medicine, Hallym University; [7]School of Medicine, Jeju National University; [8]Shinsegae Hospital*

**Background:** Evidences for antipsychotic augmentation for schizophrenic patients with sub-optimal efficacy have been lacking although it has been widespread therapeutic strategy in clinical practice. The purpose of this study was to investigate the efficacy and tolerability of blonanserin augmentation with an atypical antipsychotics (AAPs) in schizophrenic patients.
**Methods:** A total of 100 patients with schizophrenia partially or completely unresponsive to treatment with an AAP recruited in this 12-week, open-label, non-comparative, multicenter study. Blonanserin was added to existing AAPs which were maintained during the study period. Efficacy was primarily evaluated using Positive and Negative Syndrome Scale (PANSS) at baseline, week 2, 4, 8, and 12. Predictors for PANSS response (≥20% reduction) was investigated.
**Results:** The PANSS total score was significantly decreased at 12 weeks after blonanserin augmentation (-21.0 ± 18.1, F=105.849, p<0.001). Response rate on PANSS at week 12 was 51.0%. Premature discontinuation was occurred in 17 patients (17.0%) and 4 patients among them discontinued the study due to adverse events. Nine patients experienced significant weight gain during the study. Response to blonanserin augmentation was associated with severe (PANSS>85) baseline symptom (OR=10.298, p=0.007) and higher dose (>600mg/day of chlorpromazine equivalent dose) of existing AAPs (OR=4.594, p=0.014).
**Discussion:** Blonanserin augmentation improved psychiatric symptoms of schizophrenic patients in cases of partial or non-responsive to an AAP treatment with favorable tolerability. Patients with severe symptom despite treatment with higher dose of AAP were benefited from this augmentation. These results suggested that blonanserin augmentation could be an effective strategy for specific patients with schizophrenia.

### S221. QUANTITATIVE SYSTEMS PHARMACOLOGY AS AN ALTERNATIVE TO CHLORPROMAZINE EQUIVALENTS: PREDICTIVE VALIDATION FROM A CRIS DATABASE EXPERIMENT

Hugo Geerts*,[1], Athan Spiros[1], Giouliana Kadra[2], Robert Stewart[2], Richard Hayes[2], Hitesh Shetty[3], Ehtesham Iqbal[4]
*[1]In Silico Biosciences; [2]Institute of Psychiatry, King's College London; [3]BRC Nucleus, South London and Maudsley NHS Foundation Trust; [4]BRC Nucleus, South London and Maudsley*

**Background:** Polypharmacy is common in real clinical practice and in pharma-sponsored clinical trials. Chlorpromazine equivalents do not take into account pharmacodynamic interactions of drug combinations. If there is a sufficiently deep calibration set available, bio-informatics approaches can build classifiers for clinical phenotypes. However, this is not always the case which severely limits the generalizability of the predictions.
**Methods:** We applied a mechanism-based computer model of a cortico-striatal-thalamocortical loop of the dorsal motor circuit that has been calibrated with clinical data on the prevalence of extrapyramidal symptoms after antipsychotic treatment in schizophrenia patients and therapeutic interventions in Parkinson's patients[1]. The Quantitative Systems Pharmacology (QSP) model is based on the appropriate connections between basal ganglia regions and consists of 220 neurons (8 different cell types), 3500 synapses and implementations of 32 CNS active targets, based on their unique locations and coupling with intracellular pathways. Modulation of the various CNS targets were calculated on simulating the competition between the endogenous neurotransmitter and the two drugs at their appropriate concentrations and affinity.

The model was challenged to blindly predict the extrapyramidal symptoms liability of 1,124 patients prescribed two antipsychotics for six or more months (772 unique combinations). Anonymized data were derived from South London and Maudsley NHS Foundation Trust (SLAM) electronic health records (EHR). Extrapyramidal side effects were captured and identified using a combination of Natural Language Processing and a bespoke algorithm [2]. Only names and doses of the two drugs were made available without any calibration set.
**Results:** Blind prediction of the outcomes using a Receiver Operating Characteristic curve with the QSP model resulted in an Area-Under-the Curve of 0.64 (p<0.01), compared to an AUC of 0.52 for the sum of the chlorpromazine equivalents, 0.53 for the sum of affinity constants or the sum of D2R occupancies of the individual antipsychotics (AUC=0.52).
**Discussion:** QSP is a powerful approach to predict PD-PD interactions in the absence of any calibration set or with limited and unique data and is superior to chlorpromazine equivalents for predicting EPS liability. A major application is the simulation of pharmacodynamic interactions of comedications in clinical trials with novel compounds leading to possible better balance between the different treatment arms

### S222. CLINICAL UTILITY OF PHARMACOGENETIC TESTING IN SCHIZOPHRENIA TREATMENT

Daniel Mueller*[,1], Arun Tiwari[2], Clement Zai[3], James Kennedy[3]
*[1]University of Toronto; [2]Centre for Addiction and Mental Health; [3]CAMH, University of Toronto*

**Background:** Antipsychotics (APs), antidepressants (ADs) and mood stabilizers are essential components in treatment of most psychiatric disorders and in particular in schizophrenia. Unfortunately, among the various compounds which have been developed, lengthy trials are often required before the optimum medication treatment is found, i.e. with most significant symptom alleviation and minimal side effects. Thus, predictive factors would thus be extremely beneficial in clinical practice. The underlying reasons for this large inter-individual
variability in terms of treatment response are not fully understood. Important factors that influence drug dose, response and side effects include age, gender, patient compliance, constellation of symptoms, co-morbidity, and to a large extent genetic factors.
**Methods:** Methods follow two strategic concepts, i.e. 1) review of the literature and review of the clinical utility of using genetic information preemptively and 2) results of own studies evaluating treatment outcome in psychiatric care after genetic information (e.g., CYP2D6 and CYP2C19) was provided to more than 350 physicians.
**Results:** There is growing consensus among expert that genetic testing to optimize medication treatment in psychiatry meets criteria for clinical utility. However, utility remains restricted to specific gene-drug pairs and multi-gene test require further validation.
Our own research has shown that variation in genes involved in the metabolism of psychotropic drugs (pharmacokinetics) and genes encoding drug targets, such as brain receptors (pharmacodynamics) are associated with plasma drug levels, treatment response, and side effects (e.g., antipsychotic-induced weight gain). In addition, our genome-wide analyses have revealed associations with clinical outcome to antipsychotics or antidepressants and markers in neurotrophins, cell-signaling and inflammatory pathways.
With respect to our preemptive genetic testing program in more than 10,000 patients, we received supportive responses from physicians who enrolled patients in our study. Notably, while the vast majority of patients reported improvement in patient outcome, only two physicians indicated that their patient's symptoms has slightly worsened after they had used the pharmacogenetic report to guide treatment.
**Discussion:** There is emerging evidence that preemptive genetic testing for numerous gene & psychiatric-drug pairs has reached levels for clinical utility which includes validation of analytical and clinical validity. Genetic testing has become readily available but however clinicians and patients are poorly prepared to this new emerging field and proper education is of utmost importance. This presentation will review the level of evidence for 'actionable' gene-drug pairs in psychiatry in addition to present novel genomic findings and reports from our ongoing genetic testing experiences.

### S223. COMBINED TREATMENT WITH A SELECTIVE PDE10A INHIBITOR TAK-063 AND ANTIPSYCHOTICS AT SUBEFFECTIVE DOSES PRODUCES POTENT ANTIPSYCHOTIC-LIKE EFFECTS WITHOUT EXACERBATING SIDE EFFECTS PROFILE IN RODENTS

Kazunori Suzuki*[,1], Akina Harada[1], Hirobumi Suzuki[1], Atsushi Nakatani[1], Clizia Capuani[2], Annarosa Ugolini[2], Mauro Corsi[2], Nidhi Kaushal[1], Konstantin Bobkov[1], John Vekich[1], Joseph Doyle[1], Haruhide Kimura[1]
*[1]Takeda Pharmaceutical Company Limited; [2]Aptuit, Inc.*

**Background:** Activation of indirect pathway medium spiny neurons (MSNs) via promotion of cAMP production is the principal mechanism of action (MOA) of current antipsychotics with dopamine D2 receptor antagonism. Phosphodiesterase 10A (PDE10A) inhibitors activate both direct and indirect pathway MSNs by increasing both cAMP and cGMP levels by inhibiting their degradation, which might be expected to promote activation of intracellular signaling similar to that of D2 antagonists in the indirect pathway MSNs. Thus, the activation of the indirect MSN pathway through the distinct MOA of these compounds raises the possibility of augmented pharmacologic effects with combined treatment.
In this study, we compared gene-regulation patterns in the indirect pathway MSNs induced by the PDE10A inhibitors T-773 and T-609, and the D2 antagonist haloperidol, using a cell-type-specific comprehensive gene expression analysis in Drd2-bacTRAP (translating ribosome affinity purification) mice. The pharmacologic effects of combined treatment with another PDE10A inhibitor, TAK-063, and clinically used antipsychotics, haloperidol (HAL) and olanzapine (OLA), were evaluated in multiple rodent models.
**Methods:** Male ICR mice, Drd2-bacTRAP mice, and Sprague-Dawley rats were used. The indirect pathway MSN-specific gene expression changes by T-773, T-609, and HAL were investigated using RNA sequencing of striatal samples of Drd2-bacTRAP mice. The activation of MSNs in rats was evaluated by measuring glutamate receptor subunit 1 phosphorylation (pGluR1) levels. An in vitro electrophysiological study on the corticostrial pathway in rats was conducted in a slice preparation. The activation of each MSN pathway was assessed by inducing genes as pathway-specific markers: enkephalin for the indirect pathway and substance P for the direct pathway. Suppression of MK-801- or methamphetamine (METH)-induced hyperactivity was assessed by measuring locomotor activity for 2 hours after administration of these stimulants to rats. Improvement of prepulse inhibition (PPI) was investigated in a MK-801-induced PPI deficit mouse model.
**Results:** Translational profiling in Drd2-bacTRAP mice treated with the PDE10A selective inhibitors, T-773 and T-609, and with HAL suggested regulatory of a largely overlapping signaling pathway by these compounds in the indirect pathway MSNs: 87% of the genes regulated by HAL were also regulated by both T-773 and T-609. Combined treatment with TAK-063 and either HAL or OLA produced an augmented effect on pGluR1 in the rat striatum. An electrophysiological study in rat brain slices indicated that TAK-063 enhanced synaptic responses to a similar extent in both direct and indirect pathway MSNs. Additional evaluation using MSN pathway-specific markers revealed that coadministration of TAK-063 with HAL or OLA additively activated the indirect pathway, but not the direct pathway. Combined treatment with TAK-063 (0.1 mg/kg p.o.) and either HAL (0.3 mg/kg p.o.) or OLA (3 mg/kg p.o.) at subeffective doses produced augmented effects on

METH- or MK-801–induced hyperactivity in rats and MK-801–induced PPI deficits in mice. TAK-063 at 0.1 mg/kg did not affect plasma prolactin levels and cataleptic response induced by HAL or OLA in rats.

**Discussion:** PDE10A inhibitors and HAL showed similar patterns of gene regulation in indirect pathway MSNs in mice. Combined treatment with TAK-063 and either HAL or OLA at subeffective doses produced significant antipsychotic-like effects but no augmentation of the plasma prolactin level and cataleptic response. Although further preclinical and clinical studies will be needed, TAK-063 may provide a novel mechanism as a PDE10A inhibitor for use as combination therapy in schizophrenia.

### S224. DELTA-9-TETRAHYDROCANNABINOL CHALLENGE IN CANNABIS USERS AND NON-USERS DIFFERENTIALLY AFFECTS BRAIN FUNCTION AND BEHAVIOR: AN FMRI STUDY OF DEVELOPMENT OF TOLERANCE

Marco Colizzi*[,1], Philip McGuire[1], Vincent Giampietro[1], Steve Williams[1], Mick Brammer[1], Sagnik Bhattacharyya[1]
[1]*Institute of Psychiatry, King's College*

**Background:** Cannabis use can induce acute and long-lasting psychosis and cognitive dysfunction. Some evidence suggests that the acute behavioral and neurocognitive effects of the main active ingredient in cannabis, (−)-trans-Δ9-tetrahydrocannabinol (Δ9-THC), might be modulated by previous cannabis exposure. However, this has not been investigated either using a control group of non-users, or following abstinence in modest cannabis users, who represent the majority of recreational users.

**Methods:** Twenty-four healthy men participated in a double-blind, randomized, placebo-controlled, repeated-measures, within-subject, Δ9-THC challenge study.

**Results:** Compared to non-users (N=12; <5 lifetime cannabis joints smoked), abstinent modest cannabis users (N=12; 24.5 ± 9 lifetime cannabis joints smoked) showed worse performance and stronger right hemispheric activation during cognitive processing, independent of the acute challenge (all P≤0.047). Acute Δ9-THC administration produced transient anxiety and psychotomimetic symptoms (all P≤0.02), the latter being greater in non-users compared to users (P=0.040). Non-users under placebo (control group) activated specific brain areas to perform the tasks, while deactivating others. An opposite pattern was found under acute (Δ9-THC challenge in non-users) as well as residual (cannabis users under placebo) effect of Δ9-THC. Under Δ9-THC, cannabis users showed brain activity patterns intermediate between those in non-users under placebo (control group), and non-users under Δ9-THC (acute effect) and cannabis users under placebo (residual effect). In non-users, the more severe the Δ9-THC-induced psychotomimetic symptoms and cognitive impairments, the more pronounced was the neurophysiological alteration (all P≤0.036).

**Discussion:** Previous modest cannabis use blunts the acute behavioral and neurophysiological effects of Δ9-THC, which are more marked in people who have never used cannabis.

### S225. A SYSTEMATIC REVIEW AND META-ANALYSIS OF PHARMACOLOGICAL INTERVENTIONS FOR REDUCTION OR PREVENTION OF WEIGHT GAIN IN SCHIZOPHRENIA

Sri Mahavir Agarwal*[,1], Zohra Ahsan[1], Jonathan Lockwood[1], Marcus Duncan[2], Hiroyoshi Takeuchi[1], Tony Cohn[1], Valerie Taylor[3], Gary Remington[1], Guy Faulkner[2], Margaret Hahn[1]
[1]*Centre for Addiction and Mental Health;* [2]*University of British Columbia;* [3]*Women's College Hospital*

**Background:** Weight gain is an extremely common problem in schizophrenia patients and is associated with morbidity and mortality. We conducted a Cochrane meta-analysis to determine the effects of pharmacological interventions aimed at reduction or prevention of weight gain in schizophrenia.

**Methods:** We searched the Cochrane Schizophrenia Group's Trials Register that is based on regular searches of CINAHL, BIOSIS, AMED, EMBASE, PubMed, MEDLINE, PsycINFO, and registries of clinical trials. There is no language, date, document type, or publication status limitations for inclusion of records in the register. We also searched reference sections within relevant papers, hand searched key journals, and contacted the first author of each relevant study and other experts to collect further information. We included all double blind randomized controlled trials examining any adjunctive pharmacological intervention for weight loss (treatment) or weight maintenance (prevention) in patients with schizophrenia or schizophrenia-like illnesses. We reliably selected, quality assessed and extracted data from studies. As endpoint and change data were combined in the analysis, mean differences (MD) of the change from baseline were calculated. The primary outcome measure was weight loss.

**Results:** Forty-four randomized controlled trials met the inclusion criteria for this review. Ten studies examined prevention of weight gain while on antipsychotics. Reboxetine may be slightly effective in preventing weight gain (Weight: MD -2.09 kg, 95% CI -3.12 to -1.05; participants = 111; studies = 3; BMI: MD -0.70, 95% CI -1.03 to -0.36; participants = 111; studies = 3) and but the quality of evidence is low as the studies are small and have all been conducted by the same group. We are uncertain about the other agents used in a preventive role because the quality of evidence is very low.

Thirty-four studies examined reduction of weight gain with pharmacological interventions. Metformin is effective in bringing about modest weight loss (MD -3.45 kg, 95% CI -4.92 to -1.98 kg; participants = 569; studies = 8; BMI: MD -1.32 kg/m2, 95% CI -1.84 to -0.81 kg/m2; participants = 606; studies = 9). This effect is probably stronger in first episode psychosis (FEP) patients (Weight: MD -5.18 kg, 95% CI -6.22 to -4.14 kg; participants = 214; studies = 3; BMI: MD -1.87 kg/m2, 95% CI -2.19 to -1.56 kg/m2; participants = 214; studies = 3) compared to chronic patients (Weight: MD -2.0 kg, 95% CI -3.0 to -1.0 kg; participants = 355; studies = 5; BMI: MD -0.73 kg/m2, 95% CI -1.04 to -0.42 kg/m2; participants = 392; studies = 6). Metformin probably decreases fasting insulin levels and insulin resistance as well. The frequency of adverse effects did not differ between metformin and placebo groups. Other agents that may reduce weight include aripiprazole, topiramate, H2 antagonists such as nizatidine, and sibutramine but the quality of the evidence is low or very low making the effects uncertain. Importantly, none of the adjunctive treatment strategies result in higher dropout rates. Other than higher reports of anxiety with aripiprazole, none of the adjunctive treatment strategies appear to result in worsening of mental status.

**Discussion:** Accumulating evidence supports the safe use of pharmacological interventions to achieve modest weight loss. Reboxetine may be slightly effective in preventing weight gain while metformin has the most evidence for use as treatment of weight gain in schizophrenia. The small number of studies, small sample size, and short study duration limits interpretation for other agents. Future studies that are adequately powered, with longer treatment duration, will be needed in evaluating the efficacy and safety of interventions for managing weight gain further.

### S226. A STUDY COMPARING WEIGHT GAIN FROM ALKS 3831 TO OLANZAPINE IN EARLY-ILLNESS YOUNG ADULTS WITH SCHIZOPHRENIFORM, SCHIZOPHRENIA, OR BIPOLAR I DISORDER

Adam Simmons*[,1], David McDonnell[1], Ying Jiang[1], Christine Graham[1], Bernard Silverman[1]
[1]*Alkermes, Inc.*

**Background:** Optimal pharmacological efficacy is crucial in first-episode and early-episode schizophrenia and bipolar I disorder. Olanzapine (OLZ) is a highly efficacious antipsychotic indicated for the treatment of schizophrenia and bipolar I disorder. However, the clinical utility of OLZ may be limited by a propensity to cause significant weight gain and increased metabolic side effects, especially for patients early in the course of their illness. ALKS 3831 is composed of a flexible dose of olanzapine (OLZ) and a fixed dose of 10 mg of samidorphan (SAM), formulated as a bilayer tablet. ALKS 3831 has been shown in Phase 1 and Phase 2 studies to result in significantly less weight gain than OLZ while delivering equivalent antipsychotic efficacy. This Phase 3, 12-week study, is designed to evaluate the effect of ALKS 3831 on body weight in young adults early in the course of diagnosis of a serious mental illness, including a schizophreniform, schizophrenia, or bipolar I disorder diagnosis.
**Methods:** This is an international (Austria, Germany, Ireland, Israel, Italy, Poland, Spain, UK, and USA) two-arm, double-blind, active-comparator–controlled, multicentre study (planned N=250) that started enrollment in 2017. Key inclusion criteria are a primary diagnosis of schizophreniform disorder, schizophrenia, or bipolar I disorder; a body-mass index (BMI) of ≥18.0 and ≤27.0 kg/m2; and meeting specific criteria for duration of illness and prior antipsychotic exposure. Patients with a bipolar I diagnosis must be in the manic phase. In the US sites, men and women must be aged ≥16 to <40 years at screening, and in Europe, aged ≥18 to <40 years. Exclusion criteria include diagnosis of additional psychiatric conditions and use of prohibited drugs.
**Results:** Patients will be randomised 1:1 to receive either OLZ or ALKS 3831 treatment for 12 weeks. ALKS 3831 (OLZ + SAM) and matched OLZ + placebo (OLZ + PBO) will be provided as bilayer tablets to be taken by mouth once daily and doses will include ALKS 3831 (OLZ/SAM) 5/10 mg, 10/10 mg, 15/10 mg, 20/10 mg, or (OLZ/PBO) 5 mg, 10 mg, 15 mg, or 20 mg. The primary endpoint will be percent change in body weight from baseline to Week 12. Secondary and exploratory endpoints will include the proportion of patients who gain ≥7% and ≥10% of baseline body weight, metabolic parameters (change in fasting lipids and glucose), body composition measured through bioimpedance, clinical global impression, and safety, pharmacokinetic, and pharmacodynamic parameters. Patients will be offered a supportive clinical care programme during the 12-week treatment period, and also receive a daily medication-adherence monitoring and reminder system (via smartphones).
**Discussion:** Patients who complete the study will have the option to participate in an open-label 2-year extension of ALKS 3831 (based on clinician and patient decision).
EudraCT number: 2017-000497-11; ClinicalTrials.gov identifier: NCT03 187769

### S227. A PROPOSED ALTERNATIVE BETWEEN DISCONTINUATION AND MAINTENANCE OF ANTIPSYCHOTICS: A GUIDED DOSE REDUCTION TRIAL FOR PATIENTS WITH REMITTED PSYCHOSIS

Chen-Chung Liu*,[1]
[1]*National Taiwan University Hospital and College of Medicine, National Taiwan University*

**Background:** Early intervention at the beginning of schizophrenia and related psychotic disorders can get better treatment response. Once symptoms subsided, the majority of patients wish to discontinue medications, yet currently the mainstream opinions still recommend maintenance antipsychotic therapy because non-adherence to medication is the most significant risk factor to predict a relapse. However, recent longitudinal studies assessing patients in community for a longer term found that discontinuation of antipsychotics might not necessarily be parallel to poorer functioning. Also there are studies suggesting a lower percentage of dopamine occupancy by antipsychotic is acceptable in stable patients with psychosis. To elucidate such discrepancies, a hypothetical compromised approach "guided dose reduction, but not aiming at discontinuation" was proposed and an observational clinical trial was initiated since July 2017.
**Methods:** Outpatients with schizophrenia-related psychotic disorders under remitted states will be recruited and randomized into guided dose reduction group (GDR, target n = 80) and maintenance treatment group (MTG1, target n = 40), and those who eligible to dose reduction yet willing to continue medication will serve as a naturalistic observation group (MTG2, target n = 40). Patients in the GDR will reduce no more than 25% of their current dose of antipsychotics and closely monitored every 4 weeks for at least 24 weeks before next dose reduction adjustment. Patients of both MTGs receive treatment as usual. All patients will be followed up for at least 2 years. The main outcomes of interests are differences in relapse rates, personal social performance, quality of life, drug-related adverse reactions, medication satisfaction, and neurocognitive functioning between groups. Patient's actual medication status will be monitored by keeping a log and therapeutic drug monitoring on selective antipsychotics. Patient's demographics and clinical variables will be taken to test whether these variables are related to outcomes during follow-up.
**Results:** Currently 26 patients have participated in this study, including 10 males and 16 females, with a Mean (SD) age 31.8(7) years old. Eleven of them were in GDR group, 10 in MTG1, and 5 in MTG2. Their baseline PANSS scores were 36.9(5.7), 37.4(7.9), 49.2(7.4), CGI-S scores were 1.7(0.6), 1.5(0.7), 2(0), and Personal Social Performance (PSP) scores were 82.2(7.9), 83.9(6.8), 77.8(2.3) in GDR, MTG1, and MTG2, respectively. So far one patient in the GDR group has resumed her original dose due to suspected early signs of relapse and no further worsening of symptoms noticed, while one patient of the MTG1 withdrew consent due to feeling unnecessary to receive comprehensive follow-up assessments. Most of patients endorsed no significant difference between ordinary dose and reduced dose at present time.
**Discussion:** During the first 4 months of this trial, we have not seen any unexpected happening yet. We will continue case recruitment and follow-up to test if the metaphor derived from Cantor's Set and Sierpinski Triangle can serve a valid model for our dose reduction trial and see if such a slow-paced guided dose reduction approach a feasible solution for the debates between medication discontinuation and maintenance.

### S228. TOWARD EARLY DETECTION OF TREATMENT RESISTANT SCHIZOPHRENIA: PREDICTIVE INFORMATION ON NON-RESPONSE TO ANTIPSYCHOTICS BY EVALUATION OF A FEW CLINICAL FACTORS: A STUDY BY ROC CURVE ANALYSIS AND CONFIRMATORY MULTIVARIATE ANALYSIS

Felice Iasevoli*,[1], Camilla Avagliano[1], Benedetta Altavilla[1], Annarita Barone[1], Mariateresa Ciccarelli[1], Luigi D'Ambrosio[1], Danilo Notar Francesco[1], Eugenio Razzino[1], Andrea de Bartolomeis[1]
[1]*University of Naples "Federico II"*

**Background:** Treatment Resistant Schizophrenia (TRS) is associated to poor prognosis and highly disabling course. Early detection of the condition is crucial to rapidly provide targeted interventions. The aim of this study was to evaluate whether it may be possible to distinguish TRS from Antipsychotic Responder Schizophrenia (ARS) patients on the basis of a limited number of measurable clinical factors.
**Methods:** 60 out of 182 eligible patients were included. A multistep diagnostic procedure to separate TRS from ARS was then used. Clinical parameters were recorded. Rating scales were administered, including: the Neurological Evaluation Scale (NES); the Positive and Negative Syndrome Scale (PANSS); the Heinrichs' Quality of Life Scale (QLS); the UCSD

Performance-Based Skills Assessment (UPSA); the Personal and Social Performance (PSP) scale and Specific Level of Functioning (SLOF).
We used the Receiver Operating Characteristic (ROC) curves analysis to distinguish between TRS and ARS. Confirmatory logistic regression and discriminant analysis were additionally used.
**Results:** Among clinical and demographic parameters, AUCs were significant for previous hospitalizations (AUC=.71; p=.004; SE= .068); antipsychotic dose (AUC=.73; p=.002; SE=.66); duration of illness (AUC=.67; p=.02; SE=.71) and NES score (AUC=.77; p<.0005; SE=.062). Moreover, significant AUCs were found for PANSS Negative subscale score (AUC=.68; p=.013; SE=.068); PANSS total score (AUC=.64; p=.05; SE=.071); QLS score (AUC=.73; p=.003; SE=.067); PSP score (AUC=.69; p=.012; SE=.68); all SLOF areas (AUC ranging from .76 to .68, p<.05), with the exclusion of Area4. A trend toward significance was found for Problem Solving (AUC=.63; p=.08). Among the whole significant variables, the highest specificity for diagnosis was found for NES score and previous hospitalizations (75% and 78.1%, respectively); the highest sensitivity for NES score (71.4%). Accordingly, Odds Ratio of being categorized as TRS were larger for NES score <21.5 (7.5), QLS score <57 (5.49), previous hospitalizations >1.45 and SLOF Area5 <43.5 (4.76 both).
Multivariate analysis supported results of ROC curve analysis. Stepwise logistic regression showed that the following variables were significant predictors of TRS/ARS status: previous hospitalizations, NES score, and antipsychotic dose among clinical variables ($\chi(3)=27.25$, $p<.0005$, Nagelkerke $R2=.48$); PANSS Negative subscale score among psychopathology variables ($\chi(1)=7.75$, $p=.005$, Nagelkerke $R2=.16$); QLS score among quality of life variables ($\chi(1)=7.91$, $p=.005$, Nagelkerke $R2=.16$); SLOF Area2 among social functioning variables ($\chi(1)=18.05$, $p<.0005$, Nagelkerke $R2=.34$).
The descriptive discriminant analysis function was significant for clinical variables, $\chi(6)=23.84$, $p=.001$. The most relevant discriminator variables in this group were NES score, antipsychotic doses, and previous hospitalizations. Discriminant function was also significant for SLOF variables $\chi(6)=17.67$, $p=.007$, with Area1 and Area3 scores ensuring the highest discriminative power. Discriminant function was only weakly significant for psychopathology and for quality of life variables (PANSS Negative subscale score and QLS score showed the highest discriminative power, respectively).
**Discussion:** Therefore, the evaluation of a few clinical factors may give solid and predictive information about patient potential to be responsive or non-responsive to antipsychotics. A patient exhibiting a combination of 2 or more lifetime hospitalizations; high NSS; high negative symptoms; low quality of life and psychosocial functioning has low possibility (less than approximately 20%, according to our data) to be responsive to antipsychotic agents.

### S229. CAN LONG-ACTING INJECTABLE PALIPERIDONE DOSING BE OPTIMIZED WITH PLASMA LEVEL MEASUREMENTS?

Robert Zipursky*[,1], Helen Huynh[2], Ofer Agid[3], Michael Kiang[3], Gary Remington[3]
*[1]McMaster University; [2]St. Joseph's Healthcare Hamilton; [3]Centre for Addiction and Mental Health (CAMH), University of Toronto*

**Background:** Most people with schizophrenia respond robustly to antipsychotic medication but are at very high risk of relapse if these medications are stopped. Long-term maintenance treatment with antipsychotic medication can dramatically reduce the risk of relapse. With long-acting injectable antipsychotic medication (LAI), adherence is documented which may account for superior efficacy in relapse prevention reported in some studies. It is known that plasma antipsychotic levels vary greatly across individuals with standard doses of LAIs. Establishing the lowest effective plasma levels for relapse prevention may also help in minimizing side effects that may contribute to problems with adherence. This study was carried out to describe the plasma paliperidone levels associated with clinical stability in patients receiving the LAI, paliperidone palmitate. We predicted that higher paliperidone plasma levels would be associated with lower subjective well-being and greater levels of sexual dysfunction.
**Methods:** Patients with clinical diagnoses of schizophrenia and schizoaffective disorder attending specialized schizophrenia outpatient clinics at St. Joseph's Healthcare Hamilton were invited to participate if they were receiving maintenance treatment with paliperidone palmitate. The study involved two visits, 3 to 4 weeks apart, on days that subjects were scheduled to receive consecutive injections of paliperidone palmitate. Plasma paliperidone levels and prolactin levels were drawn prior to the injection at Visit 1 and a second paliperidone levels was drawn at Visit 2. At Visit 1, a series of rating scales were also completed including the Subjective Well-being under Neuroleptic scale – Short version (SWN), the Changes in Sexual Functioning Questionnaire (CSFQ) and the Drug Attitude Inventory (DAI).
**Results:** Twenty-one subjects (11F/10M) provided informed consent for this study and had plasma paliperidone levels measured. Patients had been receiving LAI paliperidone for a mean of 18 months (SD = 11.4). Mean paliperidone levels at Visit 1 (n=21) and Visit 2 (n=18) were 34.9 ng/ml (SD = 20.0 ng/ml; range = 5.1–73.9 ng/ml) and 35.1 ng/ml (SD = 17.2 ng/ml; range = 9.0–67.5 ng/ml), respectively. Plasma paliperidone levels measured at Visit 2 were highly correlated with levels from Visit 1 (n=18; r = .89, p <.001). Plasma prolactin levels were correlated with levels of plasma paliperidone (n=21, r=0.56, p < .01). Lower scores on the CSFQ – Sexual Desire factor were associated with higher levels of paliperidone (n=19, r =-.61, p<.01) and prolactin (n=19, r=-.56, p <.01). Higher paliperidone levels were associated with more negative scores on the Drug Attitude Inventory (n=19, r=-0.49, p < .05). Plasma paliperidone levels were not associated with scores on the SWN (n=21, r=-.-02).
**Discussion:** In patients receiving maintenance treatment with paliperidone palmitate, plasma paliperidone levels varied approximately 15-fold. Higher paliperidone levels were associated with more negative attitudes towards medication and more severe deficits in sexual desire but not with subjective well-being. Many stable patients had plasma level close to the 20ng/ml level which in PET studies leads to 65% dopamine D2 receptor occupancy, a level reported to be associated with antipsychotic response. Our findings raise the possibility that maintaining patients at levels just above the 20ng/ml level may be sufficient for relapse prevention but may spare the adverse effects such as sexual dysfunction associated with higher plasma levels. These results suggest that measuring plasma levels in patients receiving paliperidone as a LAI may be of value in identifying the minimum effective dose for prevention of relapse and side effects.

### S230. LONG-TERM ANTIPSYCHOTIC MEDICATION IN SCHIZOPHRENIA: BENEFITS, RISKS AND FOLLOW-UP: DATA FROM FINNISH COHORT STUDIES AND SYSTEMATIC REVIEW

Matti Isohanni*[,1], Jouko Miettunen[1], Erika Jääskeläinen[1], Jani Moilanen[1], Anja Hulkko[1], Sanna Huhtaniska[1]
*[1]University of Oulu*

**Background:** Millions of people use antipsychotic medications. Thousands of clinicians (often non-psychiatrists) prescribe and monitor them every day. Existing research reports mostly favorable risk-benefit ratio during the first years of schizophrenia, but their risk-benefit ratio and maintenance efficacy in long-term is not clear.
Our aim was to:

1. analyze long-term antipsychotic use and its determinants in Finnish cohort samples, and
2. review the studies on benefits, risks, and follow-up and monitoring practices of long-term antipsychotic treatments.

**Methods:** 1. We used the data of population-based Northern Finland Birth Cohort 1966 (NFBC1966), and also Finnish therapeutic community data.

2. We performed a systematic literature search on long-term treatment effects, risks and monitoring of antipsychotic medication in schizophrenia.
**Results:** 1. In NFBC1966 in midlife, higher lifetime doses of antipsychotics were associated with alterations in brain morphometry, poorer neurocognition, and poorer clinical outcomes. Clinical follow-up was inadequate even in half of the schizophrenia cases. In therapeutic community cohort, maximal development of psychosocial care reduced the mean dose of antipsychotics in acute psychosis ward from 370 mg/day as chlorpromazine equivalents into 160 mg/day. 2. In the literature review, three main cornerstones in the high quality longitudinal use of antipsychotic medication were: a) high, evidence-based pharmacological quality, b) optimal adjuvant psychosocial therapies, c) sophisticated long-term prescription, monitoring and follow-up practices to minimize nonadherence and psychiatric and somatic failures.
In sum, antipsychotics are effective for acute and mid-term psychosis in prevention of relapses and excess mortality. Long term antipsychotic use especially in high doses may include major iatrogenic harms, as also poorly monitored withholding or discontinuing. When aiming for an optimal benefit-risk ratio and for balancing symptomatic, functional and somatic outcomes, the goal is to aim for lower ranges of effective dosing, as well as choosing an appropriate antipsychotic agent that causes minimal side effects, and to combine adjuvant psychosocial interventions in the treatment. The often recommended personalized smallest effective dose is not so simple but still a realistic strategy in current relapse prevention practices, where doses often are too large for safety reasons.
**Discussion:** Cohort-based register studies are useful in examining long-term medication effects although they contain a risk of residual confounding due to their observational design. However, randomized controlled trials in long, over 3–7 years of follow-up, are unrealistic.
The systematic literature review demonstrates major open or conflicting questions in risk-benefit ratio related to long-term outcomes. Nonadherence and attrition are key problems in sustained antipsychotic medication. Standardized prescription and monitoring practices (not so much studied) might improve medication adherence and also outcomes. Current clinical guidelines advise us based on studies from first years of schizophrenia. There are only few and weak patient-level predictors of successful tapering and discontinuation of antipsychotic medication.
In the future, clinical follow-up of medication can be improved by structured follow-up and planned continuity. Life span view of antipsychotic medication stresses careful documentation of doses, responses and harms, longitudinal planning and realization of medication as part of the whole treatment program, as well as individualized and tailored selection, dosing (dose as low as possible or minimal effective dose) and follow-up by a well-trained team.

### S231. THE ROLE OF DOPAMINERGIC AND GLUTAMATERGIC NEUROTRANSMISSION IN DELUSIONAL IDEATION AND SENSORY INFORMATION PROCESSING OF PATIENTS WITH SCHIZOPHRENIA IN COMPARISON TO HEALTHY HUMAN PARTICIPANTS

Wolfgang Strube*[,1], Graziella Quattrocchi[2], Simon Little[2], Louise Marshall[3], Alkomiet Hasan[1], Sven Bestmann[2]
*[1]University of Munich, LMU; [2]Sobell Department, UCL; [3]Wellcome Trust*

**Background:** The primary aim of this study was to generate neurobiological evidence regarding the impact of dopaminergic and glutamatergic neurotransmission on reasoning biases related to delusional ideation in patients with schizophrenia associated with impaired processing of sensory information. The proposed respective roles of these neurotransmitter systems have been encapsulated in the so-called dopamine- and glutamate-hypotheses of schizophrenia. From a behavioural perspective both reduced glutamate and enhanced dopamine levels are currently discussed as critical contributing factors to generate aberrant beliefs (glutamate) during information sampling and to generate confidence or expected precision (dopamine) during action selection. Hence, by modulating levels of glutamate and dopamine in the brain we hypothesized to induce reported impairments of patients with schizophrenia related todelusional ideation.
**Methods:** The study consisted of three aligned experiments: In the first two experiments a prospective interventional drug study was conducted with n=192 participants employing a randomized, placebo-controlled, double-blinded design on two parallel testing-groups, receiving either dopaminergic or glutamatergic neuromodulators: Experiment I: either 2.5mg haloperidol (D1/D2-receptor antagonist; HAL), 2.5mg bromocriptine (D2-receptor agonist; BRO), or placebo (PLC-1). Experiment II: either 120mg Dextromethorphan (NMDA-receptor antagonist, DXM), 250mg D-Cycloserine (NMDA-receptor agonist, CYC), or placebo (PLC-2). In the third experiment n=45 patients with schizophrenia (SZ) and n=45 healthy control participants (HC) matched for gender, age and IQ were investigated. All experiments employed a computerized (Matlab, Cogent) version of the Beadstask (Huq, Garety et al. 1988). In total participants processes 60 Beadstask trials subdivided into three levels of difficulty: (I) easy trials with a bias of 80–90% for one predominant bead color in a sequence, (II) difficult trials (60–70% bias), and (III) ambiguous trials (no bias, 50% likelihood). Additionally, the task consisted of three parts that were presented in a fixed order: an easy draws-to-decision condition, an easy probability estimates condition, and a difficult draws-to-decision condition.
**Results:** In accordance with foregoing studies, SZ patients showed significantly less draws to decision compared to HC (all $p \leq 0.038$). Explorative analysis across experimental conditions further revealed no significant differences for participants receiving DXM (NMDA-receptor antagonist) compared SZ patients (all $p \geq 0.090$), but obtained less draws to decision in the DXM group than all other groups. Whereas following HAL intervention the number of draws increased significantly compared to any other experimental group (all $p \leq 0.048$). Analyzing the probability estimates condition we quantified changes of probability estimates on an individual subject level whenever there was a change of bead color in a sequence (so called disconfirmatory evidence score, DES). In case of easy and difficult trial types we observed significantly higher DES scores in participants with SZ compared to HC ($p \leq 0.003$) and again obtained no differences between SZ and DXM ($p=0.037$).
**Discussion:** Our findings are supportive for a hypothesized relationship between neurotransmitter state alterations of glutamate and dopamine in patients with schizophrenia and the delusional ideation. Future analysis will focus on developing a computational behavioral model of cognitive processing of the Beadstask, implementing our neurobiological findings in order to further disentangle the neurobiological underpinnings of delusional ideation in patients with schizophrenia.

### S232. ALPHA7 NICOTINIC RECEPTOR AGONISTS REVERSE THE HYPERDOPAMINERGIC STATE IN THE MAM MODEL OF SCHIZOPHRENIA

Gilda Neves*[,1], Anthony Grace[1]
*[1]University of Pittsburgh*

**Background:** Most investigations into the pharmacology of schizophrenia have revolved around dopaminergic and glutamatergic neurotransmission; however, one neurotransmitter that has not received adequate attention is the cholinergic system. Indeed, several post-mortem, genetic and epidemiologic studies link specifically the alpha7 nicotinic receptor (nAChR) to schizophrenia, and the potential use of alpha7 modulators as a treatment strategy is an active field of research. Nevertheless, studies to date have been limited to normal animals rather than on a validated neurodevelopmental model of schizophrenia. Moreover, knowledge about the differential impact of orthosteric and allosteric modulators in vivo is lacking. Thus, we investigated the effects of alpha7 nAChR modulation on dopamine

(DA) neuron activity in the ventral tegmental area (VTA) in the methylazoxymethanol acetate (MAM) animal model of schizophrenia.
**Methods:** All experimental procedures were conducted according to NIH guidelines and were approved by University of Pittsburgh Institutional Animal Care and Use Committee. Sprague-Dawley pregnant dams were treated with MAM or saline on gestational day 17. Recordings of VTA dopamine neuron activity was performed on the male offspring at adulthood. The effects of four different drugs were evaluated: PNU282987 (full agonist), SSR180711 (partial agonist) NS1738 (type I positive allosteric modulator - PAM) and PNU120596 (PAM type II).
**Results:** Intravenous administration of alpha7 selective ligands did not induce a major change in the firing profile of spontaneously active DA neurons when dosed during dopamine neuron recording. PNU120596 increased in the number of active DA neurons found in the VTA of normal rats, their mean firing rate and percentage of spikes in bursts. In contrast, the full agonist PNU282987 and the partial agonist SSR180711 reduced the hyperdopaminergic tone in MAM rats, with a more prominent decrease in the number of DA neurons recorded in the lateral VTA. In order to investigate the drug site of action, both PNU282987 and SSR1800711 were infused into the ventral hippocampus (vHipp) and basolateral amygdala (BLA). After vHipp infusion, the alpha7 nAChR agonists significantly decreased the number of active DA neurons in MAM rats, with no significant impact in control rats. Once more, the effects were more robust in the lateral VTA. In contrast, the same drugs when infused directly into the BLA increased the number of spontaneously active DA neurons in the VTA of normal rats, but not in the MAM model.
**Discussion:** In summary, our results show that alpha7 nAChR positive modulators can affect midbrain dopaminergic neuronal activity in vivo in a state-dependent manner. Interestingly, alpha7 nAChR agonists counterbalanced the hyperdopaminergic state of MAM rats and this effect is partially mediated by their action in the vHipp. This effect is consistent with the potential use of alpha7 nAChR agonists for schizophrenia treatment and fits the current search for drugs able to control dopaminergic function acting in structures upstream from the dopamine receptor. The predominant inhibition of the lateral VTA points to a lower propensity to produce unwanted side effects in comparison to current employed antipsychotic agents. Our data show that drug effects can vary according to the basal level of activity of specific brain circuits and highlights the importance of using appropriated animal models to make inferences about potential therapeutic use of new neuropsychiatric drug candidates.

### S233. EVOLUTION OF THE FUNCIONING AND ATTITUDES TO MEDICATION IN A SAMPLE WITH EARLY PSYCHOSIS IN TREATMENT WITH ARIPIPRAZOLE ONCE-MONTHLY

Marta Soto*[,1], Adolfo Benito[1]
*[1]SESCAM*

**Background:** Patients with first-episode schizophrenia usually respond well to treatment, but relapse is frequent during the first years of the illness and may be associated with clinical deterioration A major concern in treating patients with schizophrenia is non-adherence with medication, with approximately 40% stopping within 1 year and 75% by 2 years. In addition to the impact on the illness, non-adherence creates serious social and psychological consequences. Although it is one of the potentially preventable causes of relapse, hospitalization, and poor outcome non-adherence is one of the most difficult problems to solve
Aripiprazole is an atypical antipsychotic with partial agonist activity at dopamine D2 receptors and a potentially less burdensome metabolic profile compared with other atypical antipsychotics. A once-monthly, long-acting injectable (LAI) formulation, aripiprazole once-monthly 400 mg (AOM 400), is approved for the treatment of schizophrenia
**Methods:** To assess the evolution of the functioning and attitude to medication in a sample of patients recently diagnosed of schizophrenia during one year of treatment with aripiprazole once-monthly (AoM)
Schizophrenic patients from three Mental Health units in the province of Toledo (Spain) were recruited. The inclusion criteria were an age between 18 and 65 years, a diagnosis of schizophrenia (based on the ICD-10 criteria) in the last 5 years, the start of treatment with AoM in last 6 months, and 12 months of treatment with oral antipsycohtics previosly to the AoM treatment and at least one relapsed in last 4 years. A series of demographic variables were recorded and the GAF (Global Assesment Functioning) scale was used to assess function, while the Drugs Attitude Inventory (DAI) scale was used to evaluate attiudes to medication. The scales were again applied 3, 6 and 12 months after the start of treatment.
**Results:** N=18 patients (12 males and 6 females), with a mean age of 29 years. There were 1 dropouts during the year of follow-up. The results showed an improvement in GAF score during the 12 months, manifesting from the third month (ANOVA, $p<0.05$). Likewise, statistically significant differences (ANOVA, $p<0.05$) were observed with the DAI scale for attitude to treatment; these results persisted over the year of follow-up and were manifest from the third month.
**Discussion:** First years of evolution in schizophrenia are determinants for the evolution of the illness. The correct adherence to medication is associated with an adecuated functioning
Poor adherence to treatment is a recognised is predictive of poor outcomes (relapse, aggressive behaviour, suicide, substance abuse) and increased risk of hospitalisation Guidelines suggest that long-acting antipsychotic medicines may be offered to people who would prefer such treatment or in cases where avoiding covert non-adherence to antipsychotic medication (either intentional or unintentional) is a clinical priority within the treatment plan.

### S234. ONE-YEAR OUTCOME AND USE OF CLOZAPINE IN FIRST-EPISODE SCHIZOPHRENIA

Petros Drosos*[,1], Kolbjørn Brønnick[2], Rune Kroken[3], Inge Joa[2], Jan Olav Johannessen[2], Tor Ketil Larsen[4]
*[1]Stavanger University Hospital; [2]TIPS – Centre for Clinical Research in Psychosis, Stavanger University Hospital; [3]Haukeland University Hospital, University of Bergen; [4]University of Bergen*

**Background:** The aim of this study is to examine the one-year outcome in a cohort of patients with a first-episode core schizophrenia diagnosis (schizophrenia, schizophreniform psychosis, schizoaffective disorder) and the use of clozapine in the non-remitted patients at one-year control.
**Methods:** The population studied is the patients who were included with a first-episode psychosis in the TIPS project in the period 01.01.2002-31.12.2010 and had a core schizophrenia diagnosis. We divided the patients into two groups according to their remission status at one-year follow up and compared their main characteristics. We then performed a digital search in the hospital´s journal of the non-remitted group for the words "clozapine" and "Leponex".
**Results:** Out of the 78 patients with first-episode core schizophrenia diagnosis included in the TIPS project during the examined period, 53 were continuously psychotic at one-year follow up. The one-year remission rate for our sample was therefore 32%. All of the non-remitted patients during the first year could be eligible for clozapine, but clozapine was considered to only 3 of them (5.7 %) and only two of them were offered clozapine. The mean number of periods with antipsychotic treatment in this group was four (4).
**Discussion:** The findings in our study show firstly a surprisingly low one-year remission rate for first-episode schizophrenia (32 %). This is much lower than what corresponding studies of the last years show. Our results also prove the underutilization of clozapine in non-remitted patients with a first-episode core schizophrenia diagnosis. Therefore, the clinicians did not follow the recommended guidelines for the treatment of schizophrenia. The possible reasons for this low use of clozapine will be discussed, but it was not possible to verify them as there was not found any relevant information in the patients' files.

### S235. MAINTENANCE TREATMENT WITH ANTIPSYCHOTIC DRUGS IN SCHIZOPHRENIA – SYSTEMATIC REVIEW AND NETWORK META-ANALYSIS

Johannes Schneider-Thoma*,[1], Stefan Leucht[1]
[1]*Klinikum rechts der Isar, Technical University of Munich*

**Background:** Antipsychotic drugs are the mainstay of the maintenance treatment of schizophrenia and they are known to prevent psychotic relapse as compared to placebo. Nevertheless, insufficient efficacy and reoccurrence of psychotic symptoms (relapse) are frequent phenomena. Furthermore, side effects of antipsychotic drugs can be very unpleasant and even dangerous, particularly, when drugs are applied over a long time. Nowadays, many different antipsychotic substances are available and they can be used in oral or long-acting-injectable applications. However, the comparative efficacy of the different drugs in preventing relapse as well as the efficacy in specific domains of the disease (such as positive and negative symptoms) over the long term is only know in parts. Also concerning side effects it is not clear for a lot of drugs which substance should be preferred over another. The novel method of network-meta-analysis provides the possibility to use indirect evidence to compare drugs for which no direct comparison is available. Moreover, hierarchies of drugs can be created with this method, that show which drug is the best, second best, and so on, for individual outcomes.
**Methods:** We are conducting a network-meta-analysis of randomized controlled trials (RCT) in patients with schizophrenia or schizoaffective disorder. To identify eligible studies, we searched the register of the Cochrane Schizophrenia group, the most comprehensive database of clinical trials in schizophrenia. RCTs comparing antipsychotic drugs with each other or placebo are included. We focus hereby on the clinically most important newer and older antipsychotic drugs – as identified by a survey of international schizophrenia experts. The primary outcome is patients with a psychotic relapse at any time. Relapse at specific time-points as well as several other efficacy and tolerability parameters will be evaluated as additional outcomes. We include only trials of at least 3 months in duration, conducted with patients in a stable state of the disorder. Special attention is paid to the question if populations of different studies are comparable. This is of particular importance for network-meta-analysis because this method is based on the assumption of transitivity, i.e. that each patient in the analysis would have been in principle eligible for each study in the network.
**Results:** All so called second-generation antipsychotic drugs as well as several first-generation antipsychotics (list provided on the poster) were identified as clinically important drugs. A systematic literature search including the generic names of these drugs as well as search terms for maintenance treatment and stable condition found 3562 references. Screening of title and abstracts resulted in 1188 references referring to potentially eligible studies. In the ongoing full-text-screening and cross-referencing process 136 included studies are identified so far (complete search results presented on the poster). More detailed assessments of study characteristics are warranted to decide which studies are eligible to be included in the network-meta-analysis, i.e. to fulfill the transitivity criteria from a clinical point of view.
**Discussion:** Studies examining antipsychotic drugs for maintenance treatment differ in inclusion criteria for participants, in criteria for stable condition and also in criteria for psychotic relapse. Therefore, some official maintenance studies may not be eligible for network-meta-analysis. However, other studies, focusing not on maintenance but on other outcomes, may fulfill the transitivity criteria. The resulting problems for conducting network-meta-analysis as well as the reasoning for inclusion and exclusion of studies will be discussed.

### S236. IS MAINTENANCE TREATMENT NEEDED WHEN THE FIRST EPISODE OF PSYCHOSIS IS NOT DUE TO SCHIZOPHRENIA?

Gbolahan Odejayi*,[1], Robert Zipursky[1]
[1]*McMaster University*

**Background:** Debate continues about how long maintenance treatment should be continued following a first episode of psychosis (FEP). Resolving this question requires an understanding of the risk of recurrence which would be expected to vary as a function of the underlying cause of the psychosis. The range of diagnoses that may present as a FEP include schizophrenia and related schizophrenia spectrum disorders, bipolar mania, bipolar and unipolar depression, substance-induced psychosis, and unspecified psychotic disorders. The majority of FEP patients will receive the diagnosis of schizophrenia or bipolar disorder for which the 1- year risk of illness recurrence is estimated at 77% and 41%, respectively. We reviewed the literature in order to estimate the risk of relapse and the risk of developing a primary psychotic disorder following a FEP due to other diagnoses.
**Methods:** We conducted a primary literature review using Medline and PubMed. We included the following search terms: first episode, relapse, recurrence, depression with psychosis, psychotic depression, mania with psychosis, substance induced psychosis and psychosis. We included prospective and retrospective studies including those that involved medication discontinuation or naturalistic follow-up to determine the risk of recurrence following a FEP. We also reviewed the literature to determine the likelihood that FEP with these diagnoses would transition to a primary psychotic disorder (schizophrenia spectrum disorder or major mood disorder) for which published rates of recurrence would apply.
**Results:** Two studies were identified which reported on the recurrence rate following a first episode of psychotic depression. Recurrence rates ranged from 27% at eight months to 80.6% at a mean of 32 months. An additional study found that following a first episode of psychotic depression, 29.9% and 14.3% of patients were diagnosed with schizophrenia and bipolar disorder, respectively, at 10-year follow-up. The risk of developing a primary psychotic disorder following a first episode of substance-induced psychosis has been investigated in three studies which reported rates of conversion to a primary psychotic disorder of 25% at one year, 25% at 10 years and 32% at 20 years. The risk of developing a primary psychotic disorder following a cannabis-induced psychosis has been investigated in three studies which reported rates of conversion of 44.5% at three years, 46% at eight years, and 47.4% at 20 years. Patients with a first episode of unspecified psychosis have been reported in a single study to have a 73.7% risk of developing a primary psychotic disorder at 10 year follow-up.
**Discussion:** The risk of illness recurrence following a FEP not initially diagnosed as a schizophrenia spectrum or bipolar disorder was found to vary by both initial diagnosis and by follow-up duration. Psychotic depression, substance-induced psychosis and other unspecified psychoses were all associated with either substantial risks of illness recurrence or development of a primary psychotic disorder. The risk of illness recurrence following medication discontinuation has not been established for these disorders as many of these studies included patients whether they were on or off of their prescribed medications. Clinical recommendation should be informed by future research on recurrence rates with and without maintenance medication for the different causes of FEP. In the meantime, patients with a FEP and their family members should be fully informed about the risk of illness recurrence and development of a primary psychotic disorder when considering any trial of medication discontinuation.

### S237. THE ACCEPTANCE, FEASIBILITY AND PRELIMINARY EFFECTS OF DYNAMIC INTERACTIVE SOCIAL COGNITION TRAINING IN VIRTUAL REALITY (DISCOVR): A PILOT STUDY

Saskia Nijman*[,1], Wim Veling[2], Chris Geraets[2], Marieke Pijnenborg[3]
*[1]GGZ Drenthe; [2]University Medical Center Groningen; [3]GGZ Drenthe, Rijksuniversiteit Groningen*

**Background:** Many people with psychotic disorder experience problems in social functioning, such as finding and maintaining jobs and relationships, which have been shown to be strongly related to deficits in social cognition. A class of interventions called Social Cognition Training (SCT) aims to improve social cognition through practice and strategy training. SCT has been shown to have positive effects on social cognition. (Social) cognition training, however, is known to optimally translate to functional skills when it is applied to and integrated with different areas of daily life. To promote the transfer of training skills to functional domains, it may therefore be beneficial to provide SCT in virtual reality (VR), since it closely resembles real-life social situations. VR is highly realistic and interactive, allowing for practice of social situations in ecologically valid environments. VR is also controllable, allowing for personalization of situations and difficulty level. In the present study, we tested the acceptance and feasibility of a newly developed VR SCT called 'DiSCoVR' (Dynamic Interactive Social Cognition Training in Virtual Reality).
**Methods:** Twenty-two individuals with a psychotic disorder were recruited from three mental health institutions in the Netherlands. All participants received a VR SCT, which was aimed at three domains: 1) emotion perception (identifying virtual characters' emotions in a virtual street); 2) social perception and theory of mind (understanding social situations and the thoughts, emotions and behavior of virtual characters); and 3) practicing social interactions with a virtual character. The intervention strongly emphasized practice with social situations in VR between, and with, virtual characters. Participants also learned strategies to cope with difficulties they experienced in social situations. Participants were assessed at baseline and post-treatment. Acceptance of the intervention was evaluated at post-treatment using a questionnaire. Social cognition was also assessed (emotion perception, social perception and theory of mind) using video/photo tasks and stories. Finally, psychotic symptoms, social anxiety, paranoia, self-esteem and depression were measured using an interview and questionnaires.
**Results:** The results of this pilot study will be presented, focusing on the findings regarding acceptance and feasibility, but also social cognition and other secondary outcome domains.
**Discussion:** The implications of the findings of the pilot study will be discussed in the context of the preparation of a randomized controlled trial of DiSCoVR (for example, necessary alterations to the protocol and/or VR software). Plans for this randomized controlled trial will be discussed.

### S238. DEVELOPMENT OF SELF-STIGMA INVENTORY FOR THE RELATIVES OF THE PATIENTS WITH SCHIZOPHRENIA: RELIABILITY AND VALIDITY STUDY IN TURKEY

Mustafa Yildiz[1], Aysel Incedere*[,1], Fatma Kiras[1], Fatma Betül Abut[1], Ayşe Kircali[1], Kübra Ipçi[1]
*[1]Kocaeli University*

**Background:** Stigmatization refers to a disrespectful attribution to a person account of being considered as outside of society's norms. Stigmatization happens not only towards the mentally ill patients but can also occur against the relatives of the patients. Researches revealed that relatives of the patients hide this disease from other people, ashamed of their patients, and feel excluded from others. They may choose to conceal the disease or even the name of the illness. This situation is referred as self-stigma of the relatives. It is important to develop an instrument to evaluate and assess the self-stigma of the relatives since it increases their burden and negatively affects the prognosis of the illness. The purpose of this study was to develop a culturally-sensitive and user-friendly inventory for the assessment of self-stigma of families.
**Methods:** After examining the studies that investigate self-stigma and internalized stigma in people with mental illness, 25-item inventory was formed. Focus group interviews were conducted with a sample of 18 relatives of the patients with schizophrenia, and the items were reviewed and rephrased into more comprehensible and relevant statements for the relatives. Consequently, the inventory was finalized with 19 items. A pilot study was carried out with 15 relatives, and the inventory was reevaluated in terms of its comprehensibility and usability. One hundred and six relatives of the patients with schizophrenia and schizoaffective disorder were given a sociodemographic form, Self-Stigma Inventory for Relatives (SSI-R), Beck Depression Inventory (BDI), Beck Hopelessness Scale (BHS), Rosenberg Self-Esteem Scale (RSES), and Zarit Caregiver Burden Scale (ZCBS). For reliability analyses; internal consistency coefficient, item-total correlation, and split-half reliability were calculated. For validity analyses; explanatory factor analysis and convergent validity were assessed.
**Results:** The sample was consisted of 106 relatives whose 52% were female, 77% were married, and the level of education was 9 years.
Cronbach's alpha coefficient for SSI-R total score was calculated as 0.87, while Cronbach's alpha scores for subscales were found between 0.82 and 0.83. Split-half reliability coefficient was 0.79. For factor analysis, Kaiser-Meyer-Olkin value was found as 0.801 and Barlett test was significant ($p<0.001$). In explanatory factor analysis, 5 factors were detected whose eigenvalue was greater than one and the factors could explain 73% of the total variance. When the scree plot was examined, it was observed that the slope suddenly decreased and changed after the third factor. For this reason, varimax rotation was applied with three factors. Consequently, 3 factors (perceived incompetency, internalized stereotypes and social withdrawal, concealment of the illness) were detected and they could explain 66% of the total variance. Five items were omitted since they had lower factor value than 0.40. On the last form, first factor included 6 items, second factor had 5 items, and third factor had 3 items.
SSI-R was correlated with Beck Depression Inventory ($r=0.20$, $p<0.05$), Beck Hopelessness Scale ($r=0.19$, $p<0.05$), Zarit Caregiver Burden Scale ($r=0.41$, $p<0.001$), and Rosenberg Self-Esteem Scale ($r=-0.23$, $p<0.05$).
**Discussion:** This study shows that the SSI-R is a reliable and valid instrument on assessing the stigmatization in families. The scale is easy-to-use with its 14 items. It can be considered as a valuable instrument to use for research and therapeutic purposes.

### S239. PREDICTIVE ACCURACY FOR WORK OUTCOME IN PATIENTS WITH SCHIZOPHRENIA

Chika Sumiyoshi*[,1], Haruo Fujino[2], Tomiki Sumiyoshi[3], Hidenaga Yamamori[4], Noriko Kudo[5], Hirotsugu Azechi[5], Michiko Fujimoto[4], Yuka Yasuda[4], Kazutaka Ohi[6], Ryota Hashimoto[4]
*[1]Fukushima University; [2]Oita University; [3]Translational Medical Center, National Center of Neurology and Psychiatry; [4]Osaka University Graduate School of Medicine; [5]Osaka University; [6]Kanazawa Medical University*

**Background:** Functional capacity (i.e., what a person can do under optimal conditions) may not be directly transferred to functional performance (i.e., what a person actually does in a real-world situation) in patients with schizophrenia due to internal or external variables (Bowie et al., 2006). This discrepancy would be larger for relatively high functioning patients compared to those with low functional status, because of a greater likelihood of being exposed to real-world settings in the former group. The purpose of this study was to determine whether prediction of work outcomes would become less reliable in patients who work for long hours than those who do not.

**Methods:** Subjects: One-hundred and thirty-seven Japanese patients meeting DSM-IV-TR criteria for schizophrenia and 156 healthy adults entered the study. The study was approved by the Ethics Committee of Osaka University.

Assessment: Current and premorbid intelligence (WAIS-3 and Japanese version of the Adult Reading Test) and functional outcomes (the UCSD Performance-Based Skills Assessment-Brief and the Social Functioning Scale Individuals' version Modified for MATRICS-PASS) were assessed. The Positive and Negative Syndrome Scales (PANSS) and the Schizophrenia Quality of Life Scale Japanese version were also for patients. Total work hours per week, obtained from the Social Activity Assessment, was used as a measure of work outcome

Analyses: Four separate multiple logistic regression analyses were conducted to predict work outcomes. Independent variables were found to be significant by means of the group comparisons. Dependent variable, i.e., the number of work hours, was dichotomized by a criterion of 0, 10, 20, or 30 hours/week. At each level, patients were classified into either the above (=1) or the below (=0) criterion (observed outcomes). Estimated probabilities were calculated using factors that remained significant in regression models (intelligence decline, psychiatric symptoms, and social function). Predictive accuracy was calculated by summing the ratios correctly classified (i.e., a patient's observed outcome=1[0] and the estimated probability > 0.5 [< 0.5]). In addition, frequency distributions for observed outcomes were generated on the axis of estimated probabilities to examine the characteristics of correct classification.

**Results:** Overall, estimation was more accurate at higher criteria, yielding 80–87% accuracy at 20 or 30 hours/week criteria (c.f., 67–70% at 0 or 10 hours/week criteria). Distributions for the observed outcomes=0 had the peaks at the part of the estimate probabilities < 0.5, while those for observed outcomes=1 were relatively uniform, lacking the peaks at the part of estimated probabilities >0.5. These results indicate that the classification was less accurate in patients who exceeded the criteria (observed outcomes=1) compared to those who did not (observed outcomes=0).

**Discussion:** The current study showed that prediction for work outcome was less reliable in patients who attained relatively better work outcome. One of the reasons would be sextrinsic variables, such as local economy, welfare services, and stigma, may intervene between functional capacity and work outcome (Buchanan et al., 2005). Also, some of high functioning patients may have better insight into their work capacity (Gould et al., 2013), which may refrain them from engaging in long-hour work.

**References:**

1. Bowie CR et al. Am J Psychiatry. 2006;163:418–425.
2. Buchanan RW et al. Schizophr Bull. 2005;31:5–19.
3. Gould F, et al. Psychiatry Res. 2013;207:19-2.

### S240. DETERMINANT FACTORS OF REAL-WORLD FUNCTIONING IN SCHIZOPHRENIA

Leticia González-Blanco*,[1], M Paz Garcia-Portilla[1], Leticia Garcia-Alvarez[1], Lorena De La Fuente-Tomas[1], Pilar Saiz-Martinez[1], Celso Iglesias[1], Ana Coto[1], Julio Bobes[1]
[1]*University of Oviedo*

**Background:** Negative, cognitive and depressive symptoms, as well as physical comorbidities, have a great impact on the real-world functioning in patients with schizophrenia (SZ) (1, 2, 3). However, not all the studies have employed accurate psychometric instruments to assess these symptoms, nor have all these factors been studied simultaneously.

The aim of the current study is to analyze the determinants of functionality in SZ measured by the Personal and Social Performance (PSP) scale, and considering not exclusively psychopathological and cognitive variables, but also aspects related to physical health and inflammation.

**Methods:** Sample: 73 outpatients with SZ, duration of illness ≤10 years, under stable maintenance treatment [mean age (31.7 ± 6.5), males (61.6%)].
Clinical variables: PANSS, CGI-Severity, Clinical Assessment Interview of Negative Symptoms (CAINS) -Motivation/Pleasure (MAP) & Expression (EXP) domains-, Brief Negative Symptom Scale (BNSS), Calgary Depression Scale (CDS), MATRICS Consensus Cognitive Battery (MCCB), PSP.
Biological variables: Glucose, cholesterol, LDL, HDL, triglycerides, TSH, prolactin, insulin, uric acid, alkaline phosphatase (APh), C-reactive protein (CRP), TNF-α, interleukin(IL)-6, IL-2, IL-1β, IL-1RA, homocysteine, HT (% hemolysis), lipid peroxidation (LPO), catalase.
Pearson correlations were performed to select variables significantly related to PSP scores which were later included in stepwise multiple linear regression analyses. Age, sex, education, smoking, alcohol use, BMI, antipsychotic equivalent doses and other confounding factors were considered.

**Results:** Final model for PSP total score (R2=0.778, F=45.564, p<0.001) identified that CGI-Severity (β= -0.279), PANSS-NM (negative Marder Factor) (β = -0.218), Asociality subscale of BNSS (β= -0.383) and IL-2 (β= -0.269) were significant predictors.
Predicting variables included in regression models for specific PSP domains:

- Self-care (R2=0.661, F=22.947, p<0.001): PANSS-NM (β=0.458), Avolition subscale of BNSS (β=0.248), IL-2 (β=0.221), APh (β=0.201).
- Useful activities (R2=0.563, F=42.473, p<0.001): CGI-S (β=0.245), Avolition (β=0.557).
- Social relationships (R2=0.731, F=56.998, p<0.001): Asociality (β=0.578), PANSS-GP (β=0.276), CAINS-EXP (β=0.178).
- Aggresive behaviour (R2=0.335, F=16.892, p<0.001): PANSS-P (β=0.408), CDS (β=0.273).

**Discussion:** 1.Negative symptoms are the most important determinants of a deficit in the real-world functioning in SZ, especially "asociality."
2."Apathy" has a negative impact on self-care and useful activities domains.
3.Proinflammatory cytokine IL-2 marks poor functionality.

**References**

1. Harvey (2014). Assessing disability in schizophrenia: tools and contributors. J Clin Psychiatry.
2. Strassnig et al. (2015). Determinants of different aspects of everyday outcome in schizophrenia: The roles of negative symptoms, cognition, and functional capacity. Schizophr Res.
3. Menendez-Miranda et al. (2015). Predictive factors of functional capacity and real-world functioning in patients with schizophrenia. Eur Psychiatry.

### S241. COMBIMOD: A FRENCH INTEGRATIVE PROGRAMME IN PATIENTS WITH SCHIZOPHRENIA FOCUSING ON RECOVERY: PRELIMINARY RESULTS

Corentin Tarquis*,[1], Audrey Tanguy[2], sarah-Lise Farhat[1], Christophe Hochard[1], Corinne Bismuth[1], Corinne Gautier[1], Thierry Lambert[1], Sandrine Orens[1], Trang Ton[1], Marie-Cécile Bralet[1]
[1]*Unité CRISALID CHI Clermont de l'Oise;* [2]*AP-HP Paris*

**Background:** Cognitive remediation is a key tool in rehabilitation programs but not sufficient recovery. Meta-analyses in cognitive remediation insist on combinating programs in schizophrenia (Wykes and al.,2012; Lindenmayer and al., 2013). Futhermore, other subjective and clinical factors must be taking account in order to create a personalized rehabilitation program facilitating skills transfer in an ecological context. CRISALID (Center of cognitive rehabilitation and cognitive remediation), Clermont de l'Oise psychiatric department (Hauts de France area, France) offers an integrative care program, COMBIMod; it combines several modules of therapeutic education and of cognitive remediation, after an individual assessment, clinical, cognitive and functional (quantitative and qualitative) of each patient. A personalized and contractualized project of rehabilitation is then created with all the care partners around the patient. Objectives are: first to present COMBIMod with the description of each integrative education therapeutic programs based on cognitive deficits; then to proceed to a statistical neuropsychological comparative analysis. We postulate that using an integrative and ecological approach including remediation cognitive technics and ecological psychoeducation programs, can improve cognitive deficits.
**Methods:** First, we described all the steps to enter COMBIMOD program since the global personalized assessments to the creation of an individualized program including cognitive remediation (Neurocognition and Social-cognition) and therapeutics education programs (MODip, MODen). We illustrated this program with patients verbatim. Then, we recruited 15 stabilized patients with schizophrenia according to DSM-IV-TR criteria. All the patients were assessed using neuropsychological tests at the baseline and after completed the program. We used Wilcoxon test analysis with level of significancy < 0,05.
**Results:** We found significant improvement in some visuospacial memory test (p< 0,02) and executive functions (semantic verbal fluency: p<0,02; alphabetical verbal fluency: p<0,01; TMTB: p<0,003). Verbatim show some impact on the daily living and cognitive perceptions of the patient.
**Discussion:** These preliminary results confirm the importance of an integrative rehabilitation program including personalized assessments, motivation working, mixed cognitive remediation and psychoeducation programs. In order to facilitate ecological skills transfer, rehabilitation program should focus on subjective and will assessments. Further studies must be conducted in a larger cohort to better understand recovery

### S242. INTERNALIZED STIGMA AND ITS ASSOCIATION WITH CLINICAL SYMPTOMS AND PREMORBID ADJUSTMENT IN STABLE SCHIZOPHRENIA

Silvia Pardeller*[,1], Beatrice Frajo-Apor[1], Georg Kemmler[1], Annasara Meola[2], Fabienne Wartelsteiner[1], Wolfgang Fleischhacker[1], Alex Hofer[1]
*[1]Medical University Innsbruck; [2]University School of Medicine Federico II*

**Background:** Previous studies have shown that internalized stigma, i.e., the inner subjective experience of stigma resulting from applying negative stereotypes and stigmatizing attitudes to oneself, may impact negatively on schizophrenia patients' quality of life, hope, and self-esteem and hinder the recovery process. The aim of the current study was to investigate to what extent patients' internalized stigma correlates with both clinical symptoms and premorbid adjustment.
**Methods:** We recruited patients with schizophrenia on an out-patient basis. Diagnoses were confirmed with the Mini International Neuropsychiatric Interview (M.I.N.I.). Internalized stigma was assessed by the Internalized Stigma of Mental Illness (ISMI) scale. Psychopathology was assessed by the Positive and Negative Syndrome Scale (PANSS), which was divided into five factors according to Wallwork et al.: positive, negative, disorganized/concrete, excited, and depressed. In addition, the Premorbid Adjustment Scale (PAS) was used, which covers two discrete areas of functioning: school functioning (scholastic performance and adaption to school) and social functioning (sociability and withdrawal, peer relationships, and capacity to establish sociosexual relationships).
**Results:** So far, a total number of 69 patients (49.3 males, 50.7 females) with a mean age of 44.9 ± 10.4 years took part in this study. The mean duration of illness was 15.0 ± 10.5 years, the mean PANSS total score was 56.2 ± 16.9. With the exception of the Wallwork-factor "excited" (no significant relationship) clinical symptoms correlated negatively with the ISMI total score. Moreover, a negative association was seen between premorbid social functioning and internalized stigma, whereas no correlation was found between premorbid school functioning and the ISMI total score.
**Discussion:** This study illustrates the complexity of factors that influence internalized stigma in stable outpatients with schizophrenia. Next to replicating earlier reports on positive associations between residual symptoms of the disorder and internalized stigma our observations point to the relevance of premorbid social functioning in this regard.

### S243. EFFECTS OF A VIRTUAL REALITY SOCIAL TRAINING INTERVENTION ON LONELINESS AND SOCIAL COGNITION IN PATIENTS WITH SCHIZOPHRENIA

Laura Hieber Adery*[,1], Megan Ichinose[1] Lénie Torregrossa[1], Heathman Nichols[1], Dayi Bian[1], Joshua Wade[1], Eric Granholm[3], Nilanjan Sarkar[1], Sohee Park[1]
*[1]Vanderbilt University; [2]UCSD*

**Background:** Deficits in social cognition and on social perception tasks are well studied and widely found in populations with schizophrenia. In addition, our work consistently replicates findings that individuals with schizophrenia report severe loneliness, significantly higher than healthy matches. Loneliness is a chronic, gnawing condition that induces distress and impedes life satisfaction and function across the spectrum of mental health. We also find social isolation impedes interpretation of social information and may lead to socio-perceptual deficits.
The present study examines the effectiveness of a novel, adaptive virtual reality simulated social exposure training intervention (see Bekele et al, 2016) in both decreasing feelings of loneliness and improving social cognitive function in individuals with schizophrenia. We investigate baseline relationships between social isolation, loneliness and social cognition abilities, as well as pre to post intervention changes in function and subjective social well-being.
**Methods:** Fifteen medicated SZ outpatients completed 10 virtual reality social skills training sessions over the course of 5 weeks. Training sessions depicted three naturalistic social scenarios in which participants were instructed to complete 12 total social "missions" to obtain information from VR avatar characters. Prior to training and following the final training session, participants were assessed using the CogState Brief Schizophrenia Battery Social Emotional cognition task and rated loneliness using the UCLA Loneliness Scale. Independent raters conducted pre- and post-training clinical interviews to assess changes in participants' levels of positive, negative, and overall psychiatric symptoms
**Results:** Greater overall psychiatric symptoms were significantly correlated with higher levels of experienced loneliness, consistent with previous findings. There was a significant improvement in social emotional cognition accuracy, and a trend-level reduction in loneliness from pre-training to post-testing following social VR training.
**Discussion:** Previous research indicates that individuals higher on the psychosis spectrum perform worse at social cognition and social perception tasks. Our own research indicates that individuals higher on the psychosis spectrum also endorse higher levels of social distress via social isolation and loneliness. The present study attempts to enhance social cognitive and interpersonal abilities of individuals with schizophrenia while decreasing loneliness by strengthening social bonds and skills using a virtual reality training game. We find that following 10 sessions of VR social training, accuracy on measures of social cognition is improved significantly, however loneliness is reduced non-significantly. These initial results demonstrate potential feasibility of a novel VR social skills training game for improving social experience for patients with schizophrenia.

### S244. CHARACTERIZING OUTCOMES OF CLINICAL HIGH-RISK NON-CONVERTERS USING GROUP-BASED TRAJECTORY MODELING

Dana Allswede*[,1], Tyrone Cannon[1], Jean Addington[2], Carrie Bearden[3], Kristin Cadenhead[4], Barbara Cornblatt[5], Daniel Mathalon[6], McGlashan Thomas[1], Diana Perkins[7], Larry Seidman[8], Ming Tsuang[4], Elaine Walker[9], Scott Woods[1]
*[1]Yale University; [2]University of Calgary; [3]University of California, Los Angeles; [4]University of California, San Diego; [5]The Zucker Hillside Hospital; [6]University of California, San Francisco; [7]University of North Carolina; [8]Harvard Medical School; [9]Emory University*

**Background:** The development of the clinical high-risk (CHR) prodromal criteria has facilitated advancement in understanding conversion to psychosis and has provided opportunities for early intervention and treatment for these individuals. However, the majority of CHR cases do not meet full criteria for conversion, yet continue to experience clinically significant symptoms and impairment in daily functioning. It is likely that many of these individuals would also benefit from additional intervention and treatment, but the outcomes and needs of these "non-converters" are not well characterized. Identifying common longitudinal patterns of symptoms and functioning of non-converters would support the identification of individuals who continue to require treatment and tailoring of services to their specific needs.
**Methods:** We used group-based trajectory modeling to identify common longitudinal symptom and functioning trajectories among CHR cases (N=561) in the second phase of the North American Prodrome Longitudinal Study (NAPLS2). Covariant trajectories of symptoms (including positive, negative, disorganized, and general) and functioning (including role and social) were examined. Models were tested for replicability in an independent sample of CHR cases (N=291) from the first phase of NAPLS (NAPLS1).
**Results:** We identified a subgroup of individuals who exhibited symptom remission and functioning within the normal range, as well as at least two additional subgroups that exhibited different patterns of ongoing, clinically significant symptoms and functional deficits.
**Discussion:** We are currently investigating the validity of these subgroups by assessing their association with a variety of risk factors and biomarkers.

### S245. LOWER- AND HIGHER-LEVEL SOCIAL COGNITIVE FACTORS ACROSS INDIVIDUALS WITH SCHIZOPHRENIA SPECTRUM DISORDERS AND HEALTHY CONTROLS: RELATIONSHIP WITH NEUROCOGNITION AND FUNCTIONAL OUTCOME

Lindsay Oliver*[,1], John Haltigan[1], James Gold[2], George Foussias[1], Pamela DeRosse[3], Robert Buchanan[2], Anil Malhotra[3], Aristotle Voineskos[1]
*[1]Centre for Addiction and Mental Health; [2]Maryland Psychiatric Research Center; [3]Zucker Hillside Hospital*

**Background:** Individuals with schizophrenia spectrum disorders (SSDs) often suffer social cognitive deficits, which are associated with functional outcome. These include lower-level "simulation" processes (emotion recognition), thought to be subserved by a frontoparietal circuit, and higher-level "mentalizing" processes (theory of mind), involving cortical midline and lateral temporal regions. Despite evidence supporting the distinction of these constructs, little work has focused on the factor structure of social cognition. In schizophrenia, factor analytic results have been inconsistent, likely due to task and analytic approach variability, and inadequate sample sizes. Further, confirmatory factor analysis (CFA) has not been used to compare multiple models across people with SSDs and healthy controls. Thus, our objective was to elucidate the factor structure of social cognition across a large group of people with SSDs and healthy controls. We hypothesized that a two-factor model, including simulation and mentalizing factors, would demonstrate the best fit across participants. We also expected social cognitive and neurocognitive factors to load on separate respective higher-order factors, and social cognition to mediate the relationship between neurocognition and clinical and functional outcome measures.
**Methods:** Behavioural data was collected from 164 participants with SSDs and 102 healthy controls across three sites. Participants completed four tasks including measures of social cognition, ranging from basic emotion recognition to complex mental state inference. Participants also completed measures of functional outcome, symptom ratings, and the MATRICS Consensus Cognitive Battery. CFAs were conducted to test social cognitive models, as well as models of social cognition and neurocognition, and multi-group CFA was used to test measurement invariance between patients and controls.
**Results:** As predicted, a two-factor (simulation, mentalizing) model fit the social cognitive data well across participants with SSDs and healthy controls (RMSEA = .010, CFI = 1.00). This model also fit significantly better than a one-factor model ($p < .001$). Further, measurement invariance testing revealed factor structure invariance, loading invariance, and partial intercept invariance between groups, allowing for between-group comparisons. Participants with SSDs showed lower scores than controls for both simulation and mentalizing factors ($p < .001$), and scores on both factors correlated significantly with symptom ratings and functional outcome measures. Including neurocognitive data, a higher-order two-factor (social cognition, neurocognition) model fit the data well (RMSEA = .047, CFI = .971), and showed significantly better fit than a one- or two-factor model ($p < .001$). Lastly, social cognition was found to mediate the relationship between neurocognition and negative symptoms, as well as social functioning and quality of life measures ($p < .05$).
**Discussion:** Our results provide evidence that social cognition includes lower- and higher-level dimensions across both individuals with SSDs and healthy controls. They also suggest that both aspects are associated with clinical and functional outcome indices, and act as a mediator between neurocognition and these measures. This provides support for distinguishing lower- and higher-level social cognition between and across people with SSDs and healthy controls, and suggests that they may indeed have partially distinct underlying mechanisms. Further, results confirm the importance of social cognition as it relates to clinical and functional outcomes, and thereby as a potential treatment target for patients with SSDs.

### S246. PSYCHOMETRIC PROPERTIES OF THE DANISH VERSION OF BNSS (BNSS-DA)

Johannes Gehr*[,1], Birte Glenthoj[2], Mette Nielsen[2]
*[1]Center for Neuropsychiatric Research, Copenhagen University Hospital; [2]Center for Neuropsychiatric Schizophrenia Research, CNSR, and Center for Clinical Intervention and Neuropsychiatric Schizophrenia Research, Mental Health Centre Glostrup, University of Copenhagen*

**Background:** The concept of negative symptoms (NS) has been known since early 19th century but the development of assessment instruments and treatment methods has yet proved inadequate. The Brief Negative Symptoms Scale (BNSS) was designed to evaluate NS according to a consensus definition by the National Institute of Mental Health from 2005. This study examines the validity and reliability of the Danish version of BNSS (BNSS-Da).

**Methods:** 49 participants with schizophrenia or schizoaffective disorder were included, counting in- and outpatients as well as users of community housing facilities. Participants were assessed with BNSS-Da, Positive And Negative Syndrome Scale (PANSS), Scale for the Assessment of Negative Symptoms (SANS), Calgary Depression Scale for Schizophrenia (CDSS), St. Hans Rating Scale for extrapyramidal syndromes (SHRS), Personal and Social Performance Scale (PSP), Trail Making Test A and B, (TMT-A/B) and Digit Symbol Substitution Test (DSST). 19 of included subjects had their BNSS-Da interviews rated separately by two raters in order to evaluate interrater agreement. The convergent and divergent validity of BNSS-Da was assessed by its relationship with the aforementioned scales and tests.
**Results:** Of 49 included subjects, 45 were diagnosed with schizophrenia and 4 with schizoaffective disorder. The mean age was 33.1 (SD: 10.8) years and 65.3% were male. Mean duration of illness was 9.7 (SD: 9.2) years and the mean PANSS total score was 65.7 (SD: 17.6). Interrater reliability for BNSS-Da was estimated by calculating the intraclass correlation coefficient based on a mean-rating (k=2), absolute-agreement, 2-way mixed-effects model, which showed to be 0.953 (95%CI: 0.880–0.982). To examine convergent and divergent validity, Spearman's rank correlation coefficients were calculated. PANSS negative and SANS total (subgroup, n=38) were both well correlated with BNSS-Da ($\rho=0.813$, $p<0.001$ and $\rho=0.852$, $p<0.001$). Also, BNSS-Da seemed to correlate well with PANSS total ($\rho=0.736$, $p<0.001$), and to a lesser extend PANSS positive ($\rho=0.552$, $p<0.001$) and PANSS general ($\rho=0.628$, $p<0.001$). More infirm correlations were found between BNSS-Da and CDSS ($\rho=0.314$, $p=0.028$), PSP ($\rho=-0.480$, $p<0.001$), DSST (subgroup, n=47, $\rho=0.393$, $p=0.006$) and TMT-B (subgroup, n=39, $\rho=0.357$, $p=0.025$), while no significant correlations were found with TMT-A (subgroup, n=39) or SHRS, except the Parkinsonism subscale ($\rho=0.420$, $p=0.003$).
**Discussion:** The interrater reliability for the BNSS-Da proved to be excellent. Regarding convergent validity, the scale correlated well with the standardized assessment tools for NS, indicating the presence of a common construct of NS. Social functioning, as measured by PSP, was fairly correlated with BNSS-Da, demonstrating how NS are associated with functional outcome. As for divergent validity, the poor correlations between BNSS-Da and CDSS and most domains of SHRS suggested a good capacity for distinguishing between primary NS and NS secondary to depression or adverse effects of neuroleptics. However, the Parkinsonism subscale of SHRS had a rather firm correlation with BNSS-Da, probably because lack of facial expressions is measured in both scales. PANSS positive also seemed to correlate with BNSS-Da. This correlation assumedly stems from NS secondary to positive symptoms, since the present study included acutely psychotic subjects, unlike most other studies on translations of BNSS. The cognitive tests, TMT-A/B and DSST, were infirmly correlated to BNSS-Da, illustrating that cognitive function and NS likely are associated yet still separable through BNSS-Da. In conclusion, BNSS-Da holds appropriate psychometric properties in terms of reliability and validity.

### S247. GENDER DIFFERENCES IN FUNCTIONALITY IN INPATIENT POPULATION AFFECTED WITH SCHIZOPHRENIA AND OTHER PSYCHOSIS

Siddarta Acebillo*[,1], Rebeca García Collell[2], Lourdes Nieto[3], Carmina Massons[2], Noèlia Ortuño[4], Cristina Domènech[4], Jesus Cobo[2]
*[1]Corporació Sanitària Parc Taulí; [2]Corporació Sanitària i Universitària Parc Taulí. CIBERSAM, Universitat Autònoma de Barcelona; [3]Instituto Nacional de Psiquiatría Ramón de la Fuente Muñiz[4]Centre Numància, Parc Sanitari Sant Joan de Dèu*

**Background:** Studies that have examined gender differences in social functioning have found better performance in women but other studies failed to detect these differences (Ochoa et al, 2012).
We aim to study gender differences in functionality in a severe sample of schizophrenia and schizophrenia spectrum disorder patients, and to analyse the relationships between functionality, psychopathological dimensions and gender.
**Methods:** Multicenter cross-sectional naturalistic study sample of 124 (66.9% men) schizophrenia and non-affective schizophrenia spectrum disorder inpatients from a University Hospital Acute Unit setting. Diagnosis was made following DSM IV-TR. Severity of psychopathology was assessed using Positive and Negative Syndrome Scale (PANSS) Lindenmayer's Factors (Kay et al., 1987). The deficit of insight and its three dimensions were evaluated by the Scale of Unawareness of Mental Disorders (SUMD) (Amador et al., 1993). Functionality was measured by the Global Assessment of Functioning Scale (GAF) and the Personal and Social Performance scale (PSP) (Morosini et al, 2000). Premorbid Intelligence Quotient (IQ) was estimated by verbal sub-scale of WAIS. Bivariate analysis and parametric correlations were performed in order to make a multiple linear regression model of insight dimensions.
**Results:** The sample included a 42.7% of people affected of schizophrenia, with a severe psychopatology (mean total PANSS scores 83.7, sd. 23) and different clinical situations. In our sample, there were no significant differences in functionality neither with the GAF or the PSP global scores. Women performed significatively worst in the PSP self-care subscale ($p=0.024$), and men performed significatively worst in the PSP disturbing and aggressive behaviours subscale ($p=0.033$).
In the regression analysis, the total sample (men and women) showed a model for the PSP global scores including only the PANSS Lindenmayer's Desorganized/Cognitive Factor (R2 0.412). PSP self-care subscale showed a model including only PANSS Lindenmayer's Desorganized Factor (R2 0.319). PSP socially useful activities subscale showed a model including only PANSS Lindenmayer's Negative Factor (R2 0.213). PSP personal and social relationships subscale showed a model including the PANSS Lindenmayer's Negative and the Excitatory Factors (R2 0.533). PSP disturbing and aggressive behaviours subscale showed a model including only PANSS Lindenmayer's Excitative Factor (R2 0.363). Gender and other clinical, sociodemographical or outcome factors did not have influence in the models.
In men sample a model for the PSP global scores included only the PANSS Lindenmayer's Desorganized Factor (R2 0.511). PSP self-care subscale showed a model including both PANSS Lindenmayer's Desorganized Factor and IQ (R2 0.585). PSP socially useful activities subscale showed a model including only PANSS Lindenmayer's Negative Factor (R2 0.394). PSP personal and social relationships subscale showed a model including only the PANSS Lindenmayer's Negative Factor (R2 0.626). PSP disturbing and aggressive behaviours subscale showed a model including only PANSS Lindenmayer's Excitative Factor (R2 0.478).
In women in our sample, there were no explicative model. Moreover, global GAF scores were not explicated by a model in our sample.
**Discussion:** According to our data, men and women seem to be similar in levels of global functionality. Nevertheless, they showed several differences in specific domains of functionality measured by PSP. Model explainig the association of functionality with psychopatological and clinical variables showed a significative relatioship of isolated psychopatological factors and functional domains.

### S248. RELATION BETWEEN CHILDHOOD TRAUMA AND PSYCHOTIC SYMPTOMS IN SCHIZOPHRENIA

Amine Larnaout*[,1], Rahma Nefzi[1], Amina Aissa[1], Rouaa Trabelsi[1], Zouhaier El Hechmi[1]
*[1]Hospital Al Razi*

**Background:** There is renewed interest in the relationship between early childhood trauma and risk of psychosis in adulthood. Trauma and stressful events in childhood and adolescence are known to be more prevalent among individuals with schizophrenia and other psychotic disorders than in the general population. Furthermore, other findings support the role of childhood trauma as a socio-environmental risk factor for psychotic symptoms, and research on the potential etiological relationship between trauma/stressful events in childhood/adolescence and psychotic disorders is evolving. The aim of the current study was to examine relations among all items and domains of childhood trauma and schizophrenic symptoms in patients with schizophrenia. The relationship between types of trauma and their association with psychotic symptoms was analysed.
**Methods:** In this study, we collected data from 50 schizophrenic patients (39 males and 11 females). All patients met the DSM 5 criteria for schizophrenia. Psychotic symptoms were measured by the Positive and Negative Syndrome Scale (PANSS). Trauma and stressful events in childhood and adolescence were assessed using the Childhood Trauma Questionnaire (CTQ).
**Results:** We found significant correlations between emotional and sexual abuse, emotional neglect and denial scale in CTQ with positive symptoms of the PANSS ($p<0,05$).
Meanwhile, no correlations were found between CTQ domains neither with negative symptoms nor with general psychopathology scale of the PANSS.
**Discussion:** This study showed that childhood trauma could be a predictor factor for developing positive symptoms in schizophrenia. Most studies found similar results, showing a correlation between childhood trauma and hallucinations in schizophrenia. A correlation between childhood trauma and agressive behaviours was also described in litterature. These results went along with the stress sensitization model where the HPA axis is overactive and excessively reactive to the subsequent environemental stressors causing positive symptoms of the disease.

### S249. IS INTERNET HARMFUL FOR PSYCHOTIC PATIENTS?

Lucia Bonet[1], Blanca Llacer[2], Miguel Hernandez[3], David Arce[4], Ignacio Blanquer[4], Carlos Cañete[3], Maria Jose Escarti[5], Julio Sanjuan*[,2]
*[1]Universidad de Valencia; [2]CIBERSAM; [3]Clinic Hospital Valencia, CIBERSAM; [4]Universidad Politecnica de Valencia; [5]Peset Hospital*

**Background:** Developments in electronic health (e-Health) interventions for psychotic patients have been possible since the growing access and use of internet and electronic devices in past 10 years (Bonet et al. 2017). However, before proceeding further on develop these interventions; limited knowledge exists about the impact of internet and new technologies on the mental health of these psychotic patients. The aim of this study is to assess the benefits and risks of new technologies usage in a survey of patients diagnosed with psychotic disorders. We analyzed the relationship between experiences and opinions about internet and demographic and clinical characteristics of the sample and patterns of use of these technologies.
**Methods:** Structured questionnaire was designed. This questionnaire was divided in three parts: 1) clinical and demographic information, 2) access and use of technologies, and 3) experiences and opinions about internet. In total, 97 patients diagnosed with psychotic disorder participated in this cross-sectional study. Mean age of the sample was 37.06 (SD=12.9), 72.2% of participants were male, 84.5% were single and 60.8% had achieved secondary education. Main diagnoses in the sample were First Episode of Psychosis (45.4%) and Schizophrenia (34%) and 64.9% of patients had a length of illness lower than 72 months
**Results:** The percentage of patients who daily acceded to internet was 63.9% while 21.6% weekly acceded. 90.7% of participants owned a mobile phone and 68% had a social media account. Related to feelings about internet, 60.8% of patients felt socially linked due to internet usage and 78.4% felt informed. However, 22.7% felt frustrated and 19.6% felt suspicious. Internet was considered as a benefit for mental health for 46.4% of patients, while 38.1% have had unpleasant experiences related to its usage, 24.7% have had internet-related relapses and 26.8% expended excessive time online. Significant association was found between feeling informed and frequency of access to internet ($\chi2= 6.17$ $p=0.05$), however any other significant association was found between feelings about internet and clinical or demographic characteristics or patterns of use of technology. According to experiences, significant associations were found between internet-related relapses and length of illness ($\chi2= 4.74$ $p=0.03$), frequency of internet access ($\chi2= 9.76$ $p<0.01$) and social media ownership ($\chi2= 5.55$ $p=0.02$). Expending excessive time on internet was found significant associated to age of the sample ($\chi2= 6.57$ $p=0.04$), employment status ($\chi2= 10.73$ $p=0.03$), frequency of access to internet ($\chi2= 10.15$ $p<0.01$) and social media ownership ($\chi2= 9.62$ $p<0.01$). Association between stop taking medication because of information read on the internet and level of education was also found ($\chi2= 9.03$ $p=0.01$).
**Discussion:** Despite the general positive feelings about internet usage, percentages between 38-19% of patients had a negative vision of internet. Furthermore, frequency of access to internet and social media ownership have been found associated to internet-related relapses and potential pathological use of internet (excessive time on it). Younger patients, recent diagnosis of psychosis and being in a non-active employment situation seem to be related to these pathological results too. To our knowledge, this is the first study to describe the potential risks about internet usage in patients diagnosed with psychotic disorders, however further studies are needed.
**Reference:**
1. Bonet L, et al Use of mobile technologies in patients with psychosis: A systematic review. Rev Psiquiatr Salud Ment. 2017; 10 (3): 168–178

### S250. RELATION BETWEEN PSYCHOPATHOLOGY AND QUALITY OF LIFE IN SCHIZOPHRENIA PATIENTS BEFORE AND AFTER FIRST ANTIPSYCHOTIC TREATMENT

Gitte Andersen*[,1], Helle Schæbel[1], Sanne Wulff[1], Birte Glenthøj[1], Mette Ødegaard Nielsen[1]
*[1]Center for Neuropsychiatric Schizophrenia Research, CNSR, and Center for Clinical Intervention and Neuropsychiatric Schizophrenia Research, CINS, Mental Health Centre Glostrup, University of Copenhagen*

**Background:** It is common knowledge that antipsychotic treatment improves the symptomatology in schizophrenia, especially for the psychotic and general symptoms. It is also a fact that patients with schizophrenia often report a reduced quality of life compared to healthy controls. In this study we aim at examining the relation between self-reported quality of life (QLS), psychopathological symptoms and level of function before and after antipsychotic treatment. We hypothesize that there will be a correlation between QLS and severity of symptoms before treatment. Further we expect an improvement in QLS after treatment and that this improvement will correlate with improvement in symptomatology.
**Methods:** As a part of a large multimodal study on antipsychotic naïve patients with schizophrenia, 69 patients were recruited. Their psychopathology was measured with the Positive and Negative Syndrome Scale (PANSS), level of function was estimated using Global Assessment of Function (GAF), and QLS was reported by answering a questionnaire. Patients were treated with individual doses of Amisulpride for six weeks, after which they were reexamined.
The questionnaire regarding QLS counts 21 questions, divided into four domains: Self and present life (i.e. "how satisfied are you with your present life"), social relations ("how satisfied are you with your current social life"), Living situation ("how much do you like the place you live") and Work situation ("How satisfied are you with the work you do"). Higher scores indicate higher satisfaction within the domain. Since the follow up period was only 6 weeks, we focused on self and present life (SPL) and social relations (SR), as we did not expect the living and work situation to change significantly within this period.
**Results:** Baseline data were available on 48 patients, mean age 25 years (6.1), 31 males (65%).
Their PANSS total score was 84 (16.0), GAF was 41(9.4), SPL-score was 13 (5.3) SR-score 10(5.3). For SPL as well as SR, there was a negative correlation with PANSS-total, PANSS-negative and PANSS- general (p-values<0.007). Follow-up data were available on 33 patients, mean age 25 years (6.6), 19 males (58%). They received 273 (163.3) mg Amisuplride. PANSS total was 68 (14.4) and GAF was 53 (15.7), SPL-score was 14 (3.9) SR-score 11(4.6). Paired T-test showed a significant improvement in PANSS total, PANSS positive, PANSS general and GAF (all p-values<0.001). There was also an improvement in SR (p=0.003), but no significant improvement in SPL and PANSS negative score (p=0.12 and p=0.5).
There were no correlations between neither of the QLS scores and any psychopathology scores at follow up. Likewise, there was no correlation between change in QLS scores and change in psychopathology. However, there was a negative correlation with change in SPL and medication dose (p=0.009)
**Discussion:** In this study, we found that antipsychotic naïve patients with most severe symptoms had the lowest self-reported QLS. This relation was only observed for negative and general symptoms, but not for positive symptoms or GAF score. As expected there was a treatment induced improvement in positive and general symptoms as well as GAF score. Likewise, patients improved on QLS, but only on SR and not in the overall measure of SPL. This may partly be because antipsychotic medication primarily improves positive symptoms, which were not correlated with QLS. Additionally, there was even a negative correlation with medication dose, indicating that patients with higher doses had the least improvement I SPL score. The results indicate that there is not a simple relationship between antipsycotic induced improvement in psychopathology and selfreported QLS. High doses of medication may even reduce QLS.

### S251. QUALITY OF LIFE OF CHRONIC SCHIZOPHRENIA PATIENTS IN THE LONG TERM FOLLOW-UP

Diego Mendes*,[1], Gustavo Mustafé[1], Thalita Fernandes[1], Thais Martins[1], Clarissa Dantas[1]
[1]*University of Campinas*

**Background:** In recent years the goal of the treatment of patients with schizophrenia has shifted from symptom remission to the improvement in health as a whole. In this context, concepts such as quality of life (QoL), social and occupational functioning gained interest. The aim of this study is to investigate changes in QoL of chronic schizophrenia patients in the long-term follow-up and their associations with symptoms and level of community functioning.
**Methods:** We will contact 85 patients with schizophrenia, considered clinically stable in the previous year, who participated in a study about the deficit syndrome of schizophrenia in 2009/2010. Back then, they were recruited in two sites: an outpatient service of a university general hospital (49 patients) and a community-based service (36 patients). Patients will be assessed with the same instruments adopted in the first study: SAPS, SANS, Calgary Depression Scale and Quality of Life Scale (QLS), plus the Personal and Social Performance Scale (PSP), not used at baseline. We started recruitment by the patients originally treated in the outpatient clinic.
**Results:** Until now, of the 49 patients, 2 dropped out treatment, 8 had been transferred to other services, 5 refused to participate, 1 had the diagnostic changed to bipolar disorder and 3 had died precociously. Of the deaths, 1 was due to complications secondary to the use of clozapine, 1 due to suicide and 1 patient was murdered by another patient during a psychiatric hospitalization. Up to now, 20 patients completed reassessment, mean age at baseline was 36.9 ± 8.9 years, mean duration of mental illness was 16 ± 10.1 years, and 75% were men. They had in mean, 10.7 ± 3.3 years of education, only 4 had any work activity and 55% had a low socioeconomic position. Assessment interval was 6.9 years ± 0.5. Some demographic aspects slightly worsened: only 15% had an occupation at follow-up, and 60% fell in the lower socioeconomic position. Between assessments, 8 (40%) patients have had periods of noncompliance to medication, 5 had psychiatric hospitalizations, three of them involuntarily. At follow-up 70% of the patients were using clozapine and, despite that, some presented residual positive symptoms (SAPS 6.2 ± 4.8); Calgary mean score was low (2.2 ± 2.2) and, except for the patient who died, there was no new suicide attempts between assessments. Negative symptoms severity was moderate in general (mean SANS 14.8 ± 7.2). Patients as a group had no significant change in QLS scores (61.5 ± 28.2 versus 60.1 ± 28.2 at follow-up). However, 15% of patients had an improvement greater than 20% in their QLS scores, and 30% had a worsening of their scores greater than 20%. Baseline scores on SANS (p=0.005), SAPS (p=0.03) and number of hospitalizations (p=0.03) were negatively correlated with follow-up QLS scores; and years of schooling at baseline was positively correlated (p=0.2). Baseline QLS scores were strongly associated with current QLS scores, as well as with PSP scores (both with P <0.000) at follow-up. Regarding PSP outcomes, 50% of patients were classified in the 70-31 interval (disabilities of various degrees) and 25% were under 30, requiring intensive support.
**Discussion:** The results presented are partial, obtained with a provisional small sample size. Nevertheless, they show some interesting trends as the possible existence of two patterns of outcome regarding QoL in chronic schizophrenia patients: of significant improvement and one of worsening. If those initial findings are to be confirmed, our next step will be to investigate characteristics associated to improvement or deterioration of QoL in spite of a relative stability of symptoms. That sort of information is of great relevance in the pursuit of recovery for schizophrenia patients.

### S252. HEALTH CARE RESOURCE UTILISATION IS HIGHER IN PATIENTS PRIOR TO DIAGNOSIS WITH SCHIZOPHRENIA THAN NON-SCHIZOPHRENIA COMPARATORS IN A LARGE COMMERCIALLY-INSURED POPULATION IN THE UNITED STATES

Anna Wallace[1], Keith Isenberg[2], John Barron[1], Whitney York[1], Mayura Shinde[1], Matt Sidovar[3], Jessica Franchino-Elder[3], Michael Sand*,[4]
[1]*Healthcore, Inc.;* [2]*Anthem Inc.;* [3]*Health Economics and Outcomes Research, Boehringer Ingelheim Pharmaceuticals, Inc.;* [4]*Boehringer Ingelheim Pharma GmbH & Co. KG*

**Background:** Schizophrenia is associated with considerable health care resource utilisation (HCRU) and costs, yet little is known about the patterns of care and HRCU in patients with schizophrenia prior to diagnosis. To address this knowledge gap, we examined the HCRU of patients with and without schizophrenia over a 5-year pre-diagnosis period.
**Methods:** This US-based retrospective study used claims data from the HealthCore Integrated Research Database to identify newly diagnosed patients with schizophrenia (ICD-9: 295.x, ICD-10: F20.x) aged 15–54 years at diagnosis. Patients with schizophrenia were compared with a demographically matched (1:4) non-schizophrenia cohort during the 0–12 months, >1–2, >2–3, >3–4 and >4–5 years prior to schizophrenia diagnosis. During the pre-diagnosis periods, both all-cause and behavioural health-related HCRU were described.
**Results:** The schizophrenia and comparator cohorts included 6,732 and 26,928 patients, respectively. The most common types of schizophrenia were schizoaffective disorder (49%), paranoid (24%) and unspecified (19%). Patients were distributed across all major US regions (Northeast: 18%, Midwest: 27%, South: 29%, West: 27%). Average age at diagnosis was 32.8 years and most patients were male (57.4%). The percentage of patients with at least one all-cause inpatient hospitalisation in the 0–12 months prior to diagnosis was 32.7% for patients with schizophrenia versus 3.9% for comparators. Patients with schizophrenia had a greater mean number of all-cause physician office visits in all pr- diagnosis time periods versus comparators (schizophrenia: 4.5–5.5 visits, comparators: 3.1–3.2 visits). Behavioural health-related HCRU was also more substantial in patients with schizophrenia versus comparators across all time periods in terms of the mean number of visits to a psychiatrist (1.8–2.9 vs 0.1 visits, respectively) or a psychologist (1.0–1.2 vs 0.2 visits, respectively). The percentage of patients with claims for antipsychotic medication was also greater in the schizophrenia cohort vs comparators (21.8–56.6% vs 0.7–1.0% of patients, respectively).

**Discussion:** For up to 5 years prior to diagnosis, patients with schizophrenia have higher all-cause and behavioural health-related HCRU, in addition to higher use of anti-psychotic medications, compared with matched comparators. In the schizophrenia cohort, HCRU increased in frequency closer to diagnosis, compared with matched comparators, whose HCRU remained relatively stable.

This study improves our understanding of the characteristics of clinically high-risk patients who go on to develop schizophrenia, who have more frequent encounters with health care providers than comparators. These results also suggest that early identification and treatment of patients prior to schizophrenia diagnosis could be optimised and is warranted.

**Funding:**

Boehringer Ingelheim (ANTHEM)

### S253. PERSON-CENTERED PSYCHOSIS CARE (PCPC) IN AN INPATIENT SETTING: PATIENT OUTCOMES

Anneli Goulding*[,1], Katarina Allerby[1], Lilas Ali[1], Margda Waern[1]

[1]*University of Gothenburg*

**Background:** The person-centered care approach has been little tested in inpatient settings for persons with schizophrenia and similar psychoses. We developed a staff educational intervention, Person-Centered Psychosis Care (PCPC) tailored to our care setting (4 hospital wards for persons with psychoses, 43 beds). The intervention was co-created by professionals, patients, and researchers using a participatory approach. There was a focus on the patient's narrative, the creation of partnership between staff and patient, an agreement between staff and patient concerning care, and a bridging of inpatient and outpatient care and support. The present study aims to describe patient outcomes associated with PCPC.

**Methods:** The study had a before and after design. Before the PCPC intervention started, questionnaire data was collected from 50 inpatients shortly before discharge. Post intervention data are currently under collection (anticipated n=50). The primary outcome measure is self-reported empowerment (Empowerment Scale, Range 0–112) and the secondary measure is consumer satisfaction (UKU-ConSat Rating Scale, converted to range between 11 and 77). Participants also complete questionnaires related to possible confounding variables such as overall health (EQ-5D), symptom burden (PANSS), and functional ability (GAF).

**Results:** The participants (46% women) included in the pre-intervention sample had a mean age of 47.5 years (SD=14.5). The total mean empowerment score for the pre-intervention sample was 82.6 (SD=8.1) whereas the mean consumer satisfaction score was 51.5 (SD=12.9). There were no statistically significant gender differences regarding empowerment or consumer satisfaction. There were no significant correlations between age, any of the confounding variables, and empowerment and consumer satisfaction. We will present results from comparisons between the pre- and post-intervention groups regarding empowerment and consumer satisfaction.

**Discussion:** The before and after design has its limitations, but if the PCPC intervention proves beneficial, such a model could be tested with a cluster randomized study design.

### S254. IMPLEMENTATION OF A PROGRAM FOR EARLY INTERVENTION IN PSYCHOSIS ONSET: THE EXPERIENCE OF REGIONE EMILIA ROMAGNA, NORTHERN ITALY

Maria Ferrara*[,1], Sinan Guloksuz[2], Shadie Burke[1], Flavia Baccari[3], Manuela Miselli[3], Alessio Saponaro[4], Mila Ferri[4], Vinod Srihari[1], Fabrizio Starace[3], Gruppo Regionale Esordi Psicotici[4]

[1]*Yale University, AUSL Modena;* [2]*Academic Hospital Maastricht, Yale University* [3]*AUSL Modena;* [4]*Servizio Assistenza Territoriale, Area Salute Mentale e Dipendenze Patologiche, Regione Emilia-Romagna*

**Background:** Early interventions services (EIS) for psychosis are not uniformly available in the Italian public mental health care system. In 2012, Region Emilia Romagna funded the implementation of a comprehensive population based program to deliver EIS. These services provide a package of care including psychiatric consultation, family psychoeducation, case management, recovery oriented activities (e.g. supported employment, social inclusion), and physical health monitoring, consistent with international models but embedded within community mental health services (CMHS). We report feasibility, descriptors of enrolled samples, and clinical variables associated with remission.

**Methods:** Demographic and clinical data of CMHS users that accepted EIS from January 1st, 2013 to December 31st, 2016 were acquired from paper and electronic health records in each province. Inclusion criteria were: residence in Regione Emilia Romagna, age 18–35, presence of non-organic, affective and non-affective psychotic symptoms within two years of onset. Exclusion criteria included severe intellectual disability and non-fluency in Italian. Remission was defined as a total score of 8 on the Health of Nation Outcome Scale (HoNOS) at 6 months after enrollment.

**Results:** Six hundred and eighty-nine patients accepted EIS. Median age was 22, 93% had diagnoses of non-affective psychosis, whereas 7% affective psychosis, with a median duration of untreated psychosis (DUP) of 6 months [IQR=10; 0–120], 41% had comorbid substance use disorders, 31.1% had personality disorders, and 39% had a previous hospitalization. The proportion of migrants (23%) was almost twice that of the entire Region (11.9%). Psychiatric visits represented 44% of total utilization, whereas only 14% received at least one case management visit, 79% a family session, 19% a recovery oriented activity, and 1% physical health monitoring.

Of the sample, 460 subjects (67%) improved as presented with significant reduction in the 4 subscales scores of the follow up HoNOS, and 164 (35.7%) showed remission. Shorter DUP and lower HoNOS scores at baseline were associated with an increased likelihood of achieving remission (OR=1.03, p=0.0068, and OR=1.04, p=<0.0001, respectively), whereas the presence of personality disorder was associated with a reduced likelihood of remission (OR=0.48, p=0.0057).

**Discussion:** EIS was acceptable to most eligible patients in regional CMHS. EIS enrollees evidenced significant clinical improvement in the first 6 months. Only a minority was diagnosed with bipolar disorder, suggesting a possible later onset of affective psychosis and reduced chance of accessing the Program.

The correlation of comorbid personality disorder with worse outcomes, suggests the need to develop a targeted treatment. The EIS were also well accepted by the high proportion of migrants. Further work is required to understand possible social determinants of psychosis onset and pathways to care in these fragile communities. The high rate of concomitant substance use at intake must be considered for developing specific pharmacological and psychoeducational treatment.
One in five patients needed admission to the inpatient unit in the first six months after onset, showing high levels of symptomatic distress. Moreover, referrals from hospital units show also possible barriers to access outpatient mental health facilities when users present with acute and urgent clinical conditions.
This report establishes the feasibility of a regional network of EIS in Northern Italy with shared data elements that will lead to useful comparisons across EIS sites within the region, and also collaborative efforts to address specific gaps in access or outcomes.

### S255. METAPHORICAL CONCEPTUALIZATION OF SCHIZOPHRENIA AND THE CAREGIVING PROCESS FROM THE PERSPECTIVE OF PRIMARY FAMILY CAREGIVERS

Zhou De-Hui Ruth*[,1], Chiu Yu-Lung Marcus[2], Lo Tak-Lam William[3], Lo Wai Fan Alison[3], Wong Siu Sing[1]
*[1]Hong Kong Shue Yan University; [2]City University of Hong Kong; [3]Kwai Chung Hospital*

**Background:** According to Hong Kong Hospital Authority (2011), nearly 20,000 outpatients with schizophrenia in Hong Kong demand substantial family support. The family caregivers are the individuals who take care of persons with schizophrenia daily, check their medication and provide emotional support. They are enlisted as important therapeutic agents. How family caregivers understand and perceive schizophrenia and the caregiving process will influence not only the quality and their persistence of the caregiving, but also the rehabilitation process of persons with schizophrenia. Thus, it is important to investigate their conceptualization of schizophrenia and the caregiving process to inform schizophrenia mental health family recovery work.
**Methods:** This study used a mixed method of quantitative and qualitative design. In the quantitative part, a questionnaire about the metaphors for schizophrenia and the caregiving process were administrated to 194 caregivers whose family members were diagnosed with schizophrenia according to the 2016 version of ICD-10-CM Diagnosis Code. This questionnaire also included standardized instruments, such as Brief Family Relationship Scale (BFRS), Experience of Caregiving Inventory (ECI-66), Mental Health Inventory(MHI-5) and Inner Resource Scale (SAS-I). Among the participants, 147 were women caregivers and 47 were male caregivers. In the qualitative part, a focus group interview with 8 randomly selected caregiver participants were invited to talk about their caregiving experiences and their understanding of schizophrenia.
**Results:** The dominant metaphors for schizophrenia were reported as unexpected visitors, the Anakin Skywalker, a time bomb, and a fire alarm. These metaphors vividly describe the unexpectedness of episodes that schizophrenia outpatients experienced as well as the explosive damages that schizophrenia caused their families. 73.2% participants used "climbing up the mountain" to describe their caregiver experiences and emphasize on the necessity of overcoming difficulties during the caregiving process. 67.5% caregivers preferred to use the "rescue work of firefighters" to describe the nature of their caregiving. 50% participants indicated the caregiving work as a burden that they chose not to lay down no matter how heavy it is, whereas 17.5% caregivers regarded it as a burden that they cannot shake off and always restricts their freedom. The independent t-tests showed that adult children caregivers reported statistically significantly more positive personal experiences $t(94) = -2.423$, $p < .05$, $d = .26$, readiness to seek for information $t(94) = -2.860$, $p < .01$, $d = .61$, and positive communication $t(94) = -2.625$, $p < .01$, $d = .56$ than spouse caregivers.
**Discussion:** The metaphorical conceptualization for schizophrenia and the meaning of the caregiving process from the caregivers' perspectives have strong implications for schizophrenia family recovery work. They could help to frame psychotherapy sessions for caregiver group works. According to the narrative psychotherapy approach, the metaphor of "unexpected visitors" could help caregivers to externalize the problem of schizophrenia and gain a space to describe and deal with schizophrenia in alliance with their family members.
**Acknowledgement:** The reported study was funded by the Research Grant Council of HKSAR (UGC/FDS15/M01/15). However, the opinions expressed do not necessarily reflect the positions of the funding agency. We want to acknowledge our great gratitude to Kwai Chung hospital for their support in multiple aspects and all caregiver participants for their active participation in our research.

### S256. A META-ANALYSIS OF RECOVERY EDUCATIONAL AND AWARENESS INTERVENTIONS FOR MENTAL HEALTH PROFESSIONALS

Helena García-Mieres*[,1], Francisco José Eiroa-Orosa[2]
*[1]Universitat de Barcelona; [2]Universitat de Barcelona & Yale School of Medicine*

**Background:** The history of mental health care has been marked by various struggles for the dignity of service users. Some reform movements have started to use strategies aimed at professionals' beliefs and attitudes change. This conference paper intends to systematically review and synthesize all information related to awareness-raising and training of professionals in aspects related to empowerment, recovery and in general in rights-based care to achieve full citizenship of mental health services users.
**Methods:** We searched academic databases as well as web search engines, aiming at finding grey literature on the subject. Quantitative studies were included if they included mental health professionals, defined as all staff involved in the management of mental health service users, as well as mental health students. All participants included should have assisted to a recovery or psychosocial rehabilitation educational or awareness-raising program. Effect size of change in knowledge, attitudes and intention to implement recovery-based practice were meta-analyzed using a fixed effects model.
**Results:** After a preliminary search, a total of 800 articles were added to a global database, of which 50 include explicit information on concrete trainings. Of these, 25 reported information about evaluation of the effectiveness of these training activities. Finally, 13 studies were included in the analysis, with a total sample size of 1123. Six studies adopted a repeated measures design and seven an independent group design (including RCTs and quasi-experimental studies). Recovery and rehabilitation based interventions had, on average, a small-to-medium-sized effect on knowledge of recovery principles ($d+ = 0.33$, 95% CI: 0.13 to 0.49); a small-to-medium-sized effect on attitudes to recovery principles ($d+ = 0.36$, 95% CI: 0.25 to 0.46), and a small-to-medium-sized effect on intention to implement recovery practice ($d+ = 0.37$, 95% CI: 0.02 to 0.71).
**Discussion:** The results show positive effects of educational and awareness activities for mental health professionals. Elements such as duration and intensity of activities must be considered when analysing the persistence and applicability of the effects. More quality studies are needed to establish the active ingredients of these activities.

### S257. EFFICACY OF CARIPRAZINE BY BASELINE SYMPTOM SEVERITY IN PATIENTS WITH SCHIZOPHRENIA: A POST HOC ANALYSIS OF 3 RANDOMIZED CONTROLLED TRIALS

Rick Mofsen*[,1], Yan Zhong[2], György Németh[3], Ágota Barabássy[3], Willie Earley[2], Kelly Krogh[2]
*[1]NA; [2]Allergan; [3]Gedeon Richter*

**Background:** Antipsychotic efficacy across the spectrum of disease severity is important in patients with schizophrenia. Cariprazine (CAR) is a dopamine D3/D2 receptor partial agonist antipsychotic; it is FDA approved for the treatment of adults with acute schizophrenia and mixed or manic episodes of bipolar I disorder. Post hoc analyses investigated the impact of baseline illness severity on the efficacy of CAR.

**Methods:** Data were pooled from 3 positive, 6-week, randomized, double-blind, placebo-controlled Phase II/III studies of CAR in adult patients with acute exacerbation of schizophrenia (NCT01104766, NCT01104779, NCT00694707). Patients were stratified by tertile into 3 severity subgroups by baseline Positive and Negative Syndrome Scale (PANSS) total score: ≤92 (placebo=160, CAR=376), >92 and ≤100 (placebo=136, CAR=312), or >100 (placebo=146, CAR=336). Post hoc analyses evaluated mean change from baseline to week 6 in PANSS total score, PANSS Positive and Negative Subscale scores, and Clinical Global Impressions-Severity (CGI-S) score. Least squares mean differences (LSMD) with 95% confidence intervals (CI) for the CAR versus placebo groups were estimated using a mixed-effects model for repeated measures (MMRM) approach.

**Results:** The LSMDs from baseline to week 6 were statistically significant for CAR versus placebo in each subgroup on PANSS total score (≤92: LSMD [95% CI]= -4.11 [-7.26, -0.96], P=.0106; >92 and ≤100: -8.80 [-12.62, -4.98], P<.0001; >100: -10.68[-14.76, -6.60], P<.0001), PANSS Negative Subscale (≤92: -1.12 [-1.90, -0.35], P=0.0045; >92 and ≤100: -2.11 [-3.02, -1.20], P<.0001; >100: -2.18 [-3.21, -1.14], P<.0001), and CGI-S score (≤92: -0.19 [-0.38, -0.01], P=.0418; >92 and ≤100: -0.50 [-0.72, -0.28], P<.0001; >100: -0.62 [-0.84, -0.39], P<.0001). The difference for CAR versus placebo was statistically significant on the PANSS Positive Subscale in the >92 and ≤100 (-2.78 [-4.06, -1.49], P<.0001) and the >100 (-3.50 [-4.78, -2.23], P<.0001) subgroups, but not in the ≤92 group (-0.85 [-1.93, 0.23], P=.1229). When PANSS baseline scores were stratified by the median (≤96 vs >96), significantly greater change from baseline was observed for CAR versus placebo in each severity subgroup on all 4 scales (all comparisons, P<.001).

**Discussion:** When PANSS baseline scores were stratified by severity, significantly greater mean change from baseline was observed for CAR versus placebo on PANSS total score, PANSS Negative Subscale score, and CGI-S score in all severity subgroups; on the PANSS Positive Subscale, change was significantly greater for CAR versus placebo in the 2 subgroups with higher baseline scores. Overall, CAR effectively improved symptoms in patients with schizophrenia regardless of baseline disease severity and the treatment effect was greater in subgroups with higher baseline severity.

### Author Index

Aarts, Henk, S77, S242
Abdel-Baki, Amal, S17
Abdulkadir, Ahmed, S184
Abellanas, Adelina, S237
Abi-Dargham, Anissa, S2, S44, S44, S71
Aboraya, Ahmed, S365
Abramovic, Lucija, S109
Abrams, Daniel, S212
Abt, Markus, S340
Acebillo, Siddarta, S423
Acharya, Bishnu, S405
Adair, Amy, S147
Adams, Rick, S327, S385
Adams, Robert, S284
Addington, Jean M., S231
Addington, Jean, S39, S62, S78, S79, S102, S266, S355, S396, S422
Adery, Laura Hieber, S77, S195, S196, S421
Adorjan, Istvan, S192
Adres, Merav, S306
Agartz, Ingrid, S306
Agarwal, Sri Mahavir, S345, S413
Agbedjro, Deborah, S196
Agerbo, Esben, S85, S87, S167
Agid, Ofer, S267, S354, S415
Ahmed, Anthony, S175
Ahmed, Kinza, S207
Ahmed, Raees, S343
Ahn, Yong Min, S203
Ahsan, Zohra, S104, S413
Ahuir Perez, Maria Isabel, S253
Ahuir, Maribel, S251
Aikawa, Sayaka, S315
Ainsworth, John, S50, S106
Aissa, Amina, S163, S258, S371, S403, S423
Aitchison, Kathy, S295
Ajnakina, Olesya, S58, S59, S84, S98, S166, S246, S373
Ak, Sertac, S345
Akbarian, Schahram, S82
Akded, Berna, S368
Akdede, Berna Binnur, S300, S356
Akgul, Ozge, S300
Al Hadithy, Asmar, S295
Alústiza, Irene, S352
Al-Diwani, Adam, S61
Al-Radaideh, Ali, S180
Alagille, David, S176
Alameda, Luis, S25, S236, S310
Albert, Nikolai, S95, S99, S106, S129, S134
Albertini, Giulia, S20
Albuquerque, Ana, S262
Alemán-Gómez, Yasser, S395
Aleman, André, S89, S177
Aleman, Andre, S238, S260
Alena,Gizdić, S195
Alex, Fornito, S162
Alexaki, Danai, S198
Alexandria, Raissa, S262
Algora, Maria José, S303
Alho, Jussi, S172
Ali, Lilas, S426
Alison Adcock, R., S281
Alison, Lo Wai Fan, S427
Allan, Stuart, S312
Allen, Lauren, S332
Allen, Paul, S28, S174
Allerby, Katarina, S426
Allott, Kelly, S32, S95, S99, S130, S404
Allswede, Dana, S422
Almeida, Valéria, S22, S297
Almodóvar, Carmen, S145, S174
Alolayan, Yazeed, S409
Alonso, Anna, S208
Alonso-Lana, Silvia, S145
Alptekin, Köksal, S300, S356
Alptekin, Koksal, S368
Alsop, Eric, S221
Altamirano, Olivia, S265
Altamura, Carlo, S371
Altamura, Mario, S263, S264
Altavilla, Benedetta, S312, S414
Altinok, Simge, S368
Alvárez, Mar, S212
Alvarez-Astorga, Aldara, S186
Alvarez-Jimenez, Mario, S4
Alves, Tania Maria, S195, S376
Álvarez, Mar, S113
Amaral, David, S126
Amaral, Robert, S182
Amato, Davide, S337
Ameis, Stephanie, S226
Amelsvoort, Therese van, S391
Amina, Aissa, S152
Aminoff, Sofie R., S113
Ammar, Hanen Ben, S163, S258
Amminger, G. Paul, S116, S326
Amminger, Paul, S378
Amodio, Antonella, S263, S264
Amoretti, Silvia, S318
Anık, Yonca, S283
Anand, Ravi, S126, S132
Andersen, Gitte, S424
Anderson, Kelly, S108, S271
Anderson-Schmidt, Heike, S155
Anderssen, Jannicke F., S205
Andrade, Chittaranjan, S405
Andrade, Julio Cesar, S195, S376
Andrea, Alexandra, S264, S353
Andreassen, Anna Krogh, S336
Andreassen, Ole A., S113, S205, S227, S362
Andreassen, Ole, S122
Andreasson, Eva, S305, S407
Andreopoulou, Angeliki, S243, S348
Angelique, Lowrie, S370
Anhøj, Simon, S80, S83, S213
Ania, Fiksinski, S247, S376
Anmella, Gerard, S337
Anna-Karin, Olsson, S263
Anne Emilie, Stürup, S134
Anne-Kathrin, Fett, S40
Annia Rüesch,Ranganadan, S254
Annibale, Luciano, S384
Ansorge, Olaf, S192
Anticevic, Alan, S30, S327
Antonija Slaviček, Sučić, S257
Antonsen, Sussie, S167
Antonucci, Linda A., S185
Antonucci, Linda, S100
Anttonen, Esa, S345
Aparecida, Fachim Helene, S222, S227
Appadurai, Vivek, S85
Appiah-Kusi, Elizabeth, S28
Arad, Michal, S191
Arango, Celso, S49, S50, S226
Arban, Roberto, S380
Arce, David, S424
Arends, Johan, S238
Argolo, Felipe, S112, S164, S286
Argyelan, Miklos, S176, S330
Arias, Barbara, S174, S330, S332
Arias, Víctor, S96
Armando, Marco, S38
Armio, Reetta-Liina, S391
Arnold, Chelsea, S302
Arrojo, Manuel, S398
Arroyo, Miguel Bernardo, S205
Arseneault, Louise, S25, S97, S110

Ash, David, S408
Ashcroft, Darren, S388
Asmal, Laila, S261, S364, S367
Assunção-Leme, Idaiane, S184
Astorga, Aldara Alvarez, S249
Athey, Alison, S374
Atienza, Joy, S339
Atkins, Alexandra, S159
Atkins, Joshua, S296
Atkinson, Samuel, S351
Attademo, Luigi, S363
Attrotto, Maria Teresa, S100
Attwood, David, S274
Aubry, Jean-Michel, S254
Auer, Dorothee, S283
August, Sharon, S190
Austin, Stephen, S163
Avagliano, Camilla, S204, S312, S414
Averbeck, Bruno, S383
Awasthi, Swapnil, S287
Ayşe,Kircali, S419
Ayşegül,Özerdem, S356
Ayesa-Arriola, Rosa, S142, S251, S268, S373, S374, S392
Ayllon, Lourdes, S237
Azechi, Hirotsugu, S419
Azis, Matilda, S81
aan het Rot, Marije, S260

Bär, Karl, S152
Bäuml, Josef G., S283
Baños, Iris, S370
Baalbergen, Heleen, S77, S225
Baccari, Flavia, S426
Bach, Michael, S11
Bachman, Peter, S266
Bachmann, Silke, S115
Badan Ba, Maryse, S388
Badcock, Johanna C., S229
Badcock, Johanna, S365
Badia, Francina, S123
Badre, David, S244
Baek, Ji Hyun, S362
Baeza, Immaculada, S337
Baeza, Inmaculada, S123, S125, S181, S183, S229
Bahk, Won-Myong, S161, S201, S206, S218, S236, S264, S296, S304, S308, S326, S367, S401, S411
Bahk, YongChun, S344
Bak, Maarten, S160
Bak, Nikolaj, S289
Baker, Amanda, S151
Baker, Andrea, S237
Baker, Dewleen, S82
Baker, Elizabeth, S353
Bakija, Ivana, S257
Bakker, Geor, S275, S391
Bakker, P. Roberto, S295, S325
Bakstein, Eduard, S173
Balakrishnan, Narayanaswamy, S189
Balducci, Pierfrancesco, S409
Ball, Hannah, S209
Ballon, Jacob, S17
Balzan, Ryan, S73, S208
Banaszak, Beata, S252
Bang, Minji, S188
Barajas, Ana, S210, S238, S317, S348, S372
Barbeito, Sara, S255
Barber, Anita, S289
Barbera, Daniele La, S75, S258
Barbero, Juan David, S303
Barch, Deanna, S140
Bardell-Williams, Melissa, S162, S270, S376
Bargallo, Nuria, S181
Barker, Gareth J., S81, S89
Barker, Gareth, S51, S174
Barker, Peter, S20
Barnes, Catherine, S30, S250, S387
Barnes, Nicholas, S48
Barnes, Thomas, S131, S148
Barnett, Jennifer, S145, S248, S353
Barone, Annarita, S312, S414
Barret, Olivier, S176
Barrigón, Maria Luisa, S348
Barrigón, Maria Luissa, S238
Barrigon, Maria Luisa, S317, S372
Barron, David, S368
Barron, John, S425
Barron, Rachel, S27
Barrowclough, Christine, S197
Barry, Erica, S92
Bartels, Matthew, S17
Bass, Emily, S360
Bassett, Anne, S247, S376
Basso, Manuela, S193
Basson, Wilna, S200
Batalla, Albert, S183
Batty, Rachel, S138, S260
Baumann, Melissa, S126
Baumann, Philipp S., S15, S236, S279
Baumann, Philipp, S133, S395
Baumann, Philippe S, S25
Baumel, Amit, S267
Baumgartner, Markus, S331
Baune, Bernhard, S74
Bayatmokhtari, Maryam, S2
Beale, Eleanor, S374
Bear, Adam, S147
Bearden, Carrie E., S102, S231
Bearden, Carrie, S39, S43, S78, S79, S266, S396, S422
Beardon, Carrie, S62
Beasley, Clare, S71
Becerra, Juan Antonio, S255
Bech, Per, S341, S342
Beck, Katherine, S68, S330
Becker, Benedikt, S355
Bedi, Gillinder, S43
Bediat, Laurent, S388
Begemann, Marieke, S95, S109
Begemann, Martin, S85
Beilen, Marije Van, S344
Belangero, Sintia I., S266
Belangero, Sintia, S96, S184
Belger, Aysenil, S79, S266
Bell, Imogen, S279
Bell, Morris, S196
Bellomo, Antonello, S263, S264
Belsham, Denise, S313
Ben Ammar, Hanen, S371, S403
Benavides, Caridad, S233
Bendall, Sarah, S4
Benedicto, Crespo-Facorro, S384
Benito, Adolfo, S417
Bennett, Jeffrey, S126
Bennett, Melanie, S264, S353
Benros, Michael Eriksen, S273
Benros, Michael, S85, S116
Benson, Taylor, S47
Bentea, Eduard, S299
Bentsen, Havard, S122
Benzian-Olsson, Natashia, S354
Berardi, Domenico, S123
Berardinelli, Laura, S319
Berberian, Arthur de Almeida, S255
Berberich, Diana, S121
Bercik, Premysl, S190
Berciu, Cristina, S235
Berdún, Jesús, S208
Berdowski, Woutje, S287
Beresniewicz, Justyna, S293
Berg, Akiah O., S113
Berg, David, S305
Berger, Gregor, S99, S116, S132, S228, S378
Berger, Maximus, S116, S228, S326
Berger, Philipp, S195
Berk, Michael, S177, S405, S411
Berman, Karen F., S85
Bernardin, Florent, S169
Bernardini, Francesco, S363, S409

Bernardo, Miguel, S351, S360
Bernardo, Miquel, S222, S318, S332
Bernasconi, Corrado, S371
Bernhard,Müller, S128
Berretta, Sabina, S14, S16, S235
Berry, Alexandra, S156, S388
Berthelot, Nicolas, S230
Bertolino, Alessandro, S83, S85, S100, S116, S193
Bertolucci, Bruno, S161, S266, S375, S375
Besteher, Bianca, S178, S287
Bestmann, Sven, S416
Betül,Abut, S211, S316, S419
Bharath, Rose, S181
Bhatia, Triptish, S63
Bhattacharyya, Sagnik, S27, S28, S105, S154, S259, S384, S413
Bhavsar, Vishal, S67
Bhuknik-Atzil, Ortal, S165
Biagianti, Bruno, S246
Bian, Dayi, S421
Biase, Maria Di, S77, S195, S196 S74
Biedermann, Falko, S135
Biegon, Anat, S65
Bighelli, Irene, S305
Bin, Kwak Yoo, S330
Binder, Elisabeth, S26
Binnewies, Julia, S287
Bioque, Miquel, S222
Birchwood, Max, S98, S156
Bird, Jessica, S335
Birk, Merete, S134
Birkeland, Kåre I., S205
Birnbaum, Michael, S100, S259, S267
Birulés, Irene, S238
Birules, Irene, S372
Bischof, Martin, S254, S331
Bismuth, Corinne, S420
Bitsch, Florian, S195
Bittner, Robert, S28, S30, S250, S387
Bjerregaard, Marie, S387
Blackman, Graham, S72
Blackman, Rachael, S244
Blair, Nicholas, S289
Blanchard, Jack, S264, S353
Blanquer, Ignacio, S424
Blasco, Drew, S54
Blasco, Maria A., S226
Blasi, Giuseppe, S85, S100, S116, S193
Bless, Josef J., S267
Bless, Josef, S113, S261, S306
Blest-Hopley, Grace, S154, S384
Bliksted, Vibeke Fuglsang, S336
Bliksted, Vibeke, S169, S252, S350
Blizovski, Meirav, S324
Bloemen, Oswald, S183, S275, S391
Bloomfield, Michael, S21
Blum, Stefan, S61
Bo Mortensen, Preben, S167
Bo, Qijing, S241, S329
Bobes, Julio, S119, S222, S420
Bobkov, Konstantin, S412
Bocaccio, Hernán, S286
Bodor, Davor, S257
Boehme, Rebecca, S278
Boehnke, Jan, S6
Bogers, Jan, S67, S239, S256
Bohlhalter, Stephan, S184
Bohlken, Marc, S109
Boies, Sébastien, S230
Bojesen, Kirsten Borup, S383
Bojesen, Kirsten, S224, S387
Boks, Marco P.M., S109
Boks, Marco, S82
Bolhuis, Koen, S231, S402
Bolt, Luke, S99
Bonet, Lucia, S200, S424
Bonet, Meritxell Tost, S253
Bonevski, Billie, S151
Bonivento, Carolina, S144, S147, S168
Bonoldi, Ilaria, S56, S81
Booij, Jan, S275, S391
Boot, Erik, S247
Bora, Emre, S300, S356
Bora, Lee, S362
Bordón, Claudia, S150
Borgan, Faith, S291
Borgwardt, Stefan, S102, S144, S147, S199, S209, S328, S331
Borland, Ron, S151
Borlido, Carol, S309
Borsboom, Denny, S269
Bortolato, Marco, S126
Bortolon, Catherine, S359
Bortolotti, Lisa, S159
Bortz, David, S339
Bosco, Francesca M., S319
Bose, Anushree, S345
Bossong, Matthijs, S28
Bottino, Cassio, S378
Boucher, Michael, S18
Bouix, Sylvain, S90
Bourque, Francois, S58
Bousman, Chad, S74, S176, S282, S295
Bowie, Chris, S31, S404
Bowtell, Meghan, S162, S270, S376
Boyd, Adrian, S317
Boyette, Lindylou, S269
Bozaoglu, Kiymet, S137, S246
Brønnick, Kolbjørn, S284, S417
Braš, Marijana, S257
Braff, David, S35
Bralet, Marie-Cécile, S268, S420
Brambilla, Paolo, S144, S147, S209, S328
Brammer, Mick, S413
Bran, Mihai, S160
Brandl, Eva, S218
Brandl, Felix, S283
Brandon, Nicholas, S105
Brandt, Allison, S289
Brandt, Jørgen, S167
Branka Restek,Petrović, S257
Brannigan, Ross, S379
Bray, Signe, S355
Breetvelt, Elemi, S247, S376
Breier, Alan, S61, S63
Brennan, Annie, S192
Brennand, Kristen, S32, S34
Bressan, Rodrigo A., S266
Bressan, Rodrigo, S96, S112, S153, S154, S161, S164, S164, S184, S255, S286, S375, S375
Brian, O'Donoghoe, S162
Brice, Nicola, S339
Brinkman, Loek, S242
Bristow, Greg, S233, S234, S234
Brivio, Paola, S202
Brix, Sophie, S116
Broberg, Brian V., S80
Broberg, Brian, S83, S119, S213, S224
Broder, Michael S., S366
Brodeur, Mathieu, S197
Bromet, Evelyn J., S40
Bronstein, Michael, S147
Brookes, Matthew, S171
Broome, Matthew R., S280
Brophy, Lisa, S151
Broughton, Susan, S233, S234
Broussard, Beth, S409
Brouwer, Rachel, S83
Brown, Eric, S329
Brucato, Gary, S233
Bruggeman, Richard, S117
Brugger, Stefan, S13
Brunoni, Andre, S133
Bryant, Caitlin, S54
Bryce, Shayden, S359, S404
Bryl, Karolina, S315
Bubl, Anna, S11
Bubl, Emanuel, S11

Bucci, Sandra, S197
Buchanan, Robert, S63, S176, S190, S422
Buchsbaum, Monte, S268
Buck, Gabriella, S182
Buclin, Thierry, S236
Budde, Monika, S155
Buil, Alfonso, S85
Buonaguro, Elisabetta Filomena, S204
Buonaguro, Elisabetta, S313
Bureau, Alexandre, S230
Burke, Erin, S235
Burke, Shadie, S272, S426
Burnet, Phil, S254
Burns, Jonathan, S271
Burrer, Achim, S174
Burton, Birgitte Klee, S110, S245, S252, S300
Butcher, Nancy, S247
Butjosa, Anna, S113, S212
Byford, Sarah, S57
Byrne, Enda, S12
Byrne, Kelly, S148
Caan, Matthan, S275, S391
Cabezas, Ángel, S253, S303
Cabungcal, Jan Harry, S1
Cadenhead, Kristen, S396
Cadenhead, Kristin S., S102, S231
Cadenhead, Kristin, S39, S62, S78, S79, S266, S422
Caforio, Grazia, S100
Cagliarini, Daniela, S4
Cagney, Gerard, S53, S72, S74
Cahn, Wiepke, S101
Cai, Helen, S73
Caillies, Stéphanie, S381
Cairns, Murray, S296, S398, S400
Calabrese, Francesca, S202
Calamante, Fernando, S91
Calkins, Monica, S57, S305
Callahan, Jennifer, S40
Calrco, Navona, S176
Calvo, Ana, S89, S255
Calvo, Anna, S183
Camilla, Avagliano, S313
Campbell, Alastair, S1
Campbell, Dónal, S318
Campbell, Donal, S147
Campbell-Meiklejohn, Daniel, S350
Canal-Rivero, Manuel, S374, S392Cañas, Benjamín, S186
Cañete, Carlos, S157, S424
Cannon, Mary, S53, S58, S72, S89, S109, S147, S181, S318, S379, S379, S402
Cannon, Tyrone D., S78, S231
Cannon, Tyrone, S35, S39, S62, S79, S102, S147, S266, S396, S422
Cano-Domínguez, Pablo, S151
Cao, Hengyi, S79
Capdevielle, Delphine, S359
Cappucciati, Marco, S383
Capuani, Clizia, S412
Capulong, Leslie, S409
Caravaggio, Fernando, S290, S329, S393
Carey, Eleanor, S147, S402
Carletti, Francesco, S50
Carlo, Pasquale Di, S193
Carlos, González-Vivas, S384
Carlström, Eva Lindholm, S223
Carlton, Mark, S339
Carmi, Dana, S54
Carney, Rebekah, S405
Carot, Jose Miguel, S200
Carpenter, William, S190
Carpenter,, William T.,Jr. S5, S37
Carr, Matthew, S388
Carr, Vaughan J., S229
Carr, Vaughan, S347
Carr, Vaughn, S296
Carrilero, Raquel Lopez, S210
Carrillo, Facundo, S43
Carrion, Ricardo, S266
Carroll, Vincent, S176
Carruthers, Sean, S78, S137, S143, S246, S359
Carter, Cameron, S4, S126, S140
Casaus, Laurie, S18
Cascia, Caterina La, S75, S258
Caspi, Avshalom, S25, S97, S110
Castañeda, Carmen, S269, S364
Castelao, Enrique, S254
Castellani, Laura, S104
Castle, David J., S151
Castle, David, S214
Castrén, Eero, S17
Castro, Mariana, S286
Castro-Fornieles, Josefina, S125, S181
Català, Ferran, S237
Catalán, Rosa, S332
Cather, Corinne, S361
Cattaneo, Annamaria, S127
Cavalcante, Daniel Azevedo, S153, S375
Cavalcante, Daniel, S96
Cavallato, Roberto, S196
Cecchi, Guillermo, S43, S76, S100
Cederlöf, Erik, S88
Celiker, Ayce, S345
Cella, Matteo, S196
Ceren,Hıdıroğlu, S356
Cermolacce, Michel, S47
Ceylan, Deniz, S356
Cha, Saetbyeol, S387
Cha, Yun-Ji, S242
Chadha, Radhika, S400
Chah, Chloe, S134
Chaimani, Anna, S94
Chakravarty, Mallar, S182, S390, S393
Chamorro, Jacobo, S237
Chan, Kit Wa, S138, S214, S215, S372
Chan, Nathan, S329
Chan, Raymond C. K., S244
Chan, Raymond, S161, S333, S358, S382
Chan, Sherry, S97
Chan, Suet In, S138
Chan-Golston, Alec, S216, S320
Chana, Gursharan, S74
Chance, Steven Andrew, S192
Chandratilleke, Madhu, S284
Chanen, Andrew, S105
Chang, Eunice, S97, S248, S366
Chang, Trina, S361
Chang, WC, S97
Chang, Wing Chung, S138, S214, S215, S243, S372
Chaparala, Srilakshmi, S298
Charles, Ellis, S120
Charters, Beth, S209
Chat, Saetbyeol, S391
Chatham, Chris, S340
Chaudhry, Imran, S131, S148
Chaumette, Boris, S26
Chavez, Sofia, S290, S329
Chavez-Baldini, UnYoung, S6, S307
Chawner, Samuel, S86
Chee, Michael, S281
Cheer, Joseph, S204
Chekroud, Adam, S157
Chelini, Gabriele, S16, S235
Chen, Chi-Ming, S2
Chen, Eric Y. H., S132
Chen, Eric YH, S243
Chen, Eric, S97, S99, S116, S138, S214, S215, S228, S372, S378
Chen, Jianqing, S176
Chen, Jun, S241
Chen, Kao Chin, S389
Chen, Kehui, S63
Chen, Maxine, S97
Chen, Po See, S389
Chen, Qiang, S85

Chen, Te-An, S188, S189
Chen, Yu-Jen, S186
Chen, Yuxiao, S19
Chesney, Edward, S310
Cheung, Eric F.C., S333, S358
Chevallier, Emmanuel, S268
Chi, Su-Hyuk, S403
Chianca, Camille, S195, S376
Chien, Yi-Ling, S186
Chiliza, Bonga, S364
Chiliza, Bonginkosi, S261, S367
Chiramberro, Marcelo, S345
Chisholm, Katharine, S209, S328, S334, S357
Chiu, Chui-de, S161, S389
Chiu, Sanyin, S138, S214
Choe, Ann, S289
Choi, Joonho, S257, S360
Choi, Kee-Hong, S240, S242, S260, S344
Choi, Sun, S240
Chokran, Cole M., S78
Chon, Eon Sol, S47
Choudhury, Munmun De, S100
Chow, Eva, S247
Chowbay, Balram, S309
Christensen, Anne Marie Raabjerg, S333
Christiani, Camilla J., S245
Christiani, Camilla Jerlang, S110, S252, S300, S336
Christiani, Camilla, S300
Chu, Min-yi, S358
Chu, On Ki, S138, S215
Chung, In Won, S203
Chung, Jun Ku, S290, S329, S393
Chung, Yoonho, S79, S396
Churchill, Madeline J, S20
Ciampoli, Mariasole, S38
Ciantar, Sarah, S368
Ciccarelli, Mariateresa, S414
Cid, Jordi, S238, S317, S348, S372
Cieslak, Kristina, S361
Cihakova, Daniela, S190
Cilia, Jackie, S339
Cinar, Ozan, S84
Ciufolini, Simone, S98
Clapcote, Stephen, S234
Clark, Jeffries, S62
Clarke, Latoya, S370
Clarke, Mary, S89, S109, S379
Cleary, Sean, S259, S409
Clementz, Brett A., S121
Clementz, Brett, S6, S14
Clemmensen, Lars, S103, S334, S336
Cleusix, Martine, S15, S25, S133, S236, S389, S395
Cobo, Jesús, S410
Cobo, Jesus, S370, S423
Cocchi, Luca, S91
Coello, Christopher, S385
Coffman, Brian, S288, S390
Cogo-Moreira, Hugo, S266
Cohen, Neta, S198
Cohen, Reut, S202
Cohen-Woods, Sarah, S347
Cohn, Tony, S413
Colantuoni, Carlo, S85
Colizzi, Marco, S246, S384, S413
Collazzoni, Alberto, S267
Collell, Rebeca García, S423
Collia, Deanna, S339
Collier, Tracey, S81
Collier, Tracy, S383
Collin, Guusje, S104
Collins, Anne, S340
Collip, Dina, S84
Colom, Francesc, S5
Common, Stephanie, S321
Comps, Kristiina, S293
Compton, Michael T., S201
Compton, Michael, S167, S178, S259, S409
Conforti, Marco, S105
Conn, P. Jeffrey, S70, S70
Connell, Melissa, S379
Constantinescu, Cristian, S176
Conus, Philippe, S15, S25, S133, S236, S279, S310, S389, S395
Cookey, Jacob, S153
Cooper, Helen, S37
Cooper, Jonathan, S65
Cooper, Owen, S241
Cope, Zackary, S233
Corbett, Ged, S339
Corcoran, Cheryl, S42, S43, S54, S76, S331
Cordeiro, Quirino, S96, S153, S266, S375
Cordes, Joachim, S343
Cornblatt, Barbara A., S78, S102, S231
Cornblatt, Barbara, S39, S62, S79, S266, S396, S422
Cornea, Emil, S103
Coromina, Marta, S212
Corral, Ricardo, S371
Correll, Christoph U., S266, S341, S342
Correll, Christoph, S86, S93, S108, S206, S207, S236, S333, S341, S363, S375
Corripio, Iluminada, S208, S318
Corsi, Mauro, S412
Corsi-Zueli, Fabiana, S329
Corsi-Zuelli, Fabiana, S227
Cortes-Briones, Jose, S27
Cortizo, Romina, S237
Corvin, Aiden, S141
Cosci, Fiammetta, S76
Costanzi, Monise, S288
Costanzo, Elsa, S286
Costas, Javier, S398
Cotel, Marie, S191
Cotel, Marie-Caroline, S65
Coto, Ana, S119, S420
Cotter, David, S53, S72, S74
Cotter, Jack, S145, S248, S353
Coughlan, Helen, S89, S147
Coughlin, Jennifer, S8
Coull, Jennifer, S47
Courouve, Laurène, S125
Courtet, Philippe, S96
Cousins, Ann, S236
Coutinho, Luccas, S96, S153, S154
Coutrot, Antoine, S249
Covey, Daniel, S204
Covington, Michael, S409
Coyle, Joseph, S1
Crabbe, Frances, S282
Craven, Alex, S302
Craven, Alexander R., S292
Crawford, Trevor, S301
Cremers, Dimitri, S219
Crespo-Facorro, Benedicto , S259
Crespo-Facorro, Benedicto, S92, S142, S184, S251, S268, S276, S373, S374, S392
Cress, Rosemary, S4
Crichton, Jonathan, S408
Crippa, Alessio, S94
Crippa, Jose Alexandre, S28, S297
Crisafio, Anthony, S409
Critchley, Hugo, S173
Crocker, Candice, S295
Croft, Jazz, S377
Cropley, Vanessa, S74, S91, S176
Crosas, Josep Maria, S251
Crossland, Georgia, S323
Crossley, Nicolas, S202, S269, S364
Crump, Francesca, S54
Crush, Eloise, S110
Cryan, John, S59
Cuadra, Meritxell Bach, S395
Cubała, Wiesław, S27

Cucchiaro, Josephine, S114, S239, S353
Cuenod, Michel, S2, S15, S25, S133, S236
Cuesta, Manuel J., S318, S374
Cui, HuiRu, S223, S366
Cui, Xi-long, S333
Cullen, Alexis, S102, S118, S143
Cunningham, Janet, S223
Curtis, Logos, S388
Curtis, Vivienne, S72
Czajkowski, Nikolai, S250
Czamara, Darina, S26
Czepielewski, Leticia, S140, S288

D'Alfonso, Simon, S4
D'Ambrosio, Enrico, S68, S225
D'Ambrosio, Luigi, S204, S312, S414
D'Souza, Deepak, S27, S63
Dadabhoy, Maria, S191
Dahoun, Tarik, S385
Dai, Jing, S331
Dalibor,Karlović, S369
Dalman, Christina, S67, S377
Dalmau, Pere Bonet, S205
Dalrymple, Anthony, S72
Dalton, Caroline, S329
Daly, Eileen, S395
Daly-Ryan, Niamh, S349
Damo-Csorba, Bori, S192
Danese, Andrea, S97, S110
Daniel, David, S131, S135
Dantas, Clarissa De Rosalmei, S349
Dantas, Clarissa, S146, S262, S425
Dantzer, Robert, S48
Dark, Frances, S214, S237
Darrelmann, Ute, S128
Das, Tushar, S79, S82, S90, S102, S170, S179, S179, S199, S373
Daumit, Gail, S12
Dauvermann, Maria M., S325
Dauvermann, Maria, S325
Davey, Christopher G., S105
David, Anthony, S39, S58, S72, S77, S87, S92, S98, S166, S246, S247, S328, S374, S396
Davidson, Michael, S23
Davies, Cathy, S56, S162, S323, S383
Davies, Geoff, S173, S212
Davis, Bethany, S139
Davis, John, S94
Davis, Robert, S236, S320, S341
Dawson, Neil, S233, S234, S234
Dazzan, Paola, S6, S48, S50, S77, S92, S98, S118, S131, S148, S166, S246, S367
De Haan, Lieuwe, S116, S269
De Hert, Marc, S84
De Jong, Steven, S260
De La Fuente-Tomas, Lorena, S119, S420
De la Serna, Elena, S125, S229
DeLisi, Lynn, S34, S194
De Luca, Vincenzo, S290
DeMarshall, Cassandra, S328
De Micheli, Andrea, S162, S383
De Nijs, Jessica, S101
De Pino, Gabriela, S286
DeRosse, Pamela, S176, S422
DeVylder, Jordan, S379
Deakin, Bill, S131, S148
Deakin, John, S48
Dean, Brian, S70
Dean, Derek, S42
Dean, Kimberlie, S229
Decoster, Jeroen, S84
Deenik, Jeroen, S407
Degenhardt, Franziska, S287
Degirmencioglu, Banu, S368
Del Cacho, Núria, S113, S212
Del Re, Elisabetta, S90
Del-Ben, Cristina Marta, S329
Del-Ben, Cristina, S166
Delacretaz, Aurélie, S310
Delespaul, Philippe, S84
Delgado-Alvarado, Manuel, S392
Dellazizzo, Laura, S346
Delucchi, Kevin, S4
Demmin, Docia, S10, S401
Dempster, Kara, S179, S179, S207
Demro, Caroline, S43
Demyanovich, Haley, S190
Deneyer, Lauren, S20
Deng, Ling, S239, S353
Denny, Thomas, S120
Denton, Jennifer, S3
Depasquale, Erica, S299
Derogatis, Leonard, S139
Derom, Catherine, S84
Desai, Priyal, S405
Deserno, Lorenz, S278
Deshpande, Gopikrishna, S170, S181
Deshpande, Smita, S63
Devenyi, Gabriel A., S121
Devenyi, Gabriel, S182
Dey, Avyarthana, S221, S327
de Aguileta, Itziar Riera-López, S113, S212
de Apraiz, Ana, S317
de Bartolomeis, Andrea, S204, S312, S313, S414
de Freitas, Rosana, S371
de Graaf, Ron, S160
de Haan, Lieuwe, S86, S107, S132, S239
de Hann, Lieuwe, S378
de Jong, Steven, S238
de Luis-García, Rodrigo, S392
de Mamani, Amy Weisman, S265, S371
de Moura, Luciana Monteiro, S184
de Sousa, Ana Elisa Farias, S197
de Witte, Lot, S261
de la Foz, Victor Ortiz-García, S92, S184
de la Serna, Elena, S181, S183
de-Luis-Matilla, Antonia, S151
dos Santos, Antonio, S166
dos Santos, Bernardo, S371
dos Santos, Eriton, S378
Dhawan, Jasbeer, S65
Di Biase, Maria, S91
Di Carlo, Pasquale, S116
Di Fabio, Fabio, S393
Di Forti, Marta, S98, S110, S123, S158, S166, S354
Di Sarno, Elaine, S210
DiForti, Marta, S332
DiPetrillo, Lauren, S312
Diaconescu, Andreea, S254, S281, S314
Diaz, Camila, S364
Diaz-Caneja, Covadonga M., S226
Díaz, Luciana, S372
Dicker, Patrick, S74
Dickerson, Faith, S62, S63, S63, S87
Dickie, Erin, S176, S226
Dickson, Hannah, S143
Didriksen, Michael, S1
Dieset, Ingrid, S227
Dieter, Lesa, S374
Dietzek, Maren, S287
Diez, Alvaro, S186
Diez-Aja, Cristobal, S237
Dillon, Rachael, S349
Dipasquale, Ottavia, S83
Diukova, Anna, S283
Dixon, Lisa, S107
Dmitrienko, Alex, S236, S341
Do, Kim Q. Do, S395
Do, Kim Q., S2, S15, S133, S279, S310, S389
Do, Kim, S25, S25, S236
Docherty, Anna, S333

Dolan, Raymond, S385
Dollfus, Sonia, S390, S396
Dolmer, Signe, S134
Dolz, Montse, S229
Dolz, Montserrat, S183
Domènech, Cristina, S423
Dong, Fang, S241
Donkersgoed, Rozanne van, S238
Donohoe, Gary, S48, S141, S325, S325, S349
Doody, Gillian, S6, S77
Dooley, Niamh, S147, S181, S402
Dorner-Ciossek, Cornelia, S128, S380
Dotsch, Ron, S242
Douvold, Laëtitia Bouérat, S241
Downey, Linglee, S162, S270, S376
Downing, Donna, S54
Doyle, Joseph, S412
Draženka,Ostojić, S369
Drake, Richard, S50, S131, S148, S156, S197, S388
Drakesmith, Mark, S396
Drosos, Petros, S417
Drucaroff, Lucas, S286
Du Plessis, Stéfan, S367
Du, Xin, S338
Du, Ya-song, S333
Du, Yong, S8
Duan, Jubao, S34
Duarte, Rodrigo, S105
Dubath, Céline, S310
Dubovsky, Steve, S188, S189
Duburcq, Anne, S125
Dukart, Juergen, S83
Dulcis, Davide, S233
Dumais, Alexandre, S346
Duncan, Erica, S266
Duncan, Marcus, S413
Dunn, Graham, S131, S148
Dunn, Walter, S63
Dunne, Thomas Frederick, S280
Duval, Céline, S140
Dwyer, Dominic B., S185
Dwyer, Dominic, S100, S155, S246, S292, S316

Eap, Chin B., S236, S310
Eaton, Scott, S162, S270, S376
Eaton, William, S190
Ebbesen, Christine, S333
Ebdrup, Bjørn H., S289
Ebert, Dieter, S11
Edden, Richard, S20
Eduard, Bentea, S299
Edwards, Jordan, S108, S271
Egan, Michael F., S85
Egerton, Alice, S51, S81, S89, S382
Egger, Stephan, S331
Eggers, Susanne, S285
Ehlers, René, S399
Ehrenreich, Hannelore, S85
Eichhorn, Peter, S261
Eickhoff, Simon B., S393
Eiroa-Orosa, Francisco José,
Eisner, Emily, S197
Ek-Persson, Jane, S211
El Hechmi, Zouhaier, S163, S258
El Marroun, Hanan, S231
Eleftheriadis, Dina, S4
Elias, Beatriz Yukie, S375
Elise, Leroux, S390
Elkis, Hélio, S266
Elkis, Helio, S262, S371, S378
Ellersgaard, Ditte V., S110, S300
Ellersgaard, Ditte, S245, S252, S336
Elzinga, Bernet, S347
Emsley, Richard, S197
Emsley, Robin, S261, S364, S367
Ena,Ivezić, S257, S257
Engelstad, Katharina, S253
Engemann, Kristine, S59
English, Jane, S53, S72
Epdrup, Bjørn, S134
Eplov, Lene, S134
Ered, Arielle, S136
Erickson, Molly, S10
Ermiyev, Michael, S198
Ernala, Sindhu, S100
Erp, Theo van, S79
Escarti, Maria Jose, S424
Esen, Duygu, S316
Esmaeilzadeh, Abdolreza, S344
Espina-Eizaguirre, Alberto, S151
Espinosa, Justo Pinzón, S337
Espinosa, Laura, S337
Essig, Marco, S289
Esther Setién Suero, S259
Estrada, Francesc, S203, S251, S253, S303, S311
Ettinger, Ulrich, S119, S386
Eurelings-Bontekoe, Elisabeth, S341
Evans, Jonathan, S371
Evans-Lacko, Sara, S54, S55
Evenden, John, S248, S353
Everall, Ian, S74, S176, S224, S295
Evins, Anne Eden, S361
Ewertzon, Mats, S211

Fañanás, Lourdes, S330
Fabiana, Corsi-Zuelli, S222
Fachim, Helene, S329
Faerden, Ann, S369
Fagerlund, Birgitte, S80, S83, S213, S226, S232, S289
Faiola, Eliana, S119, S386
Faith, Laura, S362
Falkai, Peter, S17, S155, S261, S292, S293, S342, S343, S355, S393
Falkenberg, Irina, S195
Fallgatter, Andreas, S355
Fancis, Susan, S171
Fankam, Cédine, S17
Fanshawe, Thomas, S23
Faravelli, Laura, S126, S132
Farhall, John, S302
Farhat, sarah-Lise, S420
Farkash, Herman, S198
Farmah, Baldeep, S280
Fathian, Farivar, S324, S358
Fatjó-Vilas, Mar, S145, S174, S330, S397
Fatma Betül, Abut, S211, S419
Faulkner, Guy, S413
Fazel, Seena, S23
Fearon, Paul, S77
Fedechko, Taylor, S3
Federspiel, Andrea, S175, S184
Feher, Virginia, S192
Feixas, Guillem, S210
Feldman, Stephanie, S190
Felsenheimer, Anne, S136
Ferchiou, Aziz, S160, S166
Fernandes, Thalita, S146, S262, S349, S425
Fernandez-Castilla, Belén, S255
Fernandez-Egea, Emilio, S351, S360
Ferrara, Maria, S272, S426
Ferrari, Carina, S236, S389
Ferraro, Laura, S7, S75, S110, S158, S258, S354
Ferreira, Felipe, S255
Ferreira-Santos, Jair, S166
Ferretjans, Rodrigo, S22
Ferri, Mila, S426
Fervaha, Gagan, S267, S306, S309, S354, S393
Fethi, Guemira, S152
Figueiredo, Anna Suarez, S26
Filipova, Nina, S406
Fioramonte, Mariana, S53
Firmin, Ruth, S319
Firth, Joseph, S405
Fisher, Helen, S25, S97, S110, S118, S166
Fiszdon, Joanna, S196
FitzGerald, Thomas, S385
Fitzgerald, Zachary, S194

Fitzsimmons, Chantel, S296, S398
Flavia,Müller, S127, S234
Fleischer, Christian, S122, S228
Fleischhacker, Wolfgang, S50, S421
Fleischman, Anat, S23
Fletcher, Paul, S306
Flores, Cecillia, S37
Föcking, Melanie, S74
Foecking, Melanie, S53, S72
Foldager, Leslie, S252, S341
Foley, Fiona, S302
Fonseca, Ana Olívia, S255
Fonseca, Lais, S112
Fonseka, Trehani, S190
Fonville, Leon, S396
Forner, Mireia, S123
Fornito, Alexander, S91, S176
Forrest, Emma C., S132
Fortea, Adriana, S181, S183, S337
Forti, Marta Di, S7, S59, S75, S84, S246, S258, S364, S373
Foss-Feig, Jennifer, S266
Foster, Dan, S70
Foster, Daniel, S204
Fournier, Margot, S15, S25, S133, S236
Foussias, George, S176, S267, S306, S309, S354, S422
Fowler, David, S212
Fraccaro, Paolo, S106
Fraguas, David, S226
Frajo-Apor, Beatrice, S421
Francesco, Danilo Notar, S204, S312, S414
Francey, Shona, S162
Franchino-Elder, Jessica, S425
Francis, Andrew, S260
Francis, Emma, S84
Francis, Michael, S63
Franck, Nicolas, S47
Franck, Schürhoff, S166
Franco, Ana Luiza, S255
Frank, Michael, S340
Franscini, Maurizia, S230, S336
Fraser, Elizabeth, S118
Freeman, Daniel, S335
Freeman, Natalie, S218
Freitas, Elder Lanzani, S376
Freitas, Elder, S195
Freitas, Rosana, S262, S266, S371
Fresno, Andrés, S96
Freudenreich, Oliver, S361
Friedel, Evelyn, S11
Friedman, Zvika, S339
Friis, Svein, S272
Frisch, Katharina, S178
Fruchter, Eyal, S165
Fu, Susie, S250
Fuglø, Dan, S383
Fujimoto, Michiko, S419
Fujino, Haruo, S419
Fujito, Ryoshuke, S290
Funatogawa, Tomoyuki, S315
Funk, Adam, S19, S52
Furlan, Victoria, S210
Furnham, Adrian, S368
Furuichi, Atsushi, S394
Fusar-Poli, Paolo, S162, S323, S374
Fusar-poli, Paolo, S55, S56, S383
Fusaroli, Riccardo, S169, S350
Futatsubashi, Masami, S277

Gade, Katrin, S155
Gadelha, Ary, S96, S112, S153, S154, S161, S164, S184, S255, S266, S286, S375, S375
Gaebel, Wolfgang, S343
Gage, Suzanne, S165, S270
Gagné, Anne-Marie, S11, S124
Gago, Estefania, S251
Gagsteiger, Anna, S261
Gaisler-Salomon, Inna, S202
Gajšak, Tomislav, S257
Gajwan, Ruchika Gajwan, S385
Gajwani, Ruchika, S279, S282, S340, S409
Galbraith, Jordan, S204
Galderisi, Silvana, S50, S196, S263, S264
Gale, Tim, S220
Gallardo, Carlos, S269
Gallego, Carme, S174
Gallego, Juan, S221
Gallegos, Yuli, S123
Galletly, Cherrie, S73, S137, S208, S214, S284, S408
Galletti, Chiara, S201
Galli, Aurelio, S45
Galling, Britta, S108, S206
Gama, Clarissa, S22, S140, S288
Gamble, Clare, S178
Ganapathiraju, Madhavi, S189, S298
Ganella, Eleni, S91, S176
Gangadhar, B. N. , S136
Gantriis, Ditte L., S245, S300
Gantriis, Ditte Lou, S110, S336
Gantriis, Ditte, S252
Garcés, María Sol, S352
García-Bueno, Borja, S222
García-Mieres, Helena, S210, S317, S427
García-Rizo, Clemente, S332
Garcia, Cristina, S264, S353
Garcia, Jordi Soler, S330
Garcia, Miguel, S139
Garcia-Alvarez, Leticia, S119, S420
Garcia-Carmona, Juan-Antonio, S206
Garcia-Marti, Gracian, S276
Garcia-Portilla, M Paz, S119, S420
Garcia-Ribera, Carles, S410
Gardner-Sood, Poonam, S166
Garfinkel, Sarah, S173
Garrett, Chantel, S267
Garrido, Viviana, S96
Garverich, Suzanne, S137
Garzitto, Marco, S147
Gascoyne, Lauren, S171
Gaser, Christian, S122, S178, S287
Gass, Peter, S297
Gati, Joe, S179, S179
Gattaz, Wagner, S133, S195, S376
Gaughran, Fiona, S87, S166, S332
Gautier, Corinne, S420
Gawęda, Łukasz, S146, S252
Gawne, Timothy, S120
Gayer-Anderson, Charlotte, S7, S58, S110, S156, S158, S307, S354
Geaghan, Michael, S398, S400
Geels, Camilla, S167
Geerts, Hugo, S294, S411
Gehr, Johannes, S422
Gelao, Barbara, S100
Genberg, Maria, S407
Geng, Dawei, S69
Genske, Anna, S285
Geraets, Chris, S344, S419
Gerke, Wolfgang, S331
Gerretsen, Philip, S290, S329, S393
Gerstenberg, Miriam, S230, S336
Gertsik, Lev, S340
Gholam-Razaee, Mehdi, S236
Gholam-Rezaee, Mehdi, S310
Giacca, Adria, S104
Giampietro, Vincent, S154, S174, S413
Giannakopoulou, Alexandra, S243, S348
Giannakopoulou, Dimitra, S243, S348
Gicas, Kristina, S291
Giersch, Anne, S47
Gifford, George, S323
Gil, Roberto, S44
Gilbert, Elsa, S11, S142, S149, S230, S254

Gill, Kathryn, S1, S338
Gill, Michael, S357
Gillam, John, S316
Gilleen, James, S199
Gillespie, Amy, S382
Gillis, David, S61
Gilmore, John, S103, S194
Giménez, Anna, S337
Gingerich, Susan, S216, S320, S322
Gingras, Nathalie, S11
Giordano, Annalisa, S118, S131
Giordano, Giulia Maria, S264
Giordano, Giulia, S263
Girchenko, Polina, S26
Gisela, Sugranyes, S125
Gislum, Mette, S94
Gisselgård, Jens, S284
Giuliano, Anthony, S39
Gizdic, Alena, S196
Gjestad, Rolf, S324, S346, S358
Gladyshev, Igor, S213
Glass, Steven, S236, S341
Glatard, Anaïs, S310
Glausier, Jill , S29
Gleeson, John F, S4
Glenthøj, Birte Y., S289
Glenthøj, Birte, S80, S213, S383, S424
Glenthøj, Louise Birkedal, S119
Glenthoj, Birte, S50, S83, S119, S224, S226, S232, S314, S387, S422
Glerean, Enrico, S47
Glynn, Shirley, S216, S320, S322
Gobbicchi, Chiara, S363
Goedhart, Matthieu, S219
Goense, Jozien, S282
Gogate, Jagadish, S311
Goghari, Vina, S291, S327
Gohar, Sherif M., S227
Golay, Philippe, S236, S310, S395
Gold, James, S43, S140, S176, S190, S422
Goldman, Barbara, S103
Goldman, Howard, S4
Goldman, Robert, S239, S353
Goldring, Abraham, S244, S408
Goldstein, Benjamin I., S355
Goldzweig, Gil, S350
Gomes, Felipe, S21, S75
Gomes, Flavia C. A., S15
Gómez-Gastiasoro, Ainara, S347
Goncalves, Vanessa, S218
Gong, Qiyong, S92
González, Beatriz, S370
González, Fermin, S238, S348
González-Blanco, Leticia, S119, S420
González-Fernández, Alba, S251
González-Higueras, Fermín, S317
González-Rodríguez, Alexandre, S203, S311
Gonzales, Antonio, S312
Gonzalez, Alfonso, S269
Gonzalez, Carlos, S157, S200, S276
Gonzalez-Pinto, Ana, S222, S318
Gonzalez-Valderrama, Alfonso, S202, S364
Gooding, Diane, S198
Gooding, Patricia, S220
Goodyear, Bradley, S79
Gopal, Srihari, S130, S311
Gorbovskaya, Ilona, S190
Gordianno, Annalisa, S48
Gordon, Anne, S237
Gordon, Pedro, S133
Gore-Jones, Victoria, S237
Goulding, Anneli, S212, S426
Gowatsky, Jaimie, S331
Gowland, Penny, S180, S180
Goyal, Parth, S405
Gozlan, Guy, S125
Gräger, Stephanie, S287
Grace, Anthony, S1, S75, S338, S339, S416
Gracián, García-Martí, S384
Graff-Guerrero, Ariel, S290, S329, S354, S393
Grah, Majda, S257
Graham, Christine, S413
Graham, Stephen M, S132
Granger, Kiri, S145, S248, S353
Granholm, Eric, S77, S123, S195, S421
Grasa, Eva, S208, S210, S238, S317, S348, S372
Grases, Núria, S113, S212
Gray, David, S176
Grayson, Ben, S128
Grech, Adrienne, S338
Green, Alan, S235
Green, Mallik, S241
Green, Melissa, S229, S296, S347
Greene, Mallik, S97, S248, S366
Greenwood, Kathryn, S87, S212
Greenwood, Kathy, S173
Gregersen, Maja, S245, S300, S336
Greis, Kenneth, S52
Grent-'t-Jong, Tineke, S279, S282, S340, S385
Greve, Aja, S110, S245, S252, S300, S336
Griffa, Alessandra, S15, S25, S133, S279
Griffiths, Sian, S98
Grigis, Antoine, S172
Grigoriou, Markella, S159
Grinband, Jack, S331
Groc, Laurent, S60
Gronholm, Petra, S55
Groot, Christopher, S200
Gross, Amit, S202
Gross, Joachim, S279, S282, S340, S385, S409
Grottick, Andrew J., S128
Grove, Amber, S362
Gruber, Oliver, S275
Gruener, Daniel, S340
Gruetter, Rolf, S133, S236
Guardiola, Maria, S145, S174, S397
Gudbrandsen, Maria, S395
Guerrero-Pedraza, Amalia, S145
Guevara, Miguel, S172
Guevara, Pamela, S172
Guillory, Sylvia, S266
Guimaraes, Francisco Silveira, S28
Guimaraes, Francisco, S21
Guimond, Synthia, S121
Guinjoan, Salvador M., S286
Güliz,Özgen, S316
Guloksuz, Sinan, S84, S160, S272, S426
Guma, Elisa, S390
Gumley, Andrew, S279, S282, S385, S409
Gundersen, Steffie, S334
Gunther, Nicole, S160
Guo, Shuixia, S187
Gupta, Anoop, S405
Gupta, Tina, S42
Gur, Raquel, S57, S63
Gur, Ruben, S57, S63
Gurel, Seref Can, S345
Gurung, Bina, S405
Gurvich, Caroline, S137, S139, S143, S246, S252
Gussew, Alexander, S121, S221
Gutierrez-Zotes, Alfonso, S303
Güzide,Örüm, S316

Haaki, Willehard, S88
Haan, Lieuwe de, S99, S228
Haapea, Marianne, S144, S168
Haas, Shalaila, S168, S246
Haber, Lillian, S309

Haddock, Gill, S50
Haddock, Gillian, S220
Hafizi, Sina, S390
Häggmark-Månberg, Anna, S120
Hagen, Roger, S113
Hagi, Katsuhiko, S207, S363
Hagmann, Patric, S15, S25, S133, S279
Hahn, Margaret, S104, S190, S313, S413
Hahn, Peter, S30
Haidl, Theresa, S114, S209, S285
Hajak, Göran, S343
Hajdúk, Michal, S250, S360
Häkkinen, Katja, S88
Hakala, Mika, S94
Haker, Helene, S72, S281, S331
Hall, Emma, S171
Hall, Graham, S209
Hallak, Jaime Eduardo, S297
Hallett, Danielle, S334
Halpern, Brooke, S409
Haltigan, John, S422
Hamann, Stephan, S79
Hamilton, Emily, S85
Hamilton, Holly, S266
Hamilton, Matthew, S404
Hammerton, Gemma, S379
Hanen, Ben Ammar, S152
Hanna, Donncha, S317
Hanrahan, John, S72
Hansbauer, Maximilian, S343
Harada, Akina, S412
Hardingham, Kelton, S200
Hargovan, Hethal, S61
Hargreaves, April, S141, S349, S357
Haro, Josep Maria, S371
Harripaul, Ricardo, S296
Harris, Anthony, S192
Harris, Kamelia, S220
Harrisberger, Fabienne, S102, S199
Harte, Michael, S312
Hartley, Brigham, S34
Hartman, Catharina, S14
Hartman, Leah, S368
Hartman, Richard D., S132
Hartmann, Jessica A., S105, S124
Hartmann, Jessica, S378
Hartry, Ann, S97, S241, S248, S366
Harvey, Philip D., S353
Harvey, Philip, S40, S159, S208, S242, S320
Hasan, Alkomiet, S17, S261, S342, S343, S355, S393, S406, S416
Haselden, Morgan, S108
Hashimoto, Ryota, S85, S419
Hasking, Penelope, S379
Hasson-Ohayon, Ilanit, S260, S350
Hastrup, Lene Halling, S129
Hatada, Sanae, S290
Hathway, Pamela, S395
Haug, Elisabeth, S356, S410
Haugen, Ingvild, S356
Haugg, Amelie, S81
Hauke, Daniel, S102, S314
Hawar, Moradi, S159
Hawco, Colin, S176
Hawkins, Peter CT, S83
Hayata, Jacqueline, S18
Hayes, Richard, S411
Hazan, Hadar, S335
Hazlett, Erin, S240
He, Manxi, S331
He, Yujie, S82
He, Zhimin, S106
Healy, Colm, S109, S147, S181, S318
Heaney, Lisa, S312
Hébert, Marc, S11
Hechmi, Zouhaier EL, S371
Hechmi, Zouhaier El, S403, S423
Heffetz-Giterman, Lior, S202
Hefner, Amanda, S408
Hegelstad, Wenche ten Velden, S272
Heger, Anja, S128
Heidegger, Tonio, S340
Heilbronner, Urs, S155
Heinrichs, Walter, S149, S368
Heinsbroek, Jasper, S337
Heinz, Andreas, S172, S278
Heinze, Kareen, S209, S280, S370
Heinzle, Jakob, S314, S331
Heiskanen, Inkeri, S349
Helkis, Helio, S133
Helldin, Lars, S159, S263, S265
Hellemann, Gerhard H., S136
Hellström, Cecilia, S120
Hellvin, Tone, S113
Helm, Meghan, S188, S189
Hemager, Nicoline, S110, S245, S252, S300, S336
Henderson, David, S139
Hendriksen, Ingrid, S407
Henning, Bettina, S355
Henning, Draheim, S143
Henriksen, Maria Toft, S252
Henriques, Beatriz, S295
Herdener, Marcus, S284
Heretik, Anton, S250
Herman, Yarissa, S31
Hernandez, Miguel, S157, S424
Hernandez-Garcia, Maria, S157
Hernaus, Dennis, S182
Herold, Christina, S140, S289
Heron, Jon, S165, S270, S377, S379
Heslin, Margaret, S6
Hetrick, Sarah, S404
Heywood, Brodie, S295
Hickie, Ian B., S132
Hickie, Ian, S99, S116, S228, S378
Hickman, Matthew, S270
Hidalgo-Mazzei, Diego, S5
Hielscher, Emily, S379
Hietala, Jarmo, S69, S88, S272, S391
Higuchi, Cinthia, S153, S154, S161, S266, S375
Higueras, Fermin Gonzalez, S372
Hilker, Rikke, S80, S83, S213
Hill, Rachel, S338
Hillegers, Manon H.J., S231, S402
Hillegers, Manon, S77, S225, S287
Hinkley, Leighton, S278
Hirano, Yoji, S117
Hirjak, Dusan, S140, S289
Hirnstein, Marco, S261, S267, S302
Hitchcock, Steve, S339
Hjärthag, Fredrik, S159, S263, S265
Hjelmervik, Helene, S292
Hjorthøj, Carsten, S95, S111, S129, S163
Hjorthoej, Carsten, S365
Hjorthoj, Carsten, S99
Hjortshøj, Carsten, S134
Hlinka, Jaroslav, S173
Ho, Chun, S215
Ho, Newfei, S281
Ho, Wui Hang, S372
Hobson, Scott, S128
Hochard, Christophe, S420
Hodgins, Sheilagh, S143
Hoek, Hans W., S271
Hofer, Alex, S135, S421
Hoffman, Gabriel, S34
Hoffmeyer, Debra, S63
Hol, Elly, S82
Holland, Jessica, S325
Hollingworth, Sam, S178
Holmans, Peter, S379
Holzner, Bernhard, S135
Homan, Philipp, S330
Honer, William, S97, S290, S291, S343
Hong, Elliot, S20
Hong, Ji-Eun, S177, S411
Hong, Kyung Sue, S215, S362
Hong, L. Elliot, S327

Hong, Seok Jin, S47
Hong, Yeon-Ju, S308
Honkanen, Jarno, S68
Hood, Rebecca L, S20
Hopkins, David, S87
Hopkins, Richard, S106
Hopkins, Seth, S158
Hopkins, Stephen, S131, S148
Hopper, Shaun, S70
Hoptman, Matthew, S175, S331
Hopwood, Malcolm, S260
Horacek, Jiri, S173
Horacek, Magdalena, S128
Horga, Guillermo, S44
Hortêncio, Lucas, S195, S376
Houde, John, S278
Houenou, Josselin, S172
Houle, Sylvain, S43, S390
Houtepen, Lotte, S82
Howard, Robert, S377
Howes, Oliver, S13, S68, S81, S93, S225, S291, S330, S385, S395
Hsieh, Ming-Hsien, S186
Hsu, Jay, S114
Hsu, Yung-Chin, S186
Hu, Hui-xin, S358
Hua, Jun, S289
Hua, Lingling, S385
Huang, Debbie, S54
Huang, Jing-Ying, S186
Hubl, Daniela, S407
Huda, Samei, S156
Huerta-Ramos, Elena, S208
Hugdahl, Kenneth, S95, S113, S261, S292, S293, S302, S306
Hughes, Matthew, S78
Hughes, Rebecca, S233, S234
Huhtaniska, Sanna, S415
Hui, Christy, S97
Hui, Lai Ming, S138, S214, S215, S372
Hulkko, Anja, S144, S415
Hulshoff Pol, Hilleke E., S83
Hulshoff Pol, Hilleke, S82
Humberstone, Verity, S214
Humensky, Jennifer, S107
Hunnunen, Matti O., S379
Hurtig, Tuula, S101
Husain, Nusrat, S131, S148
Huynh, Helen, S415
Hwang, Na-Ri, S242
Hwang, Tae-Yeon, S240
Hwang, Tzung-Jeng, S186
Hwang, Wu Jeong, S294
Hwu, Hai-Gwo, S186
Hyötyläinen, Tuulia, S69
Hynninen-Sundelin, Ira, S345
Hyung, Lee Tak, S330
Hyytiäinen, Matias, S345

Iacoboni, Marco, S176
Iasevoli, Felice, S204, S312, S313, S414
Ibañez, Manuel Ignacio, S330
Ibad-Ramos, Marina, S167
Ibarretxe-Bilbao, Naroa, S347
Ichinose, Megan, S29, S77, S195, S196, S421
Idris, Nagi, S128
Iglesias, Celso, S119, S420
Iglesias, Sandra, S281, S331
Igor,Filipčić, S257, S257
Ik Cho, Kang, S186, S294
Ildiko, Piszar, S143
Ilvonen, Jenna, S345
Ilzarbe, Daniel, S125, S181, S183
Im, Chang-Hwan, S187
Immonen, Johanna, S144, S270
In Chan, Suet, S214
In Kang, Jee, S188
Incedere, Aysel, S419
Ingvild, Aase, S284
Inta, Dragos, S297
İncedere, Aysel, S211, S316
Iosif, Ana-Maria, S3, S126
Iqbal, Ehtesham, S411
Irani, Sarosh, S61
Iruretagoyena, Barbara, S202, S269, S364
Irvine, Karen, S220
Isdohanni, Matti, S168
Isenberg, Keith, S425
Ismail, Khalida, S87
Isohanni, Matti, S144, S270, S415
Isometsä, Erkki, S88
Isvoranu, Adela-Maria, S269, S367
Ithal, Dhruva, S136
Iversen, Trude S., S227
Iversen, Trude, S205, S362
Ivona Šimunović,Filipčić, S257, S257
Iwata, Yusuke, S290, S329, S393
Iyegbe, Conrad, S84, S156, S189, S332
Iyer, Srividya, S56

Jääskeläinen, Erika, S144, S168, S208, S270, S415
Jönsson, Erik, S205
Jørgensen, Hugo A, S346
Jørgensen, Hugo A., S358
Jørgensen, Robert, S153, S284
Jürgen,Dukart, S340
Jablensky, Assen, S214, S365
Jackowski, Andrea Parolin, S184, S286
Jackson, Richard, S154
Jacob, Arpitha, S181
Jacobs, Grace, S226
Jacobs, Jolien, S95
Jacobs, Nele, S84
Jaffe, Andrew E., S82, S85
Jaffee, Sara, S110
Jakobsen, Ane, S361
Jamain, Stephane, S51
James, Anthony, S229
James, Shaunequa, S322
Jang, Seon-Kyeong, S187, S260
Jansen, Philip R., S402
Janssen, Jaap, S183
Janssen, Ronald J., S101
Jauhar, Sameer, S13, S81, S395
Javadzadeh, Alborz, S221
Javitt, Daniel, S43, S331
Jeffrey Conn, P., S127, S204
Jenni, Raoul, S15, S25, S133, S236, S395
Jensen, Heidi Dorthe, S134
Jensen, Heidi, S95, S129
Jensen, Lars Thorbjørn, S383
Jensen, Maria H, S80
Jensen, Maria H., S83, S213
Jeon, Peter, S179, S179
Jeon, Youngsoon, S367
Jeong, Hyun-Ghang, S403
Jeong, Seong Hoon, S203
Jeppesen, Pia, S103, S163, S334
Jepsen, Jens Richardt Møllegaard, S226, S232, S245, S252, S300, S336
Jepsen, Jens Richardt, S111, S300
Jespersen, Cecilia Pihl, S103
Jessen, Frank, S285
Jessen, Kasper, S224, S289, S383, S387
Jessica, Graham, S162
Jewel, Charlotte, S208
Jha, Robin, S405
Jhon, Min, S411
Ji, Ellen, S172
Jiang, Wenlong, S329
Jiang, Ying, S312, S413
Jin, Huajie, S57
Jo, Anna, S411
Jo, Seongwoo, S367
Jo, Young Tak, S287
Joa, Inge, S153, S272, S284, S417
Joffe, Max, S127

Johannesen, Jason, S266
Johannessen, Jan Olav, S153, S272, S284, S417
Johansson, Anita, S211
Johansson, Daniel, S120
Johansson, Madeleine, S159, S265
Johansson, Ola, S211
Johansson, Sofia, S305
Johansson, Viktoria, S73
John, Majnu, S93
Johnsen, Erik, S95, S261, S292, S293, S324, S346, S358
Johnson-Kwochka, Annalee, S343
Jokela, Markus, S349
Jomphe, Valérie, S11, S124, S142, S149, S230
Jon, Duk-In, S161, S201, S206, S218, S264, S296, S304, S308, S367, S401, S411
Jones, Derek, S396
Jones, Hannah, S165, S270, S273, S379
Jones, Peter B., S6, S48, S379
Jones, Peter, S57, S58, S77, S131, S148
Jones-Brando, Lorraine, S150
Jongsma, Hannah, S58
Jönsson, Erik, S205
Joo, Eun-jeong, S397
Joo, Sung Woo, S287, S391
Joo, Yeon Ho, S215, S287
Joober, Ridha, S56, S148, S182, S185
Jørgensen, Hugo A, S346, S358
Jørgensen, Robert, S153, S284
Jürgen,Dukart, S340
Joutsenniemi, Kaisla, S88
Joyce, Dan, S164, S249, S375
Joyce, Eileen, S131, S148
Juárez, Alba Lubeiro, S145, S186, S205, S249, S392, S397
Jukuri, Tuomas, S88
Julio, Sanjuán, S384
Jung, Han-Yong, S257, S360
Jung, Hee Yeong, S203
Jung, Sra, S292
Jung, Wi Hoon, S386
Jung, Young-Hoon, S308
Just, David, S120, S223

Käkelä, Juha, S144
Kärgel, Christian, S128
Köhler-Forsberg, Ole, S116, S273
Kølbæk, Pernille, S342
Kübra,İpçi, S316
Kübra,Ipçi, S419
KIshimoto, Taishiro, S207
Kašpar, Ines, S257
Kadra, Giouliana, S411
Kahn, Moazzzam, S235
Kahn, René S., S51
Kahn, René, S50, S77, S82, S225, S261, S287
Kahn, Rene, S74, S247
Kaiser, Nathalie, S209
Kaiser, Stefan, S81, S284, S331
Kajanne, Risto, S88
Kakar, Rishi, S63
Kaladjian, Arthur, S381
Kalhovde, Anne Martha, S113
Kalivas, Peter W., S337
Kalman, Janos, S155
Kambeitz, Joseph, S114, S246, S283, S293
Kambeitz-Ilankovic, Lana, S147, S168, S246
Kameno, Yosuke, S277
Kaminski, Jakob, S172, S278
Kampman, Olli, S88
Kanagasundaram, Pruntha, S313
Kanazawa, Tetsu, S295
Kanba, Shigenobu, S117
Kandaswamy, Radhika, S25
Kane, John, S93, S100, S207, S216, S267, S320
Kang, Jihoon, S187
Kang, Jinsook, S240
Kang, Shi Hyun, S309
Kang, Yuree, S219
Kanji, Sarah, S190
Kao, Amy, S254
Karayiorgou, Maria, S399
Karelis, Antony, S17
Kariofillis, Daniela, S128
Karmacharya, Rakesh, S400
Karmacharya, Sarina, S184
Kasai, Kiyoto, S381
Kasparbauer, Anna, S119, S386
Katagiri, Naoyuki, S315
Kataoka, Yuzuru, S248
Kataria, Lakhan, S405
Katarina,Matić, S257
Kathuria, Annie, S400
Kato, Yasuhiko, S277
Katshu, Mohammad, S171
Katthagen, Teresa, S172, S278
Kaur, Amandeep, S129
Kaushal, Nidhi, S412
Kawasaki, Yasuhiro, S248
Kawohl, Wolfram, S331
Kebir, Oussama, S26
Keefe, Richard, S136, S159, S196, S281
Keeser, Daniel, S17, S406
Kegeles, Lawrence, S1, S2, S71
Keinänen, Jaakko, S68, S285
Kelleher, Eric, S141
Kelleher, Ian, S89, S100, S109, S147, S402
Kellendonk, Christoph, S44
Keller-Varady, Katriona, S17
Kelly, Allott, S162
Kelly, Deanna, S190
Kelly, Peter, S151
Kemmler, Georg, S135, S421
Kempton, Matthew, S307
Kennedy, James, S115, S218, S296, S399, S412
Kennedy, Sidney H., S355
Kenney, Joanne, S147
Kepinska, Adrianna, S189
Keshavan, Matcheri S., S36, S104, S121, S223, S366
Keshavan, Matcheri, S6, S14, S90, S194, S196, S236
Ketharanathan, Tharini, S224
Keysers, Christian, S89
Khadimallah, Ines, S25
Khan, Ali, S179, S179
Khan, Anzalee, S129, S159, S244, S275, S408
Khandaker, Golam, S48, S272, S325
Khanyaree, Ifrah, S246
Khelifa, Emira, S152, S163, S258, S371, S403
Kiang, Michael, S390, S415
Kidd, Martin, S364
Kidd, Sean, S31, S404
Kido, Mikio, S394
Kienzler, Anne-Kathrin, S61
Kieseppä, Tuula, S68, S80, S88, S172, S285, S345, S349
Kilian, Sanja, S261, S364, S367
Killackey, Eoin, S162, S214, S321, S334, S404
Killen, Jefferey, S120
Kilpatrick, Gavin, S339
Kim, Do-Won, S187
Kim, Edward, S130, S311
Kim, Euitae, S382, S386
Kim, Eun Jin, S344
Kim, Han-Suk, S307
Kim, Harin, S287
Kim, Hyo-Won, S232
Kim, Jae-Jin, S308
Kim, Jae-Kyeong, S177
Kim, Jae-Min, S177, S411
Kim, Jeong Hoon, S292, S293
Kim, Julia, S290, S329, S393

Kim, Jung Jin, S177
Kim, Kyung Ran, S188
Kim, Min-Woo, S308
Kim, Minah, S186, S203
Kim, Moon Doo, S161, S201, S215, S236, S296, S304, S308, S326, S411
Kim, MoonDoo, S206, S401
Kim, Pitna, S299
Kim, Se Hyun, S203
Kim, Se Joo, S188
Kim, Seoyoung, S386
Kim, Seung Hyun, S403
Kim, Soo-Jeong, S308
Kim, Sun-Min, S240
Kim, Sung-Wan, S177, S411
Kim, Sungkean, S187
Kim, Tae Yong, S257
Kim, Yong Sik, S203
Kimhy, David, S17, S233
Kimura, Haruhide, S412
Kimura, Kohei, S248
King, Daniel, S208
Kiras, Fatma, S211, S283, S316, S419
Kircher, Tilo, S276
Kirkbride, James, S58, S377
Kirschner, Matthias, S81, S174, S284
Kirstie-Kulsa, Mila, S129
Kisely, Steve, S66, S178
Kjelby, Eirik, S261
Klaasen, Nicky, S177
Klauser, Paul, S15, S25, S133, S279
Klein, Hans, S358, S360
Kleinman, Leah, S241
Kleinmann, Joel E., S82
Klim, Casimir, S43, S331
Klingberg, Stefan, S128
Klingenberg, Boris, S84
Knable, Michael, S274, S404
Knegtering, Henderikus, S177
Knevel, M.F., S256
Knox, Emma, S148
Knudsen, Christina Bruun, S336
Ko, Mi-Ae, S267
Koblan, Kenneth S., S158
Koenig, Thomas, S263, S264
Koevoets, Martijn, S225
Kohler, Christian G., S305
Kohler, Christian, S57
Koike, Shinsuke, S215, S381
Kokkinou, Michelle, S93
Kokoszka, Andrzej, S252
Kolliakou, Anna, S166
Kometer, Michael, S281
Kompus, Kristiina, S113, S261, S267, S292
Kondofersky, Ivan, S155
Kondziella, Daniel, S116
Kontis, Dimitrios, S243, S348
Kopala, Lili, S291
Koponen, Hannu, S144
Koppel, Alex, S390
Koren, Dan, S54, S198, S306, S324
Korenic, Stephanie, S20
Korpela, Hanna, S144
Kos, Claire, S177
Kosidou, Kyriaki, S67
Kothare, Hardik, S278
Kotov, Roman, S6, S40
Kott, Alan, S131, S135
Kotz, Sonja, S380
Koutsouleris, Nikolaos, S100, S114, S119, S144, S147, S155, S168, S185, S209, S246, S283, S292, S293, S316, S328, S386
Kowalchuk, Chantel, S104, S313
Kozakova, Eva, S173
Kozauer, Susan, S236, S341
Kråkvik, Bodil, S113
Krężołek, Martyna, S146, S252
Kraguljac, Nina, S41, S91
Kravitz, Richard, S4
Krebs, Marie-Odile, S26, S268, S371
Krishnan, Ranga, S281
Kristensen, Jette Kolding, S88
Kristensen, Tina Dam, S119
Krivoy, Amir, S339
Krogh, Jesper, S116
Kroken, Rune A., S95, S292
Kroken, Rune, S324, S346, S358, S417
Kruiper, Caitlyn, S314
Krynicki, Carl, S48, S131
Krystal, John, S1
Ku, Benson, S167
Kubicki, Marek, S184, S194
Kubli, Roman, S284
Kudo, Noriko, S419
Kuhnt, Katrin, S122, S228
Kulkarni, Jayashri, S214
Kumar, Devvarta, S345
Kumar, Divya, S3
Kumar, Jyothika, S171
Kumari, Veena, S89
Kundu, Prantik, S240
Kunze, Birgit, S343
Kurdyak, Paul, S108, S271
Kurzthaler, Ilse, S135
Kushner, Steven A., S231, S402
Kusztrits, Isabella, S261, S267, S302
Kwak, Yoo Bin, S294
Kwiatkowski, Molly, S233
Kwon, Jun Soo, S186, S203, S215, S294, S386
Kwon, Young Joon, S201, S218, S236, S326, S411
Kwon, Young-Joon, S206, S215, S308, S401
Kyoon An, Suk, S188
Kyung-jin, Lho, S330
kian, Alireza Armani, S344

Lähteenvuo, Markku, S88
Lässer, Marc, S140, S289
López-Carrilero, Raquel, S317, S372
López-Vílchez, Irene, S332
Lönnqvist, Jouko, S88
Løberg, Else-Marie, S292, S324, S346, S358
La Barbera, Daniele, S354
La Cascia, Caterina, S110, S354
Labad, Javier, S203, S251, S253, S303, S311, S370
Labouesse, Marie, S65
Lacerda, Acioly, S133
Lacoua, Liza, S198
Lada,Goršić, S257
Ladea, Maria, S160
Lagerberg, Trine V., S113, S362
Lahti, Adrienne, S41, S91, S120
Lahti, Jari, S26
Lally, John, S81, S87, S332, S373
Lalousis, Paris Alexandros, S280, S283
Laloyaux, Julien, S113, S261, S267
LaMantia, Anthony, S1
Lambert, Thierry, S420
Lanari, Marcello, S123
Lancaster, Tom, S30
Land, Rachel, S66
Lander, Sharon S., S202
Landgrebe, Michael, S343
Lang, Donna, S291
Lang, Undine E., S199
Langbein, Kerstin, S121, S122, S221, S228, S287
Langguth, Berthold, S342, S343
Lappin, Julia, S6
Laprevote, Vincent, S169
Larøi, Frank, S113, S261, S267
Lara, Baldwin, S162
Larnaout, Amine, S152, S163, S258, S371, S403, S423
Larsen, Tor Ketil, S417

Larsson, Henrik, S23
Laskaris, Liliana, S74
Lattanzi, Guido Maria, S393
Lau, Cindy, S108, S271
Lau, Fu Chun, S215
Laughlin, Christopher, S218
Laurens, Kristin R, S118
Laurens, Kristin R., S229
Laurens, Kristin, S55, S143
Laurikainen, Heikki, S391
Lauritsen, Marlene Briciet, S333
Laursen, Thomas Munk, S87, S88, S111
Lavie, Adi, S350
Lavoie, Suzie, S326, S378
Lawire, Stephen, S385
Lawrence, Richard, S359
Lawrie, Stephen, S131, S148, S279, S282, S340, S409
Laws, Keith, S220
Leacy, Finbarr, S379
Lebel, Catherine, S355
Leboyer, Marion, S51, S160, S166, S172
Lecardeur, Laurent, S125
Lee, Arira, S292, S293
Lee, Chewah, S176
Lee, Edwin, S97
Lee, Erin, S247
Lee, Eun, S188
Lee, Eunbyeol, S242
Lee, Ho Ming, S138, S214, S215, S372
Lee, Hoi Ching, S138, S214
Lee, Hoseon, S360
Lee, I Hui, S389
Lee, Jimmy, S123, S270, S281, S309, S324
Lee, Jong Il, S309
Lee, Ju-Yeon, S177, S411
Lee, Jung Suk, S267
Lee, Jung Sun, S219, S232, S287, S387, S391
Lee, Junhee, S186, S203
Lee, Kang-Soo, S293
Lee, Kwanghun, S161, S201, S215, S236, S264, S296, S326, S367, S411
Lee, Kyuyoung, S397
Lee, Nam Young, S203
Lee, Sang Yup, S215
Lee, Sang-Hyuk, S292, S293
Lee, Sang-Yeol, S161, S201, S215, S218, S236, S264, S296, S304, S308, S367, S411
Lee, Seon-Koo, S267
Lee, Seung-Hwan, S187, S240
Lee, Stuart, S321, S359
Lee, Su Young, S188
Lee, Sun Woo, S293
Lee, Tae Young, S186, S203, S294
Lee, Tatia M.C., S243
Lee, Won-Hye, S240
Lee, Yu Sang, S215
Legind, Christian, S80, S83, S213
Lei, Wan U, S95
Leiponen, Marita, S345
Leite, Isabel, S61
Lemvigh, Cecilie, S232
Lenartowicz, Ewelina, S65, S191
Lencz, Todd, S221, S330
Lenderking, William, S241
Lennox, Belinda, S60, S61, S254
Lepage, Martin, S56, S148, S182, S185, S197
Leroux, Elise, S396
Lesh, Tyler, S4, S126
Letendre, Elaine, S17
Leucht, Claudia, S94
Leucht, Stefan, S51, S94, S94, S115, S305, S342, S418
Leung, Patrick, S161
Levier, Axel, S310
Lewis, Cathryn, S7, S158
Lewis, Glyn, S39, S48, S53, S72, S165, S247, S270, S379
Lewis, Shon, S50, S106, S131, S148, S156
Leza, Juan Carlos, S222
Li, Anna, S188, S189
Li, Chunbo, S36, S366
Li, Fangyong, S272
Li, Gang, S103
Li, HuiJun, S36, S366
Li, Huijun, S90, S125, S218
Li, Mingli, S79, S82, S90, S394
Li, Tao, S79, S82, S90, S121, S301, S394
Li, Xianbin, S329
Li, Xiaojing, S90, S394
Li, Yinfei, S90, S394
Li, Zhi, S382
Liang, Yumin, S11
Libas, Yulia, S54
Librenjak, Dina, S257
Lichtenstein, Thorsten, S114
Liddle, Elizabeth, S171
Liddle, Peter, S170, S170, S171, S171, S180, S180, S180, S283
Lieberman, Jeffrey, S115, S218
Light, Gregory, S266
Liharska, Lora, S159
Lim, Carmen, S237
Lim, Eunsung, S201, S206, S215, S218, S304, S326, S367, S401, S411
Lim, Issel, S289
Lim, Joseph K.W., S281
Lim, Steffiany, S323
Lima, Murilo, S262
Lin, Ashleigh, S209, S334, S370
Lin, Bochao, S84
Lin, Yi-Tin, S186
Lincoln, Alisa, S137
Lincoln, Tania M., S410
Lindberg, Nina, S88
Lindemann, Lothar, S1
Linden, David EJ., S379
Linden, David, S30, S396
Lindenmayer, Jean-Pierre, S129, S175, S244, S275, S408
Lindgren, Maija, S285, S349
Lindsley, Craig, S70, S127, S204
Link, Bruce, S54
Linscott, Richard J., S229
Linscott, Richard, S150, S256, S335, S351, S399
Lisiecka, Danuta, S131, S148
Little, Simon, S416
Liu, Bangyan, S400
Liu, Chen-Chung, S186, S274, S414
Liu, Chenxing, S282, S295
Liu, Chih-Min, S186
Liu, Dennis, S73, S137, S208, S284
Liu, Fang, S190
Liu, Lu, S231
Liu, Siwei, S281
Liu, Tsz Ting, S215
Liu, Wen-hua, S244
Ljuri, Isidora, S129, S244, S275
Llacer, Blanca, S424
Llorca, Bibiana Cabrera, S222, S318
Lo, Cecilia, S298
Loas, Gwenole, S363
Lobo, Antonio, S222
Loch, Alexandre Andrade, S195
Loch, Alexandre, S376
Lockwood, Jonathan, S413
Lodge, Daniel, S101
Lodhi, Rohit, S295
Loebel, Antony, S114, S158, S239, S242, S353
Loewy, Rachel, S4, S113
Lopez, Daisy, S371
Lopez, Lorna, S53, S72
Lopez-Carrilero, Raquel, S238, S348

Lopez-Morinigo, Javier-David, S166, S374
Lorente, Esther, S200, S238, S348, S372
Lorenzo, Giorgio Di, S263, S264
Loureiro, Camila, S166, S227, S329
Lourenço, Joca Sâmia Regiane, S222
Loures Malinowski, Fernando Rocha, S161
Louzã Neto, Mario Rodrigues, S210
Louzada, Paulo, Jr., S329
Louzada-Junior, Paulo, S166, S227
Louzada-Junir, Paulo, S222
Lovko, Sandra Kocijan, S257
Lu, Wei-hong, S358
Ľubica,Forgáčová, S250
Lui, Simon S.Y., S333, S358
Lui, Su, S331
Luis, Martí-Bonmatí, S384
Luk, Sin Ki, S243
Lunden, Anya, S409
Lunding, Synve Hoffart, S362
Luo, Xue-rong, S333
Luoni, Alessia, S297
Lussier, Daphné, S11, S124, S142, S230
Luteijn, L.B., S256
Luther, Lauren, S319, S343, S350
Luykx, Jurjen, S84, S261
Lyall, Amanda, S194
Lyngstad, Siv Hege, S369
Lynn, Marquardt, S302
Lynskey, Michael, S259
Lysaker, Paul, S238, S260, S319, S350
Lystad, June U., S113
Lythgoe, David J., S81, S89

Mäki, Pirjo, S101, S164
Männynsalo, Teemu, S88
Mäntylä, Teemu, S80, S285
Månberg, Anna, S120, S223
Möller, Julian, S331
Møllegaard Jepsen, Jens Richardt, S111
Møller, Paul, S410
Mørch, Ragni H., S205, S227
Mørch, Ragni Helene, S362
Ma, Xiaohong, S90, S394
MacCabe, James, S66, S67, S81, S143, S382
MacDonald, Angus,III S140
MacDougall, Arlene, S108, S271
MacDowell, Karina, S222
MacEwan, William, S291
MacKenzie, Erin, S133
MacKenzie, Malgorzata, S190
MacQueen, Glenda, S355
Macaluso, Matthew, S63
Machin, Matt, S106
Macht, Deliah, S250
Maciukiewicz, Malgorzata, S218
Mackenzie, Lynn, S252
Mackinley, Michael, S179, S179
Mackinnon, Andrew, S378
Maclaurin, Sarah, S361
Maddalena, Giancarlo, S85
Maddock, Richard, S126
Madonia, Jennifer, S176
Magata, Yasuhiro, S277
Maguire, Áine, S357
Maguire, Gerald, S63
Mahfouda, Simone, S334
Mahmoudi, Ebrahim, S398
Maihofer, Adam, S82
Maj, Mario, S196
Makowski, Carolina, S182, S185
Makris, Nikos, S184
Makuch, Mateusz, S61
Malchow, Berend, S17, S152, S343
Malhotra, Anil, S93, S176, S221, S330, S422
Malla, Ashok, S56, S148, S182, S185, S371
Mallar Chakravarty, M., S121, S290
Mallett, Susan, S23
Mallikarjun, Pavan, S280, S283, S328
Mamo, David, S393
Mancilla, Felipe, S269
Mandl, Rene C. W., S109, S289
Mandl, Rene, S83
Mane, Anna, S237
Manoliu, Andrei, S81, S284
Manseau, Marc, S167, S201
Mansueto, Giovanni, S76
Mantere, Outi, S68, S285
Mantyla, Teemu, S172
Mao, Xiangling, S2
Maples, Natalie, S31, S31
Mar, Wanna, S393
Marcó-García, Silvia, S208
Marcelino, Loureiro Camila, S222
Marcelis, Machteld, S67, S117
Marcus, Chiu Yu-Lung, S427
Marder, Stephen, S63
Marek, Kenneth, S176
Marenco, Stefano, S85
Mareschal, Isabelle, S249
Margolis, Russell, S289
Mario, Alvarez-Jimenez, S162
Markulev, Connie, S99, S116, S132, S228, S326, S378
Markus, Schwab, S380
Marmo, Federica, S313
Marquardt, Lynn, S261
Marques, Tiago Reis, S77, S92, S98, S246, S291
Marquet, Pierre, S230, S254
Marroun, Hanan El, S402
Marshall, Louise, S416
Marshall, Max, S156
Marshe, Victoria, S190
Marsman, Jan Bernard, S177
Marta, Del-Ben Cristina, S222, S227
Marteleur, Alexander, S407
Marti-Bonmati, Luis, S276
Martin, Brice, S47
Martin, Graham, S379
Martin, Nicholas, S12
Martin-Iverson, Mat, S365
Martinez-Aran, Anabel, S123
Martins, Diana, S262
Martins, Thaís, S146, S262
Martins, Thais, S349, S425
Martins-De-Souza, Daniel, S15, S22, S41, S51, S53, S297
Marvulli, Daniela, S116
Marwaha, Steven, S48
Marzi, Sarah, S25
Marzullo, Giovanni, S193
Masana, Guillem, S332
Masellis, Rita, S100, S193
Mashhadi, Farzaneh, S368
Massie, Ann, S20
Massons, Carmina, S370, S410, S423
Massuda, Raffael, S288
Massuda, Raphael, S2
Mates, Sharon, S236, S341
Mateu, Ainoa, S5
Mathalon, Daniel H., S78, S231
Mathalon, Daniel, S62, S79, S102, S266, S396, S422
Mathews, Maju, S130, S311
Mathys, Christoph, S278, S281
Matrone, Marta, S312
Matsuda, Tokio, S394
Matsumoto, Hiroshi, S315
Matsuoka, Jun, S381
Mattaroccia, Silvia, S95
Mattila, Ismo, S69
Mawn, Lauren, S321
Mayer, Jutta, S250
Maynard, Thomas, S2

Maziade, Michel, S10, S11, S124, S142, S149, S230
Mazloomzadeh, Saeedeh, S344
McAlonan, Grainne, S183
McCLure, Margaret, S240
McCarley, Robert W., S117, S223, S366
McCarley, Robert, S90
McCarthy, Timothy, S176
McCartney, Lara, S66
McCrory, Eamon, S331
McCullumsmith, Robert E., S299
McCullumsmith, Robert, S19, S19, S52
McCutcheon, Rob, S225
McDaid, David, S59
McDonald, Mhairi, S409
McDonnell, David, S312, S413
McEwen, Sarah, S18, S79, S396
McFarlane, William, S54
McGavin, Jacob, S153
McGlashan, Thomas H., S78, S102, S231
McGlashan, Thomas, S62, S79, S266, S396
McGorry, Patrick D, S74
McGorry, Patrick D., S4, S105, S116, S124, S132, S162, S228, S270, S326
McGorry, Patrick, S99, S130, S162, S214, S376, S378
McGrath, John, S87, S167, S270
McGuire, Philip, S27, S28, S51, S56, S74, S81, S81, S83, S118, S154, S162, S174, S307, S310, S323, S328, S374, S383, S393, S413
McGurk, Susan, S244, S404
McIlwaine, Sarah, S56
McIntyre, Gina, S295
McKenna, Peter J., S174
McMillan, Greg, S216
McMullen, Katrina, S89
McNulty, James, S150
McQueen, Grant, S81, S382
Mcglashan, Thomas, S39
Meador-Woodruff, James, S52, S298, S299, S400
Mechelli, Andrea, S92, S331, S374
Medalia, Alice, S196
Medeiros, Heydrich, S262
Meecham, Sabrina, S283
Meehan, Stine R., S241
Meehan, Stine, S94, S133
Mehdyoun, Nicole, S63
Mehl, Stephanie, S410
Mehmet Buğrahan, Gürcan, S316
Mehtälä, Tuukka, S349
Mehta, Mitul A., S118
Mehta, Mitul, S83
Mehtala, Tuukka, S32
Meier, Madeline, S12
Meisenzahl, Eva, S144, S147, S209, S328
Meisner, Christoph, S128
Melau, Marianne, S95, S106, S129
Melle, Ingrid, S113, S153, S205, S227, S272, S362, S369, S410
Meller, Jarek, S52, S299
Mellgren, Zophia, S211
Meltzer, Herbert, S115, S218
Mena, Cristian, S202, S364
Mendes, Diego, S146, S262, S349, S425
Mendyk, Nina, S198
Menezes, Paulo Rossi, S329
Menezes, Paulo, S166
Meng, Ya-jing, S90, S394
Menne-Lothmann, Claudia, S84
Meola, Annasara, S421
Mercedes Perez-Rodriguez, M., S240
Mercer, Jeremy, S284
Mercuel, Alain, S167
Merette, Chantal, S149
Merlino, Sergio, S162
Merriman, Tony, S399
Mesa, Nora, S251
Mesholam-Gately, Raquelle, S194
Meshul, Charles K, S20
Mesman, Esther, S225
Metzak, Paul, S388
Metzler, Sibylle, S230, S336
Meunier-Cussac, Sophie, S125
Meyer, Denny, S139, S302
Meyer, Eric, S39
Meyer, Monet, S4
Meyer, Urs, S2, S8, S65, S234
Meyer-Kalos, Piper, S216, S320, S322
Meyer-Lindenberg, Andreas, S51
Meyhöfer, Inga, S119, S386
Mezquida, Gisela, S222, S318, S351
Michael Deans, PJ, S105
Michel, Chantal, S109, S228, S407
Mielnik, Catharine, S19
Miettunen, Jouko, S144, S168, S270, S415
Miguel, Euripedes C., S286
Mikanmaa, Emmi, S279, S282, S385
Mike, Luke, S121
Mikolajacks, Gladys, S17
Milak, Matthew, S2
Mill, Jonathan, S25
Miller, Brian, S242
Millman, Zachary, S43
Milovac, Željko, S257
Mimica, Nino, S257
Mimura, Masaru, S329
Minah, Kim, S330
Miñano, Maria José, S303
Mingoia, Gianluca, S169, S277, S380
Minichino, Amedeo, S162
Minyaycheva, Maria, S213
Miselli, Manuela, S426
Misheneva, Varvara, S150
Mitelman, Serge, S268
Mitjans, Marina, S85
Mitra, Sayantanava, S136
Mittal, Vijay, S42
Mizrahi, Romina, S43, S309, S390
Mizuiri, Danielle, S278
Mizuno, Masafumi, S315
Modinos, Gemma, S89
Moehle, Mark, S204
Moeller, Scott, S240
Moffitt, Terrie, S25, S97, S110
Mofsen, Rick, S340
Moghaddam, Bita, S45
Mohn, Christine, S141, S245, S401
Moilanen, Jani, S144, S415
Moisés, Ana Carolina, S226
Mok, Pearl L.H., S167
Molden, Espen, S205
Molero, Patricio, S352
Molina, Vicente, S186
Mollon, Josephine, S39, S77, S247
Molteni, Raffaella, S202
Mondelli, Valeria, S118, S118, S166, S246
Monenschein, Holger, S339
Mongan, David, S317
Monreal, José Antonio, S203, S251, S253, S303, S311
Monserrat, Clara, S410
Montalvo, Itziar, S253, S303, S311
Moore, Cynthia, S20
Moore, Tyler M., S305
Moore, Tyler, S57
Moradi, Hawar, S263, S265
Moreira, Fabricio, S21
Moreno, Daniel, S237
Moreno, Marcos, S174
Moreno-Kustner, Berta, S151
Moretti, Patrizia, S363
Morgan, Craig, S6, S7, S58, S58, S59, S77, S110, S156, S158, S166, S166, S307, S354

Morgan, Kevin, S77, S368
Morgan, Vera, S365
Morishita, Hirofumi, S2
Moritz, Rossner, S380
Morkved, Nina, S324
Morley, Thomas, S176
Morris, Brian, S234
Morris, Crystal, S216
Morris, Derek, S325
Morris, Peter, S171
Morrison, Paul, S27
Morrison, Sinead, S86
Mors, Ole, S111, S134, S163, S245, S252, S300, S336, S341, S342, S350
Mortensen, Erik Lykke, S252
Mortensen, Preben Bo, S59, S87
Mortensen, Preben, S85, S167
Mossaheb, Nilufar, S99, S116, S132, S228, S378
Mothersill, David, S141
Mouchlianitis, Elias, S92, S383
Mougin, Olivier, S180
Moya, Jorge, S330
Mucci, Armida, S50, S196, S263, S264
Muckenhuber-Sternbauer, Susanna, S292
Mueller, Daniel, S115, S190, S218, S239, S412
Mueller, Nikola, S155
Mueller, Toni, S298
Mueser, Kim, S216, S320
Mulert, Christoph, S9
Mulholland, Ciaran, S317
Mullier, Emeline, S279
Mulligan, Victoria, S339
Mulsant, Benoit, S176, S393
Munafò, Marcus, S165
Munafo, Marcus, S270
Munday, Jennifer, S97, S366
Munk-Olsen, Trine, S88
Muñoz, Daniel, S113
Muralidharan, Anjana, S40
Murphy, Timothy, S288
Murphy, Veronica, S103
Murray, Graham, S101, S144, S164
Murray, Robin M., S166
Murray, Robin, S6, S7, S58, S59, S75, S77, S84, S87, S92, S98, S110, S118, S123, S156, S158, S166, S189, S246, S258, S328, S332, S354, S364, S373
Muscat, Whitney, S267
Mussa, Amjad, S324
Mustafé, Gustavo, S146, S262, S349, S425
Mustafa, Sally, S56
Myers, Neely, S178
Myin-Germeys, Inez, S307
Myles, Hannah, S284
Myles, Nicholas, S284

Nagarajan, Srikantan, S278
Nagele, Robert, S328
Nagels, Arne, S195, S276
Najdenovska, Elena, S395
Nakaizumi, Kyoko, S277
Nakajima, Shinichiro, S290, S329, S393
Nakamura, Mihoko, S394
Nakatani, Atsushi, S412
Nam, Beomboo, S308
Nam, Beomwoo, S161, S206, S215, S264, S296, S326, S367, S401
Napolitano, Isabel Cristina, S210
Napolitano, Isabel, S262
Narayanaswamy, Janardhanan C., S345
Nascimento, Juliana, S15
Natesan, Sridhar, S65, S191
Naveen Kumar, C., S136
Nawani, Hema, S345
Nefzi, Rahma, S152, S163, S258, S371, S403, S423
Neill, Erica, S139, S252, S355
Neill, Joanna, S128, S312
Nelson, Barnaby, S4, S46, S74, S99, S105, S116, S124, S132, S162, S228, S326, S370, S378, S410
Nemoto, Kiyotaka, S248
Nemoto, Takahiro, S315
Nenad,Jakšić, S257
Nenadic, Igor, S169, S178, S287, S380
Nentwich, Beate, S128
Netser, Roni, S10, S401
Neuchterlein, Keith, S63
Neumann, Suzanne, S351
Nevado, Angel, S186
Neves, Gilda, S416
New, Antonia, S240
Newcomer, John W., S114
Newman, Ellie, S237
Ng, Boon Tat, S309
Ni, Hua, S358
Ni, Peiyan, S121, S301
Nichols, Heathman, S195, S196, S421
Nicholson, Timothy, S328
Nick, Medford, S173
Nicolas, Poirel, S390
Nielsen, Anita Tønder, S88
Nielsen, Mai, S134
Nielsen, Mette Ø., S289
Nielsen, Mette Ødegaard, S224, S383, S424
Nielsen, Mette Odegård, S387
Nielsen, Mette, S80, S213, S422
Nielsen, René Ernst, S333
Nielssen, Olav, S214
Nieman, Dorien H., S132, S228
Nieman, Dorien, S99, S116, S378
Niemi-Pynttäri, Jussi, S88
Niendam, Tara, S3, S4
Nieto, Lourdes, S370, S410, S423
Nievergelt, Caroline, S82
Nijman, Henk, S24
Nijman, Saskia, S419
Nikita, Setiaman, S225
Niklas, Schülert, S380
Nikolina Tunjić,Vukadinović, S257
Nilsson, Hanna, S120
Nilsson, Peter, S120, S223
Nilsson, Sandra Feodor, S111
Nimgaonkar, Vishwajit, S63
Niolu, Cinzia, S263, S264
Nishikawa, Yumiko, S394
Nissen, Wiebke, S380
Niswender, Colleen, S127
Niznikiewicz, Margaret A., S117
Niznikiewicz, Margaret, S36, S90, S223, S266, S366
Noethen, Markus M., S287
Noguchi, Kyo, S394
Norbeck, Oscar, S120
Nordentoft, Merete, S50, S85, S95, S99, S99, S106, S110, S111, S116, S116, S119, S129, S132, S134, S163, S213, S228, S245, S252, S273, S300, S300, S336, S365, S378
Nordström, Tanja, S144
Nordstroem, Anna-Lena, S371
Norman, Ross, S108, S207, S271
Norton, Joanna, S359
Nosaka, Tadashi, S363
Nossel, Ilana, S107
Noto, Cristiano, S96, S153, S154, S161, S184, S255, S266, S375, S375
Noto, Mariane, S96
Notter, Tina, S8
Nour, Matthew, S81, S385
Noviar, Galoeh Adrian, S157
Nuamah, Isaac, S130
Nudel, Ron, S85

Nuechterlein, Keith H., S136
Nuechterlein, Keith, S16, S18
Nummenmaa, Lauri, S47, S77
Nunez de Arco, Rossana Kathenca Peredo, S149
Nunez, Daniel, S96
Nurmi, Erika, S218
Nye, Kathleen, S3

O' Hanlon, Erik, S147
O'Connor, Kieron, S346
O'Daly, Owen, S174, S280, S291
O'Donnell, Colin, S130
O'Donnell, Lauren J., S194
O'Donnell, Maryanne, S73
O'Donnell, Patricio, S2
O'Donoghue, Brian, S162, S270, S376
O'Donovan, Michael C., S273, S379
O'Donovan, Michael, S7, S165
O'Donovan, Sinead, S19
O'Gorman, Aoife, S53, S72
O'Hanlon, Erik, S89, S181, S402
O'Hare, Kirstie, S399
O'Leary, Conor, S37
O'Neill, Aisling, S154, S384
O'Reilly, Nicole, S347
Ochoa, Susana, S151, S208, S210, S212, S238, S317, S348, S370, S372, S410
Ochoa, Susana, S151, S208, S210, S212, S238, S317, S348, S370, S372, S410
Odejayi, Gbolahan, S418
Oduola, Sherifat, S58
Ofray, Dayshalis, S142
Oh, Hyeonju, S260
Oh, Ji-Hye, S242
Ohi, Kazutaka, S248, S419
Ohmann, Christian, S343
Ohrablo, Peter, S250
Øie, Merete, S356, S410
Ojeda, Natalia, S347
Ojeil, Najate, S2
Okada, Naohiro, S381
Okubo, Hiroaki, S248
Olasz, Irina, S251
Olesen, Julie Aamand, S365
Olga, Soprano-Ros, S384
Oliveira, Graça, S262
Oliveira, Hianna, S154
Oliver, Dominic, S162, S323
Oliver, Lindsay, S422
Olivier, Riaan, S367
Oller, Silvia, S237
Olsson, Anna-Karin, S159, S265
Olsson-Tall, Maivor, S159, S263, S265
Olvet, Doreen, S79
Ongur, Dost, S16
Onitsuka, Toshiaki, S117
Onn, Roy, S339
Ophoff, Roel, S109
Or, Chi Fai, S215
Oranje, Bob, S95, S226, S232, S314
Orens, Sandrine, S420
Oresic, Matej, S68, S69
Oribe, Naoya, S117
Origala, Ajay, S158
Orlov, Natasza, S174, S280
Orlovska, Sonja, S116
Orlovskaya, Diana, S41
Orsini, Nicola, S67, S94
Ortet, Generós, S330
Ortiz, Bruno Bertolucci, S184, S255
Ortiz, Bruno, S96
Ortiz-Gil, Jordi, S145
Ortuño, Felipe, S352
Ortuño, Marta, S352
Ortuño, Noèlia, S423
Os, Jim van, S334
Osborn, Juston, S42
Ose, Claudia, S128
Østefjells, Tiril, S113
Østergaard, Søren D., S342
Ostergaard, Soren Dinesen, S341
Ota, Vanessa, S96, S184
Ouchi, Yasuomi, S277
Overbeek, Greg, S120
Overholser, James, S374
Owe-Larsson, Björn, S120
Owen, Michael J., S273, S379
Owen, Michael, S12, S13, S86, S165
Oyebode, Femi, S280
Ozog, Veronica, S275

Pélaez, Trinidad, S348
Pérez-Solà, Víctor, S237
Pöhö, Päivi, S69
Paccalet, Thomas, S11, S124, S142, S149, S230
Pachas, Gladys, S361
Padberg, Frank, S355
Pagsberg, Anne Katrine, S226, S232, S333
Painter, Michelle, S57
Paker, Benedicto, S244
Paladini, Maria Serena, S202
Paladini, Vittoria, S116
Palaniyappan, Lena, S79, S82, S90, S102, S108, S170, S170, S171, S171, S179, S179, S180, S180, S180, S187, S194, S199, S207, S221, S229, S271, S327, S373, S394
Palao, Diego, S203, S251, S253, S303, S311
Palm, Ulrich, S355
Palmer, Lisa, S247
Palotie, Aarno, S88
Paloyelis, Yannis, S383
Pàmias, Montserrat, S251
Pan Yuen, Hok, S162
Pan, Pedro, S112, S164, S286
Panizzutti, Bruna, S22
Paños, Montse, S253
Pantazopoulos, Charalampos, S235
Pantazopoulos, Harry, S16
Pantelis, Christos, S74, S91, S176, S209, S282, S295
Paola, Presečki, S257
Papaleo, Francesco, S37, S38
Papalino, Marco, S116
Papazova, Irina, S342, S355
Papin, Caroline, S176
Papiol, Sergi, S17, S155
Papp, Mariusz, S202
Papsuev, Oleg, S213
Parak, Mohan, S244
Paramo, Mario, S398
Pardeller, Silvia, S421
Pardeller, Sylvia, S135
Pardo, Marta, S212, S229
Parellada, Mara, S222, S318
Pariante, Carmine M., S118
Pariante, Carmine, S48, S97, S98, S118, S131, S166
Pariente, Jose, S181, S183
Park, Cheol, S411
Park, Jin Young, S188
Park, Joo Eon, S257, S360
Park, Kee Jeong, S232
Park, Kiho, S240
Park, Kyung Tae, S203
Park, Seon-Cheol, S240, S257, S360
Park, Sohee, S29, S46, S47, S77, S195, S196, S421
Park, Sung-Yong, S206, S215, S218, S236, S264, S304, S308, S367, S401
Park, Woon Ju, S29
Parker, Benedicto, S129
Parker, Stephen, S237
Parker, Theresa, S256
Parlar, Melissa, S149
Parnas, Josef, S198, S306
Parola, Alberto, S169, S319
Parrish, Emma, S404

Pasternak, Ofer, S8, S9, S194
Patel, Anita, S87
Patel, Krisna, S92
Patel, Raihaan, S290
Patel, Rashmi, S154, S310
Paterno, Danielle, S10
Patterson, Thomas, S204
Pau, Soldevila-Matias, S384
Paul Amminger, G, S105
Paul Amminger, G., S99, S132, S177, S228
Paulo, Louzada-Junior, S222
Paunio, Tiina, S88
Pauselli, Luca, S167, S178, S201, S259, S409
Pavelková, Lucia, S250
Peña, Javier, S347
Pearlson, Godfrey D., S121
Pearlson, Godfrey, S6, S14
Pedersen, Carsten Bøcker, S59
Pedersen, Carsten, S87, S167
Pedersen, Emilie, S273
Pedrini, Mariana, S2
Pegoraro, Luis Fernando, S146
Pegoraro, Luiz Fernando, S349
Peh, Oon Him, S123, S270, S324
Peitl, Milena, S369
Peitl, Vjekoslav, S369
Pekar, James, S289
Pelayo-Teran, Jose Maria, S373, S374
Pelham, Tanisha, S125
Peña, Javier, S347
Penades, Rafael, S332
Penn, David, S208, S237, S320
Penney, Danielle, S148
Pennuto, Maria, S193
Pennycook, Gordon, S147
Penttilä, Matti, S144
Penzel, Nora, S293
Pepper, Fiona, S13
Peregrino, Antônio, S262
Pereira, Avril, S224
Perez, Jesus, S57
Perez, Maria Isabel Ahuir, S303
Perez, Stephanie, S101
Perez-Iglesias, Rocio, S373
Perfect, Leo, S105
Pergola, Giulio, S100, S116, S193
Perkins, Diana O., S78, S102, S231
Perkins, Diana, S39, S62, S79, S266, S396, S422
Perquier, Florence, S167
Perry, Benjamin, S48
Persson, Mats, S120
Pery, Shani, S65
Peter, Durning, S235
Peters, Benjamin, S250, S387
Peters, Sarah, S220
Petersen, Liselotte, S273
Petersen, Lone, S163
Petra Gronholm, S55
Petros, Natalia, S374
Petrušová, Veronika, S250
Peyre, Hugo, S166
Phahladira, Lebogang, S261, S364, S367
Philip, Mariamma, S181
Philip, Noah, S244
Piccin, Sara, S147
Pichker, Theresia, S135
Pierce, Katherine, S3
Pieters, Lydia, S325
Pignon, Baptiste, S155, S166
Pijnenborg, Marieke (Gerdina),
Pijnenborg, Marieke, S238, S419
Pikalov, Andrei, S114, S239, S242
Pilar, Javier Gómez, S397
Pilecka, Izabela, S77
Pillinger, Toby, S68, S225, S330
Pilobello, Kanoelani, S235
Pilton, Marie, S321
Pinheiro, Ana, S380
Pinkham, Amy, S208, S320, S358, S360
Pinner, Anita, S298
Pinnock, Farena, S149, S368
Pionke, Renata, S252
Piontkewitz, Yael, S65, S191
Pitt, Michael, S156
Pitta, José Cássio, S375
Plessen, Kerstin J., S336
Plessen, Kerstin, S111, S245, S252, S300
Plessis, Stefan du, S364
Pletnikov, Mikhail, S150
Plewnia, Christian, S355
Plitman, Eric, S290, S329, S393
Pluijm, Marieke van der, S391
Plumley, Hyekyung, S70
Podda, Giovanni, S128
Pogarell, Oliver, S393
Poh, Joann, S281
Polacek, Romana, S65
Polari, Andrea, S378
Poletti, Michele, S46
Pollak, Thomas, S60, S72, S74, S189, S328
Pollock, Bruce, S393
Polo-Velasco, Javier, S237
Pomarol-Clotet, Edith, S145, S174
Pomper, Martin, S8
Poncelet, Patrick, S47
Ponnampalam, Meneshka, S318
Ponsford, Jennie, S260, S359
Porcelli, Annamaria, S85
Porffy, Lilla, S249
Porfy, Lilla, S92
Posselt, Christine Merrild, S365
Pot-Kolder, Roos, S344
Potkin, Steven G., S115, S312
Potvin, Stéphane, S346
Poupon, Cyril, S172
Pour, Benedek, S192
Pousa, ESther, S348
Pousa, Esther, S210, S238, S317, S370, S372, S410
Powell, Susan, S233
Pradhan, Subechhya, S20
Prasad, Konasale, S63
Prats, Claudia, S174
Pratt, Judith, S234
Prayosha Villa, L., S305
Preisig, Martin, S254
Prener, Christopher, S137
Preskorn, Sheldon, S63
Price, Jack, S105
Pries, Lotta-Katrin, S84, S160
Prikken, Merel, S77, S95
Prinzio, Patricia Di, S365
Pritchard, Megan, S72
Pross, Benjamin, S393
Provenzani, Umberto, S383
Psicotici, Gruppo Regionale Esordi, S426
Puchalski, Severin, S363
Puhl, Matthew, S2
Puig-Navarro, Olga, S123, S229
Pujol, Nuria, S237
Pulasky, Beth, S248
Pulkkinen, Ulla, S349
Pungor, Katalin, S130, S311
Punzi, Giovanna, S85
Purdon, Scot, S295
Purr, Franziska, S136
Purves-Tyson, Tertia, S64, S64

Qi, Zhenghan, S104
Qian, Yun, S333
Qian, Zhenying, S223
Qiyong, Gong, S331
Quarto, Tiziana, S116
Quattrocchi, Graziella, S416
Quattrone, Diego, S7, S59, S75, S110, S158, S246, S258, S354
Qubad, Mishal, S387
Quide, Yann, S347
Quigley, Harriet, S59

Quinn, Anne Marie, S130
Quintana, Daniel, S362

Räikkönen, Katri, S26
Rösler, Lara, S250, S387
Rössler, Wulf, S376
Rødevand, Linn, S205, S362
Røssberg, Jan Ivar, S113
Raballo, Andrea, S46
Racette, Elise, S362
Rachael, Spooner, S105
Rachel, Jenkins, S235
Radaideh, Ali, S180, S180
Radhakrishnan, Rajiv, S160
Radin, Duje, S29
Radin-Gilboa, Shulamit, S54
Radnovich, Alexander, S63
Radua, Joaquim, S221, S327
Rae, Charlotte, S173
Raffard, Stéphane, S359
Ragazzi, Taciana, S166
Ragland, J. Daniel, S4
Rahel, Flückiger, S407
Raij, Tuukka, S80, S172
Raitakari, Olli, S272
Rajarajan, Prashanth, S82
Rakhmanova, Valentina, S41
Ramaekers, Jan, S182
Ramella-Cravaro, Valentina, S383
Ramikie, Teniel, S235
Rampino, Antonio, S100, S116, S193
Ramsay, Hugh, S272
Ramsey, Amy, S19
Rand, David, S147
Randers, Lasse, S95
Ranganathan, Mohini, S27
Rannikko, Irina, S144
Rao, Naren, S181, S309
Rapisarda, Attilio, S123, S270, S324
Rapoport, Judith, S34
Rapp, Alexander, S136
Rask, Charlotte Ulrikka, S103, S334
Ratheesh, Aswin, S105, S162, S270, S376
Rathi, Yogesh, S194
Ratner, Yael, S198
Rau, Carina, S95
Rauchmann, Boris, S17
Rauschenberg, Christian, S160, S219
Rautio, Nina, S144
Ray, Jim, S339
Razzino, Eugenio, S204, S312, S414
Reay, William, S296
Recasens, Marc, S279, S282, S385
Recio, Sandra, S226
Reckziegel, Ramiro, S288
Redondo, Marina, S125, S181, S229
Reese, Elaine, S335
Reeves, Gloria, S43
Refisch, Alexander, S152
Refsum, Helge, S122
Rehen, Stevens K., S15
Reichard, Holly, S339
Reichenbach, Juergen, S121, S221
Reichenberg, Abraham, S39, S77
Reichenberg, Avi, S40, S98, S165, S247, S396
Reid, Meredith, S41, S120
Reid, Vincent, S301
Reif, Andreas, S30, S250, S387
Reilly, Thomas, S56
Reinares, Maria, S5
Reinhart, Robert, S303
Reininghaus, Ulrich, S6, S6, S7, S158, S160, S219, S307
Reixach, Elisenda, S208
Remington, Gary, S104, S115, S267, S290, S306, S309, S309, S313, S329, S354, S393, S413, S415
Rempfer, Melisa, S362
Remus, Isadora, S288
René,Müri, S184
Reneman, Liesbeth, S391
Reniers, Renate, S98, S209, S280, S328, S334, S370
Reponen, Elina J., S205
Reponen, Elina Johanna, S362
Reser, Maree, S303, S406
Ressler, Kerry, S235
Revu, Omkar, S150
Reymond, Annina R., S239
Reynolds, Gavin, S329
Reznik, Noa, S306
Rhebergen, Didi, S157
Ribeiro, Wagner, S55
Rice, Simon, S4
Richard, Jean-Romain, S155
Richards, Alex, S7
Richetto, Juliet, S65, S127
Riecher-Rössler, Anita, S99, S116, S132, S199, S228, S378
Riehle, Marcel, S410
Rietschel, Marcella, S343
Rigoux, Lionel, S314
Rikandi, Eva, S80, S172, S285
Rimvall, Martin K., S103, S334
Ripka, Stephan, S287
Ripoll, Oleguer Plana, S87
Ris, Laurence, S20
Ritsner, Michael S., S198
Riva, Marco A., S297
Riva, Marco Andrea, S202
Rivolta, Davide, S340
Rizvi, Asra, S100, S267
Rizzo, Gaia, S83
Roach, Brian, S266
Roalf, David, S57
Robbins, Trevor W, S360
Roberto Bakker, P., S157
Roberts, Ruth, S118
Roberts, Susanna, S25
Robertson, James, S194
Robertson, Sharman, S216
Robinson, Delbert, S330
Robinson, Joshua F., S85
Robson, Philip, S27
Robson, Siân, S171
Roché, Matthew, S401
Rocha, Deyvis, S161, S184, S255, S375
Roche, Ayesha, S370
Roder, Volker, S239
Rodrigues, Naielly, S21
Rodriguez, Carolyn, S2
Rodriguez, Katrina, S190
Rodriguez, Vicente Molina, S205, S249, S397
Rodriguez, Victoria, S59, S246, S354
Rodriguez-Raecke, Rea, S277
Roeh, Astrid, S342
Roepstorff, Andreas, S350
Rogdaki, Maria, S81, S291, S395
Rohde, Luis Augusto, S286
Roine, Timo, S279, S395
Roiser, Jonathan, S43
Rokita, Karolina, S325
Rolls, Asya, S47
Romain, Ahmed Jérôme, S17
Romano, Raffaella, S100, S116
Romanowska, Sylvia, S355
Romero Jiménez, Rodrigo, S259
Romm, Kristin L., S113
Roos, Johannes, S399
Rosa, Ayesa-Arriola, S184
Rosa, Eduarda, S22
Rosa,Ayesa Arriola, S259
Rosana, Shuhama, S166, S222
Rosell, Daniel, S240
Rosen, Marlene, S114, S144, S147, S209
Rosen, Raymond, S139
Rosenbrock, Holger, S380
Rosengard, Rachel, S56, S185
Rosenheck, Robert, S320

Rosenthal, Adi, S4
Ross, Christopher, S289
Rossell, Susan, S78, S137, S138, S139, S143, S246, S252, S260, S279, S302, S303, S321, S355, S359, S406
Rossetti, Andrea Carlo, S202
Rossi, Menezes Paulo, S222, S227
Rossler, Wulf, S195
Rossolatos, David, S295
Rostamzadeh, Ayda, S285
Rostrup, Egill, S80, S83, S119, S224, S289, S383, S387
Rot, Marije aan het, S238, S260
Rothbaum, Maya, S198
Rothmond, Debora, S64
Rothshield, Lily, S198
Rothwell, John, S280
Roussos, Panos, S188
Rovó, Zita, S395
Rovira, Esther Lorente, S317
Rowe, Monique, S218
Rowland, Emma, S374
Rowland, Laura, S18, S20, S186
Rowland, Toby, S48
Roy, Martin, S254
Rubia, Katya, S174
Rubinstein, Katya, S165
Rubio, Jose, S93, S207
Ruderfer, Douglas, S34
Ruef, Anne, S114, S119, S155, S168, S185, S246, S292, S316
Ruggero, Camilo, S40
Ruhrmann, Stephan, S209, S285, S328
Ruiz, Ada, S370, S410
Ruiz, Isabel, S238, S348, S370, S372
Ruiz-Delgado, Isabel, S317
Rujescu, Dan, S51, S85
Rund, Bjørn Rishovd, S253
Rund, Bjorn, S245, S401
Ruparel, Kosha, S57
Ruppa, Markku, S345
Rusjan, Pablo, S390
Russell, Anthony, S178
Russell, David, S176
Russo, Debra A, S57
Russo, Manuela, S98
Russon, Penni, S4
Ruth, Zhou De-Hui, S427
Rutigliano, Grazia, S56, S162, S383
Rutten, Bart P. F., S166
Rutten, Bart, S53, S72, S82, S84
Ruth, S259
Richetto, S49
Ryan, Alex, S61
Rydkjaer, Jacob, S226, S232

Sönmez, Nasrettin, S113
Søndergaard, Anne, S245, S300, S336
Saarela, Maria, S68
Saarinen, Maiju, S272
Sabesan, Priyadharshini, S221, S327
Sabhwerwal, Sophie, S53, S72
Sadeh, Elham, S216
Saha, Sukanta, S237
Sahakian, Barbara J., S275
Saia-Cereda, Veronica M., S15
Sailas, Eila, S32
Saillard, Jelena, S236, S341
Saini, Suriati, S295
Saiz-Martinez, Pilar, S119, S222, S420
Sakakibara, Eisuke, S381
Sakuma, Kei, S315
Sakurada, Hanako, S381
Salas, Miriam, S238
Salcedo, Tatiana Oviedo, S261
Salgado, Joao, S22
Salgado-Pineda, Pilar, S174
Salibi, Noah, S120
Salisbury, Dean, S288, S390
Salokangas, Raimo K.R., S272
Salokangas, Raimo, S144, S147, S209, S328, S391
Saltó, Carme, S113
Salum, Giovanni, S164
Salvador, Teresa, S237
Salyers, Michelle, S343
Sambataro, Fabio, S83
Sami, Musa, S259
Samuel, Sarrazin, S172
SanDiego, Christine, S176
Sanchez Blanco, Lucía , S259
Sanchez, Stella, S286
Sanchez-Cabezudo, Dolores, S410
Sanchez-Gistau, Vanessa, S303
Sanchez-Gutierrez, Teresa, S255
Sanchez-gistau, vanessa, S253
Sand, Michael, S139, S248, S353, S425
Sanders, Caroline, S106
Sandin, Sven, S77
Sandra, Iglesias, S72
Sandvik, Leiv, S113
Sanfelici, Rachele, S185, S246
Sanja,Devčić, S257
Sanjuan, Julio, S157, S200, S276, S424
Santesteban-Echarri, Olga, S151, S231
Sanz-Fuentenebro, Francisco Javier, S205
Saperia, Sarah, S306
Saponaro, Alessio, S426
Sarkar, Abhirup, S328
Sarkar, Nilanjan, S77, S195, S196, S421
Sarkkinen, Katja, S345
Sarno, Elaine Di, S262
Sarnyai, Zoltan, S326
Sarró, Salvador, S145
Sarris, Jerome, S405
Sartori, Giuseppe, S393
Sartorio, Crocettarachele, S354
Sartory, Gudrun, S128
Sasabayashi, Daiki, S394
Saskia,Jünger, S285
Sass, Louis, S46
Satlin, Andrew, S236, S341
Sato, Hideyo, S20
Sato, João Ricardo, S184
Satomura, Yoshihiro, S381
Satterthwaite, Theodore, S57
Sauer, Andreas, S340
Sauer, Heinrich, S287
Sauve, Genevieve, S148
Savadijev, Peter, S184
Savadjiev, Peter, S194
Savage, Christina, S264, S353
Savill, Mark, S4
Savulich, George, S351
Sawa, Akira, S8
Sawada, Ken, S290
Sbrini, Giulia, S202
Scarborough, Joseph, S127
Scardoelli, Maria, S262
Scarpazza, Cristina, S92, S331, S393
Scarr, Elizabeth, S70
Schäfer, Miriam R., S228
Schäfer, Miriam, S132
Schäppi, Lea, S194, S198
Schæbel, Helle, S424
Schöbi, Dario, S72, S281, S331
Schack, Stephan, S221
Schaefer, Miriam, S99, S116, S326
Schafer, Miriam, S378
Schalbroeck, Rik, S104
Schalit, Liat, S306
Schaum, Michael, S250, S387
Scheepers, Floor, S92
Scheffler, Freda, S261, S367
Scheffler, Frederika, S364
Scheller, Bertram, S340
Schieveld, Jan, S219
Schifani, Christin, S43

Schiffman, Jason, S43
Schirmbeck, Frederike, S107, S269
Schlögelhofer, Monika, S99, S132, S228
Schlagenhauf, Florian, S172, S278
Schlogelhofer, Monika, S116, S378
Schmidt, André, S102, S199, S281
Schmidt, Andre, S383
Schmidt, Lyndsay, S305
Schmidt, Stefanie, S228
Schmitt, Andrea, S17
Schnack, Hugo, S101, S287
Schneider, Karoline, S284
Schneider-Axmann, Thomas, S343
Schneider-Thoma, Johannes, S115, S418
Schooler, Nina, S216, S320
Schoretsanitis, Georgios, S198
Schork, Andrew, S85
Schröder, Johannes, S115, S140, S289
Schreuder, Marieke, S14
Schruers, Koen, S76
Schulte, Raphael, S67, S239
Schultze-Lutter, Frauke, S109, S209, S228, S407
Schulz, Steffen, S152
Schulze, Thomas G., S155
Schumann, Andy, S152
Schurhoff, Franck, S155, S160
Schutte, Maya, S109, S173
Schwan, Raymund, S169
Schwannauer, Matthias, S279, S282, S340, S409
Schwannauer, Matthis, S385
Schwartenbeck, Philipp, S385
Schwartze, Michael, S380
Schwenk, Jochen, S120
Schwippel, Tobias, S355
Schwitzer, Thomas, S169
Scodes, Jennifer, S107
Scott, James, S61, S237, S379
Seabra, Gabriela, S297
Seccomandi, Benedetta, S196
Secher, Rikke Gry, S163
See, Yuen Mei, S309
Seedat, Soraya, S364
Segan, Catherine, S151
Seibyl, John, S176
Seidel, Jan, S85
Seidl, Ulrich Seidl, S140
Seidl, Ulrich, S289
Seidman, Larry J., S78, S79, S90, S117, S194, S223, S231, S235, S366
Seidman, Larry, S39, S54, S62, S102, S104, S236, S266, S396, S422
Seifritz, Erich, S81, S174, S284
Selten, Iris, S226
Selten, Jean-Paul, S67, S104, S256, S341
Selvaggi, Pierluigi, S83
Seminerio, Fabio, S258, S354
Sendt, Kyra-Verena, S382
Seo, Ho Jun, S307
Seo, Ho-Jun, S242
Seo, Hye-Jin, S201
Seppälä, Annika, S144
Seppälä, Jussi, S144, S168
Serpa, Mauricio Henriques, S376
Serpa, Mauricio, S133, S195
Servaas, Michelle, S177
Seth, Anil, S173
Setién-Suero, Esther, S251, S268
Setiaman, Nikita, S287
Setien-Suero, Esther, S92, S142, S184, S373, S374, S392
Seves, Mauro, S114, S209, S285
Sevigny, Jeffrey, S340
Sevilla-Llewellyn-Jones, Julia, S151
Sevy, Serge, S244
Shafer, Anna, S288
Shafner, Laura, S134
Shah, Jai, S56, S182, S185
Shah, Parita, S329
Shalhoub, Huda, S241
Sham, Pak, S7, S158, S354
Shan, LeeAnn, S264, S353
Shang, Jing, S283
Shannon, Ciaran, S317
Shapiro, Dan, S54
Shapiro, Daniel I., S36, S78, S366
Sharma, Shivani, S220
Shatzi, Vasiliki, S50
Shen, Dinggang, S103
Shenton, Martha E., S36, S104, S366
Shenton, Martha, S90, S117
Shergill, Sukhi, S92, S164, S174, S249, S280, S383
Shetty, Hitesh, S411
Shi, Hai-song, S244
Shi, Li-juan, S333
Shi, Yan, S331
Shimada, Takamitsu, S248
Shin, Il-Seon, S411
Shinde, Mayura, S425
Shivakumar, Venkataram, S345
Shon, Seung-Hyun, S219, S387, S391
Shon, Seung-hyun, S287
Short, Sarah, S103
Shuhama, Rosana, S227
Shukla, Ayushi, S181
Shum, Carol, S105
Shungu, Dikoma, S2
Siamouli, Melina, S393
Siddiqui, Ishraq, S306
Sideli, Lucia, S75, S258, S354
Sidovar, Matt, S425
Siemerkus, Jakob, S331
Siettou, Eleni, S243, S348
Sigvard, Anne, S224, S383, S387
Silva, Nicole, S21
Silva, Tania Da, S390
Silvana, Jelavić, S257
Silverman, Bernard, S312, S413
Silverstein, Steven, S10, S139, S140, S401
Sim, Kang, S123, S324
Sim, Xueling, S12
Simmons, Adam, S312, S413
Simons, Claudia, S269
Simonsen, Arndis, S169, S350
Simonsen, Erik, S272
Sing, Wong Siu, S427
Singer, Wolf, S340
Singh, Arun, S130, S311
Sinkeviciute, Igne, S95, S261
Sintes, Anna, S229
Siskind, Dan, S66, S178
Siu, Cynthia, S242, S267
Sivapalan, Sudhakar, S295
Sjöstedt, Evelina, S120
Sjöström, Nils, S211, S212, S305, S407
Skidmore, Frank, S91
Sklar, Pamela, S34
Skosnik, Patrick, S27
Skovgaard, Anne Mette, S103, S334
Skuban, Aleksandar, S94
Skurnik, Norbert, S167
Skymba, Haley, S4
Slavu, Ruxandra, S160
Slezak, Diego Fernández, S43
Slifstein, Mark, S2, S71
Slikboer, Reneta, S406
Sloan, Richard, S17
Smallman, Richard, S131, S148
Smelror, Runar, S306
Smesny, Stefan, S116, S121, S122, S132, S221, S228, S378
Smesny, Stephan, S99, S228
Smith, Adam, S139
Smith, Eleanor, S301
Smith, Geoffrey, S291

Smith, George Davey, S165, S273, S379
Smith, Shubulade, S87
Smith, Thomas, S107, S108
Snodgress, Matthew, S47, S77
So, Joyce, S296, S399
So, Suzanne, S161
Soares, Bernardo, S130
Soares, Walter, S378
Solberg, Dag, S122
Soldevila-Matias, Pau, S200, S276
Soldevilla, Pablo, S157
Sole, Montserrat, S253, S303
Solida, Alessandra, S389
Somes, Nathaniel, S90, S194
Sommer, Iris E, S95
Sommer, Iris, S51, S67, S109, S173
Song, In Chan, S186
Song, Min-Kyu, S161, S218, S236, S264, S296, S304, S308, S326, S367
Sonnenschein, Susan, S338
Soo, Kwon Jun, S330
Sood, Poonam, S87, S166, S246
Sorg, Christian, S283
Sormunen, Elina, S272
Sotelo, Eva, S249, S392
Soto, Marta, S417
Spang, Katrine Søborg, S245, S336
Spang, Katrine S., S300
Spang, Katrine, S110, S252
Spaniel, Filip, S173
Sparano, Olga, S157, S200, S276
Spencer, Kevin M., S117
Spinz, Rodrigo, S288
Spiros, Athan, S294, S411
Spoorthy, Mamidipalli, S322, S405
Sportel, Bouwina, S260
Srihari, Vinod, S272, S426
Sriretnakumar, Venuja, S296, S399
Srivastava, Deepak, S105
Staal, Nina, S333
Stabell, Lena, S346
Stadsgaard, Henriette Brockdorff, S336
Stadsgaard, Henriette, S252
Stafford, Jean, S377
Stahl, Daniel, S56, S87
Stain, Helen, S321
Stainton, Alexandra, S357
Stansley, Branden, S70, S127
Starace, Fabrizio, S426
Stavely, Heather, S321
Steen, Nils E., S205
Steen, Nils Eiel, S227, S362
Steen, Vidar M., S227
Steen, Vidar Martin, S358
Stefan, Just, S143
Stefan, Kaiser, S174
Stefanics, Gabor, S281
Steffens, Maria, S119, S386
Stegmayer, Katharina, S175, S184, S194, S198
Steichen, Conny, S239
Steiner, Johann, S40
Stenstrøm, Anne Dorte, S333
Stephan, Klaas Enno, S72, S254, S278, S281, S314, S331
Stepien, Marta, S284
Steullet, Pascal, S1, S395
Stewart, Robert, S72, S84, S154, S411
Stewart, Sherry, S153
Stilo, Simona, S166, S246
Stip, Emmanuel, S17
Stochl, Jan, S48, S57, S379
Stockmeier, Craig, S374
Stockton-Powdrell, Charlotte, S106
Stoeter, Peter, S277
Stojanovski, Sonja, S226
Stolz, Erin, S2
Stone, James, S81, S328
Stone, William S., S36, S78, S90, S102, S104, S223, S366
Stowkowy, Jacqueline, S231
Strahimir, Sučić, S257
Straub, Richard, S85
Straube, Benjamin, S195
Strik, Werner, S184
Stringer, Dominic, S87
Strippoli, Marie-Pierre, S254
Strube, Wolfgang, S343, S355, S393, S406, S416
Stubberud, Jan, S356
Stubbs, Brendon, S87, S330, S405
Sturm, Elisabeth, S388
Styner, Martin, S194
Su, Wayne, S291
Subotnik, Kenneth L., S136
Subotnik, Kenneth, S18
Subramaniam, Karuna, S278
Subramanian, Siva, S235
Suckling, John, S131, S148
Suen, Yi Nam, S138, S214, S215, S372
Sugar, Catherine, S216, S320
Sugranyes, Gisela, S123, S181, S183, S229, S337
Sullivan, Courtney, S19, S19
Sultana, Tania, S129
Sumiyoshi, Chika, S419
Sumiyoshi, Tomiki, S419
Sumner, Philip, S78, S137, S143, S246, S279
Sun, Bin, S192
Sun, Xiaoqi, S161
Sundar A, Shyam, S136
Sundram, Suresh, S224
Sundvall, Jouko, S285
Suokas, Kimmo, S88
Surcel, Heljä-Marja, S272
Surti, Toral, S142
Susser, Ezra, S271
Sutterland, Arjen, S86
Suvisaari, Jaana, S68, S69, S80, S88, S172, S285, S349
Suvitaival, Tommi, S69
Suzuki, Hirobumi, S412
Suzuki, Kazunori, S412
Suzuki, Michio, S394
Suzy, Harrigan, S162
Svendsen, Ingrid, S410
Svenning, Jens-Christian, S59
Swami, Viren, S368
Sweeney, John A., S121
Sweeney, John, S6, S14
Sweeney, Rohan, S151
Sweeney, Shaun, S408
Swillen, Ann, S86
Szöke, Andrei, S166
Szele, Francis, S192
Szoke, Andrei, S155, S160, S167

Tak, Erwin, S407
Taka-Eilola, Tiina, S164
Takahashi, Tsutomu, S394
Takao, Hensch, S1
Takayanagi, Yoichiro, S394
Takegoshi, Yusuke, S394
Takei, Nori, S277
Takeuchi, Hiroyoshi, S267, S309, S354, S413
Talkowski, Michael, S33
Talor, Monica, S190
Tamagnan, Gilles, S176
Tamburri, Paul, S340
Tamminga, Carol A., S121
Tamminga, Carol, S6, S14
Tan, Eric, S78, S137, S139, S143, S246, S252, S321, S359
Tan, Wilson, S130
Tanase, Costin, S126
Tandi, Jesisca, S281
Tandon, Rajiv, S241
Tang, Charmaine, S309
Tang, Yee Man, S372
Tang, YinYin, S90
Tang, YingYing, S36, S366

Tang, Yingying, S104, S223
Tang, Yun-Xiang, S121
Tangmose, Karen, S224, S383, S387
Tanguy, Audrey, S268, S420
Tania,Kümpfel, S261
Tanja,Franćeski, S369
Tanskanen, Antti, S379
Tao, Haojuan, S187
Tardif, Christine, S182
Tarquis, Corentin, S420
Tarricone, Ilaria, S75, S123
Taylor, Adam, S27
Taylor, David, S310
Taylor, Jennifer, S61
Taylor, Matthew, S310
Taylor, Valerie, S413
Teasdale, Scott, S405
Teigset, Charlotte, S245, S401
ten Have, Margreet, S160
ten Kate, Corine, S341
Tenan, Silvia, S166
Tenback, Diederik, S407
Teng, Irving, S214
Teo, Celine, S104
Termorshuizen, Fabian, S104
Teufel, Christoph, S377
Thanju, Amod, S275, S408
Theberge, Jean, S179, S179, S221, S327
Theo, Broekman, S239
Theochari, Eirini, S243, S348
Therman, Sebastian, S349
Thermenos, Heidi, S194
Therrien, Francois, S133
Thien, Kristen, S162, S270, S376
Thiery, Evert, S84
Thimm, Jens, S324
Thirthalli, Jagadisha, S136
Thomann, Philipp, S140, S289
Thomas, Anthony, S91
Thomas, Elizabeth, S137, S143, S246
Thomas, McGlashan, S422
Thomas, Megan, S301
Thomas, Neil, S78, S260, S302
Thompson, Andrew, S99, S116, S132, S228, S321, S378
Thompson, Judy, S10
Thompson, Wesley, S85
Thomson, David, S234
Thomson, Sumana, S137
Thorndike, Anne, S361
Thornton, Allen, S291
Thorup, Anne A.E., S336
Thorup, Anne Amalie, S111, S245, S300
Thorup, Anne, S111, S163, S252
Thuné, Hanna, S279, S282, S385
Tian, Ye, S282, S295
Tibbo, Philip, S153, S295
Tiemeier, Henning, S231, S402
Tiihonen, Jari, S67, S88
Tikkala, Jenni, S345
Timmerman, Marieke, S238
Tingue, Samantha, S121
Tiwari, Arun, S115, S218, S412
Tobler, Philippe N., S81, S284
Tocco, Michael, S114, S239, S242, S353
Tognin, Stefania, S393
Toh, Wei Lin, S78, S252
Töhönen, Tuomo, S345
Tomasetti, Carmine, S313
Tomiello, Sara, S72, S281, S314, S331
Ton, Trang, S420
Tonsig, Daniela Koga, S375
Tor, Jordina, S183, S229
Tordesillas-Gutiérrez, Diana, S184, S259, S392
Tordesillas-Gutierrez, Diana, S92, S142, S251, S268
Torgalsboen, Anne-Kari, S141, S250, S253
Torniainen-Holm, Minna, S88, S285, S349
Torregrossa, Lénie, S47, S77, S195, S196, S421
Torrent, Carla, S222
Torretta, Silvia, S100, S193
Torrey, E. Fuller, S24
Tortelli, Andrea, S167
Tortorella, Alfonso, S201, S363
Tost, Meritxell, S251
Toutountzidis, Diamantis, S220
Touya, Maelys, S97
Towell, Tony, S368
Trabelsi, Rouaa, S163, S423
Traber-Walker, Nina, S230, S336
Tracy, Derek, S280
Tran, Nga, S214
Tréhout, Maxime, S396
Tripoli, Giada, S7, S59, S75, S110, S158, S246, S258, S354
Tröger, Laura, S168
Trotta, Antonella, S59, S97, S166, S246
Trudeau, Jeremiah, S139
Tsai, Joyce, S242
Tsaltas, Eleftheria, S243, S348
Tse, Desmond, S182
Tseng, Huai-Hsuan, S389
Tseng, Wen-Yih Isaac, S186
Tsirogiannis, Constantinos, S59
Tsuang, Ming T., S78, S231
Tsuang, Ming, S39, S62, S79, S102, S266, S396, S422
Tsujino, Naohisa, S315
Tucha, Oliver, S177
Tully, Laura, S3, S3
Tunca, Zeliha, S356
Tuominen, Lauri, S391
Turetsky, Bruce, S57
Turkheimer, Federico, S81, S83
Turner, Alyna, S151
Turner, Luana, S18
Tuulio-Henriksson, Annamari, S88
Tuval-Maschiach, Rivka, S165
Tyler, Teadora, S192
Tzivoni, Yair, S306
Tzoumakis, Stacy, S229

Uchino, Takashi, S315
Uehara, Takashi, S248
Ueland, Torill, S356
Ugolini, Annarosa, S412
Uher, Rudolf, S252
Uhlén, Mathias, S120
Uhlhaas, Peter, S2, S279, S282, S340, S385, S409
Ulas, Halis, S368
Ullrich, Johannes, S254
Ulshen, Danny, S159
Umbricht, Daniel, S340
Umeh, Annabel, S171
Ümit,Tural, S316
Undurraga, Juan, S202, S269, S364
Unruh, Leonhardt, S327
Unsöld, Svenja, S128
Upthegrove, Rachel, S48, S48, S131, S144, S147, S148, S159, S209, S209, S280, S283, S328, S357, S370
Uranova, Natalya, S41
Urban, Alexander, S33
Uriarte, Jose Juan Uriarte, S205
Urquijo, Maria F., S119, S386
Urquijo, Maria Fernanda, S144
Ursini, Gianluca, S82, S85
Usall, Judith, S113, S208, S212, S370, S410

V. J. Ramesh,, S136
Vahdani, Bita, S344
Väisänen, Heli, S345
Vakhrusheva, Julia, S17
Vakulin, Andrew, S284
Valiengo, Leandro, S133

Valmaggia, Lucia, S307
Van Der Water, Judy, S126
Van Haren, Neeltje, S77, S109, S242
Van Harten, Peter N., S325
Van Harten, Peter, S295
Van Keuren-Jensen, Kendall, S221
Van Os, Jim, S295
Van Rheenen, Tamsyn, S176
Van Snellenberg, Jared, S44
Van de Bilt, Martinus, S133
Van der Gaag, Mark, S344
Van der Zalm, Yvonne, S67
Vanbellingen, Tim, S184
Vandeleur, Caroline, S254
Vandenberghe, Frederik, S310
Vandevelde, Anaïs, S396
Vanes, Lucy, S92, S383
Vanover, Kimberly, S236, S320, S341
Vaquero, Mercedes, S249, S392
Varambally, Shivarama, S181
Varela, Eva, S123
Vargas, Teresa, S42
Varley, James, S61
Vasconcelos, Selene, S262
Vasile, Daniel, S27
Vaskinn, Anja, S253
Vassilouli, Spyridoula, S243, S348
Vassos, Evangelos, S7, S59, S158, S246
Vaz, Stephanie McDermid, S149
Vázquez-Bourgon, Javier, S184, S259
Veddum, Lotte, S336
Vedul-Kjelsås, Einar, S113
Veijola, Juha, S88, S101, S164, S272
Vekich, John, S412
Velarde, Amalia, S72
Velden Hegelstad, Wenche ten, S153
Veling, Wim, S271, S344, S419
Velligan, Dawn, S30, S31, S404
Vellucci, Licia, S313
Velthorst, Eva, S39, S39, S40, S266, S269
Venkatasubramanian, Ganesan, S181, S345
Ventura, Joseph, S18, S136
Verbruggen, Lise, S20
Verde, Pablo, S343
Verdu, Elena, S190
Veres, Daniel, S192
Vergaelen, Elfi, S86
Verhulst, Frank, S103, S334
Verma, Swapna, S99, S116, S132, S228, S378
Vermeulen, Jentien, S86
Vernal, Ditte Lammers, S333
Vernon, Anthony, S65, S191
Veronese, Mattia, S81, S83, S291, S395
Vestergaard, Mogens, S88
Vicente, Molina, S392
Vidrih, Branka, S369
Viechtbauer, Wolfgang, S341
Vieira, Sandra, S92, S374
Viertio, Satu, S32
Vieta, Eduard, S5, S318
Vignapiano, Ananrita, S264
Vignapiano, Annarita, S263
Viher, Petra, S184, S194
Viikari, Jorma, S272
Vikhreva, Olga, S41
Vila-Badia, Regina, S113, S210, S212
Vilaplana, Claudia, S251
Vilella, Elisabet, S253, S303
Villagonzalo, Kristi-Ann, S302
Villalobos, Anna, S176
Villaplana, Anna, S210
Villarreal, Mirta F., S286
Villers, Agnès, S20
Vilme, Mike, S361
Vilner, Yael, S339
Vincent, Andrew, S284
Vincent, Angela, S141
Vincent, John, S296
Vingerhoets, Claudia, S182, S275, S391
Vink, Matthijs, S225
Vinkers, Christiaan H., S109
Vinkers, Christiaan, S82
Vinogradov, Sophia, S278
Virdee, Gursharan, S31
Viviano, Joseph, S176, S226
Vizzotto, Adriana, S262
Vohs, Jennifer, S319
Voineskos, Aristotle, S115, S176, S226, S306, S422
Volkow, Nora, S45
Vollenweider, Franz X, S281
Vorstman, Jacob, S247, S376
Voss, Andreas, S152
Voutilainen, Greta, S349
Vreira, Sandra, S331
Vucurovic, Ksenija, S381
van Amelsvoort, Therese, S86, S183, S275
van Borkulo, Claudia, S96
van Dijk, Floor, S107
van Donkersgoed, Rozanne, S260
van Dorsselaer, Saskia, S160
van Duin, Esther, S182
van Eindhjoven, Phillip, S183
van Elst, Ludger Tebartz, S11
van Erp, Theo, S396
van Haren, Neeltje, S225, S287
van Harten, Peter, S407
van Opstal, Daniel P.J., S101
van Os, Jim, S7, S76, S84, S103, S117, S158, S160, S166, S219
van Oudenaren, Mathilde, S182
van Praag, Cassandra Gould, S173
van Rooijen, Geeske, S86
van Themaat, Anna Hester Ver Loren, S300
van Tol, Marie-José, S177
van de Bilt, Martinus Theodorus, S195, S376
van de Chapelle, Chuck, S233
van de Kerkhof, Marita, S86
van den Bree, Marianne, S86
van der Burg, Nadine, S295
van der EL, Kristi, S321
van der Gaag, Mark, S238
van der Leeuw, Christine, S117
van der Ley, Claude, S117
van der Merwe, Nicolaas, S399
van der Weiden, Anouk, S77
van-der-EL, Kristi, S404
von Nielander, Luisa, S112
von Plessen, Kerstin, S111, S300

Wade, Joshua, S77, S195, S196, S421
Waern, Marda, S305
Waern, Margda, S211, S212, S407, S426
Wagner, Michael, S386
Wainsztein, Agustina, S286
Waite, Felicity, S335
Wakuda, Tomoyasu, S277
Walitza, Susanne, S230, S336
Walker, Elaine F., S78, S102, S231
Walker, Elaine, S35, S39, S62, S79, S266, S396, S422
Wall, Matthew, S385
Wall, Melanie, S107
Wallace, Anna, S425
Walling, David, S139, S340
Walters, James, S165, S273, S379
Walther, Mario, S121
Walther, Sebastian, S42, S175, S184, S194, S198
Waltz, James, S43
Wan, Claire Ramsey, S259
Wang, Changming, S329
Wang, Chenhao, S281
Wang, Chuan-Yue, S352

Wang, Chuanyue, S241, S329
Wang, Hee Ryung, S201
Wang, JiJun, S90, S223, S366
Wang, JianLi, S355
Wang, Jijun, S36, S104
Wang, Junjie, S223, S366
Wang, Qiang, S90, S394
Wang, Yabing, S295
Wang, Yen-Chin, S274
Wang, Yi, S244
Wang, Yiming, S390
Wang, Yingcheng, S90, S394
Wang, Yunpeng, S85
Wannan, Cassandra, S176
Ward, Philip, S405
Wartelsteiner, Fabienne, S421
Waterreus, Anna, S365
Waters, Patrick, S61
Watkins, Melanie, S343
Watmuff, Bradley, S400
Watson, Andrew, S148
Webb, Roger T., S167
Webb, Roger, S388
Weber, Lilian, S72, S281, S331
Weber-Stadlbauer, Ulrike, S65, S234
Webster, Maree, S38
Wegelius, Asko, S88, S345
Wei, Deng, S90, S394
Weibell, Melissa, S153
Weickert, Cynthia Shannon, S24, S38, S64, S73, S74
Weickert, Thomas, S73
Weijers, Jonas, S341
Weimand, Bente, S211
Weinberger, Daniel R., S82
Weinberger, Daniel, S85
Weiner, Ina, S65, S191
Weingart, Michal, S236, S341
Weis, Laura, S368
Weiser, Mark, S23, S23, S165
Weiske, Johanna, S168
Weiss, Catherine, S133, S241
Weizman, Abraham, S339
Welikson, Tamara, S136
Wells, Ruth, S73
Wellstein, Katharina, S254, S314
Wen, Zhexing, S299
Wenneberg, Christina, S119
Werbeloff, Nomi, S23
Werge, Thomas, S85
Werner, Lisa, S11
Westerhausen, Rene, S293
Wexler, Bruce, S142
Wexler, Deborah, S361
Wheeler, Anne, S226
Whelan, Pauline, S106
White, Amanda, S37
White, David, S41, S91, S120
Whitfield-Gabrieli, Susan, S90, S104, S194
Whittingham-Dowd, Jayde, S233, S234
Wibral, Michael, S250, S340, S387
Wichers, Marieke, S14, S84, S124
Widman, Linnea, S67
Wiest, Roland, S175, S184
Wiffen, Ben, S246
Wigman, Hanneke, S14
Wigman, Johanna, S96, S334
Wigton, Rebekah, S249
Wijtenburg, Andrea, S20, S186
Wilkin, Jennifer, S104
Willi, Cosima, S233
William, Lo Tak-Lam, S427
Williams, Jill, S151
Williams, Lana, S411
Williams, Leanne, S192
Williams, Stephen, S131, S148
Williams, Steve, S118, S413
Williams, Steven CR, S83, S89
Wilson, Alan, S43, S390
Wilson, Clayton, S149
Wilson, Nathan, S78
Wilson, Robert, S61
Wilson, Robin, S28, S154, S384
Wiltfang, Jens, S128
Winckel, Karl, S178
Wingård, Louise, S120
Winje, Dagfinn, S324
Winkel, Ruud van, S84
Winter, Inge, S51, S74
Winterer, Georg, S343
Witte, Lot D., S82
Witte, Lot de, S117
Wittert, Gary, S284
Wo, Sui Fung, S214
Wobrock, Thomas, S343
Wojcik, Joanne, S235, S236
Wolf, Achim, S23
Wolf, Daniel, S57
Wölwer, Wolfgang, S343
Wong, Cheuk Fei, S214
Wong, Chloe, S25
Wong, Katie, S92
Wong, Keri, S333, S334
Wong, Kwok Tung Gordon, S208
Wong, Tien, S12
Woo, Tsung-Ung Wilson, S235, S236
Wood, Joel, S63
Wood, Stephen, S98, S144, S147, S209, S209, S280, S281, S316, S328, S334, S357, S370
Woodall, Tamara, S334
Woodberry, Kristen, S36, S54, S78, S366
Woods, Scott W., S78, S102, S231
Woods, Scott, S39, S62, S79, S266, S396, S422
Woods, Will, S138
Woodward, Melissa, S291
Woodward, Todd, S388
Woopen, Christiane, S285
Worbe, Yulia, S360
Wright, Abigail, S212
Wright, Stephen, S27
Wright-Martin, Jillian, S322
Wulff, Sanne, S424
Wulfman, René, S167
Wunderink, Alexander, S260
Wunderink, Lex, S238
Wykes, Til, S106, S196, S307

Xiang, Yu-Tao, S352
Xiao, Lan, S42, S297
Xin, Lijing, S15, S133, S236
Xiong, Lan, S352
Xu, Feng, S289
Xu, Junzhe, S188, S189
Xu, LiHua, S223, S366
Xu, Xin, S331
Xu, Zhida, S82

Yalcin, Nadir, S345
Yamagishi, Mika, S381
Yamaguchi, Taiju, S315
Yamamori, Hidenaga, S419
Yamasue, Hidenori, S277
Yan, Tingjian, S97, S248, S366
Yan, Wenjing, S170
Yanamori, Hidenaga, S85
Yang, Cheng, S331
Yang, Lawrence, S54
Yang, Rang, S129
Yang, So Yung, S362
Yang, Yen Kuang, S389
Yang, Ziyi, S63
Yasuda, Yuka, S419
Yasuyama, Toshiki, S248
Yazbek, Hanan, S359
Yazici, Mumin Kazim, S345
Yee, Jie Yin, S309
Yi, Jung-Seo, S403
Yi, Sang Hoon, S203
Yi, Zhen-hui, S358
Yildiz, Mustafa, S211, S283, S316, S419
Yoffe, Rinat, S23

Yohn, Samantha, S70, S127, S204
Yokokura, Masamichi, S277
Yolken, Robert H, S63
Yolken, Robert H., S150
Yolken, Robert, S62, S63, S68
Yolland, Caitlin, S252
Yoo, Taeyoung, S177
Yoon, Bo-Hyun, S161, S201, S206, S215, S218, S236, S264, S296, S304, S326, S401, S411
Yoon, Jin-Sang, S177, S411
Yoon, Woon, S219, S287, S387, S391
Yoon, Youngwoo, S186
York, Whitney, S425
Yoshida, Kazunari, S190
Yoshikawa, Etsuji, S277
Youn, Tak, S203
Young, Jared, S233
Yousefi, Vida, S344
Yu, Hua, S90, S394
Yu, Yaqin, S82
Yuan, Jianxin, S188
Yuan, Jiaxin, S189
Yuen, Hok Pan, S99, S116, S130, S132, S228, S326, S378
Yum, Jiyun, S307
Yung, Alison Ruth, S132
Yung, Alison, S99, S116, S156, S228, S312, S334, S378, S388

Zahid, Uzma, S118
Zahle, Kate Kold, S252
Zai, Clement, S115, S218, S399, S412
Zakskorn, Lauren, S3
Zakzanis, Konstantine, S306, S354
Zalesky, Andrew, S74, S91, S176, S282
Zammit, Stan, S325
Zammit, Stanley, S39, S48, S53, S72, S165, S247, S270, S273, S377, S379
Zanatta, Daniela, S166
Zandian, Arasch, S120
Zanelli, Jolanta, S77
Zang, Feifei, S331
Zaytseva, Yulia, S173
Zelaya, Fernando, S81, S382
Zenoozian, Saeedeh, S344
Zhand, Naista, S274
Zhang, Chengcheng, S121, S301
Zhang, Fan, S194
Zhang, Landan, S156
Zhang, Lei, S176
Zhang, Peter, S133
Zhang, TianHong, S36, S90, S104, S223, S366
Zhao, Liansheng, S90, S394
Zhao, Wei, S187
Zhening, Liu, S187
Zhou, Baiwan, S331
Zhou, Fuchun, S352
Zhou, Han-yu, S333
Zhou, Juan, S9, S281
Zhou, Ping, S352
Zhu, Xiyu, S75
Zia, Aqsa, S3
Zijl, Peter van, S289
Zink, Mathias, S371
Zipursky, Robert, S267, S415, S418
Zorzi, Carline, S389
Zouhaier, El Hechmi, S152
Zuardi, Antonio W., S297
Zuardi, Antonio Waldo, S28
Zubiaurre-Elorza, Leire, S347
Zugman, André, S184
Zugman, Andre, S286
Zunszain, Patricia, S118
Žarko,Bajić, S257, S257, S20, S136, S259, S275, S316
